# Supplementary material for: Temporal genetic association and temporal genetic causality methods for dissecting complex networks
Source: Nat Commun. 2018 Sep 28;9:3980. doi: 10.1038/s41467-018-06203-3 (PMC6162292; doi:10.1038/s41467-018-06203-3)
Supplement: Supplementary file 1 — Supplementary Information [file 41467_2018_6203_MOESM1_ESM.pdf]

Supplementary Materials for

Temporal Genetic Association and Temporal Genetic Causality Methods for Dissecting Complex Networks

Luan Lin[1,2], Quan Chen[1,2], Jeanne P. Hirsch[3], Seungyeul Yoo[1,2], Kayee Yeung[4], Roger E. Bumgarner[4], Zhidong Tu[1,2], Eric E. Schadt[1,2,5], and Jun Zhu[1,2,5*]

[1]Department of Genetics and Genomic Sciences, [2]Icahn Institute for Genenomics and Multiscale Biology, [3]Department of Pharmacology and Systems Therapeutics, Icahn School of Medicine at Mount Sinai, New York, NY, 10029, USA; [4]Department of Microbiology, University of Washington, Box 358070, Seattle, WA, 98195, USA; [5]Sema4, a Mount Sinai venture, Stamford, CT, 06902

[*]Correspondences should be addressed to

Dr. Jun Zhu
Department of Genetics and Genomic Sciences
Icahn Institute for Genomics and Multiscale Biology
Icahn School of Medicine at Mount Sinai
New York, NY 10029
jun.zhu@mssm.edu

Contents

1. Supplementary Discussion

- **Overfitting in temporal-genetic association methods**

2. Supplementary Figures

Supplementary Figure 1 Possible causal relationships between two traits X and Y linked to a locus L.
Supplementary Figure 2: Various patterns used to generate the simulation data sets.
Supplementary Figure 3: Distribution of auto-correlation coefficients ρ for all gene expression traits in the yeast time series data.
Supplementary Figure 4: Performance comparison among different temporal genetic association methods.
Supplementary Figure 5: Performance comparison when there are missing data.
Supplementary Figure 6: Power to distinguish causal/reactive models.
Supplementary Figure 7: The distribution of the log likelihood ratio between causal and reactive models.
Supplementary Figure 8: Power of the temporal-genetic causality test for data simulated under the causal model.
Supplementary Figure 9: The distribution of the BIC difference between the causal model and other models.
Supplementary Figure 10: Power of the temporal-genetic causality test on data simulated under the independent model.
Supplementary Figure 11: Power of the temporal-genetic causality test on data simulated under the partial model.
Supplementary Figure 12 The distribution of the BIC difference between the causal model and other models based on yeast F2 data for all causal pairs in Supplementary Table 11.
Supplementary Figure 13 The distribution of the BIC difference between the causal model and other models based for *RRD1* regulated genes.
Supplementary Figure 14: Individual expression pattern of RPP2A which was linked to the teQTL hot spot chrIX:70,000.
Supplementary Figure 15 Average *RRD1* expression levels for segregants carrying RM and BY alleles at *RRD1* locus.
Supplementary Figure 16: Comparison of p-values of MPTGA and the regression methods.
Supplementary Figure 17 Assessing overfitting problem by comparing association p-values of peak SNPs and neighboring SNPs in strong LD.
Supplementary Figure 18 QQ plots of the p-values of the MPTGA test.
Supplementary Figure 20: Gene expression examples in the yeast time series data showing insufficient power of linear or quadratic polynomial fitting.
Supplementary Figure 21: Pattern fitting examples.
Supplementary Figure 22: Goodness of fit using different degree of polynomial function for fitting the yeast time series data set.

3. Supplementary Tables

- **Supplementary Table 1:** A list of significant eQTLs at FDR<0.05 identified by the static method (considering only data at the time zero).
- **Supplementary Table 2:** A list of significant eQTLs at FDR<0.05 identified by the MPTGA method.
- **Supplementary Table 3:** A list of significant eQTLs at FDR<0.05 identified by the union method.
- **Supplementary Table 4:** A list of significant eQTLs at FDR<0.05 identified by the regression method.
- **Supplementary Table 5:** A list of significant eQTLs at FDR<0.05 identified by the Fisher's p-value method.
- **Supplementary Table 6:** A list of expression traits in eQTL hot spots defined by the static method (considering only data at the time zero).
- **Supplementary Table 7:** A list of expression traits in eQTL hot spots defined by the MPTGA method.
- **Supplementary Table 8:** A list of expression traits in eQTL hot spots defined by the union method.
- **Supplementary Table 9:** A list of expression traits in eQTL hot spots defined by the regression method.
- **Supplementary Table 10:** A list of expression traits in eQTL hot spots defined by the Fisher's p-value method.
- **Supplementary Table 11:** A list of causal relationships defined by TGCT.

Supplementary Discussion
Overfitting in temporal-genetic association methods

To assess the tendency of sporadic association or overfitting in each method (Methods), we compared p-values of significant associations in both empirical and permuted data and the p-values of neighboring SNPs that are in strong linkage disequilibrium (LD). When checking empirical data result, we collected associations of peak SNPs with p-values less than 10^{-6} and 10^{-11} for the MPTGA and the regression method (different p-value thresholds but giving rise to the same FDR), respectively, and compared associations of the traits and neighboring SNPs in high LD. Supplementary Figs. 17A and 17B show the p-values for peak SNPs and neighboring SNPs in high LD are highly correlated, with correlation coefficients of 0.89 and 0.69 for the MPTGA and the regression method, respectively. The result suggests that the MPTGA is less prone to overfitting than the regression method as we expect. However, the above result may be biased because the underlying signals are strong and associations are linked to a few eQTL hot spots. To assess the overfitting problem when signals are subtle, we performed the same test on results based on permuted data, where in such data portions of true signal will occur by chance. Similarly, we checked the results based on permuted data and collected associations of peak SNPs p-values less than 10^{-6} and 10^{-11} for the MPTGA and the regression method, respectively, and then compared associations of the traits and neighboring SNPs in high LD. Supplementary Figs. 17C and 17D show the p-values for peak SNPs and neighboring SNPs in high LD are highly correlated, with correlation coefficients of 0.71 and 0.31 for the MPTGA and the regression method, respectively. Again, the result suggests that the MPTGA is less prone to overfitting than the regression method.

Supplementary Figures

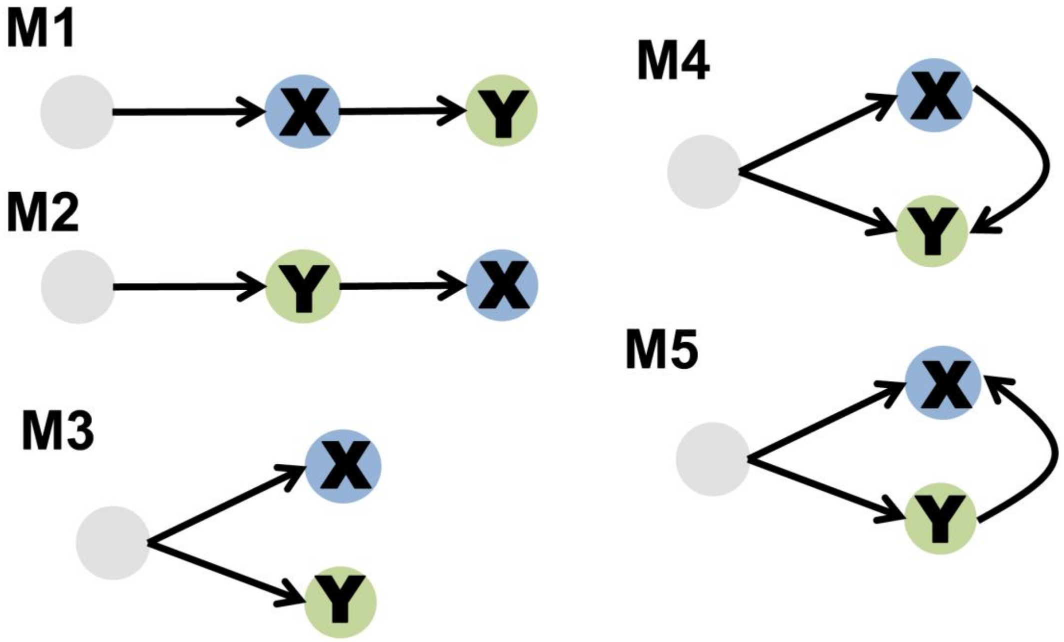

Supplementary Figure 1 Possible causal relationships between two traits X and Y linked to locus L (grey node).

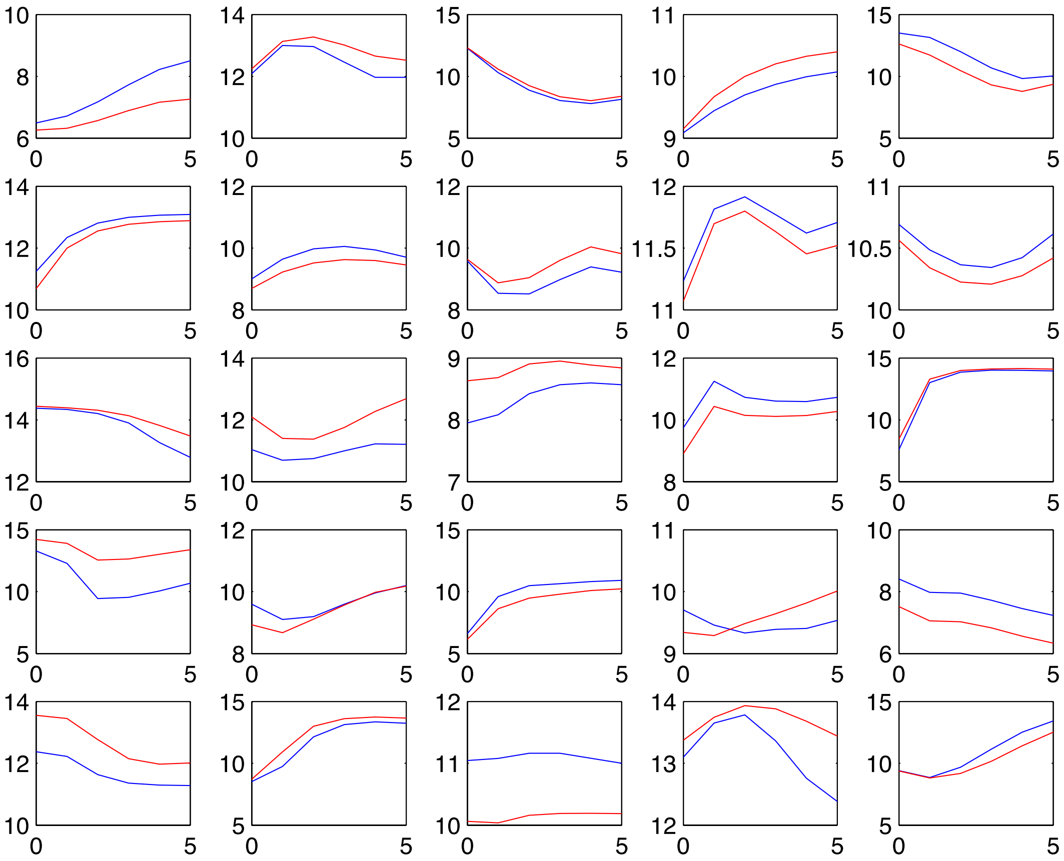

Supplementary Figure 2: Various patterns used to generate the simulation data sets which are similar to the observed patterns in the F2 yeast time series data set.

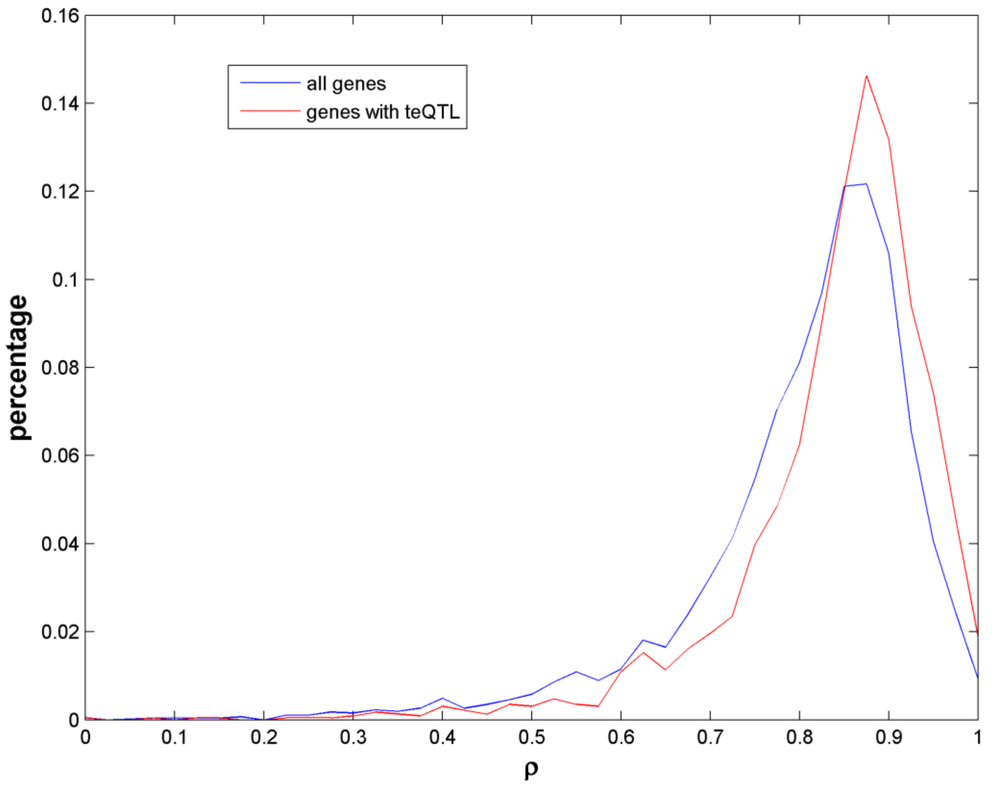

Supplementary Figure 3: Distribution of auto-correlation coefficients ρ for all gene expression traits in the yeast time series data.

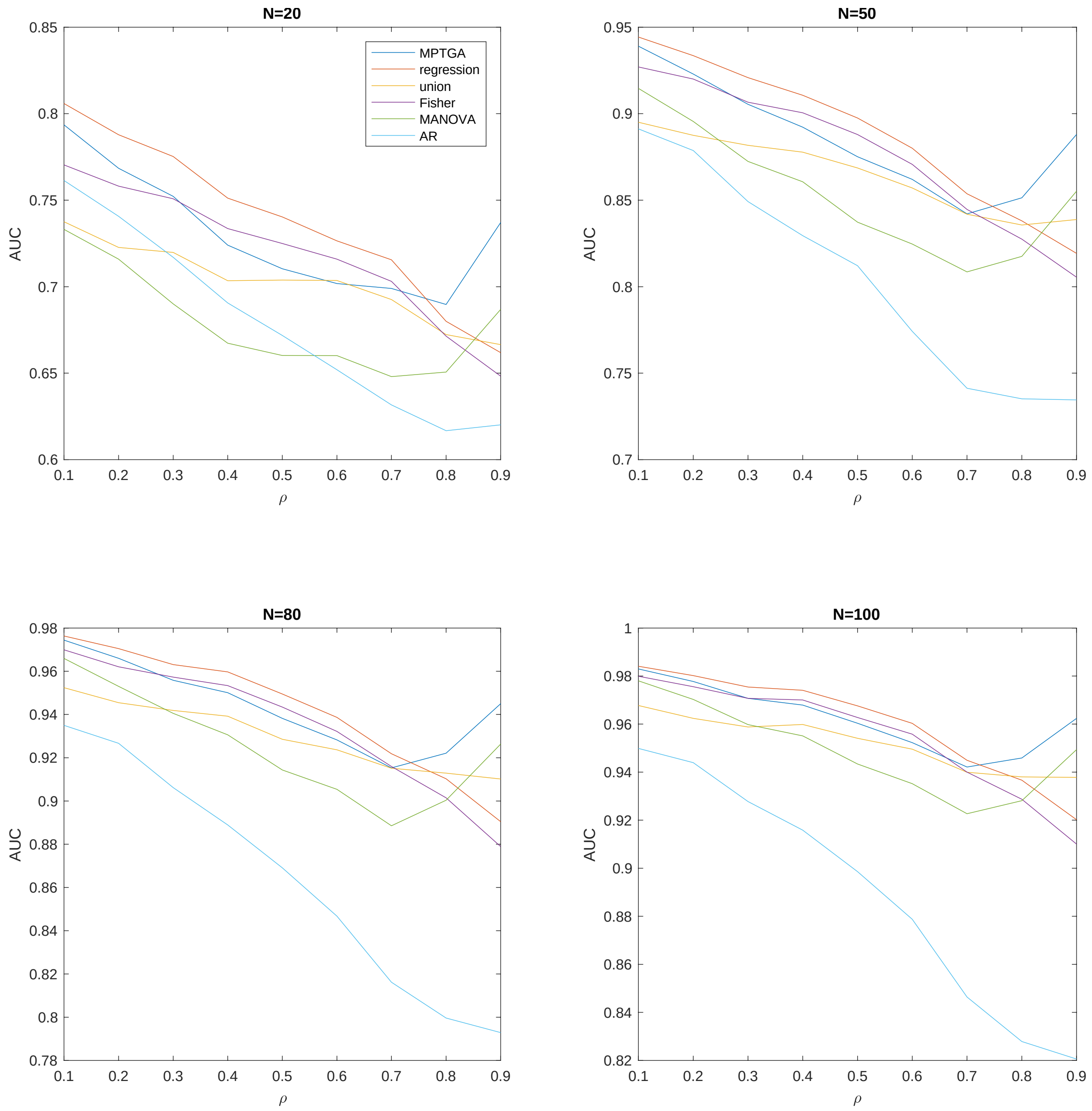

Supplementary Figure 4: Performance comparison among different temporal genetic association methods under different strength of inter time point correlation (auto-correlation) based simulated data. Area under the curve (AUC) was used to assess model accuracy. In each simulated dataset, $\rho_i \sim N(\rho, 0.02)$, $i = 1,2,\cdots,10000$. The comparison is shown for different sample sizes N=20, 50, 80, and 100 to demonstrate the general trend in statistical power of each method.

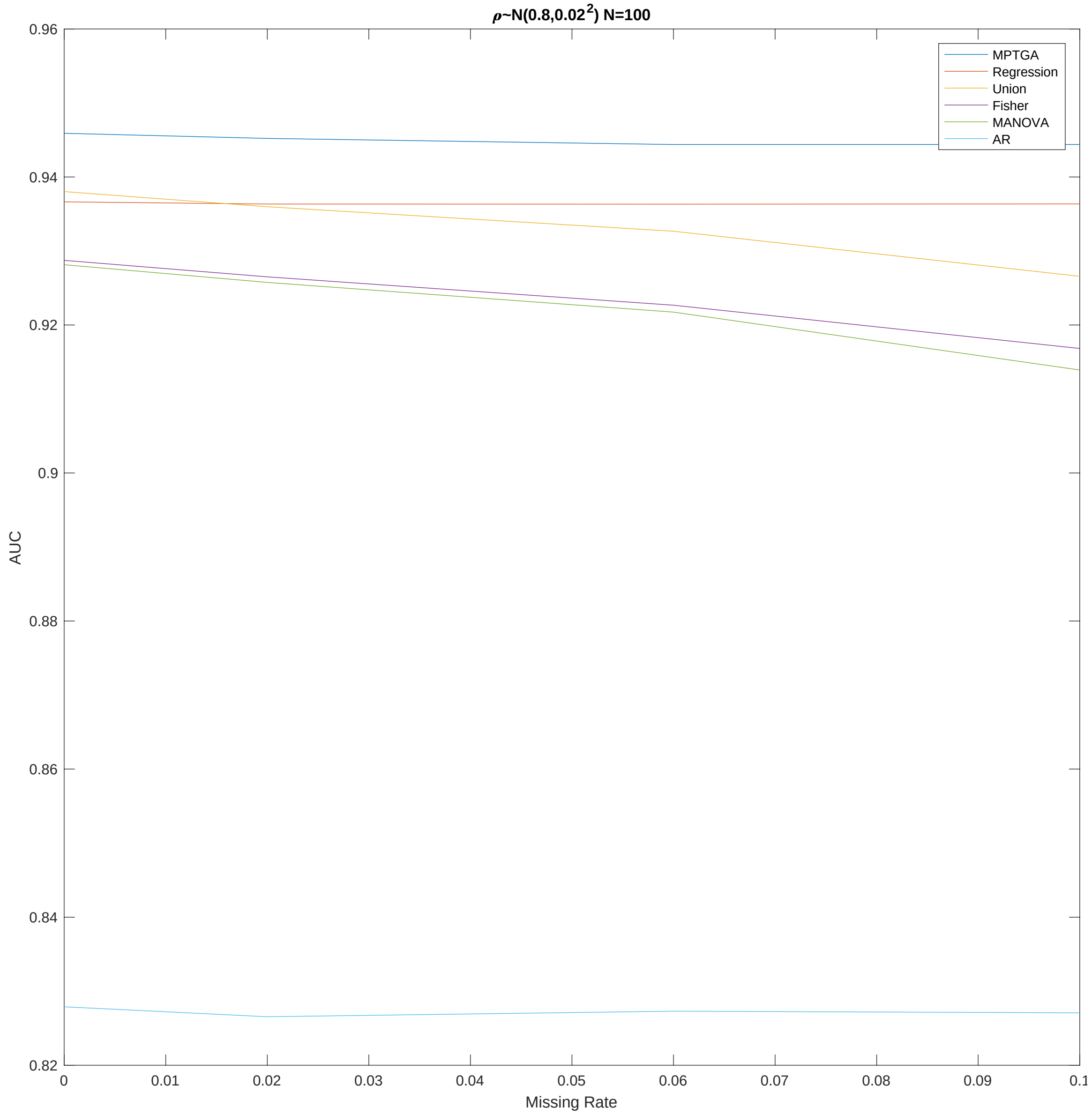

Supplementary Figure 5: Performance comparison of MPTGA and other methods when there are time points randomly dropped from the time series data with missing rate varying from 0.02 to 0.1. For methods that involve fitting a curve to the data within each genotype, i.e. MPTGA, regression and AR, the samples with missing time points were masked first, then each method was performed on the remaining samples (corresponding forms of curves fitted to the remaining data). Next, the missing time points were imputed using the fitted curves. Finally, each method was applied on the imputed data. For the other methods that do not fit curves to the data, i.e. union, Fisher and MANOVA, the samples with missing data were masked first and each method was applied on the remaining data.

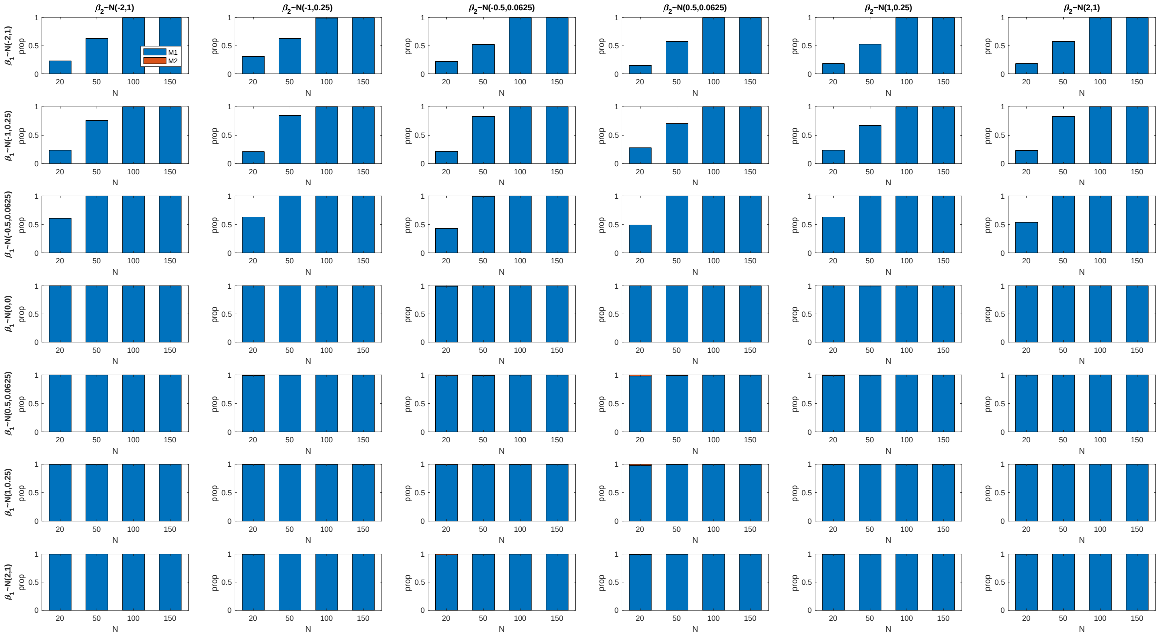

Supplementary Figure 6: Power to distinguish causal model M1(X->Y) and reactive model M2(Y->X) based on simulation according to the causal M1 model. Each bar shows the model selection result of 10,000 datasets simulated with parameters generated from the distribution specified in the corresponding row and column and varying sample sizes N=20,50,100,150. The proportion of each of the selected model is shown with different colors (M1:X->Y, M2:Y->X represented with blue and red bars, respectively). Both the simulated pair of X and Y series in each dataset had to have temporal-genetic associations at the tested locus (MPTGA $p < 10^{-6}$). All simulated time series for Trait X in the 10,000 datasets of each bar passed the p-value threshold for temporal-genetic associations. The total bar height corresponding the number of simulated time series for Trait Y out of each 10,000 datasets simulated was set as following: (1) if more than 100 simulated Y's passed the temporal-genetic association test p-value threshold, the bar height was set to 1; (2) if less than 100 simulated time series for Trait Y passed the temporal-genetic association test p-value threshold, the bar height was set to the number of simulated Trait Y with temporal-genetic associations divided by 100. The proportion of each causality model selected by TGCT is shown proportionally to the total bar height. The average accuracy (proportion of M1 selected) were 0.9954, 0.9982, 0.9995, 0.9997 for N=20, 50, 100, and150, respectively.

Supplementary Figure 7: The distribution of the log likelihood ratio between M1 and M2 based on the simulated data sets specified in Figure S6 for sample size N=20, 50, 100, and 150 (A-D), respectively.

A) sample size N=20:

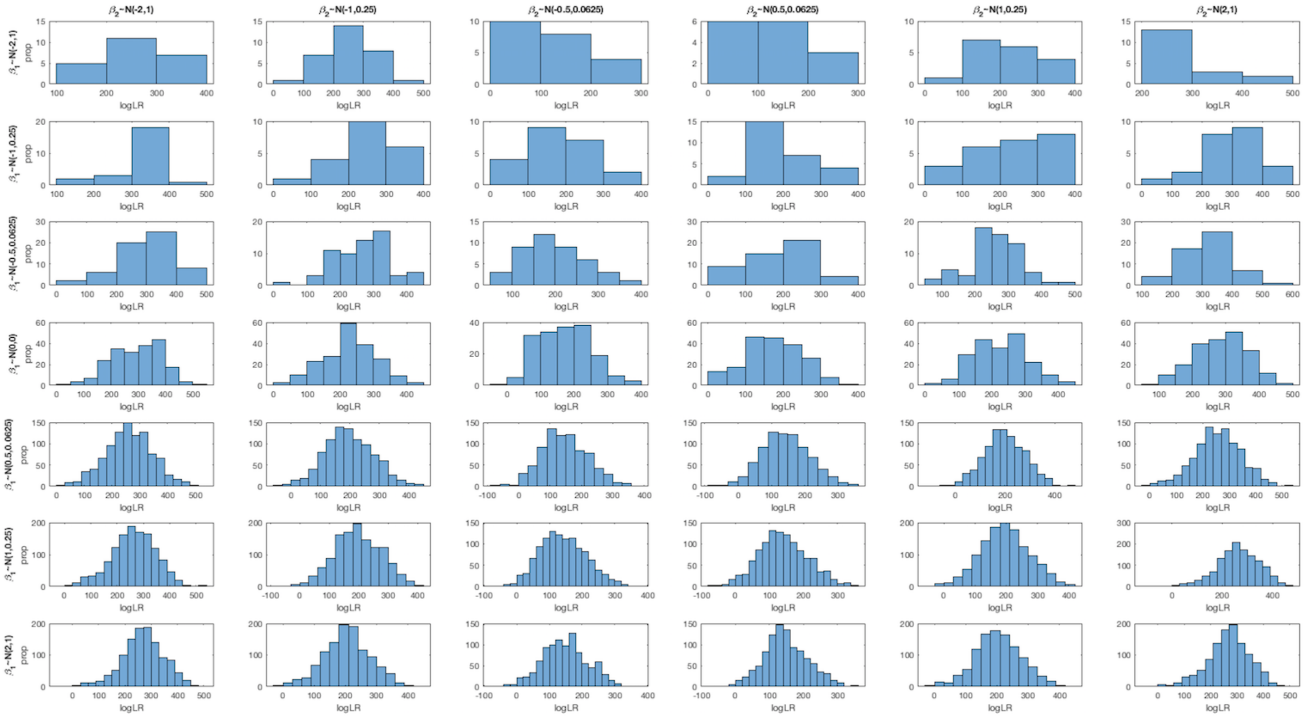

B) sample size N=50:

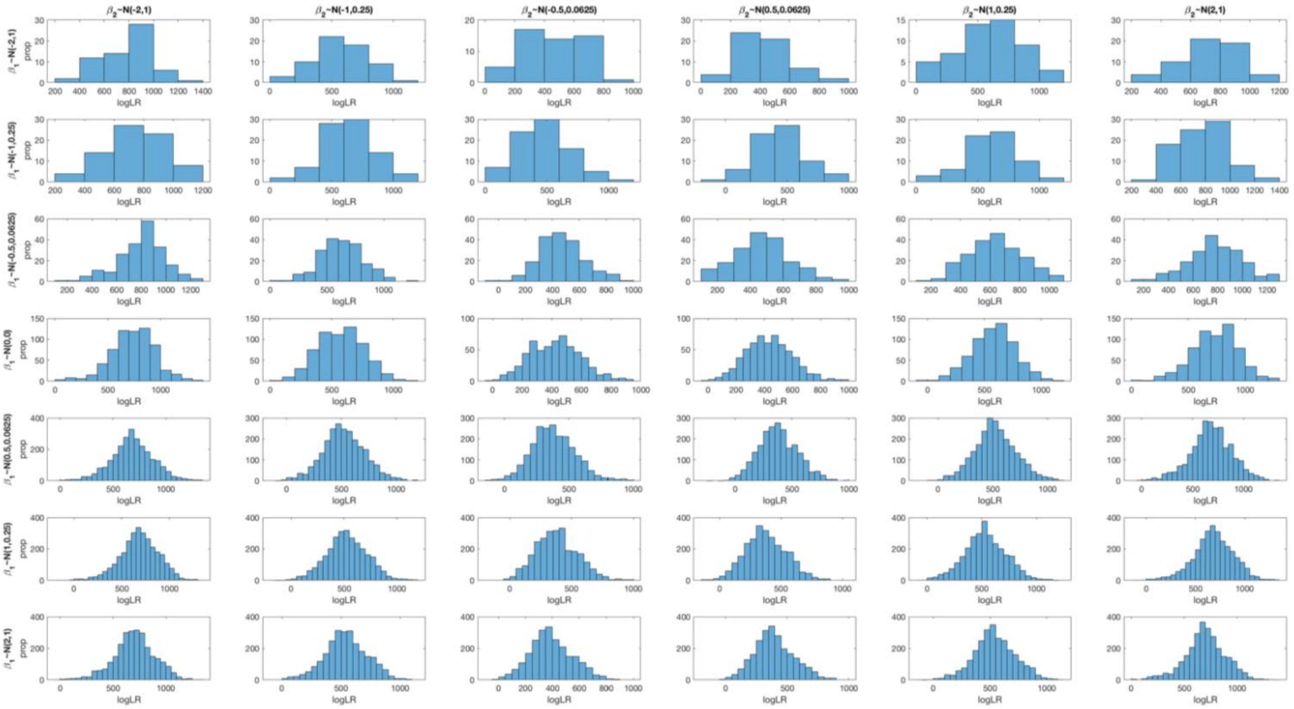

C) sample size N=100:

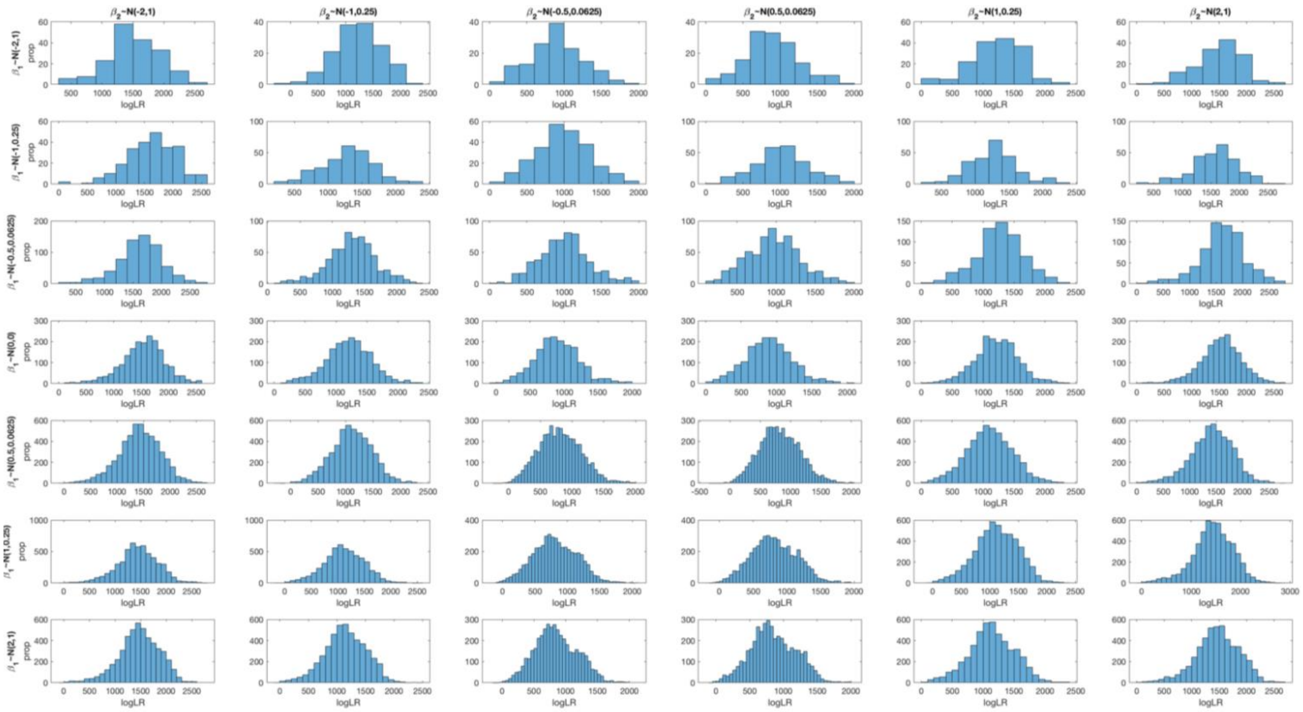

D) sample size N=150.

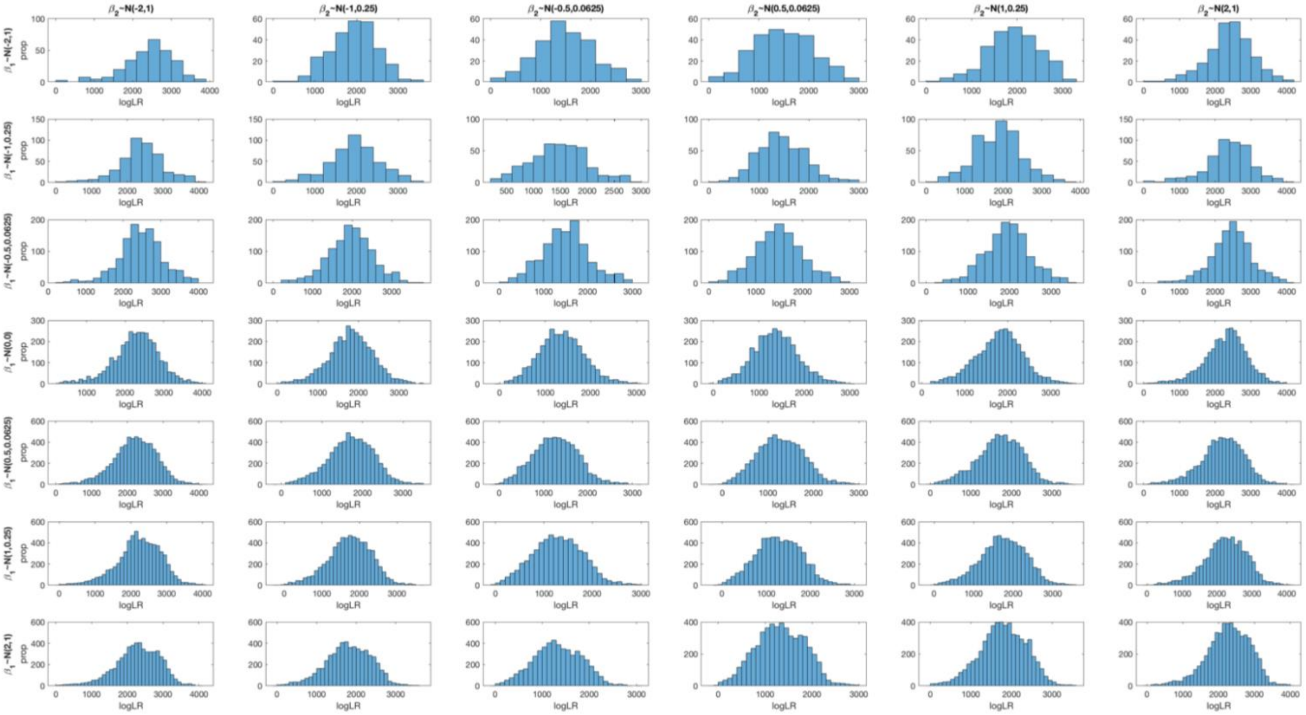

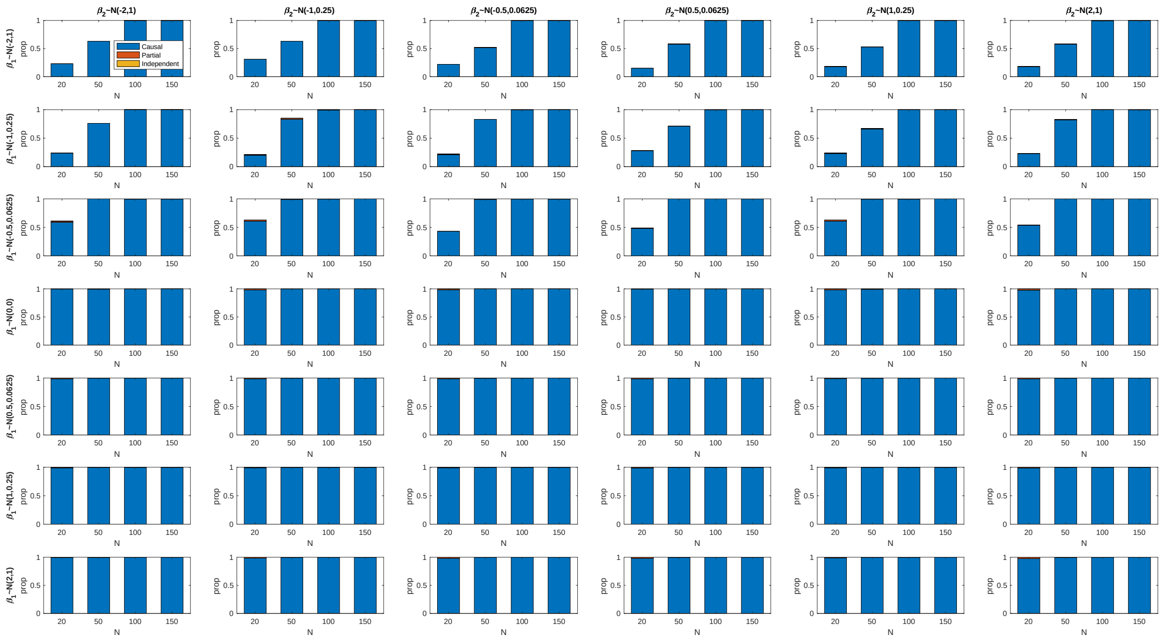

Supplementary Figure 8: Power of the temporal-genetic causality test for data simulated under the causal model. Each bar shows the model selection result of 10,000 datasets simulated under the causal model with parameters generated from the distribution specified in the corresponding row and column and different sample sizes N=20,50,100,150. The proportion of each model selected by TCGT is shown with different colors (causal, partial causal, and independent models represented with blue, red, and yellow bars, respectively). Both traits X and Y in a simulated pair had to pass the temporal-genetic association test with p-value less than 10^{-6} before we applied the temporal-genetic causality test to the pair. All simulated time series for Trait X in the 10,000 datasets passed the p-value threshold for the MPTGA test, the bar height corresponding to the number of simulated time series for Trait Y out of each 10,000 datasets simulated was set as following: (1) if more than 100 simulated time series for Trait Y passed the temporal-genetic association test p-value threshold, the bar height was set to 1; (2) if less than 100 simulated time series for Trait Y passed the temporal-genetic association test p-value threshold, the bar height is set to the number of simulated time series for Y with temporal-genetic associations divided by 100. The proportion of each causality model selected by TGCT is shown proportionally to the total bar height. The average accuracy (proportion of causal model selected) are 0.9852, 0.9958, 0.9979, 0.9987 for N=20, 50, 100, 150, respectively.

Supplementary Figure 9: The distribution of the BIC difference between the causal model and other models based on simulated data used in Figure S8 for different sample sizes N=20, 50, 100, and 150 (A-D, respectively).

A) N=20:

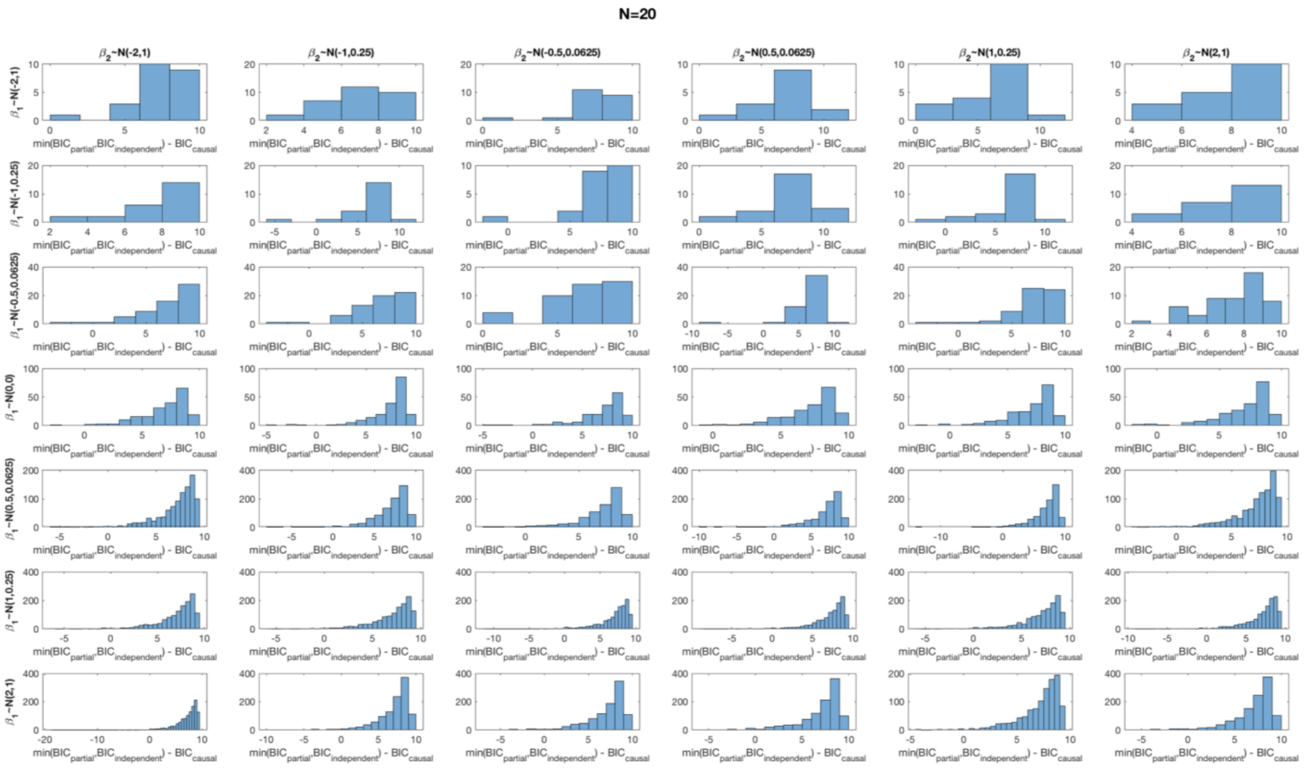

B) N=50.

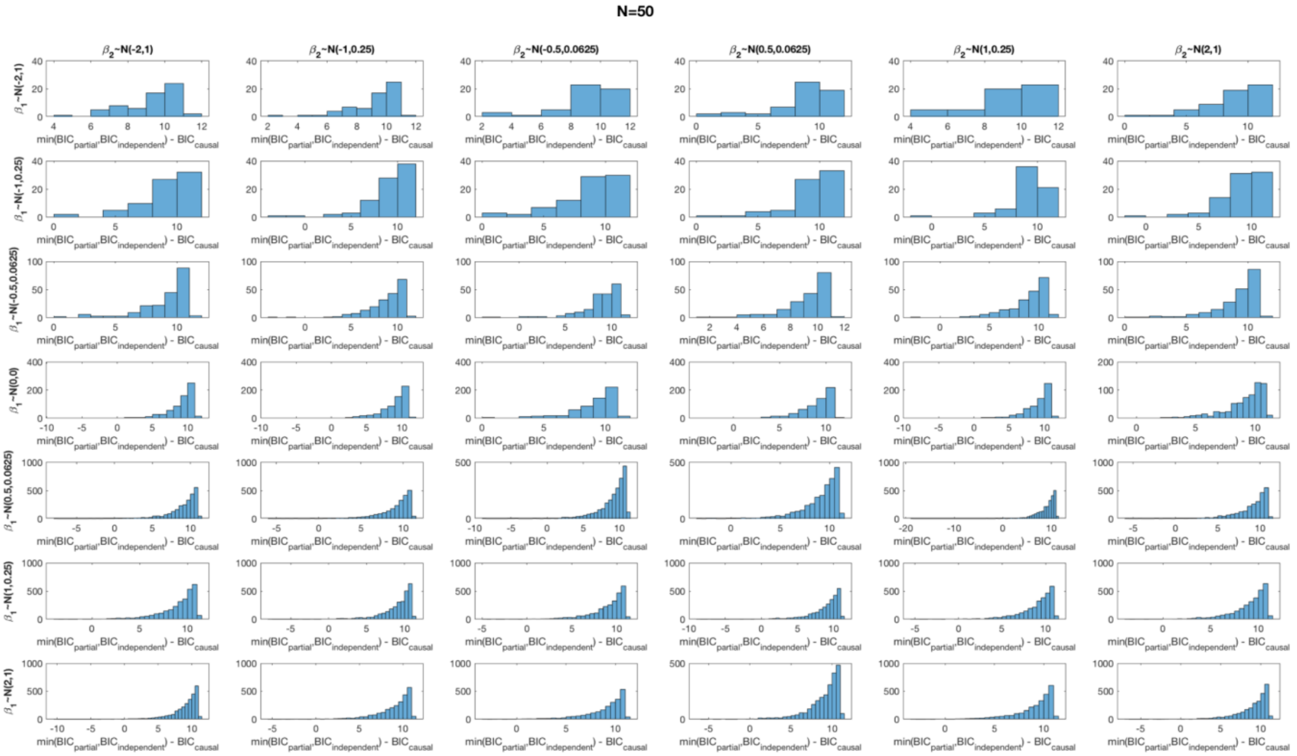
N=50
β2~N(-2,1)
β2~N(-1,0.25)
β2~N(-0.5,0.0625)
β2~N(0.5,0.0625)
β2~N(1,0.25)
β2~N(2,1)
β1~N(-2,1)
β1~N(-1,0.25)
β1~N(-0.5,0.0625)
β1~N(0,0)
β1~N(0.5,0.0625)
β1~N(1,0.25)
β1~N(2,1)
min(BICpartial,BICindependent) - BICcausal

C) N=100.

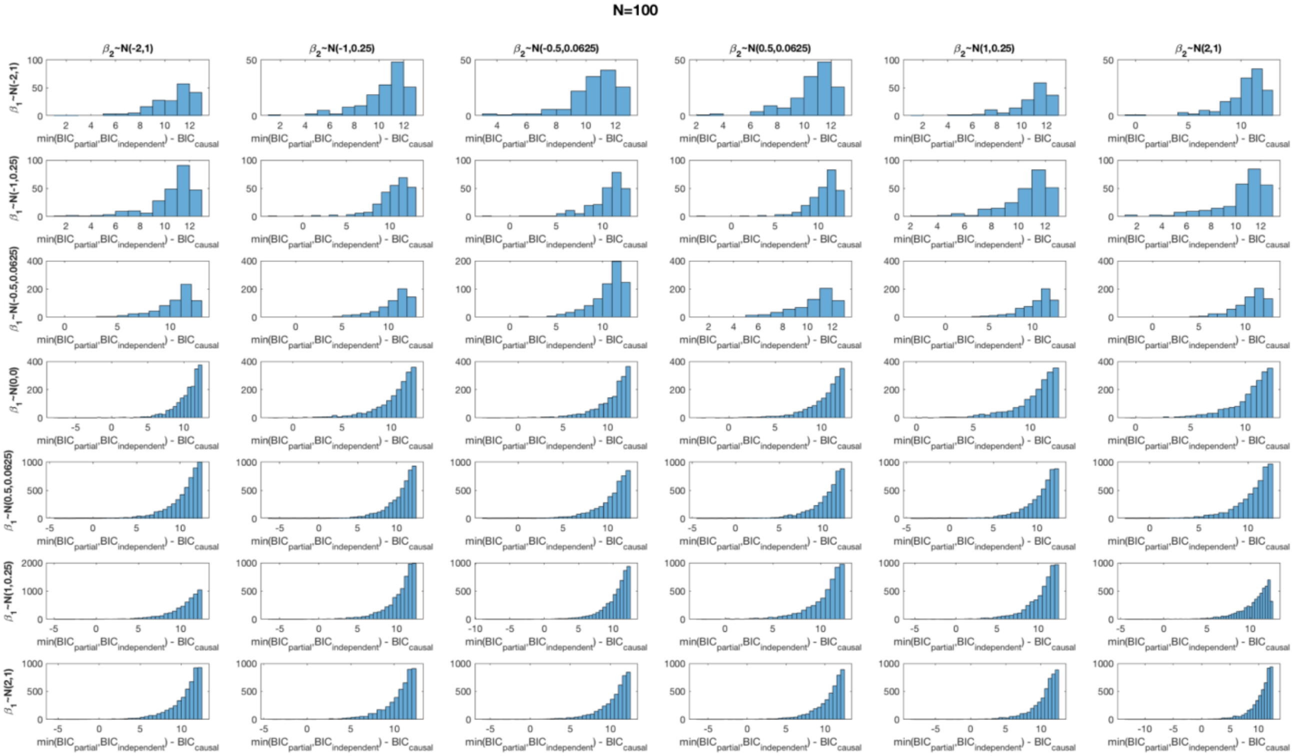
N=100
β2~N(-2,1)
β2~N(-1,0.25)
β2~N(-0.5,0.0625)
β2~N(0.5,0.0625)
β2~N(1,0.25)
β2~N(2,1)
β1~N(-2,1)
β1~N(-1,0.25)
β1~N(-0.5,0.0625)
β1~N(0,0)
β1~N(0.5,0.0625)
β1~N(1,0.25)
β1~N(2,1)
min(BICpartial,BICindependent) - BICcausal

D) N=150.

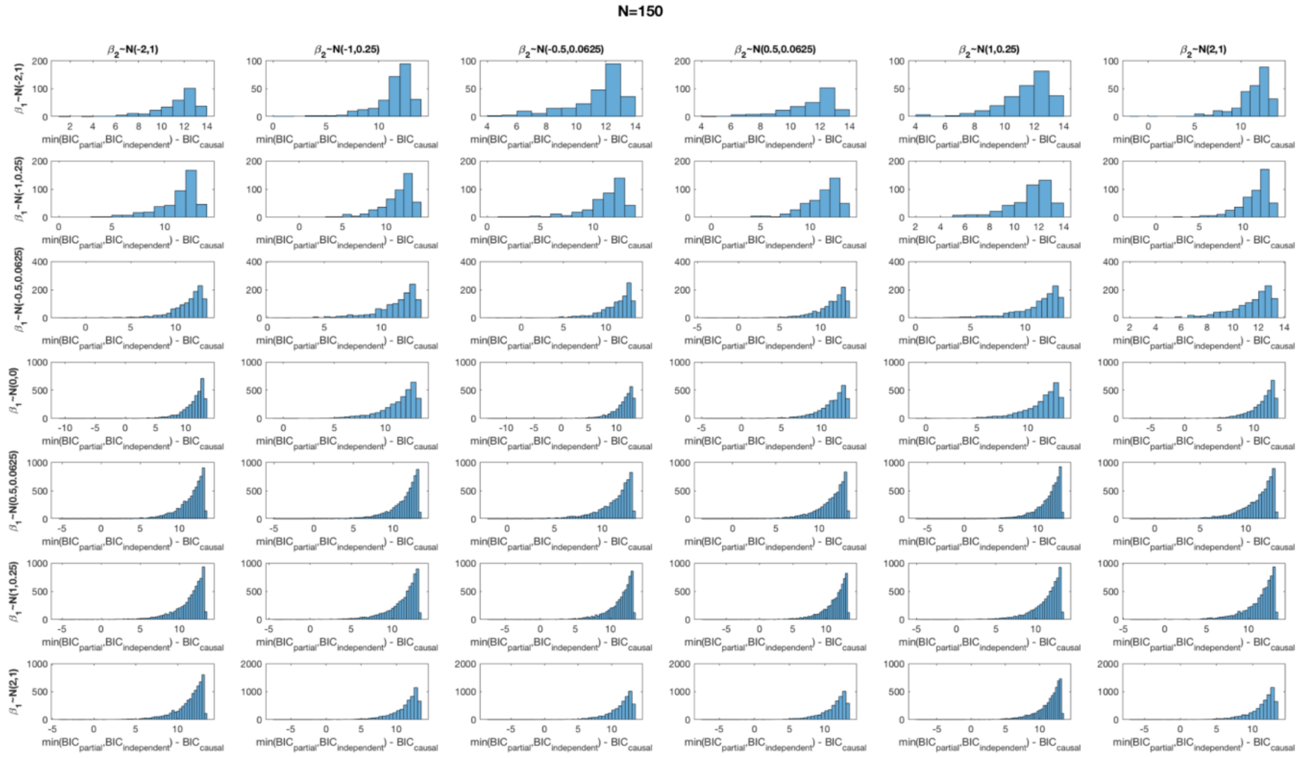
N=150
β2~N(-2,1)
β2~N(-1,0.25)
β2~N(-0.5,0.0625)
β2~N(0.5,0.0625)
β2~N(1,0.25)
β2~N(2,1)
β1~N(-2,1)
β1~N(-1,0.25)
β1~N(-0.5,0.0625)
β1~N(0,0)
β1~N(0.5,0.0625)
β1~N(1,0.25)
β1~N(2,1)
min(BICpartial,BICindependent) - BICcausal

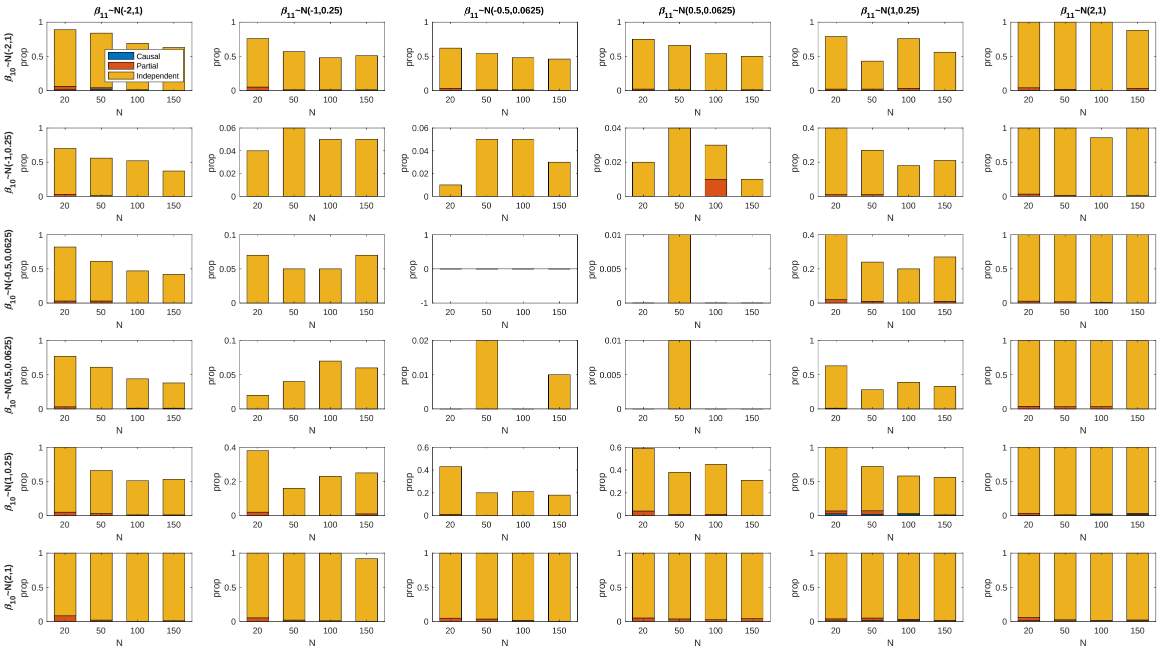

Supplementary Figure 10: Power of the temporal-genetic causality test on data simulated under the independent model. Each bar shows the model selection result of 10,000 datasets simulated with parameters generated from the distribution specified in the corresponding row and column and varying sample sizes N=20,50,100,150. The proportion of each model selected by TCGT is shown with different colors (causal, partial causal, and independent models represented with blue, red, and yellow bars, respectively). Both Traits X and Y in a simulated time series in each dataset had to pass the temporal-genetic association test with p-value less than 10^{-6} before we applied the temporal-genetic causality test on the pair. All simulated time series for X in the 10,000 datasets of each bar passed the p-value threshold, the total bar height corresponding to the number of simulated time series for Y out of each 10,000 datasets was set as the following: (1) if more than 100 simulated time series for Y passed the temporal-genetic association test p-value threshold, the bar height was set to 1; (2) if less than 100 simulated time series for Y passed the temporal-genetic association test p-value threshold, the bar height was set to the number of time series for Y with temporal-genetic associations divided by 100. The proportion of each model selected by TGCT is shown proportionally to the total bar height. The average accuracy (proportion of independent model selected) were 0.9549, 0.9718, 0.9841, and 0.9869 for N=20, 50, 100, and 150, respectively.

Supplementary Figure 11: Power of the temporal-genetic causality test on data simulated under the partial model. Each bar shows the model selection result of 10,000 datasets simulated with parameters generated from the distribution specified in the corresponding row and column and varying sample sizes N=20,50,100, and 150. The proportion of each model selected by TCGT is shown with different colors (causal, partial causal, and independent models represented with blue, red, and yellow bars, respectively). Both traits X and Y in a simulated pair in each dataset had to pass the temporal-genetic association test with p-value less than 10^{-6} before we applied the temporal-genetic causality test on the pair. All simulated time series for X in the 10,000 data sets of each bar passed the p-value threshold, the total bar height corresponding to the number of simulated time series for Y out of each 10,000 datasets simulated was set as the following: (1) if more than 100 simulated time series of Y passed the temporal-genetic association test p-value threshold, the bar height was set to 1; (2) if less than 100 simulated times series of Y passed the temporal-genetic association test p-value threshold, the bar height was set to the number of time series of Y with temporal-genetic associations divided by 100. The proportion of each causality model selected by TGCT is shown proportionally to the total bar height. The average accuracy (proportion of partial model selected, excluding the parameter settings that reduce to the causal or independent model) are 0.9980, 0.9987, 0.9988, 0.9989 for N=20, 50, 100, and 150, respectively. The model selection results for 7 sets of parameters and different β_{10} distributions are shown in A-G.

A) $\beta_{10} \sim N(-2,1)$.

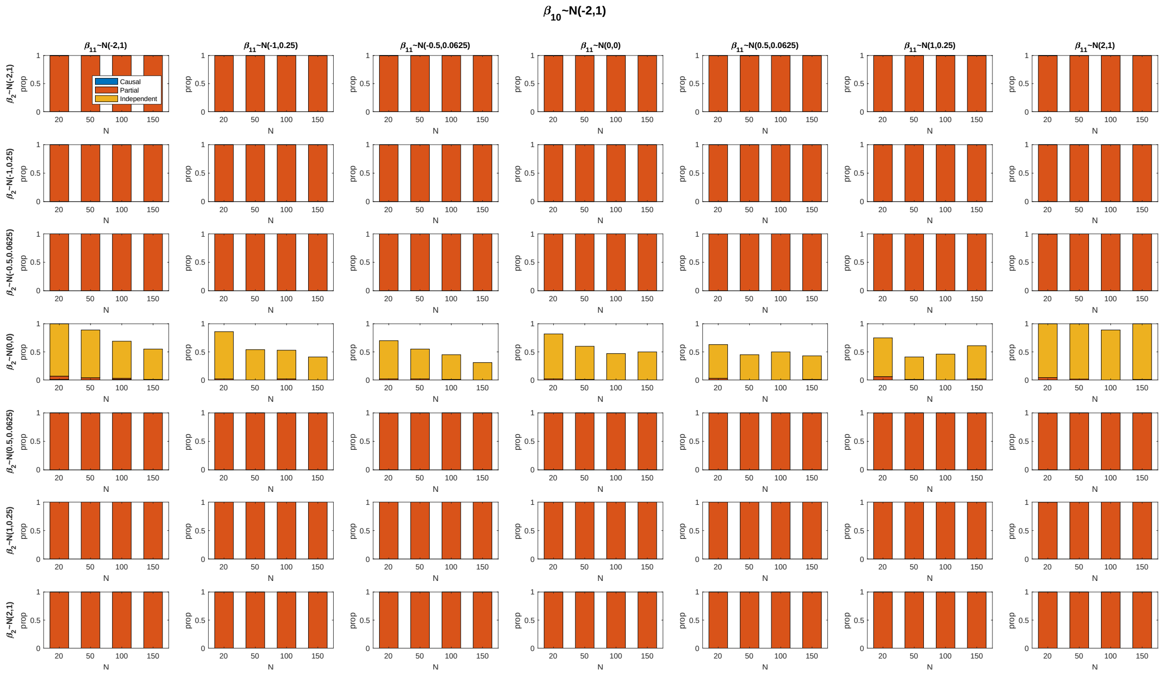

B) $\beta_{10} \sim N(-1,0.25)$.

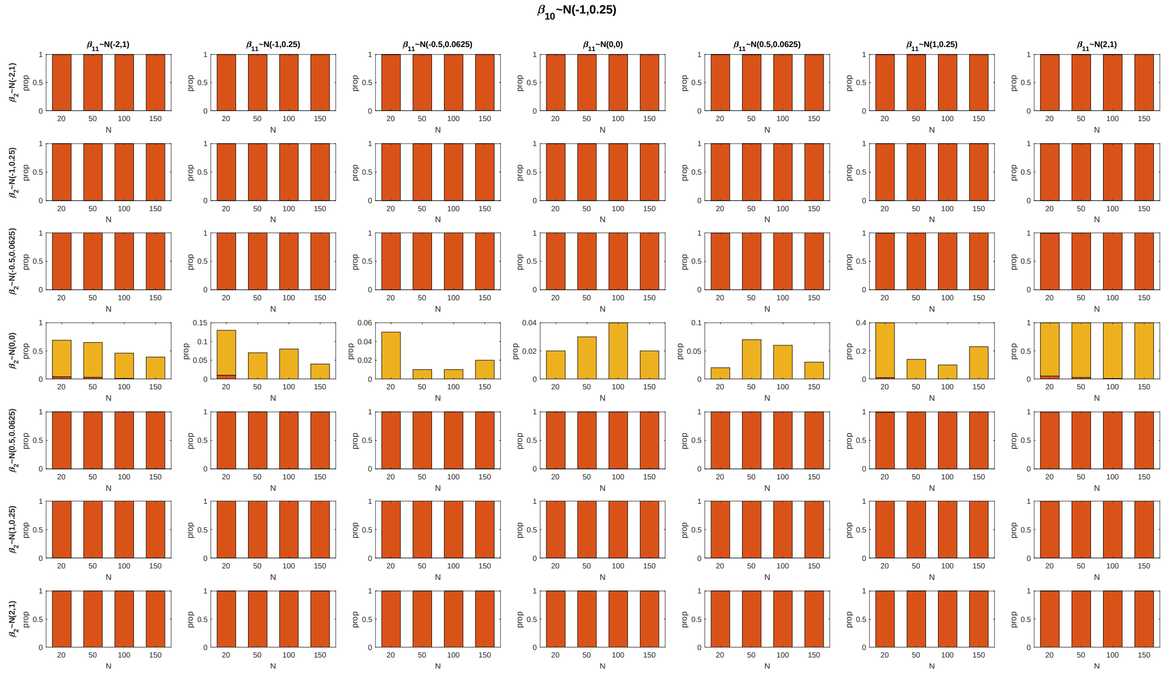

C) $\beta_{10} \sim N(-0.5,0.0625)$.

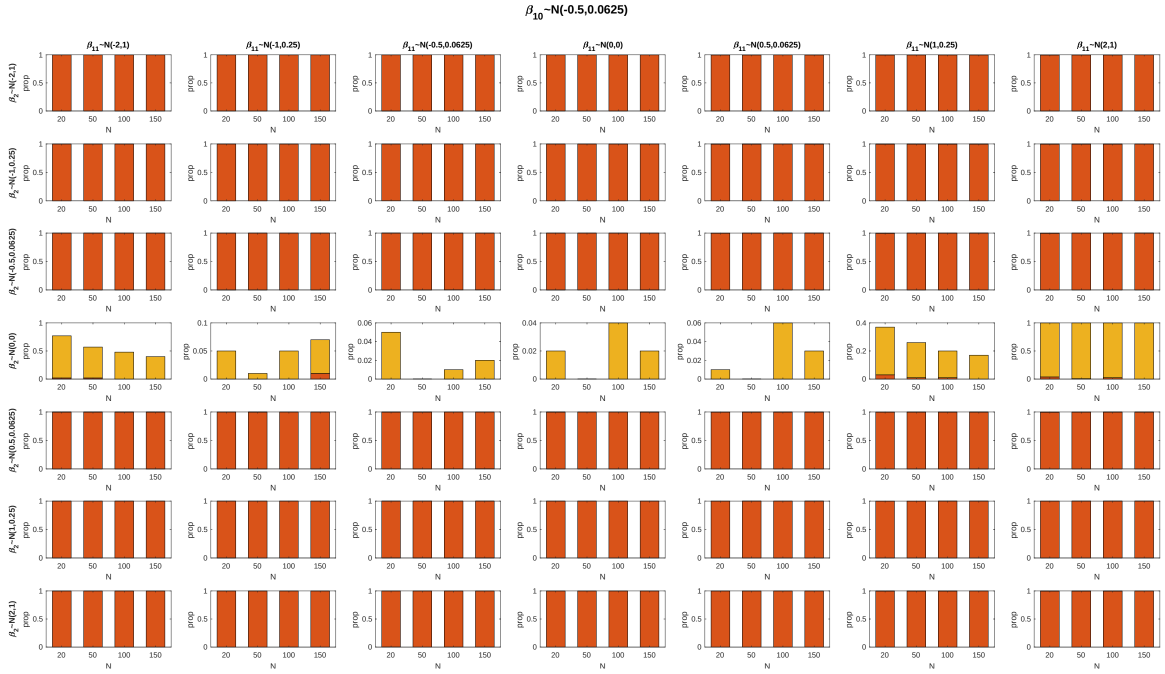

D) $\beta_{10} = 0.$

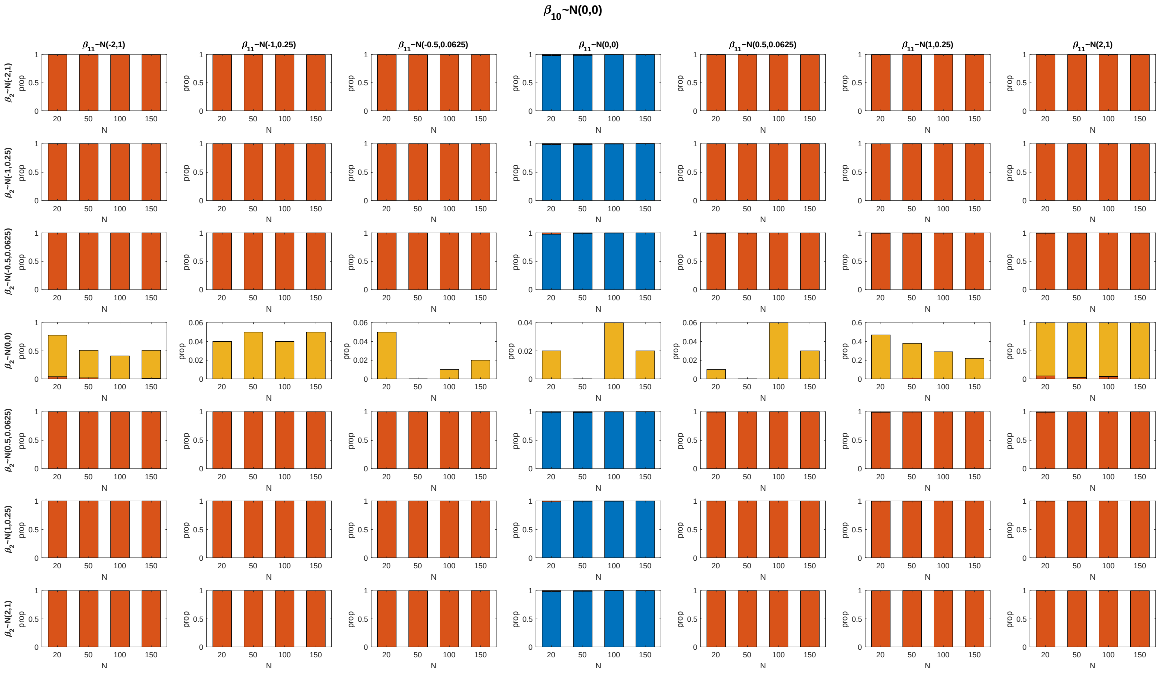

E) $\beta_{10} \sim N(0.5, 0.0625).$

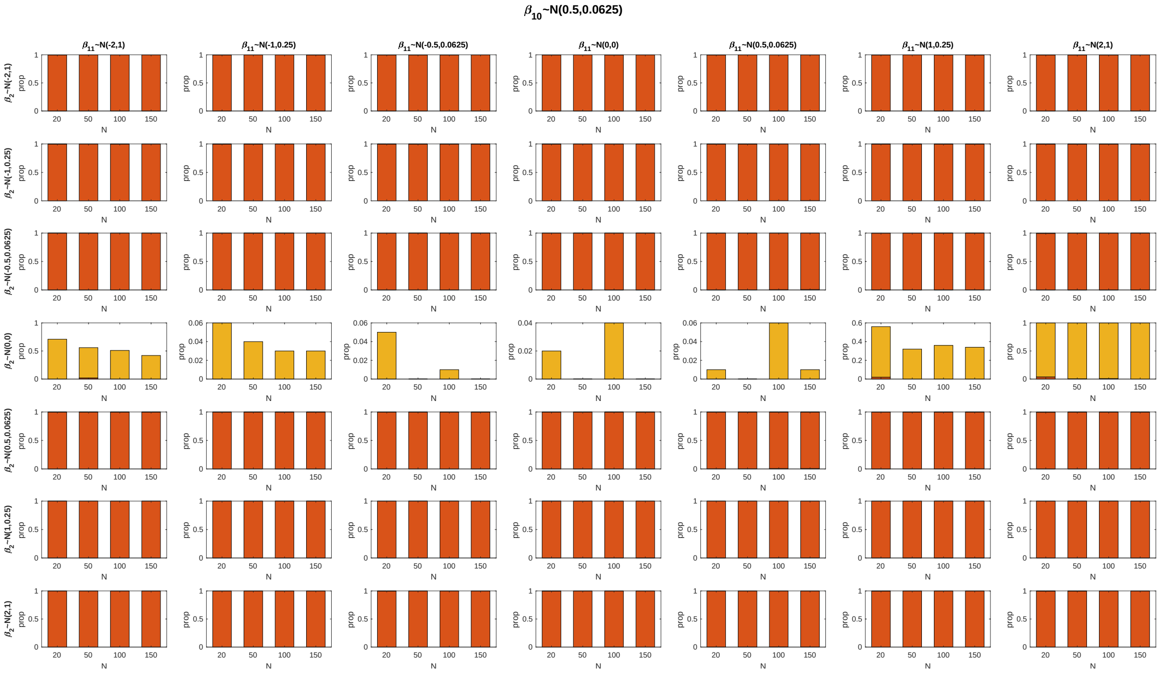

F) $\beta_{10} \sim N(1,0.25)$.

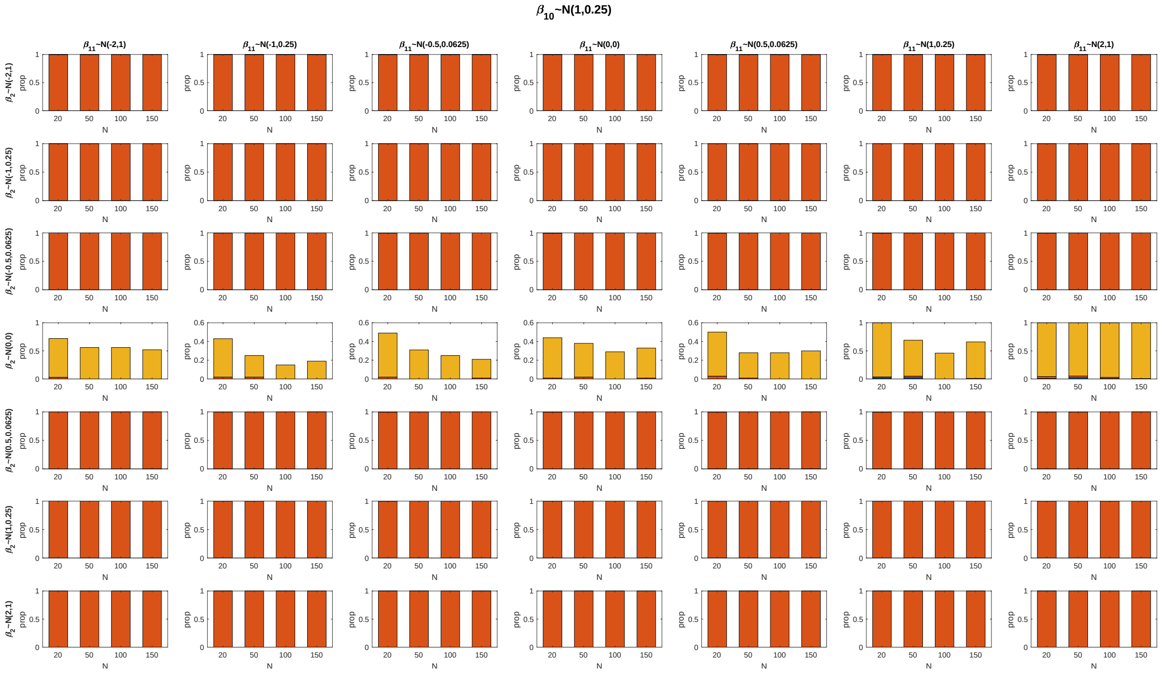

G) $\beta_{10} \sim N(2,1)$.

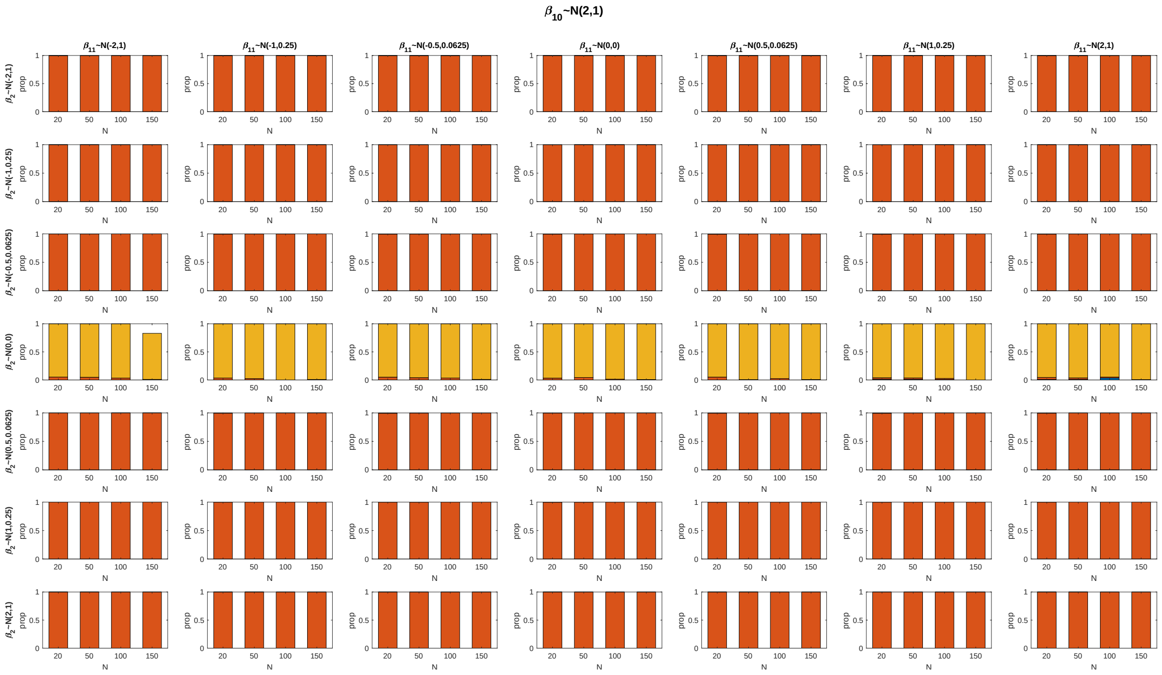

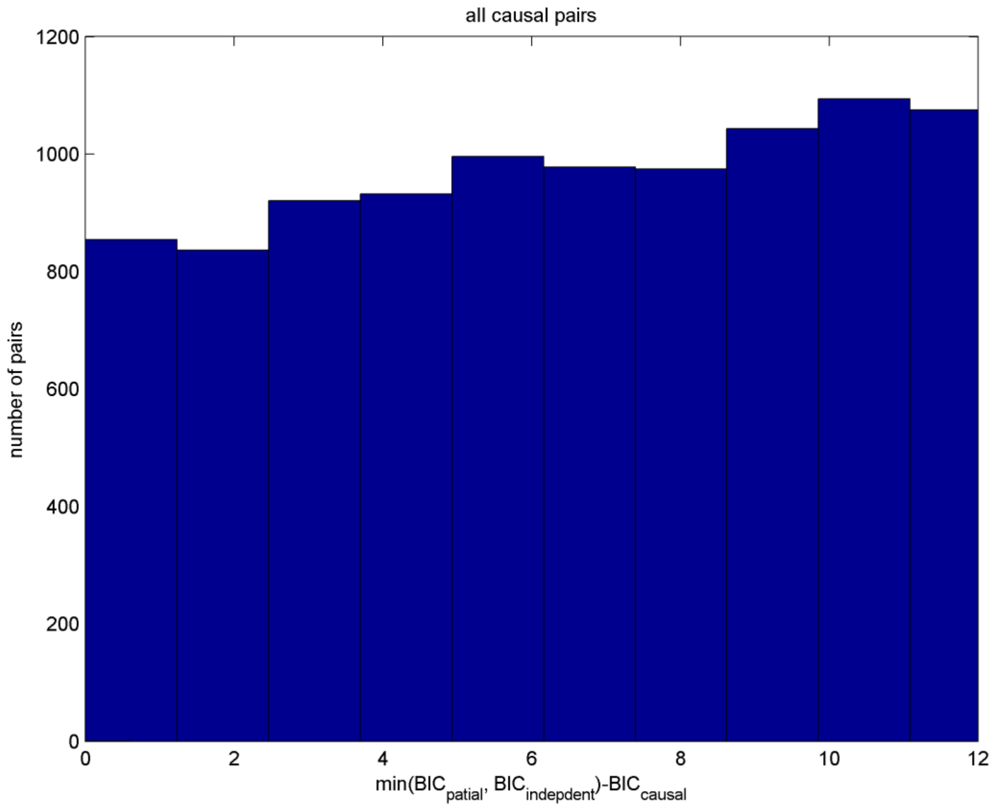

Supplementary Figure 12 The distribution of the BIC difference between the causal model and other models based on yeast F2 data for all causal pairs in Supplementary Table 11.

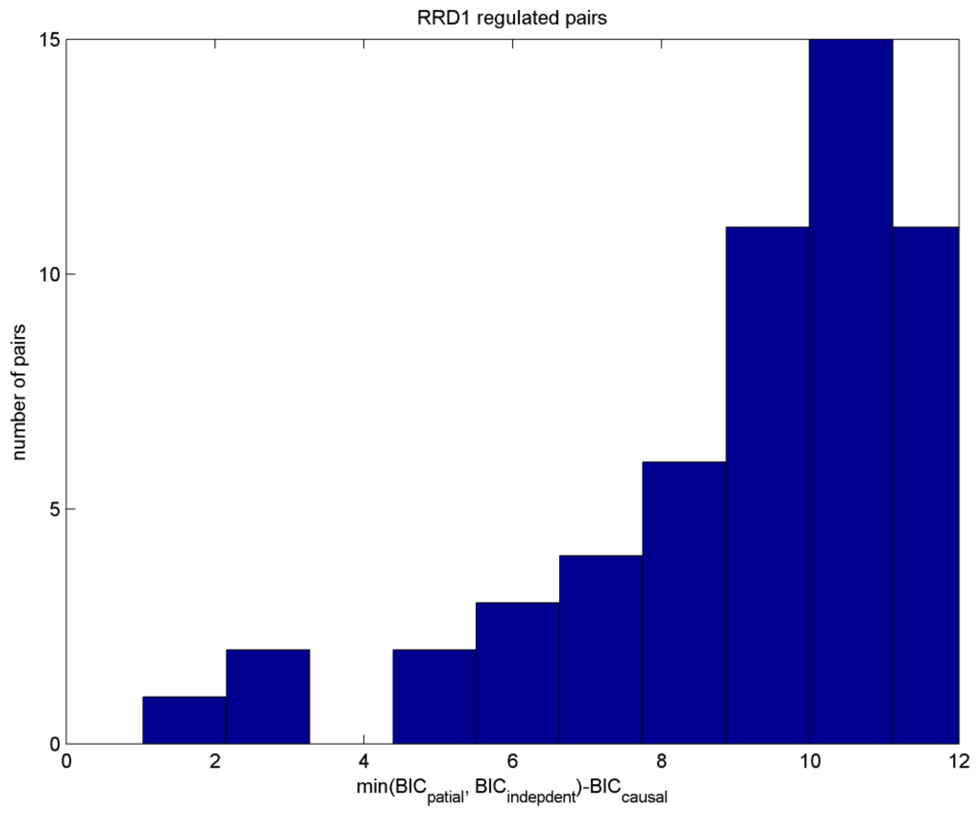

Supplementary Figure 13 The distribution of the BIC difference between the causal model and other models based on yeast F2 data for all causal pairs with *RRD1* as a causal regulator.

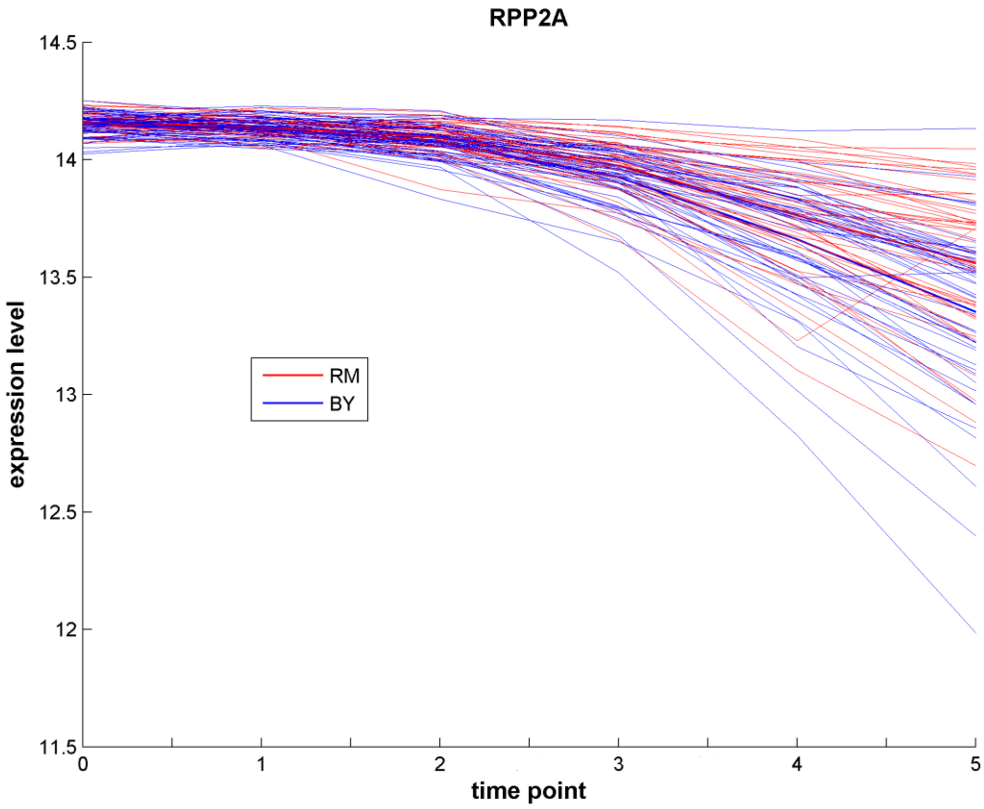

Supplementary Figure 14: Individual expression pattern of RPP2A which was linked to the teQTL hot spot chrIX:70,000.

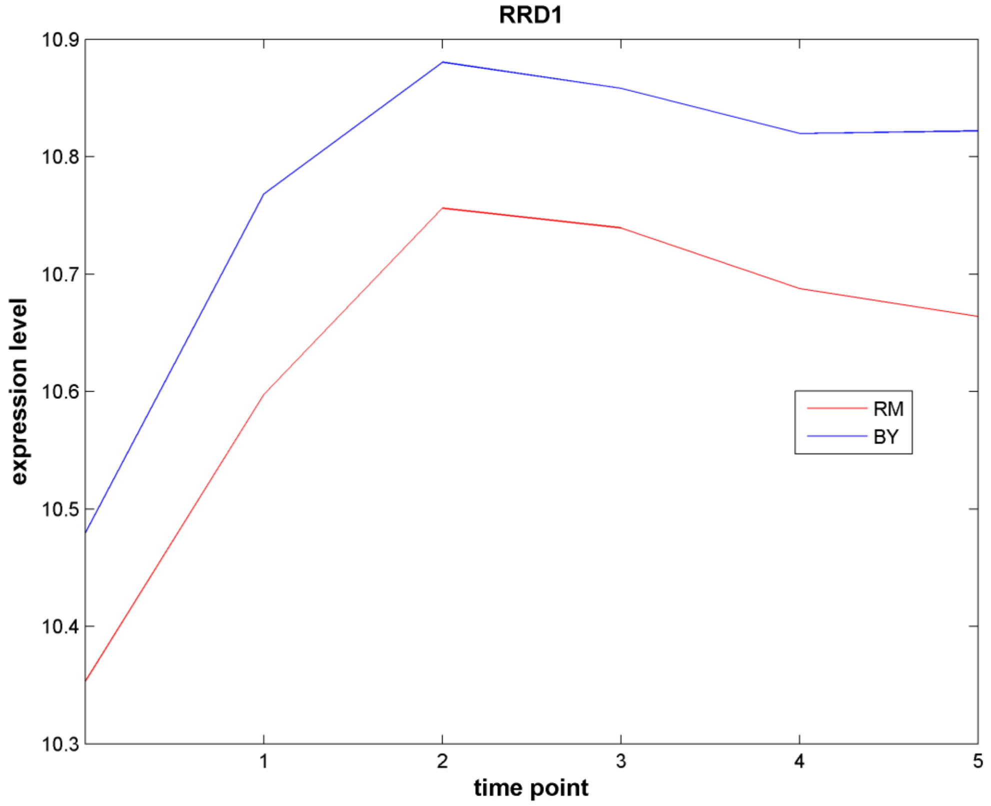

Supplementary Figure 15 Average *RRD1* expression levels for segregants carrying RM and BY alleles at *RRD1* locus.

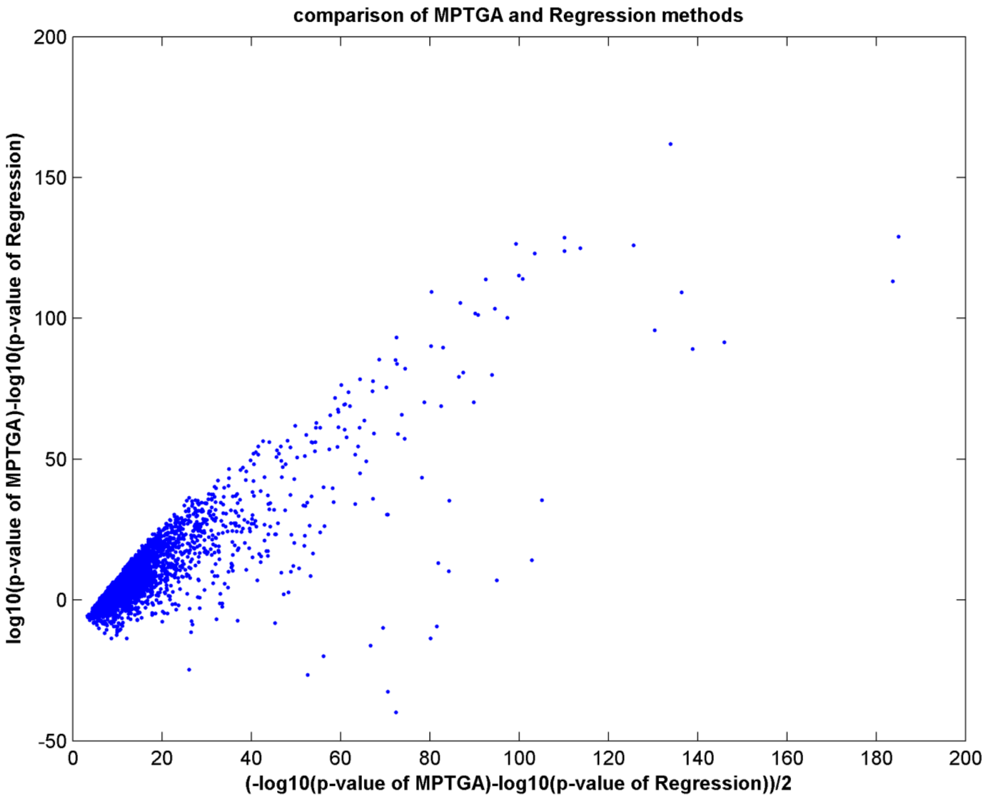

Supplementary Figure 16: Comparison all 2865 teQTLs linked to the hot spots defined by the MPTGA method (Figure 3B) with regard to p-values based on the MPTGA and the regression methods (the average of -1og$_{10}$(p-values) vs. the –log$_{10}$(p-value) difference). The p-values based on the regression method were more significant than the ones based on the MPTGA in general, but there were 22% (624/2865) of all teQTLs linked to the hot spots whose p-values based on the MPTGA were more significant.

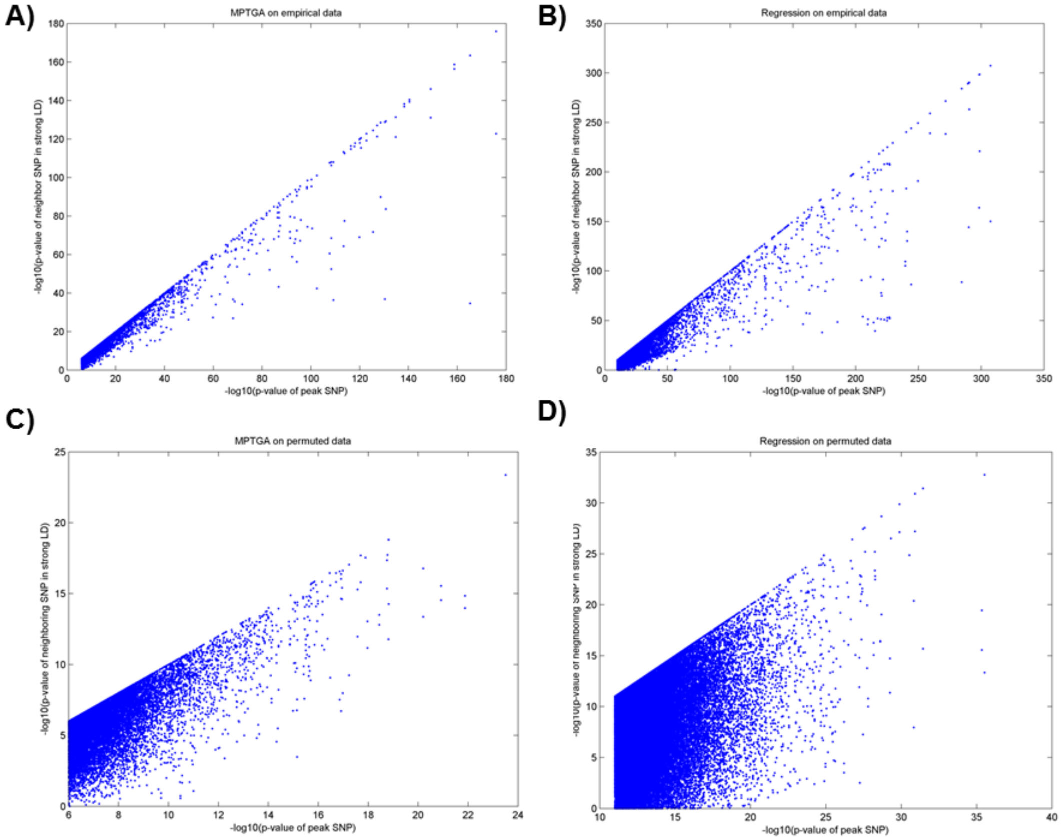

Supplementary Figure 17. Assessing overfitting problem by comparing association p-values of peak SNPs and neighboring SNPs in strong LD (the Fisher's Exact test $p < 10^{-15}$). A) result of the MPTGA on the yeast time series data; B) result of the regression method on the yeast time series; C) result of the MPTGA on permuted data; D) result of the regression method on permuted data. Only associations of peak SNP p-values less than 10^{-6} and 10^{-11} for the MPTGA and the regression method, respectively, were used for this analysis.

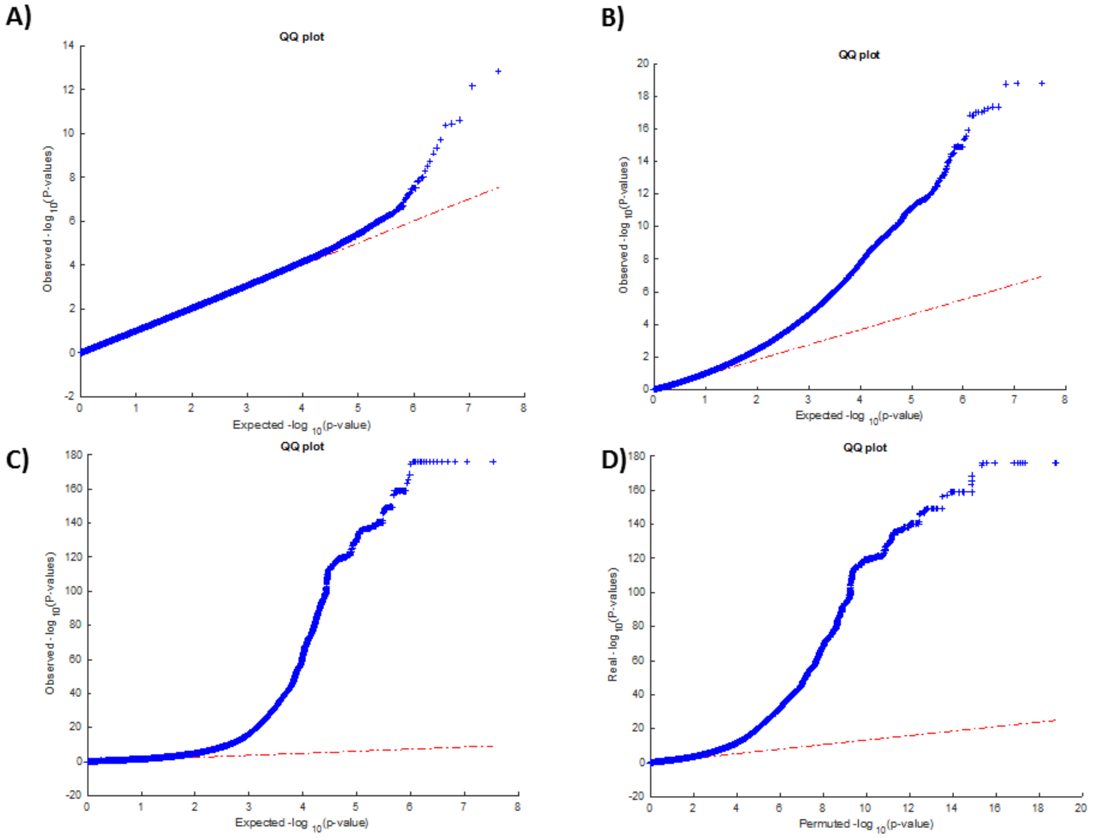

Supplementary Figure 18 QQ plots of the p-values of the MPTGA test. **A)** Simulated data; **B)** permuted data; **C)** real data; **D)** p-values for real data v.s. permuted data.

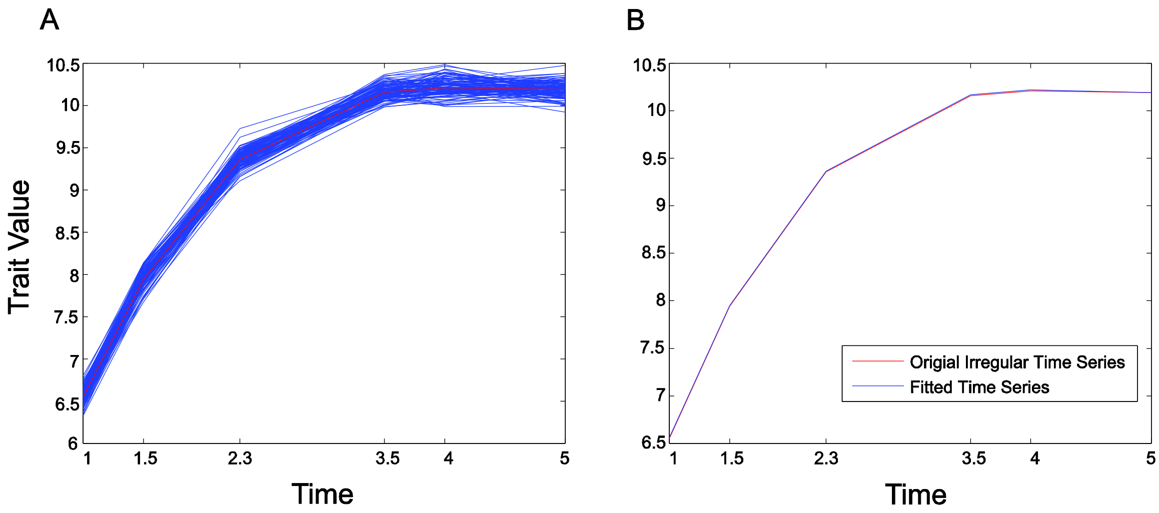

Supplementary Figure 19. Time series data with irregular time intervals. A) Given a polynomial function (red line), 100 traits were simulated by sampling 5 time points at random intervals. B) The simulated traits were fit to a cubic polynomial function which matched with the pattern underlying the simulated traits.

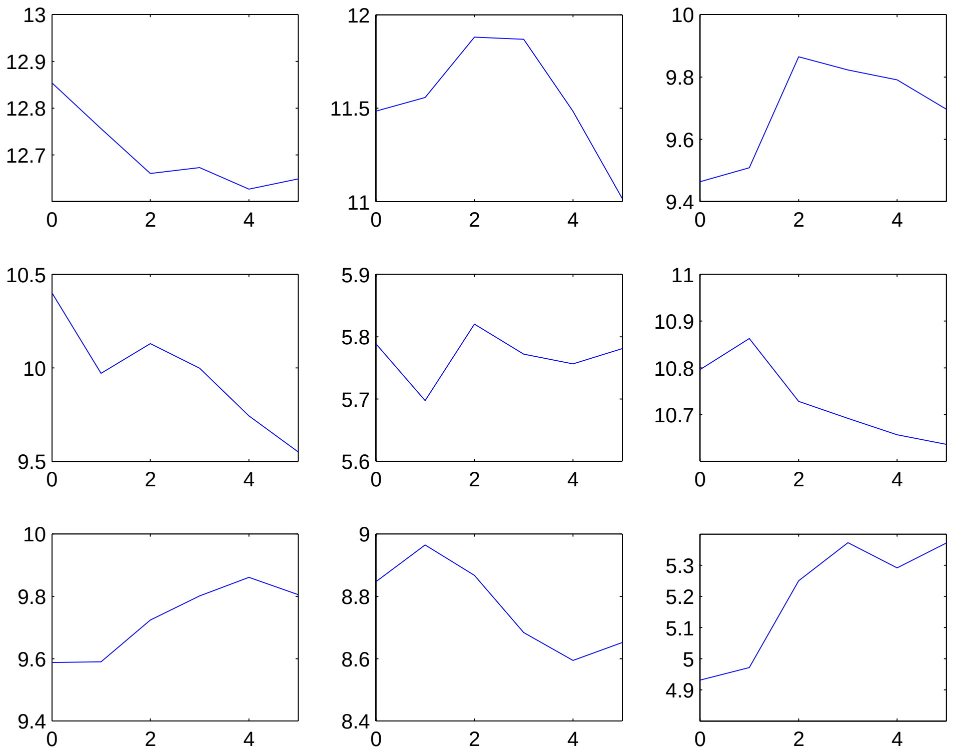

Supplementary Figure 20: Gene expression examples in the yeast time series data showing insufficient power of linear or quadratic polynomial fitting.

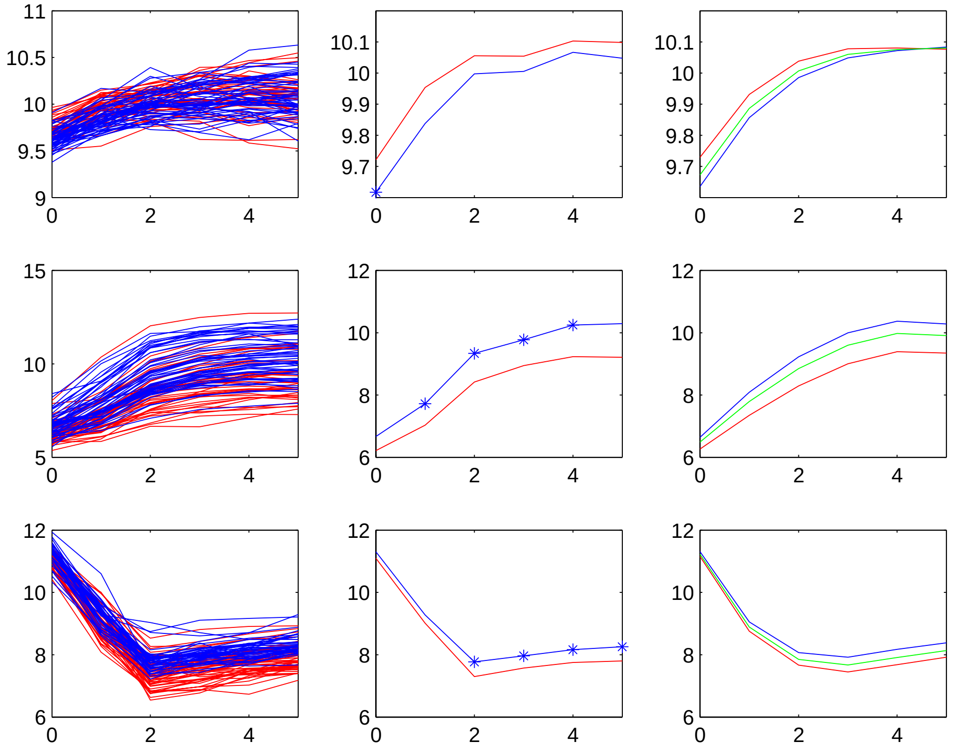

Supplementary Figure 21: Pattern fitting examples. The first column is the plots of all expression time series, in which segregants with genotype 0 is plotted in blue and segregants with genotype 1 is plotted in red; the second column plots the median value at each time point for segregants with different genotypes, and the star denotes that the expression data is declared significantly different at corresponding time point; The third column is the fitting by MPTGA, in addition to separate fitting for different genotypes, the green curve is the fitting for all segregants.

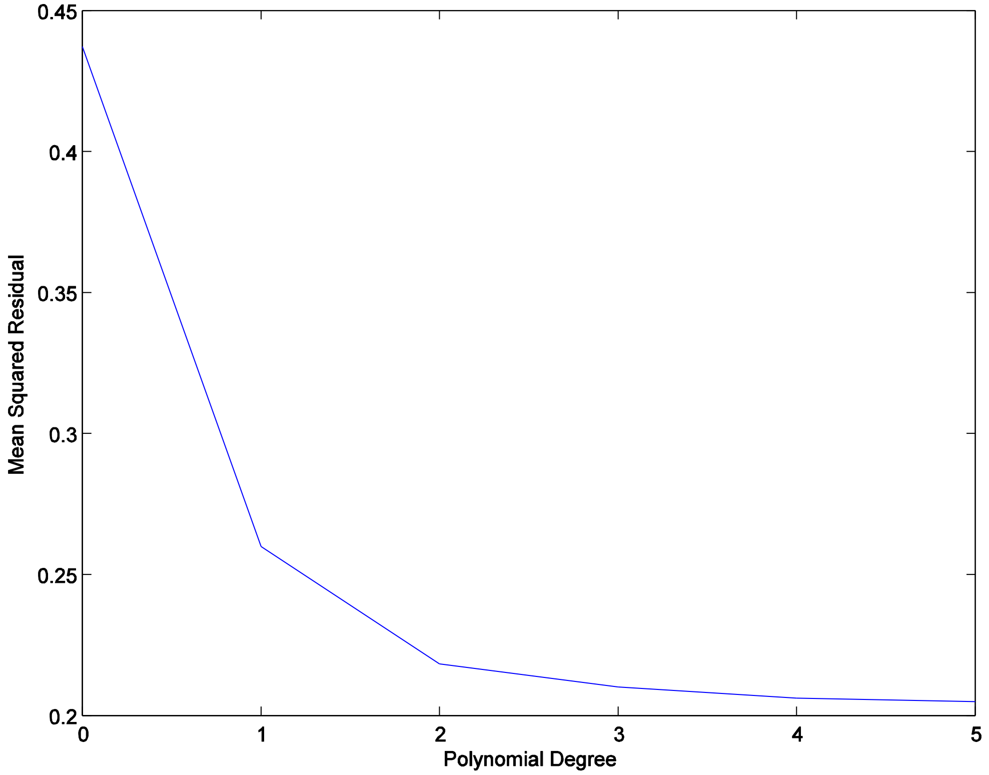

Supplementary Figure 22: Goodness of fit using different degree of polynomial function for fitting the yeast time series data set. For degree 3 or higher, the mean squared residuals are similar, suggesting a cubic polynomial function is sufficient for fitting the dataset.

Supplementary Tables

Supplementary Table 1: eQTLs based on static data (T0)

Accession ID	QTL chrom:position	p-value
YKR009C	16:492351	3.41E-05
YDL157C	4:165026	9.03E-08
YDR481C	13:33501	7.85E-14
YIL157C	15:113254	2.61E-06
YNR074C	12:662627	2.48E-05
YKL012W	4:1185630	5.14E-07
YJL147C	2:555596	3.77E-05
YJL147C	10:123859	4.51E-12
YJL147C	16:492351	2.99E-05
YDL216C	4:70901	1.43E-16
YPL223C	15:174364	1.74E-06
YKL138C	15:154177	6.87E-06
YNL126W	2:592863	1.40E-05
YML035C	13:33681	7.75E-06
YLR050C	12:238298	6.05E-15
YOL008W	15:298710	7.28E-05
YOR072W-B	14:558284	1.03E-10
YDL048C	9:139512	8.82E-07
YDL048C	15:136327	1.73E-06
YDR056C	2:537314	4.66E-05
YDR056C	12:679808	0.00014733
YNL216W	14:246135	7.48E-13
YFL034C-B	2:548401	6.25E-05
YGL222C	7:85112	6.61E-16
YIR021W	15:170945	1.42E-05
YHR014W	8:128732	1.52E-08
YDL029W	14:449639	7.58E-12
YOR192C-C	14:591237	6.62E-06
YOR192C-C	15:703771	1.74E-12
YKL195W	15:174364	6.41E-06
YDR144C	4:744330	1.37E-09
YBR149W	2:548401	1.24E-05
YMR076C	14:449639	7.75E-11
YAL013W	12:662627	0.000173634
YOR175C	12:634227	8.98E-08
YDL167C	13:99675	0.000198779
YHL010C	8:84437	1.77E-14
YHR152W	8:111682	5.77E-06
YHR152W	13:69114	1.58E-05
YER035W	1:187607	2.43E-05
YER035W	7:375499	7.68E-09
YPL020C	15:136324	1.17E-05
YPL020C	16:511406	1.99E-12
YLR286C	2:555596	7.71E-16
YMR317W	15:136324	8.20E-05

YIL125W	13:27644	4.65E-05
YIL125W	15:136324	4.33E-05
YGR130C	15:143597	3.44E-06
YDL049C	15:108577	1.88E-05
YBR065C	2:368060	5.94E-08
YMR182C	2:426887	5.13E-06
YMR182C	3.954861111	7.75E-05
YPL264C	5:272258	1.16E-11
YGR080W	13:99675	0.000147525
YAR033W	1:185122	3.04E-12
YIL010W	9:325410	1.71E-06
YGR113W	2:569420	9.45E-08
YPL050C	16:462646	2.24E-11
YDR447C	15:116709	5.65E-06
YML019W	12:659357	1.27E-07
YML019W	13:227254	8.75E-11
YKL166C	2:667125	6.57E-07
YKL166C	11:129847	3.77E-07
YDL086W	12:662627	1.05E-14
YKL096W	11:261779	2.05E-06
YKL096W	15:136327	2.75E-11
YLR064W	12:266102	5.21E-08
YBR194W	2:636958	3.56E-05
YEL003W	15:116709	5.76E-06
YKL075C	12:662627	1.34E-05
YHR198C	15:108577	5.46E-06
YMR212C	12:659357	1.22E-05
YOR271C	3:81832	1.85E-13
YAL034C	15:136327	0.000111951
YOR084W	15:174364	4.39E-05
YDR140W	4:744330	2.38E-07
YDR207C	8:111679	5.54E-05
YIL075C	4:1188862	5.31E-06
YGR010W	13:91085	2.26E-06
YDR245W	2:533268	6.55E-05
YOR187W	15:174364	1.50E-07
YDR486C	15:571105	1.49E-05
YJL186W	13:27644	7.39E-07
YBR285W	2:562409	0.000326404
YMR220W	12:662627	2.56E-14
YAR002W	1:141187	2.65E-07
YOR069W	15:461694	1.91E-06
YPR106W	2:555596	1.12E-16
YBL031W	2:537314	0.000213895
YOR124C	2:419093	1.75E-05
YOR124C	14:449639	9.40E-15
YDR072C	2:519049	1.48E-08

YGL107C	15:113251	1.74E-05
YOR394C-A	15:1065719	3.34E-14
YNL291C	12:705100	5.76E-05
YBR157C	2:555596	1.24E-13
YKL192C	15:108577	6.66E-05
YLR283W	12:705190	2.76E-14
YMR291W	3:105042	3.62E-07
YLR151C	12:433955	1.47E-14
YOR165W	4:183753	9.90E-05
YOR165W	15:632882	4.73E-07
YHR079C	13:54913	0.000130196
YBL018C	2:185450	6.52E-16
YNL095C	7:139173	4.96E-05
YCL068C	3:201166	3.83E-07
YKL167C	15:180210	2.13E-08
YOR264W	2:562409	2.22E-15
YIR039C	9:437054	4.36E-14
YKL108W	2:562415	4.50E-05
YKL208W	3:201167	6.01E-07
YPL119C	12:634227	2.56E-05
YER053C	13:28622	1.60E-06
YER053C	15:136327	1.19E-06
YER053C	16:500348	4.25E-05
YOL105C	2:533268	9.58E-05
YOL105C	15:141627	4.30E-13
YCL018W	3:81832	6.44E-16
YBR110W	13:910741	4.21E-05
YDR518W	12:672779	1.15E-07
YDR210W	2:513408	7.80E-05
YDR210W	4:878291	6.61E-07
YDR210W	15:141627	1.16E-05
YLR089C	13:49903	1.22E-07
YJL097W	7:402879	0.00021771
YNL118C	2:615927	5.37E-05
YIL161W	9:46561	1.87E-14
YCL054W	3:79091	0.00023729
YOL162W	15:10427	4.75E-17
YPR121W	16:787283	3.35E-10
YFL047W	2:555787	5.87E-08
YDR331W	12:642137	2.18E-05
YDR261C	2:551299	7.47E-05
YDR261C	12:635380	3.44E-06
YOL077W-A	15:141627	3.59E-08
YOL077W-A	16:500348	4.11E-05
YDR118W	2:565216	2.54E-05
YDL125C	4:246738	6.06E-08
YOR285W	15:846344	3.33E-16

YBR296C	13:27644	3.29E-14
YML064C	13:149075	4.49E-07
YDL176W	12:668249	8.57E-06
YJL192C	2:426887	8.90E-05
YHR005C	8:111683	3.62E-06
YCR088W	14:449639	3.24E-11
YPL215W	15:113251	2.62E-05
YGR279C	7:1048152	6.27E-14
YOR161C-C	15:619862	6.50E-07
YML023C	2:519049	0.000122965
YGL196W	7:132483	4.55E-09
YFR036W	6:227675	3.08E-06
YBL089W	2:50915	4.22E-11
YBL089W	4:1471859	1.56E-05
YHR124W	13:110808	7.07E-07
YGL036W	2:537314	4.00E-06
YDR231C	8:457580	1.77E-14
YML010W	3.954861111	4.60E-05
YHR053C	2:555596	1.01E-10
YGL104C	7:312740	2.69E-12
YMR169C	15:136327	7.24E-09
YOL071W	15:174364	3.17E-05
YDL012C	4:433589	2.56E-15
YDL012C	12:635380	7.75E-05
YDR206W	14:449639	1.40E-10
YHR191C	4:165026	3.55E-05
YOR329C	15:141627	0.000121681
YOL011W	15:301076	4.99E-06
YPL002C	16:555416	9.54E-17
YER167W	5:511793	7.36E-05
YKL194C	15:108577	3.65E-06
YDR450W	9:133693	5.73E-05
YHL020C	8:56246	9.77E-09
YGL179C	7:167587	9.84E-08
YKL140W	11:178402	2.87E-14
YPL127C	15:108577	8.28E-07
YKL211C	5:272255	8.00E-09
YKL211C	11:46633	1.48E-06
YKL085W	13:99675	6.22E-05
YKL085W	15:108577	9.68E-07
YKL085W	16:500342	6.64E-05
YJL025W	9:244902	0.000103272
YMR048W	13:371857	1.03E-16
YLR312W-A	12:757807	2.10E-09
YML091C	13:49894	3.64E-10
YML091C	15:174364	6.19E-06
YML091C	16:500348	2.28E-05

YLR299W	12:744310	1.07E-06
YMR128W	15:113251	0.000215725
YPR155C	15:174364	2.06E-05
YOR338W	7:73036	1.68E-05
YLR137W	3:79091	9.40E-05
YLR137W	13:46070	4.35E-05
YJL196C	1:41483	9.14E-06
YJL196C	7:403626	8.31E-06
YNL055C	14:542648	1.52E-07
YNL055C	15:143597	4.14E-06
YOR042W	3:90610	0.000220797
YGL252C	7:15891	2.36E-12
YGL252C	13:556726	3.07E-05
YDL244W	10:28306	2.42E-08
YBR215W	2:676540	1.38E-08
YBR215W	12:662627	1.49E-05
YKR068C	2:555778	2.71E-05
YLL027W	12:92676	1.15E-12
YKL132C	11:194611	2.92E-15
YOL005C	14:525061	6.30E-07
YJL200C	2:477206	1.13E-10
YKR092C	11:612769	4.25E-07
YKR042W	4:1400033	2.01E-05
YDL168W	4:161196	8.94E-17
YGR088W	15:136324	2.21E-09
YMR135C	15:143597	9.14E-10
YMR135C	16:500342	5.49E-05
YDL236W	12:674651	1.39E-05
YLR300W	12:721979	3.68E-13
YPR184W	15:113267	1.16E-05
YHR046C	8:193175	2.73E-10
YGR108W	9:79823	7.61E-05
YPL078C	15:108577	6.04E-07
YPL078C	16:500342	6.41E-06
YBR095C	2:427677	0.000268552
YKL135C	11:178408	6.66E-07
YNR067C	2:555596	2.10E-16
YKR086W	12:668249	4.70E-05
YKR086W	15:113251	0.000113648
YAL067C	1:10152	7.55E-16
YLR346C	12:811669	7.93E-08
YMR115W	4:1213416	5.17E-06
YMR115W	13:481541	3.31E-06
YFL052W	10:23505	4.58E-16
YEL051W	12:644082	2.99E-05
YGL006W	7:490784	2.35E-11
YDL055C	2:555596	6.99E-13

YPL206C	16:125271	3.40E-08
YGL084C	14:449639	1.13E-07
YOL090W	15:113261	1.08E-06
YLL051C	13:910375	5.37E-05
YML124C	2:567221	7.81E-10
YOR062C	15:428238	1.57E-12
YOR157C	12:611810	0.000110708
YPL067C	16:428900	1.75E-07
YMR306W	13:885665	1.14E-09
YGR178C	14:449639	1.47E-13
YBR011C	13:115474	5.34E-05
YJL133W	3.956944444	4.17E-05
YJL133W	14:449639	3.04E-13
YDR079W	15:108577	4.97E-05
YIL009W	2:553812	4.37E-08
YPL262W	2:567221	6.97E-07
YIL166C	9:33795	3.78E-09
YML125C	2:562415	0.000115193
YML125C	12:644136	8.62E-08
YML125C	16:500348	8.53E-05
YBR028C	13:33681	5.50E-07
YHL001W	15:488377	8.51E-05
YDR440W	4:1344670	2.74E-12
YDR460W	3:201166	5.60E-13
YLR313C	2:537314	9.23E-05
YLR313C	12:760763	2.78E-13
YOR178C	15:193911	0.000171628
YJL016W	10:404508	1.53E-15
YJL218W	10:23505	4.88E-16
YGR071C	12:674651	1.50E-05
YLR256W	12:662627	2.41E-14
YML072C	14:449639	3.52E-07
YEL021W	5:117056	5.22E-17
YGR183C	12:705190	0.000230283
YNL107W	6:232259	7.76E-05
YHR146W	12:644136	0.000105669
YHR097C	13:46084	1.16E-05
YHR097C	15:113251	5.06E-05
YOR390W	13:910381	0.000404081
YDL119C	2:592863	2.99E-05
YJL108C	2:555787	7.72E-07
YJL108C	12:634226	3.23E-07
YHR006W	5:272258	1.62E-06
YIR026C	9:403134	3.24E-11
YHR024C	15:113267	6.09E-06
YEL062W	5:30057	1.42E-11
YEL062W	12:1067121	3.90E-06

YDR145W	2:533268	2.36E-05
YDR145W	14:449639	4.52E-09
YGR140W	2:499012	4.29E-05
YIL151C	9:84805	1.48E-08
YGR204C-A	7:905017	7.22E-12
YKL110C	11:229040	1.34E-06
YLR052W	12:254693	8.26E-11
YNR027W	2:519049	0.000183011
YJR121W	13:28622	9.70E-05
YJR121W	15:136327	9.47E-09
YJR121W	16:500342	8.20E-06
YDR539W	4:1510883	1.43E-16
YNL313C	14:38762	1.42E-10
YPL165C	13:46070	3.10E-05
YKL116C	11:222724	3.93E-12
YNR020C	15:154177	3.77E-06
YIR034C	2:477206	2.34E-06
YPL259C	2:533268	0.000167102
YEL036C	2:517365	5.38E-05
YBR240C	13:57145	7.84E-07
YOR016C	2:658746	4.39E-05
YML093W	13:99585	2.09E-07
YPL081W	4:1188862	7.03E-07
YHR087W	15:174364	7.29E-07
YER041W	8:150330	4.51E-05
YHR038W	15:113251	0.000188875
YLL028W	12:86369	9.11E-08
YLL028W	15:144659	1.16E-15
YFR030W	5:272258	3.74E-09
YMR087W	13:81250	4.41E-05
YLL058W	3:92013	0.000189702
YLL058W	12:634226	1.61E-06
YPR055W	15:106266	1.56E-05
YFL059W	6:48218	1.67E-05
YJL088W	13:49894	2.20E-08
YCR098C	3:301446	1.09E-13
YHR043C	8:193175	3.23E-13
YCR011C	14:449639	4.22E-10
YLR181C	12:516700	1.25E-12
YOR321W	12:634226	1.14E-06
YOR321W	13:404546	1.19E-05
YKL188C	1:36900	3.63E-06
YKL188C	11:97761	5.13E-06
YGL089C	3:201166	8.94E-17
YPR069C	13:87587	3.43E-06
YOR302W	15:141627	7.34E-05
YCR073C	15:150651	1.88E-06

YDR262W	4:975086	2.07E-15
YOL109W	15:116709	1.50E-06
YLR248W	12:634225	3.00E-06
YDR530C	15:141621	0.000116131
YJL166W	15:141627	2.83E-05
YJL166W	16:500348	3.85E-05
YNL329C	14:19885	1.33E-13
YER013W	15:113254	2.82E-07
YOR094W	15:469823	1.08E-05
YOL112W	2:537314	1.42E-10
YKR062W	11:566015	2.63E-08
YML067C	13:124876	3.45E-06
YPR015C	15:141633	0.000266426
YPR015C	16:500348	0.000119649
YIL094C	2:477206	4.54E-12
YEL076C	12:1056103	1.15E-06
YER183C	5:568698	2.12E-09
YER069W	13:49894	2.45E-05
YDR001C	4:446125	4.98E-10
YPL097W	15:174364	1.86E-05
YGL143C	15:180210	5.54E-06
YMR280C	15:136324	6.44E-06
YLR218C	15:180961	0.000191267
YJR032W	10:470298	3.35E-14
YIL162W	9:38608	3.22E-10
YNL116W	12:634227	6.13E-06
YLR233C	2:530481	1.89E-05
YLR461W	15:469823	0.000245031
YPL034W	16:500342	5.37E-06
YDL103C	13:81358	0.000166253
YOL032W	7:375499	6.69E-06
YOL032W	15:255015	8.19E-14
YLR303W	8:167504	1.02E-05
YJL100W	12:662627	6.75E-10
YGL001C	12:662627	2.87E-14
YPR200C	11:652304	9.23E-10
YPR200C	16:932538	4.27E-07
YJL068C	2:427677	5.85E-05
YGR272C	15:116709	1.29E-05
YJL158C	15:136327	8.40E-07
YMR279C	12:469156	1.41E-14
YGL038C	13:100048	0.000138679
YGL038C	15:174364	5.32E-05
YML117W	8:95469	5.80E-05
YML117W	14:449639	2.19E-07
YDL105W	4:273846	1.08E-15
YGR024C	2:562415	0.000227541

YOR028C	15:380725	4.38E-12
YLR265C	12:674651	1.93E-13
YNR043W	12:659357	9.02E-13
YMR237W	2:419093	1.17E-05
YMR237W	14:449639	1.19E-10
YGR085C	15:488334	0.000382775
YDR353W	2:555575	2.42E-06
YDR353W	13:268044	5.91E-05
YOR125C	15:572404	1.56E-09
YLR307C-A	5:420595	1.30E-06
YKL027W	11:390168	3.02E-08
YPL189W	3:90610	0.000115939
YPL189W	16:182953	2.44E-08
YBR112C	2:424450	5.85E-05
YLR336C	15:116709	0.000115418
YBR291C	2:480009	2.58E-13
YAR014C	2:567221	1.10E-08
YPL134C	16:256358	1.87E-08
YGR092W	8:111690	6.88E-05
YJR074W	10:573371	8.18E-07
YFR045W	15:108577	2.22E-08
YPR175W	7:375499	5.21E-10
YDR540C	4:1511257	3.02E-15
YBR296C-A	7:375499	3.01E-06
YBR053C	14:449639	4.68E-10
YPL017C	16:547618	2.03E-07
YJR014W	2:565216	1.67E-06
YJR014W	10:461201	1.35E-07
YGL169W	7:187179	2.21E-16
YDR345C	4:183753	0.000130507
YDR345C	15:106266	7.48E-05
YDR489W	4:1441486	4.77E-10
YGR198W	12:662627	4.35E-05
YGR198W	15:632882	9.79E-10
YIL042C	9:259533	3.50E-05
YLR364W	7:707998	0.000112648
YML123C	13:27644	4.49E-15
YHR011W	15:180210	0.00013907
YOR090C	15:491172	6.26E-07
YPL258C	16:70847	1.65E-12
YAL042W	1:55221	5.07E-05
YGR205W	7:913065	1.01E-16
YLL033W	12:514835	8.32E-10
YKL190W	14:449639	5.76E-10
YBR197C	2:616262	1.03E-16
YMR044W	13:430164	4.08E-06
YOR076C	12:674651	3.41E-05

YLR020C	2:551299	0.000142927
YLR020C	12:662627	3.35E-05
YPR062W	12:672779	3.70E-06
YEL061C	2:569420	4.83E-05
YLR260W	2:479166	7.17E-05
YLR260W	12:672779	7.91E-08
YIL044C	9:272372	8.28E-10
YOR003W	12:659357	6.62E-11
YKL035W	13:28694	1.26E-05
YGL057C	1:187519	1.88E-07
YGL057C	7:375499	3.02E-13
YER124C	2:555596	5.33E-16
YBL068W	13:28334	1.03E-06
YMR318C	13:922268	9.47E-06
YMR318C	15:141633	1.75E-06
YGL153W	16:500348	6.64E-06
YKR088C	12:635380	3.62E-07
YGR168C	7:804945	1.65E-06
YDL199C	4:106892	5.50E-09
YDL199C	13:28334	9.56E-05
YDL199C	14:410244	6.43E-05
YKL045W	11:354466	1.13E-11
YIL009C-A	8:111690	6.45E-05
YIL009C-A	9:334243	8.27E-05
YFL030W	15:136327	4.19E-05
YER139C	5:458085	1.22E-14
YKL015W	10:450212	0.000127639
YKL015W	15:96849	9.36E-05
YLR139C	12:423789	2.06E-13
YOR319W	1:42591	1.87E-08
YOR319W	15:116709	5.59E-05
YNL263C	2:521415	0.000178089
YLR237W	8:167506	9.78E-05
YLR237W	12:611967	5.51E-08
YLR454W	14:449639	5.19E-10
YKL162C	14:412272	0.000127921
YDR100W	15:141633	0.000115604
YPL245W	16:84943	4.00E-12
YPR174C	1:187607	5.75E-06
YPR174C	7:375499	1.76E-14
YJL056C	10:327858	1.50E-11
YML065W	15:113251	3.40E-05
YAL017W	14:449639	5.30E-11
YOL097C	15:180961	2.09E-05
YKR087C	11:599170	1.90E-15
YDR364C	4:1185630	2.96E-06
YGR266W	12:659357	1.24E-09

YHR129C	12:668249	4.70E-05
YKR101W	11:645253	9.01E-10
YER093C	12:659357	4.95E-06
YAL001C	14:449639	9.79E-09
YMR156C	13:574867	7.18E-14
YFR033C	12:668249	8.98E-10
YFR033C	16:500342	9.75E-06
YGR287C	7:1058948	2.43E-09
YER149C	2:392138	7.08E-06
YDL237W	2:419093	1.17E-05
YDL237W	14:449639	7.92E-08
YLR275W	12:689217	1.44E-07
YBR135W	2:519049	8.97E-10
YOR144C	16:500342	0.000196489
YOR228C	2:565216	0.000104759
YOR228C	16:500342	5.98E-06
YKL187C	11:97725	2.42E-14
YLL040C	12:70121	3.54E-05
YLL040C	14:449639	7.07E-10
YML008C	12:662627	5.82E-12
YLR165C	12:472165	3.50E-15
YFL020C	1:154328	9.43E-11
YAL010C	1:136161	7.92E-05
YNL167C	14:502496	1.17E-05
YGR240C-A	7:974640	5.06E-05
YPL189C-A	8:313108	7.70E-05
YMR298W	12:634227	5.65E-07
YOL055C	8:151814	1.57E-06
YHR049W	2:477206	9.73E-06
YHR049W	13:245632	0.000154093
YHR049W	15:488384	7.51E-05
YJL030W	4:178037	6.08E-06
YJL030W	10:380085	6.88E-05
YDR325W	2:562415	5.31E-05
YDR325W	15:106266	9.00E-06
YMR275C	13:824809	8.45E-10
YDR038C	4:527458	5.75E-17
YKL048C	8:111683	0.000353096
YKL048C	13:49894	9.85E-05
YPL045W	15:113261	1.26E-06
YPR006C	16:555416	9.78E-16
YMR043W	15:113260	0.000241645
YBR273C	2:746476	1.18E-06
YIL096C	9:191491	1.25E-06
YLR038C	12:668249	1.36E-06
YIL121W	3:81832	1.50E-05
YIL121W	12:677957	2.15E-16

YMR161W	13:556841	4.43E-05
YNR066C	14:732029	7.49E-06
YDR175C	15:113251	5.63E-06
YDR375C	15:113251	4.23E-05
YER176W	5:532188	2.95E-06
YHR113W	2:427674	9.48E-05
YLR271W	12:705190	0.00016142
YPR179C	15:116709	1.25E-05
YJL063C	15:174364	7.02E-07
YBR056W	2:391856	2.02E-05
YGL178W	7:187179	2.39E-10
YFL050C	6:38648	5.09E-09
YDR234W	2:477206	1.55E-08
YDL222C	4:54224	1.17E-06
YDL222C	15:174364	2.97E-05
YMR274C	8:111680	0.000308347
YMR274C	16:500348	1.93E-05
YNL073W	15:108577	1.80E-07
YBR275C	2:750838	5.80E-14
YER093C-A	15:154177	2.10E-05
YIR007W	14:486861	3.03E-10
YIR030C	2:562409	1.16E-05
YIR030C	9:394278	3.24E-05
YKR085C	11:599170	4.86E-10
YGL068W	15:154177	5.00E-06
YCL026C-A	3:75021	2.29E-13
YCL026C-A	11:655159	0.00013489
YER170W	5:500789	9.28E-06
YER170W	15:113251	1.87E-06
YJR099W	10:612602	8.98E-13
YKL220C	13:910381	7.44E-05
YKL168C	11:114412	2.07E-08
YHR051W	15:141627	2.50E-07
YHR051W	16:500348	3.28E-05
YIL073C	13:27644	3.45E-07
YBR162C	2:628224	1.55E-05
YHR123W	14:449639	9.53E-06
YGR197C	15:632882	1.15E-12
YKL022C	11:394654	1.20E-08
YLR360W	2:592989	5.67E-05
YIL004C	8:389050	8.89E-05
YDR337W	15:174364	5.35E-08
YPR125W	14:486861	3.96E-09
YML054C	15:154177	6.60E-06
YJR134C	12:668249	9.56E-07
YKL103C	2:533262	7.73E-06
YLR342W-A	12:815498	1.30E-16

YHR072W	12:662627	6.99E-13
YBR023C	2:537314	1.74E-08
YBR023C	16:500348	5.18E-06
YEL038W	5:79647	2.02E-16
YMR202W	12:662627	1.83E-15
YDR200C	12:674651	2.03E-05
YFR016C	14:449639	1.37E-09
YGR141W	2:391856	2.31E-05
YGR141W	14:449639	1.48E-10
YNL217W	2:548401	1.22E-05
YNL217W	13:33681	1.27E-07
YKL121W	3:79091	6.06E-06
YGR177C	4:1400033	2.30E-07
YGR177C	12:611854	3.69E-05
YCL034W	14:449639	3.82E-11
YCR026C	3:175808	1.08E-08
YCR026C	12:634227	0.000110806
YOR155C	3:105042	0.000134057
YKL034W	2:376872	3.40E-05
YOR347C	13:572643	0.000188016
YOL064C	13:46084	1.25E-08
YDR349C	15:141627	0.000205051
YFR019W	2:388862	2.73E-05
YGL096W	7:311205	5.57E-09
YGL096W	12:689217	3.50E-05
YGL096W	13:410287	1.52E-06
YPL180W	12:705226	0.00023945
YDL104C	4:273846	1.11E-12
YGR218W	14:449639	5.22E-13
YGR036C	1:187640	0.000177299
YGR036C	7:543259	7.62E-06
YDL008W	4:433589	8.78E-06
YDL139C	4:201395	5.70E-10
YDL139C	13:69122	0.000338358
YOR022C	13:261725	8.25E-05
YOR022C	15:301076	0.000118232
YPL066W	2:620056	4.66E-07
YPR005C	2:537314	1.82E-06
YLR103C	2:555596	0.000114134
YKR043C	15:180961	2.74E-05
YOR171C	12:659357	0.000100341
YDR541C	4:1525327	5.78E-10
YGL012W	12:450046	0.000173634
YDR531W	4:1495582	1.32E-12
YER168C	15:116709	2.13E-06
YDR347W	15:113254	1.52E-07
YOR246C	2:584357	1.01E-05

YML075C	12:662627	1.05E-14
YMR071C	13:922258	9.11E-05
YCR052W	3:210748	1.02E-09
YFL020C	1:201024	1.44E-07
YGR166W	15:180961	0.000164359
YOL154W	15:10427	1.67E-16
YDR534C	13:910741	7.87E-06
YDR354W	12:662627	5.46E-05
YLR040C	3:201166	8.94E-17
YMR037C	15:113260	4.12E-06
YLR457C	2:553812	4.56E-05
YLR457C	12:1059928	2.71E-05
YGR213C	15:154177	4.89E-05
YHR140W	12:668249	6.98E-05
YLR231C	12:644136	6.62E-13
YPR107C	2:628224	5.10E-05
YKR003W	11:442468	3.65E-08
YOR353C	15:143597	2.04E-07
YLR214W	12:635380	6.58E-08
YFR039C	15:106164	5.46E-06
YBR151W	15:632882	8.62E-11
YPL019C	13:27644	1.19E-14
YDR502C	12:662627	3.79E-13
YDR502C	13:404546	4.22E-05
YKR104W	11:656099	5.75E-17
YHR063C	3:92247	4.25E-05
YLL015W	15:136324	7.95E-05
YKR098C	5:422612	4.55E-05
YOR389W	13:910381	1.81E-09
YER136W	12:634226	2.10E-06
YPL026C	16:486643	1.86E-06
YGR149W	2:372509	3.54E-05
YDR043C	2:427677	1.24E-07
YOR217W	2:519049	0.000225842
YOR217W	12:662627	0.000106268
YGL171W	7:187567	1.62E-07
YLR376C	12:851826	1.56E-10
YBR079C	2:388862	4.98E-06
YDL076C	15:703769	2.64E-05
YML105C	13:910381	0.000266959
YDL089W	4:289639	5.04E-10
YOR080W	15:106164	1.44E-05
YGR208W	7:913065	2.45E-11
YBR131W	15:108577	1.70E-05
YLR234W	12:635380	2.56E-10
YDR292C	12:705100	1.88E-06
YOR238W	15:804686	8.19E-12

YIL115C	9:68997	8.20E-05
YIL115C	15:113251	3.79E-05
YNL111C	12:662627	7.43E-10
YMR078C	2:533268	0.000134893
YMR078C	12:697260	5.85E-05
YPR043W	15:481586	1.23E-05
YCR090C	14:705559	0.000279775
YGL055W	12:662627	4.17E-10
YMR225C	15:113267	1.24E-07
YER089C	2:565216	1.69E-07
YML131W	3:175808	2.57E-05
YAR027W	1:185122	1.04E-15
YLR239C	15:113251	2.93E-05
YNL161W	2:569420	7.79E-07
YPL132W	15:108577	2.50E-07
YER088C	15:136327	1.68E-09
YER088C	16:511406	1.01E-05
YCR093W	3:301446	1.51E-07
YJL139C	10:151310	8.72E-16
YAR031W	1:185122	5.02E-12
YAL064W-B	5.397222222	4.88E-16
YDR520C	12:668249	0.000124398
YDR217C	13:124876	2.94E-05
YDR217C	15:113251	3.04E-05
YDR217C	16:535979	0.000103272
YOL104C	15:132423	5.29E-16
YIR031C	9:419418	5.36E-15
YPL141C	8:71742	4.08E-05
YDR067C	4:582121	1.47E-09
YDR067C	14:449639	1.79E-05
YBL067C	12:662627	1.31E-06
YDR213W	12:644136	2.16E-06
YGR089W	12:659357	7.36E-07
YDR036C	15:170945	9.89E-05
YOL127W	4:1491116	4.05E-06
YKL161C	2:537314	0.000134774
YKL161C	9:68997	7.10E-05
YDR297W	12:662627	4.82E-10
YOR298C-A	14:449639	2.29E-07
YFR025C	5:272255	1.25E-06
YIR004W	9:399057	4.09E-07
YDR453C	12:662627	9.47E-06
YGR295C	7:1081945	7.64E-17
YOR213C	14:449639	2.19E-07
YDR272W	2:530481	0.00020483
YDR272W	4:963769	1.19E-12
YDR273W	4:975086	6.83E-11

YPR191W	12:668249	0.000116768
YPR191W	13:28622	1.50E-05
YPR191W	15:113251	6.47E-07
YPR191W	16:500342	1.47E-05
YJR046W	7:375499	9.72E-09
YLR410W	1:139312	7.66E-05
YLR410W	13:27644	1.91E-13
YGR223C	7:940716	1.94E-16
YOL133W	15:59733	3.58E-13
YPR151C	12:662627	6.62E-13
YML032C	12:668249	5.96E-06
YML032C	13:180103	7.42E-08
YER100W	12:713644	8.17E-06
YML096W	13:81250	1.10E-10
YMR309C	14:449639	1.48E-07
YDR506C	2:486640	1.23E-05
YDR506C	14:449639	4.81E-08
YDL133W	12:672779	4.36E-05
YDL133W	15:703770	4.09E-05
YHL006C	8:111680	9.90E-10
YPR001W	1:208493	6.89E-05
YDL182W	2:480009	2.18E-10
YER150W	5:468641	5.76E-09
YER150W	11:211823	0.00016043
YDL243C	6:28029	1.32E-15
YDL230W	2:565216	5.31E-05
YDL230W	4:46466	0.00044826
YAL053W	3:81832	8.81E-05
YAL053W	7:708237	0.000149484
YAL053W	9:244902	1.90E-05
YAL053W	15:143597	5.79E-05
YLR243W	12:607076	6.61E-05
YJR010C-A	10:461201	3.05E-15
YDR186C	12:668249	3.36E-07
YGR084C	15:113251	5.86E-06
YNL270C	2:391856	1.83E-07
YKR052C	13:922256	6.74E-06
YFL004W	1:139312	0.000179874
YFL004W	13:27644	1.51E-14
YCL027W	8:111683	2.44E-13
YER024W	12:681096	0.000192182
YER163C	5:504714	2.87E-10
YOR101W	2:391856	5.09E-05
YLR244C	12:635380	7.18E-15
YNR037C	15:180210	5.96E-06
YBR165W	2:565216	2.05E-10
YEL030W	3.956944444	0.000135826

YEL030W	14:449639	6.62E-13
YLR356W	2:533262	1.75E-05
YLR049C	12:247886	9.76E-16
YNL045W	13:28694	2.14E-06
YNL045W	15:141633	5.54E-07
YPR118W	12:672785	4.72E-05
YML025C	15:174364	1.01E-05
YOL043C	15:248746	1.29E-15
YLR430W	12:990751	1.16E-08
YIL091C	9:244902	2.03E-05
YKL186C	11:97761	7.54E-11
YEL047C	12:659357	5.14E-07
YJL024C	10:398821	1.94E-07
YML057W	13:159533	1.36E-10
YPR020W	15:141627	1.24E-05
YPR020W	16:500348	5.56E-07
YNL252C	15:174364	4.34E-06
YDR129C	3.954861111	1.76E-05
YAL005C	1:136161	2.22E-05
YFL017C	16:511406	0.00011278
YOR385W	13:27644	1.51E-05
YAL029C	15:106164	1.34E-07
YGL063W	7:375499	1.73E-10
YBR016W	12:668249	3.47E-05
YJL131C	13:100048	1.79E-05
YPL092W	16:368296	8.98E-08
YPR165W	2:565216	3.18E-06
YFL008W	6:134096	8.26E-09
YGL066W	1:187544	2.61E-06
YGL066W	7:375499	1.41E-08
YKL072W	12:668249	1.10E-05
YKL072W	15:113251	7.06E-07
YGL062W	9:19607	0.000459577
YKR022C	12:659357	0.00015081
YLR204W	15:113251	2.05E-07
YPR141C	2:555596	3.36E-06
YDR147W	12:659357	4.04E-05
YER073W	3:91305	1.04E-12
YER073W	13:46084	1.76E-05
YIL013C	9:325410	8.27E-06
YOR308C	4:744330	3.20E-06
YMR196W	15:174364	6.19E-06
YJL144W	1:187607	2.02E-07
YJL144W	7:375499	8.61E-14
YGR031W	15:136324	3.02E-07
YPR111W	2:569420	2.73E-09
YHR209W	9:98955	7.05E-06

YMR055C	2:481439	5.52E-05
YMR055C	4.864583333	8.65E-05
YMR055C	13:390351	3.88E-08
YBR085C-A	3:91977	2.48E-05
YGR252W	7:995892	1.78E-07
YMR170C	14:449639	1.01E-10
YGL185C	2:530481	9.37E-08
YOR336W	14:449639	9.67E-07
YNR050C	2:477206	1.66E-12
YLR270W	12:674651	7.79E-06
YCR012W	2:368991	1.53E-05
YDR346C	12:644136	4.03E-06
YIL070C	15:154177	1.02E-05
YDR246W-A	4:962969	1.34E-05
YDR246W-A	7:403626	3.76E-05
YJR025C	10:472147	2.50E-14
YKR010C	2:565216	1.86E-05
YKR016W	15:113251	7.14E-06
YMR075W	15:108577	0.000110791
YKL185W	2:555596	5.42E-12
YDL235C	2:628224	7.08E-05
YGR233C	13:27644	9.34E-15
YEL011W	15:136324	3.62E-06
YEL011W	16:542295	1.45E-05
YJR078W	15:850119	9.98E-06
YFL017W-A	6:100521	2.00E-08
YCL063W	8:95289	0.000304732
YCL063W	13:46084	7.64E-05
YCL063W	14:449639	6.66E-09
YBL016W	2:533268	1.80E-05
YBL016W	8:111683	8.62E-08
YIL110W	9:133693	5.84E-06
YMR005W	13:268045	5.50E-07
YIL083C	8:111690	0.000217804
YIL083C	9:196145	1.64E-09
YIL083C	12:644082	0.000141348
YJR110W	2:551299	0.000138214
YJR110W	12:662627	2.88E-06
YGR028W	7:553877	2.50E-08
YBR040W	8:111683	3.36E-12
YNL033W	14:586789	7.84E-07
YDR244W	1:52943	5.43E-08
YKL178C	3:201166	8.94E-17
YGR033C	15:551819	1.05E-06
YHR030C	2:537314	3.86E-08
YHR030C	9:98955	0.000106881
YHR030C	15:141621	4.92E-05

YGR026W	12:634227	9.78E-07
YLR371W	16:495156	7.72E-05
YKR031C	11:482069	2.70E-13
YGR015C	2:401568	3.53E-05
YOR073W	12:662627	1.44E-05
YOR073W	15:472577	5.47E-05
YDR323C	12:644136	2.92E-07
YKL018C-A	16:500342	8.27E-05
YGR217W	2:565216	3.56E-07
YLR152C	8:457580	3.84E-12
YLR152C	12:514835	1.25E-06
YKL133C	14:449639	5.91E-11
YML030W	15:113267	1.80E-07
YER105C	14:449639	2.08E-11
YOR059C	15:438824	9.19E-13
YKR071C	3:90676	8.47E-12
YCR102C	12:1059818	3.90E-11
YCR102C	15:136324	7.96E-06
YNR058W	13:115474	1.17E-05
YKL148C	11:174008	3.11E-07
YKL148C	15:136327	3.25E-06
YLR319C	2:567221	6.15E-06
YLR319C	16:500342	9.07E-05
YBL037W	2:517123	7.53E-06
YBR048W	5:210999	8.93E-05
YKL119C	3:90610	0.000180632
YGL015C	13:81250	0.000244819
YMR063W	13:379981	1.10E-09
YDR178W	15:113254	1.04E-05
YDR178W	16:500342	0.000116052
YMR208W	12:662627	3.05E-14
YAL026C	12:662627	6.84E-05
YJL137C	13:115474	1.69E-05
YKL163W	11:153463	3.69E-08
YLR179C	12:514835	1.88E-16
YGL136C	7:256953	3.05E-12
YGR232W	13:33681	8.30E-05
YNL002C	14:449639	5.51E-08
YIL014W	9:325320	2.26E-15
YFL005W	2:636958	2.14E-05
YPR122W	8:111682	7.22E-07
YGL225W	12:634227	8.05E-07
YOR019W	15:357194	2.74E-16
YMR244C-A	15:180180	9.07E-06
YIR037W	13:115474	0.000210641
YEL034W	12:662627	1.50E-14
YGR216C	2:565216	1.31E-05

YPR091C	13:28694	9.88E-06
YDR285W	5:525380	0.000112477
YMR251W-A	2:567221	0.000281872
YMR251W-A	15:143597	7.50E-05
YMR251W-A	16:500348	2.13E-05
YHL009C	8:93002	2.04E-10
YLR225C	12:611967	4.90E-05
YHR018C	8:111680	1.51E-05
YOR038C	15:357194	5.63E-13
YHR207C	12:644082	9.48E-05
YHR207C	15:143597	3.70E-06
YFL054C	4.333333333	1.24E-09
YFL054C	10:34098	5.87E-11
YGL160W	12:674651	1.51E-08
YML016C	13:239559	4.47E-13
YOL018C	13:261719	0.000164642
YOL018C	15:298710	3.16E-11
YBR168W	12:611967	4.16E-05
YJR059W	16:500342	3.17E-05
YER016W	8:111683	1.38E-06
YPL263C	12:683463	6.61E-06
YLR056W	12:662627	2.32E-07
YML070W	2:427674	0.000301614
YML070W	13:79760	8.18E-05
YML070W	14:449639	5.22E-13
YGL229C	2:567221	2.37E-11
YGL229C	7:403626	0.000471687
YLR246W	12:635380	2.87E-15
YBR235W	2:697894	1.77E-07
YGR175C	15:141621	8.29E-07
YKR026C	11:484826	9.92E-15
YAL064C-A	0.377777778	2.39E-13
YKL070W	13:57145	3.84E-05
YJR075W	10:573371	8.58E-13
YLR108C	13:99675	4.44E-07
YLR108C	16:542295	6.00E-05
YMR091C	13:437167	1.14E-05
YJL048C	12:662627	5.01E-13
YJR054W	2:555596	3.27E-08
YOL140W	13:49894	6.98E-08
YEL058W	5:44617	3.35E-08
YAL015C	9:101011	2.92E-05
YLR154W-E	4:74695	0.000162946
YML118W	2:567221	2.68E-07
YBR097W	2:391856	9.65E-07
YBR097W	14:449639	3.23E-07
YOL110W	5:194883	8.04E-05

YMR079W	12:634225	9.91E-05
YLL029W	12:86369	1.50E-07
YBR228W	2:667083	1.18E-08
YEL020C	14:449639	9.51E-12
YOL006C	15:113261	3.14E-05
YDL189W	4:114155	7.26E-10
YDL189W	13:33681	3.09E-05
YOR054C	15:438824	1.07E-10
YIR036C	2:419093	3.84E-05
YIR036C	14:449639	8.49E-12
YNL189W	13:87587	0.000199421
YDL181W	13:28622	4.66E-05
YDL181W	15:141627	1.19E-08
YDL181W	16:500348	2.52E-06
YNL224C	1:136161	0.000112549
YNL224C	13:27644	8.65E-07
YNL224C	15:113251	3.79E-05
YHR054C	8:209167	5.06E-11
YDL093W	12:662627	1.44E-09
YDL093W	16:497425	2.61E-05
YDL225W	13:57145	1.41E-06
YPR128C	1:51324	4.36E-07
YDR276C	12:668249	3.89E-06
YGL101W	12:634227	1.02E-06
YDL185W	14:486861	4.35E-09
YDR096W	15:174364	5.37E-06
YKL062W	15:136327	8.32E-12
YKL062W	16:500342	5.15E-05
YLR342W	12:810883	1.56E-05
YML108W	15:116709	7.20E-06
YAL064W	1:11638	7.28E-13
YAL064W	8:525664	1.09E-05
YER028C	4:226317	1.34E-10
YEL050C	15:174364	1.44E-07
YDR079C-A	4:555043	9.44E-06
YDR079C-A	15:116709	4.12E-05
YAL062W	1:10152	4.93E-13
YBR067C	2:376145	9.92E-07
YBR067C	12:662627	1.66E-09
YNL047C	14:558284	6.05E-07
YNL047C	15:1075593	2.29E-05
YLR199C	2:508843	9.87E-05
YJR094C	10:604478	8.38E-17
YGR117C	13:28334	1.40E-05
YOR332W	12:634227	7.60E-06
YDR504C	15:880783	1.11E-05
YAR020C	1:154328	9.43E-11

YGR001C	7:502131	1.54E-09
YNL087W	7:129150	3.53E-05
YNL087W	15:113251	2.26E-05
YNL087W	16:500348	5.67E-05
YPL192C	4:963733	4.58E-07
YPL192C	8:111683	3.41E-08
YBR094W	2:427677	1.64E-12
YLR253W	12:644136	2.62E-05
YLR253W	15:113251	4.38E-05
YHR188C	12:705226	2.14E-05
YPL084W	3:90676	9.95E-09
YDR405W	4:1272737	5.74E-10
YDR405W	15:154309	1.17E-06
YEL065W	13:910741	3.06E-06
YNL005C	15:174364	4.83E-06
YLR073C	12:282091	1.08E-15
YJR112W-A	10:627628	1.63E-13
YLR312C	13:49894	0.000211115
YLR312C	16:500348	5.32E-05
YHR039C-A	12:659357	5.94E-06
YEL017W	5:117056	2.71E-08
YIL046W	7:707608	5.08E-05
YIL046W	9:268352	5.62E-15
YIL017C	3:201166	4.06E-14
YBR106W	12:659357	1.54E-06
YFL018C	15:136327	6.37E-05
YBR286W	12:634227	2.04E-09
YJL154C	10:135902	5.07E-09
YNL015W	2:567221	3.86E-09
YNL015W	16:547618	0.000214327
YGL023C	5:272258	3.49E-06
YGL023C	9:242417	1.25E-05
YPL016W	16:511406	3.32E-13
YKR067W	13:124876	0.000112018
YKR067W	14:449639	6.93E-07
YKL193C	10:387893	7.54E-05
YKL193C	15:174364	2.69E-05
YOL152W	13:33501	4.42E-05
YKL138C-A	11:218796	0.000125528
YIL140W	2:615927	6.21E-08
YKL080W	12:672779	3.15E-09
YHL035C	13:910381	0.000109213
YLR213C	12:611967	1.26E-08
YLR245C	12:635380	3.59E-12
YDR369C	13:99675	2.77E-07
YCL045C	14:449639	8.19E-07
YPR084W	13:492201	9.34E-06

YPR086W	2:517123	4.70E-05
YPR086W	12:674651	5.25E-06
YGR146C	3:79091	5.28E-07
YGR146C	13:28622	1.26E-05
YOL130W	13:33681	1.62E-07
YLR226W	14:542648	3.03E-05
YNL306W	15:154177	2.21E-06
YOR056C	2:537314	0.000162373
YBR262C	15:113267	0.000302327
YPL170W	12:705226	1.32E-05
YPL083C	16:387239	1.36E-09
YIL030C	16:500348	4.53E-05
YKR090W	11:612769	1.73E-13
YOL155C	9:27026	8.59E-12
YFL013C	12:808623	1.27E-08
YBL034C	2:565216	4.66E-05
YBL034C	15:136327	0.000161516
YDR169C	4:782114	7.37E-09
YKR063C	15:113251	7.14E-06
YFR009W	14:449639	3.82E-12
YIR033W	1:42591	4.58E-05
YIR033W	12:662627	4.01E-06
YBR173C	2:582419	3.03E-12
YAL028W	12:659357	2.37E-08
YMR319C	13:922258	1.07E-12
YOL017W	2:562415	0.000220739
YOL017W	15:298710	9.24E-12
YNL249C	14:168128	3.54E-15
YKR100C	2:533268	7.01E-06
YOR010C	5:350744	9.05E-07
YOR010C	12:662627	1.17E-05
YNR016C	1:42591	1.80E-06
YNR016C	2:521415	3.95E-05
YBL013W	15:136327	7.42E-06
YJL019W	10:404508	2.48E-06
YBL097W	6.628472222	3.41E-05
YHR003C	12:611967	9.09E-07
YOR034C	12:662627	4.47E-06
YOR034C	15:113251	2.35E-05
YNR034W	14:689939	1.55E-14
YNR057C	5:272258	3.94E-14
YLR352W	12:829693	2.96E-06
YDR372C	12:659357	4.46E-05
YFR005C	3:105042	0.000107334
YFR005C	13:46070	1.91E-05
YOR316C-A	2:553812	5.93E-12
YEL052W	15:136327	2.16E-06

YEL055C	15:154177	1.49E-05
YDL213C	4:70901	2.83E-08
YJR013W	10:492254	1.79E-08
YGR052W	15:141627	1.64E-09
YFR008W	13:54913	1.06E-05
YDR356W	4:1188862	3.14E-10
YJL187C	2:537314	4.64E-06
YPL168W	12:677957	9.10E-06
YJL057C	10:327858	2.04E-12
YNL178W	2:516889	0.000183692
YGL004C	2:609055	1.37E-07
YDR430C	15:174364	0.000105861
YHR154W	2:584351	2.82E-05
YPL278C	13:910381	2.99E-12
YLL013C	12:112275	1.21E-15
YER109C	2:548401	4.09E-08
YJR113C	12:668249	1.41E-05
YJR113C	15:113251	9.56E-05
YGR041W	2:567221	3.02E-15
YCR048W	12:668249	1.29E-07
YJL008C	10:450338	7.54E-05
YGL215W	14:449639	3.22E-12
YER154W	2:555596	6.17E-08
YDL067C	15:141627	0.000110791
YOL019W	2:551299	5.17E-06
YLR383W	12:881903	2.97E-07
YKR027W	3:201166	7.41E-08
YKR027W	16:511406	1.60E-05
YER046W	5:243221	2.77E-12
YIL169C	9:27026	1.27E-11
YBR003W	15:136324	1.83E-05
YMR304W	14:449639	7.58E-12
YDL117W	15:179289	1.45E-06
YOR306C	15:880783	1.24E-06
YGR281W	12:644136	1.67E-06
YMR058W	12:501528	0.000142588
YMR058W	13:922258	1.06E-06
YPL105C	14:449639	1.00E-12
YBR183W	12:659357	2.73E-06
YPL214C	1:36900	0.000114075
YKL130C	11:194611	3.32E-12
YBL005W-A	2:246129	0.000120729
YHR037W	15:108577	9.17E-05
YHR037W	16:492351	5.55E-05
YML080W	14:449639	1.26E-11
YER095W	2:592863	1.56E-05
YBR243C	14:449639	4.81E-08

YDR179W-A	15:113251		0.000246128
YNL146C-A	3:201166		8.94E-17
YOR126C	2:562415		1.43E-07
YMR089C	12:659357		0.000165454
YCL057W	14:449639		9.51E-12
YDR038C	4:527458		4.51E-16
YOR002W	12:634226		1.82E-05
YGR109W-A	7:708028		2.27E-14
YGR109W-A	8:92960		0.000164921
YGR109W-A	9:200332		1.20E-13
YLR069C	15:154177		2.95E-06
YNR010W	12:611854		3.87E-06
YNL063W	4:527511		0.00011664
YNL063W	10:548177		9.96E-10
YLR295C	13:28622		3.25E-05
YLR295C	15:136327		1.41E-08
YLR295C	16:511406		1.50E-07
YFL026W	3:201166		8.94E-17
YDR261W-B	7:553877		6.35E-06
YDR367W	4:1213416		9.08E-16
YDR307W	14:449639		1.48E-07
YGL040C	12:659357		4.01E-08
YPL173W	15:174364		2.91E-06
YIL016W	8:111686		1.82E-05
YML078W	13:115474		1.45E-15
YML078W	14:449639		3.67E-05
YIR019C	9:387985		1.91E-07
YDL170W	15:180210		2.63E-06
YBL060W	2:555596		1.25E-07
YDR034C-A		4.863888889	0.000124598
YOR278W	2:565216		4.36E-05
YOR278W	16:500342		2.97E-05
YBR259W	16:500342		5.15E-05
YHR077C	15:136324		7.21E-08
YNL261W	12:635380		8.81E-05
YNR052C	14:449639		2.03E-10
YJL099W	2:567221		4.00E-07
YGL164C	2:582419		4.62E-05
YOR292C	13:99672		6.79E-06
YPR047W	15:113261		4.86E-05
YGL212W	7:92896		2.75E-16
YHL022C	8:92978		2.24E-07
YER180C	13:27644		1.36E-05
YIR002C	13:124876		1.85E-06
YKL209C	3:201166		8.94E-17
YGR008C	15:143597		5.08E-05
YHR001W-A	15:141627		2.25E-06

YHR001W-A	16:500348	2.52E-05
YDR060W	15:136324	9.89E-05
YMR140W	13:507305	6.87E-07
YOL069W	15:206266	3.41E-06
YPL118W	15:154177	2.12E-06
YOR384W	13:410287	2.89E-07
YBL015W	16:500342	0.000177086
YHL040C	13:910741	1.10E-05
YOR363C	1:55215	1.84E-05
YDL035C	12:713644	2.59E-05
YJR004C	3:201166	8.94E-17
YNL090W	2:628224	1.13E-05
YPR134W	4:184214	3.24E-05
YHR214C-E	13:27644	4.02E-10
YNL106C	2:537314	3.45E-06
YJR017C	10:464261	1.97E-06
YLR205C	12:662627	6.93E-15
YNL293W	2:530481	0.000170747
YPL103C	6:234349	1.10E-06
YEL063C	5:40306	8.51E-05
YBR057C	12:858996	2.79E-05
YFL041W	13:922258	0.000110777
YOL003C	14:449639	7.17E-12
YKL004W	12:668249	1.59E-06
YBR037C	15:174364	2.60E-05
YFR044C	3:90610	0.000220797
YFR044C	13:77684	3.62E-07
YFR044C	15:141633	2.27E-06
YJL115W	2:548401	7.03E-06
YOL027C	11:98330	0.000226681
YOL027C	15:113267	4.36E-05
YFR011C	6:168354	0.000183774
YFR011C	15:141621	2.97E-07
YFR011C	16:497425	5.09E-05
YNL142W	2:567221	3.55E-07
YNL142W	16:500342	1.12E-05
YPL024W	16:500348	3.08E-08
YLL066W-B	4:1510883	7.34E-07
YLL066W-B	12:1056097	3.44E-07
YML086C	2:533262	0.000143441
YKL201C	11:47707	2.27E-10
YIL117C	2:533262	5.65E-11
YJL072C	5:251271	1.04E-06
YML087C	13:130069	2.90E-07
YLR382C	15:180210	5.55E-07
YIL164C	9:33795	3.44E-16
YOR221C	13:49894	5.14E-07

YAR007C	1:136161	3.28E-12
YFR017C	15:174364	2.44E-05
YOR183W	15:683415	4.16E-08
YER182W	15:154309	3.22E-05
YER014W	5:422612	2.33E-05
YER014W	12:672779	2.95E-12
YNL298W	2:537314	1.79E-07
YDL137W	4:201395	8.56E-07
YML129C	15:174364	2.91E-06
YPL183W-A	15:108577	1.32E-06
YGR263C	2:427677	0.00012553
YLR094C	12:327131	1.83E-16
YHR171W	13:158910	0.000125129
YLR087C	12:317608	6.69E-07
YOR086C	14:449639	9.28E-07
YMR185W	15:113251	3.79E-05
YJL044C	10:353027	4.23E-14
YBR004C	15:174364	2.20E-05
YER063W	2:553812	2.13E-08
YJL092W	2:569420	1.96E-05
YBR230C	13:28622	0.000241748
YBR230C	15:108577	6.54E-06
YJR010W	8:152932	1.58E-07
YOR012W	15:357194	5.39E-16
YOL081W	15:170945	1.05E-09
YLR173W	12:508029	9.93E-12
YOR214C	16:500342	7.38E-06
YOR148C	15:594024	9.73E-09
YDR259C	2:486640	4.02E-05
YNL098C	15:174364	6.41E-06
YOL002C	1:52943	0.000168953
YOL002C	7:402835	4.33E-10
YOL002C	12:659357	0.000100341
YDR514C	2:486640	1.95E-05
YGR112W	15:113254	2.08E-06
YBR250W	12:22752	1.79E-05
YDL128W	8:111686	0.000114315
YDL128W	12:22602	0.000239824
YCR037C	13:27644	9.38E-13
YGL005C	7:459354	4.64E-06
YDR300C	3:201166	2.99E-05
YDR300C	15:108577	0.000293529
YDL138W	15:141633	0.000182652
YOR381W	13:910375	9.32E-05
YJR049C	12:713638	9.87E-07
YJR048W	12:662627	5.52E-12
YBL041W	2:143721	5.78E-14

YBL033C	2:163240	0.000109571
YGL224C	13:33501	6.26E-12
YOL143C	5:117056	8.52E-16
YKL096W-A	15:174364	1.51E-10
YGL197W	7:117900	1.23E-09
YBR298C	7:1058950	1.46E-13
YDR313C	7:375499	7.21E-06
YIL074C	13:33501	2.25E-06
YNL175C	14:220948	1.08E-05
YJL141C	15:136327	1.06E-06
YJR060W	10:548177	7.38E-17
YPL184C	14:449639	6.96E-11
YPL240C	1:187607	0.000234621
YPL240C	7:375499	1.05E-10
YJL212C	10:22315	1.81E-09
YJL212C	12:697260	4.83E-06
YNR013C	14:614342	7.77E-07
YGR082W	15:174364	4.83E-06
YGL050W	1:187402	8.39E-05
YGL050W	7:402841	1.83E-06
YDL004W	13:28622	1.45E-05
YDL004W	15:136327	2.38E-07
YDL004W	16:500342	2.17E-06
YNL283C	2:548401	2.03E-08
YOL026C	12:677957	9.95E-05
YMR165C	2:499889	2.56E-05
YMR165C	13:572643	2.75E-07
YDL099W	12:719857	1.57E-06
YDR046C	3:105042	0.000110817
YDR046C	5:332264	3.98E-06
YKL114C	8:95469	9.21E-05
YDL217C	4:78583	1.20E-08
YBR021W	2:252550	6.12E-06
YOL038W	13:273244	0.000123329
YDR463W	5:312672	2.61E-08
YCL056C	3:14066	8.02E-13
YBR146W	15:180210	3.70E-07
YKR065C	15:108577	1.76E-05
YHR034C	8:185012	1.17E-08
YHR043C	8:203246	3.77E-14
YJL012C	1:139312	0.000123734
YJL012C	13:27644	2.04E-14
YGR086C	15:136327	1.33E-09
YGR086C	16:500348	7.64E-06
YJL103C	15:154309	7.36E-07
YLR369W	12:858996	1.66E-12
YIL173W	10:22273	1.80E-14

YBR199W	2:620068	4.29E-10
YER055C	5:251271	8.22E-06
YER055C	13:46084	9.54E-07
YIR016W	10:387893	1.55E-05
YIR016W	15:136324	2.64E-05
YOL084W	15:143597	3.29E-09
YPL239W	16:100527	2.72E-12
YCL048W	3:64311	0.00017212
YOR007C	12:635380	3.08E-06
YEL056W	5:44617	5.50E-07
YBL006C	2:227290	9.41E-10
YBR060C	2:388862	2.44E-09
YGR184C	7:861935	2.25E-11
YKL159C	11:146603	6.09E-16
YFL055W	6:15106	1.22E-06
YFL055W	10:34098	5.03E-15
YKR028W	2:620056	7.12E-05
YIL064W	7:707950	2.85E-05
YIL064W	9:242417	1.16E-11
YHL036W	13:99672	7.64E-06
YLR355C	3:91305	0.000110759
YLR355C	13:46084	2.21E-10
YLR355C	14:449639	2.93E-10
YDL194W	2:521415	8.12E-05
YDL194W	4:46316	2.83E-05
YBL102W	3.956944444	0.000222378
YOR060C	15:438824	1.36E-11
YGR032W	2:551299	2.05E-05
YBR114W	12:507813	2.93E-05
YJR098C	10:586311	6.53E-05
YNR036C	15:108577	8.71E-06
YNL065W	5:116530	8.15E-07
YNL065W	14:502496	3.15E-07
YGR076C	15:113254	1.29E-07
YBL009W	2:537314	9.25E-06
YNL066W	2:555596	6.41E-15
YNL066W	4:96259	0.000241293
YDL247W	10:715254	7.46E-08
YJR035W	10:503030	6.52E-17
YMR016C	2:427677	2.39E-11
YDR057W	15:113251	4.08E-05
YNL177C	15:180222	7.13E-05
YKL008C	12:634227	6.18E-09
YBR132C	2:499895	1.24E-14
YLR354C	12:852066	1.23E-09
YLR141W	12:423789	1.22E-07
YOR158W	15:180210	4.78E-06

YNR022C	15:180222	1.16E-07
YLR054C	12:247886	5.27E-07
YOR303W	15:141627	4.66E-05
YDL127W	8:111680	7.03E-05
YNL077W	1:187607	1.17E-05
YNL077W	7:375499	1.08E-15
YOR033C	2:499895	4.43E-05
YOR033C	15:357032	1.42E-05
YNL180C	14:314883	9.08E-09
YLL019C	15:174364	3.13E-06
YLL019C	16:497425	0.000125104
YJR016C	3:81832	1.55E-10
YLR099W-A	1:55215	1.98E-05
YCL049C	15:301074	0.000140311
YBR091C	2:426887	4.10E-05
YDR511W	4:1471260	4.63E-07
YDR511W	15:113251	1.72E-06
YLR395C	12:668249	7.69E-06
YLR395C	15:141627	1.01E-06
YLR395C	16:500348	1.58E-05
YOR192C	15:703771	6.83E-11
YNL128W	4:78583	0.000280142
YNL128W	14:393903	2.38E-05
YOR193W	15:703771	3.10E-15
YIL055C	9:251537	5.69E-10
YJL071W	5:272258	6.20E-12
YPL159C	12:611854	1.17E-05
YGR256W	7:1007587	1.31E-14
YJL219W	10:22315	6.48E-08
YLR353W	2:592863	1.34E-10
YLR353W	12:829265	1.90E-05
YLR273C	12:677957	1.12E-05
YJL095W	12:634227	6.06E-05
YBR203W	2:530481	1.59E-05
YBR203W	14:449639	2.23E-05
YNR028W	2:533268	5.26E-06
YPR004C	15:108577	4.51E-05
YPR004C	16:500348	1.87E-06
YGR271W	15:136324	5.59E-06
YJL145W	2:562415	7.13E-05
YHR008C	12:659357	3.17E-07
YHR008C	13:110814	7.03E-05
YIL061C	9:238345	1.18E-09
YNL155W	9:133699	0.000138295
YNL155W	13:158910	6.42E-06
YIR027C	15:842027	1.88E-05
YJL050W	8:80068	4.29E-05

YOL158C	9:19607	3.04E-05
YOL158C	13:910381	5.41E-06
YOL158C	15:44542	3.81E-06
YDL205C	4:89821	1.32E-15
YGR055W	5:272258	5.12E-09
YNL268W	2:477206	7.02E-10
YLR357W	8:95289	1.26E-05
YGL226C-A	7:73452	4.21E-14
YDR275W	12:659357	9.50E-12
YIL047C	2:562415	0.000172804
YMR031C	2:567221	5.47E-05
YMR031C	15:143597	5.92E-11
YCR030C	13:99675	3.17E-05
YCL069W	11:656099	5.39E-06
YGL129C	15:113251	8.36E-06
YOL126C	5:117056	8.02E-16
YJL178C	3.954861111	0.00014434
YOR262W	2:555575	1.16E-05
YDR017C	13:27644	4.30E-06
YJR001W	12:662627	1.17E-05
YMR266W	7:129150	2.52E-05
YMR266W	16:500342	1.01E-05
YGR030C	7:543259	3.06E-07
YOL092W	15:144659	6.20E-14
YOR388C	10:59959	6.39E-08
YDR148C	13:28622	2.94E-05
YDR148C	15:136327	1.80E-06
YPL140C	14:449639	1.41E-11
YAR015W	1:141181	5.84E-10
YEL039C	12:668249	3.16E-06
YJR065C	14:449639	1.82E-08
YDR309C	8:111690	1.38E-05
YDR309C	13:46084	7.25E-09
YMR074C	15:136324	7.00E-05
YPL003W	13:33681	2.97E-06
YPL003W	15:141633	3.56E-05
YPL091W	2:427677	0.000170871
YPL091W	16:387239	5.72E-12
YOR122C	14:410244	3.28E-05
YPL021W	16:535979	0.000132255
YAR033W	1:185122	1.54E-10
YNL006W	1:187640	1.81E-05
YNL006W	7:375499	7.29E-11
YGR002C	7:459354	2.40E-08
YCR061W	12:659357	2.10E-07
YOL045W	15:136324	9.49E-06
YOL045W	16:492351	3.30E-05

YGR247W	7:995892	1.90E-16
YPL137C	15:141633	1.04E-06
YCL017C	3:92157	2.18E-12
YGL141W	14:449639	8.18E-11
YML028W	12:644136	4.02E-05
YIR035C	12:659357	1.79E-05
YHL034C	8:46007	1.84E-05
YNL125C	14:412269	3.01E-05
YCL057C-A	15:108577	6.03E-08
YCL057C-A	16:500348	2.06E-05
YGR012W	7:457215	2.91E-11
YMR006C	13:273244	5.76E-13
YIR025W	9:403134	1.01E-12
YFR012W-A	6:168342	1.15E-07
YNL185C	15:180210	1.48E-06
YMR121C	2:673356	1.26E-05
YBL084C	2:516889	2.45E-05
YNL327W	2:555596	1.93E-15
YML024W	13:227254	2.69E-10
YER122C	12:677957	1.62E-06
YLR348C	3:90676	6.55E-11
YLR348C	13:46070	1.73E-06
YBR218C	5:99004	1.40E-05
YER116C	5:395442	2.90E-13
YDR303C	15:113251	8.92E-05
YDL231C	4:46316	1.03E-16
YIR032C	9:419722	3.02E-09
YIR029W	2:569420	2.32E-05
YJL102W	15:174364	1.33E-05
YJR091C	10:548177	1.93E-05
YDL195W	4:114155	1.56E-05
YMR305C	2:533268	8.15E-10
YJL155C	10:130933	1.03E-05
YDR326C	2:567221	5.30E-05
YDR326C	12:659357	1.85E-05
YDR326C	15:136327	7.46E-05
YDR326C	16:511406	0.000196872
YPL188W	3:201166	2.22E-09
YOL052C-A	15:174364	1.24E-06
YHL026C	8:56246	1.22E-13
YJR126C	12:611967	1.30E-05
YNR041C	15:174364	3.98E-05
YKL001C	8:152932	5.05E-05
YGL187C	15:141627	8.28E-07
YGL187C	16:500348	3.62E-06
YNL130C	8:71742	0.000125638
YNL130C	14:449639	5.17E-07

YDL084W	12:635380	9.19E-06
YDL084W	14:449639	1.54E-07
YPR101W	2:628224	3.78E-05
YOR081C	15:481586	5.29E-10
YER020W	5:196190	2.04E-16
YER020W	12:611810	5.66E-06
YLR177W	12:514835	3.25E-07
YLR177W	15:116709	6.83E-05
YDR106W	14:412758	0.000144595
YDR423C	4:1318073	1.15E-15
YNL052W	12:668249	3.59E-05
YNL052W	15:113251	1.08E-06
YNL052W	16:500342	2.60E-05
YPR035W	13:87587	5.52E-05
YPR035W	14:449639	8.68E-10
YBL019W	2:185450	7.42E-11
YJL161W	4:509811	4.94E-06
YJL161W	10:130933	7.12E-05
YMR110C	12:674651	4.77E-08
YBL035C	12:19609	0.000256472
YBL035C	15:108577	0.000151143
YJL181W	5.397222222	3.04E-05
YDR089W	4:582121	3.44E-08
YLR065C	12:288943	1.39E-09
YOR154W	15:594024	6.54E-09
YNL026W	14:571965	2.60E-06
YJL066C	15:108577	1.43E-05
YDR265W	4:975086	4.37E-10
YBR299W	7:1081945	4.04E-08
YMR250W	15:174364	1.52E-05
YHR213W-A	4.333333333	2.49E-09
YPR201W	11:655678	1.09E-09
YPR201W	16:932538	1.38E-08
YMR084W	13:437167	4.48E-07
YOR018W	12:659357	4.43E-06
YDL243C	15:180210	0.00017821
YJL183W	10:81875	3.95E-11
YPL277C	13:922258	2.38E-14
YDR073W	4:582121	2.54E-08
YGR226C	7:974640	4.74E-05
YPL249C-A	16:70853	3.79E-08
YNR019W	12:662627	3.57E-15
YBR207W	12:677957	0.000187946
YBR207W	13:910375	0.00014486
YGR062C	2:676336	6.17E-05
YGR062C	15:113251	1.06E-05
YGR248W	15:174364	2.28E-05

YER019W	5:194873	3.99E-06
YHR117W	2:499889	2.56E-05
YOL060C	15:210839	1.65E-12
YPL031C	16:492351	1.97E-09
YLR034C	13:910381	0.000197207
YLL026W	15:108577	4.72E-06
YFR007W	15:113251	2.72E-06
YGR126W	7:795132	2.98E-08
YNL201C	14:263964	1.15E-07
YBR281C	2:769107	3.87E-07
YKR070W	11:579813	1.16E-06
YOR070C	2:517365	7.25E-05
YOR313C	13:54913	6.79E-07
YGR286C	1:42639	3.71E-06
YGR286C	2:551299	7.74E-05
YGR286C	5:272258	3.46E-10
YGL173C	14:449639	1.30E-13
YLR057W	12:254693	2.42E-09
YLR057W	15:175594	0.000113706
YIL078W	14:449639	1.99E-08
YOR350C	2:380938	5.60E-05
YPL150W	13:27644	5.77E-06
YNL200C	14:254155	2.44E-07
YKL077W	12:635380	9.52E-06
YOL146W	15:47951	8.38E-11
YKL126W	11:211595	4.52E-10
YMR182W-A	2:555787	9.15E-09
YMR182W-A	4:74695	6.91E-06
YKR036C	11:508110	9.89E-14
YOR354C	15:113254	1.46E-07
YNL100W	15:113251	1.87E-06
YNL100W	16:500348	9.36E-05
YDL160C	14:449639	1.43E-12
YGL049C	7:402851	9.22E-10
YBR103W	2:477206	4.07E-12
YBR196C-B	2:616262	8.43E-15
YKR046C	12:662627	8.29E-15
YJL026W	12:508029	6.22E-11
YGL207W	7:110807	3.31E-10
YOR066W	2:620056	1.83E-05
YOR066W	8:111686	0.000129494
YIL093C	15:154177	5.63E-06
YER045C	13:33681	8.03E-06
YER045C	15:143597	5.98E-05
YML076C	13:99585	3.64E-11
YER161C	2:388862	3.44E-05
YER161C	14:449639	3.49E-08

YJL090C		3.133333333	0.000201577
YDR533C	15:144659		2.56E-15
YLR168C	12:450046		7.76E-05
YLR168C	13:28622		8.27E-06
YLR168C	15:113251		1.18E-06
YDL214C	2:615927		0.000175475
YDL214C	4:62325		1.23E-16
YKR080W	11:586769		1.57E-08
YKR080W	13:46084		8.69E-05
YOR261C	2:628224		1.44E-05
YOR261C	9:98955		0.00023243
YPL167C	16:239627		1.49E-10
YBR104W	2:427674		9.18E-05
YBR104W	15:170945		1.28E-05
YER012W	12:659357		6.63E-06
YOL136C	9:139464		3.23E-07
YOL136C	13:149075		3.47E-05
YDL234C	4:46466		7.35E-05
YIR018W	9:387979		9.82E-10
YNL124W	14:402312		3.26E-07
YPR098C	2:388862		1.34E-05
YIL116W	9:141014		1.28E-14
YPR157W	2:636958		1.88E-06
YPR157W	9:257943		3.03E-05
YJR155W	5:272255		7.74E-08
YML100W	15:143597		2.75E-06
YIL037C	8:111683		5.04E-10
YDR153C		3.95625	1.99E-05
YDR153C	14:449639		2.86E-13
YHR184W	8:389176		2.86E-05
YPL148C	16:266023		2.59E-06
YOR100C	15:488334		0.000248865
YER031C	2:553812		7.77E-05
YJR108W	10:626962		3.88E-10
YDR121W	4:744330		1.87E-07
YOR184W	2:530481		6.06E-05
YNR007C	12:668249		2.83E-05
YCR020C	8:167504		3.90E-06
YBR263W	12:607076		2.79E-05
YLR442C	8:111683		8.81E-06
YOR386W	2:582419		4.68E-07
YLR194C	12:611967		4.44E-05
YIL011W	5:350744		8.99E-11
YOL166W-A	15:44550		7.53E-06
YIL033C	9:325242		4.97E-05
YPL164C	2:555787		0.000115537
YJL159W	2:608310		2.55E-06

YJL159W	10:122312	1.49E-08
YML066C	13:129925	1.22E-13
YPL011C	2:567221	9.72E-05
YPL011C	15:108577	0.000160722
YPL011C	16:542307	4.90E-05
YNL322C	14:33361	1.13E-11
YDR522C	4:1491146	4.86E-05
YDL120W	4:246738	2.85E-07
YER173W	13:27644	7.59E-05
YER091C	5:272258	8.74E-09
YPL010W	8:389050	0.00024638
YKR017C	11:468771	3.39E-08
YMR109W	13:481542	1.71E-14
YNL284C	15:154177	1.34E-06
YJL172W	10:101193	1.88E-14
YGR222W	7:952065	1.40E-09
YJL045W	10:353027	5.05E-08
YOL166W-A	4.911111111	7.70E-08
YPR085C	16:711614	1.00E-15
YJL023C	10:453011	3.88E-06
YJR104C	12:662627	6.14E-10
YCR071C	15:113267	7.36E-07
YAR028W	1:185122	1.04E-15
YOR138C	15:594024	7.64E-10
YGL078C	14:449639	6.05E-09
YOR107W	15:136327	2.58E-07
YDR407C	14:449639	2.01E-07
YPR023C	11:219950	3.31E-06
YLR406C-A	12:927421	2.92E-09
YDL207W	8:95289	4.74E-05
YIL142W	9:84805	6.73E-09
YDL247W	10:715254	5.24E-10
YAR064W	6:43666	9.55E-08
YGR037C	1:41483	2.58E-06
YGR037C	12:679808	5.63E-05
YJL116C	2:555787	4.14E-05
YDL150W	14:449639	9.51E-12
YDR166C	15:113251	2.53E-05
YOL083W	2:519049	5.76E-05
YKL183W	11:98330	1.37E-08
YPL013C	16:523450	2.04E-05
YHR092C	9:101011	5.32E-06
YHR092C	15:143597	3.65E-05
YML128C	15:136327	6.40E-07
YEL032W	3:105042	8.13E-07
YEL032W	4:1344550	1.32E-08
YLL018C-A	15:136327	3.33E-05

YJR003C	15:116709	2.69E-06
YBR159W	2:602012	2.16E-05
YHR110W	8:95289	0.000180294
YEL023C	14:449639	4.98E-06
YDR368W	14:449639	7.75E-11
YLR157W-C	12:472165	8.38E-17
YDR004W	4:450230	1.71E-05
YPR097W	15:113251	1.44E-05
YBR115C	2:477206	2.09E-13
YPL231W	1:41483	2.64E-07
YPL231W	2:533262	6.27E-05
YOR127W	2:533268	9.94E-09
YOR127W	16:511406	5.39E-05
YCR018C	3:105042	3.89E-12
YPL187W	3:201166	8.94E-17
YOR173W	2:533262	5.50E-05
YOR173W	15:136324	8.46E-05
YPL201C	16:125271	8.33E-11
YFL002C	6:100521	1.61E-05
YER010C	2:567221	0.000197123
YDR441C	4:1344670	1.64E-12
YHR058C	8:221933	1.90E-14
YHR058C	16:126998	7.28E-05
YCR089W	8:111690	6.30E-10
YOR207C	15:113251	4.89E-05
YNL148C	13:28334	3.41E-06
YDR507C	2:565216	6.36E-11
YNL282W	2:569420	5.50E-05
YPL277C	13:910381	1.23E-12
YDR308C	8:389050	0.000200728
YJR005C-A	13:885665	7.33E-05
YNL081C	15:108577	7.78E-08
YMR223W	12:662627	2.74E-05
YOR304W	15:136327	1.49E-05
YDR351W	4:1181160	4.11E-07
YDR351W	15:141621	2.32E-05
YMR113W	13:481541	1.82E-12
YGR040W	2:567221	1.65E-12
YGR003W	7:498659	3.91E-11
YDR005C	4:433589	4.78E-12
YJL191W	3:175802	1.73E-08
YKL170W	4:527458	0.000471125
YKL170W	11:98330	1.22E-06
YKL170W	15:154177	5.25E-05
YER153C	2:555596	2.45E-11
YMR209C	13:686206	3.87E-12
YIL089W	7:708252	6.49E-09

YIL089W	9:200332	8.95E-17
YLR373C	2:513408	1.93E-05
YBR248C	2:562415	0.00017821
YBR248C	5:272258	2.17E-06
YPL257W	16:70847	2.21E-14
YDR092W	11:405226	4.70E-05
YJR131W	10:646911	1.45E-11
YJL213W	10:34098	8.13E-15
YHR183W	13:49894	3.09E-05
YNL279W	8:111683	1.18E-09
YBL052C	12:662627	5.64E-05
YAL008W	2:533268	4.03E-05
YGL033W	3:209932	6.46E-08
YHR068W	14:449639	9.30E-05
YFR041C	6:239482	4.69E-10
YMR155W	13:556841	2.73E-05
YER048W-A	5:218250	1.87E-05
YHR015W	8:137221	2.00E-14
YER050C	9:238345	0.000139271
YGL053W	1:187640	1.38E-08
YGL053W	7:403626	4.90E-17
YBL045C	13:130069	9.72E-05
YBL045C	15:141627	3.15E-06
YBL045C	16:500342	3.61E-05
YGL116W	8:111680	1.71E-06
YPR009W	15:143597	6.18E-05
YPR009W	16:500348	1.20E-06
YDR462W	15:174364	4.18E-06
YPL230W	2:562415	3.69E-06
YDL220C	4:54225	2.47E-06
YLR153C	12:662627	9.92E-15
YLR203C	15:174364	5.19E-06
YMR035W	14:449639	9.34E-09
YDR389W	2:569420	7.12E-05
YNL326C	8:111679	5.74E-05
YBL064C	13:285840	1.73E-06
YEL035C	12:662627	1.13E-05
YEL035C	13:178636	3.95E-05
YKL207W	3.953472222	0.000119115
YKL207W	14:449639	7.45E-10
YDL202W	15:113251	3.62E-06
YJR093C	10:602943	2.32E-09
YMR038C	12:668249	1.93E-10
YJL174W	2:507428	9.86E-07
YJR070C	10:570345	7.52E-06
YJL020C	10:393255	1.44E-06
YDL226C	4:46466	1.47E-15

YFL025C	6:80521	1.59E-06
YOR283W	15:842027	6.68E-16
YJL132W	10:159479	1.96E-14
YCR021C	1:187607	0.00011899
YGL223C	7:73452	0.000191095
YGL223C	8:80068	0.000141348
YGR138C	9:101011	0.000212887
YMR099C	2:419093	4.12E-05
YMR099C	14:449639	2.93E-10
YPR115W	8:111686	1.14E-07
YDL020C	12:659357	4.77E-06
YJL060W	2:419093	6.24E-05
YJL060W	10:387893	9.01E-07
YML116W	13:46084	5.33E-11
YFL027C	3:201166	3.84E-16
YIL172C	9:19607	9.02E-05
YIL172C	13:100048	1.22E-06
YFR003C	8:63314	4.98E-05
YBR042C	12:662627	9.39E-08
YFR013W	6:174552	2.45E-08
YFR013W	15:136324	1.44E-05
YCR075C	14:449639	9.28E-07
YBR068C	2:420366	1.86E-08
YBR068C	3:81832	1.65E-13
YIL024C	2:517123	1.49E-08
YHR136C	13:27644	1.61E-14
YDR493W	15:180222	1.06E-05
YML002W	13:273244	4.33E-16
YJL117W	1:139312	0.000329484
YJL117W	13:27644	6.70E-14
YBR122C	15:113254	4.23E-06
YMR027W	14:449639	1.26E-11
YLR229C	2:537314	2.60E-08
YBR026C	2:301671	1.66E-15
YPR075C	2:516889	1.79E-05
YKL109W	13:49894	1.81E-05
YKL109W	15:141627	8.30E-09
YKL109W	16:500348	3.37E-06
YHR033W	8:167506	9.24E-17
YOR334W	12:674651	6.63E-08
YLR301W	15:143597	5.86E-07
YMR198W	2:551299	1.90E-05
YEL022W	5:117046	6.45E-06
YPL028W	12:659357	2.34E-11
YDR047W	15:136327	4.33E-05
YOL135C	12:662627	5.12E-05
YHR137W	2:376145	7.10E-05

YHR137W	16:453442	5.90E-06
YLR438W	13:49894	3.97E-14
YNL164C	2:628224	3.09E-05
YHR007C	8:98513	2.65E-06
YHR007C	12:683457	9.77E-06
YHR007C	15:179289	2.84E-05
YCR004C	13:77684	6.11E-05
YCR004C	15:136327	3.22E-05
YKR051W	12:644082	2.08E-05
YDL040C	14:449639	1.52E-09
YMR065W	8:111683	5.26E-11
YDL003W	4:446125	9.29E-05
YIL014C-A	9:325320	1.15E-16
YDR182W-A	4:818354	1.25E-15
YER083C	3.953472222	0.000214555
YLR241W	12:659357	8.56E-05
YDR161W	4:782114	7.22E-11
YOR267C	4:114155	5.98E-05
YHR074W	14:449639	6.24E-11
YDR260C	2:567221	1.70E-05
YNL146W	3:201166	1.15E-16
YGL262W	7:10158	2.53E-08
YKL050C	2:530481	4.37E-05
YKL137W	11:180221	5.80E-07
YOR222W	13:96015	2.03E-05
YNL230C	12:668249	0.000170121
YML038C	13:922256	9.01E-05
YGL226W	3:91305	0.000167294
YGL226W	7:73452	2.42E-10
YOL013C	13:268045	3.65E-05
YDL073W	2:565216	0.000356304
YDL073W	12:662627	1.13E-05
YDR151C	7:375499	2.81E-08
YDR342C	13:27644	9.23E-06
YDR342C	15:174364	1.84E-08
YOR296W	2:548401	1.01E-10
YBR107C	2:477206	2.15E-14
YKR025W	7:141949	2.41E-05
YEL024W	12:668249	5.00E-09
YEL024W	15:113251	1.44E-05
YCR096C	3:201166	1.15E-16
YLR389C	12:909226	5.39E-14
YPR153W	15:438826	3.93E-05
YNL215W	8:80068	8.21E-06
YPL109C	2:616262	0.000215718
YFL007W	6:143937	0.000103182
YPR161C	14:418269	2.33E-05

YJR092W	15:116709	8.90E-05
YJR092W	16:500342	0.000135119
YJL098W	13:99672	1.85E-05
YPR088C	14:412758	3.57E-05
YBR236C	2:697894	2.65E-14
YGR220C	15:174364	3.09E-07
YLR059C	12:260119	4.52E-10
YER174C	5:556725	8.00E-05
YHR009C	8:137227	2.54E-05
YHR009C	15:136327	7.00E-05
YLL001W	15:154177	0.000182672
YDL022W	15:154177	2.93E-05
YNL207W	14:258590	4.02E-09
YMR073C	2:567221	0.000154628
YMR073C	9:133693	0.000208126
YIL124W	15:136327	2.91E-06
YOR162C	15:632882	2.80E-13
YML120C	15:113251	7.37E-07
YDL183C	15:116709	8.33E-05
YDL019C	15:143597	1.09E-07
YIL153W	9:47053	4.48E-06
YDR194C	15:180222	7.13E-05
YDR264C	4:975086	2.69E-14
YMR018W	3:92013	1.25E-05
YDR494W	15:154177	2.53E-05
YNL003C	12:659357	5.44E-10
YOR163W	13:27644	1.04E-11
YML027W	13:234545	3.82E-05
YPL070W	16:497425	8.29E-05
YEL076C-A	12:1067122	2.08E-05
YNL117W	14:402312	1.13E-06
YNL117W	15:180222	1.56E-05
YPR124W	12:611854	6.02E-05
YMR300C	13:46084	2.87E-09
YBR184W	3:105042	0.000161834
YLR288C	12:708594	6.25E-14
YNR068C	13:46084	7.88E-08
YBR006W	2:246129	2.33E-13
YLR361C-A	12:851826	3.05E-14
YPL172C	15:154177	8.39E-07
YDL059C	13:27644	2.91E-05
YLR093C	12:327131	1.04E-14
YAL065C	1:16984	2.48E-12
YOL119C	15:154309	1.84E-06
YDL144C	2:533262	6.00E-06
YJL210W	15:136327	3.02E-06
YJL210W	16:500342	3.37E-06

YDL122W	14:449639	2.03E-10
YNL094W	12:705226	0.000161977
YDL006W	2:562415	8.65E-05
YML071C	13:159533	4.66E-06
YGR065C	5:272255	2.02E-09
YJL035C	10:380085	2.07E-16
YGL243W	7:44996	3.85E-08
YGL150C	12:705100	5.76E-05
YKL041W	11:354466	4.20E-15
YGR016W	2:382536	2.56E-05
YMR272C	15:144659	4.41E-12
YGR006W	7:506254	2.19E-05
YHR076W	2:533262	3.58E-06
YHR142W	2:562415	5.42E-13
YHR142W	16:500348	3.07E-05
YHL009W-A	8:98513	3.28E-07
YHL009W-A	16:445372	8.07E-09
YER043C	12:677957	3.21E-07
YPL022W	16:511406	1.13E-11
YIL088C	9:214482	7.55E-06
YAL049C	1:52943	1.69E-12
YER047C	5:244117	2.64E-15
YLR084C	2:555575	0.000117694
YLR084C	16:497425	0.000121244
YPL098C	15:113251	0.000106085
YMR090W	15:175594	0.000127119
YIL077C	15:108577	1.37E-06
YIL077C	16:500342	2.36E-05
YMR232W	8:95469	8.12E-06
YMR002W	15:108577	1.12E-05
YMR136W	15:136327	3.04E-11
YDR116C	15:154177	7.06E-07
YIL111W	13:99675	4.20E-06
YBR234C	2:697894	1.54E-11
YNL239W	2:567221	6.86E-05
YER159C	8:95289	0.000119315
YIL136W	15:141621	6.73E-09
YDL107W	4:273840	5.27E-07
YDR226W	12:679808	8.88E-05
YPL271W	15:141627	7.96E-07
YPL271W	16:500348	3.34E-07
YOR367W	14:449639	1.33E-10
YHR047C	3:90610	6.85E-05
YHR047C	13:46084	2.34E-07
YPR159W	2:517365	3.43E-07
YNL034W	14:571965	5.93E-13
YLR412W	12:956534	3.84E-12

YBR039W	15:136327	4.50E-06
YBR039W	16:500342	7.64E-06
YNL010W	12:644082	6.40E-10
YML013W	14:449639	2.72E-07
YMR013W-A	15:136324	0.000134599
YLR088W	14:412272	2.80E-05
YGL202W	7:105279	1.85E-09
YFR053C	15:174364	9.42E-06
YNL048W	14:554606	5.57E-09
YMR315W-A	13:910381	4.43E-09
YOR317W	1:55221	0.000206427
YOR317W	13:28694	1.70E-05
YOR317W	15:113251	9.24E-05
YPL268W	1:139312	0.000163941
YPL268W	13:27644	1.83E-12
YLR254C	12:644136	6.11E-05
YCL073C	11:656099	3.75E-16
YBL098W	12:689211	0.000256764
YGL146C	13:922258	7.91E-06
YGL146C	15:174364	4.68E-05
YHR083W	2:551299	6.39E-05
YLR156W	12:472165	2.74E-16
YBR225W	8:95469	9.21E-05
YHR042W	8:193175	1.67E-08
YBR189W	2:565216	6.01E-07
YCR044C	12:611810	3.72E-05
YHR150W	14:449639	1.60E-05
YGR038W	7:553877	5.65E-15
YMR070W	12:672779	1.06E-08
YBR001C	2:216978	1.38E-07
YNL302C	5:225504	8.00E-05
YIL056W	15:136324	2.79E-07
YHR190W	12:662627	1.10E-11
YOR358W	15:108577	3.65E-06
YPL163C	2:555787	5.37E-08
YOR149C	12:705226	2.83E-06
YIL137C	3.954861111	9.43E-05
YIL137C	14:449639	8.39E-13
YGL245W	14:449639	0.000191855
YDR510W	10:572479	0.000113081
YDR510W	11:390168	0.000268552
YNR006W	12:611854	1.25E-05
YPL273W	13:922258	1.75E-14
YKL157W	2:419093	8.19E-05
YKL157W	14:449639	1.70E-12
YHR021W-A	8:150330	3.37E-10
YNR009W	13:27644	4.63E-06

YNR009W	15:136324	2.24E-05
YNR009W	16:500342	9.07E-05
YDR304C	8:80014	2.16E-05
YGR013W	2:533268	0.000183011
YGR013W	7:529376	4.59E-05
YMR289W	13:849969	2.51E-14
YNL046W	2:555596	1.90E-11
YNL046W	3.953472222	0.000114596
YNL046W	14:547071	9.51E-07
YNL145W	3:201166	8.94E-17
YLR409C	13:28334	8.92E-05
YML126C	12:659357	1.08E-14
YHR061C	2:551299	1.58E-05
YHR061C	8:111679	1.84E-06
YMR102C	5:395442	2.27E-06
YJR077C	13:28622	9.70E-05
YJR077C	15:136327	1.24E-08
YJR077C	16:500342	1.74E-05
YPR188C	4:141608	3.06E-05
YPR040W	12:659357	1.39E-07
YGL208W	7:110807	2.38E-11
YOR131C	15:571103	1.00E-11
YIL126W	9:74540	7.15E-05
YGL191W	12:668249	2.06E-08
YGL236C	15:154177	3.04E-05
YGR180C	12:450046	4.24E-07
YAL061W	15:108577	0.000176168
YJR036C	14:412758	1.22E-05
YLR426W	12:987750	2.80E-14
YKL218C	2:567221	1.08E-05
YKL218C	11:12706	2.67E-06
YMR034C	13:328865	6.51E-12
YBR072C-A	15:469823	3.28E-05
YIL050W	3:105042	1.36E-06
YIL050W	13:46084	8.82E-07
YDR132C	2:602012	5.49E-05
YDR216W	5:321708	0.000169338
YDR216W	15:144659	2.91E-07
YLR281C	12:705190	4.77E-09
YBR069C	2:376872	5.59E-12
YNL071W	14:449639	4.27E-06
YDR204W	2:426887	5.15E-05
YDR204W	14:449639	8.90E-09
YML081C-A	15:108577	2.50E-07
YML081C-A	16:511406	0.00022227
YDR461W	3:201166	8.94E-17
YFR029W	5:272258	8.81E-08

YFR029W	7:708034	0.000122738
YHR162W	13:99675	1.21E-09
YHR162W	14:449639	2.97E-07
YDR122W	4:733875	3.73E-07
YNL262W	14:449639	2.55E-12
YIL118W	2:628224	2.12E-05
YOR245C	2:551299	0.000258442
YFL016C	7:375499	2.94E-08
YFL016C	15:113254	9.47E-05
YOR123C	8:95289	3.26E-05
YGR250C	7:995892	2.11E-11
YDR077W	2:553812	2.14E-05
YDR399W	13:99675	0.000156657
YJL204C	10:51003	3.44E-07
YJL204C	12:644136	0.00019098
YMR062C	2:567221	0.000257955
YHR216W	6:28029	3.72E-06
YHR216W	13:159533	1.02E-07
YJL077W-B	2:551299	2.89E-07
YKL090W	11:266017	3.81E-14
YKR030W	2:679703	1.89E-05
YOL101C	1:42591	3.77E-09
YOL101C	7:402879	3.80E-05
YOR348C	12:668249	2.65E-05
YOR348C	15:136327	5.39E-06
YIL158W	9:47053	2.51E-14
YGL067W	7:375499	9.06E-10
YPL247C	15:174364	2.16E-07
YDR256C	2:517123	6.04E-06
YFR026C	2:372509	4.44E-06
YFR026C	6:205881	5.86E-12
YDR124W	8:111680	0.000181786
YDR411C	13:96015	0.000131621
YKR006C	15:154177	7.12E-08
YAL063C-A	1:10152	3.52E-10
YER044C-A	13:99672	2.68E-05
YLR100W	12:662627	1.72E-13
YGL017W	7:459354	7.39E-06
YGL017W	12:642137	5.30E-06
YLL066W-B	4:1511257	1.08E-11
YLL066W-B	12:1056097	5.44E-08
YLR431C	13:28334	9.56E-05
YBL029C-A	2:175071	2.10E-06
YBL029C-A	15:136327	0.000284034
YBL099W	15:136327	1.28E-06
YBL099W	16:500342	1.42E-05
YNL042W	4:297977	9.88E-06

YLR372W	12:662627	7.52E-05
YPL147W	16:275840	2.35E-07
YOL113W	15:106272	9.92E-10
YFL010C	6:104931	1.21E-09
YLL055W	12:35970	8.93E-10
YNL039W	14:558284	6.85E-05
YMR267W	15:113254	7.11E-05
YOR188W	2:567221	3.02E-07
YNL218W	12:705100	0.000330928
YLR058C	11:508110	1.62E-05
YMR181C	2:506661	3.58E-06
YMR181C	3.954166667	0.000155316
YGL021W	7:457215	1.66E-05
YGL021W	16:462646	8.59E-07
YPR196W	16:927502	9.04E-06
YNL053W	2:567221	1.29E-06
YCL038C	13:27644	7.44E-06
YNL241C	3:92247	9.49E-07
YNL311C	14:33361	2.99E-07
YBL043W	2:142262	2.16E-07
YNL323W	14:33361	1.05E-08
YDR408C	13:57145	7.89E-05
YHR090C	13:99672	0.000343426
YHR090C	16:495156	6.56E-06
YOR208W	1:55221	9.88E-05
YOR208W	2:506661	3.09E-06
YNL007C	1:187519	2.98E-05
YNL007C	7:375499	7.81E-15
YIL022W	15:106158	5.84E-05
YCL052C	3:105042	3.82E-06
YER017C	15:170945	1.52E-05
YGL209W	4:219120	3.55E-05
YGL209W	9:139462	4.27E-05
YML055W	13:163328	1.46E-13
YDL131W	2:477206	1.68E-07
YDL131W	4:226317	5.64E-10
YKL210W	11:46633	2.24E-05
YCL014W	2:516889	1.65E-05
YCL014W	15:113251	4.71E-05
YHL048W	6.176388889	4.90E-17
YML111W	13:49894	5.74E-13
YOL075C	13:410287	3.78E-06
YKL189W	8:111683	1.43E-06
YJL151C	10:130933	6.10E-09
YAR018C	2:553812	8.76E-07
YNL102W	3.956944444	4.94E-05
YNL102W	14:449639	2.87E-12

YOR324C	12:644136	4.49E-06
YOR324C	15:854265	5.08E-06
YNL187W	13:49894	1.87E-05
YNL231C	12:662627	4.08E-05
YDL037C	4:369365	2.24E-07
YDR171W	7:375499	7.48E-06
YDR171W	15:116709	0.000192054
YOL163W	15:10427	4.75E-17
YDL154W	4:183009	1.19E-05
YBR120C	15:113254	1.33E-05
YMR011W	5:395442	1.02E-07
YBL093C	12:659357	0.000117497
YPR129W	12:733838	0.000129568
YDR322W	15:174364	6.50E-07
YKL093W	11:266017	1.22E-07
YKL093W	16:511400	4.21E-06
YDR443C	4:1344670	3.25E-12
YOR382W	13:910381	1.90E-06
YLR332W	2:537314	2.92E-09
YDR019C	13:115474	9.43E-05
YCR008W	12:705100	0.000101997
YNL317W	13:69114	8.10E-06
YNL317W	14:38762	1.21E-08
YDL164C	4:165032	2.46E-09
YIL068C	8:137233	9.89E-05
YBL071C-B	2:519049	2.35E-07
YJR129C	10:646911	4.99E-11
YAL040C	1:52943	9.77E-06
YJL156C	10:122312	3.18E-06
YJL156C	15:589013	0.000109896
YJR005W	2:537314	6.94E-06
YJR005W	10:450212	1.92E-06
YPL038W-A	16:489143	1.56E-11
YER075C	2:548401	2.32E-08
YOR374W	13:99675	9.99E-11
YGL028C	2:567221	5.03E-16
YGR154C	7:913059	0.000149159
YOL095C	15:141627	5.47E-11
YBR161W	3:81832	0.000244344
YBR161W	12:644136	6.43E-07
YPR199C	11:656099	5.75E-17
YGR230W	12:719857	3.65E-05
YKR058W	11:540588	4.40E-12
YER076C	5:321714	8.69E-07
YKR095W-A	3:90676	1.60E-07
YKR095W-A	15:180961	5.93E-06
YML100W-A	15:144659	2.87E-05

YJL217W	10:23505	1.12E-16
YFL053W	6:28041	6.51E-12
YFL042C	14:410244	8.09E-05
YOL016C	13:28334	1.25E-05
YHL047C	5.218055556	4.75E-17
YGR236C	7:940716	1.27E-06
YFL021W	2:555575	3.55E-12
YFL021W	6:101519	1.94E-05
YER087W	15:174364	7.77E-05
YBR061C	2:376145	1.40E-07
YHR205W	2:533268	7.44E-05
YIL129C	14:449639	4.19E-08
YKL028W	11:394660	1.83E-09
YML130C	7:375499	8.23E-08
YGR229C	7:948578	7.20E-07
YDR487C	4:1474485	2.80E-06
YHR094C	4:217351	9.95E-10
YOR226C	3:81832	3.21E-14
YBR123C	2:477206	1.55E-05
YDL017W	4:465157	6.36E-06
YLR449W	12:1031685	1.35E-06
YLR361C	4:446125	8.71E-05
YLR361C	12:697260	8.20E-06
YKL107W	11:229040	1.50E-07
YLR216C	14:412269	0.0001023
YOR211C	13:79786	6.20E-05
YPL256C	8:111679	5.54E-05
YLR377C	12:872448	2.02E-10
YCL005W-A	12:472165	0.000338225
YCL005W-A	15:515923	4.96E-05
YMR251W	13:910741	5.52E-05
YDR011W	12:672779	4.77E-06
YDR011W	15:632894	2.47E-11
YPR119W	14:568280	0.000118113
YBR073W	2:533262	5.23E-08
YEL037C	5:79647	2.21E-15
YGL205W	7:110807	7.24E-12
YNL138W	14:449639	3.82E-12
YPL104W	15:174364	2.64E-07
YGR206W	7:905017	1.32E-05
YKL029C	3:91305	4.96E-08
YKL029C	11:382554	2.40E-06
YOL070C	15:205104	0.000121719
YPR007C	16:618575	1.07E-07
YHR080C	15:143597	1.19E-06
YIL169C	9:27026	2.59E-10
YIL169C	6.038194444	7.17E-05

YCL026C-B	3:75021	6.70E-15
YML058W-A	12:672779	1.91E-06
YJR102C	10:627628	5.35E-05
YER090W	5:272255	5.76E-08
YNL246W	14:191243	3.55E-12
YMR296C	2:516889	0.000238395
YAR042W	1:201039	1.59E-15
YAR042W	8:525664	9.42E-05
YMR127C	2:565216	0.000104759
YHL017W	12:681096	7.48E-05
YHL017W	14:449639	7.04E-06
YKL088W	4:46316	5.97E-06
YOL137W	15:59733	7.94E-08
YIL048W	9:251537	1.68E-10
YPR063C	13:130069	4.65E-05
YPR063C	16:492351	9.45E-06
YHR084W	8:111690	0.000170871
YNR015W	14:412269	8.47E-06
YPR054W	2:582419	2.82E-05
YNL131W	15:154177	8.21E-07
YMR100W	12:677957	4.52E-08
YOL057W	2:368060	7.08E-05
YBL032W	2:372509	3.51E-06
YPL135W	5:321618	1.00E-04
YPL135W	13:49894	7.42E-05
YPL135W	15:108577	9.77E-05
YOR286W	15:113251	4.18E-07
YNR049C	12:659357	2.79E-05
YOL056W	15:170945	4.67E-07
YGR289C	7:1075580	9.85E-17
YGL037C	15:174364	7.92E-06
YBR013C	2:256896	8.34E-16
YNL058C	14:525061	1.68E-11
YKL152C	2:391856	9.78E-06
YDR503C	8:92978	3.04E-05
YOR065W	12:668249	1.65E-08
YOR065W	16:500342	3.73E-05
YPL236C	15:113251	3.93E-05
YLR290C	12:705226	1.70E-07
YBR080C	2:419093	9.35E-06
YBR080C	14:449639	1.52E-09
YGL032C	3:201166	8.94E-17
YER001W	15:141627	1.36E-08
YER001W	16:511406	0.000190963
YPL052W	9:133693	2.38E-05
YPL052W	15:170945	3.09E-05
YLR455W	12:662627	6.41E-05

YKL101W	11:247944	1.40E-07
YBR274W	2:750838	1.13E-14
YLL041C	15:136327	1.02E-06
YLL041C	16:500348	8.53E-05
YGR147C	2:565216	8.88E-06
YJR133W	15:116709	9.19E-05
YBL059W	15:108577	0.000187229
YLR375W	15:106164	2.19E-05
YBL091C	14:449639	1.13E-11
YKL155C	15:174364	3.25E-06
YOR383C	13:390357	7.80E-07
YIL148W	9:74540	4.85E-15
YMR207C	15:44496	5.46E-05
YDR513W	4:1456748	5.00E-10
YGL060W	8:93002	1.27E-06
YHR179W	12:662627	1.47E-06
YHR179W	15:144659	2.96E-08
YJL006C	10:422538	8.28E-08
YDL092W	4:143910	1.36E-05
YPL046C	16:462646	9.18E-08
YNL245C	14:191243	4.04E-11
YOL159C	9:19607	1.45E-14
YLR443W	2:517365	6.79E-05
YOL164W	15:10427	4.75E-17
YLL010C	12:126934	1.70E-15
YBR272C	2:746476	1.16E-08
YCR106W	6.628472222	1.53E-06
YCR106W	0.868055556	1.03E-07
YCR106W	12:1054278	4.82E-11
YNL274C	8:457580	1.42E-10
YNL274C	15:113267	0.000193001
YNL004W	14:449639	4.55E-14
YER048C	2:530481	0.000125515
YER048C	14:449639	4.95E-07
YOL014W	15:298710	3.52E-16
YLR228C	16:500342	0.000161896
YER155C	8:111682	6.94E-07
YGR153W	7:794884	5.50E-09
YKL095W	12:19609	8.83E-05
YKL095W	15:136324	2.82E-05
YNL173C	2:567221	0.000273678
YNL173C	12:662627	5.38E-07
YNL173C	13:28622	2.85E-05
YGL103W	1:187640	4.83E-05
YGL103W	7:311205	6.10E-12
YGR109W-B	7:708028	1.14E-14
YGR109W-B	8:92960	0.000192505

YGR109W-B	9:200332	1.47E-14
YKL052C	15:144659	5.21E-14
YDL126C	4:201395	1.40E-06
YDL126C	14:449639	1.82E-10
YLR019W	12:679808	7.56E-05
YDL246C	14:394161	2.78E-05
YJR080C	15:154177	1.80E-06
YAL056W	1:41483	2.69E-15
YER188C-A	5:568698	3.98E-14
YNL315C	15:174364	2.81E-06
YFL038C	14:449639	1.24E-07
YNL316C	14:38762	4.21E-14
YML114C	2:388862	1.54E-05
YMR258C	13:99672	9.05E-05
YAL044W-A	2:537314	2.07E-05
YDL190C	2:420366	3.30E-05
YDL190C	14:449639	2.86E-13
YGR121C	2:569414	0.000117175
YPR138C	13:46084	5.94E-07
YOR281C	4:46466	3.33E-05
YOR109W	15:496730	5.19E-07
YFR012W	6:168342	6.63E-07
YBR170C	2:567221	1.32E-12
YBR170C	16:500348	6.88E-06
YHR213W	1:23780	8.46E-09
YOL089C	15:143597	4.94E-11
YOL159C-A	9:33795	1.87E-08
YOL159C-A	3.133333333	1.01E-09
YKR097W	11:632952	1.34E-06
YMR312W	13:885665	3.21E-13
YLR176C	7:375499	0.000189733
YLR176C	12:514835	3.98E-09
YIL119C	13:328865	1.38E-10
YNL134C	14:371953	2.82E-05
YNL134C	15:143597	5.77E-13
YBR148W	2:537314	4.05E-13
YHR028C	8:167504	6.61E-13
YLR250W	2:555575	8.51E-06
YLR044C	7:707950	0.000129737
YIL049W	9:268412	5.76E-08
YIR003W	9:133693	6.79E-05
YOR230W	2:555575	3.13E-06
YDL193W	4:114155	7.68E-05
YJR019C	15:136327	8.73E-05
YJR019C	16:500348	7.53E-05
YOL147C	15:108577	1.65E-07
YIL051C	13:49894	8.05E-16

YBL029W	3:91305	3.93E-05
YAR071W	13:27644	2.60E-14
YIL152W	9:74540	1.36E-14
YGL075C	7:375499	1.11E-10
YHR102W	13:46084	7.40E-05
YOR027W	7:375499	1.56E-09
YDR229W	12:644136	0.000100886
YDR229W	13:69114	2.53E-05
YBR085W	12:635380	9.41E-07
YBR085W	15:154177	3.52E-05
YBR085W	16:497425	6.61E-05
YCL012C	3:100213	2.41E-08
YML119W	13:69114	3.86E-07
YHR153C	8:389050	3.02E-05
YDR529C	12:668249	3.82E-06
YDR529C	13:28622	0.000241748
YDR529C	15:141627	3.58E-05
YDR529C	16:500348	7.77E-05
YDR446W	8:80068	5.00E-06
YKL141W	13:28622	4.19E-06
YKL141W	15:136327	1.19E-06
YKL141W	16:500348	3.17E-05
YIL065C	7:707950	1.26E-06
YIL065C	9:200332	2.98E-09
YNL137C	15:180210	6.77E-07
YBL038W	2:143721	4.07E-07
YJR148W	3:100213	2.47E-05
YKL197C	11:85465	1.88E-07
YGL181W	13:910381	4.26E-05
YOR023C	15:409778	2.90E-06
YIL082W-A	7:708028	2.14E-14
YIL082W-A	8:92960	0.000154965
YIL082W-A	9:200332	1.97E-14
YBR156C	2:555787	6.28E-14
YER118C	2:537314	5.65E-11
YPL110C	13:27644	4.70E-14
YGL009C	3:81832	3.90E-15
YGL009C	15:180222	0.000241738
YKR093W	13:46070	6.97E-08
YKR093W	15:141633	2.70E-09
YML047C	4:963733	9.69E-06
YML047C	8:111683	4.16E-10
YHR098C	8:313108	1.44E-05
YGL184C	8:167504	5.06E-11
YNR069C	13:46084	1.20E-05
YBR035C	13:124876	3.47E-05
YLL042C	12:63866	1.01E-13

YBR127C	14:449639	4.86E-09
YHR013C	8:137227	1.25E-06
YOR186W	15:703771	5.65E-07
YER029C	5:210999	3.55E-11
YHR029C	8:167504	7.96E-06
YIL043C	12:697260	1.01E-05
YGL139W	12:697260	3.28E-05
YMR174C	2:567221	2.37E-06
YDR031W	2:388862	5.09E-05
YDR173C	4:812947	1.03E-06
YKL215C	3.954861111	0.000174535
YCR107W	6.628472222	6.84E-08
YCR107W	0.868055556	6.30E-08
YCR107W	12:1054278	5.01E-09
YOR201C	15:715809	1.81E-06
YGL230C	2:569420	9.78E-05
YIL031W	2:516889	0.000151443
YIL031W	12:634227	1.54E-05
YOL164W-A	15:16691	8.70E-16
YLR219W	15:113251	5.93E-07
YPR058W	3:201167	3.18E-06
YPR158W	1:187607	0.000151377
YPR158W	7:375499	1.23E-11
YER057C	13:33681	4.90E-08
YOR111W	15:563943	7.02E-05
YBR288C	4:1213416	3.36E-05
YJL034W	8:410845	0.000135865
YJL034W	12:705100	9.58E-05
YNL278W	8:111686	3.27E-06
YNL278W	13:115474	0.000148555
YER130C	2:489202	2.72E-05
YOL091W	15:144659	1.15E-16
YIL067C	7:375499	1.40E-05
YGR067C	2:567221	0.000250415
YKL182W	1:42633	3.19E-06
YKL182W	2:516889	1.33E-06
YFL031W	2:530481	7.60E-06
YFL031W	3:100213	5.71E-05
YGL194C	7:139851	9.05E-11
YKL145W	14:449639	9.11E-11
YIL155C	15:113260	1.64E-07
YIL155C	16:500342	0.000116052
YGR243W	13:57145	8.82E-07
YGR243W	15:141627	3.65E-06
YLR155C	12:472165	4.77E-16
YBR072W	2:380938	2.09E-08
YJL027C	10:393261	3.08E-08

YJR115W	7:129150	4.61E-05
YLR285C-A	2:555787	1.01E-14
YJL005W	14:449639	2.15E-09
YML004C	13:298193	5.06E-09
YPL249C	14:449639	1.01E-10
YFR047C	2:514035	4.81E-05
YFR047C	14:486861	9.55E-09
YHR107C	2:530481	2.63E-06
YHR107C	14:449639	1.24E-05
YFR046C	6:244292	6.21E-06
YBR276C	13:91085	4.03E-05
YPR154W	12:659357	1.26E-05
YDR058C	4:582121	2.70E-12
YDL180W	4:164444	3.63E-07
YLL021W	15:113261	3.59E-05
YOR085W	12:705226	1.70E-07
YKL216W	5:117056	4.63E-16
YPR095C	12:659357	3.88E-07
YLR380W	12:644136	7.80E-07
YLL054C	2:519049	1.46E-05
YDR366C	4:1213416	3.39E-10
YGR278W	7:1051340	2.27E-09
YMR295C	15:106266	1.06E-07
YDR180W	15:113251	7.23E-05
YPR198W	11:656099	6.52E-17
YLR042C	2:562409	8.95E-15
YOR051C	15:438828	3.06E-09
YIL063C	9:214482	1.44E-09
YJL107C	2:555787	3.30E-06
YJL107C	12:659357	7.39E-06
YDR263C	4:975086	1.12E-13
YMR270C	2:565216	7.86E-05
YMR129W	2:530481	0.00017603
YMR032W	2:562415	1.18E-05
YGR096W	2:636958	3.22E-05
YDR179C	15:116709	9.19E-05
YAR075W	1:229090	1.98E-12
YPL265W	13:46084	3.12E-07
YPL265W	15:141633	2.94E-05
YMR020W	12:514835	2.16E-05
YMR020W	13:255486	2.31E-05
YKL087C	15:113251	3.83E-07
YDR357C	4:1213416	4.27E-12
YIR005W	15:116709	5.23E-05
YMR072W	15:108577	2.31E-05
YER067W	13:49894	7.90E-05
YER067W	16:500342	8.53E-05

YMR173W	2:551299	1.10E-06
YPR024W	12:697260	3.50E-05
YBL106C	12:634226	6.46E-05
YNL035C	14:571965	1.19E-16
YCR105W	6.628472222	1.14E-08
YCR105W	0.868055556	6.17E-07
YCR105W	12:1056103	1.77E-07
YOR304C-A	15:889464	1.71E-14
YJR107W	10:627628	3.14E-09
YER049W	12:611810	3.60E-05
YLR144C	2:419093	3.34E-05
YLR144C	14:449639	1.34E-06
YOL073C	15:174364	3.66E-08
YJL062W-A	10:380085	5.08E-06
YJL062W-A	15:108577	7.66E-07
YHR210C	12:634227	1.19E-06
YHR210C	15:136324	2.42E-06
YHR210C	16:511406	2.68E-05
YJL013C	10:404508	6.27E-12
YBR045C	2:328489	5.74E-17
YER074W	15:488341	8.41E-06
YJR043C	10:499854	1.29E-05
YER107C	5:422612	1.11E-07
YLR312C-B	2:533262	2.46E-06
YOR280C	16:486637	7.31E-05
YGR100W	2:530481	2.65E-05
YBL030C	15:136327	3.99E-08
YIL007C	2:676336	9.77E-05
YIL007C	12:662627	2.30E-09
YPR113W	12:677957	8.50E-07
YOR227W	3:91287	1.21E-05
YBL002W	2:569420	0.00017075
YKR045C	12:634227	2.18E-06
YER145C	12:668249	2.01E-05
YER145C	13:910381	1.28E-05
YPL014W	13:99675	2.69E-05
YPL014W	15:174364	5.98E-06
YPL014W	16:555416	5.76E-05
YCR086W	12:662627	9.38E-05
YJL170C	3:201166	8.94E-17
YJR127C	10:646911	3.35E-09
YJR127C	15:141633	2.54E-07
YCR046C	15:174364	3.09E-07
YPL191C	12:679808	7.81E-05
YPL191C	13:238291	8.82E-06
YNR059W	13:28334	0.00013917
YNR059W	15:150651	7.96E-06

YNL040W	2:513408	9.53E-07
YNL040W	14:591237	4.50E-12
YHR151C	15:113261	3.59E-05
YPL008W	12:659357	6.00E-05
YKL179C	2:555778	4.40E-08
YKL179C	16:500348	0.000143543
YGL106W	8:92978	1.40E-06
YHR039C	12:662627	1.88E-12
YGL241W	14:449639	4.59E-08
YJL198W	12:644136	8.51E-06
YPL064C	16:428900	2.62E-09
YML109W	2:555596	1.10E-08
YDR277C	13:27644	8.90E-06
YDR277C	16:500342	3.50E-05
YGR251W	7:995892	7.21E-15
YNL300W	15:174364	6.19E-06
YDR042C	4:527458	2.64E-08
YJL157C	8:111683	4.63E-12
YDL209C	4:70901	0.00016693
YGR105W	15:116709	3.72E-05
YNL151C	12:659357	0.00012908
YGR138C	12:659357	6.16E-06
YGR138C	16:511406	2.57E-07
YOR043W	15:409778	5.01E-07
YJR006W	2:551299	6.90E-07
YKL113C	11:218377	3.80E-09
YNL085W	3.956944444	0.000255337
YNL085W	14:449639	6.06E-10
YDR478W	4:1418647	1.43E-14
YKL127W	13:77684	5.39E-05
YKL127W	14:449639	1.24E-08
YGL081W	7:403626	4.26E-06
YPR105C	2:602012	3.02E-08
YDR444W	3:90676	9.95E-05
YER184C	5:561804	4.82E-05
YMR162C	12:659357	0.000211317
YJR061W	8:63386	1.16E-05
YJR061W	10:548177	1.24E-11
YGR257C	7:995892	4.58E-08
YNL078W	2:555596	5.58E-14
YNL078W	8:111680	7.97E-05
YDR488C	12:674651	2.11E-05
YPL076W	12:705226	1.93E-05
YMR222C	12:659357	9.84E-06
YIL062C	12:644082	2.37E-05
YOR142W	15:108577	0.000117944
YGR225W	7:952065	5.22E-05

YER180C-A	5:549142	9.43E-11
YBR222C	14:449639	1.01E-09
YLR163C	15:113254	2.08E-06
YMR060C	13:390351	1.23E-10
YCL028W	2:537314	1.07E-05
YGR203W	12:662627	3.57E-05
YKR064W	15:141627	6.25E-05
YBR054W	13:277071	6.78E-06
YPL129W	16:318068	8.04E-06
YHR115C	8:340251	7.04E-11
YGR021W	15:154177	1.63E-07
YPL093W	14:449639	6.59E-11
YJR084W	10:604478	6.92E-12
YLR187W	12:516700	3.01E-06
YJR015W	10:461201	1.61E-16
YGR142W	1:187607	1.42E-05
YGR142W	7:375499	3.20E-13
YLR046C	12:239578	2.14E-08
YPL252C	16:70853	7.03E-15
YKR053C	4:1188862	4.89E-05
YKR053C	11:531561	2.23E-06
YGR200C	7:913059	0.000256109
YER141W	12:674651	0.000121917
YJL051W	8:80068	8.64E-05
YJL051W	10:345059	1.55E-16
YGR234W	12:662627	2.48E-15
YNL280C	12:674651	2.06E-06
YLR462W	12:1067122	8.63E-05
YJL143W	15:180961	2.74E-05
YDR384C	12:705226	2.43E-08
YKL007W	10:572473	5.39E-07
YCR104W	12:1056103	3.35E-07
YEL025C	4:478080	9.04E-05
YHL016C	8:71742	3.13E-06
YMR157C	15:116709	3.66E-06
YNL133C	15:141621	4.74E-06
YCL025C	13:87587	7.36E-09
YBR117C	2:477206	1.68E-14
YBR087W	12:677957	8.01E-05
YGL125W	3:90676	4.24E-06
YGL125W	5:272258	8.49E-06
YHR031C	8:157605	9.18E-06
YNL056W	14:502496	7.52E-14
YJL062W	14:449639	3.90E-09
YDR034W-B	4:446125	2.61E-05
YHR022C	15:141633	1.24E-05
YJL037W	3:90610	1.29E-05

YJL118W	2:537314		8.94E-05
YDR296W	15:113254		1.00E-07
YLR085C	12:317542		2.74E-09
YBR204C	2:628224		2.28E-10
YGL035C	1:187640		0.000222962
YGL035C	2:562415		7.61E-05
YGL035C	7:427476		2.06E-09
YOL114C	15:106272		3.08E-06
YHL013C	8:84437		3.82E-05
YDR170C		3.95625	7.05E-05
YMR117C	13:498750		1.56E-12
YER187W	5:568698		1.24E-15
YOR161C	15:141633		2.48E-06
YOL065C	2:553812		3.85E-05
YMR286W	15:108577		3.60E-07
YPL229W	12:611810		2.20E-06
YML085C	13:99675		1.38E-14
YML085C	14:449639		1.24E-06
YAR035W	12:668249		0.000120526
YAR035W	15:150651		6.60E-07
YDL079C	1:166221		3.68E-05
YDL079C	4:285470		1.06E-06
YEL015W	14:449639		2.93E-10
YMR177W	7:403626		1.26E-05
YLR414C	2:537314		1.81E-05
YIL147C	16:511406		6.99E-05
YDR492W	1:187519		5.13E-08
YDR492W	7:402879		7.40E-10
YGL234W	13:49894		2.65E-06
YEL057C	5:44617		9.21E-17
YNL099C	12:679808		1.91E-05
YMR059W	4:143910		1.62E-05
YMR059W	13:390357		6.43E-07
YIL087C	7:708252		1.64E-09
YIL087C	9:196145		2.15E-16
YNR060W	12:683463		1.95E-08
YNR060W	15:179289		0.000292861
YML074C	13:158910		3.01E-06
YDL227C	4:46316		2.67E-16
YPR065W	12:659357		4.59E-13
YEL004W	2:555596		6.43E-07
YLR452C	8:111683		3.19E-12
YMR200W	2:537314		2.72E-10
YNL104C	3:90676		6.38E-09
YLR466C-B	4:1510883		5.91E-16
YLR466C-B	12:1056097		2.14E-06
YGR244C	15:143597		2.12E-08

YKR075C	7:375499	3.76E-05
YOL007C	2:548401	9.51E-09
YDR301W	3.954166667	0.000128578
YDR301W	14:449639	5.27E-08
YKL043W	11:354466	7.63E-13
YDL166C	4:143910	1.71E-08
YOL049W	12:705226	7.82E-06
YOL049W	13:481548	7.23E-05
YNL289W	2:565216	9.29E-07
YJL209W	10:36810	6.23E-08
YPR137W	14:449639	1.49E-11
YPL061W	13:77684	2.17E-12
YPL061W	14:449639	3.00E-06
YJR051W	2:420366	8.53E-05
YOR320C	12:672779	3.36E-05
YMR009W	12:662627	7.72E-14
YGL010W	7:459354	6.34E-11
YMR163C	2:517123	3.50E-08
YJR034W	15:108577	2.60E-07
YCL055W	8:111683	1.14E-12
YPL156C	8:111690	3.82E-05
YOR237W	12:644136	8.12E-10
YNL021W	14:554606	3.44E-08
YDR451C	4:1344670	6.68E-10
YFR024C-A	13:910381	0.000174432
YFR024C-A	14:449639	5.77E-08
YLR136C	13:922256	8.14E-05
YGR249W	7:974680	2.32E-08
YKL196C	12:659357	8.84E-06
YNR040W	15:180210	3.79E-08
YGL174W	7:187179	1.34E-16
YNL258C	8:56246	1.14E-05
YIL015W	3:201166	8.94E-17
YER023W	2:419093	3.98E-05
YER023W	5:201477	5.11E-06
YER023W	14:449639	1.92E-07
YGR246C	7:995892	6.14E-08
YIL114C	15:154177	5.06E-05
YKL017C	11:421190	6.77E-14
YOR129C	2:517365	3.29E-07
YDR085C	8:111682	3.24E-06
YPL088W	16:387239	1.07E-12
YHL004W	15:113251	2.34E-07
YKL016C	13:28622	4.23E-05
YKL016C	15:113251	1.30E-07
YKL016C	16:500342	1.03E-06
YNL290W	14:96321	1.52E-10

YKR001C	14:449639	8.49E-12
YMR134W	12:662627	5.66E-16
YMR085W	7:900134	3.73E-05
YCL064C	13:49894	2.04E-16
YOR063W	15:469823	2.10E-05
YLR326W	12:662627	1.81E-10
YIL123W	2:555787	1.80E-05
YPR022C	16:600664	3.10E-16
YHL027W	1:42489	0.000178266
YHL027W	8:63386	9.61E-08
YPL089C	2:533268	2.25E-07
YHR075C	12:611810	7.29E-07
YHR075C	14:449639	2.19E-07
YPR036W	14:449639	1.24E-07
YNL297C	3.956944444	0.000113498
YKR024C	3.956944444	0.000156602
YOR156C	15:516673	3.48E-05
YNL012W	13:115474	6.60E-08
YGR179C	7:858473	5.86E-06
YLR109W	13:261725	1.57E-06
YAL047C	13:124876	4.44E-06
YDL179W	2:555596	7.04E-10
YEL048C	12:659357	6.00E-05
YMR145C	12:668249	1.29E-07
YMR145C	15:113251	1.28E-05
YJR147W	2:555596	5.58E-14
YOR270C	12:668249	3.82E-06
YML003W	13:286122	1.53E-12
YOR219C	8:111683	0.000150181
YDR111C	13:96015	1.65E-05
YDR111C	15:802916	1.09E-06
YPL158C	2:555596	8.68E-11
YKL003C	15:113267	3.03E-06
YNL192W	2:537314	6.91E-05
YNL192W	15:144659	0.000347988
YGL145W	2:567221	0.000317002
YGL145W	15:108577	3.58E-05
YDL115C	4:253395	2.00E-05
YJL002C	12:634227	6.13E-06
YNR044W	8:111690	9.43E-13
YKR015C	7:1083095	0.000245145
YOR360C	14:449639	1.91E-12
YPL219W	2:419093	1.01E-05
YKL150W	12:668249	2.00E-06
YKL150W	13:28622	8.83E-05
YKL150W	15:113251	6.34E-06
YIL098C	15:113254	0.000165723

YGR194C	15:108577	5.08E-06
YGR285C	14:449639	3.52E-07
YCR069W	12:635380	1.53E-07
YLR390W	15:108577	2.83E-05
YDL210W	4:89821	8.61E-08
YDL210W	15:301074	0.000106268
YDR082W	2:562415	5.96E-06
YIL021W	8:92960	0.000285874
YKR069W	11:554200	1.11E-10
YJR056C	10:535311	1.66E-05
YGL170C	7:187179	9.91E-08
YMR246W	1:74577	9.63E-05
YMR246W	12:634226	4.93E-06
YPL111W	13:49894	1.86E-14
YLR080W	2:567221	0.000265707
YKL064W	14:502496	1.30E-05
YJL029C	10:380085	2.90E-05
YHR019C	3:201167	1.06E-05
YBL058W	12:611810	8.79E-06
YOR273C	15:144659	1.01E-07
YDR251W	8:95469	2.22E-05
YDR251W	13:57145	0.000183951
YBR163W	2:565216	9.06E-09
YOR164C	12:611810	5.21E-05
YML121W	13:27644	4.41E-12
YNR002C	13:130069	5.52E-06
YNR002C	15:141627	3.45E-07
YNR002C	16:500342	1.47E-05
YER152C	2:555596	4.71E-16
YDR041W	15:154177	0.000159722
YGL210W	11:98330	1.21E-05
YGL210W	12:672779	1.65E-06
YDR197W	15:113251	0.000170835
YLR466C-B	4:1510883	9.63E-16
YLR466C-B	12:1056097	5.50E-07
YDR003W	4:447355	1.22E-06
YAR029W	12:668249	4.70E-05
YAR029W	15:113251	5.63E-06
YAR029W	16:511406	0.000258349
YBL044W	2:151686	1.15E-16
YGR035C	15:132423	1.25E-05
YIL036W	9:277908	8.56E-06
YOR316C	2:584357	3.68E-05
YHR059W	15:180210	1.94E-06
YBL113C	12:1056097	4.74E-06
YLR045C	12:659357	1.67E-05
YML081W	12:674651	0.000106293

YNL156C	12:662627	2.14E-14
YKL120W	3:81832	6.36E-15
YKL120W	15:180222	0.000249145
YKL051W	13:27644	1.94E-12
YKL051W	15:136327	9.83E-06
YBR078W	2:401568	1.64E-07
YML009C	15:113267	7.55E-06
YOR185C	15:174364	9.36E-05
YPL038W	16:492351	3.88E-08
YPL117C	12:662627	5.66E-08
YHL031C	12:644136	2.60E-06
YHR144C	12:19609	7.08E-05
YPL047W	2:567221	0.000326404
YPL047W	5:321708	1.92E-05
YPL047W	16:428612	7.23E-06
YGL219C	7:73452	6.94E-08
YFL010W-A	6:143977	4.41E-09
YKR089C	8:92960	6.55E-07
YKR089C	11:599170	5.01E-14
YER011W	5:350744	2.76E-06
YER011W	12:662627	1.91E-09
YGR014W	2:555787	2.06E-13
YDL078C	2:506661	3.72E-06
YDL078C	4:297977	1.27E-05
YKL104C	2:537314	1.69E-05
YOR375C	3:81832	5.77E-13
YML059C	13:46070	5.50E-05
YIL072W	7:708252	9.19E-06
YIL072W	9:238345	1.44E-10
YAR050W	1:201039	1.49E-16
YOL094C	15:113267	4.87E-12
YIR042C	6:208654	7.64E-06
YIR042C	9:437054	3.04E-16
YJL087C	15:99389	0.000100656
YKR021W	11:484826	1.49E-06
YJR153W	2:592863	1.50E-06
YJR153W	5:350744	5.95E-06
YBL085W	2:533268	1.80E-06
YBR160W	12:681096	1.05E-05
YHR116W	15:108577	1.01E-06
YBR208C	2:615927	0.000124398
YBR208C	13:100048	4.79E-08
YCL066W	3:201166	8.94E-17
YLR118C	2:565216	0.000148015
YLR118C	12:469604	7.46E-05
YJL149W	13:910381	0.000197207
YNL097C	13:46084	2.16E-05

YAR073W	6:28029	3.02E-05
YAR073W	13:158910	4.04E-08
YOL097W-A	15:136324	3.49E-06
YJL011C	15:116709	1.97E-06
YDR258C	5:321714	8.61E-06
YDR258C	12:514835	1.53E-05
YPL068C	16:420441	9.52E-06
YMR314W	12:611854	2.93E-05
YOL052C	14:449639	5.77E-08
YMR105C	15:174364	5.57E-06
YCR038C	3:201166	6.40E-07
YCR038C	13:33681	4.41E-06
YOR032W-A	13:46084	3.36E-06
YDR365C	15:116709	0.000111746
YMR231W	12:705100	0.000269553
YJR105W	12:662627	1.67E-11
YKL014C	4:1213416	0.000215012
YGL020C	7:459354	2.20E-08
YLR310C	12:757570	2.24E-06
YMR013C	15:108577	0.0002313
YNL294C	2:533262	9.94E-06
YHR004C	12:662627	9.00E-08
YBR083W	2:376145	5.89E-07
YBR083W	8:111683	3.65E-05
YEL049W	1:154328	5.16E-14
YJR101W	15:113267	3.03E-05
YBR185C	15:113267	5.46E-06
YGL251C	13:46084	8.15E-05
YOR079C	15:425275	0.000177162
YER062C	15:113267	6.56E-08
YHR086W	15:170945	1.95E-06
YHR208W	3:81832	4.69E-15
YCL004W	3:105042	6.66E-07
YMR150C	13:562907	2.68E-12
YLR079W	2:562415	1.72E-11
YLR079W	16:511406	0.000131896
YBR098W	2:480009	9.58E-05
YJR103W	2:427675	0.00019894
YJR103W	14:449639	4.63E-13
YMR152W	13:562907	3.56E-12
YDL174C	12:662627	1.49E-10
YER081W	1:42591	0.000328215
YGL154C	16:486643	0.000229653
YFL001W	6:143977	7.48E-07
YGR023W	15:170945	4.40E-06
YJR011C	15:116709	7.80E-05
YOR247W	2:548401	1.24E-14

YJL171C	3:201166	1.05E-09
YJL171C	10:99921	1.06E-09
YJR076C	10:575236	1.68E-09
YPL186C	15:143597	2.70E-05
YKR103W	11:656099	5.75E-17
YOR077W	12:689211	4.21E-05
YPR194C	2:562415	8.88E-06
YJL059W	14:449639	6.93E-07
YHR134W	8:389050	1.21E-08
YKL175W	14:486861	9.16E-08
YCL021W-A	3:81832	8.82E-16
YLR258W	2:567221	1.26E-09
YLR258W	15:141621	2.59E-05
YLR258W	16:547618	0.000107211
YBR101C	7:375499	1.28E-09
YAL041W	2:567221	2.01E-07
YAL041W	16:511400	0.000272829
YDR044W	5:350744	7.05E-08
YDR044W	12:662627	7.43E-10
YGR152C	2:533268	9.58E-05
YGR152C	7:790117	1.57E-08
YLR412C-A	12:956534	4.63E-05
YHL003C	8:111682	8.44E-09
YJL153C	10:135902	4.84E-09
YMR092C	13:445622	6.37E-16
YER099C	8:95289	2.74E-05
YER099C	12:662627	0.000144671
YGL263W	7:16619	4.64E-06
YGL263W	12:1023790	9.31E-07
YBR193C	2:628224	4.43E-11
YCL040W	15:174364	4.39E-05
YBR150C	2:555596	3.69E-11
YOR288C	2:162382	3.73E-05
YAR009C	5:420595	8.40E-07
YGL014W	7:459354	3.47E-06
YLR315W	12:679808	3.66E-05
YLR006C	12:145780	5.17E-07
YLR116W	12:380269	6.01E-10
YBL061C	15:108577	0.000104049
YER044C	12:662627	1.69E-14
YGL051W	1:187640	6.98E-09
YGL051W	7:403626	4.90E-17
YLL057C	12:35970	2.03E-07
YNL310C	14:33607	1.65E-05
YLR025W	12:210539	1.53E-09
YML007W	13:245632	3.13E-13
YMR068W	2:548401	0.000107371

YMR068W	12:721988	3.55E-05
YMR068W	13:404546	8.24E-08
YGL135W	15:488341	0.000114624
YGL237C	15:113251	2.10E-05
YLR425W	12:662627	3.47E-06
YDR107C	12:611810	7.50E-05
YDL204W	4:95527	3.02E-08
YDL204W	15:141633	9.32E-05
YER096W	5:332264	2.53E-06
YHR041C	8:185882	6.23E-08
YOR259C	12:662627	4.64E-05
YML034W	8:111679	8.07E-05
YBR182C	2:508843	3.71E-05
YBL046W	2:133749	1.44E-07
YHR023W	2:514035	2.52E-05
YGR212W	7:916675	1.66E-05
YIR001C	2:628224	2.88E-05
YBR177C	15:144659	5.32E-11
YOL058W	13:49894	1.87E-05
YEL075C	5:13213	1.45E-09
YDR305C	2:609055	6.68E-05
YDR348C	15:438828	0.00020921
YJR154W	5:272258	2.17E-06
YML061C	12:674651	1.20E-06
YML061C	13:149075	1.27E-05
YLR190W	14:542648	0.000142104
YDL219W	4:89821	9.25E-05
YFL022C	6:100521	3.50E-08
YDR333C	2:555575	6.65E-06
YBR145W	4:184214	4.85E-05
YDL102W	2:555575	0.000179902
YER018C	5:193876	2.16E-12
YML104C	13:49894	2.72E-10
YOR194C	14:502316	3.03E-07
YIR018C-A	9:386046	4.09E-07
YPL144W	16:266023	4.18E-09
YER072W	13:27644	1.01E-13
YJR064W	12:662627	1.54E-05
YML079W	13:110808	5.18E-11
YPL018W	13:28694	8.29E-14
YAL024C	2:569414	7.10E-10
YAL024C	16:500348	3.85E-05
YPL274W	13:910381	8.03E-11
YIL165C	9:33795	8.67E-17
YER158C	5:350744	7.70E-05
YJR047C	12:644082	2.32E-08
YJR047C	16:497425	1.50E-05

YBR126C	15:174364	3.61E-05
YER185W	5:568650	1.38E-07
YNR001C	15:136327	8.25E-06
YKL079W	13:99675	6.63E-05
YKL079W	15:136327	1.83E-05
YAL039C	1:74577	5.56E-06
YHR035W	8:176412	4.62E-12
YDL124W	4:262796	2.39E-13
YJL078C	2:567221	1.54E-15
YDR135C	11:85465	4.94E-05
YDR135C	13:234845	2.50E-06
YMR036C	13:328865	4.11E-14
YNR014W	13:54913	8.53E-06
YNR014W	15:174364	8.94E-10
YLR329W	12:782839	8.25E-13
YLR272C	12:689217	7.32E-09
YNL079C	8:389050	0.000276669
YGL151W	2:562415	0.000183774
YGL151W	15:116709	4.27E-07
YHL023C	8:56246	6.17E-11
YKR102W	11:656099	5.74E-17
YLR420W	5:117056	2.83E-16
YGR231C	15:108577	0.000104049
YGR007W	12:662627	3.08E-07
YCR107W	6.628472222	3.88E-05
YCR107W	0.868055556	3.31E-07
YCR107W	12:1059806	1.23E-08
YDR249C	2:537314	9.40E-07
YJL176C	2:567221	0.000265707
YOR346W	15:968429	2.71E-12
YLR178C	15:174364	2.64E-07
YFL062W	7:1081945	1.36E-05
YEL040W	5:79647	3.41E-09
YNL123W	14:393903	9.97E-12
YHR175W-A	12:677957	4.53E-05
YMR316W	13:910381	5.60E-11
YOR294W	7:141955	7.05E-05
YKL221W	0.868055556	3.58E-07
YOL156W	15:113251	0.000188875
YOR114W	15:546197	1.59E-10
YOR020W-A	6:238758	0.000112018
YOR020W-A	15:407690	1.62E-06
YGR207C	15:108577	5.46E-06
YDR070C	15:136324	4.93E-05
YJR090C	15:136327	5.39E-06
YGR049W	12:662627	6.14E-10
YGR261C	14:449639	3.82E-08

YBR196C-A	2:616262	2.22E-15
YGR057C	16:492351	3.89E-05
YIL106W	9:190866	3.83E-06
YDL146W	4:183015	2.48E-07
YOR377W	12:662627	9.98E-11
YPL120W	15:589145	1.96E-05
YKL184W	16:511406	5.94E-05
YLL060C	12:19609	4.55E-11
YBR092C	8:111686	0.00012553
YDR033W	4:509817	4.72E-15
YDL132W	4:226317	1.21E-14
YGL137W	3.954861111	0.000138923
YGL137W	14:449639	2.14E-12
YDL188C	12:659357	0.000146206
YDR035W	4:527445	1.21E-05
YMR321C	13:922258	1.00E-14
YBR056W-A	13:27644	3.33E-05
YLR174W	12:505763	5.38E-06
YDR115W	15:154177	6.50E-05
YPL063W	13:124876	2.48E-06
YAL063C	5.234722222	6.43E-09
YMR310C	13:910741	1.68E-08
YDR403W	13:115474	4.44E-06
YPL039W	16:489143	6.64E-14
YLR164W	14:449639	1.34E-06
YLR114C	2:551299	1.36E-05
YFR010W	6:144191	1.85E-05
YNL168C	2:530481	2.84E-06
YNL168C	14:449639	2.25E-10
YDR482C	1:193251	2.08E-05
YDR482C	13:33681	4.70E-08
YNL159C	12:635380	1.10E-06
YPL123C	2:519049	0.000183011
YOL013W-B	15:301074	2.93E-05
YPL071C	16:420441	6.72E-09
YER039C-A	13:77684	2.79E-06
YDL165W	4:143910	1.01E-06
YKR061W	2:551299	3.90E-08
YFL014W	15:144659	4.81E-11
YJL084C	13:87587	5.88E-05
YJL084C	14:449639	9.28E-07
YBR187W	12:705100	4.59E-05
YBR176W	2:584357	2.21E-11
YLR450W	12:634227	1.26E-07
YLL012W	12:677957	5.36E-09
YIR013C	7:707950	0.000296633
YIR013C	9:254745	8.85E-06

YMR119W	12:697260	4.83E-06
YOR011W	15:382531	6.59E-10
YPL145C	16:266023	3.91E-05
YCR107W	6.628472222	8.71E-07
YCR107W	0.868055556	2.01E-08
YCR107W	12:1054278	4.77E-10
YLR350W	4:975086	3.61E-06
YBR044C	2:328489	3.48E-07
YNL059C	2:615927	4.11E-05
YNL059C	14:502496	1.22E-08
YHR026W	2:516889	4.20E-05
YHR026W	12:662627	8.22E-06
YNR065C	14:732029	2.01E-08
YGL080W	7:375499	4.77E-09
YBL040C	2:163042	8.83E-11
YGR060W	12:662627	9.88E-12
YAR023C	1:185122	2.30E-12
YJL001W	10:451832	1.09E-09
YPR160W	15:174364	4.07E-07
YHR071W	15:108577	2.93E-05
YKL057C	15:116709	0.000119203
YPL139C	8:84437	6.88E-05
YLR411W	12:956534	1.43E-16
YBR242W	12:662627	2.95E-11
YLR121C	2:519049	6.98E-05
YJL219W	10:23505	2.81E-15
YNL041C	14:553129	2.87E-09
YLR318W	15:136324	0.000126609
YKR013W	2:530481	6.18E-10
YKR013W	11:468771	1.95E-07
YAR075W	13:158910	7.50E-07
YAR075W	14:449639	0.000113687
YHR048W	12:659357	3.16E-10
YHR048W	16:500348	0.000112556
YER065C	2:676336	4.72E-05
YJL058C	16:535979	8.83E-05
YHR109W	14:502496	8.16E-05
YKL198C	13:46084	9.01E-06
YHL044W	6.176388889	5.22E-17
YER034W	12:659357	1.85E-05
YPL004C	13:28622	1.02E-05
YPL004C	15:136327	4.50E-07
YPL004C	16:500348	1.86E-05
YMR189W	13:49903	0.00011638
YKL005C	11:442468	2.05E-06
YER053C-A	12:662627	6.53E-15
YDL042C	12:469156	6.55E-05

YOL096C	15:136327	2.08E-06
YCR073W-A	12:659357	4.18E-05
YJL167W	12:659357	2.36E-14
YNL141W	13:28334	2.75E-05
YDR298C	13:28622	3.59E-05
YDR298C	15:136327	2.09E-08
YDR298C	16:500342	3.48E-07
YDR242W	2:521415	0.000118899
YPL100W	13:99675	1.08E-05
YER119C	5:395442	9.99E-10
YMR015C	12:659357	7.25E-14
YLR287C	12:674651	8.64E-05
YHR143W	2:555596	3.91E-16
YPR149W	13:28622	7.54E-05
YPR149W	15:136327	5.15E-08
YPR149W	16:500348	7.92E-06
YKR091W	2:555596	1.03E-09
YIL120W	9:136845	2.26E-11
YIL120W	13:99675	2.11E-06
YEL071W	13:49894	3.08E-06
YEL071W	15:116709	7.20E-06
YEL071W	16:500342	3.61E-05
YGL255W	13:49894	1.62E-08
YPR168W	14:591237	1.37E-05
YBR093C	13:27644	4.22E-15
YAL009W	1:136161	4.29E-11
YHL009W-B	8:95313	6.62E-09
YHL009W-B	16:445372	4.65E-09
YGR061C	13:49894	1.15E-09
YOL087C	15:154309	1.00E-06
YDL001W	13:110808	1.39E-05
YLR284C	2:530481	2.16E-05
YML046W	8:111682	7.62E-05
YLR126C	13:885665	0.000152463
YKL023W	5:251262	4.96E-05
YAR019C	1:139312	0.000229772
YDR320C	4:1108558	1.09E-08
YGR165W	15:154177	1.10E-05
YCR097W	3:201166	8.94E-17
YIL066C	12:514835	1.17E-11
YBR158W	2:567221	3.85E-15
YJR142W	14:449639	4.27E-06
YMR195W	15:141633	6.06E-07
YJR144W	16:511406	1.09E-05
YMR108W	3:91305	5.06E-12
YIL149C	9:74540	4.03E-08
YBR119W	2:480009	2.85E-12

YBR046C	13:285840	6.29E-06
YFR006W	13:46070	1.34E-05
YFR006W	15:141633	1.59E-08
YFR006W	16:500354	5.67E-06
YEL041W	8:457580	2.37E-14
YBR133C	8:111680	1.99E-06
YMR098C	15:113251	0.000222977
YDR026C	2:427675	0.000260679
YER015W	13:183097	8.65E-05
YLR162W-A	12:472165	8.38E-17
YHR032W	8:167504	9.55E-15
YGL233W	12:635380	0.000180498
YDR093W	13:46084	6.50E-05
YNL237W	14:206186	5.55E-15
YPL161C	15:179289	6.47E-05
YDR523C	4:1499294	5.78E-06
YOR031W	15:382531	1.05E-15
YJL053W	10:327858	1.44E-06
YJL180C	15:154177	4.80E-06
YFR031C	13:100048	3.47E-05
YDR435C	12:644082	2.99E-05
YDR435C	15:301074	9.38E-05
YDR379W	2:555787	5.87E-08
YDR379W	16:511400	1.09E-07
YDR281C	13:27644	5.29E-14
YOR108W	3:100213	3.43E-06
YGL144C	12:659357	5.14E-07
YGL144C	13:27644	9.21E-05
YPL113C	15:143597	6.18E-05
YPL113C	16:500348	6.04E-05
YMR252C	15:108577	3.58E-05
YIR017C	5:251647	2.55E-05
YIR017C	8:167502	2.92E-05
YER132C	5:422612	7.78E-08
YLR417W	2:537314	7.19E-06
YLR417W	8:80068	1.81E-05
YLL023C	12:111412	7.31E-11
YPL082C	2:420528	2.40E-05
YPL082C	8:111686	0.000260679
YEL027W	12:634227	0.000117925
YNL158W	12:634227	2.32E-05
YOL082W	2:548401	3.70E-05
YGR044C	2:555596	1.93E-15
YNL238W	14:245307	1.91E-06
YGR173W	15:170945	0.000105231
YOR229W	12:644136	1.18E-09
YPR192W	16:932538	3.50E-11

Supplementary Table 2: eQTLs for MPTGA

Accession ID	QTL chrom:position	p-value
YKR009C	15:150651	1.62E-09
YKR009C	16:500348	1.55E-07
YER059W	2:555575	6.64E-07
YDR481C	13:27644	5.61E-43
YML113W	13:69114	4.60E-09
YML113W	15:174364	3.80E-12
YIL157C	15:154177	4.73E-10
YNR074C	12:662627	7.48E-11
YKL012W	2:551299	5.81E-08
YJL147C	2:555575	1.94E-09
YJL147C	16:500348	2.09E-10
YDL216C	4:68082	5.42E-27
YPL223C	15:174364	9.46E-28
YKL138C	2:551299	4.00E-14
YKL138C	8:167506	7.38E-07
YKL138C	16:500348	4.15E-11
YML035C	13:49894	5.13E-09
YML035C	15:174364	7.61E-11
YER087C-B	15:170945	6.45E-08
YIL160C	13:57145	6.21E-08
YIL160C	15:174364	2.95E-27
YLR050C	12:238298	2.65E-47
YOR072W-B	14:558284	1.34E-08
YDL048C	9:133693	5.20E-09
YDL048C	15:174364	1.67E-11
YDL159W	2:551299	4.67E-08
YNL216W	2:551299	5.02E-13
YFL034C-B	2:551299	1.31E-12
YFL045C	9:101011	3.15E-09
YGL222C	7:85112	2.39E-43
YIR021W	2:551299	1.31E-08
YIR021W	16:500348	3.43E-09
YHR014W	8:137221	1.78E-09
YEL006W	2:551299	5.03E-10
YEL006W	16:500348	3.26E-08
YLR401C	12:956534	2.08E-11
YDR447C	4:96271	1.96E-08
YDR447C	5:196196	2.28E-07
YDR447C	15:170945	5.69E-08
YOR192C-C	14:591237	2.23E-10
YOR192C-C	15:703771	5.26E-22
YKL195W	2:551299	1.01E-08
YKL195W	15:154309	3.48E-08
YKL195W	16:500348	2.49E-08
YDR144C	15:150651	2.13E-08

YBR149W	2:548401	1.04E-11
YMR076C	8:111682	1.67E-09
YOR175C	12:662627	3.49E-11
YHL010C	8:95289	3.99E-20
YHR152W	8:111683	1.88E-07
YER035W	5:200848	8.28E-08
YHR002W	15:136327	1.15E-07
YPL020C	16:511406	5.67E-17
YLR286C	2:562409	#######
YJL185C	15:174364	1.73E-09
YGR130C	15:174364	5.61E-12
YBR065C	2:368060	2.45E-14
YJR082C	10:584276	1.53E-28
YMR182C	2:507282	7.90E-08
YPL264C	2:551299	1.20E-08
YPL199C	13:46084	8.35E-09
YLR266C	12:683457	2.51E-10
YDR447C	4:95527	2.32E-07
YDR447C	5:193876	3.92E-11
YDR447C	15:170945	2.04E-22
YML019W	12:672779	1.82E-07
YML019W	13:227254	2.44E-08
YKL166C	2:562415	1.17E-09
YNR038W	15:150651	1.17E-09
YJL165C	15:143597	2.38E-10
YDL086W	12:662627	4.36E-29
YKL096W	11:261779	3.88E-09
YKL096W	15:136324	7.66E-18
YLR064W	12:264911	4.89E-12
YBR194W	2:562415	2.18E-07
YHR198C	13:28622	3.59E-08
YHR198C	15:174364	6.62E-17
YNR055C	15:174364	3.96E-11
YLR149C	15:174364	3.25E-18
YOR271C	3:91287	4.88E-15
YOR271C	15:174364	5.33E-17
YHR197W	15:174364	1.35E-09
YAL034C	13:69114	6.58E-09
YAL034C	15:174364	5.54E-21
YOR084W	15:174364	1.56E-08
YDR140W	4:744330	6.10E-21
YMR216C	13:698980	3.12E-17
YDR245W	15:170945	2.26E-09
YOR187W	2:562415	1.40E-13
YOR187W	15:143597	1.37E-10
YOR187W	16:500348	3.50E-11
YGL121C	15:174364	3.74E-08

YBR285W	2:533262	1.20E-11
YBR285W	15:174364	3.16E-17
YOL028C	15:180180	7.51E-13
YMR220W	12:662627	2.07E-22
YAR002W	1:154328	1.76E-46
YPR106W	2:555596	6.79E-49
YOR124C	14:449639	2.66E-31
YDR072C	2:519049	7.51E-11
YPL269W	13:49894	6.13E-10
YGR110W	15:143597	7.48E-09
YOR394C-A	15:1065719	7.42E-24
YBR157C	2:555596	2.21E-16
YKL192C	15:170945	4.21E-08
YLR283W	12:705100	9.22E-30
YLR151C	12:450046	8.23E-40
YOR165W	15:632894	2.09E-11
YBL018C	2:185450	3.07E-49
YPL101W	2:551299	7.77E-09
YBR169C	15:174364	1.02E-13
YCL068C	3:201166	2.62E-17
YFL049W	6:33334	2.93E-07
YKL167C	15:170945	3.46E-08
YOR264W	2:562415	5.96E-36
YIR039C	6:208654	7.97E-17
YIR039C	9:435398	#######
YIR039C	12:1059925	1.55E-07
YDL061C	2:489202	6.39E-14
YDL061C	4:95527	4.26E-08
YDL061C	5:200848	5.20E-10
YDL061C	15:154309	1.27E-14
YDL061C	16:500342	2.83E-12
YOL122C	15:108577	3.80E-19
YPR117W	15:174364	6.28E-09
YER053C	5:196196	3.89E-09
YER053C	15:174364	6.98E-16
YOL105C	15:132423	2.31E-36
YCL018W	3:81832	2.21E-63
YDR518W	12:672779	1.25E-08
YDR210W	2:555575	3.75E-09
YLR089C	13:49894	9.50E-12
YMR227C	15:150651	2.86E-07
YIL161W	9:46561	2.93E-27
YCL054W	5:196196	3.05E-07
YCL054W	15:150651	4.74E-07
YDR006C	2:506661	3.50E-07
YOL162W	3.133333333	3.14E-76
YFL047W	8:111680	3.32E-09

YGR174W-A	2:537314	6.50E-08
YGR174W-A	15:174364	9.35E-12
YDR515W	13:99675	1.11E-07
YHR128W	15:170945	1.41E-08
YOL077W-A	15:141627	5.81E-08
YDL125C	4:246738	6.18E-12
YDL191W	4:95527	1.70E-10
YDL191W	5:200848	4.35E-10
YDL191W	9:74540	2.67E-08
YDL191W	15:170945	2.34E-11
YGR083C	15:170945	3.56E-10
YOR285W	15:846344	2.01E-70
YBR084C-A	2:489202	1.03E-11
YBR084C-A	5:193876	2.68E-09
YBR084C-A	15:143597	2.13E-11
YBR084C-A	16:500342	3.01E-08
YBR296C	1:141181	1.35E-13
YBR296C	13:27644	7.69E-80
YML064C	13:149075	2.41E-19
YHR005C	8:111683	2.57E-21
YHR005C	13:49894	1.17E-17
YAL003W	2:537314	3.54E-17
YAL003W	7:375499	4.44E-16
YAL003W	8:167506	1.82E-07
YAL003W	15:143597	4.33E-13
YOR161C-C	15:594024	1.43E-08
YML023C	13:243624	8.39E-10
YGL196W	2:555575	7.08E-08
YGL196W	7:139173	6.12E-08
YFR036W	6:227675	1.27E-08
YDR231C	8:457580	3.06E-42
YHR053C	2:565216	3.97E-18
YGL104C	7:311205	6.46E-19
YGL104C	15:174364	2.97E-08
YMR169C	15:174364	1.03E-28
YDL012C	2:480009	3.55E-08
YDL012C	4:433589	3.26E-56
YOL011W	13:28334	2.39E-09
YPL002C	16:555416	9.46E-31
YKL194C	2:551299	4.21E-07
YKL194C	15:174364	5.47E-09
YDR450W	4:96271	9.17E-11
YDR450W	5:200848	1.01E-08
YDR450W	9:74540	4.18E-08
YDR450W	15:170945	9.09E-11
YGL179C	7:167587	4.96E-13
YHL032C	13:46084	8.74E-08

YHL032C	15:174364	1.23E-23
YKL140W	11:178402	7.72E-31
YPL127C	8:111679	4.01E-07
YBR295W	13:46084	1.58E-08
YBR295W	15:174364	7.86E-14
YKL085W	15:174364	3.62E-10
YLR029C	2:519049	2.77E-07
YLR029C	5:196196	2.52E-07
YLR029C	15:170945	1.66E-10
YMR048W	13:371857	1.23E-49
YLR312W-A	12:757807	5.57E-14
YML091C	13:49894	1.48E-22
YML091C	15:174364	1.53E-09
YML091C	16:500348	1.00E-09
YLR299W	2:555787	8.29E-11
YLR299W	12:721988	3.02E-10
YMR128W	15:150651	1.11E-07
YPR155C	15:150651	1.03E-14
YOR338W	2:555575	1.06E-07
YOR338W	4:95437	4.16E-08
YIL052C	2:499012	1.01E-09
YIL052C	5:196196	1.88E-10
YIL052C	15:170945	4.82E-13
YJL196C	1:41483	3.50E-09
YJL196C	7:403925	7.39E-07
YHR133C	15:170945	8.76E-10
YOR179C	2:551299	1.77E-09
YMR131C	15:150651	1.94E-09
YNL055C	2:537314	1.30E-07
YNL055C	14:502496	1.24E-12
YNL055C	15:116709	1.33E-12
YLR212C	8:111682	1.10E-06
YDL108W	4:273846	2.22E-12
YGL252C	7:15891	1.87E-24
YPL131W	2:499889	2.17E-12
YPL131W	5:194873	1.24E-11
YPL131W	9:74540	2.60E-10
YPL131W	14:547071	4.63E-09
YPL131W	15:170945	8.97E-17
YPL131W	16:500342	1.84E-11
YLL027W	12:86369	1.57E-39
YBR084W	5:194883	1.02E-06
YBR084W	16:428900	1.17E-08
YKL132C	11:194611	4.51E-24
YJL200C	2:477206	5.53E-17
YKR092C	13:46084	3.69E-09
YKR092C	15:141633	2.73E-12

YKR092C	16:511406	1.91E-07
YDL168W	4:161196	#######
YGR088W	13:28622	1.89E-09
YGR088W	15:144659	2.95E-30
YMR135C	15:143597	1.46E-11
YDL085W	12:668249	9.45E-09
YDL085W	13:28622	2.90E-09
YDL085W	15:174364	2.58E-12
YDL236W	12:662627	1.58E-09
YDL236W	15:174364	1.20E-10
YLR300W	12:721988	1.42E-66
YPR184W	5:200848	4.90E-08
YPR184W	13:46084	1.54E-08
YPR184W	15:174364	4.14E-33
YHR046C	8:193175	1.88E-15
YKL135C	8:111690	6.40E-09
YKL135C	11:178411	5.46E-10
YDL223C	15:174364	2.45E-30
YNR067C	2:555596	8.31E-35
YBR186W	2:562415	2.84E-12
YAL067C	1:10152	9.36E-39
YLR346C	11:389068	1.18E-09
YLR346C	12:810883	4.82E-24
YDR516C	7:139173	3.84E-09
YDR239C	13:261725	2.93E-07
YMR115W	13:494170	4.00E-08
YDL173W	15:179289	8.43E-10
YOL066C	15:205104	1.52E-18
YFL052W	10:24469	5.71E-44
YEL051W	12:644082	1.04E-06
YGL006W	7:502131	1.17E-22
YDL055C	2:555596	1.03E-16
YDL055C	8:128732	9.56E-07
YPL206C	16:125271	1.05E-17
YOL090W	15:154177	1.13E-15
YML124C	2:507282	6.06E-16
YML124C	13:28694	1.53E-11
YML124C	15:141633	1.04E-07
YML124C	16:511406	2.02E-07
YHR065C	15:174364	2.90E-07
YOR062C	15:428238	2.35E-20
YOL036W	15:170945	2.61E-10
YMR306W	2:551299	4.62E-14
YOL098C	15:141627	9.71E-10
YGR178C	3.956944444	3.12E-08
YOL021C	2:551299	1.20E-11
YMR008C	15:108577	4.46E-07

YMR322C	12:659357	7.28E-12
YJL133W	10:159479	1.47E-16
YDR454C	15:143597	6.05E-10
YIL009W	2:555575	1.96E-08
YIL166C	9:33795	4.94E-44
YML125C	8:176670	1.12E-08
YML125C	15:150651	5.82E-09
YGL048C	7:375499	1.65E-11
YGL048C	8:176670	1.51E-07
YKL047W	16:500348	1.31E-07
YHL001W	2:519049	2.00E-09
YHL001W	5:193876	6.05E-08
YHL001W	15:154309	5.83E-10
YDR460W	3:201166	1.75E-30
YLR313C	12:757807	4.93E-31
YOR178C	15:174364	1.53E-21
YJL016W	10:404508	7.68E-20
YJL016W	15:174364	1.44E-14
YJL218W	10:23505	1.04E-56
YDL218W	2:537314	4.52E-09
YDL218W	4:95527	1.91E-16
YLR256W	12:662627	3.81E-34
YJR123W	2:499012	4.57E-12
YJR123W	4:95437	1.05E-07
YJR123W	5:194873	7.21E-11
YJR123W	9:74540	1.80E-07
YJR123W	15:170945	4.07E-14
YEL021W	5:117056	4.00E-87
YLR343W	12:811669	3.94E-42
YML102W	8:111682	2.07E-08
YGR027C	2:517123	4.77E-07
YGR027C	15:143597	3.22E-09
YHR097C	15:174364	5.50E-13
YGL253W	7:12939	6.19E-11
YGL253W	10:380085	1.08E-08
YGL253W	15:174364	1.07E-21
YOR390W	13:910741	6.95E-07
YGR258C	2:537314	5.75E-09
YGR258C	15:143597	4.92E-13
YDL229W	2:489202	2.61E-12
YDL229W	5:196196	2.74E-07
YDL229W	15:143597	1.70E-18
YDL229W	16:500342	1.62E-13
YJL108C	10:218798	5.02E-24
YJL193W	10:56788	1.89E-17
YDL155W	2:516889	8.84E-07
YER103W	13:69114	9.38E-10

YER103W	15:170945	1.65E-12
YOR045W	2:562409	3.73E-09
YOR045W	13:99675	4.77E-12
YOR045W	15:411901	4.22E-14
YEL062W	2:551299	2.89E-12
YEL062W	8:152932	6.14E-08
YIL151C	9:87577	1.76E-08
YGR204C-A	7:905017	1.66E-13
YKL110C	15:143597	2.20E-07
YMR088C	2:551299	6.35E-13
YJL205C	15:180961	1.11E-07
YJR121W	15:136327	4.81E-09
YDR539W	4:1510883	9.02E-40
YNL313C	14:38762	1.15E-12
YNL313C	15:170945	6.37E-08
YKL180W	2:499889	1.44E-15
YKL180W	5:196196	2.61E-11
YKL180W	9:74540	8.01E-07
YKL180W	15:154309	1.49E-12
YKL180W	16:500342	6.19E-11
YKL116C	11:219920	2.31E-25
YMR301C	15:154309	1.03E-09
YNR020C	2:551299	5.48E-08
YNR003C	5:251647	5.66E-09
YNR003C	8:167506	2.06E-10
YIR034C	2:477206	2.60E-10
YEL036C	15:143597	9.20E-08
YLR185W	2:499012	4.21E-10
YLR185W	5:196196	2.05E-11
YLR185W	15:170945	4.19E-15
YPR178W	12:662627	9.60E-09
YCR059C	13:57145	7.22E-08
YCR059C	15:174364	1.78E-14
YML093W	15:150651	4.47E-07
YPL081W	2:499012	1.22E-07
YHR087W	15:174364	3.81E-13
YHR038W	2:551299	2.81E-13
YHR038W	16:500348	3.07E-09
YOL063C	15:205104	2.06E-37
YLL028W	12:86369	1.03E-08
YLL028W	15:150651	7.28E-37
YFR030W	5:272255	1.33E-13
YFR030W	8:167568	8.29E-09
YHR005C-A	15:141627	9.37E-08
YKL081W	2:537314	2.06E-07
YKL081W	15:170945	4.02E-19
YNR017W	2:551299	1.87E-12

YNR017W	8:167506	8.64E-07
YNR017W	16:500348	2.20E-11
YJL088W	13:49894	6.58E-12
YCR098C	3:301446	6.34E-27
YCR098C	13:27644	4.11E-08
YHR043C	8:193175	1.84E-75
YCR011C	13:27644	3.28E-09
YOR321W	12:662627	1.12E-08
YKL188C	11:85465	6.75E-11
YGL089C	3:201166	1.77E-69
YDR243C	1:51324	5.54E-30
YCR073C	2:551299	3.31E-07
YCR073C	13:46084	9.22E-10
YCR073C	15:174364	6.06E-18
YDR262W	4:975086	1.93E-33
YOL109W	15:136327	1.35E-14
YDR530C	4:1497132	2.38E-13
YKR056W	1:41483	7.85E-08
YGL031C	5:194873	2.36E-10
YGL031C	9:101011	5.98E-08
YGL031C	15:170945	1.21E-09
YER013W	15:136327	1.40E-11
YGL013C	15:136327	2.53E-07
YOL112W	2:551299	5.57E-11
YIL094C	2:477206	5.57E-21
YBL072C	2:499012	1.38E-11
YBL072C	5:196196	2.37E-11
YBL072C	7:141955	5.07E-11
YBL072C	9:74540	3.10E-10
YBL072C	13:556835	8.14E-09
YBL072C	15:174364	2.16E-15
YBL072C	16:500342	6.99E-12
YER183C	5:568566	6.51E-08
YER069W	13:49894	1.41E-11
YDR001C	4:446125	2.02E-14
YDR001C	15:174364	8.54E-08
YMR280C	15:174364	3.10E-15
YLR218C	15:154309	2.43E-08
YJR032W	10:470298	1.82E-11
YJR032W	13:46084	1.27E-08
YJR032W	15:174364	3.34E-13
YBL027W	2:489202	3.33E-12
YBL027W	5:196196	9.93E-13
YBL027W	15:170945	2.10E-13
YER102W	2:499012	3.94E-10
YER102W	5:194873	1.19E-10
YER102W	9:74540	5.21E-08

YER102W	14:547071	2.58E-12
YER102W	15:174364	1.97E-15
YER102W	16:500342	4.88E-12
YGL189C	2:519049	2.02E-14
YGL189C	5:196196	9.85E-11
YGL189C	15:154309	9.34E-16
YGL189C	16:500342	1.03E-09
YIL162W	9:38608	2.85E-12
YJL125C	2:555575	7.98E-09
YLR233C	12:607076	1.24E-30
YDL103C	4:273846	2.75E-09
YHL018W	8:156412	9.96E-07
YOL032W	15:255015	3.06E-14
YLR303W	8:167504	1.06E-21
YJL100W	12:659357	6.54E-14
YGL001C	12:662627	1.19E-23
YPR200C	11:655678	2.39E-09
YJL068C	2:427675	3.31E-07
YCR023C	8:176670	1.13E-06
YMR279C	12:469156	1.85E-21
YDL057W	2:551299	2.31E-12
YGL038C	13:99675	1.13E-07
YGL038C	15:174364	7.97E-10
YLL038C	8:176670	1.57E-07
YDL105W	4:273846	1.61E-39
YOR028C	15:174364	2.28E-15
YLR265C	12:674651	3.03E-46
YKR057W	2:567221	1.77E-07
YKR057W	4:96271	4.77E-08
YKR057W	5:194873	5.75E-09
YKR057W	15:170945	9.27E-10
YNR043W	12:662627	1.47E-25
YNR043W	15:174364	2.19E-13
YPL036W	2:29486	4.19E-10
YPL036W	15:136327	3.52E-09
YPL036W	16:500342	1.33E-27
YGR085C	2:499012	2.11E-13
YGR085C	5:193876	1.87E-12
YGR085C	15:170945	7.42E-16
YOR125C	15:572404	1.22E-09
YKL027W	11:388373	3.31E-11
YPL189W	3:81832	1.25E-09
YPL189W	15:143597	1.13E-08
YPL189W	16:182953	2.58E-16
YGR148C	2:499889	3.56E-11
YGR148C	5:193876	3.43E-10
YGR148C	9:74540	3.33E-08

YGR148C	14:547071	4.12E-09
YGR148C	15:170945	1.98E-10
YBR291C	2:480009	4.84E-47
YAR014C	2:562415	2.27E-15
YPL134C	16:256358	2.07E-10
YJR074W	10:573371	1.67E-07
YKR044W	15:170945	8.46E-11
YFR045W	15:174364	1.86E-10
YPR175W	15:174364	3.16E-09
YDR540C	4:1511257	1.24E-40
YGR214W	2:499012	1.14E-15
YGR214W	4:95527	2.05E-07
YGR214W	5:196196	6.83E-12
YGR214W	9:74540	3.75E-07
YGR214W	15:170945	2.84E-15
YPL017C	15:174364	1.06E-19
YJR014W	10:461201	4.52E-14
YHR040W	8:176412	2.87E-08
YGL169W	7:187185	1.92E-80
YDR345C	2:551299	6.70E-12
YDR345C	15:141627	8.99E-08
YDR489W	4:1441486	4.76E-25
YLR026C	2:548401	8.55E-08
YLR364W	8:167504	4.40E-08
YML123C	13:27644	2.03E-55
YCR083W	3:258267	1.24E-15
YCR083W	5:117046	1.64E-07
YCR083W	15:174364	2.20E-14
YOR090C	15:491178	8.87E-11
YPL258C	2:636332	5.39E-17
YPL258C	16:70847	4.25E-91
YJL140W	10:151310	2.81E-17
YDR416W	15:594024	4.46E-08
YAL042W	2:551299	6.07E-08
YAL042W	15:143597	8.91E-08
YGR205W	7:913065	2.50E-41
YGR205W	15:174364	5.23E-09
YER007C-A	15:174364	6.20E-08
YBR197C	2:608310	1.19E-53
YOR076C	8:167504	1.00E-07
YLR260W	12:672779	3.33E-16
YIL044C	9:238345	2.63E-11
YOR003W	2:555575	1.54E-10
YOR003W	12:662627	4.53E-08
YNL233W	8:111690	1.57E-11
YKL035W	15:170945	6.22E-11
YGL057C	7:403626	8.65E-24

YOL061W	15:174364	4.10E-10
YER124C	2:555596	#######
YER124C	4:95437	3.48E-09
YBL068W	15:174364	1.41E-13
YPL272C	12:662627	5.19E-17
YPL272C	13:124876	1.43E-09
YPL272C	15:113251	1.23E-09
YPL272C	16:500342	2.66E-12
YDL199C	4:114155	2.98E-10
YDL199C	15:174364	1.45E-09
YIL009C-A	2:551299	2.73E-07
YIL009C-A	8:176670	4.94E-07
YIL009C-A	9:323975	2.61E-13
YFL030W	15:174364	3.60E-23
YLR139C	12:423789	2.47E-20
YPR193C	12:662627	4.17E-23
YOR319W	15:930110	1.68E-38
YLR237W	8:111683	1.14E-08
YLR237W	12:635380	2.73E-14
YKL162C	15:136327	7.13E-09
YPL245W	16:84943	1.02E-11
YHR036W	8:176994	3.01E-10
YPR174C	7:375499	2.56E-16
YJL056C	10:327858	1.01E-21
YAL017W	15:174364	3.72E-09
YOL097C	15:170945	6.23E-12
YKR087C	11:599170	2.11E-39
YOR341W	15:174364	3.74E-08
YDR364C	4:1185630	3.45E-11
YGR266W	12:662627	3.28E-11
YHR129C	15:174364	1.86E-09
YKR101W	11:645253	1.04E-09
YER093C	12:659357	8.05E-09
YMR156C	13:564142	1.28E-12
YDR090C	2:553812	1.53E-13
YDR090C	15:175594	1.23E-10
YFR033C	12:668249	3.31E-13
YGR287C	7:1063841	5.82E-49
YGR287C	15:170945	2.54E-11
YJL109C	15:174364	1.92E-07
YPL153C	8:111683	7.63E-07
YER149C	15:150651	1.25E-08
YLR275W	12:697260	1.04E-12
YBR135W	2:506661	3.50E-12
YMR142C	2:499012	3.21E-16
YMR142C	4:95527	1.10E-07
YMR142C	5:196196	6.39E-11

YMR142C	9:74540	6.03E-08
YMR142C	15:170945	9.49E-15
YOR228C	15:780225	7.58E-15
YDL229W	2:489202	1.75E-12
YDL229W	9:74540	4.10E-09
YDL229W	15:143597	1.51E-18
YDL229W	16:500348	3.77E-15
YKL187C	11:97725	3.32E-17
YKL187C	15:143597	7.37E-09
YML008C	12:662627	2.08E-12
YLR165C	12:472165	9.43E-31
YFL020C	1:154328	2.52E-15
YCR091W	15:174364	2.98E-13
YKL037W	15:174364	8.85E-12
YPL189C-A	12:674651	3.57E-08
YMR275C	13:824809	1.25E-11
YDR038C	4:527458	#######
YMR239C	15:174364	1.79E-11
YLR363C	8:175255	5.12E-07
YPR006C	16:555416	6.32E-60
YLR075W	5:200848	3.69E-08
YLR075W	9:133693	3.90E-08
YLR075W	15:174364	5.42E-14
YDR064W	2:499012	1.10E-18
YDR064W	4:62325	2.15E-11
YDR064W	5:194873	3.15E-09
YDR064W	9:74540	2.66E-13
YDR064W	15:174364	3.49E-12
YIL134W	9:98955	2.34E-10
YIL121W	3:91977	1.31E-06
YIL121W	9:136845	1.14E-16
YIL121W	12:683463	5.74E-40
YER064C	2:506661	4.45E-18
YBR077C	2:551299	3.55E-12
YNR066C	15:179289	3.91E-07
YER176W	5:549142	1.07E-09
YLR271W	15:154309	4.77E-10
YJL063C	2:555575	8.34E-10
YJL063C	16:500348	2.74E-08
YPR163C	2:555575	1.43E-07
YPR163C	15:170945	2.94E-10
YGL178W	7:187179	1.03E-14
YOR152C	15:154309	4.71E-13
YDR234W	2:477206	4.06E-10
YDL222C	13:46084	9.72E-09
YDL222C	15:174364	2.79E-41
YNL073W	2:533262	2.85E-10

YNL073W	13:99675	2.11E-07
YNL073W	15:141627	2.12E-09
YNL073W	16:500348	5.88E-09
YBR275C	2:754059	6.29E-19
YKL112W	2:551299	8.66E-08
YMR159C	8:167506	1.36E-08
YIR030C	2:551299	2.20E-15
YIR030C	9:419418	1.03E-14
YGL068W	2:555575	3.15E-18
YGL068W	7:425445	8.97E-08
YGL068W	8:167506	6.83E-09
YGL068W	16:500348	3.65E-11
YCL026C-A	3:75021	3.01E-21
YGL259W	2:562415	2.13E-15
YGL259W	4:100720	4.28E-08
YOL120C	2:499012	4.72E-13
YOL120C	5:196196	4.43E-11
YOL120C	9:74540	2.86E-07
YOL120C	15:170945	4.30E-13
YJR099W	10:612602	8.70E-13
YKL220C	13:910741	2.57E-10
YGL254W	7:15891	8.09E-23
YBR162C	2:551299	9.24E-07
YBR162C	15:174364	8.89E-08
YGR197C	7:889602	1.14E-31
YGR197C	15:632882	3.93E-26
YKL022C	11:388373	4.12E-14
YPR181C	2:506661	2.04E-08
YDL031W	15:174364	1.55E-07
YLR360W	2:555787	4.32E-13
YDR337W	15:154177	3.48E-07
YPR125W	2:551299	2.18E-11
YML054C	12:672779	3.54E-16
YML054C	15:150651	2.90E-16
YPL107W	2:551299	2.02E-14
YPL107W	12:662627	1.03E-15
YPL107W	16:500348	7.06E-17
YKL103C	2:533262	7.24E-09
YLR342W-A	12:810883	4.41E-38
YGL111W	15:174364	7.12E-09
YJL162C	10:151190	6.24E-07
YLR249W	2:481439	3.67E-09
YLR249W	9:101011	4.96E-08
YLR249W	15:170945	5.48E-21
YHR072W	12:662627	8.19E-27
YHR072W	15:174364	6.72E-07
YBR023C	15:143597	4.92E-09

YEL038W	5:79647	2.00E-43
YMR202W	12:662627	7.13E-26
YNL217W	15:174364	1.23E-17
YOL020W	2:555575	1.67E-17
YCR026C	3:177850	2.24E-08
YKL165C	8:111690	3.65E-08
YOL064C	13:27644	6.85E-08
YDR349C	13:46084	1.32E-08
YDR349C	15:174364	4.10E-17
YGL096W	15:174364	2.04E-13
YDL104C	4:273846	1.10E-12
YDL139C	4:211612	3.69E-41
YOR022C	15:384743	1.67E-17
YPL066W	2:555575	4.05E-09
YPR005C	2:555596	2.65E-08
YKR049C	15:170945	9.25E-19
YER036C	2:481439	7.61E-08
YLR103C	8:111683	7.66E-08
YKR043C	15:174364	3.81E-21
YDR541C	4:1525327	2.87E-10
YGR053C	15:170945	5.32E-09
YDR531W	4:1495582	3.00E-13
YDR347W	2:551299	5.95E-08
YOR246C	2:537314	4.58E-07
YOR246C	15:170945	1.09E-14
YLR222C	15:150651	1.22E-07
YMR241W	15:174364	2.02E-09
YML075C	12:662627	1.70E-46
YER006W	15:174364	5.76E-08
YCL059C	15:174364	4.54E-10
YHR200W	14:449639	6.30E-27
YGR166W	13:46084	2.90E-09
YGR166W	15:174364	3.09E-11
YLR267W	13:28694	2.79E-11
YLR267W	15:136324	7.26E-08
YOL154W	15:16838	1.07E-28
YDR534C	5:458085	1.28E-08
YDR534C	9:435536	8.22E-10
YLR040C	3:201166	#######
YBR121C	15:170945	5.60E-13
YHR085W	15:174364	1.73E-10
YHR140W	15:174364	1.84E-08
YHR062C	15:174364	2.95E-10
YHR139C	15:150651	6.98E-12
YBL049W	2:517365	2.98E-12
YLR231C	12:644136	7.88E-20
YOR047C	4:201395	1.18E-08

YOR047C	15:384923	5.24E-13
YKR003W	11:442468	5.44E-19
YDR490C	15:143597	1.82E-10
YFR039C	15:174364	1.28E-09
YPL019C	13:27644	8.38E-48
YPR140W	15:174364	8.41E-08
YDR502C	12:683457	3.16E-23
YDR502C	13:46070	5.26E-12
YDR502C	15:143597	5.69E-12
YKR104W	11:651668	#######
YOR389W	13:910381	8.21E-08
YOR389W	15:1065719	5.75E-21
YER136W	12:679808	8.55E-08
YLR376C	12:872448	3.63E-21
YBR079C	2:555575	2.44E-08
YML105C	8:176670	1.69E-08
YDL089W	4:289639	2.30E-18
YGR208W	2:489202	1.55E-08
YGR208W	7:911217	1.43E-18
YGR208W	15:143597	1.46E-09
YLR234W	8:111679	3.55E-07
YLR234W	12:611967	3.79E-10
YPR010C	15:174364	1.56E-09
YAL035W	2:555575	2.21E-07
YMR010W	15:174364	1.89E-09
YER126C	15:150651	6.37E-08
YGL189C	2:517123	3.60E-14
YGL189C	5:196196	3.59E-11
YGL189C	15:154309	4.14E-13
YIL115C	9:141014	2.36E-12
YDR528W	15:170945	5.86E-11
YIL069C	2:499012	5.87E-19
YIL069C	5:193876	1.61E-13
YIL069C	15:170945	1.95E-12
YIL069C	16:500342	1.84E-10
YNL111C	12:662627	1.92E-27
YPR043W	2:519049	3.45E-11
YPR043W	5:193876	3.46E-09
YPR043W	15:170945	4.07E-17
YGL055W	7:403626	2.94E-20
YGL055W	12:681096	1.12E-07
YGR170W	13:100048	5.53E-09
YMR225C	2:551299	1.71E-08
YMR225C	16:500348	4.27E-07
YER089C	2:519049	5.60E-07
YER089C	5:350744	5.20E-11
YAR027W	1:185122	8.78E-96

YOR099W	12:662627	3.66E-08
YNL161W	15:174364	4.50E-10
YPL132W	2:551299	1.38E-08
YPL132W	15:174364	3.53E-16
YER088C	15:174364	1.16E-14
YJL139C	10:151310	1.36E-16
YAR031W	1:185122	5.89E-15
YER101C	15:154309	3.04E-14
YNL242W	14:191243	1.67E-37
YNL121C	2:551299	4.15E-08
YNL113W	15:174364	2.02E-08
YAL064W-B	1:16984	3.32E-39
YOL104C	15:132423	1.72E-44
YIR031C	2:551299	3.22E-34
YIR031C	9:419722	3.99E-11
YIR031C	13:87587	5.19E-09
YIR031C	16:500342	2.90E-09
YDR067C	4:582121	2.98E-13
YDR067C	14:402312	1.62E-09
YAR002C-A	15:150651	1.37E-07
YDR524C	4:1471859	1.80E-08
YDR213W	12:662627	1.29E-07
YGR089W	12:668249	7.48E-07
YDR036C	4:518394	2.97E-18
YOL127W	2:499012	1.73E-16
YOL127W	5:196196	3.09E-14
YOL127W	9:74540	2.85E-10
YOL127W	15:174364	3.42E-12
YKL161C	11:145839	1.48E-14
YKL161C	15:174364	1.56E-10
YMR179W	8:111690	9.92E-08
YDR297W	12:662627	2.43E-12
YOR061W	2:548401	2.53E-08
YDR306C	15:150651	3.23E-08
YPR162C	2:555575	8.82E-08
YDR453C	12:681096	2.42E-08
YDR453C	13:46070	2.73E-08
YDR453C	15:174364	4.19E-29
YGR295C	7:1081945	#######
YOR213C	14:449639	5.36E-17
YDR272W	4:975086	5.77E-26
YDR273W	4:975086	3.14E-09
YDR273W	15:174364	2.77E-07
YPR191W	15:113251	6.44E-08
YPR191W	16:500342	4.70E-08
YDR237W	2:555575	6.02E-09
YLR410W	13:27644	2.07E-18

YGR223C	7:940716	2.29E-44
YGR223C	13:77684	1.63E-07
YOL133W	15:59733	1.62E-16
YPR151C	12:662627	5.73E-87
YML032C	12:674651	5.75E-07
YML032C	13:205921	3.04E-12
YML096W	13:79786	3.09E-09
YMR309C	2:555575	1.46E-08
YMR315W	8:441790	4.83E-10
YFR002W	2:507428	2.86E-07
YLR077W	12:282397	5.06E-11
YHL048C-A	5:15697	2.26E-17
YHL006C	8:111680	4.26E-14
YJR152W	2:551299	2.46E-41
YJR152W	16:500342	5.24E-13
YDL182W	2:480009	3.77E-17
YDL182W	13:27644	2.40E-09
YER150W	13:46084	3.37E-08
YER150W	15:174364	3.39E-40
YBR154C	2:555575	1.32E-07
YAR008W	1:141181	2.65E-12
YAR008W	13:28622	3.53E-11
YER066W	15:143597	1.83E-10
YGL190C	15:154309	3.46E-07
YDL243C	6:28029	1.45E-33
YMR180C	2:555575	2.35E-09
YDL230W	2:565216	2.40E-08
YDL230W	4:74695	3.63E-07
YJR010C-A	10:461201	1.16E-33
YDR186C	12:662627	2.35E-07
YGR084C	2:551299	7.22E-10
YDL095W	2:516889	2.72E-09
YNL270C	2:480009	5.79E-08
YJL208C	15:143597	1.46E-09
YKR052C	13:922256	7.42E-08
YFL004W	13:27644	1.86E-30
YNL240C	2:555575	1.12E-07
YBR175W	2:562415	1.07E-10
YCL027W	2:573491	3.59E-07
YCL027W	8:111683	8.71E-31
YCL027W	13:49894	1.41E-16
YER163C	5:504714	6.94E-14
YOR101W	15:515923	2.79E-25
YLR244C	12:635380	1.26E-24
YNR037C	2:551299	3.37E-10
YOL047C	2:562415	3.01E-34
YLR172C	15:174364	1.29E-18

YBR165W	2:567221	3.21E-17
YGR211W	7:913059	8.00E-10
YER054C	13:46084	9.52E-10
YER054C	15:174364	1.51E-36
YOL022C	15:174364	7.72E-14
YLR049C	12:238298	1.70E-72
YNL045W	15:170945	9.57E-09
YHR143W-A	15:174364	1.72E-08
YOL043C	15:248746	2.52E-23
YLR430W	12:990751	7.42E-11
YEL054C	2:499012	3.93E-12
YEL054C	5:196196	2.37E-13
YEL054C	15:170945	1.31E-13
YIL091C	15:174364	1.72E-09
YKL186C	11:98336	6.23E-19
YEL047C	12:659357	1.44E-07
YEL047C	15:144659	2.13E-08
YJL024C	10:398821	4.43E-09
YML057W	13:158910	1.65E-22
YPR020W	16:500342	3.75E-08
YDR129C	15:154309	1.08E-07
YAL005C	1:136161	3.96E-32
YAL005C	10:387893	4.58E-09
YAL005C	15:170945	5.67E-10
YFL017C	15:143597	5.59E-09
YAL029C	15:150651	2.46E-08
YGL063W	7:375499	1.85E-20
YMR125W	8:167504	2.26E-10
YJL131C	15:174364	1.48E-10
YPL092W	16:368296	7.59E-35
YGL066W	7:375499	1.06E-10
YML026C	4:95527	4.97E-08
YML026C	5:193876	1.85E-09
YML026C	15:170945	3.06E-12
YML026C	16:500342	6.16E-09
YNL160W	15:174364	4.31E-16
YMR242C	2:499012	2.34E-11
YMR242C	5:194873	1.69E-10
YMR242C	9:74540	3.81E-07
YMR242C	15:170945	1.22E-12
YKL072W	15:174364	3.43E-16
YGL062W	2:481439	7.32E-07
YGL062W	9:19607	1.66E-09
YGL062W	12:274240	3.87E-11
YMR116C	2:521415	2.56E-12
YMR116C	5:196196	9.43E-14
YMR116C	9:74540	5.53E-10

YMR116C	15:174364	1.93E-18
YMR116C	16:500342	4.74E-13
YLR204W	15:154177	7.59E-08
YGR239C	5:312672	7.22E-08
YER073W	3:91305	1.61E-19
YER073W	13:46070	2.48E-09
YMR196W	13:77684	3.74E-09
YMR196W	15:174364	6.94E-25
YJL144W	7:375499	1.54E-19
YBL105C	6.628472222	5.39E-10
YBR111C	15:174364	2.32E-08
YGR031W	13:46084	5.95E-09
YGR031W	15:136327	2.31E-09
YDL109C	4:263770	2.10E-08
YPR060C	15:174364	6.70E-12
YHR209W	2:555787	2.24E-07
YIR012W	15:174364	2.28E-10
YMR055C	13:379981	7.55E-50
YNL309W	8:111679	6.56E-08
YBR085C-A	2:419093	1.33E-17
YBR085C-A	3:79091	6.01E-08
YKL084W	13:46084	3.11E-08
YKL084W	15:174364	6.37E-18
YMR170C	2:551299	1.78E-07
YGR201C	15:174364	4.85E-24
YPL043W	15:174364	1.27E-08
YGL185C	2:530481	1.11E-10
YNR050C	2:477206	3.89E-44
YLR270W	12:683463	5.51E-11
YLR270W	15:174364	1.84E-12
YDR346C	12:662627	3.07E-11
YIL070C	2:551299	3.32E-13
YIL070C	16:500348	2.94E-10
YJR025C	10:472147	4.75E-43
YLR150W	2:489202	5.46E-12
YLR150W	15:154177	1.70E-13
YLR150W	16:500342	3.09E-11
YKL091C	13:77684	5.64E-07
YKL091C	15:154309	7.25E-25
YKR016W	15:174364	6.09E-09
YKL185W	2:555596	4.07E-18
YJR045C	2:551299	6.13E-16
YJR045C	7:375499	1.25E-07
YJR045C	8:167506	7.35E-07
YDL235C	15:174364	5.49E-08
YGR233C	13:27644	3.60E-38
YNL101W	2:555596	9.24E-13

YEL011W	15:174364	4.57E-11
YFL017W-A	6:100521	1.86E-12
YFL061W	5:420595	2.12E-09
YLR368W	2:570229	1.62E-07
YLR368W	12:855389	9.14E-11
YDR418W	2:489202	1.48E-11
YDR418W	5:196196	1.83E-10
YDR418W	15:170945	7.14E-14
YHL042W	2:551299	1.35E-07
YJR138W	2:555596	2.55E-07
YCL063W	14:449639	6.43E-22
YPL196W	15:170945	1.48E-12
YJR055W	15:174364	8.49E-12
YBL016W	2:199107	1.18E-20
YBL016W	8:111683	2.71E-27
YBL016W	13:49894	2.08E-09
YML060W	8:149618	5.65E-08
YIL083C	9:195965	4.02E-11
YJR110W	8:175255	5.54E-07
YGR028W	7:553877	5.98E-07
YFL064C	4:1511257	4.82E-08
YBR040W	8:111682	1.69E-20
YNL033W	14:586789	1.99E-20
YDR244W	1:52943	3.57E-09
YDR244W	8:151814	2.94E-08
YKL178C	3:201166	#######
YGR033C	7:543259	2.32E-14
YGR033C	15:141633	3.84E-09
YHR030C	2:555596	2.98E-13
YHR030C	9:47053	8.85E-09
YHR030C	13:69114	3.68E-07
YHR030C	15:143597	6.10E-10
YDR352W	2:551299	6.13E-08
YKR031C	2:551299	4.58E-10
YGR217W	15:143597	3.03E-12
YML030W	2:537314	3.15E-08
YML030W	13:99675	7.99E-08
YML030W	15:154309	3.41E-14
YML030W	16:500348	2.47E-08
YNL051W	14:542648	1.76E-11
YOR059C	15:438824	5.55E-19
YGL128C	15:150651	3.61E-07
YKR071C	3:90676	1.58E-20
YLR259C	2:551299	1.22E-16
YLR259C	7:375499	2.10E-16
YLR259C	8:167506	4.93E-09
YLR259C	16:500348	9.82E-08

YCR102C	12:1059928	4.30E-15
YKL148C	5:504717	4.87E-11
YKL148C	11:174008	1.13E-20
YBR048W	5:196196	7.03E-08
YIL104C	7:707950	2.53E-13
YIL104C	9:190866	6.40E-27
YMR063W	13:391767	1.32E-07
YDR178W	5:500789	3.12E-08
YMR208W	12:662627	1.43E-25
YDR202C	15:174364	1.80E-12
YAL026C	13:28694	1.87E-07
YJL137C	15:174364	1.25E-09
YJR039W	15:154309	5.31E-09
YKL163W	11:146603	7.53E-13
YHR201C	8:111683	1.78E-14
YLR179C	12:514835	2.76E-48
YLR179C	13:78655	4.27E-12
YIL014W	9:325320	4.59E-27
YPR122W	8:111680	1.19E-13
YJL189W	4:95527	3.74E-08
YJL189W	5:183958	3.31E-07
YJL189W	15:170945	9.57E-11
YOR019W	15:357194	9.62E-48
YOR250C	15:804686	6.49E-15
YMR244C-A	15:180961	1.93E-11
YIR037W	15:174364	2.30E-10
YEL034W	12:662627	4.25E-44
YPR091C	15:174364	9.19E-12
YJR097W	15:174364	8.21E-10
YMR251W-A	2:537314	1.44E-14
YMR251W-A	15:143597	4.17E-08
YMR251W-A	16:500342	5.39E-20
YHL009C	8:84437	2.07E-12
YER177W	15:150651	6.69E-08
YOR038C	15:382531	5.75E-09
YHR207C	15:174364	9.25E-09
YFL054C	6:18384	7.28E-36
YFL054C	15:154309	8.55E-08
YLR388W	2:499889	1.25E-15
YLR388W	5:200734	1.65E-09
YLR388W	15:143597	5.45E-11
YGL160W	12:662627	2.09E-26
YML016C	13:243624	1.47E-15
YHR103W	13:57145	2.34E-08
YGR264C	15:170945	1.43E-15
YPL048W	15:174364	6.78E-09
YOL018C	15:298710	1.40E-17

YBR168W	2:573491	6.45E-08
YER016W	8:111683	3.35E-12
YML070W	13:99675	7.52E-10
YML070W	15:174364	4.11E-12
YGL229C	2:567221	1.78E-15
YGL229C	15:154309	4.65E-08
YLR246W	12:635380	7.83E-35
YDR270W	4:975086	1.57E-09
YGR175C	15:170945	1.13E-13
YKR026C	15:170945	7.85E-10
YAL064C-A	1:11626	3.28E-33
YJR075W	10:573371	1.47E-15
YML063W	2:499012	1.15E-08
YML063W	4:100720	2.79E-08
YML063W	5:196196	7.22E-12
YML063W	9:74540	5.32E-10
YML063W	15:174364	4.44E-13
YML063W	16:500342	5.96E-09
YJL048C	12:659357	3.61E-26
YLL039C	2:516889	3.98E-08
YLL039C	5:196196	7.84E-08
YJR054W	2:551299	7.40E-10
YJR054W	15:174364	9.24E-09
YOL140W	13:49894	1.66E-07
YOL140W	15:43217	7.53E-15
YEL058W	2:548401	4.09E-08
YEL058W	15:174364	3.56E-09
YDR211W	15:174364	1.95E-14
YML118W	2:569420	6.58E-08
YNL044W	15:174364	7.29E-09
YAL031C	8:167504	4.00E-11
YAL023C	2:551299	1.31E-09
YGL257C	7:12939	4.13E-14
YBR228W	2:551299	8.28E-08
YCL035C	15:174364	5.33E-17
YLR388W	2:499889	4.58E-15
YLR388W	5:200734	2.70E-09
YLR388W	15:143597	3.46E-10
YOL006C	15:136327	2.05E-09
YLR175W	15:174364	1.53E-07
YDL189W	4:122293	2.08E-13
YLR251W	15:174364	1.30E-11
YPL221W	2:537314	1.29E-07
YPL221W	15:143597	3.68E-07
YOR054C	15:438824	1.97E-09
YDL181W	13:28622	4.32E-10
YDL181W	15:141627	1.84E-21

YDL181W	16:500348	1.27E-15
YHR054C	8:209167	3.30E-29
YKR094C	2:499012	7.34E-10
YKR094C	5:196196	5.37E-07
YKR094C	15:170945	6.87E-13
YGL065C	15:170945	3.69E-08
YDL093W	12:662627	1.28E-12
YPR128C	1:42633	1.89E-09
YGL101W	12:662627	4.99E-36
YMR096W	14:449639	2.22E-26
YJL080C	12:662627	6.27E-07
YDR096W	15:174364	1.05E-17
YKL062W	15:143597	5.10E-19
YAL064W	1:11638	4.96E-12
YPL128C	2:516889	1.16E-07
YAR066W	8:549634	6.98E-20
YER028C	4:226317	8.58E-17
YEL050C	15:174364	1.12E-08
YAL062W	1:10152	3.07E-42
YAL062W	2:551299	7.08E-09
YAL062W	8:525664	1.31E-06
YDL064W	8:167504	8.74E-09
YKL164C	2:555596	4.35E-13
YBR067C	8:111686	3.60E-11
YBR067C	12:662627	1.58E-11
YBR067C	13:46084	8.28E-09
YJR094C	10:602943	8.07E-47
YDR504C	4:1471859	1.57E-13
YDR504C	15:150651	6.06E-08
YAR020C	1:154328	2.23E-12
YGR001C	7:497987	3.42E-11
YGR001C	15:174364	8.55E-08
YGR068C	8:111683	2.94E-07
YNL087W	15:174364	1.33E-19
YPL192C	8:111683	6.50E-20
YHR188C	12:634226	7.02E-08
YPL084W	3:90610	5.96E-07
YPL084W	15:174364	1.18E-07
YEL065W	2:551299	4.01E-15
YEL065W	7:375499	2.73E-08
YEL065W	12:956534	1.25E-06
YEL046C	15:174364	1.68E-19
YLR073C	12:282091	2.28E-38
YLR344W	2:499889	1.32E-11
YLR344W	5:193876	2.04E-11
YLR344W	15:170945	7.72E-14
YJR112W-A	10:627628	9.91E-18

YLR312C	15:170945	1.81E-07
YEL017W	5:116530	3.16E-10
YDR018C	15:170945	3.89E-12
YIL046W	9:268412	1.32E-19
YIL046W	15:150651	7.89E-09
YDR222W	2:551299	4.30E-09
YBL082C	2:73342	7.26E-25
YBL082C	13:77684	7.14E-07
YOR020C	7:375499	3.64E-12
YPL125W	16:318068	5.77E-33
YIL017C	3:201166	4.60E-21
YBR106W	13:850520	3.01E-07
YHR089C	2:551299	8.52E-08
YHR089C	15:143597	5.31E-07
YBR286W	15:174364	2.20E-22
YJL154C	10:135902	2.13E-40
YOL117W	15:154309	5.44E-07
YIL105C	15:154177	1.52E-10
YNL015W	2:569414	7.45E-21
YNL015W	15:174364	1.21E-07
YJL163C	10:110038	4.03E-12
YJL163C	15:154309	8.08E-14
YPL016W	16:523450	2.99E-34
YKR067W	11:554200	8.78E-08
YKL193C	15:170945	2.78E-09
YOL152W	13:46084	6.66E-14
YOL152W	15:108577	1.68E-07
YOL152W	16:500348	1.84E-10
YML098W	13:81250	1.88E-09
YIL140W	2:555787	2.21E-08
YIL140W	8:111682	8.48E-14
YNL108C	15:174364	2.17E-11
YLR397C	12:899946	5.08E-16
YHL035C	13:910381	2.44E-08
YLR213C	15:150651	9.80E-09
YLR245C	12:635380	1.41E-23
YHR176W	15:144659	7.01E-08
YLR048W	2:499012	2.73E-15
YLR048W	5:194873	7.51E-14
YLR048W	15:154309	2.32E-14
YLR048W	16:500342	2.21E-10
YDR369C	13:99675	3.05E-08
YDL054C	8:176670	2.35E-07
YGR146C	8:111683	2.24E-07
YOL130W	15:143597	3.00E-09
YOR056C	2:555596	3.04E-13
YPL170W	12:662627	8.69E-10

YKR066C	1:198274	3.79E-08
YKR066C	11:532483	1.01E-10
YPL083C	16:387239	7.58E-20
YOR381W-A	13:922268	2.23E-08
YKR090W	11:612769	2.37E-13
YOL155C	9:19607	1.98E-17
YOL155C	15:47951	2.12E-12
YFL013C	6:134252	7.59E-11
YKR063C	15:170945	1.78E-13
YGR199W	8:156412	3.46E-10
YBR173C	2:582419	1.17E-08
YOR120W	5:196352	4.08E-07
YOR120W	15:174364	3.30E-21
YAL028W	12:659357	2.32E-13
YJR008W	2:517365	2.67E-10
YJR008W	10:450212	4.24E-11
YJR008W	15:174364	7.44E-19
YMR319C	13:922258	1.94E-20
YMR319C	15:154309	7.77E-10
YOL017W	8:111682	5.61E-10
YNL249C	14:171103	4.06E-41
YKL204W	2:530481	2.67E-08
YBL072C	2:499012	8.05E-09
YBL072C	5:194873	1.09E-11
YBL072C	15:174364	4.10E-15
YBL072C	16:500342	3.76E-09
YOL128C	2:551299	6.04E-13
YOL128C	12:662627	1.26E-10
YNR012W	15:174364	5.52E-14
YGR288W	7:1074345	4.02E-08
YOR010C	5:350744	8.21E-10
YDR012W	5:193876	1.00E-09
YDR012W	15:170945	8.69E-11
YGL022W	15:150651	7.13E-08
YBL013W	15:143597	1.04E-15
YPR013C	13:87587	1.61E-08
YMR171C	13:597705	7.05E-17
YBL097W	6.628472222	1.49E-07
YHR003C	12:662627	1.64E-07
YNR034W	14:689939	4.39E-13
YNR034W	15:174364	5.44E-08
YNR057C	5:272255	1.85E-15
YHR156C	15:174364	2.68E-08
YNR011C	14:568280	2.80E-07
YOR147W	8:213595	2.50E-07
YOR147W	15:589013	7.98E-09
YHR199C-A	2:551299	1.06E-09

YHR199C-A	8:175255	9.57E-09
YHR199C-A	13:100048	5.39E-08
YHR199C-A	16:500348	8.61E-11
YFR005C	3:90610	7.64E-07
YOR316C-A	2:553812	2.25E-32
YEL052W	13:49894	1.23E-08
YEL052W	15:174364	6.87E-12
YOR312C	5:196196	1.11E-08
YOR312C	15:170945	6.13E-09
YEL055C	15:174364	1.81E-07
YIL046W-A	9:268352	3.80E-14
YDL213C	15:174364	8.80E-10
YJR013W	10:461201	9.46E-09
YGR052W	15:174364	7.08E-36
YKR077W	8:111679	6.05E-07
YDR356W	4:1185630	1.54E-09
YJL187C	8:111683	1.79E-15
YPL168W	2:551299	1.18E-09
YJL206C	2:555575	2.49E-08
YJL057C	10:327858	9.23E-33
YNL178W	2:499889	1.49E-16
YNL178W	4:62325	1.02E-13
YNL178W	5:196196	2.89E-12
YNL178W	9:74540	7.71E-12
YNL178W	15:170945	1.53E-12
YGL004C	15:174364	3.85E-11
YDR430C	2:551299	2.57E-09
YDR430C	15:170945	2.74E-07
YDR430C	16:500348	2.12E-07
YCR099C	2:551299	4.48E-08
YPL278C	13:910381	8.56E-13
YPL278C	15:1065719	1.59E-20
YMR205C	2:562415	1.37E-06
YMR205C	12:662627	6.19E-08
YIL092W	7:708022	4.98E-07
YMR236W	2:567221	5.27E-08
YMR236W	8:95469	6.99E-09
YMR236W	13:49894	4.55E-08
YLL013C	12:126934	1.66E-50
YPL005W	16:555416	2.10E-11
YIL108W	15:154177	1.07E-09
YGR041W	2:562415	1.19E-26
YCR048W	12:662627	5.24E-07
YJL008C	10:451832	1.47E-07
YER154W	2:555575	2.21E-09
YOL019W	2:551299	1.10E-08
YOL019W	15:170945	1.88E-14

YKR027W	3:201166	7.09E-10
YER046W	5:243215	5.48E-15
YIL169C	9:19607	4.18E-16
YBR003W	15:154309	1.59E-09
YDR319C	4:1105243	7.50E-11
YDL117W	2:551299	3.59E-07
YOR306C	15:889464	5.56E-14
YGR281W	2:507282	1.58E-07
YGR281W	7:1081966	8.01E-19
YGR281W	12:662627	3.94E-08
YGR281W	16:500348	4.41E-08
YDR165W	15:174364	8.89E-08
YMR229C	15:174364	8.91E-09
YMR058W	2:551299	2.20E-13
YMR058W	12:956534	1.54E-06
YIL018W	2:499012	1.45E-14
YIL018W	5:196196	4.53E-11
YIL018W	9:74540	2.22E-08
YIL018W	15:154309	1.89E-13
YIL018W	16:500342	7.61E-10
YBR183W	12:659357	2.68E-10
YKL130C	11:194611	1.07E-11
YBL005W-A	2:246129	2.89E-09
YKR034W	2:551299	3.80E-50
YKR034W	16:500342	5.29E-09
YMR264W	15:136327	5.46E-08
YHR037W	16:500342	1.66E-08
YER095W	2:569420	2.31E-09
YNL146C-A	3:201166	6.09E-95
YLL002W	8:111683	1.44E-07
YOR126C	2:573491	1.01E-07
YPL054W	2:553812	1.22E-11
YPL054W	4:192248	2.62E-09
YPL054W	16:495156	1.02E-11
YOR291W	15:174364	6.24E-10
YCL057W	14:449639	4.59E-23
YDR038C	4:527458	#######
YDR038C	11:98332	1.39E-09
YGR109W-A	7:708252	5.34E-12
YGR109W-A	8:111683	5.35E-08
YGR109W-A	9:200332	1.51E-16
YLR069C	2:537314	6.65E-14
YLR069C	16:500348	5.49E-10
YNL063W	10:548177	2.15E-21
YLR295C	15:136327	7.04E-10
YFL026W	3:201167	#######
YJL079C	13:46070	1.74E-07

YDR367W	4:1213416	5.03E-60
YGL162W	7:192140	9.57E-24
YGL040C	2:555575	8.81E-15
YGL040C	15:141633	2.02E-07
YGL040C	16:500348	2.88E-12
YGR227W	8:111680	1.93E-07
YML078W	13:115474	4.08E-27
YLR200W	15:174364	1.42E-07
YMR122W-A	2:555575	1.50E-08
YNL244C	13:110808	2.33E-07
YBR167C	2:562415	1.12E-18
YGL164C	2:573491	1.56E-09
YOR292C	15:170945	3.33E-11
YDR437W	8:156412	1.05E-08
YGL212W	7:92896	3.03E-39
YHL022C	8:56246	1.12E-22
YER180C	5:549142	1.00E-69
YDR130C	13:46084	6.51E-08
YKL209C	3:201166	#######
YMR144W	8:111682	1.24E-09
YHR001W-A	15:141627	2.47E-09
YDR060W	15:174364	6.47E-11
YIL079C	15:174364	5.59E-10
YOL069W	13:49894	3.89E-09
YPL118W	15:154177	3.86E-09
YDR341C	15:174364	3.64E-11
YBL015W	2:191171	3.92E-09
YBL015W	15:174364	2.46E-09
YKL212W	15:143597	1.10E-07
YJL014W	15:150651	1.33E-09
YJR004C	3:201166	1.54E-66
YJR004C	8:111686	9.51E-14
YJR004C	10:450212	1.28E-12
YPR134W	2:551299	6.53E-08
YPR110C	15:174364	7.28E-10
YHL012W	8:95469	6.10E-27
YGR034W	2:499012	1.19E-12
YGR034W	4:95527	2.69E-08
YGR034W	5:194873	3.15E-11
YGR034W	9:74540	1.32E-09
YGR034W	15:170945	1.23E-13
YHR214C-E	13:27644	1.34E-19
YEL026W	5:196196	2.15E-11
YEL026W	15:174364	1.44E-17
YLR205C	12:662627	4.12E-76
YNL293W	2:551299	2.00E-08
YKL024C	15:143597	2.70E-09

YPR103W	8:176670	8.42E-07
YEL063C	2:553812	1.16E-30
YEL063C	16:500342	3.72E-12
YBR057C	8:111683	9.68E-09
YFL041W	2:582419	1.08E-08
YKL004W	15:150651	1.49E-08
YBR037C	2:551299	1.28E-08
YBR037C	15:174364	3.95E-09
YBR037C	16:500348	1.04E-07
YJL076W	2:516889	6.98E-07
YFR044C	13:46084	1.59E-09
YFR044C	15:141633	1.10E-07
YKR018C	15:180961	2.65E-08
YOL041C	15:174364	1.93E-08
YDR032C	8:95469	6.36E-08
YDR032C	15:174364	2.72E-08
YER165W	2:506661	7.61E-13
YER165W	13:69114	4.07E-07
YER165W	15:143597	2.40E-13
YFR011C	6:168354	7.66E-10
YFR011C	15:154177	6.72E-09
YNL142W	2:555575	1.57E-42
YNL142W	16:500342	1.02E-09
YKL100C	15:174364	4.06E-11
YMR192W	13:649250	4.48E-27
YPL024W	16:500348	2.61E-12
YBR282W	16:511406	6.65E-07
YKL201C	11:47707	4.23E-14
YIL117C	2:551299	8.76E-21
YIL117C	15:143597	3.16E-12
YER169W	15:174364	1.57E-09
YML087C	13:99675	4.05E-11
YLR382C	2:530481	5.13E-11
YLR382C	15:150651	1.98E-07
YLR382C	16:500348	2.60E-08
YIL164C	2:551299	6.78E-17
YIL164C	9:33807	3.22E-24
YAR007C	1:141181	3.45E-19
YFR017C	15:174364	1.14E-14
YER182W	15:170945	1.80E-12
YER014W	12:662627	2.55E-15
YDL137W	4:223324	3.38E-09
YML129C	2:551299	9.89E-12
YML129C	16:511406	1.20E-08
YPL183W-A	15:174364	1.17E-06
YPL106C	16:368086	1.26E-76
YEL064C	2:551299	5.01E-15

YLR094C	12:327131	1.15E-48
YDL215C	3:81832	5.64E-07
YER162C	15:174364	8.16E-10
YOR044W	8:175255	4.81E-07
YOR086C	15:174364	1.45E-08
YMR185W	15:136327	4.23E-08
YJL044C	10:353027	8.58E-19
YBR004C	2:246129	3.22E-13
YER063W	2:555575	4.71E-13
YOL080C	15:150651	1.01E-09
YBR230C	15:174364	3.70E-09
YOR376W-A	8:167506	3.52E-08
YOR376W-A	16:500348	5.89E-09
YJR010W	8:161987	6.39E-14
YJR010W	10:461201	1.62E-21
YOR012W	15:348934	1.34E-50
YOL081W	15:170945	8.03E-10
YLR173W	12:469156	4.06E-22
YOR148C	15:594024	3.49E-09
YDR471W	2:499012	4.69E-10
YDR471W	5:193876	5.33E-12
YNL098C	15:174364	3.31E-08
YOL002C	7:402835	1.77E-10
YDR514C	2:555575	1.27E-09
YGR112W	15:174364	2.05E-11
YMR281W	13:824809	1.38E-35
YDL128W	15:589013	7.22E-08
YOR205C	2:519049	1.11E-07
YCR037C	13:27644	1.79E-44
YDR300C	15:174364	1.89E-15
YMR107W	15:174364	1.93E-18
YOR381W	12:582499	4.53E-12
YOR381W	13:910381	4.46E-12
YJR048W	12:662627	4.48E-26
YBL041W	2:142262	2.74E-31
YAL036C	15:174364	3.19E-08
YOL143C	5:117056	3.82E-99
YDR508C	2:551299	7.47E-24
YDR508C	16:500348	2.20E-10
YKL096W-A	15:154177	3.10E-13
YGL197W	7:117900	5.00E-12
YJL046W	10:374040	1.02E-08
YBR298C	7:1058950	4.95E-20
YOR046C	15:170945	8.67E-09
YNL175C	14:314883	1.98E-11
YNL175C	15:174364	6.50E-08
YJL141C	15:174364	1.99E-14

YJR060W	10:548177	1.36E-60
YJR060W	13:28694	2.59E-10
YPL184C	14:449639	2.21E-44
YPL240C	7:375499	1.92E-14
YJL212C	10:39595	3.08E-08
YGR082W	2:551299	5.34E-23
YGR082W	7:375499	5.47E-09
YGR082W	8:167506	3.30E-13
YGR082W	16:500348	4.77E-17
YNL283C	2:551299	1.10E-12
YPL030W	15:170945	2.65E-13
YMR165C	15:174364	2.83E-10
YDR046C	15:180222	1.18E-09
YDL217C	4:78583	5.99E-12
YDL130W	2:519049	1.83E-16
YDL130W	4:95437	1.72E-07
YDL130W	5:194873	2.26E-12
YDL130W	15:143597	1.11E-13
YDL130W	16:500342	1.69E-12
YBR021W	2:301671	2.36E-15
YDR463W	5:312672	1.12E-09
YCL056C	3:14066	5.36E-13
YCL056C	8:111682	1.32E-10
YBR146W	2:537314	5.27E-17
YBR146W	16:500348	2.90E-07
YKR065C	2:551299	1.26E-07
YEL060C	2:507428	2.65E-09
YGR066C	2:567221	1.96E-11
YHR034C	8:167504	9.34E-09
YHR043C	8:193175	1.11E-47
YOR004W	15:150651	2.03E-08
YER042W	8:161987	1.73E-13
YJL012C	13:27644	1.52E-47
YJL012C	15:143597	3.05E-08
YGR086C	15:143597	2.11E-13
YGR086C	16:500342	4.96E-09
YBR105C	5:116554	6.05E-08
YBR105C	13:99675	1.33E-08
YBR105C	15:150651	2.64E-07
YMR261C	15:174364	2.93E-12
YJL103C	15:154309	8.55E-10
YLR369W	12:858996	5.10E-16
YIL173W	9:38608	9.40E-21
YIL173W	10:22273	2.27E-20
YPL207W	15:174364	1.79E-17
YLR446W	2:537314	3.14E-08
YLR446W	15:174364	9.91E-14

YLR394W	15:174364	8.55E-08
YBR199W	2:622993	1.26E-09
YER055C	13:33501	7.39E-08
YIR016W	15:174364	6.99E-22
YOL084W	15:144659	5.31E-50
YMR118C	2:562415	9.14E-22
YMR118C	4:100720	4.43E-12
YMR118C	15:144659	1.77E-10
YPL239W	15:174364	7.48E-14
YNL267W	13:81250	3.70E-10
YJR116W	7:403626	1.15E-06
YOR206W	15:174364	3.13E-08
YBL006C	2:216978	4.82E-11
YNL186W	5:196196	5.12E-08
YBR060C	2:364851	2.22E-09
YFR055W	9:79793	1.42E-07
YFR055W	16:511400	6.54E-13
YFR014C	15:170945	1.06E-21
YGR184C	7:860665	5.91E-18
YKL159C	11:153463	2.27E-32
YFL055W	6:18384	9.07E-25
YFL055W	10:22309	3.69E-16
YHR195W	15:174364	4.94E-13
YIL064W	9:242417	4.02E-09
YHL036W	15:174364	2.78E-11
YLR355C	3:91305	5.04E-19
YLR355C	13:46084	1.18E-18
YLR355C	15:179289	1.35E-08
YMR215W	4:100720	4.74E-08
YOR060C	15:418282	4.28E-11
YIL097W	15:154309	8.43E-08
YBR114W	15:174364	2.03E-08
YJR098C	10:612602	1.25E-17
YNR036C	2:551299	9.96E-09
YNR036C	15:141627	1.04E-10
YNL065W	5:117046	2.58E-07
YNL065W	14:502496	8.46E-39
YGR076C	2:551299	1.42E-07
YDL061C	2:499889	2.00E-13
YDL061C	4:95527	5.60E-10
YDL061C	5:200848	4.01E-10
YDL061C	15:170945	2.07E-16
YHL033C	2:499012	7.39E-12
YHL033C	5:194873	1.16E-10
YHL033C	15:170945	7.74E-14
YNL066W	2:555787	1.91E-44
YDL247W	10:703868	1.56E-14

YMR114C	15:174364	3.87E-12
YKR060W	15:174364	2.52E-09
YLR193C	13:28622	1.92E-08
YLR193C	15:174364	2.12E-11
YJR035W	10:503030	1.45E-38
YPL175W	15:174364	1.79E-13
YMR016C	2:427677	6.26E-08
YMR016C	15:150651	2.26E-15
YKL008C	2:584357	1.01E-07
YKL008C	12:634227	3.32E-08
YBR132C	2:499895	5.55E-52
YLR354C	12:852066	4.05E-14
YOR158W	2:551299	8.94E-10
YPL023C	16:511406	5.71E-44
YDL024C	2:537314	1.20E-10
YDL024C	15:170945	3.38E-14
YLR054C	2:562415	1.49E-07
YLR054C	12:247898	5.01E-16
YDL127W	8:111680	5.86E-09
YAL012W	8:176412	1.04E-07
YAL012W	13:49894	4.37E-09
YAL012W	15:174364	5.58E-10
YOL131W	2:533262	4.59E-12
YOL131W	15:143597	3.31E-25
YNL077W	7:375499	1.25E-29
YNL180C	14:281187	8.19E-12
YLL019C	15:174364	2.28E-11
YJR016C	3:81832	8.24E-21
YJR016C	15:179289	1.66E-10
YLR070C	12:264911	9.15E-14
YLR070C	15:170945	9.56E-17
YIL134C-A	6:234349	1.35E-07
YCL049C	13:46084	8.68E-09
YBR091C	2:555575	8.11E-09
YOR192C	15:703769	1.50E-11
YJR021C	8:167506	1.58E-07
YOR193W	15:703771	1.67E-43
YIL055C	9:251537	1.02E-08
YIL127C	15:150651	1.32E-07
YDL083C	2:499012	1.00E-12
YDL083C	5:196196	2.11E-10
YDL083C	15:170945	5.16E-12
YDL083C	16:500342	4.26E-10
YGR256W	15:170945	6.42E-15
YNL093W	15:174364	8.33E-20
YJL219W	10:23409	1.14E-18
YLR353W	2:533268	9.73E-09

YLR353W	12:829693	1.84E-11
YLR273C	12:677957	3.93E-12
YJL095W	2:551299	7.60E-07
YNR028W	13:81358	3.62E-07
YLR445W	12:1023790	1.37E-09
YJL145W	2:562415	1.10E-11
YIL061C	9:242934	1.27E-20
YGR019W	2:555575	1.46E-07
YIR027C	2:555575	1.99E-33
YIR027C	13:110808	1.95E-12
YIR027C	16:500348	1.06E-09
YJL050W	10:380085	9.65E-32
YMR175W	15:170945	1.77E-18
YOL158C	9:19607	1.69E-06
YOL158C	15:43057	1.36E-08
YDL205C	4:89821	1.58E-46
YGR055W	2:551299	1.78E-28
YGR055W	8:167504	4.70E-13
YLR002C	15:150651	2.02E-07
YNL268W	2:477206	6.58E-10
YPL124W	8:111683	8.89E-10
YDR098C	2:533262	1.38E-09
YDR098C	15:174364	3.21E-20
YGL226C-A	7:73452	1.43E-18
YDR275W	12:659357	3.82E-09
YIL047C	2:537314	2.46E-10
YIL047C	15:174364	1.00E-15
YMR031C	15:174364	1.11E-15
YLR345W	15:174364	1.53E-17
YDL121C	15:170945	2.00E-08
YCR030C	15:174364	4.32E-10
YCL069W	11:656099	1.15E-31
YGL129C	2:551299	1.02E-08
YOL126C	5:117056	2.58E-71
YJL178C	10:89348	9.18E-34
YDL239C	2:553812	1.61E-25
YJR001W	2:565216	7.38E-08
YOL025W	2:555575	2.49E-07
YPL244C	13:69114	2.85E-08
YPL228W	16:109106	6.84E-09
YMR012W	2:555575	9.91E-08
YMR012W	15:150651	2.40E-07
YMR012W	16:500348	2.41E-09
YOL092W	15:154309	2.13E-28
YJL047C	16:500348	6.73E-08
YBR005W	2:246129	3.48E-09
YOR388C	10:59959	2.43E-10

YNR032W	8:111683	1.34E-10
YNR032W	13:99675	6.77E-11
YNL195C	15:174364	1.05E-22
YAR015W	1:141181	6.38E-19
YDR309C	13:96015	3.64E-10
YDR309C	15:174364	1.32E-11
YPL091W	16:387239	3.49E-19
YMR271C	2:517365	1.64E-08
YMR271C	15:143597	1.56E-08
YGR111W	15:174364	1.90E-11
YMR307W	8:111682	1.05E-07
YNL006W	7:375499	7.58E-13
YLR448W	2:499012	4.96E-15
YLR448W	5:200848	4.33E-12
YLR448W	15:170945	7.94E-12
YCR061W	12:662627	2.67E-09
YOL045W	2:562415	1.07E-09
YGR247W	7:995892	7.33E-37
YCL017C	3:81832	1.19E-25
YIR035C	15:136327	1.81E-07
YHL034C	15:174364	3.26E-09
YNL125C	2:537314	2.86E-12
YNL125C	15:143597	1.70E-08
YCL057C-A	15:108577	4.26E-07
YGR012W	7:514308	1.12E-36
YGL061C	8:95289	4.07E-14
YMR006C	13:277071	2.27E-31
YJR072C	8:176412	6.31E-07
YIR025W	9:403134	5.03E-14
YFR012W-A	6:168342	3.59E-09
YNL185C	2:551299	4.39E-12
YNL185C	16:500348	5.51E-10
YNL327W	2:555778	2.01E-30
YNL273W	8:111680	1.98E-08
YML024W	4:96271	1.25E-08
YML024W	5:196196	2.07E-08
YML024W	9:101011	2.65E-08
YML024W	15:174364	6.06E-10
YNL209W	2:555575	3.96E-12
YNL209W	5:196196	8.06E-09
YNL209W	15:143597	4.13E-19
YNL209W	16:500348	2.67E-14
YDR339C	15:174364	3.78E-09
YER122C	12:659357	1.00E-09
YLR348C	3:91287	3.07E-24
YLR348C	13:46070	2.80E-16
YDL142C	2:555575	2.23E-08

YDL142C	8:149618	2.07E-07
YDR303C	15:113260	3.94E-08
YDL231C	2:553812	4.18E-07
YDL231C	4:46316	1.56E-85
YDR436W	15:174364	7.37E-11
YIR032C	2:551299	2.11E-35
YIR032C	9:419722	6.68E-13
YIR032C	16:500342	8.05E-09
YIR029W	2:551299	1.96E-31
YIR029W	16:500342	3.00E-11
YOR167C	5:196196	1.09E-07
YOR167C	15:170945	6.77E-11
YOR310C	5:196190	2.50E-07
YOR310C	15:174364	5.97E-11
YMR305C	2:533262	2.55E-12
YOL048C	15:143597	1.95E-08
YDR185C	15:143597	1.20E-12
YOL052C-A	15:174364	7.37E-12
YHL026C	8:56246	2.93E-25
YLR392C	15:174364	1.68E-11
YNR041C	12:677957	1.61E-17
YER002W	15:174364	8.39E-09
YKL001C	8:175255	2.64E-12
YGL187C	15:141627	1.44E-07
YGL187C	16:500342	1.84E-10
YPL143W	2:499012	5.42E-10
YPL143W	5:196196	6.78E-10
YML042W	15:150651	8.86E-09
YOR081C	15:481586	4.04E-31
YER020W	5:196190	3.30E-34
YLR177W	15:174364	5.87E-16
YDR423C	4:1318073	3.46E-54
YPR035W	2:419093	1.59E-12
YPR035W	13:115474	2.20E-10
YBL019W	2:186293	4.20E-09
YDR131C	2:551299	5.53E-11
YDR131C	16:500348	1.57E-09
YJL161W	2:533262	2.24E-09
YJL161W	10:123859	1.47E-13
YJL161W	15:170945	7.92E-17
YMR110C	12:674651	1.62E-08
YBL035C	15:174364	2.44E-08
YJL181W	15:143597	9.93E-13
YNL236W	14:209852	1.16E-13
YDR089W	4:582121	4.22E-18
YOL039W	2:517123	2.80E-11
YOL039W	9:74540	2.57E-07

YOL039W	15:170945	2.02E-13
YOR154W	15:594024	4.04E-15
YJL066C	15:174364	7.78E-10
YLL024C	7:375499	8.74E-32
YDR265W	4:975086	1.11E-18
YBR299W	7:1081978	1.62E-12
YMR250W	5:196352	5.46E-08
YMR250W	15:174364	1.44E-13
YHR213W-A	6:38648	8.00E-15
YPR201W	11:655678	4.58E-19
YPR201W	16:932538	3.99E-16
YMR214W	15:174364	1.41E-13
YNR033W	15:174364	4.06E-11
YJL183W	10:81875	3.99E-09
YPL277C	13:922258	7.13E-15
YDR073W	4:579813	2.89E-08
YPL249C-A	2:499889	5.31E-10
YPL249C-A	5:200734	2.15E-09
YPL249C-A	15:170945	9.79E-08
YNR019W	12:662627	2.03E-31
YGR062C	15:174364	3.66E-08
YGR248W	5:196196	8.58E-11
YGR248W	7:974625	4.55E-17
YGR248W	15:174364	6.67E-24
YLR196W	15:150651	1.78E-07
YER019W	2:555575	3.01E-16
YER019W	5:194496	2.26E-07
YHR117W	2:565216	8.67E-09
YHR117W	13:149075	8.62E-08
YOL060C	15:210839	4.00E-23
YNL281W	7:375499	1.17E-15
YPL031C	16:500342	2.25E-12
YLL026W	15:174364	2.04E-12
YFR007W	2:551299	4.83E-10
YFR007W	16:500348	2.91E-08
YNL201C	8:72233	1.39E-08
YBR281C	2:769107	3.20E-15
YHR202W	2:555596	5.46E-10
YKR070W	11:574956	5.51E-16
YGR286C	2:537314	1.85E-11
YGR286C	5:272258	6.66E-22
YGR286C	15:154309	6.43E-08
YML026C	4:100720	3.13E-09
YML026C	5:193876	2.17E-10
YML026C	15:170945	9.97E-13
YGL173C	14:449639	3.77E-34
YIL078W	15:170945	1.10E-08

YIL154C	12:644136	1.04E-07
YNL200C	14:258590	5.74E-10
YNL200C	15:174364	5.77E-10
YMR194W	2:537314	1.59E-15
YMR194W	5:196196	6.58E-10
YMR194W	15:143597	7.19E-08
YOL146W	15:47951	4.94E-19
YKL126W	11:211595	1.98E-14
YMR182W-A	15:143597	2.11E-11
YKR036C	11:510933	2.96E-16
YOR354C	2:555778	2.30E-08
YOR354C	15:141627	1.29E-09
YOR354C	16:500348	4.81E-08
YNL100W	13:49894	7.25E-10
YNL100W	15:154309	3.94E-17
YHR020W	15:170945	2.77E-13
YLR023C	2:517123	7.15E-07
YLR023C	15:141621	1.83E-07
YHR161C	8:439711	5.96E-08
YBR171W	2:555575	1.28E-11
YGL049C	7:402851	4.88E-14
YBR103W	2:477206	1.56E-23
YBR196C-B	2:616262	2.79E-36
YPL108W	15:174364	7.31E-10
YKR046C	12:662627	4.42E-88
YHR012W	2:516889	1.59E-07
YJL026W	12:508029	2.64E-20
YGL207W	7:110813	1.10E-17
YOR066W	13:49894	5.00E-09
YPL015C	15:174364	2.09E-09
YIL093C	2:551299	2.56E-07
YER045C	15:141633	4.45E-11
YML076C	13:99672	4.32E-31
YBR088C	8:152932	3.10E-08
YDR533C	4:1501004	2.79E-09
YDR533C	15:143597	9.05E-49
YLR168C	12:450045	3.39E-11
YLR168C	15:141627	6.00E-09
YDL214C	4:62325	1.12E-28
YDL214C	15:174364	5.02E-13
YKR080W	11:586769	3.68E-14
YPL167C	16:239627	#######
YBR104W	2:486640	2.99E-21
YBR104W	15:170945	9.36E-10
YIR018W	9:387985	4.75E-11
YNL124W	14:412269	1.24E-09
YLR421C	2:517123	2.38E-07

YIL116W	9:141014	2.18E-32
YML100W	15:174364	5.55E-12
YPL012W	15:174364	2.22E-09
YER129W	12:693790	9.83E-08
YIL037C	8:111686	7.53E-11
YIL037C	9:277908	8.93E-09
YPL148C	16:266023	5.56E-12
YGR043C	15:143597	4.27E-26
YOR100C	15:524972	1.90E-08
YDL052C	13:115474	4.52E-07
YDL052C	16:500348	4.96E-11
YJR108W	10:627628	7.99E-66
YDR121W	8:167506	3.62E-07
YOR184W	15:704058	3.59E-07
YDR025W	2:499012	2.05E-15
YDR025W	5:196196	2.79E-11
YDR025W	15:170945	2.79E-12
YDR025W	16:500342	3.95E-11
YCR020C	2:551299	2.99E-09
YBR263W	2:551299	2.98E-07
YBR263W	15:143597	1.02E-07
YOR386W	2:499012	2.76E-11
YOR386W	15:174364	4.20E-11
YLR194C	15:143597	1.69E-10
YKL056C	15:170945	1.16E-09
YIL011W	5:350744	7.39E-19
YIL011W	9:341216	9.93E-17
YOL166W-A	15:17946	4.83E-19
YLL009C	2:551299	3.90E-09
YLL009C	16:500348	2.23E-07
YIL033C	15:170945	1.44E-10
YJL159W	2:565216	8.24E-08
YJL159W	10:122312	2.00E-09
YML066C	13:129925	3.33E-33
YPL011C	15:141621	2.75E-08
YPL011C	16:542307	3.86E-14
YNL322C	14:33361	3.55E-15
YDR522C	4:1491146	1.01E-08
YDL120W	8:152932	2.55E-07
YDL120W	16:511406	8.02E-10
YER091C	5:272258	3.52E-18
YER091C	8:167504	8.50E-10
YIL001W	8:167506	3.25E-08
YMR148W	15:143597	3.88E-18
YKR017C	11:468771	9.46E-24
YKR017C	15:144659	1.49E-07
YLR215C	12:582499	1.62E-27

YMR109W	13:481542	3.84E-19
YDL005C	4:463264	6.30E-08
YNL284C	15:154177	5.07E-07
YJL172W	2:551299	3.26E-07
YJL172W	10:99921	2.84E-25
YGR222W	7:940716	8.13E-14
YOR327C	10:548177	1.83E-17
YJL045W	2:537314	6.02E-09
YJL045W	10:380085	6.66E-16
YJL045W	15:143597	7.31E-12
YLR102C	2:562415	6.38E-07
YLR102C	15:143597	3.51E-12
YOL166W-A	3.133333333	1.03E-34
YPR085C	16:711614	1.77E-29
YJL023C	2:548401	2.03E-07
YJR104C	10:627628	4.67E-08
YJR104C	12:659357	1.86E-08
YCR071C	2:551299	1.37E-09
YAR028W	1:185122	2.06E-56
YOR138C	15:589145	4.68E-12
YGL078C	15:154177	2.89E-08
YHL038C	2:562415	2.01E-06
YOR107W	15:136327	7.73E-10
YPR023C	16:600664	5.16E-18
YLR406C-A	12:909226	2.93E-18
YDL207W	4:85846	2.20E-16
YGR158C	7:805409	3.32E-19
YIL142W	9:74540	1.00E-08
YPL183C	2:537314	1.88E-07
YGL006W-A	7:490784	7.36E-21
YDL247W	10:715254	1.35E-12
YAR064W	6:38648	4.16E-10
YLR183C	8:111683	1.54E-14
YGR037C	1:41483	1.03E-08
YLR092W	12:332574	1.07E-13
YJL116C	2:548401	1.25E-08
YJL116C	10:204137	7.20E-12
YDL150W	2:551299	2.92E-08
YDL150W	8:176412	1.15E-06
YDR166C	12:668249	1.13E-07
YML056C	5:196196	1.07E-07
YML056C	15:174364	4.15E-14
YPR144C	2:551299	4.76E-10
YNL264C	12:635380	6.69E-07
YGR187C	15:174364	8.45E-09
YJL146W	8:167506	1.98E-07
YHR092C	15:154309	1.67E-09

YML128C	1:41483	7.95E-13
YML128C	13:77684	1.49E-07
YML128C	15:174364	9.32E-29
YLL018C-A	13:46084	5.31E-08
YLL018C-A	15:174364	2.74E-22
YNL050C	14:542648	9.61E-11
YJR003C	15:174364	9.27E-16
YEL023C	2:551299	1.24E-08
YLR157W-C	12:472165	2.38E-94
YBR115C	2:477206	3.79E-83
YBR115C	5:251262	3.36E-08
YBR115C	8:80068	1.74E-08
YOR127W	2:537314	4.83E-07
YGR128C	15:174364	9.49E-11
YCR018C	3:105042	1.99E-31
YGL019W	7:460945	1.78E-10
YPL187W	3:201166	#######
YOR173W	5:200848	4.49E-10
YOR173W	15:174364	4.19E-44
YPL176C	2:555575	3.39E-08
YPL201C	16:90266	4.07E-14
YMR288W	8:111680	7.92E-13
YNL072W	8:111690	1.02E-10
YDR441C	4:1344670	7.33E-18
YCL051W	15:174364	2.40E-08
YGR125W	2:551299	3.47E-28
YCR089W	8:111683	1.38E-40
YCR089W	13:49894	1.50E-14
YOR207C	15:174364	9.64E-13
YHL019C	8:167506	1.07E-06
YDL203C	4:89821	7.89E-09
YDR507C	8:111683	1.58E-09
YDR382W	9:74540	8.79E-07
YDR382W	15:16691	4.14E-10
YML092C	8:176994	1.40E-07
YML092C	13:49894	3.99E-10
YNL282W	2:551299	1.41E-09
YPL277C	13:910381	7.20E-14
YPL277C	15:1065719	1.76E-19
YCR031C	2:519049	5.96E-10
YCR031C	4:95437	4.31E-09
YCR031C	15:174364	1.35E-12
YPL211W	15:174364	1.92E-08
YNL081C	15:141627	1.37E-08
YDL241W	15:154309	3.35E-15
YDR351W	15:174364	4.58E-08
YER131W	2:499012	1.79E-14

YER131W	5:196196	6.33E-11
YER131W	15:170945	1.10E-09
YER131W	16:500342	2.56E-10
YMR113W	12:679808	2.01E-07
YMR113W	13:494170	8.62E-25
YGR040W	2:567221	1.90E-17
YGR040W	7:530473	1.34E-07
YHR127W	8:111679	2.53E-07
YGR003W	7:502131	1.65E-15
YJL191W	2:489202	1.59E-08
YJL191W	5:193876	1.05E-17
YJL191W	15:170945	3.99E-13
YKL170W	2:551299	5.87E-07
YER153C	2:555596	1.59E-15
YIL089W	2:555575	4.52E-09
YIL089W	9:196145	8.86E-56
YFL036W	2:562409	1.17E-11
YFL036W	15:154309	4.45E-08
YPL257W	16:70847	2.91E-31
YDL197C	4:115570	7.98E-14
YDR092W	4:582121	2.74E-11
YMR240C	13:100048	3.62E-07
YMR240C	16:500348	2.28E-08
YJR131W	10:646911	2.83E-35
YJL213W	10:34098	5.41E-49
YJL213W	13:49903	1.35E-10
YHR183W	2:507282	2.33E-10
YHR183W	9:133693	4.76E-07
YHR183W	13:77684	1.84E-22
YHR183W	15:143597	2.07E-16
YNL279W	8:111683	9.79E-30
YNL279W	13:91085	1.10E-15
YBR255C-A	4:155454	1.56E-07
YBL052C	8:95469	1.75E-11
YBR063C	2:368060	6.82E-10
YPL266W	15:174364	2.09E-10
YHR068W	15:174364	8.44E-19
YMR155W	2:555596	1.68E-09
YHR015W	8:137221	2.14E-34
YBR179C	2:565216	1.69E-08
YGL053W	7:403626	#######
YLR188W	2:537314	6.87E-09
YLR188W	16:387239	1.55E-13
YGL116W	8:111680	1.92E-07
YPR009W	16:555416	2.15E-15
YDR462W	2:551299	4.30E-09
YPL230W	15:174364	1.02E-21

YLR153C	12:662627	1.07E-26
YLR203C	15:174364	2.36E-12
YGR156W	13:57145	3.37E-08
YGR156W	15:154177	2.20E-10
YNL326C	8:111682	7.00E-09
YDR321W	4:1109729	8.34E-35
YDR321W	15:154177	2.37E-16
YBL064C	15:298710	1.04E-07
YPL119C-A	15:174364	1.48E-07
YEL035C	12:662627	4.94E-09
YHR187W	15:174364	2.04E-09
YDL045W-A	2:551299	1.90E-09
YDL045W-A	16:511406	5.79E-07
YDL202W	2:551299	3.35E-08
YJR093C	10:604478	1.04E-14
YMR038C	12:668249	1.91E-08
YMR038C	15:154309	1.04E-13
YOR233W	15:174364	1.48E-11
YPL171C	2:562415	6.31E-08
YPL171C	15:143597	2.19E-14
YDL169C	2:555596	1.16E-08
YMR255W	2:551299	2.40E-11
YLR209C	2:565216	1.79E-08
YJL020C	10:393255	4.36E-12
YDL226C	4:46316	2.79E-23
YFL025C	6:80521	2.66E-09
YOR283W	15:846344	1.71E-30
YJL132W	10:159479	1.46E-18
YGL223C	7:73452	5.73E-07
YGL223C	8:95469	7.59E-09
YGR138C	16:511406	2.74E-08
YPR115W	8:111686	6.02E-11
YJL060W	10:330165	5.05E-11
YFL027C	3:201166	6.28E-37
YJL052W	1:41483	1.55E-08
YJL052W	5:222998	9.48E-11
YJL052W	10:374040	5.03E-14
YJL052W	15:170945	4.51E-15
YFR013W	6:174552	1.41E-08
YKL046C	13:46084	3.14E-07
YKL046C	15:174364	1.38E-08
YBR068C	3:105042	7.57E-12
YHR136C	13:27644	6.08E-48
YLR325C	2:499889	1.49E-11
YLR325C	4:95527	3.54E-07
YLR325C	5:194873	7.09E-09
YLR325C	15:170945	2.62E-10

YHL008C	8:93002	5.48E-93
YHL008C	11:599170	1.69E-15
YHL008C	16:438365	1.21E-08
YML002W	13:273244	1.83E-42
YJL117W	13:27644	1.06E-27
YMR027W	14:449639	3.29E-20
YNL286W	14:96321	3.14E-14
YGL077C	12:683457	2.87E-09
YGL077C	15:174364	5.40E-08
YMR056C	15:143597	1.17E-06
YBR026C	2:301671	7.97E-69
YPR075C	2:551299	7.40E-07
YKL109W	15:174364	3.75E-13
YHR033W	8:167504	1.14E-54
YHR033W	15:180961	1.44E-11
YOR177C	15:690367	1.38E-11
YOR334W	12:662627	5.47E-11
YDR500C	2:506661	1.12E-12
YDR500C	5:193876	3.27E-08
YDR500C	15:143597	2.02E-11
YDR500C	16:500342	3.24E-09
YLR301W	15:141627	1.13E-06
YDL051W	15:174364	2.38E-08
YPL079W	2:499012	7.00E-17
YPL079W	5:200848	5.05E-11
YPL079W	15:143597	4.32E-09
YPL079W	16:500342	3.63E-11
YMR198W	8:111683	3.62E-07
YMR198W	13:46084	2.11E-08
YEL022W	5:116554	2.49E-07
YPL233W	16:105278	5.67E-09
YPL028W	12:662627	3.05E-15
YDL208W	15:143597	1.13E-08
YDR047W	15:89217	5.27E-12
YOL135C	12:662627	6.28E-08
YLR438W	12:1023795	6.42E-13
YLR438W	13:49894	6.35E-47
YHR007C	2:481439	1.19E-07
YHR007C	8:111686	7.56E-11
YHR007C	13:46084	6.39E-09
YHR007C	15:170945	3.41E-10
YCR004C	15:174364	8.86E-12
YMR065W	8:111683	3.63E-36
YMR065W	13:49894	5.74E-13
YDL003W	8:111682	6.53E-13
YIL014C-A	9:325320	#######
YIL014C-A	12:956534	5.72E-07

YDR161W	15:174364	1.66E-08
YLR082C	12:264911	1.47E-10
YCR051W	15:174364	1.67E-08
YDR260C	2:551299	5.02E-11
YDR260C	15:141633	2.00E-08
YDR260C	16:500348	1.39E-08
YGR155W	15:174364	8.13E-10
YER060W-A	13:79786	1.27E-07
YOR356W	12:662627	4.38E-08
YLR061W	2:519049	1.28E-17
YLR061W	5:194873	3.53E-12
YLR061W	12:260113	6.84E-12
YLR061W	15:154309	2.78E-11
YLR061W	16:500342	9.76E-11
YNL146W	3:201166	2.48E-53
YGL262W	4:46466	5.62E-07
YGL262W	7:10158	2.08E-26
YKL050C	2:521415	5.87E-16
YOR222W	15:945781	3.73E-11
YNL230C	8:167506	2.51E-08
YGL226W	7:73452	1.31E-21
YDL073W	13:28622	1.42E-08
YDR151C	7:375499	1.64E-07
YDR342C	15:174364	3.19E-15
YOR296W	2:548401	3.64E-13
YBR107C	2:477206	7.09E-67
YMR001C	2:551299	1.67E-13
YHR141C	5:193876	1.56E-09
YHR141C	15:170945	3.42E-10
YLL007C	1:55215	2.66E-11
YLL007C	4:1507292	4.35E-09
YLL007C	12:131338	3.35E-92
YNL183C	2:551299	6.79E-16
YGR169C	2:555575	4.15E-07
YEL024W	12:659357	2.39E-24
YEL024W	15:113251	1.71E-09
YEL024W	16:500342	3.91E-08
YCR096C	3:201166	3.91E-38
YJR132W	15:150651	1.40E-10
YLR293C	5:196196	5.73E-08
YLR293C	15:150651	2.32E-14
YLR389C	12:927421	6.36E-33
YMR186W	7:375499	1.06E-32
YPL109C	15:150651	4.28E-09
YOL034W	15:170945	5.27E-08
YPR161C	15:154177	2.88E-10
YJR092W	2:551299	8.07E-18

YJL098W	13:49894	5.22E-08
YPR088C	12:697260	1.14E-07
YBR236C	2:697894	2.07E-29
YGR220C	2:551299	1.34E-07
YGR220C	15:141627	9.54E-08
YLR059C	2:555787	1.22E-06
YLR059C	12:260119	6.03E-15
YHR009C	8:137227	1.94E-07
YHR009C	15:174364	2.13E-07
YLL001W	15:154177	1.13E-07
YDL022W	15:174364	1.57E-08
YNL207W	14:245313	5.80E-14
YIL124W	2:562415	1.37E-06
YOR162C	15:632894	3.82E-36
YLR142W	2:555575	1.96E-10
YLR142W	13:49894	6.46E-09
YLR142W	16:500342	3.04E-10
YDL183C	15:174364	2.31E-11
YDL019C	15:144659	4.26E-13
YMR308C	15:150651	2.08E-07
YIL153W	9:98955	1.38E-06
YDR194C	2:555575	5.08E-11
YDR194C	16:500348	2.08E-09
YDR264C	4:975086	4.76E-35
YDR494W	2:555575	6.11E-07
YDR494W	15:174364	1.61E-07
YIL146C	2:555575	3.63E-19
YIL146C	9:74540	4.86E-08
YNL003C	12:662627	2.07E-10
YLR413W	8:111690	6.66E-07
YOR163W	13:27644	5.79E-22
YOR005C	15:154309	6.08E-07
YNL117W	2:551299	1.27E-15
YOR243C	15:170945	3.19E-10
YPR124W	2:555575	3.51E-08
YMR300C	13:99720	4.83E-12
YER156C	15:170945	5.96E-10
YLR288C	12:708594	3.06E-15
YDL206W	15:174364	2.98E-09
YNR068C	2:551299	3.08E-12
YBR006W	2:246129	2.90E-22
YLR361C-A	12:851826	6.34E-29
YLL022C	8:156412	8.48E-09
YCL010C	8:167506	2.64E-09
YLR197W	5:196196	3.96E-08
YLR197W	15:174364	3.74E-10
YBL055C	2:133749	2.05E-09

YMR101C	2:562415	7.87E-10
YLR093C	12:327131	2.65E-37
YAL065C	5.234722222	4.49E-34
YIL103W	9:190866	9.36E-14
YOL119C	15:106266	1.08E-10
YBR221W-A	14:412269	4.19E-08
YJL210W	15:143597	1.36E-11
YDL122W	2:555575	1.24E-07
YDL006W	4:463264	8.40E-10
YNL194C	13:69114	5.79E-09
YNL194C	15:174364	8.48E-36
YML071C	15:180961	9.23E-09
YJL035C	10:380085	5.00E-33
YML031W	2:551299	1.83E-08
YKL041W	11:354466	3.95E-24
YPL267W	8:111679	5.83E-10
YMR272C	15:150651	1.71E-16
YGR006W	8:175255	1.32E-07
YKL026C	15:170945	1.17E-21
YHR142W	2:562415	9.71E-12
YHL009W-A	8:95469	1.07E-15
YHL009W-A	16:442503	1.13E-16
YER043C	2:567221	2.99E-07
YER043C	13:57145	3.37E-08
YER043C	15:143597	1.08E-16
YPL022W	16:511406	8.00E-13
YIL088C	2:555596	1.41E-08
YAL049C	1:52943	4.06E-50
YER047C	5:243299	1.63E-16
YIL099W	9:244902	2.70E-09
YIL099W	15:174364	2.12E-20
YLR084C	2:537314	2.07E-08
YLR084C	15:141627	1.88E-10
YPL098C	2:551299	2.94E-14
YPL098C	8:167506	6.73E-09
YPL098C	16:500348	1.08E-11
YMR090W	15:174364	1.89E-22
YIL077C	15:143597	3.23E-14
YMR232W	8:111683	4.90E-33
YMR002W	2:582419	3.15E-07
YMR136W	15:143597	1.45E-12
YIL111W	9:155027	1.16E-29
YIL111W	13:99675	8.48E-14
YBR234C	2:697894	2.26E-30
YNL239W	2:555575	5.48E-10
YMR041C	15:143597	9.49E-12
YIL136W	5:196352	3.32E-08

YIL136W	9:101011	4.72E-07
YIL136W	13:28622	5.05E-09
YIL136W	15:174364	1.46E-34
YDR226W	2:562415	1.64E-07
YLR333C	2:499889	1.32E-07
YLR333C	5:196196	1.42E-10
YLR333C	15:170945	2.93E-14
YLR333C	16:500342	2.18E-08
YPL271W	15:108577	2.44E-09
YPL271W	16:500342	9.40E-12
YMR030W-A	2:551299	4.08E-11
YBR166C	2:567221	3.69E-92
YBR166C	4:95437	2.02E-08
YHR047C	3:90610	6.87E-09
YHR047C	13:46084	1.71E-19
YHR047C	15:141633	1.28E-19
YNL034W	14:577299	1.38E-25
YGL140C	16:497425	3.05E-07
YLR412W	12:956534	5.03E-10
YNL010W	12:672779	3.79E-09
YNL064C	7:375499	7.71E-18
YNL064C	8:176670	6.41E-09
YGL202W	2:555575	1.87E-09
YFR053C	15:174364	3.79E-10
YHR045W	15:170945	3.96E-09
YOR317W	13:28694	2.22E-07
YPL268W	13:27644	5.12E-13
YPL087W	16:387239	7.18E-15
YCL073C	11:652304	4.03E-58
YGL146C	15:174364	1.95E-10
YLR156W	12:472165	3.03E-99
YOR254C	15:143597	7.89E-09
YJL190C	4:95527	4.44E-08
YJL190C	5:194873	7.30E-11
YJL190C	9:74540	1.50E-07
YJL190C	15:170945	3.25E-12
YER110C	15:174364	3.54E-15
YOL012C	8:152932	7.65E-09
YOR136W	15:594024	1.21E-07
YHR042W	8:185882	4.41E-08
YBR189W	2:519049	4.60E-20
YBR189W	4:95527	4.04E-08
YBR189W	5:196196	3.82E-11
YBR189W	9:74540	8.63E-08
YBR189W	15:170945	5.56E-13
YPL160W	15:170945	4.34E-16
YHR150W	8:399409	1.10E-11

YHR100C	15:174364	1.27E-09
YGR038W	7:553877	3.13E-44
YMR070W	12:672779	3.34E-10
YCR033W	2:516889	2.65E-09
YBR001C	15:174364	2.73E-08
YNL302C	5:196196	5.74E-10
YNL302C	15:170945	7.72E-10
YIL056W	9:251495	1.33E-08
YHR190W	12:662627	7.78E-22
YOR358W	15:150651	4.86E-08
YPL163C	2:537314	1.78E-15
YPL163C	8:111682	2.81E-09
YOR149C	15:589013	9.02E-12
YDR510W	4:1455509	5.80E-13
YPL273W	13:922256	1.15E-16
YKL157W	14:449639	2.20E-26
YHR021W-A	8:149618	1.85E-17
YMR289W	13:849969	2.12E-33
YHL011C	15:174364	1.69E-11
YLR264W	5:193876	9.06E-11
YNL046W	2:555575	3.71E-13
YNL145W	3:201166	#######
YLR409C	15:174364	2.65E-07
YML126C	12:659357	8.17E-33
YML126C	15:143597	3.23E-07
YHR061C	8:111679	1.40E-11
YJR077C	15:136327	9.54E-09
YGL208W	7:110813	7.47E-16
YOR131C	15:571103	8.85E-27
YGL191W	12:668249	7.24E-09
YMR206W	15:174364	9.33E-11
YBR070C	2:388862	6.93E-12
YBR070C	8:111683	5.03E-15
YGR180C	12:450046	2.36E-14
YAL061W	15:150651	3.35E-11
YLR097C	2:555787	2.67E-08
YJR036C	2:533262	3.22E-10
YJR036C	15:143597	7.78E-11
YLR426W	12:987750	1.31E-27
YKL218C	2:551299	1.97E-11
YMR034C	13:328865	2.52E-23
YOR335C	15:170945	1.33E-08
YPR030W	15:170945	1.69E-09
YIL050W	3:90610	9.10E-14
YIL050W	13:49903	5.78E-13
YDR132C	15:136327	6.91E-08
YDR216W	15:143597	7.27E-33

YLR281C	12:705190	4.24E-12
YBR069C	2:376872	1.63E-13
YPR132W	2:555575	2.15E-09
YPR132W	4:100720	2.56E-12
YPR132W	5:194873	8.02E-08
YPR132W	9:74540	2.59E-07
YPR132W	15:170945	2.34E-12
YPR132W	16:500342	6.08E-09
YKL151C	15:174364	1.34E-22
YDR204W	15:174364	2.98E-10
YDR461W	3:201166	8.93E-62
YHR162W	13:99675	2.57E-10
YDR122W	4:733875	2.14E-16
YPL053C	15:174364	1.61E-08
YPL222W	15:174364	3.67E-15
YER039C	8:150330	7.08E-07
YDR284C	12:662627	1.12E-12
YPR037C	8:111679	2.53E-07
YFL016C	7:375499	2.52E-11
YGR250C	7:995892	5.47E-18
YDR077W	2:555787	3.77E-15
YPL055C	8:167506	5.03E-07
YDR399W	8:176412	7.63E-07
YJL204C	10:59959	1.21E-08
YER151C	2:551299	1.21E-07
YNL096C	2:499012	1.99E-10
YNL096C	5:200734	4.88E-11
YKL090W	11:266017	2.89E-32
YKR030W	8:152932	3.62E-07
YOL101C	1:42591	1.32E-10
YOR348C	12:668249	4.02E-12
YOR348C	15:150651	6.25E-07
YOR348C	16:500342	2.17E-08
YIL158W	2:555778	1.75E-09
YIL158W	15:144659	9.03E-07
YPL247C	15:174364	7.16E-23
YDR256C	15:144659	2.27E-13
YFR026C	6:205881	1.98E-24
YCL001W-B	2:551299	5.17E-10
YDR124W	8:111682	1.77E-14
YDR124W	13:49894	1.08E-08
YKR006C	2:551299	1.05E-09
YAL063C-A	1:10152	1.72E-08
YCL042W	13:46084	1.12E-09
YPR114W	2:517123	8.70E-12
YLR100W	12:662627	3.26E-14
YGL017W	7:457215	2.03E-19

YLR143W	15:174364	1.27E-08
YLR289W	2:551299	1.70E-08
YKL181W	2:555575	2.64E-10
YKL006W	2:499889	7.65E-17
YKL006W	5:196196	7.39E-12
YKL006W	9:74540	1.25E-10
YKL006W	15:170945	4.46E-13
YLL066W-B	4:1511257	2.64E-11
YBL029C-A	15:170945	2.53E-09
YMR211W	15:174364	3.33E-08
YMR323W	12:1042072	5.10E-09
YMR323W	13:922268	2.94E-09
YMR323W	15:690367	7.07E-10
YNL042W	14:549682	2.67E-10
YLR372W	5:196190	2.03E-07
YLR372W	8:111690	1.34E-06
YLR372W	15:170945	6.18E-11
YER074W	2:499012	2.34E-16
YER074W	4:95437	1.84E-07
YER074W	5:196196	3.37E-13
YER074W	9:74540	6.50E-07
YER074W	15:154309	9.13E-15
YER074W	16:500342	7.78E-11
YFR031C-A	2:499012	1.20E-13
YFR031C-A	5:196196	6.92E-12
YFR031C-A	15:170945	6.52E-16
YOL113W	15:106272	6.91E-08
YFL010C	6:134096	3.03E-11
YLL055W	12:26184	5.61E-70
YLL055W	13:27644	9.90E-15
YLL055W	15:136327	2.81E-15
YMR267W	15:170945	1.24E-08
YBL022C	2:551299	2.71E-08
YBL022C	7:375499	1.11E-08
YOR188W	2:537314	6.94E-14
YOR188W	15:141627	3.12E-07
YNL218W	13:28694	5.15E-08
YNL218W	15:174364	5.91E-07
YMR181C	2:507282	2.20E-09
YMR181C	13:28694	3.97E-10
YMR181C	15:174364	2.01E-16
YDR362C	2:573491	6.64E-07
YPR196W	16:932538	5.23E-13
YNL053W	15:143597	1.09E-08
YBR280C	15:174364	1.42E-09
YNL241C	3:92247	2.31E-10
YBL043W	2:142262	2.45E-09

YBL043W	13:99675	5.72E-11
YNL323W	2:551299	1.48E-08
YNL323W	13:245625	8.42E-10
YNL323W	14:33643	8.15E-12
YNL007C	7:375499	1.41E-18
YHL033C	2:519049	1.30E-11
YHL033C	5:193876	1.61E-12
YHL033C	15:170945	2.63E-16
YGL209W	2:537314	2.03E-07
YGL209W	15:154309	2.11E-07
YML055W	13:163328	2.61E-15
YDL131W	2:477206	1.00E-12
YDL131W	4:217399	4.21E-22
YKL210W	11:46633	1.75E-09
YCL014W	2:551299	2.32E-10
YCL014W	8:149618	2.89E-08
YHL048W	6.176388889	2.06E-89
YML111W	13:49894	4.11E-18
YMR093W	15:174364	6.24E-09
YKL189W	8:111683	3.24E-22
YKL189W	13:64970	2.70E-07
YBR231C	2:667125	3.29E-22
YDL082W	2:499012	7.67E-15
YDL082W	5:196196	5.66E-12
YDL082W	15:170945	2.95E-11
YJL151C	10:138712	4.89E-26
YNL102W	8:111690	1.07E-10
YDL021W	15:170945	1.42E-08
YGL093W	7:311205	6.98E-13
YDR074W	15:174364	1.75E-16
YDL037C	1:23780	1.12E-08
YDL037C	4:369365	1.25E-17
YDR171W	15:174364	1.39E-13
YOL163W	3.133333333	3.32E-77
YDL154W	2:551299	1.52E-11
YBR120C	2:551299	1.60E-07
YMR011W	2:555575	3.26E-10
YMR011W	5:350744	6.81E-18
YMR011W	9:74540	9.06E-07
YMR011W	15:141627	1.44E-12
YMR011W	16:500348	1.22E-09
YGR131W	12:659357	3.08E-09
YDR322W	2:551299	1.34E-07
YKL093W	11:269734	1.59E-13
YKL093W	15:174364	4.65E-15
YDR443C	4:1344670	6.85E-17
YLR332W	15:174364	2.55E-07

YDR019C	13:49894	2.33E-11
YNL317W	14:33643	2.46E-12
YPR133W-A	2:555778	2.67E-17
YPR133W-A	8:167506	2.34E-08
YPR133W-A	16:500348	1.13E-11
YDL164C	8:111683	1.85E-06
YDR350C	8:156412	1.63E-07
YKL142W	15:170945	3.00E-08
YML106W	2:537314	1.57E-10
YML106W	15:170945	3.96E-10
YBL071C-B	2:519049	3.94E-07
YAL040C	15:174364	6.17E-11
YJR005W	2:537314	8.23E-08
YJR005W	15:143597	2.84E-08
YJR041C	15:174364	7.70E-11
YPL038W-A	16:489143	1.03E-18
YER075C	2:562415	2.01E-12
YOR374W	13:99675	1.73E-15
YGL028C	2:562415	1.57E-82
YGR154C	2:551299	3.19E-08
YGR154C	16:500348	4.25E-11
YOL095C	15:141627	1.16E-16
YNL247W	15:170945	1.74E-11
YPR199C	11:656099	5.01E-33
YKR058W	15:174364	1.08E-17
YKR095W-A	3:91287	6.59E-08
YKR004C	11:421190	2.31E-14
YML068W	2:551299	2.68E-09
YML068W	8:167506	6.27E-08
YML068W	16:500348	6.18E-07
YJL217W	10:23505	#######
YFL053W	6:30378	5.98E-38
YER112W	8:175255	2.26E-07
YFL042C	15:154309	2.41E-09
YOL016C	13:27644	2.31E-12
YOL103W	15:132423	3.40E-09
YOR276W	15:174364	2.27E-11
YHL047C	5.217361111	3.57E-81
YGR236C	7:948578	2.19E-16
YGR236C	15:150651	1.04E-13
YPR018W	8:111690	1.55E-06
YPR018W	15:144659	1.48E-07
YPL040C	2:551299	4.03E-10
YPL040C	15:141627	1.60E-10
YPL040C	16:500348	5.91E-10
YFL021W	2:555575	3.25E-33
YFL021W	6:101519	8.73E-13

YKL028W	11:394660	1.04E-08
YLR432W	2:499012	9.05E-09
YLR432W	5:196196	7.42E-09
YLR432W	15:154309	2.96E-11
YGL115W	15:143597	1.72E-08
YDL002C	4:446125	1.20E-25
YHR094C	4:201395	1.34E-10
YOR226C	3:90676	3.08E-28
YOL029C	15:174364	3.99E-16
YMR052W	13:255486	6.87E-09
YHR027C	8:167504	4.92E-18
YER056C-A	2:499889	9.52E-09
YER056C-A	5:193876	1.65E-08
YER056C-A	15:170945	1.36E-11
YLR419W	2:506661	1.10E-08
YLR449W	15:174364	3.49E-19
YLR361C	12:851826	1.05E-08
YOR140W	2:548401	2.34E-12
YKL107W	2:517365	8.40E-11
YKL107W	4:100720	3.27E-07
YLR216C	7:375499	7.16E-15
YAL037W	2:555575	3.53E-10
YAL037W	8:111690	2.34E-07
YDL039C	1:23813	2.39E-08
YDL039C	4:369365	2.41E-26
YDL039C	5:321618	3.13E-14
YDL039C	10:28306	3.89E-13
YLL062C	8:167504	2.15E-15
YLR377C	12:872448	4.96E-14
YDR011W	12:672779	2.91E-07
YDR011W	15:632894	9.45E-17
YPR119W	2:562415	9.13E-08
YDR177W	5:321618	2.81E-08
YDR177W	8:176670	1.63E-07
YDR177W	12:292523	5.83E-10
YGL205W	1:42489	3.65E-10
YGL205W	7:110807	3.04E-13
YBR261C	15:174364	3.84E-11
YGR189C	13:46084	5.70E-11
YGR189C	15:170945	5.25E-14
YPL104W	2:537314	3.67E-08
YPL104W	15:174364	1.39E-10
YOR048C	15:174364	5.69E-11
YHR106W	15:174364	4.41E-08
YKL029C	3:91305	9.03E-09
YKL029C	11:382553	1.13E-21
YPR007C	2:569420	3.72E-07

YPR007C	16:547618	4.99E-23
YPR172W	15:170945	1.97E-09
YHR080C	15:144659	2.82E-17
YBR029C	15:143597	3.21E-07
YCL026C-B	3:75021	4.57E-38
YCL026C-B	4:733875	2.19E-22
YML058W-A	12:469156	7.81E-10
YJR102C	10:627628	1.50E-08
YDL038C	1:23813	6.68E-09
YDL038C	4:369365	1.66E-21
YDL038C	10:28306	3.53E-12
YMR230W	5:194873	5.21E-08
YMR230W	15:170945	7.78E-08
YMR124W	13:513778	7.77E-28
YNL246W	14:191243	8.68E-33
YMR296C	13:57145	6.05E-09
YMR296C	15:150651	4.76E-07
YDL014W	2:551299	6.58E-09
YDL014W	15:170945	8.00E-08
YAR042W	1:201039	2.64E-40
YPR008W	15:174364	4.59E-10
YDR023W	15:174364	2.56E-11
YIL048W	9:242417	1.67E-12
YHR084W	8:111683	2.90E-28
YHR084W	13:91085	1.58E-08
YLR146C	15:174364	3.38E-09
YMR100W	12:677957	8.21E-10
YGL147C	5:196196	2.80E-08
YGL147C	9:74540	2.19E-10
YGL147C	14:731633	7.81E-10
YGL147C	15:174364	2.64E-11
YOR286W	2:551299	5.14E-10
YOR286W	8:167506	2.95E-07
YOR286W	15:141627	4.21E-08
YOR286W	16:500348	1.78E-11
YNL115C	15:174364	1.04E-07
YGL064C	2:530481	4.59E-11
YOL056W	15:170945	7.19E-11
YOR311C	15:170945	1.86E-08
YNL302C	2:499012	2.28E-13
YNL302C	5:193876	6.21E-11
YNL302C	15:170945	3.28E-10
YGR289C	7:1075580	1.77E-57
YIR009W	2:551299	2.93E-08
YGL037C	15:116709	2.13E-09
YBR013C	2:256896	3.00E-40
YNL058C	14:525061	2.47E-11

YHR105W	8:167506	3.94E-07
YOR065W	5:500783	7.04E-10
YOR065W	12:662627	1.33E-24
YOR065W	13:130069	1.56E-07
YOR065W	16:500342	3.56E-10
YOR034C-A	2:555596	1.34E-06
YPL236C	15:150651	1.45E-07
YLR290C	12:705226	1.05E-10
YGL032C	3:201166	9.07E-84
YER001W	2:551299	2.32E-08
YER001W	15:141627	2.77E-08
YNL255C	5:196196	1.49E-08
YNL255C	15:174364	1.21E-11
YPL052W	13:46070	1.92E-07
YPL052W	15:174364	3.37E-14
YKL101W	8:111683	1.80E-12
YBR274W	2:746476	6.04E-24
YLL041C	15:113251	3.64E-08
YGR147C	2:562409	1.30E-06
YGR237C	15:174364	1.18E-11
YJR133W	2:537314	5.44E-09
YHR052W	8:209167	1.18E-09
YOR212W	8:111683	1.17E-21
YOR212W	13:49894	7.10E-09
YLR375W	12:852066	1.49E-06
YLR375W	15:136327	3.26E-08
YLR375W	16:511400	4.03E-07
YFR040W	2:555575	3.09E-07
YKL155C	15:174364	7.44E-07
YIL148W	9:79793	4.05E-10
YDR513W	4:1468373	4.58E-20
YDR513W	15:170945	7.29E-08
YGL060W	8:111683	7.46E-26
YGL060W	13:91085	6.17E-12
YHR179W	5:196196	1.43E-07
YHR179W	12:662627	1.15E-13
YPR026W	15:174364	2.26E-18
YJL006C	10:451832	2.52E-12
YPL046C	16:462646	8.73E-15
YNL245C	14:191243	1.42E-21
YGR143W	15:174364	8.46E-09
YOL159C	9:19607	2.78E-12
YOL159C	13:46084	1.30E-09
YOL164W	15:10427	#######
YLL010C	12:126934	3.90E-44
YLL046C	15:141627	3.31E-08
YCR106W	6.628472222	7.25E-09

YCR106W	0.868055556	9.27E-09
YCR106W	12:1054278	4.52E-09
YML073C	2:519049	1.23E-12
YML073C	5:196196	1.68E-11
YML073C	15:170945	2.59E-12
YML073C	16:500342	5.05E-09
YNL274C	15:174364	4.26E-12
YDR294C	2:555575	3.31E-07
YPR120C	8:111690	1.19E-07
YOL014W	15:298710	4.03E-78
YLR228C	15:174364	1.53E-07
YER155C	8:111682	3.30E-13
YIL131C	2:551299	3.75E-15
YIL131C	16:500348	1.66E-08
YNL173C	12:634226	3.88E-07
YGL103W	1:187544	3.75E-09
YGL103W	9:101011	1.47E-08
YGL103W	15:170945	8.88E-12
YGR109W-B	7:708345	1.58E-41
YGR109W-B	9:200332	7.82E-23
YKL052C	15:143597	9.29E-17
YDL126C	14:449639	1.13E-21
YJR080C	2:551299	6.54E-10
YJR080C	15:174364	2.32E-07
YAL056W	1:41483	2.30E-73
YER188C-A	5:568716	2.53E-19
YNL315C	2:555575	2.66E-09
YNL316C	14:33643	1.04E-24
YGR121C	2:551299	7.87E-09
YPR138C	2:555575	8.33E-17
YOR109W	15:524972	2.49E-12
YFR012W	6:168342	2.00E-10
YBR170C	2:579459	7.72E-08
YHR213W	1:23780	1.11E-07
YOL089C	15:154309	5.87E-36
YOL159C-A	9:19607	7.36E-08
YOL159C-A	3.133333333	4.33E-09
YBR052C	15:174364	3.98E-08
YKR097W	2:569420	4.71E-15
YKR097W	11:632952	1.66E-22
YJR030C	10:472146	2.52E-60
YMR312W	15:174364	1.13E-08
YJL010C	15:174364	6.45E-10
YGR109C	8:111690	2.48E-10
YLR176C	15:154309	2.15E-09
YIL071C	2:555575	4.10E-08
YIL119C	9:139462	3.44E-18

YNL134C	15:150651	3.44E-26
YBR148W	2:548401	3.61E-33
YBR148W	15:174364	1.83E-12
YGR127W	15:174364	2.74E-13
YBL087C	2:519049	5.81E-12
YBL087C	4:95527	2.67E-07
YBL087C	5:196196	3.41E-10
YBL087C	9:74540	6.33E-09
YBL087C	15:174364	2.32E-16
YHR028C	8:167504	6.19E-33
YGR174C	2:537314	1.03E-07
YGR174C	15:154309	7.22E-09
YGL258W-A	2:562415	1.05E-17
YOL121C	2:489202	3.13E-12
YOL121C	5:193876	8.88E-12
YOL121C	15:154309	1.89E-08
YFR052W	8:176670	1.28E-08
YOR230W	2:565216	8.59E-14
YGL123W	2:521415	7.26E-13
YGL123W	4:95527	4.66E-11
YGL123W	5:196196	3.74E-09
YGL123W	9:74540	4.25E-09
YGL123W	15:170945	3.36E-11
YGL123W	16:500342	8.73E-10
YOR160W	15:632894	1.23E-27
YOL147C	15:54769	1.56E-08
YIL051C	13:49894	9.80E-50
YCR009C	8:111690	5.67E-09
YAR071W	1:141181	2.22E-12
YAR071W	7.024305556	8.37E-07
YAR071W	13:27644	1.96E-67
YLR157W-C	3:175808	1.76E-10
YGR136W	2:551299	2.00E-07
YIL152W	9:74540	2.06E-26
YGL075C	16:500348	2.12E-09
YOR027W	7:375499	5.59E-24
YBR085W	12:634227	3.23E-09
YIL130W	9:101011	1.38E-10
YHR025W	15:170945	5.35E-12
YML119W	13:27644	3.58E-09
YHR153C	8:389050	2.50E-09
YDR529C	12:634226	1.48E-08
YDR529C	13:28334	2.82E-07
YDL090C	13:49894	2.10E-09
YIL065C	9:200332	1.93E-09
YNL137C	2:551299	6.01E-08
YNL137C	15:141627	7.11E-08

YNL137C	16:500348	2.07E-08
YNL202W	14:258590	1.15E-13
YJR148W	2:533268	1.47E-10
YJR148W	3:100213	9.79E-13
YAL060W	5.280555556	5.87E-21
YDR410C	8:111690	2.39E-11
YGL181W	7:143756	1.60E-35
YOL102C	15:132423	3.49E-15
YPR041W	2:551299	5.21E-09
YOR023C	15:407684	3.26E-08
YIL082W-A	7:708034	3.73E-42
YIL082W-A	9:200332	7.09E-24
YBR156C	2:555575	7.43E-18
YBR156C	8:111680	5.37E-14
YER118C	2:537314	8.59E-12
YPL110C	13:27644	1.95E-15
YGL009C	3:81832	8.75E-42
YGL009C	12:644136	7.13E-10
YKR093W	3:90610	1.99E-10
YKR093W	12:22602	2.09E-08
YKR093W	13:46084	7.58E-32
YKR093W	15:141633	3.52E-39
YOR312C	5:196196	1.25E-09
YOR312C	15:170945	7.85E-12
YDL156W	2:517123	4.90E-07
YOR092W	15:491178	6.69E-08
YML047C	8:111683	9.64E-46
YML047C	13:46084	2.16E-09
YGL184C	8:167504	2.22E-15
YNR069C	2:551299	2.98E-13
YLL042C	12:63866	1.88E-15
YHR013C	13:49894	5.14E-08
YOR186W	15:143597	4.91E-10
YHR088W	15:174364	8.58E-10
YDR385W	2:517365	1.15E-08
YGL045W	7:403626	2.05E-24
YER029C	5:218250	2.76E-26
YEL072W	2:551299	9.24E-10
YEL072W	16:500342	5.96E-08
YHR029C	8:167504	3.61E-08
YGL139W	2:551299	7.70E-12
YMR174C	2:537314	7.96E-08
YMR174C	15:170945	5.45E-18
YAR068W	8:549634	1.06E-22
YAR068W	15:524956	2.73E-08
YCR107W	6.628472222	2.53E-07
YNL069C	2:519049	1.93E-14

YNL069C	5:196196	4.02E-11
YNL069C	7:141955	7.36E-12
YNL069C	9:74540	2.78E-07
YNL069C	15:170945	6.40E-13
YJR122W	15:141627	2.90E-09
YJR122W	16:500348	6.23E-08
YIL031W	2:516889	2.34E-08
YOL164W-A	3.133333333	2.16E-45
YLR219W	15:174364	4.41E-17
YPR158W	7:375499	3.12E-20
YCR034W	15:143597	2.93E-12
YNL227C	15:174364	2.83E-08
YOR111W	2:551299	1.16E-09
YBR256C	2:551299	3.93E-09
YJL034W	7:375499	1.14E-11
YJL034W	15:150651	1.56E-11
YNL278W	2:508843	7.72E-08
YNL278W	8:111683	1.61E-08
YER130C	15:143597	3.21E-10
YOR215C	15:174364	3.42E-09
YOL091W	15:144659	1.58E-41
YOR220W	15:174364	2.75E-08
YGR067C	2:584357	2.07E-07
YKL182W	12:659357	6.55E-09
YGL194C	7:167587	2.74E-12
YFL065C	4:1511257	1.05E-07
YKL145W	14:449639	4.31E-32
YHR010W	2:537314	2.16E-08
YHR010W	4:96271	3.16E-09
YHR010W	5:193876	3.25E-11
YHR010W	15:170945	1.10E-14
YIL155C	15:174364	5.16E-10
YKL128C	11:194611	7.43E-14
YGR243W	13:57145	2.80E-08
YGR243W	15:154309	1.20E-11
YGR077C	15:136327	1.82E-07
YLR155C	12:472165	#######
YBR034C	15:150651	4.89E-09
YBR072W	2:380932	2.35E-33
YBR072W	5:225484	5.17E-08
YBR072W	9:101011	5.19E-10
YBR072W	15:170945	1.60E-14
YGR102C	15:174364	1.85E-12
YJR115W	7:110807	1.59E-09
YLR154C	12:662627	7.86E-14
YLR285C-A	2:555787	5.19E-20
YJL005W	15:174364	1.35E-13

YML004C	13:298187	4.56E-11
YER082C	15:174364	4.49E-17
YHR107C	2:551299	4.58E-13
YHR107C	8:176670	1.41E-11
YFR046C	5:321618	1.54E-07
YFR046C	16:500348	6.98E-11
YML018C	15:170945	2.88E-09
YDR058C	4:569429	3.73E-55
YDR058C	15:132423	5.33E-09
YLR138W	2:551299	9.62E-08
YOR085W	12:705226	1.02E-09
YKL216W	5:117056	2.50E-29
YKL216W	15:174364	2.14E-09
YPR095C	12:659357	3.64E-10
YGR123C	15:174364	2.26E-07
YOL116W	15:136327	1.06E-10
YDR366C	4:1213416	2.53E-36
YBL003C	10:511213	1.19E-08
YGR278W	13:255486	3.95E-09
YMR295C	15:106164	6.43E-07
YPR198W	11:656099	1.10E-41
YLR042C	2:562415	2.48E-37
YOR051C	15:424522	1.13E-12
YJL107C	10:214773	3.05E-22
YDR263C	4:975086	9.24E-37
YDR263C	15:143597	1.37E-07
YIL101C	9:244902	1.36E-12
YIL101C	15:174364	1.30E-17
YMR129W	2:517365	1.85E-07
YMR032W	2:551299	1.12E-16
YNL328C	8:167506	1.11E-08
YNL328C	14:33643	2.48E-10
YAR075W	1:229140	1.78E-32
YPL265W	13:46084	1.88E-13
YPL265W	15:141633	2.01E-09
YGL058W	15:154309	9.76E-15
YKL087C	2:551299	5.91E-11
YKL087C	13:99675	9.89E-08
YKL087C	15:141627	4.42E-09
YKL087C	16:500348	8.66E-14
YDR357C	4:1213416	1.23E-15
YMR072W	15:174364	1.00E-09
YGR174W-A	2:499012	1.41E-09
YGR174W-A	15:174364	1.24E-12
YER067W	15:174364	5.24E-09
YDR332W	15:150651	2.91E-07
YMR173W	2:551299	7.00E-15

YNL035C	14:591234	2.06E-30
YCR105W	2:29483	1.37E-08
YCR105W	12:1056103	3.11E-08
YOR304C-A	15:889464	1.87E-23
YJR107W	10:627628	2.55E-08
YER049W	15:174364	3.37E-13
YJL062W-A	2:551299	8.66E-10
YJL062W-A	15:108577	7.57E-08
YJL062W-A	16:500348	8.24E-09
YJL013C	10:404508	1.03E-16
YBR045C	2:328489	2.96E-49
YDL238C	2:555575	2.32E-20
YER074W	2:489202	6.47E-12
YER074W	5:196196	9.83E-11
YER074W	9:74540	3.17E-08
YER074W	15:143597	1.03E-14
YER074W	16:428900	5.71E-10
YJR043C	2:499012	1.47E-07
YJR043C	15:150651	1.37E-07
YER107C	15:174364	1.84E-11
YLR312C-B	12:760763	9.02E-18
YBL030C	15:136327	1.30E-07
YIL007C	12:662627	1.19E-11
YOR227W	3:91287	5.63E-10
YOR227W	15:170945	3.78E-11
YBL002W	8:111690	7.50E-08
YER145C	2:506661	1.41E-07
YGL248W	7:15891	5.11E-58
YGL248W	8:176670	3.82E-10
YPL014W	13:77684	2.01E-07
YPL014W	15:174364	5.40E-11
YCR086W	2:616262	1.70E-09
YCR086W	3:258303	1.20E-10
YJL170C	3:201166	#######
YJL170C	8:111682	2.11E-07
YJR127C	15:150651	9.26E-11
YCR046C	2:551299	5.71E-15
YCR046C	8:167506	1.33E-07
YCR046C	16:500348	2.85E-10
YDR218C	12:672779	3.69E-12
YPL191C	8:167506	6.91E-07
YIL023C	9:308854	3.98E-27
YPL213W	2:551299	6.04E-13
YNL040W	2:514035	1.74E-10
YNL040W	14:553129	9.37E-18
YKL179C	2:567221	8.05E-07
YGL106W	8:111683	7.55E-12

YHR039C	12:662627	3.89E-17
YGL241W	7:44996	2.19E-11
YPL064C	16:428900	4.18E-10
YDR277C	15:174364	7.17E-10
YGR251W	7:995892	2.01E-15
YNL300W	8:111683	3.38E-20
YJL157C	8:111683	4.33E-27
YJL157C	13:46084	2.74E-11
YMR194C-B	15:174364	2.63E-15
YDL209C	4:80849	3.58E-11
YGR138C	2:567221	2.72E-09
YGR138C	12:659357	2.07E-07
YGR138C	15:136327	3.37E-13
YGR138C	16:500348	1.20E-15
YDR425W	2:551299	5.92E-10
YGR118W	15:113251	1.06E-09
YKL113C	8:111683	5.13E-09
YKL127W	13:46084	6.80E-12
YKL127W	15:170945	7.54E-12
YGL081W	2:562415	2.26E-07
YGL081W	7:375499	2.18E-09
YGL081W	15:174364	3.77E-10
YPR105C	2:551299	2.46E-09
YDR421W	2:551299	1.64E-11
YDR421W	15:150651	4.53E-10
YDR421W	16:500348	6.34E-18
YOR096W	2:499012	4.50E-09
YOR096W	5:200848	5.94E-12
YOR096W	9:74540	1.25E-07
YOR096W	15:170945	7.47E-16
YDR444W	4:1344670	1.74E-26
YLR406C	2:499889	8.14E-16
YLR406C	5:193876	3.53E-14
YLR406C	15:170945	1.27E-11
YOR293W	2:499012	1.61E-07
YOR293W	4:96271	2.45E-07
YOR293W	5:200848	3.18E-11
YOR293W	15:143597	1.46E-10
YJR061W	10:548177	1.57E-23
YNL078W	2:582419	3.62E-15
YNL078W	8:111680	7.39E-07
YHR016C	8:137227	1.39E-11
YHR016C	15:174364	9.21E-12
YGR225W	7:948590	3.23E-09
YER180C-A	5:568698	1.93E-12
YPL157W	16:266023	3.98E-30
YDR091C	15:150651	1.20E-08

YKR076W	15:174364	1.10E-07
YMR060C	16:500348	4.86E-11
YLR086W	12:317542	1.41E-19
YBR054W	13:277071	4.81E-08
YGR021W	15:174364	6.93E-12
YJR084W	10:602943	1.59E-12
YOR380W	2:551299	3.92E-09
YBR284W	15:170945	3.26E-14
YJR015W	10:461201	1.70E-87
YGR142W	7:375499	8.02E-26
YLR046C	12:239578	2.82E-22
YPL252C	16:70853	3.81E-17
YKR053C	11:554200	1.07E-57
YGR200C	7:889602	2.43E-12
YGR200C	15:174364	4.30E-10
YER141W	12:659357	9.79E-09
YHR064C	15:154309	1.80E-12
YJL051W	10:345059	3.53E-30
YGR234W	7:956838	6.23E-45
YGR234W	12:662627	6.50E-21
YNL280C	12:662627	3.15E-13
YLR462W	4:1511257	3.47E-09
YLR462W	12:1067088	1.67E-08
YMR297W	15:174364	9.73E-09
YKL007W	10:548177	7.35E-13
YHR017W	15:174364	4.56E-13
YHL016C	2:480009	7.85E-11
YHL016C	8:71742	5.73E-56
YHL016C	13:96015	1.35E-12
YMR157C	13:572643	2.74E-09
YMR157C	15:180961	8.50E-07
YGL076C	2:499012	3.41E-16
YGL076C	5:196196	1.88E-10
YGL076C	9:74540	5.10E-08
YGL076C	15:174364	1.05E-16
YNL133C	15:141627	9.24E-10
YCL025C	2:565216	8.87E-07
YCL025C	13:87587	1.16E-15
YCL025C	16:500342	8.04E-10
YBR117C	2:477206	2.02E-12
YBR117C	15:174364	2.44E-19
YGL125W	3:91049	6.85E-09
YHR031C	8:167502	1.94E-10
YNL056W	14:502496	7.60E-22
YDR034W-B	2:537314	1.02E-07
YDR034W-B	15:174364	4.33E-23
YHR022C	2:537314	2.18E-10

YHR022C	8:149510	1.04E-07
YHR022C	13:33501	4.24E-09
YHR022C	15:174364	6.72E-09
YHR022C	16:500348	6.44E-09
YJL118W	15:154309	8.47E-11
YDR296W	15:170945	2.01E-07
YLR085C	12:317542	1.67E-10
YOR241W	15:174364	2.68E-16
YBR204C	2:628224	2.52E-18
YGL035C	7:427476	9.88E-14
YLR378C	2:551299	1.25E-09
YPL166W	8:167506	5.66E-07
YKL222C	11:13042	3.19E-17
YHL013C	8:152932	5.56E-07
YMR120C	4:1272737	4.55E-10
YMR117C	13:492453	3.72E-29
YBR038W	2:569420	7.62E-07
YGL183C	7:141949	2.54E-22
YER187W	5:568698	1.73E-44
YOR001W	15:143597	3.71E-10
YOR161C	15:174364	1.23E-22
YMR286W	15:141627	2.53E-07
YER056C	5:272255	4.36E-27
YER056C	15:170945	2.74E-09
YLR327C	15:170945	5.39E-09
YML085C	13:99675	1.23E-22
YML085C	14:412758	6.81E-09
YDL079C	15:174364	2.09E-12
YCR043C	15:170945	2.52E-08
YOR298W	2:551299	1.54E-10
YOR064C	8:167504	3.69E-07
YMR177W	13:77684	1.05E-08
YMR177W	15:174364	7.07E-19
YOR274W	1:41483	3.22E-08
YOR274W	9:254745	1.75E-08
YOR274W	13:77684	2.81E-10
YOR274W	15:174364	1.67E-19
YDR492W	7:403925	3.63E-09
YGL234W	13:99720	9.15E-10
YEL057C	5:44617	8.35E-47
YEL057C	15:170945	3.46E-09
YIL087C	7:708252	3.42E-08
YIL087C	9:195965	3.97E-18
YIL087C	15:174364	2.88E-14
YNR060W	12:683463	4.42E-22
YNR060W	15:180210	6.63E-07
YOR180C	8:111690	1.36E-06

YDL227C	4:46316	1.70E-78
YPR065W	12:662627	8.26E-22
YGR121W-A	7:708285	1.33E-27
YEL004W	2:555778	8.56E-07
YLR452C	8:111683	1.37E-28
YLR452C	13:49894	1.87E-15
YMR200W	2:551299	3.54E-08
YNL104C	3:81832	1.28E-25
YLR466C-B	4:1511257	1.66E-29
YLR466C-B	12:1056097	5.33E-09
YJR114W	10:621762	1.70E-11
YGR244C	15:143597	2.42E-09
YBR264C	8:111680	3.56E-09
YOL007C	2:537314	2.87E-08
YOL007C	8:111683	5.09E-14
YFR032C-A	4:95527	1.65E-07
YFR032C-A	5:193876	1.17E-08
YFR032C-A	15:170945	2.43E-11
YKL054C	8:167504	3.36E-07
YKL054C	16:500348	1.44E-11
YKL043W	11:354466	9.48E-24
YDL166C	15:174364	2.83E-09
YOL049W	12:697260	5.22E-08
YNL289W	8:111683	6.85E-10
YJL209W	10:40238	3.50E-13
YGL097W	2:551299	6.44E-09
YPL061W	2:562415	5.15E-13
YPL061W	13:77684	6.14E-08
YPL061W	15:141627	1.85E-08
YPL061W	16:428900	1.90E-15
YIL008W	13:46070	3.01E-09
YIL008W	15:150651	4.84E-12
YDL211C	15:180961	1.49E-07
YMR009W	12:662627	1.22E-25
YGL010W	7:459354	2.89E-12
YMR163C	2:519049	3.58E-09
YJR034W	2:551299	6.66E-08
YJR034W	15:180961	1.11E-06
YGR047C	8:111683	3.71E-07
YML062C	8:111682	1.50E-07
YCL055W	2:506661	1.08E-07
YCL055W	8:111683	4.28E-56
YCL055W	13:49894	8.08E-17
YPL156C	8:111683	1.46E-13
YPL156C	13:46084	1.37E-11
YPL156C	15:174364	6.89E-09
YPL156C	16:618575	1.27E-07

YOR237W	12:659357	1.91E-18
YLR180W	8:176412	7.29E-09
YLR180W	9:254745	2.44E-08
YLR180W	13:46084	6.75E-14
YNL021W	14:586789	2.97E-19
YOL141W	15:43217	6.90E-30
YBL086C	15:174364	2.23E-11
YGR249W	7:974640	5.14E-12
YLR405W	2:517365	2.30E-07
YNL054W	14:502316	5.90E-14
YGL174W	7:187179	3.84E-39
YMR244W	12:662627	3.62E-14
YNL258C	8:95469	4.68E-10
YNL258C	13:46084	1.45E-07
YIL015W	3:201166	1.49E-72
YHR111W	15:174364	4.06E-11
YGR246C	7:985414	1.51E-09
YKL017C	11:421190	1.50E-29
YIL039W	15:143597	2.87E-07
YOR129C	2:537314	1.26E-15
YDR085C	8:111682	2.31E-07
YER117W	4:95527	6.71E-09
YER117W	5:193876	1.01E-09
YER117W	15:170945	3.12E-13
YPL088W	16:387239	7.68E-50
YBR191W	2:499012	1.42E-14
YBR191W	4:95437	3.43E-08
YBR191W	5:196196	7.26E-14
YBR191W	15:170945	2.38E-13
YBR191W	16:500342	7.86E-11
YNL290W	8:111690	9.99E-09
YNL290W	14:96321	1.25E-11
YMR134W	12:662627	1.46E-38
YCL064C	4:1443910	1.82E-10
YCL064C	7:110813	1.75E-08
YCL064C	13:49903	1.36E-87
YCL064C	14:13845	1.07E-10
YOR063W	5:193876	8.55E-08
YOR063W	15:170945	2.90E-14
YLR326W	12:782839	7.15E-36
YJR063W	15:154309	3.54E-09
YKR079C	15:174364	1.05E-18
YPR022C	16:600664	5.64E-30
YBR139W	2:533262	1.24E-08
YBR139W	8:128732	6.25E-09
YPL089C	2:555575	6.05E-08
YPL089C	15:154177	8.60E-09

YPR074C	2:562415	2.63E-10
YPR074C	13:69122	3.91E-08
YPR036W	15:143597	8.10E-08
YKR024C	8:111679	7.05E-10
YBR162W-A	15:136327	2.89E-07
YBR007C	15:174364	1.87E-12
YLR109W	13:261725	5.85E-10
YAL047C	1:55215	3.26E-09
YDL179W	2:555596	4.49E-09
YMR145C	15:154177	1.46E-07
YOL054W	15:174364	4.88E-15
YJR147W	2:548401	1.53E-16
YML003W	13:255486	9.89E-15
YGR085C	2:499012	1.31E-08
YGR085C	4:95437	5.17E-11
YOR219C	8:111683	3.32E-09
YDR111C	13:96015	9.23E-08
YPL158C	2:555596	5.98E-11
YKL003C	2:551299	6.48E-08
YDR420W	8:167504	9.96E-07
YDR420W	15:143597	3.54E-07
YDL087C	4:289639	4.33E-07
YBR025C	15:174364	7.38E-15
YGL145W	15:170945	1.48E-09
YNR044W	8:111683	7.48E-27
YNR044W	13:91085	9.00E-14
YLR441C	5:200848	7.27E-10
YLR441C	15:170945	1.25E-11
YPL219W	15:174364	3.85E-08
YKL150W	12:659357	2.89E-10
YIL098C	2:551299	3.82E-08
YIL098C	16:500348	1.15E-07
YGR194C	15:174364	3.74E-09
YGR285C	15:174364	1.28E-13
YCR069W	12:634227	2.91E-10
YLR390W	2:551299	3.18E-10
YLR390W	8:167506	8.91E-07
YDL210W	2:555575	1.32E-28
YDL210W	4:95437	3.56E-08
YDL210W	16:500342	7.42E-09
YIL021W	8:167506	3.15E-07
YIL021W	15:154309	1.36E-07
YKR069W	11:566015	1.80E-23
YIL156W	12:693790	3.19E-08
YJR056C	10:537157	2.98E-11
YGL170C	7:187179	2.00E-11
YLL034C	15:174364	9.81E-09

YPL111W	13:49894	1.23E-38
YLR080W	2:562415	6.32E-09
YML110C	13:46084	1.61E-07
YML110C	15:174364	5.71E-11
YHR096C	15:174364	5.09E-20
YHR019C	15:170945	5.28E-09
YOR058C	15:438828	5.03E-09
YNL042W-B	8:111683	1.13E-08
YOR273C	15:842003	6.06E-17
YBR111W-A	2:477206	1.38E-08
YPR033C	15:170945	1.33E-11
YML121W	13:27644	2.02E-14
YNR002C	13:99720	2.21E-21
YNR002C	15:174364	8.33E-22
YER152C	2:555596	1.75E-43
YDR041W	15:174364	2.48E-09
YGL210W	8:167506	5.33E-07
YDR197W	15:174364	2.34E-17
YDR214W	7:375499	2.44E-15
YLR466C-B	4:1511257	1.28E-24
YDR003W	4:450230	4.04E-09
YAR029W	1:184405	1.88E-28
YAR029W	15:175594	3.57E-09
YBR031W	5:194873	1.41E-09
YBR031W	15:170945	2.40E-17
YLL045C	2:519049	3.09E-13
YLL045C	5:196196	4.05E-09
YLL045C	9:74540	2.34E-07
YLL045C	15:170945	6.52E-15
YBL044W	2:143721	3.55E-68
YGR035C	13:27644	3.26E-09
YGR035C	15:174364	5.64E-12
YER025W	2:555575	3.75E-08
YER025W	15:170945	1.48E-10
YOR315W	2:569420	1.98E-19
YOR315W	15:143597	5.69E-09
YIL036W	15:174364	8.21E-10
YOR316C	2:584357	1.78E-07
YOR316C	13:99720	1.82E-10
YHR059W	2:551299	2.91E-08
YHR193C	3.954861111	7.18E-09
YNL156C	12:662627	9.96E-42
YKL120W	3:81832	1.01E-87
YKL120W	13:28622	4.43E-08
YKL120W	15:180222	2.59E-11
YKL051W	13:33501	9.37E-13
YKL051W	15:143597	2.97E-21

YML009C	2:555575	2.89E-10
YML009C	16:500348	4.85E-08
YOL115W	2:551299	4.15E-08
YPL038W	16:489143	5.47E-16
YPL117C	12:662627	7.92E-22
YNL234W	15:136327	7.40E-12
YOR330C	2:562415	1.16E-07
YHR144C	15:174364	1.53E-11
YLR340W	2:519049	1.21E-07
YLR340W	5:194873	3.80E-08
YLR340W	15:170945	2.38E-10
YGL219C	7:85112	8.48E-20
YNL301C	2:499012	2.71E-15
YNL301C	4:95437	1.83E-07
YNL301C	5:194873	6.27E-12
YNL301C	15:170945	7.02E-14
YFL010W-A	6:144815	1.53E-12
YKR089C	11:599170	3.29E-17
YER011W	5:350744	3.57E-10
YER011W	12:662627	7.35E-11
YER011W	15:154177	1.44E-09
YGR014W	2:555787	1.23E-19
YDL078C	2:507282	2.42E-09
YKL104C	15:143597	2.73E-07
YOR375C	3:81832	4.09E-75
YOR375C	4:251013	1.02E-15
YOR375C	5:183958	9.49E-14
YOR375C	13:99717	5.00E-09
YIL072W	9:214482	1.33E-14
YAR050W	1:199778	6.77E-23
YOL094C	15:154177	3.20E-09
YIR042C	6:234349	7.59E-25
YIR042C	9:437054	#######
YLL056C	12:26184	2.78E-12
YLL056C	13:27644	2.06E-07
YLL056C	15:136324	1.05E-08
YKR021W	2:551299	2.14E-08
YBL085W	2:519049	6.99E-07
YPL027W	12:662627	6.32E-08
YBR208C	13:81250	1.09E-16
YCL066W	3:201166	#######
YLR118C	15:170945	7.61E-10
YDR198C	15:174364	1.11E-10
YOL040C	2:519049	1.07E-13
YOL040C	5:194873	1.74E-09
YOL040C	9:74540	3.36E-08
YOL040C	14:547071	6.41E-08

YOL040C	15:174364	2.10E-13
YOL040C	16:500342	9.05E-11
YJL011C	15:154309	8.30E-08
YGR020C	2:555575	4.65E-09
YMR314W	6.628472222	1.78E-07
YMR314W	8:176670	1.34E-10
YMR314W	15:594024	5.69E-08
YOL052C	2:499012	4.01E-09
YMR105C	15:174364	1.47E-12
YNL061W	15:150651	4.11E-08
YJL160C	2:565216	1.22E-09
YJL160C	10:122312	1.31E-15
YCR038C	3:201166	3.22E-33
YER070W	8:111690	8.74E-12
YOR032W-A	13:46084	4.90E-10
YDR365C	4:1213416	6.25E-94
YDR365C	9:98955	4.86E-10
YBR181C	2:499889	1.16E-12
YBR181C	4:95437	1.46E-07
YBR181C	5:194873	1.07E-10
YBR181C	9:74540	3.27E-08
YBR181C	15:170945	2.45E-14
YBR181C	16:500342	8.07E-10
YJR105W	12:662627	5.50E-12
YJR096W	2:533262	1.66E-08
YJR096W	15:170945	8.62E-24
YKL014C	15:174364	6.94E-09
YGL105W	2:553812	6.65E-08
YMR013C	2:517365	7.74E-07
YDL081C	4:95437	7.43E-09
YDL081C	5:194873	8.57E-08
YDL081C	9:74540	2.64E-08
YDL081C	15:170945	1.35E-08
YNL294C	15:143597	5.46E-07
YHR004C	12:662627	8.10E-09
YPL072W	2:551299	3.34E-09
YPL072W	16:500348	1.63E-09
YBR083W	2:391856	7.93E-09
YBR083W	8:111683	1.08E-18
YBR083W	13:46084	3.99E-09
YNL176C	2:551299	1.07E-07
YEL049W	1:154328	2.49E-27
YDR406W	15:144659	1.85E-28
YJR101W	2:555575	1.22E-07
YJR101W	15:150651	2.64E-07
YBR185C	2:562409	3.57E-10
YBR185C	15:154309	3.73E-08

YDR324C	15:174364	1.14E-07
YJR086W	8:111683	1.94E-08
YER062C	15:113267	7.36E-18
YHR208W	3:81832	1.42E-42
YHR208W	15:180222	2.57E-09
YFR028C	2:537314	1.89E-09
YCL004W	3:100213	1.76E-09
YMR150C	13:562907	5.40E-16
YLR079W	2:562415	1.29E-13
YBR098W	2:477206	3.22E-21
YHL024W	15:174364	2.95E-13
YHL024W	16:495156	7.11E-09
YMR152W	13:562907	3.88E-15
YDL174C	12:662627	2.37E-16
YER081W	13:46084	7.65E-08
YFL001W	2:555575	7.74E-08
YFL001W	6:144755	1.61E-07
YKL010C	11:421190	1.11E-14
YOR247W	2:537314	5.93E-13
YOR247W	13:77684	1.30E-08
YOR247W	15:141633	7.23E-10
YJL171C	3:201167	6.08E-16
YJL171C	10:99927	2.44E-16
YOR232W	2:551299	5.30E-18
YOR232W	7:375499	1.76E-08
YOR232W	8:167506	6.41E-12
YOR232W	16:500348	2.13E-13
YJR076C	10:579935	6.81E-09
YPL186C	13:28694	2.06E-08
YPL186C	15:174364	3.77E-35
YKR103W	11:649438	#######
YJL069C	15:174364	4.57E-09
YPR194C	2:555596	6.34E-09
YPR194C	16:927506	3.47E-31
YJL059W	10:388461	4.31E-09
YFL040W	6:57455	3.02E-29
YPL049C	13:49894	2.66E-08
YGL025C	15:154177	4.16E-08
YIR028W	2:551299	7.01E-29
YIR028W	13:110808	9.58E-10
YIR028W	16:500342	4.66E-12
YCL021W-A	3:81832	5.55E-95
YLR258W	2:567221	7.19E-13
YLR258W	15:174364	3.48E-10
YBR101C	7:375499	1.48E-23
YAL041W	2:569414	2.16E-09
YAL041W	15:143597	8.74E-07

YDR044W	5:350744	9.32E-14
YDR044W	12:662627	1.85E-10
YGR152C	2:499012	3.30E-08
YGR152C	5:196196	2.81E-07
YGR152C	8:185012	1.67E-07
YGR152C	15:143597	3.46E-07
YHL003C	8:111679	1.51E-16
YPR139C	13:99675	1.28E-08
YMR092C	13:445622	8.86E-26
YGR238C	13:79760	2.64E-07
YGR238C	15:150651	7.23E-07
YML052W	2:555787	3.80E-08
YGL130W	15:174364	7.68E-09
YBR193C	2:567221	6.24E-12
YCL040W	13:46084	7.83E-10
YCL040W	15:174364	3.55E-08
YBR237W	2:567221	2.81E-08
YHR203C	2:499012	9.03E-14
YHR203C	5:194873	2.19E-10
YHR203C	9:74540	4.88E-09
YHR203C	14:547071	1.17E-09
YHR203C	15:170945	1.94E-12
YMR143W	4:95527	1.15E-07
YMR143W	5:200848	7.43E-09
YMR143W	15:170945	6.16E-09
YBR150C	2:555596	1.37E-27
YOR288C	15:179289	6.93E-08
YLR315W	8:167506	5.96E-07
YMR024W	2:537314	4.75E-09
YDR394W	15:594024	2.75E-07
YOL088C	15:174364	2.36E-26
YBL061C	2:551299	6.31E-08
YBL061C	15:174364	4.07E-17
YER044C	12:662627	4.88E-39
YGL051W	7:403626	#######
YKL068W-A	2:553812	7.46E-08
YLL057C	12:27765	3.22E-14
YKL073W	15:154309	5.57E-08
YBR048W	2:499012	5.94E-13
YBR048W	15:170945	1.58E-11
YBR048W	16:500342	2.93E-09
YML007W	13:245632	2.05E-14
YGL135W	2:537314	5.99E-08
YGL135W	4:96271	1.04E-10
YGL135W	5:196196	8.49E-08
YGL135W	9:74540	6.96E-09
YGL135W	15:174364	4.88E-15

YDR329C	15:174364	2.43E-09
YER060W	2:548401	9.62E-10
YER060W	15:179289	6.18E-16
YGL237C	15:174364	4.93E-12
YDR037W	12:662627	2.02E-08
YDR037W	15:170945	4.85E-07
YAL016W	12:674651	2.23E-08
YBL042C	5:116722	4.30E-15
YOR342C	2:567221	1.93E-19
YBR071W	8:111679	1.10E-08
YBR071W	15:154309	2.91E-15
YDL204W	4:95437	8.09E-07
YDL204W	10:380085	1.60E-07
YDL204W	15:174364	2.11E-34
YER096W	2:537314	7.24E-15
YER096W	15:143597	5.52E-12
YGL056C	12:674651	3.19E-12
YHR104W	15:174364	4.65E-18
YBL046W	2:133749	3.28E-14
YNR053C	2:551299	5.44E-09
YGR212W	7:913059	4.04E-18
YBR177C	15:143597	1.88E-26
YOL058W	13:49894	7.32E-13
YCR092C	10:511123	5.80E-09
YEL075C	4.607638889	2.74E-15
YOR198C	15:703769	8.19E-13
YFL022C	2:499012	7.60E-10
YFL022C	15:174364	3.40E-11
YJL054W	2:551299	1.71E-08
YDR333C	2:555575	1.60E-08
YER018C	5:193876	4.17E-14
YNL165W	2:551299	2.58E-08
YOL100W	15:113261	3.53E-17
YNL256W	15:174364	3.21E-13
YBR147W	15:174364	3.08E-11
YML104C	13:49894	2.20E-10
YOR134W	2:567221	5.96E-07
YOR134W	15:170945	1.42E-11
YIR018C-A	9:386046	3.55E-12
YPL144W	16:280629	2.31E-22
YER072W	13:27644	7.76E-39
YML079W	13:110808	5.66E-16
YPL018W	13:28694	1.41E-24
YAL024C	2:551299	3.23E-12
YAL024C	16:500348	8.36E-09
YPL274W	13:910381	3.15E-13
YIL165C	2:551299	6.31E-17

YIL165C	9:47053	1.82E-26
YER158C	5:420595	1.44E-09
YJR047C	12:634227	2.86E-08
YBR126C	2:533268	1.59E-07
YBR126C	15:174364	5.97E-09
YER185W	5:568650	1.55E-18
YNR001C	13:99675	3.35E-08
YNR001C	15:136324	1.66E-12
YNR001C	16:500342	7.21E-10
YKL079W	13:99675	4.92E-07
YJR062C	10:548177	5.59E-10
YAL039C	2:537314	1.23E-07
YAL039C	12:672779	1.73E-07
YAL039C	15:141627	1.68E-07
YHR035W	8:176994	2.76E-25
YJL105W	12:659357	2.65E-13
YDL124W	2:667125	1.22E-10
YDL124W	4:262796	1.28E-51
YDL124W	11:421190	1.38E-11
YJL078C	2:562415	7.18E-41
YDR135C	15:143597	1.66E-09
YGR195W	15:154309	1.48E-09
YNR014W	15:174364	7.57E-23
YLR329W	12:790025	2.50E-42
YHL023C	2:551299	9.94E-08
YKR102W	11:656099	4.52E-52
YLR420W	5:117056	4.18E-42
YLR420W	12:964986	1.83E-32
YGR007W	13:69114	1.50E-07
YGR007W	15:150651	4.94E-12
YCR107W	10:28306	2.78E-12
YCR107W	0.868055556	1.02E-07
YNL037C	2:533262	5.82E-08
YGL167C	13:69114	5.35E-07
YLR178C	5:196352	2.90E-08
YLR178C	12:516242	4.30E-14
YLR178C	13:77684	7.08E-09
YLR178C	15:174364	1.23E-35
YFL062W	7:1083095	1.11E-12
YEL040W	15:174364	2.96E-18
YNL123W	14:371953	6.54E-13
YMR316W	13:910381	1.92E-16
YJL122W	2:555575	1.41E-07
YOR114W	15:524972	5.76E-14
YJL177W	2:499012	9.45E-15
YJL177W	5:193876	1.22E-12
YJL177W	15:170945	4.08E-12

YJR042W	2:562415	7.21E-07
YLR083C	15:170945	6.99E-09
YML069W	13:99720	7.98E-12
YCL016C	3:100213	5.44E-25
YJR094W-A	2:489202	8.30E-09
YJR094W-A	4:95527	1.26E-07
YJR094W-A	5:196196	1.38E-09
YJR094W-A	15:170945	8.52E-13
YHR160C	15:174364	1.48E-22
YGR207C	15:108577	2.98E-07
YDR070C	15:174364	1.95E-35
YBR050C	15:842027	6.01E-12
YJR090C	15:141627	1.02E-07
YGR049W	12:662627	9.65E-26
YBR196C-A	2:608310	4.45E-43
YIL060W	9:251537	2.20E-09
YOR377W	12:659357	4.76E-19
YPL120W	8:213595	2.51E-07
YDL075W	2:519049	7.94E-09
YDL075W	5:194873	2.54E-09
YDL075W	15:170945	4.95E-13
YLL060C	12:22752	1.44E-09
YBR092C	2:506661	1.20E-14
YJL110C	2:553812	2.49E-18
YEL073C	5:15697	1.01E-22
YBR030W	15:174364	1.64E-09
YDR033W	4:509817	1.77E-72
YDR033W	10:122312	6.15E-10
YDR033W	16:523450	2.84E-12
YDL132W	4:226317	1.84E-24
YMR201C	12:662627	4.07E-10
YMR321C	13:922256	1.65E-16
YJL112W	15:150651	2.77E-09
YJL112W	16:511406	1.19E-07
YDR115W	2:551299	1.73E-07
YDR115W	16:511406	1.90E-07
YPL063W	2:551299	5.40E-08
YAL063C	1:16984	2.92E-30
YAL063C	8:525664	2.13E-09
YMR310C	15:143597	5.57E-08
YPL039W	16:486643	1.37E-20
YLR164W	2:537314	4.38E-14
YLR164W	12:469604	4.24E-20
YLR164W	13:78655	1.84E-09
YLR164W	15:141627	8.30E-11
YML006C	15:174364	3.54E-07
YHL001W	2:499889	2.34E-14

YHL001W	5:194873	1.39E-11
YHL001W	15:170945	1.22E-12
YHL001W	16:500348	3.42E-11
YGR255C	15:174364	9.18E-10
YFR010W	6:152824	2.29E-11
YNL168C	2:553812	3.30E-10
YDR482C	13:27644	1.92E-13
YDR482C	15:174364	2.54E-07
YNL159C	8:151814	3.18E-08
YPL123C	2:517365	4.91E-08
YPL123C	15:174364	2.76E-08
YOL013W-B	15:301074	1.92E-08
YHR021C	2:499889	8.11E-11
YHR021C	4:95527	1.11E-08
YHR021C	5:193876	4.73E-13
YHR021C	9:74540	8.81E-08
YHR021C	15:170945	1.65E-13
YHR021C	16:500342	1.42E-09
YPL071C	2:551299	3.33E-08
YMR217W	12:317608	8.47E-10
YMR217W	15:174364	3.13E-16
YER039C-A	8:176670	5.25E-09
YER039C-A	16:500348	1.03E-07
YKR061W	2:551299	1.84E-07
YFL014W	15:143597	8.42E-31
YFL014W	16:500354	1.15E-13
YBR187W	15:174364	5.59E-11
YGL249W	16:500348	1.48E-09
YBR176W	2:584357	4.06E-15
YLR450W	12:1031688	2.27E-13
YLL012W	12:662627	5.09E-11
YLL012W	15:174364	1.75E-11
YJL133C-A	12:634227	2.86E-08
YBR191W	2:519049	1.04E-11
YBR191W	5:196196	8.49E-10
YBR191W	9:74540	5.12E-08
YBR191W	15:170945	1.56E-12
YMR119W	13:506838	2.63E-09
YOR011W	14:449639	8.10E-16
YOR011W	15:438824	2.75E-29
YCR107W	6.628472222	2.20E-07
YCR107W	0.868055556	1.69E-08
YCR107W	12:1054278	1.18E-09
YNL299W	2:555575	2.63E-08
YLR350W	8:111690	2.50E-07
YLR350W	12:829693	1.40E-15
YLR438C-A	12:1019347	4.23E-08

YOR234C	5:193876	2.56E-10
YOR234C	15:170945	1.23E-12
YNL059C	14:502496	1.76E-10
YNR065C	15:179289	8.58E-12
YMR285C	2:521415	1.61E-07
YBL040C	2:133749	1.79E-16
YGR060W	12:662627	3.57E-14
YGR060W	15:170945	6.76E-08
YAR023C	1:185122	3.63E-35
YJL001W	10:450212	3.27E-10
YPR160W	5:196196	1.44E-08
YPR160W	15:174364	7.52E-10
YJL074C	8:111680	2.77E-09
YLR411W	12:956534	#######
YBR242W	12:662627	2.36E-24
YLR121C	13:77684	2.71E-09
YLR121C	15:154309	7.02E-16
YJL219W	10:22453	1.22E-47
YNL041C	14:553129	9.50E-11
YKR013W	2:562415	4.46E-16
YKR013W	15:174364	1.98E-08
YAR075W	6:33334	1.94E-10
YAR075W	13:205921	1.04E-11
YAR075W	15:154177	5.71E-16
YNR034W-A	1:41483	8.14E-16
YNR034W-A	5:222998	2.67E-07
YNR034W-A	15:174364	8.95E-29
YHR048W	12:659357	1.45E-13
YER065C	16:500342	1.93E-07
YHL044W	6.176388889	8.89E-69
YER034W	13:57145	3.07E-08
YPL004C	13:28622	9.83E-08
YPL004C	15:143597	9.92E-10
YPL004C	16:500342	1.31E-13
YER053C-A	12:662627	6.25E-29
YDR104C	12:956534	1.89E-06
YDR104C	15:174364	9.21E-10
YLR201C	15:174364	2.75E-12
YOL096C	15:154309	1.36E-15
YCR073W-A	12:659357	8.96E-08
YJL167W	12:659357	4.97E-21
YNL141W	13:27644	4.19E-08
YDR298C	15:136324	9.83E-08
YDR242W	2:551299	4.99E-17
YPL085W	2:530481	1.55E-08
YPL100W	15:170945	8.52E-09
YMR015C	12:662627	1.32E-28

YHR143W	2:555596	#######
YHR143W	4:95437	4.04E-09
YPR149W	5:200848	1.70E-07
YPR149W	15:143597	2.33E-26
YKR091W	2:555596	2.64E-25
YIL120W	9:136845	4.75E-16
YIL120W	13:99675	6.97E-09
YEL071W	15:113251	9.21E-07
YGL255W	13:69122	1.61E-07
YGL255W	15:174364	1.85E-08
YBL092W	2:519049	1.95E-14
YBL092W	5:196196	2.06E-10
YBL092W	15:143597	5.02E-16
YBL092W	16:500342	1.28E-09
YBR093C	1:141181	7.39E-12
YBR093C	13:27644	1.49E-96
YAL009W	1:136161	2.27E-21
YHL009W-B	8:84437	2.35E-15
YEL042W	8:167506	6.45E-07
YGR061C	13:79760	1.59E-08
YDR075W	15:174364	9.26E-09
YOL087C	15:174364	4.04E-10
YML046W	8:111683	5.25E-32
YLR053C	2:551299	1.47E-20
YMR238W	15:180961	7.96E-08
YDR320C	4:1108558	5.57E-10
YCR097W	3:201166	#######
YIL066C	12:514835	5.41E-27
YBR158W	2:562415	1.05E-42
YPL203W	15:170945	9.69E-10
YMR195W	15:141633	4.13E-16
YMR195W	16:500348	2.15E-08
YGR094W	15:170945	9.02E-12
YMR108W	3:91049	2.29E-15
YMR108W	15:180222	2.04E-10
YDR434W	15:174364	1.87E-08
YIL149C	2:551299	9.31E-12
YBR119W	2:477206	3.99E-20
YKR005C	2:551299	3.57E-12
YFR006W	3:90610	5.13E-09
YFR006W	13:46084	1.30E-13
YFR006W	15:136327	1.27E-16
YPR127W	15:170945	9.75E-08
YBR041W	15:594024	1.49E-06
YBR041W	16:511406	1.20E-07
YEL041W	5:79647	1.02E-20
YEL041W	8:457580	9.72E-36

YDR381W	2:555778	4.98E-15
YLR162W-A	12:472165	2.80E-96
YML082W	15:150651	1.01E-10
YHR032W	8:167504	7.00E-37
YNL237W	14:206186	1.40E-47
YHR099W	2:551299	6.16E-10
YHR099W	16:500348	8.62E-08
YJL136C	2:499889	2.82E-10
YJL136C	5:193876	2.30E-10
YJL136C	15:170945	3.52E-13
YOR116C	2:551299	5.00E-09
YOR031W	15:392765	8.62E-44
YJL053W	2:555575	2.51E-08
YFL041W-A	15:143597	1.01E-08
YDR379W	2:555787	3.12E-08
YDR379W	16:511406	8.44E-08
YDR281C	1:141181	3.77E-09
YDR281C	13:27644	2.89E-57
YOR108W	15:490329	1.03E-07
YIL145C	15:143597	8.60E-12
YIL133C	2:499012	3.55E-13
YIL133C	5:193876	2.34E-12
YIL133C	15:170945	1.62E-13
YIL133C	16:500342	1.24E-09
YER132C	5:422612	4.78E-13
YER132C	13:99675	4.21E-08
YER132C	15:170945	5.74E-09
YOL030W	15:154177	6.31E-08
YFR031C-A	2:519049	3.98E-17
YFR031C-A	4:95437	3.03E-08
YFR031C-A	5:196196	1.64E-12
YFR031C-A	9:74540	1.03E-09
YFR031C-A	14:547071	7.79E-10
YFR031C-A	15:170945	8.80E-17
YFR031C-A	16:500342	1.11E-14
YOR293W	2:489202	2.18E-08
YOR293W	4:96271	4.96E-08
YOR293W	5:200848	2.41E-11
YOR293W	15:170945	2.97E-11
YOR293W	16:500342	1.90E-09
YLR167W	9:74540	1.89E-08
YLL023C	12:99261	4.81E-11
YLL023C	15:174364	1.85E-10
YPL082C	2:506661	5.00E-13
YGL030W	5:200848	3.92E-09
YGL030W	9:101011	7.54E-08
YGL030W	15:170945	1.48E-17

YMR143W	5:200848	1.75E-07
YNL158W	8:167504	3.13E-12
YGR070W	15:174364	1.78E-14
YOL082W	2:553812	2.81E-07
YOL082W	15:179289	4.81E-31
YGR044C	2:555596	1.74E-31
YML029W	13:100048	1.15E-09
YMR111C	8:167504	4.45E-08
YDR225W	8:111680	9.27E-08
YNL312W	8:111683	1.03E-06
YNL238W	14:245313	6.49E-09
YPR028W	15:174364	3.33E-09
YOR229W	12:644136	2.82E-10
YPR192W	16:927500	5.81E-74

Supplementary Table 3: eQTLs for Union

Accession ID	QTL chrom:position	p-value
YKR009C	15:143597	1.48E-08
YKR009C	16:500348	5.57E-06
YDL157C	4:165026	9.03E-08
YER059W	2:537314	1.10E-06
YDR481C	13:33501	2.02E-14
YML113W	13:69114	2.74E-07
YML113W	15:174364	1.08E-09
YIL157C	15:154309	2.68E-09
YNR074C	12:662627	1.22E-10
YKL012W	15:180180	5.21E-06
YJL147C	2:555575	1.53E-07
YJL147C	10:123859	4.51E-12
YJL147C	16:500348	9.53E-07
YDL216C	2:555575	1.01E-05
YDL216C	4:70901	1.43E-16
YPL223C	15:174364	6.20E-12
YKL138C	2:551299	5.33E-07
YKL138C	8:167506	1.16E-05
YKL138C	15:174364	1.22E-07
YNL126W	2:569420	7.90E-06
YML035C	15:174364	1.34E-06
YIL160C	15:174364	4.09E-09
YLR050C	12:238298	6.05E-15
YOL008W	15:298710	8.55E-08
YOR072W-B	14:558284	1.03E-10
YPL253C	16:70853	3.12E-07
YDL048C	15:174364	8.74E-09
YDR056C	2:537314	1.28E-06
YNL083W	15:106266	5.66E-08
YPL154C	15:174364	3.81E-08
YNL216W	14:246135	7.48E-13
YFL034C-B	2:551299	7.23E-09
YGL222C	7:85112	6.61E-16
YIR021W	2:551299	2.62E-06
YIR021W	15:170945	2.26E-06
YEL006W	2:551299	3.91E-06
YLR401C	12:956534	5.22E-09
YLR401C	15:154309	2.78E-06
YOR192C-C	15:703771	1.56E-12
YKL195W	15:174364	3.49E-09
YDR144C	4:744330	1.37E-09
YDR144C	15:150651	7.68E-06
YBR149W	15:174364	2.98E-09
YOR175C	12:662627	1.41E-08
YHL010C	2:555575	2.91E-06

YHL010C	8:84437	1.77E-14
YHR152W	8:111683	1.02E-06
YHR152W	13:46084	1.98E-07
YER035W	5:200848	9.15E-07
YER035W	7:375499	7.68E-09
YER035W	15:174364	2.74E-07
YHR002W	15:154309	1.31E-06
YPL020C	15:108577	5.37E-07
YPL020C	16:511406	1.99E-12
YLR286C	2:562409	3.24E-16
YJL185C	15:174364	2.95E-08
YMR317W	15:154177	2.04E-06
YIL125W	15:174364	8.11E-08
YGR130C	15:174364	2.03E-10
YDL049C	13:81358	3.97E-06
YBR065C	2:368060	3.91E-14
YJR082C	10:579935	1.13E-12
YMR182C	2:486640	6.82E-07
YPL264C	5:272258	1.16E-11
YGR080W	12:674651	2.24E-06
YLR125W	2:507282	1.29E-06
YAR033W	1:185122	3.04E-12
YGR113W	2:569420	9.45E-08
YPL050C	16:462646	2.24E-11
YDR447C	15:170945	7.20E-07
YML019W	12:659357	4.99E-08
YML019W	13:227254	8.75E-11
YKL166C	11:129847	3.77E-07
YNR038W	15:150651	5.63E-07
YJL165C	15:143597	2.03E-06
YDL086W	12:662627	3.36E-15
YKL096W	11:261779	3.35E-09
YKL096W	15:136327	2.75E-11
YLR064W	12:266102	5.21E-08
YEL003W	15:136324	3.49E-06
YKL075C	12:674651	2.75E-06
YHR198C	15:174364	7.40E-10
YMR212C	12:659357	5.09E-09
YLR149C	15:174364	1.18E-10
YOR271C	3:81832	1.85E-13
YOR271C	15:174364	9.57E-08
YHR197W	15:174364	2.71E-08
YAL034C	15:174364	2.60E-08
YOR084W	15:174364	6.32E-08
YDR140W	4:744330	1.72E-12
YMR216C	13:698980	5.60E-11
YJL042W	15:170945	4.25E-09

YGR010W	13:91085	2.26E-06
YDR245W	15:170945	1.04E-07
YOR187W	15:174364	1.49E-09
YGL121C	15:174364	8.83E-07
YJL186W	13:27644	2.59E-08
YBR285W	15:143597	6.86E-08
YOL028C	15:179289	4.76E-09
YMR220W	12:662627	2.41E-14
YAR002W	1:154328	1.02E-14
YMR049C	15:174364	4.50E-06
YOR069W	15:461694	1.91E-06
YPR106W	2:555596	1.12E-16
YBL031W	12:634227	1.44E-06
YOR124C	14:449639	9.40E-15
YDL149W	15:174364	2.09E-06
YDR072C	2:519049	1.48E-08
YGL107C	2:506661	1.65E-09
YGR110W	15:143597	8.04E-07
YOR394C-A	15:1065719	3.34E-14
YBR157C	2:555596	1.24E-13
YKL192C	15:170945	4.64E-08
YLR283W	12:705190	2.76E-14
YMR291W	3:105042	3.62E-07
YMR291W	15:193911	3.81E-06
YLR151C	12:433955	1.47E-14
YOR165W	15:632882	4.73E-07
YBL018C	2:185450	6.52E-16
YNL095C	15:174364	9.90E-07
YPL101W	2:517123	7.26E-06
YBR169C	15:174364	1.17E-07
YCL068C	3:201166	2.86E-13
YFL049W	6:33688	2.25E-07
YKL167C	15:170945	5.59E-09
YOR264W	2:562409	1.97E-15
YIR039C	9:437054	1.84E-16
YOL122C	15:108577	1.86E-08
YER053C	13:28622	1.60E-06
YER053C	15:174364	5.12E-09
YOL105C	2:499895	4.32E-08
YOL105C	15:141627	4.30E-13
YCL018W	3:81832	6.44E-16
YDR518W	12:672779	6.17E-10
YDR210W	2:548401	2.33E-06
YLR089C	13:49903	1.22E-07
YMR227C	15:143597	1.05E-07
YIL161W	9:46561	1.87E-14
YCL054W	15:150651	1.35E-07

YOL162W	15:10427	4.75E-17
YFL047W	2:537314	2.60E-08
YFL047W	8:111680	5.21E-08
YDR261C	12:635380	3.44E-06
YOL077W-A	15:141627	9.95E-09
YDR119W	2:582419	7.69E-06
YDL125C	4:246738	1.31E-10
YGR083C	15:174364	2.71E-08
YOR285W	15:846344	3.33E-16
YBR084C-A	15:154309	7.66E-07
YBR296C	13:27644	2.45E-14
YML064C	13:28694	6.04E-08
YHR005C	8:111683	1.63E-11
YHR005C	13:91085	1.34E-06
YPL215W	15:174364	5.78E-07
YGR279C	7:1048152	4.42E-14
YAL003W	2:537314	7.73E-06
YML023C	13:243624	1.19E-08
YGL196W	2:555596	3.27E-08
YGL196W	7:131386	2.68E-10
YFR036W	6:227675	3.08E-06
YBL089W	2:50915	3.79E-12
YHR124W	13:110808	7.07E-07
YDR231C	15:170945	3.59E-08
YHR053C	2:555596	1.80E-11
YGL104C	7:312740	2.69E-12
YMR169C	15:136327	7.48E-12
YOL071W	15:174364	2.19E-08
YDL012C	4:433589	5.71E-16
YOL011W	13:28334	1.27E-06
YPL002C	16:555416	9.54E-17
YER167W	15:180961	6.61E-06
YKL194C	15:174364	2.84E-09
YHL020C	8:71742	2.42E-09
YGL179C	7:167587	7.58E-11
YHL032C	15:174364	2.39E-12
YKL140W	11:178402	2.87E-14
YPL127C	15:108577	8.28E-07
YKL211C	5:272255	8.00E-09
YKL211C	11:46633	1.48E-06
YBR142W	15:174364	4.66E-06
YBR295W	13:46084	3.05E-07
YBR295W	15:143597	4.34E-09
YKL085W	15:174364	7.32E-09
YMR048W	13:371857	1.03E-16
YLR312W-A	12:757807	1.84E-10
YML091C	13:49894	3.64E-10

YML091C	15:174364	9.90E-07
YLR299W	2:592863	1.14E-06
YLR299W	12:744310	1.07E-06
YMR128W	15:150651	2.55E-06
YPR155C	15:174364	1.93E-10
YOR338W	4:95437	4.75E-07
YJL196C	1:41483	1.09E-07
YJL196C	7:402879	1.42E-06
YOR179C	2:551299	3.77E-07
YMR131C	15:174364	1.56E-07
YNL055C	14:525061	1.04E-11
YNL055C	15:143597	2.96E-06
YBR128C	15:143597	2.55E-06
YCR094W	15:141627	2.71E-07
YLR212C	8:111682	3.49E-06
YDL108W	4:273846	1.64E-09
YOR042W	15:170945	5.47E-07
YGL252C	7:15891	7.00E-14
YDL244W	10:24469	4.46E-09
YDL244W	11:657281	8.39E-06
YDR076W	15:179289	2.55E-07
YPL131W	15:170945	5.77E-06
YMR221C	15:180222	1.67E-06
YBR215W	2:658746	1.51E-10
YBR215W	12:662627	4.81E-06
YLL027W	12:92676	1.15E-12
YKL132C	11:194611	2.92E-15
YJL200C	2:477206	2.33E-11
YJL200C	15:193911	1.22E-06
YKR092C	11:612769	4.25E-07
YKR092C	15:141633	2.37E-06
YJL082W	15:143597	4.14E-06
YDL168W	4:154436	7.38E-17
YGR088W	15:154309	2.60E-10
YMR135C	15:154309	1.54E-11
YDL085W	15:174364	1.22E-07
YDL236W	12:662627	3.11E-08
YLR300W	12:721979	3.68E-13
YPR184W	15:174364	6.43E-10
YHR046C	8:193175	9.13E-11
YHR046C	15:143597	7.15E-06
YPL078C	15:154309	7.34E-08
YPL078C	16:500348	3.62E-06
YBR095C	15:174364	1.11E-06
YKL135C	8:111690	1.19E-07
YKL135C	11:178411	7.20E-09
YDL223C	15:174364	6.43E-10

YNR067C	2:555596	1.35E-16
YBR186W	2:562415	1.09E-08
YAL067C	1:10152	3.20E-16
YLR346C	12:811669	7.93E-08
YLR346C	15:136327	6.22E-06
YDR516C	15:174364	1.13E-07
YDR239C	15:141627	1.24E-05
YMR115W	13:492453	3.90E-09
YOL066C	15:205104	2.67E-11
YFL052W	10:23505	4.58E-16
YEL051W	12:644082	1.06E-06
YGL006W	7:490784	2.35E-11
YDL055C	2:555596	2.88E-13
YPL206C	16:125271	3.40E-08
YGL084C	2:551299	6.06E-07
YOL090W	15:154177	9.70E-11
YML124C	2:562409	2.50E-11
YML124C	16:511406	1.72E-06
YHR065C	15:170945	3.28E-06
YOR062C	15:428238	1.57E-12
YOL036W	15:174364	8.81E-08
YPL067C	16:428900	2.19E-08
YOL098C	15:141627	1.52E-07
YOL021C	2:551299	1.48E-07
YMR008C	15:143597	5.86E-07
YJL133W	2:555596	7.66E-06
YJL133W	10:159479	7.72E-09
YDR079W	15:154177	1.63E-07
YDR454C	15:154309	6.47E-08
YIL009W	2:553812	4.37E-08
YPL262W	2:567221	6.97E-07
YIL166C	9:33795	7.64E-17
YFR038W	2:562415	1.80E-06
YML125C	12:644136	8.62E-08
YML125C	15:150651	1.44E-06
YBR028C	4:154436	6.87E-07
YBR028C	13:33681	5.50E-07
YHL001W	15:154309	2.14E-06
YDR440W	4:1344670	2.74E-12
YDR460W	3:201166	2.81E-16
YLR313C	12:760763	2.93E-14
YDR062W	15:141627	6.04E-07
YOR178C	15:174364	2.98E-09
YJL016W	10:404508	1.53E-15
YJL218W	10:23505	2.27E-16
YDL218W	2:533262	1.56E-06
YDL218W	4:95527	7.58E-10

YLR256W	12:662627	8.81E-15
YBL095W	12:662627	2.73E-07
YEL021W	5:117056	5.22E-17
YLR343W	12:815480	4.30E-14
YML102W	8:111682	4.52E-07
YDR361C	15:174364	4.91E-08
YHR097C	13:46084	3.36E-06
YHR097C	15:174364	1.88E-09
YGL253W	15:154309	7.80E-07
YOR390W	13:910381	3.62E-07
YGR258C	2:537314	1.72E-07
YGR258C	15:143597	8.64E-09
YDL229W	15:154309	1.99E-06
YJL108C	2:555787	7.72E-07
YJL108C	10:218798	2.22E-12
YJL108C	12:634226	3.23E-07
YJL193W	10:56788	3.54E-10
YDL155W	2:562415	4.28E-06
YER103W	15:174364	2.26E-06
YIR026C	9:403134	1.48E-11
YHR024C	15:113267	6.09E-06
YOR045W	15:16838	9.08E-07
YEL062W	2:555575	5.92E-08
YEL062W	5:30057	1.42E-11
YEL062W	12:1067121	3.90E-06
YDR145W	2:533268	5.45E-06
YDR145W	14:449639	1.12E-09
YIL151C	9:84805	1.48E-08
YGR204C-A	7:905017	7.22E-12
YKL110C	11:229040	1.34E-06
YMR088C	2:551299	1.18E-07
YJL205C	15:179289	3.25E-07
YLR052W	12:254693	8.26E-11
YLR052W	15:175594	5.01E-07
YNL011C	15:136327	1.65E-07
YLL049W	13:100048	8.35E-07
YJR121W	15:174364	7.00E-09
YDR539W	4:1510883	1.34E-16
YJR088C	15:136324	4.50E-06
YNL313C	14:38762	2.79E-12
YNL313C	15:170945	3.26E-07
YKL180W	15:154309	4.63E-06
YKL116C	11:218805	2.03E-15
YMR301C	15:154309	3.86E-06
YNR020C	2:555787	1.39E-09
YNR020C	15:154177	2.21E-06
YHR204W	15:132423	8.19E-06

YEL036C	2:519049	1.77E-08
YEL036C	15:141627	5.16E-07
YLR185W	15:174364	5.57E-06
YBR240C	13:57145	4.49E-07
YPR178W	12:662627	5.43E-08
YMR257C	2:565216	9.20E-06
YCR059C	15:174364	1.56E-09
YML093W	13:99585	2.09E-07
YML093W	15:150651	8.55E-06
YPL081W	4:1188862	7.03E-07
YHR087W	15:174364	5.07E-10
YDR055W	2:555575	8.81E-06
YHR038W	2:551299	1.91E-08
YHR038W	15:170945	3.68E-07
YOR266W	15:842027	2.83E-07
YOL063C	15:205104	5.29E-16
YLL028W	12:86369	9.11E-08
YLL028W	15:144659	4.59E-16
YHR005C-A	15:180222	2.53E-06
YKL059C	2:569420	2.96E-06
YKL081W	15:174364	7.58E-07
YLL058W	12:634226	2.43E-07
YPR055W	15:136324	1.09E-05
YJL088W	13:49894	2.20E-08
YCR098C	3:301446	1.09E-13
YHR043C	8:193175	6.91E-14
YPR048W	15:170945	3.26E-07
YLR181C	12:516700	1.25E-12
YOR321W	12:713644	1.12E-07
YKL188C	11:97761	1.86E-06
YBR108W	12:644136	2.91E-06
YGL089C	3:201166	8.94E-17
YPR069C	13:87587	3.43E-06
YDR415C	4:1291675	2.58E-06
YDR243C	1:51324	6.58E-14
YOR302W	15:154309	2.14E-06
YCR073C	15:174364	9.72E-11
YLR105C	13:49894	5.56E-07
YDR262W	4:975086	1.00E-15
YOL109W	15:136327	3.02E-10
YLR248W	12:634226	7.45E-07
YLR248W	15:174364	1.87E-06
YDR530C	4:1497132	2.68E-08
YDR530C	15:143597	6.60E-07
YKR056W	15:150651	3.99E-06
YML007C-A	12:681096	8.71E-06
YJL166W	15:174364	9.90E-07

YER013W	13:27644	1.51E-05
YER013W	15:136327	4.04E-10
YGL013C	15:141627	4.06E-07
YOL112W	2:537314	1.42E-10
YKR062W	11:566015	2.63E-08
YML067C	13:129925	6.70E-08
YPR015C	15:108577	6.80E-07
YIL094C	2:477206	4.54E-12
YBL072C	15:154309	5.07E-06
YER183C	5:568698	2.12E-09
YDR001C	2:533262	3.60E-07
YDR001C	4:446125	4.98E-10
YDR001C	15:174364	3.62E-07
YPL097W	15:174364	2.91E-06
YGL143C	15:174364	3.25E-06
YMR280C	15:174364	4.09E-09
YJL148W	15:174364	2.34E-07
YLR218C	15:170945	2.02E-10
YJR032W	10:470298	3.35E-14
YJR032W	15:174364	1.80E-09
YBL027W	15:154309	3.46E-06
YIL162W	9:38608	3.22E-10
YJL125C	10:159479	9.36E-08
YNL116W	15:141627	5.87E-06
YLR233C	12:607076	8.53E-13
YDL112W	15:174364	5.35E-07
YPL034W	16:500342	5.37E-06
YDL103C	4:273846	2.40E-09
YHL018W	13:81250	1.91E-06
YOL032W	15:255015	8.19E-14
YLR303W	8:167504	1.80E-06
YJL100W	12:662627	1.95E-11
YGL001C	12:662627	2.87E-14
YPR200C	11:652304	9.23E-10
YPR200C	16:932535	3.37E-07
YGR272C	15:136327	3.36E-08
YJL158C	15:136327	8.40E-07
YMR279C	12:469156	8.17E-15
YDL057W	2:551299	1.30E-09
YDL105W	4:273846	8.92E-16
YOR028C	15:380725	4.38E-12
YLR265C	12:674651	4.84E-15
YNR043W	12:662627	1.60E-14
YPL036W	16:500342	6.53E-12
YDR353W	2:555575	2.42E-06
YOR125C	15:572404	1.56E-09
YKL027W	11:388373	1.25E-11

YPL189W	2:567221	8.48E-06
YPL189W	16:182953	8.73E-10
YBR112C	2:480009	2.92E-07
YLR336C	12:782839	1.21E-06
YBR251W	2:551299	2.13E-06
YBR251W	15:174364	2.83E-08
YBR291C	2:480009	2.58E-13
YBR291C	13:81358	1.33E-05
YAR014C	2:567221	2.14E-11
YPL134C	16:256358	1.87E-08
YGR092W	8:111690	8.11E-06
YJR074W	10:573371	8.18E-07
YKR044W	15:174364	2.95E-08
YFR045W	15:174364	1.84E-11
YPR175W	7:375499	5.21E-10
YDR540C	4:1511257	1.62E-16
YPL017C	15:174364	1.36E-09
YPL017C	16:547618	2.03E-07
YJR014W	2:565216	1.67E-06
YER123W	13:57145	8.82E-07
YGL169W	7:187567	5.22E-17
YDR345C	2:555575	4.49E-06
YDR345C	4:201395	3.26E-06
YLR026C	2:548401	3.39E-06
YGR198W	12:662627	2.68E-06
YGR198W	15:632882	9.79E-10
YLR364W	8:167506	1.48E-05
YML123C	13:27644	5.06E-16
YHR011W	15:174364	1.95E-06
YOR090C	15:491172	1.18E-08
YPL258C	16:70847	5.19E-15
YJL140W	10:151310	5.49E-11
YDR416W	8:185882	6.58E-06
YDR416W	15:594024	1.61E-06
YAL042W	2:562415	5.15E-06
YGR205W	7:913065	1.01E-16
YER007C-A	15:174364	2.26E-06
YLL033W	12:514835	8.32E-10
YLL033W	15:589013	1.22E-06
YBR197C	2:616262	1.03E-16
YLR020C	12:662627	3.10E-06
YPR062W	12:672779	8.67E-07
YLR260W	12:672779	6.55E-11
YIL044C	9:272372	8.28E-10
YOR003W	2:555575	3.88E-08
YOR003W	12:659357	6.62E-11
YNL233W	8:111690	2.22E-08

YKL035W	15:174364	8.36E-09
YGL057C	7:403626	1.10E-13
YOL061W	15:174364	2.60E-08
YER124C	2:555596	1.74E-16
YBL068W	13:28334	1.03E-06
YBL068W	15:174364	2.72E-10
YPL272C	12:662627	1.42E-07
YMR318C	13:922268	9.47E-06
YKR088C	12:635380	3.62E-07
YDL199C	4:106892	5.50E-09
YDL199C	15:174364	7.32E-09
YKL045W	9:74540	5.91E-06
YKL045W	11:354466	4.63E-13
YFL030W	15:143597	1.11E-09
YLR139C	12:423789	6.44E-14
YPR193C	12:662627	2.56E-10
YOR319W	1:42591	1.87E-08
YOR319W	15:930110	5.31E-14
YHR149C	15:143597	1.45E-05
YLR237W	12:635380	1.30E-11
YKL162C	11:129847	5.15E-07
YKL162C	15:170945	2.89E-07
YDR100W	15:143597	1.02E-06
YPL245W	16:84943	1.90E-12
YHR036W	8:176412	7.31E-11
YPR174C	7:375499	1.76E-14
YJL056C	10:327858	6.24E-14
YMR282C	15:113261	4.85E-07
YAL017W	15:174364	4.48E-09
YOL097C	15:174364	8.14E-10
YKR087C	11:599170	7.57E-16
YOR341W	15:170945	1.44E-06
YDR364C	4:1188862	5.36E-10
YLR433C	8:93002	3.27E-06
YGR266W	2:530481	4.58E-06
YGR266W	12:662627	3.41E-12
YHR129C	15:174364	4.23E-07
YKL191W	15:170945	1.50E-06
YKR101W	11:645253	9.01E-10
YER093C	12:659357	4.99E-08
YMR156C	13:574867	7.18E-14
YDR090C	15:180961	7.35E-06
YFR033C	12:668249	1.42E-10
YGR287C	7:1063841	3.25E-15
YJL109C	15:170945	2.10E-06
YPL153C	8:111683	2.30E-07
YER149C	15:174364	3.37E-06

YLR275W	12:697260	6.21E-11
YBR135W	2:506661	6.95E-11
YDL148C	15:150651	7.15E-07
YOR228C	15:143597	6.10E-07
YDL229W	15:143597	2.36E-06
YKL187C	11:97725	2.42E-14
YML008C	12:662627	1.62E-13
YLR165C	12:472165	7.31E-16
YFL020C	1:154328	1.55E-12
YCR091W	15:174364	9.55E-09
YKL037W	15:174364	2.37E-09
YIR011C	12:662627	1.84E-06
YBL050W	2:530481	2.02E-06
YMR298W	12:634227	1.26E-07
YOL055C	2:555596	1.33E-05
YOL055C	8:151814	3.01E-07
YHR049W	15:488334	8.75E-06
YDR325W	15:106266	9.00E-06
YMR275C	13:824809	6.89E-10
YDR038C	4:527458	5.75E-17
YPL045W	15:113261	1.26E-06
YMR239C	15:174364	3.81E-08
YPR006C	16:555416	9.78E-16
YMR043W	15:106272	1.82E-06
YLR363W-A	15:174364	1.30E-08
YHR070W	15:170945	2.18E-06
YIL096C	9:191491	1.25E-06
YIL096C	15:170945	4.64E-08
YLR038C	12:668249	1.21E-06
YIL134W	9:98955	7.85E-08
YIL121W	12:677957	1.90E-16
YER064C	2:506661	7.65E-10
YIL006W	15:136324	2.33E-06
YDR175C	2:551299	8.38E-09
YDR175C	15:174364	1.07E-06
YMR025W	15:143597	9.44E-08
YDR375C	15:174364	7.58E-07
YER176W	15:150651	2.41E-07
YHR113W	2:537314	2.28E-08
YDL060W	15:150651	1.68E-06
YJL063C	2:555787	1.02E-06
YJL063C	15:174364	9.57E-08
YPR163C	15:143597	2.03E-06
YGL178W	7:187179	2.84E-13
YFL050C	6:33688	2.53E-09
YDR234W	2:477206	5.47E-10
YDR234W	15:174364	1.09E-08

YDL222C	15:174364	1.22E-11
YMR274C	8:111683	1.30E-07
YNL073W	15:174364	1.38E-07
YBR275C	2:750838	5.80E-14
YER093C-A	15:174364	9.98E-09
YKL112W	2:569420	3.69E-06
YLR063W	15:174364	4.18E-06
YIR007W	2:533262	1.95E-07
YIR030C	2:555575	2.49E-09
YIR030C	9:419418	7.86E-11
YKR085C	11:599170	4.86E-10
YGL068W	2:555596	7.93E-08
YGL068W	15:180222	2.63E-06
YCL026C-A	3:75021	2.29E-13
YER170W	15:113251	1.87E-06
YGL259W	2:562415	8.99E-08
YOL120C	15:154309	2.93E-06
YJR099W	2:567221	1.20E-06
YJR099W	10:612602	8.98E-13
YGL254W	7:15891	2.80E-14
YKL168C	11:114412	2.07E-08
YHR051W	15:141627	2.50E-07
YIL073C	13:27644	3.45E-07
YGR197C	15:632882	1.03E-12
YKL022C	11:389068	1.20E-10
YKL022C	15:174364	1.95E-06
YPR181C	2:506661	3.45E-06
YDL031W	15:174364	2.85E-07
YLR360W	2:551299	6.60E-10
YDR337W	15:174364	5.35E-08
YPR125W	2:555575	5.67E-08
YPR125W	14:486861	2.04E-09
YML054C	15:150651	4.34E-09
YIL038C	12:672779	1.32E-06
YIL038C	15:170945	9.09E-07
YJR134C	12:668249	9.56E-07
YPL107W	2:551299	3.08E-06
YPL107W	16:500342	9.53E-07
YKL103C	2:537314	7.72E-09
YLR342W-A	12:815480	6.52E-17
YGL111W	15:174364	1.33E-07
YJL162C	10:151190	3.25E-06
YGL091C	12:681096	5.13E-07
YLR249W	15:154309	9.43E-08
YHR072W	12:662627	1.90E-14
YBR023C	2:537314	1.36E-09
YBR023C	15:143597	1.81E-07

YLR224W	15:136324	8.18E-08
YEL038W	5:79647	2.02E-16
YMR202W	12:662627	1.52E-15
YDR200C	12:662627	1.65E-06
YNL217W	13:46084	3.47E-08
YNL217W	15:174364	3.81E-08
YKL121W	15:174364	2.49E-08
YOL020W	2:533262	1.43E-09
YCR026C	3:175808	1.08E-08
YOR155C	3:105042	7.17E-06
YKL165C	8:111690	1.16E-05
YLR306W	12:705100	1.52E-05
YOR347C	15:170945	1.06E-06
YOL064C	13:46084	1.25E-08
YDR349C	15:174364	5.60E-09
YGL096W	15:136327	8.08E-07
YDL104C	4:273846	1.11E-12
YGR036C	7:543259	1.60E-07
YDL139C	4:211612	6.29E-15
YOR022C	15:392765	1.02E-11
YPL066W	2:620056	4.66E-07
YPR005C	2:537314	1.56E-06
YKR049C	15:174364	1.64E-09
YKR043C	15:174364	9.25E-11
YDR541C	4:1525327	5.78E-10
YGR053C	15:174364	4.71E-08
YDR531W	4:1495582	1.32E-12
YER168C	15:143597	2.96E-07
YDR347W	15:113254	1.52E-07
YOR246C	2:567221	3.44E-06
YOR246C	15:143597	7.54E-09
YLR222C	15:154309	3.92E-07
YML075C	12:662627	2.07E-15
YER006W	15:150651	1.68E-06
YFL020C	1:154328	1.52E-09
YCL059C	15:174364	8.18E-07
YGR166W	15:174364	5.12E-09
YDL046W	15:174364	4.23E-07
YLR267W	12:956534	8.16E-06
YLR267W	13:27644	1.88E-06
YOL154W	15:10427	7.87E-17
YDR534C	13:910741	3.06E-06
YLR040C	3:201166	8.94E-17
YMR037C	15:113260	4.12E-06
YBR121C	15:174364	4.40E-07
YLR457C	2:533262	2.56E-06
YHR085W	15:174364	2.10E-08

YHR062C	15:174364	1.55E-08
YHR139C	15:150651	1.56E-06
YLR231C	12:644136	6.62E-13
YOR047C	4:201395	9.24E-07
YOR047C	15:384923	9.62E-12
YDR370C	2:565216	2.18E-06
YLR437C	15:154309	3.10E-06
YOR353C	15:143597	2.04E-07
YDR490C	15:174364	4.15E-08
YLR214W	12:635380	6.58E-08
YFR039C	15:174364	2.52E-06
YBR151W	15:632882	8.62E-11
YOL077C	15:174364	4.90E-09
YPL019C	13:27644	1.89E-15
YPR140W	15:170945	3.29E-08
YDR502C	12:662627	1.42E-14
YKR104W	11:656099	5.74E-17
YDL025C	15:174364	2.02E-06
YHR063C	3:91287	3.24E-06
YKR098C	13:49903	3.29E-07
YOR389W	13:910381	9.24E-10
YOR389W	15:1065719	3.64E-10
YER136W	12:634226	2.10E-06
YPL026C	2:562415	7.69E-06
YPL026C	16:500348	4.40E-07
YGR149W	2:555778	3.13E-06
YDR043C	2:427677	1.24E-07
YGL171W	7:187567	1.62E-07
YLR376C	12:872448	1.58E-14
YBR079C	2:567221	2.86E-06
YDL089W	4:289639	2.18E-15
YGR208W	7:913065	2.45E-11
YGR208W	15:174364	2.81E-06
YBR131W	15:174364	9.97E-08
YLR234W	8:111682	1.94E-06
YLR234W	12:635380	2.56E-10
YPR010C	15:143597	9.24E-08
YDR292C	12:705100	1.19E-06
YDR292C	15:170945	2.67E-07
YMR010W	15:174364	1.04E-07
YOR238W	15:804686	8.19E-12
YDR528W	15:141627	5.16E-07
YNL111C	12:662627	1.03E-12
YMR078C	2:530481	4.63E-07
YPR043W	15:170945	2.09E-07
YCR090C	15:136324	5.59E-06
YNL152W	2:517365	1.07E-06

YGL055W	7:403626	1.90E-09
YGL055W	12:662627	4.17E-10
YGR170W	13:100048	1.66E-07
YMR225C	2:555787	7.27E-06
YMR225C	15:180222	1.69E-09
YER089C	2:530481	8.25E-08
YOR349W	15:144659	1.79E-07
YML131W	13:124876	3.70E-06
YOL144W	15:44484	9.08E-07
YAR027W	1:185122	1.04E-15
YNL149C	15:136327	1.60E-06
YLR239C	15:174364	2.52E-06
YNL161W	2:569420	7.79E-07
YNL161W	15:170945	6.66E-07
YPL132W	15:174364	8.38E-11
YER088C	15:174364	5.62E-11
YER088C	16:511406	1.01E-05
YCR093W	3:301446	1.51E-07
YJL139C	10:151310	8.72E-16
YAR031W	1:185122	1.04E-12
YPL193W	12:642137	2.87E-06
YER101C	15:154177	9.90E-09
YNL242W	14:191243	2.92E-14
YNL113W	15:174364	6.06E-08
YDR395W	15:174364	3.76E-07
YAL064W-B	1:10152	1.62E-16
YDR520C	12:677957	1.50E-06
YOL104C	15:132423	5.29E-16
YIR031C	2:551299	9.55E-11
YIR031C	9:419418	5.36E-15
YDR067C	4:582121	5.83E-13
YDR067C	14:402312	2.87E-08
YAR002C-A	15:144659	1.02E-05
YDR524C	4:1471859	9.99E-08
YBL067C	12:662627	9.06E-09
YBL067C	16:500348	3.49E-06
YDR213W	12:662627	7.57E-09
YGR089W	12:659357	1.21E-08
YDR036C	4:509817	1.74E-09
YKL161C	15:143597	3.48E-07
YMR179W	8:111683	1.15E-07
YDR297W	12:662627	6.14E-12
YOR298C-A	14:449639	6.40E-12
YIL112W	2:506661	7.46E-06
YIR004W	9:369587	2.61E-08
YPR162C	2:555596	6.18E-07
YDR453C	15:174364	2.98E-09

YGR295C	7:1081945	7.64E-17
YOR213C	13:69122	2.37E-06
YOR213C	14:449639	5.76E-10
YDR272W	2:519049	1.87E-06
YDR272W	4:963769	1.19E-12
YDR273W	4:975086	6.83E-11
YPR191W	15:136327	3.28E-07
YJR046W	7:375499	2.08E-10
YNL212W	8:98513	6.37E-06
YDR237W	2:555596	8.62E-08
YLR410W	13:27644	1.91E-13
YGR223C	7:940716	1.94E-16
YOL133W	8:111683	1.88E-07
YOL133W	15:59733	3.58E-13
YPR151C	12:662627	7.36E-15
YML032C	12:668249	5.96E-06
YML032C	13:180103	7.42E-08
YML096W	13:81250	1.10E-10
YML096W	15:170945	2.67E-07
YDR227W	15:170945	1.10E-06
YMR309C	2:551299	2.41E-06
YDR506C	2:486640	1.02E-06
YFR002W	2:507428	2.28E-06
YDL133W	12:644136	7.50E-07
YIL090W	2:519049	3.93E-06
YLR077W	12:282397	7.20E-07
YHL048C-A	5:15697	7.12E-12
YHL006C	8:98513	9.44E-11
YJR152W	2:551299	7.47E-13
YJR152W	16:500342	9.53E-07
YDL182W	2:480009	2.55E-11
YER150W	15:174364	2.64E-11
YBR154C	2:555596	7.22E-07
YAR008W	13:28694	6.17E-07
YER066W	15:143597	1.19E-06
YGL190C	7:141949	1.12E-07
YDL243C	6:28029	1.32E-15
YMR180C	2:555596	5.57E-06
YDL230W	2:569420	1.11E-06
YDL230W	4:54225	1.90E-07
YAL053W	13:46084	3.52E-07
YAL053W	15:154177	3.35E-07
YLR243W	12:634226	3.10E-07
YLR243W	15:174364	2.02E-06
YJR010C-A	10:461201	3.05E-15
YDR186C	12:662627	1.61E-08
YGR084C	2:555575	5.39E-09

YGR084C	15:113251	5.86E-06
YNL270C	2:477206	2.45E-08
YJL208C	15:150651	1.33E-06
YKR052C	13:922256	6.74E-06
YFL004W	13:27644	1.51E-14
YNL240C	2:555575	9.45E-06
YBR175W	2:562415	4.18E-07
YBR175W	13:46084	4.69E-06
YBR175W	15:174364	9.17E-07
YCL027W	8:111683	8.33E-14
YER024W	12:683463	2.86E-06
YER163C	5:504714	4.33E-12
YOR101W	15:515923	8.87E-14
YLR244C	12:635380	7.18E-15
YNR037C	2:555596	6.42E-06
YNR037C	14:694765	1.66E-06
YNR037C	15:180180	2.67E-07
YOL047C	2:562415	1.72E-11
YLR172C	15:174364	1.50E-07
YDL130W-A	4:217402	4.46E-06
YDL130W-A	15:174364	1.42E-08
YBR165W	2:565216	2.05E-10
YGR211W	7:916675	8.95E-11
YLR356W	15:174364	9.18E-08
YER054C	15:174364	3.14E-10
YOL022C	15:174364	5.86E-09
YKL174C	15:170945	1.06E-06
YLR049C	12:247886	9.76E-16
YNL045W	13:46084	1.03E-06
YNL045W	15:174364	2.16E-09
YHR067W	2:517123	3.57E-06
YPR118W	15:174364	2.49E-08
YCR057C	15:174364	8.81E-08
YHR143W-A	15:154309	1.43E-07
YML025C	2:548401	3.75E-08
YML025C	15:170945	2.01E-07
YOL043C	15:248746	1.29E-15
YLR430W	12:990751	2.20E-09
YIL091C	15:174364	2.28E-08
YKL186C	11:103658	4.01E-12
YEL047C	12:662627	5.20E-08
YJL024C	10:398821	1.94E-07
YML057W	13:159533	1.36E-10
YPR020W	16:542295	9.23E-09
YNL252C	2:551299	2.51E-06
YNL252C	15:174364	8.18E-07
YAL005C	1:136161	5.13E-09

YAL005C	15:170945	3.13E-07
YFL017C	15:143597	2.13E-07
YAL029C	15:106164	1.34E-07
YGL063W	7:375499	3.27E-11
YMR125W	8:167504	5.02E-06
YJL131C	15:174364	4.90E-09
YPL092W	16:368296	8.98E-08
YPR165W	2:565216	3.18E-06
YGL066W	1:187544	2.61E-06
YGL066W	7:375499	3.04E-12
YNL160W	15:174364	3.26E-09
YKL072W	15:174364	2.16E-09
YGL062W	15:89217	6.52E-07
YMR116C	15:174364	4.95E-07
YJL164C	15:170945	4.66E-09
YLR204W	15:170945	1.71E-08
YPR141C	2:565216	2.18E-06
YDR147W	12:705100	2.63E-06
YGR239C	12:662627	8.59E-07
YER073W	3:91305	1.04E-12
YMR196W	15:174364	4.39E-10
YJL144W	7:375499	8.61E-14
YBL105C	6.628472222	2.83E-08
YBR111C	15:154309	2.99E-06
YGR031W	15:136324	2.54E-09
YKL124W	11:212278	8.93E-07
YKL124W	15:136324	1.46E-07
YDL109C	4:263770	1.55E-06
YPR111W	2:569420	2.73E-09
YPR060C	15:174364	1.58E-08
YHR209W	9:98955	7.05E-06
YIR012W	15:174364	2.10E-08
YMR055C	13:379981	5.21E-11
YNL309W	8:111679	9.97E-07
YGR252W	7:995892	1.78E-07
YKL084W	15:174364	7.16E-08
YMR170C	2:555596	1.89E-09
YGR201C	15:174364	3.14E-10
YPL043W	15:154309	8.27E-07
YGL185C	2:530481	9.37E-08
YOL053W	15:220337	7.03E-10
YOR336W	15:141627	4.90E-06
YNR050C	2:477206	8.88E-13
YLR270W	15:174364	2.24E-10
YDR346C	12:644136	9.64E-09
YIL070C	2:555575	1.80E-07
YIL070C	15:113251	4.18E-07

YJR025C	10:472147	2.50E-14
YKL091C	15:174364	2.13E-10
YKR016W	15:154309	1.31E-07
YKL185W	2:555596	5.42E-12
YOR128C	15:563943	6.20E-09
YDL235C	15:174364	1.99E-07
YGR233C	13:27644	1.15E-15
YGR233C	15:174364	1.39E-06
YNL101W	2:553812	7.77E-07
YEL011W	15:170945	4.66E-09
YJR078W	15:850119	9.12E-07
YFL017W-A	6:100521	9.47E-11
YFL061W	10:703868	1.91E-06
YLR368W	12:851826	1.38E-09
YDR418W	15:154309	5.35E-06
YHL042W	4.390972222	3.00E-06
YJR138W	2:555596	1.25E-07
YJR138W	16:500342	1.50E-06
YCL063W	14:449639	1.26E-10
YPL196W	15:174364	1.55E-08
YJR055W	15:174364	3.36E-08
YBL016W	2:199101	7.69E-09
YBL016W	8:111683	4.08E-11
YAL002W	15:174364	9.97E-08
YIL110W	9:133693	5.84E-06
YIL110W	15:174364	2.16E-07
YMR005W	13:268045	5.50E-07
YIL083C	9:196145	3.63E-10
YJR110W	12:662627	2.88E-06
YJR110W	15:106152	2.22E-06
YGR028W	7:553877	2.50E-08
YBR172C	2:555575	1.66E-07
YFL064C	4:1525327	7.54E-07
YBR040W	8:111683	3.36E-12
YNL033W	14:571965	1.55E-13
YDR244W	1:52943	5.43E-08
YKL178C	3:201166	8.94E-17
YGR033C	7:543259	1.03E-08
YGR033C	15:141633	1.23E-08
YHR030C	2:537314	3.86E-08
YHR030C	15:143597	2.07E-08
YGR026W	12:634227	9.78E-07
YLR371W	12:662627	4.77E-07
YDR352W	2:553812	1.13E-05
YDR352W	15:175594	2.11E-07
YDL175C	4:154436	1.72E-06
YKR031C	11:482069	2.70E-13

YGR015C	2:553812	8.12E-06
YOR073W	12:672779	8.35E-07
YDR323C	12:644136	5.44E-08
YKL018C-A	15:143597	1.33E-06
YGR217W	2:555778	3.30E-07
YGR217W	15:143597	1.86E-08
YGR217W	16:511406	1.30E-05
YJR002W	15:174364	2.71E-06
YML030W	15:174364	2.84E-09
YNL051W	14:542648	5.08E-08
YER105C	14:449639	2.08E-11
YOR059C	15:438824	9.19E-13
YKR071C	3:90676	8.47E-12
YKR071C	15:180961	6.70E-07
YLR259C	15:180222	3.86E-07
YCR102C	12:1059818	2.69E-11
YCR102C	15:144659	3.49E-06
YKL148C	11:174008	8.76E-10
YKL148C	15:154309	5.60E-07
YLR319C	2:555575	5.98E-06
YBL037W	2:537314	1.89E-06
YBL037W	13:79760	1.43E-06
YIL104C	7:707950	2.16E-07
YIL104C	9:190866	5.89E-12
YMR063W	13:379981	1.10E-09
YDR178W	15:174364	7.66E-09
YMR208W	12:662627	3.80E-15
YDL066W	2:551299	3.08E-06
YDR202C	15:143597	4.55E-09
YPL212C	15:170945	1.68E-06
YJL137C	13:28622	6.11E-07
YJL137C	15:143597	3.44E-08
YJR039W	15:154309	1.58E-06
YKL163W	11:153463	3.69E-08
YHR201C	8:111683	6.17E-08
YLR179C	12:514835	1.88E-16
YGL136C	7:256953	4.95E-13
YNL002C	15:174364	2.25E-07
YIL014W	9:325320	2.26E-15
YPR122W	8:111680	8.10E-09
YGL225W	12:634227	8.05E-07
YOR019W	15:357194	8.38E-17
YOR250C	15:819015	7.25E-11
YKL067W	15:174364	9.18E-08
YMR244C-A	15:180961	6.38E-09
YIR037W	15:174364	1.69E-07
YEL034W	12:662627	1.30E-15

YGR216C	2:517123	3.85E-06
YGR216C	15:108577	1.37E-06
YPR091C	13:46070	3.07E-06
YPR091C	15:174364	1.14E-08
YJR097W	15:174364	1.62E-08
YMR251W-A	15:143597	3.14E-09
YHL009C	8:93002	2.04E-10
YER061C	2:555596	5.18E-06
YLR225C	5:420595	6.61E-07
YHR018C	2:555575	7.39E-06
YOR038C	15:382531	5.22E-13
YHR207C	15:174364	7.32E-09
YFL054C	6:28029	6.33E-14
YFL054C	10:34098	5.87E-11
YGL160W	12:662627	7.39E-13
YML016C	13:243624	3.24E-13
YHR103W	13:57145	6.43E-07
YGR264C	15:170945	1.64E-08
YPL048W	15:174364	4.34E-06
YOL018C	15:298710	3.16E-11
YBR168W	12:611854	9.81E-06
YJR059W	15:174364	1.38E-07
YER016W	8:111683	1.50E-08
YER016W	13:91085	2.83E-06
YPL263C	12:683463	6.61E-06
YLR056W	12:662627	1.31E-07
YML070W	15:174364	1.56E-07
YGL229C	2:537314	1.99E-11
YLR246W	2:562409	2.65E-06
YLR246W	12:635380	2.87E-15
YDR270W	4:975086	3.65E-09
YBR235W	2:553812	9.39E-10
YGR175C	15:141621	5.95E-08
YKR026C	11:484826	9.92E-15
YKR026C	15:174364	3.81E-08
YAL064C-A	0.377777778	2.39E-13
YJR075W	10:572473	3.32E-13
YLR108C	13:99675	4.44E-07
YLR108C	15:174364	2.43E-06
YJL197W	2:506661	6.45E-06
YJL048C	12:662627	6.49E-14
YLL039C	15:180222	3.42E-06
YJR054W	2:555596	3.27E-08
YOL140W	13:49894	6.98E-08
YOL140W	15:43153	5.88E-08
YEL058W	5:44617	3.35E-08
YEL058W	15:143597	1.96E-06

YDR211W	15:174364	5.36E-09
YML118W	2:570229	2.50E-07
YML118W	13:28622	1.38E-07
YBR097W	2:391856	2.87E-08
YLL029W	12:86369	1.50E-07
YAL031C	8:156412	5.44E-06
YAL023C	2:551299	8.18E-07
YGL257C	7:12939	7.58E-10
YIL034C	15:174364	1.07E-06
YBR228W	2:667083	1.18E-08
YCL035C	15:174364	4.91E-08
YOL006C	15:136327	5.05E-09
YLR175W	15:174364	6.25E-07
YLR251W	15:174364	8.45E-08
YPL221W	2:537314	3.32E-07
YOR054C	15:438824	1.07E-10
YDL181W	15:174364	5.86E-09
YOR035C	2:551299	8.18E-07
YNL224C	13:27644	8.65E-07
YHR054C	8:209167	5.06E-11
YDL093W	12:662627	8.16E-11
YJR124C	15:174364	5.00E-06
YDL225W	2:551299	2.22E-07
YDL225W	13:57145	1.41E-06
YOR195W	15:136327	5.27E-07
YPR128C	1:42633	1.12E-08
YBR102C	15:180180	1.11E-06
YGL101W	12:662627	3.60E-12
YEL007W	9:139462	2.86E-06
YDR096W	15:174364	1.80E-09
YKL062W	15:136327	5.75E-12
YLR342W	12:810883	7.51E-10
YML108W	15:174364	4.33E-08
YAL064W	1:11638	7.28E-13
YAL064W	8:525664	7.76E-06
YPL128C	2:551299	3.30E-07
YER028C	4:217351	8.58E-11
YEL050C	15:174364	4.52E-08
YDR412W	15:170945	1.88E-06
YAL062W	1:10152	1.95E-16
YBR067C	12:662627	1.10E-10
YJR094C	10:602943	7.87E-17
YOR332W	2:537314	5.58E-06
YOR332W	12:634227	1.61E-06
YDR504C	4:1456748	5.24E-10
YDR504C	15:150651	5.20E-07
YAR020C	1:154328	9.43E-11

YGR001C	7:497987	7.33E-11
YGR068C	8:111683	3.43E-07
YNL087W	15:174364	9.98E-09
YCR101C	2:537314	1.19E-06
YPL192C	4:963733	4.58E-07
YPL192C	8:111683	1.17E-10
YBR094W	2:427677	1.64E-12
YGR188C	2:565216	3.69E-06
YLR253W	2:508843	3.19E-06
YLR253W	12:644082	1.67E-06
YPL084W	3:90676	9.95E-09
YDR405W	4:1272737	3.65E-14
YDR405W	15:154309	1.17E-06
YEL065W	13:910741	3.06E-06
YEL046C	15:136327	1.86E-07
YJR149W	10:703868	2.84E-16
YLR073C	12:282091	1.08E-15
YJR112W-A	10:627628	1.63E-13
YLR312C	15:174364	2.09E-06
YEL017W	5:117056	8.25E-10
YIL046W	9:268352	5.62E-15
YIL046W	15:174364	8.18E-07
YBL082C	2:73342	9.96E-10
YPL125W	16:318068	9.74E-11
YIL017C	3:201166	4.06E-14
YBR106W	12:668249	5.93E-07
YBR106W	15:136327	1.02E-06
YFL018C	13:87587	9.05E-07
YFL018C	15:170945	8.75E-07
YHR089C	15:170945	1.19E-06
YBR286W	12:634227	2.04E-09
YJL154C	10:135902	2.60E-14
YIL105C	15:174364	1.92E-08
YNL015W	2:567221	3.86E-09
YNL015W	15:174364	3.08E-08
YJL163C	10:110038	3.25E-07
YJL163C	15:154309	1.11E-07
YPL016W	16:523450	4.10E-15
YKR067W	11:566015	6.93E-08
YKL193C	15:170945	1.71E-08
YML098W	13:110814	3.75E-06
YNL023C	15:136327	3.41E-07
YIL140W	2:537314	1.11E-08
YIL140W	8:111682	9.45E-06
YNL108C	15:174364	1.08E-07
YKL080W	12:672779	3.15E-09
YLR397C	12:909226	1.44E-08

YLR397C	15:174364	1.07E-06
YLR213C	12:611854	3.83E-09
YLR245C	12:635380	7.31E-16
YHR176W	2:584351	4.63E-06
YHR176W	15:144659	2.60E-08
YLR048W	15:154309	1.47E-06
YDR369C	13:99675	3.87E-08
YPR086W	13:28694	3.81E-08
YGR146C	3:79091	5.28E-07
YGR146C	8:111683	2.49E-07
YGR146C	15:141627	2.25E-06
YGL250W	15:141627	2.81E-06
YOL130W	13:33681	2.60E-08
YOL130W	15:143597	9.46E-09
YLR226W	15:180180	5.95E-08
YNL306W	15:154177	2.17E-08
YOR056C	2:555596	8.44E-09
YER037W	5:226503	1.48E-08
YBR262C	15:154177	1.23E-06
YPL170W	12:662627	3.21E-07
YPL170W	13:99675	4.05E-06
YDR477W	2:555787	3.85E-06
YDR477W	15:174364	1.11E-06
YPL083C	16:387239	1.86E-11
YML101C	13:81250	1.48E-06
YGR090W	15:143597	1.39E-06
YOL138C	15:76724	3.00E-07
YIL030C	15:141627	1.27E-06
YKR090W	11:612769	1.73E-13
YOL155C	9:27026	8.59E-12
YOL155C	6.038194444	9.17E-07
YFL013C	6:143977	1.52E-07
YFL013C	12:796771	1.07E-08
YBL034C	15:136327	2.60E-06
YDR169C	4:782114	7.37E-09
YKR063C	15:170945	2.12E-09
YIR033W	1:41483	1.62E-06
YIR033W	12:662627	2.68E-06
YBR173C	2:582419	7.38E-13
YOR120W	15:174364	3.74E-09
YKL172W	15:174364	1.11E-06
YAL028W	12:662627	2.90E-12
YJR008W	10:450212	1.59E-06
YJR008W	15:154309	1.55E-07
YMR319C	13:922258	1.07E-12
YOL017W	8:111686	1.08E-05
YOL017W	15:298710	9.24E-12

YNL249C	2:513408	2.75E-06
YKL204W	2:530481	1.23E-06
YMR053C	13:390357	2.81E-07
YOL128C	2:551299	3.08E-08
YOL128C	12:662627	2.52E-07
YNR012W	15:174364	2.10E-08
YGR288W	7:1074345	1.80E-07
YOR010C	12:662627	3.69E-08
YGL022W	15:113251	1.06E-05
YNR016C	1:42591	1.80E-06
YBL013W	15:144659	2.48E-10
YPR013C	13:99675	9.24E-07
YMR171C	13:597711	1.05E-10
YBL024W	15:170945	8.75E-07
YJL055W	15:174364	4.83E-06
YHR003C	12:677957	6.13E-09
YOR034C	12:662627	9.00E-08
YOR034C	16:500348	6.41E-06
YNR034W	14:689939	1.55E-14
YNR034W	15:113260	3.07E-07
YNR057C	5:272258	3.94E-14
YHR156C	15:174364	6.06E-08
YNR011C	14:571965	6.17E-09
YBR269C	15:174364	1.38E-07
YFR005C	3:90610	6.62E-06
YOR316C-A	2:553812	2.12E-14
YEL052W	15:174364	1.18E-09
YEL055C	15:174364	1.09E-08
YIL046W-A	7:708022	1.02E-06
YIL046W-A	9:268352	3.58E-11
YDL213C	4:70901	2.83E-08
YDL213C	15:174364	9.14E-09
YJR013W	10:492254	1.79E-08
YGR052W	15:174364	3.37E-13
YFR008W	2:551299	5.17E-06
YDR356W	4:1188862	4.60E-13
YJL187C	2:514035	1.67E-06
YJL187C	8:111683	3.27E-08
YPL168W	2:507428	4.95E-07
YJL206C	2:555575	9.45E-06
YJL057C	10:327858	7.71E-15
YGL004C	2:609055	1.37E-07
YGL004C	15:144659	7.24E-09
YDR430C	15:174364	2.26E-09
YPL278C	13:910381	2.99E-12
YOR189W	2:551299	1.60E-06
YMR236W	2:537314	5.46E-08

YMR236W	8:111683	1.47E-07
YLL013C	12:112275	1.21E-15
YPL005W	16:555416	1.95E-08
YER109C	2:548401	4.09E-08
YIL108W	15:143597	7.73E-07
YJR113C	12:668249	9.56E-07
YGR041W	2:555596	1.05E-15
YCR048W	12:668249	3.84E-08
YJL008C	10:451940	5.57E-06
YEL029C	15:170945	3.44E-08
YER154W	2:555596	6.17E-08
YOL019W	2:551299	5.17E-06
YLR383W	12:881579	3.39E-09
YKR027W	3:201166	7.41E-08
YER046W	5:243215	1.81E-12
YIL169C	9:27026	1.27E-11
YIL169C	6.038194444	4.36E-06
YBR003W	15:174364	1.24E-08
YMR304W	15:179289	1.06E-06
YDL117W	2:551299	6.67E-06
YDL117W	15:179289	1.45E-06
YOR306C	15:174364	7.29E-07
YGR281W	12:644136	1.67E-06
YOR040W	15:170945	3.01E-07
YDR165W	15:174364	1.50E-07
YMR229C	15:174364	4.91E-08
YMR058W	13:922258	8.01E-07
YPL105C	14:449639	1.00E-12
YBR081C	15:150651	3.48E-07
YBR183W	12:662627	1.56E-10
YNL132W	15:174364	3.21E-07
YPL214C	2:551299	1.10E-06
YKL130C	11:194611	1.25E-12
YKR034W	2:555575	2.34E-14
YMR264W	15:136327	7.52E-08
YHR037W	8:185012	1.73E-06
YHR037W	16:492351	9.23E-09
YML080W	2:551299	2.05E-06
YER095W	2:592863	7.73E-07
YGR283C	7:1058948	1.67E-06
YGR283C	9:74540	8.68E-06
YNL146C-A	3:201166	8.94E-17
YLL002W	8:111683	2.70E-06
YOR126C	2:567221	4.65E-08
YPL054W	2:553812	2.17E-06
YOR291W	15:174364	9.17E-07
YPR190C	15:143597	4.79E-06

YDR038C	4:527458	4.51E-16
YDR038C	15:106272	5.29E-07
YOR002W	12:713644	6.01E-07
YOR002W	15:141627	4.55E-06
YJL038C	15:846344	1.99E-06
YGR109W-A	7:708028	2.27E-14
YGR109W-A	9:200332	3.13E-14
YLR069C	2:555575	8.98E-08
YLR069C	15:174364	2.19E-08
YNR010W	12:611810	9.26E-07
YNL063W	7:375499	2.68E-09
YNL063W	10:548177	9.96E-10
YLR295C	15:136327	1.41E-08
YLR295C	16:511406	1.50E-07
YDR034C	2:419093	8.81E-07
YFL026W	3:201166	8.94E-17
YDR261W-B	7:553877	6.35E-06
YDR367W	4:1213416	9.08E-16
YGL162W	7:192140	2.75E-13
YGL040C	12:659357	9.66E-09
YPL173W	2:551299	5.33E-07
YPL173W	15:174364	3.09E-07
YGR227W	8:111680	3.69E-07
YBL107C	13:96015	3.12E-06
YML078W	13:115474	7.11E-16
YIR019C	9:387985	5.86E-09
YDL170W	15:180961	2.21E-09
YBL060W	2:555596	1.25E-07
YBL060W	15:174364	2.02E-06
YOR278W	2:555575	7.93E-06
YOR278W	16:500342	1.15E-06
YBR259W	15:174364	2.16E-07
YHR077C	15:136324	7.21E-08
YNR052C	14:449639	4.26E-11
YBR167C	2:567221	1.64E-10
YJL099W	2:567221	4.00E-07
YGL164C	2:537314	5.34E-09
YOR292C	15:170945	3.01E-07
YPR047W	15:113251	2.40E-06
YGL212W	7:92896	2.43E-16
YER079W	13:49894	2.86E-06
YER079W	15:174364	2.09E-06
YHL022C	8:56246	4.56E-12
YER180C	5:549142	8.42E-16
YIR002C	9:362631	3.91E-09
YIR002C	13:124876	1.85E-06
YDR130C	13:28622	1.27E-07

YKL209C	3:201166	8.94E-17
YGR008C	15:174364	1.24E-08
YMR144W	8:111682	1.64E-05
YHR001W-A	15:141627	1.66E-06
YDR060W	15:170945	2.43E-08
YIL079C	15:174364	4.07E-07
YMR140W	13:507305	6.87E-07
YMR140W	15:174364	2.34E-06
YOL069W	13:49903	1.38E-07
YPL118W	15:154177	7.12E-08
YOR384W	13:410287	4.34E-09
YDR341C	15:193911	5.48E-07
YBL015W	15:174364	2.91E-06
YBR198C	2:562415	5.77E-07
YHL040C	13:910741	5.02E-06
YKL212W	15:154177	4.66E-08
YOR363C	1:42633	1.60E-06
YGL163C	7:128898	4.11E-07
YJL014W	15:154177	4.66E-08
YJR004C	3:201166	8.94E-17
YPR134W	2:551299	1.53E-06
YPR110C	15:174364	4.15E-08
YLR014C	15:150651	6.35E-07
YHL012W	8:95289	2.45E-12
YHR214C-E	13:27644	4.02E-10
YEL026W	15:174364	6.87E-08
YJR017C	10:450212	1.18E-06
YLR205C	12:662627	4.28E-15
YKL024C	15:143597	5.75E-06
YPR070W	12:668249	3.68E-06
YPL103C	16:252119	4.44E-08
YEL063C	2:553812	4.15E-13
YBR057C	8:111683	3.41E-08
YFL041W	6:48218	2.22E-07
YOL003C	14:449639	7.17E-12
YKL004W	12:668249	1.59E-06
YKL004W	15:141627	1.18E-06
YBR037C	15:174364	1.55E-08
YFR044C	13:46084	4.31E-08
YFR044C	15:141633	5.29E-07
YOL041C	15:150651	3.34E-07
YDR032C	15:174364	1.84E-08
YJL115W	10:163850	2.02E-06
YER165W	15:143597	2.03E-06
YOL027C	2:582419	2.33E-06
YFR011C	6:168354	1.30E-10
YFR011C	15:154177	4.04E-08

YNL142W	2:555596	2.58E-13
YKL100C	15:174364	1.04E-08
YPL024W	16:500348	7.97E-11
YLL066W-B	4:1510883	4.89E-07
YLL066W-B	12:1056097	4.90E-08
YKL201C	11:47707	2.27E-10
YIL117C	2:533262	5.65E-11
YIL117C	15:143597	2.84E-07
YJL072C	5:251271	1.04E-06
YML087C	13:99675	1.80E-10
YLR382C	2:530481	5.65E-07
YLR382C	15:174364	2.95E-08
YGL073W	3:90610	3.92E-06
YIL164C	2:551299	8.18E-07
YIL164C	9:33795	1.96E-16
YDR398W	15:154309	8.80E-06
YOR221C	13:49894	5.14E-07
YAR007C	1:136161	3.28E-12
YAR007C	13:27644	3.99E-06
YFR017C	15:174364	8.14E-10
YER182W	15:174364	4.61E-10
YER014W	12:662627	2.16E-13
YNL298W	2:537314	2.18E-08
YLR367W	4:1213416	3.36E-07
YML129C	2:551299	1.10E-06
YML129C	15:174364	2.60E-09
YPL183W-A	15:174364	3.66E-08
YPL106C	16:368086	2.31E-14
YEL064C	2:551299	1.66E-08
YLR094C	12:327131	1.42E-16
YHR171W	2:555596	7.80E-07
YER162C	15:174364	1.76E-08
YOR086C	15:174364	7.66E-09
YMR185W	15:174364	3.62E-07
YJR119C	15:846344	9.17E-07
YJL044C	10:353027	4.23E-14
YBR004C	2:252538	7.25E-10
YBR004C	15:154309	3.10E-06
YER063W	2:553812	2.13E-08
YOL080C	15:174364	1.18E-09
YJL092W	2:569420	7.20E-07
YBR230C	15:143597	5.47E-09
YJR010W	8:152932	1.58E-07
YJR010W	10:461201	6.05E-15
YOR012W	15:357194	1.67E-16
YOL081W	15:170945	1.05E-09
YLR173W	12:508029	9.93E-12

YOR148C	15:594024	9.73E-09
YNL098C	15:174364	2.21E-09
YOL002C	7:402835	4.33E-10
YDR514C	2:567221	1.11E-06
YGR112W	15:174364	4.68E-09
YMR281W	13:824809	1.55E-12
YOR205C	2:521415	3.54E-08
YCR037C	2:551299	8.92E-06
YCR037C	13:27644	9.38E-13
YCR037C	16:511406	4.73E-07
YDR300C	15:174364	2.16E-09
YMR107W	15:174364	9.97E-08
YJR049C	12:713638	9.87E-07
YJR048W	12:662627	3.03E-13
YBL041W	2:143721	1.80E-14
YBL033C	12:679808	1.52E-06
YGL224C	13:33501	6.26E-12
YAL036C	15:143597	4.97E-06
YOL143C	5:117056	6.68E-16
YDR508C	2:555575	6.17E-08
YDR508C	4:1456748	1.58E-10
YKL096W-A	15:174364	1.51E-10
YGL197W	7:117900	2.41E-12
YJL046W	10:341703	1.64E-08
YPR021C	15:154309	8.80E-06
YBR298C	7:1058950	1.46E-13
YDR313C	15:170945	3.01E-07
YIL074C	13:33501	6.01E-07
YNL175C	14:314883	2.20E-11
YNL175C	15:174364	3.81E-08
YJL141C	15:154309	5.83E-09
YJR060W	10:548177	6.92E-17
YPL240C	7:375499	1.05E-10
YJL212C	2:555787	3.06E-06
YJL212C	10:22315	1.81E-09
YJL212C	12:681096	1.44E-07
YNR013C	14:614342	1.29E-07
YCL001W-A	2:508843	4.84E-07
YGR082W	2:551299	7.98E-09
YGR082W	8:167506	6.89E-06
YGR082W	15:174364	1.22E-07
YGL050W	7:402841	1.83E-06
YDL004W	15:174364	1.28E-07
YDL004W	16:500342	2.17E-06
YOL009C	15:298710	7.55E-08
YNL283C	2:548401	6.61E-10
YPL030W	15:170945	1.21E-09

YOL026C	12:659357	7.36E-07
YBR202W	2:569420	5.47E-07
YMR165C	2:517365	4.31E-06
YMR165C	13:572643	2.75E-07
YDL099W	12:635380	9.98E-07
YDL217C	4:74695	9.28E-11
YDL130W	15:143597	1.68E-06
YBR021W	2:301671	2.48E-10
YDR463W	5:312672	2.61E-08
YCL056C	3:14066	1.86E-13
YBR146W	2:537314	1.92E-10
YBR146W	15:180210	3.70E-07
YKR065C	15:170945	1.64E-08
YEL060C	2:567221	1.29E-05
YEL060C	15:136327	2.80E-06
YGR066C	2:567221	5.32E-06
YHR034C	8:176412	1.51E-09
YHR043C	8:203246	3.77E-14
YOR004W	15:150651	1.24E-06
YLR407W	15:170945	1.24E-06
YPR176C	15:136324	5.20E-06
YJL012C	13:27644	8.38E-16
YGR086C	15:174364	1.84E-11
YBR105C	13:99675	2.18E-06
YMR261C	15:174364	1.80E-09
YJL103C	15:154309	9.14E-09
YLR369W	12:858996	1.66E-12
YIL173W	9:19607	6.79E-10
YIL173W	10:22273	1.80E-14
YPL207W	15:174364	1.93E-10
YLR446W	15:174364	1.63E-07
YBR199W	2:620056	1.86E-12
YER055C	13:46084	1.98E-07
YLR008C	2:555596	6.43E-07
YIR016W	15:174364	4.39E-10
YOL084W	15:144659	2.88E-15
YMR118C	2:562415	1.62E-07
YPL239W	15:174364	1.49E-09
YPL239W	16:100527	2.72E-12
YNL267W	13:81250	7.20E-07
YMR153W	15:179289	1.68E-06
YCL048W	2:562415	9.88E-06
YOR007C	12:635380	3.08E-06
YOR007C	13:28334	7.56E-06
YOR206W	15:174364	1.07E-06
YEL056W	5:46078	1.61E-07
YBL006C	2:216978	1.01E-11

YNL186W	5:194883	2.90E-06
YBR060C	2:388862	2.44E-09
YFR055W	15:179289	1.12E-07
YFR014C	15:174364	1.31E-10
YGR184C	7:861935	2.25E-11
YKL159C	11:146603	6.09E-16
YFL055W	4.333333333	2.85E-09
YFL055W	10:23505	3.18E-15
YHR195W	15:174364	8.45E-08
YIL064W	9:242417	1.16E-11
YNL243W	2:592863	1.73E-07
YHL036W	15:174364	6.87E-08
YLR355C	3:100213	2.21E-10
YLR355C	13:46084	2.21E-10
YDL233W	2:551299	3.47E-06
YDL194W	15:174364	6.01E-07
YHR081W	15:174364	9.17E-07
YOR060C	15:438824	4.05E-13
YIL097W	15:136327	2.70E-06
YBR114W	2:555778	8.75E-07
YJR098C	10:612602	2.04E-13
YNR036C	15:170945	4.64E-08
YNL065W	14:502496	1.24E-14
YGR076C	15:180180	4.57E-08
YDL061C	15:174364	1.95E-06
YBL009W	2:533268	7.36E-08
YNL066W	2:555778	1.34E-15
YMR114C	15:170945	6.53E-08
YKR060W	15:174364	1.62E-08
YBR233W	12:677957	3.76E-06
YLR193C	15:174364	8.11E-08
YJR035W	10:503030	6.52E-17
YPL175W	15:174364	1.31E-10
YML048W	12:644082	1.14E-06
YMR016C	2:427677	2.39E-11
YKL008C	2:582419	4.61E-09
YKL008C	12:634227	6.18E-09
YBR132C	2:499889	3.06E-16
YLR354C	12:852066	1.23E-09
YLR141W	12:423789	4.80E-13
YOR158W	2:551299	3.77E-07
YOR158W	15:174364	1.76E-07
YPL023C	16:511406	6.21E-14
YNR022C	15:180222	4.13E-08
YDL024C	2:562415	7.15E-06
YLR054C	12:246579	3.91E-12
YOR303W	2:551299	2.72E-06

YOR303W	15:154309	1.16E-07
YDL127W	8:111680	1.09E-06
YOL131W	15:170945	1.26E-08
YNL077W	7:375499	1.08E-15
YNL180C	14:281187	1.48E-09
YLL019C	15:174364	4.09E-09
YJR016C	3:81832	2.72E-11
YLR070C	15:143597	2.32E-08
YCL049C	13:54913	7.07E-08
YBR091C	2:555596	3.57E-07
YDR511W	4:1471260	4.63E-07
YDR511W	15:170945	4.86E-07
YLR395C	15:141627	8.28E-07
YOR192C	15:703769	2.10E-11
YNL128W	14:412269	5.81E-07
YOR193W	15:703771	3.10E-15
YIL055C	9:251537	5.69E-10
YKL086W	8:167506	5.98E-06
YIL127C	15:174364	3.48E-07
YLL008W	15:174364	1.19E-08
YJL071W	5:272258	6.20E-12
YPL159C	12:611854	4.41E-07
YGR256W	15:150651	2.19E-06
YNL093W	15:150651	4.55E-09
YJL219W	10:23409	1.56E-12
YLR353W	2:592863	1.34E-10
YLR353W	12:829693	1.06E-10
YLR273C	12:697260	4.91E-08
YJL095W	2:569420	7.90E-06
YJL095W	12:662627	1.21E-06
YNR028W	2:533268	5.26E-06
YNR028W	12:965218	4.34E-06
YPR004C	16:500348	1.87E-06
YGR271W	15:136324	5.59E-06
YJL145W	2:567221	2.77E-08
YHR008C	12:668249	4.78E-08
YIL061C	9:242934	2.11E-14
YDL245C	10:703868	1.21E-06
YGR019W	2:555596	4.81E-10
YIR027C	2:553812	1.03E-11
YIR027C	13:79786	1.37E-06
YJL050W	10:345059	2.06E-12
YMR175W	15:174364	3.98E-08
YOL158C	9:33795	3.41E-07
YOL158C	13:910381	3.35E-06
YOL158C	3.133333333	2.56E-10
YDL205C	4:89821	1.32E-15

YGR055W	2:551299	4.11E-10
YGR055W	5:272258	5.12E-09
YLR002C	15:174364	2.64E-07
YNL268W	2:477206	3.66E-10
YPL124W	8:111682	3.01E-06
YDR098C	15:143597	3.29E-09
YLR357W	2:519049	7.16E-07
YGL226C-A	7:73452	4.21E-14
YDR275W	2:553812	2.08E-07
YDR275W	12:659357	9.50E-12
YIL047C	2:537314	5.79E-06
YIL047C	15:154309	5.01E-08
YMR031C	15:174364	2.81E-12
YLR345W	15:174364	2.27E-12
YCR030C	15:174364	1.30E-08
YCL069W	11:656099	2.61E-14
YGL129C	2:551299	2.89E-07
YJL178C	10:89348	3.46E-14
YDL239C	2:537314	1.16E-10
YDL239C	4:46316	4.99E-06
YOR262W	15:141627	2.30E-07
YDR017C	13:27644	4.30E-06
YOL025W	2:537314	1.06E-06
YNL139C	2:506661	3.32E-06
YPL244C	13:49894	1.35E-06
YMR266W	15:143597	1.22E-05
YMR266W	16:500342	1.01E-05
YPL228W	2:567221	7.35E-06
YPL228W	16:114235	2.03E-07
YGR030C	7:543259	3.06E-07
YOL092W	15:154309	2.02E-15
YBR005W	2:246129	7.28E-07
YBR005W	12:662627	6.43E-08
YOR388C	10:59959	7.61E-10
YNR032W	2:565216	4.61E-06
YNR032W	8:111683	1.15E-07
YDR148C	15:174364	1.36E-08
YNL195C	15:174364	2.26E-09
YAR015W	1:141181	5.84E-10
YJR065C	13:77684	2.50E-06
YDR309C	13:46084	7.25E-09
YPL003W	15:143597	8.37E-07
YPL091W	16:387239	4.30E-12
YMR271C	2:537314	5.58E-06
YER027C	15:76724	1.23E-06
YGR111W	15:174364	1.04E-07
YPL021W	16:542295	7.94E-06

YAR033W	1:185122	1.01E-13
YNL006W	7:375499	7.29E-11
YLR028C	15:141633	1.83E-06
YNL144C	15:174364	4.66E-06
YGR002C	7:459354	2.40E-08
YCR061W	12:659357	2.10E-07
YOL045W	12:662627	3.34E-06
YGR247W	7:995892	1.57E-16
YCL017C	3:81832	4.58E-14
YGL141W	2:537314	1.18E-07
YML028W	12:644136	3.43E-07
YIR035C	15:174364	1.20E-06
YHL034C	15:170945	2.10E-06
YMR290C	15:174364	8.11E-08
YDR221W	15:113254	3.52E-06
YCL057C-A	15:174364	5.57E-08
YGR012W	7:524216	3.50E-15
YGL061C	8:95289	1.67E-08
YMR006C	13:277071	6.87E-14
YIR025W	9:403134	1.01E-12
YFR012W-A	6:168342	2.04E-08
YNL185C	2:555575	2.92E-07
YNL185C	15:174364	1.33E-07
YNL327W	2:562409	1.36E-15
YNL273W	8:111680	3.57E-06
YML024W	13:227254	2.69E-10
YNL209W	15:143597	2.46E-06
YDR339C	15:174364	8.50E-07
YER122C	12:672779	1.79E-08
YLR348C	3:81832	1.77E-14
YLR348C	13:46070	1.73E-06
YDL142C	2:555575	3.36E-06
YER116C	5:395442	2.90E-13
YDR303C	15:113260	2.77E-08
YDL231C	4:46316	8.00E-17
YPL217C	15:174364	2.71E-06
YDR436W	15:174364	8.74E-09
YHL002W	12:611854	4.07E-07
YIR032C	2:555575	2.79E-14
YIR032C	9:419722	3.02E-09
YIR029W	2:551299	2.20E-12
YHR135C	2:537314	1.10E-06
YJL102W	15:174364	1.62E-06
YOR310C	15:174364	1.22E-07
YMR305C	2:533268	3.41E-10
YOL048C	15:143597	2.42E-08
YDR326C	2:565216	1.27E-06

YDR326C	12:662627	1.54E-07
YKL009W	15:174364	1.74E-06
YPL188W	3:201166	2.22E-09
YDR185C	15:143597	3.75E-08
YOL052C-A	15:174364	3.14E-10
YHL026C	8:56246	1.22E-13
YJR126C	12:662627	2.62E-07
YLR392C	15:150651	1.70E-08
YNR041C	12:677957	8.37E-09
YER002W	15:174364	3.34E-07
YJL039C	15:170945	4.15E-07
YGL187C	15:141627	8.28E-07
YGL187C	16:500348	2.09E-06
YDL084W	12:659357	1.54E-06
YOR081C	15:481586	3.71E-13
YER020W	5:196190	7.87E-17
YLR177W	15:174364	1.13E-09
YDR423C	4:1318073	9.50E-16
YNL052W	12:668249	6.42E-07
YNL052W	15:113251	1.08E-06
YBL019W	2:185450	7.42E-11
YBR297W	7:1081945	3.98E-06
YDR131C	2:555575	4.04E-08
YJL161W	15:170945	1.81E-06
YMR110C	12:674651	4.77E-08
YMR110C	15:170945	3.01E-07
YGR171C	2:530481	1.26E-07
YBL035C	15:174364	1.99E-07
YJL181W	15:174364	6.88E-06
YDR089W	4:582121	7.28E-13
YLR065C	12:288943	1.39E-09
YOR154W	15:594024	5.00E-10
YER007W	8:111680	2.67E-06
YNL026W	14:614342	1.68E-09
YJL066C	15:174364	9.82E-10
YLL024C	7:375499	4.14E-09
YDR248C	13:115474	1.73E-07
YDR265W	4:975086	1.24E-12
YBR299W	7:1081945	9.44E-10
YMR250W	15:174364	6.13E-10
YHR213W-A	6:28029	3.89E-11
YLR033W	15:174364	2.34E-07
YPR201W	11:656099	2.53E-10
YPR201W	16:932538	1.38E-08
YKL099C	15:136327	4.50E-06
YMR214W	15:150651	5.97E-06
YNR033W	15:174364	1.30E-08

YJL183W	2:569420	3.43E-06
YJL183W	10:81875	3.95E-11
YOR110W	8:185882	1.13E-06
YOL108C	15:108577	5.66E-06
YPL277C	13:922258	2.38E-14
YPL277C	15:1065809	2.97E-10
YDR073W	4:582121	4.68E-09
YPL249C-A	16:109106	2.18E-09
YNR019W	12:662627	3.57E-15
YGR062C	15:174364	2.74E-07
YGR248W	7:974625	9.03E-08
YGR248W	15:174364	1.76E-08
YLR196W	15:154309	1.36E-06
YER019W	2:555596	1.60E-07
YER019W	5:194873	3.99E-06
YHR117W	13:129925	7.91E-08
YOL060C	15:220337	3.88E-13
YGL018C	15:174364	2.97E-07
YNL281W	7:375499	6.61E-07
YPL031C	16:492351	1.97E-09
YLR034C	15:141627	3.52E-06
YLL026W	15:174364	3.57E-09
YFR007W	15:174364	3.22E-08
YGR126W	7:795132	2.98E-08
YNL201C	8:72233	9.36E-07
YNL201C	14:254154	4.61E-09
YBR281C	2:769107	1.88E-10
YHR202W	2:555596	9.64E-09
YKR070W	11:574956	2.33E-11
YOR313C	13:54913	6.79E-07
YGR286C	1:42639	3.71E-06
YGR286C	5:272258	3.46E-10
YCL047C	5:422612	9.31E-07
YGL173C	14:449639	4.07E-15
YLR057W	12:254693	2.42E-09
YIL154C	12:662627	4.07E-07
YNL200C	14:254155	2.44E-07
YNL200C	15:174364	1.72E-09
YKL077W	12:635380	9.78E-07
YMR194W	2:567221	5.93E-06
YOL146W	15:47951	5.54E-13
YKL126W	11:211595	4.52E-10
YMR182W-A	2:555787	9.15E-09
YMR182W-A	4:74695	6.91E-06
YMR182W-A	15:143597	3.77E-07
YKR036C	11:510933	1.05E-15
YOR354C	2:555787	2.65E-07

YOR354C	15:170945	1.39E-07
YNL100W	15:150651	4.15E-09
YHR020W	15:170945	3.59E-08
YLR023C	15:141633	3.67E-07
YDL160C	14:449639	2.43E-14
YBR171W	2:555575	2.03E-08
YGL049C	7:402851	1.82E-10
YBR103W	2:477206	4.07E-12
YBR196C-B	2:608310	1.33E-16
YPL108W	15:174364	7.32E-09
YKR046C	12:662627	8.22E-16
YJL026W	12:508029	1.32E-12
YGL207W	7:111785	6.12E-13
YOR066W	13:49894	8.61E-08
YPL015C	15:174364	1.11E-06
YIL093C	2:555575	1.25E-07
YIL093C	15:180210	2.44E-06
YER045C	15:143597	1.09E-07
YML076C	13:115474	1.73E-13
YNL308C	15:150651	2.36E-06
YDR533C	15:144659	1.02E-15
YLR168C	12:505763	9.28E-11
YLR168C	15:113251	1.18E-06
YDL214C	4:62325	1.23E-16
YDL214C	15:174364	4.15E-08
YPR082C	15:589013	1.72E-06
YKR080W	11:586769	5.21E-11
YBR104W	2:486640	6.79E-08
YBR104W	15:170945	1.19E-06
YOL136C	9:139464	3.23E-07
YOL136C	13:149075	1.65E-06
YDL234C	4:46466	3.31E-07
YIR018W	9:387985	9.82E-10
YGR162W	15:143597	6.87E-07
YNL124W	14:402312	3.26E-07
YIL116W	9:141014	9.01E-15
YML100W	15:174364	1.75E-10
YPL012W	15:150651	3.62E-07
YDR286C	15:143597	1.02E-06
YER129W	12:693790	6.57E-07
YGR185C	15:174364	5.57E-06
YIL037C	8:111686	1.47E-12
YPL148C	16:266023	4.15E-11
YGR043C	15:143597	1.05E-11
YJR108W	10:627628	4.90E-17
YDR121W	4:744330	1.87E-07
YOR184W	15:174364	1.03E-06

YNR007C	12:672779	3.27E-08
YNR007C	15:141627	1.09E-06
YCR020C	2:551299	7.15E-08
YCR020C	8:167504	3.90E-06
YLR442C	8:111683	4.83E-06
YHR091C	2:533268	7.16E-07
YOR386W	2:582419	4.68E-07
YOR386W	15:143597	1.96E-07
YLR277C	15:170945	5.69E-07
YLR381W	12:881579	1.15E-07
YLR194C	15:174364	9.17E-07
YIL011W	5:350744	6.60E-12
YOL166W-A	15:16691	3.56E-09
YIL033C	15:170945	4.64E-10
YPL164C	2:579459	4.33E-06
YJL159W	2:555596	4.02E-07
YJL159W	10:122312	1.49E-08
YML066C	13:129925	2.58E-15
YPL011C	2:567221	9.43E-06
YPL011C	15:141621	1.23E-07
YPL011C	16:542307	6.80E-09
YNL322C	14:33361	1.13E-11
YDL120W	4:246738	1.31E-10
YER091C	5:272258	8.74E-09
YMR148W	15:136327	1.68E-09
YKR017C	11:482069	4.00E-13
YLR215C	12:582499	2.91E-13
YMR109W	13:481542	1.71E-14
YDL005C	4:465157	4.17E-07
YNL284C	2:555787	3.30E-06
YNL284C	15:154177	1.34E-06
YJL172W	2:551299	1.67E-06
YJL172W	10:101193	1.88E-14
YGR222W	7:952065	1.40E-09
YOR327C	10:548177	2.20E-11
YJL045W	10:380085	7.04E-09
YLR102C	15:136327	1.03E-08
YOL166W-A	15:43051	5.84E-15
YPR085C	16:711614	8.81E-16
YJL023C	2:537314	2.28E-06
YJL023C	10:453011	3.88E-06
YJR104C	12:662627	6.14E-10
YCR071C	2:551299	1.55E-07
YCR071C	8:167506	1.16E-05
YCR071C	15:180222	2.17E-07
YAR028W	1:185122	1.04E-15
YOR138C	15:594024	1.36E-11

YGL078C	15:150651	6.87E-07
YHL038C	2:562415	4.53E-07
YOR107W	15:136327	2.58E-07
YCR016W	15:170945	6.40E-07
YPR023C	11:219950	3.31E-06
YPR023C	16:600664	2.38E-11
YLR406C-A	12:919638	1.98E-11
YDL207W	4:85846	1.95E-12
YBR214W	15:174364	4.33E-08
YGR158C	7:805409	8.27E-10
YIL142W	9:79823	2.21E-10
YLR330W	12:796771	9.54E-07
YCR053W	15:174364	4.18E-06
YPL183C	15:170945	7.20E-07
YGL006W-A	7:490784	1.13E-11
YDL247W	10:715254	7.09E-11
YAR064W	6:43666	9.55E-08
YLR183C	8:111683	5.29E-07
YJL116C	2:548401	8.61E-07
YJL116C	10:214773	8.36E-09
YDL150W	2:551299	5.17E-06
YML056C	15:154309	1.11E-07
YPR144C	2:551299	7.84E-07
YNL264C	12:635380	1.14E-07
YLL011W	15:170945	4.86E-07
YGR187C	15:174364	1.03E-09
YKL105C	12:634227	1.16E-07
YKL183W	11:98330	1.37E-08
YPL041C	12:674651	1.82E-06
YHR092C	15:143597	2.85E-06
YML128C	15:174364	3.63E-10
YEL032W	4:1407833	3.21E-09
YEL032W	13:46084	1.31E-07
YLL018C-A	15:174364	2.92E-11
YNL050C	2:551299	9.28E-07
YNL050C	14:525061	3.64E-08
YJR003C	15:174364	4.39E-10
YBR159W	2:565216	7.05E-07
YHR110W	15:174364	2.53E-07
YLR157W-C	12:472165	8.38E-17
YDR004W	4:450230	1.42E-06
YPR061C	15:136327	7.96E-06
YBR115C	2:477206	4.19E-14
YPL231W	1:41483	2.64E-07
YPL231W	12:659357	4.94E-07
YOR127W	2:533268	7.59E-09
YOR127W	15:143597	3.99E-06

YGR128C	15:174364	7.32E-09
YGR095C	15:143597	1.24E-07
YCR018C	3:105042	3.69E-12
YGL019W	7:460945	3.26E-08
YPL187W	3:201166	8.94E-17
YOR173W	15:174364	2.72E-10
YPL201C	16:125271	8.33E-11
YMR288W	8:111680	1.19E-10
YNL072W	8:111683	2.92E-07
YER010C	13:49894	2.22E-07
YDR441C	4:1344670	1.64E-12
YCL051W	15:174364	2.85E-07
YHR058C	8:221933	3.20E-15
YGR125W	2:551299	6.14E-11
YCR089W	8:111683	1.21E-12
YOR207C	15:174364	2.38E-08
YDL203C	4:96271	8.02E-07
YDL203C	15:175594	1.28E-06
YNL148C	13:33681	3.08E-08
YDR507C	2:565216	6.36E-11
YDR507C	8:111683	8.81E-06
YBR155W	15:174364	2.49E-08
YNL282W	2:551299	2.43E-08
YPL277C	13:910381	1.23E-12
YJR005C-A	10:453011	3.79E-07
YPL211W	15:174364	9.90E-07
YNL081C	15:180222	1.86E-09
YMR223W	12:662627	3.47E-07
YDR351W	15:143597	1.14E-07
YMR113W	12:679808	7.78E-07
YMR113W	13:494170	1.19E-12
YGR040W	2:567221	8.52E-13
YGR040W	7:557230	3.19E-09
YHR127W	8:111679	3.76E-06
YGR003W	7:498659	3.91E-11
YDR005C	4:433589	4.78E-12
YJL191W	3:175802	1.73E-08
YJL191W	5:196196	6.26E-07
YKL170W	2:553812	1.17E-05
YKL170W	11:98330	2.96E-09
YKL170W	15:154177	7.37E-07
YER153C	2:555596	1.74E-13
YIL089W	7:708252	6.49E-09
YIL089W	9:200332	6.52E-17
YFL036W	2:555787	4.31E-06
YFL036W	6:70818	1.94E-07
YFL036W	15:174364	1.28E-07

YPL257W	16:70847	2.21E-14
YDR092W	4:579813	8.59E-09
YJR131W	10:646911	1.45E-11
YJL213W	10:34098	5.23E-16
YHR183W	13:77684	3.58E-10
YNL279W	8:111683	1.50E-10
YBL052C	2:567221	1.54E-05
YBL052C	8:95469	4.92E-08
YBR063C	2:364851	1.09E-10
YAL008W	15:174364	1.50E-06
YPL266W	15:174364	8.11E-08
YHR068W	15:154309	2.06E-07
YFR041C	6:239482	4.69E-10
YMR155W	2:555596	1.20E-07
YMR155W	16:500342	2.71E-06
YHR015W	8:137221	1.39E-14
YBR179C	2:565216	2.36E-11
YGL053W	7:403626	4.90E-17
YBL045C	15:154309	1.26E-06
YLR188W	16:387239	1.50E-09
YGL116W	8:111690	1.14E-07
YPR009W	16:547618	4.18E-09
YDR462W	2:555575	2.42E-08
YDR462W	15:170945	1.57E-07
YPL230W	2:562415	3.69E-06
YPL230W	15:174364	5.36E-09
YLR153C	12:662627	2.20E-15
YGR163W	3:100213	1.22E-06
YLR203C	15:174364	7.40E-10
YGR156W	15:154177	6.47E-07
YNL326C	8:80068	9.36E-08
YDR321W	4:1109729	6.76E-10
YBL064C	15:170945	9.14E-08
YHR187W	15:174364	1.17E-07
YKL207W	14:449639	7.45E-10
YDL202W	2:555575	3.71E-07
YDL202W	15:174364	3.13E-06
YJR093C	10:604478	5.06E-11
YMR038C	12:668249	1.35E-11
YOR233W	15:174364	6.87E-08
YPL171C	2:562415	3.97E-06
YPL171C	15:143597	2.86E-09
YJL174W	2:507428	9.86E-07
YDL169C	2:555596	2.39E-07
YMR255W	2:551299	1.24E-08
YJR070C	15:174364	2.91E-06
YLR209C	2:565216	5.60E-08

YJL020C	10:400191	4.14E-10
YJL020C	13:77684	4.41E-07
YDL226C	4:46466	1.47E-15
YGL120C	15:174364	4.76E-07
YOR283W	15:842027	4.63E-16
YJL132W	2:562415	4.78E-06
YJL132W	10:159479	1.96E-14
YGL223C	7:85112	2.36E-09
YGL223C	8:95469	2.63E-06
YGL223C	13:28622	2.90E-06
YPR115W	8:111683	2.64E-08
YPR115W	15:170945	1.15E-06
YJL060W	2:555575	5.37E-06
YML116W	13:46084	5.33E-11
YFL027C	3:201166	1.08E-16
YIL172C	9:33795	3.57E-06
YIL172C	13:100048	1.22E-06
YGR271C-A	15:136327	2.18E-08
YBR042C	12:662627	9.39E-08
YFR013W	6:174552	2.45E-08
YFR013W	15:136324	1.23E-06
YKL046C	13:49894	6.82E-06
YKL046C	15:174364	1.22E-07
YBR068C	2:420366	1.86E-08
YBR068C	3:81832	1.65E-13
YIL024C	2:517123	1.49E-08
YHR136C	13:27644	1.47E-15
YDR493W	2:565216	6.41E-06
YDR493W	15:180222	2.95E-06
YHL008C	8:84437	6.74E-13
YML002W	13:273244	4.33E-16
YJL117W	13:27644	4.43E-14
YBR122C	15:113254	4.23E-06
YBL008W	13:28334	4.74E-07
YNL286W	14:96321	6.15E-10
YLR229C	2:537314	4.93E-10
YBR026C	2:301671	1.21E-15
YKL109W	15:170945	1.93E-09
YKL109W	16:500348	3.37E-06
YHR033W	8:167506	9.24E-17
YOR177C	15:683415	1.65E-07
YOR334W	12:674651	6.63E-08
YDR500C	15:154309	5.75E-06
YLR301W	15:143597	5.86E-07
YDL051W	15:174364	3.08E-08
YMR198W	8:111683	3.89E-06
YMR198W	13:46084	7.24E-07

YEL022W	5:117046	6.45E-06
YPL233W	16:105278	6.93E-07
YPL028W	12:662627	2.87E-14
YDL208W	15:174364	5.86E-09
YDR047W	15:89217	2.21E-09
YOL135C	12:662627	1.16E-07
YLR438W	13:77684	8.49E-15
YNL164C	2:567221	1.34E-05
YHR007C	8:111683	7.61E-08
YHR007C	15:170945	5.92E-07
YCR004C	13:69114	4.23E-06
YCR004C	15:170945	1.64E-08
YKR051W	12:683463	3.15E-07
YMR065W	8:111683	2.31E-12
YDL003W	8:111682	8.42E-07
YIL014C-A	9:325320	1.15E-16
YLR241W	12:634227	9.37E-08
YDR161W	4:782114	7.22E-11
YDR161W	15:174364	1.50E-07
YLR082C	12:264911	3.51E-09
YOR267C	15:174364	3.50E-08
YGR221C	2:517365	1.72E-06
YCR051W	15:174364	9.57E-08
YDR260C	2:551299	7.20E-07
YLL035W	15:174364	2.38E-08
YER060W-A	13:79786	2.50E-06
YNL146W	3:201166	1.15E-16
YGL262W	7:10158	5.28E-11
YKL050C	2:521415	2.82E-07
YDR097C	15:174364	2.97E-07
YKL137W	11:180221	5.80E-07
YBR182C-A	12:659357	3.37E-07
YJR106W	10:621762	2.52E-07
YNL230C	12:674651	1.48E-06
YGL226W	7:73452	2.42E-10
YGL226W	15:170945	2.27E-07
YDL073W	12:662627	1.13E-05
YDL073W	13:28622	2.50E-06
YDL073W	15:170945	1.63E-07
YDR151C	7:375499	5.59E-11
YDR342C	15:174364	1.75E-10
YOR296W	2:548401	1.34E-11
YBR107C	2:477206	2.15E-14
YMR001C	2:551299	1.78E-07
YNL183C	2:553812	8.22E-11
YGR169C	2:506661	4.82E-06
YEL024W	12:668249	5.00E-09

YEL024W	15:141627	1.13E-06
YCR096C	3:201166	8.94E-17
YJR132W	15:150651	1.09E-07
YLR293C	15:143597	2.03E-06
YLR389C	12:909226	5.39E-14
YNL215W	8:80068	8.21E-06
YBL039C	15:150651	2.65E-06
YPL109C	15:143597	2.84E-07
YFL007W	14:449639	4.28E-14
YLR314C	2:551299	1.97E-06
YOL034W	15:174364	1.33E-07
YPR161C	15:154177	2.76E-08
YJR092W	2:551299	2.68E-10
YJL098W	13:46084	6.43E-07
YJL098W	15:174364	6.75E-07
YPR088C	12:697260	2.61E-06
YBR236C	2:697894	2.65E-14
YGR220C	2:555787	4.18E-07
YGR220C	15:180222	8.61E-08
YLR059C	12:260119	4.52E-10
YHR163W	13:77684	1.33E-06
YER174C	5:538875	1.89E-07
YHR009C	8:137227	1.33E-07
YHR009C	15:174364	2.07E-07
YLL001W	15:174364	4.48E-09
YDL022W	15:174364	1.64E-09
YNL207W	14:246135	2.14E-12
YNL207W	15:174364	3.13E-06
YMR073C	2:565216	1.94E-06
YIL124W	15:174364	9.14E-09
YOR162C	15:632894	4.02E-14
YLR142W	2:567221	4.51E-07
YLR142W	13:49894	1.30E-08
YLR142W	16:500342	8.81E-08
YML120C	15:174364	4.15E-08
YDL183C	15:106266	4.99E-08
YDL019C	15:174364	6.18E-13
YMR308C	15:150651	6.35E-07
YIL153W	9:74540	2.64E-07
YDR194C	2:555596	1.01E-08
YDR264C	4:975086	2.69E-14
YMR018W	3:92013	1.25E-05
YFL048C	15:143597	1.19E-06
YIL146C	2:555596	4.16E-10
YNL003C	12:659357	5.44E-10
YNL103W	15:136327	3.49E-06
YOR163W	13:27644	1.04E-11

YOR005C	15:180180	2.95E-06
YEL076C-A	12:1067122	7.09E-06
YNL117W	2:551299	4.29E-07
YNL117W	14:412269	1.27E-08
YOR243C	15:170945	1.33E-07
YMR300C	13:46084	2.87E-09
YLR288C	12:708594	1.40E-14
YNR068C	2:555596	2.12E-08
YNR068C	13:46084	7.88E-08
YBR006W	2:246129	1.97E-13
YLR361C-A	12:851826	3.05E-14
YPL172C	15:154177	8.03E-07
YPR089W	2:569420	2.65E-06
YBR205W	2:551299	1.47E-06
YBR205W	16:511406	1.25E-05
YLR197W	15:170945	5.06E-07
YBL055C	2:133749	1.43E-08
YMR101C	2:562415	4.02E-07
YOR113W	15:174364	1.08E-07
YLR093C	12:327131	1.04E-14
YAL065C	1:10152	8.29E-14
YIL103W	9:190866	1.60E-06
YOL119C	15:89217	4.74E-10
YBR221W-A	14:412269	3.03E-07
YFL029C	6:74709	1.32E-07
YJL210W	15:143597	8.64E-09
YJL210W	16:500342	3.37E-06
YDL122W	2:555575	1.73E-07
YOR260W	15:174364	3.48E-07
YDL006W	4:465337	1.64E-07
YNL194C	15:174364	3.14E-10
YDL178W	12:662627	5.59E-07
YML071C	15:170945	1.57E-07
YGR065C	5:272255	2.02E-09
YJL035C	10:380085	1.72E-16
YML031W	2:517365	4.05E-07
YKL041W	11:354466	4.20E-15
YPL267W	8:111679	3.63E-07
YNL025C	13:81250	1.59E-06
YGR016W	2:537314	1.28E-06
YGR016W	15:703769	6.32E-06
YHL021C	15:174364	3.91E-07
YMR272C	15:108577	3.72E-12
YKL026C	15:143597	2.73E-09
YHR142W	2:537314	2.04E-13
YHL009W-A	8:80068	5.82E-10
YHL009W-A	16:445372	1.82E-09

YER043C	12:644136	2.73E-09
YPL022W	16:511406	1.13E-11
YBR130C	2:537314	2.82E-07
YIL088C	2:555596	2.60E-09
YAL049C	1:52943	1.07E-15
YER047C	5:244117	2.64E-15
YIL099W	15:143597	1.14E-06
YMR293C	15:154309	5.55E-06
YLR084C	2:537314	8.04E-08
YLR084C	15:141627	1.29E-07
YPL098C	15:89217	6.27E-07
YMR090W	15:174364	3.26E-09
YHL039W	15:174364	6.50E-07
YIL077C	15:174364	8.94E-10
YMR232W	8:111683	3.81E-13
YMR002W	15:154177	1.04E-06
YMR136W	15:143597	2.33E-11
YDR116C	2:555575	2.80E-06
YDR116C	15:154177	7.06E-07
YIL111W	12:681096	1.37E-07
YIL111W	13:99675	4.20E-06
YBR234C	2:697894	1.42E-13
YNL239W	2:555596	2.01E-10
YMR041C	15:143597	1.86E-08
YIL136W	15:174364	3.29E-10
YDL107W	4:273840	5.27E-07
YDR226W	2:569420	8.48E-06
YDR226W	12:679808	2.79E-06
YLR333C	15:174364	2.97E-07
YPL271W	15:174364	3.21E-07
YPL271W	16:500348	3.34E-07
YMR030W-A	2:551299	3.72E-08
YBR166C	2:567221	4.61E-15
YHR047C	3:91977	4.24E-06
YHR047C	13:46084	1.25E-07
YGR058W	12:659357	6.53E-07
YPR159W	2:517365	1.55E-08
YNL034W	14:553129	6.00E-16
YGL140C	2:553812	5.21E-06
YLR412W	12:956534	3.84E-12
YGR169C-A	12:644136	5.67E-08
YBR039W	15:136327	4.50E-06
YNL010W	12:644082	6.40E-10
YGL202W	2:555596	5.77E-06
YGL202W	7:110806	2.10E-10
YPL224C	15:174364	1.24E-06
YFR053C	15:174364	9.55E-09

YNL048W	14:554606	5.57E-09
YMR315W-A	13:910381	4.66E-10
YHR045W	15:170945	2.73E-06
YOR317W	15:150651	8.70E-07
YPL268W	13:27644	1.83E-12
YPL087W	2:507428	4.45E-06
YPL087W	15:174364	9.53E-07
YCL073C	11:656099	5.75E-17
YBL098W	12:681096	2.20E-06
YGL146C	15:174364	1.09E-08
YLR156W	12:472165	2.74E-16
YOR254C	15:143597	2.46E-06
YDR087C	15:174364	1.20E-06
YER110C	15:150651	9.24E-08
YOR136W	15:571103	5.35E-07
YHR042W	8:193175	1.67E-08
YBR189W	2:565216	6.01E-07
YPL160W	15:170945	1.70E-07
YHR150W	8:389050	7.81E-09
YHR100C	15:174364	2.34E-07
YGR038W	7:553877	7.57E-16
YMR070W	12:672779	1.06E-08
YCR033W	12:662627	7.07E-07
YBR001C	2:216978	1.38E-07
YBR001C	15:154309	2.75E-08
YIL056W	15:136324	1.58E-07
YHR190W	12:659357	9.16E-14
YOR358W	15:174364	4.71E-08
YPL163C	2:537314	3.03E-11
YOR149C	15:589013	1.87E-08
YDR510W	4:1474482	8.34E-10
YNR006W	12:611854	1.25E-05
YPL273W	13:922258	1.75E-14
YKL157W	2:533262	2.30E-07
YHR021W-A	8:150330	3.37E-10
YIR008C	9:372093	3.50E-10
YGR013W	2:533262	2.94E-07
YMR289W	13:849969	1.97E-14
YHL011C	15:174364	3.08E-08
YNL046W	2:555787	1.22E-11
YNL046W	14:547071	1.86E-07
YNL145W	3:201166	8.94E-17
YLR409C	15:174364	2.28E-08
YML126C	12:659357	6.29E-16
YHR061C	8:111690	5.30E-08
YJR077C	15:136327	1.24E-08
YPR040W	2:508843	4.64E-07

YPR040W	12:659357	1.39E-07
YGL208W	7:110813	6.87E-13
YGL208W	15:174364	3.62E-06
YOR131C	12:662627	1.65E-06
YOR131C	15:571103	1.00E-11
YCR100C	2:555596	4.49E-06
YGL191W	12:668249	4.51E-10
YGL236C	15:154177	4.25E-06
YBR070C	2:388862	1.09E-08
YBR070C	8:111683	1.55E-06
YGR180C	12:450046	1.84E-08
YAL061W	15:143597	2.84E-07
YLR097C	2:567221	9.51E-07
YKR072C	2:555575	1.43E-05
YDR358W	15:174364	2.43E-07
YJR036C	2:562415	2.35E-06
YJR036C	15:143597	6.35E-07
YLR426W	12:987750	1.17E-15
YKL218C	2:555778	7.38E-10
YKL218C	11:12706	9.33E-07
YMR034C	13:328865	2.75E-14
YOR335C	15:170945	1.10E-06
YPR030W	15:170945	4.08E-08
YIL050W	3:105042	8.30E-08
YIL050W	13:46084	8.82E-07
YDR216W	15:144659	5.60E-11
YDL110C	15:174364	1.24E-09
YLR281C	12:705190	3.48E-09
YBR069C	2:376872	3.79E-12
YKL151C	15:174364	1.36E-08
YDR204W	15:174364	1.72E-09
YML081C-A	15:108577	2.50E-07
YML081C-A	16:511400	4.06E-06
YDR461W	3:201166	8.94E-17
YHR162W	13:79760	8.62E-10
YHR162W	14:449639	2.76E-09
YDR122W	4:733875	3.73E-07
YPL053C	15:174364	1.22E-07
YPL222W	15:174364	4.28E-09
YKR019C	11:468771	4.21E-09
YDR284C	12:662627	8.28E-09
YNL262W	14:449639	2.55E-12
YFL016C	7:375499	6.66E-09
YFL016C	15:108577	8.41E-06
YLR090W	15:113260	3.29E-06
YGR250C	7:995892	2.11E-11
YJL204C	10:59959	5.22E-09

YMR062C	13:404546	2.62E-09
YHR216W	6:28029	3.72E-06
YHR216W	13:180103	7.74E-08
YJL077W-B	2:551299	2.94E-08
YKL090W	11:266017	2.50E-16
YKR030W	12:668249	4.11E-06
YOL101C	1:42591	4.51E-10
YOR348C	12:662627	4.17E-10
YOR348C	15:150651	6.35E-07
YIL158W	2:555778	7.93E-06
YIL158W	9:47053	2.12E-15
YDR409W	2:569420	4.85E-07
YGL067W	7:375499	2.55E-10
YPL247C	15:174364	6.87E-11
YDR256C	15:154309	4.86E-09
YFR026C	6:205881	3.88E-14
YCL001W-B	2:537314	3.06E-07
YDR124W	8:111683	8.62E-08
YKR006C	2:555787	1.02E-06
YKR006C	15:154177	7.12E-08
YAL063C-A	1:10152	3.52E-10
YBR125C	13:49894	4.94E-07
YLR100W	12:662627	1.72E-13
YGL017W	12:662627	6.05E-07
YLR143W	15:174364	2.28E-08
YLR289W	2:555575	1.55E-06
YLR289W	15:154177	1.18E-06
YKL181W	2:555575	1.47E-07
YOR176W	15:174364	2.71E-06
YDR021W	15:174364	3.21E-07
YLL066W-B	4:1511257	1.08E-11
YLL066W-B	12:1056097	8.25E-10
YBL029C-A	15:180961	1.05E-08
YBL099W	15:174364	3.81E-08
YMR323W	15:690367	1.56E-07
YNL042W	14:549682	2.83E-08
YDL036C	15:175594	5.45E-08
YLR372W	15:170945	2.26E-06
YER074W	15:154309	2.78E-06
YFR031C-A	15:170945	2.27E-07
YOL113W	15:108577	4.71E-10
YFL010C	6:134096	6.23E-11
YLL055W	12:26184	1.29E-13
YNL039W	14:591237	5.00E-08
YMR267W	15:170945	5.36E-10
YOR188W	2:537314	4.60E-11
YNL218W	15:180180	8.12E-06

YLR058C	13:79760	7.79E-07
YMR181C	2:506661	3.58E-06
YMR181C	15:174364	2.13E-10
YPR196W	16:932538	5.77E-12
YDR464W	2:565216	7.97E-06
YNL053W	2:567221	1.29E-06
YKL139W	15:174364	1.34E-06
YNL241C	3:92247	9.49E-07
YNL311C	14:33361	2.99E-07
YBL043W	2:142262	4.92E-10
YBL043W	12:659357	4.99E-08
YNL323W	14:33643	1.14E-10
YDR408C	13:46084	3.01E-06
YOR208W	2:506661	3.09E-06
YOR208W	13:46084	1.99E-06
YOR208W	15:143597	3.93E-07
YNL007C	7:375499	7.81E-15
YER017C	15:174364	4.66E-06
YHL033C	15:170945	1.15E-06
YGL209W	2:555575	1.80E-06
YML055W	13:163328	1.46E-13
YDL131W	2:477206	2.89E-09
YDL131W	4:226317	5.64E-10
YKL210W	11:46633	8.65E-07
YCL014W	2:551299	1.43E-08
YHL048W	6.176388889	4.90E-17
YML111W	13:49894	5.74E-13
YMR093W	15:174364	3.48E-07
YKL189W	8:111683	6.71E-10
YBR231C	2:679703	2.40E-11
YJL151C	10:130933	6.10E-09
YJL151C	15:179289	4.31E-07
YBL028C	15:170945	1.95E-06
YAR018C	2:553812	8.76E-07
YNL102W	14:449639	2.87E-12
YDL021W	15:170945	2.23E-08
YDR074W	15:174364	1.51E-10
YNL231C	12:662627	3.34E-06
YDR171W	15:174364	2.98E-09
YOL163W	15:10427	4.75E-17
YBR120C	15:174364	1.24E-06
YGR131W	12:634226	1.55E-06
YDR322W	15:174364	6.50E-07
YKL093W	11:266017	4.84E-08
YKL093W	15:174364	9.90E-07
YDR443C	4:1344670	8.25E-13
YLR332W	2:537314	2.92E-09

YLR332W	15:143597	2.96E-07
YGR093W	15:174364	1.44E-06
YDR019C	13:115474	9.20E-07
YNL317W	14:37071	5.18E-11
YPR133W-A	2:555575	5.67E-08
YDL164C	4:165032	2.46E-09
YKL142W	15:174364	5.36E-09
YBL071C-B	2:519049	2.35E-07
YAL040C	15:174364	3.08E-08
YJR005W	2:537314	8.76E-08
YJR005W	10:450212	1.92E-06
YJR005W	15:143597	1.29E-07
YJR041C	15:174364	2.48E-09
YPL038W-A	16:489143	1.40E-12
YKL144C	15:174364	6.75E-07
YER075C	2:548401	2.32E-08
YOR374W	13:99675	9.99E-11
YOR374W	15:174364	2.38E-08
YGL028C	2:562409	2.36E-16
YGR154C	2:567221	1.11E-06
YGR154C	16:500348	1.29E-06
YOL095C	15:141627	5.47E-11
YNL247W	15:174364	2.43E-07
YBR161W	12:634225	1.15E-07
YPR199C	11:656099	5.75E-17
YKR058W	11:540588	4.40E-12
YKR058W	15:174364	3.81E-08
YER076C	5:321714	8.69E-07
YKR095W-A	3:91287	1.21E-07
YKR095W-A	15:180961	2.01E-07
YML068W	2:555575	8.42E-07
YJL217W	10:23505	1.12E-16
YFL053W	6:28029	7.58E-16
YFL053W	10:22273	4.07E-06
YFL042C	6:48218	4.26E-07
YFL042C	15:154309	3.09E-07
YOL125W	15:174364	7.38E-06
YOL016C	2:569420	9.84E-07
YHL047C	5.218055556	4.75E-17
YGR236C	15:150651	2.32E-08
YPR018W	15:174364	2.43E-07
YPL040C	2:551299	1.15E-06
YPL040C	15:174364	3.41E-09
YFL021W	2:555575	3.55E-12
YER087W	15:174364	7.02E-07
YKL028W	11:394660	1.83E-09
YLR432W	13:184113	1.44E-06

YGL115W	15:154309	3.62E-07
YML130C	7:375499	8.23E-08
YGR229C	2:569420	8.10E-07
YGR229C	7:954692	3.52E-08
YDR487C	4:1474485	2.80E-06
YDL002C	4:463264	4.30E-14
YHR094C	4:217351	9.95E-10
YOR226C	3:81832	3.21E-14
YOR226C	15:174364	1.44E-06
YOL029C	15:174364	5.31E-10
YMR052W	13:379981	2.44E-09
YHR027C	8:175255	2.96E-08
YER056C-A	15:154309	2.22E-06
YLR419W	2:506661	1.02E-06
YLR419W	15:150651	8.49E-08
YDL017W	4:465157	5.38E-09
YLR449W	12:1031685	1.35E-06
YLR449W	15:174364	5.86E-09
YIR038C	15:174364	3.50E-08
YLR361C	12:851826	1.20E-07
YOR140W	2:548401	9.27E-10
YKL107W	2:562415	5.15E-06
YKL107W	11:229040	1.50E-07
YOR211C	13:81250	1.78E-06
YAL037W	2:548401	6.29E-07
YLR377C	12:872448	2.02E-10
YDR011W	12:672779	4.77E-06
YDR011W	15:632894	2.47E-11
YPR119W	2:555787	2.95E-06
YBR073W	2:533262	5.23E-08
YBR073W	15:170945	5.69E-07
YMR081C	15:174364	3.34E-07
YDR177W	4:812947	3.12E-07
YEL037C	5:79647	2.21E-15
YGL205W	7:110807	7.24E-12
YBR261C	15:154177	8.19E-08
YGR189C	13:46084	1.75E-07
YGR189C	15:174364	3.13E-06
YPL104W	15:174364	1.88E-09
YOR048C	15:150651	1.87E-09
YKL029C	3:91305	4.96E-08
YKL029C	11:382553	2.55E-13
YOL070C	15:179289	2.08E-07
YPR007C	16:547618	8.04E-13
YDL135C	4:143910	2.99E-07
YPR172W	15:144659	7.84E-08
YHR080C	15:143597	6.56E-11

YIL169C	9:27026	2.59E-10
YCL026C-B	3:75021	6.70E-15
YGL079W	12:683457	3.77E-06
YML058W-A	12:469156	8.70E-08
YJR102C	2:548401	1.32E-06
YMR124W	13:513778	1.14E-11
YNL246W	14:191243	3.00E-15
YMR296C	2:519049	4.23E-06
YMR296C	13:57145	1.99E-06
YML088W	13:57145	1.98E-07
YML088W	15:174364	5.78E-07
YDL014W	15:170945	2.02E-06
YDL010W	4:433589	1.21E-08
YAR042W	1:201039	1.59E-15
YKL088W	4:46316	5.97E-06
YLR399C	12:662627	1.20E-07
YPR008W	15:174364	2.19E-08
YOL137W	15:59733	1.81E-10
YIL048W	9:251537	1.68E-10
YHR084W	8:111683	7.84E-12
YPL232W	12:662627	4.97E-07
YLR146C	15:174364	1.84E-08
YJL077C	2:592989	1.64E-05
YNL131W	15:154177	8.21E-07
YMR100W	12:677957	1.69E-08
YBL032W	2:162382	8.06E-07
YOR286W	15:170945	1.93E-07
YNL115C	15:174364	1.95E-06
YGL064C	2:537314	3.68E-09
YGL064C	7:375499	1.88E-06
YOL056W	15:170945	7.67E-09
YOR311C	15:180961	2.65E-06
YGR289C	7:1075580	9.85E-17
YGL037C	15:174364	2.48E-09
YBR013C	2:256896	8.34E-16
YNL058C	14:525061	1.68E-11
YOR065W	12:668249	1.65E-08
YOR034C-A	2:517365	1.25E-06
YPL236C	15:136327	8.08E-07
YLR290C	12:705226	1.70E-07
YGL032C	3:201166	8.94E-17
YER001W	2:551299	3.45E-07
YER001W	15:141627	1.36E-08
YER001W	16:511406	2.93E-06
YNL255C	15:150651	5.55E-06
YPL052W	15:174364	1.18E-09
YKL101W	8:111690	4.58E-07

YKL101W	11:247944	1.19E-07
YBR274W	2:750838	1.13E-14
YLL041C	15:174364	3.08E-08
YGR147C	2:567221	1.39E-07
YGR237C	15:170945	8.06E-08
YOR212W	8:111683	4.11E-09
YOR212W	13:49894	1.90E-06
YKL155C	15:174364	3.02E-06
YIL148W	9:74540	4.85E-15
YDR513W	4:1456748	5.00E-10
YDR513W	15:174364	6.01E-07
YGL060W	8:111683	1.71E-11
YLR439W	2:551299	3.03E-07
YLR439W	15:174364	9.18E-08
YHR179W	12:662627	5.85E-10
YHR179W	15:144659	2.96E-08
YPR026W	15:174364	8.37E-09
YHR168W	15:174364	6.25E-07
YJL006C	10:451946	4.71E-09
YPL046C	16:462646	1.33E-09
YNL245C	14:191243	2.93E-13
YGR143W	15:170945	7.20E-07
YOL159C	9:19607	1.45E-14
YOL164W	15:10427	4.75E-17
YBR272C	2:746476	1.16E-08
YCR106W	6.628472222	5.89E-09
YCR106W	0.868055556	4.12E-09
YCR106W	12:1054278	4.82E-11
YML073C	15:154309	3.66E-06
YNL274C	15:174364	1.43E-09
YGL227W	15:174364	2.34E-07
YNL004W	14:449639	4.55E-14
YPR120C	8:111690	2.71E-06
YER048C	2:537314	5.18E-06
YOL014W	15:298710	3.52E-16
YLR228C	15:174364	3.09E-07
YER155C	8:111683	3.27E-09
YGR153W	7:794884	5.50E-09
YIL131C	2:551299	1.24E-08
YNL173C	2:567221	5.50E-07
YNL173C	12:662627	5.38E-07
YGL103W	7:311205	6.10E-12
YGR109W-B	7:708028	7.35E-15
YGR109W-B	9:200332	1.47E-14
YKL052C	15:144659	5.21E-14
YJR080C	15:174364	1.36E-09
YAL056W	1:41483	2.69E-15

YAL056W	16:500348	1.91E-07
YER188C-A	5:568698	3.98E-14
YNL315C	15:174364	2.81E-06
YFL038C	15:180222	2.26E-06
YNL316C	14:38762	7.83E-15
YGR121C	2:519049	6.29E-06
YPR138C	2:555575	1.43E-08
YPR138C	13:46084	5.94E-07
YOR109W	15:546197	3.63E-10
YFR012W	6:168342	6.63E-07
YBR170C	2:567221	1.32E-12
YBR170C	16:500348	1.22E-07
YHR213W	1:23780	8.46E-09
YOL089C	15:154309	2.02E-15
YOL159C-A	9:33795	3.61E-09
YOL159C-A	3.133333333	1.01E-09
YBR052C	15:174364	1.69E-08
YKR097W	2:569420	2.08E-07
YKR097W	11:632952	2.72E-12
YJR030C	10:472146	1.83E-16
YMR312W	13:885665	1.32E-13
YMR312W	15:174364	5.57E-10
YJL010C	15:174364	1.09E-08
YLR176C	12:514835	3.98E-09
YIL071C	2:537314	6.69E-06
YIL119C	9:139462	4.68E-10
YIL119C	13:328865	1.38E-10
YNL134C	15:150651	6.90E-14
YBR148W	2:548401	8.62E-15
YGR127W	15:174364	1.09E-08
YBL087C	15:154177	4.77E-07
YHR028C	2:555596	4.35E-07
YHR028C	8:167504	6.61E-13
YLR250W	2:555596	3.36E-06
YGR174C	15:174364	1.18E-09
YGL258W-A	2:562415	6.11E-08
YFR052W	15:154177	1.63E-07
YOR230W	2:555575	3.13E-06
YDL193W	4:114155	7.60E-07
YDL193W	12:634226	2.86E-07
YJR019C	15:136327	4.32E-07
YOL147C	15:108577	1.65E-07
YIL051C	13:49894	8.05E-16
YCR009C	8:111690	1.05E-06
YAR071W	13:27644	1.05E-14
YGR136W	2:551299	1.74E-06
YBL071W-A	15:174364	9.18E-08

YIL152W	9:74540	4.18E-15
YGL075C	7:375499	1.11E-10
YHR102W	15:141627	6.09E-06
YOR027W	7:375499	1.72E-12
YDR229W	13:46084	2.44E-07
YBR085W	12:635380	5.00E-07
YHR025W	15:170945	6.66E-07
YJR007W	15:174364	1.03E-06
YCL012C	3:100213	2.41E-08
YML119W	13:27644	5.68E-10
YDR446W	8:80068	5.00E-06
YKL141W	13:28622	4.19E-06
YKL141W	15:174364	1.24E-08
YIL065C	7:707950	1.26E-06
YIL065C	9:200332	1.49E-09
YNL137C	2:555787	2.25E-06
YNL137C	15:180210	6.77E-07
YBL038W	2:138648	1.17E-07
YNL202W	14:258590	4.83E-09
YKL197C	11:85465	1.88E-07
YAL060W	1:36900	1.11E-12
YAL060W	15:179289	2.28E-06
YDR410C	8:111682	1.25E-07
YGL181W	7:143756	7.02E-12
YDR138W	15:141627	6.54E-06
YPR041W	15:170945	3.94E-06
YOR023C	15:409778	2.90E-06
YIL082W-A	7:708028	1.38E-14
YIL082W-A	9:200332	1.97E-14
YBR156C	2:555787	6.28E-14
YBR156C	8:111680	4.79E-08
YER118C	2:537314	6.85E-12
YPL110C	13:27644	4.70E-14
YPL110C	15:174364	2.71E-06
YGR150C	7:790117	1.49E-06
YGL009C	3:81832	3.90E-15
YKR093W	13:46070	3.30E-08
YKR093W	15:141633	2.70E-09
YML047C	4:963733	6.09E-06
YML047C	8:111683	2.48E-14
YGL184C	8:167504	8.76E-12
YNR069C	2:555596	5.92E-08
YLL042C	12:63866	1.01E-13
YMR047C	2:499889	2.81E-06
YHR013C	8:137227	1.25E-06
YHR148W	15:174364	5.98E-06
YHR088W	15:174364	4.71E-08

YGL045W	7:402841	7.26E-09
YER029C	5:210999	6.10E-15
YHR029C	8:167504	2.60E-08
YGL139W	2:551299	2.80E-09
YMR174C	2:517365	1.72E-06
YMR174C	15:174364	2.74E-07
YDL224C	2:516889	2.92E-06
YDR173C	4:812947	5.64E-10
YDR173C	15:144659	1.67E-06
YKL215C	2:519049	5.45E-06
YCR107W	6.628472222	5.15E-09
YCR107W	0.868055556	2.60E-08
YCR107W	12:1056103	1.34E-09
YMR191W	13:649250	3.62E-14
YIL031W	12:662627	4.38E-08
YOL164W-A	15:10427	4.75E-17
YLR219W	15:174364	8.48E-12
YPR158W	7:375499	2.05E-12
YCR034W	15:154309	1.36E-06
YER057C	13:33681	2.10E-08
YNL227C	15:174364	4.23E-07
YOR111W	2:551299	7.49E-08
YNL278W	8:111683	3.04E-07
YER130C	15:150651	2.19E-06
YOR215C	15:143597	3.48E-07
YOL091W	15:144659	1.15E-16
YOR220W	13:28622	3.62E-06
YOR220W	15:170945	4.15E-07
YIL067C	15:143597	1.62E-06
YGR067C	2:584357	4.15E-06
YKL182W	2:516889	1.33E-06
YKL182W	12:659357	6.03E-07
YFL031W	6:75460	5.95E-08
YGL194C	7:139851	9.05E-11
YFL065C	4:1511257	1.08E-07
YKL145W	12:644136	4.49E-06
YHR010W	15:154309	6.40E-06
YIL155C	15:174364	2.37E-09
YKL128C	11:194611	5.69E-10
YGR243W	13:57145	8.82E-07
YGR243W	15:154309	2.04E-10
YGR077C	15:136327	1.58E-07
YLR155C	12:472165	1.89E-16
YBR034C	15:174364	8.81E-08
YBR072W	2:380938	2.09E-08
YJL027C	10:393261	3.08E-08
YGR102C	15:174364	4.95E-07

YJR115W	7:139851	1.01E-07
YPL075W	15:136327	7.77E-07
YLR154C	12:662627	3.62E-11
YLR285C-A	2:555787	1.01E-14
YJL005W	15:174364	3.66E-08
YML004C	13:298193	1.18E-09
YER082C	15:174364	1.56E-09
YHR107C	2:506661	6.14E-09
YHR107C	8:167504	3.02E-07
YLR066W	15:174364	3.89E-06
YPR154W	12:659357	5.14E-07
YML018C	15:174364	1.84E-07
YDR058C	4:582121	2.70E-12
YDL180W	4:164444	3.63E-07
YLL021W	15:174364	6.01E-07
YMR210W	5:333252	7.11E-07
YOR085W	12:705226	1.70E-07
YKL216W	5:117056	4.63E-16
YKL216W	15:174364	1.91E-07
YPR095C	12:659357	1.30E-09
YLR129W	15:150651	5.86E-07
YLR380W	12:644136	7.80E-07
YOL116W	15:141627	3.99E-09
YDR366C	4:1213416	2.21E-16
YNL271C	12:644082	2.60E-06
YMR295C	15:106266	1.06E-07
YLR305C	12:697260	6.75E-07
YCR020C-A	15:136324	3.37E-06
YPR198W	11:656099	6.12E-17
YLR042C	2:567221	1.45E-15
YOR051C	15:438828	3.06E-09
YIL063C	9:214482	1.44E-09
YJL107C	2:555787	3.30E-06
YJL107C	10:218798	1.09E-12
YJL107C	12:659357	7.39E-06
YDR263C	4:975086	1.12E-13
YDR263C	15:143597	1.81E-06
YIL101C	9:244902	1.01E-06
YIL101C	15:174364	4.71E-08
YMR129W	2:516889	1.07E-06
YMR032W	2:551299	5.37E-09
YMR032W	15:141633	7.75E-08
YNL328C	8:152932	4.92E-06
YLR186W	15:174364	7.02E-07
YAR075W	1:229090	1.98E-12
YPL265W	13:46084	3.12E-07
YGL058W	15:174364	1.69E-07

YKL087C	2:551299	4.97E-06
YKL087C	15:174364	3.22E-08
YLR278C	13:255564	7.15E-07
YDR357C	4:1213416	6.53E-13
YNL223W	15:154309	3.48E-07
YER067W	15:174364	1.76E-07
YBL054W	2:133741	3.07E-07
YMR173W	2:551299	1.10E-06
YNL035C	14:571965	1.19E-16
YCR105W	6.628472222	1.09E-08
YCR105W	0.868055556	2.32E-07
YCR105W	12:1056103	8.16E-09
YOR304C-A	15:889464	6.71E-15
YJR107W	10:627628	3.14E-09
YER049W	15:174364	1.64E-09
YOL073C	15:174364	3.66E-08
YAL043C	2:508843	8.97E-07
YOR337W	15:174364	3.62E-07
YJL062W-A	2:555778	9.12E-06
YJL062W-A	15:170945	1.10E-08
YHR210C	12:634227	1.19E-06
YHR210C	15:136327	3.02E-07
YJL013C	10:404508	5.31E-12
YBR045C	2:328489	5.74E-17
YDL238C	2:555575	7.81E-13
YJR043C	2:533262	1.79E-07
YER107C	5:422612	1.11E-07
YER107C	15:174364	8.11E-08
YLR312C-B	2:533262	2.46E-06
YLR312C-B	12:744310	1.67E-09
YGR097W	15:150651	1.62E-06
YGR100W	2:555596	3.89E-06
YBL030C	15:141627	1.63E-08
YIL007C	12:662627	2.30E-09
YPR113W	12:677957	3.66E-08
YOR227W	3:91287	2.52E-08
YOR227W	15:174364	2.99E-10
YBL002W	2:569420	2.11E-06
YKR045C	11:522777	4.96E-10
YER145C	12:659357	2.35E-06
YER145C	13:910381	1.24E-05
YGL248W	7:15891	2.36E-14
YGR091W	2:551299	5.81E-07
YPL014W	15:174364	6.01E-07
YCR086W	3:258303	1.45E-07
YJL170C	3:201166	8.94E-17
YJR127C	15:144659	4.57E-11

YCR046C	2:555787	3.85E-06
YCR046C	15:174364	1.08E-09
YJR085C	15:180961	9.10E-06
YDR218C	12:672779	1.51E-09
YIL023C	9:308854	3.69E-09
YPL213W	2:551299	9.00E-08
YNR059W	15:143597	3.57E-06
YNL040W	2:513408	2.20E-07
YNL040W	14:591237	4.50E-12
YHR151C	12:668249	2.62E-06
YPL008W	12:668249	4.11E-06
YKL179C	2:555778	4.40E-08
YGL106W	2:562415	9.54E-06
YGL106W	8:111683	1.20E-08
YHR039C	12:662627	1.88E-12
YIL035C	2:562415	2.26E-06
YGL241W	7:44996	9.43E-10
YJL198W	12:644136	3.13E-06
YPL064C	16:428900	6.84E-10
YML109W	2:555596	1.10E-08
YDR277C	15:174364	1.55E-08
YGR251W	7:995892	7.21E-15
YNL300W	2:521415	4.21E-08
YNL300W	8:111683	2.85E-09
YDR042C	4:527458	2.64E-08
YLL063C	2:569420	1.39E-06
YKL078W	13:79760	1.67E-06
YJL157C	8:111683	8.71E-13
YJL157C	13:46084	2.23E-06
YLR117C	4:1188862	1.70E-06
YEL005C	15:174364	1.68E-06
YMR194C-B	15:174364	1.83E-12
YDL209C	4:80849	4.26E-07
YNL151C	12:662627	1.83E-09
YNL151C	15:154309	2.14E-06
YGR138C	12:659357	2.18E-06
YGR138C	16:511406	2.57E-07
YDR425W	2:507282	4.22E-07
YPR016C	15:170945	1.95E-06
YOR043W	15:409778	7.04E-09
YGR118W	15:113251	1.02E-05
YJR006W	2:551299	6.90E-07
YKL113C	11:218377	3.80E-09
YPL096W	15:170945	8.09E-07
YDR478W	4:1418647	1.43E-14
YGL081W	2:562415	1.18E-05
YGL081W	7:375499	7.18E-07

YPR105C	2:548401	2.65E-08
YDR421W	16:500348	8.50E-07
YOR096W	15:154309	3.10E-06
YDR444W	4:1344670	1.30E-10
YJR061W	10:548177	1.24E-11
YGR257C	7:1007587	6.87E-11
YNL078W	2:555596	5.58E-14
YNL078W	8:111680	6.63E-07
YDR488C	12:674651	1.01E-06
YHR016C	15:174364	5.12E-09
YIL062C	9:242417	5.91E-08
YOR142W	15:143597	7.80E-08
YGR225W	7:948590	1.14E-07
YER180C-A	5:568500	1.18E-11
YLR163C	15:113254	2.08E-06
YPL157W	15:180180	2.63E-06
YPL157W	16:252119	5.34E-14
YDR091C	15:150651	2.04E-07
YKR076W	15:174364	3.91E-09
YMR060C	13:390351	6.62E-13
YLR086W	12:317542	5.56E-12
YKR064W	15:144659	4.68E-07
YBR054W	13:277071	2.60E-07
YHR115C	8:340251	3.08E-12
YGR021W	15:174364	9.55E-09
YJR084W	10:604478	6.92E-12
YJR015W	10:461201	1.61E-16
YGR142W	7:375499	3.20E-13
YML005W	15:170945	8.41E-07
YLR046C	12:239578	2.14E-08
YPL252C	16:70853	7.03E-15
YKR053C	11:554200	9.07E-14
YGR200C	7:889602	3.04E-07
YGR200C	15:174364	3.76E-07
YER141W	12:662627	4.64E-06
YHR064C	15:154177	8.03E-07
YJL051W	10:345059	1.55E-16
YGR234W	7:948578	3.35E-08
YGR234W	12:662627	8.22E-16
YNL280C	12:662627	3.83E-09
YNL092W	15:174364	1.91E-07
YLR462W	4:1511257	2.38E-08
YMR297W	15:174364	1.56E-07
YDR384C	12:705226	2.43E-08
YKL007W	10:548177	2.31E-11
YAR003W	12:662627	1.04E-06
YLR017W	15:174364	1.95E-06

YCR104W	12:1056103	1.28E-07
YHR017W	15:174364	2.83E-08
YHL016C	8:71742	2.29E-13
YMR157C	13:572643	8.09E-09
YMR157C	15:113261	5.00E-08
YNL133C	15:141627	1.12E-09
YCL025C	13:87587	7.36E-09
YCL025C	16:500342	1.99E-07
YBR117C	2:477206	1.68E-14
YGL125W	3:100213	2.55E-07
YHR031C	8:167502	2.48E-11
YNL056W	14:502496	7.52E-14
YDR034W-B	15:174364	4.15E-08
YHR022C	15:141633	7.26E-07
YJL037W	10:380085	2.17E-10
YJL118W	15:143597	1.33E-06
YDR296W	15:174364	2.01E-08
YLR085C	12:317542	2.74E-09
YER092W	9:98955	3.72E-06
YOR241W	15:174364	7.32E-09
YBR204C	2:565216	2.13E-11
YGL035C	7:427476	2.06E-09
YLR378C	2:551299	1.15E-06
YOL114C	15:106272	3.08E-06
YKL222C	0.868055556	2.65E-14
YHL013C	8:93002	1.48E-05
YMR120C	13:77684	4.76E-08
YGL099W	15:174364	2.17E-06
YMR117C	13:492453	3.77E-15
YBR038W	2:551299	1.77E-09
YGL183C	7:141949	9.52E-13
YER187W	5:568698	3.37E-16
YOR001W	15:150651	2.53E-08
YOR161C	15:174364	1.67E-10
YLR035C	12:705100	3.53E-06
YOL065C	2:427677	4.23E-06
YMR286W	15:154177	3.34E-08
YER056C	5:272258	7.22E-11
YLR327C	15:174364	1.14E-08
YOR224C	15:174364	3.50E-08
YPL229W	12:672779	1.13E-06
YML085C	13:99675	1.38E-14
YAR035W	15:150651	6.60E-07
YDL079C	4:285470	1.06E-06
YDL079C	15:174364	9.71E-07
YCR043C	15:174364	1.62E-08
YEL015W	2:480009	4.74E-06

YEL015W	14:449639	2.93E-10
YMR177W	7:403626	1.26E-05
YMR177W	15:174364	8.00E-09
YLR414C	2:537314	8.35E-07
YLR414C	12:659357	1.27E-06
YOR274W	15:174364	1.99E-07
YDR492W	7:402879	7.40E-10
YGL234W	13:99720	3.67E-07
YEL057C	5:44617	9.21E-17
YNL099C	2:521415	9.14E-08
YIL087C	7:708252	1.64E-09
YIL087C	9:196145	2.15E-16
YIL087C	15:136327	4.00E-07
YNR060W	12:683463	1.71E-08
YNR060W	15:179289	1.45E-06
YOR180C	8:111690	3.79E-06
YML074C	13:163180	1.25E-06
YDL227C	4:46316	2.67E-16
YPR065W	12:659357	4.59E-13
YGR121W-A	7:705697	4.68E-11
YEL004W	2:555596	6.43E-07
YLR452C	8:111683	3.95E-14
YMR200W	2:537314	2.72E-10
YNL104C	3:105042	2.78E-10
YLR466C-B	4:1510883	5.91E-16
YLR466C-B	12:1056097	1.49E-07
YJR114W	10:621762	3.90E-08
YGR244C	15:141627	8.66E-11
YBR264C	8:111683	1.43E-08
YKR075C	7:375499	7.42E-10
YOL007C	2:530481	8.10E-11
YOL007C	8:111683	1.10E-07
YKL043W	11:354466	1.07E-13
YDL166C	4:143910	1.71E-08
YDL166C	15:174364	1.29E-06
YNL289W	2:533262	2.84E-08
YNL289W	8:111683	5.09E-07
YJL209W	10:40238	1.00E-10
YGL097W	15:174364	5.78E-07
YPR137W	15:174364	9.18E-08
YPL061W	13:77684	2.17E-12
YIL008W	15:136327	3.69E-07
YJR051W	12:668249	1.66E-07
YDL211C	15:180961	4.60E-06
YMR009W	12:662627	3.63E-14
YGL010W	7:459354	6.34E-11
YGL010W	15:179289	3.52E-07

YMR163C	2:517123	3.50E-08
YJR034W	15:170945	4.21E-10
YGR047C	8:111683	2.21E-07
YML062C	8:111682	2.08E-06
YCL055W	8:111683	9.15E-15
YPL156C	8:111682	6.17E-08
YOR237W	12:662627	6.34E-11
YNL021W	14:591234	4.69E-14
YOL141W	15:43153	7.89E-14
YDR451C	4:1400033	3.63E-11
YBL086C	15:144659	1.24E-08
YGR249W	7:974640	3.06E-11
YIL002C	13:110808	1.53E-06
YNL054W	12:662627	3.73E-06
YNL054W	14:502496	5.54E-10
YNR040W	2:555575	5.29E-07
YNR040W	15:180210	3.79E-08
YBL021C	2:163240	3.82E-06
YDR279W	15:174364	7.58E-07
YGL174W	7:187179	1.34E-16
YMR244W	12:662627	1.81E-10
YNL258C	8:95469	1.27E-08
YIL015W	3:201166	8.94E-17
YHR111W	15:174364	9.82E-10
YER023W	5:201477	5.11E-06
YGR246C	7:995892	5.89E-08
YIL114C	15:174364	1.11E-06
YKL017C	11:421190	6.77E-14
YIL039W	15:144659	4.67E-06
YNR046W	15:154309	5.55E-06
YOR129C	2:537314	5.10E-11
YDR085C	8:111683	1.23E-06
YPL088W	15:174364	3.98E-08
YPL088W	16:387239	1.07E-12
YHL004W	2:530481	2.63E-06
YHL004W	15:174364	4.15E-08
YKL016C	13:28622	1.66E-06
YKL016C	15:113251	1.30E-07
YKL016C	16:500342	1.03E-06
YNL277W-A	2:533268	1.10E-06
YNL290W	8:111690	1.42E-06
YNL290W	14:96321	1.52E-10
YMR134W	12:662627	5.66E-16
YCL064C	2:553812	1.36E-06
YCL064C	13:49894	2.04E-16
YCL064C	16:500342	1.34E-06
YLR326W	12:782839	5.99E-14

YIL123W	2:551299	2.80E-08
YJR063W	15:174364	2.43E-07
YKR079C	15:174364	2.47E-10
YJL129C	15:136324	2.68E-07
YPR022C	16:600664	3.10E-16
YHL027W	8:63386	9.21E-08
YDR422C	4:1318073	1.35E-12
YBR139W	2:533262	1.39E-07
YPL089C	2:533268	2.25E-07
YPL089C	15:143597	4.43E-07
YHR075C	12:662627	6.54E-07
YPR074C	2:567221	1.75E-06
YKR024C	8:111680	3.71E-08
YBR162W-A	15:136324	4.50E-07
YNL012W	13:115474	6.60E-08
YGR179C	7:853863	7.86E-07
YBR007C	15:174364	6.59E-08
YBR287W	2:537314	2.56E-06
YAL047C	1:55215	5.92E-07
YAL047C	13:124876	4.44E-06
YDL179W	2:555596	7.04E-10
YMR145C	12:668249	1.29E-07
YMR145C	15:113251	5.41E-06
YDR022C	2:533262	1.10E-06
YOL054W	15:174364	2.49E-08
YJR147W	2:555596	5.58E-14
YML050W	15:154177	3.51E-08
YOR270C	12:672779	1.20E-07
YML003W	13:286122	1.53E-12
YOR219C	8:111683	6.43E-07
YDR111C	15:802916	2.64E-08
YPL158C	2:555596	8.68E-11
YKL003C	2:551299	3.61E-06
YKL003C	15:180222	1.49E-08
YDR420W	15:143597	2.04E-07
YDL087C	4:297977	2.04E-08
YGL145W	15:174364	2.10E-08
YNR044W	8:111683	1.11E-13
YPL219W	15:154309	8.60E-07
YKL150W	12:668249	2.00E-06
YKL150W	15:174364	1.42E-08
YIL098C	15:154309	1.61E-07
YGR194C	15:174364	1.97E-09
YKR084C	11:596215	6.56E-08
YGR285C	15:154177	9.95E-07
YCR069W	12:662627	1.28E-11
YLR390W	2:555575	4.03E-06

YLR390W	15:170945	2.89E-08
YDL210W	2:555596	1.74E-13
YDL210W	4:89821	8.61E-08
YDR082W	2:569414	1.82E-06
YKR069W	11:566015	4.06E-14
YIL156W	12:634225	5.15E-07
YJR056C	10:537157	1.15E-10
YMR017W	15:804686	3.53E-06
YGL170C	7:187179	9.91E-08
YLL034C	15:174364	1.28E-07
YPL111W	13:49894	1.86E-14
YLR080W	2:562415	2.33E-08
YML110C	15:170945	4.87E-09
YHR096C	15:154309	4.23E-10
YDR009W	4:450230	2.65E-08
YOR058C	15:438828	1.13E-06
YNL042W-B	8:111683	1.88E-07
YOR273C	15:842006	4.08E-09
YDR251W	15:174364	2.25E-07
YBR163W	2:565216	4.12E-10
YLR206W	12:553064	1.47E-14
YPR033C	15:170945	3.15E-08
YML121W	13:27644	4.41E-12
YNR002C	13:77684	2.22E-08
YNR002C	15:174364	1.64E-09
YER152C	2:555596	4.71E-16
YGL210W	12:662627	1.65E-06
YDR197W	15:174364	1.39E-09
YLR466C-B	4:1510883	9.63E-16
YLR466C-B	12:1056097	4.14E-08
YDR003W	4:447355	1.22E-06
YDR003W	15:154309	1.22E-06
YAR029W	1:185122	3.47E-13
YAR029W	12:672779	9.01E-07
YAR029W	15:170945	6.40E-07
YBL044W	2:142262	7.87E-17
YGR035C	15:174364	1.69E-07
YER025W	15:170945	9.82E-07
YOR315W	2:569420	3.17E-10
YIL036W	15:174364	4.15E-08
YOR316C	2:582419	3.62E-06
YHR059W	2:551299	8.60E-08
YHR059W	15:174364	1.76E-07
YHR193C	3.954861111	8.07E-06
YML081W	12:662627	5.36E-06
YNL156C	12:662627	2.14E-14
YKL120W	3:81832	6.36E-15

YKL120W	15:180222	3.86E-07
YKL051W	13:27644	1.94E-12
YKL051W	15:143597	2.06E-09
YBR058C	15:113251	9.04E-06
YBR078W	2:401568	1.64E-07
YML009C	2:555787	4.15E-06
YML009C	15:154177	1.95E-06
YOR185C	15:174364	4.09E-09
YOL115W	2:551299	4.29E-07
YOL115W	8:167504	1.09E-05
YPL038W	16:492351	3.88E-08
YPL117C	12:662627	1.88E-12
YHL031C	12:644136	2.60E-06
YNL234W	15:136327	4.02E-09
YHR144C	15:174364	2.71E-08
YGL219C	7:85112	1.35E-12
YFL010W-A	6:101519	2.42E-09
YKR089C	8:92960	6.55E-07
YKR089C	11:599170	7.18E-15
YKR089C	13:46084	4.67E-07
YER011W	12:662627	1.91E-09
YGR014W	2:567221	7.04E-14
YDL078C	2:499889	2.16E-06
YOR375C	3:81832	5.77E-13
YIL072W	9:238345	2.81E-12
YAR050W	1:201039	1.49E-16
YOL094C	15:113254	4.87E-12
YIR042C	9:437054	3.04E-16
YLL056C	12:634226	2.70E-08
YJL087C	15:170945	3.39E-07
YKR021W	2:551299	2.41E-06
YJL168C	15:174364	4.34E-06
YJR153W	2:608310	1.07E-06
YJR153W	10:703868	1.82E-07
YBL085W	2:516889	1.05E-07
YHR116W	15:174364	4.09E-09
YBR208C	13:49903	4.01E-08
YCL066W	3:201166	8.94E-17
YLR118C	15:170945	1.24E-06
YLR106C	15:174364	2.64E-07
YGL086W	2:562415	5.12E-07
YDR198C	15:174364	1.09E-08
YIL144W	15:170945	1.39E-06
YJL011C	15:136324	2.91E-07
YGR020C	2:551299	7.95E-06
YDR258C	15:174364	2.97E-07
YOL052C	2:499012	3.36E-08

YMR105C	15:174364	2.72E-09
YNL061W	14:449639	7.58E-12
YNL061W	15:174364	2.34E-06
YJL160C	2:569420	5.25E-07
YJL160C	10:159473	4.85E-07
YCR038C	3:201166	2.99E-16
YER070W	8:111680	1.15E-05
YOR032W-A	13:46084	3.36E-06
YDR365C	4:1213416	1.83E-16
YJR105W	12:662627	1.67E-11
YOR289W	15:174364	1.68E-06
YJR096W	15:170945	3.70E-09
YKL014C	15:174364	7.29E-07
YGL020C	7:459354	1.35E-10
YLR310C	12:757570	9.73E-09
YBR192W	2:567221	3.59E-08
YGL105W	2:553812	5.18E-07
YNL294C	15:143597	3.62E-07
YHR004C	12:662627	6.71E-08
YPL072W	2:551299	2.62E-06
YBR083W	8:111683	1.25E-11
YKL021C	15:174364	1.44E-06
YEL049W	1:154328	2.04E-14
YDR406W	15:144659	3.66E-10
YJR101W	2:555596	2.91E-06
YBR185C	2:555787	8.74E-09
YBR185C	15:174364	4.52E-08
YDR324C	15:174364	7.66E-09
YJR086W	8:111683	2.49E-07
YDL100C	12:662627	3.22E-06
YDR480W	12:659357	7.12E-06
YER062C	15:143597	3.02E-11
YHR086W	15:170945	5.47E-07
YHR208W	3:81832	3.90E-15
YFR028C	2:537314	1.34E-07
YCL004W	3:100213	2.28E-11
YMR150C	13:562907	2.68E-12
YLR079W	2:562415	1.72E-11
YBR098W	2:477206	2.89E-11
YMR313C	13:28694	4.53E-07
YMR152W	13:562907	3.56E-12
YMR152W	15:174364	8.83E-07
YDL174C	12:662627	4.74E-13
YFL001W	2:551299	6.61E-07
YFL001W	6:144755	7.99E-09
YGR023W	15:174364	5.57E-08
YJR011C	15:108577	1.19E-07

YKL010C	11:446685	7.10E-12
YOR247W	2:548401	9.16E-15
YJL171C	3:201166	1.05E-09
YJL171C	10:110038	2.81E-10
YOR232W	2:555596	3.13E-06
YJR076C	10:575236	1.68E-09
YPL186C	15:174364	9.37E-10
YLR257W	2:569420	5.32E-06
YKR103W	11:656099	5.75E-17
YJL069C	15:143597	2.22E-07
YLR264C-A	15:108577	1.08E-05
YPR194C	2:555778	3.13E-06
YPR194C	16:927500	1.38E-11
YJL059W	10:388461	8.16E-07
YFL040W	6:44753	1.23E-13
YPL049C	13:96015	2.50E-06
YHR134W	8:389050	1.21E-08
YGL025C	15:150651	5.35E-06
YIR028W	2:555575	2.21E-11
YCL021W-A	3:81832	3.21E-16
YLR258W	2:567221	1.26E-09
YLR258W	15:174364	2.38E-08
YBR101C	7:375499	3.25E-14
YAL041W	2:569420	1.41E-08
YDR044W	5:350744	1.06E-09
YDR044W	12:662627	3.44E-11
YGR152C	7:790117	1.57E-08
YLR412C-A	12:956534	2.81E-06
YHL003C	8:111679	2.36E-12
YPR139C	13:87587	6.42E-07
YJL153C	10:135902	5.78E-12
YMR092C	13:445622	6.37E-16
YCR072C	15:150651	9.05E-07
YGL130W	15:174364	2.64E-07
YGL263W	12:1019347	8.32E-07
YMR030W	15:150651	3.08E-06
YBR193C	2:582419	4.49E-13
YCL040W	3:14066	1.49E-05
YCL040W	15:174364	3.81E-08
YBR150C	2:537314	1.28E-13
YOR288C	15:174364	2.26E-06
YLR315W	12:681096	4.25E-06
YOR091W	15:143597	1.56E-06
YMR024W	2:537314	3.19E-07
YLR116W	12:380269	6.01E-10
YOL088C	15:170945	4.64E-10
YBL061C	15:174364	8.14E-10

YER044C	12:662627	1.69E-14
YGL051W	7:403626	4.90E-17
YKL068W-A	2:555596	3.36E-06
YLL057C	12:35970	2.36E-09
YNL310C	14:33607	3.24E-08
YNL310C	15:136324	9.07E-07
YML007W	13:245632	3.13E-13
YMR068W	13:404546	8.24E-08
YDR329C	15:143597	6.86E-08
YER060W	15:175594	5.01E-07
YGL237C	15:154309	1.00E-08
YLR425W	12:662627	3.47E-06
YAL016W	12:674651	4.85E-07
YAL059W	0.377777778	2.78E-07
YBL042C	2:138648	2.32E-06
YOR342C	2:567221	4.97E-14
YDL204W	4:95527	3.02E-08
YDL204W	15:174364	1.84E-10
YER096W	2:537314	1.79E-07
YHR041C	8:185882	6.23E-08
YGL056C	12:659357	8.35E-08
YHR104W	15:174364	3.63E-10
YBL046W	2:133749	1.87E-08
YLR067C	2:555575	1.20E-05
YGR212W	7:913059	9.00E-10
YBR177C	15:144659	5.32E-11
YCR092C	10:510007	1.78E-07
YEL075C	3.956944444	3.28E-12
YCR079W	15:154309	1.53E-06
YDR212W	15:174364	5.98E-06
YDR348C	8:111690	5.27E-06
YPR143W	15:136327	4.00E-07
YML061C	12:674651	1.20E-06
YML061C	13:195863	4.54E-06
YLR190W	2:551299	1.62E-07
YFL022C	6:100521	3.50E-08
YFL022C	15:143597	1.50E-06
YJL054W	2:551299	1.25E-06
YDR333C	2:555575	8.27E-08
YER018C	5:193876	2.16E-12
YBR180W	2:602012	8.12E-08
YHR147C	15:154177	4.98E-07
YOL100W	15:113267	2.16E-11
YNL256W	15:174364	3.36E-08
YBR147W	15:150651	6.87E-07
YML104C	13:49894	2.72E-10
YOR194C	14:502316	3.03E-07

YIR018C-A	9:393834	1.57E-08
YPL144W	16:280629	2.96E-13
YER072W	13:27644	2.01E-15
YJR064W	12:662627	4.77E-07
YJR064W	15:174364	3.76E-06
YML079W	13:110808	5.18E-11
YPL018W	13:28694	8.29E-14
YDL027C	15:136327	5.69E-07
YAL024C	2:569414	7.10E-10
YPL274W	13:910381	8.03E-11
YIL165C	2:551299	1.41E-06
YIL165C	9:33795	8.67E-17
YER158C	5:420595	1.86E-08
YJR047C	12:634227	2.26E-08
YBR126C	15:174364	5.84E-10
YER185W	5:568650	1.59E-11
YOR305W	15:589013	2.41E-06
YJR062C	10:535311	2.23E-08
YAL039C	12:672779	8.35E-07
YHR035W	8:176412	4.62E-12
YJL105W	12:659357	4.57E-08
YDL124W	4:262796	2.39E-13
YDL124W	15:144659	4.53E-08
YJL078C	2:567221	5.19E-16
YDR135C	13:234845	2.50E-06
YDR135C	15:141627	2.40E-07
YGR195W	15:154177	4.98E-07
YMR036C	13:328865	4.11E-14
YNR014W	15:174364	4.07E-12
YLR329W	12:782839	8.25E-13
YLR272C	12:689217	7.32E-09
YLR272C	15:141627	5.87E-06
YGL151W	15:116709	4.27E-07
YHL023C	8:56246	6.17E-11
YKR102W	11:656099	5.74E-17
YLR420W	5:117056	2.83E-16
YLR420W	12:964989	6.18E-15
YDR063W	15:143597	9.52E-06
YGR231C	15:170945	5.59E-09
YGR007W	12:662627	3.08E-07
YGR007W	15:150651	3.84E-06
YCR107W	10:28306	2.77E-09
YCR107W	0.868055556	2.01E-08
YCR107W	12:1059806	1.23E-08
YDR249C	2:537314	9.40E-07
YDR249C	8:111683	1.86E-06
YOR346W	15:968429	2.71E-12

YGL167C	13:69114	6.39E-07
YLR178C	15:174364	1.84E-11
YFL062W	7:1081945	1.36E-05
YEL040W	5:79647	3.41E-09
YEL040W	15:174364	1.87E-06
YNL123W	14:393903	9.97E-12
YOR284W	15:842006	1.74E-06
YMR316W	13:910381	5.60E-11
YDL198C	4:95527	2.70E-06
YOL156W	15:108577	8.95E-07
YOR114W	15:546197	1.59E-10
YOR020W-A	15:407690	1.09E-06
YJR042W	2:562415	4.13E-06
YNL075W	15:174364	3.62E-07
YLR083C	15:154309	9.29E-07
YML069W	13:130069	9.16E-08
YCL016C	3:79091	5.19E-13
YJR094W-A	15:174364	1.50E-06
YHR160C	15:174364	4.90E-09
YGR207C	15:113261	1.15E-09
YDR070C	15:174364	5.07E-10
YBR050C	15:842027	1.01E-06
YJR090C	10:586311	7.28E-08
YGR049W	12:662627	2.56E-13
YML041C	13:184113	1.33E-06
YBR196C-A	2:616262	1.61E-16
YDR059C	15:174364	5.77E-06
YIL060W	9:214482	8.24E-08
YIL106W	2:562415	6.41E-06
YIL106W	9:244902	3.24E-06
YIL106W	15:180961	8.78E-06
YDL146W	4:183015	2.48E-07
YOR377W	12:662627	4.01E-11
YPL120W	8:167506	4.83E-06
YDL075W	15:170945	9.45E-07
YLL060C	12:19609	4.55E-11
YBR092C	2:517123	2.84E-06
YBR092C	15:154309	8.68E-08
YJL110C	2:555575	4.63E-12
YBR030W	15:174364	9.53E-07
YDR033W	4:509817	2.00E-15
YDL132W	4:226317	1.21E-14
YDL132W	14:449639	1.34E-12
YNL112W	14:412269	4.08E-13
YMR321C	13:922258	1.00E-14
YCR028C	15:141627	6.56E-08
YBR056W-A	13:69122	5.51E-06

YJL112W	15:154177	6.99E-09
YPL174C	2:551299	2.62E-06
YPL174C	15:174364	8.50E-07
YDR115W	15:180180	1.53E-06
YPL063W	13:129925	2.48E-06
YIL020C	15:174364	1.74E-06
YAL063C	5.234722222	1.40E-11
YAL063C	8:525664	2.02E-05
YMR310C	13:910741	1.68E-08
YMR310C	15:174364	6.87E-08
YDR403W	13:115474	3.40E-06
YPL039W	16:489143	2.61E-14
YLR164W	2:537314	6.08E-07
YLR164W	12:501510	1.90E-08
YML006C	15:174364	6.25E-07
YGR134W	2:516889	1.74E-06
YGR134W	15:154177	6.87E-06
YGR255C	15:174364	7.66E-09
YFR010W	6:152824	1.52E-07
YNL168C	2:555596	4.58E-10
YDR482C	13:27644	2.85E-12
YDR482C	15:141633	6.06E-07
YNL159C	8:167506	1.16E-05
YNL159C	12:635380	1.10E-06
YCR035C	15:170945	1.24E-06
YPL123C	2:537314	8.03E-07
YPL071C	16:420441	6.72E-09
YMR217W	15:174364	2.85E-07
YER039C-A	13:77684	2.79E-06
YDL165W	4:143910	1.01E-06
YER058W	15:154177	6.80E-08
YKR061W	2:551299	1.12E-08
YLR435W	15:174364	3.22E-08
YFL014W	15:143597	8.51E-12
YKL038W	15:174364	6.75E-07
YJL084C	13:100048	8.69E-06
YBR187W	15:170945	3.80E-06
YBR176W	2:584357	2.21E-11
YLR450W	12:1031688	2.23E-12
YLL012W	12:677957	5.36E-09
YLL012W	15:174364	5.35E-07
YLR003C	15:174364	5.78E-07
YBR191W	15:154309	2.06E-06
YNR063W	15:154309	8.20E-06
YMR119W	12:697260	7.87E-07
YOR011W	15:382531	6.59E-10
YPL145C	16:266023	2.64E-07

YDR240C	15:170945	3.41E-06
YCR107W	6.628472222	1.75E-07
YCR107W	0.868055556	1.69E-08
YCR107W	12:1054278	4.77E-10
YNL299W	2:551299	7.07E-06
YLR350W	12:829693	6.06E-09
YOR118W	12:659357	2.48E-08
YLR438C-A	12:1019347	4.78E-08
YNL059C	14:525061	6.82E-11
YHR026W	12:662627	8.22E-06
YNR065C	14:732029	2.01E-08
YMR285C	2:569420	3.98E-07
YBL040C	2:133749	8.92E-12
YGR060W	12:662627	9.88E-12
YJL173C	9:133663	2.41E-06
YOR039W	15:382531	2.69E-06
YAR023C	1:185122	3.41E-14
YJL001W	10:451832	9.06E-10
YPR160W	15:174364	4.09E-09
YJL074C	13:28694	2.39E-06
YLR411W	12:956534	1.18E-16
YBR242W	12:662627	1.15E-13
YLR121C	15:143597	2.64E-08
YJL219W	10:23505	2.58E-16
YNL041C	14:553129	2.87E-09
YMR160W	15:174364	2.53E-07
YKR013W	2:533268	1.15E-10
YAR075W	13:180103	4.27E-08
YNR034W-A	15:174364	4.09E-09
YHR048W	12:659357	1.65E-10
YHR109W	2:530481	5.02E-07
YHL044W	6.176388889	4.90E-17
YER034W	13:57145	1.70E-06
YPL004C	15:174364	1.88E-09
YKL005C	11:482069	4.67E-07
YER053C-A	12:662627	6.53E-15
YDR049W	4:555043	5.93E-08
YDR049W	15:89229	4.04E-06
YLR201C	15:174364	4.52E-08
YOL096C	15:154309	1.02E-10
YJL167W	12:662627	6.15E-15
YNL141W	13:27644	1.94E-07
YDR298C	15:136327	2.09E-08
YDR298C	16:500342	3.48E-07
YDR242W	2:555575	3.51E-11
YPL085W	2:530481	3.50E-07
YPL100W	15:170945	1.10E-06

YER119C	2:555596	9.45E-06
YMR015C	12:662627	2.87E-14
YMR139W	15:174364	1.92E-08
YHR143W	2:555596	2.10E-16
YPR149W	15:174364	3.46E-10
YPR149W	16:500348	7.92E-06
YKR091W	2:555596	1.03E-09
YIL120W	9:136845	2.26E-11
YIL120W	13:99675	2.11E-06
YEL071W	13:49894	3.08E-06
YEL071W	15:143597	2.46E-06
YGL255W	13:49894	1.62E-08
YGL255W	15:174364	3.50E-08
YBR093C	13:27644	2.14E-15
YAL009W	1:136161	3.09E-13
YHL009W-B	8:84437	2.75E-09
YHL009W-B	16:445372	4.65E-09
YGR061C	13:49894	1.15E-09
YDR075W	15:174364	8.18E-07
YOL087C	15:154309	1.00E-06
YFR049W	15:154309	1.65E-07
YML046W	8:111683	3.41E-13
YLR126C	13:27644	7.99E-06
YLR053C	2:551299	1.36E-07
YMR238W	13:46084	9.74E-08
YMR238W	15:174364	6.32E-08
YDR320C	4:1108558	1.09E-08
YGR165W	2:506661	1.50E-06
YGR165W	15:174364	4.34E-06
YCR097W	3:201166	8.94E-17
YIL066C	12:514835	9.93E-12
YBR158W	2:567221	4.72E-16
YPL203W	15:174364	3.98E-08
YMR195W	15:143597	2.12E-08
YMR195W	16:500348	3.89E-06
YGR094W	15:154309	7.65E-08
YJR144W	16:511406	3.94E-06
YMR108W	3:91305	2.46E-12
YMR108W	15:180222	7.41E-06
YDR434W	15:136324	1.19E-07
YIL149C	2:551299	2.89E-07
YIL149C	9:74540	4.03E-08
YBR119W	2:480009	4.30E-13
YBR119W	8:98513	1.25E-06
YKR005C	2:516889	5.43E-07
YFR006W	13:46084	1.41E-06
YFR006W	15:141633	6.97E-09

YEL041W	5:79647	2.21E-14
YBR133C	8:111680	3.54E-07
YDR381W	2:555778	1.25E-05
YLR162W-A	12:472165	8.38E-17
YML082W	15:150651	9.04E-09
YDL013W	8:167506	1.12E-05
YDL013W	13:69114	8.03E-07
YHR032W	8:167504	5.88E-16
YDR093W	2:507282	7.35E-06
YNL237W	14:209852	1.35E-15
YPL161C	15:174364	2.91E-06
YHR099W	2:508843	8.61E-07
YJL136C	15:170945	3.05E-06
YDR523C	4:1495582	6.11E-07
YOR116C	2:551299	8.53E-07
YOR116C	15:143597	9.19E-06
YOR031W	15:382531	6.39E-16
YJL053W	2:555596	3.49E-06
YJL053W	10:345059	9.34E-08
YJL180C	2:506661	6.22E-06
YFR031C	13:28622	6.60E-07
YFL041W-A	15:143597	1.29E-07
YDR379W	2:555787	5.87E-08
YDR379W	16:511400	1.09E-07
YDR281C	13:27644	6.10E-15
YOR108W	3:100213	3.43E-06
YOR108W	15:488377	4.51E-06
YGL144C	12:659357	5.14E-07
YPL113C	13:28694	2.41E-07
YPL113C	15:143597	9.24E-08
YPL270W	13:28694	6.98E-06
YIL145C	15:143597	6.57E-09
YMR252C	12:662627	1.54E-07
YIR017C	5:251647	1.18E-06
YER132C	5:422612	7.78E-08
YER132C	15:170945	2.44E-06
YOL030W	15:141627	6.54E-06
YFR031C-A	15:154309	3.59E-06
YLL023C	12:111412	7.31E-11
YPL082C	2:506661	1.46E-08
YBR024W	15:174364	3.76E-07
YOR052C	15:174364	3.37E-06
YNL158W	12:662627	2.96E-07
YGR070W	15:174364	4.71E-08
YOL082W	2:553812	6.28E-06
YOL082W	15:179289	6.80E-12
YGR044C	2:555596	1.93E-15

YML029W	13:100048	3.02E-07
YNL238W	14:235423	5.87E-08
YPR028W	15:174364	1.39E-06
YOR229W	12:644136	1.18E-09
YPR192W	16:927500	3.75E-14

Supplementary Table 4: eQTLs for the regression method

Accession ID	QTL chrom:position	p-value
YKR009C	2:553812	1.31E-19
YKR009C	15:144659	2.92E-26
YKR009C	16:497425	4.14E-18
YDR481C	3:90610	7.51E-17
YDR481C	13:33501	9.64E-87
YML113W	13:28694	4.78E-20
YML113W	15:180961	2.17E-28
YIL157C	9:238345	2.95E-13
YIL157C	15:154309	2.52E-35
YNR074C	12:662627	1.26E-20
YJL147C	2:553812	2.25E-32
YJL147C	10:163850	1.16E-18
YJL147C	16:497425	4.31E-26
YDL216C	2:555596	4.20E-17
YDL216C	4:70901	1.67E-96
YPL223C	15:174364	7.47E-55
YKL138C	2:555787	1.73E-14
YKL138C	15:180180	1.73E-16
YML035C	15:174364	1.71E-18
YIL160C	15:174364	1.04E-46
YLR050C	12:238298	6.61E-83
YOL008W	15:301074	5.00E-24
YOR072W-B	14:549682	1.47E-29
YPL253C	16:70847	7.38E-30
YDL048C	9:133693	9.49E-26
YDL048C	15:174364	5.88E-36
YDR056C	5.397222222	8.76E-17
YDR056C	2:533268	1.85E-20
YDR056C	12:677957	1.23E-15
YNL083W	15:106266	3.77E-14
YPL154C	15:180961	1.72E-24
YDR020C	2:479161	7.26E-20
YNL216W	2:553812	3.61E-26
YNL216W	14:254145	4.67E-25
YFL034C-B	2:553812	2.65E-35
YEL031W	12:705190	1.02E-18
YBR238C	5:350744	2.60E-32
YFL045C	2:579459	2.19E-14
YGL222C	7:92848	2.98E-115
YHR014W	8:137221	2.44E-20
YEL006W	2:555596	8.74E-20
YLR401C	12:956534	2.37E-17
YDR447C	15:170945	1.29E-20
YKL195W	15:179289	6.82E-25
YOR175C	12:672779	1.26E-40

YHL010C	2:553812	1.68E-19
YHL010C	8:80068	8.70E-33
YHR152W	8:111683	7.00E-27
YHR152W	13:46084	9.91E-21
YER035W	5:200854	1.22E-25
YPL020C	15:136324	8.27E-23
YPL020C	16:500354	8.27E-22
YLR286C	2:555596	1.19E-197
YJL185C	15:180961	8.67E-29
YIL125W	15:180961	2.25E-30
YGR130C	15:180961	3.20E-44
YDL049C	13:81250	1.50E-14
YBR065C	2:364851	1.85E-42
YJR082C	10:575236	4.07E-39
YMR182C	2:427683	3.32E-28
YMR182C	3.955555556	2.35E-16
YPL264C	2:507282	1.22E-25
YPL264C	5:272258	7.37E-36
YAR033W	1:187544	2.09E-45
YAR033W	7:410146	6.34E-21
YIL010W	9:325320	1.00E-13
YLR266C	8:111683	1.14E-14
YDR447C	15:174364	2.00E-32
YML019W	12:672779	2.52E-26
YML019W	13:227254	2.45E-19
YKL166C	2:553812	3.70E-27
YKL166C	11:146603	2.41E-21
YDL086W	12:672779	7.99E-109
YIL176C	4:289639	4.92E-18
YKL096W	11:261779	6.13E-23
YKL096W	15:136324	5.44E-42
YEL003W	15:136327	4.06E-22
YKL075C	12:672779	2.27E-23
YHR198C	13:28622	2.62E-22
YHR198C	15:180961	2.43E-38
YMR212C	12:662627	7.82E-23
YLR149C	15:180961	8.11E-51
YOR271C	3:92247	1.92E-45
YOR271C	15:179289	6.17E-32
YAL034C	15:180961	2.49E-41
YOR084W	15:180961	1.03E-35
YDR140W	4:744330	3.04E-72
YJL042W	15:179289	2.99E-30
YOR187W	15:180961	2.80E-38
YJL186W	13:27644	4.07E-27
YBR285W	2:553812	2.10E-22
YBR285W	15:180961	6.31E-37

YBR285W	16:497425	1.14E-21
YOL028C	15:179289	1.40E-33
YMR220W	12:662627	1.27E-76
YAR002W	1:141181	6.53E-93
YPR106W	2:555596	8.77E-173
YPR106W	16:497425	2.27E-21
YOR124C	2:419093	1.01E-20
YOR124C	14:449639	3.19E-59
YDR072C	2:519049	1.18E-25
YOR394C-A	15:1065719	4.80E-68
YBR157C	2:555596	2.50E-38
YKL192C	15:174364	8.21E-28
YLR283W	12:705190	2.02E-48
YLR151C	12:433958	2.70E-71
YOR165W	2:479166	4.48E-15
YBL018C	2:185450	2.10E-176
YGR124W	3:92013	5.23E-15
YCL068C	3:201167	8.32E-66
YFL049W	6:33688	1.08E-19
YKL167C	15:174364	1.22E-27
YOR264W	2:555596	8.03E-144
YIR039C	6:208654	7.26E-26
YIR039C	9:437054	2.66E-224
YDL061C	2:489202	2.49E-17
YOL122C	15:108577	9.08E-21
YKL208W	3:201166	3.42E-33
YPR117W	15:174364	4.41E-19
YER053C	5:200854	3.29E-16
YER053C	13:28694	1.01E-23
YER053C	15:180961	1.01E-43
YOL105C	2:477206	3.31E-15
YOL105C	15:136324	3.07E-36
YCL018W	2:420528	3.91E-15
YCL018W	3:92157	3.08E-291
YDR518W	12:672779	7.13E-45
YDR210W	2:537314	9.10E-24
YLR089C	13:49894	7.09E-23
YBL036C	15:180961	2.06E-21
YJL097W	12:697260	9.31E-22
YIL161W	9:47053	2.41E-47
YCL054W	5:200854	4.75E-16
YDR006C	2:507282	2.74E-18
YOL162W	15:10427	2.27E-198
YFL047W	2:533268	1.49E-29
YFL047W	8:111683	6.08E-20
YGR174W-A	2:481439	1.61E-16
YDR515W	16:497425	4.35E-19

YOL077W-A	15:154309	4.21E-33
YOL077W-A	16:500354	8.88E-21
YDR119W	2:579459	3.43E-22
YDL125C	4:262796	6.22E-47
YDL191W	9:79793	3.25E-16
YMR178W	2:499895	1.50E-15
YOR285W	9:242417	6.66E-15
YOR285W	15:846344	1.18E-77
YBR017C	14:449639	4.27E-34
YBR084C-A	2:481439	4.52E-19
YBR084C-A	5:200854	1.45E-18
YBR296C	13:28694	3.61E-99
YML064C	13:33501	6.16E-22
YJL192C	12:634227	5.44E-21
YHR005C	8:111683	6.28E-53
YCR088W	14:449639	3.25E-38
YGR279C	7:1048152	6.62E-83
YAL003W	2:553812	1.62E-35
YAL003W	15:180961	5.10E-21
YOR161C-C	15:596079	2.76E-12
YML023C	13:234851	1.78E-27
YGL196W	2:555575	7.54E-29
YGL196W	7:139173	1.64E-30
YBL089W	4:1471224	7.96E-21
YHR124W	13:100048	5.80E-20
YDR231C	15:174364	4.72E-22
YHR053C	2:548401	7.48E-62
YGL104C	7:311205	6.52E-39
YMR169C	15:144659	2.43E-47
YOL071W	15:180961	3.73E-26
YDL012C	4:463267	2.03E-120
YDL012C	12:635380	4.99E-16
YDR206W	2:481439	2.10E-16
YDR206W	14:449639	4.77E-37
YHR191C	8:389050	2.20E-17
YOL011W	15:301076	6.61E-21
YPL002C	16:555416	6.43E-109
YKL194C	15:174364	2.80E-26
YHL020C	8:71742	3.61E-40
YHL020C	12:634225	4.66E-19
YGL179C	7:187567	2.48E-52
YHL032C	15:180961	3.46E-52
YKL140W	11:171771	5.12E-42
YPL127C	16:497425	4.37E-21
YBR295W	15:174364	2.12E-31
YDR223W	4:928745	6.74E-33
YKL085W	15:180961	2.00E-39

YMR048W	13:371857	1.36E-146
YLR312W-A	12:757807	1.51E-55
YML091C	13:33501	6.88E-44
YLR299W	2:592989	5.74E-27
YPR155C	15:180961	3.74E-41
YOR338W	2:565216	3.24E-19
YOR338W	4:95527	5.44E-18
YJL196C	5.234722222	2.23E-27
YJL196C	7:402891	2.19E-21
YHR133C	12:713644	3.77E-13
YOR179C	2:507428	1.82E-29
YNL055C	14:502496	2.54E-48
YNL055C	15:144659	2.18E-26
YCR094W	15:144659	1.42E-17
YDL108W	4:263770	1.90E-39
YOR042W	15:180961	1.05E-21
YGL252C	7:15891	1.77E-89
YDL244W	10:28306	6.07E-33
YDL244W	11:657287	3.28E-17
YPL131W	15:174364	3.34E-20
YMR221C	15:179289	3.51E-20
YBR258C	8:111680	5.14E-15
YLL027W	12:86369	5.55E-29
YKL132C	11:194611	9.08E-57
YOL005C	14:502316	2.16E-20
YJL200C	2:477206	4.41E-24
YKR092C	15:108577	1.42E-25
YKR042W	2:553812	1.26E-17
YDL168W	4:161196	1.68E-213
YGL195W	14:486861	1.22E-42
YGR088W	15:144659	4.11E-57
YMR135C	15:144659	4.14E-46
YMR135C	16:497425	1.24E-18
YDL236W	12:662627	5.06E-38
YLR300W	12:721988	4.77E-40
YPR184W	15:180961	4.38E-55
YHR046C	8:203246	1.81E-52
YPL078C	15:154309	6.07E-32
YPL078C	16:500354	8.41E-23
YKL135C	8:111682	4.60E-19
YKL135C	11:178408	4.24E-40
YDL223C	15:174364	1.31E-42
YNR067C	2:555596	2.19E-140
YBR186W	2:565216	7.45E-27
YAL067C	1:10152	2.10E-139
YDR516C	15:180961	2.57E-28
YMR115W	13:481542	3.40E-29

YOL066C	15:206266	1.07E-35
YCL043C	12:689211	1.12E-14
YFL052W	4:297977	2.63E-15
YFL052W	10:23505	6.59E-124
YEL051W	5:48665	2.27E-18
YEL051W	12:644082	7.01E-21
YDL055C	2:555596	9.46E-73
YPL206C	3:90610	2.01E-13
YPL206C	16:127004	5.41E-21
YOL090W	15:154309	6.36E-41
YML124C	2:553812	4.85E-61
YML124C	16:500354	4.64E-24
YOR062C	15:427159	2.81E-55
YOL036W	15:174364	4.08E-20
YPL067C	2:427683	6.98E-17
YPL067C	16:428900	9.45E-21
YMR306W	2:565216	1.95E-14
YMR306W	13:922268	1.22E-23
YOL098C	15:141627	2.99E-23
YGR178C	14:486861	1.01E-57
YOL021C	2:553812	1.20E-35
YMR008C	3:91287	3.81E-17
YMR322C	12:659357	2.22E-20
YJL133W	10:159479	2.14E-21
YDR079W	9:251537	9.59E-15
YIL166C	9:33795	3.38E-124
YFR038W	2:553812	1.47E-27
YML125C	12:672779	1.27E-20
YML125C	15:144659	1.63E-21
YML125C	16:497425	3.56E-25
YGL048C	8:167510	3.49E-16
YKL047W	2:565216	4.61E-14
YHL001W	2:521415	4.04E-20
YHL001W	5:196196	5.41E-15
YDR460W	3:201166	1.47E-89
YLR313C	12:757807	1.79E-100
YOR178C	15:180961	1.29E-45
YJL016W	10:400191	9.83E-67
YJL016W	15:89217	7.16E-22
YJL218W	10:23505	4.84E-211
YDL218W	2:553812	4.57E-23
YDL218W	4:95527	2.23E-39
YLR256W	12:672779	1.18E-125
YEL021W	5:117056	0
YLR343W	12:815480	1.89E-69
YML102W	8:111683	7.96E-18
YHR097C	8:111683	4.29E-16

YHR097C	13:46084	2.99E-24
YHR097C	15:180961	4.21E-43
YGL253W	7:12939	3.62E-15
YOR390W	13:910381	6.53E-32
YGR258C	2:533268	3.52E-25
YGR258C	15:150651	4.30E-27
YDL229W	2:489202	1.42E-17
YJL108C	10:218798	3.04E-44
YJL108C	12:634226	1.24E-17
YHR157W	2:427683	3.28E-20
YDL155W	2:553812	7.54E-31
YER103W	15:174364	2.38E-23
YEL062W	2:555575	1.03E-25
YEL062W	16:500354	1.59E-23
YPL195W	14:449639	2.87E-26
YDR145W	2:533262	1.21E-17
YDR145W	14:449639	1.17E-43
YGR204C-A	7:905017	1.33E-45
YMR088C	2:555596	5.41E-27
YJL205C	15:179289	1.14E-24
YNR027W	2:427683	3.70E-17
YJR121W	13:28694	3.13E-20
YJR121W	15:180961	7.61E-38
YJR121W	16:500342	2.42E-17
YDR539W	4:1510883	4.67E-128
YNL313C	15:174364	1.37E-20
YKL180W	2:521415	3.12E-20
YKL180W	5:200854	3.98E-15
YKL116C	11:219950	9.28E-104
YNR020C	2:555787	3.97E-18
YNR003C	5:251267	1.93E-16
YNR003C	8:167510	2.08E-15
YPL259C	2:521415	8.96E-19
YEL036C	2:521415	4.05E-16
YEL036C	15:108577	5.22E-22
YLR185W	5:200854	8.95E-17
YBR240C	13:57145	1.88E-23
YPR178W	12:662627	2.55E-20
YMR257C	12:662627	3.25E-18
YCR059C	13:77684	2.41E-16
YCR059C	15:174364	3.06E-33
YHR087W	15:180961	1.38E-54
YDR055W	2:553812	2.76E-17
YER041W	5:210999	2.06E-14
YHR038W	2:555575	2.26E-16
YHR038W	15:179289	8.03E-18
YOL063C	10:575236	3.27E-17

YOL063C	15:205104	4.62E-92
YLL028W	12:86369	2.07E-27
YLL028W	15:144659	1.28E-187
YFR030W	5:272258	2.86E-33
YFR004W	14:486861	4.95E-27
YMR087W	14:449639	1.44E-50
YKL059C	2:565216	2.57E-20
YKL081W	15:180961	2.27E-31
YPR055W	15:106152	3.36E-23
YFL059W	6:33688	1.86E-23
YMR149W	13:562907	6.14E-24
YNR017W	8:167504	1.30E-12
YJL088W	13:54913	1.23E-16
YCR098C	3:301446	1.25E-53
YCR098C	12:956534	1.88E-12
YHR043C	8:203266	1.18E-144
YPR048W	15:170945	1.07E-20
YOR321W	12:708260	6.02E-37
YOR321W	13:404546	5.90E-25
YFL028C	2:507282	4.67E-20
YKL188C	11:97761	2.54E-17
YGL089C	3:201166	2.57E-206
YOR355W	2:479166	4.29E-15
YOR355W	14:449639	3.07E-26
YDR243C	1:51324	5.45E-48
YCR073C	3:90610	4.60E-14
YCR073C	15:174364	1.97E-42
YDR262W	4:975086	9.11E-96
YOL109W	15:136327	1.93E-42
YLR248W	15:180961	4.21E-22
YDR530C	15:180961	4.18E-21
YML007C-A	12:659357	2.97E-18
YJL166W	16:500354	1.53E-19
YNL329C	14:19885	3.19E-67
YER013W	15:136324	7.42E-31
YOL112W	2:486640	8.23E-18
YAL011W	2:513408	3.26E-18
YML067C	13:129925	3.17E-34
YIL094C	2:477206	1.46E-32
YEL076C	12:1067121	5.91E-30
YBL072C	15:174364	2.36E-22
YER183C	5:568566	5.83E-24
YOR032C	13:99585	1.42E-20
YOR032C	15:136324	5.03E-17
YPL097W	15:180961	6.78E-17
YGL143C	15:113260	2.84E-19
YKR039W	11:510933	1.24E-27

YMR280C	15:180961	1.51E-42
YMR280C	16:497425	9.35E-17
YLR218C	15:180180	1.22E-27
YJR032W	15:180961	5.27E-37
YBL027W	2:489202	5.23E-19
YBL027W	5:200854	2.36E-20
YGL189C	2:519049	4.94E-18
YIL162W	9:19607	5.78E-27
YJL125C	2:553812	1.34E-23
YJL125C	10:159479	1.83E-17
YPL034W	16:486637	1.02E-19
YDL103C	3:92013	1.11E-18
YDL103C	4:262796	8.31E-33
YLR303W	2:519049	1.64E-16
YLR303W	8:167504	6.35E-17
YJL100W	12:672779	1.47E-28
YGL001C	12:662627	2.32E-82
YPR200C	3:90610	4.98E-14
YPR200C	11:649300	6.60E-57
YPR200C	16:932535	5.21E-31
YJL068C	2:427683	8.22E-22
YJL068C	14:449639	8.68E-34
YGR272C	15:136327	2.11E-22
YJL158C	10:101187	8.01E-15
YCR023C	13:28334	2.22E-15
YMR279C	12:501510	4.51E-64
YDL057W	2:553812	1.92E-32
YGL038C	2:517365	3.58E-16
YDL105W	4:273846	1.22E-128
YDR127W	4:95437	3.44E-16
YOR028C	15:180961	2.31E-40
YLR265C	12:672779	1.12E-149
YNR043W	12:662627	1.05E-86
YNR043W	15:175594	2.04E-17
YMR237W	2:427675	6.79E-21
YPL036W	16:486637	4.00E-59
YGR085C	2:481439	9.66E-20
YGR085C	5:200854	2.86E-17
YOR125C	15:571111	1.54E-35
YKL027W	11:388373	6.88E-45
YPL189W	3:81832	1.63E-14
YPL189W	16:182953	3.38E-41
YBR112C	2:486640	4.13E-23
YGR148C	2:486640	4.91E-16
YBR291C	2:480009	5.02E-49
YAR014C	2:537314	1.15E-45
YGR092W	8:111690	2.98E-16

YJR074W	10:575236	2.68E-21
YCR017C	15:136324	1.10E-17
YKR044W	11:508575	4.50E-22
YKR044W	15:180961	1.70E-35
YFR045W	15:180961	1.46E-47
YDR540C	4:1510883	1.55E-146
YGR214W	2:519049	3.30E-19
YGR214W	5:200854	5.67E-16
YPL017C	13:28622	9.72E-20
YPL017C	15:180961	1.12E-52
YHR040W	2:553812	2.51E-19
YGL169W	7:187567	4.35E-216
YDR345C	2:555596	1.48E-17
YLR026C	2:507282	3.22E-16
YGR198W	15:683415	3.39E-23
YIL042C	16:497425	3.96E-18
YML123C	13:28694	2.80E-128
YCR083W	15:174364	3.45E-22
YHR011W	15:180210	9.05E-18
YOR090C	15:491172	8.21E-37
YPL258C	16:70853	1.47E-105
YJL140W	10:151310	9.15E-42
YDR416W	15:589145	1.03E-17
YAL042W	2:553812	1.41E-20
YGR205W	7:913065	7.04E-163
YLL033W	12:508029	4.16E-23
YBR197C	2:616262	3.33E-219
YPR062W	12:672785	1.34E-22
YLR260W	12:672779	7.85E-53
YIL044C	2:252538	4.40E-19
YIL044C	9:238345	9.47E-43
YOR003W	2:555596	5.14E-29
YOR003W	12:662627	1.95E-29
YNL233W	2:427675	5.27E-12
YNL233W	8:111683	6.80E-22
YKL035W	13:46084	6.50E-24
YKL035W	15:180961	9.37E-43
YGL057C	1:187602	4.39E-29
YGL057C	7:403626	1.00E-86
YOL061W	15:180961	2.12E-28
YER124C	2:555596	2.87E-250
YER124C	4:95527	6.11E-15
YBL068W	15:180961	5.58E-33
YPL272C	16:500354	1.97E-17
YMR318C	13:910381	2.32E-21
YGL153W	3:90610	7.02E-16
YGL153W	10:387893	4.94E-14

YKR088C	12:672779	4.40E-24
YDL199C	13:28694	4.11E-18
YDL199C	15:180961	5.07E-31
YKL045W	9:79793	1.13E-20
YKL045W	11:354466	3.57E-69
YFL030W	15:174364	1.23E-47
YCL039W	2:555787	1.08E-14
YCL037C	5:194883	2.79E-17
YLR139C	12:433958	9.79E-67
YPR193C	12:672779	1.69E-50
YOR319W	15:889464	1.18E-60
YLR237W	12:634225	3.37E-37
YKL162C	15:180961	2.05E-25
YDR100W	15:144659	6.35E-22
YPL245W	16:84943	2.86E-36
YHR036W	8:176412	4.79E-34
YJL056C	10:327858	2.10E-72
YAL017W	15:180961	1.16E-31
YOL097C	15:180961	2.38E-33
YKR087C	11:596215	3.93E-179
YKL089W	15:594024	5.87E-15
YGR266W	2:553812	7.44E-26
YGR266W	12:672779	7.40E-40
YOR174W	2:555596	4.24E-19
YHR214C-D	5.234722222	1.50E-12
YKR101W	11:646049	2.94E-23
YER093C	12:672779	3.26E-27
YMR156C	13:574867	4.24E-30
YFR033C	12:672779	2.45E-41
YFR033C	16:497425	3.14E-20
YGR287C	7:1063841	1.03E-83
YER149C	2:533268	1.14E-16
YDL237W	2:427683	4.72E-15
YLR275W	12:689217	5.85E-44
YBR135W	2:519049	2.86E-34
YMR142C	2:521415	4.73E-23
YOR228C	15:180961	3.44E-29
YOR228C	16:497425	1.20E-16
YKL187C	11:97761	5.02E-72
YML008C	12:672779	4.18E-55
YLR165C	12:472165	3.27E-145
YPL115C	3:90610	6.24E-14
YFL020C	1:154328	1.39E-50
YCR091W	15:180961	3.17E-36
YKL037W	15:180961	1.66E-32
YMR298W	12:634227	9.54E-34
YOL055C	2:565216	1.95E-13

YHR049W	2:477206	7.16E-19
YHR049W	15:488373	2.74E-17
YMR275C	13:849906	2.22E-39
YDR038C	4:527457	0
YPL045W	15:106266	1.07E-16
YMR239C	15:180961	1.78E-27
YPR006C	16:590622	1.08E-165
YNL008C	14:614342	4.80E-37
YLR038C	12:705190	2.35E-19
YLR038C	16:500354	4.23E-20
YDR064W	2:519049	3.82E-16
YDR064W	9:79793	8.22E-14
YIL134W	9:98955	1.62E-14
YIL121W	3:91977	6.66E-13
YIL121W	12:677957	2.07E-221
YIL121W	13:481544	4.62E-16
YER064C	2:553812	5.31E-41
YDR175C	2:513408	7.37E-14
YDR175C	15:113260	1.75E-14
YDR375C	15:113260	5.27E-21
YER176W	11:648430	1.48E-13
YHR113W	2:555596	1.86E-28
YLR271W	2:507428	1.05E-23
YJL063C	15:179289	3.44E-20
YBR056W	2:477206	1.80E-19
YBR056W	14:449639	5.54E-32
YPR163C	2:553812	9.56E-19
YGL178W	7:187179	9.39E-69
YDR234W	2:479166	2.11E-20
YDL222C	15:174364	3.22E-64
YMR274C	8:111683	7.24E-23
YNL073W	15:174364	4.99E-23
YBR275C	2:746476	1.90E-32
YER093C-A	15:180961	6.72E-23
YKL112W	2:569420	2.08E-22
YIR007W	2:533268	9.03E-27
YMR159C	8:167510	4.64E-16
YIR030C	2:555596	5.22E-24
YIR030C	9:419418	1.54E-43
YKR085C	11:599170	8.84E-32
YGL068W	2:555787	7.49E-20
YCL026C-A	2:407410	1.27E-14
YCL026C-A	3:76127	2.02E-42
YCL026C-A	11:648430	4.13E-19
YER170W	15:113267	1.15E-16
YGL259W	2:553812	9.48E-35
YJR099W	10:612602	3.42E-42

YKL220C	13:910381	9.31E-21
YGL254W	7:15891	2.98E-40
YKL168C	2:553812	8.84E-18
YHR051W	15:141627	6.31E-18
YHR051W	16:500354	1.79E-24
YIL073C	13:27644	1.64E-30
YBR162C	15:174364	4.01E-19
YHL028W	9:133693	2.23E-21
YHL028W	15:180961	4.20E-24
YNL265C	13:33681	2.71E-19
YGR197C	15:632894	9.85E-96
YKL022C	11:388373	1.33E-46
YPR181C	2:507428	4.94E-24
YLR360W	2:555787	6.18E-36
YDR337W	15:113260	1.22E-23
YPR125W	2:507428	4.68E-30
YML054C	15:144659	3.53E-33
YPL107W	2:553812	6.21E-27
YPL107W	16:500354	5.04E-29
YKL103C	2:555596	1.05E-32
YLR342W-A	12:815498	5.94E-134
YLR249W	15:180961	6.54E-33
YHR072W	12:662627	1.04E-93
YLR328W	2:489202	4.30E-18
YBR023C	2:521415	5.41E-22
YBR023C	15:108577	3.64E-28
YEL038W	5:79647	1.31E-140
YMR202W	12:662627	2.91E-104
YDR200C	12:662627	7.23E-18
YFR016C	6:172572	2.23E-16
YFR016C	14:486861	9.10E-38
YGR141W	2:427683	1.23E-14
YGR141W	14:449639	7.46E-48
YNL217W	13:77684	3.53E-28
YNL217W	15:180961	4.72E-30
YOL020W	2:521415	1.19E-36
YKL165C	8:111683	4.25E-17
YLR306W	12:719857	1.48E-19
YOR347C	15:180961	3.64E-27
YDR349C	15:174364	5.81E-38
YGL096W	15:136324	9.22E-25
YDL104C	4:273840	1.95E-22
YGR218W	2:419093	6.14E-17
YGR218W	14:449639	1.28E-56
YGR036C	7:530473	7.74E-18
YDL139C	4:201395	1.69E-92
YOR022C	15:379157	1.24E-54

YKR049C	15:174364	1.97E-41
YKR043C	15:174364	8.70E-48
YOR171C	5:332264	1.76E-16
YDR541C	4:1510883	2.43E-24
YGL012W	8:167510	3.12E-16
YGL012W	12:469156	4.82E-16
YGR053C	9:244902	1.63E-17
YGR053C	15:179289	7.45E-30
YER168C	16:500342	2.01E-19
YDR347W	15:113260	5.41E-19
YOR246C	2:537314	3.24E-21
YOR246C	15:150651	2.88E-42
YMR241W	15:180961	7.89E-22
YML075C	12:644082	3.84E-105
YCR052W	3:210748	1.51E-48
YFL020C	1:154328	2.60E-34
YHR200W	14:449639	1.42E-33
YGR166W	15:174364	8.53E-28
YOL154W	15:10427	4.18E-106
YDR534C	13:910381	4.14E-31
YDR354W	14:502496	8.27E-13
YLR040C	3:201166	4.68E-216
YLR457C	2:519049	7.43E-15
YGR213C	3:90610	3.63E-21
YHR062C	15:180961	1.29E-24
YBL049W	2:519049	1.86E-20
YLR231C	12:644082	4.29E-73
YOR047C	15:357194	1.31E-17
YDR370C	2:555787	9.11E-19
YOR353C	3:90610	6.93E-17
YOR353C	15:179289	2.14E-27
YDR490C	15:180961	1.73E-26
YLR214W	12:611997	1.30E-15
YFR039C	15:106272	7.10E-25
YBR151W	12:611854	2.63E-14
YPL019C	13:27644	1.21E-128
YDR502C	12:662627	5.52E-74
YKR104W	11:656099	0
YHR063C	2:508843	8.72E-15
YHR063C	3:79091	3.96E-15
YOR389W	13:910741	2.01E-33
YOR389W	15:1065719	2.96E-24
YER136W	12:634226	3.21E-18
YGR149W	2:553812	1.36E-24
YOR217W	12:662627	4.85E-16
YLR376C	12:872448	6.05E-76
YML099C	13:110808	8.46E-16

YBR079C	2:553812	4.22E-21
YDL089W	4:289639	5.75E-87
YOR080W	3:90676	6.22E-19
YOR080W	15:108577	7.12E-19
YGR208W	15:180961	2.53E-23
YBR131W	15:174364	1.30E-22
YLR234W	8:111683	3.71E-15
YPR010C	16:497425	3.76E-19
YDR292C	14:502496	8.93E-17
YMR010W	3:90610	1.72E-14
YMR010W	10:387893	1.93E-13
YMR010W	15:179289	5.51E-26
YOR238W	15:804686	1.82E-19
YGL189C	2:519049	5.61E-19
YIL115C	9:101011	1.87E-41
YDR528W	15:144659	4.52E-24
YIL069C	2:521415	1.45E-26
YIL069C	5:200854	3.40E-18
YNL111C	12:672779	4.65E-64
YMR078C	2:516889	1.59E-22
YPR043W	15:170945	7.28E-27
YGL055W	7:403626	6.53E-34
YGL055W	12:677957	1.31E-31
YGR170W	13:100048	2.52E-20
YMR225C	15:179289	4.95E-20
YER089C	2:516889	1.65E-32
YER089C	5:332264	3.69E-16
YML131W	13:124876	5.12E-21
YAR027W	1:185122	2.50E-134
YOR099W	12:713638	1.90E-24
YLR239C	15:180961	4.01E-24
YPL132W	15:179289	6.20E-44
YER088C	15:180961	6.10E-55
YER088C	16:500342	4.97E-17
YCR093W	3:258303	1.54E-17
YJL139C	10:151310	3.97E-48
YAR031W	1:185122	2.51E-56
YER101C	15:154309	7.59E-37
YMR004W	13:261719	7.97E-16
YAL064W-B	1:10152	9.57E-146
YDR520C	12:644082	5.95E-19
YOL104C	15:136324	8.70E-226
YIR031C	2:555575	4.78E-45
YIR031C	9:420076	5.79E-23
YDR067C	4:582121	3.55E-59
YDR067C	14:402312	3.04E-37
YDR524C	3:91049	7.23E-14

YDR524C	4:1456748	2.46E-24
YBL067C	16:500354	6.81E-17
YDR213W	12:672779	5.91E-34
YGR089W	12:672785	9.84E-26
YDR036C	4:509817	2.51E-34
YOL127W	2:499889	5.81E-18
YOL127W	5:200854	1.15E-15
YKL161C	15:174364	1.53E-24
YMR179W	8:111683	6.88E-16
YDR297W	12:662627	6.52E-47
YOR061W	3:90610	1.72E-18
YOR061W	14:502316	6.73E-18
YFR025C	5:272258	6.18E-25
YPR162C	2:555575	3.52E-19
YDR453C	12:681096	1.02E-20
YDR453C	15:180961	2.98E-51
YGR295C	7:1081939	0
YOR213C	14:449639	6.60E-31
YDR272W	2:521415	1.37E-23
YPR191W	13:28694	1.55E-23
YPR191W	16:497425	4.75E-22
YJR046W	7:403626	3.12E-18
YNL212W	8:111680	4.30E-18
YDR237W	2:555787	1.03E-15
YLR410W	13:27644	3.59E-35
YGR223C	7:946196	4.21E-129
YOL133W	15:59733	3.46E-39
YPR151C	12:672779	5.41E-102
YML096W	13:79760	1.82E-24
YMR309C	2:553812	4.03E-24
YDR506C	2:486640	9.41E-26
YFR002W	2:521415	8.80E-20
YDL133W	12:611854	3.59E-15
YIL090W	2:548401	1.12E-14
YHL048C-A	5:15697	2.28E-44
YHL006C	8:111680	1.13E-45
YJR152W	2:555575	7.92E-54
YJR152W	16:497425	4.47E-20
YDL182W	2:480009	1.30E-26
YER150W	15:180961	3.99E-63
YBR154C	2:553812	1.11E-18
YAR008W	1:141181	4.18E-25
YAR008W	13:28694	7.49E-25
YER066W	5:210999	1.24E-15
YER066W	15:144659	3.65E-24
YBR257W	15:180961	1.26E-19
YDL243C	3:91049	1.05E-23

YDL243C	6:28029	6.93E-83
YMR180C	2:555596	1.83E-16
YDL230W	2:569420	8.96E-23
YDL230W	4:54225	2.76E-26
YAL053W	9:242417	2.47E-18
YAL053W	13:54913	2.15E-18
YAL053W	15:179289	1.52E-19
YJR010C-A	10:461201	1.31E-140
YDR186C	12:644136	5.73E-29
YLR436C	15:180961	2.59E-19
YGR084C	2:530481	3.11E-17
YDL095W	2:507282	2.57E-20
YNL270C	2:382536	2.61E-17
YFL004W	13:28694	1.84E-43
YNL240C	2:553812	8.07E-18
YBR175W	15:174364	1.05E-18
YCL027W	8:111683	5.40E-105
YCR095C	15:136327	1.77E-24
YER163C	5:504714	4.56E-58
YOR101W	15:516673	7.90E-36
YLR244C	12:635380	8.55E-86
YNR037C	14:731633	1.53E-16
YOL047C	2:553812	9.97E-59
YLR172C	15:180961	8.96E-28
YDL130W-A	15:180961	6.15E-29
YGR211W	7:913065	8.04E-21
YEL030W	14:449639	1.81E-54
YDL145C	2:553812	3.52E-23
YLR356W	2:565216	2.69E-16
YLR356W	15:180961	3.21E-31
YER054C	15:180961	1.39E-52
YOL022C	15:180961	1.84E-37
YLR049C	12:247898	1.06E-59
YNL045W	13:33681	3.76E-22
YNL045W	15:144659	1.52E-36
YHR067W	2:553812	1.25E-21
YHR143W-A	15:180961	3.71E-26
YML025C	15:180961	5.97E-21
YOL043C	15:255012	8.92E-71
YLR430W	12:987750	1.02E-28
YEL054C	2:519049	4.42E-16
YEL054C	5:200854	4.13E-19
YKL186C	2:565216	3.62E-18
YKL186C	11:97761	1.33E-39
YEL047C	12:469161	1.43E-38
YJL024C	10:393261	3.79E-19
YML057W	13:149075	7.50E-28

YPR020W	16:500354	3.27E-38
YNL252C	15:180961	4.29E-22
YDR129C	14:449639	5.55E-43
YDR129C	15:180961	1.62E-22
YAL005C	1:136161	8.63E-39
YFL017C	14:449639	1.91E-36
YFL017C	15:174364	2.90E-24
YAL029C	15:180961	4.08E-27
YAL029C	16:497425	1.38E-17
YKL149C	2:567221	1.79E-17
YGL063W	7:375499	6.61E-55
YMR125W	8:167504	6.42E-16
YJL131C	15:180961	6.65E-30
YFL008W	6:167184	1.41E-17
YNL160W	15:180961	4.87E-45
YKL072W	15:180961	7.70E-42
YMR116C	15:180961	5.18E-26
YJL164C	15:180961	1.24E-30
YLR204W	15:113260	9.93E-26
YPR141C	2:553812	2.73E-17
YDR147W	12:713686	3.02E-16
YGR239C	5:350744	3.10E-20
YGR239C	12:644082	8.19E-15
YER073W	3:92157	3.70E-31
YIL013C	2:584357	2.97E-16
YIL013C	9:325242	5.00E-19
YMR196W	15:180961	3.98E-53
YBL105C	6.628472222	1.13E-24
YGR031W	15:136324	6.53E-39
YKL124W	11:194611	5.21E-22
YPR060C	15:180961	1.62E-31
YMR055C	13:379981	1.50E-44
YKL084W	15:180961	5.71E-29
YGL110C	2:420528	5.02E-16
YGR201C	15:174364	5.16E-41
YGL185C	2:521415	5.03E-32
YNR050C	2:477206	3.03E-68
YLR270W	15:180961	7.80E-44
YDR346C	12:672779	5.37E-27
YIL070C	2:553812	2.49E-14
YIL070C	15:179289	4.93E-14
YDR246W-A	7:375499	8.14E-25
YJR025C	10:471990	6.74E-85
YKL091C	15:180961	3.42E-44
YKR016W	13:99585	2.11E-20
YKR016W	15:180961	1.06E-31
YKR016W	16:497425	1.55E-18

YKL185W	2:555596	8.33E-61
YKL185W	4:100720	9.73E-18
YOR128C	15:551819	8.56E-27
YDL235C	15:180961	8.26E-21
YGR233C	13:28694	2.65E-96
YEL011W	15:180961	3.29E-40
YJR078W	2:565216	4.34E-16
YJR078W	15:842003	8.90E-30
YDL248W	11:652684	5.65E-17
YFL017W-A	6:100521	4.81E-60
YLR368W	12:852066	6.40E-23
YDR418W	2:489202	1.46E-17
YDR418W	5:200854	1.75E-16
YDR418W	15:174364	6.35E-22
YJR138W	2:555575	1.48E-19
YJR138W	16:497425	5.17E-20
YCL063W	14:449639	3.26E-49
YPL196W	15:180961	2.62E-27
YBL016W	2:199101	7.33E-36
YBL016W	8:111683	2.49E-54
YIL083C	9:197948	8.86E-19
YIL083C	16:500354	2.65E-20
YFL064C	4:1501004	2.52E-25
YBR040W	4:963733	2.62E-24
YBR040W	8:111683	1.48E-43
YNL033W	14:591234	1.28E-61
YDR244W	13:28622	6.02E-14
YKL178C	3:201166	7.54E-228
YGR033C	7:557230	2.19E-33
YGR033C	15:136327	2.29E-31
YHR030C	15:108577	2.62E-21
YGR026W	12:634227	2.50E-22
YDR352W	3:91287	9.61E-13
YKR031C	11:508110	1.77E-27
YGR015C	2:555575	8.50E-16
YOR073W	12:672779	1.38E-16
YDR323C	12:644082	2.06E-18
YKL018C-A	3:90676	2.58E-13
YKL018C-A	16:497425	1.98E-17
YGR217W	2:521415	1.06E-19
YGR217W	15:136324	2.26E-33
YGR217W	16:497425	1.30E-20
YKL133C	2:427683	5.35E-17
YKL133C	14:449639	4.25E-45
YML030W	15:179289	1.03E-35
YOR059C	15:428238	4.72E-28
YKR071C	3:100213	2.23E-36

YKR071C	15:174364	5.56E-21
YCR102C	12:1059806	1.40E-57
YCR102C	15:150651	8.50E-25
YNR058W	2:553812	1.65E-12
YKL148C	11:171771	8.01E-39
YLR319C	2:553812	1.25E-17
YBL037W	2:486640	9.58E-17
YIL104C	7:707950	3.01E-20
YIL104C	9:191491	2.17E-42
YMR063W	13:404546	2.88E-30
YDR178W	15:180961	1.00E-30
YDR178W	16:500342	3.09E-16
YMR208W	12:662627	2.15E-99
YDL066W	2:555575	4.01E-19
YDR202C	15:180961	6.07E-41
YJL137C	13:28694	9.39E-27
YJL137C	15:180961	4.09E-32
YJR039W	15:150651	2.03E-20
YHR201C	8:111682	3.84E-17
YLR179C	3:90676	6.22E-15
YLR179C	12:507813	1.95E-111
YIL014W	9:325320	2.23E-100
YPR122W	8:111680	5.95E-26
YGL225W	2:519049	3.26E-15
YOR019W	15:357194	2.55E-170
YOR250C	12:677957	1.01E-16
YOR250C	15:819015	2.77E-35
YKL067W	15:174364	6.62E-27
YMR244C-A	15:179289	2.36E-41
YIR037W	15:180961	1.44E-29
YEL034W	12:644082	5.14E-122
YPR091C	13:28694	1.21E-17
YPR091C	15:174364	1.04E-32
YJR097W	15:180961	3.62E-34
YMR251W-A	16:500354	3.06E-25
YHL009C	8:95289	4.16E-22
YER177W	15:136327	1.47E-23
YHR018C	2:551299	9.00E-15
YOR038C	15:380725	3.26E-23
YHR207C	15:180961	1.80E-38
YFL054C	6:28029	1.21E-90
YFL054C	10:23409	7.15E-50
YLR388W	2:521415	3.79E-24
YGL160W	12:672779	6.55E-65
YML016C	13:239556	5.09E-59
YPL048W	15:174364	1.11E-16
YOL018C	15:301074	4.51E-46

YJR059W	15:180961	1.45E-21
YBL039W-A	2:151674	2.13E-16
YER016W	8:111683	3.23E-37
YML070W	13:77684	1.28E-23
YML070W	14:449639	2.98E-40
YML070W	15:174364	1.93E-27
YGL229C	2:533268	5.35E-44
YLR246W	2:555787	8.39E-23
YLR246W	12:635380	1.30E-33
YLR246W	16:497425	1.72E-19
YGR175C	15:144659	9.80E-36
YKR026C	15:180961	3.85E-28
YAL064C-A	1:10152	4.07E-64
YJR075W	10:573371	2.11E-73
YLR108C	15:174364	1.83E-20
YMR091C	12:713644	2.43E-15
YMR091C	13:437167	2.85E-36
YJL197W	2:481439	2.51E-16
YJL048C	12:672779	2.55E-84
YOL140W	13:49894	4.54E-19
YOL140W	15:43153	9.15E-25
YDR211W	15:180961	1.35E-40
YML118W	2:565216	5.19E-33
YML118W	13:28694	6.35E-23
YLL029W	12:70127	7.92E-20
YAL031C	8:167510	8.66E-16
YAL031C	13:99585	7.37E-20
YAL023C	2:553812	3.33E-26
YGL257C	7:12939	1.41E-22
YBR228W	2:553812	2.48E-17
YCL035C	15:174364	2.46E-31
YEL020C	2:427674	4.76E-14
YEL020C	14:449639	2.70E-43
YLR388W	2:521415	1.08E-21
YOL006C	15:136324	2.94E-25
YPL221W	2:479161	7.36E-24
YOR054C	15:427159	2.68E-14
YIR036C	2:427674	5.99E-15
YIR036C	3:90610	1.02E-13
YIR036C	14:449639	1.28E-43
YDL181W	13:28694	5.66E-20
YDL181W	15:180961	1.00E-41
YDL181W	16:500354	9.85E-25
YOR035C	2:555596	2.22E-19
YNL224C	3:91977	7.87E-16
YHR054C	8:213595	3.50E-36
YKR094C	2:486640	3.30E-17

YDL093W	12:672779	1.26E-51
YOR195W	15:136324	1.50E-27
YJR053W	10:510235	1.33E-12
YPR128C	1:51324	1.93E-30
YGL101W	12:644082	7.67E-59
YDL185W	12:681096	9.24E-18
YMR096W	14:486861	1.31E-22
YDR096W	15:180961	2.67E-46
YKL062W	15:136324	3.62E-58
YKL062W	16:497425	3.29E-24
YLR342W	12:808623	2.36E-28
YML108W	13:69122	3.15E-17
YML108W	15:180961	3.24E-30
YAL064W	1:11638	1.06E-48
YPL128C	2:514035	9.97E-23
YAR066W	6:48218	5.60E-29
YER028C	4:217351	4.17E-51
YEL050C	15:180961	7.45E-31
YAL062W	1:10152	2.31E-142
YBR067C	2:380932	5.70E-31
YBR067C	12:662627	5.93E-46
YJR094C	10:604478	1.72E-173
YOR332W	12:634227	4.67E-16
YDR504C	4:1456748	9.91E-34
YAR020C	1:154328	1.30E-38
YGR001C	7:502131	5.16E-45
YGR068C	8:111683	6.00E-16
YNL087W	15:180961	9.00E-42
YCR101C	2:573491	6.22E-16
YPL192C	4:963733	9.46E-23
YPL192C	8:111683	5.41E-48
YKR041W	11:508110	1.50E-21
YBR094W	2:477206	5.03E-41
YLR253W	15:106158	4.33E-13
YHR188C	12:634226	6.56E-17
YDR405W	4:1272737	1.91E-54
YEL065W	13:922268	2.02E-18
YNL005C	15:113260	3.25E-14
YEL046C	15:174364	4.19E-31
YJR149W	11:632285	1.55E-20
YLR073C	12:288943	2.99E-105
YNL314W	16:497425	1.89E-20
YIL041W	2:427674	1.40E-14
YIL041W	3:79091	3.12E-14
YLR344W	5:200854	1.97E-16
YJR112W-A	10:627628	7.01E-78
YHR039C-A	12:659357	6.59E-18

YEL017W	5:117056	2.71E-46
YDR018C	15:180961	9.90E-26
YIL046W	15:144659	7.00E-14
YBL082C	2:73342	5.03E-35
YBL082C	13:69114	3.63E-21
YPL125W	16:318068	5.27E-49
YBR106W	16:500342	3.64E-18
YFL018C	13:87587	1.46E-24
YFL018C	15:180961	4.92E-20
YBR286W	12:611854	2.84E-27
YBR286W	15:180961	4.73E-24
YJL154C	10:110044	4.28E-34
YNL015W	2:521415	4.18E-28
YNL015W	15:180961	2.51E-25
YJL163C	10:110038	1.26E-21
YJL163C	15:154309	6.96E-30
YPL016W	16:492351	2.04E-73
YKR067W	11:566015	2.63E-20
YKL193C	15:180961	4.95E-34
YNL108C	15:174364	1.73E-26
YKL080W	12:672779	2.51E-22
YLR397C	12:919638	4.08E-31
YHL035C	13:910381	6.58E-13
YLR213C	12:635380	6.74E-33
YNL067W	7:187570	1.88E-24
YLR245C	12:635380	2.65E-95
YBL111C	4:1501004	1.29E-22
YBL111C	12:1067089	2.46E-20
YHR176W	2:584357	2.14E-16
YHR176W	15:150651	1.86E-26
YLR048W	2:507282	1.06E-18
YLR048W	5:200854	6.60E-21
YDR369C	13:99585	5.42E-41
YPR084W	12:634227	8.49E-17
YPR086W	13:28694	1.63E-21
YGR146C	3:79091	8.21E-14
YGR146C	7:403626	1.45E-17
YGR146C	8:92960	3.69E-16
YOL130W	15:144659	8.61E-26
YNL306W	15:113260	3.81E-25
YOR056C	2:553812	4.00E-33
YLR285W	1:41483	9.42E-18
YER037W	5:226497	8.87E-29
YPL083C	16:408883	2.46E-43
YGR090W	5:200854	1.88E-14
YIL030C	16:497425	4.42E-23
YKR090W	11:612769	2.00E-38

YPL006W	15:180961	1.74E-19
YOL155C	9:19607	2.87E-37
YOL155C	15:48335	3.78E-25
YFL013C	6:100371	2.63E-32
YFL013C	12:796771	1.66E-21
YDR169C	4:782114	6.52E-30
YKR063C	15:180961	1.97E-38
YFR009W	14:449639	1.15E-69
YIR033W	1:11638	4.16E-24
YIR033W	12:662627	1.16E-26
YBR173C	2:582419	6.95E-44
YOR120W	15:180961	3.91E-47
YKL203C	2:555787	2.28E-13
YAL028W	12:634227	7.44E-38
YJR008W	2:553812	1.29E-20
YJR008W	10:451946	2.75E-23
YMR319C	10:336317	4.43E-16
YMR319C	13:910381	1.24E-53
YOL017W	15:298704	6.35E-24
YKL204W	2:499895	7.18E-22
YBL072C	15:174364	9.61E-24
YOL128C	2:555787	4.12E-32
YNR012W	15:180961	1.09E-28
YGR288W	7:1058950	6.54E-26
YOR010C	5:350744	5.88E-46
YOR010C	12:672779	6.03E-24
YGL022W	12:705190	1.08E-14
YGL022W	15:113260	5.53E-17
YMR095C	14:486861	2.07E-23
YBL013W	15:144659	4.85E-52
YMR171C	13:597711	3.48E-57
YBL097W	6.628472222	2.11E-16
YJL055W	12:683463	1.15E-14
YJL055W	15:175594	2.52E-20
YHR003C	12:644082	1.65E-26
YLL006W-A	12:131338	3.20E-145
YOR034C	12:644082	6.58E-18
YOR034C	15:384695	2.07E-14
YOR034C	16:497425	1.31E-19
YNR034W	14:689939	3.07E-28
YNR057C	5:272258	3.28E-45
YHR156C	15:180961	1.51E-24
YNR011C	14:554606	4.51E-23
YHR199C-A	8:167510	1.90E-13
YOR316C-A	2:555778	5.85E-80
YEL052W	15:180961	1.38E-38
YIL046W-A	9:268352	5.35E-36

YDL213C	15:180961	1.29E-24
YGR052W	15:174364	1.28E-72
YDR356W	4:1185630	2.93E-43
YJL187C	8:111683	5.75E-26
YPL168W	2:507282	7.82E-33
YJL206C	2:555596	7.51E-23
YJL057C	10:327858	1.57E-96
YNL178W	9:79793	6.82E-15
YGL004C	15:150651	1.44E-23
YDR430C	15:174364	8.94E-23
YCR099C	2:573491	9.47E-18
YDR219C	16:497425	8.27E-18
YPL278C	13:910741	8.05E-50
YOL151W	15:144659	8.68E-25
YMR205C	12:644082	6.90E-16
YIL092W	7:708345	1.36E-17
YIL092W	9:238345	8.10E-18
YMR236W	2:533268	3.57E-20
YMR236W	8:84437	7.24E-18
YLL013C	12:126934	3.57E-240
YPL005W	16:555416	2.84E-29
YER109C	2:553812	2.07E-22
YER109C	5:350744	1.65E-45
YJR113C	12:672779	4.61E-16
YGR041W	2:555596	8.93E-139
YGR041W	4:95527	1.78E-15
YCR048W	12:672779	9.63E-31
YCR048W	15:116709	1.00E-15
YEL029C	15:170945	2.15E-28
YER154W	2:584351	5.52E-20
YDL067C	16:500354	4.00E-21
YLR383W	12:899898	3.62E-21
YER046W	5:243305	5.77E-71
YIL169C	9:19607	9.34E-41
YIL169C	15:48335	4.40E-24
YBR003W	15:180961	2.94E-27
YMR058W	13:910381	2.21E-17
YIL018W	2:519049	8.33E-18
YPL105C	2:427683	9.32E-16
YPL105C	14:449639	9.95E-46
YBR183W	3:79091	5.66E-14
YBR183W	12:672779	7.43E-33
YKL130C	11:209627	6.95E-32
YBL005W-A	2:246129	5.71E-29
YBL005W-A	13:362310	2.12E-69
YKR034W	2:555575	1.81E-59
YMR264W	15:136324	5.38E-21

YHR037W	8:185882	5.24E-19
YHR037W	16:500342	9.90E-37
YML080W	2:553812	1.28E-19
YER095W	2:592863	1.98E-23
YDR179W-A	15:136324	6.35E-20
YNL146C-A	3:201166	3.57E-186
YNL229C	14:486861	4.64E-24
YLL002W	8:111683	9.84E-18
YOR126C	2:582419	3.81E-29
YPL054W	16:497425	5.46E-22
YOR291W	15:180961	3.30E-23
YDR038C	4:527458	9.84E-108
YOR002W	12:672779	6.27E-22
YGR109W-A	7:708237	2.24E-51
YGR109W-A	8:111683	8.56E-32
YGR109W-A	9:196145	2.88E-74
YLR069C	2:530481	8.43E-16
YLR069C	15:113260	2.23E-16
YNR010W	12:677957	7.12E-18
YLR295C	13:28622	1.34E-21
YLR295C	15:136324	2.07E-37
YLR295C	16:511406	2.95E-24
YFL026W	3:201166	6.73E-215
YDR261W-B	7:543259	3.19E-21
YDR261W-B	12:808707	1.44E-19
YDR367W	4:1213416	1.19E-51
YDR307W	2:486640	2.47E-18
YPL173W	15:180961	9.64E-18
YGR227W	8:111680	1.94E-19
YIL016W	8:111683	1.68E-22
YBL107C	13:87587	1.67E-24
YML078W	13:100048	8.24E-102
YIR019C	5:350744	9.97E-36
YDL170W	15:89217	4.64E-25
YOR297C	2:507428	6.63E-23
YOR278W	2:548401	2.43E-19
YOR278W	16:497425	7.83E-24
YBR259W	15:174364	3.18E-22
YNR052C	14:410244	3.39E-21
YGL164C	2:553812	1.98E-35
YPR047W	15:113260	2.84E-17
YGL212W	7:92896	1.45E-182
YHL022C	8:56252	3.74E-72
YER180C	5:549142	4.02E-137
YKL209C	3:201166	3.58E-218
YGR008C	15:180961	1.05E-33
YMR144W	8:111683	2.00E-14

YHR001W-A	15:141627	4.30E-17
YHR001W-A	16:500354	2.68E-22
YIL079C	15:180961	2.76E-26
YOL069W	13:81250	1.26E-21
YPL118W	15:113260	1.96E-20
YOR384W	13:410287	5.40E-35
YOR363C	1:51324	6.53E-16
YJR004C	3:201166	1.25E-158
YPR134W	2:507428	2.91E-22
YHL012W	8:111683	4.34E-41
YHR214C-E	5.280555556	1.28E-15
YHR214C-E	13:33681	4.28E-31
YEL026W	5:194883	4.62E-17
YEL026W	15:180961	8.19E-33
YLR205C	12:672779	3.07E-108
YKL024C	11:389068	2.60E-22
YPL103C	16:252119	3.10E-21
YEL063C	2:555596	3.53E-34
YBR057C	8:111683	8.98E-21
YFL041W	6:43672	1.47E-26
YKL004W	15:144659	1.39E-21
YBR037C	15:180961	1.21E-24
YJL076W	2:507282	1.17E-18
YFR044C	3:90610	1.63E-14
YFR044C	13:77684	7.77E-28
YFR044C	15:108577	3.28E-30
YFR044C	16:500342	2.14E-19
YKR018C	15:174364	1.06E-17
YDR032C	15:180961	9.29E-35
YPL060W	16:428900	1.71E-12
YER165W	2:507282	1.15E-22
YFR011C	6:168342	2.54E-32
YFR011C	15:113254	4.15E-29
YNL142W	2:555575	2.59E-52
YNL142W	16:500354	1.86E-14
YKL100C	9:133693	1.61E-12
YKL100C	15:180961	2.71E-39
YMR192W	13:649250	1.04E-60
YPL024W	16:500354	1.55E-50
YLL066W-B	4:1525327	1.50E-25
YLL066W-B	12:1056097	5.54E-31
YML086C	15:89217	1.06E-17
YKL201C	11:46635	1.64E-46
YMR133W	2:553812	2.38E-15
YIL117C	2:553812	3.15E-28
YEL066W	3:90610	2.29E-15
YFR018C	6:168354	1.34E-12

YML087C	13:99675	2.36E-46
YLR382C	2:530481	1.85E-15
YLR382C	15:180210	1.08E-21
YIL164C	2:551299	9.89E-31
YIL164C	9:33795	6.74E-83
YPL077C	16:497425	6.76E-20
YAR007C	1:141181	1.54E-44
YAR007C	13:28694	1.07E-16
YFR017C	15:180961	8.38E-46
YER182W	15:180961	2.25E-41
YER014W	5:420595	5.06E-30
YER014W	12:644082	2.94E-68
YNL298W	2:521415	1.15E-20
YML129C	15:179289	7.29E-21
YPL183W-A	15:180961	2.66E-26
YPL106C	16:368296	2.75E-82
YFR015C	15:180961	2.27E-24
YEL064C	2:555575	7.64E-32
YLR094C	12:327131	1.42E-161
YDL215C	3:105042	6.06E-17
YOR044W	8:167510	6.58E-14
YLR087C	12:317542	7.64E-20
YOR086C	15:174364	9.62E-27
YJL044C	10:353027	2.63E-55
YBR004C	2:234820	7.77E-36
YBR004C	9:244902	8.14E-22
YBR004C	15:174364	5.02E-21
YBR049C	2:514035	1.01E-22
YER063W	2:555575	3.89E-29
YJL092W	2:570229	7.65E-16
YBR230C	15:174364	1.75E-31
YOR376W-A	8:167510	5.72E-16
YJR010W	10:461201	8.84E-72
YOR012W	15:357194	5.55E-176
YOR148C	15:589013	1.99E-29
YDR471W	5:193876	2.80E-17
YDR259C	5:350744	9.23E-47
YNL098C	15:180961	2.27E-36
YOL002C	7:402851	6.58E-43
YOL002C	12:713644	1.91E-17
YDR514C	2:555575	1.25E-27
YGR112W	15:180961	6.23E-39
YMR281W	13:843356	4.90E-53
YDL128W	12:22602	7.66E-15
YOR205C	2:519049	3.46E-14
YCR037C	13:27644	1.29E-54
YDR300C	15:180961	1.07E-42

YMR107W	15:180961	2.82E-32
YOR381W	13:910381	7.45E-16
YJR049C	12:634227	6.18E-19
YJR048W	12:672779	3.63E-63
YBL041W	2:151686	2.94E-108
YMR284W	2:507428	4.79E-18
YBL033C	12:681096	3.81E-18
YOL143C	5:117056	2.96E-153
YDR508C	2:553812	1.57E-38
YKL096W-A	15:154309	2.29E-43
YGL197W	7:110813	3.22E-53
YJL046W	10:380085	3.53E-26
YMR199W	13:649250	7.85E-26
YBR298C	7:1075580	1.31E-74
YDR313C	15:174364	1.68E-20
YIL074C	11:394654	9.48E-18
YJL141C	15:180961	1.56E-32
YJR060W	10:548177	1.75E-170
YPL184C	14:449639	1.20E-90
YJL212C	12:693790	1.66E-17
YNR013C	14:554606	2.77E-18
YGR082W	2:553812	4.48E-26
YGR082W	8:167506	3.46E-15
YDL004W	13:28622	1.98E-24
YDL004W	15:180961	1.08E-31
YDL004W	16:497425	3.62E-24
YNL283C	2:517365	8.72E-26
YPL030W	15:180961	6.26E-30
YOL026C	12:677957	5.27E-23
YDL099W	6.628472222	1.70E-12
YAL068C	4:289639	1.97E-21
YDL217C	4:70901	1.16E-29
YDL240W	2:517365	4.53E-15
YDL130W	2:519049	5.62E-21
YBR021W	2:252640	2.76E-28
YPR180W	2:499895	7.71E-20
YCL056C	3:43867	2.70E-59
YBR146W	2:530481	5.15E-24
YBR146W	15:180210	4.76E-14
YKR065C	3:79091	1.29E-15
YKR065C	15:170945	4.49E-24
YEL060C	15:180961	1.42E-22
YGR066C	2:565216	4.25E-21
YHR034C	8:176670	5.25E-31
YHR043C	8:203246	1.33E-115
YJL012C	13:28694	4.25E-128
YGR086C	15:180961	4.26E-56

YGR086C	16:500342	2.83E-21
YMR261C	15:180961	1.57E-34
YJL103C	15:180961	3.39E-30
YIL173W	9:19607	1.36E-60
YIL173W	10:23505	2.26E-87
YPL207W	15:180961	2.20E-40
YLR446W	15:180961	1.26E-33
YLR394W	15:180961	1.86E-23
YBR199W	2:620056	4.32E-36
YLR008C	15:113260	2.93E-12
YIR016W	15:180961	1.09E-49
YOL084W	15:144659	6.56E-86
YMR118C	2:565216	2.78E-31
YMR118C	4:100720	1.40E-16
YIL053W	12:672785	1.05E-15
YPL239W	15:180961	2.02E-40
YJR116W	13:850520	9.51E-19
YCL048W	2:565216	7.65E-17
YOR007C	16:497425	3.84E-23
YEL056W	5:44617	4.16E-28
YBL006C	2:234826	9.64E-43
YNL186W	5:194883	3.36E-19
YFR055W	15:174364	3.97E-22
YFR014C	15:180961	1.73E-54
YGR184C	7:853863	1.33E-40
YKL159C	11:147886	1.38E-43
YFL055W	10:23409	3.29E-88
YHR195W	2:553812	8.92E-15
YHR195W	15:180961	1.64E-33
YIL064W	9:242934	3.88E-35
YHL036W	15:180961	1.23E-29
YLR355C	2:427683	7.86E-16
YLR355C	3:92157	2.54E-45
YLR355C	13:77684	1.06E-25
YDL233W	2:555596	2.08E-16
YOR060C	15:427159	3.85E-43
YIL097W	2:553812	6.40E-20
YIL097W	15:136327	1.73E-22
YJR098C	10:612602	4.26E-56
YNR036C	15:179289	1.03E-23
YNL065W	14:502496	5.06E-57
YGR076C	15:113260	8.90E-22
YLR390W-A	4:161196	2.82E-16
YDL061C	2:489202	2.92E-17
YDL061C	15:174364	1.51E-25
YBL009W	2:553812	1.08E-22
YHL033C	2:519049	8.55E-18

YNL066W	2:555778	6.22E-141
YNL066W	4:95527	4.14E-16
YMR114C	15:180961	6.00E-35
YKR060W	15:180961	2.95E-25
YLR193C	15:180961	1.67E-31
YJR035W	10:503030	8.29E-95
YPL175W	15:174364	3.09E-35
YMR016C	2:427675	1.33E-36
YMR016C	3:90610	2.68E-14
YNL177C	15:113260	1.43E-14
YKL008C	2:612245	1.70E-30
YKL008C	12:634225	3.40E-33
YBR132C	2:499889	4.01E-186
YLR354C	12:829705	1.65E-31
YLR141W	12:433958	2.79E-43
YOR158W	15:179289	1.52E-20
YPL023C	16:511406	2.85E-70
YNR022C	15:113260	9.58E-24
YDL024C	2:565216	3.92E-20
YLR054C	3:90610	2.65E-14
YLR054C	12:247898	2.49E-47
YDL127W	4:963769	3.52E-21
YDL127W	8:111683	6.39E-24
YOL131W	2:533268	6.69E-17
YOL131W	15:170945	1.74E-39
YNL077W	7:375499	5.93E-91
YOR033C	2:479166	7.22E-15
YOR033C	3:90610	6.74E-14
YNL180C	14:263964	6.31E-46
YLL019C	15:180961	1.62E-44
YJR016C	3:100213	1.42E-55
YLR070C	15:174364	3.55E-33
YCL049C	13:49894	1.28E-22
YBR091C	2:553812	5.04E-22
YDR511W	4:1455509	7.76E-20
YDR511W	15:106266	1.22E-20
YLR395C	15:136324	1.23E-23
YLR395C	16:500354	2.63E-32
YNL128W	4:68082	4.41E-19
YOR193W	15:704062	1.57E-101
YIL055C	2:555787	2.12E-20
YIL055C	9:251495	1.95E-29
YDR108W	15:596079	3.41E-13
YNR029C	14:689939	1.53E-51
YDL101C	4:297977	2.42E-18
YPL159C	12:672779	6.15E-22
YDL083C	2:519049	4.50E-17

YDL083C	5:200854	3.87E-16
YGR256W	7:994645	2.81E-29
YCR087C-A	5:194883	1.48E-14
YNL093W	15:180961	5.58E-40
YJL219W	10:23505	1.79E-45
YCL061C	3:90676	6.59E-14
YLR353W	2:521415	2.70E-34
YLR273C	12:677957	3.87E-24
YJL095W	2:553812	8.68E-19
YJL095W	12:662627	1.69E-25
YPR004C	15:108577	1.93E-15
YPR004C	16:500354	2.38E-17
YLR445W	12:1023795	4.33E-28
YJL145W	2:553812	3.86E-45
YFL037W	2:507282	1.52E-17
YHR008C	12:672779	2.67E-26
YIL061C	9:242934	7.15E-48
YGR019W	2:555596	4.83E-22
YIR027C	2:555575	1.29E-48
YIR027C	13:91085	2.64E-21
YIR027C	16:500354	1.28E-16
YJL050W	10:380085	1.57E-61
YMR175W	15:174364	7.76E-33
YOL158C	9:45863	8.78E-33
YOL158C	13:910381	1.81E-19
YOL158C	15:10427	1.27E-38
YDL205C	4:89821	3.82E-175
YGR055W	2:555596	1.54E-38
YGR055W	8:167504	5.02E-16
YPL124W	8:111683	1.99E-18
YDR098C	15:180961	4.53E-37
YLR357W	2:519049	8.16E-20
YGL226C-A	7:69250	1.75E-26
YDR275W	12:644082	2.05E-19
YIL047C	2:521415	9.86E-24
YIL047C	15:154309	2.64E-33
YMR031C	15:174364	2.32E-65
YLR345W	15:180961	2.85E-48
YDL121C	15:136324	3.72E-21
YCL069W	11:656099	5.20E-82
YGL129C	15:179289	6.88E-17
YOL126C	5:117046	2.03E-68
YJL178C	10:96190	3.54E-55
YPR094W	13:124876	2.71E-17
YDL239C	2:519049	1.99E-44
YOL025W	2:555575	1.20E-23
YPL244C	16:500354	5.87E-17

YPL228W	2:562415	8.19E-15
YOL092W	15:154309	3.30E-97
YBR005W	2:252550	3.16E-22
YBR005W	12:677957	2.63E-23
YOR388C	10:59959	1.19E-34
YNR032W	8:111683	5.35E-21
YDR148C	15:180961	1.77E-31
YNL195C	15:174364	1.03E-26
YAR015W	1:141181	1.83E-30
YEL039C	5:79653	1.69E-27
YOL044W	2:514035	2.56E-15
YOL044W	15:174364	2.17E-17
YDR309C	13:91085	9.82E-21
YPL091W	2:427683	5.63E-14
YPL091W	16:368296	1.00E-82
YMR271C	2:553812	3.62E-24
YER027C	15:96633	1.90E-17
YGR111W	15:180961	7.59E-28
YAR033W	1:185122	3.90E-66
YNL006W	7:403926	5.48E-32
YNL330C	16:500342	1.11E-15
YLR448W	2:521415	7.93E-20
YLR448W	5:200854	5.73E-20
YCR061W	12:672779	8.47E-20
YGR247W	7:995892	1.23E-163
YCL017C	3:92157	7.12E-97
YGL141W	2:530481	1.30E-16
YML028W	12:644136	4.27E-18
YIR035C	12:659357	6.05E-18
YIR035C	15:154309	2.83E-15
YHL034C	15:170945	1.53E-21
YNL125C	15:144659	2.80E-19
YNL125C	16:500342	3.46E-19
YCL057C-A	15:180961	6.96E-33
YCL057C-A	16:500342	3.37E-14
YGR012W	3:201166	1.19E-21
YGR012W	7:524216	2.22E-107
YGL061C	8:111679	1.34E-24
YDR183W	9:133693	4.38E-15
YMR006C	13:273244	1.20E-87
YIR025W	9:403134	5.26E-27
YFR012W-A	6:168342	3.19E-42
YNL185C	2:555787	3.07E-15
YMR121C	2:530481	1.60E-14
YNL327W	2:555778	1.28E-115
YNL327W	4:95527	2.81E-17
YNL273W	8:111683	3.33E-17

YCR060W	13:100048	1.15E-20
YNL209W	2:553812	1.56E-23
YDR339C	15:180961	4.59E-22
YER122C	12:672779	7.61E-33
YLR348C	2:420528	6.59E-19
YLR348C	3:92157	5.88E-93
YDL142C	2:553812	6.53E-22
YDL231C	4:46316	5.85E-245
YDR436W	15:180961	2.63E-28
YHL002W	12:668249	1.71E-20
YIR032C	2:555575	1.45E-47
YIR032C	16:500354	4.90E-18
YIR029W	2:555575	8.88E-55
YIR029W	16:497425	8.26E-26
YHR135C	2:533262	2.46E-20
YOR310C	5:194883	5.28E-15
YMR305C	2:533268	5.21E-29
YDR326C	2:553812	1.79E-25
YDR326C	12:672779	4.35E-15
YDR326C	16:500354	1.34E-20
YPL188W	2:553812	2.64E-16
YPL188W	13:28622	1.14E-16
YPL188W	16:497425	4.85E-24
YDR185C	15:144659	1.34E-30
YOL052C-A	15:180961	6.72E-51
YHL026C	8:63386	2.10E-37
YJR126C	12:635380	1.24E-20
YDR388W	15:180961	3.65E-21
YLR392C	15:180961	1.38E-30
YNR041C	12:672779	1.28E-19
YER002W	15:180961	1.27E-26
YGL187C	13:28694	6.76E-19
YGL187C	16:500354	2.56E-29
YDL084W	12:668249	5.86E-21
YOR081C	15:469823	1.78E-80
YER020W	5:200848	1.22E-104
YLR177W	15:180961	8.56E-40
YDR423C	4:1318073	5.54E-116
YNL052W	15:141627	6.95E-16
YNL052W	16:497425	4.13E-23
YBR297W	7:1081978	4.85E-14
YDR131C	2:553812	5.59E-27
YJL161W	10:122312	8.58E-25
YJL161W	15:180961	1.55E-29
YJL161W	16:497425	4.37E-19
YMR277W	2:530481	6.52E-22
YMR277W	13:850520	1.40E-16

YMR110C	12:672779	1.14E-16
YBL035C	15:174364	5.64E-24
YJL181W	12:693790	6.34E-18
YDR089W	4:582121	1.59E-68
YLR065C	12:288943	1.39E-26
YOL039W	15:170945	2.18E-19
YOR154W	15:594024	1.33E-40
YNL026W	14:614342	3.03E-28
YJL066C	15:180961	1.07E-34
YDR248C	13:100048	8.00E-27
YDR265W	4:963769	2.78E-30
YBR299W	7:1081945	1.98E-51
YMR250W	15:180961	3.65E-50
YHR213W-A	6:28029	1.08E-39
YPR201W	3:90610	2.76E-15
YPR201W	11:649300	1.83E-54
YPR201W	16:932535	7.93E-44
YDL243C	3:90610	1.79E-13
YNR033W	15:174364	2.49E-26
YJL183W	2:553812	3.41E-20
YJL183W	10:122312	1.17E-26
YOR110W	8:111680	3.63E-21
YOL108C	15:96633	2.72E-16
YPL277C	13:910381	2.87E-47
YPL277C	15:1065719	2.88E-25
YPL249C-A	2:519049	1.16E-15
YNR019W	12:662627	6.36E-92
YGR248W	7:974644	1.18E-36
YGR248W	15:180961	1.36E-45
YER019W	2:609055	1.24E-21
YER019W	5:183958	8.26E-20
YHR117W	13:149075	4.51E-26
YOL060C	15:205104	6.19E-60
YGL018C	15:180961	6.71E-19
YPL031C	16:497425	1.29E-23
YLL026W	15:174364	2.17E-34
YFR007W	15:180961	4.03E-17
YGL071W	2:507428	1.16E-14
YHR202W	2:555575	2.17E-28
YKR070W	11:574956	7.44E-50
YKR070W	12:677957	8.54E-17
YGR286C	1:21628	6.21E-22
YGR286C	5:272258	3.41E-34
YCL047C	5:420595	1.11E-23
YGL173C	14:449639	1.87E-98
YLR057W	5:201477	1.35E-20
YLR057W	12:254693	5.27E-30

YLR057W	16:497425	4.47E-18
YIL154C	12:644082	2.33E-21
YNL200C	15:174364	1.34E-35
YKL077W	12:642137	8.75E-20
YMR194W	2:553812	1.33E-26
YMR194W	5:193876	7.69E-17
YOL146W	15:48335	5.17E-72
YKL126W	11:209627	2.48E-38
YMR182W-A	2:555787	8.55E-17
YKL094W	1:41483	3.22E-19
YMR302C	13:910741	3.65E-16
YKR036C	11:510933	3.12E-52
YOR354C	15:113260	8.22E-20
YNL100W	16:500354	1.08E-17
YHR020W	15:174364	3.06E-26
YLR023C	2:508843	6.23E-21
YDL160C	2:419093	1.10E-13
YDL160C	14:449639	1.97E-68
YBR171W	2:565216	7.21E-21
YGL049C	7:402851	2.62E-57
YBR103W	2:480009	1.43E-55
YBR196C-B	2:608310	5.57E-154
YPL108W	15:180961	9.80E-29
YKR046C	12:672779	3.10E-145
YJL026W	12:469156	2.59E-82
YGL207W	7:110807	1.37E-72
YOR066W	8:111683	4.37E-16
YOR066W	12:681096	2.20E-16
YOR066W	13:99585	9.27E-29
YPL015C	9:98955	5.00E-17
YPL015C	15:180961	5.11E-23
YIL093C	15:180180	1.09E-13
YER045C	3:90610	1.63E-16
YER045C	15:144659	4.93E-32
YML076C	13:99675	3.37E-65
YML076C	14:410244	3.71E-18
YER161C	14:449639	7.96E-32
YDR533C	15:144659	2.80E-136
YLR168C	12:501510	1.01E-45
YDL214C	4:46316	7.38E-33
YDL214C	15:174364	2.91E-22
YPR082C	15:594024	1.24E-16
YPL167C	16:239627	4.34E-208
YBR104W	2:427674	1.24E-30
YBR104W	15:170945	1.45E-27
YOL136C	13:149075	1.96E-19
YIR018W	9:387985	9.47E-40

YNL124W	14:410244	4.04E-26
YPR098C	2:427683	1.76E-20
YIL116W	9:141014	7.88E-84
YPR157W	9:251495	1.66E-20
YJR155W	2:570229	7.46E-18
YML100W	15:180961	1.65E-49
YDR286C	9:251537	9.08E-15
YBL112C	4:1471859	7.44E-21
YBL112C	12:1067089	1.43E-16
YIL037C	8:111683	2.97E-47
YIL037C	9:197948	1.71E-25
YDR153C	3.953472222	1.19E-22
YPL148C	16:266023	7.87E-42
YGR043C	15:174364	9.80E-53
YJR108W	10:627628	1.15E-159
YDR025W	2:489202	4.96E-21
YDR025W	5:200854	7.27E-19
YCR020C	2:555575	2.17E-16
YHR108W	2:427683	4.84E-12
YLR442C	8:111683	1.09E-21
YKL020C	12:713644	2.60E-21
YHR091C	2:533262	2.03E-19
YOR386W	2:553812	1.52E-33
YOR386W	15:180961	6.32E-26
YLR277C	2:548401	9.06E-15
YLR194C	12:681096	4.69E-20
YIL011W	5:350744	3.54E-80
YIL011W	9:341216	2.37E-29
YIL011W	12:634227	2.09E-16
YOL166W-A	7.374305556	3.06E-38
YIL033C	15:180961	1.17E-40
YGR157W	5.397222222	2.58E-13
YJL159W	2:569420	3.30E-20
YML066C	13:129925	5.11E-91
YPL011C	2:565216	8.34E-18
YPL011C	15:144659	2.35E-18
YPL011C	16:533282	8.23E-35
YDL120W	3:105042	1.49E-16
YER173W	13:28694	5.35E-17
YER091C	5:272258	1.70E-37
YMR148W	15:180961	5.77E-43
YKR017C	11:482069	1.19E-71
YLR215C	12:582499	8.21E-56
YMR109W	13:481094	8.17E-66
YNL284C	15:113260	8.00E-15
YJL172W	10:99921	5.01E-38
YGR222W	7:952065	5.10E-28

YOR327C	10:548177	7.75E-45
YJL045W	2:521415	1.18E-14
YJL045W	10:380085	6.58E-40
YLR102C	15:136327	2.35E-37
YOL166W-A	3.133333333	4.22E-60
YPR085C	16:711614	2.28E-77
YJR104C	12:672779	8.43E-22
YAR028W	1:185122	2.27E-169
YOR138C	15:594024	1.60E-42
YOR107W	9:133663	1.37E-17
YOR107W	15:136324	1.21E-21
YOR231W	3:100213	4.20E-18
YPR023C	11:220519	3.55E-18
YPR023C	16:600664	1.90E-57
YLR406C-A	12:899898	2.81E-50
YDL207W	4:70901	3.33E-39
YGR158C	7:823424	2.19E-39
YIL142W	9:79823	5.89E-35
YPL183C	2:553812	2.72E-18
YGL006W-A	7:497987	9.88E-44
YDL247W	11:652684	2.01E-13
YAR064W	6:28041	9.56E-29
YLR183C	8:111683	2.38E-19
YGR037C	1:11638	2.09E-21
YJL116C	2:555596	1.89E-29
YJL116C	10:218798	6.25E-39
YDL150W	2:553812	1.50E-19
YDR166C	12:672779	3.00E-15
YML056C	15:180961	1.08E-29
YNL264C	12:642137	5.92E-31
YOL083W	2:521415	2.10E-20
YGL126W	15:180961	1.01E-17
YGL206C	14:502316	2.28E-16
YPL041C	3:90610	2.05E-13
YPL041C	8:111680	7.40E-19
YPL041C	12:705190	9.12E-26
YHR092C	9:101011	3.12E-17
YHR092C	15:180961	1.80E-25
YML128C	15:180961	2.11E-57
YEL032W	4:1344670	1.89E-31
YLL018C-A	15:180961	4.81E-46
YNL050C	2:592863	9.58E-20
YNL050C	14:553129	6.19E-23
YJR003C	15:180961	1.35E-49
YLR157W-C	12:472165	8.19E-291
YBR115C	2:477206	5.86E-179
YBR115C	5:251262	7.28E-14

YPL231W	12:644136	5.16E-25
YOR127W	2:533268	5.53E-26
YOR127W	3:90610	2.67E-15
YCR018C	3:105042	2.47E-115
YPL187W	3:201166	1.47E-227
YOR173W	15:180961	1.05E-57
YPL201C	16:90266	2.85E-32
YMR288W	8:111680	1.31E-23
YNL072W	8:111683	9.58E-22
YER010C	13:99585	1.43E-25
YDR441C	4:1344670	1.43E-26
YGR125W	2:555596	1.70E-46
YCR089W	8:111683	6.06E-67
YOR207C	15:180961	3.67E-29
YHR177W	12:689217	2.99E-15
YDR507C	2:533268	8.26E-24
YNL282W	2:553812	5.72E-22
YPL277C	13:910741	2.35E-49
YCR031C	2:519049	1.03E-16
YJR005C-A	10:453011	5.95E-20
YJR005C-A	13:885665	1.02E-25
YPL211W	15:180961	3.12E-24
YNL081C	15:179289	4.54E-27
YPL001W	9:101011	1.51E-13
YMR223W	12:662627	2.43E-20
YDR351W	3:90610	3.25E-12
YDR351W	15:174364	1.73E-25
YER131W	2:489202	2.40E-21
YER131W	5:196196	3.49E-17
YMR113W	12:677957	1.17E-17
YMR113W	13:481542	4.18E-60
YGR040W	2:567221	1.87E-51
YGR040W	3:100213	1.28E-16
YGR003W	7:502131	7.12E-48
YDR005C	4:465337	2.55E-22
YJL191W	5:200854	3.15E-28
YKL170W	11:103658	7.91E-30
YER153C	2:555596	5.09E-68
YIL089W	7:707950	1.11E-49
YIL089W	9:196145	2.11E-205
YIL089W	12:957108	3.05E-17
YFL036W	15:180210	1.90E-16
YLR373C	2:514035	5.96E-19
YPL257W	7:790857	1.57E-18
YPL257W	16:70847	6.06E-75
YDL197C	4:114155	1.94E-50
YJR131W	10:646911	6.68E-46

YJL213W	10:34098	2.77E-161
YHR183W	13:77684	8.14E-50
YNL279W	4:963733	1.24E-25
YNL279W	8:111683	7.88E-62
YBL052C	8:111679	1.62E-18
YAL008W	15:174364	5.78E-24
YHR068W	15:180961	1.62E-32
YFR041C	6:238758	1.82E-32
YOR150W	15:594024	3.12E-15
YMR155W	2:555575	8.18E-25
YMR155W	16:497425	7.53E-28
YHR015W	8:137221	1.42E-95
YBR179C	2:562415	2.45E-19
YGL053W	1:187544	3.35E-59
YGL053W	7:403626	0
YBL045C	13:99585	2.04E-23
YBL045C	16:497425	6.48E-22
YLR188W	16:428900	9.77E-33
YGL116W	8:111680	1.26E-24
YPR009W	16:555416	4.35E-45
YPL230W	15:180961	4.68E-46
YLR153C	12:662627	4.94E-112
YLR203C	15:180961	4.02E-40
YNL326C	8:71742	8.13E-21
YDR321W	4:1108558	4.27E-56
YDL202W	15:113260	6.35E-14
YJR093C	10:604478	1.24E-52
YMR038C	12:672779	3.87E-36
YOR233W	15:179289	1.49E-24
YPL171C	2:553812	5.66E-21
YPL171C	15:144659	4.77E-41
YDL169C	2:553812	4.48E-22
YDL169C	16:500354	1.20E-15
YMR255W	2:553812	1.13E-34
YMR255W	16:497425	8.38E-21
YJL020C	10:393261	4.06E-41
YDL226C	4:46316	2.14E-77
YOR283W	15:842027	6.54E-130
YJL132W	2:565216	1.57E-15
YJL132W	10:159479	9.42E-26
YGL223C	7:73452	2.15E-19
YGL223C	8:111680	5.56E-23
YPR115W	8:111683	1.64E-40
YPR115W	15:180961	1.20E-23
YJL060W	2:555575	3.65E-17
YJL060W	10:330165	2.13E-23
YJL060W	16:497425	1.49E-21

YML116W	14:486861	3.98E-22
YFL027C	3:201166	3.76E-126
YJL052W	15:180961	2.12E-22
YIL172C	2:602012	5.10E-15
YIL172C	9:33795	8.60E-22
YIL172C	13:100048	8.20E-29
YFR003C	8:95469	4.67E-21
YGR271C-A	15:136327	9.42E-23
YFR013W	15:136327	3.67E-24
YBR068C	3:100213	2.98E-33
YHR136C	13:27644	3.33E-148
YHL008C	8:95313	3.33E-53
YHL008C	10:43261	3.23E-20
YML002W	13:273244	8.91E-197
YJL117W	10:185319	2.83E-17
YJL117W	13:27644	3.23E-62
YMR027W	14:449639	4.91E-43
YNL286W	14:104765	2.45E-33
YGL077C	15:174364	3.95E-17
YLR229C	2:537314	6.59E-28
YLR229C	12:611854	3.22E-17
YBR026C	2:301671	3.18E-129
YKL109W	15:180961	1.06E-33
YHR033W	8:167504	1.08E-134
YOR177C	15:683415	8.43E-34
YDR500C	2:521415	6.00E-21
YNR032C-A	13:124876	2.59E-16
YPL079W	2:553812	2.90E-26
YPL079W	5:200854	3.72E-18
YMR198W	8:92960	2.39E-12
YPL233W	16:109106	4.41E-23
YPL028W	12:644082	9.69E-70
YDR047W	3:91977	3.15E-20
YDR047W	15:89217	8.98E-36
YOL135C	12:668249	8.04E-28
YLR438W	13:77684	3.44E-95
YHR007C	8:92960	1.56E-32
YHR007C	13:54913	8.98E-17
YHR007C	15:170945	1.28E-26
YCR004C	3:90610	9.08E-17
YCR004C	13:77684	1.13E-24
YCR004C	15:174364	2.52E-35
YKR051W	12:683463	2.24E-20
YMR065W	8:111683	1.57E-69
YDL003W	8:111683	2.14E-17
YIL014C-A	9:325320	0
YIL014C-A	12:956534	6.56E-16

YLR241W	12:634227	4.21E-21
YLR082C	12:264911	1.55E-32
YGR221C	7:916675	9.72E-16
YDR260C	2:553812	1.94E-23
YDR260C	16:500354	2.04E-17
YGR155W	15:180961	7.20E-18
YLL035W	15:59733	3.90E-19
YER060W-A	13:79786	2.63E-25
YJL065C	2:573491	2.65E-18
YLR061W	2:507282	3.03E-27
YLR061W	5:200854	3.71E-16
YNL146W	3:201166	2.25E-111
YGL262W	7:10170	1.50E-50
YKL050C	2:553812	2.37E-23
YOR222W	15:945781	1.15E-27
YNL230C	2:427683	4.45E-14
YNL230C	3:100213	1.47E-15
YGL226W	3:100213	3.71E-14
YDL073W	12:672779	5.67E-16
YDL073W	13:28622	4.05E-19
YDR151C	7:402841	9.93E-31
YDR342C	15:180961	1.48E-62
YOR296W	2:553812	1.80E-52
YBR107C	2:480009	9.63E-99
YMR001C	2:553812	6.21E-30
YLL007C	12:145780	2.37E-165
YNL183C	14:486861	8.27E-28
YGR169C	15:180210	1.65E-16
YEL024W	12:672779	7.06E-30
YEL024W	15:136324	3.58E-17
YEL024W	16:500354	1.46E-23
YCR096C	3:209932	5.43E-111
YLR389C	12:919638	1.08E-33
YML053C	2:555787	1.25E-14
YNL215W	14:246135	2.63E-20
YPL109C	15:180961	1.01E-20
YFL007W	14:449639	1.76E-68
YPR161C	15:174364	4.29E-24
YJR092W	2:553812	6.13E-43
YPR088C	12:672785	7.80E-21
YGR220C	15:180180	1.14E-20
YHR163W	9:196145	2.82E-15
YHR163W	13:77684	5.43E-26
YHR009C	8:137221	9.66E-31
YHR009C	15:174364	1.34E-32
YLL001W	15:179289	1.06E-27
YGL213C	3:209932	1.88E-17

YGL213C	14:449639	1.11E-28
YDL022W	15:180961	7.23E-35
YNL207W	14:235423	4.27E-27
YIL124W	9:101011	4.58E-22
YIL124W	15:180961	1.10E-40
YOR162C	15:632894	2.65E-68
YLR142W	2:555575	1.20E-26
YLR142W	13:49894	2.19E-25
YLR142W	16:500342	3.93E-31
YDL183C	15:180961	1.55E-37
YDL019C	15:144659	1.30E-47
YIL153W	9:79820	1.89E-22
YDR194C	2:555787	9.72E-21
YDR264C	4:963769	2.49E-82
YIL146C	2:555596	3.94E-38
YNL003C	12:672779	7.62E-30
YOR163W	13:33501	4.84E-31
YOR005C	15:154309	1.36E-18
YPL070W	3:90610	4.98E-17
YEL076C-A	12:1067121	4.04E-25
YNL117W	2:553812	2.12E-29
YNL117W	16:497425	3.18E-19
YMR300C	2:427674	2.78E-15
YMR300C	13:77684	9.37E-34
YLR288C	12:708594	2.35E-62
YDL206W	4:46466	5.67E-15
YNR068C	2:555575	7.14E-31
YNR068C	4:95527	7.99E-17
YBR006W	2:246129	1.75E-65
YLR361C-A	12:851826	7.76E-115
YPL172C	15:180961	3.73E-21
YPR089W	2:555787	1.11E-23
YDL059C	13:33501	1.03E-18
YBL055C	2:142262	1.09E-27
YMR101C	2:565216	1.28E-21
YLR093C	12:327131	5.82E-55
YAL065C	1:10152	1.33E-74
YIL103W	9:191491	1.27E-22
YOL119C	15:106152	2.13E-35
YDL144C	2:533268	4.29E-17
YJL210W	15:144659	1.71E-37
YJL210W	16:500342	9.60E-25
YDL122W	2:553812	4.68E-27
YDL006W	4:465157	7.45E-27
YNL194C	15:180961	2.60E-53
YDL178W	12:634226	7.10E-18
YML071C	15:170945	3.21E-24

YJL035C	10:380085	2.20E-158
YJL035C	13:87587	3.49E-17
YGL243W	7:52613	9.31E-31
YML031W	2:553812	3.75E-28
YKL041W	11:354466	5.91E-89
YMR272C	15:144659	2.16E-75
YKL026C	2:521415	8.69E-15
YKL026C	15:174364	4.97E-44
YHR142W	2:521415	1.36E-27
YHL009W-A	8:98513	2.73E-55
YER043C	12:681096	9.87E-24
YER043C	15:174364	1.57E-21
YPL022W	16:500354	1.00E-29
YBR130C	2:519049	2.68E-15
YIL088C	2:555596	1.14E-22
YAL049C	1:52943	4.52E-104
YER047C	5:243305	1.85E-53
YIL099W	15:180961	2.94E-29
YMR293C	15:180961	1.04E-17
YLR084C	2:553812	4.49E-36
YLR084C	15:144659	7.23E-26
YLR084C	16:500354	5.04E-26
YPL098C	2:555787	2.94E-15
YMR090W	15:180961	1.20E-47
YIL077C	15:180961	1.76E-42
YIL077C	16:500354	9.91E-20
YMR232W	8:111683	2.84E-60
YMR136W	15:154309	1.47E-47
YDR116C	15:180961	9.62E-19
YIL111W	9:154733	3.98E-56
YIL111W	12:683457	1.55E-24
YNL239W	2:555596	8.61E-39
YMR041C	15:136324	3.09E-30
YMR041C	16:500342	5.76E-17
YIL136W	15:180961	9.52E-59
YDR226W	2:553812	9.13E-20
YDR226W	12:677957	1.24E-18
YLR333C	5:200854	7.41E-15
YPL271W	15:180961	7.91E-26
YPL271W	16:500354	3.56E-24
YMR030W-A	2:553812	3.87E-16
YOR367W	14:449639	2.96E-44
YBR166C	2:567221	3.44E-99
YHR047C	3:91977	1.78E-26
YHR047C	13:33681	2.20E-22
YPR159W	2:517365	9.26E-20
YNL034W	14:591234	1.15E-121

YLR412W	12:957108	1.67E-27
YBR039W	16:497425	8.22E-17
YDR232W	12:644082	5.33E-17
YNL010W	12:672779	5.11E-32
YGL202W	2:551299	4.84E-15
YFR053C	15:180961	4.07E-38
YMR315W-A	13:910741	4.45E-19
YOR317W	15:136324	1.63E-19
YOR317W	16:500342	5.75E-19
YPL268W	13:28694	1.32E-34
YPL087W	2:507428	2.30E-16
YCL073C	11:656099	2.59E-182
YBL098W	12:683463	2.04E-21
YGL146C	13:33501	7.24E-20
YGL146C	15:180961	1.42E-44
YLR156W	12:472165	3.52E-308
YJL190C	15:180961	8.90E-20
YOL012C	8:167504	1.55E-14
YBR189W	2:519049	1.09E-23
YPL160W	15:180961	2.08E-26
YCR044C	12:677957	6.68E-20
YHR150W	8:389050	2.56E-33
YHR100C	15:174364	1.30E-21
YGR038W	7:557230	2.33E-114
YMR070W	12:672779	1.45E-22
YCR033W	2:507282	1.67E-20
YCR033W	12:662627	3.04E-19
YBR001C	2:252538	2.78E-19
YHR190W	12:672779	1.18E-66
YOR358W	15:180961	4.02E-29
YPL163C	2:530481	1.77E-46
YDR359C	2:420528	4.54E-18
YDR359C	3:81832	5.62E-17
YIL137C	14:449639	1.63E-56
YDR510W	4:1455131	4.69E-30
YPL273W	13:910381	1.72E-65
YKL157W	2:533268	3.58E-22
YHR021W-A	8:149652	1.11E-20
YIR008C	9:362631	9.97E-35
YDR304C	8:92960	9.08E-24
YDR304C	12:681096	7.68E-17
YGR013W	2:486640	1.44E-23
YMR289W	13:849969	4.67E-80
YHL011C	15:180961	7.33E-30
YNL046W	2:555596	5.21E-49
YNL046W	14:547072	1.05E-27
YNL145W	3:201166	1.70E-223

YML126C	12:672779	6.08E-103
YHR061C	8:111680	1.28E-29
YJR077C	15:180961	2.58E-26
YJR077C	16:500342	1.04E-18
YPR040W	2:507282	2.34E-22
YPR040W	12:681096	6.13E-16
YGL208W	7:110807	6.33E-69
YGL208W	15:174364	7.14E-18
YOR131C	15:546197	3.27E-26
YIL126W	9:98955	3.09E-21
YIL126W	10:570753	2.74E-16
YCR100C	2:573491	1.38E-16
YGL191W	12:672779	1.60E-27
YGL236C	15:113260	5.12E-17
YDR475C	9:133693	1.13E-15
YMR206W	15:180961	2.57E-22
YBR070C	2:388862	2.40E-33
YGR180C	12:469156	6.40E-46
YAL061W	15:144659	6.88E-31
YLR097C	2:555787	2.59E-26
YPR011C	15:113260	1.10E-18
YJR036C	2:533268	2.42E-21
YJR036C	15:150651	2.76E-21
YLR426W	12:987750	3.58E-100
YKL218C	2:565216	6.07E-38
YMR034C	13:328865	2.80E-74
YPR030W	15:180961	4.65E-28
YIL050W	3:105042	4.14E-15
YDR216W	15:150651	1.08E-62
YLR281C	12:705100	1.79E-42
YBR069C	2:376872	9.92E-42
YPR132W	15:180961	8.38E-18
YKL151C	15:180961	7.01E-41
YBR058C-A	15:136327	4.89E-18
YDR204W	13:28694	1.06E-20
YDR204W	15:180961	3.21E-39
YML081C-A	15:174364	1.55E-24
YML081C-A	16:500354	3.14E-16
YDR461W	3:201166	1.72E-210
YFR029W	5:321618	4.03E-29
YHR162W	13:77684	2.97E-20
YPL222W	15:180961	3.10E-35
YKR019C	11:508110	2.05E-22
YDR284C	2:516889	5.08E-27
YDR284C	12:662627	9.81E-32
YIL118W	9:133693	8.26E-13
YIL118W	15:180961	2.59E-24

YPR037C	5:321708	4.16E-18
YJL204C	10:59959	2.77E-37
YJL204C	12:644082	1.05E-19
YMR062C	2:565216	4.94E-19
YMR062C	13:379975	6.48E-19
YHR216W	6:33688	1.33E-21
YHR216W	13:205921	7.21E-29
YJL077W-B	2:521415	3.55E-19
YNL096C	5:200854	4.04E-18
YKL090W	11:266017	4.91E-129
YKR030W	11:508575	2.98E-23
YOL101C	1:10152	3.77E-51
YOR348C	12:672779	1.93E-28
YOR348C	16:497425	5.25E-30
YIL158W	2:553812	5.64E-20
YIL158W	9:47053	5.82E-68
YDR409W	2:507428	5.51E-16
YGL067W	7:375499	2.76E-33
YPL247C	15:180961	3.39E-57
YDR256C	15:144659	8.92E-30
YFR026C	6:205881	2.13E-93
YCL001W-B	2:553812	1.66E-16
YDR124W	8:111683	1.73E-26
YKR006C	15:179289	5.09E-16
YAL063C-A	1:10152	3.98E-32
YPR114W	2:517123	1.03E-19
YPR114W	16:744590	2.31E-70
YLR100W	12:662627	8.39E-51
YGL017W	12:642137	2.01E-16
YLR143W	15:174364	2.11E-23
YKL181W	2:553812	3.86E-28
YKL006W	2:519049	6.75E-24
YKL006W	5:196196	4.90E-16
YKL006W	9:79793	2.51E-15
YOR176W	15:174364	1.74E-20
YLL066W-B	4:1510883	5.55E-44
YLL066W-B	12:1056097	3.13E-38
YBL029C-A	15:179289	3.70E-31
YBL099W	15:180961	4.46E-31
YBL099W	16:497425	7.21E-19
YMR323W	12:1042072	8.02E-24
YMR323W	13:923511	1.69E-20
YOR089C	15:491172	6.81E-17
YER074W	2:519049	6.27E-22
YER074W	5:200854	4.13E-16
YFR031C-A	2:489202	3.60E-18
YFR031C-A	5:200854	1.15E-15

YFR031C-A	15:170945	6.00E-29
YOL113W	15:96633	5.72E-26
YFL010C	6:100371	2.29E-58
YLL055W	12:22752	2.17E-67
YLL055W	13:28694	2.83E-18
YMR267W	15:174364	7.28E-28
YOR188W	2:553812	1.56E-55
YNL218W	15:179289	6.79E-20
YLR058C	13:77684	1.70E-15
YMR181C	15:180961	9.93E-46
YPR196W	16:932535	2.24E-55
YDR464W	2:553812	4.16E-18
YLR099C	2:481439	3.85E-23
YNL241C	3:92013	4.89E-13
YBL043W	12:672785	1.13E-28
YNL323W	14:33361	5.17E-36
YHR090C	16:495156	2.35E-15
YNL007C	7:375499	1.04E-46
YER017C	15:106266	8.11E-15
YHL033C	15:170945	1.67E-26
YGL209W	2:537314	1.87E-17
YML055W	13:163322	7.76E-39
YDL131W	2:477206	2.37E-18
YDL131W	4:217351	3.03E-25
YKL210W	11:46632	1.07E-28
YCL014W	2:553812	8.51E-44
YCL014W	16:511406	3.20E-18
YHL048W	5.215972222	0
YML111W	3:90610	1.47E-14
YML111W	13:28694	5.46E-48
YOL075C	13:410287	8.42E-22
YKL189W	8:111683	1.13E-35
YBR231C	2:667125	4.15E-27
YDL082W	2:489202	4.09E-20
YDL082W	5:200854	9.90E-17
YJL151C	14:502316	2.47E-17
YAR018C	2:551299	1.23E-16
YDL021W	15:174364	1.29E-29
YDR074W	15:180961	9.38E-44
YKL117W	15:174364	3.63E-20
YNL231C	12:683463	5.51E-18
YDR171W	15:174364	6.02E-32
YOL163W	15:10427	6.01E-183
YDL154W	2:553812	6.54E-19
YDL154W	8:111686	2.77E-17
YBR120C	15:179289	1.04E-16
YOR372C	2:514035	5.84E-21

YMR011W	5:350744	7.82E-33
YGR131W	12:659357	1.96E-21
YDR322W	15:180961	4.61E-20
YKL093W	15:174364	1.86E-27
YDR443C	4:1344670	2.17E-52
YOR382W	13:910381	2.59E-26
YLR332W	15:150651	3.22E-19
YDR019C	13:110808	3.98E-19
YCR008W	12:662627	4.82E-19
YNL317W	2:555575	1.40E-15
YNL317W	16:497425	5.74E-18
YPR133W-A	2:553812	1.95E-25
YDL164C	4:165032	6.36E-19
YKL142W	15:180961	2.43E-36
YML106W	2:553812	4.27E-24
YBL071C-B	2:521415	8.52E-24
YJL156C	13:362346	1.09E-16
YJR005W	2:521415	1.38E-26
YJR041C	15:180961	7.19E-24
YPL038W-A	16:486637	1.03E-66
YKL144C	15:180961	2.74E-25
YER075C	2:553812	7.02E-34
YOR374W	13:99585	1.16E-44
YOR374W	15:180961	1.02E-31
YGL028C	2:555596	3.98E-230
YGL028C	4:95527	1.03E-16
YGR154C	2:565216	4.97E-20
YGR154C	16:500354	7.01E-27
YOL095C	15:141627	8.87E-49
YBR161W	12:611967	5.99E-17
YPR199C	11:656099	1.11E-182
YGR230W	3:92247	9.05E-19
YKR058W	15:180961	1.34E-34
YER076C	5:272258	3.06E-22
YKR095W-A	3:92247	1.15E-27
YKR095W-A	15:179289	4.12E-24
YKL106W	15:170945	2.29E-18
YKR004C	11:421190	4.17E-49
YML068W	2:553812	3.94E-28
YML068W	8:167510	7.38E-16
YJL217W	10:23505	5.10E-260
YFL053W	4.328472222	7.93E-116
YFL053W	10:23409	6.52E-21
YFL042C	15:154309	3.09E-26
YOL016C	2:553812	1.61E-17
YOR276W	9:133693	9.47E-16
YHL047C	6.176388889	0

YGR236C	15:144659	1.12E-35
YPL040C	2:555787	6.33E-15
YPL040C	15:174364	4.22E-21
YFL021W	2:555596	9.77E-38
YGL159W	2:508843	1.87E-20
YGL159W	7:192140	7.04E-30
YHR205W	3:92247	7.49E-15
YHR205W	14:449639	1.15E-38
YKL028W	11:394660	1.63E-37
YML130C	8:167510	3.74E-14
YGR229C	2:555778	1.40E-19
YGR229C	7:952041	2.13E-24
YDL002C	4:463264	3.96E-63
YHR094C	4:201395	1.55E-38
YOR226C	2:481439	2.97E-17
YOR226C	3:92157	3.06E-53
YOR226C	15:179289	2.48E-25
YOL029C	15:179289	2.86E-41
YMR052W	13:379981	9.43E-32
YPL169C	2:513408	3.31E-18
YHR027C	8:167510	2.65E-30
YLR419W	2:553812	1.11E-26
YDL017W	4:463264	8.89E-33
YLR449W	15:180961	1.54E-33
YIR038C	15:150651	3.55E-23
YOR140W	2:548401	1.11E-37
YKL107W	2:553812	3.70E-23
YKL107W	4:100720	3.05E-15
YOR211C	13:78655	2.12E-16
YPL256C	4:95437	1.18E-12
YPL256C	14:486861	2.80E-30
YAL037W	2:553812	4.59E-19
YAL037W	8:111690	2.91E-19
YMR251W	13:910381	4.04E-14
YDR011W	12:672779	3.60E-19
YDR011W	15:632894	3.24E-38
YBR073W	15:174364	1.08E-22
YDR177W	4:782114	2.01E-22
YGL205W	7:110807	3.02E-49
YPL104W	15:180961	1.01E-38
YOR048C	15:180961	1.24E-26
YGR206W	7:913065	1.41E-17
YGR101W	7.374305556	1.57E-14
YKL029C	3:100213	3.76E-19
YKL029C	11:388373	1.03E-51
YPR007C	2:555787	2.32E-19
YPR007C	16:590622	1.82E-63

YPR172W	15:174364	6.31E-27
YHR080C	15:144659	3.18E-44
YBR029C	1:29975	6.33E-14
YIL169C	9:19607	9.11E-37
YIL169C	4.911111111	2.19E-25
YCL026C-B	2:407410	3.39E-16
YCL026C-B	3:76127	2.45E-129
YML058W-A	12:469156	4.57E-36
YJR102C	2:553812	3.26E-19
YMR124W	13:513778	1.36E-45
YNL246W	14:209852	2.44E-93
YMR296C	2:481439	3.11E-19
YML088W	13:28622	1.01E-21
YML088W	15:180961	5.08E-26
YDL010W	4:463264	4.22E-32
YAR042W	1:199858	1.09E-41
YMR127C	2:553812	4.10E-19
YKL071W	8:525664	7.93E-14
YHL017W	12:611854	5.74E-22
YOL137W	15:59733	8.24E-31
YIL048W	9:251537	5.11E-24
YHR084W	8:111683	1.12E-52
YGL232W	2:419093	4.29E-16
YPL232W	12:662627	1.52E-26
YPL232W	16:438365	9.86E-20
YNL131W	15:113260	8.20E-23
YMR100W	12:677957	1.10E-26
YPL135W	5:321708	2.98E-20
YOR286W	15:113260	6.03E-21
YNL115C	15:180961	9.20E-23
YGL064C	2:530481	7.24E-27
YOL056W	15:180961	4.02E-37
YOR311C	5:420595	7.76E-21
YGR289C	7:1081945	3.14E-104
YGL037C	15:180961	9.15E-41
YBR013C	2:252640	4.22E-173
YOL086C	15:89211	4.74E-19
YGL098W	2:553812	1.00E-13
YNL058C	14:502496	1.97E-25
YOR065W	12:672779	6.30E-22
YOR065W	16:500354	1.80E-24
YOR034C-A	2:555596	2.42E-18
YGL032C	3:201166	2.93E-210
YER001W	2:553812	3.05E-28
YER001W	15:136324	4.22E-26
YER001W	16:497425	3.05E-25
YDL171C	4:161196	1.37E-17

YPL052W	15:180961	3.47E-37
YKL101W	8:111683	2.13E-17
YBR274W	2:746476	5.87E-44
YLL041C	13:28694	5.36E-20
YLL041C	15:180961	4.38E-38
YLL041C	16:500354	6.82E-17
YGR147C	2:555575	9.80E-19
YGR237C	15:180961	1.13E-28
YOR204W	2:553812	3.71E-20
YOR212W	8:111683	6.88E-31
YOR212W	13:87587	4.56E-20
YOR383C	13:410287	1.10E-39
YIL148W	9:79823	3.27E-26
YER032W	2:553812	2.30E-19
YDR513W	4:1474479	4.69E-27
YGL060W	8:111683	4.87E-56
YOR352W	15:1031657	8.32E-21
YLR439W	15:180961	7.18E-19
YHR179W	12:662627	1.10E-43
YHR179W	15:154177	1.62E-19
YPR026W	15:180961	6.87E-44
YJL006C	10:451940	2.13E-39
YPL046C	16:462646	3.96E-41
YMR166C	14:486861	3.51E-28
YNL245C	14:209852	1.30E-57
YGR143W	15:180961	5.65E-23
YOL159C	9:19607	2.51E-58
YOL164W	4.911111111	0
YLL010C	12:112275	1.26E-165
YCR106W	6.628472222	2.70E-41
YCR106W	0.868055556	1.62E-39
YCR106W	12:1054302	2.71E-49
YDR312W	15:180961	4.84E-22
YML073C	2:521415	2.14E-19
YML073C	5:196196	1.41E-16
YNL274C	15:180961	6.75E-40
YGL227W	15:180961	1.35E-21
YNL004W	14:486861	9.39E-33
YER048C	2:521415	3.33E-25
YER048C	15:150651	2.04E-17
YOL014W	15:298710	4.56E-201
YER155C	8:111683	5.52E-41
YKL095W	13:99585	6.75E-16
YIL131C	2:553812	3.36E-38
YNL173C	13:28622	1.83E-20
YNL173C	16:497425	1.83E-22
YGR109W-B	7:708028	1.62E-154

YGR109W-B	8:95313	2.35E-15
YGR109W-B	9:196145	3.58E-104
YKL052C	15:150651	3.08E-74
YDL126C	14:449639	2.01E-40
YJR080C	15:174364	7.29E-29
YAL056W	1:41483	8.07E-52
YER188C-A	5:568500	4.45E-76
YNL315C	15:106266	3.58E-14
YFL038C	15:170945	6.06E-20
YNL316C	14:38762	3.79E-102
YDL190C	2:419093	5.99E-22
YDL190C	14:449639	1.15E-41
YPR138C	2:555596	3.93E-26
YOR109W	15:524978	1.03E-46
YBR170C	2:582419	5.61E-27
YBR170C	16:500342	4.60E-18
YHR213W	1:10152	2.83E-24
YOL089C	15:154309	1.63E-70
YOL159C-A	9:19607	2.38E-40
YOL159C-A	15:10529	3.86E-41
YBR052C	15:180961	2.67E-29
YKR097W	2:569420	2.21E-28
YKR097W	11:632952	5.92E-56
YJR030C	10:472146	1.51E-116
YMR312W	13:910381	1.59E-23
YMR312W	15:174364	4.19E-22
YLR176C	12:634227	6.02E-16
YIL071C	2:553812	3.24E-24
YIL119C	9:139462	1.07E-39
YNL134C	14:371953	2.25E-18
YNL134C	15:144659	7.62E-97
YBR148W	2:537314	1.45E-109
YGR127W	15:180961	2.57E-32
YBL087C	15:174364	5.62E-25
YHR028C	2:555575	3.44E-18
YHR028C	8:167504	1.55E-21
YDR317W	4:1109729	5.43E-164
YGR174C	15:180961	3.44E-27
YGL258W-A	2:553812	4.49E-37
YOL121C	2:489202	8.47E-20
YOL121C	5:210999	3.91E-20
YIL049W	9:268412	1.52E-27
YDL193W	4:114155	1.61E-28
YDL193W	12:634226	7.84E-23
YNL208W	4:527458	9.15E-12
YNL208W	5:251262	6.53E-15
YNL208W	15:99389	4.68E-16

YJR019C	15:136324	3.15E-26
YJR019C	16:500354	2.99E-20
YOL147C	15:43153	1.07E-18
YDR267C	12:693790	2.93E-22
YIL051C	13:49894	2.98E-58
YCR009C	8:111690	3.53E-20
YCR009C	9:254745	7.13E-17
YBL029W	3:100213	9.92E-16
YAR071W	13:28694	1.60E-107
YPR133C	14:449639	8.07E-28
YDL111C	4:263770	1.52E-17
YDL111C	12:697260	4.19E-13
YIL152W	9:79823	7.85E-136
YIL152W	11:382548	2.48E-21
YGL075C	7:375499	6.54E-33
YOR027W	7:375499	1.90E-50
YPL074W	13:78655	1.20E-13
YBR085W	12:634227	4.37E-23
YBR085W	15:144659	2.93E-17
YBR085W	16:500342	3.07E-19
YHR025W	15:59733	9.08E-25
YML119W	13:28694	4.64E-34
YHR153C	8:389050	4.20E-29
YDR529C	12:705190	1.20E-15
YDR529C	16:500354	1.63E-20
YDL090C	2:548401	5.23E-17
YDL090C	5:332264	7.58E-17
YDL090C	8:111690	5.66E-18
YKL141W	13:28694	1.25E-26
YKL141W	15:180961	5.69E-29
YKL141W	16:500354	2.86E-17
YIL065C	9:196145	1.63E-41
YNL137C	15:180210	1.89E-19
YBL038W	2:142262	4.73E-32
YNL202W	2:489202	7.41E-20
YNL202W	14:281187	2.73E-43
YJR148W	2:533262	4.91E-15
YJR148W	3:105042	5.10E-18
YAL060W	15:174364	1.16E-17
YDR410C	8:80014	3.65E-20
YGL181W	7:143756	9.37E-46
YPR041W	2:553812	1.19E-23
YOR023C	15:409778	2.06E-24
YIL082W-A	7:708028	7.68E-172
YIL082W-A	9:196145	6.37E-104
YBR156C	2:521415	4.99E-29
YBR156C	8:111680	1.81E-19

YER118C	2:530481	2.96E-46
YPL110C	13:33501	3.63E-37
YGR150C	7:804945	7.07E-29
YGL009C	3:92157	1.13E-135
YKR093W	13:54913	1.59E-27
YKR093W	15:108577	5.07E-37
YDL156W	2:517123	4.55E-16
YML047C	4:963733	6.09E-27
YML047C	8:111683	4.33E-57
YCR077C	2:513408	1.78E-16
YCR077C	3:258303	2.71E-25
YGL184C	8:167504	1.49E-21
YNR069C	2:555575	5.16E-29
YLL042C	12:65842	1.74E-64
YMR047C	2:481439	1.65E-21
YGL045W	7:402851	2.35E-23
YER029C	5:218250	5.34E-94
YEL072W	2:508843	1.73E-22
YEL072W	16:500354	3.27E-20
YHR029C	2:553812	2.22E-14
YHR029C	8:167504	6.46E-30
YGL139W	2:555575	8.72E-35
YMR174C	2:521415	3.65E-21
YMR174C	15:180961	4.62E-32
YAR068W	6:48218	5.61E-29
YKL215C	2:533268	1.79E-13
YCR107W	6.628472222	8.43E-42
YCR107W	12:1054278	8.96E-43
YOR201C	15:715809	3.78E-30
YMR191W	13:649250	2.69E-55
YIL031W	2:553812	5.44E-25
YIL031W	12:662627	1.47E-23
YOL164W-A	15:10427	9.56E-136
YLR219W	15:180961	7.07E-52
YPR158W	7:375499	4.17E-52
YER057C	13:33681	5.13E-24
YOR111W	2:507282	1.77E-28
YJL034W	15:144659	3.58E-16
YNL278W	2:427675	8.55E-21
YNL278W	8:111683	5.42E-25
YER130C	2:489202	5.76E-17
YOR215C	9:244902	4.69E-15
YOL091W	15:144659	2.26E-107
YGR067C	2:565216	1.52E-29
YKL182W	2:519049	1.44E-18
YKL182W	12:644136	1.69E-23
YGL194C	7:139383	1.43E-38

YFL065C	4:1501004	2.82E-28
YKL145W	14:449639	5.54E-46
YHR010W	15:180961	1.29E-23
YIL155C	15:174364	6.14E-34
YKL128C	11:194611	3.27E-28
YGR243W	15:179289	2.60E-43
YLR155C	12:472165	0
YBR034C	15:180961	2.25E-25
YBR072W	9:101011	1.53E-17
YGR102C	15:174364	2.45E-31
YLR154C	12:662627	3.03E-33
YLR285C-A	2:555787	2.56E-62
YJL005W	15:180961	8.23E-36
YML004C	13:298185	9.33E-44
YPL249C	14:486861	1.96E-24
YER082C	15:180961	1.22E-27
YFR047C	2:521415	3.73E-20
YHR107C	2:553812	2.76E-37
YHR107C	8:167504	2.51E-19
YFR046C	16:497425	3.11E-24
YLR066W	9:244902	1.96E-16
YPR154W	12:634227	7.40E-17
YML018C	15:174364	1.55E-21
YDR058C	4:582121	3.75E-24
YLR138W	3:90676	4.22E-14
YOR085W	12:672779	2.76E-25
YKL216W	5:117056	1.28E-80
YPR095C	12:672779	3.16E-26
YLR380W	2:479166	1.12E-13
YLR380W	8:14953	2.49E-17
YOL116W	15:136324	1.34E-29
YDR366C	4:1213416	2.06E-111
YGR278W	7:1063841	1.58E-22
YMR295C	15:106152	5.46E-15
YDR338C	5:350744	3.84E-32
YCR020C-A	15:136327	4.22E-18
YEL070W	5:17399	1.01E-26
YPR198W	11:656099	2.49E-205
YLR042C	2:567221	4.80E-103
YOR051C	15:154309	2.17E-20
YIL063C	9:242417	5.47E-20
YJL107C	10:218798	6.77E-43
YIL101C	9:244902	1.44E-34
YIL101C	15:180961	2.12E-39
YGL157W	12:1054302	7.07E-18
YMR129W	2:521415	8.42E-27
YMR032W	2:553812	7.90E-21

YNL328C	8:167510	4.33E-17
YNL328C	14:33643	1.17E-14
YLR186W	5:194883	7.84E-15
YDR179C	15:136327	1.85E-23
YAR075W	1:201039	1.24E-46
YGL058W	15:180961	4.60E-32
YKL087C	15:180222	5.93E-17
YLR278C	13:255486	6.43E-20
YDR357C	4:1213416	2.32E-62
YMR072W	15:174364	1.04E-21
YGR174W-A	2:481439	3.09E-17
YLL031C	2:553812	2.43E-16
YER067W	15:180961	6.02E-30
YMR173W	2:592863	5.95E-26
YBL106C	16:497425	6.51E-21
YNL035C	14:591234	3.62E-141
YCR105W	6.628472222	2.54E-42
YCR105W	0.868055556	5.37E-37
YCR105W	12:1054302	7.49E-44
YOR304C-A	15:889464	1.53E-96
YJR107W	10:627628	6.57E-30
YER049W	12:681096	1.35E-15
YER049W	15:180961	1.13E-30
YOL073C	15:180961	4.80E-31
YAL043C	2:513408	6.56E-29
YJL062W-A	15:179289	1.79E-24
YLR128W	15:170945	6.60E-19
YHR210C	3:91977	3.34E-17
YHR210C	12:644082	1.17E-18
YHR210C	15:108577	1.13E-24
YHR210C	16:500342	1.12E-15
YJL013C	10:451946	1.86E-62
YBR045C	2:328489	5.35E-114
YDL238C	2:555596	5.49E-56
YDL238C	4:100720	8.60E-15
YJR043C	2:533268	2.32E-23
YER107C	15:180961	2.13E-28
YLR312C-B	12:762930	1.91E-35
YGR097W	3:90610	4.51E-13
YGR100W	2:553812	6.03E-20
YBL030C	15:180961	1.77E-32
YBL030C	16:500342	1.17E-17
YIL007C	12:662627	9.63E-40
YPR113W	12:677957	9.68E-32
YOR227W	3:79091	3.20E-23
YOR227W	15:180961	6.16E-36
YKR045C	11:510933	4.20E-31

YNL097C-A	8:95469	2.33E-17
YER145C	2:499012	1.66E-16
YER145C	13:910381	1.38E-13
YGL248W	7:15891	1.88E-94
YGL248W	13:849969	4.21E-13
YPL014W	15:180961	1.27E-29
YCR086W	3:258303	2.51E-26
YJL170C	3:201166	5.49E-174
YJR127C	15:144659	3.97E-49
YCR046C	15:179289	3.47E-26
YDR218C	12:672779	3.13E-26
YIL023C	9:308848	1.43E-41
YPL213W	2:553812	2.15E-20
YNL040W	2:517365	7.38E-37
YNL040W	14:553117	9.00E-57
YHR151C	12:672779	9.25E-18
YPL008W	12:672785	3.06E-17
YKL179C	2:555596	3.10E-22
YKL179C	16:497425	7.35E-17
YGL106W	8:95313	4.49E-38
YHR039C	12:662627	2.04E-65
YIL035C	2:555787	4.35E-15
YGL241W	7:44996	2.11E-40
YGL241W	8:167510	7.39E-14
YPL064C	16:428612	1.29E-31
YML109W	2:553812	6.82E-25
YDR277C	15:180961	1.36E-28
YNL300W	8:111683	1.46E-22
YDR042C	4:527458	1.77E-20
YLL063C	2:555787	5.94E-23
YJL157C	8:111683	3.04E-78
YMR194C-B	15:174364	3.84E-50
YDL209C	4:80849	4.70E-24
YGR138C	12:644082	5.71E-23
YGR138C	16:497425	9.77E-29
YDR425W	2:555575	4.54E-27
YOR043W	15:409778	1.51E-28
YGR118W	15:106152	3.54E-18
YDR478W	4:1418647	8.96E-32
YGL081W	2:565216	9.27E-22
YGL081W	15:180961	2.38E-22
YPR105C	2:602012	3.39E-33
YDR421W	16:500354	4.47E-26
YOR096W	15:174364	3.40E-24
YDR444W	4:1344670	1.62E-38
YLR406C	2:499889	4.16E-19
YLR406C	5:200854	2.29E-17

YJR061W	4:95437	1.02E-13
YGR257C	3.954166667	3.25E-18
YGR257C	7:994478	1.29E-52
YNL078W	2:582419	1.45E-71
YNL078W	8:111680	2.92E-24
YDR488C	12:662627	3.48E-24
YHR016C	15:180961	3.98E-35
YMR222C	14:449639	1.07E-40
YIL062C	9:242934	1.39E-19
YOR142W	2:533268	1.91E-16
YOR142W	16:500354	6.61E-19
YKR023W	11:484826	1.11E-37
YGR225W	7:954692	3.53E-30
YER180C-A	5:568698	2.51E-46
YLR163C	15:113260	4.60E-14
YPL157W	16:266023	6.36E-53
YKL040C	15:180961	5.90E-22
YMR060C	13:390351	2.29E-36
YLR086W	12:317542	1.98E-41
YKR064W	15:144659	4.60E-19
YHR115C	2:391856	4.89E-19
YHR115C	8:340251	4.16E-66
YGR021W	15:174364	5.71E-30
YJR084W	10:604478	1.07E-50
YJR015W	10:461201	1.46E-212
YGR142W	7:375499	5.08E-63
YPL252C	16:70847	1.82E-25
YKR053C	11:553792	5.35E-80
YNL135C	14:393903	3.95E-23
YHR064C	15:180961	5.77E-24
YJL051W	10:380085	1.59E-79
YGR234W	12:644082	1.75E-74
YGR234W	16:438365	1.45E-14
YNL280C	12:662627	2.50E-33
YLR462W	4:1500947	1.77E-35
YLR462W	12:1067120	1.57E-33
YMR297W	2:553812	2.72E-15
YDR384C	2:477206	4.62E-14
YDR384C	12:689217	3.80E-18
YKL007W	10:548177	6.31E-42
YAR003W	12:672779	6.40E-19
YCR104W	15:589013	2.18E-20
YHR017W	15:180961	4.11E-29
YHL016C	2:479161	2.94E-15
YHL016C	8:71742	2.78E-86
YMR157C	13:566144	1.04E-16
YMR157C	15:113260	3.33E-25

YGL076C	2:489202	4.64E-21
YNL133C	15:144659	4.07E-37
YCL025C	13:87587	1.82E-15
YBR117C	2:477206	5.27E-59
YGL125W	3:100213	8.94E-16
YHR031C	8:167504	3.12E-29
YNL056W	14:502496	2.82E-78
YDR034W-B	15:180961	3.74E-31
YHR022C	2:565216	5.22E-16
YHR022C	13:28694	2.86E-15
YHR022C	15:136324	5.08E-23
YHR022C	16:500342	1.82E-21
YJL037W	10:380085	1.34E-23
YJL118W	15:154309	2.04E-20
YDR296W	15:179289	3.68E-23
YLR085C	12:317542	5.81E-37
YER092W	9:98955	7.56E-16
YOR241W	15:180961	1.54E-42
YBR204C	2:636332	4.85E-29
YLR378C	2:553812	5.47E-27
YPL166W	8:167510	3.95E-13
YOL114C	15:113260	2.07E-18
YKL222C	0.868055556	4.15E-53
YMR120C	11:508110	3.06E-16
YMR120C	13:49894	1.39E-20
YMR117C	13:503713	9.10E-92
YBR038W	2:553812	2.24E-23
YGL183C	7:141949	2.64E-49
YER187W	5:568698	1.66E-222
YDL129W	5:321714	3.73E-18
YOR161C	3:90610	1.43E-17
YOR161C	12:19609	6.06E-17
YOR161C	15:174364	1.46E-54
YOL065C	2:427683	4.14E-17
YMR286W	15:179289	4.25E-22
YER056C	5:272258	2.96E-40
YLR327C	15:180961	9.03E-34
YPL229W	12:672779	1.49E-20
YML085C	13:99675	4.41E-99
YDL079C	4:273846	1.15E-24
YDL079C	15:180961	5.73E-26
YCR043C	15:180961	2.81E-27
YLR096W	2:507282	5.33E-15
YEL015W	2:479166	6.43E-18
YEL015W	14:449639	3.01E-41
YOR298W	2:555787	1.30E-13
YMR177W	15:180961	9.43E-38

YLR414C	12:668249	7.74E-20
YOR274W	1:42489	2.22E-19
YOR274W	9:242417	1.48E-19
YOR274W	13:46084	5.34E-25
YOR274W	15:180961	3.77E-34
YDR492W	7:402891	6.11E-33
YGL234W	13:99585	5.83E-26
YEL057C	5:44617	1.95E-96
YIL087C	7:707608	1.95E-24
YIL087C	9:196145	4.12E-53
YNR060W	12:677957	4.22E-37
YNR060W	15:175594	2.82E-19
YOR180C	8:111686	3.33E-14
YML074C	13:163322	2.19E-26
YDL227C	4:46316	4.53E-285
YPR065W	12:672779	1.92E-58
YGR121W-A	7:790117	1.06E-45
YEL004W	2:565216	5.97E-23
YLR452C	8:111683	2.22E-81
YNL104C	3:100213	3.87E-31
YLR466C-B	4:1510883	1.64E-120
YLR466C-B	12:1067087	1.62E-37
YGR244C	15:136324	9.26E-41
YBR264C	8:92960	1.51E-16
YKR075C	7:375499	6.22E-26
YOL007C	2:513408	1.79E-21
YOL007C	8:111683	2.12E-18
YKL054C	16:500354	2.53E-15
YKL043W	11:354466	2.71E-81
YOL049W	12:642137	5.73E-21
YJL209W	10:51003	1.09E-36
YPL061W	2:565216	3.08E-21
YPL061W	14:402312	9.42E-15
YPL061W	16:511400	3.08E-17
YIL008W	15:136327	5.07E-29
YJR051W	12:681096	1.68E-17
YDL211C	15:179289	1.08E-18
YMR009W	12:662627	3.45E-91
YGL010W	7:460945	2.15E-48
YGL010W	15:174364	3.74E-22
YMR163C	2:553812	1.32E-27
YJR034W	15:179289	9.81E-28
YGR047C	8:111683	5.99E-19
YML062C	8:111679	1.50E-15
YCL055W	8:111683	1.58E-86
YPL156C	8:111680	3.87E-21
YOR237W	12:644082	1.05E-49

YLR180W	9:254745	6.66E-21
YLR180W	13:46084	3.77E-24
YNL021W	14:591234	2.37E-77
YOL141W	15:43153	7.19E-51
YDR451C	4:1407833	1.49E-50
YBL086C	15:180961	2.61E-27
YLR136C	13:922268	4.47E-15
YGR249W	7:974680	2.54E-38
YNL054W	12:697260	1.83E-16
YNL054W	14:502496	7.45E-23
YNR040W	2:555787	3.26E-14
YBL021C	2:163042	7.43E-20
YGL174W	7:187567	5.99E-157
YMR244W	12:662627	2.99E-24
YIL015W	3:201166	8.46E-221
YHR111W	15:180961	1.12E-32
YGR246C	7:994478	5.40E-38
YIL114C	15:179289	3.18E-23
YKL017C	11:421190	5.19E-43
YOR129C	2:553812	4.36E-54
YDR085C	8:111683	3.95E-22
YER117W	15:170945	2.93E-23
YDR391C	2:553812	3.69E-17
YPL088W	16:368296	7.88E-42
YBR191W	2:507282	1.60E-16
YBR191W	5:200854	3.02E-19
YBR191W	15:174364	5.39E-22
YHL004W	15:106266	1.30E-21
YFL060C	3:90676	2.14E-13
YFL060C	6:33688	1.34E-25
YFL060C	11:645253	2.03E-12
YKL016C	13:28694	4.43E-23
YKL016C	15:180961	3.55E-32
YKL016C	16:497425	1.11E-20
YNL277W-A	2:548401	1.27E-17
YNL290W	8:111690	3.76E-17
YNL290W	14:104765	2.68E-41
YMR134W	12:672779	7.46E-102
YMR085W	2:553812	6.64E-18
YCL064C	9:268412	1.21E-17
YCL064C	13:46084	1.65E-75
YLR326W	12:713644	1.81E-67
YIL123W	2:553812	3.88E-27
YAL021C	2:427683	8.62E-14
YKR079C	15:180961	3.19E-30
YJL129C	15:136324	3.16E-27
YPR022C	16:600664	2.40E-35

YHL027W	5:321618	1.47E-13
YDR422C	4:1318073	1.96E-48
YPR074C	2:553812	2.45E-20
YBR162W-A	15:136327	1.48E-25
YNL012W	13:115474	7.49E-24
YGR179C	7:853863	9.83E-24
YBR007C	3:90610	2.32E-14
YBR007C	15:174364	3.20E-28
YBR287W	2:553812	2.60E-15
YAL047C	1:52943	2.34E-20
YDL179W	2:555575	6.84E-19
YDL179W	12:677957	9.15E-16
YMR145C	12:672779	2.48E-23
YMR145C	15:106266	2.46E-18
YDR022C	2:521415	2.26E-18
YOL054W	15:180961	4.31E-34
YJR147W	2:562409	9.06E-54
YOR219C	8:111683	1.43E-25
YPL158C	2:555596	2.94E-25
YKL003C	15:113251	1.15E-14
YDR420W	15:144659	2.49E-24
YBR025C	15:180961	1.19E-20
YGL145W	15:180961	4.14E-33
YJL002C	12:705190	2.21E-21
YNR044W	8:111683	3.59E-96
YLR441C	15:180961	3.75E-23
YOR360C	14:449639	1.43E-49
YOR360C	15:594024	2.29E-16
YKL150W	3:90610	6.52E-15
YIL098C	15:113260	1.10E-17
YGR194C	15:180961	5.97E-36
YGR285C	15:180961	2.60E-25
YCR069W	12:672779	3.36E-34
YLR390W	15:179289	2.72E-17
YDL210W	2:555596	1.15E-44
YDL210W	4:95527	2.82E-15
YDL210W	16:500354	4.83E-16
YKR069W	11:566015	3.88E-97
YJR056C	2:481439	1.26E-15
YJR056C	10:537157	2.26E-27
YDR128W	4:744330	2.48E-30
YPL111W	13:77684	4.68E-52
YLR080W	2:565216	1.07E-34
YML110C	15:174364	2.74E-31
YHR096C	15:180961	4.30E-43
YDR009W	15:842003	3.91E-16
YOR058C	15:438832	1.43E-18

YOR273C	7:402853	3.39E-15
YOR273C	15:144659	6.77E-26
YOR164C	12:668249	5.45E-27
YLR206W	12:553064	5.62E-51
YPR033C	15:180961	1.67E-29
YNR002C	13:99585	3.99E-40
YNR002C	15:180961	1.85E-53
YER152C	2:555596	5.08E-147
YER152C	16:500354	1.80E-18
YDR041W	9:238345	3.55E-16
YGL210W	12:693790	4.14E-18
YDR197W	15:180961	2.04E-46
YLR466C-B	4:1510883	2.21E-103
YLR466C-B	12:1056097	2.12E-39
YDR003W	4:450230	1.59E-30
YAR029W	1:185122	2.90E-55
YAR029W	15:170945	8.43E-28
YLL045C	2:519049	1.13E-18
YBL044W	2:151686	5.67E-221
YGR035C	15:180961	3.55E-35
YER025W	2:553812	3.64E-22
YOR315W	2:555787	9.98E-40
YOR316C	2:555787	5.44E-22
YHR059W	2:530481	2.44E-15
YHR059W	15:179289	3.90E-14
YBL113C	4:1525327	6.41E-20
YBL113C	12:1067087	2.34E-28
YML081W	12:662627	5.80E-20
YNL156C	12:672779	9.60E-89
YKL120W	3:92157	2.44E-110
YKL120W	15:179289	4.61E-23
YKL051W	13:28694	3.99E-42
YKL051W	15:136327	5.84E-47
YBR078W	2:401568	3.17E-21
YML009C	15:113260	2.15E-19
YOR185C	15:180961	1.20E-40
YOL115W	2:553812	3.78E-26
YOL115W	8:167510	1.18E-13
YPL038W	2:555596	3.01E-13
YPL038W	16:486637	4.58E-34
YPL117C	12:662627	3.92E-60
YNL234W	9:139462	8.04E-13
YNL234W	15:136324	1.31E-36
YHR144C	15:180961	1.62E-27
YMR235C	2:507282	1.57E-17
YGL219C	7:69250	2.65E-66
YGL219C	13:28622	6.49E-16

YNL301C	2:521415	3.68E-19
YNL301C	15:174364	8.38E-22
YFL010W-A	6:100371	8.42E-41
YKR089C	8:84437	7.60E-17
YKR089C	13:46084	1.55E-17
YER011W	5:350744	3.14E-30
YER011W	12:644082	2.62E-49
YGR014W	2:553812	1.30E-92
YDL078C	2:521415	5.71E-26
YOR375C	3:92157	2.35E-83
YIL072W	7:707950	3.32E-19
YIL072W	9:214482	6.35E-64
YAR050W	1:199778	4.22E-71
YOL094C	15:154309	8.64E-29
YIR042C	6:226146	4.22E-28
YIR042C	9:437054	1.32E-241
YKR021W	2:555596	3.80E-18
YKR021W	3:91287	1.08E-12
YJR153W	2:609055	5.14E-35
YJR153W	5:350744	5.49E-30
YBL085W	2:519049	6.71E-23
YHR116W	15:179289	4.28E-22
YBR208C	13:100048	6.26E-32
YCL066W	3:201166	8.55E-226
YLR118C	15:180961	3.39E-22
YLR106C	15:180222	2.25E-16
YGL086W	2:567221	1.74E-14
YDR198C	15:180961	5.94E-29
YIL040W	1:52943	5.32E-15
YIL040W	15:580354	6.62E-16
YOL040C	2:519049	5.34E-19
YJL011C	15:136327	3.65E-24
YDR258C	5:200848	4.24E-16
YMR314W	8:167510	8.29E-16
YOL052C	2:507428	5.84E-27
YMR105C	15:180961	1.85E-48
YJL160C	2:569420	5.25E-18
YCR038C	3:201167	4.27E-112
YDR365C	4:1213416	1.59E-177
YDR365C	9:79820	2.00E-18
YMR231W	7.374305556	1.82E-13
YBR181C	15:180961	4.71E-24
YJR105W	12:662627	1.63E-54
YJR105W	15:175594	5.32E-15
YJR096W	2:533268	1.89E-16
YJR096W	15:174364	1.30E-40
YGL020C	7:460945	6.66E-47

YBR192W	2:582419	1.36E-19
YGL105W	2:555575	6.59E-23
YNL294C	3:90610	6.71E-14
YHR004C	12:642137	3.42E-20
YPL072W	2:553812	3.34E-16
YBR083W	8:111683	1.07E-55
YEL049W	1:154328	1.08E-99
YEL049W	13:27644	9.75E-16
YDR406W	15:108577	1.11E-33
YBR185C	15:113260	7.64E-18
YJR086W	8:111683	1.12E-20
YDL100C	12:642137	1.58E-20
YOR079C	15:180961	3.01E-22
YER062C	15:144659	9.88E-49
YHR208W	3:92157	3.87E-126
YFR028C	2:553812	2.41E-32
YCL004W	3:105042	8.59E-37
YCL004W	8:389050	9.80E-15
YMR150C	13:562907	3.64E-55
YLR079W	2:555787	5.15E-51
YBR098W	2:427677	5.07E-49
YJR103W	2:427683	1.14E-17
YJR103W	14:449639	9.23E-56
YHL024W	15:180961	1.04E-24
YHL024W	16:497425	1.10E-13
YMR152W	13:566144	1.20E-55
YMR152W	15:180961	3.26E-27
YDL174C	12:672779	3.80E-43
YGL154C	2:530481	5.17E-18
YFL001W	6:143937	2.00E-22
YJR011C	15:136324	9.18E-27
YKL010C	11:421190	5.94E-49
YOR247W	2:537314	1.07E-52
YJL171C	3:201166	3.02E-41
YJL171C	10:110038	1.34E-48
YOR232W	2:555787	6.46E-20
YOR232W	8:167504	5.53E-12
YJR076C	10:575236	1.93E-35
YPL186C	15:180961	2.30E-51
YKR103W	11:656099	2.08E-299
YPR194C	2:553812	2.75E-25
YPR194C	16:932535	2.61E-49
YER113C	2:521415	3.22E-20
YJL059W	10:388461	2.14E-22
YFL040W	6:57455	1.07E-68
YGL025C	13:33501	1.15E-17
YIR028W	2:555575	1.28E-48

YIR028W	16:500354	4.82E-23
YCL021W-A	3:92157	1.97E-272
YLR095C	2:521415	1.05E-18
YLR258W	2:553812	1.20E-23
YLR258W	15:180961	6.50E-40
YBR101C	7:375499	3.48E-62
YAL041W	2:567221	1.36E-29
YDR044W	5:350744	3.96E-61
YDR044W	12:644082	4.66E-50
YGR152C	2:479161	3.66E-21
YGR152C	7:790857	1.18E-24
YLR412C-A	12:956534	1.04E-14
YHL003C	2:427683	2.41E-18
YHL003C	8:111682	1.88E-57
YPR139C	13:99585	1.25E-33
YMR092C	12:1059925	2.22E-23
YMR092C	13:445622	2.36E-136
YGL130W	12:681096	1.56E-20
YGL130W	15:174364	3.16E-25
YGL263W	12:994739	1.06E-31
YBR193C	2:582419	1.14E-45
YCL040W	3:90610	3.34E-15
YCL040W	15:180961	6.28E-28
YHR203C	2:486640	3.76E-16
YBR150C	2:548401	3.24E-58
YOR288C	15:174364	6.75E-22
YOL088C	15:174364	4.32E-39
YBL061C	15:174364	2.09E-39
YER044C	12:672779	5.69E-80
YGL051W	1:187544	8.98E-58
YGL051W	7:403626	0
YKL068W-A	2:555596	5.27E-20
YLL057C	12:22752	1.65E-39
YNL310C	15:180180	1.30E-16
YBR048W	2:519049	2.94E-18
YLR025W	12:195531	1.01E-42
YML007W	13:245674	5.80E-38
YMR068W	13:390357	5.91E-23
YGL135W	15:180961	2.38E-23
YDR329C	3:90610	7.26E-14
YDR329C	15:180961	1.57E-26
YER060W	2:555787	3.73E-14
YER060W	15:179289	1.81E-26
YGL237C	15:180961	6.25E-36
YDR037W	15:174364	3.30E-14
YAL016W	12:659357	2.73E-26
YOR342C	2:584351	4.38E-50

YDR107C	2:427683	2.20E-13
YDR107C	12:710924	3.93E-16
YDL204W	15:180961	2.67E-58
YER096W	2:553812	8.71E-26
YHR041C	12:909226	6.16E-16
YGL056C	12:662627	4.08E-20
YHR104W	15:180961	1.64E-47
YML034W	16:511400	7.05E-16
YBR182C	2:427683	1.80E-15
YBR182C	3:90610	1.55E-24
YBR182C	9:244902	3.28E-21
YHR023W	2:513408	1.46E-19
YNR053C	2:553812	1.34E-19
YGR212W	7:912558	2.24E-30
YBR177C	2:553812	4.84E-17
YBR177C	15:144659	4.51E-56
YOL058W	13:54913	4.93E-17
YCR092C	10:510007	5.44E-24
YEL075C	4.607638889	5.30E-57
YEL075C	12:1067122	1.35E-14
YML061C	13:149075	4.11E-17
YLR190W	2:553812	8.38E-20
YJL054W	2:507282	7.33E-19
YDR333C	2:553812	1.45E-34
YDL102W	2:553812	1.76E-15
YER018C	5:183958	1.25E-51
YBR180W	2:602012	6.64E-17
YOL100W	15:113267	2.43E-35
YNL256W	15:180961	4.14E-39
YML104C	13:28622	5.90E-28
YOR194C	15:704062	2.85E-55
YOR134W	2:565216	6.79E-16
YIR018C-A	9:386046	2.05E-33
YPL144W	16:266023	2.89E-73
YER072W	13:27644	1.84E-128
YML079W	13:99675	9.14E-25
YPL018W	13:28694	4.47E-42
YAL024C	2:553812	1.76E-37
YPL274W	13:910381	6.98E-43
YIL165C	2:551299	5.36E-29
YIL165C	9:33795	1.16E-86
YPL227C	9:101011	4.88E-16
YER158C	5:420595	4.92E-38
YJR047C	12:634227	1.26E-38
YJR047C	16:500342	1.52E-21
YBR126C	15:180961	2.64E-46
YER185W	5:568500	2.51E-47

YNL277W	2:508843	2.18E-18
YNL277W	16:497425	5.80E-17
YOR305W	9:133693	9.36E-16
YJR062C	10:511213	3.18E-20
YAL039C	12:672779	2.58E-24
YHR035W	8:176994	1.15E-49
YJL105W	12:644082	7.05E-17
YDL124W	4:262796	4.51E-66
YDL124W	15:144659	3.95E-26
YJL078C	2:582419	1.68E-158
YJL078C	16:500354	3.28E-18
YDR135C	15:136327	1.65E-24
YGR195W	15:174364	1.76E-24
YMR036C	13:328865	2.54E-107
YNR014W	15:180961	7.95E-61
YLR329W	12:790025	2.28E-44
YKR102W	11:656099	8.45E-199
YLR420W	12:964989	5.63E-39
YDR063W	15:154309	1.12E-19
YGR231C	15:96633	7.15E-23
YGR007W	12:705190	3.79E-17
YCR107W	2:14291	3.88E-21
YCR107W	10:28306	2.60E-44
YCR107W	0.868055556	2.19E-34
YCR107W	12:1054302	2.03E-33
YDR249C	8:111683	4.13E-19
YNL037C	2:555787	4.53E-14
YOR346W	15:968429	1.06E-97
YGL167C	13:77684	6.69E-19
YLR178C	15:180961	4.90E-72
YFL062W	7:1083095	8.75E-14
YEL040W	15:174364	8.39E-25
YMR316W	2:427683	9.92E-17
YMR316W	3:90610	2.24E-19
YMR316W	13:910381	4.71E-25
YJL122W	2:553812	2.14E-21
YDL198C	2:565216	4.48E-13
YDL198C	4:95437	5.79E-21
YOR114W	15:519776	2.83E-34
YOR114W	16:497425	1.28E-19
YOR020W-A	15:379157	4.00E-25
YJL177W	2:489202	3.69E-20
YJL177W	5:210999	8.11E-17
YJR042W	2:569420	4.24E-16
YKL068W	2:521415	2.12E-14
YKL068W	14:449639	7.00E-48
YML069W	13:99585	8.32E-28

YCL016C	3:79091	1.43E-58
YOR251C	10:387893	6.44E-18
YJR094W-A	5:200854	1.82E-16
YHR160C	15:180961	1.08E-39
YGR207C	7:940716	7.02E-18
YDR070C	15:180961	3.37E-54
YBR050C	15:842027	1.14E-20
YGR049W	12:662627	2.54E-76
YBR196C-A	2:608310	2.57E-158
YIL060W	9:246551	7.12E-18
YIL106W	2:521415	1.11E-20
YIL106W	9:244902	5.41E-17
YIL106W	12:681096	3.51E-20
YIL106W	15:150651	1.68E-23
YKR008W	2:507282	3.87E-24
YOR377W	12:662627	5.87E-46
YPL120W	8:188671	7.67E-19
YCL008C	2:555575	6.23E-13
YCL008C	13:28622	2.77E-17
YBR092C	15:150651	8.52E-21
YJL110C	2:555596	9.90E-20
YEL073C	5:15697	1.04E-34
YBR030W	15:174364	2.08E-24
YDR033W	4:518394	3.86E-134
YDL132W	4:217351	4.60E-23
YDL132W	14:449639	3.45E-35
YGL137W	14:449639	2.71E-35
YOL062C	2:427683	1.21E-14
YDR035W	4:527458	3.81E-12
YNL112W	14:412269	6.72E-56
YMR321C	13:910381	7.88E-65
YBR056W-A	13:77684	8.00E-22
YBR056W-A	14:449639	3.89E-19
YPL174C	15:596079	1.63E-14
YPL063W	13:124876	1.76E-21
YAL063C	5.234722222	2.87E-36
YAL063C	8:525664	1.55E-14
YDR403W	13:99720	3.94E-25
YJL130C	5:117046	4.89E-16
YPL039W	6.628472222	1.08E-21
YPL039W	16:486637	8.33E-96
YLR164W	2:530481	3.14E-25
YLR164W	12:469604	2.39E-43
YML006C	3:91049	6.16E-20
YML006C	15:96849	4.55E-21
YHL001W	2:489202	2.49E-23
YHL001W	5:196196	1.63E-16

YLR114C	2:533268	5.16E-19
YGR255C	15:180961	6.97E-34
YFR010W	6:144755	4.22E-28
YNL168C	2:548401	2.00E-37
YDR482C	1:198265	1.67E-16
YDR482C	13:33681	1.01E-53
YNL159C	12:634227	2.92E-15
YCR035C	2:553812	4.45E-17
YCR035C	15:180961	1.09E-27
YPL123C	2:507428	5.15E-32
YMR217W	15:180961	1.95E-30
YER058W	15:113260	1.03E-15
YFL014W	15:144659	1.91E-78
YFL014W	16:500342	2.99E-20
YJL084C	13:99585	3.67E-24
YNL288W	2:427683	1.97E-19
YGL249W	2:565216	4.19E-18
YLR450W	12:1039501	2.72E-45
YLL012W	12:662627	1.13E-32
YLL012W	15:174364	1.33E-26
YBR191W	15:174364	4.33E-21
YOR011W	15:392765	6.06E-21
YCR107W	6.628472222	2.57E-36
YCR107W	0.868055556	1.19E-43
YCR107W	12:1054302	4.94E-52
YNL299W	2:553812	4.44E-18
YLR350W	12:829693	2.49E-38
YOR118W	12:668249	7.38E-26
YLR438C-A	12:1023795	2.25E-24
YOR234C	5:200848	3.57E-15
YOR234C	15:180961	4.03E-22
YNL059C	14:502316	5.11E-32
YNR065C	3:91287	5.28E-14
YNR065C	14:732029	8.06E-17
YMR285C	2:519049	2.45E-23
YBL040C	2:151674	9.10E-61
YGR060W	12:677957	9.63E-34
YGR060W	15:179289	6.55E-19
YAR023C	1:184243	4.72E-73
YJL001W	10:451940	5.77E-38
YPR160W	15:180961	7.33E-49
YLR411W	12:956534	0
YDR483W	12:634227	2.83E-15
YBR242W	12:662627	1.50E-72
YLR121C	15:154309	4.55E-27
YJL219W	10:22453	3.87E-129
YNL041C	14:553117	7.81E-38

YMR160W	15:180961	2.32E-22
YKR013W	2:533268	1.30E-46
YKR013W	8:95313	1.83E-15
YKR013W	11:482069	1.15E-32
YNR034W-A	15:180961	2.96E-47
YHR048W	3:90610	6.01E-19
YHR048W	12:672779	1.79E-55
YER065C	16:497425	1.49E-21
YHR109W	2:530481	2.41E-13
YKL198C	11:85465	2.51E-15
YHL044W	6.176388889	5.07E-290
YPL004C	10:387893	1.49E-14
YPL004C	13:28694	1.98E-20
YPL004C	15:180961	4.66E-44
YER053C-A	12:672779	1.94E-88
YLR201C	15:180961	8.08E-34
YOL096C	15:154309	4.22E-47
YJL167W	12:672779	2.76E-93
YDR298C	13:28694	9.58E-22
YDR298C	15:136324	1.30E-32
YDR298C	16:497425	4.16E-29
YDR242W	2:555575	1.15E-41
YPL085W	2:530481	3.25E-23
YPL100W	15:180961	3.93E-22
YER119C	2:548401	4.65E-19
YER119C	5:395442	9.67E-22
YMR015C	12:644136	2.61E-67
YHR143W	2:555596	5.10E-241
YHR143W	4:95527	1.57E-17
YPR149W	3:90610	5.36E-16
YPR149W	15:174364	1.67E-50
YKR091W	2:555596	1.75E-23
YIL120W	9:136845	2.89E-29
YGL255W	13:77684	6.31E-29
YGL255W	15:174364	6.41E-30
YBL092W	2:521415	4.89E-19
YBR093C	13:28694	1.19E-117
YAL009W	1:136161	1.30E-67
YHL009W-B	8:80068	9.99E-50
YHL009W-B	16:442503	3.92E-39
YGR061C	13:77684	3.74E-27
YLR047C	12:247886	4.21E-28
YFR049W	15:154309	3.29E-21
YML046W	8:111683	4.79E-58
YLR126C	13:28694	3.49E-15
YLR053C	2:507282	8.43E-33
YGR165W	15:113260	3.01E-18

YCR097W	3:201166	1.29E-225
YIL066C	12:469656	1.60E-60
YBR158W	2:567221	1.30E-173
YMR195W	15:108577	9.15E-28
YFR027W	6:205893	2.44E-74
YJR144W	15:571113	2.83E-20
YJR144W	16:497425	4.68E-21
YMR108W	3:92157	1.36E-39
YMR108W	15:180180	5.77E-22
YIL149C	2:507282	1.80E-23
YBR119W	2:477206	1.27E-74
YBR119W	8:80068	1.94E-16
YFR006W	3:81832	1.25E-16
YFR006W	13:33681	3.46E-26
YFR006W	15:136327	9.20E-42
YFR006W	16:500342	3.51E-19
YGR260W	15:180961	7.79E-20
YEL041W	5:79647	2.25E-54
YEL041W	8:457580	3.21E-43
YBR133C	8:111680	1.13E-17
YMR098C	15:180961	2.15E-19
YDR381W	2:569420	9.40E-20
YDR026C	2:481439	2.45E-18
YLR162W-A	12:472165	5.26E-299
YHR032W	8:167504	5.01E-77
YDR093W	2:481439	1.89E-22
YDR093W	16:500354	2.29E-22
YNL237W	14:209852	1.10E-120
YHR099W	2:507428	3.55E-20
YDR523C	4:1491116	1.44E-23
YOR116C	2:553812	5.94E-31
YOR116C	16:497425	4.85E-25
YOR031W	15:380725	2.27E-110
YJL053W	2:555596	1.05E-16
YJL180C	15:180210	8.03E-16
YFR031C	13:28622	2.38E-21
YFR031C	15:180961	2.31E-22
YFL041W-A	15:144659	2.91E-24
YDR379W	2:548401	2.78E-20
YDR379W	3:81832	2.80E-19
YDR281C	13:28694	3.92E-107
YOR108W	15:488384	1.96E-17
YGL144C	3:91977	4.17E-14
YPL113C	3:90610	1.06E-17
YPL113C	13:33681	7.72E-29
YPL113C	15:144659	1.44E-30
YPL270W	13:28334	4.41E-20

YIL145C	15:174364	6.54E-30
YMR252C	12:681096	7.19E-25
YIL133C	2:489202	4.27E-18
YIL133C	5:200854	1.04E-17
YER132C	15:180961	1.57E-26
YOL030W	15:141627	3.10E-15
YFR031C-A	2:519049	7.01E-20
YOR293W	5:200848	1.36E-14
YNR021W	12:634227	1.30E-20
YLL023C	12:99261	2.45E-49
YPL082C	2:499889	2.59E-39
YNL158W	12:634227	6.82E-18
YGR070W	15:180961	6.13E-34
YOL082W	15:179289	1.81E-49
YGR044C	2:555596	6.09E-85
YGR044C	4:95527	4.07E-18
YGR044C	16:497425	4.73E-19
YML029W	13:99585	7.90E-25
YMR154C	2:555596	1.69E-19
YNL238W	14:245313	4.27E-29
YPR028W	16:618575	2.97E-22
YOR229W	5:420595	9.83E-23
YOR229W	12:668249	1.68E-43
YPR192W	16:932535	1.25E-90

Supplementary Table 5: eQTLs for Fisher's p-value method

Accession ID	QTL chrom:position	p-value
YKR009C	15:143597	1.58E-33
YER059W	2:555596	1.24E-25
YDR481C	3:91305	1.32E-22
YDR481C	13:33681	9.75E-59
YML113W	13:69114	1.03E-28
YML113W	15:174364	1.32E-36
YIL157C	15:154177	4.94E-41
YNR074C	12:662627	1.90E-48
YJL147C	2:555596	1.51E-35
YJL147C	10:123859	1.98E-33
YJL147C	16:500342	2.21E-27
YDL216C	2:533262	1.18E-22
YDL216C	4:68082	1.22E-77
YNL320W	14:33361	4.58E-24
YPL223C	15:174364	3.00E-57
YKL138C	15:180210	1.18E-29
YIL160C	15:174364	7.06E-39
YLR050C	12:238298	6.28E-60
YOL008W	15:298710	3.17E-28
YOR072W-B	14:549682	9.76E-41
YPL253C	16:90266	1.00E-28
YDL048C	9:133693	2.17E-28
YDL048C	15:174364	1.68E-43
YDR056C	2:537314	1.19E-26
YNL083W	15:154177	7.81E-25
YPL154C	15:174364	1.89E-32
YNL216W	14:254155	1.40E-40
YFL034C-B	2:551299	2.19E-36
YEL031W	12:705100	1.84E-24
YBR238C	15:154177	7.15E-26
YGL222C	7:85112	4.87E-89
YHR014W	8:137221	9.02E-26
YLR401C	12:956534	3.89E-22
YOR192C-C	15:703771	5.39E-59
YKL195W	15:170945	3.44E-36
YBR149W	15:174364	1.47E-39
YOR175C	12:662627	2.27E-42
YHL010C	8:95289	6.17E-35
YHR152W	8:111683	5.75E-31
YHR152W	13:46084	2.05E-31
YER035W	5:200848	2.14E-31
YER035W	15:174364	2.06E-31
YPL020C	15:136324	1.94E-26
YPL020C	16:511406	1.75E-23
YLR286C	2:562409	1.42E-92

YJL185C	15:174364	3.11E-33
YMR317W	15:154177	9.71E-28
YIL125W	15:174364	2.41E-34
YGR130C	15:174364	9.12E-47
YDL049C	13:81358	5.52E-24
YBR065C	2:368060	3.09E-53
YJR082C	10:581691	6.55E-32
YMR182C	2:477206	4.69E-30
YMR182C	3.953472222	5.61E-23
YPL264C	5:272258	4.72E-45
YAR033W	1:184405	4.54E-60
YDR447C	15:113267	8.72E-29
YML019W	12:672779	1.81E-32
YKL166C	2:555596	1.45E-28
YNR038W	15:150651	1.17E-26
YJL165C	15:143597	1.44E-24
YDL086W	12:662627	3.15E-81
YKL096W	11:261779	4.06E-41
YKL096W	15:136327	1.11E-48
YEL003W	15:136324	3.22E-27
YKL075C	12:674651	3.20E-29
YHR198C	15:174364	2.68E-42
YMR212C	12:659357	1.38E-32
YLR149C	15:174364	3.33E-47
YOR271C	3:81832	1.62E-51
YOR271C	15:180961	2.50E-32
YHR197W	15:174364	3.46E-29
YAL034C	15:174364	5.42E-39
YOR084W	15:174364	6.59E-36
YDR140W	4:744330	9.20E-61
YJL042W	15:170945	2.81E-35
YDR245W	15:170945	1.38E-24
YOR187W	15:174364	3.72E-43
YGL121C	15:174364	3.63E-31
YJL186W	13:27644	2.06E-29
YBR285W	2:533262	7.20E-28
YBR285W	15:136327	1.15E-34
YOL028C	15:179289	6.03E-39
YMR220W	12:662627	4.96E-71
YAR002W	1:154328	6.25E-74
YMR049C	15:174364	9.01E-21
YPR106W	2:555596	7.27E-94
YBL031W	12:674651	3.86E-23
YOR124C	14:449639	1.56E-61
YDR072C	2:519049	6.26E-34
YGR110W	15:143597	4.07E-24
YOR394C-A	15:1065719	1.12E-72

YBR157C	2:537314	3.90E-46
YKL192C	15:170945	1.06E-34
YLR283W	12:705100	1.44E-44
YLR151C	12:433955	1.22E-64
YBL018C	2:185450	9.18E-90
YBR169C	15:174364	4.82E-33
YCL068C	3:201166	1.94E-62
YFL049W	6:33688	6.33E-24
YKL167C	15:170945	3.85E-41
YOR264W	2:562409	1.52E-86
YIR039C	9:437054	5.34E-92
YOL122C	15:108577	1.10E-23
YKL208W	3:201167	1.62E-37
YER053C	13:28622	3.94E-27
YER053C	15:174364	1.17E-43
YOL105C	15:113251	3.89E-38
YCL018W	3:81832	1.57E-88
YDR518W	12:672779	6.70E-49
YDR210W	2:513408	2.38E-23
YDR210W	4:878291	1.83E-40
YLR089C	13:57145	3.77E-26
YMR227C	15:150651	8.10E-24
YJL097W	1:42591	1.38E-25
YJL097W	12:697260	7.71E-27
YIL161W	9:33807	1.06E-43
YCL054W	15:143597	4.08E-26
YOL162W	15:10427	1.15E-98
YFL047W	2:537314	1.67E-35
YFL047W	8:111683	1.83E-21
YOL077W-A	15:141627	7.77E-39
YOL077W-A	16:500348	1.69E-22
YDL125C	4:246738	2.81E-45
YGR083C	15:174364	1.70E-30
YOR285W	15:865782	3.41E-46
YBR296C	13:27644	4.10E-67
YML064C	13:28694	4.17E-27
YDL176W	4:150743	2.47E-26
YHR005C	8:111683	1.70E-49
YCR088W	14:449639	5.01E-50
YPL215W	15:174364	1.24E-24
YGR279C	7:1048152	1.50E-70
YML023C	13:243624	1.30E-31
YGL196W	2:553812	1.15E-33
YGL196W	7:139173	5.84E-38
YBL089W	2:50915	2.02E-61
YBL089W	4:1474482	1.61E-23
YHR124W	13:110808	7.90E-27

YDR231C	15:170945	2.73E-32
YHR053C	2:562415	1.52E-58
YGL104C	7:312740	5.28E-38
YMR169C	15:144659	1.02E-55
YOL071W	15:170945	1.80E-36
YDL012C	4:433589	1.76E-89
YHR191C	8:389050	3.78E-22
YOL011W	15:301076	1.75E-29
YPL002C	16:555416	4.54E-85
YKL194C	15:174364	6.99E-35
YHL020C	8:56246	2.16E-43
YGL179C	7:167587	9.87E-57
YHL032C	15:174364	3.56E-45
YKL140W	11:174008	1.04E-37
YPL127C	15:113251	1.27E-28
YKL211C	11:46633	2.86E-26
YBR295W	13:46084	4.11E-25
YBR295W	15:174364	3.26E-33
YKL085W	15:174364	6.52E-41
YMR048W	13:371857	1.54E-83
YLR312W-A	12:757807	1.06E-54
YML091C	13:49894	1.67E-43
YLR299W	2:592989	1.70E-30
YLR299W	12:662627	1.77E-23
YMR128W	15:150651	4.90E-26
YPR155C	15:143597	1.19E-47
YOR338W	2:553812	1.51E-24
YLR137W	3:79091	2.17E-28
YJL196C	1:41483	1.07E-35
YJL196C	7:402879	2.84E-31
YOR179C	2:551299	3.41E-26
YMR131C	15:174364	4.09E-23
YNL055C	14:502496	5.22E-55
YNL055C	15:143597	6.16E-31
YBR128C	15:143597	2.35E-24
YCR094W	15:141627	8.76E-25
YDL108W	4:273846	3.66E-38
YOR042W	15:170945	2.29E-27
YGL252C	7:15891	1.20E-73
YDL244W	10:24469	2.26E-34
YMR221C	15:180222	5.07E-23
YBR215W	2:658746	5.16E-44
YBR215W	12:662627	4.70E-24
YLL027W	12:86369	8.05E-42
YBR084W	16:387239	1.88E-29
YKL132C	11:194611	4.80E-48
YJL200C	2:477206	6.02E-27

YJL200C	15:170945	2.90E-22
YKR092C	11:650334	5.12E-22
YMR283C	15:170945	1.24E-25
YDL168W	4:161196	1.96E-97
YGR088W	15:144659	1.09E-53
YMR135C	15:144659	3.36E-50
YDL085W	15:174364	1.89E-28
YDL236W	12:662627	2.79E-38
YPR184W	15:174364	5.96E-50
YHR046C	8:193175	9.10E-53
YHR046C	15:154177	5.03E-24
YPL078C	13:49894	2.58E-26
YPL078C	15:154309	3.64E-39
YPL078C	16:500342	3.14E-25
YBR095C	2:489202	7.89E-25
YBR095C	15:174364	1.30E-22
YKL135C	11:178411	1.57E-40
YDL223C	15:174364	3.85E-40
YNR067C	2:555596	1.28E-92
YBR186W	2:562415	2.05E-27
YAL067C	1:10152	4.81E-91
YDR516C	15:174364	3.84E-29
YMR115W	13:494170	3.44E-36
YOL066C	15:210839	6.38E-48
YFL052W	10:23505	1.47E-88
YEL051W	12:644082	6.72E-29
YDL055C	2:555596	4.09E-70
YOL090W	15:113251	8.79E-46
YML124C	2:567221	2.73E-55
YML124C	16:500348	8.97E-27
YOR062C	15:445656	1.55E-59
YOL036W	15:174364	1.32E-24
YPL067C	16:428900	8.44E-29
YOL098C	15:113251	3.42E-27
YGR178C	14:449639	3.35E-74
YOL021C	2:537314	2.09E-30
YMR008C	13:79760	1.18E-26
YMR008C	15:150651	3.01E-23
YJL133W	10:159479	7.71E-25
YDR079W	15:154177	3.86E-34
YDR454C	15:154177	2.73E-25
YIL166C	9:33795	1.15E-84
YFR038W	2:551299	7.18E-29
YML125C	12:662627	1.26E-31
YML125C	15:150651	3.45E-28
YML125C	16:500348	1.45E-25
YHL001W	15:170945	1.34E-24

YDR440W	4:1344670	6.44E-43
YDR460W	3:201166	3.26E-84
YLR313C	12:757807	6.45E-77
YDR062W	15:141627	2.07E-24
YOR178C	15:174364	3.03E-43
YJL016W	10:404508	1.21E-68
YJL218W	10:23505	1.38E-91
YDL218W	2:551299	1.20E-23
YDL218W	4:95527	8.59E-37
YGR071C	12:662627	2.15E-23
YLR256W	12:662627	1.20E-82
YEL021W	5:117056	1.64E-96
YLR343W	12:811669	8.20E-50
YHR097C	8:111683	8.33E-22
YHR097C	13:46084	1.02E-31
YHR097C	15:174364	2.46E-42
YGL253W	15:174364	7.44E-24
YOR390W	13:910381	3.47E-29
YGR258C	2:537314	3.33E-27
YGR258C	15:143597	8.81E-27
YJL108C	10:214773	2.94E-46
YJL108C	12:659357	2.36E-25
YDL155W	2:517123	2.92E-24
YER103W	15:170945	8.97E-25
YIR026C	9:403134	8.75E-38
YEL062W	2:507282	1.05E-31
YEL062W	5:30057	1.24E-27
YGR204C-A	7:905017	4.75E-46
YMR088C	2:551299	1.21E-34
YJL205C	15:143597	5.23E-30
YLR052W	15:175594	2.51E-25
YNL011C	15:136327	5.87E-24
YJR121W	15:136327	1.88E-44
YDR539W	4:1510883	7.79E-93
YNL313C	15:170945	6.86E-26
YKL116C	11:222724	9.10E-79
YNR020C	2:555787	7.69E-22
YNR020C	15:180222	2.44E-25
YPL259C	2:519049	3.05E-22
YEL036C	2:519049	2.37E-23
YEL036C	15:143597	1.15E-26
YBR240C	13:54913	1.78E-31
YPR178W	12:662627	1.46E-25
YCR059C	13:57145	1.62E-23
YCR059C	15:174364	4.48E-31
YHR087W	15:174364	6.64E-50
YHR038W	15:180222	4.21E-27

YOR266W	15:842027	8.69E-31
YOL063C	15:205104	3.02E-70
YLL028W	12:35970	4.64E-36
YLL028W	15:144659	9.18E-92
YHR005C-A	15:180222	4.25E-25
YKL059C	2:569420	2.96E-24
YPR055W	15:106266	3.72E-26
YFL059W	6:48218	3.31E-28
YCR098C	3:301446	1.81E-66
YHR043C	8:193175	7.74E-78
YPR048W	15:170945	4.96E-26
YOR321W	12:713644	8.00E-39
YOR321W	13:404546	3.02E-29
YGL089C	3:201166	5.12E-97
YDR243C	1:51324	3.81E-60
YCR073C	15:174364	3.29E-41
YDR262W	4:975086	1.38E-88
YOL109W	15:136324	3.84E-47
YLR248W	15:174364	1.52E-24
YDR530C	4:1503330	1.50E-27
YDR530C	15:143597	7.09E-25
YKR056W	15:150651	6.46E-22
YML007C-A	12:659357	4.67E-23
YJL166W	15:174364	1.84E-31
YER013W	15:132423	9.14E-37
YGL013C	15:141627	2.59E-25
YOL112W	2:533262	1.73E-22
YHR001W	12:668249	7.43E-26
YML067C	13:129925	9.18E-38
YPR015C	15:136327	9.32E-26
YPR015C	16:500348	7.05E-23
YIL094C	2:477206	8.35E-33
YEL076C	12:1067122	1.31E-30
YER183C	5:568566	6.73E-30
YDR001C	4:446125	2.54E-24
YOR032C	13:100048	1.68E-23
YPL097W	15:154177	2.32E-25
YGL143C	15:174364	7.41E-29
YMR280C	15:174364	2.24E-41
YLR218C	15:170945	1.21E-34
YJR032W	10:471555	5.20E-24
YJR032W	15:174364	2.55E-31
YIL162W	9:38608	6.54E-37
YJL125C	10:159479	1.33E-23
YLR233C	12:607076	5.12E-53
YDL103C	3:92247	5.96E-22
YDL103C	4:273846	8.73E-39

YHL018W	13:81250	3.77E-27
YOL032W	15:255015	3.79E-38
YJL100W	12:662627	4.00E-42
YGL001C	12:662627	1.34E-70
YPR200C	11:655678	5.64E-51
YPR200C	16:932535	2.34E-33
YJL068C	2:427674	1.69E-21
YGR272C	15:136324	8.90E-32
YMR279C	12:469156	8.12E-60
YDL057W	2:555575	1.46E-35
YGL038C	2:603790	1.65E-22
YDL105W	4:273846	4.92E-88
YOR028C	15:174364	1.80E-42
YLR265C	12:674651	1.92E-83
YNR043W	12:662627	1.78E-77
YMR237W	2:419093	6.93E-28
YPL036W	16:489143	1.13E-47
YDR353W	2:553812	9.04E-23
YDR353W	13:234541	2.83E-26
YOR125C	15:563943	5.14E-38
YKL027W	11:388373	1.35E-46
YPL189W	16:182953	7.49E-40
YBR112C	2:427677	8.27E-33
YBR251W	15:174364	7.99E-29
YBR291C	2:480009	5.66E-27
YAR014C	2:565216	6.42E-48
YPL134C	16:256358	4.59E-39
YGR092W	8:111690	2.88E-23
YJR074W	10:575236	1.39E-30
YCR017C	15:141627	1.59E-24
YKR044W	15:170945	8.08E-36
YFR045W	15:174364	5.60E-51
YDR540C	4:1511257	1.38E-91
YPL017C	15:174364	6.14E-43
YGL169W	7:187179	1.18E-97
YDR345C	4:188469	1.36E-28
YGR198W	12:662627	9.44E-24
YGR198W	15:683415	5.41E-33
YIL042C	15:113251	6.61E-25
YLR364W	12:852066	7.08E-23
YML123C	13:27644	5.61E-88
YCR083W	15:193911	5.40E-29
YHR011W	15:174364	1.96E-23
YOR090C	15:491178	3.08E-41
YPL258C	16:70847	1.63E-67
YJL140W	10:151310	5.67E-43
YDR416W	15:594024	7.21E-23

YGR205W	7:913065	5.62E-92
YLL033W	12:514835	4.72E-33
YBR197C	2:608310	4.85E-95
YLR020C	2:551299	4.22E-24
YLR020C	12:662627	1.12E-24
YPR062W	12:672779	8.35E-32
YLR260W	12:672779	4.33E-50
YIL044C	9:238345	1.39E-44
YOR003W	2:555575	4.25E-32
YOR003W	12:662627	3.39E-39
YNL233W	8:111690	6.94E-23
YKL035W	13:46084	4.63E-29
YKL035W	15:174364	5.20E-41
YGL057C	7:403626	1.23E-70
YOL061W	15:174364	2.77E-30
YER124C	2:555596	9.60E-94
YBL068W	15:174364	3.26E-41
YPL272C	12:662627	1.14E-26
YMR318C	13:910381	1.57E-26
YKR088C	12:674651	1.66E-29
YGR168C	13:69114	1.10E-26
YGR168C	15:170945	3.57E-26
YDL199C	13:28694	2.51E-25
YDL199C	15:174364	2.13E-37
YKL045W	9:74540	5.72E-24
YKL045W	11:354466	1.98E-63
YIL009C-A	8:111690	2.33E-22
YFL030W	15:143597	1.93E-47
YER139C	5:458085	4.92E-78
YLR139C	12:423789	9.84E-74
YPR193C	12:662627	7.23E-45
YOR319W	15:930110	2.96E-39
YLR237W	12:635380	6.06E-40
YKL162C	15:136327	8.58E-31
YDR100W	15:143597	1.85E-30
YPL245W	16:84943	6.00E-44
YHR036W	8:176994	5.67E-36
YJL056C	10:327858	1.79E-66
YAL017W	15:174364	2.80E-37
YOL097C	15:174364	2.45E-39
YKR087C	11:599170	5.22E-90
YJL083W	15:193911	1.01E-29
YLR433C	8:93002	1.72E-25
YGR266W	2:530481	9.68E-26
YGR266W	12:662627	1.02E-53
YHR129C	15:174364	1.47E-25
YKR101W	11:645253	8.22E-23

YER093C	12:659357	5.14E-37
YMR156C	13:574867	2.39E-39
YDR090C	15:179289	3.26E-22
YFR033C	12:668249	7.11E-48
YGR287C	7:1063841	1.58E-65
YJL109C	15:170945	3.21E-24
YLR275W	12:679808	4.45E-45
YBR135W	2:519049	1.32E-54
YOR228C	15:143597	7.61E-30
YKL187C	11:97725	3.42E-72
YML008C	12:662627	1.67E-64
YLR165C	12:472165	6.00E-89
YFL020C	1:154328	6.04E-61
YCR091W	15:174364	2.44E-37
YKL037W	15:174364	1.81E-31
YBL050W	2:530481	3.04E-23
YMR298W	12:634227	9.37E-37
YHR049W	2:477206	6.92E-27
YHR049W	15:488377	5.51E-25
YDR325W	15:136324	2.78E-24
YMR275C	13:824809	6.15E-46
YDR038C	4:527458	3.60E-98
YPL045W	15:106266	1.94E-23
YMR239C	15:174364	1.14E-33
YPR006C	16:555416	1.78E-87
YMR043W	15:180961	6.15E-24
YLR038C	12:668249	5.90E-27
YIL121W	3:92247	2.00E-24
YIL121W	12:677957	1.05E-94
YER064C	2:506661	3.63E-40
YIL006W	15:136324	1.53E-29
YDR175C	15:174364	3.37E-26
YDR375C	15:174364	1.67E-27
YHR113W	2:537314	6.84E-35
YDL060W	15:150651	3.08E-23
YLR271W	2:481439	1.37E-24
YJL063C	15:180222	2.20E-34
YPR163C	15:170945	6.12E-22
YGL178W	7:187179	9.43E-62
YDR234W	2:477206	2.64E-27
YDR234W	15:174364	1.03E-23
YDL222C	15:174364	5.66E-57
YMR274C	8:111683	1.48E-28
YNL073W	15:174364	1.89E-31
YBR275C	2:746476	2.13E-51
YER093C-A	15:174364	4.28E-37
YKL112W	2:569420	2.18E-25

YIR007W	2:533262	3.29E-32
YIR030C	2:551299	3.48E-29
YIR030C	9:419418	1.13E-42
YKR085C	11:599170	1.94E-43
YGL068W	15:180222	1.96E-25
YCL026C-A	3:75021	4.81E-51
YER170W	15:113251	2.24E-26
YGL259W	2:562415	1.07E-27
YJR099W	10:612602	5.03E-51
YKL220C	12:672779	3.71E-28
YKL220C	13:910381	5.87E-25
YGL254W	7:15891	7.55E-42
YHR051W	15:141627	3.03E-33
YHR051W	16:500348	1.46E-25
YIL073C	13:27644	3.08E-33
YBR162C	2:608310	1.38E-23
YHL028W	9:133693	2.44E-25
YHL028W	13:49894	3.08E-27
YHL028W	15:174364	2.22E-27
YNL265C	13:46084	5.68E-26
YGR197C	15:632882	2.67E-71
YKL022C	11:388373	7.83E-50
YDL031W	15:150651	2.65E-23
YLR360W	2:551299	1.67E-32
YDR337W	15:180210	6.01E-35
YPR125W	2:506661	1.10E-28
YML054C	15:150651	8.64E-40
YJR134C	12:668249	6.99E-24
YPL107W	16:500342	2.41E-27
YKL103C	2:533262	6.15E-43
YKL103C	15:143597	4.42E-28
YLR342W-A	12:815498	5.56E-90
YGL111W	15:174364	1.89E-28
YHR072W	12:662627	9.74E-78
YBR023C	2:537314	1.00E-29
YBR023C	15:143597	4.83E-32
YLR224W	15:136324	3.04E-24
YEL038W	5:79647	6.98E-88
YMR202W	12:662627	5.52E-87
YDR200C	12:659357	5.58E-25
YGR141W	14:449639	5.27E-53
YNL217W	13:46084	5.72E-36
YNL217W	15:174364	3.15E-32
YKL121W	15:170945	1.05E-28
YOL020W	2:533262	1.56E-32
YOR155C	3:105042	5.46E-21
YLR306W	12:705100	1.46E-24

YOR347C	15:170945	4.74E-30
YDR349C	15:174364	8.75E-38
YGL096W	15:132423	1.28E-27
YDL104C	4:273846	5.65E-31
YGR218W	14:449639	9.59E-74
YDL139C	4:211612	4.00E-67
YOR022C	15:380725	4.72E-49
YKR049C	15:174364	7.37E-42
YKR043C	15:174364	2.19E-45
YDR541C	4:1525327	4.60E-31
YGR053C	15:174364	2.46E-34
YER168C	15:143597	5.03E-32
YDR347W	15:170945	7.01E-29
YOR246C	2:537314	3.77E-26
YOR246C	15:143597	2.64E-37
YLR222C	15:150651	2.51E-27
YMR241W	5:116680	1.65E-30
YML075C	12:662627	1.44E-84
YCR052W	3:210748	1.08E-49
YFL020C	1:154328	6.53E-46
YGR166W	13:46084	5.34E-24
YGR166W	15:174364	7.21E-34
YDL046W	15:170945	2.81E-28
YOL154W	15:10427	1.65E-95
YLR040C	3:201166	5.12E-97
YLR457C	2:517365	1.70E-22
YHR085W	15:174364	1.90E-28
YGR213C	15:154177	3.85E-24
YHR062C	15:174364	7.08E-27
YLR231C	12:644136	2.05E-69
YOR047C	4:201395	1.64E-27
YDR370C	2:565216	8.41E-22
YOR353C	15:154177	2.01E-30
YDR490C	15:170945	4.65E-26
YFR039C	15:174364	1.67E-27
YBR151W	15:632894	2.50E-37
YOL077C	15:174364	8.23E-26
YPL019C	13:27644	5.39E-84
YPR140W	15:170945	5.93E-28
YDR502C	12:662627	1.64E-72
YKR104W	11:656099	3.60E-98
YDL025C	15:150651	2.48E-23
YOR389W	13:922258	5.35E-36
YER136W	12:634226	1.02E-25
YPL026C	16:500348	2.82E-23
YGR149W	2:555575	2.78E-24
YLR376C	12:872448	4.07E-75

YDL089W	4:289639	2.37E-73
YOR080W	15:106164	2.80E-24
YGR208W	13:57145	9.00E-23
YGR208W	15:174364	1.91E-25
YBR131W	15:174364	6.01E-34
YPR010C	15:174364	2.56E-25
YDR292C	12:705100	4.75E-22
YDR292C	15:170945	4.72E-28
YMR010W	15:174364	2.24E-27
YOR238W	15:798209	9.84E-31
YIL115C	9:141014	7.03E-44
YDR528W	15:150651	2.45E-27
YNL111C	12:662627	1.11E-65
YMR078C	2:533262	4.07E-26
YCR090C	15:136324	9.65E-24
YNL152W	2:514035	9.81E-23
YGL055W	7:403626	6.03E-32
YGL055W	12:659357	1.18E-40
YMR225C	15:180222	6.05E-39
YER089C	2:530481	8.49E-34
YML131W	3:175808	1.23E-24
YML131W	13:124876	1.40E-25
YAR027W	1:185122	5.77E-90
YNL149C	13:115474	2.14E-22
YOR099W	12:713644	6.78E-30
YLR239C	15:174364	2.99E-27
YNL161W	15:170945	7.88E-26
YPL132W	15:174364	3.53E-48
YER088C	15:174364	4.19E-53
YCR093W	3:258303	6.38E-26
YJL139C	10:151310	1.77E-65
YAR031W	1:185122	1.66E-67
YER101C	15:154309	1.95E-37
YNL242W	14:191183	1.14E-58
YNL113W	15:143597	5.53E-24
YDR395W	15:174364	8.95E-27
YAL064W-B	1:10152	1.77E-91
YDR520C	12:662627	4.65E-28
YOL104C	15:132423	2.36E-91
YIR031C	2:551299	8.22E-42
YIR031C	9:419418	8.22E-31
YPL141C	8:111683	2.00E-23
YDR067C	4:582121	1.26E-59
YDR067C	14:402312	5.15E-37
YDR524C	4:1484268	1.69E-30
YBL067C	12:662627	6.29E-28
YDR213W	12:662627	4.62E-41

YGR089W	12:659357	1.80E-33
YDR036C	4:509817	1.94E-37
YKL161C	15:143597	1.91E-30
YDR297W	12:662627	1.63E-56
YFR025C	6:206151	2.64E-25
YIR004W	9:369587	9.98E-31
YDR306C	15:150651	4.92E-24
YDR453C	15:174364	6.16E-42
YGR295C	7:1081945	1.99E-97
YOR213C	14:449639	1.52E-48
YDR272W	2:514035	4.09E-27
YDR272W	4:963769	1.59E-32
YPR191W	13:28622	3.40E-29
YPR191W	15:136327	5.33E-32
YPR191W	16:500342	5.10E-24
YNL212W	8:98513	2.74E-26
YLR410W	13:27644	6.35E-37
YGR223C	7:940716	9.38E-88
YOL133W	8:111683	1.16E-24
YOL133W	15:59733	2.95E-42
YPR151C	12:662627	2.83E-76
YML096W	13:79786	1.73E-30
YML096W	15:170945	1.47E-23
YDR506C	2:486640	5.00E-33
YFR002W	2:506661	3.27E-23
YDL133W	12:644136	1.10E-24
YHL048C-A	5:15697	1.16E-47
YHL006C	8:111680	1.54E-49
YJR152W	2:551299	1.13E-52
YJR152W	16:500342	7.24E-28
YDL182W	2:480009	3.50E-25
YER150W	13:46084	9.05E-25
YER150W	15:174364	2.27E-51
YBR154C	2:555596	2.31E-22
YAR008W	13:28622	8.36E-30
YER066W	15:143597	1.30E-27
YGL190C	7:141949	7.27E-29
YDL243C	6:30378	8.65E-60
YDL230W	2:569420	1.11E-24
YDL230W	4:46466	9.99E-33
YAL053W	9:244902	6.36E-23
YAL053W	13:46084	3.62E-27
YAL053W	15:154177	2.56E-24
YJR010C-A	10:461201	2.32E-87
YDR186C	12:659357	2.39E-35
YNL270C	2:477206	2.71E-24
YFL004W	13:27644	1.04E-37

YBR175W	13:46070	1.04E-21
YBR175W	15:174364	4.08E-26
YCL027W	8:111683	3.59E-77
YCR095C	15:136324	1.00E-27
YER163C	5:504714	8.57E-57
YOR101W	15:515923	4.74E-35
YLR244C	12:635380	3.57E-80
YNR037C	15:180222	2.48E-30
YOL047C	2:562415	1.02E-34
YLR172C	15:174364	2.12E-27
YDL130W-A	4:201395	2.43E-25
YDL130W-A	15:174364	3.15E-37
YBR165W	2:565216	3.47E-27
YGR211W	7:913059	1.53E-33
YLR356W	2:567221	1.65E-21
YLR356W	15:174364	2.09E-32
YER054C	15:174364	4.93E-45
YOL022C	15:174364	1.68E-34
YLR049C	12:246579	2.84E-31
YNL045W	13:46084	2.13E-31
YNL045W	15:143597	2.02E-44
YHR067W	2:517123	6.08E-24
YPR118W	15:174364	8.73E-26
YCR057C	15:174364	4.45E-28
YHR143W-A	15:170945	4.27E-27
YML025C	15:174364	2.03E-31
YOL043C	15:248746	1.39E-69
YKL206C	11:25990	1.39E-27
YLR430W	12:990751	1.54E-41
YIL091C	15:174364	5.26E-29
YKL186C	2:555575	4.62E-22
YKL186C	11:97761	3.07E-37
YEL047C	12:659357	4.58E-40
YJL024C	10:398821	1.30E-25
YML057W	13:149075	1.43E-27
YPR020W	15:141627	1.47E-24
YPR020W	16:500348	6.85E-40
YNL252C	15:174364	2.95E-30
YAL005C	1:136161	1.40E-31
YAL005C	15:170945	1.23E-24
YFL017C	15:179289	3.22E-27
YAL029C	15:150651	1.20E-34
YFR001W	15:136327	2.80E-24
YGL063W	7:375499	1.26E-54
YJL131C	15:174364	7.53E-37
YPL092W	16:368296	1.94E-28
YFL008W	6:174552	2.71E-28

YNL160W	15:174364	2.31E-43
YKL072W	15:174364	3.06E-43
YJL164C	15:174364	6.23E-38
YLR204W	15:180222	8.44E-37
YPR141C	2:565216	4.78E-24
YDR147W	12:705100	2.46E-24
YGR239C	5:312672	5.99E-26
YGR239C	12:662627	6.68E-24
YER073W	3:91305	5.17E-32
YMR196W	13:46084	4.04E-24
YMR196W	15:174364	1.56E-47
YJL144W	15:113254	1.96E-21
YBL105C	6.628472222	3.81E-28
YGR031W	15:136324	1.76E-43
YKL124W	11:212278	3.03E-25
YDL109C	4:263770	5.35E-28
YPR060C	15:174364	7.66E-35
YIR012W	15:174364	2.44E-28
YMR055C	13:379981	2.79E-36
YKL084W	15:174364	4.73E-28
YMR170C	2:555596	6.95E-36
YGR201C	15:174364	1.65E-38
YPL043W	15:150651	1.36E-23
YGL185C	2:519049	2.56E-33
YNR050C	2:477206	4.04E-46
YLR270W	15:174364	3.22E-45
YDR346C	12:662627	2.14E-34
YIL070C	15:180210	8.80E-27
YDR246W-A	7:375499	1.77E-30
YJR025C	10:472147	6.31E-73
YKL091C	15:174364	1.53E-45
YKR016W	15:141627	2.04E-36
YKL185W	2:553812	2.83E-57
YOR128C	15:563943	3.44E-40
YDL235C	15:170945	1.25E-23
YGR233C	13:27644	4.39E-72
YEL011W	15:174364	2.24E-43
YJR078W	15:850119	3.59E-31
YDL248W	11:655159	1.01E-22
YFL017W-A	6:100521	2.58E-55
YFL061W	10:703868	2.80E-29
YLR368W	12:851826	4.89E-30
YJR138W	2:555596	8.24E-25
YJR138W	16:497425	1.72E-25
YCL063W	14:449639	2.79E-54
YPL196W	15:170945	1.30E-32
YJR055W	15:174364	9.19E-30

YBL016W	2:199101	2.96E-37
YBL016W	8:111683	2.56E-48
YIL110W	15:174364	1.88E-25
YIL083C	9:191491	8.78E-31
YJR110W	15:106266	1.59E-24
YBR172C	2:555575	4.94E-27
YFL064C	4:1510883	7.98E-27
YBR040W	8:111683	1.49E-60
YNL033W	14:586789	3.98E-57
YKL178C	3:201166	5.12E-97
YGR033C	7:543259	9.66E-32
YGR033C	15:141633	4.64E-33
YHR030C	15:143597	2.96E-24
YGR026W	12:634227	8.57E-29
YLR371W	12:662627	5.22E-29
YKR031C	11:508110	1.05E-31
YOR073W	12:672779	1.55E-23
YKL018C-A	15:136327	1.81E-29
YKL018C-A	16:500342	2.86E-23
YGR217W	2:567221	1.00E-28
YGR217W	15:136327	7.44E-34
YGR217W	16:500348	5.72E-23
YKL133C	14:449639	1.11E-52
YML030W	15:174364	6.59E-43
YOR059C	15:438824	3.09E-31
YKR071C	3:90610	1.63E-36
YKR071C	15:179289	8.34E-28
YLR259C	15:180222	3.15E-25
YCR102C	12:1059818	1.06E-59
YCR102C	15:132423	3.07E-28
YKL148C	11:174008	8.94E-43
YKL148C	15:136327	1.02E-30
YLR319C	2:555575	5.34E-23
YBL037W	2:537314	2.10E-28
YIL104C	7:707950	4.37E-29
YIL104C	9:190866	6.01E-50
YMR063W	13:391767	6.07E-39
YDR178W	15:174364	1.19E-40
YMR208W	12:662627	7.34E-81
YDL066W	2:551299	2.11E-24
YDR202C	15:170945	2.66E-39
YJL137C	13:28694	1.10E-31
YJL137C	15:143597	1.41E-35
YJR039W	15:154309	3.75E-23
YLR179C	12:514835	5.61E-94
YGL136C	7:256953	1.04E-48
YNL002C	15:174364	2.94E-23

YIL014W	9:325320	6.66E-73
YPR122W	8:111680	3.57E-30
YOR019W	15:357194	1.33E-91
YOR250C	15:804686	4.00E-37
YKL067W	15:180961	7.27E-33
YMR244C-A	15:180180	4.97E-41
YIR037W	2:567221	5.00E-21
YIR037W	15:170945	1.50E-27
YEL034W	12:662627	2.07E-87
YGR216C	15:174364	4.71E-24
YPR091C	13:46070	9.24E-24
YPR091C	15:174364	9.54E-37
YJR097W	15:174364	4.66E-34
YMR251W-A	15:143597	1.03E-41
YHL009C	8:93002	3.01E-24
YER177W	15:136327	2.03E-26
YHR018C	2:555575	6.00E-24
YOR038C	15:382531	3.73E-36
YHR207C	15:174364	6.37E-42
YFL054C	6:28029	2.30E-72
YFL054C	10:34086	4.30E-39
YGL160W	12:674651	5.78E-62
YML016C	13:239559	1.45E-58
YGR264C	15:170945	2.48E-28
YOL018C	15:298710	1.04E-45
YJR059W	15:174364	3.87E-29
YER016W	8:111683	4.63E-38
YML070W	13:77684	4.77E-26
YML070W	15:174364	1.23E-30
YGL229C	2:533262	2.86E-43
YLR246W	2:562409	8.30E-27
YLR246W	12:635380	1.33E-26
YDR270W	4:975086	4.53E-48
YBR235W	2:553812	3.69E-44
YGR175C	15:143597	3.21E-39
YKR026C	11:484826	3.85E-37
YKR026C	15:170945	1.58E-29
YAL064C-A	5.397222222	2.14E-60
YKL070W	13:79760	4.03E-24
YJR075W	10:573371	4.65E-65
YLR108C	13:99720	5.52E-26
YLR108C	15:174364	1.60E-28
YMR091C	13:445622	1.98E-40
YJL048C	12:662627	1.69E-72
YOL140W	13:49894	6.93E-27
YOL140W	15:43153	8.84E-27
YDR211W	15:174364	2.91E-35

YML118W	2:569420	6.75E-35
YML118W	13:28622	3.74E-28
YBR097W	2:391856	1.26E-33
YLL029W	12:70121	1.35E-26
YIL034C	15:170945	4.55E-26
YBR228W	2:551299	2.92E-35
YCL035C	15:170945	4.79E-32
YEL020C	14:449639	2.40E-51
YOL006C	15:136327	6.93E-31
YLR175W	15:170945	5.37E-23
YLR251W	15:174364	4.00E-29
YPL221W	2:477206	9.18E-27
YIR036C	14:449639	2.84E-50
YDL181W	15:174364	1.39E-47
YDL181W	16:500348	6.84E-27
YOR035C	2:553812	8.88E-25
YNL224C	15:108577	1.42E-26
YHR054C	8:209167	7.92E-34
YDL093W	12:662627	1.18E-55
YJR124C	15:154177	1.00E-23
YLR242C	15:136324	3.91E-22
YOR195W	15:136324	5.61E-31
YPR128C	1:42633	5.53E-44
YBR102C	15:180180	6.02E-23
YDR276C	15:150651	2.25E-23
YGL101W	12:662627	4.10E-56
YEL007W	9:139462	3.99E-23
YDR096W	15:174364	8.94E-48
YKL062W	15:136327	4.10E-57
YKL062W	16:500348	1.49E-25
YLR342W	12:810883	7.87E-37
YML108W	15:136324	2.68E-33
YAL064W	1:11638	1.20E-58
YAL064W	8:525664	2.03E-27
YER028C	4:226317	2.65E-54
YEL050C	15:174364	4.39E-37
YAL062W	1:10152	2.26E-88
YBR067C	2:376145	2.26E-34
YBR067C	12:662627	1.37E-53
YJR068W	10:572479	9.44E-24
YJR094C	10:602943	1.13E-95
YDR504C	4:1471859	6.89E-41
YDR504C	15:150651	1.12E-23
YAR020C	1:154328	2.07E-44
YGR001C	7:502131	1.68E-50
YNL087W	15:174364	2.21E-38
YPL192C	4:963733	4.74E-29

YPL192C	8:111683	1.55E-54
YKR041W	11:508110	3.28E-27
YBR094W	2:427677	2.34E-57
YPL084W	3:100213	1.25E-21
YDR405W	4:1272737	9.26E-65
YDR405W	15:154309	2.53E-25
YEL065W	13:922258	5.58E-26
YEL046C	15:174364	4.84E-31
YJR149W	10:703868	1.71E-88
YLR073C	12:282091	2.21E-83
YJR112W-A	10:627628	4.19E-67
YEL017W	5:117056	8.07E-49
YBL082C	2:73342	1.26E-34
YPL125W	16:318068	1.46E-41
YIL017C	3:201166	3.86E-55
YBR106W	12:668249	5.87E-25
YBR106W	13:49894	3.64E-24
YFL018C	13:69114	1.10E-26
YFL018C	15:170945	8.14E-29
YBR286W	12:607076	2.48E-38
YJL154C	10:135902	2.77E-42
YIL105C	15:154177	2.87E-34
YNL015W	2:567221	6.30E-29
YNL015W	15:174364	3.54E-34
YJL163C	10:110044	1.68E-29
YJL163C	15:154309	2.76E-34
YPL016W	16:523450	4.56E-77
YKL193C	15:170945	2.14E-39
YNL023C	15:174364	7.94E-25
YIL140W	2:537314	6.81E-25
YNL108C	15:174364	1.55E-29
YKL080W	12:672779	8.72E-29
YLR397C	12:909226	1.25E-32
YLR397C	15:174364	6.26E-26
YLR213C	12:611967	2.03E-38
YLR245C	12:635380	4.19E-79
YBL111C	4:1503330	8.71E-28
YBL111C	12:1056097	2.07E-26
YHR176W	15:144659	5.97E-30
YDR369C	13:99675	2.30E-40
YPR086W	12:668249	8.27E-24
YPR086W	13:28622	4.51E-28
YGR146C	3:91287	2.18E-22
YGR146C	7:402879	4.24E-21
YGR146C	8:111682	4.63E-21
YGL250W	15:141627	1.07E-26
YOL130W	13:33681	2.74E-25

YOL130W	15:143597	2.31E-33
YLR226W	15:170945	3.08E-29
YNL306W	15:180222	4.22E-38
YOR056C	2:555575	2.72E-34
YER037W	5:226503	1.89E-37
YBR262C	15:154177	2.55E-28
YPL083C	16:387239	5.49E-57
YGR090W	15:143597	1.81E-24
YIL030C	15:113251	6.82E-27
YIL030C	16:500348	9.46E-26
YKR090W	11:612769	7.76E-44
YOL155C	9:27026	5.72E-48
YOL155C	6.038194444	1.18E-28
YFL013C	6:144755	5.66E-29
YFL013C	12:796771	4.31E-32
YBL034C	2:565216	1.78E-22
YDR169C	4:782114	7.71E-39
YKR063C	15:170945	4.18E-41
YFR009W	14:449639	1.11E-75
YIR033W	1:41483	3.24E-30
YIR033W	12:662627	1.24E-33
YBR173C	2:582419	3.88E-56
YOR120W	15:174364	1.31E-42
YAL028W	12:634227	1.78E-44
YJR008W	10:450212	7.03E-26
YJR008W	15:154309	4.66E-26
YMR319C	13:910741	8.27E-48
YMR319C	15:174364	1.38E-26
YOL017W	15:298704	2.06E-34
YOL128C	2:551299	3.55E-32
YOL128C	12:662627	2.74E-28
YNR012W	15:174364	3.45E-34
YOR010C	5:350744	9.13E-39
YOR010C	12:662627	3.31E-39
YNR016C	1:42591	7.28E-24
YNR016C	2:521415	2.60E-23
YBL013W	15:144659	1.06E-48
YMR171C	13:597705	4.49E-51
YBL097W	6.628472222	2.03E-20
YHR003C	12:662627	6.32E-36
YOR034C	12:662627	1.93E-31
YOR034C	16:500342	9.97E-25
YNR034W	14:695114	3.02E-41
YNR034W	15:113260	5.72E-25
YNR057C	5:272258	1.26E-48
YHR156C	15:174364	1.11E-26
YNR011C	14:554606	4.03E-29

YBR269C	15:174364	3.05E-34
YOR316C-A	2:553812	1.18E-69
YBR230W-A	15:143597	1.22E-21
YEL052W	15:174364	2.86E-44
YEL055C	15:174364	1.15E-33
YIL046W-A	9:268352	3.17E-38
YDL213C	15:174364	4.76E-31
YGR052W	15:144659	2.32E-67
YDR356W	4:1185630	5.62E-51
YJL187C	8:111690	2.41E-22
YJL206C	2:555596	9.71E-24
YJL057C	10:327858	7.39E-79
YGL004C	15:144659	1.27E-27
YDR430C	15:170945	4.45E-34
YPL278C	13:922258	1.11E-49
YOL151W	15:143597	2.04E-26
YMR236W	2:537314	4.22E-28
YLL013C	12:126934	5.40E-88
YPL005W	16:600658	5.87E-30
YER109C	2:555575	1.11E-28
YIL108W	15:143597	5.17E-24
YJR113C	12:672779	1.44E-23
YGR041W	2:555778	2.00E-88
YCR048W	12:668249	1.16E-39
YCR048W	15:113251	1.53E-25
YEL029C	15:170945	3.69E-31
YER154W	2:584357	2.95E-22
YDL067C	16:500348	2.90E-22
YLR383W	12:881579	2.31E-30
YKR027W	3:201166	2.49E-29
YER046W	5:243215	2.94E-65
YIL169C	9:27026	1.15E-49
YIL169C	6.038194444	1.03E-27
YBR003W	15:174364	1.82E-34
YOR306C	15:154309	7.10E-25
YOR040W	15:170945	2.60E-27
YDR165W	15:174364	1.99E-26
YMR229C	15:174364	1.15E-24
YMR058W	13:922258	1.96E-30
YBR183W	12:659357	1.83E-40
YNL132W	15:174364	1.07E-23
YKL130C	11:194611	1.37E-39
YBL005W-A	2:246129	7.38E-29
YKR034W	2:551299	3.58E-43
YMR264W	15:136324	4.69E-24
YHR037W	8:185882	1.88E-26
YHR037W	15:113251	9.06E-26

YHR037W	16:500342	1.44E-41
YER095W	2:573491	1.04E-25
YDR179W-A	15:136324	2.07E-24
YNL146C-A	3:201166	5.12E-97
YOR126C	2:562415	2.22E-36
YDR038C	4:527458	2.35E-27
YOR002W	12:668249	8.70E-27
YGR109W-A	7:708028	8.86E-82
YGR109W-A	8:92960	9.17E-23
YGR109W-A	9:200332	6.42E-78
YLR069C	15:174364	3.45E-30
YNR010W	12:659357	1.33E-31
YLR295C	13:28622	6.19E-27
YLR295C	15:136327	1.65E-41
YLR295C	16:511406	1.01E-29
YFL026W	3:201166	5.12E-97
YDR367W	4:1213416	1.03E-34
YGL040C	12:659357	1.36E-24
YPL173W	15:174364	2.03E-28
YGR227W	8:111680	4.37E-24
YIL016W	8:111686	4.67E-26
YBL107C	13:87587	1.10E-29
YML078W	13:115474	5.76E-90
YIR019C	9:387985	1.44E-30
YDL170W	15:179289	1.19E-29
YOR278W	2:555596	5.48E-25
YOR278W	16:500342	2.17E-28
YBR259W	15:174364	6.85E-28
YHR077C	15:136324	1.21E-28
YNR052C	14:449639	5.12E-52
YBR167C	2:562415	1.30E-22
YGL164C	2:573491	7.42E-40
YGL212W	7:92896	1.63E-91
YER079W	15:174364	2.18E-25
YHL022C	8:56246	2.31E-59
YER180C	5:549142	1.08E-72
YIR002C	9:362631	1.61E-40
YIR002C	13:100048	4.27E-24
YKL209C	3:201166	5.12E-97
YGR008C	15:174364	5.63E-39
YHR001W-A	16:500348	1.42E-24
YDR060W	15:170945	8.39E-32
YIL079C	15:174364	1.43E-31
YOL069W	13:49903	6.03E-25
YPL118W	15:154177	3.22E-33
YOR384W	13:410287	2.06E-42
YBL015W	15:174364	6.02E-23

YBR198C	2:562415	1.98E-25
YHL040C	13:923511	2.80E-24
YKL212W	15:143597	2.38E-33
YOR363C	1:51324	3.24E-25
YJL014W	15:154177	2.24E-30
YJR004C	3:201166	2.88E-95
YPR134W	2:551299	6.60E-23
YPR110C	15:174364	1.07E-29
YLR014C	15:141633	2.94E-23
YHL012W	8:95289	6.46E-43
YHR214C-E	13:33681	9.16E-28
YHR214C-E	15:136324	4.66E-22
YEL026W	15:174364	1.45E-27
YJR017C	10:451832	1.87E-28
YLR205C	12:662627	1.58E-72
YKL024C	15:143597	3.59E-24
YPL103C	16:252119	1.24E-29
YEL063C	2:555575	1.68E-25
YBR057C	8:111686	1.69E-24
YKL004W	15:150651	4.31E-25
YBR037C	15:174364	4.91E-36
YFR044C	13:77684	2.88E-31
YFR044C	15:141633	3.90E-35
YKR018C	11:508110	1.65E-25
YOL041C	15:174364	1.91E-24
YDR032C	15:174364	4.62E-37
YBR137W	15:116709	6.50E-23
YFR011C	6:168354	9.04E-41
YFR011C	15:154177	2.09E-38
YNL142W	2:555575	2.60E-34
YKL100C	15:174364	4.49E-36
YPL024W	16:500348	1.88E-51
YLL066W-B	4:1510883	1.41E-27
YLL066W-B	12:1056097	9.76E-41
YKL201C	11:47707	1.44E-50
YIL117C	2:533262	3.23E-27
YIL117C	15:143597	9.59E-25
YML087C	13:99675	1.81E-46
YLR382C	15:174364	4.38E-33
YIL164C	2:555787	6.34E-28
YIL164C	9:33795	2.81E-76
YAR007C	1:141181	5.68E-45
YAR007C	13:27644	1.73E-24
YFR017C	15:174364	1.46E-46
YER182W	15:174364	1.87E-46
YER014W	12:662627	1.67E-63
YNL298W	2:537314	2.91E-28

YML129C	15:174364	1.37E-36
YPL183W-A	15:174364	7.52E-37
YPL106C	16:368086	1.88E-52
YFR015C	15:174364	5.55E-27
YEL064C	2:551299	1.63E-33
YLR094C	12:327131	1.78E-94
YHR171W	2:555596	3.74E-26
YHR171W	13:110808	1.54E-22
YER162C	15:143597	3.20E-26
YLR087C	12:317542	4.63E-27
YOR086C	15:174364	2.15E-30
YMR185W	15:136327	2.15E-29
YJR119C	15:846344	1.54E-26
YJL044C	10:353027	3.14E-61
YBR004C	2:252538	1.02E-39
YBR004C	15:174364	3.79E-27
YER063W	2:555596	1.97E-32
YOL080C	15:150651	5.79E-26
YJL092W	2:569420	6.85E-25
YBR230C	15:174364	6.77E-44
YJR010W	10:461201	1.65E-60
YOR012W	15:357194	1.26E-93
YOR148C	15:594024	6.92E-30
YNL098C	15:174364	3.33E-41
YOL002C	7:402851	5.05E-43
YDR514C	2:555575	3.26E-30
YDR514C	15:143597	3.88E-24
YGR112W	15:174364	3.91E-40
YOR205C	2:519049	1.81E-26
YCR037C	13:27644	4.53E-54
YCR037C	16:511406	9.99E-23
YDR300C	15:174364	1.11E-40
YMR107W	15:193911	2.95E-31
YJR049C	12:662627	8.77E-26
YJR048W	12:662627	4.29E-65
YBL041W	2:142262	1.18E-81
YBL033C	12:679808	3.59E-23
YGL224C	13:46084	5.35E-28
YAL036C	15:174364	7.29E-23
YOL143C	5:117056	1.32E-78
YDR508C	4:1456748	9.12E-25
YKL096W-A	15:174364	5.88E-52
YGL197W	7:117900	1.07E-52
YJL046W	10:372838	1.09E-31
YMR199W	13:649250	4.30E-37
YBR298C	7:1075580	6.23E-69
YDR313C	15:170945	4.46E-31

YNL175C	14:314883	2.80E-29
YJL141C	15:154309	1.58E-42
YJR060W	10:548177	1.25E-97
YPL184C	14:449639	5.68E-75
YJL212C	12:662627	1.12E-23
YGR082W	15:174364	3.13E-28
YDL004W	13:49894	4.52E-29
YDL004W	15:174364	1.14E-37
YDL004W	16:500342	1.08E-26
YNL283C	2:537314	1.25E-33
YPL030W	15:170945	1.69E-34
YOL026C	12:659357	1.08E-28
YMR165C	2:517365	1.81E-24
YDL099W	12:659357	1.81E-24
YDL217C	4:78583	3.11E-43
YBR021W	2:301671	1.87E-34
YPR180W	3.956944444	7.30E-25
YCL056C	3:14066	2.91E-70
YBR146W	2:530481	1.01E-24
YBR146W	15:174364	3.68E-28
YKR065C	3:79091	3.73E-22
YKR065C	15:170945	1.52E-33
YEL060C	15:136327	2.98E-26
YGR066C	2:567221	1.58E-21
YHR034C	8:167504	5.26E-37
YHR043C	8:193175	1.28E-75
YOR115C	15:136324	1.41E-24
YLR407W	15:170945	3.51E-29
YJL012C	13:27644	4.84E-84
YGR086C	15:143597	3.47E-57
YMR261C	15:174364	1.92E-36
YJL103C	15:154309	2.86E-38
YIL173W	9:19607	1.17E-50
YIL173W	10:22273	8.78E-73
YPL207W	15:174364	6.42E-41
YLR446W	15:174364	4.00E-30
YBR199W	2:608310	3.29E-56
YIR016W	15:174364	6.79E-46
YOL084W	15:144659	1.18E-75
YMR118C	2:562415	8.48E-23
YPL239W	15:174364	5.86E-37
YOR007C	16:500348	8.32E-23
YOR206W	15:143597	2.12E-23
YEL056W	5:44605	1.28E-34
YBL006C	2:216978	1.32E-53
YNL186W	5:194883	2.39E-23
YBR060C	2:388862	2.30E-38

YFR055W	15:179289	2.33E-33
YFR014C	15:174364	6.83E-45
YGR184C	7:865575	1.60E-38
YKL159C	11:153463	1.83E-29
YFL055W	6:15106	5.31E-43
YFL055W	10:22309	5.56E-75
YHR195W	15:150651	2.38E-33
YIL064W	7:707950	2.49E-23
YIL064W	9:242417	1.14E-46
YHL036W	15:174364	6.33E-29
YLR355C	3:91305	1.30E-41
YLR355C	13:46084	1.14E-37
YHR081W	15:174364	1.47E-24
YOR060C	15:428238	6.26E-53
YIL097W	2:537314	3.39E-23
YIL097W	15:136327	1.26E-27
YJR098C	10:612602	6.27E-55
YNR036C	15:170945	4.06E-31
YNL065W	14:502496	2.16E-63
YGR076C	15:180222	1.20E-36
YBL009W	2:537314	1.21E-33
YNL066W	2:555596	3.11E-88
YDL247W	10:703868	1.60E-33
YMR114C	15:174364	1.80E-34
YKR060W	15:174364	3.58E-30
YBR233W	12:677957	9.91E-25
YLR193C	15:113251	2.27E-32
YJR035W	10:503030	7.86E-68
YPL175W	15:180961	2.94E-39
YMR016C	2:427677	3.35E-38
YNL177C	15:154177	1.58E-24
YKL008C	2:615927	6.92E-34
YKL008C	12:634226	4.39E-37
YBR132C	2:499889	8.60E-91
YLR354C	12:852066	3.47E-33
YLR141W	12:423789	1.09E-62
YOR158W	15:180210	1.66E-30
YPL023C	16:511406	1.03E-43
YNR022C	15:180222	2.43E-36
YLR054C	12:247886	4.54E-55
YDL127W	8:111682	2.06E-26
YOL131W	15:170945	1.38E-28
YNL077W	7:375499	8.53E-83
YNL180C	14:281187	1.55E-48
YLL019C	15:174364	4.45E-42
YJR016C	3:81832	1.18E-53
YJR016C	15:179289	1.06E-23

YLR070C	15:174364	1.76E-33
YCL049C	13:54913	7.96E-32
YBR091C	2:426887	2.59E-30
YDR511W	4:1471859	1.90E-26
YDR511W	15:106164	7.39E-29
YLR395C	15:141627	9.98E-35
YLR395C	16:500348	3.53E-30
YOR192C	15:703769	2.43E-36
YNL128W	4:78583	2.94E-24
YOR193W	15:703771	3.23E-76
YIL055C	2:569420	6.27E-24
YIL055C	9:251537	9.27E-34
YIL127C	15:150651	7.64E-22
YLL008W	15:174364	1.80E-31
YPL159C	12:668249	1.41E-28
YNL093W	15:174364	4.63E-33
YJL219W	10:23409	2.50E-51
YLR353W	2:533268	3.64E-37
YLR353W	12:829265	4.70E-37
YLR273C	12:677957	7.57E-25
YJL095W	12:662627	3.53E-30
YNR028W	12:965218	6.32E-24
YPR004C	15:108577	1.59E-24
YPR004C	16:500348	7.13E-23
YLR445W	12:1023795	1.07E-34
YGR271W	15:136327	1.68E-23
YJL145W	2:562415	1.38E-37
YHR008C	12:668249	1.07E-34
YHR008C	15:150651	1.23E-24
YIL061C	9:242934	1.83E-59
YDL245C	10:703868	1.06E-22
YGR019W	2:555575	5.30E-35
YIR027C	2:551299	8.57E-43
YIR027C	13:110808	2.84E-25
YJL050W	10:345059	4.97E-44
YMR175W	15:174364	3.37E-33
YOL158C	9:33795	1.00E-32
YOL158C	13:910381	2.78E-24
YOL158C	15:44488	5.26E-43
YDL205C	4:89821	1.88E-87
YGR055W	2:551299	8.52E-29
YLR002C	15:150651	4.59E-25
YNL268W	2:477206	2.63E-29
YDR098C	15:143597	9.97E-35
YLR357W	2:519049	2.12E-24
YGL226C-A	7:73452	1.24E-30
YDR275W	12:644136	2.89E-27

YIL047C	2:481439	1.90E-24
YIL047C	15:154309	3.14E-34
YMR031C	15:174364	1.76E-61
YLR345W	15:174364	1.59E-53
YDL121C	15:136324	2.13E-23
YCR030C	15:174364	2.32E-29
YCL069W	11:656099	5.99E-61
YGL129C	15:174364	4.83E-24
YJL178C	10:89348	1.05E-50
YDL239C	2:537314	6.38E-41
YDL239C	4:46466	1.59E-20
YOR262W	15:174364	5.29E-25
YOL025W	2:555596	3.12E-28
YGR030C	7:530473	3.70E-25
YOL092W	15:154309	9.56E-81
YBR005W	2:246129	2.18E-26
YBR005W	12:677957	1.75E-29
YOR388C	10:59959	3.94E-47
YNR032W	8:111690	6.72E-29
YDR148C	15:174364	8.02E-40
YNL195C	15:143597	5.67E-30
YAR015W	1:141181	5.51E-26
YDR309C	13:91085	3.19E-30
YPL091W	16:387239	6.11E-68
YMR271C	2:519049	1.67E-24
YMR271C	15:143597	2.37E-24
YGR111W	15:174364	1.37E-31
YPL021W	16:535979	3.37E-22
YAR033W	1:185122	3.11E-73
YNL006W	7:375499	1.21E-46
YLR028C	15:136324	8.66E-22
YCR061W	12:674651	2.88E-27
YGR247W	7:995892	1.50E-92
YCL017C	3:81832	6.35E-76
YGL141W	2:537314	4.04E-23
YML028W	12:644136	5.14E-23
YCL057C-A	15:174364	2.10E-40
YGR012W	3:201166	2.45E-26
YGR012W	7:524216	3.87E-78
YMR006C	13:277071	1.99E-75
YIR025W	9:403134	8.11E-30
YFR012W-A	6:168342	2.12E-41
YFR012W-A	15:842027	3.05E-28
YNL185C	15:180222	1.09E-28
YNL327W	2:567221	2.02E-84
YML024W	13:227254	3.95E-29
YER122C	12:659357	4.21E-38

YLR348C	3:81832	9.57E-75
YDR303C	15:170945	3.32E-30
YDL231C	4:46316	1.07E-96
YDR436W	15:174364	1.54E-31
YHL002W	12:677957	3.54E-24
YIR032C	2:548401	1.24E-30
YIR029W	2:551299	1.41E-50
YIR029W	16:500342	2.83E-28
YHR135C	2:537314	9.38E-26
YJL102W	15:174364	8.35E-29
YOR310C	15:174364	4.98E-25
YMR305C	2:533268	1.70E-43
YOL048C	15:143597	2.53E-36
YDR326C	2:567221	3.90E-28
YDR326C	12:659357	1.05E-26
YDR326C	15:136327	3.08E-22
YDR326C	16:500348	1.98E-24
YDR185C	15:143597	1.10E-35
YOL052C-A	15:174364	9.28E-51
YHL026C	8:63314	5.59E-33
YJR126C	12:635380	5.80E-27
YLR392C	15:150651	2.53E-32
YNR041C	12:662627	6.59E-27
YER002W	15:174364	3.76E-26
YJL039C	15:180180	7.31E-25
YGL187C	13:28622	1.27E-25
YGL187C	15:141627	7.20E-33
YGL187C	16:500348	1.26E-31
YDL084W	12:672779	4.93E-28
YOR081C	15:481586	1.54E-64
YER020W	5:196190	8.95E-95
YLR177W	15:174364	4.64E-44
YDR423C	4:1318073	1.27E-85
YNL052W	12:668249	8.05E-31
YNL052W	15:113251	1.11E-27
YBR297W	7:1081945	3.69E-24
YDR131C	2:551299	4.23E-26
YJL161W	10:123859	1.50E-31
YJL161W	15:170945	4.43E-29
YMR277W	2:530481	1.76E-24
YMR110C	12:674651	3.09E-23
YMR110C	15:170945	1.16E-26
YLR276C	12:689217	6.86E-24
YBL035C	15:174364	1.94E-28
YJL181W	1:42591	1.14E-21
YJL181W	12:679808	5.52E-23
YDR089W	4:582121	8.45E-65

YLR065C	12:288943	2.19E-34
YOR154W	15:594024	5.08E-45
YNL026W	14:614342	1.67E-38
YJL066C	15:170945	4.02E-39
YDR248C	13:100048	4.85E-32
YDR265W	4:975086	6.95E-53
YBR299W	7:1081978	1.08E-49
YMR250W	15:174364	9.67E-48
YHR213W-A	6:28029	2.66E-53
YLR033W	15:174364	1.92E-25
YPR201W	11:655678	3.53E-52
YPR201W	16:932538	4.28E-45
YMR214W	15:174364	3.15E-25
YNR033W	15:170945	1.06E-35
YJL183W	2:569420	2.21E-25
YJL183W	10:123859	3.66E-35
YOR110W	8:111680	2.11E-25
YPL277C	13:922258	3.96E-56
YPL277C	15:1065809	4.66E-44
YNR019W	12:662627	8.24E-82
YGR062C	15:113251	5.00E-29
YGR248W	7:974625	2.65E-38
YGR248W	15:174364	2.97E-41
YLR196W	15:154309	1.22E-25
YER019W	5:183958	4.66E-24
YHR117W	13:149075	8.24E-29
YOL060C	15:210839	5.75E-65
YGL018C	15:170945	2.34E-26
YPL031C	16:500342	5.53E-27
YLL026W	15:174364	8.74E-37
YFR007W	15:170945	8.50E-29
YGR126W	7:794884	7.08E-30
YNL201C	8:72233	1.66E-25
YNL201C	14:258590	1.54E-29
YHR202W	2:555596	1.59E-33
YKR070W	11:574956	1.25E-48
YGR286C	1:42591	4.89E-24
YCL047C	5:422612	1.01E-26
YGL173C	14:449639	4.48E-82
YLR057W	12:257512	1.67E-40
YIL154C	12:644136	3.55E-29
YNL200C	15:174364	3.39E-42
YKL077W	12:642137	5.17E-26
YOL146W	15:47951	4.69E-66
YKL126W	11:211820	1.95E-41
YMR182W-A	2:562415	2.98E-24
YKL094W	1:41483	6.53E-26

YKR036C	11:510933	3.77E-53
YOR354C	15:170945	7.34E-32
YNL100W	15:154177	7.03E-43
YHR020W	15:170945	1.86E-29
YDL160C	14:449639	5.41E-77
YBR171W	2:567221	2.20E-28
YGL049C	7:402851	4.08E-54
YBR103W	2:480009	2.93E-61
YBR196C-B	2:616262	9.49E-93
YPL108W	15:174364	2.89E-29
YKR046C	12:662627	2.47E-82
YJL026W	12:508029	4.55E-67
YGL207W	7:110807	7.59E-64
YOR066W	13:49894	2.32E-30
YPL015C	9:98955	2.29E-21
YIL093C	15:180222	2.79E-26
YER045C	15:143597	3.05E-35
YML076C	13:99585	3.92E-56
YDR533C	15:144659	3.73E-89
YLR168C	12:450045	7.00E-45
YDL214C	4:78583	1.05E-24
YDL214C	15:174364	4.98E-28
YPL167C	16:239627	2.97E-85
YBR104W	2:427674	1.04E-27
YBR104W	15:170945	7.08E-30
YOL136C	13:149075	1.22E-25
YIR018W	9:387985	7.43E-45
YNL124W	14:412269	4.63E-30
YPR098C	2:427677	6.18E-22
YPR098C	15:143597	1.44E-23
YIL116W	9:141014	3.16E-66
YPR157W	9:251495	1.59E-24
YML100W	15:174364	2.56E-51
YPL012W	15:174364	3.35E-24
YIL037C	8:111686	2.96E-58
YDR153C	3.955555556	7.82E-24
YPL148C	16:266023	8.74E-47
YGR043C	15:143597	6.25E-50
YJR108W	10:627628	3.98E-90
YNR007C	12:668249	7.61E-32
YLR442C	8:111683	1.20E-26
YOR386W	2:537314	2.71E-31
YOR386W	15:143597	6.30E-31
YLR277C	15:180180	3.72E-27
YLR194C	15:143597	8.47E-25
YIL011W	5:350744	8.66E-63
YIL011W	9:341216	9.59E-32

YOL166W-A	15:17946	2.85E-40
YIL033C	15:170945	1.31E-41
YJL159W	2:569420	7.46E-26
YML066C	13:129925	5.45E-82
YPL011C	2:567221	1.22E-24
YPL011C	15:141621	6.95E-25
YPL011C	16:542307	4.03E-44
YNL322C	14:33361	5.77E-45
YDL120W	4:246738	1.36E-45
YER173W	13:69122	2.10E-24
YMR148W	15:136327	2.55E-41
YKR017C	11:482069	4.02E-65
YLR215C	12:582499	6.48E-44
YMR109W	13:481549	1.99E-66
YNL284C	15:154177	1.49E-27
YJL172W	2:551299	9.40E-24
YJL172W	10:99921	7.46E-40
YOR327C	10:548177	3.06E-50
YJL045W	10:387893	2.08E-42
YLR102C	15:136327	5.59E-36
YOL166W-A	15:17946	1.05E-58
YPR085C	16:711614	1.25E-80
YJR104C	12:668249	4.42E-29
YJR104C	15:150651	5.47E-23
YCR071C	15:180222	7.10E-30
YAR028W	1:185122	1.25E-90
YOR138C	15:589145	2.31E-48
YGL078C	15:154309	3.69E-21
YOR107W	15:136327	1.77E-25
YPR023C	11:219950	2.48E-24
YPR023C	16:600664	4.20E-56
YLR406C-A	12:927421	4.48E-52
YDL207W	4:85846	5.90E-42
YBR214W	15:174364	6.96E-31
YGR158C	7:805409	1.10E-45
YIL142W	9:79823	4.26E-42
YGL006W-A	7:490784	2.30E-47
YDL247W	10:715254	3.84E-52
YAR064W	6:43672	1.68E-35
YGR037C	1:41483	1.86E-25
YJL116C	2:555787	1.91E-32
YJL116C	10:218798	4.05E-44
YML056C	15:154177	2.27E-30
YNL264C	12:635380	2.10E-34
YLL011W	15:174364	3.87E-26
YGR187C	15:174364	6.72E-30
YOL083W	2:533262	1.58E-25

YPL041C	8:111679	5.92E-25
YPL041C	12:705190	1.65E-29
YHR092C	15:143597	6.27E-27
YML128C	15:174364	2.53E-50
YLL018C-A	15:150651	4.83E-44
YNL050C	14:525061	1.87E-34
YJR003C	15:174364	1.02E-47
YHR110W	15:174364	6.67E-27
YLR157W-C	12:472165	2.53E-94
YPR061C	15:150651	2.44E-25
YBR115C	2:477206	2.23E-78
YPL231W	12:644136	4.27E-29
YOR127W	2:533268	1.53E-32
YOR127W	15:143597	6.28E-25
YGR128C	15:174364	5.07E-30
YGR095C	15:143597	2.59E-24
YCR018C	3:105042	7.45E-68
YPL187W	3:201166	5.12E-97
YOR173W	15:174364	1.22E-48
YPL201C	16:90266	1.32E-33
YMR288W	8:111680	2.40E-24
YNL072W	8:111679	2.11E-23
YER010C	2:553812	2.28E-22
YER010C	13:49894	4.34E-27
YDR441C	4:1344670	1.14E-43
YDR441C	13:27644	1.57E-24
YCL051W	15:154309	2.02E-23
YHR058C	8:221933	4.00E-79
YGR125W	2:555575	7.85E-43
YCR089W	8:111683	3.86E-66
YOR207C	15:174364	7.56E-35
YDL203C	15:170945	8.80E-27
YNL148C	13:28334	7.26E-27
YDR507C	2:533262	3.97E-34
YBR155W	15:174364	1.66E-29
YNL282W	2:517365	2.34E-28
YPL277C	13:922258	1.28E-50
YJR005C-A	10:453011	1.89E-31
YJR005C-A	13:885665	1.18E-27
YNL081C	15:180222	1.25E-40
YMR223W	12:662627	3.65E-28
YDR351W	15:143597	2.66E-31
YMR113W	13:494170	4.34E-63
YGR040W	2:567221	6.45E-53
YGR040W	7:530473	2.73E-29
YGR003W	7:502131	1.16E-49
YDR005C	4:433589	2.14E-24

YKL170W	11:98330	8.96E-39
YER153C	2:555596	4.55E-67
YIL089W	9:196145	1.12E-97
YPL257W	16:70853	4.70E-61
YDL197C	4:114155	4.24E-50
YJR131W	10:646911	1.43E-40
YJL213W	10:34098	1.10E-88
YHR183W	13:77684	1.38E-46
YNL279W	4:963733	3.06E-26
YNL279W	8:111683	2.19E-54
YBL052C	8:92960	1.18E-24
YBR063C	2:376872	1.88E-34
YAL008W	15:170945	1.60E-29
YGL033W	3:201166	1.68E-24
YPL266W	15:174364	1.72E-28
YHR068W	15:154177	3.36E-27
YFR041C	6:239482	3.24E-39
YMR155W	2:555596	7.90E-28
YMR155W	13:556841	4.14E-25
YMR155W	16:500342	7.93E-24
YHR015W	8:137221	2.63E-65
YBR179C	2:565216	2.41E-33
YGL053W	7:403626	1.39E-98
YBL045C	13:28622	7.21E-25
YBL045C	15:141627	6.40E-30
YLR188W	16:387239	2.65E-42
YGL116W	8:111680	4.07E-33
YPR009W	15:150651	6.85E-24
YPR009W	16:542295	1.25E-46
YDR462W	15:170945	3.13E-29
YPL230W	15:174364	1.43E-43
YLR153C	12:662627	1.11E-86
YLR203C	15:174364	4.33E-40
YDR389W	2:569420	5.37E-23
YNL326C	8:71742	8.23E-31
YDR321W	4:1109729	1.08E-44
YBL064C	15:170945	1.29E-35
YHR187W	15:174364	1.97E-27
YDL202W	15:174364	1.17E-25
YJR093C	10:604478	5.29E-50
YMR038C	12:668249	2.17E-45
YOR233W	15:174364	6.46E-28
YPL171C	2:562415	3.54E-23
YPL171C	15:144659	4.29E-41
YDL169C	2:555596	4.22E-29
YMR255W	2:551299	3.00E-37
YJL020C	10:393255	7.32E-45

YJL020C	13:77684	3.37E-28
YDL226C	4:46316	2.58E-69
YGL120C	15:154177	5.69E-23
YOR283W	15:846344	4.29E-86
YJL132W	2:562415	1.16E-22
YJL132W	10:159479	6.38E-27
YGL223C	7:73452	9.51E-28
YGL223C	8:95289	6.09E-27
YGL223C	13:28622	2.24E-25
YPR115W	8:111686	8.86E-42
YPR115W	15:170945	6.06E-26
YJL060W	2:555575	4.93E-22
YML116W	13:46084	3.22E-24
YML116W	14:449639	7.91E-44
YFL027C	3:201166	2.11E-94
YIL172C	9:19607	4.56E-28
YIL172C	13:100048	2.11E-32
YGR271C-A	15:136324	3.10E-31
YBR042C	12:662627	7.10E-27
YFR013W	6:169671	2.54E-30
YFR013W	15:136324	1.44E-29
YKL046C	15:174364	1.10E-29
YBR068C	3:92157	6.70E-49
YHR136C	13:27644	1.17E-84
YDR493W	15:180222	1.51E-25
YHL008C	8:84437	1.64E-34
YML002W	13:273244	8.70E-91
YJL117W	13:27644	2.55E-52
YBL008W	13:28694	1.80E-23
YNL286W	14:96321	6.52E-35
YLR229C	2:537314	8.66E-38
YBR026C	2:301671	4.07E-89
YKL109W	15:170945	6.02E-39
YHR033W	8:167504	1.40E-81
YOR177C	15:683415	3.98E-36
YOR334W	12:662627	1.24E-32
YDL051W	15:174364	1.81E-31
YPL233W	16:105278	4.83E-29
YPL028W	12:662627	2.37E-68
YDL208W	15:143597	1.12E-23
YDR047W	3:90610	1.15E-22
YDR047W	15:106266	2.52E-35
YOL135C	12:662627	5.37E-31
YLR438W	13:77684	4.25E-78
YNL164C	2:567221	3.86E-21
YHR007C	8:111686	8.06E-35
YHR007C	15:170945	9.20E-31

YCR004C	13:77684	5.92E-27
YCR004C	15:170945	7.57E-39
YKR051W	12:679808	1.01E-26
YMR065W	8:111690	1.13E-62
YIL014C-A	9:325320	2.36E-96
YIL014C-A	12:956534	1.22E-21
YLR241W	12:634227	1.22E-30
YDR161W	15:174364	2.47E-26
YLR082C	12:264911	7.53E-40
YOR267C	15:747543	1.01E-27
YCR051W	15:174364	1.89E-25
YDR260C	2:555778	1.11E-24
YLL035W	15:170945	9.49E-31
YER060W-A	13:49903	6.56E-27
YOR356W	15:108577	6.45E-22
YNL146W	3:201166	3.75E-76
YGL262W	7:10158	9.63E-48
YKL050C	2:521415	1.89E-23
YGL226W	15:179289	3.53E-27
YDL073W	12:668249	2.73E-22
YDL073W	15:113251	1.25E-30
YDR151C	7:375499	5.38E-38
YDR342C	15:174364	2.57E-56
YOR296W	2:548401	7.04E-53
YBR107C	2:477206	3.40E-72
YEL024W	12:668249	4.82E-37
YEL024W	15:113251	1.15E-30
YCR096C	3:201166	3.61E-91
YJR132W	15:150651	1.56E-33
YLR293C	15:150651	1.36E-22
YLR389C	12:927421	4.28E-36
YNL215W	14:245307	4.14E-42
YBL039C	15:174364	4.06E-25
YPL109C	15:143597	2.44E-26
YFL007W	14:449639	6.87E-72
YPR161C	15:154177	7.69E-30
YJR092W	2:551299	2.12E-31
YJL098W	13:49894	1.02E-24
YPR088C	12:677957	3.00E-23
YGR220C	15:180222	1.57E-32
YHR163W	13:77684	8.21E-31
YHR009C	8:137227	8.91E-32
YHR009C	15:174364	2.13E-32
YLL001W	15:174364	6.16E-32
YDL022W	15:174364	1.96E-41
YNL207W	14:258590	6.93E-31
YMR073C	2:565216	1.27E-21

YIL124W	9:101011	2.03E-25
YIL124W	15:174364	6.31E-41
YOR162C	15:632882	3.68E-43
YLR142W	2:567221	3.27E-30
YLR142W	13:49894	1.64E-33
YLR142W	16:500342	2.68E-32
YML120C	15:113251	3.72E-36
YDL183C	15:174364	1.12E-35
YDL019C	15:174364	2.76E-62
YMR308C	15:150651	3.54E-27
YIL153W	9:74540	1.38E-27
YDR264C	4:975086	3.79E-80
YFL048C	15:143597	4.98E-25
YIL146C	2:555596	1.29E-37
YNL003C	12:659357	7.96E-44
YLR413W	12:662627	2.54E-24
YOR163W	13:27644	3.68E-27
YOR005C	15:141627	4.09E-22
YEL076C-A	12:1067122	8.35E-28
YNL117W	2:551299	1.49E-24
YOR243C	15:170945	6.98E-29
YMR300C	13:77684	3.94E-38
YLR288C	12:708594	1.09E-71
YNR068C	2:555575	2.74E-29
YBR006W	2:246129	5.82E-60
YLR361C-A	12:851826	5.16E-80
YPL172C	15:174364	4.26E-30
YPR089W	2:569420	9.92E-28
YLR197W	15:174364	5.03E-24
YBL055C	2:133749	1.09E-35
YOR113W	15:174364	1.79E-28
YLR093C	12:327131	2.19E-41
YAL065C	5.234722222	8.76E-68
YIL103W	9:190866	2.77E-25
YOL119C	15:106272	5.80E-44
YFL029C	6:78241	5.66E-29
YJL210W	15:143597	7.39E-44
YJL210W	16:500348	8.27E-30
YDL122W	2:530481	7.64E-30
YOR260W	15:154177	3.43E-25
YDL006W	4:465337	4.18E-30
YNL194C	15:174364	5.31E-48
YDL178W	12:662627	8.66E-30
YML071C	15:170945	5.67E-28
YJL035C	10:380085	1.23E-91
YML031W	2:519049	1.50E-27
YKL041W	11:354466	3.32E-73

YNL025C	13:100048	4.65E-23
YGR016W	2:506661	2.35E-24
YHL021C	15:174364	2.02E-28
YMR272C	15:108577	3.15E-68
YKL026C	15:174364	5.79E-40
YHR142W	2:533268	3.58E-37
YHL009W-A	8:98513	4.03E-47
YHL009W-A	16:445372	1.78E-50
YER043C	12:683463	5.25E-35
YPL022W	16:523450	1.10E-38
YBR130C	2:537314	4.87E-31
YIL088C	2:555596	1.26E-32
YAL049C	1:52943	3.04E-86
YER047C	5:243299	6.63E-54
YIL099W	15:174364	3.00E-27
YMR293C	15:154177	2.88E-26
YOL042W	15:276469	1.27E-24
YLR084C	2:537314	1.63E-34
YLR084C	15:141627	3.49E-31
YLR084C	16:500342	1.14E-26
YPL098C	15:89229	1.01E-24
YMR090W	15:174364	2.48E-44
YHL039W	15:174364	5.84E-25
YIL077C	15:143597	3.00E-47
YMR232W	8:111683	6.68E-60
YMR002W	15:113261	1.34E-25
YMR136W	15:143597	7.24E-53
YDR116C	15:174364	7.18E-27
YIL111W	12:681096	1.14E-31
YIL111W	13:87587	4.04E-26
YNL239W	2:555596	2.59E-43
YMR041C	15:136327	2.33E-31
YMR041C	16:500348	9.08E-23
YIL136W	15:174364	3.43E-53
YDR226W	12:677957	3.94E-26
YPL271W	13:49894	9.11E-24
YPL271W	15:174364	1.64E-36
YPL271W	16:500342	6.12E-27
YBR166C	2:569420	9.53E-59
YHR047C	3:91977	2.14E-28
YPR159W	2:517365	7.88E-25
YNL034W	14:571965	4.66E-85
YLR412W	12:957108	6.80E-35
YBR039W	15:136327	3.21E-27
YNL010W	12:644136	5.92E-39
YNL064C	14:502496	2.98E-27
YGL202W	7:110806	5.85E-31

YFR053C	15:174364	1.91E-39
YNL048W	14:525061	1.42E-29
YMR315W-A	13:910381	3.17E-36
YOR317W	13:28622	1.62E-25
YOR317W	15:150651	2.02E-29
YPL268W	13:28694	8.24E-38
YPL087W	2:506661	1.59E-22
YPL087W	15:143597	9.73E-27
YCL073C	11:656099	1.06E-96
YBL098W	12:662627	1.89E-27
YGL146C	15:174364	7.22E-40
YLR156W	12:472165	5.72E-92
YOR254C	15:113254	7.10E-24
YER110C	15:150651	8.89E-26
YOR136W	15:571103	5.61E-25
YBR189W	2:565216	2.17E-22
YPL160W	15:170945	1.46E-27
YHR150W	8:389050	1.76E-36
YGR038W	7:553877	1.54E-87
YMR070W	12:672779	2.47E-31
YCR033W	12:662627	8.19E-27
YBR001C	2:252538	1.53E-26
YBR001C	15:150651	7.48E-35
YHR190W	12:662627	7.18E-66
YOR358W	15:108577	4.48E-36
YPL163C	2:537314	4.72E-46
YDR510W	4:1474479	1.22E-28
YPL273W	13:922256	3.76E-68
YKL157W	2:533262	1.18E-30
YIR008C	9:372093	5.70E-41
YDR304C	8:80014	7.40E-24
YGR013W	2:486640	2.36E-27
YMR289W	13:849969	2.88E-72
YHL011C	15:174364	1.47E-32
YNL046W	2:533262	1.79E-55
YNL046W	14:547071	2.11E-35
YNL145W	3:201166	5.12E-97
YLR409C	15:174364	1.99E-26
YML126C	12:659357	9.21E-87
YHR061C	8:111679	9.92E-32
YJR077C	15:136324	1.43E-34
YJR077C	16:500342	9.07E-22
YPR040W	12:681096	1.54E-23
YGL208W	7:110813	2.73E-66
YGL208W	15:174364	4.11E-24
YIL126W	9:98955	1.53E-24
YGL191W	12:668249	3.28E-39

YBR070C	2:388862	8.77E-33
YGR180C	12:450046	5.28E-42
YAL061W	15:143597	4.25E-35
YDR358W	15:174364	1.43E-26
YJR036C	2:533262	8.82E-26
YJR036C	15:143597	6.20E-26
YLR426W	12:987750	3.31E-84
YKL218C	2:551299	1.33E-43
YMR034C	13:328865	5.50E-61
YPR030W	15:170945	1.41E-29
YDR216W	15:144659	2.15E-52
YDL110C	15:174364	2.70E-33
YLR281C	12:705190	5.83E-46
YBR069C	2:376872	8.38E-63
YKL151C	15:174364	1.28E-39
YDR204W	15:174364	1.09E-40
YML081C-A	15:174364	2.63E-35
YML081C-A	16:511400	1.60E-23
YDR461W	3:201166	5.12E-97
YFR029W	5:321618	1.74E-30
YHR162W	13:81358	4.18E-25
YPL053C	15:174364	4.03E-29
YPL222W	15:170945	3.71E-36
YDR284C	2:517123	4.18E-23
YDR284C	12:662627	1.45E-33
YFL016C	7:375499	2.17E-36
YFL016C	15:108577	5.40E-24
YJL204C	10:59959	1.32E-40
YMR062C	13:390351	5.53E-28
YHR216W	6:28029	8.49E-29
YHR216W	13:180103	5.48E-35
YJL077W-B	2:551299	2.13E-26
YKL090W	11:266017	9.54E-89
YOL101C	1:42591	4.04E-55
YOR348C	12:668249	1.96E-38
YOR348C	15:150651	2.80E-31
YOR348C	16:500342	5.77E-33
YIL158W	9:47053	6.44E-73
YGL067W	7:375499	4.54E-53
YPL247C	15:174364	1.36E-52
YDR256C	15:144659	5.99E-30
YFR026C	6:205881	3.13E-74
YDR124W	8:111683	3.56E-27
YKR006C	15:170945	1.35E-30
YAL063C-A	1:10152	6.59E-48
YLR100W	12:662627	4.24E-52
YGL017W	7:459354	3.16E-36

YGL017W	12:644082	3.03E-25
YLR143W	15:174364	1.73E-37
YKL181W	2:555575	1.29E-26
YOR176W	15:170945	9.61E-27
YDR021W	15:174364	8.60E-28
YLL066W-B	4:1511257	6.80E-45
YLL066W-B	12:1056097	1.54E-48
YBL029C-A	15:143597	4.62E-38
YBL099W	15:174364	1.06E-35
YMR323W	12:1042072	2.77E-24
YMR323W	13:923511	4.03E-26
YDL036C	15:180180	1.24E-27
YLR372W	15:170945	7.90E-24
YPL147W	16:266023	2.99E-28
YOL113W	15:108577	2.77E-36
YFL010C	6:134096	5.75E-57
YLL055W	12:26184	6.75E-43
YMR267W	15:170945	1.27E-39
YOR188W	2:537314	4.49E-51
YLR058C	13:79760	3.76E-23
YMR181C	15:174364	5.52E-45
YPR196W	16:932538	4.71E-53
YDR464W	2:565216	3.03E-22
YDR464W	15:141627	4.79E-24
YKL139W	15:174364	1.28E-23
YBL043W	12:672779	3.18E-33
YNL323W	14:19885	1.44E-50
YDR408C	13:77684	9.75E-23
YHR090C	13:33681	2.39E-24
YHR090C	15:136324	3.82E-25
YNL007C	7:375499	8.12E-55
YGL209W	2:551299	3.42E-22
YML055W	13:163328	1.31E-39
YDL131W	4:217399	1.26E-30
YKL210W	11:46633	2.72E-31
YCL014W	2:555575	2.96E-38
YHL048W	6.176388889	1.39E-98
YML111W	13:49894	9.81E-59
YMR093W	15:174364	2.87E-24
YKL189W	8:111683	2.53E-35
YKL189W	13:46084	5.84E-26
YBR231C	2:681442	6.20E-35
YDL082W	4:289639	8.72E-25
YJL151C	15:174364	1.40E-23
YLL018C	3:81832	6.17E-22
YAR018C	2:553812	7.24E-24
YDL021W	15:170945	3.76E-36

YDR074W	15:174364	2.89E-47
YKL117W	15:179289	7.96E-25
YNL231C	12:659357	1.06E-24
YDR171W	15:174364	2.20E-41
YOL163W	15:10427	1.22E-98
YBR120C	15:180222	3.00E-28
YMR011W	5:350744	4.18E-39
YGR131W	12:659357	2.06E-26
YDR322W	15:174364	2.31E-30
YKL093W	15:174364	1.30E-27
YDR443C	4:1344670	9.26E-66
YOR382W	13:885665	6.14E-35
YLR332W	15:143597	1.40E-27
YGR093W	15:174364	1.02E-24
YNL317W	14:37071	3.28E-55
YDL164C	4:165032	2.46E-29
YKL142W	15:174364	2.33E-38
YML106W	2:533262	2.99E-25
YBL071C-B	2:519049	2.80E-33
YAL040C	15:174364	3.99E-30
YJR005W	2:537314	8.26E-33
YJR041C	15:174364	5.07E-33
YPL038W-A	16:489143	1.05E-63
YER075C	2:562415	1.82E-38
YOR374W	13:77684	8.07E-45
YOR374W	15:174364	4.23E-35
YGL028C	2:562409	4.91E-92
YOL095C	15:141627	8.98E-52
YNL247W	15:174364	1.87E-24
YBR161W	12:662627	1.43E-31
YPR199C	11:656099	7.63E-95
YGR230W	3:90610	9.77E-23
YGR230W	12:744310	1.18E-30
YKR058W	15:174364	3.24E-32
YER076C	5:272258	2.76E-27
YKR095W-A	3:91287	2.42E-37
YKR095W-A	15:180961	8.09E-31
YKR004C	11:421190	8.12E-48
YML068W	2:555575	3.79E-26
YJL217W	10:23505	2.34E-92
YFL053W	6:30378	7.97E-79
YFL042C	15:154309	1.33E-29
YOL016C	2:567221	7.97E-27
YHL047C	6.176388889	1.39E-98
YGR236C	15:143597	2.59E-37
YPL040C	15:174364	3.55E-31
YER087W	15:170945	1.52E-26

YKL028W	11:394660	7.71E-40
YGR229C	2:553812	2.66E-24
YGR229C	7:954692	3.03E-33
YDL002C	4:465337	8.61E-52
YHR094C	4:217351	6.08E-46
YOR226C	3:79091	5.10E-47
YOR226C	15:143597	4.27E-29
YOL029C	15:174364	2.81E-42
YMR052W	13:379981	1.27E-36
YBR123C	3.133333333	5.42E-21
YPL169C	2:514035	1.60E-23
YLR419W	2:537314	6.24E-26
YLR419W	15:141627	2.37E-25
YDL017W	4:465157	1.06E-39
YLR449W	15:174364	3.14E-31
YIR038C	15:143597	1.05E-32
YOR140W	2:548401	2.64E-43
YOR211C	13:79786	1.67E-23
YDR011W	12:672779	1.88E-24
YDR011W	15:632882	3.98E-41
YBR073W	15:170945	3.50E-27
YMR081C	15:174364	1.17E-25
YGL205W	7:110807	1.14E-46
YBR261C	15:154177	3.43E-31
YPL104W	15:174364	2.89E-43
YOR048C	15:150651	1.59E-30
YGR206W	7:905017	2.44E-26
YKL029C	3:100213	2.52E-26
YKL029C	11:382553	8.95E-62
YPR007C	2:569420	1.23E-23
YPR007C	16:547618	7.90E-59
YDL135C	4:143910	4.19E-32
YPR172W	15:170945	1.28E-33
YHR080C	15:143597	6.10E-48
YIL169C	9:27026	1.61E-40
YIL169C	6.038194444	2.90E-28
YCL026C-B	3:75021	1.15E-83
YML058W-A	12:469156	4.94E-38
YJR102C	2:548401	1.52E-25
YMR124W	13:513778	1.35E-42
YNL246W	14:191243	5.32E-79
YMR296C	2:519049	4.43E-23
YML088W	13:28622	2.52E-26
YML088W	15:174364	9.45E-26
YAR042W	1:201039	2.09E-30
YMR127C	2:567221	1.64E-25
YKL071W	13:77684	2.09E-24

YPR008W	15:174364	4.84E-27
YOL137W	15:59733	1.65E-41
YIL048W	9:251537	1.54E-25
YPR063C	13:124876	1.24E-22
YHR084W	8:111683	8.74E-41
YPL232W	12:662627	8.53E-32
YLR146C	15:174364	9.60E-30
YNL131W	15:113261	6.05E-27
YMR100W	12:677957	4.30E-34
YBL032W	2:162382	3.20E-27
YPL135W	5:321618	2.18E-26
YOR286W	15:113254	7.59E-32
YNL115C	15:174364	8.75E-27
YGL064C	2:530481	8.13E-27
YOL056W	15:170945	1.38E-37
YGR289C	7:1081945	4.92E-65
YGL037C	15:174364	7.95E-45
YBR013C	2:252640	5.32E-89
YNL058C	14:525061	6.72E-37
YOR065W	12:668249	1.86E-30
YOR065W	15:113251	2.99E-27
YOR065W	16:500348	1.63E-26
YOR034C-A	2:553812	1.99E-23
YPL236C	15:136327	9.46E-31
YGL032C	3:201166	5.12E-97
YER001W	2:551299	1.97E-31
YER001W	15:141627	1.07E-28
YER001W	16:511406	1.21E-29
YDL171C	4:164444	1.44E-27
YPL052W	15:174364	4.10E-39
YKL101W	11:247944	3.20E-24
YBR274W	2:746476	1.20E-69
YLL041C	13:28622	1.73E-25
YLL041C	15:174364	7.02E-41
YGR147C	2:565216	3.22E-30
YGR237C	15:170945	1.51E-31
YOR212W	8:111683	1.50E-29
YOR212W	13:49894	9.42E-28
YOR383C	13:390357	3.47E-38
YIL148W	9:79793	1.17E-41
YDR513W	4:1456748	1.90E-27
YDR513W	15:170945	2.73E-29
YGL060W	8:111690	3.06E-48
YLR439W	15:174364	2.69E-29
YHR179W	12:662627	1.14E-45
YPR026W	15:174364	1.03E-39
YJL006C	10:451946	7.45E-42

YPL046C	16:462646	1.48E-47
YNL245C	14:191243	3.20E-62
YOL159C	9:19607	4.54E-62
YOL164W	15:10427	1.15E-98
YCR106W	6.628472222	4.31E-42
YCR106W	0.868055556	3.49E-42
YCR106W	12:1054278	2.82E-52
YML073C	15:154177	4.56E-23
YNL274C	15:143597	4.65E-41
YGL227W	15:174364	5.29E-29
YNL004W	14:486861	1.49E-51
YER048C	2:537314	2.84E-24
YOL014W	15:298710	1.30E-91
YLR228C	15:174364	1.51E-29
YER155C	8:111682	7.72E-41
YGR153W	15:108577	1.99E-24
YKL095W	13:110814	1.40E-22
YIL131C	2:551299	1.02E-27
YNL173C	2:562415	9.52E-27
YNL173C	12:662627	3.26E-24
YNL173C	15:141627	4.78E-25
YGL103W	7:311205	1.82E-52
YGR109W-B	7:708028	2.51E-83
YGR109W-B	8:92960	4.79E-23
YGR109W-B	9:200332	2.53E-79
YKL052C	15:143597	7.79E-67
YDL126C	14:486861	3.76E-48
YJR080C	15:174364	1.72E-39
YAL056W	1:41483	2.38E-35
YAL056W	16:500348	1.86E-24
YER188C-A	5:568716	8.88E-72
YNL315C	15:170945	7.00E-25
YFL038C	15:170945	1.13E-25
YNL316C	14:38762	2.29E-79
YOR196C	13:849969	9.15E-22
YDL190C	2:419093	2.28E-26
YPR138C	2:555575	8.41E-36
YOR109W	15:524972	1.05E-44
YFR012W	6:168342	3.22E-27
YBR170C	2:567221	1.90E-47
YBR170C	16:500348	4.17E-30
YHR213W	1:11626	2.05E-38
YOL089C	15:141627	1.61E-54
YOL159C-A	9:33795	1.03E-45
YOL159C-A	3.133333333	1.02E-44
YBR052C	15:174364	1.68E-40
YKR097W	2:569420	1.55E-26

YKR097W	11:632952	3.11E-56
YJR030C	10:472146	2.00E-61
YMR312W	15:174364	7.43E-30
YJL010C	15:174364	8.75E-30
YLR176C	12:634225	4.66E-26
YLR022C	15:174364	2.00E-25
YIL119C	9:139462	2.54E-41
YNL134C	15:150651	1.23E-77
YBR148W	2:548401	6.03E-82
YGR127W	15:170945	2.00E-34
YBL087C	15:154177	7.79E-24
YHR028C	2:537314	2.08E-25
YGR174C	15:174364	1.53E-42
YGL258W-A	2:562415	3.89E-29
YIL049W	9:268412	1.02E-30
YOR230W	2:555596	9.18E-22
YDL193W	4:114155	1.18E-29
YJR019C	15:136327	1.78E-32
YDR267C	12:681096	8.85E-25
YIL051C	13:49894	4.98E-42
YCR009C	2:480009	7.09E-21
YCR009C	8:111690	1.33E-23
YAR071W	13:27644	3.39E-74
YBL071W-A	15:174364	6.44E-27
YIL152W	9:74540	1.11E-83
YGL075C	7:375499	9.44E-46
YOR027W	7:375499	6.35E-51
YDR229W	15:170945	1.95E-26
YBR085W	12:662627	1.00E-31
YBR085W	15:113251	5.94E-26
YJR007W	15:143597	1.20E-21
YCL012C	3:91977	1.78E-21
YML119W	13:27644	5.18E-39
YHR153C	8:389050	1.90E-34
YDR529C	12:705100	1.17E-20
YDR529C	15:141627	1.87E-25
YDL090C	8:111690	1.00E-21
YKL141W	13:28694	4.74E-32
YKL141W	15:174364	5.29E-43
YKL141W	16:500342	1.10E-22
YIL065C	7:707950	1.61E-25
YIL065C	9:200332	9.18E-44
YNL137C	15:180210	3.44E-29
YBL038W	2:143721	2.21E-39
YNL202W	14:258590	6.40E-42
YJR148W	3:81832	3.96E-24
YAL060W	1:36900	5.67E-39

YAL060W	15:179289	1.21E-24
YDR410C	8:95289	1.41E-22
YGL181W	7:143756	4.42E-32
YOR023C	15:407684	6.65E-28
YIL082W-A	7:708028	1.38E-82
YIL082W-A	8:92960	7.70E-24
YIL082W-A	9:200332	8.67E-81
YBR156C	2:537314	3.85E-45
YER118C	2:537314	2.88E-53
YPL110C	13:46084	3.43E-45
YGL009C	3:81832	3.11E-80
YGL009C	15:180222	5.25E-25
YKR093W	13:46070	1.23E-30
YKR093W	15:143597	1.50E-41
YDL156W	2:517123	8.25E-23
YML047C	4:963733	3.99E-27
YML047C	8:111683	2.50E-64
YGL184C	8:167504	3.01E-24
YNR069C	2:555575	1.11E-25
YLL042C	12:63866	4.82E-61
YMR047C	2:499889	5.83E-23
YOR186W	15:136327	9.54E-28
YHR088W	15:174364	2.67E-25
YGL045W	7:402841	5.99E-26
YER029C	5:218250	4.59E-79
YHR029C	8:167504	5.68E-37
YIL043C	12:674651	5.94E-24
YGL139W	2:551299	1.36E-37
YMR174C	2:517365	1.66E-27
YMR174C	15:143597	7.92E-29
YCR107W	6.628472222	8.51E-47
YCR107W	0.868055556	2.26E-41
YCR107W	12:1054278	6.29E-50
YOR201C	15:715809	3.07E-36
YMR191W	13:649250	2.76E-68
YIL031W	12:662627	8.14E-32
YOL164W-A	15:10427	1.56E-96
YLR219W	15:174364	1.04E-50
YPR158W	7:375499	4.11E-60
YER057C	13:33681	6.97E-30
YNL227C	15:174364	8.92E-28
YOR111W	2:551299	3.87E-33
YBR288C	15:174364	1.58E-23
YNL278W	8:111683	1.88E-31
YER130C	15:143597	6.77E-28
YOR215C	15:143597	6.89E-31
YOL091W	15:144659	6.44E-65

YOR220W	13:49894	3.48E-24
YOR220W	15:170945	1.79E-28
YIL067C	15:143597	2.39E-25
YGR067C	2:584357	5.82E-27
YKL182W	2:516889	6.14E-26
YKL182W	12:644136	6.86E-26
YGL194C	7:139389	1.13E-46
YFL065C	4:1503330	2.15E-30
YHR010W	15:108577	7.80E-25
YIL155C	15:154309	3.25E-41
YKL128C	11:194611	7.27E-31
YGR243W	15:174364	8.35E-47
YGR077C	15:136327	4.43E-23
YLR155C	12:472165	1.89E-92
YBR034C	15:150651	1.92E-28
YGR102C	15:174364	1.19E-32
YLR154C	12:662627	2.76E-45
YLR285C-A	2:565216	8.97E-57
YJL005W	15:174364	1.40E-33
YML004C	13:266326	3.94E-47
YML004C	15:141627	7.35E-23
YER082C	15:174364	1.45E-36
YFR047C	2:514035	4.64E-25
YHR107C	2:530481	7.36E-38
YLR066W	15:154177	5.35E-26
YPR154W	12:659357	1.16E-24
YML018C	15:170945	2.88E-32
YLL021W	15:150651	2.49E-28
YOR085W	12:705226	5.84E-31
YKL216W	5:117056	6.64E-66
YPR095C	12:659357	1.20E-37
YLR129W	15:154309	1.43E-27
YLR380W	12:662627	3.22E-27
YOL116W	15:132423	3.11E-34
YDR366C	4:1213416	2.28E-85
YNL271C	12:662627	1.80E-24
YCR020C-A	15:136324	7.92E-23
YPR198W	11:656099	1.12E-97
YLR042C	2:562409	1.86E-79
YOR051C	15:154309	1.23E-25
YIL063C	9:238345	1.46E-33
YJL107C	10:218798	8.57E-46
YIL101C	9:244902	2.33E-33
YIL101C	15:174364	1.65E-38
YMR129W	2:517365	7.92E-30
YMR032W	2:562415	9.72E-22
YLR186W	15:143597	8.23E-26

YDR179C	15:136324	1.08E-26
YAR075W	1:229090	3.03E-41
YGL058W	15:174364	2.26E-29
YKL087C	15:180222	1.12E-32
YLR278C	13:255564	7.16E-24
YDR357C	4:1213416	7.08E-58
YDR378C	15:136324	7.28E-23
YNL223W	15:136327	1.73E-25
YMR072W	15:170945	9.04E-25
YER067W	15:174364	9.36E-34
YBL054W	2:133741	9.92E-23
YMR173W	2:592863	2.89E-24
YNL035C	14:591234	4.73E-91
YCR105W	6.628472222	1.96E-41
YCR105W	0.868055556	9.44E-38
YCR105W	12:1056103	7.98E-46
YOR304C-A	15:889464	3.29E-81
YJR107W	10:627628	1.38E-36
YER049W	15:174364	2.69E-32
YDR280W	15:174364	4.59E-26
YOL073C	15:174364	2.05E-32
YAL043C	2:508843	6.10E-27
YJL062W-A	10:380085	2.42E-23
YJL062W-A	15:170945	3.19E-34
YLR128W	15:136327	5.26E-24
YHR210C	12:644082	7.49E-25
YHR210C	15:136327	3.38E-27
YJL013C	10:404508	2.80E-62
YBR045C	2:328489	1.30E-82
YDL238C	2:555575	1.24E-53
YDL238C	4:46466	1.40E-22
YJR043C	2:533262	3.20E-27
YER107C	15:174364	1.06E-26
YLR312C-B	12:760763	2.30E-31
YBL030C	15:136327	1.48E-41
YIL007C	12:662627	3.24E-45
YPR113W	12:677957	4.65E-35
YOR227W	3:91287	5.01E-23
YOR227W	15:170945	8.02E-40
YNL097C-A	8:95289	4.87E-23
YER145C	12:683463	1.85E-22
YGL248W	7.024305556	5.27E-56
YPL014W	13:77684	7.14E-22
YPL014W	15:174364	7.96E-33
YCR086W	3:258303	3.55E-25
YJL170C	3:201166	6.61E-97
YJR127C	15:141633	9.55E-51

YCR046C	15:174364	5.50E-41
YDR218C	12:672779	1.95E-31
YPL213W	2:551299	8.05E-30
YNR059W	15:143597	4.93E-27
YNL040W	2:513408	8.09E-38
YNL040W	14:591237	4.87E-64
YHR151C	12:662627	1.73E-24
YPL008W	12:668249	3.68E-24
YKL179C	2:562409	2.71E-29
YGL106W	8:93002	5.51E-36
YHR039C	12:662627	7.10E-62
YGL241W	7:44996	8.24E-46
YJL198W	12:662627	5.91E-23
YPL064C	16:428900	5.97E-36
YML109W	2:533262	9.31E-33
YDR277C	15:174364	5.33E-31
YDR042C	4:527458	9.89E-26
YLL063C	2:573491	4.93E-25
YJL157C	8:111683	1.15E-70
YMR194C-B	15:174364	6.68E-47
YDL209C	4:80849	7.56E-33
YGR105W	15:136324	4.19E-22
YGR138C	12:662627	3.26E-30
YGR138C	16:511406	1.45E-30
YDR425W	2:551299	1.84E-29
YOR043W	15:409778	1.26E-35
YGR118W	15:113251	1.29E-23
YKL113C	11:218805	1.93E-25
YGL081W	2:567221	2.24E-24
YPR105C	2:602012	9.57E-38
YOR096W	15:108577	4.63E-22
YDR444W	4:1344670	9.14E-43
YGR257C	7:1007587	1.48E-51
YNL078W	2:582419	4.59E-63
YNL078W	8:111680	1.23E-29
YDR488C	12:674651	6.49E-32
YHR016C	15:174364	7.60E-40
YOR142W	15:108577	1.37E-31
YOR142W	16:500342	1.05E-23
YGR225W	7:952041	2.42E-33
YER180C-A	5:568698	5.31E-52
YLR163C	15:113251	2.25E-22
YPL157W	16:266023	1.80E-52
YDR091C	15:143597	2.03E-25
YKR076W	15:174364	1.61E-34
YMR060C	13:390351	3.22E-51
YGR203W	12:662627	7.87E-24

YLR086W	12:317542	2.98E-44
YKR064W	15:143597	9.33E-25
YBR054W	13:277071	7.44E-34
YHR115C	8:340251	6.36E-64
YGR021W	15:170945	9.80E-40
YJR084W	10:602943	1.62E-53
YJR015W	10:461201	1.62E-92
YGR142W	7:375499	1.14E-70
YML005W	15:174364	4.76E-25
YPL252C	16:70853	3.20E-28
YKR053C	11:553792	1.94E-63
YGR200C	7:889602	1.01E-25
YGR200C	15:174364	3.78E-24
YHR064C	15:154177	2.92E-26
YJL051W	10:345059	1.50E-76
YGR234W	12:662627	2.20E-84
YNL280C	12:662627	8.40E-37
YNL092W	15:174364	2.88E-24
YLR462W	4:1510883	1.22E-34
YLR462W	12:1067122	5.02E-33
YMR297W	15:174364	2.17E-28
YDR384C	12:693610	4.49E-30
YKL007W	10:548177	2.95E-52
YAR003W	12:674651	3.91E-24
YCR104W	12:1056103	4.15E-36
YHR017W	15:150651	5.89E-31
YHL016C	8:71742	2.36E-64
YMR157C	13:572643	7.71E-22
YMR157C	15:113261	1.84E-32
YNL133C	15:141627	2.08E-42
YCL025C	16:500342	2.57E-29
YBR117C	2:477206	7.15E-53
YHR031C	8:157605	1.71E-36
YNL056W	14:502496	8.29E-70
YDR034W-B	15:174364	1.22E-28
YHR022C	15:141633	6.65E-27
YHR022C	16:511406	1.32E-22
YJL037W	10:374040	1.51E-28
YDR296W	15:170945	6.60E-37
YLR085C	12:317542	9.34E-44
YOR241W	15:174364	1.06E-39
YBR204C	2:628224	4.10E-46
YLR378C	2:551299	4.74E-25
YOL114C	15:113254	1.10E-24
YKL222C	0.868055556	7.88E-55
YMR120C	13:77684	1.21E-26
YMR117C	13:492453	1.54E-76

YBR038W	2:548401	3.18E-36
YGL183C	7:143756	1.47E-42
YER187W	5:568698	2.71E-91
YDL129W	5:321714	6.65E-24
YOR001W	15:143597	4.62E-29
YOR161C	15:174364	1.47E-50
YMR286W	15:154177	1.68E-35
YER056C	5:272258	3.11E-36
YLR327C	15:174364	9.01E-35
YOR224C	15:174364	2.26E-26
YPL229W	12:668249	2.71E-27
YML085C	13:99675	2.09E-77
YML085C	14:412758	6.03E-29
YDL079C	4:285470	7.81E-30
YDL079C	15:174364	2.40E-27
YCR043C	15:170945	1.87E-35
YEL015W	2:480009	1.62E-23
YEL015W	14:449639	1.52E-46
YMR177W	15:174364	7.41E-36
YLR414C	12:659357	5.83E-28
YOR274W	15:174364	2.28E-29
YDR492W	7:403626	1.68E-36
YGL234W	13:49894	8.60E-34
YEL057C	5:44617	1.63E-68
YNL099C	2:521415	7.74E-25
YIL087C	7:707983	2.70E-31
YIL087C	9:200332	4.36E-53
YIL087C	15:136327	1.02E-29
YNR060W	12:683463	4.20E-32
YNR060W	15:179289	8.48E-28
YDL227C	4:46316	4.18E-92
YPR065W	12:659357	9.91E-60
YGR121W-A	7:705697	1.35E-43
YEL004W	2:555778	7.40E-27
YLR452C	8:111683	1.42E-72
YMR200W	2:537314	3.64E-25
YNL104C	3:105042	2.48E-31
YLR466C-B	4:1510883	4.57E-85
YLR466C-B	12:1056097	3.86E-38
YGR244C	15:143597	4.97E-50
YBR264C	8:95289	2.30E-23
YKR075C	7:375499	6.96E-35
YOL007C	2:517365	6.41E-33
YDL116W	15:174364	1.35E-26
YKL043W	11:354466	4.64E-71
YOL049W	12:634226	5.40E-26
YJL209W	10:51003	2.45E-44

YPR137W	15:170945	3.84E-26
YPL061W	13:77684	5.32E-36
YIL008W	15:136327	1.77E-30
YJR051W	12:668249	1.40E-26
YDL211C	15:179289	1.32E-22
YMR009W	12:662627	9.34E-80
YGL010W	7:459354	1.81E-49
YGL010W	15:179289	3.80E-31
YMR163C	2:521415	3.21E-31
YJR034W	15:180222	1.07E-39
YGR047C	8:111683	7.99E-24
YCL055W	8:111683	7.11E-70
YPL156C	8:111690	3.98E-25
YOR237W	12:662627	2.43E-59
YNL021W	14:591228	4.39E-69
YOL141W	15:43153	7.57E-39
YDR451C	4:1344670	1.17E-49
YBL086C	15:144659	8.70E-34
YGR249W	7:974640	7.60E-52
YLR405W	2:499012	2.17E-22
YNL054W	12:705100	5.03E-24
YNL054W	14:502496	4.04E-31
YNR040W	15:180222	7.85E-27
YBL021C	2:163240	8.49E-25
YDR279W	15:174364	1.88E-27
YGL174W	7:187179	9.21E-91
YMR244W	12:662627	8.06E-27
YIL015W	3:201166	5.12E-97
YHR111W	15:174364	1.82E-35
YGR246C	7:995892	1.84E-39
YIL114C	15:174364	1.37E-27
YKL017C	11:421190	6.04E-43
YOR129C	2:507428	1.12E-46
YDR085C	8:111683	4.92E-32
YER117W	15:108577	2.68E-22
YPL088W	15:174364	4.82E-28
YPL088W	16:387239	2.39E-30
YHL004W	15:174364	5.21E-34
YKL016C	13:28622	5.69E-29
YKL016C	15:174364	6.24E-37
YKL016C	16:500342	5.49E-24
YNL277W-A	2:548401	2.50E-24
YNL290W	14:96321	2.71E-45
YMR134W	12:662627	2.26E-85
YIL138C	15:136324	8.86E-22
YCL064C	2:553812	7.05E-23
YCL064C	13:49894	1.10E-30

YLR326W	12:782839	6.11E-61
YIL123W	2:551299	5.42E-30
YJR063W	15:154309	3.54E-26
YKR079C	15:174364	2.10E-35
YJL129C	15:136324	5.59E-29
YPR022C	16:600664	1.97E-29
YHL027W	8:63386	3.42E-32
YDR422C	4:1318073	2.85E-53
YBR139W	2:533262	9.05E-28
YBR162W-A	15:136324	5.93E-28
YNL012W	13:115474	3.13E-32
YGR179C	7:858473	9.47E-26
YBR007C	15:174364	8.50E-31
YBR287W	2:537314	1.23E-25
YAL047C	1:55215	1.37E-22
YDL179W	2:555596	3.28E-24
YMR145C	12:668249	5.37E-36
YMR145C	15:113251	9.19E-28
YDR022C	2:537314	2.67E-24
YDR022C	15:143597	1.38E-23
YOL054W	15:174364	2.04E-33
YJR147W	2:548401	1.12E-53
YML050W	15:154177	4.07E-38
YOR270C	12:705100	3.08E-24
YML003W	13:273244	9.58E-52
YOR219C	8:111683	4.15E-27
YPL158C	2:548401	4.52E-30
YKL003C	15:180222	8.69E-30
YDR420W	15:143597	3.38E-29
YDL087C	4:297977	1.77E-32
YGL145W	15:170945	3.53E-36
YJL002C	12:705190	7.46E-28
YNR044W	8:111690	1.31E-74
YOR360C	14:486861	5.54E-55
YOR360C	15:594024	1.86E-22
YPL219W	15:174364	4.45E-29
YKL150W	15:150651	4.83E-39
YIL098C	15:113267	6.79E-28
YGR194C	15:143597	7.12E-44
YCR069W	12:662627	1.34E-45
YLR390W	15:170945	2.15E-28
YDL210W	2:555575	1.04E-41
YDL210W	4:95437	1.47E-24
YKR069W	11:566015	7.24E-72
YIL156W	12:634225	3.59E-26
YJR056C	10:548177	5.55E-36
YMR017W	15:804686	1.61E-22

YDR128W	4:733875	3.57E-35
YLL034C	15:170945	1.73E-27
YPL111W	13:49894	2.35E-50
YLR080W	2:567221	1.44E-38
YML110C	15:170945	1.21E-35
YHR096C	15:144659	9.02E-42
YDR009W	4:450230	8.23E-30
YOR273C	15:141633	1.36E-25
YBR163W	2:565216	5.24E-37
YLR206W	12:553124	5.14E-61
YPR033C	15:174364	2.79E-29
YNR002C	13:99675	5.01E-39
YNR002C	15:174364	4.58E-47
YER152C	2:555596	4.06E-88
YGL210W	12:681096	1.84E-26
YDR197W	15:174364	2.76E-40
YLR466C-B	4:1510883	3.52E-79
YLR466C-B	12:1056097	2.01E-41
YDR003W	4:450230	2.53E-32
YDR003W	15:136327	1.00E-24
YAR029W	1:184405	3.70E-51
YAR029W	15:150651	4.26E-29
YBL044W	2:142262	1.24E-96
YGR035C	15:174364	1.75E-35
YOR315W	2:569420	5.10E-40
YIL036W	15:174364	1.03E-29
YOR316C	2:582419	7.33E-25
YHR059W	15:180222	9.08E-30
YBL113C	12:1056097	5.73E-31
YML081W	12:662627	1.96E-29
YNL156C	12:662627	8.58E-73
YKL120W	3:81832	8.88E-70
YKL120W	15:180222	2.49E-31
YKL051W	13:27644	9.24E-45
YKL051W	15:143597	8.97E-45
YBR058C	15:113251	1.31E-22
YBR078W	2:401568	2.13E-33
YML009C	15:180222	1.84E-26
YOR185C	15:174364	8.98E-44
YOL115W	2:551299	3.81E-26
YPL038W	16:492351	1.73E-32
YPL117C	12:662627	1.21E-59
YHL031C	8:17636	5.97E-30
YNL234W	15:141633	3.48E-38
YHR144C	15:174364	1.92E-29
YMR235C	2:519049	2.48E-21
YGL219C	7:73452	7.18E-59

YFL010W-A	6:134252	5.99E-47
YKR089C	8:111683	2.77E-22
YKR089C	11:599170	6.42E-46
YKR089C	13:46084	8.52E-29
YER011W	5:350744	8.30E-37
YER011W	12:644136	5.90E-50
YGR014W	2:569414	1.10E-73
YDL078C	2:506661	5.96E-28
YOR375C	3:100213	1.29E-47
YIL072W	7:708252	1.39E-27
YIL072W	9:238345	9.57E-62
YAR050W	1:201039	1.91E-95
YOL094C	15:154177	3.52E-40
YIR042C	9:437054	2.83E-92
YLL056C	12:634226	7.15E-28
YJL087C	15:170945	2.69E-27
YJL168C	15:174364	4.22E-24
YJR153W	2:592863	6.03E-33
YJR153W	5:350744	7.38E-30
YJR153W	10:703868	3.38E-33
YBL085W	2:519049	1.82E-34
YHR116W	15:180961	1.23E-36
YBR208C	2:419093	2.34E-23
YBR208C	13:81358	8.69E-31
YCL066W	3:201166	5.12E-97
YOR256C	12:659357	2.13E-22
YAR073W	6:28029	1.10E-25
YAR073W	13:180103	4.98E-35
YDR198C	15:174364	2.26E-34
YIL040W	1:51324	3.32E-22
YJL011C	15:136324	4.37E-31
YDR258C	15:174364	1.62E-27
YOL052C	2:499012	2.13E-30
YMR105C	15:174364	8.59E-46
YJL160C	2:565216	3.24E-22
YCR038C	3:201166	2.62E-80
YDR365C	4:1213416	3.03E-71
YJR105W	12:662627	3.45E-56
YOR289W	15:174364	1.61E-27
YJR096W	15:170945	2.89E-36
YKL014C	15:136327	1.55E-25
YGL020C	7:459354	5.85E-51
YLR310C	12:757570	3.50E-36
YBR192W	2:567221	7.07E-29
YGL105W	2:553812	1.36E-27
YHR004C	12:662627	4.44E-27
YBR083W	8:111683	3.75E-51

YEL049W	1:154328	7.75E-74
YDR406W	15:144659	2.04E-45
YBR185C	15:154177	4.69E-31
YDR324C	15:154309	1.10E-27
YJR086W	8:111682	9.83E-22
YDL100C	12:662627	3.86E-30
YOR079C	15:174364	5.76E-25
YER062C	15:143597	3.24E-53
YHR208W	3:81832	4.61E-84
YHR208W	15:180222	1.52E-24
YFR028C	2:537314	1.13E-32
YCL004W	3:105042	3.73E-44
YMR150C	13:562907	7.69E-52
YLR079W	2:567221	7.57E-54
YLR079W	16:500348	7.03E-23
YBR098W	2:480009	2.74E-53
YMR313C	13:28694	1.12E-25
YJR103W	14:449639	9.72E-62
YMR152W	13:572643	6.90E-51
YMR152W	15:170945	1.01E-28
YDL174C	12:672779	8.78E-50
YFL001W	6:144755	3.31E-30
YJR011C	15:116709	2.87E-32
YKL010C	11:421190	1.41E-46
YOR247W	2:537314	3.52E-65
YJL171C	3:201166	4.46E-40
YJL171C	10:110038	5.49E-48
YJR076C	10:575236	5.58E-41
YPL186C	15:143597	8.29E-47
YKR103W	11:656099	3.60E-98
YJL069C	15:143597	7.92E-29
YPR194C	2:555778	7.78E-31
YPR194C	16:927500	5.58E-54
YER113C	2:519049	4.01E-23
YJL059W	10:388461	8.93E-26
YFL040W	6:57455	1.85E-55
YIR028W	2:551299	5.39E-44
YCL021W-A	3:81832	1.15E-92
YLR095C	2:517365	1.41E-23
YLR258W	2:533262	8.91E-31
YLR258W	15:174364	6.99E-38
YBR101C	7:375499	4.22E-67
YAL041W	2:569414	2.02E-34
YDR044W	5:350744	9.42E-48
YDR044W	12:662627	5.46E-55
YGR152C	2:499012	2.30E-24
YGR152C	7:790857	7.02E-29

YLR412C-A	12:956534	1.64E-24
YHL003C	8:111679	1.28E-56
YPR139C	13:87587	4.22E-32
YJL153C	10:135908	6.23E-27
YMR092C	12:1059925	9.33E-26
YMR092C	13:445622	3.39E-89
YCR072C	15:150651	1.18E-26
YGL130W	12:681096	1.44E-23
YGL130W	15:174364	4.46E-26
YGL263W	12:1019347	1.14E-32
YBR193C	2:582419	1.44E-65
YCL040W	13:46084	3.15E-26
YCL040W	15:174364	4.86E-37
YBR150C	2:555596	2.59E-60
YOR288C	15:179289	6.57E-24
YOL088C	15:170945	2.42E-31
YBL061C	15:174364	2.19E-40
YER044C	12:662627	5.65E-71
YGL051W	7:403626	1.39E-98
YKL068W-A	2:553812	3.06E-23
YLL057C	12:27765	1.96E-42
YNL310C	14:33607	5.70E-33
YLR025W	12:195531	6.61E-47
YML007W	13:245674	4.82E-43
YDR329C	15:174364	6.46E-32
YER060W	15:175594	1.35E-27
YGL237C	15:154309	2.29E-39
YLR425W	12:662627	3.42E-25
YAL016W	12:674651	3.07E-29
YBL042C	5:116722	6.85E-24
YOR342C	2:567221	8.38E-50
YDL204W	15:174364	2.80E-50
YGL056C	12:659357	7.97E-32
YHR104W	15:170945	4.64E-45
YML034W	8:111679	5.17E-23
YBL046W	2:133749	5.86E-33
YGR212W	7:913059	7.23E-37
YBR177C	15:144659	3.72E-51
YCR092C	10:510007	4.05E-28
YEL075C	3.956944444	1.11E-61
YPR143W	15:136327	2.09E-24
YFL022C	6:100521	2.29E-23
YFL022C	15:143597	2.82E-23
YDR333C	2:555575	1.96E-32
YER018C	5:183958	5.91E-60
YHR147C	15:180222	3.91E-26
YOL100W	15:113261	3.44E-42

YNL256W	15:174364	2.57E-35
YBR147W	15:150651	4.27E-25
YML104C	13:49894	7.68E-33
YOR194C	15:703771	9.23E-40
YOR134W	2:562415	2.61E-22
YIR018C-A	9:386046	1.02E-37
YPL144W	16:280629	5.54E-64
YER072W	13:27644	2.29E-83
YML079W	13:110808	1.13E-27
YPL018W	13:28694	4.34E-35
YDL027C	15:136327	1.53E-27
YAL024C	2:570229	4.06E-36
YPL274W	13:910381	1.16E-47
YIL165C	2:551299	1.51E-25
YIL165C	9:33795	1.72E-78
YER158C	5:420595	2.31E-39
YJR047C	12:635380	7.22E-42
YJR047C	16:497425	4.45E-28
YBR126C	15:174364	2.38E-46
YER185W	5:568716	7.26E-47
YNR001C	15:174364	5.25E-25
YKL079W	13:99675	3.75E-23
YAL039C	12:672779	6.27E-28
YHR035W	8:176994	1.03E-45
YJL105W	12:659357	6.07E-25
YDL124W	4:246738	1.79E-50
YDL124W	15:144659	4.01E-36
YJL078C	2:567221	1.14E-90
YDR135C	15:141627	3.97E-29
YGR195W	15:154177	9.59E-30
YMR036C	13:328865	4.93E-76
YNR014W	13:46084	4.92E-25
YNR014W	15:174364	9.56E-63
YLR329W	12:790025	2.34E-42
YLR272C	12:672785	1.52E-23
YGL151W	15:106266	1.85E-22
YKR102W	11:656099	3.60E-98
YLR420W	5:116722	9.63E-49
YLR420W	12:965218	2.58E-41
YGR231C	15:170945	5.67E-32
YGR007W	12:705100	1.66E-24
YCR107W	6.628472222	6.08E-22
YCR107W	10:28306	1.98E-35
YCR107W	0.868055556	3.16E-36
YCR107W	12:1059806	2.21E-35
YDR249C	8:111683	4.49E-24
YOR346W	15:968429	1.18E-65

YGL167C	13:69114	2.12E-24
YLR178C	15:174364	2.15E-58
YEL040W	5:79647	9.97E-31
YEL040W	15:143597	4.99E-27
YMR316W	13:57145	2.58E-31
YDL198C	4:95527	1.72E-28
YOL156W	15:108577	3.72E-25
YOR114W	15:524972	4.39E-38
YOR020W-A	15:407690	1.68E-28
YLR083C	15:174364	1.29E-22
YML069W	13:130069	9.06E-25
YCL016C	3:79091	2.06E-46
YJR094W-A	15:170945	7.34E-25
YHR160C	15:174364	3.29E-30
YGR207C	15:108577	3.12E-39
YDR070C	15:174364	3.90E-48
YJR090C	10:586311	6.50E-26
YGR049W	12:662627	1.46E-66
YBR196C-A	2:616262	2.40E-92
YGR057C	15:136324	1.67E-24
YIL060W	9:246551	4.10E-27
YIL106W	2:569420	1.17E-22
YIL106W	12:681096	1.42E-24
YIL106W	15:180961	1.33E-24
YOR377W	12:662627	9.44E-49
YBR092C	15:144659	7.83E-27
YEL073C	5:15817	1.73E-33
YBR030W	15:174364	2.51E-29
YDR033W	4:509817	4.64E-87
YDL132W	14:449639	4.73E-54
YGL137W	14:449639	1.79E-54
YNL112W	14:412269	6.94E-42
YMR321C	13:922256	1.32E-68
YCR028C	15:141627	1.31E-24
YBR056W-A	13:69122	1.10E-27
YLR174W	12:514835	1.41E-26
YJL112W	15:154177	1.99E-25
YDR115W	15:180222	1.97E-23
YPL063W	13:124876	6.11E-26
YIL020C	15:174364	9.78E-28
YAL063C	5.234722222	1.46E-62
YAL063C	8:525664	8.00E-25
YMR310C	15:143597	3.07E-27
YDR403W	13:99672	5.50E-28
YPL039W	6.628472222	2.24E-26
YPL039W	16:489143	8.93E-75
YLR164W	2:537314	8.79E-30

YLR164W	12:501510	7.32E-38
YML006C	15:96849	8.05E-25
YLR114C	2:533262	6.17E-24
YGR134W	15:141627	1.97E-24
YGR255C	15:174364	2.12E-36
YFR010W	6:144755	2.36E-30
YNL168C	2:553812	1.59E-45
YDR482C	1:199760	3.73E-21
YDR482C	13:27644	5.40E-53
YCR035C	15:170945	8.37E-28
YPL123C	2:517365	1.63E-31
YPL123C	15:143597	3.41E-24
YMR217W	15:174364	8.11E-23
YDL165W	4:143910	1.25E-30
YER058W	15:154177	8.95E-26
YKR061W	2:551299	3.56E-22
YLR435W	15:174364	9.87E-27
YFL014W	15:143597	3.03E-65
YJL084C	13:100048	2.34E-27
YNL288W	2:499895	5.01E-22
YLR450W	12:1031688	3.08E-44
YLL012W	12:679808	2.49E-38
YLL012W	15:174364	2.68E-32
YLR003C	15:174364	5.07E-25
YOR011W	15:384923	1.40E-25
YCR107W	6.628472222	3.75E-37
YCR107W	0.868055556	1.21E-43
YCR107W	12:1054278	6.79E-52
YLR350W	12:829693	2.74E-35
YOR118W	12:659357	1.74E-29
YBR044C	2:328489	9.99E-45
YLR438C-A	12:1019347	6.36E-33
YNL059C	14:525061	1.44E-52
YNR065C	14:732029	5.89E-27
YMR285C	2:521415	1.05E-28
YBL040C	2:142262	6.54E-59
YGR060W	12:677957	3.33E-36
YGR060W	15:180222	9.19E-24
YAR023C	1:185122	6.94E-67
YJL001W	10:451832	3.55E-45
YPR160W	15:174364	4.36E-46
YLR411W	12:956534	6.58E-95
YBR242W	12:662627	1.59E-70
YLR121C	15:143597	8.13E-31
YJL219W	10:23505	5.40E-91
YNL041C	14:553129	4.34E-40
YMR160W	15:174364	1.33E-27

YKR013W	2:533268	3.99E-49
YKR013W	11:468771	1.10E-36
YAR075W	13:180103	2.72E-29
YNR034W-A	15:174364	3.04E-42
YHR048W	12:659357	1.93E-55
YHL044W	6.176388889	1.58E-98
YER034W	13:57145	3.19E-23
YPL004C	13:28622	2.07E-25
YPL004C	15:174364	1.58E-47
YKL005C	11:482069	2.11E-29
YER053C-A	12:662627	1.21E-78
YDR049W	4:555043	1.45E-29
YLR201C	15:174364	2.13E-32
YOL096C	15:154309	1.24E-48
YJL167W	12:662627	1.35E-80
YNL141W	13:27644	1.35E-25
YDR298C	13:49894	3.74E-28
YDR298C	15:136327	5.39E-40
YDR298C	16:500342	2.13E-31
YDR242W	2:555575	4.68E-45
YPL085W	2:530481	1.54E-24
YER119C	2:555596	1.63E-26
YMR015C	12:659357	3.48E-65
YMR139W	15:154309	8.56E-27
YHR143W	2:555596	2.93E-93
YPR149W	13:28622	1.91E-23
YPR149W	15:174364	2.54E-51
YIL120W	9:136845	3.21E-30
YGL255W	13:69122	6.04E-34
YGL255W	15:174364	8.63E-33
YBR093C	13:27644	2.11E-79
YAL009W	1:136161	4.29E-64
YHL009W-B	8:84437	7.38E-49
YHL009W-B	16:442503	5.37E-40
YGR061C	13:49894	1.71E-37
YLR047C	12:238298	4.16E-32
YFR049W	15:154309	7.19E-30
YML046W	8:111683	8.82E-43
YLR126C	13:27644	9.56E-22
YLR053C	2:562415	1.09E-30
YMR238W	13:46084	2.65E-31
YMR238W	15:174364	1.03E-23
YGR165W	15:180210	3.54E-25
YCR097W	3:201166	5.12E-97
YIL066C	12:514835	2.60E-57
YBR158W	2:567221	2.11E-89
YPL203W	15:174364	2.33E-29

YMR195W	15:143597	1.47E-37
YMR195W	16:500348	7.75E-24
YFR027W	6:205881	2.21E-60
YGR094W	15:154177	2.88E-26
YJR144W	15:563943	6.28E-26
YJR144W	16:511400	1.17E-25
YMR108W	3:91305	9.33E-48
YMR108W	15:180222	1.96E-25
YDR434W	15:106266	8.74E-29
YBR119W	2:480009	2.80E-65
YBR119W	8:95289	1.71E-21
YFR006W	13:46070	4.56E-25
YFR006W	15:141633	3.52E-45
YFR006W	16:500342	8.58E-29
YEL041W	5:73010	3.84E-58
YBR133C	8:111680	5.95E-30
YMR098C	15:174364	1.07E-26
YLR162W-A	12:472165	4.16E-94
YML082W	15:150651	9.62E-33
YHR032W	8:167504	4.74E-51
YDR093W	2:480009	1.39E-24
YNL237W	14:209852	1.57E-84
YDR523C	4:1497132	1.03E-27
YOR116C	2:555575	3.11E-28
YOR116C	15:150651	1.97E-23
YOR031W	15:392765	7.38E-86
YJL180C	15:174364	4.86E-24
YFR031C	13:69114	4.30E-27
YFL041W-A	15:143597	2.15E-27
YDR379W	2:555787	2.28E-23
YDR379W	3:81832	6.64E-23
YDR281C	13:27644	1.28E-71
YPL113C	13:28694	8.03E-32
YPL113C	15:143597	1.16E-36
YPL270W	13:28694	2.53E-25
YIL145C	15:143597	8.29E-36
YMR252C	12:681096	3.59E-29
YIR017C	5:251647	1.58E-28
YER132C	15:143597	1.20E-25
YLL023C	12:99261	1.85E-50
YPL082C	2:506661	4.09E-39
YBR024W	15:180961	2.16E-23
YNL158W	12:634227	4.63E-26
YGR070W	15:174364	1.13E-34
YOL082W	2:553812	1.22E-24
YOL082W	15:179289	9.52E-56
YGR044C	2:555575	8.92E-65

YML029W	13:115474	1.21E-28
YNL238W	14:245307	1.95E-33
YPR028W	15:174364	4.84E-25
YOR229W	12:662627	2.94E-43
YPR192W	16:927500	2.70E-76

Supplementary Table 6: eQTL hot spot for the static method (T0)

Hot Spot 1:190000

Accession ID	p-value
YAR033W	3.04E-12
YFL020C	9.43E-11
YFL020C	1.44E-07
YAR027W	1.04E-15
YAR031W	5.02E-12
YPR001W	6.89E-05
YGL066W	2.61E-06
YAR020C	9.43E-11
YAR033W	1.54E-10
YAR028W	1.04E-15
YAR042W	1.59E-15
YAR075W	1.98E-12
YAR050W	1.49E-16
YEL049W	5.16E-14
YAR023C	2.30E-12

Hot Spot 1:50000

Accession ID	p-value
YJL196C	9.14E-06
YAL067C	7.55E-16
YKL188C	3.63E-06
YOR319W	1.87E-08
YAL064W-B	4.88E-16
YDR244W	5.43E-08
YPR128C	4.36E-07
YAL064W	7.28E-13
YAL062W	4.93E-13
YIR033W	4.58E-05
YNR016C	1.80E-06
YOR363C	1.84E-05
YOL002C	0.000169
YLR099W-A	1.98E-05
YJL181W	3.04E-05
YGR286C	3.71E-06
YGR037C	2.58E-06
YPL231W	2.64E-07
YAL065C	2.48E-12
YAL049C	1.69E-12
YOL101C	3.77E-09
YAL063C-A	3.52E-10
YAL040C	9.77E-06
YAL056W	2.69E-15
YHR213W	8.46E-09
YKL182W	3.19E-06
YER081W	0.000328

YAL063C	6.43E-09
Hot Spot 2:550000	
Accession ID	p-value
YJL147C	3.77E-05
YNL126W	1.40E-05
YDR056C	4.66E-05
YLR286C	7.71E-16
YGR113W	9.45E-08
YPR106W	1.12E-16
YDR072C	1.48E-08
YBR157C	1.24E-13
YOR264W	2.22E-15
YKL108W	4.50E-05
YNL118C	5.37E-05
YFL047W	5.87E-08
YDR261C	7.47E-05
YDR118W	2.54E-05
YML023C	0.000123
YGL036W	4.00E-06
YHR053C	1.01E-10
YKR068C	2.71E-05
YJL200C	1.13E-10
YNR067C	2.10E-16
YDL055C	6.99E-13
YML124C	7.81E-10
YIL009W	4.37E-08
YPL262W	6.97E-07
YLR313C	9.23E-05
YDL119C	2.99E-05
YJL108C	7.72E-07
YNR027W	0.000183
YIR034C	2.34E-06
YEL036C	5.38E-05
YOL112W	1.42E-10
YIL094C	4.54E-12
YLR233C	1.89E-05
YDR353W	2.42E-06
YBR291C	2.58E-13
YAR014C	1.10E-08
YJR014W	1.67E-06
YBR197C	1.03E-16
YLR260W	7.17E-05
YER124C	5.33E-16
YBR135W	8.97E-10
YOR228C	0.000105
YHR049W	9.73E-06
YDR234W	1.55E-08

YIR030C	1.16E-05
YBR162C	1.55E-05
YKL103C	7.73E-06
YBR023C	1.74E-08
YNL217W	1.22E-05
YPL066W	4.66E-07
YPR005C	1.82E-06
YLR103C	0.000114
YOR246C	1.01E-05
YLR457C	4.56E-05
YOR217W	0.000226
YMR078C	0.000135
YER089C	1.69E-07
YNL161W	7.79E-07
YDR272W	0.000205
YDR506C	1.23E-05
YDL182W	2.18E-10
YDL230W	5.31E-05
YBR165W	2.05E-10
YLR356W	1.75E-05
YPR165W	3.18E-06
YPR141C	3.36E-06
YPR111W	2.73E-09
YMR055C	5.52E-05
YGL185C	9.37E-08
YNR050C	1.66E-12
YKR010C	1.86E-05
YKL185W	5.42E-12
YBL016W	1.80E-05
YHR030C	3.86E-08
YGR217W	3.56E-07
YLR319C	6.15E-06
YBL037W	7.53E-06
YFL005W	2.14E-05
YGR216C	1.31E-05
YGL229C	2.37E-11
YJR054W	3.27E-08
YML118W	2.68E-07
YBR228W	1.18E-08
YNL015W	3.86E-09
YIL140W	6.21E-08
YPR086W	4.70E-05
YBL034C	4.66E-05
YBR173C	3.03E-12
YKR100C	7.01E-06
YNR016C	3.95E-05
YOR316C-A	5.93E-12

YJL187C	4.64E-06
YGL004C	1.37E-07
YHR154W	2.82E-05
YER109C	4.09E-08
YGR041W	3.02E-15
YER154W	6.17E-08
YOL019W	5.17E-06
YER095W	1.56E-05
YOR126C	1.43E-07
YBL060W	1.25E-07
YOR278W	4.36E-05
YJL099W	4.00E-07
YGL164C	4.62E-05
YNL090W	1.13E-05
YNL106C	3.45E-06
YNL293W	0.000171
YJL115W	7.03E-06
YNL142W	3.55E-07
YIL117C	5.65E-11
YNL298W	1.79E-07
YER063W	2.13E-08
YJL092W	1.96E-05
YNL283C	2.03E-08
YMR165C	2.56E-05
YBR199W	4.29E-10
YKR028W	7.12E-05
YGR032W	2.05E-05
YNL066W	6.41E-15
YBR132C	1.24E-14
YLR353W	1.34E-10
YBR203W	1.59E-05
YNR028W	5.26E-06
YJL145W	7.13E-05
YNL268W	7.02E-10
YMR031C	5.47E-05
YOR262W	1.16E-05
YNL327W	1.93E-15
YIR029W	2.32E-05
YMR305C	8.15E-10
YDR326C	5.30E-05
YPR101W	3.78E-05
YHR117W	2.56E-05
YOR070C	7.25E-05
YGR286C	7.74E-05
YMR182W-A	9.15E-09
YBR103W	4.07E-12
YBR196C-B	8.43E-15

YOR066W	1.83E-05
YOR261C	1.44E-05
YPR157W	1.88E-06
YOR184W	6.06E-05
YOR386W	4.68E-07
YPL164C	0.000116
YJL159W	2.55E-06
YPL011C	9.72E-05
YJL116C	4.14E-05
YOL083W	5.76E-05
YBR159W	2.16E-05
YBR115C	2.09E-13
YPL231W	6.27E-05
YOR127W	9.94E-09
YDR507C	6.36E-11
YNL282W	5.50E-05
YGR040W	1.65E-12
YER153C	2.45E-11
YLR373C	1.93E-05
YBR248C	0.000178
YJL174W	9.86E-07
YIL024C	1.49E-08
YLR229C	2.60E-08
YDR260C	1.70E-05
YKL050C	4.37E-05
YDL073W	0.000356
YOR296W	1.01E-10
YBR107C	2.15E-14
YPL109C	0.000216
YMR073C	0.000155
YDL144C	6.00E-06
YDL006W	8.65E-05
YHR076W	3.58E-06
YHR142W	5.42E-13
YLR084C	0.000118
YNL239W	6.86E-05
YPR159W	3.43E-07
YHR083W	6.39E-05
YBR189W	6.01E-07
YPL163C	5.37E-08
YNL046W	1.90E-11
YKL218C	1.08E-05
YDR132C	5.49E-05
YDR077W	2.14E-05
YMR062C	0.000258
YJL077W-B	2.89E-07
YDR256C	6.04E-06

YOR188W	3.02E-07
YMR181C	3.58E-06
YNL053W	1.29E-06
YOR208W	3.09E-06
YDL131W	1.68E-07
YCL014W	1.65E-05
YAR018C	8.76E-07
YLR332W	2.92E-09
YBL071C-B	2.35E-07
YJR005W	6.94E-06
YER075C	2.32E-08
YGL028C	5.03E-16
YFL021W	3.55E-12
YBR073W	5.23E-08
YMR127C	0.000105
YPR054W	2.82E-05
YGR147C	8.88E-06
YER048C	0.000126
YAL044W-A	2.07E-05
YGR121C	0.000117
YBR170C	1.32E-12
YBR148W	4.05E-13
YLR250W	8.51E-06
YOR230W	3.13E-06
YBR156C	6.28E-14
YER118C	5.65E-11
YMR174C	2.37E-06
YER130C	2.72E-05
YKL182W	1.33E-06
YFL031W	7.60E-06
YLR285C-A	1.01E-14
YHR107C	2.63E-06
YLL054C	1.46E-05
YLR042C	8.95E-15
YJL107C	3.30E-06
YMR270C	7.86E-05
YMR129W	0.000176
YMR032W	1.18E-05
YGR096W	3.22E-05
YMR173W	1.10E-06
YLR312C-B	2.46E-06
YGR100W	2.65E-05
YBL002W	0.000171
YNL040W	9.53E-07
YKL179C	4.40E-08
YML109W	1.10E-08
YJR006W	6.90E-07

YPR105C	3.02E-08
YNL078W	5.58E-14
YCL028W	1.07E-05
YBR117C	1.68E-14
YJL118W	8.94E-05
YBR204C	2.28E-10
YGL035C	7.61E-05
YLR414C	1.81E-05
YEL004W	6.43E-07
YMR200W	2.72E-10
YOL007C	9.51E-09
YNL289W	9.29E-07
YMR163C	3.50E-08
YOR129C	3.29E-07
YPL089C	2.25E-07
YDL179W	7.04E-10
YJR147W	5.58E-14
YPL158C	8.68E-11
YDR082W	5.96E-06
YLR080W	0.000266
YBR163W	9.06E-09
YER152C	4.71E-16
YOR316C	3.68E-05
YGR014W	2.06E-13
YDL078C	3.72E-06
YKL104C	1.69E-05
YJR153W	1.50E-06
YBL085W	1.80E-06
YBR208C	0.000124
YLR118C	0.000148
YNL294C	9.94E-06
YLR079W	1.72E-11
YBR098W	9.58E-05
YOR247W	1.24E-14
YPR194C	8.88E-06
YLR258W	1.26E-09
YAL041W	2.01E-07
YGR152C	9.58E-05
YBR193C	4.43E-11
YBR150C	3.69E-11
YBR182C	3.71E-05
YIR001C	2.88E-05
YDR305C	6.68E-05
YDR333C	6.65E-06
YDL102W	0.00018
YAL024C	7.10E-10
YJL078C	1.54E-15

YDR249C	9.40E-07
YBR196C-A	2.22E-15
YLR114C	1.36E-05
YNL168C	2.84E-06
YPL123C	0.000183
YKR061W	3.90E-08
YBR176W	2.21E-11
YNL059C	4.11E-05
YLR121C	6.98E-05
YKR013W	6.18E-10
YHR143W	3.91E-16
YKR091W	1.03E-09
YLR284C	2.16E-05
YBR158W	3.85E-15
YBR119W	2.85E-12
YDR379W	5.87E-08
YLR417W	7.19E-06
YOL082W	3.70E-05
YGR044C	1.93E-15

Hot Spot 2:430000

Accession ID	p-value
YMR182C	5.13E-06
YOR124C	1.75E-05
YBR095C	0.000269
YJL068C	5.85E-05
YMR237W	1.17E-05
YDL237W	1.17E-05
YHR113W	9.48E-05
YBR056W	2.02E-05
YGR141W	2.31E-05
YDR043C	1.24E-07
YNL270C	1.83E-07
YOR101W	5.09E-05
YGR015C	3.53E-05
YBR097W	9.65E-07
YBR094W	1.64E-12
YGR263C	0.000126
YBR060C	2.44E-09
YMR016C	2.39E-11
YBR091C	4.10E-05
YPL091W	0.000171
YBR104W	9.18E-05
YPR098C	1.34E-05
YBR068C	1.86E-08
YDR204W	5.15E-05
YBR080C	9.35E-06
YDL190C	3.30E-05

YPL219W	1.01E-05
YBR078W	1.64E-07
YJR103W	0.000199
YDR026C	0.000261
YPL082C	2.40E-05
Hot Spot 3:90000	
Accession ID	p-value
YOR271C	1.85E-13
YMR291W	3.62E-07
YCL018W	6.44E-16
YPL189W	0.000116
YKL121W	6.06E-06
YHR063C	4.25E-05
YER073W	1.04E-12
YBR085C-A	2.48E-05
YKR071C	8.47E-12
YPL084W	9.95E-09
YLR355C	0.000111
YJR016C	1.55E-10
YCL017C	2.18E-12
YLR348C	6.55E-11
YEL032W	8.13E-07
YCR018C	3.89E-12
YBR068C	1.65E-13
YMR018W	1.25E-05
YHR047C	6.85E-05
YIL050W	1.36E-06
YNL241C	9.49E-07
YCL052C	3.82E-06
YKR095W-A	1.60E-07
YOR226C	3.21E-14
YKL029C	4.96E-08
YCL026C-B	6.70E-15
YBL029W	3.93E-05
YCL012C	2.41E-08
YGL009C	3.90E-15
YOR227W	1.21E-05
YDR444W	9.95E-05
YGL125W	4.24E-06
YJL037W	1.29E-05
YNL104C	6.38E-09
YKL120W	6.36E-15
YOR375C	5.77E-13
YHR208W	4.69E-15
YCL004W	6.66E-07
YCL021W-A	8.82E-16
YMR108W	5.06E-12

Hot Spot 3:170000

Accession ID	p-value
YCL068C	3.83E-07
YKL208W	6.01E-07
YDR460W	5.60E-13
YGL089C	8.94E-17
YCR026C	1.08E-08
YCR052W	1.02E-09
YLR040C	8.94E-17
YML131W	2.57E-05
YKL178C	8.94E-17
YIL017C	4.06E-14
YKR027W	7.41E-08
YNL146C-A	8.94E-17
YFL026W	8.94E-17
YKL209C	8.94E-17
YJR004C	8.94E-17
YPL188W	2.22E-09
YPL187W	8.94E-17
YJL191W	1.73E-08
YFL027C	3.84E-16
YNL146W	1.15E-16
YCR096C	1.15E-16
YNL145W	8.94E-17
YDR461W	8.94E-17
YGL032C	8.94E-17
YPR058W	3.18E-06
YJL170C	8.94E-17
YIL015W	8.94E-17
YHR019C	1.06E-05
YCL066W	8.94E-17
YCR038C	6.40E-07
YJL171C	1.05E-09
YCR097W	8.94E-17

Hot Spot 7:410000

Accession ID	p-value
YER035W	7.68E-09
YJL097W	0.000218
YJL196C	8.31E-06
YGL057C	3.02E-13
YPR174C	1.76E-14
YGL063W	1.73E-10
YGL066W	1.41E-08
YJL144W	8.61E-14
YDR246W-A	3.76E-05
YGL229C	0.000472
YOL002C	4.33E-10

YPL240C	1.05E-10
YGL050W	1.83E-06
YNL077W	1.08E-15
YNL006W	7.29E-11
YGL049C	9.22E-10
YGL053W	4.90E-17
YDR151C	2.81E-08
YFL016C	2.94E-08
YOL101C	3.80E-05
YGL067W	9.06E-10
YNL007C	7.81E-15
YDR171W	7.48E-06
YGL075C	1.11E-10
YOR027W	1.56E-09
YPR158W	1.23E-11
YGL081W	4.26E-06
YGR142W	3.20E-13
YGL035C	2.06E-09
YMR177W	1.26E-05
YDR492W	7.40E-10
YKR075C	3.76E-05
YBR101C	1.28E-09
YGL051W	4.90E-17

Hot Spot 8:110000

Accession ID	p-value
YHR014W	1.52E-08
YHL010C	1.77E-14
YHR152W	5.77E-06
YDR207C	5.54E-05
YHR005C	3.62E-06
YHL020C	9.77E-09
YKL048C	0.000353
YMR274C	0.000308
YHL006C	9.90E-10
YCL027W	2.44E-13
YBL016W	8.62E-08
YBR040W	3.36E-12
YPR122W	7.22E-07
YHL009C	2.04E-10
YHR018C	1.51E-05
YER016W	1.38E-06
YPL192C	3.41E-08
YGR109W-A	0.000165
YIL016W	1.82E-05
YHL022C	2.24E-07
YDL128W	0.000114
YJL050W	4.29E-05

YLR357W	1.26E-05
YHL026C	1.22E-13
YOR066W	0.000129
YIL037C	5.04E-10
YLR442C	8.81E-06
YCR089W	6.30E-10
YNL279W	1.18E-09
YHR015W	2.00E-14
YGL116W	1.71E-06
YNL326C	5.74E-05
YGL223C	0.000141
YPR115W	1.14E-07
YHR007C	2.65E-06
YMR065W	5.26E-11
YNL215W	8.21E-06
YHL009W-A	3.28E-07
YBR225W	9.21E-05
YDR304C	2.16E-05
YHR061C	1.84E-06
YOR123C	3.26E-05
YKL189W	1.43E-06
YDR503C	3.04E-05
YGL060W	1.27E-06
YER155C	6.94E-07
YGR109W-B	0.000193
YDR446W	5.00E-06
YIL082W-A	0.000155
YML047C	4.16E-10
YNL278W	3.27E-06
YGL106W	1.40E-06
YJL157C	4.63E-12
YJL051W	8.64E-05
YHL013C	3.82E-05
YLR452C	3.19E-12
YCL055W	1.14E-12
YDR085C	3.24E-06
YOR219C	0.00015
YNR044W	9.43E-13
YKR089C	6.55E-07
YBR083W	3.65E-05
YHL003C	8.44E-09
YER099C	2.74E-05
YHL023C	6.17E-11
YBR092C	0.000126
YPL139C	6.88E-05
YHL009W-B	6.62E-09
YML046W	7.62E-05

YBR133C	1.99E-06
YLR417W	1.81E-05
YPL082C	0.000261

Hot Spot 12:670000

Accession ID	p-value
YNR074C	2.48E-05
YOR175C	8.98E-08
YML019W	1.27E-07
YDL086W	1.05E-14
YKL075C	1.34E-05
YMR212C	1.22E-05
YMR220W	2.56E-14
YNL291C	5.76E-05
YLR283W	2.76E-14
YDR518W	1.15E-07
YDR331W	2.18E-05
YDR261C	3.44E-06
YDL176W	8.57E-06
YLR299W	1.07E-06
YBR215W	1.49E-05
YLR300W	3.68E-13
YKR086W	4.70E-05
YEL051W	2.99E-05
YML125C	8.62E-08
YGR071C	1.50E-05
YLR256W	2.41E-14
YHR146W	0.000106
YJL108C	3.23E-07
YOR321W	1.14E-06
YLR248W	3.00E-06
YNL116W	6.13E-06
YJL100W	6.75E-10
YGL001C	2.87E-14
YLR265C	1.93E-13
YNR043W	9.02E-13
YGR198W	4.35E-05
YLR020C	3.35E-05
YPR062W	3.70E-06
YLR260W	7.91E-08
YOR003W	6.62E-11
YKR088C	3.62E-07
YLR237W	5.51E-08
YGR266W	1.24E-09
YHR129C	4.70E-05
YER093C	4.95E-06
YFR033C	8.98E-10
YLR275W	1.44E-07

YML008C	5.82E-12
YMR298W	5.65E-07
YLR038C	1.36E-06
YIL121W	2.15E-16
YLR271W	0.000161
YJR134C	9.56E-07
YHR072W	6.99E-13
YMR202W	1.83E-15
YGR177C	3.69E-05
YGL096W	3.50E-05
YML075C	1.05E-14
YHR140W	6.98E-05
YLR231C	6.62E-13
YLR214W	6.58E-08
YDR502C	3.79E-13
YER136W	2.10E-06
YOR217W	0.000106
YLR234W	2.56E-10
YDR292C	1.88E-06
YNL111C	7.43E-10
YGL055W	4.17E-10
YDR520C	0.000124
YBL067C	1.31E-06
YDR213W	2.16E-06
YGR089W	7.36E-07
YDR297W	4.82E-10
YDR453C	9.47E-06
YPR191W	0.000117
YPR151C	6.62E-13
YML032C	5.96E-06
YER100W	8.17E-06
YDL133W	4.36E-05
YDR186C	3.36E-07
YER024W	0.000192
YLR244C	7.18E-15
YPR118W	4.72E-05
YEL047C	5.14E-07
YBR016W	3.47E-05
YKL072W	1.10E-05
YDR147W	4.04E-05
YLR270W	7.79E-06
YDR346C	4.03E-06
YJR110W	2.88E-06
YGR026W	9.78E-07
YOR073W	1.44E-05
YDR323C	2.92E-07
YMR208W	3.05E-14

YGL225W	8.05E-07
YEL034W	1.50E-14
YHR207C	9.48E-05
YGL160W	1.51E-08
YPL263C	6.61E-06
YLR056W	2.32E-07
YLR246W	2.87E-15
YJL048C	5.01E-13
YDL093W	1.44E-09
YDR276C	3.89E-06
YGL101W	1.02E-06
YBR067C	1.66E-09
YOR332W	7.60E-06
YLR253W	2.62E-05
YHR039C-A	5.94E-06
YBR106W	1.54E-06
YBR286W	2.04E-09
YKL080W	3.15E-09
YLR213C	1.26E-08
YLR245C	3.59E-12
YPR086W	5.25E-06
YIR033W	4.01E-06
YAL028W	2.37E-08
YOR010C	1.17E-05
YOR034C	4.47E-06
YDR372C	4.46E-05
YPL168W	9.10E-06
YJR113C	1.41E-05
YCR048W	1.29E-07
YGR281W	1.67E-06
YBR183W	2.73E-06
YNR010W	3.87E-06
YGL040C	4.01E-08
YNL261W	8.81E-05
YLR205C	6.93E-15
YKL004W	1.59E-06
YER014W	2.95E-12
YOL002C	0.0001
YJR049C	9.87E-07
YJR048W	5.52E-12
YOL026C	9.95E-05
YDL099W	1.57E-06
YOR007C	3.08E-06
YKL008C	6.18E-09
YLR395C	7.69E-06
YPL159C	1.17E-05
YLR273C	1.12E-05

YJL095W	6.06E-05
YHR008C	3.17E-07
YDR275W	9.50E-12
YJR001W	1.17E-05
YEL039C	3.16E-06
YCR061W	2.10E-07
YML028W	4.02E-05
YIR035C	1.79E-05
YER122C	1.62E-06
YDR326C	1.85E-05
YJR126C	1.30E-05
YDL084W	9.19E-06
YNL052W	3.59E-05
YMR110C	4.77E-08
YOR018W	4.43E-06
YNR019W	3.57E-15
YKL077W	9.52E-06
YKR046C	8.29E-15
YER012W	6.63E-06
YNR007C	2.83E-05
YJR104C	6.14E-10
YMR223W	2.74E-05
YLR153C	9.92E-15
YEL035C	1.13E-05
YMR038C	1.93E-10
YDL020C	4.77E-06
YBR042C	9.39E-08
YOR334W	6.63E-08
YPL028W	2.34E-11
YHR007C	9.77E-06
YKR051W	2.08E-05
YDL073W	1.13E-05
YEL024W	5.00E-09
YNL003C	5.44E-10
YPR124W	6.02E-05
YLR288C	6.25E-14
YGL150C	5.76E-05
YER043C	3.21E-07
YNL010W	6.40E-10
YLR254C	6.11E-05
YMR070W	1.06E-08
YHR190W	1.10E-11
YNR006W	1.25E-05
YML126C	1.08E-14
YPR040W	1.39E-07
YGL191W	2.06E-08
YLR281C	4.77E-09

YJL204C	0.000191
YOR348C	2.65E-05
YLR100W	1.72E-13
YGL017W	5.30E-06
YLR372W	7.52E-05
YNL218W	0.000331
YOR324C	4.49E-06
YCR008W	0.000102
YBR161W	6.43E-07
YGR230W	3.65E-05
YLR361C	8.20E-06
YDR011W	4.77E-06
YML058W-A	1.91E-06
YHL017W	7.48E-05
YMR100W	4.52E-08
YNR049C	2.79E-05
YOR065W	1.65E-08
YLR290C	1.70E-07
YHR179W	1.47E-06
YNL173C	5.38E-07
YDR229W	0.000101
YBR085W	9.41E-07
YDR529C	3.82E-06
YGL139W	3.28E-05
YIL031W	1.54E-05
YJL034W	9.58E-05
YOR085W	1.70E-07
YPR095C	3.88E-07
YLR380W	7.80E-07
YJL107C	7.39E-06
YPR024W	3.50E-05
YHR210C	1.19E-06
YIL007C	2.30E-09
YPR113W	8.50E-07
YKR045C	2.18E-06
YER145C	2.01E-05
YHR039C	1.88E-12
YJL198W	8.51E-06
YNL151C	0.000129
YGR138C	6.16E-06
YIL062C	2.37E-05
YGR203W	3.57E-05
YGR234W	2.48E-15
YNL280C	2.06E-06
YDR384C	2.43E-08
YAR035W	0.000121
YNR060W	1.95E-08

YPR065W	4.59E-13
YOR320C	3.36E-05
YMR009W	7.72E-14
YOR237W	8.12E-10
YKL196C	8.84E-06
YMR134W	5.66E-16
YLR326W	1.81E-10
YEL048C	6.00E-05
YMR145C	1.29E-07
YOR270C	3.82E-06
YJL002C	6.13E-06
YKL150W	2.00E-06
YCR069W	1.53E-07
YBL058W	8.79E-06
YGL210W	1.65E-06
YAR029W	4.70E-05
YNL156C	2.14E-14
YPL117C	5.66E-08
YHL031C	2.60E-06
YER011W	1.91E-09
YBR160W	1.05E-05
YMR314W	2.93E-05
YMR231W	0.00027
YJR105W	1.67E-11
YHR004C	9.00E-08
YDL174C	1.49E-10
YOR077W	4.21E-05
YDR044W	7.43E-10
YER099C	0.000145
YLR315W	3.66E-05
YER044C	1.69E-14
YLR425W	3.47E-06
YOR259C	4.64E-05
YML061C	1.20E-06
YJR064W	1.54E-05
YJR047C	2.32E-08
YLR272C	7.32E-09
YGR007W	3.08E-07
YGR049W	6.14E-10
YOR377W	9.98E-11
YNL159C	1.10E-06
YBR187W	4.59E-05
YLR450W	1.26E-07
YLL012W	5.36E-09
YMR119W	4.83E-06
YHR026W	8.22E-06
YGR060W	9.88E-12

YBR242W	2.95E-11
YHR048W	3.16E-10
YER034W	1.85E-05
YER053C-A	6.53E-15
YCR073W-A	4.18E-05
YJL167W	2.36E-14
YMR015C	7.25E-14
YGL233W	0.00018
YDR435C	2.99E-05
YGL144C	5.14E-07
YEL027W	0.000118
YNL158W	2.32E-05
YOR229W	1.18E-09

Hot Spot 13:70000

Accession ID	p-value
YDR481C	7.85E-14
YML035C	7.75E-06
YDL167C	0.000199
YIL125W	4.65E-05
YGR080W	0.000148
YGR010W	2.26E-06
YJL186W	7.39E-07
YHR079C	0.00013
YER053C	1.60E-06
YLR089C	1.22E-07
YBR296C	3.29E-14
YML064C	4.49E-07
YHR124W	7.07E-07
YKL085W	6.22E-05
YML091C	3.64E-10
YLR137W	4.35E-05
YBR011C	5.34E-05
YBR028C	5.50E-07
YHR097C	1.16E-05
YJR121W	9.70E-05
YPL165C	3.10E-05
YBR240C	7.84E-07
YML093W	2.09E-07
YMR087W	4.41E-05
YJL088W	2.20E-08
YPR069C	3.43E-06
YML067C	3.45E-06
YER069W	2.45E-05
YML123C	4.49E-15
YKL035W	1.26E-05
YBL068W	1.03E-06
YDL199C	9.56E-05

YKL048C	9.85E-05
YIL073C	3.45E-07
YNL217W	1.27E-07
YOL064C	1.25E-08
YDL139C	0.000338
YPL019C	1.19E-14
YDR217C	2.94E-05
YPR191W	1.50E-05
YLR410W	1.91E-13
YML096W	1.10E-10
YFL004W	1.51E-14
YNL045W	2.14E-06
YML057W	1.36E-10
YOR385W	1.51E-05
YJL131C	1.79E-05
YER073W	1.76E-05
YGR233C	9.34E-15
YJL137C	1.69E-05
YIR037W	0.000211
YPR091C	9.88E-06
YML070W	8.18E-05
YKL070W	3.84E-05
YLR108C	4.44E-07
YOL140W	6.98E-08
YDL181W	4.66E-05
YNL224C	8.65E-07
YDL225W	1.41E-06
YGR117C	1.40E-05
YDR369C	2.77E-07
YGR146C	1.26E-05
YOL130W	1.62E-07
YFR008W	1.06E-05
YLR295C	3.25E-05
YML078W	1.45E-15
YOR292C	6.79E-06
YER180C	1.36E-05
YIR002C	1.85E-06
YHR214C-E	4.02E-10
YFR044C	3.62E-07
YML087C	2.90E-07
YOR221C	5.14E-07
YCR037C	9.38E-13
YGL224C	6.26E-12
YIL074C	2.25E-06
YDL004W	1.45E-05
YJL012C	2.04E-14
YER055C	9.54E-07

YHL036W	7.64E-06
YLR355C	2.21E-10
YHR008C	7.03E-05
YNL155W	6.42E-06
YCR030C	3.17E-05
YDR017C	4.30E-06
YDR148C	2.94E-05
YDR309C	7.25E-09
YPL003W	2.97E-06
YLR348C	1.73E-06
YPR035W	5.52E-05
YOR313C	6.79E-07
YPL150W	5.77E-06
YML076C	3.64E-11
YLR168C	8.27E-06
YOL136C	3.47E-05
YML066C	1.22E-13
YER173W	7.59E-05
YNL148C	3.41E-06
YHR183W	3.09E-05
YML116W	5.33E-11
YIL172C	1.22E-06
YHR136C	1.61E-14
YJL117W	6.70E-14
YKL109W	1.81E-05
YLR438W	3.97E-14
YCR004C	6.11E-05
YDR342C	9.23E-06
YOR163W	1.04E-11
YMR300C	2.87E-09
YNR068C	7.88E-08
YDL059C	2.91E-05
YML071C	4.66E-06
YIL111W	4.20E-06
YHR047C	2.34E-07
YOR317W	1.70E-05
YPL268W	1.83E-12
YNR009W	4.63E-06
YLR409C	8.92E-05
YJR077C	9.70E-05
YIL050W	8.82E-07
YHR162W	1.21E-09
YDR399W	0.000157
YHR216W	1.02E-07
YLR431C	9.56E-05
YCL038C	7.44E-06
YML055W	1.46E-13

YML111W	5.74E-13
YNL187W	1.87E-05
YOR374W	9.99E-11
YOL016C	1.25E-05
YOR211C	6.20E-05
YNL173C	2.85E-05
YPR138C	5.94E-07
YIL051C	8.05E-16
YAR071W	2.60E-14
YHR102W	7.40E-05
YDR229W	2.53E-05
YML119W	3.86E-07
YKL141W	4.19E-06
YPL110C	4.70E-14
YKR093W	6.97E-08
YBR035C	3.47E-05
YER057C	4.90E-08
YGR243W	8.82E-07
YPL265W	3.12E-07
YER067W	7.90E-05
YPL014W	2.69E-05
YNR059W	0.000139
YDR277C	8.90E-06
YCL025C	7.36E-09
YML085C	1.38E-14
YGL234W	2.65E-06
YML074C	3.01E-06
YPL061W	2.17E-12
YKL016C	4.23E-05
YCL064C	2.04E-16
YNL012W	6.60E-08
YAL047C	4.44E-06
YPL111W	1.86E-14
YML121W	4.41E-12
YNR002C	5.52E-06
YKL051W	1.94E-12
YBR208C	4.79E-08
YNL097C	2.16E-05
YAR073W	4.04E-08
YCR038C	4.41E-06
YOR032W-A	3.36E-06
YOL058W	1.87E-05
YML061C	1.27E-05
YML104C	2.72E-10
YER072W	1.01E-13
YML079W	5.18E-11
YPL018W	8.29E-14

YKL079W	6.63E-05
YNR014W	8.53E-06
YBR056W-A	3.33E-05
YPL063W	2.48E-06
YDR482C	4.70E-08
YER039C-A	2.79E-06
YAR075W	7.50E-07
YKL198C	9.01E-06
YPL004C	1.02E-05
YNL141W	2.75E-05
YDR298C	3.59E-05
YPL100W	1.08E-05
YPR149W	7.54E-05
YIL120W	2.11E-06
YEL071W	3.08E-06
YGL255W	1.62E-08
YBR093C	4.22E-15
YGR061C	1.15E-09
YDL001W	1.39E-05
YFR006W	1.34E-05
YFR031C	3.47E-05
YDR281C	5.29E-14
YGL144C	9.21E-05

Hot Spot 13:910000

Accession ID	p-value
YBR110W	4.21E-05
YLL051C	5.37E-05
YOR390W	0.000404
YMR318C	9.47E-06
YKL220C	7.44E-05
YMR071C	9.11E-05
YDR534C	7.87E-06
YOR389W	1.81E-09
YML105C	0.000267
YKR052C	6.74E-06
YEL065W	3.06E-06
YHL035C	0.000109
YMR319C	1.07E-12
YPL278C	2.99E-12
YMR058W	1.06E-06
YHL040C	1.10E-05
YFL041W	0.000111
YOR381W	9.32E-05
YOL158C	5.41E-06
YPL277C	2.38E-14
YBR207W	0.000145
YLR034C	0.000197

YPL277C	1.23E-12
YJR005C-A	7.33E-05
YML038C	9.01E-05
YMR315W-A	4.43E-09
YGL146C	7.91E-06
YPL273W	1.75E-14
YOR382W	1.90E-06
YMR251W	5.52E-05
YMR312W	3.21E-13
YGL181W	4.26E-05
YER145C	1.28E-05
YFR024C-A	0.000174
YLR136C	8.14E-05
YJL149W	0.000197
YPL274W	8.03E-11
YMR316W	5.60E-11
YMR321C	1.00E-14
YMR310C	1.68E-08

Hot Spot 14:410000

Accession ID	p-value
YDL029W	7.58E-12
YMR076C	7.75E-11
YOR124C	9.40E-15
YCR088W	3.24E-11
YGR178C	1.47E-13
YJL133W	3.04E-13
YDR145W	4.52E-09
YCR011C	4.22E-10
YKL190W	5.76E-10
YLR454W	5.19E-10
YLL040C	7.07E-10
YFR016C	1.37E-09
YGR141W	1.48E-10
YCL034W	3.82E-11
YGR218W	5.22E-13
YDR506C	4.81E-08
YEL030W	6.62E-13
YMR170C	1.01E-10
YCL063W	6.66E-09
YKL133C	5.91E-11
YER105C	2.08E-11
YML070W	5.22E-13
YEL020C	9.51E-12
YIR036C	8.49E-12
YFR009W	3.82E-12
YPL105C	1.00E-12
YML080W	1.26E-11

YCL057W	9.51E-12
YNR052C	2.03E-10
YOL003C	7.17E-12
YOR086C	9.28E-07
YPL184C	6.96E-11
YLR355C	2.93E-10
YPL140C	1.41E-11
YOR122C	3.28E-05
YGL141W	8.18E-11
YNL125C	3.01E-05
YGL173C	1.30E-13
YDL160C	1.43E-12
YNL124W	3.26E-07
YDR153C	2.86E-13
YDR368W	7.75E-11
YKL207W	7.45E-10
YMR099C	2.93E-10
YMR027W	1.26E-11
YHR074W	6.24E-11
YPR088C	3.57E-05
YNL117W	1.13E-06
YOR367W	1.33E-10
YLR088W	2.80E-05
YIL137C	8.39E-13
YKL157W	1.70E-12
YJR036C	1.22E-05
YNL262W	2.55E-12
YNL102W	2.87E-12
YLR216C	0.000102
YNR015W	8.47E-06
YBL091C	1.13E-11
YNL004W	4.55E-14
YDL126C	1.82E-10
YDL190C	2.86E-13
YPL249C	1.01E-10
YNL085W	6.06E-10
YJL062W	3.90E-09
YEL015W	2.93E-10
YPR137W	1.49E-11
YFR024C-A	5.77E-08
YOR360C	1.91E-12
YJR103W	4.63E-13
YNL123W	9.97E-12
YGL137W	2.14E-12
YNL168C	2.25E-10

Hot Spot 15:150000

Accession ID	p-value

YIL157C	2.61E-06
YPL223C	1.74E-06
YKL138C	6.87E-06
YDL048C	1.73E-06
YKL195W	6.41E-06
YPL020C	1.17E-05
YMR317W	8.20E-05
YIL125W	4.33E-05
YGR130C	3.44E-06
YDL049C	1.88E-05
YDR447C	5.65E-06
YKL096W	2.75E-11
YEL003W	5.76E-06
YHR198C	5.46E-06
YAL034C	0.000112
YOR187W	1.50E-07
YGL107C	1.74E-05
YKL192C	6.66E-05
YKL167C	2.13E-08
YER053C	1.19E-06
YOL105C	4.30E-13
YDR210W	1.16E-05
YOL077W-A	3.59E-08
YPL215W	2.62E-05
YMR169C	7.24E-09
YOL071W	3.17E-05
YKL194C	3.65E-06
YPL127C	8.28E-07
YKL085W	9.68E-07
YML091C	6.19E-06
YPR155C	2.06E-05
YNL055C	4.14E-06
YGR088W	2.21E-09
YMR135C	9.14E-10
YPR184W	1.16E-05
YPL078C	6.04E-07
YKR086W	0.000114
YOL090W	1.08E-06
YHR024C	6.09E-06
YJR121W	9.47E-09
YNR020C	3.77E-06
YHR087W	7.29E-07
YLL028W	1.16E-15
YPR055W	1.56E-05
YOR302W	7.34E-05
YCR073C	1.88E-06
YOL109W	1.50E-06

YJL166W	2.83E-05
YER013W	2.82E-07
YGL143C	5.54E-06
YMR280C	6.44E-06
YGR272C	1.29E-05
YJL158C	8.40E-07
YFR045W	2.22E-08
YDR345C	7.48E-05
YHR011W	0.000139
YMR318C	1.75E-06
YFL030W	4.19E-05
YKL015W	9.36E-05
YOR319W	5.59E-05
YML065W	3.40E-05
YDR325W	9.00E-06
YPL045W	1.26E-06
YMR043W	0.000242
YDR175C	5.63E-06
YDR375C	4.23E-05
YPR179C	1.25E-05
YJL063C	7.02E-07
YNL073W	1.80E-07
YER093C-A	2.10E-05
YGL068W	5.00E-06
YER170W	1.87E-06
YHR051W	2.50E-07
YDR337W	5.35E-08
YML054C	6.60E-06
YER168C	2.13E-06
YDR347W	1.52E-07
YMR037C	4.12E-06
YOR353C	2.04E-07
YFR039C	5.46E-06
YLL015W	7.95E-05
YOR080W	1.44E-05
YBR131W	1.70E-05
YIL115C	3.79E-05
YMR225C	1.24E-07
YLR239C	2.93E-05
YPL132W	2.50E-07
YER088C	1.68E-09
YDR217C	3.04E-05
YOL104C	5.29E-16
YPR191W	6.47E-07
YOL133W	3.58E-13
YAL053W	5.79E-05
YGR084C	5.86E-06

YNR037C	5.96E-06
YNL045W	5.54E-07
YML025C	1.01E-05
YPR020W	1.24E-05
YNL252C	4.34E-06
YAL029C	1.34E-07
YKL072W	7.06E-07
YLR204W	2.05E-07
YMR196W	6.19E-06
YGR031W	3.02E-07
YIL070C	1.02E-05
YKR016W	7.14E-06
YEL011W	3.62E-06
YML030W	1.80E-07
YCR102C	7.96E-06
YKL148C	3.25E-06
YDR178W	1.04E-05
YMR244C-A	9.07E-06
YHR207C	3.70E-06
YGR175C	8.29E-07
YOL006C	3.14E-05
YDL181W	1.19E-08
YDR096W	5.37E-06
YKL062W	8.32E-12
YML108W	7.20E-06
YEL050C	1.44E-07
YDR079C-A	4.12E-05
YLR253W	4.38E-05
YDR405W	1.17E-06
YNL005C	4.83E-06
YFL018C	6.37E-05
YKL193C	2.69E-05
YNL306W	2.21E-06
YBL034C	0.000162
YKR063C	7.14E-06
YBL013W	7.42E-06
YOR034C	2.35E-05
YEL052W	2.16E-06
YGR052W	1.64E-09
YJR113C	9.56E-05
YBR003W	1.83E-05
YDL117W	1.45E-06
YLR069C	2.95E-06
YLR295C	1.41E-08
YPL173W	2.91E-06
YDL170W	2.63E-06
YHR077C	7.21E-08

YPR047W	4.86E-05
YHR001W-A	2.25E-06
YPL118W	2.12E-06
YBR037C	2.60E-05
YFR044C	2.27E-06
YOL027C	4.36E-05
YFR011C	2.97E-07
YLR382C	5.55E-07
YER182W	3.22E-05
YML129C	2.91E-06
YPL183W-A	1.32E-06
YMR185W	3.79E-05
YBR004C	2.20E-05
YBR230C	6.54E-06
YOL081W	1.05E-09
YNL098C	6.41E-06
YGR112W	2.08E-06
YKL096W-A	1.51E-10
YJL141C	1.06E-06
YGR082W	4.83E-06
YDL004W	2.38E-07
YBR146W	3.70E-07
YKR065C	1.76E-05
YGR086C	1.33E-09
YJL103C	7.36E-07
YIR016W	2.64E-05
YOL084W	3.29E-09
YNR036C	8.71E-06
YGR076C	1.29E-07
YOR158W	4.78E-06
YOR303W	4.66E-05
YLL019C	3.13E-06
YDR511W	1.72E-06
YLR395C	1.01E-06
YGR271W	5.59E-06
YMR031C	5.92E-11
YGL129C	8.36E-06
YOL092W	6.20E-14
YDR148C	1.80E-06
YMR074C	7.00E-05
YPL003W	3.56E-05
YOL045W	9.49E-06
YPL137C	1.04E-06
YCL057C-A	6.03E-08
YNL185C	1.48E-06
YJL102W	1.33E-05
YDR326C	7.46E-05

YOL052C-A	1.24E-06
YGL187C	8.28E-07
YLR177W	6.83E-05
YNL052W	1.08E-06
YJL066C	1.43E-05
YMR250W	1.52E-05
YDL243C	0.000178
YGR062C	1.06E-05
YLL026W	4.72E-06
YFR007W	2.72E-06
YOR354C	1.46E-07
YNL100W	1.87E-06
YIL093C	5.63E-06
YER045C	5.98E-05
YDR533C	2.56E-15
YLR168C	1.18E-06
YBR104W	1.28E-05
YML100W	2.75E-06
YNL284C	1.34E-06
YCR071C	7.36E-07
YOR107W	2.58E-07
YDR166C	2.53E-05
YHR092C	3.65E-05
YML128C	6.40E-07
YLL018C-A	3.33E-05
YJR003C	2.69E-06
YPR097W	1.44E-05
YOR173W	8.46E-05
YOR207C	4.89E-05
YNL081C	7.78E-08
YOR304W	1.49E-05
YDR351W	2.32E-05
YKL170W	5.25E-05
YBL045C	3.15E-06
YDR462W	4.18E-06
YLR203C	5.19E-06
YDL202W	3.62E-06
YFR013W	1.44E-05
YBR122C	4.23E-06
YKL109W	8.30E-09
YLR301W	5.86E-07
YDR047W	4.33E-05
YHR007C	2.84E-05
YCR004C	3.22E-05
YDR342C	1.84E-08
YEL024W	1.44E-05
YJR092W	8.90E-05

YGR220C	3.09E-07
YHR009C	7.00E-05
YLL001W	0.000183
YDL022W	2.93E-05
YIL124W	2.91E-06
YML120C	7.37E-07
YDL183C	8.33E-05
YDL019C	1.09E-07
YDR494W	2.53E-05
YPL172C	8.39E-07
YOL119C	1.84E-06
YJL210W	3.02E-06
YMR272C	4.41E-12
YPL098C	0.000106
YMR090W	0.000127
YIL077C	1.37E-06
YMR002W	1.12E-05
YMR136W	3.04E-11
YDR116C	7.06E-07
YIL136W	6.73E-09
YPL271W	7.96E-07
YBR039W	4.50E-06
YMR013W-A	0.000135
YFR053C	9.42E-06
YIL056W	2.79E-07
YOR358W	3.65E-06
YNR009W	2.24E-05
YJR077C	1.24E-08
YGL236C	3.04E-05
YDR216W	2.91E-07
YML081C-A	2.50E-07
YFL016C	9.47E-05
YOR348C	5.39E-06
YPL247C	2.16E-07
YKR006C	7.12E-08
YBL029C-A	0.000284
YBL099W	1.28E-06
YOL113W	9.92E-10
YMR267W	7.11E-05
YCL014W	4.71E-05
YBR120C	1.33E-05
YDR322W	6.50E-07
YOL095C	5.47E-11
YML100W-A	2.87E-05
YPL104W	2.64E-07
YHR080C	1.19E-06
YOL137W	7.94E-08

YNL131W	8.21E-07
YOR286W	4.18E-07
YOL056W	4.67E-07
YGL037C	7.92E-06
YPL236C	3.93E-05
YER001W	1.36E-08
YLL041C	1.02E-06
YLR375W	2.19E-05
YKL155C	3.25E-06
YHR179W	2.96E-08
YNL274C	0.000193
YKL052C	5.21E-14
YJR080C	1.80E-06
YNL315C	2.81E-06
YOL089C	4.94E-11
YNL134C	5.77E-13
YJR019C	8.73E-05
YOL147C	1.65E-07
YDR529C	3.58E-05
YKL141W	1.19E-06
YNL137C	6.77E-07
YKR093W	2.70E-09
YLR219W	5.93E-07
YOL091W	1.15E-16
YIL155C	1.64E-07
YGR243W	3.65E-06
YLL021W	3.59E-05
YMR295C	1.06E-07
YDR180W	7.23E-05
YPL265W	2.94E-05
YKL087C	3.83E-07
YMR072W	2.31E-05
YOL073C	3.66E-08
YJL062W-A	7.66E-07
YHR210C	2.42E-06
YBL030C	3.99E-08
YPL014W	5.98E-06
YJR127C	2.54E-07
YCR046C	3.09E-07
YNR059W	7.96E-06
YHR151C	3.59E-05
YNL300W	6.19E-06
YGR105W	3.72E-05
YLR163C	2.08E-06
YKR064W	6.25E-05
YGR021W	1.63E-07
YMR157C	3.66E-06

YNL133C	4.74E-06
YHR022C	1.24E-05
YDR296W	1.00E-07
YOL114C	3.08E-06
YOR161C	2.48E-06
YMR286W	3.60E-07
YAR035W	6.60E-07
YGR244C	2.12E-08
YJR034W	2.60E-07
YNR040W	3.79E-08
YIL114C	5.06E-05
YHL004W	2.34E-07
YKL016C	1.30E-07
YMR145C	1.28E-05
YKL003C	3.03E-06
YGL145W	3.58E-05
YKL150W	6.34E-06
YIL098C	0.000166
YGR194C	5.08E-06
YLR390W	2.83E-05
YOR273C	1.01E-07
YNR002C	3.45E-07
YGR035C	1.25E-05
YHR059W	1.94E-06
YKL051W	9.83E-06
YML009C	7.55E-06
YOR185C	9.36E-05
YOL094C	4.87E-12
YJL087C	0.000101
YHR116W	1.01E-06
YOL097W-A	3.49E-06
YJL011C	1.97E-06
YMR105C	5.57E-06
YJR101W	3.03E-05
YBR185C	5.46E-06
YER062C	6.56E-08
YHR086W	1.95E-06
YGR023W	4.40E-06
YJR011C	7.80E-05
YLR258W	2.59E-05
YGL237C	2.10E-05
YBR177C	5.32E-11
YBR126C	3.61E-05
YNR001C	8.25E-06
YKL079W	1.83E-05
YNR014W	8.94E-10
YGL151W	4.27E-07

YLR178C	2.64E-07
YGR207C	5.46E-06
YDR070C	4.93E-05
YJR090C	5.39E-06
YFL014W	4.81E-11
YPR160W	4.07E-07
YKL057C	0.000119
YPL004C	4.50E-07
YOL096C	2.08E-06
YDR298C	2.09E-08
YPR149W	5.15E-08
YEL071W	7.20E-06
YOL087C	1.00E-06
YGR165W	1.10E-05
YMR195W	6.06E-07
YFR006W	1.59E-08
YPL161C	6.47E-05
YJL180C	4.80E-06
YPL113C	6.18E-05

Hot Spot 16:510000

Accession ID	p-value
YJL147C	2.99E-05
YPL020C	1.99E-12
YPL050C	2.24E-11
YER053C	4.25E-05
YOL077W-A	4.11E-05
YPL002C	9.54E-17
YKL085W	6.64E-05
YML091C	2.28E-05
YMR135C	5.49E-05
YPL078C	6.41E-06
YML125C	8.53E-05
YJR121W	8.20E-06
YJL166W	3.85E-05
YPR015C	0.00012
YPL034W	5.37E-06
YPL017C	2.03E-07
YGL153W	6.64E-06
YFR033C	9.75E-06
YOR228C	5.98E-06
YPR006C	9.78E-16
YMR274C	1.93E-05
YHR051W	3.28E-05
YBR023C	5.18E-06
YPL026C	1.86E-06
YDR217C	0.000103
YPR191W	1.47E-05

YPR020W	5.56E-07
YEL011W	1.45E-05
YLR371W	7.72E-05
YKL018C-A	8.27E-05
YLR319C	9.07E-05
YMR251W-A	2.13E-05
YJR059W	3.17E-05
YLR108C	6.00E-05
YDL181W	2.52E-06
YKL062W	5.15E-05
YNL087W	5.67E-05
YLR312C	5.32E-05
YPL016W	3.32E-13
YIL030C	4.53E-05
YLR295C	1.50E-07
YOR278W	2.97E-05
YBR259W	5.15E-05
YHR001W-A	2.52E-05
YBL015W	0.000177
YNL142W	1.12E-05
YPL024W	3.08E-08
YOR214C	7.38E-06
YDL004W	2.17E-06
YGR086C	7.64E-06
YLL019C	0.000125
YLR395C	1.58E-05
YPR004C	1.87E-06
YMR266W	1.01E-05
YPL021W	0.000132
YCL057C-A	2.06E-05
YDR326C	0.000197
YGL187C	3.62E-06
YNL052W	2.60E-05
YPL031C	1.97E-09
YNL100W	9.36E-05
YPL011C	4.90E-05
YPL013C	2.04E-05
YBL045C	3.61E-05
YPR009W	1.20E-06
YKL109W	3.37E-06
YJR092W	0.000135
YJL210W	3.37E-06
YHR142W	3.07E-05
YPL022W	1.13E-11
YIL077C	2.36E-05
YPL271W	3.34E-07
YBR039W	7.64E-06

YJR077C	1.74E-05
YBL099W	1.42E-05
YGL021W	8.59E-07
YHR090C	6.56E-06
YKL093W	4.21E-06
YPL038W-A	1.56E-11
YPR063C	9.45E-06
YOR065W	3.73E-05
YPL046C	9.18E-08
YLR228C	0.000162
YBR170C	6.88E-06
YJR019C	7.53E-05
YDR529C	7.77E-05
YKL141W	3.17E-05
YIL155C	0.000116
YER067W	8.53E-05
YDR277C	3.50E-05
YGR138C	2.57E-07
YKL016C	1.03E-06
YNR002C	1.47E-05
YPL038W	3.88E-08
YAL024C	3.85E-05
YJR047C	1.50E-05
YPL039W	6.64E-14
YJL058C	8.83E-05
YPL004C	1.86E-05
YDR298C	3.48E-07
YPR149W	7.92E-06
YEL071W	3.61E-05
YFR006W	5.67E-06
YDR379W	1.09E-07
YPL113C	6.04E-05

Supplementary Table 7: eQTL hot spots for the MPTGA method

Hot Spot 2:550000

Accession ID	p-value
YER059W	6.64E-07
YKL012W	5.81E-08
YJL147C	1.94E-09
YKL138C	4.00E-14
YDL159W	4.67E-08
YNL216W	5.02E-13
YFL034C-B	1.31E-12
YIR021W	1.31E-08
YEL006W	5.03E-10
YKL195W	1.01E-08
YBR149W	1.04E-11
YLR286C	6.52E-101
YMR182C	7.90E-08
YPL264C	1.20E-08
YKL166C	1.17E-09
YBR194W	2.18E-07
YOR187W	1.40E-13
YBR285W	1.20E-11
YPR106W	6.79E-49
YDR072C	7.51E-11
YBR157C	2.21E-16
YPL101W	7.77E-09
YOR264W	5.96E-36
YDL061C	6.39E-14
YDR210W	3.75E-09
YDR006C	3.50E-07
YGR174W-A	6.50E-08
YBR084C-A	1.03E-11
YAL003W	3.54E-17
YGL196W	7.08E-08
YHR053C	3.97E-18
YDL012C	3.55E-08
YKL194C	4.21E-07
YLR029C	2.77E-07
YLR299W	8.29E-11
YOR338W	1.06E-07
YIL052C	1.01E-09
YOR179C	1.77E-09
YNL055C	1.30E-07
YPL131W	2.17E-12
YJL200C	5.53E-17
YNR067C	8.31E-35
YBR186W	2.84E-12
YDL055C	1.03E-16

YML124C	6.06E-16
YMR306W	4.62E-14
YOL021C	1.20E-11
YIL009W	1.96E-08
YHL001W	2.00E-09
YDL218W	4.52E-09
YJR123W	4.57E-12
YGR027C	4.77E-07
YGR258C	5.75E-09
YDL229W	2.61E-12
YDL155W	8.84E-07
YOR045W	3.73E-09
YEL062W	2.89E-12
YMR088C	6.35E-13
YKL180W	1.44E-15
YNR020C	5.48E-08
YIR034C	2.60E-10
YLR185W	4.21E-10
YPL081W	1.22E-07
YHR038W	2.81E-13
YKL081W	2.06E-07
YNR017W	1.87E-12
YCR073C	3.31E-07
YOL112W	5.57E-11
YIL094C	5.57E-21
YBL072C	1.38E-11
YBL027W	3.33E-12
YER102W	3.94E-10
YGL189C	2.02E-14
YJL125C	7.98E-09
YDL057W	2.31E-12
YKR057W	1.77E-07
YGR085C	2.11E-13
YGR148C	3.56E-11
YBR291C	4.84E-47
YAR014C	2.27E-15
YGR214W	1.14E-15
YDR345C	6.70E-12
YLR026C	8.55E-08
YAL042W	6.07E-08
YBR197C	1.19E-53
YOR003W	1.54E-10
YER124C	2.97E-121
YIL009C-A	2.73E-07
YDR090C	1.53E-13
YBR135W	3.50E-12
YMR142C	3.21E-16

YDL229W	1.75E-12
YDR064W	1.10E-18
YER064C	4.45E-18
YBR077C	3.55E-12
YJL063C	8.34E-10
YPR163C	1.43E-07
YDR234W	4.06E-10
YNL073W	2.85E-10
YKL112W	8.66E-08
YIR030C	2.20E-15
YGL068W	3.15E-18
YGL259W	2.13E-15
YOL120C	4.72E-13
YBR162C	9.24E-07
YPR181C	2.04E-08
YLR360W	4.32E-13
YPR125W	2.18E-11
YPL107W	2.02E-14
YKL103C	7.24E-09
YLR249W	3.67E-09
YOL020W	1.67E-17
YPL066W	4.05E-09
YPR005C	2.65E-08
YER036C	7.61E-08
YDR347W	5.95E-08
YOR246C	4.58E-07
YBL049W	2.98E-12
YBR079C	2.44E-08
YGR208W	1.55E-08
YAL035W	2.21E-07
YGL189C	3.60E-14
YIL069C	5.87E-19
YPR043W	3.45E-11
YMR225C	1.71E-08
YER089C	5.60E-07
YPL132W	1.38E-08
YNL121C	4.15E-08
YIR031C	3.22E-34
YOL127W	1.73E-16
YOR061W	2.53E-08
YPR162C	8.82E-08
YDR237W	6.02E-09
YMR309C	1.46E-08
YFR002W	2.86E-07
YJR152W	2.46E-41
YDL182W	3.77E-17
YBR154C	1.32E-07

YMR180C	2.35E-09
YDL230W	2.40E-08
YGR084C	7.22E-10
YDL095W	2.72E-09
YNL270C	5.79E-08
YNL240C	1.12E-07
YBR175W	1.07E-10
YCL027W	3.59E-07
YNR037C	3.37E-10
YOL047C	3.01E-34
YBR165W	3.21E-17
YEL054C	3.93E-12
YMR242C	2.34E-11
YGL062W	7.32E-07
YMR116C	2.56E-12
YHR209W	2.24E-07
YMR170C	1.78E-07
YGL185C	1.11E-10
YNR050C	3.89E-44
YIL070C	3.32E-13
YLR150W	5.46E-12
YKL185W	4.07E-18
YJR045C	6.13E-16
YNL101W	9.24E-13
YLR368W	1.62E-07
YDR418W	1.48E-11
YHL042W	1.35E-07
YJR138W	2.55E-07
YHR030C	2.98E-13
YDR352W	6.13E-08
YKR031C	4.58E-10
YML030W	3.15E-08
YLR259C	1.22E-16
YMR251W-A	1.44E-14
YLR388W	1.25E-15
YBR168W	6.45E-08
YGL229C	1.78E-15
YML063W	1.15E-08
YLL039C	3.98E-08
YJR054W	7.40E-10
YEL058W	4.09E-08
YML118W	6.58E-08
YAL023C	1.31E-09
YBR228W	8.28E-08
YLR388W	4.58E-15
YPL221W	1.29E-07
YKR094C	7.34E-10

YPL128C	1.16E-07
YAL062W	7.08E-09
YKL164C	4.35E-13
YEL065W	4.01E-15
YLR344W	1.32E-11
YDR222W	4.30E-09
YHR089C	8.52E-08
YNL015W	7.45E-21
YIL140W	2.21E-08
YLR048W	2.73E-15
YOR056C	3.04E-13
YBR173C	1.17E-08
YJR008W	2.67E-10
YKL204W	2.67E-08
YBL072C	8.05E-09
YOL128C	6.04E-13
YHR199C-A	1.06E-09
YOR316C-A	2.25E-32
YPL168W	1.18E-09
YJL206C	2.49E-08
YNL178W	1.49E-16
YDR430C	2.57E-09
YCR099C	4.48E-08
YMR205C	1.37E-06
YMR236W	5.27E-08
YGR041W	1.19E-26
YER154W	2.21E-09
YOL019W	1.10E-08
YDL117W	3.59E-07
YGR281W	1.58E-07
YMR058W	2.20E-13
YIL018W	1.45E-14
YKR034W	3.80E-50
YER095W	2.31E-09
YOR126C	1.01E-07
YPL054W	1.22E-11
YLR069C	6.65E-14
YGL040C	8.81E-15
YMR122W-A	1.50E-08
YBR167C	1.12E-18
YGL164C	1.56E-09
YPR134W	6.53E-08
YGR034W	1.19E-12
YNL293W	2.00E-08
YEL063C	1.16E-30
YFL041W	1.08E-08
YBR037C	1.28E-08

YJL076W	6.98E-07
YER165W	7.61E-13
YNL142W	1.57E-42
YIL117C	8.76E-21
YLR382C	5.13E-11
YIL164C	6.78E-17
YML129C	9.89E-12
YEL064C	5.01E-15
YER063W	4.71E-13
YDR471W	4.69E-10
YDR514C	1.27E-09
YOR205C	1.11E-07
YDR508C	7.47E-24
YGR082W	5.34E-23
YNL283C	1.10E-12
YDL130W	1.83E-16
YBR146W	5.27E-17
YKR065C	1.26E-07
YEL060C	2.65E-09
YGR066C	1.96E-11
YLR446W	3.14E-08
YMR118C	9.14E-22
YNR036C	9.96E-09
YGR076C	1.42E-07
YDL061C	2.00E-13
YHL033C	7.39E-12
YNL066W	1.91E-44
YKL008C	1.01E-07
YBR132C	5.55E-52
YOR158W	8.94E-10
YDL024C	1.20E-10
YLR054C	1.49E-07
YOL131W	4.59E-12
YBR091C	8.11E-09
YDL083C	1.00E-12
YLR353W	9.73E-09
YJL095W	7.60E-07
YJL145W	1.10E-11
YGR019W	1.46E-07
YIR027C	1.99E-33
YGR055W	1.78E-28
YNL268W	6.58E-10
YDR098C	1.38E-09
YIL047C	2.46E-10
YGL129C	1.02E-08
YDL239C	1.61E-25
YJR001W	7.38E-08

YOL025W	2.49E-07
YMR012W	9.91E-08
YMR271C	1.64E-08
YLR448W	4.96E-15
YOL045W	1.07E-09
YNL125C	2.86E-12
YNL185C	4.39E-12
YNL327W	2.01E-30
YNL209W	3.96E-12
YDL142C	2.23E-08
YDL231C	4.18E-07
YIR032C	2.11E-35
YIR029W	1.96E-31
YMR305C	2.55E-12
YPL143W	5.42E-10
YDR131C	5.53E-11
YJL161W	2.24E-09
YOL039W	2.80E-11
YPL249C-A	5.31E-10
YER019W	3.01E-16
YHR117W	8.67E-09
YFR007W	4.83E-10
YHR202W	5.46E-10
YGR286C	1.85E-11
YMR194W	1.59E-15
YOR354C	2.30E-08
YLR023C	7.15E-07
YBR171W	1.28E-11
YBR103W	1.56E-23
YBR196C-B	2.79E-36
YHR012W	1.59E-07
YIL093C	2.56E-07
YBR104W	2.99E-21
YLR421C	2.38E-07
YDR025W	2.05E-15
YCR020C	2.99E-09
YBR263W	2.98E-07
YOR386W	2.76E-11
YLL009C	3.90E-09
YJL159W	8.24E-08
YJL172W	3.26E-07
YJL045W	6.02E-09
YLR102C	6.38E-07
YJL023C	2.03E-07
YCR071C	1.37E-09
YHL038C	2.01E-06
YPL183C	1.88E-07

YJL116C	1.25E-08
YDL150W	2.92E-08
YPR144C	4.76E-10
YEL023C	1.24E-08
YBR115C	3.79E-83
YOR127W	4.83E-07
YPL176C	3.39E-08
YGR125W	3.47E-28
YNL282W	1.41E-09
YCR031C	5.96E-10
YER131W	1.79E-14
YGR040W	1.90E-17
YJL191W	1.59E-08
YKL170W	5.87E-07
YER153C	1.59E-15
YIL089W	4.52E-09
YFL036W	1.17E-11
YHR183W	2.33E-10
YMR155W	1.68E-09
YBR179C	1.69E-08
YLR188W	6.87E-09
YDR462W	4.30E-09
YDL045W-A	1.90E-09
YDL202W	3.35E-08
YPL171C	6.31E-08
YDL169C	1.16E-08
YMR255W	2.40E-11
YLR209C	1.79E-08
YLR325C	1.49E-11
YPR075C	7.40E-07
YDR500C	1.12E-12
YPL079W	7.00E-17
YHR007C	1.19E-07
YDR260C	5.02E-11
YLR061W	1.28E-17
YKL050C	5.87E-16
YOR296W	3.64E-13
YBR107C	7.09E-67
YMR001C	1.67E-13
YNL183C	6.79E-16
YGR169C	4.15E-07
YJR092W	8.07E-18
YGR220C	1.34E-07
YLR059C	1.22E-06
YIL124W	1.37E-06
YLR142W	1.96E-10
YDR194C	5.08E-11

YDR494W	6.11E-07
YIL146C	3.63E-19
YNL117W	1.27E-15
YPR124W	3.51E-08
YNR068C	3.08E-12
YMR101C	7.87E-10
YDL122W	1.24E-07
YML031W	1.83E-08
YHR142W	9.71E-12
YER043C	2.99E-07
YIL088C	1.41E-08
YLR084C	2.07E-08
YPL098C	2.94E-14
YMR002W	3.15E-07
YNL239W	5.48E-10
YDR226W	1.64E-07
YLR333C	1.32E-07
YMR030W-A	4.08E-11
YBR166C	3.69E-92
YGL202W	1.87E-09
YBR189W	4.60E-20
YCR033W	2.65E-09
YPL163C	1.78E-15
YNL046W	3.71E-13
YLR097C	2.67E-08
YJR036C	3.22E-10
YKL218C	1.97E-11
YPR132W	2.15E-09
YDR077W	3.77E-15
YER151C	1.21E-07
YNL096C	1.99E-10
YIL158W	1.75E-09
YCL001W-B	5.17E-10
YKR006C	1.05E-09
YPR114W	8.70E-12
YLR289W	1.70E-08
YKL181W	2.64E-10
YKL006W	7.65E-17
YER074W	2.34E-16
YFR031C-A	1.20E-13
YBL022C	2.71E-08
YOR188W	6.94E-14
YMR181C	2.20E-09
YDR362C	6.64E-07
YNL323W	1.48E-08
YHL033C	1.30E-11
YGL209W	2.03E-07

YDL131W	1.00E-12
YCL014W	2.32E-10
YDL082W	7.67E-15
YDL154W	1.52E-11
YBR120C	1.60E-07
YMR011W	3.26E-10
YDR322W	1.34E-07
YPR133W-A	2.67E-17
YML106W	1.57E-10
YBL071C-B	3.94E-07
YJR005W	8.23E-08
YER075C	2.01E-12
YGL028C	1.57E-82
YGR154C	3.19E-08
YML068W	2.68E-09
YPL040C	4.03E-10
YFL021W	3.25E-33
YLR432W	9.05E-09
YER056C-A	9.52E-09
YLR419W	1.10E-08
YOR140W	2.34E-12
YKL107W	8.40E-11
YAL037W	3.53E-10
YPR119W	9.13E-08
YPL104W	3.67E-08
YPR007C	3.72E-07
YDL014W	6.58E-09
YOR286W	5.14E-10
YGL064C	4.59E-11
YNL302C	2.28E-13
YIR009W	2.93E-08
YOR034C-A	1.34E-06
YER001W	2.32E-08
YGR147C	1.30E-06
YJR133W	5.44E-09
YFR040W	3.09E-07
YML073C	1.23E-12
YDR294C	3.31E-07
YIL131C	3.75E-15
YJR080C	6.54E-10
YNL315C	2.66E-09
YGR121C	7.87E-09
YPR138C	8.33E-17
YBR170C	7.72E-08
YKR097W	4.71E-15
YIL071C	4.10E-08
YBR148W	3.61E-33

YBL087C	5.81E-12
YGR174C	1.03E-07
YGL258W-A	1.05E-17
YOL121C	3.13E-12
YOR230W	8.59E-14
YGL123W	7.26E-13
YGR136W	2.00E-07
YNL137C	6.01E-08
YJR148W	1.47E-10
YPR041W	5.21E-09
YBR156C	7.43E-18
YER118C	8.59E-12
YDL156W	4.90E-07
YNR069C	2.98E-13
YDR385W	1.15E-08
YEL072W	9.24E-10
YGL139W	7.70E-12
YMR174C	7.96E-08
YNL069C	1.93E-14
YIL031W	2.34E-08
YOR111W	1.16E-09
YBR256C	3.93E-09
YNL278W	7.72E-08
YGR067C	2.07E-07
YHR010W	2.16E-08
YLR285C-A	5.19E-20
YHR107C	4.58E-13
YLR138W	9.62E-08
YLR042C	2.48E-37
YMR129W	1.85E-07
YMR032W	1.12E-16
YKL087C	5.91E-11
YGR174W-A	1.41E-09
YMR173W	7.00E-15
YJL062W-A	8.66E-10
YDL238C	2.32E-20
YER074W	6.47E-12
YJR043C	1.47E-07
YER145C	1.41E-07
YCR086W	1.70E-09
YCR046C	5.71E-15
YPL213W	6.04E-13
YNL040W	1.74E-10
YKL179C	8.05E-07
YGR138C	2.72E-09
YDR425W	5.92E-10
YGL081W	2.26E-07

YPR105C	2.46E-09
YDR421W	1.64E-11
YOR096W	4.50E-09
YLR406C	8.14E-16
YOR293W	1.61E-07
YNL078W	3.62E-15
YOR380W	3.92E-09
YHL016C	7.85E-11
YGL076C	3.41E-16
YCL025C	8.87E-07
YBR117C	2.02E-12
YDR034W-B	1.02E-07
YHR022C	2.18E-10
YLR378C	1.25E-09
YBR038W	7.62E-07
YOR298W	1.54E-10
YEL004W	8.56E-07
YMR200W	3.54E-08
YOL007C	2.87E-08
YGL097W	6.44E-09
YPL061W	5.15E-13
YMR163C	3.58E-09
YJR034W	6.66E-08
YCL055W	1.08E-07
YLR405W	2.30E-07
YOR129C	1.26E-15
YBR191W	1.42E-14
YBR139W	1.24E-08
YPL089C	6.05E-08
YPR074C	2.63E-10
YDL179W	4.49E-09
YJR147W	1.53E-16
YGR085C	1.31E-08
YPL158C	5.98E-11
YKL003C	6.48E-08
YIL098C	3.82E-08
YLR390W	3.18E-10
YDL210W	1.32E-28
YLR080W	6.32E-09
YBR111W-A	1.38E-08
YER152C	1.75E-43
YLL045C	3.09E-13
YER025W	3.75E-08
YOR315W	1.98E-19
YOR316C	1.78E-07
YHR059W	2.91E-08
YML009C	2.89E-10

YOL115W	4.15E-08
YOR330C	1.16E-07
YLR340W	1.21E-07
YNL301C	2.71E-15
YGR014W	1.23E-19
YDL078C	2.42E-09
YKR021W	2.14E-08
YBL085W	6.99E-07
YOL040C	1.07E-13
YGR020C	4.65E-09
YOL052C	4.01E-09
YJL160C	1.22E-09
YBR181C	1.16E-12
YJR096W	1.66E-08
YGL105W	6.65E-08
YMR013C	7.74E-07
YPL072W	3.34E-09
YNL176C	1.07E-07
YJR101W	1.22E-07
YBR185C	3.57E-10
YFR028C	1.89E-09
YLR079W	1.29E-13
YBR098W	3.22E-21
YFL001W	7.74E-08
YOR247W	5.93E-13
YOR232W	5.30E-18
YPR194C	6.34E-09
YIR028W	7.01E-29
YLR258W	7.19E-13
YAL041W	2.16E-09
YGR152C	3.30E-08
YML052W	3.80E-08
YBR193C	6.24E-12
YBR237W	2.81E-08
YHR203C	9.03E-14
YBR150C	1.37E-27
YMR024W	4.75E-09
YBL061C	6.31E-08
YKL068W-A	7.46E-08
YBR048W	5.94E-13
YGL135W	5.99E-08
YER060W	9.62E-10
YOR342C	1.93E-19
YER096W	7.24E-15
YNR053C	5.44E-09
YFL022C	7.60E-10
YJL054W	1.71E-08

YDR333C	1.60E-08
YNL165W	2.58E-08
YOR134W	5.96E-07
YAL024C	3.23E-12
YIL165C	6.31E-17
YBR126C	1.59E-07
YAL039C	1.23E-07
YJL078C	7.18E-41
YHL023C	9.94E-08
YNL037C	5.82E-08
YJL122W	1.41E-07
YJL177W	9.45E-15
YJR042W	7.21E-07
YJR094W-A	8.30E-09
YBR196C-A	4.45E-43
YDL075W	7.94E-09
YBR092C	1.20E-14
YJL110C	2.49E-18
YDR115W	1.73E-07
YPL063W	5.40E-08
YLR164W	4.38E-14
YHL001W	2.34E-14
YNL168C	3.30E-10
YPL123C	4.91E-08
YHR021C	8.11E-11
YPL071C	3.33E-08
YKR061W	1.84E-07
YBR176W	4.06E-15
YBR191W	1.04E-11
YNL299W	2.63E-08
YMR285C	1.61E-07
YKR013W	4.46E-16
YDR242W	4.99E-17
YPL085W	1.55E-08
YHR143W	7.74E-128
YKR091W	2.64E-25
YBL092W	1.95E-14
YLR053C	1.47E-20
YBR158W	1.05E-42
YIL149C	9.31E-12
YBR119W	3.99E-20
YKR005C	3.57E-12
YDR381W	4.98E-15
YHR099W	6.16E-10
YJL136C	2.82E-10
YOR116C	5.00E-09
YJL053W	2.51E-08

YDR379W	3.12E-08
YIL133C	3.55E-13
YFR031C-A	3.98E-17
YOR293W	2.18E-08
YPL082C	5.00E-13
YOL082W	2.81E-07
YGR044C	1.74E-31

Hot Spot 3:90000

Accession ID	p-value
YOR271C	4.88E-15
YCL018W	2.21E-63
YPL189W	1.25E-09
YIL121W	1.31E-06
YCL026C-A	3.01E-21
YER073W	1.61E-19
YBR085C-A	6.01E-08
YKR071C	1.58E-20
YPL084W	5.96E-07
YFR005C	7.64E-07
YDL215C	5.64E-07
YLR355C	5.04E-19
YJR016C	8.24E-21
YCL017C	1.19E-25
YLR348C	3.07E-24
YCR018C	1.99E-31
YBR068C	7.57E-12
YHR047C	6.87E-09
YIL050W	9.10E-14
YNL241C	2.31E-10
YKR095W-A	6.59E-08
YOR226C	3.08E-28
YKL029C	9.03E-09
YCL026C-B	4.57E-38
YJR148W	9.79E-13
YGL009C	8.75E-42
YKR093W	1.99E-10
YOR227W	5.63E-10
YGL125W	6.85E-09
YNL104C	1.28E-25
YKL120W	1.01E-87
YOR375C	4.09E-75
YHR208W	1.42E-42
YCL004W	1.76E-09
YCL021W-A	5.55E-95
YCL016C	5.44E-25
YMR108W	2.29E-15
YFR006W	5.13E-09

Hot Spot 4:90000

Accession ID	p-value
YDL216C	5.42E-27
YDR447C	1.96E-08
YDR447C	2.32E-07
YDL061C	4.26E-08
YDL191W	1.70E-10
YDR450W	9.17E-11
YOR338W	4.16E-08
YDL218W	1.91E-16
YJR123W	1.05E-07
YKR057W	4.77E-08
YGR214W	2.05E-07
YER124C	3.48E-09
YDL199C	2.98E-10
YMR142C	1.10E-07
YDR064W	2.15E-11
YGL259W	4.28E-08
YML026C	4.97E-08
YJL189W	3.74E-08
YML063W	2.79E-08
YDL189W	2.08E-13
YNL178W	1.02E-13
YGR034W	2.69E-08
YDL217C	5.99E-12
YDL130W	1.72E-07
YMR118C	4.43E-12
YMR215W	4.74E-08
YDL061C	5.60E-10
YDL205C	1.58E-46
YML024W	1.25E-08
YML026C	3.13E-09
YDL214C	1.12E-28
YDL207W	2.20E-16
YDL203C	7.89E-09
YCR031C	4.31E-09
YDL197C	7.98E-14
YLR325C	3.54E-07
YBR166C	2.02E-08
YJL190C	4.44E-08
YBR189W	4.04E-08
YPR132W	2.56E-12
YER074W	1.84E-07
YKL107W	3.27E-07
YBL087C	2.67E-07
YGL123W	4.66E-11
YHR010W	3.16E-09

YDL209C	3.58E-11
YOR293W	2.45E-07
YFR032C-A	1.65E-07
YER117W	6.71E-09
YBR191W	3.43E-08
YGR085C	5.17E-11
YDL210W	3.56E-08
YNL301C	1.83E-07
YBR181C	1.46E-07
YDL081C	7.43E-09
YMR143W	1.15E-07
YGL135W	1.04E-10
YDL204W	8.09E-07
YJR094W-A	1.26E-07
YHR021C	1.11E-08
YHR143W	4.04E-09
YFR031C-A	3.03E-08
YOR293W	4.96E-08

Hot Spot 5:190000

Accession ID	p-value
YDR447C	2.28E-07
YER035W	8.28E-08
YDR447C	3.92E-11
YDL061C	5.20E-10
YER053C	3.89E-09
YCL054W	3.05E-07
YDL191W	4.35E-10
YBR084C-A	2.68E-09
YDR450W	1.01E-08
YLR029C	2.52E-07
YIL052C	1.88E-10
YPL131W	1.24E-11
YBR084W	1.02E-06
YPR184W	4.90E-08
YHL001W	6.05E-08
YJR123W	7.21E-11
YDL229W	2.74E-07
YKL180W	2.61E-11
YLR185W	2.05E-11
YGL031C	2.36E-10
YBL072C	2.37E-11
YBL027W	9.93E-13
YER102W	1.19E-10
YGL189C	9.85E-11
YKR057W	5.75E-09
YGR085C	1.87E-12
YGR148C	3.43E-10

YGR214W	6.83E-12
YMR142C	6.39E-11
YLR075W	3.69E-08
YDR064W	3.15E-09
YOL120C	4.43E-11
YGL189C	3.59E-11
YIL069C	1.61E-13
YPR043W	3.46E-09
YOL127W	3.09E-14
YEL054C	2.37E-13
YML026C	1.85E-09
YMR242C	1.69E-10
YMR116C	9.43E-14
YDR418W	1.83E-10
YBR048W	7.03E-08
YJL189W	3.31E-07
YLR388W	1.65E-09
YML063W	7.22E-12
YLL039C	7.84E-08
YLR388W	2.70E-09
YKR094C	5.37E-07
YLR344W	2.04E-11
YLR048W	7.51E-14
YOR120W	4.08E-07
YBL072C	1.09E-11
YDR012W	1.00E-09
YOR312C	1.11E-08
YNL178W	2.89E-12
YER046W	5.48E-15
YIL018W	4.53E-11
YGR034W	3.15E-11
YEL026W	2.15E-11
YDR471W	5.33E-12
YDL130W	2.26E-12
YNL186W	5.12E-08
YDL061C	4.01E-10
YHL033C	1.16E-10
YDL083C	2.11E-10
YLR448W	4.33E-12
YML024W	2.07E-08
YNL209W	8.06E-09
YOR167C	1.09E-07
YOR310C	2.50E-07
YPL143W	6.78E-10
YER020W	3.30E-34
YMR250W	5.46E-08
YPL249C-A	2.15E-09

YGR248W	8.58E-11
YER019W	2.26E-07
YML026C	2.17E-10
YMR194W	6.58E-10
YDR025W	2.79E-11
YML056C	1.07E-07
YOR173W	4.49E-10
YER131W	6.33E-11
YJL191W	1.05E-17
YJL052W	9.48E-11
YLR325C	7.09E-09
YDR500C	3.27E-08
YPL079W	5.05E-11
YLR061W	3.53E-12
YHR141C	1.56E-09
YLR293C	5.73E-08
YLR197W	3.96E-08
YER047C	1.63E-16
YIL136W	3.32E-08
YLR333C	1.42E-10
YJL190C	7.30E-11
YBR189W	3.82E-11
YNL302C	5.74E-10
YLR264W	9.06E-11
YPR132W	8.02E-08
YNL096C	4.88E-11
YKL006W	7.39E-12
YLR372W	2.03E-07
YER074W	3.37E-13
YFR031C-A	6.92E-12
YHL033C	1.61E-12
YDL082W	5.66E-12
YLR432W	7.42E-09
YER056C-A	1.65E-08
YMR230W	5.21E-08
YGL147C	2.80E-08
YNL302C	6.21E-11
YNL255C	1.49E-08
YHR179W	1.43E-07
YML073C	1.68E-11
YBL087C	3.41E-10
YOL121C	8.88E-12
YGL123W	3.74E-09
YOR312C	1.25E-09
YER029C	2.76E-26
YNL069C	4.02E-11
YHR010W	3.25E-11

YER074W	9.83E-11
YOR096W	5.94E-12
YLR406C	3.53E-14
YOR293W	3.18E-11
YGL076C	1.88E-10
YFR032C-A	1.17E-08
YER117W	1.01E-09
YBR191W	7.26E-14
YOR063W	8.55E-08
YLR441C	7.27E-10
YBR031W	1.41E-09
YLL045C	4.05E-09
YLR340W	3.80E-08
YNL301C	6.27E-12
YOR375C	9.49E-14
YOL040C	1.74E-09
YBR181C	1.07E-10
YDL081C	8.57E-08
YGR152C	2.81E-07
YHR203C	2.19E-10
YMR143W	7.43E-09
YGL135W	8.49E-08
YER018C	4.17E-14
YLR178C	2.90E-08
YJL177W	1.22E-12
YJR094W-A	1.38E-09
YDL075W	2.54E-09
YHL001W	1.39E-11
YHR021C	4.73E-13
YBR191W	8.49E-10
YOR234C	2.56E-10
YPR160W	1.44E-08
YNR034W-A	2.67E-07
YPR149W	1.70E-07
YBL092W	2.06E-10
YJL136C	2.30E-10
YIL133C	2.34E-12
YFR031C-A	1.64E-12
YOR293W	2.41E-11
YGL030W	3.92E-09
YMR143W	1.75E-07

Hot Spot 7:410000

Accession ID	p-value
YAL003W	4.44E-16
YJL196C	7.39E-07
YGL048C	1.65E-11
YGL057C	8.65E-24

YPR174C	2.56E-16
YGL068W	8.97E-08
YGL055W	2.94E-20
YGL063W	1.85E-20
YGL066W	1.06E-10
YJL144W	1.54E-19
YJR045C	1.25E-07
YLR259C	2.10E-16
YEL065W	2.73E-08
YOR020C	3.64E-12
YOL002C	1.77E-10
YPL240C	1.92E-14
YGR082W	5.47E-09
YJR116W	1.15E-06
YNL077W	1.25E-29
YNL006W	7.58E-13
YLL024C	8.74E-32
YNL281W	1.17E-15
YGL049C	4.88E-14
YGL053W	1.40E-176
YDR151C	1.64E-07
YMR186W	1.06E-32
YNL064C	7.71E-18
YFL016C	2.52E-11
YBL022C	1.11E-08
YNL007C	1.41E-18
YLR216C	7.16E-15
YOR027W	5.59E-24
YGL045W	2.05E-24
YPR158W	3.12E-20
YJL034W	1.14E-11
YGL081W	2.18E-09
YGR142W	8.02E-26
YGL035C	9.88E-14
YDR492W	3.63E-09
YDR214W	2.44E-15
YOR232W	1.76E-08
YBR101C	1.48E-23
YGL051W	4.35E-114

Hot Spot 8:110000

Accession ID	p-value
YKL138C	7.38E-07
YHR014W	1.78E-09
YMR076C	1.67E-09
YHL010C	3.99E-20
YHR152W	1.88E-07
YFL047W	3.32E-09

YHR005C	2.57E-21
YAL003W	1.82E-07
YPL127C	4.01E-07
YLR212C	1.10E-06
YHR046C	1.88E-15
YKL135C	6.40E-09
YDL055C	9.56E-07
YML125C	1.12E-08
YGL048C	1.51E-07
YML102W	2.07E-08
YEL062W	6.14E-08
YNR003C	2.06E-10
YFR030W	8.29E-09
YNR017W	8.64E-07
YHR043C	1.84E-75
YHL018W	9.96E-07
YLR303W	1.06E-21
YCR023C	1.13E-06
YLL038C	1.57E-07
YHR040W	2.87E-08
YLR364W	4.40E-08
YOR076C	1.00E-07
YNL233W	1.57E-11
YIL009C-A	4.94E-07
YLR237W	1.14E-08
YHR036W	3.01E-10
YPL153C	7.63E-07
YLR363C	5.12E-07
YMR159C	1.36E-08
YGL068W	6.83E-09
YKL165C	3.65E-08
YLR103C	7.66E-08
YML105C	1.69E-08
YLR234W	3.55E-07
YMR179W	9.92E-08
YHL006C	4.26E-14
YCL027W	8.71E-31
YMR125W	2.26E-10
YNL309W	6.56E-08
YJR045C	7.35E-07
YBL016W	2.71E-27
YML060W	5.65E-08
YJR110W	5.54E-07
YBR040W	1.69E-20
YDR244W	2.94E-08
YLR259C	4.93E-09
YHR201C	1.78E-14

YPR122W	1.19E-13
YHL009C	2.07E-12
YER016W	3.35E-12
YAL031C	4.00E-11
YHR054C	3.30E-29
YDL064W	8.74E-09
YBR067C	3.60E-11
YGR068C	2.94E-07
YPL192C	6.50E-20
YIL140W	8.48E-14
YDL054C	2.35E-07
YGR146C	2.24E-07
YGR199W	3.46E-10
YOL017W	5.61E-10
YOR147W	2.50E-07
YHR199C-A	9.57E-09
YKR077W	6.05E-07
YJL187C	1.79E-15
YMR236W	6.99E-09
YLL002W	1.44E-07
YGR109W-A	5.35E-08
YGR227W	1.93E-07
YDR437W	1.05E-08
YHL022C	1.12E-22
YMR144W	1.24E-09
YJR004C	9.51E-14
YHL012W	6.10E-27
YPR103W	8.42E-07
YBR057C	9.68E-09
YDR032C	6.36E-08
YOR044W	4.81E-07
YOR376W-A	3.52E-08
YJR010W	6.39E-14
YGR082W	3.30E-13
YCL056C	1.32E-10
YHR034C	9.34E-09
YHR043C	1.11E-47
YER042W	1.73E-13
YDL127W	5.86E-09
YAL012W	1.04E-07
YJR021C	1.58E-07
YGR055W	4.70E-13
YPL124W	8.89E-10
YNR032W	1.34E-10
YMR307W	1.05E-07
YGL061C	4.07E-14
YJR072C	6.31E-07

YNL273W	1.98E-08
YDL142C	2.07E-07
YHL026C	2.93E-25
YKL001C	2.64E-12
YNL201C	1.39E-08
YBR088C	3.10E-08
YIL037C	7.53E-11
YDR121W	3.62E-07
YDL120W	2.55E-07
YER091C	8.50E-10
YIL001W	3.25E-08
YLR183C	1.54E-14
YDL150W	1.15E-06
YJL146W	1.98E-07
YBR115C	1.74E-08
YMR288W	7.92E-13
YNL072W	1.02E-10
YCR089W	1.38E-40
YHL019C	1.07E-06
YDR507C	1.58E-09
YML092C	1.40E-07
YHR127W	2.53E-07
YNL279W	9.79E-30
YBL052C	1.75E-11
YHR015W	2.14E-34
YGL116W	1.92E-07
YNL326C	7.00E-09
YGL223C	7.59E-09
YPR115W	6.02E-11
YHL008C	5.48E-93
YHR033W	1.14E-54
YMR198W	3.62E-07
YHR007C	7.56E-11
YMR065W	3.63E-36
YDL003W	6.53E-13
YNL230C	2.51E-08
YHR009C	1.94E-07
YLR413W	6.66E-07
YLL022C	8.48E-09
YCL010C	2.64E-09
YPL267W	5.83E-10
YGR006W	1.32E-07
YHL009W-A	1.07E-15
YPL098C	6.73E-09
YMR232W	4.90E-33
YNL064C	6.41E-09
YOL012C	7.65E-09

YHR042W	4.41E-08
YPL163C	2.81E-09
YHR021W-A	1.85E-17
YHR061C	1.40E-11
YBR070C	5.03E-15
YER039C	7.08E-07
YPR037C	2.53E-07
YPL055C	5.03E-07
YDR399W	7.63E-07
YKR030W	3.62E-07
YDR124W	1.77E-14
YLR372W	1.34E-06
YCL014W	2.89E-08
YKL189W	3.24E-22
YNL102W	1.07E-10
YPR133W-A	2.34E-08
YDL164C	1.85E-06
YDR350C	1.63E-07
YML068W	6.27E-08
YER112W	2.26E-07
YPR018W	1.55E-06
YHR027C	4.92E-18
YAL037W	2.34E-07
YLL062C	2.15E-15
YDR177W	1.63E-07
YHR084W	2.90E-28
YOR286W	2.95E-07
YHR105W	3.94E-07
YKL101W	1.80E-12
YHR052W	1.18E-09
YOR212W	1.17E-21
YGL060W	7.46E-26
YPR120C	1.19E-07
YER155C	3.30E-13
YGR109C	2.48E-10
YHR028C	6.19E-33
YFR052W	1.28E-08
YCR009C	5.67E-09
YDR410C	2.39E-11
YBR156C	5.37E-14
YML047C	9.64E-46
YGL184C	2.22E-15
YHR029C	3.61E-08
YNL278W	1.61E-08
YHR107C	1.41E-11
YNL328C	1.11E-08
YBL002W	7.50E-08

YGL248W	3.82E-10
YJL170C	2.11E-07
YCR046C	1.33E-07
YPL191C	6.91E-07
YGL106W	7.55E-12
YNL300W	3.38E-20
YJL157C	4.33E-27
YKL113C	5.13E-09
YNL078W	7.39E-07
YHR016C	1.39E-11
YHL016C	5.73E-56
YHR031C	1.94E-10
YHR022C	1.04E-07
YPL166W	5.66E-07
YHL013C	5.56E-07
YOR064C	3.69E-07
YOR180C	1.36E-06
YLR452C	1.37E-28
YBR264C	3.56E-09
YOL007C	5.09E-14
YKL054C	3.36E-07
YNL289W	6.85E-10
YGR047C	3.71E-07
YML062C	1.50E-07
YCL055W	4.28E-56
YPL156C	1.46E-13
YLR180W	7.29E-09
YNL258C	4.68E-10
YDR085C	2.31E-07
YNL290W	9.99E-09
YBR139W	6.25E-09
YKR024C	7.05E-10
YOR219C	3.32E-09
YDR420W	9.96E-07
YNR044W	7.48E-27
YLR390W	8.91E-07
YIL021W	3.15E-07
YNL042W-B	1.13E-08
YGL210W	5.33E-07
YMR314W	1.34E-10
YER070W	8.74E-12
YBR083W	1.08E-18
YJR086W	1.94E-08
YOR232W	6.41E-12
YGR152C	1.67E-07
YHL003C	1.51E-16
YLR315W	5.96E-07

YBR071W	1.10E-08
YHR035W	2.76E-25
YPL120W	2.51E-07
YNL159C	3.18E-08
YER039C-A	5.25E-09
YLR350W	2.50E-07
YJL074C	2.77E-09
YHL009W-B	2.35E-15
YEL042W	6.45E-07
YML046W	5.25E-32
YHR032W	7.00E-37
YNL158W	3.13E-12
YMR111C	4.45E-08
YDR225W	9.27E-08
YNL312W	1.03E-06

Hot Spot 9:70000

Accession ID	p-value
YFL045C	3.15E-09
YDL191W	2.67E-08
YDR450W	4.18E-08
YPL131W	2.60E-10
YJR123W	1.80E-07
YIL151C	1.76E-08
YKL180W	8.01E-07
YGL031C	5.98E-08
YBL072C	3.10E-10
YER102W	5.21E-08
YGR148C	3.33E-08
YGR214W	3.75E-07
YMR142C	6.03E-08
YDL229W	4.10E-09
YDR064W	2.66E-13
YIL134W	2.34E-10
YOL120C	2.86E-07
YLR249W	4.96E-08
YOL127W	2.85E-10
YMR242C	3.81E-07
YMR116C	5.53E-10
YML063W	5.32E-10
YNL178W	7.71E-12
YIL018W	2.22E-08
YGR034W	1.32E-09
YIL164C	3.22E-24
YFR055W	1.42E-07
YML024W	2.65E-08
YOL039W	2.57E-07
YIL142W	1.00E-08

YDR382W	8.79E-07
YIL153W	1.38E-06
YIL146C	4.86E-08
YIL136W	4.72E-07
YJL190C	1.50E-07
YBR189W	8.63E-08
YPR132W	2.59E-07
YKL006W	1.25E-10
YER074W	6.50E-07
YMR011W	9.06E-07
YGL147C	2.19E-10
YIL148W	4.05E-10
YGL103W	1.47E-08
YBL087C	6.33E-09
YGL123W	4.25E-09
YIL152W	2.06E-26
YIL130W	1.38E-10
YNL069C	2.78E-07
YBR072W	5.19E-10
YER074W	3.17E-08
YOR096W	1.25E-07
YGL076C	5.10E-08
YLL045C	2.34E-07
YOL040C	3.36E-08
YDR365C	4.86E-10
YBR181C	3.27E-08
YDL081C	2.64E-08
YHR203C	4.88E-09
YGL135W	6.96E-09
YIL165C	1.82E-26
YHR021C	8.81E-08
YBR191W	5.12E-08
YFR031C-A	1.03E-09
YLR167W	1.89E-08
YGL030W	7.54E-08

Hot Spot 12:670000

Accession ID	p-value
YNR074C	7.48E-11
YOR175C	3.49E-11
YLR266C	2.51E-10
YML019W	1.82E-07
YDL086W	4.36E-29
YMR220W	2.07E-22
YLR283W	9.22E-30
YDR518W	1.25E-08
YDL085W	9.45E-09
YDL236W	1.58E-09

YLR300W	1.42E-66
YEL051W	1.04E-06
YMR322C	7.28E-12
YLR256W	3.81E-34
YPR178W	9.60E-09
YOR321W	1.12E-08
YLR233C	1.24E-30
YJL100W	6.54E-14
YGL001C	1.19E-23
YLR265C	3.03E-46
YNR043W	1.47E-25
YLR260W	3.33E-16
YOR003W	4.53E-08
YPL272C	5.19E-17
YPR193C	4.17E-23
YLR237W	2.73E-14
YGR266W	3.28E-11
YER093C	8.05E-09
YFR033C	3.31E-13
YLR275W	1.04E-12
YML008C	2.08E-12
YPL189C-A	3.57E-08
YIL121W	5.74E-40
YML054C	3.54E-16
YPL107W	1.03E-15
YHR072W	8.19E-27
YMR202W	7.13E-26
YML075C	1.70E-46
YLR231C	7.88E-20
YDR502C	3.16E-23
YER136W	8.55E-08
YLR234W	3.79E-10
YNL111C	1.92E-27
YGL055W	1.12E-07
YOR099W	3.66E-08
YDR213W	1.29E-07
YGR089W	7.48E-07
YDR297W	2.43E-12
YDR453C	2.42E-08
YPR151C	5.73E-87
YML032C	5.75E-07
YDR186C	2.35E-07
YLR244C	1.26E-24
YEL047C	1.44E-07
YLR270W	5.51E-11
YDR346C	3.07E-11
YMR208W	1.43E-25

YEL034W	4.25E-44
YGL160W	2.09E-26
YLR246W	7.83E-35
YJL048C	3.61E-26
YDL093W	1.28E-12
YGL101W	4.99E-36
YJL080C	6.27E-07
YBR067C	1.58E-11
YHR188C	7.02E-08
YLR245C	1.41E-23
YPL170W	8.69E-10
YAL028W	2.32E-13
YOL128C	1.26E-10
YHR003C	1.64E-07
YMR205C	6.19E-08
YCR048W	5.24E-07
YGR281W	3.94E-08
YBR183W	2.68E-10
YLR205C	4.12E-76
YER014W	2.55E-15
YJR048W	4.48E-26
YKL008C	3.32E-08
YLR273C	3.93E-12
YDR275W	3.82E-09
YCR061W	2.67E-09
YER122C	1.00E-09
YNR041C	1.61E-17
YMR110C	1.62E-08
YNR019W	2.03E-31
YIL154C	1.04E-07
YKR046C	4.42E-88
YER129W	9.83E-08
YJR104C	1.86E-08
YDR166C	1.13E-07
YNL264C	6.69E-07
YMR113W	2.01E-07
YLR153C	1.07E-26
YEL035C	4.94E-09
YMR038C	1.91E-08
YGL077C	2.87E-09
YOR334W	5.47E-11
YPL028W	3.05E-15
YOL135C	6.28E-08
YOR356W	4.38E-08
YEL024W	2.39E-24
YPR088C	1.14E-07
YNL003C	2.07E-10

YLR288C	3.06E-15
YNL010W	3.79E-09
YMR070W	3.34E-10
YHR190W	7.78E-22
YML126C	8.17E-33
YGL191W	7.24E-09
YDR284C	1.12E-12
YOR348C	4.02E-12
YLR100W	3.26E-14
YGR131W	3.08E-09
YDR011W	2.91E-07
YMR100W	8.21E-10
YOR065W	1.33E-24
YHR179W	1.15E-13
YNL173C	3.88E-07
YBR085W	3.23E-09
YDR529C	1.48E-08
YGL009C	7.13E-10
YKL182W	6.55E-09
YLR154C	7.86E-14
YOR085W	1.02E-09
YPR095C	3.64E-10
YIL007C	1.19E-11
YDR218C	3.69E-12
YHR039C	3.89E-17
YGR138C	2.07E-07
YER141W	9.79E-09
YGR234W	6.50E-21
YNL280C	3.15E-13
YNR060W	4.42E-22
YPR065W	8.26E-22
YOL049W	5.22E-08
YMR009W	1.22E-25
YOR237W	1.91E-18
YMR244W	3.62E-14
YMR134W	1.46E-38
YKL150W	2.89E-10
YCR069W	2.91E-10
YIL156W	3.19E-08
YNL156C	9.96E-42
YPL117C	7.92E-22
YER011W	7.35E-11
YPL027W	6.32E-08
YJR105W	5.50E-12
YHR004C	8.10E-09
YDL174C	2.37E-16
YDR044W	1.85E-10

YER044C	4.88E-39
YDR037W	2.02E-08
YAL016W	2.23E-08
YGL056C	3.19E-12
YJR047C	2.86E-08
YAL039C	1.73E-07
YJL105W	2.65E-13
YGR049W	9.65E-26
YOR377W	4.76E-19
YMR201C	4.07E-10
YLL012W	5.09E-11
YJL133C-A	2.86E-08
YGR060W	3.57E-14
YBR242W	2.36E-24
YHR048W	1.45E-13
YER053C-A	6.25E-29
YCR073W-A	8.96E-08
YJL167W	4.97E-21
YMR015C	1.32E-28
YOR229W	2.82E-10

Hot Spot 13:70000

Accession ID	p-value
YDR481C	5.61E-43
YML113W	4.60E-09
YML035C	5.13E-09
YIL160C	6.21E-08
YPL199C	8.35E-09
YHR198C	3.59E-08
YAL034C	6.58E-09
YPL269W	6.13E-10
YLR089C	9.50E-12
YDR515W	1.11E-07
YBR296C	7.69E-80
YHR005C	1.17E-17
YOL011W	2.39E-09
YHL032C	8.74E-08
YBR295W	1.58E-08
YML091C	1.48E-22
YKR092C	3.69E-09
YGR088W	1.89E-09
YDL085W	2.90E-09
YPR184W	1.54E-08
YML124C	1.53E-11
YER103W	9.38E-10
YOR045W	4.77E-12
YCR059C	7.22E-08
YJL088W	6.58E-12

YCR098C	4.11E-08
YCR011C	3.28E-09
YCR073C	9.22E-10
YER069W	1.41E-11
YJR032W	1.27E-08
YGL038C	1.13E-07
YML123C	2.03E-55
YPL272C	1.43E-09
YDL222C	9.72E-09
YNL073W	2.11E-07
YOL064C	6.85E-08
YDR349C	1.32E-08
YGR166W	2.90E-09
YLR267W	2.79E-11
YPL019C	8.38E-48
YDR502C	5.26E-12
YGR170W	5.53E-09
YIR031C	5.19E-09
YDR453C	2.73E-08
YLR410W	2.07E-18
YGR223C	1.63E-07
YML096W	3.09E-09
YDL182W	2.40E-09
YER150W	3.37E-08
YAR008W	3.53E-11
YFL004W	1.86E-30
YCL027W	1.41E-16
YER054C	9.52E-10
YER073W	2.48E-09
YMR196W	3.74E-09
YGR031W	5.95E-09
YKL084W	3.11E-08
YKL091C	5.64E-07
YGR233C	3.60E-38
YBL016W	2.08E-09
YHR030C	3.68E-07
YML030W	7.99E-08
YAL026C	1.87E-07
YLR179C	4.27E-12
YHR103W	2.34E-08
YML070W	7.52E-10
YOL140W	1.66E-07
YDL181W	4.32E-10
YBR067C	8.28E-09
YBL082C	7.14E-07
YOL152W	6.66E-14
YML098W	1.88E-09

YDR369C	3.05E-08
YPR013C	1.61E-08
YHR199C-A	5.39E-08
YEL052W	1.23E-08
YMR236W	4.55E-08
YJL079C	1.74E-07
YML078W	4.08E-27
YNL244C	2.33E-07
YDR130C	6.51E-08
YOL069W	3.89E-09
YHR214C-E	1.34E-19
YFR044C	1.59E-09
YER165W	4.07E-07
YML087C	4.05E-11
YCR037C	1.79E-44
YJR060W	2.59E-10
YJL012C	1.52E-47
YBR105C	1.33E-08
YER055C	7.39E-08
YNL267W	3.70E-10
YLR355C	1.18E-18
YLR193C	1.92E-08
YAL012W	4.37E-09
YCL049C	8.68E-09
YNR028W	3.62E-07
YIR027C	1.95E-12
YPL244C	2.85E-08
YNR032W	6.77E-11
YDR309C	3.64E-10
YLR348C	2.80E-16
YPR035W	2.20E-10
YNL100W	7.25E-10
YOR066W	5.00E-09
YML076C	4.32E-31
YDL052C	4.52E-07
YML066C	3.33E-33
YML128C	1.49E-07
YLL018C-A	5.31E-08
YCR089W	1.50E-14
YML092C	3.99E-10
YMR240C	3.62E-07
YJL213W	1.35E-10
YHR183W	1.84E-22
YNL279W	1.10E-15
YGR156W	3.37E-08
YKL046C	3.14E-07
YHR136C	6.08E-48

YJL117W	1.06E-27
YMR198W	2.11E-08
YLR438W	6.35E-47
YHR007C	6.39E-09
YMR065W	5.74E-13
YER060W-A	1.27E-07
YDL073W	1.42E-08
YJL098W	5.22E-08
YLR142W	6.46E-09
YOR163W	5.79E-22
YMR300C	4.83E-12
YNL194C	5.79E-09
YER043C	3.37E-08
YIL111W	8.48E-14
YIL136W	5.05E-09
YHR047C	1.71E-19
YOR317W	2.22E-07
YPL268W	5.12E-13
YIL050W	5.78E-13
YHR162W	2.57E-10
YDR124W	1.08E-08
YCL042W	1.12E-09
YLL055W	9.90E-15
YNL218W	5.15E-08
YMR181C	3.97E-10
YBL043W	5.72E-11
YML111W	4.11E-18
YKL189W	2.70E-07
YDR019C	2.33E-11
YOR374W	1.73E-15
YOL016C	2.31E-12
YGR189C	5.70E-11
YMR296C	6.05E-09
YHR084W	1.58E-08
YOR065W	1.56E-07
YPL052W	1.92E-07
YOR212W	7.10E-09
YGL060W	6.17E-12
YOL159C	1.30E-09
YIL051C	9.80E-50
YAR071W	1.96E-67
YML119W	3.58E-09
YDR529C	2.82E-07
YDL090C	2.10E-09
YPL110C	1.95E-15
YKR093W	7.58E-32
YML047C	2.16E-09

YHR013C	5.14E-08
YGR243W	2.80E-08
YPL265W	1.88E-13
YKL087C	9.89E-08
YPL014W	2.01E-07
YJL157C	2.74E-11
YKL127W	6.80E-12
YHL016C	1.35E-12
YCL025C	1.16E-15
YHR022C	4.24E-09
YML085C	1.23E-22
YMR177W	1.05E-08
YOR274W	2.81E-10
YGL234W	9.15E-10
YLR452C	1.87E-15
YPL061W	6.14E-08
YIL008W	3.01E-09
YCL055W	8.08E-17
YPL156C	1.37E-11
YLR180W	6.75E-14
YNL258C	1.45E-07
YCL064C	1.36E-87
YPR074C	3.91E-08
YDR111C	9.23E-08
YNR044W	9.00E-14
YPL111W	1.23E-38
YML110C	1.61E-07
YML121W	2.02E-14
YNR002C	2.21E-21
YGR035C	3.26E-09
YOR316C	1.82E-10
YKL120W	4.43E-08
YKL051W	9.37E-13
YOR375C	5.00E-09
YLL056C	2.06E-07
YBR208C	1.09E-16
YOR032W-A	4.90E-10
YBR083W	3.99E-09
YER081W	7.65E-08
YOR247W	1.30E-08
YPL186C	2.06E-08
YPL049C	2.66E-08
YIR028W	9.58E-10
YPR139C	1.28E-08
YGR238C	2.64E-07
YCL040W	7.83E-10
YOL058W	7.32E-13

YML104C	2.20E-10
YER072W	7.76E-39
YML079W	5.66E-16
YPL018W	1.41E-24
YNR001C	3.35E-08
YKL079W	4.92E-07
YGR007W	1.50E-07
YGL167C	5.35E-07
YLR178C	7.08E-09
YML069W	7.98E-12
YLR164W	1.84E-09
YDR482C	1.92E-13
YLR121C	2.71E-09
YER034W	3.07E-08
YPL004C	9.83E-08
YNL141W	4.19E-08
YIL120W	6.97E-09
YGL255W	1.61E-07
YBR093C	1.49E-96
YGR061C	1.59E-08
YFR006W	1.30E-13
YDR281C	2.89E-57
YER132C	4.21E-08
YML029W	1.15E-09

Hot Spot 15:150000

Accession ID	p-value
YKR009C	1.62E-09
YML113W	3.80E-12
YIL157C	4.73E-10
YPL223C	9.46E-28
YML035C	7.61E-11
YER087C-B	6.45E-08
YIL160C	2.95E-27
YDL048C	1.67E-11
YDR447C	5.69E-08
YKL195W	3.48E-08
YDR144C	2.13E-08
YHR002W	1.15E-07
YJL185C	1.73E-09
YGR130C	5.61E-12
YDR447C	2.04E-22
YNR038W	1.17E-09
YJL165C	2.38E-10
YKL096W	7.66E-18
YHR198C	6.62E-17
YNR055C	3.96E-11
YLR149C	3.25E-18

YOR271C	5.33E-17
YHR197W	1.35E-09
YAL034C	5.54E-21
YOR084W	1.56E-08
YDR245W	2.26E-09
YOR187W	1.37E-10
YGL121C	3.74E-08
YBR285W	3.16E-17
YOL028C	7.51E-13
YGR110W	7.48E-09
YKL192C	4.21E-08
YBR169C	1.02E-13
YKL167C	3.46E-08
YDL061C	1.27E-14
YOL122C	3.80E-19
YPR117W	6.28E-09
YER053C	6.98E-16
YOL105C	2.31E-36
YMR227C	2.86E-07
YCL054W	4.74E-07
YGR174W-A	9.35E-12
YHR128W	1.41E-08
YOL077W-A	5.81E-08
YDL191W	2.34E-11
YGR083C	3.56E-10
YBR084C-A	2.13E-11
YAL003W	4.33E-13
YGL104C	2.97E-08
YMR169C	1.03E-28
YKL194C	5.47E-09
YDR450W	9.09E-11
YHL032C	1.23E-23
YBR295W	7.86E-14
YKL085W	3.62E-10
YLR029C	1.66E-10
YML091C	1.53E-09
YMR128W	1.11E-07
YPR155C	1.03E-14
YIL052C	4.82E-13
YHR133C	8.76E-10
YMR131C	1.94E-09
YNL055C	1.33E-12
YPL131W	8.97E-17
YKR092C	2.73E-12
YGR088W	2.95E-30
YMR135C	1.46E-11
YDL085W	2.58E-12

YDL236W	1.20E-10
YPR184W	4.14E-33
YDL223C	2.45E-30
YDL173W	8.43E-10
YOL090W	1.13E-15
YML124C	1.04E-07
YHR065C	2.90E-07
YOL036W	2.61E-10
YOL098C	9.71E-10
YMR008C	4.46E-07
YDR454C	6.05E-10
YML125C	5.82E-09
YHL001W	5.83E-10
YOR178C	1.53E-21
YJL016W	1.44E-14
YJR123W	4.07E-14
YGR027C	3.22E-09
YHR097C	5.50E-13
YGL253W	1.07E-21
YGR258C	4.92E-13
YDL229W	1.70E-18
YER103W	1.65E-12
YKL110C	2.20E-07
YJL205C	1.11E-07
YJR121W	4.81E-09
YNL313C	6.37E-08
YKL180W	1.49E-12
YMR301C	1.03E-09
YEL036C	9.20E-08
YLR185W	4.19E-15
YCR059C	1.78E-14
YML093W	4.47E-07
YHR087W	3.81E-13
YLL028W	7.28E-37
YHR005C-A	9.37E-08
YKL081W	4.02E-19
YCR073C	6.06E-18
YOL109W	1.35E-14
YGL031C	1.21E-09
YER013W	1.40E-11
YGL013C	2.53E-07
YBL072C	2.16E-15
YDR001C	8.54E-08
YMR280C	3.10E-15
YLR218C	2.43E-08
YJR032W	3.34E-13
YBL027W	2.10E-13

YER102W	1.97E-15
YGL189C	9.34E-16
YGL038C	7.97E-10
YOR028C	2.28E-15
YKR057W	9.27E-10
YNR043W	2.19E-13
YPL036W	3.52E-09
YGR085C	7.42E-16
YPL189W	1.13E-08
YGR148C	1.98E-10
YKR044W	8.46E-11
YFR045W	1.86E-10
YPR175W	3.16E-09
YGR214W	2.84E-15
YPL017C	1.06E-19
YDR345C	8.99E-08
YCR083W	2.20E-14
YAL042W	8.91E-08
YGR205W	5.23E-09
YER007C-A	6.20E-08
YKL035W	6.22E-11
YOL061W	4.10E-10
YBL068W	1.41E-13
YPL272C	1.23E-09
YDL199C	1.45E-09
YFL030W	3.60E-23
YKL162C	7.13E-09
YAL017W	3.72E-09
YOL097C	6.23E-12
YOR341W	3.74E-08
YHR129C	1.86E-09
YDR090C	1.23E-10
YGR287C	2.54E-11
YJL109C	1.92E-07
YER149C	1.25E-08
YMR142C	9.49E-15
YDL229W	1.51E-18
YKL187C	7.37E-09
YCR091W	2.98E-13
YKL037W	8.85E-12
YMR239C	1.79E-11
YLR075W	5.42E-14
YDR064W	3.49E-12
YNR066C	3.91E-07
YLR271W	4.77E-10
YPR163C	2.94E-10
YOR152C	4.71E-13

YDL222C	2.79E-41
YNL073W	2.12E-09
YOL120C	4.30E-13
YBR162C	8.89E-08
YDL031W	1.55E-07
YDR337W	3.48E-07
YML054C	2.90E-16
YGL111W	7.12E-09
YLR249W	5.48E-21
YHR072W	6.72E-07
YBR023C	4.92E-09
YNL217W	1.23E-17
YDR349C	4.10E-17
YGL096W	2.04E-13
YKR049C	9.25E-19
YKR043C	3.81E-21
YGR053C	5.32E-09
YOR246C	1.09E-14
YLR222C	1.22E-07
YMR241W	2.02E-09
YER006W	5.76E-08
YCL059C	4.54E-10
YGR166W	3.09E-11
YLR267W	7.26E-08
YBR121C	5.60E-13
YHR085W	1.73E-10
YHR140W	1.84E-08
YHR062C	2.95E-10
YHR139C	6.98E-12
YDR490C	1.82E-10
YFR039C	1.28E-09
YPR140W	8.41E-08
YDR502C	5.69E-12
YGR208W	1.46E-09
YPR010C	1.56E-09
YMR010W	1.89E-09
YER126C	6.37E-08
YGL189C	4.14E-13
YDR528W	5.86E-11
YIL069C	1.95E-12
YPR043W	4.07E-17
YNL161W	4.50E-10
YPL132W	3.53E-16
YER088C	1.16E-14
YER101C	3.04E-14
YNL113W	2.02E-08
YOL104C	1.72E-44

YAR002C-A	1.37E-07
YOL127W	3.42E-12
YKL161C	1.56E-10
YDR306C	3.23E-08
YDR453C	4.19E-29
YDR273W	2.77E-07
YPR191W	6.44E-08
YER150W	3.39E-40
YER066W	1.83E-10
YGL190C	3.46E-07
YJL208C	1.46E-09
YLR172C	1.29E-18
YER054C	1.51E-36
YOL022C	7.72E-14
YNL045W	9.57E-09
YHR143W-A	1.72E-08
YEL054C	1.31E-13
YIL091C	1.72E-09
YEL047C	2.13E-08
YDR129C	1.08E-07
YAL005C	5.67E-10
YFL017C	5.59E-09
YAL029C	2.46E-08
YJL131C	1.48E-10
YML026C	3.06E-12
YNL160W	4.31E-16
YMR242C	1.22E-12
YKL072W	3.43E-16
YMR116C	1.93E-18
YLR204W	7.59E-08
YMR196W	6.94E-25
YBR111C	2.32E-08
YGR031W	2.31E-09
YPR060C	6.70E-12
YIR012W	2.28E-10
YKL084W	6.37E-18
YGR201C	4.85E-24
YPL043W	1.27E-08
YLR270W	1.84E-12
YLR150W	1.70E-13
YKL091C	7.25E-25
YKR016W	6.09E-09
YDL235C	5.49E-08
YEL011W	4.57E-11
YDR418W	7.14E-14
YPL196W	1.48E-12
YJR055W	8.49E-12

YGR033C	3.84E-09
YHR030C	6.10E-10
YGR217W	3.03E-12
YML030W	3.41E-14
YGL128C	3.61E-07
YDR202C	1.80E-12
YJL137C	1.25E-09
YJR039W	5.31E-09
YJL189W	9.57E-11
YMR244C-A	1.93E-11
YIR037W	2.30E-10
YPR091C	9.19E-12
YJR097W	8.21E-10
YMR251W-A	4.17E-08
YER177W	6.69E-08
YHR207C	9.25E-09
YFL054C	8.55E-08
YLR388W	5.45E-11
YGR264C	1.43E-15
YPL048W	6.78E-09
YML070W	4.11E-12
YGL229C	4.65E-08
YGR175C	1.13E-13
YKR026C	7.85E-10
YML063W	4.44E-13
YJR054W	9.24E-09
YEL058W	3.56E-09
YDR211W	1.95E-14
YNL044W	7.29E-09
YCL035C	5.33E-17
YLR388W	3.46E-10
YOL006C	2.05E-09
YLR175W	1.53E-07
YLR251W	1.30E-11
YPL221W	3.68E-07
YDL181W	1.84E-21
YKR094C	6.87E-13
YGL065C	3.69E-08
YDR096W	1.05E-17
YKL062W	5.10E-19
YEL050C	1.12E-08
YDR504C	6.06E-08
YGR001C	8.55E-08
YNL087W	1.33E-19
YPL084W	1.18E-07
YEL046C	1.68E-19
YLR344W	7.72E-14

YLR312C	1.81E-07
YDR018C	3.89E-12
YIL046W	7.89E-09
YHR089C	5.31E-07
YBR286W	2.20E-22
YOL117W	5.44E-07
YIL105C	1.52E-10
YNL015W	1.21E-07
YJL163C	8.08E-14
YKL193C	2.78E-09
YOL152W	1.68E-07
YNL108C	2.17E-11
YLR213C	9.80E-09
YHR176W	7.01E-08
YLR048W	2.32E-14
YOL130W	3.00E-09
YKR063C	1.78E-13
YOR120W	3.30E-21
YJR008W	7.44E-19
YMR319C	7.77E-10
YBL072C	4.10E-15
YNR012W	5.52E-14
YDR012W	8.69E-11
YGL022W	7.13E-08
YBL013W	1.04E-15
YNR034W	5.44E-08
YHR156C	2.68E-08
YEL052W	6.87E-12
YOR312C	6.13E-09
YEL055C	1.81E-07
YDL213C	8.80E-10
YGR052W	7.08E-36
YNL178W	1.53E-12
YGL004C	3.85E-11
YDR430C	2.74E-07
YIL108W	1.07E-09
YOL019W	1.88E-14
YBR003W	1.59E-09
YDR165W	8.89E-08
YMR229C	8.91E-09
YIL018W	1.89E-13
YMR264W	5.46E-08
YOR291W	6.24E-10
YLR295C	7.04E-10
YGL040C	2.02E-07
YLR200W	1.42E-07
YOR292C	3.33E-11

YHR001W-A	2.47E-09
YDR060W	6.47E-11
YIL079C	5.59E-10
YPL118W	3.86E-09
YDR341C	3.64E-11
YBL015W	2.46E-09
YKL212W	1.10E-07
YJL014W	1.33E-09
YPR110C	7.28E-10
YGR034W	1.23E-13
YEL026W	1.44E-17
YKL024C	2.70E-09
YKL004W	1.49E-08
YBR037C	3.95E-09
YFR044C	1.10E-07
YKR018C	2.65E-08
YOL041C	1.93E-08
YDR032C	2.72E-08
YER165W	2.40E-13
YFR011C	6.72E-09
YKL100C	4.06E-11
YIL117C	3.16E-12
YER169W	1.57E-09
YLR382C	1.98E-07
YFR017C	1.14E-14
YER182W	1.80E-12
YPL183W-A	1.17E-06
YER162C	8.16E-10
YOR086C	1.45E-08
YMR185W	4.23E-08
YOL080C	1.01E-09
YBR230C	3.70E-09
YOL081W	8.03E-10
YNL098C	3.31E-08
YGR112W	2.05E-11
YDR300C	1.89E-15
YMR107W	1.93E-18
YAL036C	3.19E-08
YKL096W-A	3.10E-13
YOR046C	8.67E-09
YNL175C	6.50E-08
YJL141C	1.99E-14
YPL030W	2.65E-13
YMR165C	2.83E-10
YDR046C	1.18E-09
YDL130W	1.11E-13
YOR004W	2.03E-08

YJL012C	3.05E-08
YGR086C	2.11E-13
YBR105C	2.64E-07
YMR261C	2.93E-12
YJL103C	8.55E-10
YPL207W	1.79E-17
YLR446W	9.91E-14
YLR394W	8.55E-08
YIR016W	6.99E-22
YOL084W	5.31E-50
YMR118C	1.77E-10
YPL239W	7.48E-14
YOR206W	3.13E-08
YFR014C	1.06E-21
YHR195W	4.94E-13
YHL036W	2.78E-11
YLR355C	1.35E-08
YIL097W	8.43E-08
YBR114W	2.03E-08
YNR036C	1.04E-10
YDL061C	2.07E-16
YHL033C	7.74E-14
YMR114C	3.87E-12
YKR060W	2.52E-09
YLR193C	2.12E-11
YPL175W	1.79E-13
YMR016C	2.26E-15
YDL024C	3.38E-14
YAL012W	5.58E-10
YOL131W	3.31E-25
YLL019C	2.28E-11
YJR016C	1.66E-10
YLR070C	9.56E-17
YIL127C	1.32E-07
YDL083C	5.16E-12
YGR256W	6.42E-15
YNL093W	8.33E-20
YMR175W	1.77E-18
YLR002C	2.02E-07
YDR098C	3.21E-20
YIL047C	1.00E-15
YMR031C	1.11E-15
YLR345W	1.53E-17
YDL121C	2.00E-08
YCR030C	4.32E-10
YMR012W	2.40E-07
YOL092W	2.13E-28

YNL195C	1.05E-22
YDR309C	1.32E-11
YMR271C	1.56E-08
YGR111W	1.90E-11
YLR448W	7.94E-12
YIR035C	1.81E-07
YHL034C	3.26E-09
YNL125C	1.70E-08
YCL057C-A	4.26E-07
YML024W	6.06E-10
YNL209W	4.13E-19
YDR339C	3.78E-09
YDR303C	3.94E-08
YDR436W	7.37E-11
YOR167C	6.77E-11
YOR310C	5.97E-11
YOL048C	1.95E-08
YDR185C	1.20E-12
YOL052C-A	7.37E-12
YLR392C	1.68E-11
YER002W	8.39E-09
YGL187C	1.44E-07
YML042W	8.86E-09
YLR177W	5.87E-16
YJL161W	7.92E-17
YBL035C	2.44E-08
YJL181W	9.93E-13
YOL039W	2.02E-13
YJL066C	7.78E-10
YMR250W	1.44E-13
YMR214W	1.41E-13
YNR033W	4.06E-11
YPL249C-A	9.79E-08
YGR062C	3.66E-08
YGR248W	6.67E-24
YLR196W	1.78E-07
YLL026W	2.04E-12
YGR286C	6.43E-08
YML026C	9.97E-13
YIL078W	1.10E-08
YNL200C	5.77E-10
YMR194W	7.19E-08
YMR182W-A	2.11E-11
YOR354C	1.29E-09
YNL100W	3.94E-17
YHR020W	2.77E-13
YLR023C	1.83E-07

YPL108W	7.31E-10
YPL015C	2.09E-09
YER045C	4.45E-11
YDR533C	9.05E-49
YLR168C	6.00E-09
YDL214C	5.02E-13
YBR104W	9.36E-10
YML100W	5.55E-12
YPL012W	2.22E-09
YGR043C	4.27E-26
YDR025W	2.79E-12
YBR263W	1.02E-07
YOR386W	4.20E-11
YLR194C	1.69E-10
YKL056C	1.16E-09
YIL033C	1.44E-10
YPL011C	2.75E-08
YMR148W	3.88E-18
YKR017C	1.49E-07
YNL284C	5.07E-07
YJL045W	7.31E-12
YLR102C	3.51E-12
YGL078C	2.89E-08
YOR107W	7.73E-10
YML056C	4.15E-14
YGR187C	8.45E-09
YHR092C	1.67E-09
YML128C	9.32E-29
YLL018C-A	2.74E-22
YJR003C	9.27E-16
YGR128C	9.49E-11
YOR173W	4.19E-44
YCL051W	2.40E-08
YOR207C	9.64E-13
YCR031C	1.35E-12
YPL211W	1.92E-08
YNL081C	1.37E-08
YDL241W	3.35E-15
YDR351W	4.58E-08
YER131W	1.10E-09
YJL191W	3.99E-13
YFL036W	4.45E-08
YHR183W	2.07E-16
YPL266W	2.09E-10
YHR068W	8.44E-19
YPL230W	1.02E-21
YLR203C	2.36E-12

YGR156W	2.20E-10
YDR321W	2.37E-16
YPL119C-A	1.48E-07
YHR187W	2.04E-09
YMR038C	1.04E-13
YOR233W	1.48E-11
YPL171C	2.19E-14
YJL052W	4.51E-15
YKL046C	1.38E-08
YLR325C	2.62E-10
YGL077C	5.40E-08
YMR056C	1.17E-06
YKL109W	3.75E-13
YHR033W	1.44E-11
YDR500C	2.02E-11
YLR301W	1.13E-06
YDL051W	2.38E-08
YPL079W	4.32E-09
YDL208W	1.13E-08
YDR047W	5.27E-12
YHR007C	3.41E-10
YCR004C	8.86E-12
YDR161W	1.66E-08
YCR051W	1.67E-08
YDR260C	2.00E-08
YGR155W	8.13E-10
YLR061W	2.78E-11
YDR342C	3.19E-15
YHR141C	3.42E-10
YEL024W	1.71E-09
YJR132W	1.40E-10
YLR293C	2.32E-14
YPL109C	4.28E-09
YOL034W	5.27E-08
YPR161C	2.88E-10
YGR220C	9.54E-08
YHR009C	2.13E-07
YLL001W	1.13E-07
YDL022W	1.57E-08
YDL183C	2.31E-11
YDL019C	4.26E-13
YMR308C	2.08E-07
YDR494W	1.61E-07
YOR005C	6.08E-07
YOR243C	3.19E-10
YER156C	5.96E-10
YDL206W	2.98E-09

YLR197W	3.74E-10
YOL119C	1.08E-10
YJL210W	1.36E-11
YNL194C	8.48E-36
YML071C	9.23E-09
YMR272C	1.71E-16
YKL026C	1.17E-21
YER043C	1.08E-16
YIL099W	2.12E-20
YLR084C	1.88E-10
YMR090W	1.89E-22
YIL077C	3.23E-14
YMR136W	1.45E-12
YMR041C	9.49E-12
YIL136W	1.46E-34
YLR333C	2.93E-14
YPL271W	2.44E-09
YHR047C	1.28E-19
YFR053C	3.79E-10
YHR045W	3.96E-09
YGL146C	1.95E-10
YOR254C	7.89E-09
YJL190C	3.25E-12
YER110C	3.54E-15
YBR189W	5.56E-13
YPL160W	4.34E-16
YHR100C	1.27E-09
YBR001C	2.73E-08
YNL302C	7.72E-10
YOR358W	4.86E-08
YHL011C	1.69E-11
YLR409C	2.65E-07
YML126C	3.23E-07
YJR077C	9.54E-09
YMR206W	9.33E-11
YAL061W	3.35E-11
YJR036C	7.78E-11
YOR335C	1.33E-08
YPR030W	1.69E-09
YDR132C	6.91E-08
YDR216W	7.27E-33
YPR132W	2.34E-12
YKL151C	1.34E-22
YDR204W	2.98E-10
YPL053C	1.61E-08
YPL222W	3.67E-15
YOR348C	6.25E-07

YIL158W	9.03E-07
YPL247C	7.16E-23
YDR256C	2.27E-13
YLR143W	1.27E-08
YKL006W	4.46E-13
YBL029C-A	2.53E-09
YMR211W	3.33E-08
YLR372W	6.18E-11
YER074W	9.13E-15
YFR031C-A	6.52E-16
YOL113W	6.91E-08
YLL055W	2.81E-15
YMR267W	1.24E-08
YOR188W	3.12E-07
YNL218W	5.91E-07
YMR181C	2.01E-16
YNL053W	1.09E-08
YBR280C	1.42E-09
YHL033C	2.63E-16
YGL209W	2.11E-07
YMR093W	6.24E-09
YDL082W	2.95E-11
YDL021W	1.42E-08
YDR074W	1.75E-16
YDR171W	1.39E-13
YMR011W	1.44E-12
YKL093W	4.65E-15
YLR332W	2.55E-07
YKL142W	3.00E-08
YML106W	3.96E-10
YAL040C	6.17E-11
YJR005W	2.84E-08
YJR041C	7.70E-11
YOL095C	1.16E-16
YNL247W	1.74E-11
YKR058W	1.08E-17
YFL042C	2.41E-09
YOL103W	3.40E-09
YOR276W	2.27E-11
YGR236C	1.04E-13
YPR018W	1.48E-07
YPL040C	1.60E-10
YLR432W	2.96E-11
YGL115W	1.72E-08
YOL029C	3.99E-16
YER056C-A	1.36E-11
YLR449W	3.49E-19

YBR261C	3.84E-11
YGR189C	5.25E-14
YPL104W	1.39E-10
YOR048C	5.69E-11
YHR106W	4.41E-08
YPR172W	1.97E-09
YHR080C	2.82E-17
YBR029C	3.21E-07
YMR230W	7.78E-08
YMR296C	4.76E-07
YDL014W	8.00E-08
YPR008W	4.59E-10
YDR023W	2.56E-11
YLR146C	3.38E-09
YGL147C	2.64E-11
YOR286W	4.21E-08
YNL115C	1.04E-07
YOL056W	7.19E-11
YOR311C	1.86E-08
YNL302C	3.28E-10
YGL037C	2.13E-09
YPL236C	1.45E-07
YER001W	2.77E-08
YNL255C	1.21E-11
YPL052W	3.37E-14
YLL041C	3.64E-08
YGR237C	1.18E-11
YLR375W	3.26E-08
YKL155C	7.44E-07
YDR513W	7.29E-08
YPR026W	2.26E-18
YGR143W	8.46E-09
YLL046C	3.31E-08
YML073C	2.59E-12
YNL274C	4.26E-12
YLR228C	1.53E-07
YGL103W	8.88E-12
YKL052C	9.29E-17
YJR080C	2.32E-07
YOL089C	5.87E-36
YBR052C	3.98E-08
YMR312W	1.13E-08
YJL010C	6.45E-10
YLR176C	2.15E-09
YNL134C	3.44E-26
YBR148W	1.83E-12
YGR127W	2.74E-13

YBL087C	2.32E-16
YGR174C	7.22E-09
YOL121C	1.89E-08
YGL123W	3.36E-11
YHR025W	5.35E-12
YNL137C	7.11E-08
YOL102C	3.49E-15
YKR093W	3.52E-39
YOR312C	7.85E-12
YOR186W	4.91E-10
YHR088W	8.58E-10
YMR174C	5.45E-18
YNL069C	6.40E-13
YJR122W	2.90E-09
YLR219W	4.41E-17
YCR034W	2.93E-12
YNL227C	2.83E-08
YJL034W	1.56E-11
YER130C	3.21E-10
YOR215C	3.42E-09
YOL091W	1.58E-41
YOR220W	2.75E-08
YHR010W	1.10E-14
YIL155C	5.16E-10
YGR243W	1.20E-11
YGR077C	1.82E-07
YBR034C	4.89E-09
YBR072W	1.60E-14
YGR102C	1.85E-12
YJL005W	1.35E-13
YER082C	4.49E-17
YML018C	2.88E-09
YDR058C	5.33E-09
YKL216W	2.14E-09
YGR123C	2.26E-07
YOL116W	1.06E-10
YMR295C	6.43E-07
YDR263C	1.37E-07
YIL101C	1.30E-17
YPL265W	2.01E-09
YGL058W	9.76E-15
YKL087C	4.42E-09
YMR072W	1.00E-09
YGR174W-A	1.24E-12
YER067W	5.24E-09
YDR332W	2.91E-07
YER049W	3.37E-13

YJL062W-A	7.57E-08
YER074W	1.03E-14
YJR043C	1.37E-07
YER107C	1.84E-11
YBL030C	1.30E-07
YOR227W	3.78E-11
YPL014W	5.40E-11
YJR127C	9.26E-11
YDR277C	7.17E-10
YMR194C-B	2.63E-15
YGR138C	3.37E-13
YGR118W	1.06E-09
YKL127W	7.54E-12
YGL081W	3.77E-10
YDR421W	4.53E-10
YOR096W	7.47E-16
YLR406C	1.27E-11
YOR293W	1.46E-10
YHR016C	9.21E-12
YDR091C	1.20E-08
YKR076W	1.10E-07
YGR021W	6.93E-12
YBR284W	3.26E-14
YGR200C	4.30E-10
YHR064C	1.80E-12
YMR297W	9.73E-09
YHR017W	4.56E-13
YMR157C	8.50E-07
YGL076C	1.05E-16
YNL133C	9.24E-10
YBR117C	2.44E-19
YDR034W-B	4.33E-23
YHR022C	6.72E-09
YJL118W	8.47E-11
YDR296W	2.01E-07
YOR241W	2.68E-16
YOR001W	3.71E-10
YOR161C	1.23E-22
YMR286W	2.53E-07
YER056C	2.74E-09
YLR327C	5.39E-09
YDL079C	2.09E-12
YCR043C	2.52E-08
YMR177W	7.07E-19
YOR274W	1.67E-19
YEL057C	3.46E-09
YIL087C	2.88E-14

YNR060W	6.63E-07
YGR244C	2.42E-09
YFR032C-A	2.43E-11
YDL166C	2.83E-09
YPL061W	1.85E-08
YIL008W	4.84E-12
YDL211C	1.49E-07
YJR034W	1.11E-06
YPL156C	6.89E-09
YBL086C	2.23E-11
YHR111W	4.06E-11
YIL039W	2.87E-07
YER117W	3.12E-13
YBR191W	2.38E-13
YOR063W	2.90E-14
YJR063W	3.54E-09
YKR079C	1.05E-18
YPL089C	8.60E-09
YPR036W	8.10E-08
YBR162W-A	2.89E-07
YBR007C	1.87E-12
YMR145C	1.46E-07
YOL054W	4.88E-15
YDR420W	3.54E-07
YBR025C	7.38E-15
YGL145W	1.48E-09
YLR441C	1.25E-11
YPL219W	3.85E-08
YGR194C	3.74E-09
YGR285C	1.28E-13
YIL021W	1.36E-07
YLL034C	9.81E-09
YML110C	5.71E-11
YHR096C	5.09E-20
YHR019C	5.28E-09
YPR033C	1.33E-11
YNR002C	8.33E-22
YDR041W	2.48E-09
YDR197W	2.34E-17
YAR029W	3.57E-09
YBR031W	2.40E-17
YLL045C	6.52E-15
YGR035C	5.64E-12
YER025W	1.48E-10
YOR315W	5.69E-09
YIL036W	8.21E-10
YKL120W	2.59E-11

YKL051W	2.97E-21
YNL234W	7.40E-12
YHR144C	1.53E-11
YLR340W	2.38E-10
YNL301C	7.02E-14
YER011W	1.44E-09
YKL104C	2.73E-07
YOL094C	3.20E-09
YLL056C	1.05E-08
YLR118C	7.61E-10
YDR198C	1.11E-10
YOL040C	2.10E-13
YJL011C	8.30E-08
YMR105C	1.47E-12
YNL061W	4.11E-08
YBR181C	2.45E-14
YJR096W	8.62E-24
YKL014C	6.94E-09
YDL081C	1.35E-08
YNL294C	5.46E-07
YDR406W	1.85E-28
YJR101W	2.64E-07
YBR185C	3.73E-08
YDR324C	1.14E-07
YER062C	7.36E-18
YHR208W	2.57E-09
YHL024W	2.95E-13
YOR247W	7.23E-10
YPL186C	3.77E-35
YJL069C	4.57E-09
YGL025C	4.16E-08
YLR258W	3.48E-10
YAL041W	8.74E-07
YGR152C	3.46E-07
YGR238C	7.23E-07
YGL130W	7.68E-09
YCL040W	3.55E-08
YHR203C	1.94E-12
YMR143W	6.16E-09
YOR288C	6.93E-08
YOL088C	2.36E-26
YBL061C	4.07E-17
YKL073W	5.57E-08
YBR048W	1.58E-11
YGL135W	4.88E-15
YDR329C	2.43E-09
YER060W	6.18E-16

YGL237C	4.93E-12
YDR037W	4.85E-07
YBR071W	2.91E-15
YDL204W	2.11E-34
YER096W	5.52E-12
YHR104W	4.65E-18
YBR177C	1.88E-26
YFL022C	3.40E-11
YOL100W	3.53E-17
YNL256W	3.21E-13
YBR147W	3.08E-11
YOR134W	1.42E-11
YBR126C	5.97E-09
YNR001C	1.66E-12
YAL039C	1.68E-07
YDR135C	1.66E-09
YGR195W	1.48E-09
YNR014W	7.57E-23
YGR007W	4.94E-12
YLR178C	1.23E-35
YEL040W	2.96E-18
YJL177W	4.08E-12
YLR083C	6.99E-09
YJR094W-A	8.52E-13
YHR160C	1.48E-22
YGR207C	2.98E-07
YDR070C	1.95E-35
YJR090C	1.02E-07
YDL075W	4.95E-13
YBR030W	1.64E-09
YJL112W	2.77E-09
YMR310C	5.57E-08
YLR164W	8.30E-11
YML006C	3.54E-07
YHL001W	1.22E-12
YGR255C	9.18E-10
YDR482C	2.54E-07
YPL123C	2.76E-08
YHR021C	1.65E-13
YMR217W	3.13E-16
YFL014W	8.42E-31
YBR187W	5.59E-11
YLL012W	1.75E-11
YBR191W	1.56E-12
YOR234C	1.23E-12
YNR065C	8.58E-12
YGR060W	6.76E-08

YPR160W	7.52E-10
YLR121C	7.02E-16
YKR013W	1.98E-08
YAR075W	5.71E-16
YNR034W-A	8.95E-29
YPL004C	9.92E-10
YDR104C	9.21E-10
YLR201C	2.75E-12
YOL096C	1.36E-15
YDR298C	9.83E-08
YPL100W	8.52E-09
YPR149W	2.33E-26
YEL071W	9.21E-07
YGL255W	1.85E-08
YBL092W	5.02E-16
YDR075W	9.26E-09
YOL087C	4.04E-10
YMR238W	7.96E-08
YPL203W	9.69E-10
YMR195W	4.13E-16
YGR094W	9.02E-12
YMR108W	2.04E-10
YDR434W	1.87E-08
YFR006W	1.27E-16
YPR127W	9.75E-08
YML082W	1.01E-10
YJL136C	3.52E-13
YFL041W-A	1.01E-08
YIL145C	8.60E-12
YIL133C	1.62E-13
YER132C	5.74E-09
YOL030W	6.31E-08
YFR031C-A	8.80E-17
YOR293W	2.97E-11
YLL023C	1.85E-10
YGL030W	1.48E-17
YGR070W	1.78E-14
YOL082W	4.81E-31
YPR028W	3.33E-09

Hot Spot 16:510000

Accession ID	p-value
YKR009C	1.55E-07
YJL147C	2.09E-10
YKL138C	4.15E-11
YIR021W	3.43E-09
YEL006W	3.26E-08
YKL195W	2.49E-08

YPL020C	5.67E-17
YOR187W	3.50E-11
YDL061C	2.83E-12
YBR084C-A	3.01E-08
YML091C	1.00E-09
YPL131W	1.84E-11
YKR092C	1.91E-07
YML124C	2.02E-07
YKL047W	1.31E-07
YDL229W	1.62E-13
YKL180W	6.19E-11
YHR038W	3.07E-09
YNR017W	2.20E-11
YBL072C	6.99E-12
YER102W	4.88E-12
YGL189C	1.03E-09
YPL036W	1.33E-27
YPL272C	2.66E-12
YDL229W	3.77E-15
YJL063C	2.74E-08
YNL073W	5.88E-09
YGL068W	3.65E-11
YPL107W	7.06E-17
YIL069C	1.84E-10
YMR225C	4.27E-07
YIR031C	2.90E-09
YPR191W	4.70E-08
YJR152W	5.24E-13
YPR020W	3.75E-08
YML026C	6.16E-09
YMR116C	4.74E-13
YIL070C	2.94E-10
YLR150W	3.09E-11
YML030W	2.47E-08
YLR259C	9.82E-08
YMR251W-A	5.39E-20
YML063W	5.96E-09
YDL181W	1.27E-15
YPL016W	2.99E-34
YOL152W	1.84E-10
YLR048W	2.21E-10
YBL072C	3.76E-09
YHR199C-A	8.61E-11
YDR430C	2.12E-07
YGR281W	4.41E-08
YIL018W	7.61E-10
YKR034W	5.29E-09

YHR037W	1.66E-08
YPL054W	1.02E-11
YLR069C	5.49E-10
YGL040C	2.88E-12
YEL063C	3.72E-12
YBR037C	1.04E-07
YNL142W	1.02E-09
YPL024W	2.61E-12
YBR282W	6.65E-07
YLR382C	2.60E-08
YML129C	1.20E-08
YOR376W-A	5.89E-09
YDR508C	2.20E-10
YGR082W	4.77E-17
YDL130W	1.69E-12
YBR146W	2.90E-07
YGR086C	4.96E-09
YFR055W	6.54E-13
YPL023C	5.71E-44
YDL083C	4.26E-10
YIR027C	1.06E-09
YMR012W	2.41E-09
YJL047C	6.73E-08
YNL185C	5.51E-10
YNL209W	2.67E-14
YIR032C	8.05E-09
YIR029W	3.00E-11
YGL187C	1.84E-10
YDR131C	1.57E-09
YPL031C	2.25E-12
YFR007W	2.91E-08
YOR354C	4.81E-08
YDL052C	4.96E-11
YDR025W	3.95E-11
YLL009C	2.23E-07
YDL120W	8.02E-10
YER131W	2.56E-10
YMR240C	2.28E-08
YDL045W-A	5.79E-07
YGR138C	2.74E-08
YDR500C	3.24E-09
YPL079W	3.63E-11
YDR260C	1.39E-08
YLR061W	9.76E-11
YEL024W	3.91E-08
YLR142W	3.04E-10
YDR194C	2.08E-09

YPL022W	8.00E-13
YPL098C	1.08E-11
YLR333C	2.18E-08
YPL271W	9.40E-12
YGL140C	3.05E-07
YPR132W	6.08E-09
YOR348C	2.17E-08
YER074W	7.78E-11
YMR011W	1.22E-09
YPR133W-A	1.13E-11
YPL038W-A	1.03E-18
YGR154C	4.25E-11
YML068W	6.18E-07
YPL040C	5.91E-10
YOR286W	1.78E-11
YOR065W	3.56E-10
YLR375W	4.03E-07
YPL046C	8.73E-15
YML073C	5.05E-09
YIL131C	1.66E-08
YGL123W	8.73E-10
YGL075C	2.12E-09
YNL137C	2.07E-08
YEL072W	5.96E-08
YJR122W	6.23E-08
YFR046C	6.98E-11
YKL087C	8.66E-14
YJL062W-A	8.24E-09
YCR046C	2.85E-10
YGR138C	1.20E-15
YDR421W	6.34E-18
YMR060C	4.86E-11
YCL025C	8.04E-10
YHR022C	6.44E-09
YKL054C	1.44E-11
YBR191W	7.86E-11
YIL098C	1.15E-07
YDL210W	7.42E-09
YML009C	4.85E-08
YPL038W	5.47E-16
YOL040C	9.05E-11
YBR181C	8.07E-10
YPL072W	1.63E-09
YHL024W	7.11E-09
YOR232W	2.13E-13
YIR028W	4.66E-12
YBR048W	2.93E-09

YAL024C	8.36E-09
YNR001C	7.21E-10
YDR033W	2.84E-12
YJL112W	1.19E-07
YDR115W	1.90E-07
YPL039W	1.37E-20
YHL001W	3.42E-11
YHR021C	1.42E-09
YER039C-A	1.03E-07
YFL014W	1.15E-13
YGL249W	1.48E-09
YER065C	1.93E-07
YPL004C	1.31E-13
YBL092W	1.28E-09
YMR195W	2.15E-08
YBR041W	1.20E-07
YHR099W	8.62E-08
YDR379W	8.44E-08
YIL133C	1.24E-09
YFR031C-A	1.11E-14
YOR293W	1.90E-09

Supplementary Table 8: eQTL hot spots for the union method

Hot Spot 2:550000

Accession ID	p-value
YER059W	1.10E-06
YJL147C	1.53E-07
YDL216C	1.01E-05
YKL138C	5.33E-07
YFL034C-B	7.23E-09
YIR021W	2.62E-06
YHL010C	2.91E-06
YLR286C	3.24E-16
YLR125W	1.29E-06
YGR113W	9.45E-08
YPR106W	1.12E-16
YDR072C	1.48E-08
YGL107C	1.65E-09
YBR157C	1.24E-13
YOR264W	1.97E-15
YOL105C	4.32E-08
YFL047W	2.60E-08
YGL196W	3.27E-08
YHR053C	1.80E-11
YLR299W	1.14E-06
YOR179C	3.77E-07
YJL200C	2.33E-11
YNR067C	1.35E-16
YBR186W	1.09E-08
YDL055C	2.88E-13
YGL084C	6.06E-07
YML124C	2.50E-11
YOL021C	1.48E-07
YJL133W	7.66E-06
YIL009W	4.37E-08
YPL262W	6.97E-07
YFR038W	1.80E-06
YDL218W	1.56E-06
YGR258C	1.72E-07
YJL108C	7.72E-07
YDL155W	4.28E-06
YEL062W	5.92E-08
YMR088C	1.18E-07
YNR020C	1.39E-09
YEL036C	1.77E-08
YMR257C	9.20E-06
YDR055W	8.81E-06
YHR038W	1.91E-08
YOL112W	1.42E-10

YIL094C	4.54E-12
YDR001C	3.60E-07
YDL057W	1.30E-09
YDR353W	2.42E-06
YBR112C	2.92E-07
YBR291C	2.58E-13
YAR014C	2.14E-11
YJR014W	1.67E-06
YDR345C	4.49E-06
YBR197C	1.03E-16
YOR003W	3.88E-08
YER124C	1.74E-16
YGR266W	4.58E-06
YBR135W	6.95E-11
YBL050W	2.02E-06
YOL055C	1.33E-05
YER064C	7.65E-10
YDR175C	8.38E-09
YHR113W	2.28E-08
YJL063C	1.02E-06
YDR234W	5.47E-10
YIR007W	1.95E-07
YIR030C	2.49E-09
YGL068W	7.93E-08
YGL259W	8.99E-08
YJR099W	1.20E-06
YPR181C	3.45E-06
YLR360W	6.60E-10
YPR125W	5.67E-08
YKL103C	7.72E-09
YBR023C	1.36E-09
YOL020W	1.43E-09
YPL066W	4.66E-07
YOR246C	3.44E-06
YLR457C	2.56E-06
YDR370C	2.18E-06
YGR149W	3.13E-06
YBR079C	2.86E-06
YMR078C	4.63E-07
YNL152W	1.07E-06
YER089C	8.25E-08
YNL161W	7.79E-07
YIR031C	9.55E-11
YIL112W	7.46E-06
YPR162C	6.18E-07
YDR272W	1.87E-06
YDR237W	8.62E-08

YMR309C	2.41E-06
YFR002W	2.28E-06
YIL090W	3.93E-06
YJR152W	7.47E-13
YDL182W	2.55E-11
YBR154C	7.22E-07
YMR180C	5.57E-06
YDL230W	1.11E-06
YGR084C	5.39E-09
YNL270C	2.45E-08
YBR175W	4.18E-07
YNR037C	6.42E-06
YOL047C	1.72E-11
YBR165W	2.05E-10
YML025C	3.75E-08
YNL252C	2.51E-06
YPR165W	3.18E-06
YPR141C	2.18E-06
YPR111W	2.73E-09
YMR170C	1.89E-09
YGL185C	9.37E-08
YNR050C	8.88E-13
YIL070C	1.80E-07
YKL185W	5.42E-12
YNL101W	7.77E-07
YJR138W	1.25E-07
YBR172C	1.66E-07
YHR030C	3.86E-08
YGR217W	3.30E-07
YLR319C	5.98E-06
YGR216C	3.85E-06
YER061C	5.18E-06
YHR018C	7.39E-06
YGL229C	1.99E-11
YLR246W	2.65E-06
YBR235W	9.39E-10
YJR054W	3.27E-08
YML118W	2.50E-07
YAL023C	8.18E-07
YOR035C	8.18E-07
YDL225W	2.22E-07
YCR101C	1.19E-06
YGR188C	3.69E-06
YLR253W	3.19E-06
YNL015W	3.86E-09
YIL140W	1.11E-08
YOR056C	8.44E-09

YBR173C	7.38E-13
YKL204W	1.23E-06
YOL128C	3.08E-08
YOR316C-A	2.12E-14
YPL168W	4.95E-07
YJL206C	9.45E-06
YGL004C	1.37E-07
YMR236W	5.46E-08
YER109C	4.09E-08
YGR041W	1.05E-15
YER154W	6.17E-08
YOL019W	5.17E-06
YKR034W	2.34E-14
YML080W	2.05E-06
YER095W	7.73E-07
YOR126C	4.65E-08
YLR069C	8.98E-08
YPL173W	5.33E-07
YBL060W	1.25E-07
YOR278W	7.93E-06
YBR167C	1.64E-10
YJL099W	4.00E-07
YGL164C	5.34E-09
YBR198C	5.77E-07
YEL063C	4.15E-13
YNL142W	2.58E-13
YIL117C	5.65E-11
YLR382C	5.65E-07
YNL298W	2.18E-08
YEL064C	1.66E-08
YHR171W	7.80E-07
YER063W	2.13E-08
YJL092W	7.20E-07
YDR514C	1.11E-06
YOR205C	3.54E-08
YDR508C	6.17E-08
YGR082W	7.98E-09
YNL283C	6.61E-10
YBR202W	5.47E-07
YMR165C	4.31E-06
YBR146W	1.92E-10
YGR066C	5.32E-06
YBR199W	1.86E-12
YLR008C	6.43E-07
YMR118C	1.62E-07
YNL243W	1.73E-07
YBR114W	8.75E-07

YBL009W	7.36E-08
YNL066W	1.34E-15
YKL008C	4.61E-09
YBR132C	3.06E-16
YOR158W	3.77E-07
YDL024C	7.15E-06
YBR091C	3.57E-07
YLR353W	1.34E-10
YNR028W	5.26E-06
YJL145W	2.77E-08
YGR019W	4.81E-10
YIR027C	1.03E-11
YGR055W	4.11E-10
YNL268W	3.66E-10
YLR357W	7.16E-07
YDR275W	2.08E-07
YGL129C	2.89E-07
YDL239C	1.16E-10
YOL025W	1.06E-06
YNL139C	3.32E-06
YPL228W	7.35E-06
YNR032W	4.61E-06
YGL141W	1.18E-07
YNL185C	2.92E-07
YNL327W	1.36E-15
YDL142C	3.36E-06
YIR032C	2.79E-14
YIR029W	2.20E-12
YHR135C	1.10E-06
YMR305C	3.41E-10
YDR326C	1.27E-06
YDR131C	4.04E-08
YGR171C	1.26E-07
YJL183W	3.43E-06
YER019W	1.60E-07
YHR202W	9.64E-09
YMR194W	5.93E-06
YMR182W-A	9.15E-09
YOR354C	2.65E-07
YBR171W	2.03E-08
YBR103W	4.07E-12
YBR196C-B	1.33E-16
YIL093C	1.25E-07
YCR020C	7.15E-08
YOR386W	4.68E-07
YJL159W	4.02E-07
YJL023C	2.28E-06

YCR071C	1.55E-07
YHL038C	4.53E-07
YPR144C	7.84E-07
YBR159W	7.05E-07
YBR115C	4.19E-14
YOR127W	7.59E-09
YGR125W	6.14E-11
YDR507C	6.36E-11
YNL282W	2.43E-08
YGR040W	8.52E-13
YER153C	1.74E-13
YFL036W	4.31E-06
YMR155W	1.20E-07
YBR179C	2.36E-11
YDR462W	2.42E-08
YDL202W	3.71E-07
YPL171C	3.97E-06
YJL174W	9.86E-07
YDL169C	2.39E-07
YMR255W	1.24E-08
YLR209C	5.60E-08
YJL060W	5.37E-06
YIL024C	1.49E-08
YDR493W	6.41E-06
YLR229C	4.93E-10
YGR221C	1.72E-06
YDR260C	7.20E-07
YKL050C	2.82E-07
YOR296W	1.34E-11
YBR107C	2.15E-14
YMR001C	1.78E-07
YNL183C	8.22E-11
YJR092W	2.68E-10
YGR220C	4.18E-07
YMR073C	1.94E-06
YLR142W	4.51E-07
YDR194C	1.01E-08
YIL146C	4.16E-10
YNL117W	4.29E-07
YNR068C	2.12E-08
YPR089W	2.65E-06
YDL122W	1.73E-07
YML031W	4.05E-07
YGR016W	1.28E-06
YHR142W	2.04E-13
YBR130C	2.82E-07
YIL088C	2.60E-09

YLR084C	8.04E-08
YDR116C	2.80E-06
YNL239W	2.01E-10
YBR166C	4.61E-15
YPR159W	1.55E-08
YGL202W	5.77E-06
YPL087W	4.45E-06
YBR189W	6.01E-07
YPL163C	3.03E-11
YKL157W	2.30E-07
YGR013W	2.94E-07
YNL046W	1.22E-11
YPR040W	4.64E-07
YCR100C	4.49E-06
YLR097C	9.51E-07
YKR072C	1.43E-05
YJR036C	2.35E-06
YKL218C	7.38E-10
YJL077W-B	2.94E-08
YIL158W	7.93E-06
YDR409W	4.85E-07
YCL001W-B	3.06E-07
YKR006C	1.02E-06
YLR289W	1.55E-06
YKL181W	1.47E-07
YOR188W	4.60E-11
YDR464W	7.97E-06
YNL053W	1.29E-06
YGL209W	1.80E-06
YDL131W	2.89E-09
YCL014W	1.43E-08
YLR332W	2.92E-09
YPR133W-A	5.67E-08
YBL071C-B	2.35E-07
YJR005W	8.76E-08
YER075C	2.32E-08
YGL028C	2.36E-16
YGR154C	1.11E-06
YML068W	8.42E-07
YOL016C	9.84E-07
YPL040C	1.15E-06
YFL021W	3.55E-12
YGR229C	8.10E-07
YLR419W	1.02E-06
YOR140W	9.27E-10
YBR073W	5.23E-08
YJR102C	1.32E-06

YMR296C	4.23E-06
YGL064C	3.68E-09
YER001W	3.45E-07
YGR147C	1.39E-07
YLR439W	3.03E-07
YIL131C	1.24E-08
YNL173C	5.50E-07
YPR138C	1.43E-08
YBR170C	1.32E-12
YKR097W	2.08E-07
YBR148W	8.62E-15
YHR028C	4.35E-07
YLR250W	3.36E-06
YGL258W-A	6.11E-08
YOR230W	3.13E-06
YBR156C	6.28E-14
YER118C	6.85E-12
YNR069C	5.92E-08
YGL139W	2.80E-09
YMR174C	1.72E-06
YKL215C	5.45E-06
YOR111W	7.49E-08
YGR067C	4.15E-06
YKL182W	1.33E-06
YLR285C-A	1.01E-14
YHR107C	6.14E-09
YLR042C	1.45E-15
YMR129W	1.07E-06
YMR032W	5.37E-09
YJL062W-A	9.12E-06
YDL238C	7.81E-13
YJR043C	1.79E-07
YGR100W	3.89E-06
YBL002W	2.11E-06
YPL213W	9.00E-08
YNL040W	2.20E-07
YKL179C	4.40E-08
YIL035C	2.26E-06
YML109W	1.10E-08
YNL300W	4.21E-08
YLL063C	1.39E-06
YDR425W	4.22E-07
YJR006W	6.90E-07
YPR105C	2.65E-08
YNL078W	5.58E-14
YBR117C	1.68E-14
YBR204C	2.13E-11

YLR378C	1.15E-06
YBR038W	1.77E-09
YEL015W	4.74E-06
YNL099C	9.14E-08
YEL004W	6.43E-07
YMR200W	2.72E-10
YOL007C	8.10E-11
YNL289W	2.84E-08
YMR163C	3.50E-08
YNR040W	5.29E-07
YOR129C	5.10E-11
YHL004W	2.63E-06
YNL277W-A	1.10E-06
YCL064C	1.36E-06
YIL123W	2.80E-08
YBR139W	1.39E-07
YPL089C	2.25E-07
YPR074C	1.75E-06
YDL179W	7.04E-10
YDR022C	1.10E-06
YJR147W	5.58E-14
YPL158C	8.68E-11
YLR390W	4.03E-06
YDL210W	1.74E-13
YDR082W	1.82E-06
YLR080W	2.33E-08
YBR163W	4.12E-10
YER152C	4.71E-16
YOR315W	3.17E-10
YOR316C	3.62E-06
YHR059W	8.60E-08
YML009C	4.15E-06
YGR014W	7.04E-14
YDL078C	2.16E-06
YJR153W	1.07E-06
YBL085W	1.05E-07
YOL052C	3.36E-08
YJL160C	5.25E-07
YBR192W	3.59E-08
YGL105W	5.18E-07
YPL072W	2.62E-06
YJR101W	2.91E-06
YBR185C	8.74E-09
YFR028C	1.34E-07
YLR079W	1.72E-11
YBR098W	2.89E-11
YFL001W	6.61E-07

YOR247W	9.16E-15
YOR232W	3.13E-06
YPR194C	3.13E-06
YIR028W	2.21E-11
YLR258W	1.26E-09
YAL041W	1.41E-08
YBR193C	4.49E-13
YBR150C	1.28E-13
YMR024W	3.19E-07
YKL068W-A	3.36E-06
YOR342C	4.97E-14
YER096W	1.79E-07
YLR067C	1.20E-05
YLR190W	1.62E-07
YJL054W	1.25E-06
YDR333C	8.27E-08
YBR180W	8.12E-08
YAL024C	7.10E-10
YJL078C	5.19E-16
YDR249C	9.40E-07
YBR196C-A	1.61E-16
YJL110C	4.63E-12
YLR164W	6.08E-07
YGR134W	1.74E-06
YNL168C	4.58E-10
YPL123C	8.03E-07
YKR061W	1.12E-08
YBR176W	2.21E-11
YMR285C	3.98E-07
YKR013W	1.15E-10
YHR109W	5.02E-07
YDR242W	3.51E-11
YPL085W	3.50E-07
YHR143W	2.10E-16
YKR091W	1.03E-09
YLR053C	1.36E-07
YBR158W	4.72E-16
YIL149C	2.89E-07
YBR119W	4.30E-13
YKR005C	5.43E-07
YDR381W	1.25E-05
YHR099W	8.61E-07
YOR116C	8.53E-07
YJL053W	3.49E-06
YDR379W	5.87E-08
YPL082C	1.46E-08
YGR044C	1.93E-15

Hot Spot 3:90000

Accession ID	p-value
YOR271C	1.85E-13
YMR291W	3.62E-07
YCL018W	6.44E-16
YHR063C	3.24E-06
YER073W	1.04E-12
YKR071C	8.47E-12
YPL084W	9.95E-09
YFR005C	6.62E-06
YLR355C	2.21E-10
YJR016C	2.72E-11
YCL017C	4.58E-14
YLR348C	1.77E-14
YCR018C	3.69E-12
YBR068C	1.65E-13
YHR047C	4.24E-06
YNL241C	9.49E-07
YKR095W-A	1.21E-07
YOR226C	3.21E-14
YKL029C	4.96E-08
YCL026C-B	6.70E-15
YGL009C	3.90E-15
YOR227W	2.52E-08
YKL120W	6.36E-15
YOR375C	5.77E-13
YHR208W	3.90E-15
YCL004W	2.28E-11
YCL021W-A	3.21E-16
YCL016C	5.19E-13
YMR108W	2.46E-12

Hot Spot 7:410000

Accession ID	p-value
YER035W	7.68E-09
YJL196C	1.42E-06
YGL057C	1.10E-13
YPR174C	1.76E-14
YGL055W	1.90E-09
YGL063W	3.27E-11
YGL066W	3.04E-12
YJL144W	8.61E-14
YOL002C	4.33E-10
YGL050W	1.83E-06
YNL077W	1.08E-15
YNL006W	7.29E-11
YGL049C	1.82E-10
YGL053W	4.90E-17

YDR151C	5.59E-11
YFL016C	6.66E-09
YNL007C	7.81E-15
YOR027W	1.72E-12
YGL045W	7.26E-09
YPR158W	2.05E-12
YGR142W	3.20E-13
YGL035C	2.06E-09
YMR177W	1.26E-05
YDR492W	7.40E-10
YKR075C	7.42E-10
YBR101C	3.25E-14
YGL051W	4.90E-17

Hot Spot 8:110000

Accession ID	p-value
YHL010C	1.77E-14
YHR152W	1.02E-06
YFL047W	5.21E-08
YHR005C	1.63E-11
YHL020C	2.42E-09
YLR212C	3.49E-06
YML102W	4.52E-07
YLR433C	3.27E-06
YPL153C	2.30E-07
YMR274C	1.30E-07
YLR234W	1.94E-06
YMR179W	1.15E-07
YOL133W	1.88E-07
YHL006C	9.44E-11
YCL027W	8.33E-14
YBL016W	4.08E-11
YBR040W	3.36E-12
YHR201C	6.17E-08
YPR122W	8.10E-09
YHL009C	2.04E-10
YER016W	1.50E-08
YGR068C	3.43E-07
YPL192C	1.17E-10
YGR146C	2.49E-07
YOL017W	1.08E-05
YJL187C	3.27E-08
YMR236W	1.47E-07
YLL002W	2.70E-06
YGR227W	3.69E-07
YHL022C	4.56E-12
YHL012W	2.45E-12
YBR057C	3.41E-08

YPL124W	3.01E-06
YNR032W	1.15E-07
YGL061C	1.67E-08
YHL026C	1.22E-13
YER007W	2.67E-06
YNL201C	9.36E-07
YIL037C	1.47E-12
YLR442C	4.83E-06
YLR183C	5.29E-07
YMR288W	1.19E-10
YNL072W	2.92E-07
YCR089W	1.21E-12
YNL279W	1.50E-10
YBL052C	4.92E-08
YHR015W	1.39E-14
YNL326C	9.36E-08
YPR115W	2.64E-08
YHL008C	6.74E-13
YMR198W	3.89E-06
YHR007C	7.61E-08
YMR065W	2.31E-12
YDL003W	8.42E-07
YNL215W	8.21E-06
YHL009W-A	5.82E-10
YMR232W	3.81E-13
YBR070C	1.55E-06
YDR124W	8.62E-08
YKL189W	6.71E-10
YHR084W	7.84E-12
YKL101W	4.58E-07
YOR212W	4.11E-09
YGL060W	1.71E-11
YER155C	3.27E-09
YDR446W	5.00E-06
YDR410C	1.25E-07
YBR156C	4.79E-08
YML047C	2.48E-14
YNL278W	3.04E-07
YGL106W	1.20E-08
YNL300W	2.85E-09
YJL157C	8.71E-13
YHL016C	2.29E-13
YHL013C	1.48E-05
YLR452C	3.95E-14
YBR264C	1.43E-08
YOL007C	1.10E-07
YNL289W	5.09E-07

YGR047C	2.21E-07
YML062C	2.08E-06
YCL055W	9.15E-15
YPL156C	6.17E-08
YNL258C	1.27E-08
YDR085C	1.23E-06
YKR024C	3.71E-08
YOR219C	6.43E-07
YNR044W	1.11E-13
YNL042W-B	1.88E-07
YKR089C	6.55E-07
YBR083W	1.25E-11
YJR086W	2.49E-07
YHL003C	2.36E-12
YDR249C	1.86E-06
YHL009W-B	2.75E-09
YML046W	3.41E-13
YBR119W	1.25E-06
YBR133C	3.54E-07

Hot Spot 12:670000

Accession ID	p-value
YNR074C	1.22E-10
YOR175C	1.41E-08
YML019W	4.99E-08
YDL086W	3.36E-15
YMR212C	5.09E-09
YMR220W	2.41E-14
YBL031W	1.44E-06
YLR283W	2.76E-14
YDR518W	6.17E-10
YBR215W	4.81E-06
YDL236W	3.11E-08
YLR300W	3.68E-13
YEL051W	1.06E-06
YML125C	8.62E-08
YLR256W	8.81E-15
YBL095W	2.73E-07
YPR178W	5.43E-08
YOR321W	1.12E-07
YBR108W	2.91E-06
YLR248W	7.45E-07
YML007C-A	8.71E-06
YJL100W	1.95E-11
YGL001C	2.87E-14
YLR265C	4.84E-15
YNR043W	1.60E-14
YGR198W	2.68E-06

YPR062W	8.67E-07
YLR260W	6.55E-11
YOR003W	6.62E-11
YPL272C	1.42E-07
YKR088C	3.62E-07
YPR193C	2.56E-10
YLR237W	1.30E-11
YGR266W	3.41E-12
YER093C	4.99E-08
YFR033C	1.42E-10
YLR275W	6.21E-11
YML008C	1.62E-13
YMR298W	1.26E-07
YLR038C	1.21E-06
YIL121W	1.90E-16
YIL038C	1.32E-06
YJR134C	9.56E-07
YGL091C	5.13E-07
YHR072W	1.90E-14
YMR202W	1.52E-15
YDR200C	1.65E-06
YML075C	2.07E-15
YLR231C	6.62E-13
YLR214W	6.58E-08
YDR502C	1.42E-14
YLR234W	2.56E-10
YNL111C	1.03E-12
YGL055W	4.17E-10
YDR520C	1.50E-06
YBL067C	9.06E-09
YDR213W	7.57E-09
YGR089W	1.21E-08
YDR297W	6.14E-12
YPR151C	7.36E-15
YML032C	5.96E-06
YDL133W	7.50E-07
YDR186C	1.61E-08
YER024W	2.86E-06
YLR244C	7.18E-15
YEL047C	5.20E-08
YDR346C	9.64E-09
YGR026W	9.78E-07
YLR371W	4.77E-07
YOR073W	8.35E-07
YDR323C	5.44E-08
YMR208W	3.80E-15
YGL225W	8.05E-07

YEL034W	1.30E-15
YGL160W	7.39E-13
YPL263C	6.61E-06
YLR056W	1.31E-07
YLR246W	2.87E-15
YJL048C	6.49E-14
YDL093W	8.16E-11
YGL101W	3.60E-12
YBR067C	1.10E-10
YOR332W	1.61E-06
YLR253W	1.67E-06
YBR106W	5.93E-07
YBR286W	2.04E-09
YKL080W	3.15E-09
YLR213C	3.83E-09
YLR245C	7.31E-16
YPL170W	3.21E-07
YAL028W	2.90E-12
YOL128C	2.52E-07
YOR010C	3.69E-08
YHR003C	6.13E-09
YOR034C	9.00E-08
YJR113C	9.56E-07
YCR048W	3.84E-08
YGR281W	1.67E-06
YBR183W	1.56E-10
YOR002W	6.01E-07
YGL040C	9.66E-09
YLR205C	4.28E-15
YPR070W	3.68E-06
YKL004W	1.59E-06
YER014W	2.16E-13
YJR048W	3.03E-13
YJL212C	1.44E-07
YOL026C	7.36E-07
YML048W	1.14E-06
YKL008C	6.18E-09
YLR273C	4.91E-08
YHR008C	4.78E-08
YDR275W	9.50E-12
YBR005W	6.43E-08
YCR061W	2.10E-07
YOL045W	3.34E-06
YML028W	3.43E-07
YER122C	1.79E-08
YHL002W	4.07E-07
YDR326C	1.54E-07

YJR126C	2.62E-07
YNR041C	8.37E-09
YDL084W	1.54E-06
YNL052W	6.42E-07
YNR019W	3.57E-15
YIL154C	4.07E-07
YKL077W	9.78E-07
YKR046C	8.22E-16
YNR007C	3.27E-08
YJR104C	6.14E-10
YNL264C	1.14E-07
YKL105C	1.16E-07
YPL231W	4.94E-07
YMR223W	3.47E-07
YMR113W	7.78E-07
YLR153C	2.20E-15
YMR038C	1.35E-11
YBR042C	9.39E-08
YOR334W	6.63E-08
YPL028W	2.87E-14
YOL135C	1.16E-07
YKR051W	3.15E-07
YLR241W	9.37E-08
YBR182C-A	3.37E-07
YEL024W	5.00E-09
YNL003C	5.44E-10
YLR288C	1.40E-14
YDL178W	5.59E-07
YER043C	2.73E-09
YIL111W	1.37E-07
YGR169C-A	5.67E-08
YNL010W	6.40E-10
YBL098W	2.20E-06
YMR070W	1.06E-08
YHR190W	9.16E-14
YML126C	6.29E-16
YPR040W	1.39E-07
YGL191W	4.51E-10
YDR284C	8.28E-09
YKR030W	4.11E-06
YOR348C	4.17E-10
YLR100W	1.72E-13
YGL017W	6.05E-07
YBL043W	4.99E-08
YBR161W	1.15E-07
YDR011W	4.77E-06
YLR399C	1.20E-07

YPL232W	4.97E-07
YMR100W	1.69E-08
YOR065W	1.65E-08
YHR179W	5.85E-10
YNL173C	5.38E-07
YBR085W	5.00E-07
YIL031W	4.38E-08
YKL182W	6.03E-07
YKL145W	4.49E-06
YLR154C	3.62E-11
YPR095C	1.30E-09
YLR380W	7.80E-07
YIL007C	2.30E-09
YPR113W	3.66E-08
YDR218C	1.51E-09
YHR151C	2.62E-06
YPL008W	4.11E-06
YHR039C	1.88E-12
YJL198W	3.13E-06
YNL151C	1.83E-09
YGR138C	2.18E-06
YDR488C	1.01E-06
YER141W	4.64E-06
YGR234W	8.22E-16
YNL280C	3.83E-09
YDR384C	2.43E-08
YAR003W	1.04E-06
YPL229W	1.13E-06
YNR060W	1.71E-08
YPR065W	4.59E-13
YJR051W	1.66E-07
YMR009W	3.63E-14
YOR237W	6.34E-11
YMR244W	1.81E-10
YMR134W	5.66E-16
YHR075C	6.54E-07
YMR145C	1.29E-07
YOR270C	1.20E-07
YKL150W	2.00E-06
YCR069W	1.28E-11
YGL210W	1.65E-06
YML081W	5.36E-06
YNL156C	2.14E-14
YPL117C	1.88E-12
YHL031C	2.60E-06
YER011W	1.91E-09
YJR105W	1.67E-11

YHR004C	6.71E-08
YDL100C	3.22E-06
YDL174C	4.74E-13
YDR044W	3.44E-11
YER044C	1.69E-14
YLR425W	3.47E-06
YAL016W	4.85E-07
YGL056C	8.35E-08
YJR064W	4.77E-07
YJR047C	2.26E-08
YAL039C	8.35E-07
YJL105W	4.57E-08
YLR272C	7.32E-09
YGR007W	3.08E-07
YGR049W	2.56E-13
YOR377W	4.01E-11
YNL159C	1.10E-06
YLL012W	5.36E-09
YMR119W	7.87E-07
YOR118W	2.48E-08
YGR060W	9.88E-12
YBR242W	1.15E-13
YHR048W	1.65E-10
YER053C-A	6.53E-15
YJL167W	6.15E-15
YMR015C	2.87E-14
YGL144C	5.14E-07
YMR252C	1.54E-07
YNL158W	2.96E-07
YOR229W	1.18E-09

Hot Spot 13:50000

Accession ID	p-value
YDR481C	2.02E-14
YML113W	2.74E-07
YHR152W	1.98E-07
YJL186W	2.59E-08
YER053C	1.60E-06
YLR089C	1.22E-07
YBR296C	2.45E-14
YML064C	6.04E-08
YHR124W	7.07E-07
YOL011W	1.27E-06
YBR295W	3.05E-07
YML091C	3.64E-10
YHR097C	3.36E-06
YLL049W	8.35E-07
YML093W	2.09E-07

YJL088W	2.20E-08
YER013W	1.51E-05
YML067C	6.70E-08
YHL018W	1.91E-06
YER123W	8.82E-07
YML123C	5.06E-16
YBL068W	1.03E-06
YIL073C	3.45E-07
YNL217W	3.47E-08
YOL064C	1.25E-08
YLR267W	1.88E-06
YPL019C	1.89E-15
YGR170W	1.66E-07
YOR213C	2.37E-06
YLR410W	1.91E-13
YML096W	1.10E-10
YAR008W	6.17E-07
YFL004W	1.51E-14
YGR233C	1.15E-15
YBL037W	1.43E-06
YJL137C	6.11E-07
YHR103W	6.43E-07
YLR108C	4.44E-07
YOL140W	6.98E-08
YML118W	1.38E-07
YNL224C	8.65E-07
YML098W	3.75E-06
YDR369C	3.87E-08
YPR086W	3.81E-08
YOL130W	2.60E-08
YPL170W	4.05E-06
YML101C	1.48E-06
YPR013C	9.24E-07
YML078W	7.11E-16
YIR002C	1.85E-06
YDR130C	1.27E-07
YOL069W	1.38E-07
YHR214C-E	4.02E-10
YFR044C	4.31E-08
YML087C	1.80E-10
YOR221C	5.14E-07
YAR007C	3.99E-06
YCR037C	9.38E-13
YGL224C	6.26E-12
YIL074C	6.01E-07
YJL012C	8.38E-16
YBR105C	2.18E-06

YER055C	1.98E-07
YNL267W	7.20E-07
YOR007C	7.56E-06
YLR355C	2.21E-10
YCL049C	7.07E-08
YIR027C	1.37E-06
YDR017C	4.30E-06
YDR309C	7.25E-09
YDR248C	1.73E-07
YHR117W	7.91E-08
YOR066W	8.61E-08
YML076C	1.73E-13
YML066C	2.58E-15
YEL032W	1.31E-07
YNL148C	3.08E-08
YHR183W	3.58E-10
YJL020C	4.41E-07
YGL223C	2.90E-06
YML116W	5.33E-11
YIL172C	1.22E-06
YHR136C	1.47E-15
YJL117W	4.43E-14
YBL008W	4.74E-07
YLR438W	8.49E-15
YCR004C	4.23E-06
YER060W-A	2.50E-06
YLR142W	1.30E-08
YOR163W	1.04E-11
YMR300C	2.87E-09
YNR068C	7.88E-08
YNL025C	1.59E-06
YIL111W	4.20E-06
YHR047C	1.25E-07
YPL268W	1.83E-12
YHR162W	8.62E-10
YBR125C	4.94E-07
YLR058C	7.79E-07
YML111W	5.74E-13
YDR019C	9.20E-07
YOR374W	9.99E-11
YOR211C	1.78E-06
YMR296C	1.99E-06
YML088W	1.98E-07
YIL051C	8.05E-16
YAR071W	1.05E-14
YDR229W	2.44E-07
YML119W	5.68E-10

YPL110C	4.70E-14
YKR093W	3.30E-08
YER057C	2.10E-08
YGR243W	8.82E-07
YKL078W	1.67E-06
YCL025C	7.36E-09
YMR120C	4.76E-08
YML085C	1.38E-14
YGL234W	3.67E-07
YPL061W	2.17E-12
YKL016C	1.66E-06
YCL064C	2.04E-16
YNL012W	6.60E-08
YAL047C	4.44E-06
YPL111W	1.86E-14
YML121W	4.41E-12
YNR002C	2.22E-08
YKL051W	1.94E-12
YKR089C	4.67E-07
YBR208C	4.01E-08
YMR313C	4.53E-07
YML104C	2.72E-10
YER072W	2.01E-15
YML079W	5.18E-11
YPL018W	8.29E-14
YGL167C	6.39E-07
YDR482C	2.85E-12
YER034W	1.70E-06
YNL141W	1.94E-07
YIL120W	2.11E-06
YEL071W	3.08E-06
YGL255W	1.62E-08
YBR093C	2.14E-15
YGR061C	1.15E-09
YLR126C	7.99E-06
YMR238W	9.74E-08
YFR006W	1.41E-06
YDL013W	8.03E-07
YFR031C	6.60E-07
YDR281C	6.10E-15
YPL113C	2.41E-07
YML029W	3.02E-07

Hot Spot 15:150000

Accession ID	p-value
YKR009C	1.48E-08
YML113W	1.08E-09
YIL157C	2.68E-09

YPL223C	6.20E-12
YKL138C	1.22E-07
YIL160C	4.09E-09
YDL048C	8.74E-09
YNL083W	5.66E-08
YPL154C	3.81E-08
YLR401C	2.78E-06
YKL195W	3.49E-09
YDR144C	7.68E-06
YBR149W	2.98E-09
YER035W	2.74E-07
YHR002W	1.31E-06
YPL020C	5.37E-07
YJL185C	2.95E-08
YIL125W	8.11E-08
YGR130C	2.03E-10
YDR447C	7.20E-07
YNR038W	5.63E-07
YKL096W	2.75E-11
YEL003W	3.49E-06
YHR198C	7.40E-10
YLR149C	1.18E-10
YOR271C	9.57E-08
YHR197W	2.71E-08
YAL034C	2.60E-08
YOR084W	6.32E-08
YJL042W	4.25E-09
YDR245W	1.04E-07
YOR187W	1.49E-09
YGL121C	8.83E-07
YBR285W	6.86E-08
YOL028C	4.76E-09
YGR110W	8.04E-07
YKL192C	4.64E-08
YBR169C	1.17E-07
YKL167C	5.59E-09
YOL122C	1.86E-08
YER053C	5.12E-09
YOL105C	4.30E-13
YCL054W	1.35E-07
YOL077W-A	9.95E-09
YGR083C	2.71E-08
YBR084C-A	7.66E-07
YDR231C	3.59E-08
YMR169C	7.48E-12
YOL071W	2.19E-08
YKL194C	2.84E-09

YHL032C	2.39E-12
YPL127C	8.28E-07
YBR142W	4.66E-06
YBR295W	4.34E-09
YKL085W	7.32E-09
YMR128W	2.55E-06
YPR155C	1.93E-10
YMR131C	1.56E-07
YCR094W	2.71E-07
YDR076W	2.55E-07
YKR092C	2.37E-06
YGR088W	2.60E-10
YMR135C	1.54E-11
YDL085W	1.22E-07
YPR184W	6.43E-10
YPL078C	7.34E-08
YDL223C	6.43E-10
YDR516C	1.13E-07
YOL090W	9.70E-11
YOL036W	8.81E-08
YOL098C	1.52E-07
YMR008C	5.86E-07
YDR079W	1.63E-07
YDR454C	6.47E-08
YML125C	1.44E-06
YHL001W	2.14E-06
YDR062W	6.04E-07
YOR178C	2.98E-09
YDR361C	4.91E-08
YHR097C	1.88E-09
YGL253W	7.80E-07
YGR258C	8.64E-09
YDL229W	1.99E-06
YHR024C	6.09E-06
YJL205C	3.25E-07
YLR052W	5.01E-07
YNL011C	1.65E-07
YJR121W	7.00E-09
YKL180W	4.63E-06
YMR301C	3.86E-06
YNR020C	2.21E-06
YEL036C	5.16E-07
YCR059C	1.56E-09
YML093W	8.55E-06
YHR087W	5.07E-10
YHR038W	3.68E-07
YLL028W	4.59E-16

YOR302W	2.14E-06
YCR073C	9.72E-11
YOL109W	3.02E-10
YDR530C	6.60E-07
YKR056W	3.99E-06
YER013W	4.04E-10
YGL013C	4.06E-07
YPR015C	6.80E-07
YBL072C	5.07E-06
YDR001C	3.62E-07
YMR280C	4.09E-09
YJL148W	2.34E-07
YLR218C	2.02E-10
YJR032W	1.80E-09
YBL027W	3.46E-06
YNL116W	5.87E-06
YDL112W	5.35E-07
YGR272C	3.36E-08
YJL158C	8.40E-07
YBR251W	2.83E-08
YKR044W	2.95E-08
YFR045W	1.84E-11
YPL017C	1.36E-09
YKL035W	8.36E-09
YOL061W	2.60E-08
YBL068W	2.72E-10
YDL199C	7.32E-09
YFL030W	1.11E-09
YKL162C	2.89E-07
YMR282C	4.85E-07
YAL017W	4.48E-09
YOL097C	8.14E-10
YHR129C	4.23E-07
YDL148C	7.15E-07
YOR228C	6.10E-07
YCR091W	9.55E-09
YKL037W	2.37E-09
YDR325W	9.00E-06
YPL045W	1.26E-06
YMR239C	3.81E-08
YLR363W-A	1.30E-08
YHR070W	2.18E-06
YIL096C	4.64E-08
YDR175C	1.07E-06
YMR025W	9.44E-08
YER176W	2.41E-07
YDL060W	1.68E-06

YJL063C	9.57E-08
YDR234W	1.09E-08
YDL222C	1.22E-11
YER093C-A	9.98E-09
YER170W	1.87E-06
YOL120C	2.93E-06
YHR051W	2.50E-07
YKL022C	1.95E-06
YDL031W	2.85E-07
YDR337W	5.35E-08
YML054C	4.34E-09
YGL111W	1.33E-07
YLR249W	9.43E-08
YBR023C	1.81E-07
YLR224W	8.18E-08
YNL217W	3.81E-08
YKL121W	2.49E-08
YDR349C	5.60E-09
YGL096W	8.08E-07
YKR049C	1.64E-09
YKR043C	9.25E-11
YGR053C	4.71E-08
YDR347W	1.52E-07
YOR246C	7.54E-09
YLR222C	3.92E-07
YER006W	1.68E-06
YCL059C	8.18E-07
YGR166W	5.12E-09
YDL046W	4.23E-07
YMR037C	4.12E-06
YHR085W	2.10E-08
YHR062C	1.55E-08
YHR139C	1.56E-06
YLR437C	3.10E-06
YOR353C	2.04E-07
YDR490C	4.15E-08
YFR039C	2.52E-06
YOL077C	4.90E-09
YPR140W	3.29E-08
YBR131W	9.97E-08
YPR010C	9.24E-08
YMR010W	1.04E-07
YDR528W	5.16E-07
YPR043W	2.09E-07
YOR349W	1.79E-07
YPL132W	8.38E-11
YER088C	5.62E-11

YER101C	9.90E-09
YNL113W	6.06E-08
YDR395W	3.76E-07
YOL104C	5.29E-16
YKL161C	3.48E-07
YDR453C	2.98E-09
YPR191W	3.28E-07
YML096W	2.67E-07
YER150W	2.64E-11
YER066W	1.19E-06
YAL053W	3.35E-07
YJL208C	1.33E-06
YBR175W	9.17E-07
YNR037C	2.67E-07
YLR172C	1.50E-07
YDL130W-A	1.42E-08
YLR356W	9.18E-08
YER054C	3.14E-10
YOL022C	5.86E-09
YNL045W	2.16E-09
YPR118W	2.49E-08
YCR057C	8.81E-08
YHR143W-A	1.43E-07
YML025C	2.01E-07
YIL091C	2.28E-08
YAL005C	3.13E-07
YFL017C	2.13E-07
YAL029C	1.34E-07
YJL131C	4.90E-09
YNL160W	3.26E-09
YKL072W	2.16E-09
YMR116C	4.95E-07
YJL164C	4.66E-09
YLR204W	1.71E-08
YMR196W	4.39E-10
YBR111C	2.99E-06
YGR031W	2.54E-09
YKL124W	1.46E-07
YPR060C	1.58E-08
YIR012W	2.10E-08
YKL084W	7.16E-08
YGR201C	3.14E-10
YPL043W	8.27E-07
YLR270W	2.24E-10
YIL070C	4.18E-07
YKL091C	2.13E-10
YKR016W	1.31E-07

YDL235C	1.99E-07
YEL011W	4.66E-09
YDR418W	5.35E-06
YPL196W	1.55E-08
YJR055W	3.36E-08
YAL002W	9.97E-08
YIL110W	2.16E-07
YJR110W	2.22E-06
YGR033C	1.23E-08
YHR030C	2.07E-08
YDR352W	2.11E-07
YKL018C-A	1.33E-06
YGR217W	1.86E-08
YJR002W	2.71E-06
YML030W	2.84E-09
YKL148C	5.60E-07
YDR178W	7.66E-09
YDR202C	4.55E-09
YJL137C	3.44E-08
YJR039W	1.58E-06
YNL002C	2.25E-07
YKL067W	9.18E-08
YIR037W	1.69E-07
YGR216C	1.37E-06
YPR091C	1.14E-08
YJR097W	1.62E-08
YMR251W-A	3.14E-09
YHR207C	7.32E-09
YGR264C	1.64E-08
YJR059W	1.38E-07
YML070W	1.56E-07
YGR175C	5.95E-08
YKR026C	3.81E-08
YDR211W	5.36E-09
YIL034C	1.07E-06
YCL035C	4.91E-08
YOL006C	5.05E-09
YLR251W	8.45E-08
YDL181W	5.86E-09
YOR195W	5.27E-07
YDR096W	1.80E-09
YKL062W	5.75E-12
YML108W	4.33E-08
YEL050C	4.52E-08
YDR504C	5.20E-07
YNL087W	9.98E-09
YDR405W	1.17E-06

YEL046C	1.86E-07
YIL046W	8.18E-07
YBR106W	1.02E-06
YIL105C	1.92E-08
YNL015W	3.08E-08
YJL163C	1.11E-07
YKL193C	1.71E-08
YNL023C	3.41E-07
YNL108C	1.08E-07
YLR397C	1.07E-06
YHR176W	2.60E-08
YLR048W	1.47E-06
YGR146C	2.25E-06
YOL130W	9.46E-09
YLR226W	5.95E-08
YNL306W	2.17E-08
YBR262C	1.23E-06
YIL030C	1.27E-06
YKR063C	2.12E-09
YOR120W	3.74E-09
YJR008W	1.55E-07
YNR012W	2.10E-08
YBL013W	2.48E-10
YBL024W	8.75E-07
YNR034W	3.07E-07
YHR156C	6.06E-08
YBR269C	1.38E-07
YEL052W	1.18E-09
YEL055C	1.09E-08
YDL213C	9.14E-09
YGR052W	3.37E-13
YGL004C	7.24E-09
YDR430C	2.26E-09
YIL108W	7.73E-07
YEL029C	3.44E-08
YBR003W	1.24E-08
YMR304W	1.06E-06
YDL117W	1.45E-06
YOR306C	7.29E-07
YOR040W	3.01E-07
YDR165W	1.50E-07
YMR229C	4.91E-08
YBR081C	3.48E-07
YNL132W	3.21E-07
YMR264W	7.52E-08
YDR038C	5.29E-07
YLR069C	2.19E-08

YLR295C	1.41E-08
YPL173W	3.09E-07
YBR259W	2.16E-07
YHR077C	7.21E-08
YPR047W	2.40E-06
YGR008C	1.24E-08
YHR001W-A	1.66E-06
YDR060W	2.43E-08
YIL079C	4.07E-07
YPL118W	7.12E-08
YKL212W	4.66E-08
YJL014W	4.66E-08
YPR110C	4.15E-08
YLR014C	6.35E-07
YEL026W	6.87E-08
YKL004W	1.18E-06
YBR037C	1.55E-08
YFR044C	5.29E-07
YOL041C	3.34E-07
YDR032C	1.84E-08
YER165W	2.03E-06
YFR011C	4.04E-08
YKL100C	1.04E-08
YIL117C	2.84E-07
YLR382C	2.95E-08
YDR398W	8.80E-06
YFR017C	8.14E-10
YER182W	4.61E-10
YML129C	2.60E-09
YPL183W-A	3.66E-08
YER162C	1.76E-08
YOR086C	7.66E-09
YMR185W	3.62E-07
YBR004C	3.10E-06
YOL080C	1.18E-09
YBR230C	5.47E-09
YOL081W	1.05E-09
YNL098C	2.21E-09
YGR112W	4.68E-09
YDR300C	2.16E-09
YMR107W	9.97E-08
YKL096W-A	1.51E-10
YDR313C	3.01E-07
YNL175C	3.81E-08
YJL141C	5.83E-09
YGR082W	1.22E-07
YDL004W	1.28E-07

YPL030W	1.21E-09
YBR146W	3.70E-07
YKR065C	1.64E-08
YEL060C	2.80E-06
YOR004W	1.24E-06
YGR086C	1.84E-11
YMR261C	1.80E-09
YJL103C	9.14E-09
YPL207W	1.93E-10
YLR446W	1.63E-07
YIR016W	4.39E-10
YOL084W	2.88E-15
YPL239W	1.49E-09
YFR055W	1.12E-07
YFR014C	1.31E-10
YHR195W	8.45E-08
YHL036W	6.87E-08
YIL097W	2.70E-06
YNR036C	4.64E-08
YGR076C	4.57E-08
YMR114C	6.53E-08
YKR060W	1.62E-08
YLR193C	8.11E-08
YPL175W	1.31E-10
YOR158W	1.76E-07
YOR303W	1.16E-07
YOL131W	1.26E-08
YLL019C	4.09E-09
YLR070C	2.32E-08
YLR395C	8.28E-07
YIL127C	3.48E-07
YLL008W	1.19E-08
YNL093W	4.55E-09
YMR175W	3.98E-08
YLR002C	2.64E-07
YDR098C	3.29E-09
YIL047C	5.01E-08
YMR031C	2.81E-12
YLR345W	2.27E-12
YCR030C	1.30E-08
YOR262W	2.30E-07
YOL092W	2.02E-15
YDR148C	1.36E-08
YNL195C	2.26E-09
YPL003W	8.37E-07
YGR111W	1.04E-07
YLR028C	1.83E-06

YMR290C	8.11E-08
YDR221W	3.52E-06
YCL057C-A	5.57E-08
YNL185C	1.33E-07
YDR303C	2.77E-08
YDR436W	8.74E-09
YOR310C	1.22E-07
YOL048C	2.42E-08
YDR185C	3.75E-08
YOL052C-A	3.14E-10
YLR392C	1.70E-08
YER002W	3.34E-07
YJL039C	4.15E-07
YGL187C	8.28E-07
YLR177W	1.13E-09
YMR110C	3.01E-07
YBL035C	1.99E-07
YJL066C	9.82E-10
YMR250W	6.13E-10
YKL099C	4.50E-06
YMR214W	5.97E-06
YNR033W	1.30E-08
YGR248W	1.76E-08
YLR196W	1.36E-06
YGL018C	2.97E-07
YLR034C	3.52E-06
YLL026W	3.57E-09
YFR007W	3.22E-08
YNL200C	1.72E-09
YOR354C	1.39E-07
YNL100W	4.15E-09
YHR020W	3.59E-08
YLR023C	3.67E-07
YPL108W	7.32E-09
YIL093C	2.44E-06
YER045C	1.09E-07
YNL308C	2.36E-06
YDR533C	1.02E-15
YLR168C	1.18E-06
YDL214C	4.15E-08
YML100W	1.75E-10
YPL012W	3.62E-07
YGR043C	1.05E-11
YOR184W	1.03E-06
YNR007C	1.09E-06
YOR386W	1.96E-07
YLR277C	5.69E-07

YLR194C	9.17E-07
YIL033C	4.64E-10
YPL011C	1.23E-07
YMR148W	1.68E-09
YNL284C	1.34E-06
YLR102C	1.03E-08
YGL078C	6.87E-07
YOR107W	2.58E-07
YCR016W	6.40E-07
YBR214W	4.33E-08
YPL183C	7.20E-07
YML056C	1.11E-07
YLL011W	4.86E-07
YGR187C	1.03E-09
YML128C	3.63E-10
YLL018C-A	2.92E-11
YJR003C	4.39E-10
YHR110W	2.53E-07
YPR061C	7.96E-06
YGR128C	7.32E-09
YGR095C	1.24E-07
YOR173W	2.72E-10
YOR207C	2.38E-08
YDL203C	1.28E-06
YBR155W	2.49E-08
YDR351W	1.14E-07
YKL170W	7.37E-07
YFL036W	1.28E-07
YAL008W	1.50E-06
YPL266W	8.11E-08
YHR068W	2.06E-07
YBL045C	1.26E-06
YDR462W	1.57E-07
YPL230W	5.36E-09
YLR203C	7.40E-10
YBL064C	9.14E-08
YHR187W	1.17E-07
YOR233W	6.87E-08
YPL171C	2.86E-09
YGL120C	4.76E-07
YGR271C-A	2.18E-08
YFR013W	1.23E-06
YKL046C	1.22E-07
YBR122C	4.23E-06
YKL109W	1.93E-09
YLR301W	5.86E-07
YDL051W	3.08E-08

YDL208W	5.86E-09
YDR047W	2.21E-09
YHR007C	5.92E-07
YCR004C	1.64E-08
YDR161W	1.50E-07
YOR267C	3.50E-08
YCR051W	9.57E-08
YLL035W	2.38E-08
YGL226W	2.27E-07
YDR342C	1.75E-10
YEL024W	1.13E-06
YJR132W	1.09E-07
YBL039C	2.65E-06
YPL109C	2.84E-07
YOL034W	1.33E-07
YPR161C	2.76E-08
YJL098W	6.75E-07
YHR009C	2.07E-07
YLL001W	4.48E-09
YDL022W	1.64E-09
YIL124W	9.14E-09
YML120C	4.15E-08
YDL183C	4.99E-08
YDL019C	6.18E-13
YMR308C	6.35E-07
YFL048C	1.19E-06
YOR243C	1.33E-07
YPL172C	8.03E-07
YLR197W	5.06E-07
YOR113W	1.08E-07
YOL119C	4.74E-10
YJL210W	8.64E-09
YOR260W	3.48E-07
YNL194C	3.14E-10
YML071C	1.57E-07
YMR272C	3.72E-12
YKL026C	2.73E-09
YIL099W	1.14E-06
YMR293C	5.55E-06
YLR084C	1.29E-07
YPL098C	6.27E-07
YMR090W	3.26E-09
YHL039W	6.50E-07
YIL077C	8.94E-10
YMR002W	1.04E-06
YMR136W	2.33E-11
YDR116C	7.06E-07

YMR041C	1.86E-08
YIL136W	3.29E-10
YPL271W	3.21E-07
YBR039W	4.50E-06
YFR053C	9.55E-09
YOR317W	8.70E-07
YGL146C	1.09E-08
YDR087C	1.20E-06
YER110C	9.24E-08
YPL160W	1.70E-07
YHR100C	2.34E-07
YBR001C	2.75E-08
YIL056W	1.58E-07
YOR358W	4.71E-08
YHL011C	3.08E-08
YLR409C	2.28E-08
YJR077C	1.24E-08
YGL236C	4.25E-06
YAL061W	2.84E-07
YDR358W	2.43E-07
YJR036C	6.35E-07
YPR030W	4.08E-08
YDR216W	5.60E-11
YDL110C	1.24E-09
YKL151C	1.36E-08
YDR204W	1.72E-09
YML081C-A	2.50E-07
YPL053C	1.22E-07
YPL222W	4.28E-09
YFL016C	8.41E-06
YLR090W	3.29E-06
YOR348C	6.35E-07
YPL247C	6.87E-11
YDR256C	4.86E-09
YKR006C	7.12E-08
YLR143W	2.28E-08
YLR289W	1.18E-06
YDR021W	3.21E-07
YBL099W	3.81E-08
YDL036C	5.45E-08
YER074W	2.78E-06
YFR031C-A	2.27E-07
YOL113W	4.71E-10
YMR267W	5.36E-10
YMR181C	2.13E-10
YOR208W	3.93E-07
YHL033C	1.15E-06

YMR093W	3.48E-07
YDL021W	2.23E-08
YDR074W	1.51E-10
YDR171W	2.98E-09
YBR120C	1.24E-06
YKL093W	9.90E-07
YLR332W	2.96E-07
YKL142W	5.36E-09
YAL040C	3.08E-08
YJR005W	1.29E-07
YJR041C	2.48E-09
YOR374W	2.38E-08
YOL095C	5.47E-11
YNL247W	2.43E-07
YKR058W	3.81E-08
YFL042C	3.09E-07
YGR236C	2.32E-08
YPR018W	2.43E-07
YPL040C	3.41E-09
YGL115W	3.62E-07
YOL029C	5.31E-10
YER056C-A	2.22E-06
YLR419W	8.49E-08
YLR449W	5.86E-09
YIR038C	3.50E-08
YBR261C	8.19E-08
YPL104W	1.88E-09
YOR048C	1.87E-09
YPR172W	7.84E-08
YHR080C	6.56E-11
YML088W	5.78E-07
YPR008W	2.19E-08
YLR146C	1.84E-08
YNL131W	8.21E-07
YOR286W	1.93E-07
YOL056W	7.67E-09
YGL037C	2.48E-09
YER001W	1.36E-08
YNL255C	5.55E-06
YPL052W	1.18E-09
YLL041C	3.08E-08
YGR237C	8.06E-08
YDR513W	6.01E-07
YLR439W	9.18E-08
YHR179W	2.96E-08
YPR026W	8.37E-09
YHR168W	6.25E-07

YML073C	3.66E-06
YNL274C	1.43E-09
YGL227W	2.34E-07
YLR228C	3.09E-07
YKL052C	5.21E-14
YJR080C	1.36E-09
YOL089C	2.02E-15
YBR052C	1.69E-08
YMR312W	5.57E-10
YJL010C	1.09E-08
YNL134C	6.90E-14
YGR127W	1.09E-08
YBL087C	4.77E-07
YGR174C	1.18E-09
YFR052W	1.63E-07
YJR019C	4.32E-07
YOL147C	1.65E-07
YBL071W-A	9.18E-08
YHR102W	6.09E-06
YHR025W	6.66E-07
YKL141W	1.24E-08
YNL137C	6.77E-07
YDR138W	6.54E-06
YPL110C	2.71E-06
YKR093W	2.70E-09
YHR088W	4.71E-08
YMR174C	2.74E-07
YDR173C	1.67E-06
YLR219W	8.48E-12
YCR034W	1.36E-06
YNL227C	4.23E-07
YER130C	2.19E-06
YOR215C	3.48E-07
YOL091W	1.15E-16
YOR220W	4.15E-07
YIL067C	1.62E-06
YHR010W	6.40E-06
YIL155C	2.37E-09
YGR243W	2.04E-10
YGR077C	1.58E-07
YBR034C	8.81E-08
YPL075W	7.77E-07
YJL005W	3.66E-08
YER082C	1.56E-09
YML018C	1.84E-07
YLL021W	6.01E-07
YKL216W	1.91E-07

YLR129W	5.86E-07
YOL116W	3.99E-09
YMR295C	1.06E-07
YCR020C-A	3.37E-06
YIL101C	4.71E-08
YMR032W	7.75E-08
YLR186W	7.02E-07
YKL087C	3.22E-08
YNL223W	3.48E-07
YER067W	1.76E-07
YER049W	1.64E-09
YOL073C	3.66E-08
YOR337W	3.62E-07
YJL062W-A	1.10E-08
YHR210C	3.02E-07
YER107C	8.11E-08
YGR097W	1.62E-06
YBL030C	1.63E-08
YOR227W	2.99E-10
YJR127C	4.57E-11
YCR046C	1.08E-09
YDR277C	1.55E-08
YMR194C-B	1.83E-12
YNL151C	2.14E-06
YPL096W	8.09E-07
YOR096W	3.10E-06
YHR016C	5.12E-09
YOR142W	7.80E-08
YLR163C	2.08E-06
YPL157W	2.63E-06
YDR091C	2.04E-07
YKR076W	3.91E-09
YKR064W	4.68E-07
YGR021W	9.55E-09
YGR200C	3.76E-07
YHR064C	8.03E-07
YNL092W	1.91E-07
YMR297W	1.56E-07
YHR017W	2.83E-08
YMR157C	5.00E-08
YNL133C	1.12E-09
YDR034W-B	4.15E-08
YHR022C	7.26E-07
YDR296W	2.01E-08
YOR241W	7.32E-09
YOR001W	2.53E-08
YOR161C	1.67E-10

YMR286W	3.34E-08
YLR327C	1.14E-08
YOR224C	3.50E-08
YAR035W	6.60E-07
YCR043C	1.62E-08
YMR177W	8.00E-09
YOR274W	1.99E-07
YIL087C	4.00E-07
YGR244C	8.66E-11
YPR137W	9.18E-08
YIL008W	3.69E-07
YGL010W	3.52E-07
YJR034W	4.21E-10
YBL086C	1.24E-08
YNR040W	3.79E-08
YHR111W	9.82E-10
YIL114C	1.11E-06
YIL039W	4.67E-06
YNR046W	5.55E-06
YPL088W	3.98E-08
YHL004W	4.15E-08
YKL016C	1.30E-07
YJR063W	2.43E-07
YKR079C	2.47E-10
YJL129C	2.68E-07
YPL089C	4.43E-07
YBR162W-A	4.50E-07
YBR007C	6.59E-08
YOL054W	2.49E-08
YML050W	3.51E-08
YDR420W	2.04E-07
YGL145W	2.10E-08
YPL219W	8.60E-07
YKL150W	1.42E-08
YIL098C	1.61E-07
YGR194C	1.97E-09
YGR285C	9.95E-07
YLR390W	2.89E-08
YLL034C	1.28E-07
YML110C	4.87E-09
YHR096C	4.23E-10
YDR251W	2.25E-07
YPR033C	3.15E-08
YNR002C	1.64E-09
YDR197W	1.39E-09
YDR003W	1.22E-06
YAR029W	6.40E-07

YGR035C	1.69E-07
YIL036W	4.15E-08
YHR059W	1.76E-07
YKL051W	2.06E-09
YML009C	1.95E-06
YOR185C	4.09E-09
YNL234W	4.02E-09
YHR144C	2.71E-08
YOL094C	4.87E-12
YHR116W	4.09E-09
YLR106C	2.64E-07
YDR198C	1.09E-08
YJL011C	2.91E-07
YDR258C	2.97E-07
YMR105C	2.72E-09
YJR096W	3.70E-09
YKL014C	7.29E-07
YNL294C	3.62E-07
YDR406W	3.66E-10
YBR185C	4.52E-08
YDR324C	7.66E-09
YER062C	3.02E-11
YGR023W	5.57E-08
YJR011C	1.19E-07
YPL186C	9.37E-10
YJL069C	2.22E-07
YGL025C	5.35E-06
YLR258W	2.38E-08
YCR072C	9.05E-07
YGL130W	2.64E-07
YMR030W	3.08E-06
YCL040W	3.81E-08
YOL088C	4.64E-10
YBL061C	8.14E-10
YNL310C	9.07E-07
YDR329C	6.86E-08
YER060W	5.01E-07
YGL237C	1.00E-08
YDL204W	1.84E-10
YHR104W	3.63E-10
YBR177C	5.32E-11
YCR079W	1.53E-06
YPR143W	4.00E-07
YHR147C	4.98E-07
YOL100W	2.16E-11
YNL256W	3.36E-08
YBR147W	6.87E-07

YDL027C	5.69E-07
YBR126C	5.84E-10
YDL124W	4.53E-08
YDR135C	2.40E-07
YGR195W	4.98E-07
YNR014W	4.07E-12
YGL151W	4.27E-07
YGR231C	5.59E-09
YLR178C	1.84E-11
YEL040W	1.87E-06
YOL156W	8.95E-07
YNL075W	3.62E-07
YLR083C	9.29E-07
YHR160C	4.90E-09
YGR207C	1.15E-09
YDR070C	5.07E-10
YDL075W	9.45E-07
YBR092C	8.68E-08
YBR030W	9.53E-07
YCR028C	6.56E-08
YJL112W	6.99E-09
YDR115W	1.53E-06
YIL020C	1.74E-06
YMR310C	6.87E-08
YML006C	6.25E-07
YGR255C	7.66E-09
YDR482C	6.06E-07
YCR035C	1.24E-06
YMR217W	2.85E-07
YER058W	6.80E-08
YLR435W	3.22E-08
YFL014W	8.51E-12
YLL012W	5.35E-07
YLR003C	5.78E-07
YBR191W	2.06E-06
YNR063W	8.20E-06
YPR160W	4.09E-09
YLR121C	2.64E-08
YMR160W	2.53E-07
YNR034W-A	4.09E-09
YPL004C	1.88E-09
YLR201C	4.52E-08
YOL096C	1.02E-10
YDR298C	2.09E-08
YMR139W	1.92E-08
YPR149W	3.46E-10
YEL071W	2.46E-06

YGL255W	3.50E-08
YDR075W	8.18E-07
YOL087C	1.00E-06
YFR049W	1.65E-07
YMR238W	6.32E-08
YPL203W	3.98E-08
YMR195W	2.12E-08
YGR094W	7.65E-08
YDR434W	1.19E-07
YFR006W	6.97E-09
YML082W	9.04E-09
YFL041W-A	1.29E-07
YPL113C	9.24E-08
YIL145C	6.57E-09
YFR031C-A	3.59E-06
YBR024W	3.76E-07
YGR070W	4.71E-08
YOL082W	6.80E-12
YPR028W	1.39E-06

Hot Spot 16:510000

Accession ID	p-value
YJL147C	9.53E-07
YPL020C	1.99E-12
YPL036W	6.53E-12
YPL107W	9.53E-07
YPL026C	4.40E-07
YJR152W	9.53E-07
YJR138W	1.50E-06
YPL016W	4.10E-15
YOR034C	6.41E-06
YHR037W	9.23E-09
YOR278W	1.15E-06
YPL024W	7.97E-11
YDL004W	2.17E-06
YPL023C	6.21E-14
YPR004C	1.87E-06
YMR266W	1.01E-05
YGL187C	2.09E-06
YPL031C	1.97E-09
YMR155W	2.71E-06
YLR142W	8.81E-08
YPL022W	1.13E-11
YPL271W	3.34E-07
YPL038W-A	1.40E-12
YGR154C	1.29E-06
YAL056W	1.91E-07
YBR170C	1.22E-07

YGR138C	2.57E-07
YDR421W	8.50E-07
YCL025C	1.99E-07
YKL016C	1.03E-06
YCL064C	1.34E-06
YPL038W	3.88E-08
YPL039W	2.61E-14
YDR298C	3.48E-07
YMR195W	3.89E-06
YDR379W	1.09E-07

Supplementary Table 9: eQTL hot spots for the regression method

Hot Spot 2:550000

Accession ID	p-value
YKR009C	1.31E-19
YJL147C	2.25E-32
YDL216C	4.20E-17
YDR056C	1.85E-20
YNL216W	3.61E-26
YFL034C-B	2.65E-35
YEL006W	8.74E-20
YHL010C	1.68E-19
YLR286C	1.19E-197
YBR285W	2.10E-22
YPR106W	8.77E-173
YDR072C	1.18E-25
YBR157C	2.50E-38
YOR165W	4.48E-15
YOR264W	8.03E-144
YFL047W	1.49E-29
YGR174W-A	1.61E-16
YMR178W	1.50E-15
YBR084C-A	4.52E-19
YGL196W	7.54E-29
YHR053C	7.48E-62
YDR206W	2.10E-16
YLR299W	5.74E-27
YOR338W	3.24E-19
YOR179C	1.82E-29
YJL200C	4.41E-24
YNR067C	2.19E-140
YBR186W	7.45E-27
YDL055C	9.46E-73
YML124C	4.85E-61
YMR306W	1.95E-14
YOL021C	1.20E-35
YFR038W	1.47E-27
YDL218W	4.57E-23
YGR258C	3.52E-25
YDL155W	7.54E-31
YEL062W	1.03E-25
YDR145W	1.21E-17
YMR088C	5.41E-27
YKL180W	3.12E-20
YNR020C	3.97E-18
YPL259C	8.96E-19
YHR038W	2.26E-16
YKL059C	2.57E-20

YFL028C	4.67E-20
YOR355W	4.29E-15
YIL094C	1.46E-32
YJL125C	1.34E-23
YDL057W	1.92E-32
YGR085C	9.66E-20
YBR112C	4.13E-23
YBR291C	5.02E-49
YAR014C	1.15E-45
YHR040W	2.51E-19
YDR345C	1.48E-17
YLR026C	3.22E-16
YOR003W	5.14E-29
YER124C	2.87E-250
YGR266W	7.44E-26
YOR174W	4.24E-19
YER149C	1.14E-16
YBR135W	2.86E-34
YMR142C	4.73E-23
YOL055C	1.95E-13
YER064C	5.31E-41
YHR113W	1.86E-28
YLR271W	1.05E-23
YBR056W	1.80E-19
YPR163C	9.56E-19
YDR234W	2.11E-20
YKL112W	2.08E-22
YIR007W	9.03E-27
YIR030C	5.22E-24
YGL068W	7.49E-20
YGL259W	9.48E-35
YKL168C	8.84E-18
YPR181C	4.94E-24
YLR360W	6.18E-36
YPR125W	4.68E-30
YPL107W	6.21E-27
YKL103C	1.05E-32
YLR328W	4.30E-18
YBR023C	5.41E-22
YOL020W	1.19E-36
YLR457C	7.43E-15
YBL049W	1.86E-20
YDR370C	9.11E-19
YGR149W	1.36E-24
YBR079C	4.22E-21
YIL069C	1.45E-26
YMR078C	1.59E-22

YER089C	1.65E-32
YIR031C	4.78E-45
YOL127W	5.81E-18
YPR162C	3.52E-19
YDR272W	1.37E-23
YMR309C	4.03E-24
YDR506C	9.41E-26
YFR002W	8.80E-20
YJR152W	7.92E-54
YDL182W	1.30E-26
YMR180C	1.83E-16
YDL230W	8.96E-23
YGR084C	3.11E-17
YNL240C	8.07E-18
YOL047C	9.97E-59
YLR356W	2.69E-16
YHR067W	1.25E-21
YKL186C	3.62E-18
YKL149C	1.79E-17
YIL013C	2.97E-16
YGL185C	5.03E-32
YNR050C	3.03E-68
YIL070C	2.49E-14
YKL185W	8.33E-61
YJR078W	4.34E-16
YJR138W	1.48E-19
YGR015C	8.50E-16
YLR319C	1.25E-17
YDL066W	4.01E-19
YLR388W	3.79E-24
YGL229C	5.35E-44
YLR246W	8.39E-23
YJL197W	2.51E-16
YML118W	5.19E-33
YAL023C	3.33E-26
YPL221W	7.36E-24
YOR035C	2.22E-19
YBR094W	5.03E-41
YNL015W	4.18E-28
YHR176W	2.14E-16
YLR048W	1.06E-18
YOR056C	4.00E-33
YBR173C	6.95E-44
YKL204W	7.18E-22
YOL128C	4.12E-32
YOR316C-A	5.85E-80
YPL168W	7.82E-33

YJL206C	7.51E-23
YMR236W	3.57E-20
YER109C	2.07E-22
YGR041W	8.93E-139
YER154W	5.52E-20
YKR034W	1.81E-59
YML080W	1.28E-19
YER095W	1.98E-23
YOR126C	3.81E-29
YLR069C	8.43E-16
YOR297C	6.63E-23
YOR278W	2.43E-19
YGL164C	1.98E-35
YPR134W	2.91E-22
YEL063C	3.53E-34
YNL142W	2.59E-52
YIL117C	3.15E-28
YLR382C	1.85E-15
YIL164C	9.89E-31
YEL064C	7.64E-32
YER063W	3.89E-29
YDR514C	1.25E-27
YDR508C	1.57E-38
YGR082W	4.48E-26
YNL283C	8.72E-26
YBR146W	5.15E-24
YGR066C	4.25E-21
YMR118C	2.78E-31
YCL048W	7.65E-17
YDL233W	2.08E-16
YIL097W	6.40E-20
YBL009W	1.08E-22
YNL066W	6.22E-141
YBR132C	4.01E-186
YDL024C	3.92E-20
YOL131W	6.69E-17
YOR033C	7.22E-15
YBR091C	5.04E-22
YIL055C	2.12E-20
YLR353W	2.70E-34
YJL095W	8.68E-19
YJL145W	3.86E-45
YGR019W	4.83E-22
YIR027C	1.29E-48
YGR055W	1.54E-38
YLR357W	8.16E-20
YIL047C	9.86E-24

YDL239C	1.99E-44
YOL025W	1.20E-23
YOL044W	2.56E-15
YMR271C	3.62E-24
YGL141W	1.30E-16
YNL327W	1.28E-115
YNL209W	1.56E-23
YDL142C	6.53E-22
YIR032C	1.45E-47
YIR029W	8.88E-55
YHR135C	2.46E-20
YMR305C	5.21E-29
YDR326C	1.79E-25
YPL188W	2.64E-16
YDR131C	5.59E-27
YMR277W	6.52E-22
YER019W	1.24E-21
YGL071W	1.16E-14
YHR202W	2.17E-28
YMR194W	1.33E-26
YMR182W-A	8.55E-17
YBR171W	7.21E-21
YBR103W	1.43E-55
YBR196C-B	5.57E-154
YDR025W	4.96E-21
YCR020C	2.17E-16
YHR091C	2.03E-19
YOR386W	1.52E-33
YJL159W	3.30E-20
YPL011C	8.34E-18
YPL183C	2.72E-18
YJL116C	1.89E-29
YDL150W	1.50E-19
YOL083W	2.10E-20
YNL050C	9.58E-20
YBR115C	5.86E-179
YOR127W	5.53E-26
YGR125W	1.70E-46
YDR507C	8.26E-24
YNL282W	5.72E-22
YER131W	2.40E-21
YGR040W	1.87E-51
YER153C	5.09E-68
YMR155W	8.18E-25
YBR179C	2.45E-19
YMR255W	1.13E-34
YJL132W	1.57E-15

YJL060W	3.65E-17
YIL172C	5.10E-15
YLR229C	6.59E-28
YLR061W	3.03E-27
YOR296W	1.80E-52
YBR107C	9.63E-99
YMR001C	6.21E-30
YML053C	1.25E-14
YJR092W	6.13E-43
YLR142W	1.20E-26
YDR194C	9.72E-21
YIL146C	3.94E-38
YNL117W	2.12E-29
YNR068C	7.14E-31
YPR089W	1.11E-23
YMR101C	1.28E-21
YDL144C	4.29E-17
YDL122W	4.68E-27
YML031W	3.75E-28
YHR142W	1.36E-27
YIL088C	1.14E-22
YLR084C	4.49E-36
YNL239W	8.61E-39
YBR166C	3.44E-99
YPL087W	2.30E-16
YBR189W	1.09E-23
YPL163C	1.77E-46
YKL157W	3.58E-22
YGR013W	1.44E-23
YNL046W	5.21E-49
YPR040W	2.34E-22
YLR097C	2.59E-26
YJR036C	2.42E-21
YKL218C	6.07E-38
YMR062C	4.94E-19
YIL158W	5.64E-20
YDR409W	5.51E-16
YCL001W-B	1.66E-16
YPR114W	1.03E-19
YKL181W	3.86E-28
YKL006W	6.75E-24
YER074W	6.27E-22
YOR188W	1.56E-55
YLR099C	3.85E-23
YCL014W	8.51E-44
YDL082W	4.09E-20
YDL154W	6.54E-19

YNL317W	1.40E-15
YPR133W-A	1.95E-25
YBL071C-B	8.52E-24
YJR005W	1.38E-26
YER075C	7.02E-34
YGL028C	3.98E-230
YGR154C	4.97E-20
YML068W	3.94E-28
YFL021W	9.77E-38
YGL159W	1.87E-20
YGR229C	1.40E-19
YOR226C	2.97E-17
YLR419W	1.11E-26
YOR140W	1.11E-37
YKL107W	3.70E-23
YAL037W	4.59E-19
YPR007C	2.32E-19
YMR296C	3.11E-19
YGL064C	7.24E-27
YOR034C-A	2.42E-18
YER001W	3.05E-28
YGR147C	9.80E-19
YOR204W	3.71E-20
YER048C	3.33E-25
YIL131C	3.36E-38
YPR138C	3.93E-26
YBR170C	5.61E-27
YKR097W	2.21E-28
YIL071C	3.24E-24
YBR148W	1.45E-109
YHR028C	3.44E-18
YGL258W-A	4.49E-37
YOL121C	8.47E-20
YJR148W	4.91E-15
YPR041W	1.19E-23
YBR156C	4.99E-29
YER118C	2.96E-46
YNR069C	5.16E-29
YMR047C	1.65E-21
YHR029C	2.22E-14
YGL139W	8.72E-35
YMR174C	3.65E-21
YOR111W	1.77E-28
YGR067C	1.52E-29
YLR285C-A	2.56E-62
YFR047C	3.73E-20
YHR107C	2.76E-37

YLR380W	1.12E-13
YLR042C	4.80E-103
YMR129W	8.42E-27
YMR032W	7.90E-21
YGR174W-A	3.09E-17
YLL031C	2.43E-16
YMR173W	5.95E-26
YAL043C	6.56E-29
YDL238C	5.49E-56
YJR043C	2.32E-23
YPL213W	2.15E-20
YNL040W	7.38E-37
YKL179C	3.10E-22
YML109W	6.82E-25
YLL063C	5.94E-23
YDR425W	4.54E-27
YGL081W	9.27E-22
YPR105C	3.39E-33
YLR406C	4.16E-19
YNL078W	1.45E-71
YOR142W	1.91E-16
YGL076C	4.64E-21
YBR117C	5.27E-59
YHR022C	5.22E-16
YLR378C	5.47E-27
YBR038W	2.24E-23
YLR096W	5.33E-15
YEL015W	6.43E-18
YEL004W	5.97E-23
YPL061W	3.08E-21
YOR129C	4.36E-54
YBR191W	1.60E-16
YIL123W	3.88E-27
YPR074C	2.45E-20
YDL179W	6.84E-19
YJR147W	9.06E-54
YPL158C	2.94E-25
YDL210W	1.15E-44
YJR056C	1.26E-15
YLR080W	1.07E-34
YER152C	5.08E-147
YER025W	3.64E-22
YOR315W	9.98E-40
YOR316C	5.44E-22
YHR059W	2.44E-15
YOL115W	3.78E-26
YGR014W	1.30E-92

YDL078C	5.71E-26
YKR021W	3.80E-18
YBL085W	6.71E-23
YGL086W	1.74E-14
YOL052C	5.84E-27
YJL160C	5.25E-18
YJR096W	1.89E-16
YBR192W	1.36E-19
YGL105W	6.59E-23
YPL072W	3.34E-16
YFR028C	2.41E-32
YLR079W	5.15E-51
YGL154C	5.17E-18
YOR247W	1.07E-52
YOR232W	6.46E-20
YPR194C	2.75E-25
YIR028W	1.28E-48
YLR095C	1.05E-18
YLR258W	1.20E-23
YAL041W	1.36E-29
YBR193C	1.14E-45
YBR150C	3.24E-58
YKL068W-A	5.27E-20
YOR342C	4.38E-50
YER096W	8.71E-26
YNR053C	1.34E-19
YLR190W	8.38E-20
YDR333C	1.45E-34
YBR180W	6.64E-17
YOR134W	6.79E-16
YAL024C	1.76E-37
YIL165C	5.36E-29
YJL078C	1.68E-158
YJL177W	3.69E-20
YJR042W	4.24E-16
YBR196C-A	2.57E-158
YIL106W	1.11E-20
YCL008C	6.23E-13
YJL110C	9.90E-20
YLR164W	3.14E-25
YHL001W	2.49E-23
YLR114C	5.16E-19
YNL168C	2.00E-37
YPL123C	5.15E-32
YNL299W	4.44E-18
YMR285C	2.45E-23
YKR013W	1.30E-46

YHR109W	2.41E-13
YDR242W	1.15E-41
YPL085W	3.25E-23
YHR143W	5.10E-241
YKR091W	1.75E-23
YBR158W	1.30E-173
YBR119W	1.27E-74
YDR381W	9.40E-20
YDR026C	2.45E-18
YDR093W	1.89E-22
YHR099W	3.55E-20
YOR116C	5.94E-31
YJL053W	1.05E-16
YPL082C	2.59E-39
YGR044C	6.09E-85
YMR154C	1.69E-19
Hot Spot 2:430000	
Accession ID	p-value
YMR182C	3.32E-28
YOR124C	1.01E-20
YPL067C	6.98E-17
YHR157W	3.28E-20
YMR237W	6.79E-21
YNL233W	5.27E-12
YPL105C	9.32E-16
YMR016C	1.33E-36
YBR104W	1.24E-30
YCL026C-B	3.39E-16
YDL190C	5.99E-22
YNL278W	8.55E-21
YBR098W	5.07E-49
YJR103W	1.14E-17
YHL003C	2.41E-18
YNL288W	1.97E-19
Hot Spot 3:90000	
Accession ID	p-value
YOR271C	1.92E-45
YCL018W	3.08E-291
YMR008C	3.81E-17
YDL103C	1.11E-18
YGL153W	7.02E-16
YCL026C-A	2.02E-42
YOR080W	6.22E-19
YDR524C	7.23E-14
YDL243C	1.05E-23
YER073W	3.70E-31
YKL018C-A	2.58E-13

YLR179C	6.22E-15
YLR355C	2.54E-45
YJR016C	1.42E-55
YCL061C	6.59E-14
YCL017C	7.12E-97
YLR348C	5.88E-93
YCR018C	2.47E-115
YBR068C	2.98E-33
YDR047W	3.15E-20
YHR047C	1.78E-26
YGR230W	9.05E-19
YKR095W-A	1.15E-27
YHR205W	7.49E-15
YOR226C	3.06E-53
YCL026C-B	2.45E-129
YGL009C	1.13E-135
YLR138W	4.22E-14
YFL060C	2.14E-13
YKL120W	2.44E-110
YOR375C	2.35E-83
YHR208W	3.87E-126
YCL004W	8.59E-37
YCL021W-A	1.97E-272
YCL016C	1.43E-58
YML006C	6.16E-20
YNR065C	5.28E-14
YMR108W	1.36E-39
YPL113C	1.06E-17

Hot Spot 3:210000

Accession ID	p-value
YCL068C	8.32E-66
YKL208W	3.42E-33
YDR460W	1.47E-89
YGL089C	2.57E-206
YCR052W	1.51E-48
YLR040C	4.68E-216
YKL178C	7.54E-228
YNL146C-A	3.57E-186
YFL026W	6.73E-215
YKL209C	3.58E-218
YJR004C	1.25E-158
YGR012W	1.19E-21
YPL187W	1.47E-227
YFL027C	3.76E-126
YNL146W	2.25E-111
YNL145W	1.70E-223
YDR461W	1.72E-210

YGL032C	2.93E-210
YJL170C	5.49E-174
YIL015W	8.46E-221
YCL066W	8.55E-226
YCR038C	4.27E-112
YJL171C	3.02E-41
YCR097W	1.29E-225

Hot Spot 4:90000

Accession ID	p-value
YDL216C	1.67E-96
YDL218W	2.23E-39
YKL185W	9.73E-18
YDL217C	1.16E-29
YNL066W	4.14E-16
YDL205C	3.82E-175
YNL327W	2.81E-17
YDL207W	3.33E-39
YDL197C	1.94E-50
YDL209C	4.70E-24

Hot Spot 5:210000

Accession ID	p-value
YER035W	1.22E-25
YER053C	3.29E-16
YCL054W	4.75E-16
YBR084C-A	1.45E-18
YBL027W	2.36E-20
YGR085C	2.86E-17
YCL037C	2.79E-17
YIL069C	3.40E-18
YEL054C	4.13E-19
YDR418W	1.75E-16
YLR344W	1.97E-16
YLR048W	6.60E-21
YER037W	8.87E-29
YEL026W	4.62E-17
YDR471W	2.80E-17
YNL186W	3.36E-19
YCR087C-A	1.48E-14
YLR448W	5.73E-20
YOR310C	5.28E-15
YER020W	1.22E-104
YLR057W	1.35E-20
YMR194W	7.69E-17
YDR025W	7.27E-19
YER131W	3.49E-17
YJL191W	3.15E-28
YNL096C	4.04E-18

YDL082W	9.90E-17
YER029C	5.34E-94
YLR186W	7.84E-15
YLR406C	2.29E-17
YBR191W	3.02E-19
YDR258C	4.24E-16
YER018C	1.25E-51
YHL001W	1.63E-16
YIL133C	1.04E-17
Hot Spot 8:110000	
Accession ID	p-value
YHL010C	8.70E-33
YHR152W	7.00E-27
YLR266C	1.14E-14
YFL047W	6.08E-20
YHR005C	6.28E-53
YHL020C	3.61E-40
YML102W	7.96E-18
YHR097C	4.29E-16
YNL233W	6.80E-22
YMR274C	7.24E-23
YKL165C	4.25E-17
YLR234W	3.71E-15
YMR179W	6.88E-16
YNL212W	4.30E-18
YHL006C	1.13E-45
YCL027W	5.40E-105
YBL016W	2.49E-54
YBR040W	1.48E-43
YHR201C	3.84E-17
YPR122W	5.95E-26
YHL009C	4.16E-22
YER016W	3.23E-37
YGR068C	6.00E-16
YPL192C	5.41E-48
YJL187C	5.75E-26
YMR236W	7.24E-18
YLL002W	9.84E-18
YGR109W-A	8.56E-32
YIL016W	1.68E-22
YMR144W	2.00E-14
YHL012W	4.34E-41
YBR057C	8.98E-21
YDL127W	6.39E-24
YPL124W	1.99E-18
YNR032W	5.35E-21
YGL061C	1.34E-24

YNL273W	3.33E-17
YOR066W	4.37E-16
YIL037C	2.97E-47
YLR442C	1.09E-21
YLR183C	2.38E-19
YPL041C	7.40E-19
YMR288W	1.31E-23
YNL072W	9.58E-22
YCR089W	6.06E-67
YNL279W	7.88E-62
YBL052C	1.62E-18
YNL326C	8.13E-21
YPR115W	1.64E-40
YHL008C	3.33E-53
YMR065W	1.57E-69
YDL003W	2.14E-17
YHL009W-A	2.73E-55
YMR232W	2.84E-60
YHR061C	1.28E-29
YDR124W	1.73E-26
YKL189W	1.13E-35
YDL154W	2.77E-17
YHR084W	1.12E-52
YKL101W	2.13E-17
YOR212W	6.88E-31
YGL060W	4.87E-56
YER155C	5.52E-41
YDR410C	3.65E-20
YML047C	4.33E-57
YNL278W	5.42E-25
YGL106W	4.49E-38
YNL300W	1.46E-22
YJL157C	3.04E-78
YHL016C	2.78E-86
YOR180C	3.33E-14
YLR452C	2.22E-81
YOL007C	2.12E-18
YGR047C	5.99E-19
YCL055W	1.58E-86
YPL156C	3.87E-21
YDR085C	3.95E-22
YOR219C	1.43E-25
YNR044W	3.59E-96
YBR083W	1.07E-55
YJR086W	1.12E-20
YHL003C	1.88E-57
YDR249C	4.13E-19

YHL009W-B	9.99E-50
YML046W	4.79E-58
YBR119W	1.94E-16
Hot Spot 9:250000	
Accession ID	p-value
YOR285W	6.66E-15
YIL044C	9.47E-43
YAL053W	2.47E-18
YBR004C	8.14E-22
YIL064W	3.88E-35
YIL061C	7.15E-48
YIL048W	5.11E-24
YIL049W	1.52E-27
YIL063C	5.47E-20
YIL101C	1.44E-34
YIL062C	1.39E-19
YOR274W	1.48E-19
YBR182C	3.28E-21
YIL106W	5.41E-17
Hot Spot 12:650000	
Accession ID	p-value
YNR074C	1.26E-20
YEL031W	1.02E-18
YOR175C	1.26E-40
YML019W	2.52E-26
YDL086W	7.99E-109
YKL075C	2.27E-23
YMR212C	7.82E-23
YMR220W	1.27E-76
YLR283W	2.02E-48
YDR518W	7.13E-45
YJL192C	5.44E-21
YDL012C	4.99E-16
YHL020C	4.66E-19
YDL236W	5.06E-38
YLR300W	4.77E-40
YCL043C	1.12E-14
YEL051W	7.01E-21
YML125C	1.27E-20
YLR256W	1.18E-125
YJL108C	1.24E-17
YOR321W	6.02E-37
YML007C-A	2.97E-18
YJL100W	1.47E-28
YGL001C	2.32E-82
YLR265C	1.12E-149
YNR043W	1.05E-86

YPR062W	1.34E-22
YLR260W	7.85E-53
YOR003W	1.95E-29
YPR193C	1.69E-50
YLR237W	3.37E-37
YGR266W	7.40E-40
YER093C	3.26E-27
YFR033C	2.45E-41
YLR275W	5.85E-44
YML008C	4.18E-55
YMR298W	9.54E-34
YLR038C	2.35E-19
YIL121W	2.07E-221
YHR072W	1.04E-93
YMR202W	2.91E-104
YDR200C	7.23E-18
YLR306W	1.48E-19
YML075C	3.84E-105
YLR231C	4.29E-73
YDR502C	5.52E-74
YNL111C	4.65E-64
YOR099W	1.90E-24
YDR213W	5.91E-34
YGR089W	9.84E-26
YDR297W	6.52E-47
YDR453C	1.02E-20
YPR151C	5.41E-102
YDR186C	5.73E-29
YLR244C	8.55E-86
YDR346C	5.37E-27
YGR026W	2.50E-22
YDR323C	2.06E-18
YMR208W	2.15E-99
YEL034W	5.14E-122
YGL160W	6.55E-65
YLR246W	1.30E-33
YJL048C	2.55E-84
YDL093W	1.26E-51
YGL101W	7.67E-59
YDL185W	9.24E-18
YBR067C	5.93E-46
YOR332W	4.67E-16
YHR039C-A	6.59E-18
YLR213C	6.74E-33
YLR245C	2.65E-95
YPR084W	8.49E-17
YIR033W	1.16E-26

YAL028W	7.44E-38
YOR010C	6.03E-24
YHR003C	1.65E-26
YCR048W	9.63E-31
YBR183W	7.43E-33
YLR205C	3.07E-108
YER014W	2.94E-68
YOL002C	1.91E-17
YJR049C	6.18E-19
YJR048W	3.63E-63
YBL033C	3.81E-18
YIL053W	1.05E-15
YPL159C	6.15E-22
YHR008C	2.67E-26
YDR275W	2.05E-19
YCR061W	8.47E-20
YML028W	4.27E-18
YER122C	7.61E-33
YHL002W	1.71E-20
YDR326C	4.35E-15
YJR126C	1.24E-20
YMR110C	1.14E-16
YNR019W	6.36E-92
YIL154C	2.33E-21
YKL077W	8.75E-20
YKR046C	3.10E-145
YLR194C	4.69E-20
YIL011W	2.09E-16
YJR104C	8.43E-22
YDR166C	3.00E-15
YNL264C	5.92E-31
YPL041C	9.12E-26
YPL231W	5.16E-25
YMR223W	2.43E-20
YLR153C	4.94E-112
YMR038C	3.87E-36
YPL028W	9.69E-70
YOL135C	8.04E-28
YKR051W	2.24E-20
YLR241W	4.21E-21
YDL073W	5.67E-16
YEL024W	7.06E-30
YNL003C	7.62E-30
YLR288C	2.35E-62
YDL178W	7.10E-18
YER043C	9.87E-24
YIL111W	1.55E-24

YNL010W	5.11E-32
YBL098W	2.04E-21
YHR190W	1.18E-66
YDR304C	7.68E-17
YML126C	6.08E-103
YPR040W	6.13E-16
YGL191W	1.60E-27
YLR281C	1.79E-42
YDR284C	9.81E-32
YOR348C	1.93E-28
YLR100W	8.39E-51
YGL017W	2.01E-16
YBL043W	1.13E-28
YNL231C	5.51E-18
YGR131W	1.96E-21
YDR011W	3.60E-19
YPL232W	1.52E-26
YOR065W	6.30E-22
YHR179W	1.10E-43
YLR176C	6.02E-16
YDL193W	7.84E-23
YBR085W	4.37E-23
YKL182W	1.69E-23
YLR154C	3.03E-33
YPR154W	7.40E-17
YOR085W	2.76E-25
YPR095C	3.16E-26
YHR210C	1.17E-18
YIL007C	9.63E-40
YPR113W	9.68E-32
YDR218C	3.13E-26
YPL008W	3.06E-17
YHR039C	2.04E-65
YGR138C	5.71E-23
YDR488C	3.48E-24
YGR234W	1.75E-74
YNL280C	2.50E-33
YDR384C	3.80E-18
YPL229W	1.49E-20
YLR414C	7.74E-20
YNR060W	4.22E-37
YPR065W	1.92E-58
YOL049W	5.73E-21
YJR051W	1.68E-17
YMR009W	3.45E-91
YOR237W	1.05E-49
YMR244W	2.99E-24

YMR134W	7.46E-102
YLR326W	1.81E-67
YMR145C	2.48E-23
YJL002C	2.21E-21
YCR069W	3.36E-34
YOR164C	5.45E-27
YML081W	5.80E-20
YNL156C	9.60E-89
YPL117C	3.92E-60
YER011W	2.62E-49
YJR105W	1.63E-54
YHR004C	3.42E-20
YDL100C	1.58E-20
YDL174C	3.80E-43
YDR044W	4.66E-50
YGL130W	1.56E-20
YER044C	5.69E-80
YAL016W	2.73E-26
YGL056C	4.08E-20
YJR047C	1.26E-38
YAL039C	2.58E-24
YJL105W	7.05E-17
YGR049W	2.54E-76
YIL106W	3.51E-20
YOR377W	5.87E-46
YNL159C	2.92E-15
YLL012W	1.13E-32
YOR118W	7.38E-26
YGR060W	9.63E-34
YDR483W	2.83E-15
YBR242W	1.50E-72
YHR048W	1.79E-55
YER053C-A	1.94E-88
YJL167W	2.76E-93
YMR015C	2.61E-67
YMR252C	7.19E-25
YNR021W	1.30E-20
YNL158W	6.82E-18
YOR229W	1.68E-43

Hot Spot 13:70000

Accession ID	p-value
YDR481C	9.64E-87
YML113W	4.78E-20
YDL049C	1.50E-14
YHR198C	2.62E-22
YJL186W	4.07E-27
YER053C	1.01E-23

YLR089C	7.09E-23
YBR296C	3.61E-99
YHR124W	5.80E-20
YML091C	6.88E-44
YJR121W	3.13E-20
YML067C	3.17E-34
YCR023C	2.22E-15
YML123C	2.80E-128
YDL199C	4.11E-18
YIL073C	1.64E-30
YNL265C	2.71E-19
YPL019C	1.21E-128
YML099C	8.46E-16
YML131W	5.12E-21
YPR191W	1.55E-23
YLR410W	3.59E-35
YML096W	1.82E-24
YAR008W	7.49E-25
YFL004W	1.84E-43
YGR233C	2.65E-96
YJL137C	9.39E-27
YPR091C	1.21E-17
YML118W	6.35E-23
YDL181W	5.66E-20
YML108W	3.15E-17
YDR369C	5.42E-41
YPR086W	1.63E-21
YLR295C	1.34E-21
YML078W	8.24E-102
YOL069W	1.26E-21
YHR214C-E	4.28E-31
YFR044C	7.77E-28
YML087C	2.36E-46
YAR007C	1.07E-16
YCR037C	1.29E-54
YDL004W	1.98E-24
YJL012C	4.25E-128
YCL049C	1.28E-22
YPR094W	2.71E-17
YGL187C	6.76E-19
YDR248C	8.00E-27
YML076C	3.37E-65
YML066C	5.11E-91
YER173W	5.35E-17
YHR183W	8.14E-50
YIL172C	8.20E-29
YHR136C	3.33E-148

YJL117W	3.23E-62
YLR438W	3.44E-95
YER060W-A	2.63E-25
YDL073W	4.05E-19
YLR142W	2.19E-25
YOR163W	4.84E-31
YMR300C	9.37E-34
YHR047C	2.20E-22
YPL268W	1.32E-34
YDR204W	1.06E-20
YLL055W	2.83E-18
YML111W	5.46E-48
YDR019C	3.98E-19
YOR374W	1.16E-44
YOR211C	2.12E-16
YLL041C	5.36E-20
YNL173C	1.83E-20
YIL051C	2.98E-58
YAR071W	1.60E-107
YML119W	4.64E-34
YKL141W	1.25E-26
YPL110C	3.63E-37
YKR093W	1.59E-27
YER057C	5.13E-24
YMR120C	1.39E-20
YML085C	4.41E-99
YOR274W	5.34E-25
YLR180W	3.77E-24
YKL016C	4.43E-23
YCL064C	1.65E-75
YNL012W	7.49E-24
YPL111W	4.68E-52
YNR002C	3.99E-40
YKL051W	3.99E-42
YBR208C	6.26E-32
YEL049W	9.75E-16
YPR139C	1.25E-33
YML104C	5.90E-28
YER072W	1.84E-128
YML079W	9.14E-25
YPL018W	4.47E-42
YBR056W-A	8.00E-22
YPL063W	1.76E-21
YDR403W	3.94E-25
YDR482C	1.01E-53
YPL004C	1.98E-20
YDR298C	9.58E-22

YGL255W	6.31E-29
YBR093C	1.19E-117
YGR061C	3.74E-27
YLR126C	3.49E-15
YFR006W	3.46E-26
YFR031C	2.38E-21
YDR281C	3.92E-107
Hot Spot 13:910000	
Accession ID	p-value
YMR306W	1.22E-23
YOR390W	6.53E-32
YMR318C	2.32E-21
YKL220C	9.31E-21
YDR534C	4.14E-31
YOR389W	2.01E-33
YEL065W	2.02E-18
YHL035C	6.58E-13
YMR319C	1.24E-53
YPL278C	8.05E-50
YMR058W	2.21E-17
YOR381W	7.45E-16
YOL158C	1.81E-19
YPL277C	2.87E-47
YMR302C	3.65E-16
YPL277C	2.35E-49
YMR315W-A	4.45E-19
YPL273W	1.72E-65
YOR382W	2.59E-26
YMR251W	4.04E-14
YMR312W	1.59E-23
YER145C	1.38E-13
YLR136C	4.47E-15
YPL274W	6.98E-43
YMR316W	4.71E-25
YMR321C	7.88E-65
Hot Spot 14:410000	
Accession ID	p-value
YOR124C	3.19E-59
YBR017C	4.27E-34
YCR088W	3.25E-38
YDR206W	4.77E-37
YPL195W	2.87E-26
YDR145W	1.17E-43
YMR087W	1.44E-50
YOR355W	3.07E-26
YJL068C	8.68E-34
YBR056W	5.54E-32

YGR141W	7.46E-48
YGR218W	1.28E-56
YHR200W	1.42E-33
YOR213C	6.60E-31
YEL030W	1.81E-54
YDR129C	5.55E-43
YFL017C	1.91E-36
YCL063W	3.26E-49
YKL133C	4.25E-45
YML070W	2.98E-40
YEL020C	2.70E-43
YIR036C	1.28E-43
YFR009W	1.15E-69
YPL105C	9.95E-46
YPL184C	1.20E-90
YGL173C	1.87E-98
YDL160C	1.97E-68
YER161C	7.96E-32
YMR027W	4.91E-43
YFL007W	1.76E-68
YGL213C	1.11E-28
YOR367W	2.96E-44
YIL137C	1.63E-56
YHR205W	1.15E-38
YDL126C	2.01E-40
YDL190C	1.15E-41
YPR133C	8.07E-28
YKL145W	5.54E-46
YMR222C	1.07E-40
YEL015W	3.01E-41
YOR360C	1.43E-49
YJR103W	9.23E-56
YKL068W	7.00E-48
YDL132W	3.45E-35
YGL137W	2.71E-35
YBR056W-A	3.89E-19

Hot Spot 15:150000

Accession ID	p-value
YIL157C	2.52E-35
YPL223C	7.47E-55
YML035C	1.71E-18
YIL160C	1.04E-46
YDL048C	5.88E-36
YKL195W	6.82E-25
YPL020C	8.27E-23
YDR447C	2.00E-32
YKL096W	5.44E-42

YEL003W	4.06E-22
YOR271C	6.17E-32
YOL028C	1.40E-33
YKL192C	8.21E-28
YKL167C	1.22E-27
YOL122C	9.08E-21
YPR117W	4.41E-19
YOL105C	3.07E-36
YOL077W-A	4.21E-33
YMR169C	2.43E-47
YKL194C	2.80E-26
YBR295W	2.12E-31
YPL131W	3.34E-20
YGR088W	4.11E-57
YMR135C	4.14E-46
YPL078C	6.07E-32
YDL223C	1.31E-42
YOL090W	6.36E-41
YOL036W	4.08E-20
YOL098C	2.99E-23
YGR258C	4.30E-27
YER103W	2.38E-23
YJL205C	1.14E-24
YNL313C	1.37E-20
YCR059C	3.06E-33
YLL028W	1.28E-187
YPR055W	3.36E-23
YCR073C	1.97E-42
YOL109W	1.93E-42
YER013W	7.42E-31
YBL072C	2.36E-22
YOR032C	5.03E-17
YGL143C	2.84E-19
YLR218C	1.22E-27
YGR272C	2.11E-22
YCR017C	1.10E-17
YCR083W	3.45E-22
YHR011W	9.05E-18
YFL030W	1.23E-47
YDR375C	5.27E-21
YJL063C	3.44E-20
YDL222C	3.22E-64
YNL073W	4.99E-23
YER170W	1.15E-16
YDR337W	1.22E-23
YBR023C	3.64E-28
YDR349C	5.81E-38

YGL096W	9.22E-25
YKR049C	1.97E-41
YKR043C	8.70E-48
YGR053C	7.45E-30
YDR347W	5.41E-19
YOR246C	2.88E-42
YGR166W	8.53E-28
YOR353C	2.14E-27
YFR039C	7.10E-25
YBR131W	1.30E-22
YMR225C	4.95E-20
YPL132W	6.20E-44
YER101C	7.59E-37
YOL104C	8.70E-226
YKL161C	1.53E-24
YAL053W	1.52E-19
YBR175W	1.05E-18
YCR095C	1.77E-24
YNL045W	1.52E-36
YFL017C	2.90E-24
YLR204W	9.93E-26
YGR031W	6.53E-39
YGR201C	5.16E-41
YDR418W	6.35E-22
YGR033C	2.29E-31
YHR030C	2.62E-21
YGR217W	2.26E-33
YML030W	1.03E-35
YKR071C	5.56E-21
YCR102C	8.50E-25
YJR039W	2.03E-20
YKL067W	6.62E-27
YMR244C-A	2.36E-41
YPR091C	1.04E-32
YER177W	1.47E-23
YPL048W	1.11E-16
YML070W	1.93E-27
YGR175C	9.80E-36
YLR108C	1.83E-20
YCL035C	2.46E-31
YOL006C	2.94E-25
YOR195W	1.50E-27
YKL062W	3.62E-58
YEL046C	4.19E-31
YJL163C	6.96E-30
YNL108C	1.73E-26
YHR176W	1.86E-26

YNL306W	3.81E-25
YBL072C	9.61E-24
YJL055W	2.52E-20
YGR052W	1.28E-72
YGL004C	1.44E-23
YDR430C	8.94E-23
YCR048W	1.00E-15
YEL029C	2.15E-28
YMR264W	5.38E-21
YDR179W-A	6.35E-20
YLR069C	2.23E-16
YLR295C	2.07E-37
YDL170W	4.64E-25
YBR259W	3.18E-22
YPR047W	2.84E-17
YPL118W	1.96E-20
YFR044C	3.28E-30
YKR018C	1.06E-17
YFR011C	4.15E-29
YLR382C	1.08E-21
YML129C	7.29E-21
YOR086C	9.62E-27
YBR004C	5.02E-21
YBR230C	1.75E-31
YKL096W-A	2.29E-43
YDR313C	1.68E-20
YBR146W	4.76E-14
YOL084W	6.56E-86
YFR055W	3.97E-22
YIL097W	1.73E-22
YNR036C	1.03E-23
YGR076C	8.90E-22
YDL061C	1.51E-25
YPL175W	3.09E-35
YNL177C	1.43E-14
YNR022C	9.58E-24
YOL131W	1.74E-39
YLR070C	3.55E-33
YDR511W	1.22E-20
YLR395C	1.23E-23
YMR175W	7.76E-33
YIL047C	2.64E-33
YMR031C	2.32E-65
YDL121C	3.72E-21
YOL092W	3.30E-97
YNL195C	1.03E-26
YER027C	1.90E-17

YIR035C	2.83E-15
YHL034C	1.53E-21
YBL035C	5.64E-24
YNR033W	2.49E-26
YLL026W	2.17E-34
YNL200C	1.34E-35
YOR354C	8.22E-20
YHR020W	3.06E-26
YDR533C	2.80E-136
YDL214C	2.91E-22
YBR104W	1.45E-27
YGR043C	9.80E-53
YLR102C	2.35E-37
YOR107W	1.21E-21
YNL081C	4.54E-27
YDR351W	1.73E-25
YFL036W	1.90E-16
YAL008W	5.78E-24
YGR271C-A	9.42E-23
YFR013W	3.67E-24
YGL077C	3.95E-17
YDR047W	8.98E-36
YCR004C	2.52E-35
YGR169C	1.65E-16
YEL024W	3.58E-17
YPR161C	4.29E-24
YGR220C	1.14E-20
YHR009C	1.34E-32
YLL001W	1.06E-27
YOR005C	1.36E-18
YOL119C	2.13E-35
YMR272C	2.16E-75
YKL026C	4.97E-44
YER043C	1.57E-21
YMR136W	1.47E-47
YMR041C	3.09E-30
YOR317W	1.63E-19
YGL208W	7.14E-18
YGL236C	5.12E-17
YPR011C	1.10E-18
YJR036C	2.76E-21
YDR216W	1.08E-62
YBR058C-A	4.89E-18
YML081C-A	1.55E-24
YLR143W	2.11E-23
YOR176W	1.74E-20
YBL029C-A	3.70E-31

YOL113W	5.72E-26
YMR267W	7.28E-28
YHL033C	1.67E-26
YDL021W	1.29E-29
YKL117W	3.63E-20
YDR171W	6.02E-32
YKL093W	1.86E-27
YLR332W	3.22E-19
YOL095C	8.87E-49
YKR095W-A	4.12E-24
YFL042C	3.09E-26
YPL040C	4.22E-21
YOR226C	2.48E-25
YOL029C	2.86E-41
YIR038C	3.55E-23
YBR073W	1.08E-22
YPR172W	6.31E-27
YHR080C	3.18E-44
YNL131W	8.20E-23
YOR286W	6.03E-21
YER001W	4.22E-26
YER048C	2.04E-17
YKL052C	3.08E-74
YJR080C	7.29E-29
YOL089C	1.63E-70
YNL134C	7.62E-97
YBL087C	5.62E-25
YNL208W	4.68E-16
YJR019C	3.15E-26
YNL137C	1.89E-19
YAL060W	1.16E-17
YKR093W	5.07E-37
YOL091W	2.26E-107
YIL155C	6.14E-34
YGR243W	2.60E-43
YGR102C	2.45E-31
YOL116W	1.34E-29
YOR051C	2.17E-20
YDR179C	1.85E-23
YMR072W	1.04E-21
YJL062W-A	1.79E-24
YJR127C	3.97E-49
YCR046C	3.47E-26
YMR194C-B	3.84E-50
YOR096W	3.40E-24
YGR021W	5.71E-30
YMR157C	3.33E-25

YNL133C	4.07E-37
YJL118W	2.04E-20
YDR296W	3.68E-23
YOL114C	2.07E-18
YOR161C	1.46E-54
YMR286W	4.25E-22
YNR060W	2.82E-19
YGR244C	9.26E-41
YIL008W	5.07E-29
YGL010W	3.74E-22
YJR034W	9.81E-28
YER117W	2.93E-23
YBR191W	5.39E-22
YHL004W	1.30E-21
YJL129C	3.16E-27
YBR162W-A	1.48E-25
YBR007C	3.20E-28
YMR145C	2.46E-18
YIL098C	1.10E-17
YLR390W	2.72E-17
YML110C	2.74E-31
YKL120W	4.61E-23
YKL051W	5.84E-47
YML009C	2.15E-19
YNL234W	1.31E-36
YNL301C	8.38E-22
YOL094C	8.64E-29
YHR116W	4.28E-22
YJL011C	3.65E-24
YJR096W	1.30E-40
YDR406W	1.11E-33
YBR185C	7.64E-18
YER062C	9.88E-49
YJR011C	9.18E-27
YGL130W	3.16E-25
YOR288C	6.75E-22
YOL088C	4.32E-39
YBL061C	2.09E-39
YER060W	1.81E-26
YBR177C	4.51E-56
YOL100W	2.43E-35
YDR135C	1.65E-24
YGR195W	1.76E-24
YDR063W	1.12E-19
YEL040W	8.39E-25
YIL106W	1.68E-23
YBR092C	8.52E-21

YBR030W	2.08E-24
YER058W	1.03E-15
YFL014W	1.91E-78
YLL012W	1.33E-26
YBR191W	4.33E-21
YGR060W	6.55E-19
YLR121C	4.55E-27
YOL096C	4.22E-47
YDR298C	1.30E-32
YPR149W	1.67E-50
YGL255W	6.41E-30
YFR049W	3.29E-21
YGR165W	3.01E-18
YMR195W	9.15E-28
YMR108W	5.77E-22
YFR006W	9.20E-42
YJL180C	8.03E-16
YIL145C	6.54E-30
YOL082W	1.81E-49

Hot Spot 16:510000

Accession ID	p-value
YJL147C	4.31E-26
YPL020C	8.27E-22
YPR106W	2.27E-21
YDR515W	4.35E-19
YOL077W-A	8.88E-21
YPL127C	4.37E-21
YPL078C	8.41E-23
YJR121W	2.42E-17
YPL036W	4.00E-59
YFR033C	3.14E-20
YPL107W	5.04E-29
YER168C	2.01E-19
YER088C	4.97E-17
YPR191W	4.75E-22
YJR152W	4.47E-20
YPR020W	3.27E-38
YJR138W	5.17E-20
YKL018C-A	1.98E-17
YGR217W	1.30E-20
YDR178W	3.09E-16
YLR246W	1.72E-19
YDL181W	9.85E-25
YKL062W	3.29E-24
YNL314W	1.89E-20
YBR106W	3.64E-18
YPL016W	2.04E-73

YIL030C	4.42E-23
YOR034C	1.31E-19
YDL067C	4.00E-21
YHR037W	9.90E-37
YPL054W	5.46E-22
YOR278W	7.83E-24
YHR001W-A	2.68E-22
YFR044C	2.14E-19
YPL024W	1.55E-50
YDL004W	3.62E-24
YGR086C	2.83E-21
YPL023C	2.85E-70
YLR395C	2.63E-32
YPR004C	2.38E-17
YNL330C	1.11E-15
YNL125C	3.46E-19
YCL057C-A	3.37E-14
YIR029W	8.26E-26
YDR326C	1.34E-20
YPL188W	4.85E-24
YGL187C	2.56E-29
YNL052W	4.13E-23
YJL161W	4.37E-19
YPL031C	1.29E-23
YLR057W	4.47E-18
YMR155W	7.53E-28
YBL045C	6.48E-22
YMR255W	8.38E-21
YJL060W	1.49E-21
YDR260C	2.04E-17
YLR142W	3.93E-31
YJL210W	9.60E-25
YPL022W	1.00E-29
YMR041C	5.76E-17
YPL271W	3.56E-24
YOR317W	5.75E-19
YJR077C	1.04E-18
YML081C-A	3.14E-16
YOR348C	5.25E-30
YBL099W	7.21E-19
YHR090C	2.35E-15
YNL317W	5.74E-18
YPL038W-A	1.03E-66
YER001W	3.05E-25
YNL173C	1.83E-22
YBR170C	4.60E-18
YBR085W	3.07E-19

YFR046C	3.11E-24
YHR210C	1.12E-15
YBL030C	1.17E-17
YKL179C	7.35E-17
YGR138C	9.77E-29
YDR421W	4.47E-26
YHR022C	1.82E-21
YKL054C	2.53E-15
YKL016C	1.11E-20
YJR047C	1.52E-21
YOR114W	1.28E-19
YPL039W	8.33E-96
YFL014W	2.99E-20
YER065C	1.49E-21
YDR298C	4.16E-29
YJR144W	4.68E-21
YFR006W	3.51E-19
YOR116C	4.85E-25
YGR044C	4.73E-19

Supplementary Table 10: eQTL hot spots for the Fisher's p-value method

Hot Spot 2:550000

Accession ID	p-value
YER059W	1.24E-25
YJL147C	1.51E-35
YDR056C	1.19E-26
YFL034C-B	2.19E-36
YLR286C	1.42E-92
YMR182C	4.69E-30
YKL166C	1.45E-28
YBR285W	7.20E-28
YPR106W	7.27E-94
YDR072C	6.26E-34
YBR157C	3.90E-46
YOR264W	1.52E-86
YFL047W	1.67E-35
YGL196W	1.15E-33
YHR053C	1.52E-58
YLR299W	1.70E-30
YOR338W	1.51E-24
YOR179C	3.41E-26
YNR067C	1.28E-92
YBR186W	2.05E-27
YDL055C	4.09E-70
YML124C	2.73E-55
YOL021C	2.09E-30
YFR038W	7.18E-29
YDL218W	1.20E-23
YEL062W	1.05E-31
YMR088C	1.21E-34
YPL259C	3.05E-22
YEL036C	2.37E-23
YKL059C	2.96E-24
YIL094C	8.35E-33
YDL057W	1.46E-35
YBR291C	5.66E-27
YAR014C	6.42E-48
YBR197C	4.85E-95
YOR003W	4.25E-32
YER124C	9.60E-94
YGR266W	9.68E-26
YBR135W	1.32E-54
YBL050W	3.04E-23
YER064C	3.63E-40
YHR113W	6.84E-35
YLR271W	1.37E-24
YIR007W	3.29E-32

YIR030C	3.48E-29
YGL259W	1.07E-27
YLR360W	1.67E-32
YPR125W	1.10E-28
YKL103C	6.15E-43
YBR023C	1.00E-29
YOL020W	1.56E-32
YOR246C	3.77E-26
YDR370C	8.41E-22
YGR149W	2.78E-24
YMR078C	4.07E-26
YER089C	8.49E-34
YIR031C	8.22E-42
YDR272W	4.09E-27
YDR506C	5.00E-33
YFR002W	3.27E-23
YJR152W	1.13E-52
YDL182W	3.50E-25
YNL270C	2.71E-24
YOL047C	1.02E-34
YBR165W	3.47E-27
YKL186C	4.62E-22
YPR141C	4.78E-24
YMR170C	6.95E-36
YGL185C	2.56E-33
YNR050C	4.04E-46
YKL185W	2.83E-57
YJR138W	8.24E-25
YBR172C	4.94E-27
YGR217W	1.00E-28
YLR319C	5.34E-23
YBL037W	2.10E-28
YHR018C	6.00E-24
YGL229C	2.86E-43
YLR246W	8.30E-27
YBR235W	3.69E-44
YML118W	6.75E-35
YBR228W	2.92E-35
YPL221W	9.18E-27
YOR035C	8.88E-25
YNL015W	6.30E-29
YIL140W	6.81E-25
YOR056C	2.72E-34
YBL034C	1.78E-22
YBR173C	3.88E-56
YOL128C	3.55E-32
YOR316C-A	1.18E-69

YJL206C	9.71E-24
YER109C	1.11E-28
YGR041W	2.00E-88
YKR034W	3.58E-43
YER095W	1.04E-25
YOR126C	2.22E-36
YOR278W	5.48E-25
YGL164C	7.42E-40
YBR198C	1.98E-25
YEL063C	1.68E-25
YNL142W	2.60E-34
YIL117C	3.23E-27
YIL164C	6.34E-28
YNL298W	2.91E-28
YEL064C	1.63E-33
YHR171W	3.74E-26
YER063W	1.97E-32
YJL092W	6.85E-25
YDR514C	3.26E-30
YOR205C	1.81E-26
YNL283C	1.25E-33
YMR165C	1.81E-24
YBR146W	1.01E-24
YGR066C	1.58E-21
YBR199W	3.29E-56
YBL009W	1.21E-33
YNL066W	3.11E-88
YKL008C	6.92E-34
YBR132C	8.60E-91
YIL055C	6.27E-24
YLR353W	3.64E-37
YJL145W	1.38E-37
YGR019W	5.30E-35
YIR027C	8.57E-43
YGR055W	8.52E-29
YNL268W	2.63E-29
YLR357W	2.12E-24
YIL047C	1.90E-24
YDL239C	6.38E-41
YOL025W	3.12E-28
YMR271C	1.67E-24
YNL327W	2.02E-84
YIR032C	1.24E-30
YIR029W	1.41E-50
YHR135C	9.38E-26
YMR305C	1.70E-43
YDR326C	3.90E-28

YMR277W	1.76E-24
YJL183W	2.21E-25
YHR202W	1.59E-33
YMR182W-A	2.98E-24
YBR171W	2.20E-28
YBR103W	2.93E-61
YOR386W	2.71E-31
YJL159W	7.46E-26
YPL011C	1.22E-24
YJL116C	1.91E-32
YOL083W	1.58E-25
YBR115C	2.23E-78
YOR127W	1.53E-32
YGR125W	7.85E-43
YDR507C	3.97E-34
YNL282W	2.34E-28
YGR040W	6.45E-53
YER153C	4.55E-67
YMR155W	7.90E-28
YBR179C	2.41E-33
YPL171C	3.54E-23
YDL169C	4.22E-29
YMR255W	3.00E-37
YJL060W	4.93E-22
YLR229C	8.66E-38
YDR260C	1.11E-24
YKL050C	1.89E-23
YOR296W	7.04E-53
YBR107C	3.40E-72
YJR092W	2.12E-31
YMR073C	1.27E-21
YLR142W	3.27E-30
YIL146C	1.29E-37
YNR068C	2.74E-29
YPR089W	9.92E-28
YDL122W	7.64E-30
YML031W	1.50E-27
YHR142W	3.58E-37
YBR130C	4.87E-31
YIL088C	1.26E-32
YLR084C	1.63E-34
YNL239W	2.59E-43
YBR166C	9.53E-59
YPR159W	7.88E-25
YBR189W	2.17E-22
YPL163C	4.72E-46
YKL157W	1.18E-30

YGR013W	2.36E-27
YNL046W	1.79E-55
YJR036C	8.82E-26
YKL218C	1.33E-43
YJL077W-B	2.13E-26
YKL181W	1.29E-26
YOR188W	4.49E-51
YDR464W	3.03E-22
YGL209W	3.42E-22
YCL014W	2.96E-38
YML106W	2.99E-25
YBL071C-B	2.80E-33
YJR005W	8.26E-33
YER075C	1.82E-38
YGL028C	4.91E-92
YML068W	3.79E-26
YOL016C	7.97E-27
YGR229C	2.66E-24
YLR419W	6.24E-26
YOR140W	2.64E-43
YPR007C	1.23E-23
YJR102C	1.52E-25
YMR296C	4.43E-23
YMR127C	1.64E-25
YGL064C	8.13E-27
YOR034C-A	1.99E-23
YER001W	1.97E-31
YGR147C	3.22E-30
YIL131C	1.02E-27
YNL173C	9.52E-27
YPR138C	8.41E-36
YBR170C	1.90E-47
YKR097W	1.55E-26
YBR148W	6.03E-82
YHR028C	2.08E-25
YGL258W-A	3.89E-29
YOR230W	9.18E-22
YBR156C	3.85E-45
YER118C	2.88E-53
YDL156W	8.25E-23
YNR069C	1.11E-25
YGL139W	1.36E-37
YMR174C	1.66E-27
YOR111W	3.87E-33
YGR067C	5.82E-27
YKL182W	6.14E-26
YLR285C-A	8.97E-57

YHR107C	7.36E-38
YLR042C	1.86E-79
YMR129W	7.92E-30
YMR032W	9.72E-22
YMR173W	2.89E-24
YDL238C	1.24E-53
YJR043C	3.20E-27
YNL040W	8.09E-38
YKL179C	2.71E-29
YML109W	9.31E-33
YLL063C	4.93E-25
YDR425W	1.84E-29
YGL081W	2.24E-24
YPR105C	9.57E-38
YNL078W	4.59E-63
YBR117C	7.15E-53
YBR038W	3.18E-36
YEL015W	1.62E-23
YNL099C	7.74E-25
YEL004W	7.40E-27
YMR200W	3.64E-25
YOL007C	6.41E-33
YMR163C	3.21E-31
YOR129C	1.12E-46
YCL064C	7.05E-23
YIL123W	5.42E-30
YBR139W	9.05E-28
YBR287W	1.23E-25
YDL179W	3.28E-24
YDR022C	2.67E-24
YJR147W	1.12E-53
YPL158C	4.52E-30
YDL210W	1.04E-41
YLR080W	1.44E-38
YBR163W	5.24E-37
YER152C	4.06E-88
YOR315W	5.10E-40
YOR316C	7.33E-25
YOL115W	3.81E-26
YGR014W	1.10E-73
YDL078C	5.96E-28
YJR153W	6.03E-33
YBL085W	1.82E-34
YOL052C	2.13E-30
YJL160C	3.24E-22
YBR192W	7.07E-29
YGL105W	1.36E-27

YFR028C	1.13E-32
YLR079W	7.57E-54
YBR098W	2.74E-53
YOR247W	3.52E-65
YPR194C	7.78E-31
YER113C	4.01E-23
YIR028W	5.39E-44
YLR095C	1.41E-23
YLR258W	8.91E-31
YAL041W	2.02E-34
YBR193C	1.44E-65
YBR150C	2.59E-60
YOR342C	8.38E-50
YDR333C	1.96E-32
YAL024C	4.06E-36
YJL078C	1.14E-90
YIL106W	1.17E-22
YLR164W	8.79E-30
YLR114C	6.17E-24
YNL168C	1.59E-45
YPL123C	1.63E-31
YKR061W	3.56E-22
YMR285C	1.05E-28
YKR013W	3.99E-49
YDR242W	4.68E-45
YPL085W	1.54E-24
YER119C	1.63E-26
YHR143W	2.93E-93
YLR053C	1.09E-30
YBR158W	2.11E-89
YBR119W	2.80E-65
YDR093W	1.39E-24
YOR116C	3.11E-28
YPL082C	4.09E-39
YGR044C	8.92E-65

Hot Spot 3:90000

Accession ID	p-value
YDR481C	1.32E-22
YOR271C	1.62E-51
YCL018W	1.57E-88
YDL103C	5.96E-22
YIL121W	2.00E-24
YER073W	5.17E-32
YKR071C	1.63E-36
YLR355C	1.30E-41
YJR016C	1.18E-53
YCL017C	6.35E-76

YLR348C	9.57E-75
YCR018C	7.45E-68
YBR068C	6.70E-49
YDR047W	1.15E-22
YHR047C	2.14E-28
YGR230W	9.77E-23
YKR095W-A	2.42E-37
YOR226C	5.10E-47
YCL026C-B	1.15E-83
YGL009C	3.11E-80
YOR227W	5.01E-23
YKL120W	8.88E-70
YOR375C	1.29E-47
YHR208W	4.61E-84
YCL004W	3.73E-44
YCL021W-A	1.15E-92
YCL016C	2.06E-46
YMR108W	9.33E-48

Hot Spot 3:170000

Accession ID	p-value
YCL068C	1.94E-62
YKL208W	1.62E-37
YDR460W	3.26E-84
YGL089C	5.12E-97
YLR040C	5.12E-97
YKL178C	5.12E-97
YIL017C	3.86E-55
YKR027W	2.49E-29
YNL146C-A	5.12E-97
YFL026W	5.12E-97
YKL209C	5.12E-97
YJR004C	2.88E-95
YPL187W	5.12E-97
YGL033W	1.68E-24
YFL027C	2.11E-94
YNL146W	3.75E-76
YCR096C	3.61E-91
YNL145W	5.12E-97
YDR461W	5.12E-97
YGL032C	5.12E-97
YJL170C	6.61E-97
YIL015W	5.12E-97
YCL066W	5.12E-97
YCR038C	2.62E-80
YJL171C	4.46E-40
YCR097W	5.12E-97

Hot Spot 8:110000

Accession ID	p-value
YHL010C	6.17E-35
YHR152W	5.75E-31
YFL047W	1.83E-21
YHR005C	1.70E-49
YLR433C	1.72E-25
YMR274C	1.48E-28
YPL141C	2.00E-23
YNL212W	2.74E-26
YOL133W	1.16E-24
YHL006C	1.54E-49
YCL027W	3.59E-77
YBL016W	2.56E-48
YBR040W	1.49E-60
YPR122W	3.57E-30
YHL009C	3.01E-24
YER016W	4.63E-38
YPL192C	1.55E-54
YGR109W-A	9.17E-23
YIL016W	4.67E-26
YHL012W	6.46E-43
YBR057C	1.69E-24
YDL127W	2.06E-26
YIL037C	2.96E-58
YLR442C	1.20E-26
YCR089W	3.86E-66
YNL279W	2.19E-54
YBL052C	1.18E-24
YGL116W	4.07E-33
YNL326C	8.23E-31
YGL223C	6.09E-27
YPR115W	8.86E-42
YHL008C	1.64E-34
YHR007C	8.06E-35
YMR065W	1.13E-62
YHL009W-A	4.03E-47
YMR232W	6.68E-60
YDR304C	7.40E-24
YHR061C	9.92E-32
YDR124W	3.56E-27
YKL189W	2.53E-35
YHR084W	8.74E-41
YOR212W	1.50E-29
YGL060W	3.06E-48
YER155C	7.72E-41
YGR109W-B	4.79E-23
YIL082W-A	7.70E-24

YML047C	2.50E-64
YNL278W	1.88E-31
YGL106W	5.51E-36
YJL157C	1.15E-70
YHL016C	2.36E-64
YLR452C	1.42E-72
YGR047C	7.99E-24
YCL055W	7.11E-70
YDR085C	4.92E-32
YOR219C	4.15E-27
YNR044W	1.31E-74
YBR083W	3.75E-51
YHL003C	1.28E-56
YDR249C	4.49E-24
YHL009W-B	7.38E-49
YML046W	8.82E-43
YBR133C	5.95E-30

Hot Spot 12:670000

Accession ID	p-value
YNR074C	1.90E-48
YOR175C	2.27E-42
YML019W	1.81E-32
YDL086W	3.15E-81
YMR212C	1.38E-32
YMR220W	4.96E-71
YDR518W	6.70E-49
YLR299W	1.77E-23
YBR215W	4.70E-24
YDL236W	2.79E-38
YEL051W	6.72E-29
YML125C	1.26E-31
YLR256W	1.20E-82
YPR178W	1.46E-25
YOR321W	8.00E-39
YHR001W	7.43E-26
YJL100W	4.00E-42
YGL001C	1.34E-70
YLR265C	1.92E-83
YNR043W	1.78E-77
YPR062W	8.35E-32
YLR260W	4.33E-50
YOR003W	3.39E-39
YPL272C	1.14E-26
YKR088C	1.66E-29
YPR193C	7.23E-45
YLR237W	6.06E-40
YGR266W	1.02E-53

YER093C	5.14E-37
YFR033C	7.11E-48
YLR275W	4.45E-45
YML008C	1.67E-64
YMR298W	9.37E-37
YLR038C	5.90E-27
YIL121W	1.05E-94
YKL220C	3.71E-28
YJR134C	6.99E-24
YHR072W	9.74E-78
YMR202W	5.52E-87
YDR200C	5.58E-25
YML075C	1.44E-84
YLR231C	2.05E-69
YDR502C	1.64E-72
YNL111C	1.11E-65
YGL055W	1.18E-40
YOR099W	6.78E-30
YDR520C	4.65E-28
YBL067C	6.29E-28
YDR213W	4.62E-41
YGR089W	1.80E-33
YDR297W	1.63E-56
YPR151C	2.83E-76
YDL133W	1.10E-24
YDR186C	2.39E-35
YLR244C	3.57E-80
YEL047C	4.58E-40
YDR346C	2.14E-34
YLR371W	5.22E-29
YOR073W	1.55E-23
YMR208W	7.34E-81
YEL034W	2.07E-87
YGL160W	5.78E-62
YJL048C	1.69E-72
YDL093W	1.18E-55
YGL101W	4.10E-56
YBR067C	1.37E-53
YBR106W	5.87E-25
YKL080W	8.72E-29
YLR245C	4.19E-79
YPR086W	8.27E-24
YIR033W	1.24E-33
YAL028W	1.78E-44
YOL128C	2.74E-28
YOR010C	3.31E-39
YHR003C	6.32E-36

YOR034C	1.93E-31
YCR048W	1.16E-39
YBR183W	1.83E-40
YOR002W	8.70E-27
YNR010W	1.33E-31
YGL040C	1.36E-24
YLR205C	1.58E-72
YER014W	1.67E-63
YJR049C	8.77E-26
YJR048W	4.29E-65
YOL026C	1.08E-28
YPL159C	1.41E-28
YJL095W	3.53E-30
YHR008C	1.07E-34
YDR275W	2.89E-27
YBR005W	1.75E-29
YML028W	5.14E-23
YER122C	4.21E-38
YDR326C	1.05E-26
YJR126C	5.80E-27
YNR041C	6.59E-27
YDL084W	4.93E-28
YNL052W	8.05E-31
YLR276C	6.86E-24
YNR019W	8.24E-82
YIL154C	3.55E-29
YKL077W	5.17E-26
YKR046C	2.47E-82
YNR007C	7.61E-32
YJR104C	4.42E-29
YNL264C	2.10E-34
YPL041C	1.65E-29
YPL231W	4.27E-29
YMR223W	3.65E-28
YLR153C	1.11E-86
YMR038C	2.17E-45
YBR042C	7.10E-27
YOR334W	1.24E-32
YPL028W	2.37E-68
YOL135C	5.37E-31
YLR241W	1.22E-30
YDL073W	2.73E-22
YEL024W	4.82E-37
YNL003C	7.96E-44
YLR288C	1.09E-71
YDL178W	8.66E-30
YER043C	5.25E-35

YIL111W	1.14E-31
YDR226W	3.94E-26
YNL010W	5.92E-39
YBL098W	1.89E-27
YMR070W	2.47E-31
YHR190W	7.18E-66
YML126C	9.21E-87
YPR040W	1.54E-23
YGL191W	3.28E-39
YLR281C	5.83E-46
YDR284C	1.45E-33
YOR348C	1.96E-38
YLR100W	4.24E-52
YGL017W	3.03E-25
YBL043W	3.18E-33
YNL231C	1.06E-24
YGR131W	2.06E-26
YBR161W	1.43E-31
YDR011W	1.88E-24
YPL232W	8.53E-32
YMR100W	4.30E-34
YOR065W	1.86E-30
YHR179W	1.14E-45
YNL173C	3.26E-24
YBR085W	1.00E-31
YIL031W	8.14E-32
YKL182W	6.86E-26
YLR154C	2.76E-45
YOR085W	5.84E-31
YPR095C	1.20E-37
YLR380W	3.22E-27
YHR210C	7.49E-25
YIL007C	3.24E-45
YPR113W	4.65E-35
YDR218C	1.95E-31
YPL008W	3.68E-24
YHR039C	7.10E-62
YJL198W	5.91E-23
YGR138C	3.26E-30
YDR488C	6.49E-32
YGR203W	7.87E-24
YGR234W	2.20E-84
YNL280C	8.40E-37
YDR384C	4.49E-30
YPL229W	2.71E-27
YLR414C	5.83E-28
YNR060W	4.20E-32

YPR065W	9.91E-60
YJR051W	1.40E-26
YMR009W	9.34E-80
YOR237W	2.43E-59
YMR244W	8.06E-27
YMR134W	2.26E-85
YMR145C	5.37E-36
YCR069W	1.34E-45
YGL210W	1.84E-26
YML081W	1.96E-29
YNL156C	8.58E-73
YPL117C	1.21E-59
YER011W	5.90E-50
YJR105W	3.45E-56
YHR004C	4.44E-27
YDL100C	3.86E-30
YDL174C	8.78E-50
YDR044W	5.46E-55
YGL130W	1.44E-23
YER044C	5.65E-71
YLR425W	3.42E-25
YAL016W	3.07E-29
YGL056C	7.97E-32
YJR047C	7.22E-42
YAL039C	6.27E-28
YJL105W	6.07E-25
YLR272C	1.52E-23
YGR049W	1.46E-66
YIL106W	1.42E-24
YOR377W	9.44E-49
YLL012W	2.49E-38
YOR118W	1.74E-29
YGR060W	3.33E-36
YBR242W	1.59E-70
YHR048W	1.93E-55
YER053C-A	1.21E-78
YJL167W	1.35E-80
YMR015C	3.48E-65
YMR252C	3.59E-29
YNL158W	4.63E-26
YOR229W	2.94E-43

Hot Spot 13:70000

Accession ID	p-value
YDR481C	9.75E-59
YML113W	1.03E-28
YHR152W	2.05E-31
YJL186W	2.06E-29

YER053C	3.94E-27
YBR296C	4.10E-67
YML064C	4.17E-27
YHR124W	7.90E-27
YML091C	1.67E-43
YMR008C	1.18E-26
YHR097C	1.02E-31
YBR240C	1.78E-31
YML067C	9.18E-38
YHL018W	3.77E-27
YML123C	5.61E-88
YKL035W	4.63E-29
YGR168C	1.10E-26
YDL199C	2.51E-25
YIL073C	3.08E-33
YNL265C	5.68E-26
YNL217W	5.72E-36
YPL019C	5.39E-84
YGR208W	9.00E-23
YML131W	1.40E-25
YNL149C	2.14E-22
YPR191W	3.40E-29
YLR410W	6.35E-37
YML096W	1.73E-30
YAR008W	8.36E-30
YFL004W	1.04E-37
YNL045W	2.13E-31
YMR196W	4.04E-24
YGR233C	4.39E-72
YJL137C	1.10E-31
YML070W	4.77E-26
YKL070W	4.03E-24
YOL140W	6.93E-27
YML118W	3.74E-28
YFL018C	1.10E-26
YDR369C	2.30E-40
YPR086W	4.51E-28
YLR295C	6.19E-27
YML078W	5.76E-90
YOL069W	6.03E-25
YHR214C-E	9.16E-28
YFR044C	2.88E-31
YML087C	1.81E-46
YAR007C	1.73E-24
YCR037C	4.53E-54
YGL224C	5.35E-28
YDL004W	4.52E-29

YJL012C	4.84E-84
YLR355C	1.14E-37
YCL049C	7.96E-32
YIR027C	2.84E-25
YDR309C	3.19E-30
YGL187C	1.27E-25
YDR248C	4.85E-32
YOR066W	2.32E-30
YML076C	3.92E-56
YML066C	5.45E-82
YER173W	2.10E-24
YDR441C	1.57E-24
YNL148C	7.26E-27
YHR183W	1.38E-46
YBL045C	7.21E-25
YJL020C	3.37E-28
YGL223C	2.24E-25
YIL172C	2.11E-32
YHR136C	1.17E-84
YJL117W	2.55E-52
YBL008W	1.80E-23
YLR438W	4.25E-78
YCR004C	5.92E-27
YJL098W	1.02E-24
YHR163W	8.21E-31
YLR142W	1.64E-33
YOR163W	3.68E-27
YMR300C	3.94E-38
YOR317W	1.62E-25
YPL268W	8.24E-38
YHR162W	4.18E-25
YLR058C	3.76E-23
YML111W	9.81E-59
YOR374W	8.07E-45
YOR211C	1.67E-23
YML088W	2.52E-26
YLL041C	1.73E-25
YOR212W	9.42E-28
YIL051C	4.98E-42
YAR071W	3.39E-74
YML119W	5.18E-39
YKL141W	4.74E-32
YPL110C	3.43E-45
YKR093W	1.23E-30
YER057C	6.97E-30
YOR220W	3.48E-24
YMR120C	1.21E-26

YML085C	2.09E-77
YGL234W	8.60E-34
YPL061W	5.32E-36
YKL016C	5.69E-29
YCL064C	1.10E-30
YNL012W	3.13E-32
YPL111W	2.35E-50
YNR002C	5.01E-39
YKL051W	9.24E-45
YKR089C	8.52E-29
YBR208C	8.69E-31
YMR313C	1.12E-25
YPR139C	4.22E-32
YML104C	7.68E-33
YER072W	2.29E-83
YML079W	1.13E-27
YPL018W	4.34E-35
YKL079W	3.75E-23
YGL167C	2.12E-24
YMR316W	2.58E-31
YBR056W-A	1.10E-27
YPL063W	6.11E-26
YDR403W	5.50E-28
YDR482C	5.40E-53
YJL084C	2.34E-27
YPL004C	2.07E-25
YNL141W	1.35E-25
YDR298C	3.74E-28
YGL255W	6.04E-34
YBR093C	2.11E-79
YGR061C	1.71E-37
YLR126C	9.56E-22
YMR238W	2.65E-31
YFR006W	4.56E-25
YFR031C	4.30E-27
YDR281C	1.28E-71
YPL113C	8.03E-32
YPL270W	2.53E-25
YML029W	1.21E-28

Hot Spot 15:150000

Accession ID	p-value
YKR009C	1.58E-33
YML113W	1.32E-36
YIL157C	4.94E-41
YPL223C	3.00E-57
YKL138C	1.18E-29
YIL160C	7.06E-39

YDL048C	1.68E-43
YPL154C	1.89E-32
YBR238C	7.15E-26
YKL195W	3.44E-36
YBR149W	1.47E-39
YER035W	2.06E-31
YPL020C	1.94E-26
YJL185C	3.11E-33
YMR317W	9.71E-28
YIL125W	2.41E-34
YGR130C	9.12E-47
YDR447C	8.72E-29
YNR038W	1.17E-26
YKL096W	1.11E-48
YEL003W	3.22E-27
YHR198C	2.68E-42
YLR149C	3.33E-47
YHR197W	3.46E-29
YAL034C	5.42E-39
YOR084W	6.59E-36
YJL042W	2.81E-35
YOR187W	3.72E-43
YGL121C	3.63E-31
YBR285W	1.15E-34
YOL028C	6.03E-39
YKL192C	1.06E-34
YBR169C	4.82E-33
YKL167C	3.85E-41
YER053C	1.17E-43
YOL105C	3.89E-38
YMR227C	8.10E-24
YCL054W	4.08E-26
YOL077W-A	7.77E-39
YGR083C	1.70E-30
YDR231C	2.73E-32
YMR169C	1.02E-55
YOL071W	1.80E-36
YKL194C	6.99E-35
YHL032C	3.56E-45
YPL127C	1.27E-28
YBR295W	3.26E-33
YKL085W	6.52E-41
YMR128W	4.90E-26
YPR155C	1.19E-47
YNL055C	6.16E-31
YBR128C	2.35E-24
YCR094W	8.76E-25

YGR088W	1.09E-53
YMR135C	3.36E-50
YDL085W	1.89E-28
YPR184W	5.96E-50
YHR046C	5.03E-24
YPL078C	3.64E-39
YDL223C	3.85E-40
YDR516C	3.84E-29
YOL090W	8.79E-46
YOL036W	1.32E-24
YMR008C	3.01E-23
YDR079W	3.86E-34
YML125C	3.45E-28
YOR178C	3.03E-43
YHR097C	2.46E-42
YGR258C	8.81E-27
YER103W	8.97E-25
YJL205C	5.23E-30
YLR052W	2.51E-25
YNL011C	5.87E-24
YJR121W	1.88E-44
YEL036C	1.15E-26
YCR059C	4.48E-31
YHR087W	6.64E-50
YLL028W	9.18E-92
YPR055W	3.72E-26
YCR073C	3.29E-41
YOL109W	3.84E-47
YDR530C	7.09E-25
YKR056W	6.46E-22
YJL166W	1.84E-31
YER013W	9.14E-37
YGL013C	2.59E-25
YPR015C	9.32E-26
YPL097W	2.32E-25
YGL143C	7.41E-29
YMR280C	2.24E-41
YLR218C	1.21E-34
YJR032W	2.55E-31
YGR272C	8.90E-32
YOR028C	1.80E-42
YBR251W	7.99E-29
YKR044W	8.08E-36
YFR045W	5.60E-51
YPL017C	6.14E-43
YIL042C	6.61E-25
YKL035W	5.20E-41

YOL061W	2.77E-30
YBL068W	3.26E-41
YDL199C	2.13E-37
YFL030W	1.93E-47
YKL162C	8.58E-31
YDR100W	1.85E-30
YAL017W	2.80E-37
YOL097C	2.45E-39
YDR090C	3.26E-22
YOR228C	7.61E-30
YCR091W	2.44E-37
YKL037W	1.81E-31
YPL045W	1.94E-23
YMR239C	1.14E-33
YIL006W	1.53E-29
YDR175C	3.37E-26
YDR375C	1.67E-27
YDL060W	3.08E-23
YDL222C	5.66E-57
YNL073W	1.89E-31
YER093C-A	4.28E-37
YER170W	2.24E-26
YHR051W	3.03E-33
YDL031W	2.65E-23
YDR337W	6.01E-35
YML054C	8.64E-40
YKL103C	4.42E-28
YGL111W	1.89E-28
YBR023C	4.83E-32
YLR224W	3.04E-24
YNL217W	3.15E-32
YOR347C	4.74E-30
YDR349C	8.75E-38
YGL096W	1.28E-27
YKR049C	7.37E-42
YKR043C	2.19E-45
YGR053C	2.46E-34
YER168C	5.03E-32
YDR347W	7.01E-29
YOR246C	2.64E-37
YLR222C	2.51E-27
YGR166W	7.21E-34
YHR085W	1.90E-28
YHR062C	7.08E-27
YOR353C	2.01E-30
YFR039C	1.67E-27
YOL077C	8.23E-26

YDL025C	2.48E-23
YOR080W	2.80E-24
YBR131W	6.01E-34
YPR010C	2.56E-25
YMR010W	2.24E-27
YDR528W	2.45E-27
YPL132W	3.53E-48
YER088C	4.19E-53
YER101C	1.95E-37
YDR395W	8.95E-27
YOL104C	2.36E-91
YKL161C	1.91E-30
YDR306C	4.92E-24
YDR453C	6.16E-42
YPR191W	5.33E-32
YER150W	2.27E-51
YER066W	1.30E-27
YAL053W	2.56E-24
YCR095C	1.00E-27
YLR172C	2.12E-27
YDL130W-A	3.15E-37
YLR356W	2.09E-32
YER054C	4.93E-45
YOL022C	1.68E-34
YNL045W	2.02E-44
YCR057C	4.45E-28
YML025C	2.03E-31
YIL091C	5.26E-29
YNL252C	2.95E-30
YAL029C	1.20E-34
YJL131C	7.53E-37
YNL160W	2.31E-43
YKL072W	3.06E-43
YJL164C	6.23E-38
YMR196W	1.56E-47
YGR031W	1.76E-43
YPR060C	7.66E-35
YIR012W	2.44E-28
YKL084W	4.73E-28
YGR201C	1.65E-38
YPL043W	1.36E-23
YLR270W	3.22E-45
YIL070C	8.80E-27
YKL091C	1.53E-45
YKR016W	2.04E-36
YEL011W	2.24E-43
YPL196W	1.30E-32

YJR055W	9.19E-30
YJR110W	1.59E-24
YGR033C	4.64E-33
YHR030C	2.96E-24
YKL018C-A	1.81E-29
YGR217W	7.44E-34
YML030W	6.59E-43
YKR071C	8.34E-28
YCR102C	3.07E-28
YKL148C	1.02E-30
YDR178W	1.19E-40
YDR202C	2.66E-39
YJL137C	1.41E-35
YJR039W	3.75E-23
YMR244C-A	4.97E-41
YPR091C	9.54E-37
YJR097W	4.66E-34
YMR251W-A	1.03E-41
YER177W	2.03E-26
YHR207C	6.37E-42
YGR264C	2.48E-28
YJR059W	3.87E-29
YML070W	1.23E-30
YGR175C	3.21E-39
YKR026C	1.58E-29
YDR211W	2.91E-35
YCL035C	4.79E-32
YOL006C	6.93E-31
YLR251W	4.00E-29
YDL181W	1.39E-47
YNL224C	1.42E-26
YOR195W	5.61E-31
YDR276C	2.25E-23
YDR096W	8.94E-48
YKL062W	4.10E-57
YML108W	2.68E-33
YEL050C	4.39E-37
YDR504C	1.12E-23
YNL087W	2.21E-38
YDR405W	2.53E-25
YEL046C	4.84E-31
YIL105C	2.87E-34
YNL015W	3.54E-34
YJL163C	2.76E-34
YKL193C	2.14E-39
YNL108C	1.55E-29
YLR397C	6.26E-26

YHR176W	5.97E-30
YGL250W	1.07E-26
YOL130W	2.31E-33
YLR226W	3.08E-29
YBR262C	2.55E-28
YKR063C	4.18E-41
YOR120W	1.31E-42
YJR008W	4.66E-26
YMR319C	1.38E-26
YNR012W	3.45E-34
YBL013W	1.06E-48
YNR034W	5.72E-25
YBR269C	3.05E-34
YEL052W	2.86E-44
YEL055C	1.15E-33
YDL213C	4.76E-31
YGR052W	2.32E-67
YGL004C	1.27E-27
YDR430C	4.45E-34
YEL029C	3.69E-31
YBR003W	1.82E-34
YOR306C	7.10E-25
YOR040W	2.60E-27
YDR165W	1.99E-26
YMR229C	1.15E-24
YNL132W	1.07E-23
YMR264W	4.69E-24
YDR179W-A	2.07E-24
YLR069C	3.45E-30
YLR295C	1.65E-41
YPL173W	2.03E-28
YDL170W	1.19E-29
YHR077C	1.21E-28
YGR008C	5.63E-39
YDR060W	8.39E-32
YIL079C	1.43E-31
YPL118W	3.22E-33
YKL212W	2.38E-33
YJL014W	2.24E-30
YPR110C	1.07E-29
YLR014C	2.94E-23
YHR214C-E	4.66E-22
YEL026W	1.45E-27
YKL004W	4.31E-25
YBR037C	4.91E-36
YFR044C	3.90E-35
YDR032C	4.62E-37

YFR011C	2.09E-38
YKL100C	4.49E-36
YIL117C	9.59E-25
YLR382C	4.38E-33
YFR017C	1.46E-46
YER182W	1.87E-46
YML129C	1.37E-36
YPL183W-A	7.52E-37
YER162C	3.20E-26
YOR086C	2.15E-30
YMR185W	2.15E-29
YBR004C	3.79E-27
YOL080C	5.79E-26
YBR230C	6.77E-44
YNL098C	3.33E-41
YDR514C	3.88E-24
YGR112W	3.91E-40
YDR300C	1.11E-40
YKL096W-A	5.88E-52
YDR313C	4.46E-31
YJL141C	1.58E-42
YDL004W	1.14E-37
YPL030W	1.69E-34
YBR146W	3.68E-28
YKR065C	1.52E-33
YEL060C	2.98E-26
YOR115C	1.41E-24
YLR407W	3.51E-29
YGR086C	3.47E-57
YMR261C	1.92E-36
YJL103C	2.86E-38
YPL207W	6.42E-41
YLR446W	4.00E-30
YIR016W	6.79E-46
YOL084W	1.18E-75
YPL239W	5.86E-37
YFR055W	2.33E-33
YFR014C	6.83E-45
YHR195W	2.38E-33
YHL036W	6.33E-29
YIL097W	1.26E-27
YNR036C	4.06E-31
YMR114C	1.80E-34
YKR060W	3.58E-30
YLR193C	2.27E-32
YOR158W	1.66E-30
YOL131W	1.38E-28

YLL019C	4.45E-42
YLR070C	1.76E-33
YDR511W	7.39E-29
YLR395C	9.98E-35
YLL008W	1.80E-31
YNL093W	4.63E-33
YPR004C	1.59E-24
YGR271W	1.68E-23
YHR008C	1.23E-24
YMR175W	3.37E-33
YLR002C	4.59E-25
YDR098C	9.97E-35
YIL047C	3.14E-34
YMR031C	1.76E-61
YLR345W	1.59E-53
YDL121C	2.13E-23
YCR030C	2.32E-29
YOL092W	9.56E-81
YDR148C	8.02E-40
YNL195C	5.67E-30
YGR111W	1.37E-31
YCL057C-A	2.10E-40
YDR303C	3.32E-30
YDR436W	1.54E-31
YOL048C	2.53E-36
YDR326C	3.08E-22
YDR185C	1.10E-35
YOL052C-A	9.28E-51
YLR392C	2.53E-32
YER002W	3.76E-26
YGL187C	7.20E-33
YLR177W	4.64E-44
YNL052W	1.11E-27
YMR110C	1.16E-26
YBL035C	1.94E-28
YJL066C	4.02E-39
YMR250W	9.67E-48
YNR033W	1.06E-35
YGR062C	5.00E-29
YGR248W	2.97E-41
YLR196W	1.22E-25
YLL026W	8.74E-37
YFR007W	8.50E-29
YNL200C	3.39E-42
YOR354C	7.34E-32
YNL100W	7.03E-43
YHR020W	1.86E-29

YER045C	3.05E-35
YDR533C	3.73E-89
YDL214C	4.98E-28
YBR104W	7.08E-30
YML100W	2.56E-51
YGR043C	6.25E-50
YOR386W	6.30E-31
YIL033C	1.31E-41
YPL011C	6.95E-25
YMR148W	2.55E-41
YNL284C	1.49E-27
YLR102C	5.59E-36
YJR104C	5.47E-23
YOR107W	1.77E-25
YBR214W	6.96E-31
YML056C	2.27E-30
YGR187C	6.72E-30
YHR092C	6.27E-27
YML128C	2.53E-50
YLL018C-A	4.83E-44
YJR003C	1.02E-47
YHR110W	6.67E-27
YPR061C	2.44E-25
YOR127W	6.28E-25
YGR128C	5.07E-30
YOR173W	1.22E-48
YCL051W	2.02E-23
YOR207C	7.56E-35
YDL203C	8.80E-27
YBR155W	1.66E-29
YDR351W	2.66E-31
YAL008W	1.60E-29
YPL266W	1.72E-28
YHR068W	3.36E-27
YBL045C	6.40E-30
YPR009W	6.85E-24
YDR462W	3.13E-29
YPL230W	1.43E-43
YLR203C	4.33E-40
YBL064C	1.29E-35
YHR187W	1.97E-27
YOR233W	6.46E-28
YPL171C	4.29E-41
YGL120C	5.69E-23
YGR271C-A	3.10E-31
YFR013W	1.44E-29
YKL046C	1.10E-29

YKL109W	6.02E-39
YDL051W	1.81E-31
YDR047W	2.52E-35
YHR007C	9.20E-31
YCR004C	7.57E-39
YDR161W	2.47E-26
YCR051W	1.89E-25
YLL035W	9.49E-31
YDL073W	1.25E-30
YDR342C	2.57E-56
YEL024W	1.15E-30
YJR132W	1.56E-33
YLR293C	1.36E-22
YPL109C	2.44E-26
YPR161C	7.69E-30
YHR009C	2.13E-32
YLL001W	6.16E-32
YDL022W	1.96E-41
YIL124W	6.31E-41
YML120C	3.72E-36
YDL183C	1.12E-35
YDL019C	2.76E-62
YMR308C	3.54E-27
YFL048C	4.98E-25
YOR243C	6.98E-29
YPL172C	4.26E-30
YOL119C	5.80E-44
YJL210W	7.39E-44
YOR260W	3.43E-25
YNL194C	5.31E-48
YML071C	5.67E-28
YHL021C	2.02E-28
YMR272C	3.15E-68
YKL026C	5.79E-40
YMR293C	2.88E-26
YLR084C	3.49E-31
YPL098C	1.01E-24
YMR090W	2.48E-44
YHL039W	5.84E-25
YIL077C	3.00E-47
YMR002W	1.34E-25
YMR136W	7.24E-53
YMR041C	2.33E-31
YIL136W	3.43E-53
YPL271W	1.64E-36
YBR039W	3.21E-27
YFR053C	1.91E-39

YOR317W	2.02E-29
YPL087W	9.73E-27
YGL146C	7.22E-40
YOR254C	7.10E-24
YER110C	8.89E-26
YPL160W	1.46E-27
YBR001C	7.48E-35
YOR358W	4.48E-36
YHL011C	1.47E-32
YLR409C	1.99E-26
YJR077C	1.43E-34
YGL208W	4.11E-24
YAL061W	4.25E-35
YDR358W	1.43E-26
YDR216W	2.15E-52
YDL110C	2.70E-33
YKL151C	1.28E-39
YDR204W	1.09E-40
YML081C-A	2.63E-35
YPL053C	4.03E-29
YPL222W	3.71E-36
YFL016C	5.40E-24
YOR348C	2.80E-31
YPL247C	1.36E-52
YDR256C	5.99E-30
YKR006C	1.35E-30
YLR143W	1.73E-37
YOR176W	9.61E-27
YDR021W	8.60E-28
YBL029C-A	4.62E-38
YBL099W	1.06E-35
YDL036C	1.24E-27
YOL113W	2.77E-36
YMR267W	1.27E-39
YMR181C	5.52E-45
YDR464W	4.79E-24
YDL021W	3.76E-36
YDR074W	2.89E-47
YKL117W	7.96E-25
YDR171W	2.20E-41
YDR322W	2.31E-30
YKL093W	1.30E-27
YLR332W	1.40E-27
YKL142W	2.33E-38
YAL040C	3.99E-30
YJR041C	5.07E-33
YOR374W	4.23E-35

YOL095C	8.98E-52
YKR058W	3.24E-32
YFL042C	1.33E-29
YGR236C	2.59E-37
YPL040C	3.55E-31
YOR226C	4.27E-29
YOL029C	2.81E-42
YLR419W	2.37E-25
YLR449W	3.14E-31
YIR038C	1.05E-32
YBR261C	3.43E-31
YPL104W	2.89E-43
YOR048C	1.59E-30
YPR172W	1.28E-33
YHR080C	6.10E-48
YLR146C	9.60E-30
YNL131W	6.05E-27
YOR286W	7.59E-32
YOL056W	1.38E-37
YGL037C	7.95E-45
YOR065W	2.99E-27
YPL236C	9.46E-31
YER001W	1.07E-28
YPL052W	4.10E-39
YLL041C	7.02E-41
YGR237C	1.51E-31
YLR439W	2.69E-29
YPR026W	1.03E-39
YML073C	4.56E-23
YNL274C	4.65E-41
YGL227W	5.29E-29
YLR228C	1.51E-29
YNL173C	4.78E-25
YKL052C	7.79E-67
YJR080C	1.72E-39
YFL038C	1.13E-25
YOL089C	1.61E-54
YBR052C	1.68E-40
YMR312W	7.43E-30
YJL010C	8.75E-30
YNL134C	1.23E-77
YGR127W	2.00E-34
YBL087C	7.79E-24
YGR174C	1.53E-42
YJR019C	1.78E-32
YDR529C	1.87E-25
YKL141W	5.29E-43

YNL137C	3.44E-29
YKR093W	1.50E-41
YOR186W	9.54E-28
YMR174C	7.92E-29
YLR219W	1.04E-50
YNL227C	8.92E-28
YER130C	6.77E-28
YOR215C	6.89E-31
YOL091W	6.44E-65
YOR220W	1.79E-28
YIL067C	2.39E-25
YHR010W	7.80E-25
YIL155C	3.25E-41
YGR243W	8.35E-47
YBR034C	1.92E-28
YGR102C	1.19E-32
YJL005W	1.40E-33
YML004C	7.35E-23
YER082C	1.45E-36
YLR066W	5.35E-26
YML018C	2.88E-32
YLL021W	2.49E-28
YLR129W	1.43E-27
YOL116W	3.11E-34
YOR051C	1.23E-25
YIL101C	1.65E-38
YDR179C	1.08E-26
YNL223W	1.73E-25
YMR072W	9.04E-25
YER067W	9.36E-34
YER049W	2.69E-32
YOL073C	2.05E-32
YJL062W-A	3.19E-34
YLR128W	5.26E-24
YHR210C	3.38E-27
YBL030C	1.48E-41
YOR227W	8.02E-40
YPL014W	7.96E-33
YJR127C	9.55E-51
YCR046C	5.50E-41
YNR059W	4.93E-27
YDR277C	5.33E-31
YMR194C-B	6.68E-47
YGR118W	1.29E-23
YHR016C	7.60E-40
YOR142W	1.37E-31
YKR076W	1.61E-34

YKR064W	9.33E-25
YGR021W	9.80E-40
YHR064C	2.92E-26
YNL092W	2.88E-24
YMR297W	2.17E-28
YHR017W	5.89E-31
YMR157C	1.84E-32
YNL133C	2.08E-42
YDR034W-B	1.22E-28
YHR022C	6.65E-27
YDR296W	6.60E-37
YOR241W	1.06E-39
YOL114C	1.10E-24
YOR001W	4.62E-29
YOR161C	1.47E-50
YMR286W	1.68E-35
YLR327C	9.01E-35
YOR224C	2.26E-26
YDL079C	2.40E-27
YCR043C	1.87E-35
YMR177W	7.41E-36
YOR274W	2.28E-29
YIL087C	1.02E-29
YNR060W	8.48E-28
YGR244C	4.97E-50
YPR137W	3.84E-26
YIL008W	1.77E-30
YDL211C	1.32E-22
YGL010W	3.80E-31
YBL086C	8.70E-34
YHR111W	1.82E-35
YIL114C	1.37E-27
YPL088W	4.82E-28
YHL004W	5.21E-34
YKL016C	6.24E-37
YJR063W	3.54E-26
YKR079C	2.10E-35
YJL129C	5.59E-29
YBR162W-A	5.93E-28
YBR007C	8.50E-31
YMR145C	9.19E-28
YOL054W	2.04E-33
YML050W	4.07E-38
YDR420W	3.38E-29
YGL145W	3.53E-36
YKL150W	4.83E-39
YIL098C	6.79E-28

YGR194C	7.12E-44
YLR390W	2.15E-28
YLL034C	1.73E-27
YML110C	1.21E-35
YHR096C	9.02E-42
YPR033C	2.79E-29
YNR002C	4.58E-47
YDR197W	2.76E-40
YAR029W	4.26E-29
YGR035C	1.75E-35
YIL036W	1.03E-29
YKL051W	8.97E-45
YOR185C	8.98E-44
YNL234W	3.48E-38
YHR144C	1.92E-29
YOL094C	3.52E-40
YJL168C	4.22E-24
YDR198C	2.26E-34
YJL011C	4.37E-31
YDR258C	1.62E-27
YMR105C	8.59E-46
YOR289W	1.61E-27
YJR096W	2.89E-36
YKL014C	1.55E-25
YDR406W	2.04E-45
YBR185C	4.69E-31
YDR324C	1.10E-27
YER062C	3.24E-53
YJR011C	2.87E-32
YPL186C	8.29E-47
YJL069C	7.92E-29
YLR258W	6.99E-38
YCR072C	1.18E-26
YGL130W	4.46E-26
YCL040W	4.86E-37
YOL088C	2.42E-31
YBL061C	2.19E-40
YDR329C	6.46E-32
YER060W	1.35E-27
YGL237C	2.29E-39
YDL204W	2.80E-50
YHR104W	4.64E-45
YBR177C	3.72E-51
YPR143W	2.09E-24
YOL100W	3.44E-42
YNL256W	2.57E-35
YBR147W	4.27E-25

YDL027C	1.53E-27
YBR126C	2.38E-46
YDL124W	4.01E-36
YDR135C	3.97E-29
YGR195W	9.59E-30
YNR014W	9.56E-63
YGL151W	1.85E-22
YGR231C	5.67E-32
YLR178C	2.15E-58
YEL040W	4.99E-27
YOL156W	3.72E-25
YHR160C	3.29E-30
YGR207C	3.12E-39
YDR070C	3.90E-48
YBR092C	7.83E-27
YBR030W	2.51E-29
YCR028C	1.31E-24
YJL112W	1.99E-25
YIL020C	9.78E-28
YML006C	8.05E-25
YGR134W	1.97E-24
YGR255C	2.12E-36
YCR035C	8.37E-28
YER058W	8.95E-26
YFL014W	3.03E-65
YLL012W	2.68E-32
YPR160W	4.36E-46
YLR121C	8.13E-31
YMR160W	1.33E-27
YNR034W-A	3.04E-42
YPL004C	1.58E-47
YLR201C	2.13E-32
YOL096C	1.24E-48
YDR298C	5.39E-40
YMR139W	8.56E-27
YPR149W	2.54E-51
YGL255W	8.63E-33
YFR049W	7.19E-30
YMR238W	1.03E-23
YGR165W	3.54E-25
YPL203W	2.33E-29
YMR195W	1.47E-37
YGR094W	2.88E-26
YDR434W	8.74E-29
YFR006W	3.52E-45
YML082W	9.62E-33
YFL041W-A	2.15E-27

YPL113C	1.16E-36
YIL145C	8.29E-36
YER132C	1.20E-25
YGR070W	1.13E-34
YOL082W	9.52E-56
Hot Spot 16:510000	
Accession ID	p-value
YJL147C	2.21E-27
YPL078C	3.14E-25
YML124C	8.97E-27
YML125C	1.45E-25
YPR015C	7.05E-23
YPL036W	1.13E-47
YHR051W	1.46E-25
YPL107W	2.41E-27
YPL026C	2.82E-23
YJR152W	7.24E-28
YPR020W	6.85E-40
YJR138W	1.72E-25
YDL181W	6.84E-27
YKL062W	1.49E-25
YPL016W	4.56E-77
YIL030C	9.46E-26
YOR034C	9.97E-25
YHR037W	1.44E-41
YOR278W	2.17E-28
YPL024W	1.88E-51
YDL004W	1.08E-26
YOR007C	8.32E-23
YPL023C	1.03E-43
YLR395C	3.53E-30
YPR004C	7.13E-23
YIR029W	2.83E-28
YGL187C	1.26E-31
YPL031C	5.53E-27
YMR155W	7.93E-24
YLR142W	2.68E-32
YJL210W	8.27E-30
YPL022W	1.10E-38
YLR084C	1.14E-26
YMR041C	9.08E-23
YPL271W	6.12E-27
YOR348C	5.77E-33
YPL038W-A	1.05E-63
YOR065W	1.63E-26
YBR170C	4.17E-30
YGR138C	1.45E-30

YOR142W	1.05E-23
YCL025C	2.57E-29
YPL038W	1.73E-32
YJR047C	4.45E-28
YPL039W	8.93E-75
YDR298C	2.13E-31
YMR195W	7.75E-24
YFR006W	8.58E-29

Supplementary Table 11: causality test results for TGCT

HotSpot(Chr:Pos)	Regulator	Target	BIC_causal	BIC_partial	BIC_independent
2:550000	YBR149W	YER059W	-482.253	-482.249	-343.431
2:550000	YBR149W	YLR286C	-411.208	-407.179	-393.692
2:550000	YBR149W	YMR182C	26.7719	37.3243	96.1602
2:550000	YBR149W	YKL166C	-169.713	-162.608	-120.717
2:550000	YBR149W	YBR194W	-516.055	-515.504	-502.558
2:550000	YBR149W	YBR285W	703.919	712.706	732.554
2:550000	YBR149W	YBR157C	601.938	608.644	659.858
2:550000	YBR149W	YPL101W	-612.362	-602.871	-573.167
2:550000	YBR149W	YOR264W	-648.454	-636.549	-642.467
2:550000	YBR149W	YDR006C	-819.658	-818.609	-749.297
2:550000	YBR149W	YGL196W	-306.756	-304.271	-193.926
2:550000	YBR149W	YNL055C	-1196.91	-1188.5	-1192.58
2:550000	YBR149W	YNR067C	-599.635	-587.835	-588.208
2:550000	YBR149W	YIL009W	-492.666	-482.727	-488.174
2:550000	YBR149W	YOL112W	-535.44	-530.069	-488.057
2:550000	YBR149W	YAR014C	-599.121	-592.113	-330.921
2:550000	YBR149W	YDR090C	-72.2436	-68.6523	72.9335
2:550000	YBR149W	YDR234W	521.453	531.338	558.947
2:550000	YBR149W	YIR030C	793.682	805.435	922.995
2:550000	YBR149W	YKL103C	80.1017	80.5975	91.5384
2:550000	YBR149W	YPL066W	-798.424	-790.738	-796.58
2:550000	YBR149W	YPR005C	109.237	114.205	196.22
2:550000	YBR149W	YOR246C	-987.768	-978.746	-959.988
2:550000	YBR149W	YGR208W	-620.627	-609.443	-540.883
2:550000	YBR149W	YER089C	-874.327	-870.648	-768.57
2:550000	YBR149W	YBR154C	270.862	272.821	291.638
2:550000	YBR149W	YGL062W	-988.554	-979.765	-966.464
2:550000	YBR149W	YMR170C	371.993	374.43	374.632
2:550000	YBR149W	YGL229C	92.2785	94.8378	140.653
2:550000	YBR149W	YML118W	100.667	107.422	101.936
2:550000	YBR149W	YBR173C	-340.079	-333.936	-212.483
2:550000	YBR149W	YJR008W	90.4135	92.7508	117.356
2:550000	YBR149W	YCR099C	240.542	247.8	246.337
2:550000	YBR149W	YMR205C	-1070.18	-1067.62	-1069.25
2:550000	YBR149W	YMR236W	-997.938	-992.167	-872.797
2:550000	YBR149W	YGR281W	-416.781	-404.476	-302.513
2:550000	YBR149W	YOR126C	-620.101	-609.153	-540.693
2:550000	YBR149W	YNL283C	-538.118	-527.747	-531.941
2:550000	YBR149W	YLR446W	243.244	252.181	278.459
2:550000	YBR149W	YLR353W	-695.948	-683.909	-602.367
2:550000	YBR149W	YJL145W	-535.739	-530.419	-493.496
2:550000	YBR149W	YGR019W	79.4863	87.447	122.724
2:550000	YBR149W	YNL268W	-905.659	-894.383	-871.103
2:550000	YBR149W	YDR098C	-1072.77	-1065.73	-1071.72
2:550000	YBR149W	YIL047C	-241.404	-232.214	-64.5592

2:550000	YBR149W	YJR001W	-970.022	-966.802	-918.638
2:550000	YBR149W	YHR117W	-743.51	-731.545	-728.233
2:550000	YBR149W	YBR104W	334.494	339.728	539.652
2:550000	YBR149W	YLR421C	-892.84	-883.122	-867.9
2:550000	YBR149W	YOR386W	127.047	131.009	158.856
2:550000	YBR149W	YJL116C	294.253	303.836	449.97
2:550000	YBR149W	YPR144C	468.023	468.092	485.871
2:550000	YBR149W	YBR115C	-41.1501	-28.9139	-17.5223
2:550000	YBR149W	YGR040W	-368.592	-362.003	-349.693
2:550000	YBR149W	YIL089W	129.535	133.163	152.774
2:550000	YBR149W	YPL171C	102.082	104.79	102.806
2:550000	YBR149W	YHR007C	-890.402	-885.733	-814.243
2:550000	YBR149W	YBR107C	-519.088	-512.974	-448.749
2:550000	YBR149W	YLR084C	-392.372	-380.136	-290.525
2:550000	YBR149W	YPL163C	-493.861	-485.395	-491.322
2:550000	YBR149W	YMR181C	59.6949	64.9783	61.4629
2:550000	YBR149W	YDR362C	-695.328	-692.854	-693.188
2:550000	YBR149W	YGL209W	345.133	354.261	411.8
2:550000	YBR149W	YBL071C-B	-324.483	-312.611	-282.669
2:550000	YBR149W	YFL021W	212.484	222.513	578.986
2:550000	YBR149W	YOR286W	-349.712	-348.441	-345.948
2:550000	YBR149W	YJR133W	-256.562	-251.557	-208.297
2:550000	YBR149W	YLR285C-A	-55.018	-44.3466	-47.333
2:550000	YBR149W	YLR042C	85.6365	91.0803	137.147
2:550000	YBR149W	YMR129W	-703.65	-701.995	-702.671
2:550000	YBR149W	YMR173W	-535.052	-527.513	-531.169
2:550000	YBR149W	YER145C	-101.101	-98.5201	-78.1947
2:550000	YBR149W	YCR086W	-265.321	-258.619	-84.1924
2:550000	YBR149W	YGL081W	113.112	123.187	214.718
2:550000	YBR149W	YPR105C	-829.103	-824.388	-762.698
2:550000	YBR149W	YNL078W	-483.807	-472.335	-450.587
2:550000	YBR149W	YHL016C	999.399	1008.4	1075.18
2:550000	YBR149W	YEL004W	-596.859	-596.287	-543.128
2:550000	YBR149W	YPR074C	-1226.34	-1214.73	-1180.5
2:550000	YBR149W	YDL179W	-177.224	-165.317	-169.518
2:550000	YBR149W	YPL158C	-535.643	-526.494	-530.846
2:550000	YBR149W	YBR111W-A	76.9249	85.9445	100.805
2:550000	YBR149W	YOR316C	-730.532	-720.084	-630.357
2:550000	YBR149W	YJR096W	283.78	296.044	336.4
2:550000	YBR149W	YMR013C	-381.927	-375.983	-372.82
2:550000	YBR149W	YLR079W	-779.31	-767.659	-742.511
2:550000	YBR149W	YOR247W	-1062.98	-1062.79	-934.936
2:550000	YBR149W	YPR194C	1251.72	1253.27	1375.63
2:550000	YBR149W	YLR258W	761.666	764.856	825.8
2:550000	YBR149W	YAL041W	-769.611	-765.95	-766.261
2:550000	YBR149W	YKL068W-A	418.6	418.819	421.563
2:550000	YBR149W	YDR333C	-509.569	-505.047	-507.609

2:550000	YBR149W	YAL024C	-505.629	-499.897	-387.865
2:550000	YBR149W	YAL039C	-111.232	-107.19	-89.1917
2:550000	YBR149W	YJL110C	47.9975	51.0998	48.4962
2:550000	YBR149W	YBR119W	-207.985	-196.304	-197.374
2:550000	YBR149W	YOL082W	80.0812	89.3325	99.3885
2:550000	YBR149W	YGR044C	-111.516	-103.125	-96.6237
2:550000	YBR157C	YJL147C	-239.833	-230.837	-196.454
2:550000	YBR157C	YDL159W	-779.568	-770.746	-756.36
2:550000	YBR157C	YNL216W	-399.11	-398.42	-393.466
2:550000	YBR157C	YEL006W	-431.178	-428.966	-393.455
2:550000	YBR157C	YBR149W	-699.806	-696.165	-669.411
2:550000	YBR157C	YPL264C	693.533	694.855	749.648
2:550000	YBR157C	YBR194W	-512.285	-508.364	-510.5
2:550000	YBR157C	YOR264W	-657.803	-646.618	-651.252
2:550000	YBR157C	YDL061C	-790.048	-785.229	-783.671
2:550000	YBR157C	YDR210W	-517.812	-512.977	-516.569
2:550000	YBR157C	YBR084C-A	-343.793	-332.398	-323.031
2:550000	YBR157C	YAL003W	-711.794	-704.428	-697.112
2:550000	YBR157C	YDL012C	-465.243	-453.543	-427.921
2:550000	YBR157C	YLR029C	-996.783	-984.52	-981.419
2:550000	YBR157C	YOR338W	754.591	764.972	797.189
2:550000	YBR157C	YIL052C	-461.736	-449.671	-447.651
2:550000	YBR157C	YOR179C	-465.681	-454.377	-433.091
2:550000	YBR157C	YNL055C	-1213.13	-1208.43	-1204.67
2:550000	YBR157C	YPL131W	-770.39	-762.828	-760.957
2:550000	YBR157C	YJL200C	776.926	788.049	782.556
2:550000	YBR157C	YNR067C	-599.698	-587.556	-593.391
2:550000	YBR157C	YDL055C	-1148.8	-1146.09	-1148.11
2:550000	YBR157C	YMR306W	-20.1668	-10.2843	15.9914
2:550000	YBR157C	YHL001W	-212.555	-200.431	-169.59
2:550000	YBR157C	YDL218W	221.769	230.467	291.195
2:550000	YBR157C	YJR123W	-1010.03	-999.672	-1002.61
2:550000	YBR157C	YGR027C	-1042.58	-1030.89	-1031.58
2:550000	YBR157C	YDL229W	17.4228	26.7667	24.6507
2:550000	YBR157C	YDL155W	-439.088	-433.78	-383.682
2:550000	YBR157C	YMR088C	160.163	172.193	235.82
2:550000	YBR157C	YKL180W	-456.101	-445.836	-445.774
2:550000	YBR157C	YIR034C	391.255	395.984	482.985
2:550000	YBR157C	YLR185W	-360.596	-348.937	-348.923
2:550000	YBR157C	YPL081W	591.28	593.22	592.137
2:550000	YBR157C	YHR038W	410.246	417.801	418.777
2:550000	YBR157C	YKL081W	-678.441	-666.892	-650.901
2:550000	YBR157C	YCR073C	755.778	758.953	813.25
2:550000	YBR157C	YOL112W	-496.724	-489.904	-495.8
2:550000	YBR157C	YIL094C	-355.463	-346.373	-325.886
2:550000	YBR157C	YBL072C	-1338.63	-1329.19	-1309.62
2:550000	YBR157C	YBL027W	-414.751	-406.503	-406.737

2:550000	YBR157C	YER102W	-628.927	-623.866	-617.109
2:550000	YBR157C	YDL057W	50.5265	59.9565	86.5576
2:550000	YBR157C	YKR057W	-243.575	-233.628	-233.439
2:550000	YBR157C	YGR085C	-86.3108	-74.2276	-52.5588
2:550000	YBR157C	YGR148C	-1021.66	-1013.42	-1013.05
2:550000	YBR157C	YGR214W	-235.793	-227.095	-226.17
2:550000	YBR157C	YDR345C	-177.248	-172.301	-132.611
2:550000	YBR157C	YBR197C	90.7113	101.78	148.537
2:550000	YBR157C	YOR003W	-71.0372	-61.0672	-11.6305
2:550000	YBR157C	YIL009C-A	-295.977	-291.443	-264.843
2:550000	YBR157C	YMR142C	-750.607	-741.81	-743.363
2:550000	YBR157C	YDL229W	-441.549	-435.985	-437.32
2:550000	YBR157C	YDR064W	-1021.72	-1019.53	-1020.4
2:550000	YBR157C	YER064C	855.928	863.671	986.926
2:550000	YBR157C	YPR163C	129.159	137.572	186.768
2:550000	YBR157C	YDR234W	539.06	549.373	568.853
2:550000	YBR157C	YKL112W	-572.879	-567.23	-565.829
2:550000	YBR157C	YIR030C	923.567	928.773	930.47
2:550000	YBR157C	YOL120C	-521.072	-514.892	-515.382
2:550000	YBR157C	YPR181C	-964.932	-953.331	-951.961
2:550000	YBR157C	YPL107W	-212.639	-208.611	-185.009
2:550000	YBR157C	YLR249W	-359.249	-347.348	-344.786
2:550000	YBR157C	YPL066W	-813.537	-805.074	-806.418
2:550000	YBR157C	YER036C	13.971	22.1766	65.2264
2:550000	YBR157C	YBL049W	308.581	318.214	358.761
2:550000	YBR157C	YBR079C	199.798	210	249.575
2:550000	YBR157C	YAL035W	-223.625	-221.025	-155.798
2:550000	YBR157C	YGL189C	-959.084	-950.533	-947.822
2:550000	YBR157C	YIL069C	256.599	266.007	276.942
2:550000	YBR157C	YPR043W	-1158.33	-1147.99	-1150.34
2:550000	YBR157C	YER089C	-786.739	-775.272	-780.376
2:550000	YBR157C	YPL132W	16.5976	24.8972	21.5996
2:550000	YBR157C	YOL127W	-1119.84	-1109.6	-1110.48
2:550000	YBR157C	YOR061W	-908.103	-896.635	-841.669
2:550000	YBR157C	YPR162C	-519.199	-510.971	-442.259
2:550000	YBR157C	YDL182W	154.959	162.377	214.159
2:550000	YBR157C	YMR180C	-51.7219	-42.0614	7.98762
2:550000	YBR157C	YDL095W	-824.472	-813.66	-810.663
2:550000	YBR157C	YNL240C	670.68	682.065	700.645
2:550000	YBR157C	YBR175W	-546.455	-546.252	-526.67
2:550000	YBR157C	YCL027W	88.2736	98.1571	107.039
2:550000	YBR157C	YEL054C	320.719	332.984	346.166
2:550000	YBR157C	YMR242C	-387.718	-378.065	-376.237
2:550000	YBR157C	YGL062W	-988.849	-979.515	-974.307
2:550000	YBR157C	YMR116C	-1110.81	-1104.87	-1088.11
2:550000	YBR157C	YHR209W	262.148	272.161	297.926
2:550000	YBR157C	YMR170C	349.619	353.463	374.891

2:550000	YBR157C	YGL185C	-583.174	-582.122	-574.557
2:550000	YBR157C	YNR050C	-537.293	-528.322	-470.542
2:550000	YBR157C	YLR150W	-1238.14	-1231.7	-1227.93
2:550000	YBR157C	YJR045C	-678.987	-670.437	-661.903
2:550000	YBR157C	YNL101W	-405.08	-400.263	-369.208
2:550000	YBR157C	YLR368W	-463.232	-450.93	-426.69
2:550000	YBR157C	YDR418W	-666.58	-656.095	-652.526
2:550000	YBR157C	YHL042W	357.908	362.808	374.528
2:550000	YBR157C	YJR138W	-375.063	-368.235	-324.643
2:550000	YBR157C	YDR352W	-334.363	-324.831	-273.848
2:550000	YBR157C	YML030W	-127.597	-126.306	-123.787
2:550000	YBR157C	YLR259C	-852.802	-845.317	-841.864
2:550000	YBR157C	YLR388W	-78.9004	-69.6489	-67.0657
2:550000	YBR157C	YML063W	-678.527	-667.544	-665.026
2:550000	YBR157C	YJR054W	-513.552	-508.777	-489.082
2:550000	YBR157C	YEL058W	-709.091	-697.727	-660.073
2:550000	YBR157C	YML118W	72.8839	81.0257	95.2718
2:550000	YBR157C	YAL023C	-963.672	-951.861	-949.807
2:550000	YBR157C	YLR388W	-184.501	-175.064	-171.556
2:550000	YBR157C	YKR094C	-490.495	-479.248	-478.647
2:550000	YBR157C	YAL062W	542.487	548.411	584.957
2:550000	YBR157C	YEL065W	189.181	192.685	204.367
2:550000	YBR157C	YLR344W	-153.323	-141.124	-128.418
2:550000	YBR157C	YDR222W	290.867	294.647	306.099
2:550000	YBR157C	YHR089C	717.345	724.672	787.127
2:550000	YBR157C	YNL015W	-151.548	-145.782	-151.047
2:550000	YBR157C	YLR048W	-19.7805	-9.45159	-8.11837
2:550000	YBR157C	YBR173C	-219.448	-214.001	-218.837
2:550000	YBR157C	YKL204W	-536.036	-524.312	-513.373
2:550000	YBR157C	YBL072C	-814.995	-804.972	-795.525
2:550000	YBR157C	YOL128C	760.808	764.823	816.522
2:550000	YBR157C	YHR199C-A	-207.09	-204.499	-203.169
2:550000	YBR157C	YJL206C	-80.385	-68.958	-18.587
2:550000	YBR157C	YNL178W	-730.793	-728.244	-729.47
2:550000	YBR157C	YMR205C	-1090.07	-1084.08	-1083.84
2:550000	YBR157C	YMR236W	-947.574	-946.815	-873.585
2:550000	YBR157C	YOL019W	-240.635	-237.775	-226.242
2:550000	YBR157C	YDL117W	-436.094	-433.127	-371.554
2:550000	YBR157C	YIL018W	-231.422	-220.874	-223.809
2:550000	YBR157C	YOR126C	-590.727	-583.552	-549.052
2:550000	YBR157C	YMR122W-A	-1305.06	-1294.76	-1275.49
2:550000	YBR157C	YBR167C	-411.252	-399.099	-294.39
2:550000	YBR157C	YGR034W	-249.447	-237.592	-234.829
2:550000	YBR157C	YJL076W	-692.563	-686.329	-681.341
2:550000	YBR157C	YER165W	-891.592	-881.3	-868.017
2:550000	YBR157C	YIL164C	453.826	461.337	508.005
2:550000	YBR157C	YER063W	-612.125	-601.27	-559.018

2:550000	YBR157C	YDR471W	168.487	180.324	199.782
2:550000	YBR157C	YDR508C	297.914	309.513	360.975
2:550000	YBR157C	YNL283C	-566.292	-564.081	-541.952
2:550000	YBR157C	YDL130W	-674.934	-664.634	-662.196
2:550000	YBR157C	YMR118C	636.345	640.542	649.758
2:550000	YBR157C	YDL061C	-854.201	-845.85	-843.639
2:550000	YBR157C	YHL033C	-360.631	-353.931	-356.449
2:550000	YBR157C	YDL024C	52.6627	64.123	81.0755
2:550000	YBR157C	YLR054C	-30.3189	-19.2082	2.19766
2:550000	YBR157C	YOL131W	197.008	207.623	229.299
2:550000	YBR157C	YDL083C	59.6775	69.8568	68.5216
2:550000	YBR157C	YGR019W	90.6081	96.0176	118.016
2:550000	YBR157C	YNL268W	-889.665	-878.915	-883.683
2:550000	YBR157C	YGL129C	-95.5795	-89.8729	-95.3982
2:550000	YBR157C	YOL025W	-714.392	-708.363	-701.75
2:550000	YBR157C	YMR271C	646.897	654.377	740.167
2:550000	YBR157C	YLR448W	-144.247	-132.616	-121.78
2:550000	YBR157C	YOL045W	99.0686	103.9	103.837
2:550000	YBR157C	YNL209W	-147.346	-136.79	-131.868
2:550000	YBR157C	YPL143W	-836.595	-827.993	-828.677
2:550000	YBR157C	YDR131C	-532.483	-532.004	-522.049
2:550000	YBR157C	YOL039W	-762.503	-750.481	-749.792
2:550000	YBR157C	YPL249C-A	-878.476	-868.511	-867.991
2:550000	YBR157C	YHR117W	-770.126	-759.472	-741.06
2:550000	YBR157C	YHR202W	62.13	69.9532	130.935
2:550000	YBR157C	YMR194W	-182.524	-171.666	-152.987
2:550000	YBR157C	YHR012W	-693.288	-689.159	-689.432
2:550000	YBR157C	YDR025W	-29.6181	-18.9883	-16.726
2:550000	YBR157C	YCR020C	316.645	324.29	379.953
2:550000	YBR157C	YBR263W	-850.584	-846.159	-848.232
2:550000	YBR157C	YJL159W	-1129.8	-1122.06	-1097.94
2:550000	YBR157C	YJL172W	0.895425	5.10281	104.979
2:550000	YBR157C	YJL045W	89.7391	99.1112	101.188
2:550000	YBR157C	YJL023C	-418.603	-410.739	-402.943
2:550000	YBR157C	YPR144C	441.714	443.225	483.644
2:550000	YBR157C	YEL023C	-412.808	-401.535	-395.235
2:550000	YBR157C	YBR115C	-28.899	-19.5693	-19.7675
2:550000	YBR157C	YOR127W	-261.315	-250.311	-218.457
2:550000	YBR157C	YPL176C	-485.836	-474.155	-469.658
2:550000	YBR157C	YCR031C	-987.673	-980.638	-983.148
2:550000	YBR157C	YER131W	321.945	333.103	339.108
2:550000	YBR157C	YGR040W	-398.144	-388.66	-353.425
2:550000	YBR157C	YJL191W	96.1478	107.251	140.7
2:550000	YBR157C	YIL089W	137.533	142.449	152.791
2:550000	YBR157C	YFL036W	-222.427	-212.049	-169.593
2:550000	YBR157C	YHR183W	-793.016	-784.476	-770.128
2:550000	YBR157C	YPL171C	96.7275	102.093	103.358

2:550000	YBR157C	YDL169C	383.973	388.637	421.237
2:550000	YBR157C	YLR325C	-214.633	-204.25	-204.746
2:550000	YBR157C	YDR500C	-229.557	-220.619	-216.529
2:550000	YBR157C	YPL079W	-410.4	-401.294	-399.884
2:550000	YBR157C	YHR007C	-808.515	-796.654	-802.765
2:550000	YBR157C	YDR260C	-428.012	-422.429	-365.698
2:550000	YBR157C	YLR061W	-216.787	-207.791	-204.415
2:550000	YBR157C	YOR296W	-511.687	-505.78	-505.028
2:550000	YBR157C	YMR001C	-444.909	-438.18	-377.498
2:550000	YBR157C	YDR494W	20.8894	28.7901	26.7342
2:550000	YBR157C	YIL146C	204.989	215.469	324.279
2:550000	YBR157C	YNL117W	440.315	447.087	497.741
2:550000	YBR157C	YMR101C	50.9995	62.9683	88.849
2:550000	YBR157C	YHR142W	139.391	151.677	163.624
2:550000	YBR157C	YER043C	-984.865	-977.71	-952.049
2:550000	YBR157C	YIL088C	-359.266	-351.033	-267.676
2:550000	YBR157C	YNL239W	-358.804	-348.749	-291.23
2:550000	YBR157C	YLR333C	-506.009	-494.915	-492.61
2:550000	YBR157C	YMR030W-A	-308.956	-306.876	-268.064
2:550000	YBR157C	YGL202W	-592.583	-584.059	-546.622
2:550000	YBR157C	YBR189W	-355.556	-353.528	-353.257
2:550000	YBR157C	YCR033W	-786.27	-778.813	-772.325
2:550000	YBR157C	YPL163C	-503.231	-495.289	-494.798
2:550000	YBR157C	YNL046W	-300.377	-292.834	-250.5
2:550000	YBR157C	YKL218C	722.064	729.689	798.801
2:550000	YBR157C	YPR132W	-1024.3	-1019.93	-1019.9
2:550000	YBR157C	YDR077W	-1445.76	-1442.38	-1444.76
2:550000	YBR157C	YER151C	-863.878	-856.1	-854.832
2:550000	YBR157C	YNL096C	-112.083	-100.274	-71.6528
2:550000	YBR157C	YCL001W-B	-871.288	-869.567	-863.01
2:550000	YBR157C	YLR289W	196.008	200.839	198.5
2:550000	YBR157C	YKL181W	64.8406	75.9868	136.982
2:550000	YBR157C	YKL006W	-527.021	-515.109	-514.989
2:550000	YBR157C	YER074W	-456.123	-447.32	-449.267
2:550000	YBR157C	YFR031C-A	-191.72	-180.722	-181.594
2:550000	YBR157C	YNL323W	-934.282	-924.517	-918.856
2:550000	YBR157C	YHL033C	-456.08	-453.785	-451.554
2:550000	YBR157C	YDL131W	259.632	265.769	335.304
2:550000	YBR157C	YCL014W	-480.066	-471.987	-444.487
2:550000	YBR157C	YDL082W	262.973	274.942	287.836
2:550000	YBR157C	YMR011W	587.118	592.63	644.461
2:550000	YBR157C	YML106W	-801.438	-793.02	-773.04
2:550000	YBR157C	YJR005W	-583.695	-574.393	-548.472
2:550000	YBR157C	YGR154C	383.007	394.841	446.559
2:550000	YBR157C	YLR432W	17.3039	29.3639	63.7306
2:550000	YBR157C	YER056C-A	-240.759	-229.301	-225.595
2:550000	YBR157C	YKL107W	363.889	375.666	429.446

2:550000	YBR157C	YAL037W	384.185	396.303	423.791
2:550000	YBR157C	YPR119W	-315.102	-306.099	-298.425
2:550000	YBR157C	YPR007C	-125.529	-117.396	-117.816
2:550000	YBR157C	YDL014W	695.282	702.285	752.472
2:550000	YBR157C	YNL302C	-893.495	-882.305	-882.387
2:550000	YBR157C	YIR009W	-226.449	-216.534	-146.816
2:550000	YBR157C	YER001W	-123.528	-115.439	-76.8883
2:550000	YBR157C	YFR040W	-360.455	-352.468	-316.149
2:550000	YBR157C	YML073C	-326.194	-316.931	-312.084
2:550000	YBR157C	YIL131C	-296.886	-291.497	-290.367
2:550000	YBR157C	YPR138C	566.81	569.814	632.019
2:550000	YBR157C	YKR097W	291.315	293.699	305.324
2:550000	YBR157C	YIL071C	19.9483	20.5149	31.4618
2:550000	YBR157C	YBL087C	-732.74	-721.382	-713.913
2:550000	YBR157C	YOL121C	71.0272	82.923	87.4709
2:550000	YBR157C	YGL123W	-937.992	-928.238	-931.217
2:550000	YBR157C	YJR148W	-893.426	-887.906	-872.205
2:550000	YBR157C	YPR041W	-151.002	-149.286	-120.263
2:550000	YBR157C	YER118C	-454.081	-452.149	-447.758
2:550000	YBR157C	YDL156W	-301.651	-291.582	-295.932
2:550000	YBR157C	YDR385W	-1613.9	-1610.26	-1599.81
2:550000	YBR157C	YGL139W	-686.823	-675.05	-661.634
2:550000	YBR157C	YMR174C	447.46	458.27	489.789
2:550000	YBR157C	YNL069C	-687.611	-675.922	-674.877
2:550000	YBR157C	YOR111W	-195.085	-184.967	-140.938
2:550000	YBR157C	YBR256C	-378.721	-367.91	-331.399
2:550000	YBR157C	YNL278W	-34.2213	-22.6134	-25.5296
2:550000	YBR157C	YGR067C	61.9602	71.568	76.3658
2:550000	YBR157C	YHR010W	-534.287	-521.965	-519.145
2:550000	YBR157C	YLR285C-A	-52.5572	-42.3809	-48.4532
2:550000	YBR157C	YHR107C	-592.664	-582.425	-575.378
2:550000	YBR157C	YLR042C	127.423	138.329	138.557
2:550000	YBR157C	YKL087C	60.7048	69.7639	76.1374
2:550000	YBR157C	YMR173W	-552.295	-546.251	-542.447
2:550000	YBR157C	YER074W	-647.827	-635.712	-639.671
2:550000	YBR157C	YER145C	-82.2866	-81.1151	-78.0858
2:550000	YBR157C	YKL179C	-814.441	-804.327	-770.827
2:550000	YBR157C	YGR138C	259.719	265.556	306.138
2:550000	YBR157C	YOR096W	-487.617	-476.494	-476.329
2:550000	YBR157C	YLR406C	311.914	323.915	339.245
2:550000	YBR157C	YOR293W	-41.0303	-30.0761	-27.5986
2:550000	YBR157C	YGL076C	-1199.16	-1190.87	-1188.88
2:550000	YBR157C	YBR117C	604.637	614.945	631.146
2:550000	YBR157C	YDR034W-B	354.462	356.708	357.328
2:550000	YBR157C	YLR378C	-896.754	-884.581	-872.369
2:550000	YBR157C	YBR038W	-329.154	-325.197	-251.389
2:550000	YBR157C	YOR298W	380.661	389.868	429.695

2:550000	YBR157C	YEL004W	-576.52	-567.145	-549.038
2:550000	YBR157C	YOL007C	-194.288	-191.605	-167.243
2:550000	YBR157C	YPL061W	-28.0942	-17.7433	23.6929
2:550000	YBR157C	YMR163C	-176.777	-165.243	-127.848
2:550000	YBR157C	YCL055W	-22.0792	-10.6069	6.83511
2:550000	YBR157C	YBR191W	-707.851	-696.766	-694.57
2:550000	YBR157C	YPL089C	168.04	176.467	244.023
2:550000	YBR157C	YDL179W	-212.616	-206.597	-171.438
2:550000	YBR157C	YGR085C	-1051.83	-1039.69	-1039.9
2:550000	YBR157C	YPL158C	-575.429	-563.244	-534.017
2:550000	YBR157C	YIL098C	390.501	397.522	391.855
2:550000	YBR157C	YDL210W	1254	1255.67	1300.98
2:550000	YBR157C	YLR080W	271.678	278.183	316.299
2:550000	YBR157C	YBR111W-A	92.9825	104.323	103.661
2:550000	YBR157C	YLL045C	-690.771	-678.72	-681.799
2:550000	YBR157C	YER025W	-357.651	-350.264	-308.302
2:550000	YBR157C	YOR315W	449.871	456.924	545.895
2:550000	YBR157C	YOR330C	-175.555	-164.122	-150.018
2:550000	YBR157C	YLR340W	-971.184	-959.64	-953.094
2:550000	YBR157C	YNL301C	-1060.92	-1052.65	-1054.03
2:550000	YBR157C	YGR014W	-556.224	-546.087	-519.494
2:550000	YBR157C	YDL078C	-908.934	-902.728	-887.38
2:550000	YBR157C	YOL040C	-740.869	-729.765	-731.361
2:550000	YBR157C	YGR020C	-1083.8	-1082.97	-1082.6
2:550000	YBR157C	YJL160C	1.03679	12.3517	35.469
2:550000	YBR157C	YBR181C	-1057.56	-1045.57	-1039.34
2:550000	YBR157C	YJR096W	352.844	355.53	352.985
2:550000	YBR157C	YMR013C	-379.046	-372.937	-374.178
2:550000	YBR157C	YNL176C	-478.887	-469.326	-446.125
2:550000	YBR157C	YFR028C	-634.745	-630.977	-629.695
2:550000	YBR157C	YLR079W	-765.61	-760.525	-751.294
2:550000	YBR157C	YBR098W	-234.653	-225.737	-214.667
2:550000	YBR157C	YAL041W	-771.199	-765.159	-771.116
2:550000	YBR157C	YGR152C	-429.616	-418.185	-413.994
2:550000	YBR157C	YML052W	-466.077	-457.331	-371.44
2:550000	YBR157C	YBR193C	-441.217	-437.088	-358.061
2:550000	YBR157C	YHR203C	-1386.89	-1376.32	-1370.72
2:550000	YBR157C	YBL061C	245.92	250.665	330.468
2:550000	YBR157C	YKL068W-A	400.967	402.091	418.796
2:550000	YBR157C	YBR048W	-795.695	-783.817	-778.754
2:550000	YBR157C	YGL135W	-1300.64	-1288.98	-1285.95
2:550000	YBR157C	YER060W	116.475	128.772	155.424
2:550000	YBR157C	YER096W	130.096	132.701	141.072
2:550000	YBR157C	YFL022C	-737.534	-730.686	-720.579
2:550000	YBR157C	YDR333C	-529.017	-526.241	-522.089
2:550000	YBR157C	YNL165W	-278.326	-270.469	-250.615
2:550000	YBR157C	YOR134W	553.91	565.06	595.794

2:550000	YBR157C	YAL024C	-409.8	-406.133	-395.39
2:550000	YBR157C	YIL165C	486.691	491.84	537.1
2:550000	YBR157C	YBR126C	308.388	319.395	313.305
2:550000	YBR157C	YHL023C	-635.992	-626.158	-589.511
2:550000	YBR157C	YJL177W	24.8582	36.2641	41.23
2:550000	YBR157C	YJR094W-A	-36.0036	-24.854	-20.7721
2:550000	YBR157C	YDL075W	-648.435	-636.252	-636.063
2:550000	YBR157C	YBR092C	538.713	548.331	580.529
2:550000	YBR157C	YJL110C	-19.3244	-11.8578	44.0235
2:550000	YBR157C	YHL001W	-914.382	-903.066	-900.48
2:550000	YBR157C	YHR021C	-495.808	-484.793	-485.961
2:550000	YBR157C	YPL071C	-280.961	-271.902	-240.862
2:550000	YBR157C	YKR061W	547.544	550.09	602.747
2:550000	YBR157C	YBR191W	-244.663	-232.947	-227.997
2:550000	YBR157C	YMR285C	-884.128	-878.303	-881.292
2:550000	YBR157C	YKR013W	-181.951	-172.457	-165.014
2:550000	YBR157C	YBL092W	-932.056	-919.776	-914.529
2:550000	YBR157C	YBR119W	-219.742	-216.538	-202.329
2:550000	YBR157C	YDR381W	-214.661	-205.356	-172.618
2:550000	YBR157C	YHR099W	-312.384	-305.145	-283.649
2:550000	YBR157C	YJL136C	-861.037	-849.099	-846.426
2:550000	YBR157C	YIL133C	-50.891	-40.8991	-37.3229
2:550000	YBR157C	YFR031C-A	-941.659	-932.695	-932.436
2:550000	YBR157C	YOR293W	-80.7798	-69.8837	-68.5068
2:550000	YBR157C	YOL082W	41.1262	51.517	91.5858
2:550000	YBR157C	YGR044C	-145.461	-138.214	-108.854
2:550000	YBR162C	YBR149W	-619.755	-611.899	-617.097
2:550000	YBR162C	YLR286C	-403.673	-399.59	-361.821
2:550000	YBR162C	YMR182C	101.474	101.995	101.478
2:550000	YBR162C	YPL264C	683.022	689.174	702.977
2:550000	YBR162C	YDR072C	146.134	150.586	157.482
2:550000	YBR162C	YOR264W	-612.267	-601.114	-606.718
2:550000	YBR162C	YLR029C	-955.293	-951.498	-914.43
2:550000	YBR162C	YIL052C	-436.709	-435.233	-405.682
2:550000	YBR162C	YNL055C	-1138.99	-1129.92	-1133.58
2:550000	YBR162C	YPL131W	-728.761	-728.153	-708.012
2:550000	YBR162C	YJL200C	685.448	692.321	737.579
2:550000	YBR162C	YNR067C	-571.572	-559.842	-562.724
2:550000	YBR162C	YDL055C	-1091.25	-1086.68	-1090.5
2:550000	YBR162C	YIL009W	-462.101	-455.462	-460.692
2:550000	YBR162C	YJR123W	-938.907	-933.319	-926.127
2:550000	YBR162C	YGR027C	-976.135	-972.922	-952.066
2:550000	YBR162C	YDL155W	-375.474	-371.4	-352.829
2:550000	YBR162C	YIR034C	437.636	439.8	450.285
2:550000	YBR162C	YLR185W	-352.915	-349.737	-316.344
2:550000	YBR162C	YCR073C	707.9	711.005	761.249
2:550000	YBR162C	YIL094C	-373.467	-372.181	-302.923

2:550000	YBR162C	YBL072C	-1234.13	-1230.19	-1212.68
2:550000	YBR162C	YGR148C	-966.228	-964.679	-943.777
2:550000	YBR162C	YAR014C	-373.1	-367.89	-309.389
2:550000	YBR162C	YBR197C	103.447	106.563	124.968
2:550000	YBR162C	YDR234W	523.562	533.962	529.483
2:550000	YBR162C	YIR030C	851.422	854.12	867.469
2:550000	YBR162C	YPL066W	-759.766	-750.484	-756.37
2:550000	YBR162C	YPR005C	159.443	165.499	185.133
2:550000	YBR162C	YER036C	62.8361	62.9884	64.5521
2:550000	YBR162C	YGL189C	-893.512	-892.091	-866.463
2:550000	YBR162C	YPR043W	-1094.45	-1090.07	-1072.28
2:550000	YBR162C	YER089C	-757.039	-746.08	-720.199
2:550000	YBR162C	YDL182W	139.714	143.617	208.828
2:550000	YBR162C	YGL062W	-911.183	-903.919	-906.192
2:550000	YBR162C	YML030W	-123.148	-120.329	-114.939
2:550000	YBR162C	YML063W	-656.527	-650.575	-622.982
2:550000	YBR162C	YAL023C	-888.722	-887.463	-880.665
2:550000	YBR162C	YBL072C	-744.849	-736.486	-725.97
2:550000	YBR162C	YMR236W	-854.358	-848.612	-831.802
2:550000	YBR162C	YOL019W	-317.835	-317.744	-209.502
2:550000	YBR162C	YDL117W	-389.561	-385.522	-352.386
2:550000	YBR162C	YGR281W	-299.074	-297.922	-296.613
2:550000	YBR162C	YIL018W	-219.107	-218.554	-190.723
2:550000	YBR162C	YOR126C	-523.708	-512.668	-510.748
2:550000	YBR162C	YNL283C	-507.152	-498.67	-504.707
2:550000	YBR162C	YLR353W	-616.701	-607.232	-579.613
2:550000	YBR162C	YNL268W	-836.603	-825.756	-824.881
2:550000	YBR162C	YNL327W	-609.304	-604.84	-608.42
2:550000	YBR162C	YOL039W	-704.991	-698.495	-688.325
2:550000	YBR162C	YPL249C-A	-812.132	-810.708	-803.008
2:550000	YBR162C	YHR117W	-713.201	-701.667	-698.675
2:550000	YBR162C	YBR104W	463.567	470.923	516.119
2:550000	YBR162C	YJL159W	-1057.09	-1047.37	-1024.51
2:550000	YBR162C	YJL116C	364.691	370.732	423.8
2:550000	YBR162C	YBR115C	-36.7824	-25.4658	-27.601
2:550000	YBR162C	YPL176C	-432.815	-430.44	-429.28
2:550000	YBR162C	YGR040W	-340.115	-328.953	-334.681
2:550000	YBR162C	YJL191W	112.098	113.792	133.513
2:550000	YBR162C	YHR007C	-829.849	-823.802	-767.658
2:550000	YBR162C	YBR107C	-480.237	-474.125	-421.867
2:550000	YBR162C	YMR101C	63.5053	63.602	88.1051
2:550000	YBR162C	YLR084C	-277.238	-272.283	-274.108
2:550000	YBR162C	YGL202W	-576.069	-575.256	-513.912
2:550000	YBR162C	YPL163C	-459.869	-451.61	-456.601
2:550000	YBR162C	YKL006W	-495.823	-492.849	-476.652
2:550000	YBR162C	YMR181C	66.0564	69.1411	66.4559
2:550000	YBR162C	YGL209W	386.931	389.063	400.543

2:550000	YBR162C	YDL131W	293.649	295.654	326.535
2:550000	YBR162C	YCL014W	-433.54	-426.362	-417.597
2:550000	YBR162C	YJR005W	-515.041	-514.155	-514.283
2:550000	YBR162C	YFL021W	476.295	483.008	551.074
2:550000	YBR162C	YER056C-A	-236.182	-234.995	-204.633
2:550000	YBR162C	YNL302C	-825.531	-823.823	-811.246
2:550000	YBR162C	YJR133W	-201.328	-192.819	-190.03
2:550000	YBR162C	YGL123W	-859.969	-859.09	-851.687
2:550000	YBR162C	YNL069C	-639.546	-637.437	-615.845
2:550000	YBR162C	YIL031W	-442.862	-438.948	-413.522
2:550000	YBR162C	YHR010W	-504.666	-501.373	-471.684
2:550000	YBR162C	YLR285C-A	-51.7708	-42.6014	-48.1021
2:550000	YBR162C	YHR107C	-567.911	-561.009	-535.619
2:550000	YBR162C	YLR042C	114.756	123.302	124.884
2:550000	YBR162C	YMR173W	-514.343	-505.398	-510.917
2:550000	YBR162C	YER074W	-619.643	-611.908	-595.128
2:550000	YBR162C	YCR086W	-162.671	-151.457	-84.0055
2:550000	YBR162C	YNL040W	-35.6214	-26.985	-16.7057
2:550000	YBR162C	YKL179C	-748.981	-745.172	-717.099
2:550000	YBR162C	YGR138C	275.951	282.45	291.753
2:550000	YBR162C	YPR105C	-734.575	-733.701	-726.31
2:550000	YBR162C	YOR096W	-460.529	-458.145	-430.135
2:550000	YBR162C	YOR293W	-53.3321	-52.9148	-17.4203
2:550000	YBR162C	YNL078W	-431.785	-422.873	-426.576
2:550000	YBR162C	YEL004W	-534.298	-526.496	-512.443
2:550000	YBR162C	YOL007C	-164.47	-157.256	-151.638
2:550000	YBR162C	YPR074C	-1126.95	-1118.23	-1112.37
2:550000	YBR162C	YDL179W	-167.529	-157.715	-161.554
2:550000	YBR162C	YGR085C	-995.49	-991.838	-970.462
2:550000	YBR162C	YPL158C	-512.316	-505.216	-510.745
2:550000	YBR162C	YIL098C	345.96	349.113	369.287
2:550000	YBR162C	YBR111W-A	73.5311	83.0952	96.033
2:550000	YBR162C	YLL045C	-636.657	-629.303	-621.552
2:550000	YBR162C	YOR316C	-607.202	-596.251	-586.186
2:550000	YBR162C	YLR340W	-904.373	-899.707	-876.579
2:550000	YBR162C	YOL040C	-688.884	-684.935	-668.98
2:550000	YBR162C	YBR181C	-981.851	-981.469	-961.373
2:550000	YBR162C	YLR079W	-721.77	-710.714	-709.167
2:550000	YBR162C	YOR247W	-946.033	-941.483	-873.493
2:550000	YBR162C	YAL041W	-714.075	-707.385	-709.864
2:550000	YBR162C	YGR152C	-402.551	-396.848	-384.249
2:550000	YBR162C	YHR203C	-1302.12	-1301.15	-1268.29
2:550000	YBR162C	YBR048W	-739.256	-736.548	-714.84
2:550000	YBR162C	YGL135W	-1216.46	-1212.9	-1190.08
2:550000	YBR162C	YNL165W	-245.651	-236.109	-233.567
2:550000	YBR162C	YOR134W	547.689	549.75	562.123
2:550000	YBR162C	YAL024C	-402.553	-393.889	-364.687

2:550000	YBR162C	YBR126C	286.716	298.664	294.644
2:550000	YBR162C	YAL039C	-79.3659	-76.5406	-75.2644
2:550000	YBR162C	YDL075W	-601.725	-596.147	-580.91
2:550000	YBR162C	YHL001W	-843.529	-843.173	-829.771
2:550000	YBR162C	YNL168C	-148.51	-142.015	-140.666
2:550000	YBR162C	YKR061W	503.657	511.532	565.024
2:550000	YBR162C	YBL092W	-865.472	-859.966	-845.491
2:550000	YBR162C	YBR119W	-184.919	-173.85	-177.31
2:550000	YBR162C	YJL136C	-804.645	-801.949	-781.567
2:550000	YBR162C	YOL082W	74.106	74.9231	85.9282
2:550000	YBR154C	YKL012W	-957.418	-950.203	-825.002
2:550000	YBR154C	YKL138C	-9.58868	1.66952	252.515
2:550000	YBR154C	YKL195W	-315.512	-308.411	6.44149
2:550000	YBR154C	YBR149W	-682.501	-678.282	-669.411
2:550000	YBR154C	YLR286C	-441.055	-441.024	-403.106
2:550000	YBR154C	YOR187W	-447.816	-447.456	-396.358
2:550000	YBR154C	YDR072C	80.7397	83.5143	168.763
2:550000	YBR154C	YOR264W	-658.032	-646.047	-651.252
2:550000	YBR154C	YDL061C	-927.607	-922.843	-783.671
2:550000	YBR154C	YBR084C-A	-439.681	-431.381	-323.031
2:550000	YBR154C	YDL012C	-535.458	-526.377	-427.921
2:550000	YBR154C	YKL194C	-176.104	-168.215	99.7679
2:550000	YBR154C	YLR029C	-1053.3	-1042.54	-981.419
2:550000	YBR154C	YOR338W	439.11	440.841	797.189
2:550000	YBR154C	YIL052C	-616.087	-604.564	-447.651
2:550000	YBR154C	YOR179C	-474.033	-471.724	-433.091
2:550000	YBR154C	YNL055C	-1209.27	-1198.88	-1204.67
2:550000	YBR154C	YPL131W	-880.807	-872.214	-760.957
2:550000	YBR154C	YJL200C	523.851	534.573	782.556
2:550000	YBR154C	YNR067C	-604.224	-592.172	-593.391
2:550000	YBR154C	YDL055C	-1164.99	-1161.51	-1148.11
2:550000	YBR154C	YMR306W	-151.582	-149.82	15.9914
2:550000	YBR154C	YIL009W	-502.69	-492.656	-497.548
2:550000	YBR154C	YHL001W	-294.395	-289.337	-169.59
2:550000	YBR154C	YJR123W	-1120.68	-1109.54	-1002.61
2:550000	YBR154C	YGR027C	-1110.83	-1100.73	-1031.58
2:550000	YBR154C	YDL229W	-147.873	-137.811	24.6507
2:550000	YBR154C	YDL155W	-661.857	-655.328	-383.682
2:550000	YBR154C	YKL180W	-622.256	-613.745	-445.774
2:550000	YBR154C	YIR034C	336.839	341.326	482.985
2:550000	YBR154C	YLR185W	-487.34	-478.536	-348.923
2:550000	YBR154C	YHR038W	-17.7167	-16.1635	418.777
2:550000	YBR154C	YKL081W	-866.976	-856.095	-650.901
2:550000	YBR154C	YCR073C	420.696	423.441	813.25
2:550000	YBR154C	YIL094C	-641.362	-632.809	-325.886
2:550000	YBR154C	YBL072C	-1447.39	-1435.29	-1309.62
2:550000	YBR154C	YBL027W	-550.907	-544.372	-406.737

2:550000	YBR154C	YER102W	-749.12	-741.559	-617.109
2:550000	YBR154C	YGL189C	-1185.24	-1178.85	-1093.69
2:550000	YBR154C	YKR057W	-420.68	-411.53	-233.439
2:550000	YBR154C	YGR085C	-278.142	-269.441	-52.5588
2:550000	YBR154C	YGR148C	-1130.74	-1120.82	-1013.05
2:550000	YBR154C	YBR291C	71.4771	74.9653	392.094
2:550000	YBR154C	YGR214W	-382.121	-378.415	-226.17
2:550000	YBR154C	YLR026C	-396.559	-387.202	-317.175
2:550000	YBR154C	YBR197C	-4.93441	2.34325	148.537
2:550000	YBR154C	YIL009C-A	-386.004	-378.675	-264.843
2:550000	YBR154C	YMR142C	-855.84	-851.557	-743.363
2:550000	YBR154C	YDL229W	-595.726	-589.488	-437.32
2:550000	YBR154C	YJL063C	-20.4134	-13.1231	107.657
2:550000	YBR154C	YPR163C	-102.322	-92.006	186.768
2:550000	YBR154C	YDR234W	531.547	540.066	568.853
2:550000	YBR154C	YNL073W	-443.797	-434.366	-274.729
2:550000	YBR154C	YIR030C	836.151	839.785	930.47
2:550000	YBR154C	YGL068W	118.975	126.662	239.505
2:550000	YBR154C	YOL120C	-643.602	-642.031	-515.382
2:550000	YBR154C	YBR162C	-772.844	-768.284	-737.254
2:550000	YBR154C	YPR181C	-1067.46	-1060.2	-951.961
2:550000	YBR154C	YLR249W	-524.732	-513.369	-344.786
2:550000	YBR154C	YPL066W	-809.36	-800.878	-806.418
2:550000	YBR154C	YER036C	-143.034	-131.747	65.2264
2:550000	YBR154C	YOR246C	-991.774	-987.385	-967.012
2:550000	YBR154C	YBR079C	82.7197	85.2162	249.575
2:550000	YBR154C	YAL035W	-245.925	-243.704	-155.798
2:550000	YBR154C	YGL189C	-1043.93	-1032.96	-947.822
2:550000	YBR154C	YPR043W	-1237.21	-1226.19	-1150.34
2:550000	YBR154C	YMR225C	-8.94712	-5.15666	32.833
2:550000	YBR154C	YER089C	-885.636	-874.472	-780.376
2:550000	YBR154C	YPL132W	-289.207	-280.074	21.5996
2:550000	YBR154C	YNL121C	-849.112	-847.457	-800.586
2:550000	YBR154C	YOL127W	-1234.03	-1223.28	-1110.48
2:550000	YBR154C	YOR061W	-979.211	-973.958	-841.669
2:550000	YBR154C	YPR162C	-771.534	-764.544	-442.259
2:550000	YBR154C	YDR237W	71.6978	81.428	222.031
2:550000	YBR154C	YDL182W	24.8724	30.6804	214.159
2:550000	YBR154C	YMR180C	-438.583	-429.269	7.98762
2:550000	YBR154C	YGR084C	20.6422	23.0454	90.8587
2:550000	YBR154C	YDL095W	-928.839	-923.077	-810.663
2:550000	YBR154C	YNL270C	-30.6398	-20.9061	85.7966
2:550000	YBR154C	YNL240C	-56.2556	-54.4926	700.645
2:550000	YBR154C	YCL027W	-73.5549	-63.2951	107.039
2:550000	YBR154C	YNR037C	26.7952	30.9276	71.0211
2:550000	YBR154C	YEL054C	11.6131	21.6987	346.166
2:550000	YBR154C	YMR242C	-551.371	-544.602	-376.237

2:550000	YBR154C	YGL062W	-976.802	-968.502	-974.307
2:550000	YBR154C	YHR209W	273.923	277.995	297.926
2:550000	YBR154C	YNR050C	-645.41	-634.421	-470.542
2:550000	YBR154C	YIL070C	413.984	421.817	530.22
2:550000	YBR154C	YLR150W	-1393.08	-1384.23	-1227.93
2:550000	YBR154C	YJR045C	-899.485	-892.819	-661.903
2:550000	YBR154C	YLR368W	-555.753	-546.937	-426.69
2:550000	YBR154C	YDR418W	-816.619	-810.774	-652.526
2:550000	YBR154C	YHL042W	214.522	220.533	374.528
2:550000	YBR154C	YHR030C	-344.734	-341.248	-255.429
2:550000	YBR154C	YML030W	-339.593	-335.134	-123.787
2:550000	YBR154C	YLR259C	-1013.52	-1008.57	-841.864
2:550000	YBR154C	YLR388W	-280.18	-278.374	-67.0657
2:550000	YBR154C	YML063W	-737.205	-725.03	-665.026
2:550000	YBR154C	YLL039C	-662.483	-655.233	-628.626
2:550000	YBR154C	YEL058W	-825.263	-817.384	-660.073
2:550000	YBR154C	YAL023C	-1037.51	-1031.91	-949.807
2:550000	YBR154C	YBR228W	-234.118	-232.371	-183.76
2:550000	YBR154C	YLR388W	-360.634	-358.107	-171.556
2:550000	YBR154C	YKR094C	-623.281	-614.07	-478.647
2:550000	YBR154C	YPL128C	-644.588	-644.013	-639.975
2:550000	YBR154C	YKL164C	-977.883	-973.807	-955.669
2:550000	YBR154C	YLR344W	-338.986	-331.053	-128.418
2:550000	YBR154C	YHR089C	471.652	481.761	787.127
2:550000	YBR154C	YNL015W	-190.865	-188.665	-151.047
2:550000	YBR154C	YIL140W	-492.335	-491.694	-480.054
2:550000	YBR154C	YLR048W	-222.137	-213.934	-8.11837
2:550000	YBR154C	YBR173C	-411.408	-400.546	-218.837
2:550000	YBR154C	YKL204W	-746.959	-739.124	-513.373
2:550000	YBR154C	YBL072C	-889.755	-879.51	-795.525
2:550000	YBR154C	YJL206C	-147.013	-144.777	-18.587
2:550000	YBR154C	YNL178W	-802.994	-801.988	-729.47
2:550000	YBR154C	YDR430C	-162.828	-155.496	90.9889
2:550000	YBR154C	YCR099C	245.318	248.813	245.59
2:550000	YBR154C	YMR236W	-1037.85	-1037.53	-873.585
2:550000	YBR154C	YER154W	-404.831	-397.269	-318.211
2:550000	YBR154C	YDL117W	-594.865	-584.376	-371.554
2:550000	YBR154C	YIL018W	-369.506	-360.575	-223.809
2:550000	YBR154C	YOR126C	-671.25	-668.311	-549.052
2:550000	YBR154C	YLR069C	38.3282	45.0366	242.756
2:550000	YBR154C	YGL040C	-571.66	-570.822	-345.805
2:550000	YBR154C	YMR122W-A	-1354.18	-1348.81	-1275.49
2:550000	YBR154C	YGR034W	-387.335	-376.833	-234.829
2:550000	YBR154C	YBR037C	-98.3984	-93.7378	319.633
2:550000	YBR154C	YER165W	-1065.06	-1061.1	-868.017
2:550000	YBR154C	YIL117C	-73.3144	-64.5721	83.7008
2:550000	YBR154C	YLR382C	-166.981	-158.899	92.099

2:550000	YBR154C	YML129C	-251.383	-241.36	124.227
2:550000	YBR154C	YER063W	-565.821	-564.057	-559.018
2:550000	YBR154C	YDR471W	-34.773	-25.8343	199.782
2:550000	YBR154C	YNL283C	-549.143	-539.3	-541.952
2:550000	YBR154C	YDL130W	-797.502	-791.305	-662.196
2:550000	YBR154C	YKR065C	-595.052	-590.893	-590.806
2:550000	YBR154C	YNR036C	-486.506	-479.614	-427.707
2:550000	YBR154C	YGR076C	-58.5349	-51.2404	64.001
2:550000	YBR154C	YDL061C	-964.303	-957.794	-843.639
2:550000	YBR154C	YHL033C	-459.504	-456.979	-356.449
2:550000	YBR154C	YKL008C	-647.279	-642.58	-586.631
2:550000	YBR154C	YOR158W	-110.31	-100.847	138.76
2:550000	YBR154C	YDL024C	44.6808	46.5374	81.0755
2:550000	YBR154C	YLR054C	-18.8812	-16.3416	2.19766
2:550000	YBR154C	YDL083C	-123.821	-114.759	68.5216
2:550000	YBR154C	YLR353W	-693.128	-688.142	-612.339
2:550000	YBR154C	YJL095W	-774.403	-773.777	-699.045
2:550000	YBR154C	YNL268W	-918.616	-907.991	-883.683
2:550000	YBR154C	YGL129C	-384.93	-380.818	-95.3982
2:550000	YBR154C	YMR012W	-825.344	-824.872	-667.092
2:550000	YBR154C	YMR271C	562.621	564.361	740.167
2:550000	YBR154C	YLR448W	-339.405	-334.251	-121.78
2:550000	YBR154C	YOL045W	-287.984	-277.526	103.837
2:550000	YBR154C	YNL185C	128.136	136.272	283.193
2:550000	YBR154C	YNL327W	-660.004	-656.996	-650.477
2:550000	YBR154C	YNL209W	-324.515	-316.093	-131.868
2:550000	YBR154C	YMR305C	-472.867	-465.053	-346.394
2:550000	YBR154C	YPL143W	-932.422	-927.537	-828.677
2:550000	YBR154C	YOL039W	-846.338	-835.362	-749.792
2:550000	YBR154C	YPL249C-A	-956.256	-950.343	-867.991
2:550000	YBR154C	YHR117W	-754.281	-744.896	-741.06
2:550000	YBR154C	YFR007W	-367.851	-365.872	-96.9866
2:550000	YBR154C	YGR286C	-208.447	-208.309	-152.301
2:550000	YBR154C	YOR354C	-451.973	-449.109	-310.571
2:550000	YBR154C	YIL093C	303.611	310.694	467.733
2:550000	YBR154C	YLR421C	-902.949	-898.722	-880.74
2:550000	YBR154C	YDR025W	-251.754	-242.257	-16.726
2:550000	YBR154C	YCR020C	-86.5455	-77.5438	379.953
2:550000	YBR154C	YBR263W	-885.652	-885.216	-848.232
2:550000	YBR154C	YLL009C	372.347	379.473	427.34
2:550000	YBR154C	YJL159W	-1284.06	-1275.76	-1097.94
2:550000	YBR154C	YCR071C	218.445	226.458	311.759
2:550000	YBR154C	YEL023C	-532.797	-524.34	-395.235
2:550000	YBR154C	YBR115C	-48.9304	-39.8636	-19.7675
2:550000	YBR154C	YOR127W	-516.524	-506.042	-218.457
2:550000	YBR154C	YPL176C	-644.271	-634.221	-469.658
2:550000	YBR154C	YCR031C	-1070	-1069.4	-983.148

2:550000	YBR154C	YER131W	75.0653	86.4986	339.108
2:550000	YBR154C	YGR040W	-383.803	-372.602	-353.425
2:550000	YBR154C	YJL191W	-4.05803	5.52389	140.7
2:550000	YBR154C	YKL170W	-86.5954	-77.7322	162.747
2:550000	YBR154C	YFL036W	-495.548	-483.554	-169.593
2:550000	YBR154C	YHR183W	-1045.84	-1037.76	-770.128
2:550000	YBR154C	YDR462W	-99.1638	-96.3706	64.3765
2:550000	YBR154C	YDL045W-A	-184.658	-177.155	-110.66
2:550000	YBR154C	YDL202W	107.827	113.565	202.606
2:550000	YBR154C	YLR325C	-398.073	-390.121	-204.746
2:550000	YBR154C	YPR075C	-543.248	-537.022	-536.551
2:550000	YBR154C	YPL079W	-605.886	-602.17	-399.884
2:550000	YBR154C	YHR007C	-893.732	-881.544	-802.765
2:550000	YBR154C	YLR061W	-402.155	-400.261	-204.415
2:550000	YBR154C	YKL050C	-345.853	-339.543	-1.81962
2:550000	YBR154C	YBR107C	-516.848	-516.815	-449.743
2:550000	YBR154C	YGR169C	-297.552	-295.645	-282.734
2:550000	YBR154C	YGR220C	-274.482	-266.806	-142.201
2:550000	YBR154C	YDR494W	-261.708	-257.295	26.7342
2:550000	YBR154C	YPR124W	-243.688	-240.442	-213.578
2:550000	YBR154C	YMR101C	-109.022	-103.79	88.849
2:550000	YBR154C	YHR142W	-324.694	-317.555	163.624
2:550000	YBR154C	YER043C	-953.067	-952.194	-952.049
2:550000	YBR154C	YPL098C	-533.544	-527.865	-404.824
2:550000	YBR154C	YMR002W	-500.428	-493.235	-276.069
2:550000	YBR154C	YLR333C	-709.43	-698.845	-492.61
2:550000	YBR154C	YGL202W	-945.659	-943.085	-546.622
2:550000	YBR154C	YCR033W	-906.119	-898.622	-772.325
2:550000	YBR154C	YPL163C	-499.253	-491.532	-494.798
2:550000	YBR154C	YPR132W	-1115.67	-1109.93	-1019.9
2:550000	YBR154C	YDR077W	-1503.5	-1494.49	-1444.76
2:550000	YBR154C	YNL096C	-243.736	-233.686	-71.6528
2:550000	YBR154C	YKR006C	5.11783	6.61093	26.3845
2:550000	YBR154C	YLR289W	-190.57	-183.037	198.5
2:550000	YBR154C	YKL006W	-644.993	-634.583	-514.989
2:550000	YBR154C	YER074W	-575.086	-569.232	-449.267
2:550000	YBR154C	YFR031C-A	-317.172	-306.989	-181.594
2:550000	YBR154C	YBL022C	-416.535	-404.431	-212.187
2:550000	YBR154C	YNL323W	-1087.15	-1081.55	-918.856
2:550000	YBR154C	YDL131W	161.72	170.812	335.304
2:550000	YBR154C	YCL014W	-621.164	-616.654	-444.487
2:550000	YBR154C	YDL082W	-24.3112	-15.5244	287.836
2:550000	YBR154C	YDL154W	61.9486	68.4777	302.347
2:550000	YBR154C	YBR120C	70.035	75.3006	419.952
2:550000	YBR154C	YMR011W	467.044	472.911	644.461
2:550000	YBR154C	YDR322W	183.239	192.51	278.16
2:550000	YBR154C	YBL071C-B	-319.483	-314.241	-284.906

2:550000	YBR154C	YPL040C	-180.852	-176.29	-14.8478
2:550000	YBR154C	YLR432W	-153.405	-142.481	63.7306
2:550000	YBR154C	YER056C-A	-397.392	-388.955	-225.595
2:550000	YBR154C	YKL107W	220.571	224.618	429.446
2:550000	YBR154C	YAL037W	180.126	183.078	423.791
2:550000	YBR154C	YPL104W	-156.092	-151.826	-118.947
2:550000	YBR154C	YPR007C	-132.793	-130.439	-117.816
2:550000	YBR154C	YDL014W	349.308	359.445	752.472
2:550000	YBR154C	YNL302C	-984.88	-975.518	-882.387
2:550000	YBR154C	YIR009W	-250.879	-241.041	-146.816
2:550000	YBR154C	YFR040W	-462.109	-457.464	-316.149
2:550000	YBR154C	YML073C	-488.996	-484.06	-312.084
2:550000	YBR154C	YJR080C	-243.07	-233.372	33.8248
2:550000	YBR154C	YNL315C	96.8968	98.8004	105.882
2:550000	YBR154C	YBR170C	-654.533	-646.425	-253.473
2:550000	YBR154C	YBL087C	-818.923	-810.472	-713.913
2:550000	YBR154C	YOL121C	-159.038	-149.983	87.4709
2:550000	YBR154C	YGL123W	-1044.77	-1035.28	-931.217
2:550000	YBR154C	YNL137C	-280.478	-271.618	-108.316
2:550000	YBR154C	YDL156W	-385.496	-376.056	-295.932
2:550000	YBR154C	YDR385W	-1752.04	-1745.32	-1599.81
2:550000	YBR154C	YMR174C	474.924	475.592	489.789
2:550000	YBR154C	YNL069C	-805.472	-794.956	-674.877
2:550000	YBR154C	YIL031W	-622.715	-611.844	-438.165
2:550000	YBR154C	YBR256C	-434.245	-433.275	-331.399
2:550000	YBR154C	YNL278W	-313.189	-303.808	-25.5296
2:550000	YBR154C	YHR010W	-642.961	-631.545	-519.145
2:550000	YBR154C	YLR285C-A	-51.5793	-42.4294	-48.4532
2:550000	YBR154C	YHR107C	-846.579	-835.707	-575.378
2:550000	YBR154C	YLR138W	-635.908	-634.556	-584.468
2:550000	YBR154C	YLR042C	73.3245	84.6568	138.557
2:550000	YBR154C	YMR032W	-328.934	-328.663	-140.274
2:550000	YBR154C	YKL087C	-268.692	-266.921	76.1374
2:550000	YBR154C	YER074W	-751.404	-739.139	-639.671
2:550000	YBR154C	YJR043C	-593.274	-592.332	-549.048
2:550000	YBR154C	YNL040W	-133.989	-126.3	-18.6792
2:550000	YBR154C	YKL179C	-913.022	-903.181	-770.827
2:550000	YBR154C	YGR138C	208.93	219.603	306.138
2:550000	YBR154C	YOR096W	-584.498	-572.791	-476.329
2:550000	YBR154C	YLR406C	116.288	122.489	339.245
2:550000	YBR154C	YOR293W	-223.296	-214.987	-27.5986
2:550000	YBR154C	YNL078W	-494.444	-484.083	-454.627
2:550000	YBR154C	YGL076C	-1276.13	-1270.05	-1188.88
2:550000	YBR154C	YBR117C	629.13	630.873	631.146
2:550000	YBR154C	YOR298W	135.046	146.115	429.695
2:550000	YBR154C	YEL004W	-624.522	-616.026	-549.038
2:550000	YBR154C	YMR200W	-813.174	-804.406	-789.35

2:550000	YBR154C	YOL007C	-271.656	-261.776	-167.243
2:550000	YBR154C	YPL061W	-151.753	-151.206	23.6929
2:550000	YBR154C	YMR163C	-447.288	-439.767	-127.848
2:550000	YBR154C	YJR034W	68.3836	78.3689	185.585
2:550000	YBR154C	YCL055W	-156.386	-147.128	6.83511
2:550000	YBR154C	YBR191W	-851.278	-841.589	-694.57
2:550000	YBR154C	YPL089C	15.1815	25.6377	244.023
2:550000	YBR154C	YPR074C	-1236.98	-1234.33	-1193.24
2:550000	YBR154C	YDL179W	-177.173	-166.625	-171.438
2:550000	YBR154C	YJR147W	553.676	560.231	557.698
2:550000	YBR154C	YGR085C	-1151.18	-1140.36	-1039.9
2:550000	YBR154C	YPL158C	-540.087	-532.528	-534.017
2:550000	YBR154C	YKL003C	-16.4257	-7.29514	156.811
2:550000	YBR154C	YIL098C	220.537	227.324	391.855
2:550000	YBR154C	YLR390W	-155.751	-146.036	-21.5558
2:550000	YBR154C	YLL045C	-797.494	-785.806	-681.799
2:550000	YBR154C	YER025W	-443.946	-443.561	-308.302
2:550000	YBR154C	YOR316C	-708.718	-700.087	-641.811
2:550000	YBR154C	YHR059W	284.936	286.801	330.681
2:550000	YBR154C	YLR340W	-1108.44	-1099	-953.094
2:550000	YBR154C	YNL301C	-1173.47	-1168.77	-1054.03
2:550000	YBR154C	YBL085W	-490.713	-480.92	-174.442
2:550000	YBR154C	YOL040C	-832.511	-821.71	-731.361
2:550000	YBR154C	YJL160C	-15.0932	-11.701	35.469
2:550000	YBR154C	YBR181C	-1181.47	-1171.51	-1039.34
2:550000	YBR154C	YPL072W	-116.101	-108.285	72.8463
2:550000	YBR154C	YNL176C	-541.657	-539.351	-446.125
2:550000	YBR154C	YJR101W	-159.593	-153.896	-68.7677
2:550000	YBR154C	YBR185C	-55.8078	-46.8097	135.686
2:550000	YBR154C	YLR079W	-790.072	-784.25	-751.294
2:550000	YBR154C	YBR098W	-422.59	-412.164	-214.667
2:550000	YBR154C	YOR247W	-1047.63	-1046.21	-931.071
2:550000	YBR154C	YOR232W	-326.221	-326.15	-266.367
2:550000	YBR154C	YGR152C	-572.733	-566.426	-413.994
2:550000	YBR154C	YML052W	-624.257	-615.884	-371.44
2:550000	YBR154C	YBR237W	-577.696	-571.304	-560.925
2:550000	YBR154C	YHR203C	-1518.89	-1507.7	-1370.72
2:550000	YBR154C	YMR024W	-113.452	-112.843	-75.8836
2:550000	YBR154C	YBL061C	-130.685	-120.526	330.468
2:550000	YBR154C	YBR048W	-896.908	-885.912	-778.754
2:550000	YBR154C	YGL135W	-1463.99	-1451.8	-1285.95
2:550000	YBR154C	YER060W	52.4751	57.9969	155.424
2:550000	YBR154C	YER096W	20.9321	28.8875	141.072
2:550000	YBR154C	YFL022C	-870.548	-869.234	-720.579
2:550000	YBR154C	YNL165W	-511.689	-505.042	-250.615
2:550000	YBR154C	YOR134W	473.048	480.805	595.794
2:550000	YBR154C	YAL024C	-599.58	-587.585	-395.39

2:550000	YBR154C	YAL039C	-111.693	-108.689	-91.5118
2:550000	YBR154C	YNL037C	-23.1254	-20.3813	7.37951
2:550000	YBR154C	YJL177W	-154.482	-146.08	41.23
2:550000	YBR154C	YJR094W-A	-251.498	-241.56	-20.7721
2:550000	YBR154C	YDL075W	-761.3	-750.487	-636.063
2:550000	YBR154C	YJL110C	27.2288	28.598	44.0235
2:550000	YBR154C	YDR115W	-40.6685	-32.9637	119.448
2:550000	YBR154C	YHL001W	-1036.82	-1028.89	-900.48
2:550000	YBR154C	YNL168C	-335.299	-331.985	-161.825
2:550000	YBR154C	YHR021C	-636.756	-625.996	-485.961
2:550000	YBR154C	YPL071C	-263.83	-261.784	-240.862
2:550000	YBR154C	YBR191W	-346.102	-336.083	-227.997
2:550000	YBR154C	YKR013W	-476.469	-465.178	-165.014
2:550000	YBR154C	YPL085W	-707.793	-706.124	-665.575
2:550000	YBR154C	YBL092W	-1004.85	-993.342	-914.529
2:550000	YBR154C	YBR158W	-531.541	-524.574	-342.581
2:550000	YBR154C	YBR119W	-219.586	-211.236	-202.329
2:550000	YBR154C	YKR005C	41.2962	50.6524	420.052
2:550000	YBR154C	YDR381W	-642.162	-631.286	-172.618
2:550000	YBR154C	YJL136C	-956.616	-945.588	-846.426
2:550000	YBR154C	YDR379W	-769.654	-764.866	-750.464
2:550000	YBR154C	YIL133C	-270.839	-263.008	-37.3229
2:550000	YBR154C	YFR031C-A	-1017.37	-1010.98	-932.436
2:550000	YBR154C	YOR293W	-263.987	-255.442	-68.5068
2:550000	YBR165W	YJL147C	-188.285	-185.029	-187.016
2:550000	YBR165W	YDL159W	-746.983	-743.743	-743.882
2:550000	YBR165W	YBR149W	-668.561	-657.668	-661.218
2:550000	YBR165W	YMR182C	107.043	110.824	109.697
2:550000	YBR165W	YPL264C	724.57	736.413	740.707
2:550000	YBR165W	YDR072C	158.46	169.479	164.402
2:550000	YBR165W	YOR264W	-653.337	-641.225	-646.454
2:550000	YBR165W	YBR084C-A	-332.311	-321.218	-317.826
2:550000	YBR165W	YAL003W	-695.971	-695.087	-686.403
2:550000	YBR165W	YDL012C	-431.297	-420.315	-419.632
2:550000	YBR165W	YLR029C	-982.333	-970.107	-966.428
2:550000	YBR165W	YIL052C	-457.211	-446.47	-440.695
2:550000	YBR165W	YOR179C	-431.786	-425.951	-427.828
2:550000	YBR165W	YNL055C	-1196.64	-1193.43	-1193.2
2:550000	YBR165W	YPL131W	-766.672	-761.354	-748.008
2:550000	YBR165W	YJL200C	764.313	776.572	774.689
2:550000	YBR165W	YNR067C	-592.324	-581.659	-587.799
2:550000	YBR165W	YDL055C	-1132.24	-1123.61	-1129.69
2:550000	YBR165W	YMR306W	20.2976	21.4526	21.2181
2:550000	YBR165W	YIL009W	-497.779	-486.854	-487.416
2:550000	YBR165W	YHL001W	-178.089	-169.688	-162.635
2:550000	YBR165W	YDL218W	265.987	275.601	287.377
2:550000	YBR165W	YJR123W	-1008.34	-999.66	-987.729

2:550000	YBR165W	YGR027C	-1044.86	-1033.81	-1023.09
2:550000	YBR165W	YDL229W	10.7543	21.0583	30.3147
2:550000	YBR165W	YDL155W	-391.714	-379.749	-378.668
2:550000	YBR165W	YKL180W	-463.869	-454.477	-436.122
2:550000	YBR165W	YIR034C	469.297	480.774	481.299
2:550000	YBR165W	YLR185W	-367.132	-355.36	-340.376
2:550000	YBR165W	YHR038W	411.444	417.897	420.065
2:550000	YBR165W	YKL081W	-664.822	-658.168	-641.896
2:550000	YBR165W	YCR073C	791.847	803.879	808.179
2:550000	YBR165W	YOL112W	-491.221	-483.654	-489.458
2:550000	YBR165W	YIL094C	-323.279	-312.667	-317.656
2:550000	YBR165W	YBL072C	-1299.51	-1289.64	-1292.55
2:550000	YBR165W	YBL027W	-413.8	-403.249	-396.461
2:550000	YBR165W	YER102W	-609.594	-605.593	-605.716
2:550000	YBR165W	YKR057W	-241.643	-232.03	-225.827
2:550000	YBR165W	YGR085C	-66.0987	-58.0106	-47.0059
2:550000	YBR165W	YGR148C	-1014.5	-1009.81	-999.493
2:550000	YBR165W	YGR214W	-244.882	-236.326	-217.922
2:550000	YBR165W	YLR026C	-319.014	-311.825	-312.11
2:550000	YBR165W	YOR003W	-22.0481	-18.8298	-7.99305
2:550000	YBR165W	YIL009C-A	-262.199	-255.684	-256.09
2:550000	YBR165W	YMR142C	-754.785	-744.992	-730.351
2:550000	YBR165W	YDL229W	-444.644	-437.526	-428.864
2:550000	YBR165W	YPR163C	179.872	185.857	186.532
2:550000	YBR165W	YDR234W	559.787	570.83	564.727
2:550000	YBR165W	YNL073W	-267.765	-265.821	-267.698
2:550000	YBR165W	YKL112W	-566.852	-565.627	-559.245
2:550000	YBR165W	YOL120C	-521.868	-514.308	-505.076
2:550000	YBR165W	YPR181C	-951.803	-942.861	-941.476
2:550000	YBR165W	YLR249W	-351.634	-342.399	-336.88
2:550000	YBR165W	YPL066W	-810.317	-801.146	-807.248
2:550000	YBR165W	YER036C	60.7067	67.5717	65.2484
2:550000	YBR165W	YOR246C	-971.157	-969.986	-958.724
2:550000	YBR165W	YBL049W	345.957	348.444	350.801
2:550000	YBR165W	YBR079C	229.137	229.189	249.487
2:550000	YBR165W	YGR208W	-541.401	-538.401	-538.884
2:550000	YBR165W	YGL189C	-948.955	-940.178	-933.637
2:550000	YBR165W	YIL069C	266.884	269.885	278.244
2:550000	YBR165W	YPR043W	-1148.21	-1141.82	-1136.37
2:550000	YBR165W	YER089C	-788.161	-776.876	-776.592
2:550000	YBR165W	YPL132W	17.3559	25.0831	25.7205
2:550000	YBR165W	YOL127W	-1109.01	-1100.74	-1093.96
2:550000	YBR165W	YPR162C	-448.554	-441.669	-438.212
2:550000	YBR165W	YDR237W	222.52	227.102	222.815
2:550000	YBR165W	YDL182W	191.354	203.482	215.417
2:550000	YBR165W	YMR180C	-13.7497	-3.47645	8.19439
2:550000	YBR165W	YGR084C	94.2837	99.3416	94.9941

2:550000	YBR165W	YDL095W	-820.591	-811.645	-796.717
2:550000	YBR165W	YNL270C	58.5821	67.1272	81.8229
2:550000	YBR165W	YNL240C	644.208	653.943	696.822
2:550000	YBR165W	YCL027W	106.979	114.2	112.026
2:550000	YBR165W	YEL054C	326.936	336.62	346.134
2:550000	YBR165W	YMR242C	-386.916	-376.038	-366.024
2:550000	YBR165W	YGL062W	-965.933	-956.073	-958.552
2:550000	YBR165W	YHR209W	286.84	292.975	294.257
2:550000	YBR165W	YLR150W	-1236.91	-1229.85	-1211.11
2:550000	YBR165W	YLR368W	-433.073	-430.585	-426.84
2:550000	YBR165W	YDR418W	-663.768	-653.292	-639.939
2:550000	YBR165W	YHL042W	366.195	371.05	373.219
2:550000	YBR165W	YJR138W	-321.752	-313.864	-308.186
2:550000	YBR165W	YDR352W	-263.083	-259.674	-260.624
2:550000	YBR165W	YML030W	-118.08	-108.411	-112.509
2:550000	YBR165W	YLR388W	-74.1604	-66.8026	-60.7069
2:550000	YBR165W	YML063W	-662.284	-650.966	-652.566
2:550000	YBR165W	YLL039C	-652.224	-645.948	-612.283
2:550000	YBR165W	YEL058W	-654.078	-645.579	-647.146
2:550000	YBR165W	YAL023C	-959.387	-949.487	-938.865
2:550000	YBR165W	YLR388W	-178.543	-170.477	-164.183
2:550000	YBR165W	YKR094C	-490.89	-480.455	-470.477
2:550000	YBR165W	YLR344W	-140.201	-132.228	-123.816
2:550000	YBR165W	YDR222W	305.145	309.811	305.172
2:550000	YBR165W	YHR089C	777.553	783.308	783.887
2:550000	YBR165W	YLR048W	-21.0339	-12.3214	-3.5445
2:550000	YBR165W	YBR173C	-243.93	-235.492	-215.873
2:550000	YBR165W	YKL204W	-514.233	-504.863	-502.618
2:550000	YBR165W	YBL072C	-790.634	-778.491	-781.41
2:550000	YBR165W	YJL206C	-24.0557	-14.4779	-11.7254
2:550000	YBR165W	YCR099C	239.687	247.577	244.141
2:550000	YBR165W	YOL019W	-238.347	-229.292	-221.758
2:550000	YBR165W	YDL117W	-386.021	-373.716	-363.076
2:550000	YBR165W	YGR281W	-315.202	-305.859	-295.489
2:550000	YBR165W	YIL018W	-237.876	-226.271	-216.236
2:550000	YBR165W	YOR126C	-546.142	-535.124	-540.4
2:550000	YBR165W	YMR122W-A	-1258.78	-1256.18	-1256.1
2:550000	YBR165W	YGR034W	-246.543	-235.437	-227.117
2:550000	YBR165W	YNL293W	-272.638	-271.084	-266.428
2:550000	YBR165W	YBR037C	316.813	325.324	321.749
2:550000	YBR165W	YJL076W	-671.463	-671.288	-670.367
2:550000	YBR165W	YER165W	-860.117	-855.792	-855.492
2:550000	YBR165W	YIL164C	490.201	493.003	504.288
2:550000	YBR165W	YER063W	-556.674	-549.781	-551.172
2:550000	YBR165W	YDR471W	179.909	187.574	202.32
2:550000	YBR165W	YDR508C	352.955	354.41	358.959
2:550000	YBR165W	YNL283C	-538.752	-529.38	-535.148

2:550000	YBR165W	YDL130W	-668.491	-659.63	-650.316
2:550000	YBR165W	YMR118C	629.91	630.687	644.755
2:550000	YBR165W	YDL061C	-834.929	-831.972	-829.706
2:550000	YBR165W	YHL033C	-374.03	-364.9	-348.266
2:550000	YBR165W	YDL024C	65.5184	70.5696	82.9375
2:550000	YBR165W	YOL131W	220.026	220.539	226.113
2:550000	YBR165W	YDL083C	49.9355	59.7375	71.5985
2:550000	YBR165W	YLR353W	-610.587	-604.293	-606.58
2:550000	YBR165W	YJL095W	-695.083	-689.486	-693.617
2:550000	YBR165W	YNL268W	-882.373	-872.707	-875.906
2:550000	YBR165W	YDR098C	-1097.11	-1088	-1063.35
2:550000	YBR165W	YGL129C	-94.837	-88.2761	-89.9726
2:550000	YBR165W	YOL025W	-691.736	-688.306	-684.788
2:550000	YBR165W	YMR271C	716.821	726.966	733.002
2:550000	YBR165W	YLR448W	-137.678	-128.97	-115.789
2:550000	YBR165W	YNL185C	281.617	288.203	288.303
2:550000	YBR165W	YNL209W	-154.381	-144.185	-129.171
2:550000	YBR165W	YPL143W	-835.539	-825.905	-816.471
2:550000	YBR165W	YOL039W	-754.534	-743.296	-740.352
2:550000	YBR165W	YPL249C-A	-870.508	-859.93	-855.704
2:550000	YBR165W	YHR117W	-741.99	-735.597	-734.376
2:550000	YBR165W	YHR202W	136.85	138.562	146.297
2:550000	YBR165W	YGR286C	-181.982	-181.926	-150.437
2:550000	YBR165W	YMR194W	-158.189	-152.219	-144.61
2:550000	YBR165W	YBR103W	-978.654	-974.674	-931.366
2:550000	YBR165W	YBR104W	535.7	546.805	541.447
2:550000	YBR165W	YLR421C	-871.238	-867.125	-870.675
2:550000	YBR165W	YDR025W	-31.2905	-22.7812	-11.5838
2:550000	YBR165W	YCR020C	371.202	373.554	380.127
2:550000	YBR165W	YBR263W	-859.61	-850.807	-839.312
2:550000	YBR165W	YJL159W	-1101.07	-1089.35	-1084.5
2:550000	YBR165W	YJL045W	91.8069	96.077	101.426
2:550000	YBR165W	YJL023C	-394.774	-393.292	-393.769
2:550000	YBR165W	YJL116C	449.134	452.143	456.71
2:550000	YBR165W	YEL023C	-397.309	-387.724	-385.685
2:550000	YBR165W	YBR115C	-22.6855	-12.9306	-15.9507
2:550000	YBR165W	YOR127W	-228.517	-217.457	-211.973
2:550000	YBR165W	YPL176C	-469.005	-460.53	-464.019
2:550000	YBR165W	YCR031C	-995.813	-985.94	-968.323
2:550000	YBR165W	YER131W	322.357	330.021	338.821
2:550000	YBR165W	YGR040W	-360.563	-348.386	-347.178
2:550000	YBR165W	YJL191W	116.947	127.199	143.786
2:550000	YBR165W	YKL170W	163.685	167.603	165.093
2:550000	YBR165W	YFL036W	-167.311	-161.908	-166.602
2:550000	YBR165W	YHR183W	-799.41	-793.48	-766.317
2:550000	YBR165W	YLR188W	-817.224	-817.155	-815.952
2:550000	YBR165W	YLR325C	-222.184	-212.702	-199.576

2:550000	YBR165W	YDR500C	-231.052	-223.417	-211.459
2:550000	YBR165W	YPL079W	-410.487	-404.402	-393.78
2:550000	YBR165W	YHR007C	-796.216	-784.171	-789.921
2:550000	YBR165W	YDR260C	-362.318	-356.726	-357.313
2:550000	YBR165W	YLR061W	-216.318	-209.139	-198.066
2:550000	YBR165W	YBR107C	-488.677	-487.126	-443.018
2:550000	YBR165W	YDR494W	23.2167	29.1382	30.7436
2:550000	YBR165W	YIL146C	311.745	314.23	326.473
2:550000	YBR165W	YNR068C	319.296	322.357	346.556
2:550000	YBR165W	YMR101C	62.579	71.0056	83.77
2:550000	YBR165W	YHR142W	148.337	158.199	162.771
2:550000	YBR165W	YIL088C	-267.965	-259.474	-259.592
2:550000	YBR165W	YMR002W	-277.854	-269.784	-271.707
2:550000	YBR165W	YNL239W	-308.17	-296.726	-283.741
2:550000	YBR165W	YLR333C	-500.504	-492.713	-482.793
2:550000	YBR165W	YGL202W	-553.687	-541.437	-539.363
2:550000	YBR165W	YPL163C	-497.749	-494.197	-495.018
2:550000	YBR165W	YNL046W	-245.166	-241.046	-242.159
2:550000	YBR165W	YKL218C	787.103	791.786	793.564
2:550000	YBR165W	YPR132W	-1008.72	-1005.97	-1005.16
2:550000	YBR165W	YDR077W	-1425.81	-1419.98	-1417.88
2:550000	YBR165W	YER151C	-840.361	-836.338	-840.154
2:550000	YBR165W	YNL096C	-82.594	-75.9522	-69.7265
2:550000	YBR165W	YKL181W	138.967	143.677	143.204
2:550000	YBR165W	YKL006W	-526.925	-515.053	-505.811
2:550000	YBR165W	YER074W	-461.518	-451.225	-437.563
2:550000	YBR165W	YFR031C-A	-193.643	-184.295	-175.107
2:550000	YBR165W	YBL022C	-214.769	-208.55	-205.622
2:550000	YBR165W	YMR181C	52.4515	58.8354	68.3039
2:550000	YBR165W	YNL323W	-931.01	-920.727	-911.599
2:550000	YBR165W	YHL033C	-452.294	-446.728	-436.182
2:550000	YBR165W	YDL131W	321.032	332.586	334.874
2:550000	YBR165W	YCL014W	-444.353	-432.83	-437.795
2:550000	YBR165W	YDL082W	269.483	278.644	289.141
2:550000	YBR165W	YDL154W	288.967	289.373	308.514
2:550000	YBR165W	YBR120C	412.87	419.528	421.534
2:550000	YBR165W	YBL071C-B	-296.216	-290.112	-280.096
2:550000	YBR165W	YJR005W	-556.971	-551.905	-552.47
2:550000	YBR165W	YLR432W	52.5521	58.6299	64.799
2:550000	YBR165W	YER056C-A	-239.628	-227.736	-217.458
2:550000	YBR165W	YKL107W	409.098	415.948	423.172
2:550000	YBR165W	YDL014W	741.991	747.738	748.178
2:550000	YBR165W	YOR286W	-348.377	-344.773	-339.147
2:550000	YBR165W	YNL302C	-885.429	-876.075	-868.825
2:550000	YBR165W	YIR009W	-147.444	-138.231	-139.207
2:550000	YBR165W	YJR133W	-217.943	-211.457	-209.321
2:550000	YBR165W	YML073C	-322.827	-313.615	-305.281

2:550000	YBR165W	YIL131C	-284.179	-281.16	-282.835
2:550000	YBR165W	YJR080C	35.9435	41.6324	35.9872
2:550000	YBR165W	YGR121C	510.717	513.835	514.038
2:550000	YBR165W	YBR148W	96.8027	101.832	105.161
2:550000	YBR165W	YBL087C	-713.167	-704.04	-699.723
2:550000	YBR165W	YGR174C	331.012	340.902	340.916
2:550000	YBR165W	YOL121C	74.4872	85.2585	91.1967
2:550000	YBR165W	YGL123W	-933.802	-925.176	-917.118
2:550000	YBR165W	YNL137C	-103.386	-98.55	-103.227
2:550000	YBR165W	YDL156W	-301.76	-291.788	-289.478
2:550000	YBR165W	YDR385W	-1592.69	-1590.6	-1580.98
2:550000	YBR165W	YGL139W	-654.744	-647.016	-649.037
2:550000	YBR165W	YMR174C	469.185	479.288	488.523
2:550000	YBR165W	YNL069C	-682.196	-671.078	-663.961
2:550000	YBR165W	YIL031W	-445.11	-433.101	-436.887
2:550000	YBR165W	YBR256C	-344.388	-337.58	-321.237
2:550000	YBR165W	YNL278W	-41.027	-30.144	-23.4996
2:550000	YBR165W	YGR067C	72.6172	77.4267	76.2982
2:550000	YBR165W	YHR010W	-524.919	-513.817	-510.36
2:550000	YBR165W	YHR107C	-578.125	-566.163	-565.52
2:550000	YBR165W	YLR042C	132.339	140.696	135.452
2:550000	YBR165W	YKL087C	76.1451	80.8014	79.7954
2:550000	YBR165W	YER074W	-646.853	-635.051	-629.298
2:550000	YBR165W	YJR043C	-564.352	-553.572	-540.423
2:550000	YBR165W	YCR086W	-98.5832	-96.3165	-86.8864
2:550000	YBR165W	YNL040W	-29.3948	-17.2702	-16.1603
2:550000	YBR165W	YKL179C	-797.976	-788.119	-764.466
2:550000	YBR165W	YGR138C	303.048	313.671	307.63
2:550000	YBR165W	YPR105C	-760.73	-758.314	-760.381
2:550000	YBR165W	YOR096W	-478.342	-470.104	-468.188
2:550000	YBR165W	YLR406C	324.273	332.432	341.085
2:550000	YBR165W	YOR293W	-43.2105	-31.7983	-22.0463
2:550000	YBR165W	YNL078W	-448.361	-440.584	-446.477
2:550000	YBR165W	YHL016C	1084.62	1091.32	1085.45
2:550000	YBR165W	YGL076C	-1186.75	-1180.93	-1170.21
2:550000	YBR165W	YBR117C	617.279	618.753	625.612
2:550000	YBR165W	YHR022C	748.013	752.939	771.86
2:550000	YBR165W	YLR378C	-885.779	-874.101	-862.32
2:550000	YBR165W	YOR298W	419.117	427.281	428.62
2:550000	YBR165W	YEL004W	-562.029	-552.115	-544.181
2:550000	YBR165W	YOL007C	-167.348	-156.264	-162.115
2:550000	YBR165W	YMR163C	-141.546	-139.343	-130.425
2:550000	YBR165W	YJR034W	188.753	191.515	188.912
2:550000	YBR165W	YCL055W	2.66931	12.1055	13.6604
2:550000	YBR165W	YBR191W	-691.321	-684.39	-682.588
2:550000	YBR165W	YPL089C	233.113	242.15	243.379
2:550000	YBR165W	YDL179W	-177.816	-166.051	-167.274

2:550000	YBR165W	YGR085C	-1045.7	-1033.57	-1024.85
2:550000	YBR165W	YPL158C	-531.869	-522.062	-528.158
2:550000	YBR165W	YKL003C	157.551	163.459	158.47
2:550000	YBR165W	YIL098C	388.279	399.026	392.874
2:550000	YBR165W	YBR111W-A	90.5221	92.8878	108.407
2:550000	YBR165W	YLL045C	-700.379	-688.77	-671.819
2:550000	YBR165W	YOR315W	516.497	525.147	534.959
2:550000	YBR165W	YOR316C	-656.105	-646.167	-634.975
2:550000	YBR165W	YOR330C	-160.314	-150.513	-150.074
2:550000	YBR165W	YLR340W	-959.138	-947.233	-939.469
2:550000	YBR165W	YNL301C	-1058.64	-1049.69	-1036.07
2:550000	YBR165W	YBL085W	-193.434	-181.505	-172.358
2:550000	YBR165W	YOL040C	-740.966	-731.443	-720.029
2:550000	YBR165W	YJL160C	23.8016	29.6718	36.5971
2:550000	YBR165W	YBR181C	-1030.52	-1021.37	-1023.53
2:550000	YBR165W	YNL176C	-439.931	-433.347	-435.938
2:550000	YBR165W	YJR101W	-73.1331	-64.687	-64.7175
2:550000	YBR165W	YFR028C	-643.229	-639.537	-626.889
2:550000	YBR165W	YLR079W	-750.347	-743.918	-748.723
2:550000	YBR165W	YBR098W	-240.676	-237.973	-209.109
2:550000	YBR165W	YOR247W	-941.325	-938.584	-918.126
2:550000	YBR165W	YAL041W	-761.782	-756.191	-760.892
2:550000	YBR165W	YGR152C	-420.935	-409.004	-405.372
2:550000	YBR165W	YML052W	-376.268	-370.913	-368.518
2:550000	YBR165W	YHR203C	-1384.38	-1376.21	-1353.1
2:550000	YBR165W	YBL061C	313.73	323.382	329.078
2:550000	YBR165W	YBR048W	-783.84	-775.885	-766.464
2:550000	YBR165W	YGL135W	-1283.16	-1273.71	-1268.05
2:550000	YBR165W	YER096W	125.336	126.972	138.541
2:550000	YBR165W	YNL165W	-254.238	-244.523	-250.388
2:550000	YBR165W	YOR134W	555.126	557.485	583.571
2:550000	YBR165W	YAL024C	-392.479	-383.423	-387.553
2:550000	YBR165W	YIL165C	517.418	519.512	534.093
2:550000	YBR165W	YBR126C	303.023	311.833	305.691
2:550000	YBR165W	YAL039C	-87.7236	-83.3164	-84.7794
2:550000	YBR165W	YJL177W	22.8761	32.9038	45.1394
2:550000	YBR165W	YJR094W-A	-32.123	-21.1606	-14.9571
2:550000	YBR165W	YDL075W	-649.201	-636.929	-627.216
2:550000	YBR165W	YJL110C	45.3542	54.9095	50.7596
2:550000	YBR165W	YHL001W	-913.463	-902.299	-887.732
2:550000	YBR165W	YNL168C	-165.256	-154.855	-155.235
2:550000	YBR165W	YHR021C	-495.859	-485.819	-475.783
2:550000	YBR165W	YKR061W	580.549	590.203	594.477
2:550000	YBR165W	YBR191W	-234.76	-226.275	-220.107
2:550000	YBR165W	YMR285C	-880.133	-874.868	-870.379
2:550000	YBR165W	YPL085W	-665.898	-658.076	-655.747
2:550000	YBR165W	YBL092W	-909.072	-897.386	-900.057

2:550000	YBR165W	YBR119W	-213.499	-208.768	-197.196
2:550000	YBR165W	YDR381W	-184.851	-172.918	-168.105
2:550000	YBR165W	YJL136C	-848.285	-838.544	-836.21
2:550000	YBR165W	YIL133C	-48.4786	-42.9019	-33.3422
2:550000	YBR165W	YFR031C-A	-931.386	-925.376	-916.43
2:550000	YBR165W	YOR293W	-81.7358	-70.7614	-62.4484
2:550000	YBR165W	YGR044C	-108.747	-107.935	-95.5061
2:550000	YBR146W	YBR149W	-669.736	-658.855	-660.954
2:550000	YBR146W	YMR182C	78.762	85.7452	103.616
2:550000	YBR146W	YPL264C	730.154	737.499	742.716
2:550000	YBR146W	YDR072C	165.673	174.334	168.271
2:550000	YBR146W	YOR264W	-656.71	-645.398	-649.196
2:550000	YBR146W	YDR210W	-507.518	-506.656	-505.584
2:550000	YBR146W	YDR006C	-765.048	-763.856	-751.089
2:550000	YBR146W	YLR029C	-987.461	-986.667	-971.823
2:550000	YBR146W	YNL055C	-1195.77	-1186.02	-1188.67
2:550000	YBR146W	YJL200C	739.828	746.469	774.678
2:550000	YBR146W	YNR067C	-603.396	-591.339	-591.792
2:550000	YBR146W	YDL055C	-1138.18	-1130.68	-1130.69
2:550000	YBR146W	YIL009W	-508.524	-503.304	-489.743
2:550000	YBR146W	YJR123W	-999.823	-999.436	-990.744
2:550000	YBR146W	YDL155W	-405.33	-404.263	-378.068
2:550000	YBR146W	YOR045W	-689.012	-680.89	-679.798
2:550000	YBR146W	YMR088C	238.525	243.927	241.648
2:550000	YBR146W	YIR034C	476.285	484.224	478.165
2:550000	YBR146W	YCR073C	769.273	771.789	806.504
2:550000	YBR146W	YOL112W	-503.401	-492.001	-484.524
2:550000	YBR146W	YDL057W	96.4939	99.4554	97.1527
2:550000	YBR146W	YAR014C	-380.479	-373.357	-332.272
2:550000	YBR146W	YIL009C-A	-263.09	-261.678	-259.523
2:550000	YBR146W	YDR090C	29.9097	34.2949	81.6739
2:550000	YBR146W	YDR234W	553.555	564.699	564.181
2:550000	YBR146W	YIR030C	905.518	915.936	925.717
2:550000	YBR146W	YBR162C	-722.07	-714.494	-719.599
2:550000	YBR146W	YPL066W	-803.139	-797.82	-802.576
2:550000	YBR146W	YER036C	67.6933	70.6045	69.8149
2:550000	YBR146W	YGL189C	-948.8	-946.592	-938.699
2:550000	YBR146W	YER089C	-793.814	-783.956	-773.484
2:550000	YBR146W	YDL182W	186.024	188.059	215.374
2:550000	YBR146W	YGL062W	-970.234	-961.456	-960.935
2:550000	YBR146W	YDR352W	-264.446	-259.66	-263.099
2:550000	YBR146W	YML030W	-122.672	-116.169	-119.202
2:550000	YBR146W	YML063W	-664.899	-659.105	-658.588
2:550000	YBR146W	YKL164C	-944.596	-937.567	-941.613
2:550000	YBR146W	YBL072C	-836.684	-834.387	-801.493
2:550000	YBR146W	YJL206C	-12.1528	-7.88458	-12.1313
2:550000	YBR146W	YCR099C	241.996	248.208	242.074

2:550000	YBR146W	YMR205C	-1081.39	-1075.72	-1067.52
2:550000	YBR146W	YMR236W	-873.966	-873.039	-864.734
2:550000	YBR146W	YOL019W	-246.149	-242.546	-225.839
2:550000	YBR146W	YDL117W	-386.592	-380.507	-364.082
2:550000	YBR146W	YGR281W	-292.763	-286.914	-291.024
2:550000	YBR146W	YOR126C	-553.338	-543.958	-541.95
2:550000	YBR146W	YBR037C	319.449	327.621	322.172
2:550000	YBR146W	YER063W	-569.297	-557.692	-548.786
2:550000	YBR146W	YNL283C	-545.507	-533.944	-531.101
2:550000	YBR146W	YKR065C	-574.542	-567.157	-573.183
2:550000	YBR146W	YLR353W	-630.141	-618.694	-609.601
2:550000	YBR146W	YNL268W	-891.656	-880.099	-877.476
2:550000	YBR146W	YJR001W	-947.754	-946.304	-920.522
2:550000	YBR146W	YDL231C	-436.871	-433.23	-436.424
2:550000	YBR146W	YMR305C	-362.02	-350.859	-333.789
2:550000	YBR146W	YHR117W	-756.745	-744.616	-738.51
2:550000	YBR146W	YHR012W	-706.998	-705.056	-686.923
2:550000	YBR146W	YBR104W	519.749	530.821	545.08
2:550000	YBR146W	YLR421C	-914.399	-902.977	-869.15
2:550000	YBR146W	YJL159W	-1107.26	-1100.71	-1089.88
2:550000	YBR146W	YJL116C	420.916	431.087	463.573
2:550000	YBR146W	YBR115C	-25.7958	-13.8	-15.6089
2:550000	YBR146W	YOR127W	-236.665	-235.1	-212.923
2:550000	YBR146W	YPL176C	-478.558	-478.041	-467.246
2:550000	YBR146W	YGR040W	-354.966	-344.303	-348.531
2:550000	YBR146W	YLR209C	-1004.48	-999.003	-955.293
2:550000	YBR146W	YPR075C	-528.817	-527.971	-528.626
2:550000	YBR146W	YHR007C	-798.721	-788.867	-791.119
2:550000	YBR146W	YOR296W	-501.014	-495.112	-499.512
2:550000	YBR146W	YIL088C	-261.585	-257.184	-261.108
2:550000	YBR146W	YNL239W	-284.624	-281.314	-284.127
2:550000	YBR146W	YPL163C	-489.399	-485.493	-488.227
2:550000	YBR146W	YDL131W	329.642	337.405	332.877
2:550000	YBR146W	YCL014W	-447.658	-438.004	-435.979
2:550000	YBR146W	YJR005W	-544.302	-536.248	-539.405
2:550000	YBR146W	YFL021W	524.834	535.907	576.169
2:550000	YBR146W	YOR286W	-358.006	-352.54	-350.092
2:550000	YBR146W	YIR009W	-143.039	-136.143	-142.267
2:550000	YBR146W	YJR133W	-221.511	-211.477	-212.978
2:550000	YBR146W	YDR294C	-967.637	-966.379	-966.824
2:550000	YBR146W	YBR148W	86.9079	88.1892	101.096
2:550000	YBR146W	YIL031W	-439.474	-430.692	-432.467
2:550000	YBR146W	YOR111W	-135.425	-133.419	-134.753
2:550000	YBR146W	YLR285C-A	-47.9382	-39.3151	-45.4501
2:550000	YBR146W	YHR107C	-605.04	-598.368	-567.156
2:550000	YBR146W	YLR042C	135.319	147.166	141.021
2:550000	YBR146W	YMR173W	-537.167	-535.292	-534.277

2:550000	YBR146W	YCR086W	-171.28	-162.914	-88.9939
2:550000	YBR146W	YNL040W	-28.8732	-17.8714	-23.9828
2:550000	YBR146W	YGR138C	300.618	309.549	304.886
2:550000	YBR146W	YNL078W	-450.254	-439.977	-446.126
2:550000	YBR146W	YBR117C	627.566	630.376	629.135
2:550000	YBR146W	YEL004W	-554.077	-543.264	-548.917
2:550000	YBR146W	YOL007C	-171.937	-161.548	-166.787
2:550000	YBR146W	YJR034W	186.69	192.285	187.278
2:550000	YBR146W	YPL089C	243.025	245.672	245.141
2:550000	YBR146W	YPR074C	-1190.65	-1181.43	-1179.27
2:550000	YBR146W	YDL179W	-171.089	-160.3	-166.179
2:550000	YBR146W	YJR147W	554.634	557.782	557.405
2:550000	YBR146W	YGR085C	-1046.2	-1045.08	-1036.94
2:550000	YBR146W	YIL098C	383.926	390.012	388.542
2:550000	YBR146W	YBR111W-A	76.8924	84.774	93.7636
2:550000	YBR146W	YOR316C	-642.366	-630.954	-633.263
2:550000	YBR146W	YBL085W	-184.257	-179.286	-172.493
2:550000	YBR146W	YJL160C	31.1678	35.1437	35.5661
2:550000	YBR146W	YLR079W	-751.799	-744.105	-750.086
2:550000	YBR146W	YOR247W	-947.22	-936.16	-914.582
2:550000	YBR146W	YAL041W	-764.766	-756.52	-759.33
2:550000	YBR146W	YNL165W	-261.568	-252.049	-248.086
2:550000	YBR146W	YAL024C	-426.571	-416.548	-392.721
2:550000	YBR146W	YBR126C	301.835	306.234	306.006
2:550000	YBR146W	YHL023C	-585.631	-581.316	-577.238
2:550000	YBR146W	YJL110C	43.3225	52.4119	49.058
2:550000	YBR146W	YNL168C	-160.238	-149.525	-155.674
2:550000	YBR146W	YPL071C	-234.071	-229.285	-231.063
2:550000	YBR146W	YKR061W	557.108	564.199	594.398
2:550000	YBR146W	YBR119W	-201.849	-192.007	-197.953
2:550000	YBR146W	YOL082W	85.8484	92.9736	102.02
2:550000	YBR166C	YKL138C	220.574	231.502	254.069
2:550000	YBR166C	YIR021W	-60.0444	-48.0841	-25.2941
2:550000	YBR166C	YKL195W	-32.3608	-22.1549	12.3832
2:550000	YBR166C	YBR149W	-687.907	-686.457	-661.218
2:550000	YBR166C	YLR286C	-424.222	-415.365	-391.473
2:550000	YBR166C	YMR182C	108.912	115.575	109.697
2:550000	YBR166C	YPL264C	720.8	722.996	740.707
2:550000	YBR166C	YOR187W	-464.495	-459.45	-386.059
2:550000	YBR166C	YDR072C	152.589	158.546	164.402
2:550000	YBR166C	YBR157C	641.005	652.576	669.376
2:550000	YBR166C	YOR264W	-652.851	-640.554	-646.454
2:550000	YBR166C	YDR006C	-765.783	-756.89	-750.855
2:550000	YBR166C	YBR084C-A	-321.136	-318.684	-317.826
2:550000	YBR166C	YDL012C	-426.95	-426.779	-419.632
2:550000	YBR166C	YKL194C	58.3772	68.7217	105.927
2:550000	YBR166C	YLR029C	-974.792	-967.195	-966.428

2:550000	YBR166C	YIL052C	-443.578	-439.772	-440.695
2:550000	YBR166C	YNL055C	-1216.67	-1216.4	-1193.2
2:550000	YBR166C	YJL200C	752.488	760.814	774.689
2:550000	YBR166C	YNR067C	-592.736	-582.911	-587.799
2:550000	YBR166C	YDL055C	-1140.52	-1128.49	-1129.69
2:550000	YBR166C	YIL009W	-491.17	-481.412	-487.416
2:550000	YBR166C	YJR123W	-989.839	-982.715	-987.729
2:550000	YBR166C	YGR027C	-1025.83	-1018.2	-1023.09
2:550000	YBR166C	YDL155W	-389.872	-383.977	-378.668
2:550000	YBR166C	YOR045W	-719.446	-708.407	-682.642
2:550000	YBR166C	YNR020C	11.7877	21.5935	32.7017
2:550000	YBR166C	YLR185W	-345.42	-344.519	-340.376
2:550000	YBR166C	YHR038W	412.999	421.988	420.065
2:550000	YBR166C	YOL112W	-490.369	-487.713	-489.458
2:550000	YBR166C	YIL094C	-327.504	-316.413	-317.656
2:550000	YBR166C	YBL072C	-1297.81	-1286.63	-1292.55
2:550000	YBR166C	YBL027W	-396.981	-393.532	-396.461
2:550000	YBR166C	YLR026C	-321.303	-312.773	-312.11
2:550000	YBR166C	YJL063C	63.4327	75.5062	112.027
2:550000	YBR166C	YNL073W	-312.248	-303.713	-267.698
2:550000	YBR166C	YGL068W	210.294	220.741	241.89
2:550000	YBR166C	YBR162C	-784.387	-773.346	-728.511
2:550000	YBR166C	YPR181C	-942.711	-935.597	-941.476
2:550000	YBR166C	YPL066W	-814.378	-807.184	-807.248
2:550000	YBR166C	YPR005C	187.166	198.959	201.245
2:550000	YBR166C	YDR347W	-38.5935	-29.8441	7.45929
2:550000	YBR166C	YGL189C	-936.862	-929.273	-933.637
2:550000	YBR166C	YMR225C	-5.82195	6.00202	41.0046
2:550000	YBR166C	YER089C	-782.235	-772.053	-776.592
2:550000	YBR166C	YPL132W	19.0778	29.1731	25.7205
2:550000	YBR166C	YOL127W	-1095.88	-1089.95	-1093.96
2:550000	YBR166C	YDR237W	195.556	207.364	222.815
2:550000	YBR166C	YDL182W	207.279	211.775	215.417
2:550000	YBR166C	YGR084C	79.6653	90.5265	94.9941
2:550000	YBR166C	YCL027W	111.786	118.176	112.026
2:550000	YBR166C	YNR037C	14.2191	24.5966	74.7635
2:550000	YBR166C	YBR165W	-710.694	-705.402	-698.728
2:550000	YBR166C	YMR242C	-368.439	-367.779	-366.024
2:550000	YBR166C	YGL062W	-963.444	-952.403	-958.552
2:550000	YBR166C	YIL070C	490.942	502.881	530.612
2:550000	YBR166C	YJR045C	-659.583	-649.945	-647.536
2:550000	YBR166C	YKR031C	-247.012	-245.882	-223.299
2:550000	YBR166C	YML030W	-143.548	-134.418	-112.509
2:550000	YBR166C	YLR259C	-845.089	-834.119	-825.938
2:550000	YBR166C	YML063W	-657.992	-646.643	-652.566
2:550000	YBR166C	YKR094C	-472.301	-471.49	-470.477
2:550000	YBR166C	YKL164C	-962.311	-950.558	-940.51

2:550000	YBR166C	YIL140W	-486.595	-477.608	-473.457
2:550000	YBR166C	YKL204W	-504.762	-496.683	-502.618
2:550000	YBR166C	YBL072C	-786.351	-775.257	-781.41
2:550000	YBR166C	YDR430C	47.1544	56.335	95.4493
2:550000	YBR166C	YMR236W	-859.573	-854.737	-859.467
2:550000	YBR166C	YER154W	-329.892	-326.7	-311.311
2:550000	YBR166C	YOL019W	-229.09	-222.366	-221.758
2:550000	YBR166C	YDL117W	-377.309	-375.195	-363.076
2:550000	YBR166C	YIL018W	-218.124	-212.122	-216.236
2:550000	YBR166C	YER095W	-563.213	-557.793	-554.752
2:550000	YBR166C	YOR126C	-545.323	-535.195	-540.4
2:550000	YBR166C	YLR069C	215.394	224.698	249.262
2:550000	YBR166C	YBR167C	-304.103	-298.181	-297.25
2:550000	YBR166C	YGR034W	-230.072	-225.103	-227.117
2:550000	YBR166C	YBR037C	295.39	306.446	321.749
2:550000	YBR166C	YLR382C	57.3055	65.4093	102.479
2:550000	YBR166C	YML129C	91.1053	102.158	129.191
2:550000	YBR166C	YGR082W	-150.902	-147.356	-143.546
2:550000	YBR166C	YNL283C	-571.041	-562.136	-535.148
2:550000	YBR166C	YBR146W	-92.8111	-86.2594	-62.9164
2:550000	YBR166C	YKR065C	-649.527	-640.753	-581.835
2:550000	YBR166C	YNR036C	-467.598	-456.694	-419.769
2:550000	YBR166C	YGR076C	39.097	50.1579	69.259
2:550000	YBR166C	YKL008C	-579.67	-575.274	-579.512
2:550000	YBR166C	YOR158W	109.694	120.275	143.346
2:550000	YBR166C	YBR091C	10.3748	18.0554	15.6324
2:550000	YBR166C	YLR353W	-610.59	-601.049	-606.58
2:550000	YBR166C	YJL095W	-694.449	-687.905	-693.617
2:550000	YBR166C	YNL268W	-882.081	-875.159	-875.906
2:550000	YBR166C	YGL129C	-108.289	-96.6555	-89.9726
2:550000	YBR166C	YNL185C	274.572	285.18	288.303
2:550000	YBR166C	YMR305C	-373.325	-370.95	-341.697
2:550000	YBR166C	YOL039W	-743.126	-736.294	-740.352
2:550000	YBR166C	YPL249C-A	-856.298	-851.041	-855.704
2:550000	YBR166C	YHR117W	-736.062	-731.795	-734.376
2:550000	YBR166C	YFR007W	-119.236	-107.397	-90.8147
2:550000	YBR166C	YOR354C	-324.607	-315.019	-301.769
2:550000	YBR166C	YIL093C	436.455	448.48	466.623
2:550000	YBR166C	YBR104W	536.152	546.555	541.447
2:550000	YBR166C	YLL009C	390.956	402.984	428.058
2:550000	YBR166C	YCR071C	275.726	287.944	313.227
2:550000	YBR166C	YHL038C	-466.993	-456.507	-446.061
2:550000	YBR166C	YJL116C	456.318	461.75	456.71
2:550000	YBR166C	YEL023C	-388.874	-379.873	-385.685
2:550000	YBR166C	YBR115C	-27.7597	-15.8049	-15.9507
2:550000	YBR166C	YOR127W	-215.705	-208.41	-211.973
2:550000	YBR166C	YGR040W	-355.289	-344.339	-347.178

2:550000	YBR166C	YKL170W	150.038	160.353	165.093
2:550000	YBR166C	YFL036W	-172.157	-163.291	-166.602
2:550000	YBR166C	YDR462W	43.4629	53.4836	70.7903
2:550000	YBR166C	YDL045W-A	-145.033	-132.769	-105.458
2:550000	YBR166C	YDL202W	184.936	196.595	205.494
2:550000	YBR166C	YPR075C	-536.521	-526.43	-526.318
2:550000	YBR166C	YHR007C	-808.892	-800.111	-789.921
2:550000	YBR166C	YBR107C	-460.358	-455.9	-443.018
2:550000	YBR166C	YGR169C	-296.17	-285.511	-273.529
2:550000	YBR166C	YGR220C	-173.18	-160.982	-134.838
2:550000	YBR166C	YDR194C	-217.826	-210.101	-205.836
2:550000	YBR166C	YDR494W	21.9683	31.4872	30.7436
2:550000	YBR166C	YPR124W	-223.751	-215.442	-210.504
2:550000	YBR166C	YHR142W	158.707	168.922	162.771
2:550000	YBR166C	YPL098C	-405.833	-402.879	-388.357
2:550000	YBR166C	YMR002W	-301.47	-289.473	-271.707
2:550000	YBR166C	YGL202W	-547.961	-539.243	-539.363
2:550000	YBR166C	YCR033W	-762.06	-757.052	-761.006
2:550000	YBR166C	YKR006C	-20.7618	-10.0686	33.2436
2:550000	YBR166C	YLR289W	175.022	186.908	206.29
2:550000	YBR166C	YKL006W	-509.214	-502.661	-505.811
2:550000	YBR166C	YFR031C-A	-176.122	-173.712	-175.107
2:550000	YBR166C	YBL022C	-221.662	-210.606	-205.622
2:550000	YBR166C	YNL323W	-912.03	-909.622	-911.599
2:550000	YBR166C	YCL014W	-447.963	-438.557	-437.795
2:550000	YBR166C	YBR120C	408.024	417.143	421.534
2:550000	YBR166C	YDR322W	228.311	237.184	280.028
2:550000	YBR166C	YGL028C	207.482	208.711	236.073
2:550000	YBR166C	YPL040C	-18.2597	-9.16036	-7.09279
2:550000	YBR166C	YER056C-A	-225.019	-224.003	-217.458
2:550000	YBR166C	YPL104W	-175.424	-167.694	-112.866
2:550000	YBR166C	YOR286W	-404.536	-400.935	-339.147
2:550000	YBR166C	YJR133W	-237.759	-228.352	-209.321
2:550000	YBR166C	YJR080C	-10.7505	-2.72905	35.9872
2:550000	YBR166C	YNL315C	56.3392	62.4407	112.692
2:550000	YBR166C	YGR121C	488.045	494.686	514.038
2:550000	YBR166C	YBR148W	91.4952	103.528	105.161
2:550000	YBR166C	YNL137C	-130.688	-118.668	-103.227
2:550000	YBR166C	YDL156W	-290.723	-284.213	-289.478
2:550000	YBR166C	YNL069C	-666.097	-658.851	-663.961
2:550000	YBR166C	YIL031W	-443.715	-434.338	-436.887
2:550000	YBR166C	YNL278W	-25.4362	-17.3794	-23.4996
2:550000	YBR166C	YHR010W	-514.004	-506.311	-510.36
2:550000	YBR166C	YLR285C-A	-50.4299	-44.4989	-49.8306
2:550000	YBR166C	YHR107C	-573.8	-562.517	-565.52
2:550000	YBR166C	YLR042C	110.848	122.013	135.452
2:550000	YBR166C	YKL087C	75.2151	82.1898	79.7954

2:550000	YBR166C	YJL062W-A	-232.31	-222.727	-144.242
2:550000	YBR166C	YER074W	-634.864	-623.171	-629.298
2:550000	YBR166C	YJR043C	-542.222	-537.549	-540.423
2:550000	YBR166C	YCR086W	-87.8797	-80.7337	-86.8864
2:550000	YBR166C	YCR046C	-128.736	-116.737	-66.3095
2:550000	YBR166C	YGR138C	303.335	313.479	307.63
2:550000	YBR166C	YPR105C	-760.594	-754.229	-760.381
2:550000	YBR166C	YOR096W	-470.418	-462.695	-468.188
2:550000	YBR166C	YOR293W	-25.585	-22.396	-22.0463
2:550000	YBR166C	YNL078W	-451.278	-444.426	-446.477
2:550000	YBR166C	YLR378C	-869.015	-866.619	-862.32
2:550000	YBR166C	YOR298W	428.598	433.493	428.62
2:550000	YBR166C	YEL004W	-551.738	-546.228	-544.181
2:550000	YBR166C	YOL007C	-171.206	-161.35	-162.115
2:550000	YBR166C	YJR034W	124.649	132.413	188.912
2:550000	YBR166C	YBR191W	-683.582	-676.607	-682.588
2:550000	YBR166C	YDL179W	-190.472	-190.182	-167.274
2:550000	YBR166C	YJR147W	529.147	534.604	551.418
2:550000	YBR166C	YGR085C	-1031.21	-1022.84	-1024.85
2:550000	YBR166C	YKL003C	124.347	136.614	158.47
2:550000	YBR166C	YIL098C	356.26	362.891	392.874
2:550000	YBR166C	YLR390W	-67.656	-58.3226	-15.9846
2:550000	YBR166C	YBR111W-A	100.586	112.255	108.407
2:550000	YBR166C	YLL045C	-674.447	-667.547	-671.819
2:550000	YBR166C	YHR059W	300.668	310.344	333.887
2:550000	YBR166C	YML009C	-190.615	-178.315	-136.093
2:550000	YBR166C	YOR330C	-159.372	-148.013	-150.074
2:550000	YBR166C	YLR340W	-943.439	-937.01	-939.469
2:550000	YBR166C	YBL085W	-181.302	-176.652	-172.358
2:550000	YBR166C	YOL040C	-721.72	-718.722	-720.029
2:550000	YBR166C	YBR181C	-1027.36	-1017.5	-1023.53
2:550000	YBR166C	YPL072W	48.6166	60.2164	78.1404
2:550000	YBR166C	YJR101W	-71.2915	-61.0858	-64.7175
2:550000	YBR166C	YBR185C	114.01	123.566	139.464
2:550000	YBR166C	YLR079W	-756.815	-747.236	-748.723
2:550000	YBR166C	YOR247W	-944.901	-934.446	-918.126
2:550000	YBR166C	YOR232W	-261.149	-249.604	-246.164
2:550000	YBR166C	YGR152C	-416.602	-408.841	-405.372
2:550000	YBR166C	YBR237W	-563.673	-557.702	-559.778
2:550000	YBR166C	YHR203C	-1353.76	-1353.17	-1353.1
2:550000	YBR166C	YMR024W	-113.628	-101.497	-71.5151
2:550000	YBR166C	YBR048W	-768.226	-765.089	-766.464
2:550000	YBR166C	YGL135W	-1271.86	-1265.12	-1268.05
2:550000	YBR166C	YNL165W	-269.305	-265.753	-250.388
2:550000	YBR166C	YAL024C	-391.309	-381.894	-387.553
2:550000	YBR166C	YNL037C	-9.65748	-1.38439	12.0376
2:550000	YBR166C	YDL075W	-633.21	-625.339	-627.216

2:550000	YBR166C	YJL110C	48.19	52.5565	50.7596
2:550000	YBR166C	YDR115W	88.7915	100.918	121.525
2:550000	YBR166C	YPL063W	-1007.91	-1001.19	-1004.2
2:550000	YBR166C	YHR021C	-478.353	-477.672	-475.783
2:550000	YBR166C	YKR061W	590.261	600.611	594.477
2:550000	YBR166C	YBR191W	-221.275	-215.053	-220.107
2:550000	YBR166C	YKR013W	-169.377	-159.464	-161.614
2:550000	YBR166C	YPL085W	-656.497	-649.708	-655.747
2:550000	YBR166C	YBL092W	-904.871	-893.904	-900.057
2:550000	YBR166C	YIL149C	-810.597	-810.303	-807.385
2:550000	YBR166C	YBR119W	-224.023	-215.537	-197.196
2:550000	YBR166C	YDR381W	-172.985	-163.108	-168.105
2:550000	YBR166C	YJL136C	-837.836	-834.143	-836.21
2:550000	YBR166C	YOR293W	-64.0996	-61.2886	-62.4484
2:550000	YBR148W	YER059W	-362.586	-354.413	-343.431
2:550000	YBR148W	YJL147C	-194.904	-184.309	-189.148
2:550000	YBR148W	YNL216W	-384.317	-378.772	-384.072
2:550000	YBR148W	YEL006W	-390.778	-382.899	-385.432
2:550000	YBR148W	YBR149W	-667.243	-655.563	-661.191
2:550000	YBR148W	YMR182C	88.9104	98.0837	96.1602
2:550000	YBR148W	YPL264C	740.387	750.157	744.428
2:550000	YBR148W	YBR285W	719.704	722.118	732.554
2:550000	YBR148W	YDR072C	161.633	170.95	168.23
2:550000	YBR148W	YOR264W	-648.349	-636.322	-642.467
2:550000	YBR148W	YBR084C-A	-317.541	-315.611	-316.684
2:550000	YBR148W	YGL196W	-228.222	-217.262	-193.926
2:550000	YBR148W	YDL012C	-422.332	-418.059	-420.481
2:550000	YBR148W	YLR029C	-970.033	-965.268	-966.9
2:550000	YBR148W	YOR179C	-434.159	-422.302	-424.369
2:550000	YBR148W	YNR067C	-595.179	-583.524	-588.208
2:550000	YBR148W	YML124C	-1237.82	-1228.36	-1233.3
2:550000	YBR148W	YHL001W	-180.223	-170.188	-168.426
2:550000	YBR148W	YDL218W	291.449	291.903	292.786
2:550000	YBR148W	YDL155W	-384.954	-378.351	-380.843
2:550000	YBR148W	YMR088C	219.182	226.02	233.819
2:550000	YBR148W	YIR034C	465.784	476.01	477.802
2:550000	YBR148W	YLR185W	-346.046	-345.503	-343.363
2:550000	YBR148W	YKL081W	-643.159	-636.908	-642.053
2:550000	YBR148W	YOL112W	-496.478	-485.567	-488.057
2:550000	YBR148W	YDL057W	78.035	88.5365	91.8531
2:550000	YBR148W	YGR085C	-59.7287	-49.9959	-56.0407
2:550000	YBR148W	YDR345C	-139.552	-130.215	-129.511
2:550000	YBR148W	YOR003W	-22.8878	-20.3186	-6.32642
2:550000	YBR148W	YIL009C-A	-263.031	-256.52	-262.567
2:550000	YBR148W	YBR135W	-648.593	-636.989	-631.682
2:550000	YBR148W	YER064C	974.561	983.181	978.83
2:550000	YBR148W	YPR163C	176.54	188.662	184.201

2:550000	YBR148W	YKL112W	-570.717	-558.91	-554.47
2:550000	YBR148W	YPL107W	-182.022	-171.825	-174.703
2:550000	YBR148W	YOL020W	-484.99	-476.429	-477.408
2:550000	YBR148W	YPL066W	-803.173	-797.869	-796.58
2:550000	YBR148W	YPR005C	189.736	200.828	196.22
2:550000	YBR148W	YER036C	55.2956	67.3363	66.6118
2:550000	YBR148W	YOR246C	-963.933	-958.838	-959.988
2:550000	YBR148W	YBL049W	354.065	357.332	355.606
2:550000	YBR148W	YBR079C	232.609	244.838	247.723
2:550000	YBR148W	YGR208W	-553.019	-544.89	-540.883
2:550000	YBR148W	YAL035W	-163.176	-152.715	-155.601
2:550000	YBR148W	YPR043W	-1139.04	-1137.16	-1135.7
2:550000	YBR148W	YBR154C	266.057	278.264	291.638
2:550000	YBR148W	YEL054C	340.223	343.683	340.475
2:550000	YBR148W	YGL062W	-966.962	-960.311	-966.464
2:550000	YBR148W	YHR209W	281.88	283.724	298.837
2:550000	YBR148W	YMR170C	338.603	346.495	374.632
2:550000	YBR148W	YGL185C	-571.537	-563.308	-567.618
2:550000	YBR148W	YJR138W	-320.298	-319.993	-315.57
2:550000	YBR148W	YDR352W	-279.217	-268.95	-269.294
2:550000	YBR148W	YML063W	-669.315	-661.793	-665.3
2:550000	YBR148W	YML118W	96.5993	107.001	101.936
2:550000	YBR148W	YAL023C	-937.816	-935.159	-935.334
2:550000	YBR148W	YBR228W	-178.646	-171.95	-178.02
2:550000	YBR148W	YKR094C	-477.174	-473.899	-477.04
2:550000	YBR148W	YLR344W	-136.085	-126.847	-132.997
2:550000	YBR148W	YDR222W	283.925	284.31	300.289
2:550000	YBR148W	YHR089C	773.08	784.777	779.879
2:550000	YBR148W	YNL015W	-160.738	-150.779	-146.364
2:550000	YBR148W	YOR056C	237.62	240.685	239.563
2:550000	YBR148W	YJL206C	-23.3981	-12.2919	-18.1506
2:550000	YBR148W	YCR099C	242.635	251.169	246.337
2:550000	YBR148W	YMR205C	-1087.87	-1075.75	-1069.25
2:550000	YBR148W	YMR058W	-181.375	-169.538	-151.044
2:550000	YBR148W	YOR126C	-546.541	-539.023	-540.693
2:550000	YBR148W	YGL164C	-856.232	-850.533	-850.848
2:550000	YBR148W	YPR134W	-284.789	-276.359	-277.002
2:550000	YBR148W	YJL076W	-680.435	-672.095	-676.214
2:550000	YBR148W	YER063W	-568.792	-558.215	-550.322
2:550000	YBR148W	YDR471W	185.663	197.107	192.93
2:550000	YBR148W	YOR205C	-45.1524	-41.5846	-42.7396
2:550000	YBR148W	YEL060C	-396.626	-391.952	-393.474
2:550000	YBR148W	YDL024C	81.8901	87.4808	83.1665
2:550000	YBR148W	YOL131W	206.054	217.637	214.005
2:550000	YBR148W	YLR353W	-628.701	-619.84	-602.367
2:550000	YBR148W	YJL145W	-506.801	-496.973	-493.496
2:550000	YBR148W	YGR019W	84.1921	87.5878	122.724

2:550000	YBR148W	YGR055W	560.69	566.835	567.31
2:550000	YBR148W	YNL268W	-885.876	-883.034	-871.103
2:550000	YBR148W	YDR098C	-1076.28	-1065.71	-1071.72
2:550000	YBR148W	YJR001W	-930.442	-924.61	-918.638
2:550000	YBR148W	YMR271C	720.56	729.227	730.447
2:550000	YBR148W	YLR448W	-124.835	-118.012	-124.09
2:550000	YBR148W	YOL039W	-742.39	-738.264	-738.476
2:550000	YBR148W	YER019W	-645.264	-640.119	-633.766
2:550000	YBR148W	YMR194W	-160.213	-151.927	-156.252
2:550000	YBR148W	YBR196C-B	22.6681	23.0465	61.725
2:550000	YBR148W	YHR012W	-715.829	-706.262	-686.449
2:550000	YBR148W	YLR421C	-886.283	-882	-867.9
2:550000	YBR148W	YBR263W	-853.758	-845.917	-838.538
2:550000	YBR148W	YJL159W	-1091.84	-1080.44	-1086.53
2:550000	YBR148W	YJL172W	95.9968	96.4054	105.271
2:550000	YBR148W	YJL045W	92.5234	101.241	95.0883
2:550000	YBR148W	YJL116C	402.694	405.615	449.97
2:550000	YBR148W	YPR144C	469.758	481.294	485.871
2:550000	YBR148W	YBR115C	-26.3922	-14.7903	-17.5223
2:550000	YBR148W	YPL176C	-463.912	-456.004	-461.905
2:550000	YBR148W	YNL282W	177.289	181.701	193.602
2:550000	YBR148W	YGR040W	-355.283	-343.604	-349.693
2:550000	YBR148W	YJL191W	129.199	138.131	138.141
2:550000	YBR148W	YMR155W	-261.933	-257.7	-253.945
2:550000	YBR148W	YPL171C	92.117	103.031	102.806
2:550000	YBR148W	YDL169C	411.634	415.105	425.01
2:550000	YBR148W	YMR255W	-673.15	-664.381	-665.333
2:550000	YBR148W	YLR209C	-1003.85	-992.329	-962.815
2:550000	YBR148W	YDR260C	-367.646	-356.344	-360.697
2:550000	YBR148W	YOR296W	-510.739	-500.705	-501.136
2:550000	YBR148W	YNL117W	489.869	498.378	496.347
2:550000	YBR148W	YER043C	-951.119	-940.843	-945.658
2:550000	YBR148W	YIL088C	-275.583	-271.318	-261.359
2:550000	YBR148W	YLR084C	-308.077	-298.531	-290.525
2:550000	YBR148W	YNL239W	-296.176	-289.968	-285.84
2:550000	YBR148W	YLR333C	-487.366	-478.715	-484.549
2:550000	YBR148W	YPL163C	-494.876	-486.305	-491.322
2:550000	YBR148W	YNL046W	-258.131	-247.187	-250.888
2:550000	YBR148W	YKL218C	784.361	786.825	793.151
2:550000	YBR148W	YNL096C	-84.3016	-73.0252	-72.8074
2:550000	YBR148W	YKL181W	124.153	135.43	136.914
2:550000	YBR148W	YKL006W	-512.877	-512.845	-510.928
2:550000	YBR148W	YFR031C-A	-178.062	-175.557	-177.141
2:550000	YBR148W	YGL209W	386.498	395.815	411.8
2:550000	YBR148W	YDL131W	329.037	338.537	332.435
2:550000	YBR148W	YCL014W	-441.679	-431.957	-435.567
2:550000	YBR148W	YDL082W	280.503	287	281.211

2:550000	YBR148W	YMR011W	638.84	645.066	639.174
2:550000	YBR148W	YML106W	-778.423	-767.256	-770.99
2:550000	YBR148W	YBL071C-B	-304.491	-293.875	-282.669
2:550000	YBR148W	YJR005W	-552.447	-544.19	-544.879
2:550000	YBR148W	YGR154C	436.398	445.042	443.064
2:550000	YBR148W	YLR432W	58.8191	70.2582	65.0874
2:550000	YBR148W	YER056C-A	-224.022	-218.44	-222.885
2:550000	YBR148W	YKL107W	414.837	425.737	423.191
2:550000	YBR148W	YDL014W	739.571	750.774	745.247
2:550000	YBR148W	YNL302C	-876.144	-872.617	-875.342
2:550000	YBR148W	YIR009W	-153.153	-141.715	-146.655
2:550000	YBR148W	YER001W	-81.9658	-78.1188	-74.1983
2:550000	YBR148W	YJR133W	-212.476	-209.131	-208.297
2:550000	YBR148W	YOL121C	85.2736	89.8215	86.0337
2:550000	YBR148W	YJR148W	-868.992	-859.126	-858.742
2:550000	YBR148W	YPR041W	-122.533	-113.286	-117.239
2:550000	YBR148W	YGL139W	-650.517	-645.333	-650.143
2:550000	YBR148W	YMR174C	468.833	475.784	478.283
2:550000	YBR148W	YOR111W	-165.377	-153.81	-138.028
2:550000	YBR148W	YBR256C	-331.464	-323.888	-329.841
2:550000	YBR148W	YGR067C	75.3607	80.9621	75.4991
2:550000	YBR148W	YHR010W	-517.311	-514.09	-513.697
2:550000	YBR148W	YLR285C-A	-51.036	-45.4828	-47.333
2:550000	YBR148W	YHR107C	-581.828	-579.996	-571.295
2:550000	YBR148W	YLR042C	132.004	138.789	137.147
2:550000	YBR148W	YMR173W	-537.667	-526.446	-531.169
2:550000	YBR148W	YER145C	-111.891	-100.78	-78.1947
2:550000	YBR148W	YPL213W	-515.243	-511.555	-508.852
2:550000	YBR148W	YNL040W	-23.9125	-14.3877	-17.4354
2:550000	YBR148W	YGR138C	296.612	297.727	298.12
2:550000	YBR148W	YGL081W	185.923	197.138	214.718
2:550000	YBR148W	YPR105C	-777.196	-766.166	-762.698
2:550000	YBR148W	YLR406C	327.929	336.852	330.934
2:550000	YBR148W	YNL078W	-454.64	-444.471	-450.587
2:550000	YBR148W	YHL016C	1067.17	1069.54	1075.18
2:550000	YBR148W	YBR117C	608.052	611.217	610.883
2:550000	YBR148W	YDR034W-B	337.822	346.669	343.521
2:550000	YBR148W	YEL004W	-548.848	-536.976	-543.128
2:550000	YBR148W	YPL089C	244.364	249.768	244.368
2:550000	YBR148W	YPR074C	-1206.41	-1199.49	-1180.5
2:550000	YBR148W	YDL179W	-185.305	-183.215	-169.518
2:550000	YBR148W	YGR085C	-1028.57	-1022.91	-1026.35
2:550000	YBR148W	YPL158C	-546.743	-535.98	-530.846
2:550000	YBR148W	YLR080W	311.013	317.726	316.837
2:550000	YBR148W	YBR111W-A	81.7732	92.252	100.805
2:550000	YBR148W	YLL045C	-678.459	-677.789	-672.224
2:550000	YBR148W	YER025W	-313.657	-301.845	-304.857

2:550000	YBR148W	YOR315W	542.529	544.033	542.737
2:550000	YBR148W	YOR316C	-650.609	-645.035	-630.357
2:550000	YBR148W	YLR340W	-944.813	-942.053	-941.745
2:550000	YBR148W	YGR014W	-522.548	-512.766	-514.886
2:550000	YBR148W	YDL078C	-895.288	-884.734	-876.041
2:550000	YBR148W	YOL052C	-332.995	-328.119	-329.987
2:550000	YBR148W	YJL160C	33.2346	43.2316	37.1567
2:550000	YBR148W	YJR096W	310.485	318.154	336.4
2:550000	YBR148W	YLR079W	-749.06	-737.49	-742.511
2:550000	YBR148W	YOR247W	-945.517	-940.202	-934.936
2:550000	YBR148W	YAL041W	-779.109	-767.025	-766.261
2:550000	YBR148W	YGR152C	-413.374	-403.139	-409.277
2:550000	YBR148W	YBR193C	-351.357	-342.771	-344.394
2:550000	YBR148W	YKL068W-A	400.913	405.387	421.563
2:550000	YBR148W	YDR333C	-523.614	-512.728	-507.609
2:550000	YBR148W	YOR134W	587.271	595.598	590.071
2:550000	YBR148W	YBR126C	301.791	312.91	306.874
2:550000	YBR148W	YHL023C	-596.768	-586.297	-584.467
2:550000	YBR148W	YJR094W-A	-22.488	-17.9177	-22.3532
2:550000	YBR148W	YDL075W	-627.947	-625.624	-624.639
2:550000	YBR148W	YBR092C	537.131	542.888	573.845
2:550000	YBR148W	YJL110C	46.8268	54.6092	48.4962
2:550000	YBR148W	YLR164W	303.887	304.833	322.029
2:550000	YBR148W	YNL168C	-165.36	-153.554	-156.253
2:550000	YBR148W	YPL071C	-248.948	-237.202	-238.278
2:550000	YBR148W	YMR285C	-872.527	-863.547	-867.377
2:550000	YBR148W	YBR119W	-202.216	-194.272	-197.374
2:550000	YBR148W	YHR099W	-281.302	-273.513	-278.409
2:550000	YBR148W	YJL136C	-840.078	-834.603	-837.87
2:550000	YBR148W	YOR116C	105.888	111.202	108.148
2:550000	YBR148W	YOL082W	74.4185	76.5013	99.3885
2:550000	YBR148W	YGR044C	-110.484	-98.1904	-96.6237
2:550000	YBR156C	YBR149W	-695.051	-691.394	-669.411
2:550000	YBR156C	YPL264C	743.308	747.41	749.648
2:550000	YBR156C	YBR285W	657.323	660.432	735.538
2:550000	YBR156C	YDR072C	163.821	168.161	168.763
2:550000	YBR156C	YOR264W	-661.188	-649.787	-651.252
2:550000	YBR156C	YGR174W-A	580.894	582.006	598.881
2:550000	YBR156C	YBR084C-A	-323.274	-321.571	-323.031
2:550000	YBR156C	YDL012C	-430.699	-429.804	-427.921
2:550000	YBR156C	YLR029C	-985.897	-975.369	-981.419
2:550000	YBR156C	YIL052C	-449.615	-441.642	-447.651
2:550000	YBR156C	YOR179C	-436.707	-429.204	-433.091
2:550000	YBR156C	YNL055C	-1218.94	-1215.42	-1204.67
2:550000	YBR156C	YNR067C	-599.658	-587.928	-593.391
2:550000	YBR156C	YML124C	-1276.29	-1269.13	-1246.29
2:550000	YBR156C	YIL009W	-499.895	-491.471	-497.548

2:550000	YBR156C	YHL001W	-181.344	-171.357	-169.59
2:550000	YBR156C	YDL218W	290.322	293.105	291.195
2:550000	YBR156C	YJR123W	-1004.65	-999.399	-1002.61
2:550000	YBR156C	YGR027C	-1034.34	-1026.8	-1031.58
2:550000	YBR156C	YGR258C	-674.557	-669.776	-658.868
2:550000	YBR156C	YDL155W	-395.31	-393.537	-383.682
2:550000	YBR156C	YIR034C	480.913	489.134	482.985
2:550000	YBR156C	YLR185W	-351.46	-342.765	-348.923
2:550000	YBR156C	YKL081W	-651.29	-645.277	-650.901
2:550000	YBR156C	YGR085C	-64.4346	-54.1634	-52.5588
2:550000	YBR156C	YAR014C	-517.812	-506.17	-333.618
2:550000	YBR156C	YDR345C	-134.381	-133.876	-132.611
2:550000	YBR156C	YPR163C	178.2	187.885	186.768
2:550000	YBR156C	YDR234W	503.934	507.756	568.853
2:550000	YBR156C	YKL112W	-566.265	-561.961	-565.829
2:550000	YBR156C	YIR030C	884.379	895.603	930.47
2:550000	YBR156C	YLR249W	-345.57	-339.923	-344.786
2:550000	YBR156C	YPL066W	-813.53	-804.439	-806.418
2:550000	YBR156C	YER036C	63.5062	70.9925	65.2264
2:550000	YBR156C	YOR246C	-1035.06	-1028.51	-967.012
2:550000	YBR156C	YBR079C	239.898	246.847	249.575
2:550000	YBR156C	YGR208W	-635.101	-626.302	-548.723
2:550000	YBR156C	YGL189C	-952.514	-948.949	-947.822
2:550000	YBR156C	YPR043W	-1153.18	-1144.22	-1150.34
2:550000	YBR156C	YER089C	-808.023	-801.471	-780.376
2:550000	YBR156C	YOL127W	-1111.64	-1105.6	-1110.48
2:550000	YBR156C	YDL182W	201.999	203.955	214.159
2:550000	YBR156C	YBR154C	271.291	272.205	287.62
2:550000	YBR156C	YEL054C	345.761	352.325	346.166
2:550000	YBR156C	YGL062W	-980.826	-976.311	-974.307
2:550000	YBR156C	YHR209W	296.823	301.132	297.926
2:550000	YBR156C	YMR170C	359.77	365.398	374.891
2:550000	YBR156C	YJR138W	-329.684	-326.616	-324.643
2:550000	YBR156C	YMR251W-A	-972.508	-964.782	-925.371
2:550000	YBR156C	YML063W	-669.774	-659.892	-665.026
2:550000	YBR156C	YLL039C	-642.638	-635.631	-628.626
2:550000	YBR156C	YML118W	84.535	91.7482	95.2718
2:550000	YBR156C	YAL023C	-951.033	-946.339	-949.807
2:550000	YBR156C	YKR094C	-478.718	-472.509	-478.647
2:550000	YBR156C	YAL062W	574.598	576.348	584.957
2:550000	YBR156C	YLR344W	-135.639	-125.935	-128.418
2:550000	YBR156C	YDR222W	305.841	308.076	306.099
2:550000	YBR156C	YHR089C	785.574	792.089	787.127
2:550000	YBR156C	YNL015W	-247.829	-242.794	-151.047
2:550000	YBR156C	YBL072C	-808.693	-806.173	-795.525
2:550000	YBR156C	YMR205C	-1098.58	-1090.3	-1083.84
2:550000	YBR156C	YOL019W	-228.691	-220.124	-226.242

2:550000	YBR156C	YGR281W	-422.381	-421.408	-296.81
2:550000	YBR156C	YIL018W	-224.197	-221.697	-223.809
2:550000	YBR156C	YOR126C	-581.861	-576.957	-549.052
2:550000	YBR156C	YGR034W	-235.163	-230.435	-234.829
2:550000	YBR156C	YJL076W	-697.998	-690.854	-681.341
2:550000	YBR156C	YER063W	-569.386	-559.165	-559.018
2:550000	YBR156C	YDR471W	194.455	203.124	199.782
2:550000	YBR156C	YOR205C	-87.9815	-86.551	-46.9023
2:550000	YBR156C	YNL283C	-567.389	-564.782	-541.952
2:550000	YBR156C	YEL060C	-482.793	-479.622	-408.696
2:550000	YBR156C	YLR446W	249.397	255.806	282.399
2:550000	YBR156C	YDL024C	68.1469	78.4171	81.0755
2:550000	YBR156C	YOL131W	203.711	214.378	229.299
2:550000	YBR156C	YLR353W	-636.585	-624.537	-612.339
2:550000	YBR156C	YJL145W	-567.651	-564.202	-507.41
2:550000	YBR156C	YGR019W	59.6893	68.3997	118.016
2:550000	YBR156C	YNL268W	-903.169	-894.277	-883.683
2:550000	YBR156C	YDR098C	-1109.2	-1097.46	-1074.74
2:550000	YBR156C	YIL047C	-230.486	-224.782	-72.6364
2:550000	YBR156C	YJR001W	-970.743	-963.85	-932.78
2:550000	YBR156C	YMR271C	739.292	746.098	740.167
2:550000	YBR156C	YLR448W	-124.609	-117.4	-121.78
2:550000	YBR156C	YJL161W	639.065	641.494	682.77
2:550000	YBR156C	YOL039W	-754.763	-744.335	-749.792
2:550000	YBR156C	YHR117W	-751.64	-739.584	-741.06
2:550000	YBR156C	YHR012W	-720.184	-714.302	-689.432
2:550000	YBR156C	YLR421C	-919.055	-909.123	-880.74
2:550000	YBR156C	YBR263W	-883.212	-871.281	-848.232
2:550000	YBR156C	YJL159W	-1105.33	-1093.3	-1097.94
2:550000	YBR156C	YJL045W	75.7188	86.9922	101.188
2:550000	YBR156C	YJL116C	424.525	435.327	461.231
2:550000	YBR156C	YPR144C	470.435	470.855	483.644
2:550000	YBR156C	YBR115C	-25.7963	-14.1518	-19.7675
2:550000	YBR156C	YNL282W	61.2165	67.8531	189.764
2:550000	YBR156C	YGR040W	-380.833	-375.159	-353.425
2:550000	YBR156C	YJL191W	124.631	136.045	140.7
2:550000	YBR156C	YIL089W	126.213	134.482	152.791
2:550000	YBR156C	YPL171C	88.0156	95.4536	103.358
2:550000	YBR156C	YDR260C	-368.148	-360.607	-365.698
2:550000	YBR156C	YIL124W	-713.632	-706.475	-660.237
2:550000	YBR156C	YNR068C	338.724	338.752	340.388
2:550000	YBR156C	YER043C	-967.868	-960.138	-952.049
2:550000	YBR156C	YIL088C	-270.043	-263.835	-267.676
2:550000	YBR156C	YLR084C	-385.808	-376.392	-292.888
2:550000	YBR156C	YNL239W	-295.004	-286.924	-291.23
2:550000	YBR156C	YDR226W	-1544.47	-1539.42	-1542.12
2:550000	YBR156C	YLR333C	-494.968	-486.762	-492.61

2:550000	YBR156C	YPL163C	-509.921	-499.87	-494.798
2:550000	YBR156C	YNL096C	-76.1456	-67.8055	-71.6528
2:550000	YBR156C	YKL181W	133.972	139.726	136.982
2:550000	YBR156C	YKL006W	-516.606	-509.46	-514.989
2:550000	YBR156C	YFR031C-A	-181.917	-175.727	-181.594
2:550000	YBR156C	YMR181C	46.529	54.7661	67.3455
2:550000	YBR156C	YGL209W	335.438	347.208	417.567
2:550000	YBR156C	YDL131W	333.469	336.19	335.304
2:550000	YBR156C	YCL014W	-455.448	-448.388	-444.487
2:550000	YBR156C	YML106W	-798.522	-788.859	-773.04
2:550000	YBR156C	YBL071C-B	-354.343	-342.322	-284.906
2:550000	YBR156C	YJR005W	-581.677	-570.638	-548.472
2:550000	YBR156C	YFL021W	518.387	529.03	580.622
2:550000	YBR156C	YLR432W	58.7453	67.0141	63.7306
2:550000	YBR156C	YER056C-A	-225.832	-220.096	-225.595
2:550000	YBR156C	YKL107W	426.43	433.97	429.446
2:550000	YBR156C	YDL014W	750.694	757.552	752.472
2:550000	YBR156C	YER001W	-88.6576	-84.6093	-76.8883
2:550000	YBR156C	YJR133W	-215.38	-207.787	-212.095
2:550000	YBR156C	YBR148W	99.3169	102.881	100.907
2:550000	YBR156C	YOL121C	87.3165	93.4459	87.4709
2:550000	YBR156C	YER118C	-457.273	-453.959	-447.758
2:550000	YBR156C	YDL156W	-298.544	-297.448	-295.932
2:550000	YBR156C	YEL072W	-278.555	-270.879	-232.659
2:550000	YBR156C	YMR174C	465.594	475.648	489.789
2:550000	YBR156C	YNL069C	-675.737	-670.036	-674.877
2:550000	YBR156C	YOR111W	-145.366	-141.799	-140.938
2:550000	YBR156C	YGR067C	73.7345	80.6808	76.3658
2:550000	YBR156C	YHR010W	-522.366	-513.361	-519.145
2:550000	YBR156C	YLR285C-A	-51.6132	-43.0844	-48.4532
2:550000	YBR156C	YHR107C	-588.134	-579.715	-575.378
2:550000	YBR156C	YMR129W	-738.618	-735.294	-713.978
2:550000	YBR156C	YMR173W	-550.894	-541.713	-542.447
2:550000	YBR156C	YER074W	-646.014	-638.686	-639.671
2:550000	YBR156C	YER145C	-101.394	-94.8796	-78.0858
2:550000	YBR156C	YCR086W	-122.296	-118.167	-88.0681
2:550000	YBR156C	YNL040W	-23.662	-12.9948	-18.6792
2:550000	YBR156C	YKL179C	-778.949	-775.786	-770.827
2:550000	YBR156C	YGL081W	120.566	132.289	212.465
2:550000	YBR156C	YPR105C	-779.764	-772.952	-771.537
2:550000	YBR156C	YOR096W	-479.322	-471.596	-476.329
2:550000	YBR156C	YLR406C	332.854	341.502	339.245
2:550000	YBR156C	YNL078W	-466.325	-454.793	-454.627
2:550000	YBR156C	YGL076C	-1191.82	-1185.19	-1188.88
2:550000	YBR156C	YCL025C	-401.388	-396.682	-344.652
2:550000	YBR156C	YBR117C	598.285	603.007	631.146
2:550000	YBR156C	YDR034W-B	322.307	329.382	357.328

2:550000	YBR156C	YLR378C	-879.243	-877.463	-872.369
2:550000	YBR156C	YEL004W	-556.478	-546.423	-549.038
2:550000	YBR156C	YOL007C	-199.893	-195.71	-167.243
2:550000	YBR156C	YPR074C	-1256.59	-1244.51	-1193.24
2:550000	YBR156C	YDL179W	-190.502	-180.494	-171.438
2:550000	YBR156C	YGR085C	-1042.34	-1034.2	-1039.9
2:550000	YBR156C	YPL158C	-562.025	-549.911	-534.017
2:550000	YBR156C	YLR080W	305.821	307.457	316.299
2:550000	YBR156C	YBR111W-A	97.311	106.653	103.661
2:550000	YBR156C	YLL045C	-685.414	-676.574	-681.799
2:550000	YBR156C	YER025W	-318.749	-311.824	-308.302
2:550000	YBR156C	YOR316C	-706.564	-697.886	-641.811
2:550000	YBR156C	YLR340W	-955.563	-949.189	-953.094
2:550000	YBR156C	YGR014W	-530.54	-525.529	-519.494
2:550000	YBR156C	YDL078C	-925.103	-915.899	-887.38
2:550000	YBR156C	YOL040C	-733.233	-729.416	-731.361
2:550000	YBR156C	YJR096W	240.627	252.582	352.985
2:550000	YBR156C	YMR013C	-416.79	-412.413	-374.178
2:550000	YBR156C	YFR028C	-667.216	-657.514	-629.695
2:550000	YBR156C	YLR079W	-767.219	-755.33	-751.294
2:550000	YBR156C	YOR247W	-932.247	-925.846	-931.071
2:550000	YBR156C	YLR258W	740.796	749.373	822.971
2:550000	YBR156C	YAL041W	-796.832	-787.419	-771.116
2:550000	YBR156C	YGR152C	-417.174	-409.051	-413.994
2:550000	YBR156C	YBR048W	-781.847	-778.376	-778.754
2:550000	YBR156C	YGL135W	-1288.83	-1280.39	-1285.95
2:550000	YBR156C	YDR333C	-527.211	-523.465	-522.089
2:550000	YBR156C	YOR134W	595.58	601.249	595.794
2:550000	YBR156C	YAL024C	-452.55	-447.899	-395.39
2:550000	YBR156C	YBR126C	249.456	254.572	313.305
2:550000	YBR156C	YDL075W	-639.176	-630.536	-636.063
2:550000	YBR156C	YBR092C	557.422	564.109	580.529
2:550000	YBR156C	YLR164W	258.655	264.712	325.737
2:550000	YBR156C	YNL168C	-164.768	-156.624	-161.825
2:550000	YBR156C	YHR021C	-486.404	-481.096	-485.961
2:550000	YBR156C	YMR285C	-895.623	-886.214	-881.292
2:550000	YBR156C	YBR119W	-207.083	-196.912	-202.329
2:550000	YBR156C	YJL136C	-848.578	-840.275	-846.426
2:550000	YBR156C	YGR044C	-121.883	-114.429	-108.854
2:550000	YBR150C	YER059W	-470.68	-459.483	-358.67
2:550000	YBR150C	YFL034C-B	-340.404	-337.65	-331.696
2:550000	YBR150C	YIR021W	-48.9284	-39.2137	-32.858
2:550000	YBR150C	YBR149W	-676.951	-670.293	-669.411
2:550000	YBR150C	YMR182C	68.409	78.4285	94.672
2:550000	YBR150C	YKL166C	-201.646	-201.061	-142.623
2:550000	YBR150C	YBR157C	636.306	642.609	658.979
2:550000	YBR150C	YPL101W	-607.217	-601.687	-590.176

2:550000	YBR150C	YOR264W	-658.424	-646.744	-651.252
2:550000	YBR150C	YDR006C	-806.343	-796.899	-761.499
2:550000	YBR150C	YLR029C	-985.707	-979.606	-981.419
2:550000	YBR150C	YIL052C	-450.671	-449.349	-447.651
2:550000	YBR150C	YNL055C	-1209.37	-1200.43	-1204.67
2:550000	YBR150C	YNR067C	-609.363	-598.221	-593.391
2:550000	YBR150C	YML124C	-1262.06	-1254.98	-1246.29
2:550000	YBR150C	YIL009W	-512.884	-501.029	-497.548
2:550000	YBR150C	YJR123W	-1004.44	-999.458	-1002.61
2:550000	YBR150C	YGR027C	-1034.33	-1026.99	-1031.58
2:550000	YBR150C	YGR258C	-684.498	-675.307	-658.868
2:550000	YBR150C	YOR045W	-704.119	-694.399	-690.923
2:550000	YBR150C	YNR020C	23.0035	29.8826	28.7324
2:550000	YBR150C	YLR185W	-351.471	-348.338	-348.923
2:550000	YBR150C	YPL081W	564.757	575.378	592.137
2:550000	YBR150C	YNR017W	-268.816	-266.378	-242.084
2:550000	YBR150C	YOL112W	-495.804	-489.692	-495.8
2:550000	YBR150C	YJL125C	-137.462	-135.917	-100.381
2:550000	YBR150C	YAR014C	-390.001	-377.715	-333.618
2:550000	YBR150C	YBR135W	-650.943	-648.253	-642.603
2:550000	YBR150C	YDR234W	564.332	574.857	568.853
2:550000	YBR150C	YIR030C	915.584	923.849	930.47
2:550000	YBR150C	YPR125W	-418.759	-410.712	-402.078
2:550000	YBR150C	YLR249W	-345.609	-341.351	-344.786
2:550000	YBR150C	YPL066W	-808.869	-800.255	-806.418
2:550000	YBR150C	YPR005C	136.319	144.485	201.829
2:550000	YBR150C	YOR246C	-1012.83	-1008.38	-967.012
2:550000	YBR150C	YGR208W	-660.812	-660.613	-548.723
2:550000	YBR150C	YGL189C	-951.621	-949.764	-947.822
2:550000	YBR150C	YPR043W	-1152.06	-1145.77	-1150.34
2:550000	YBR150C	YER089C	-801.74	-793.624	-780.376
2:550000	YBR150C	YOL127W	-1111.72	-1107.19	-1110.48
2:550000	YBR150C	YMR309C	68.999	69.7853	71.5108
2:550000	YBR150C	YFR002W	-878.46	-869.25	-823.427
2:550000	YBR150C	YBR154C	251.592	259.149	287.62
2:550000	YBR150C	YDL230W	-613.107	-602.778	-522.305
2:550000	YBR150C	YGL062W	-976.936	-968.149	-974.307
2:550000	YBR150C	YGL185C	-579.296	-574.936	-574.557
2:550000	YBR150C	YMR251W-A	-979.687	-971.927	-925.371
2:550000	YBR150C	YGL229C	63.0139	73.4631	144.155
2:550000	YBR150C	YML063W	-673.335	-667.811	-665.026
2:550000	YBR150C	YLL039C	-688.885	-677.551	-628.626
2:550000	YBR150C	YML118W	92.0348	97.2794	95.2718
2:550000	YBR150C	YAL023C	-951.454	-944.029	-949.807
2:550000	YBR150C	YNL015W	-199.991	-191.154	-151.047
2:550000	YBR150C	YJR008W	7.05114	13.7013	111.605
2:550000	YBR150C	YBL072C	-807.673	-803.602	-795.525

2:550000	YBR150C	YCR099C	244.142	250.963	245.59
2:550000	YBR150C	YMR205C	-1084.66	-1079.6	-1083.84
2:550000	YBR150C	YOL019W	-232.558	-225.203	-226.242
2:550000	YBR150C	YGR281W	-356.581	-355.159	-296.81
2:550000	YBR150C	YIL018W	-224.701	-222.921	-223.809
2:550000	YBR150C	YER095W	-563.704	-559.311	-556.439
2:550000	YBR150C	YNL293W	-283.935	-281.49	-279.816
2:550000	YBR150C	YDR514C	-45.1728	-36.2802	76.312
2:550000	YBR150C	YOR205C	-56.418	-54.6403	-46.9023
2:550000	YBR150C	YNL283C	-552.232	-543.53	-541.952
2:550000	YBR150C	YEL060C	-501.444	-491.487	-408.696
2:550000	YBR150C	YLR446W	170.496	178.636	282.399
2:550000	YBR150C	YBR091C	-33.0536	-22.879	11.0925
2:550000	YBR150C	YLR353W	-650.475	-639.076	-612.339
2:550000	YBR150C	YNL268W	-893.394	-882.957	-883.683
2:550000	YBR150C	YDR098C	-1096.26	-1084.39	-1074.74
2:550000	YBR150C	YIL047C	-141.697	-133.983	-72.6364
2:550000	YBR150C	YJR001W	-981.425	-976.696	-932.78
2:550000	YBR150C	YDL142C	-590.418	-579.974	-496.195
2:550000	YBR150C	YOL039W	-754.032	-748.918	-749.792
2:550000	YBR150C	YER019W	-688.693	-686.495	-648.801
2:550000	YBR150C	YHR117W	-749.845	-737.892	-741.06
2:550000	YBR150C	YGR286C	-167.06	-159.843	-152.301
2:550000	YBR150C	YLR421C	-881.224	-875.056	-880.74
2:550000	YBR150C	YLL009C	424.546	430.778	427.34
2:550000	YBR150C	YJL159W	-1109.79	-1103.1	-1097.94
2:550000	YBR150C	YJL045W	95.6749	103.791	101.188
2:550000	YBR150C	YLR102C	-503.056	-502.565	-401.714
2:550000	YBR150C	YHL038C	-458.785	-449.811	-451.504
2:550000	YBR150C	YPL183C	166.8	173.249	220.219
2:550000	YBR150C	YJL116C	422.141	433.665	461.231
2:550000	YBR150C	YBR115C	-31.2318	-19.5274	-19.7675
2:550000	YBR150C	YNL282W	159.218	164.563	189.764
2:550000	YBR150C	YLR188W	-842.453	-834.155	-826.552
2:550000	YBR150C	YLR209C	-985.117	-982.73	-979.716
2:550000	YBR150C	YPR075C	-543.699	-536.215	-536.551
2:550000	YBR150C	YHR007C	-816.855	-811.584	-802.765
2:550000	YBR150C	YBR107C	-451.537	-449.494	-449.743
2:550000	YBR150C	YGR169C	-292.191	-284.575	-282.734
2:550000	YBR150C	YIL124W	-743.625	-736.678	-660.237
2:550000	YBR150C	YDR194C	-239.099	-228.942	-212.262
2:550000	YBR150C	YPR124W	-219.087	-212.035	-213.578
2:550000	YBR150C	YDL122W	-395.207	-391.55	-327.007
2:550000	YBR150C	YML031W	-766.891	-765.363	-761.168
2:550000	YBR150C	YLR084C	-384.829	-374.802	-292.888
2:550000	YBR150C	YDR226W	-1550.93	-1542.95	-1542.12
2:550000	YBR150C	YPL163C	-503.676	-494.328	-494.798

2:550000	YBR150C	YJR036C	25.976	29.8462	89.2493
2:550000	YBR150C	YIL158W	-232.77	-222.182	-161.395
2:550000	YBR150C	YPR114W	-713.489	-703.745	-692.599
2:550000	YBR150C	YKL006W	-517.1	-513.198	-514.989
2:550000	YBR150C	YMR181C	-4.16762	0.00102396	67.3455
2:550000	YBR150C	YGL209W	390.729	401.089	417.567
2:550000	YBR150C	YCL014W	-466.955	-464.918	-444.487
2:550000	YBR150C	YBL071C-B	-343.876	-332.676	-284.906
2:550000	YBR150C	YER075C	74.9747	83.8598	188.481
2:550000	YBR150C	YFL021W	503.969	510.657	580.622
2:550000	YBR150C	YLR419W	-297.651	-291.138	-232.234
2:550000	YBR150C	YOR286W	-352.08	-348.621	-351.249
2:550000	YBR150C	YGL064C	-32.6036	-29.6079	-10.2632
2:550000	YBR150C	YOR034C-A	-622.139	-617.162	-587.235
2:550000	YBR150C	YDR294C	-1002.56	-994.525	-982.789
2:550000	YBR150C	YBR148W	83.5666	91.1772	100.907
2:550000	YBR150C	YGL123W	-931.703	-925.063	-931.217
2:550000	YBR150C	YER118C	-456.429	-452.298	-447.758
2:550000	YBR150C	YDL156W	-296.088	-292.07	-295.932
2:550000	YBR150C	YNL069C	-677.129	-675.431	-674.877
2:550000	YBR150C	YNL278W	-29.7674	-28.138	-25.5296
2:550000	YBR150C	YHR010W	-526.409	-524.294	-519.145
2:550000	YBR150C	YLR285C-A	-53.8125	-43.5366	-48.4532
2:550000	YBR150C	YHR107C	-595.196	-589.815	-575.378
2:550000	YBR150C	YMR173W	-565.483	-554.002	-542.447
2:550000	YBR150C	YER074W	-645.19	-637.415	-639.671
2:550000	YBR150C	YJR043C	-560.846	-552.575	-549.048
2:550000	YBR150C	YER145C	-81.6224	-78.0166	-78.0858
2:550000	YBR150C	YCR086W	-141.669	-139.672	-88.0681
2:550000	YBR150C	YCR046C	-75.0821	-70.6006	-72.9792
2:550000	YBR150C	YKL179C	-786.391	-785.947	-770.827
2:550000	YBR150C	YGL081W	142.526	154.647	212.465
2:550000	YBR150C	YPR105C	-798.483	-788.384	-771.537
2:550000	YBR150C	YDR421W	-347.259	-341.506	-328.546
2:550000	YBR150C	YOR096W	-483.368	-481.92	-476.329
2:550000	YBR150C	YNL078W	-475.144	-466.025	-454.627
2:550000	YBR150C	YOR380W	-643.946	-635.967	-619.039
2:550000	YBR150C	YCL025C	-397.883	-388.268	-344.652
2:550000	YBR150C	YBR117C	629.881	635.59	631.146
2:550000	YBR150C	YDR034W-B	332.501	340.742	357.328
2:550000	YBR150C	YEL004W	-558.762	-550.048	-549.038
2:550000	YBR150C	YPR074C	-1222.6	-1210.44	-1193.24
2:550000	YBR150C	YDL179W	-176.287	-165.942	-171.438
2:550000	YBR150C	YGR085C	-1042.77	-1036.74	-1039.9
2:550000	YBR150C	YIL098C	385.099	389.102	391.855
2:550000	YBR150C	YBR111W-A	95.9408	106.539	103.661
2:550000	YBR150C	YLL045C	-685.411	-676.584	-681.799

2:550000	YBR150C	YLR340W	-955.438	-948.782	-953.094
2:550000	YBR150C	YOL040C	-732.819	-728.67	-731.361
2:550000	YBR150C	YJR096W	293.985	304.799	352.985
2:550000	YBR150C	YMR013C	-382.505	-377.354	-374.178
2:550000	YBR150C	YJR101W	-70.6056	-63.2671	-68.7677
2:550000	YBR150C	YLR079W	-795.174	-783.77	-751.294
2:550000	YBR150C	YFL001W	-299.08	-295.59	-264.303
2:550000	YBR150C	YOR247W	-940.221	-931.791	-931.071
2:550000	YBR150C	YPR194C	1144.65	1150.47	1384.24
2:550000	YBR150C	YLR258W	672.116	683.414	822.971
2:550000	YBR150C	YAL041W	-776.445	-767.656	-771.116
2:550000	YBR150C	YGR152C	-417.447	-409.412	-413.994
2:550000	YBR150C	YBR048W	-781.448	-778.406	-778.754
2:550000	YBR150C	YGL135W	-1290.38	-1286.6	-1285.95
2:550000	YBR150C	YJL054W	-811.516	-802.881	-792.357
2:550000	YBR150C	YAL024C	-434.256	-425.261	-395.39
2:550000	YBR150C	YDL075W	-639.668	-632.835	-636.063
2:550000	YBR150C	YPL063W	-1020.39	-1012.82	-1014.78
2:550000	YBR150C	YPL123C	-88.0971	-88.053	-52.4532
2:550000	YBR150C	YHR021C	-487.234	-486.164	-485.961
2:550000	YBR150C	YNL299W	407.078	412.011	521.045
2:550000	YBR150C	YKR091W	-290.431	-283.82	-270.153
2:550000	YBR150C	YBL092W	-920.459	-916.57	-914.529
2:550000	YBR150C	YBR119W	-214.632	-202.445	-202.329
2:550000	YBR150C	YJL136C	-847.963	-844.257	-846.426
2:550000	YBR150C	YJL053W	-688.63	-680.371	-642.303
2:550000	YBR150C	YPL082C	-331.594	-331.417	-266.948
2:550000	YBR150C	YGR044C	-115.163	-109.028	-108.854
2:550000	YBR158W	YER059W	-357.748	-352.667	-326.644
2:550000	YBR158W	YJL147C	-181.556	-174.79	-180.378
2:550000	YBR158W	YFL034C-B	-317.132	-309.855	-315.56
2:550000	YBR158W	YIR021W	-37.8097	-26.8125	-23.8927
2:550000	YBR158W	YBR149W	-654.163	-643.636	-649.714
2:550000	YBR158W	YLR286C	-423.799	-411.952	-383.034
2:550000	YBR158W	YMR182C	74.6504	78.0447	96.2704
2:550000	YBR158W	YKL166C	-143.121	-141.874	-141.602
2:550000	YBR158W	YBR194W	-525.017	-516.152	-512.225
2:550000	YBR158W	YOR187W	-383.012	-381.295	-380.97
2:550000	YBR158W	YPL101W	-587.5	-576.461	-568.816
2:550000	YBR158W	YOR264W	-651.446	-639.193	-645.191
2:550000	YBR158W	YHR053C	-152.549	-141.853	-140.086
2:550000	YBR158W	YNL055C	-1191.63	-1185.37	-1182.96
2:550000	YBR158W	YJL200C	758.669	765.261	764.984
2:550000	YBR158W	YNR067C	-595.606	-585.687	-578.723
2:550000	YBR158W	YDL055C	-1119	-1109.12	-1115.13
2:550000	YBR158W	YOL021C	-605.061	-596.196	-598.111
2:550000	YBR158W	YIL009W	-496.181	-483.982	-489.111

2:550000	YBR158W	YGR258C	-638.567	-630.38	-636.425
2:550000	YBR158W	YOR045W	-697.371	-689.963	-673.735
2:550000	YBR158W	YEL062W	-615.087	-607.767	-607.864
2:550000	YBR158W	YNR020C	23.8664	35.4426	31.5115
2:550000	YBR158W	YPL081W	571.982	582.672	588.266
2:550000	YBR158W	YOL112W	-494.583	-482.701	-486.758
2:550000	YBR158W	YBL072C	-1279.78	-1278.59	-1276.31
2:550000	YBR158W	YJL125C	-95.6243	-85.4501	-80.699
2:550000	YBR158W	YAR014C	-343.433	-331.931	-333.643
2:550000	YBR158W	YER124C	531.004	541.416	604.764
2:550000	YBR158W	YDR090C	67.6074	78.3364	83.0523
2:550000	YBR158W	YBR077C	-798.399	-796.548	-785.351
2:550000	YBR158W	YJL063C	109.458	118.184	112.517
2:550000	YBR158W	YDR234W	554.126	557.457	558.982
2:550000	YBR158W	YNL073W	-263.731	-256.493	-262.56
2:550000	YBR158W	YIR030C	913.14	921.736	916.434
2:550000	YBR158W	YGL068W	239.306	246.849	240.707
2:550000	YBR158W	YBR162C	-732.68	-725.899	-721.203
2:550000	YBR158W	YLR360W	-159.656	-150.362	-141.618
2:550000	YBR158W	YPR125W	-392.847	-382.012	-386.468
2:550000	YBR158W	YPL066W	-798.583	-791.686	-795.831
2:550000	YBR158W	YGR208W	-545.371	-538.757	-532.277
2:550000	YBR158W	YMR225C	28.9766	37.993	41.3101
2:550000	YBR158W	YER089C	-778.711	-774.422	-766.217
2:550000	YBR158W	YNL121C	-792.733	-786.794	-788.175
2:550000	YBR158W	YDR237W	220.09	227.022	221.36
2:550000	YBR158W	YFR002W	-803.209	-796.624	-802.294
2:550000	YBR158W	YDL230W	-539.37	-528.438	-513.663
2:550000	YBR158W	YGR084C	90.5526	100.102	93.9668
2:550000	YBR158W	YNR037C	60.641	72.3235	76.0776
2:550000	YBR158W	YIL070C	518.335	529.284	523.868
2:550000	YBR158W	YKL185W	-548.399	-541.048	-544.4
2:550000	YBR158W	YNL101W	-349.833	-343.928	-348.895
2:550000	YBR158W	YML030W	-118.752	-107.523	-113.499
2:550000	YBR158W	YGL229C	126.07	136.341	146.282
2:550000	YBR158W	YLL039C	-604.33	-598.408	-603.22
2:550000	YBR158W	YPL128C	-657.482	-646.532	-651.959
2:550000	YBR158W	YKL164C	-956.121	-944.69	-946.494
2:550000	YBR158W	YOR056C	253.501	260.219	254.519
2:550000	YBR158W	YJR008W	125.454	131.076	126.658
2:550000	YBR158W	YOR316C-A	-379.964	-368.384	-349.787
2:550000	YBR158W	YDR430C	92.0554	98.5226	92.6882
2:550000	YBR158W	YGR281W	-291.751	-285.324	-291.311
2:550000	YBR158W	YER095W	-549.074	-546.765	-548.405
2:550000	YBR158W	YFL041W	-826.286	-821.702	-825.852
2:550000	YBR158W	YEL064C	-466.278	-458.093	-438.488
2:550000	YBR158W	YDR514C	85.2563	95.0859	94.8714

2:550000	YBR158W	YOR205C	-78.3242	-69.949	-38.8073
2:550000	YBR158W	YNL283C	-544.958	-535.04	-537.344
2:550000	YBR158W	YBR146W	-64.504	-57.9842	-60.2152
2:550000	YBR158W	YKR065C	-597.659	-595.687	-574.729
2:550000	YBR158W	YLR446W	259.344	268.685	268.262
2:550000	YBR158W	YNR036C	-423.94	-411.934	-414.294
2:550000	YBR158W	YGR076C	66.9315	76.0861	69.9487
2:550000	YBR158W	YBR091C	-0.736467	11.1009	13.1087
2:550000	YBR158W	YLR353W	-614.154	-611.928	-596.521
2:550000	YBR158W	YJL095W	-691.36	-687.338	-690.651
2:550000	YBR158W	YNL268W	-871.876	-862.664	-867.92
2:550000	YBR158W	YJR001W	-913.08	-907.132	-909.455
2:550000	YBR158W	YDL142C	-496.203	-488.422	-471.442
2:550000	YBR158W	YMR305C	-336.693	-330.133	-336.195
2:550000	YBR158W	YHR117W	-730.108	-720.661	-726.737
2:550000	YBR158W	YBR171W	-1077.29	-1068.05	-1073.11
2:550000	YBR158W	YIL093C	462.391	469.401	463.664
2:550000	YBR158W	YLR421C	-859.639	-853.139	-858.046
2:550000	YBR158W	YLL009C	410.586	420.09	422.769
2:550000	YBR158W	YCR071C	305.098	316.602	311.352
2:550000	YBR158W	YHL038C	-443.552	-433.746	-438.626
2:550000	YBR158W	YPL183C	232.894	243.396	239.948
2:550000	YBR158W	YJL116C	433.333	440.224	453.35
2:550000	YBR158W	YDL150W	340.343	349.321	344.215
2:550000	YBR158W	YBR115C	-24.9214	-19.2386	-11.9144
2:550000	YBR158W	YGR040W	-346.93	-336.036	-339.662
2:550000	YBR158W	YER153C	-338.217	-330.234	-335.049
2:550000	YBR158W	YIL089W	162.028	170.304	164.162
2:550000	YBR158W	YDL045W-A	-111.874	-100.131	-104.647
2:550000	YBR158W	YDL202W	202.507	211.071	204.973
2:550000	YBR158W	YMR255W	-648.336	-642.272	-647.964
2:550000	YBR158W	YLR209C	-972.081	-969.13	-969.684
2:550000	YBR158W	YPR075C	-529.911	-524.585	-521.536
2:550000	YBR158W	YHR007C	-793.469	-783.435	-787.939
2:550000	YBR158W	YOR296W	-498.263	-489.859	-495.997
2:550000	YBR158W	YGR169C	-278.749	-272.401	-268.667
2:550000	YBR158W	YGR220C	-136.544	-126.505	-132.336
2:550000	YBR158W	YLR059C	-612.33	-601.46	-604.18
2:550000	YBR158W	YIL124W	-644.693	-640.343	-637.506
2:550000	YBR158W	YLR142W	828.818	838.47	848.555
2:550000	YBR158W	YDR194C	-221.843	-210.995	-202.033
2:550000	YBR158W	YPR124W	-218.291	-206.779	-210.308
2:550000	YBR158W	YDL122W	-298.543	-293.042	-295.589
2:550000	YBR158W	YLR084C	-295.728	-285.802	-279.386
2:550000	YBR158W	YMR002W	-274.93	-268.967	-273.405
2:550000	YBR158W	YDR226W	-1514.24	-1504.92	-1509.92
2:550000	YBR158W	YJR036C	89.3059	95.2338	93.4096

2:550000	YBR158W	YIL158W	-169.393	-157.454	-146.657
2:550000	YBR158W	YKR006C	28.9209	35.2921	34.0981
2:550000	YBR158W	YPR114W	-679.295	-668.43	-663.976
2:550000	YBR158W	YMR181C	61.2717	68.5865	63.5581
2:550000	YBR158W	YGL209W	403.276	413.88	413.289
2:550000	YBR158W	YCL014W	-435.579	-435.341	-428.354
2:550000	YBR158W	YDR322W	272.018	283.162	278.101
2:550000	YBR158W	YBL071C-B	-275.824	-267.85	-272.924
2:550000	YBR158W	YER075C	140.54	152.065	171.96
2:550000	YBR158W	YGL028C	132.214	139.407	234.712
2:550000	YBR158W	YFL021W	552.626	552.95	575.065
2:550000	YBR158W	YLR419W	-213.301	-207.969	-202.287
2:550000	YBR158W	YOR140W	-144.918	-136.898	-137.122
2:550000	YBR158W	YPL104W	-119.925	-112.778	-111.161
2:550000	YBR158W	YGL064C	-25.6637	-16.7482	5.10943
2:550000	YBR158W	YOR034C-A	-577.949	-565.956	-558.096
2:550000	YBR158W	YDR294C	-967.368	-957.884	-959.312
2:550000	YBR158W	YJR080C	32.8632	40.0998	35.1776
2:550000	YBR158W	YGR174C	335.433	344.671	339.667
2:550000	YBR158W	YNL137C	-100.47	-95.8341	-100.328
2:550000	YBR158W	YLR285C-A	-56.0014	-48.4151	-51.5384
2:550000	YBR158W	YLR042C	128.818	139.057	132.989
2:550000	YBR158W	YMR129W	-688.323	-681.646	-687.721
2:550000	YBR158W	YER074W	-630.308	-624.101	-624.639
2:550000	YBR158W	YJR043C	-531.976	-530.228	-531.152
2:550000	YBR158W	YER145C	-76.5534	-66.6199	-71.5026
2:550000	YBR158W	YCR046C	-82.6152	-73.0451	-62.9153
2:550000	YBR158W	YPL213W	-489.086	-477.64	-481.1
2:550000	YBR158W	YGR138C	302.374	306.323	306.651
2:550000	YBR158W	YDR425W	-510.598	-498.538	-474.742
2:550000	YBR158W	YGL081W	198.852	207.524	203.294
2:550000	YBR158W	YPR105C	-769.997	-763.716	-748.797
2:550000	YBR158W	YDR421W	-308.439	-300.565	-306.13
2:550000	YBR158W	YNL078W	-451.103	-443.393	-441.772
2:550000	YBR158W	YOR380W	-608.494	-600.663	-600.915
2:550000	YBR158W	YMR200W	-837.455	-829.511	-835.627
2:550000	YBR158W	YGL097W	-882.535	-875.743	-881.782
2:550000	YBR158W	YJR034W	177.362	188.3	188.776
2:550000	YBR158W	YCL055W	10.216	13.6391	11.486
2:550000	YBR158W	YLR405W	67.0664	77.1449	81.8332
2:550000	YBR158W	YOR129C	-512.245	-501.861	-500.132
2:550000	YBR158W	YBR139W	-453.431	-446.696	-433.409
2:550000	YBR158W	YPR074C	-1187.61	-1175.42	-1177.17
2:550000	YBR158W	YDL179W	-166.522	-162.338	-160.643
2:550000	YBR158W	YJR147W	533.023	541.545	545.833
2:550000	YBR158W	YPL158C	-525.933	-516.482	-521.761
2:550000	YBR158W	YKL003C	154.501	164.415	158.282

2:550000	YBR158W	YIL098C	382.927	394.493	389.597
2:550000	YBR158W	YLR390W	-19.7729	-9.06062	-15.0005
2:550000	YBR158W	YBR111W-A	87.1284	91.7807	104.911
2:550000	YBR158W	YOR316C	-640.35	-632.313	-627.319
2:550000	YBR158W	YHR059W	325.645	335.972	331.629
2:550000	YBR158W	YML009C	-151.712	-143.828	-135.736
2:550000	YBR158W	YOL115W	-443.144	-433.835	-420.694
2:550000	YBR158W	YPL072W	76.9704	83.0097	77.3103
2:550000	YBR158W	YJR101W	-64.0029	-55.0799	-60.9165
2:550000	YBR158W	YLR079W	-759.015	-749.542	-747.382
2:550000	YBR158W	YFL001W	-259.91	-248.035	-244.886
2:550000	YBR158W	YOR232W	-240.756	-234.478	-240.362
2:550000	YBR158W	YPR194C	1352.85	1361.74	1366.64
2:550000	YBR158W	YLR258W	794.716	806.989	811.249
2:550000	YBR158W	YAL041W	-750.532	-742.491	-748.353
2:550000	YBR158W	YMR024W	-79.035	-67.3185	-69.0733
2:550000	YBR158W	YNR053C	621.289	633.407	637.782
2:550000	YBR158W	YJL054W	-776.822	-765.502	-768.587
2:550000	YBR158W	YAL024C	-397.446	-387.793	-386.329
2:550000	YBR158W	YBR126C	295.274	296.553	306.836
2:550000	YBR158W	YHL023C	-561.755	-555.682	-561.459
2:550000	YBR158W	YNL037C	7.76807	18.3785	13.4888
2:550000	YBR158W	YJL122W	782.037	789.091	783.913
2:550000	YBR158W	YJL110C	50.7191	59.2678	53.9046
2:550000	YBR158W	YDR115W	119.823	129.011	122.903
2:550000	YBR158W	YPL063W	-1010.02	-1000.39	-995.997
2:550000	YBR158W	YNL299W	491.057	494.177	519.875
2:550000	YBR158W	YHR143W	506.042	512.153	587.789
2:550000	YBR158W	YKR091W	-271.264	-259.842	-258.003
2:550000	YBR158W	YIL149C	-819.472	-809.25	-795.732
2:550000	YBR158W	YJL053W	-644.404	-638.146	-618.564
2:550000	YBR158W	YPL082C	-288.491	-280.488	-241.363
2:550000	YBR158W	YOL082W	97.1994	106.985	104.207
2:550000	YBR158W	YGR044C	-90.7495	-83.8587	-89.6898
3:90000	YCL018W	YIL121W	40.8795	49.5865	45.2002
3:90000	YCL018W	YER073W	787.523	791.238	795.716
3:90000	YCL018W	YKR071C	-100.128	-98.6248	-96.9154
3:90000	YCL018W	YDL215C	629.456	637.654	635.342
3:90000	YCL018W	YLR355C	-278.199	-271.772	-274.217
3:90000	YCL018W	YCL017C	-549.455	-537.904	-543.832
3:90000	YCL018W	YLR348C	-90.1865	-84.3809	-89.0237
3:90000	YCL018W	YCR018C	-152.423	-140.759	-146.8
3:90000	YCL018W	YBR068C	890.386	895.654	897.336
3:90000	YCL018W	YHR047C	-326.951	-321.07	-320.679
3:90000	YCL018W	YIL050W	14.4616	25.8664	19.998
3:90000	YCL018W	YNL241C	-787.489	-781.737	-783.491
3:90000	YCL018W	YKR095W-A	-308.383	-298.706	-304.858

3:90000	YCL018W	YOR226C	-429.292	-417.056	-422.324
3:90000	YCL018W	YKL029C	656.532	664.268	661.279
3:90000	YCL018W	YCL026C-B	0.658558	11.3966	5.37921
3:90000	YCL018W	YGL125W	-18.384	-6.77211	-12.5446
3:90000	YCL018W	YNL104C	-484.341	-472.424	-477.699
3:90000	YCL018W	YCL004W	-439.803	-432.04	-437.973
3:90000	YCL018W	YCL021W-A	-237.288	-227.358	-233.508
3:90000	YCL018W	YCL016C	-667.425	-658.055	-657.051
3:90000	YCL018W	YMR108W	-18.6874	-17.2697	-13.5031
3:90000	YCL026C-A	YCL018W	-104.478	-95.3292	-100.201
3:90000	YCL026C-A	YPL189W	-163.649	-162.319	-163.363
3:90000	YCL026C-A	YER073W	744.775	756.937	753.593
3:90000	YCL026C-A	YBR085C-A	-119.758	-116.079	-109.133
3:90000	YCL026C-A	YKR071C	-115.179	-108.352	-99.4983
3:90000	YCL026C-A	YCL017C	-528.302	-518.568	-516.35
3:90000	YCL026C-A	YCR018C	-154.573	-142.761	-144.994
3:90000	YCL026C-A	YHR047C	-317.818	-311.143	-301.907
3:90000	YCL026C-A	YIL050W	7.53499	15.5577	19.1759
3:90000	YCL026C-A	YNL241C	-732.18	-729.071	-726.741
3:90000	YCL026C-A	YKR095W-A	-277.894	-265.978	-271.676
3:90000	YCL026C-A	YOR226C	-402.491	-396.794	-401.865
3:90000	YCL026C-A	YKL029C	627.263	634.972	629.068
3:90000	YCL026C-A	YCL026C-B	-10.6417	1.32983	3.55578
3:90000	YCL026C-A	YOR227W	-315.622	-315.584	-306.574
3:90000	YCL026C-A	YGL125W	-13.1068	-7.78574	-2.35386
3:90000	YCL026C-A	YKL120W	463.908	470.68	475.725
3:90000	YCL026C-A	YCL021W-A	-215.974	-204.092	-208.15
3:90000	YCL017C	YIL121W	22.5231	29.0898	45.2002
3:90000	YCL017C	YCL026C-A	210.855	215.824	214.227
3:90000	YCL017C	YCR018C	-152.857	-141.257	-146.8
3:90000	YCL017C	YJR148W	-878.798	-873.283	-862.806
3:90000	YCL017C	YGL125W	-25.8569	-13.5662	-12.5446
3:90000	YCL017C	YCL021W-A	-239.411	-229.908	-233.508
3:90000	YCL026C-B	YCL018W	-105.564	-97.8126	-100.201
3:90000	YCL026C-B	YIL121W	35.6683	46.254	48.0395
3:90000	YCL026C-B	YDL215C	595.375	607.241	607.48
3:90000	YCL026C-B	YLR355C	-264.221	-255.061	-241.568
3:90000	YCL026C-B	YJR016C	-117.061	-106.913	-102.778
3:90000	YCL026C-B	YCL017C	-524.152	-515.793	-516.35
3:90000	YCL026C-B	YCR018C	-151.69	-139.706	-144.994
3:90000	YCL026C-B	YBR068C	849.04	859.987	855.904
3:90000	YCL026C-B	YKR095W-A	-277.116	-268.981	-271.676
3:90000	YCL026C-B	YKL029C	612.131	619.107	629.068
3:90000	YCL026C-B	YJR148W	-809.047	-797.742	-803.437
3:90000	YCL026C-B	YGL125W	-6.2746	3.70604	-2.35386
3:90000	YCL026C-B	YHR208W	-25.0639	-16.1766	-11.7728
3:90000	YCL026C-B	YCL021W-A	-214.238	-202.076	-208.15

3:90000	YCL026C-B	YMR108W	-30.905	-26.5726	-6.58891
3:90000	YCL004W	YCL018W	-128.669	-121.744	-124.584
3:90000	YCL004W	YPL189W	-181.242	-180.753	-177.181
3:90000	YCL004W	YIL121W	26.2282	33.2792	37.9769
3:90000	YCL004W	YBR085C-A	-117.29	-113.87	-115.893
3:90000	YCL004W	YLR348C	-160.017	-159.376	-94.2249
3:90000	YCL004W	YCR018C	-155.49	-145.034	-148.741
3:90000	YCL004W	YHR047C	-342.095	-332.529	-327.817
3:90000	YCL004W	YIL050W	11.3681	21.594	17.8377
3:90000	YCL004W	YNL241C	-789.463	-778.261	-784.344
3:90000	YCL004W	YKR095W-A	-317.383	-315.725	-301.237
3:90000	YCL004W	YOR226C	-430.145	-419.285	-420.392
3:90000	YCL004W	YKL029C	647.154	654.232	660.298
3:90000	YCL004W	YCL026C-B	-18.5279	-12.0465	-8.90532
3:90000	YCL004W	YGL125W	-26.3394	-16.3088	-13.7282
3:90000	YCL004W	YCL021W-A	-233.23	-224.527	-223.766
3:90000	YCL004W	YMR108W	-40.9325	-36.1899	-19.2938
3:90000	YCL021W-A	YER073W	789.812	801.11	795.716
3:90000	YCL021W-A	YKR071C	-99.4811	-93.1937	-96.9154
3:90000	YCL021W-A	YPL084W	-612.851	-605.437	-610.15
3:90000	YCL021W-A	YDL215C	630.125	640.038	635.342
3:90000	YCL021W-A	YJR016C	-124.343	-118.732	-120.608
3:90000	YCL021W-A	YCL017C	-549.496	-540.631	-543.832
3:90000	YCL021W-A	YLR348C	-94.6806	-86.9769	-89.0237
3:90000	YCL021W-A	YCR018C	-152.074	-141.237	-146.8
3:90000	YCL021W-A	YBR068C	891.506	901.256	897.336
3:90000	YCL021W-A	YHR047C	-327.81	-322.299	-320.679
3:90000	YCL021W-A	YNL241C	-787.244	-777.433	-783.491
3:90000	YCL021W-A	YKR095W-A	-309.155	-300.219	-304.858
3:90000	YCL021W-A	YOR226C	-436.304	-429.411	-422.324
3:90000	YCL021W-A	YCL026C-B	1.56034	9.19147	5.37921
3:90000	YCL021W-A	YGL125W	-17.8303	-14.6996	-12.5446
3:90000	YCL021W-A	YNL104C	-481.312	-471.551	-477.699
3:90000	YCL021W-A	YCL004W	-446.848	-435.049	-437.973
3:90000	YCL021W-A	YMR108W	-13.8978	-7.8385	-13.5031
3:90000	YCL016C	YCL018W	-132.081	-123.276	-124.584
3:90000	YCL016C	YPL189W	-187.486	-177.348	-177.181
3:90000	YCL016C	YCL026C-A	195.829	199.895	209.546
3:90000	YCL016C	YKR071C	-131.502	-124.864	-100.889
3:90000	YCL016C	YFR005C	-407.922	-401.536	-389.537
3:90000	YCL016C	YCL017C	-554.474	-547.551	-545.431
3:90000	YCL016C	YCR018C	-154.051	-142.869	-148.741
3:90000	YCL016C	YHR047C	-345.859	-335.571	-327.817
3:90000	YCL016C	YIL050W	9.48524	18.8184	17.8377
3:90000	YCL016C	YNL241C	-791.886	-780.092	-784.344
3:90000	YCL016C	YKR095W-A	-304.843	-295.485	-301.237
3:90000	YCL016C	YCL026C-B	-59.7654	-49.1167	-8.90532

3:90000	YCL016C	YGL125W	-20.0247	-7.77586	-13.7282
3:90000	YCL016C	YNL104C	-511.805	-501.475	-477.612
3:90000	YCL016C	YCL021W-A	-229.475	-218.037	-223.766
4:90000	YDL216C	YGL259W	129.973	141.92	152.396
4:90000	YDL216C	YJL189W	-575.705	-574.161	-533.814
4:90000	YDL216C	YNL178W	-780.012	-774.02	-723.397
4:90000	YDL216C	YDL217C	-67.613	-58.0495	85.3573
4:90000	YDL216C	YDL205C	103.274	108.454	119.85
4:90000	YDL216C	YDL214C	362.889	370.794	367.586
4:90000	YDL216C	YDL207W	-734.334	-722.752	-650.689
4:90000	YDL216C	YCR031C	-1026.71	-1023.13	-977.105
4:90000	YDL216C	YBR189W	-413.89	-413.707	-334.508
4:90000	YDL216C	YPR132W	-1059.99	-1058.52	-1013.51
4:90000	YDL216C	YDL209C	-305.797	-294.204	-232.827
4:90000	YDL216C	YDL204W	959.119	966.876	960.993
4:90000	YDL199C	YDR450W	-904.272	-903.548	-830.241
4:90000	YDL199C	YER124C	503.686	512.43	658.617
4:90000	YDL199C	YML063W	-727.767	-721.934	-666.779
4:90000	YDL199C	YGR034W	-339.416	-337.62	-231.422
4:90000	YDL199C	YML024W	-968.739	-960.97	-913.642
4:90000	YDL199C	YDL214C	351.055	359.444	369.8
4:90000	YDL199C	YPR132W	-1073.62	-1071.78	-1012.62
4:90000	YDL199C	YBR191W	-789.701	-787.332	-689.842
4:90000	YDL199C	YHR143W	551.372	559.638	650.468
4:90000	YDL205C	YDL216C	-117.115	-105.324	-25.8483
4:90000	YDL205C	YGL259W	143.207	155.074	151.554
4:90000	YDL205C	YDL214C	354.111	357.368	356.158
4:90000	YDL205C	YDL210W	1308.98	1320.22	1317.09
4:90000	YDL214C	YDR447C	-215.872	-209.1	-204.344
4:90000	YDL214C	YDL061C	-774.518	-770.011	-765.269
4:90000	YDL214C	YDR450W	-847.02	-842.388	-820.01
4:90000	YDL214C	YOR338W	603.323	615.337	795.36
4:90000	YDL214C	YDL218W	192.209	202.142	278.961
4:90000	YDL214C	YJR123W	-1010.09	-1006.72	-994.523
4:90000	YDL214C	YKR057W	-245.478	-234.714	-225.56
4:90000	YDL214C	YGR214W	-236.226	-229.297	-218.347
4:90000	YDL214C	YER124C	606.325	611.721	653.494
4:90000	YDL214C	YMR142C	-738.908	-733.055	-729.193
4:90000	YDL214C	YML026C	-260.014	-252.201	-245.307
4:90000	YDL214C	YJL189W	-543.586	-535.408	-525.796
4:90000	YDL214C	YML063W	-682.662	-679.896	-660.755
4:90000	YDL214C	YGR034W	-252.711	-245.837	-229.232
4:90000	YDL214C	YDL217C	11.2527	12.474	86.1008
4:90000	YDL214C	YDL130W	-660.034	-649.012	-646.129
4:90000	YDL214C	YMR118C	622.827	632.367	657.947
4:90000	YDL214C	YDL205C	121.969	123.071	127.455
4:90000	YDL214C	YML024W	-920.636	-913.113	-904.824

4:90000	YDL214C	YML026C	-1109.05	-1108.9	-1085.33
4:90000	YDL214C	YCR031C	-973.897	-967.144	-968.223
4:90000	YDL214C	YLR325C	-216.77	-206.121	-195.32
4:90000	YDL214C	YBR166C	-461.109	-453.977	-370.71
4:90000	YDL214C	YJL190C	-137.488	-132.363	-115.716
4:90000	YDL214C	YBR189W	-354.682	-346.871	-331.434
4:90000	YDL214C	YPR132W	-1017.67	-1011.42	-1002.19
4:90000	YDL214C	YER074W	-448.759	-441.646	-438.13
4:90000	YDL214C	YKL107W	399.236	408.967	426.568
4:90000	YDL214C	YHR010W	-531.291	-521.831	-510.529
4:90000	YDL214C	YOR293W	-55.2339	-43.4289	-20.824
4:90000	YDL214C	YFR032C-A	-560.827	-555.757	-543.995
4:90000	YDL214C	YER117W	-533.439	-525.016	-520.039
4:90000	YDL214C	YBR191W	-712.644	-705.281	-681.057
4:90000	YDL214C	YDL210W	1309.89	1317.84	1317.61
4:90000	YDL214C	YNL301C	-1044.86	-1035.95	-1033.24
4:90000	YDL214C	YBR181C	-1054.9	-1052.78	-1029.6
4:90000	YDL214C	YDL081C	-774.293	-771.285	-753.486
4:90000	YDL214C	YMR143W	-815.965	-812.911	-800.313
4:90000	YDL214C	YGL135W	-1299.2	-1290.54	-1272.19
4:90000	YDL214C	YJR094W-A	-46.6796	-37.3066	-17.2367
4:90000	YDL214C	YHR021C	-493.281	-482.911	-475.347
4:90000	YDL214C	YHR143W	576.29	582.579	646.579
4:90000	YDL214C	YFR031C-A	-918.967	-914.234	-916.709
4:90000	YDL214C	YOR293W	-93.3199	-81.5801	-61.4479
4:90000	YDL207W	YDL199C	64.1401	68.1864	65.8454
4:90000	YDL207W	YML024W	-932.046	-930.933	-901.781
4:90000	YDL207W	YDL214C	357.603	364.547	361.955
4:90000	YDL207W	YDL203C	-574.923	-571.548	-562.669
4:90000	YDL207W	YDL197C	-389.246	-378.239	-336.221
4:90000	YDL207W	YPR132W	-1012.74	-1011.57	-997.001
4:90000	YDL203C	YDR447C	-660.008	-653.656	-608.124
4:90000	YDL203C	YDR447C	-284.689	-273.488	-200.669
4:90000	YDL203C	YDL061C	-836.279	-826.379	-764.003
4:90000	YDL203C	YDL191W	-1153.93	-1147.74	-1094.52
4:90000	YDL203C	YDR450W	-877.64	-866.102	-817.24
4:90000	YDL203C	YOR338W	699.291	702.031	790.009
4:90000	YDL203C	YJR123W	-1030.8	-1020.74	-992.413
4:90000	YDL203C	YKR057W	-271.333	-263.142	-224.61
4:90000	YDL203C	YGR214W	-280.605	-270.422	-214.531
4:90000	YDL203C	YER124C	608.043	613.296	654.371
4:90000	YDL203C	YDL199C	62.9403	70.6701	76.5492
4:90000	YDL203C	YMR142C	-785.901	-776.006	-730.144
4:90000	YDL203C	YML026C	-321.236	-310.413	-246.836
4:90000	YDL203C	YJL189W	-592.331	-584.565	-532.279
4:90000	YDL203C	YML063W	-699.043	-686.826	-654.782
4:90000	YDL203C	YNL178W	-748.557	-746.943	-716.13

4:90000	YDL203C	YGR034W	-311.131	-299.771	-225.727
4:90000	YDL203C	YDL130W	-706.742	-696.915	-646.441
4:90000	YDL203C	YMR215W	-403.661	-402.068	-348
4:90000	YDL203C	YDL061C	-916.939	-910.245	-834.885
4:90000	YDL203C	YML024W	-949.531	-937.775	-900.617
4:90000	YDL203C	YML026C	-1135.93	-1132.86	-1093.95
4:90000	YDL203C	YDL214C	331.044	341.009	356.158
4:90000	YDL203C	YCR031C	-1027.97	-1018.92	-969.795
4:90000	YDL203C	YLR325C	-270.637	-260.743	-197.554
4:90000	YDL203C	YJL190C	-173.014	-162.296	-118.022
4:90000	YDL203C	YBR189W	-380.93	-373.981	-330.905
4:90000	YDL203C	YPR132W	-1051.85	-1040.23	-999.83
4:90000	YDL203C	YER074W	-497.91	-487.086	-435.294
4:90000	YDL203C	YKL107W	358.4	362.093	425.393
4:90000	YDL203C	YBL087C	-762.062	-753.038	-705.683
4:90000	YDL203C	YHR010W	-580.107	-569.529	-510.053
4:90000	YDL203C	YOR293W	-93.4336	-83.0743	-18.9454
4:90000	YDL203C	YFR032C-A	-615.808	-604.872	-545.739
4:90000	YDL203C	YER117W	-590.296	-579.541	-522.979
4:90000	YDL203C	YBR191W	-747.54	-736.044	-680.394
4:90000	YDL203C	YGR085C	-1082.91	-1080.81	-1045.36
4:90000	YDL203C	YNL301C	-1086.96	-1076.76	-1034.43
4:90000	YDL203C	YBR181C	-1096.44	-1084.45	-1024.8
4:90000	YDL203C	YDL081C	-802.303	-792.906	-758.043
4:90000	YDL203C	YMR143W	-874.378	-865.015	-801.486
4:90000	YDL203C	YGL135W	-1325.58	-1315.94	-1272.13
4:90000	YDL203C	YDL204W	947.287	948.92	947.652
4:90000	YDL203C	YJR094W-A	-83.9039	-74.9831	-14.8274
4:90000	YDL203C	YHR021C	-541.373	-530.244	-474.428
4:90000	YDL203C	YHR143W	612.013	620.234	647.511
4:90000	YDL203C	YFR031C-A	-946.806	-936.339	-914.724
4:90000	YDL203C	YOR293W	-132.966	-122.793	-58.7744
4:90000	YDL197C	YDR447C	-225.038	-213.776	-206.378
4:90000	YDL197C	YDL061C	-796.745	-786.992	-773.959
4:90000	YDL197C	YDL191W	-1135.31	-1128.88	-1108.16
4:90000	YDL197C	YDR450W	-832.612	-824.938	-830.043
4:90000	YDL197C	YOR338W	801.971	808.639	806.628
4:90000	YDL197C	YDL218W	221.868	232.829	283.635
4:90000	YDL197C	YJR123W	-1010.68	-1003.38	-1005.44
4:90000	YDL197C	YKR057W	-253.877	-241.957	-228.99
4:90000	YDL197C	YGR214W	-247.497	-237.962	-219.068
4:90000	YDL197C	YMR142C	-753.906	-744.994	-742.071
4:90000	YDL197C	YGL259W	140.312	148.898	150.497
4:90000	YDL197C	YML026C	-267.377	-256.95	-249.876
4:90000	YDL197C	YJL189W	-548.37	-546.194	-544.651
4:90000	YDL197C	YML063W	-671.045	-661.724	-666.737
4:90000	YDL197C	YNL178W	-732.375	-722.868	-721.86

4:90000	YDL197C	YGR034W	-246.925	-236.121	-230.916
4:90000	YDL197C	YDL217C	41.0908	47.7698	92.4554
4:90000	YDL197C	YDL130W	-679.078	-667.354	-655.162
4:90000	YDL197C	YMR118C	621.18	627.63	662.121
4:90000	YDL197C	YDL061C	-857.275	-852.142	-846.2
4:90000	YDL197C	YML024W	-918.251	-906.98	-913.089
4:90000	YDL197C	YDL214C	366.497	377.048	371.175
4:90000	YDL197C	YCR031C	-1000.72	-991.797	-983.176
4:90000	YDL197C	YLR325C	-226.382	-214.859	-201.035
4:90000	YDL197C	YBR166C	-401.812	-398.938	-373.391
4:90000	YDL197C	YJL190C	-130.01	-121.864	-120.961
4:90000	YDL197C	YBR189W	-348.546	-336.892	-335.922
4:90000	YDL197C	YPR132W	-1016.24	-1006.14	-1012.24
4:90000	YDL197C	YER074W	-461.614	-450.827	-443.89
4:90000	YDL197C	YKL107W	354.047	357.901	432.254
4:90000	YDL197C	YBL087C	-742.144	-734.377	-713.099
4:90000	YDL197C	YGL123W	-947.692	-945.848	-942.097
4:90000	YDL197C	YHR010W	-529.43	-517.17	-518.782
4:90000	YDL197C	YDL209C	-221.915	-216.556	-203.884
4:90000	YDL197C	YOR293W	-43.4578	-32.8395	-22.5275
4:90000	YDL197C	YFR032C-A	-564.98	-557.883	-555.792
4:90000	YDL197C	YER117W	-548.895	-540.277	-529.927
4:90000	YDL197C	YBR191W	-702.762	-691.696	-689.674
4:90000	YDL197C	YDL210W	1284.94	1292.35	1330.67
4:90000	YDL197C	YNL301C	-1070.09	-1059.95	-1049.49
4:90000	YDL197C	YBR181C	-1047.55	-1039	-1039.97
4:90000	YDL197C	YDL081C	-770.921	-765.617	-765.852
4:90000	YDL197C	YMR143W	-820.576	-817.246	-815.946
4:90000	YDL197C	YGL135W	-1305.33	-1294.49	-1288.96
4:90000	YDL197C	YDL204W	944.383	955.231	958.196
4:90000	YDL197C	YJR094W-A	-56.6829	-46.7318	-17.7194
4:90000	YDL197C	YHR021C	-503.513	-492.563	-482.415
4:90000	YDL197C	YHR143W	634.627	641.403	649.579
4:90000	YDL197C	YFR031C-A	-942.955	-935.054	-927.987
4:90000	YDL197C	YOR293W	-81.9887	-70.7016	-62.903
4:90000	YDL209C	YJR123W	-961.328	-961.315	-935.476
4:90000	YDL209C	YDL199C	-18.9186	-15.5571	76.2553
4:90000	YDL209C	YML026C	-253.019	-252.598	-225.15
4:90000	YDL209C	YJL189W	-528.738	-526.829	-496.62
4:90000	YDL209C	YDL130W	-633.201	-629.356	-604.273
4:90000	YDL209C	YML024W	-889.305	-880.677	-862.914
4:90000	YDL209C	YDL214C	347.22	353.6	348.956
4:90000	YDL209C	YDL203C	-600.526	-594.412	-518.726
4:90000	YDL209C	YDL197C	-394.196	-382.616	-308.898
4:90000	YDL209C	YHR010W	-514.441	-512.466	-478.909
4:90000	YDL209C	YOR293W	-56.0693	-53.6706	-7.78737
4:90000	YDL209C	YDL204W	914.396	922.132	918.221

4:90000	YDL209C	YHR021C	-483.336	-481.58	-442.142
4:90000	YDL209C	YFR031C-A	-879.548	-878.528	-857.873
4:90000	YDL209C	YOR293W	-88.9836	-87.4378	-45.7661
4:90000	YDL210W	YDR447C	-285.798	-283.402	-196.353
4:90000	YDL210W	YOR338W	450.138	450.219	778.04
4:90000	YDL210W	YGR214W	-325.441	-322.39	-209.869
4:90000	YDL210W	YER124C	571.072	579.165	635.025
4:90000	YDL210W	YML063W	-693.444	-688.868	-646.676
4:90000	YDL210W	YDL189W	-914.71	-908.908	-832.93
4:90000	YDL210W	YGR034W	-337.598	-333.484	-221.775
4:90000	YDL210W	YDL130W	-746.118	-741.262	-642.245
4:90000	YDL210W	YMR118C	468.434	473.688	648.158
4:90000	YDL210W	YML024W	-945.829	-937.286	-889.53
4:90000	YDL210W	YDL214C	321.926	331.051	351.713
4:90000	YDL210W	YBR166C	-608.485	-600.999	-375.663
4:90000	YDL210W	YBR189W	-468.923	-464.789	-335.157
4:90000	YDL210W	YPR132W	-1067.41	-1066	-1001.38
4:90000	YDL210W	YER074W	-525.661	-523.365	-431.495
4:90000	YDL210W	YKL107W	330.623	338.326	422.143
4:90000	YDL210W	YHR010W	-625.63	-621.596	-528.97
4:90000	YDL210W	YOR293W	-165.358	-160.167	-18.2959
4:90000	YDL210W	YBR191W	-803.079	-798.382	-674.142
4:90000	YDL210W	YJR094W-A	-181.876	-177.915	-18.463
4:90000	YDL210W	YHR021C	-574.205	-569.731	-467.301
4:90000	YDL210W	YHR143W	545.509	555.105	628.523
4:90000	YDL210W	YOR293W	-207.662	-202.319	-57.5564
4:90000	YDL204W	YDR447C	-303.629	-298.959	-196.353
4:90000	YDL204W	YDL061C	-868.587	-867.798	-754.805
4:90000	YDL204W	YDR450W	-866.242	-860.502	-808.673
4:90000	YDL204W	YJR123W	-1034.86	-1031.71	-982.8
4:90000	YDL204W	YGR214W	-291.691	-289.044	-209.869
4:90000	YDL204W	YER124C	596.749	605.16	635.025
4:90000	YDL204W	YDL199C	-56.7266	-50.323	76.1382
4:90000	YDL204W	YML026C	-340.912	-338.596	-247.066
4:90000	YDL204W	YML063W	-686.127	-677.125	-646.676
4:90000	YDL204W	YGR034W	-309.858	-302.643	-221.775
4:90000	YDL204W	YDL130W	-734.94	-733.078	-642.245
4:90000	YDL204W	YML024W	-950.796	-940.675	-889.53
4:90000	YDL204W	YDL214C	300.144	311.326	351.713
4:90000	YDL204W	YLR325C	-292.533	-292.424	-196.975
4:90000	YDL204W	YJL190C	-180.126	-177.59	-119.581
4:90000	YDL204W	YPR132W	-1066.35	-1061.39	-1001.38
4:90000	YDL204W	YER074W	-504.245	-501.187	-431.495
4:90000	YDL204W	YHR010W	-612.358	-607.47	-528.97
4:90000	YDL204W	YOR293W	-105.208	-98.7209	-18.2959
4:90000	YDL204W	YFR032C-A	-599.903	-595.636	-536.898
4:90000	YDL204W	YBR191W	-750.324	-743.872	-674.142

4:90000	YDL204W	YNL301C	-1099.22	-1097.9	-1025.33
4:90000	YDL204W	YBR181C	-1090.74	-1087.36	-1014.68
4:90000	YDL204W	YDL081C	-790.019	-789.803	-747.64
4:90000	YDL204W	YJR094W-A	-123.151	-122.905	-18.463
4:90000	YDL204W	YHR021C	-561.412	-556.691	-467.301
4:90000	YDL204W	YHR143W	600.165	610.053	628.523
4:90000	YDL204W	YFR031C-A	-985.279	-982.388	-916.958
4:90000	YDL204W	YOR293W	-148.85	-142.874	-57.5564
5:190000	YER020W	YDR447C	-616.978	-610.193	-596.015
5:190000	YER020W	YDR447C	-229.894	-220.475	-203.443
5:190000	YER020W	YDL061C	-802.174	-796.58	-762.988
5:190000	YER020W	YCL054W	705.019	707.918	772.039
5:190000	YER020W	YDL191W	-1128.28	-1125.92	-1087.79
5:190000	YER020W	YBR084C-A	-338.41	-334.994	-314.647
5:190000	YER020W	YDR450W	-827.753	-819.245	-812.351
5:190000	YER020W	YLR029C	-999.043	-992.128	-970.266
5:190000	YER020W	YIL052C	-477.31	-469.153	-445.109
5:190000	YER020W	YPL131W	-783.139	-776.539	-754.982
5:190000	YER020W	YJR123W	-1010.2	-1000.76	-988.493
5:190000	YER020W	YDL229W	9.91012	17.1028	32.1068
5:190000	YER020W	YKL180W	-466.307	-463.347	-435.15
5:190000	YER020W	YLR185W	-373.743	-367.034	-342.711
5:190000	YER020W	YGL031C	-900.185	-888.353	-890.639
5:190000	YER020W	YBL027W	-420.619	-413.308	-394.261
5:190000	YER020W	YER102W	-630.697	-624.553	-603.945
5:190000	YER020W	YGL189C	-1101.64	-1099.36	-1076
5:190000	YER020W	YKR057W	-267.799	-263.58	-226.423
5:190000	YER020W	YGR148C	-1030.94	-1022.45	-999.734
5:190000	YER020W	YGR214W	-250.462	-243.933	-217.191
5:190000	YER020W	YMR142C	-756.451	-749.491	-729.868
5:190000	YER020W	YLR075W	-1715.96	-1715.12	-1694.16
5:190000	YER020W	YDR064W	-1016.78	-1006.4	-1000.49
5:190000	YER020W	YOL120C	-521.317	-515.008	-497.275
5:190000	YER020W	YPR043W	-1160.84	-1151.28	-1139.12
5:190000	YER020W	YOL127W	-1111.99	-1102.14	-1096.17
5:190000	YER020W	YML026C	-270.855	-262.513	-246.938
5:190000	YER020W	YMR242C	-399.143	-394.38	-364.729
5:190000	YER020W	YMR116C	-1114.01	-1111.14	-1075.2
5:190000	YER020W	YDR418W	-679.551	-677.879	-638.365
5:190000	YER020W	YBR048W	-146.206	-141.157	-129.002
5:190000	YER020W	YJL189W	-538.97	-529.45	-522.371
5:190000	YER020W	YLR388W	-103.527	-102.906	-58.7373
5:190000	YER020W	YML063W	-670.422	-660.872	-654.044
5:190000	YER020W	YLR388W	-207.169	-206.206	-163.052
5:190000	YER020W	YKR094C	-494.374	-489.925	-468.778
5:190000	YER020W	YLR048W	-41.2254	-37.7222	-3.11586
5:190000	YER020W	YBL072C	-808.303	-802.391	-791.866

5:190000	YER020W	YDR012W	-292.015	-282.142	-255.13
5:190000	YER020W	YOR312C	-573.032	-569.388	-540.33
5:190000	YER020W	YNL178W	-726.518	-715.176	-706.756
5:190000	YER020W	YIL018W	-236.232	-226.425	-216.816
5:190000	YER020W	YGR034W	-255.904	-249.157	-231.522
5:190000	YER020W	YDL130W	-685.167	-678.494	-649.001
5:190000	YER020W	YDL061C	-861.427	-854.177	-826.282
5:190000	YER020W	YHL033C	-366.561	-357.421	-344.16
5:190000	YER020W	YDL083C	42.8017	49.9235	71.8035
5:190000	YER020W	YML024W	-914.395	-909.209	-909.819
5:190000	YER020W	YOR167C	-616.819	-614.64	-576.403
5:190000	YER020W	YPL143W	-848.261	-841.553	-817.102
5:190000	YER020W	YPL249C-A	-880.334	-872.974	-856.013
5:190000	YER020W	YDR025W	-52.1802	-48.9294	-11.6778
5:190000	YER020W	YER131W	303.309	307.359	339.711
5:190000	YER020W	YLR325C	-239.9	-237.861	-198.77
5:190000	YER020W	YPL079W	-429.6	-425.495	-390.752
5:190000	YER020W	YLR061W	-235.27	-232.063	-197.153
5:190000	YER020W	YHR141C	-879.44	-872.783	-848.588
5:190000	YER020W	YJL190C	-139.359	-129.445	-116.245
5:190000	YER020W	YBR189W	-356.914	-348.098	-329.909
5:190000	YER020W	YNL302C	-434.277	-427.019	-415.21
5:190000	YER020W	YPR132W	-1015.55	-1003.54	-1001.65
5:190000	YER020W	YKL006W	-530.082	-521.776	-506.394
5:190000	YER020W	YER074W	-466.629	-457.801	-437.24
5:190000	YER020W	YFR031C-A	-205.556	-195.348	-178.952
5:190000	YER020W	YHL033C	-473.346	-462.653	-444.855
5:190000	YER020W	YER056C-A	-256.857	-256.453	-219.151
5:190000	YER020W	YGL147C	-342.384	-330.864	-333.485
5:190000	YER020W	YNL302C	-899.367	-891.11	-869.886
5:190000	YER020W	YHR179W	-830.507	-820.806	-805.776
5:190000	YER020W	YML073C	-340.87	-338.323	-302.934
5:190000	YER020W	YBL087C	-737.896	-732.086	-701.25
5:190000	YER020W	YGL123W	-942.913	-932.321	-918.26
5:190000	YER020W	YOR312C	-679.027	-671.864	-654.822
5:190000	YER020W	YNL069C	-683.776	-678.296	-666.647
5:190000	YER020W	YHR010W	-536.631	-529.264	-512.006
5:190000	YER020W	YER074W	-646.321	-635.055	-631.588
5:190000	YER020W	YOR096W	-486.558	-475.671	-468.445
5:190000	YER020W	YOR293W	-48.6188	-42.1644	-20.8399
5:190000	YER020W	YGL076C	-1215.8	-1211.07	-1171.76
5:190000	YER020W	YFR032C-A	-558.112	-548.471	-540.106
5:190000	YER020W	YER117W	-555.087	-547.573	-524.127
5:190000	YER020W	YBR191W	-708.479	-701.993	-680.183
5:190000	YER020W	YOR063W	-1160.44	-1158.51	-1154.06
5:190000	YER020W	YBR031W	-1373.62	-1370.32	-1343.49
5:190000	YER020W	YLL045C	-697.636	-688.549	-673.411

5:190000	YER020W	YLR340W	-982.002	-980.417	-942.288
5:190000	YER020W	YNL301C	-1070.32	-1063.17	-1034.87
5:190000	YER020W	YOL040C	-742.201	-732.089	-721.208
5:190000	YER020W	YBR181C	-1046.96	-1045.92	-1021.25
5:190000	YER020W	YDL081C	-775.043	-767.928	-750.532
5:190000	YER020W	YGR152C	-449.042	-446.156	-411.746
5:190000	YER020W	YMR143W	-817.209	-809.07	-794.761
5:190000	YER020W	YGL135W	-1308.75	-1306.54	-1272.05
5:190000	YER020W	YJL177W	2.49475	5.91	43.5799
5:190000	YER020W	YDL075W	-654.734	-646.665	-629.561
5:190000	YER020W	YHL001W	-927.158	-922.338	-889.346
5:190000	YER020W	YHR021C	-514.367	-508.184	-481.739
5:190000	YER020W	YBR191W	-238.214	-232.811	-217.928
5:190000	YER020W	YBL092W	-914.088	-904.447	-900.43
5:190000	YER020W	YJL136C	-869.36	-860.624	-839.385
5:190000	YER020W	YFR031C-A	-945.885	-936.602	-918.379
5:190000	YER020W	YOR293W	-87.6609	-79.7635	-61.2395
5:190000	YER020W	YGL030W	-1153.24	-1149.07	-1125.94
5:190000	YER020W	YMR143W	-722.484	-715.339	-700.087
5:190000	YER019W	YDR447C	-618.732	-607.355	-589.972
5:190000	YER019W	YDR447C	-231.93	-220.765	-201.329
5:190000	YER019W	YDL061C	-795.488	-784.255	-750.082
5:190000	YER019W	YDL191W	-1091.61	-1082.23	-1074.59
5:190000	YER019W	YBR084C-A	-343.338	-332.542	-312.608
5:190000	YER019W	YDR450W	-831.876	-820.06	-801.928
5:190000	YER019W	YLR029C	-975.356	-963.761	-956.001
5:190000	YER019W	YIL052C	-479.502	-468.095	-437.182
5:190000	YER019W	YPL131W	-777.743	-769.329	-742.176
5:190000	YER019W	YBR084W	290.341	300.669	417.068
5:190000	YER019W	YHL001W	-202.44	-192.602	-161.82
5:190000	YER019W	YJR123W	-1016.42	-1004.27	-976.242
5:190000	YER019W	YDL229W	-10.974	-2.54571	34.0791
5:190000	YER019W	YKL180W	-481.338	-472.528	-427.744
5:190000	YER019W	YLR185W	-372.006	-361.64	-336.859
5:190000	YER019W	YGL031C	-901.218	-889.092	-887.517
5:190000	YER019W	YBL072C	-1326.32	-1317.19	-1281.48
5:190000	YER019W	YBL027W	-425.32	-414.589	-390.992
5:190000	YER019W	YER102W	-635.634	-624.315	-595.471
5:190000	YER019W	YGL189C	-1100.97	-1091.16	-1061.08
5:190000	YER019W	YKR057W	-250.306	-240.149	-220.156
5:190000	YER019W	YGR085C	-93.5833	-84.9838	-50.4445
5:190000	YER019W	YGR148C	-1021.3	-1010.32	-987.181
5:190000	YER019W	YGR214W	-252.972	-243.915	-211.119
5:190000	YER019W	YMR142C	-748.503	-740.065	-721.513
5:190000	YER019W	YLR075W	-1705.02	-1694.3	-1673.65
5:190000	YER019W	YDR064W	-990.138	-988.337	-986.922
5:190000	YER019W	YOL120C	-529.815	-519.533	-489.515

5:190000	YER019W	YGL189C	-957.954	-955.937	-934.181
5:190000	YER019W	YIL069C	227.839	236.138	277.738
5:190000	YER019W	YPR043W	-1142.99	-1134.7	-1129.15
5:190000	YER019W	YOL127W	-1119.01	-1107.52	-1087.37
5:190000	YER019W	YEL054C	269.05	279.43	340.188
5:190000	YER019W	YML026C	-273.249	-263.127	-245.573
5:190000	YER019W	YMR242C	-402.563	-390.785	-359.537
5:190000	YER019W	YDR418W	-671.321	-660.529	-632.101
5:190000	YER019W	YBR048W	-157.546	-146.53	-137.006
5:190000	YER019W	YJL189W	-532.743	-520.632	-513.74
5:190000	YER019W	YLR388W	-99.6117	-90.0708	-56.8156
5:190000	YER019W	YML063W	-671.458	-659.237	-646.723
5:190000	YER019W	YLR388W	-199.116	-189.697	-160.251
5:190000	YER019W	YKR094C	-496.114	-485.713	-460.805
5:190000	YER019W	YLR344W	-168.696	-159.886	-126.085
5:190000	YER019W	YLR048W	-54.7081	-45.549	-1.09291
5:190000	YER019W	YBL072C	-816.4	-808.709	-793.835
5:190000	YER019W	YDR012W	-264.363	-259.421	-253.349
5:190000	YER019W	YOR312C	-557.437	-547.65	-532.718
5:190000	YER019W	YNL178W	-732.407	-722.091	-699.233
5:190000	YER019W	YIL018W	-254.918	-243.996	-213.6
5:190000	YER019W	YGR034W	-272.3	-260.914	-227.601
5:190000	YER019W	YEL026W	467.958	478.976	560.049
5:190000	YER019W	YDR471W	149.77	157.208	196.951
5:190000	YER019W	YDL130W	-678.082	-667.5	-641.544
5:190000	YER019W	YDL061C	-841.743	-831.453	-813.012
5:190000	YER019W	YHL033C	-370.147	-360.487	-340.335
5:190000	YER019W	YDL083C	30.9247	40.9235	74.0883
5:190000	YER019W	YLR448W	-161.701	-154.445	-113.564
5:190000	YER019W	YML024W	-904.3	-898.659	-898.282
5:190000	YER019W	YNL209W	-181.122	-172.105	-122.289
5:190000	YER019W	YOR167C	-593.934	-583.544	-565.922
5:190000	YER019W	YOR310C	871.883	884	948.768
5:190000	YER019W	YPL143W	-829.009	-819.515	-804.838
5:190000	YER019W	YPL249C-A	-863.495	-852.755	-842.722
5:190000	YER019W	YML026C	-1092.3	-1088.22	-1072.51
5:190000	YER019W	YMR194W	-206.569	-197.148	-140.209
5:190000	YER019W	YDR025W	-66.0479	-55.4341	-10.2233
5:190000	YER019W	YML056C	655.384	665.627	751.516
5:190000	YER019W	YER131W	264.012	275.534	335.803
5:190000	YER019W	YJL191W	98.8189	100.703	127.647
5:190000	YER019W	YLR325C	-225.873	-215.984	-192.946
5:190000	YER019W	YDR500C	-242.773	-234.326	-201.383
5:190000	YER019W	YPL079W	-433.706	-424.218	-382.383
5:190000	YER019W	YLR061W	-247.199	-239.568	-190.915
5:190000	YER019W	YHR141C	-859.226	-848.123	-837.704
5:190000	YER019W	YLR293C	-1283.95	-1273.82	-1241.8

5:190000	YER019W	YLR197W	770.952	782.317	887.483
5:190000	YER019W	YLR333C	-533.934	-525.283	-480.957
5:190000	YER019W	YJL190C	-146.009	-134.765	-113.574
5:190000	YER019W	YBR189W	-360.846	-350.616	-323.667
5:190000	YER019W	YNL302C	-428.856	-416.995	-407.991
5:190000	YER019W	YLR264W	-309.66	-304.596	-295.089
5:190000	YER019W	YPR132W	-1010.49	-999.123	-986.892
5:190000	YER019W	YNL096C	-136.447	-127.024	-72.8229
5:190000	YER019W	YKL006W	-534.691	-522.92	-499.085
5:190000	YER019W	YLR372W	-232.692	-227.345	-225.118
5:190000	YER019W	YER074W	-464.473	-453.872	-428.232
5:190000	YER019W	YFR031C-A	-208.124	-197.516	-173.959
5:190000	YER019W	YDL082W	212.942	221.42	288.821
5:190000	YER019W	YLR432W	-1.88995	10.2445	65.1276
5:190000	YER019W	YER056C-A	-255.253	-244.878	-213.69
5:190000	YER019W	YMR230W	-214.886	-203.87	-177.891
5:190000	YER019W	YGL147C	-352.35	-340.444	-328.807
5:190000	YER019W	YNL302C	-886.668	-876.255	-860.96
5:190000	YER019W	YNL255C	-156.452	-144.969	-76.4965
5:190000	YER019W	YML073C	-342.396	-332.271	-296.024
5:190000	YER019W	YBL087C	-721.915	-711.627	-689.8
5:190000	YER019W	YOL121C	41.5243	51.4767	89.4621
5:190000	YER019W	YGL123W	-942.034	-930.929	-904.511
5:190000	YER019W	YOR312C	-669.318	-657.385	-645.93
5:190000	YER019W	YNL069C	-689.648	-681.216	-657.891
5:190000	YER019W	YHR010W	-530.083	-519.245	-504.503
5:190000	YER019W	YER074W	-643.942	-631.971	-622.752
5:190000	YER019W	YOR096W	-491.686	-479.699	-461.385
5:190000	YER019W	YLR406C	293.716	302.603	334.057
5:190000	YER019W	YOR293W	-65.4477	-53.2747	-17.7825
5:190000	YER019W	YGL076C	-1176.12	-1167.71	-1160.2
5:190000	YER019W	YFR032C-A	-555.85	-544.048	-534.031
5:190000	YER019W	YER117W	-547.248	-536.424	-519.086
5:190000	YER019W	YBR191W	-717.403	-705.15	-672.224
5:190000	YER019W	YOR063W	-1151.28	-1149.72	-1142.4
5:190000	YER019W	YLR441C	-411.428	-400.738	-378.478
5:190000	YER019W	YBR031W	-1346.81	-1343.45	-1328.2
5:190000	YER019W	YLL045C	-696.251	-684.611	-662.765
5:190000	YER019W	YLR340W	-980.042	-969.565	-931.984
5:190000	YER019W	YNL301C	-1061.04	-1050.22	-1022.48
5:190000	YER019W	YOL040C	-740.297	-729.052	-711.166
5:190000	YER019W	YBR181C	-1048.88	-1037.52	-1008.23
5:190000	YER019W	YDL081C	-771.854	-759.662	-739.266
5:190000	YER019W	YGR152C	-448.776	-440.989	-403.383
5:190000	YER019W	YHR203C	-1383.8	-1371.77	-1331.29
5:190000	YER019W	YMR143W	-805.585	-793.804	-784.142
5:190000	YER019W	YGL135W	-1308.9	-1299.4	-1254.27

5:190000	YER019W	YJL177W	-4.33402	5.59701	46.2444
5:190000	YER019W	YJR094W-A	-63.5213	-52.1707	-15.0644
5:190000	YER019W	YDL075W	-652.507	-640.635	-620.873
5:190000	YER019W	YHL001W	-904.819	-894.759	-878.446
5:190000	YER019W	YHR021C	-511.675	-501.626	-474.878
5:190000	YER019W	YBR191W	-258.354	-248.119	-213.974
5:190000	YER019W	YOR234C	-122.222	-117.197	-99.1087
5:190000	YER019W	YBL092W	-917.266	-905.21	-889.736
5:190000	YER019W	YJL136C	-851.681	-841.346	-828.457
5:190000	YER019W	YIL133C	-82.6529	-72.974	-34.9663
5:190000	YER019W	YFR031C-A	-929.373	-920.22	-904.691
5:190000	YER019W	YOR293W	-103.06	-91.0471	-56.7654
5:190000	YER019W	YGL030W	-1145.72	-1134.42	-1111.86
5:190000	YER019W	YMR143W	-712.306	-700.544	-689.962
5:190000	YER018C	YDR447C	-648.554	-639.917	-596.849
5:190000	YER018C	YDR447C	-279.731	-270.941	-203.508
5:190000	YER018C	YDL061C	-852.531	-843.829	-762.567
5:190000	YER018C	YDL191W	-1136.86	-1126.96	-1088.33
5:190000	YER018C	YBR084C-A	-362.149	-352.803	-314.975
5:190000	YER018C	YDR450W	-876.795	-872.953	-812.18
5:190000	YER018C	YLR029C	-1026.99	-1017.02	-970.126
5:190000	YER018C	YIL052C	-528.38	-519.455	-445.396
5:190000	YER018C	YPL131W	-815.186	-806.825	-754.586
5:190000	YER018C	YHL001W	-266.75	-259.806	-165.985
5:190000	YER018C	YJR123W	-1036.94	-1026.74	-989.485
5:190000	YER018C	YDL229W	-42.3793	-37.1073	32.0291
5:190000	YER018C	YKL180W	-515.251	-507.996	-435.139
5:190000	YER018C	YLR185W	-415.833	-405.824	-342.907
5:190000	YER018C	YGL031C	-930.189	-919.708	-891.156
5:190000	YER018C	YBL072C	-1362.09	-1359.53	-1296.24
5:190000	YER018C	YBL027W	-461.198	-451.344	-394.541
5:190000	YER018C	YER102W	-684.851	-678.879	-603.363
5:190000	YER018C	YGL189C	-1152.61	-1151.77	-1076.45
5:190000	YER018C	YKR057W	-301.65	-291.183	-227.021
5:190000	YER018C	YGR085C	-156.964	-147.933	-51.5892
5:190000	YER018C	YGR148C	-1072.17	-1061.71	-1000.49
5:190000	YER018C	YGR214W	-295.622	-285.268	-217.198
5:190000	YER018C	YMR142C	-785.505	-774.682	-729.928
5:190000	YER018C	YLR075W	-1751.51	-1747.94	-1695.26
5:190000	YER018C	YDR064W	-1030.11	-1021.23	-1000.89
5:190000	YER018C	YOL120C	-555.199	-546.087	-497.934
5:190000	YER018C	YIL069C	167.842	176.416	279.513
5:190000	YER018C	YPR043W	-1185.35	-1176.13	-1141.02
5:190000	YER018C	YOL127W	-1154.04	-1145.2	-1097.27
5:190000	YER018C	YEL054C	197.026	197.426	342.718
5:190000	YER018C	YML026C	-319.643	-309.574	-247.479
5:190000	YER018C	YMR242C	-444.518	-436.395	-365.489

5:190000	YER018C	YMR116C	-1116.09	-1112.54	-1074.58
5:190000	YER018C	YDR418W	-715.425	-706.711	-638.481
5:190000	YER018C	YBR048W	-167.114	-160.512	-129.818
5:190000	YER018C	YJL189W	-571.333	-561.766	-523.236
5:190000	YER018C	YLR388W	-155.65	-148.272	-59.5008
5:190000	YER018C	YML063W	-697.151	-687.996	-654.217
5:190000	YER018C	YLL039C	-628.844	-622.704	-624.33
5:190000	YER018C	YLR388W	-249.865	-241.505	-163.41
5:190000	YER018C	YKR094C	-526.877	-518.112	-468.98
5:190000	YER018C	YLR344W	-232.344	-222.763	-128.676
5:190000	YER018C	YLR048W	-102.063	-94.3512	-3.8039
5:190000	YER018C	YBL072C	-835.061	-829.747	-792.39
5:190000	YER018C	YDR012W	-294.799	-285.334	-259.349
5:190000	YER018C	YOR312C	-594.396	-584.931	-539.932
5:190000	YER018C	YNL178W	-752.086	-740.865	-708.518
5:190000	YER018C	YIL018W	-289.565	-281.677	-216.569
5:190000	YER018C	YGR034W	-307.077	-301.313	-231.136
5:190000	YER018C	YDR471W	102.476	113.458	195.296
5:190000	YER018C	YDL130W	-723.254	-712.68	-649.445
5:190000	YER018C	YDL061C	-896.713	-885.867	-826.338
5:190000	YER018C	YHL033C	-392.575	-381.108	-344.725
5:190000	YER018C	YDL083C	-15.5172	-7.30988	71.4212
5:190000	YER018C	YLR448W	-215.589	-209.816	-117.462
5:190000	YER018C	YML024W	-950.739	-946.947	-910.538
5:190000	YER018C	YOR167C	-645.083	-634.909	-576.628
5:190000	YER018C	YPL143W	-869.053	-859.65	-817.676
5:190000	YER018C	YPL249C-A	-893.79	-883.977	-856.057
5:190000	YER018C	YMR194W	-259.398	-255.02	-142.428
5:190000	YER018C	YDR025W	-126.845	-121.423	-11.8456
5:190000	YER018C	YER131W	219.958	221.096	339.731
5:190000	YER018C	YJL191W	29.3347	41.5583	124.483
5:190000	YER018C	YLR325C	-274.974	-265.138	-199.35
5:190000	YER018C	YDR500C	-295.273	-288.408	-207.292
5:190000	YER018C	YPL079W	-482.001	-473.393	-390.544
5:190000	YER018C	YLR061W	-297.832	-291.006	-196.721
5:190000	YER018C	YHR141C	-910.228	-899.253	-849.295
5:190000	YER018C	YLR293C	-1334.82	-1327.48	-1258.48
5:190000	YER018C	YLR333C	-606.402	-601.028	-486.84
5:190000	YER018C	YJL190C	-187.202	-177.613	-116.144
5:190000	YER018C	YBR189W	-395.978	-385.088	-330.655
5:190000	YER018C	YNL302C	-464.987	-457.344	-415.124
5:190000	YER018C	YLR264W	-334.709	-323.578	-296.896
5:190000	YER018C	YPR132W	-1063.8	-1054.37	-1000.96
5:190000	YER018C	YNL096C	-184.837	-180.25	-73.054
5:190000	YER018C	YKL006W	-569.628	-561.582	-505.835
5:190000	YER018C	YLR372W	-266.127	-253.884	-228.72
5:190000	YER018C	YER074W	-499.667	-488.475	-437.645

5:190000	YER018C	YFR031C-A	-246.874	-235.777	-178.537
5:190000	YER018C	YHL033C	-475.1	-470.681	-449.215
5:190000	YER018C	YER056C-A	-307.448	-301.425	-219.4
5:190000	YER018C	YMR230W	-260.535	-252.035	-183.385
5:190000	YER018C	YGL147C	-381.812	-376.992	-333.462
5:190000	YER018C	YNL302C	-922.352	-910.659	-869.881
5:190000	YER018C	YML073C	-395.613	-389.362	-302.885
5:190000	YER018C	YBL087C	-772.611	-762.341	-701.091
5:190000	YER018C	YOL121C	-9.27535	-2.39207	89.0219
5:190000	YER018C	YGL123W	-981.397	-970.532	-918.206
5:190000	YER018C	YOR312C	-704.9	-694.508	-655.137
5:190000	YER018C	YNL069C	-726.902	-720.979	-666.246
5:190000	YER018C	YHR010W	-575.714	-566.061	-512.254
5:190000	YER018C	YER074W	-679.291	-669.199	-631.713
5:190000	YER018C	YOR096W	-531.651	-523.144	-467.858
5:190000	YER018C	YLR406C	235.69	245.363	337.143
5:190000	YER018C	YOR293W	-102.973	-96.4814	-20.7166
5:190000	YER018C	YGL076C	-1216.97	-1206.14	-1171.58
5:190000	YER018C	YFR032C-A	-597.337	-587.766	-540.727
5:190000	YER018C	YER117W	-592.232	-581.217	-524.418
5:190000	YER018C	YBR191W	-755.39	-747.061	-681.317
5:190000	YER018C	YOR063W	-1176.58	-1174.88	-1156.21
5:190000	YER018C	YLR441C	-461.013	-453.325	-385.86
5:190000	YER018C	YBR031W	-1387.85	-1381.62	-1342.59
5:190000	YER018C	YLL045C	-722.261	-711.61	-673.096
5:190000	YER018C	YLR340W	-1020.3	-1012.45	-942.27
5:190000	YER018C	YNL301C	-1100.68	-1089.85	-1035.88
5:190000	YER018C	YOL040C	-779.954	-770.426	-722.937
5:190000	YER018C	YBR181C	-1085.38	-1081.33	-1021.56
5:190000	YER018C	YDL081C	-820.615	-813.507	-750.457
5:190000	YER018C	YGR152C	-496.849	-489.91	-410.909
5:190000	YER018C	YHR203C	-1420.34	-1412.92	-1350.32
5:190000	YER018C	YMR143W	-846.859	-836.676	-794.826
5:190000	YER018C	YGL135W	-1363.04	-1358.07	-1271.51
5:190000	YER018C	YJL177W	-52.2541	-43.8819	42.7584
5:190000	YER018C	YJR094W-A	-125.625	-121.314	-15.1408
5:190000	YER018C	YDL075W	-687.49	-677.446	-629.751
5:190000	YER018C	YHL001W	-956.263	-945.154	-889.782
5:190000	YER018C	YHR021C	-559.108	-551.195	-482.769
5:190000	YER018C	YBR191W	-282.123	-276.17	-217.624
5:190000	YER018C	YOR234C	-161.298	-149.53	-104.834
5:190000	YER018C	YBL092W	-950.824	-943.45	-900.763
5:190000	YER018C	YJL136C	-901.943	-890.761	-840.499
5:190000	YER018C	YIL133C	-141.591	-132.08	-34.8139
5:190000	YER018C	YFR031C-A	-971.989	-961.805	-919.634
5:190000	YER018C	YOR293W	-147.816	-141.296	-60.9883
5:190000	YER018C	YGL030W	-1189.48	-1181.94	-1127.22

5:190000	YER018C	YMR143W	-747.134	-736.8	-700.014
7:410000	YGL048C	YGL068W	218.741	228.126	236.743
7:410000	YGL048C	YGL066W	-769.94	-765.921	-765.937
7:410000	YGL048C	YGR082W	-198.538	-186.558	-133.839
7:410000	YGL048C	YJR116W	-595.732	-585.655	-591.768
7:410000	YGL048C	YGL049C	-670.617	-665.809	-625.109
7:410000	YGL048C	YDR151C	-309.381	-306.565	-308.783
7:410000	YGL048C	YGL035C	147.794	154.749	200.56
7:410000	YGL048C	YOR232W	-257.801	-248.142	-238.157
7:410000	YGL057C	YPR174C	-340.573	-338.863	-337.941
7:410000	YGL057C	YGL066W	-814.059	-804.933	-807.298
7:410000	YGL057C	YJL144W	707.128	712.524	714.479
7:410000	YGL057C	YOL002C	-422.405	-412.214	-417.09
7:410000	YGL057C	YJR116W	-616.984	-605.098	-611.201
7:410000	YGL057C	YNL077W	13.8276	23.0893	23.0352
7:410000	YGL057C	YPR158W	665.683	677.997	689.708
7:410000	YGL057C	YGL081W	181.292	191.962	206.148
7:410000	YGL057C	YGR142W	1133.48	1141.75	1136.05
7:410000	YGL057C	YGL035C	201.896	212.519	208.141
7:410000	YGL057C	YBR101C	138.381	146.196	146.181
7:410000	YGL055W	YJL196C	-739.446	-728.527	-681.399
7:410000	YGL055W	YPR174C	-346.186	-343.321	-337.941
7:410000	YGL055W	YGL066W	-811.654	-802.007	-807.298
7:410000	YGL055W	YOL002C	-422.282	-411.364	-417.09
7:410000	YGL055W	YPL240C	744.646	745.669	752.898
7:410000	YGL055W	YJR116W	-616.52	-605.458	-611.201
7:410000	YGL055W	YGL049C	-662.193	-660.892	-652.832
7:410000	YGL055W	YDR151C	-346.06	-336.33	-331.544
7:410000	YGL055W	YNL064C	427.452	433.92	433.59
7:410000	YGL055W	YFL016C	-53.6042	-52.6487	-51.3314
7:410000	YGL055W	YPR158W	681.286	687.309	689.708
7:410000	YGL055W	YGL035C	202.217	214.187	208.141
7:410000	YGL049C	YGL048C	-563.465	-552.361	-546.701
7:410000	YGL049C	YGL057C	-359.882	-352.57	-354.798
7:410000	YGL049C	YGL068W	209.08	217.712	250.902
7:410000	YGL049C	YGL055W	-470.98	-468.173	-461.217
7:410000	YGL049C	YGL066W	-813.328	-802.183	-806.773
7:410000	YGL049C	YJR045C	-682.144	-670.244	-655.23
7:410000	YGL049C	YLR259C	-860.305	-852.299	-847.828
7:410000	YGL049C	YEL065W	203.042	214.157	219.557
7:410000	YGL049C	YOR020C	-1021.48	-1011.43	-1004.9
7:410000	YGL049C	YOL002C	-422.765	-413.907	-417.958
7:410000	YGL049C	YPL240C	749.264	753.858	753.793
7:410000	YGL049C	YGR082W	-149.733	-139.483	-132.287
7:410000	YGL049C	YJR116W	-616.839	-607.27	-611.364
7:410000	YGL049C	YFL016C	-51.5831	-45.5389	-50.2046
7:410000	YGL049C	YLR216C	-447.21	-440.529	-446.484

7:410000	YGL049C	YGL035C	197.754	206.53	207.046
7:410000	YGL049C	YOR232W	-267.535	-257.961	-250.31
7:410000	YGL053W	YGL055W	-471.414	-462.256	-464.896
7:410000	YGL053W	YGL063W	-448.62	-440.64	-446.6
7:410000	YGL053W	YGL066W	-812.098	-808.216	-807.298
7:410000	YGL053W	YGR082W	-136.526	-130.17	-135.155
7:410000	YGL053W	YJR116W	-617.285	-605.224	-611.201
7:410000	YGL053W	YNL077W	22.576	25.1867	23.0352
7:410000	YGL053W	YPR158W	686.781	692.985	689.708
7:410000	YGL053W	YBR101C	145.862	149.806	146.181
7:410000	YGL045W	YJL196C	-702.307	-693.907	-681.399
7:410000	YGL045W	YPR174C	-398.655	-396.237	-337.941
7:410000	YGL045W	YGL066W	-823.009	-813.071	-807.298
7:410000	YGL045W	YOL002C	-422.288	-411.385	-417.09
7:410000	YGL045W	YJR116W	-623.371	-613.583	-611.201
7:410000	YGL045W	YGL049C	-694.155	-691.491	-652.832
7:410000	YGL045W	YDR151C	-509.665	-501.315	-331.544
7:410000	YGL045W	YGL081W	159.757	162.855	206.148
7:410000	YGL045W	YGL035C	202.16	214.265	208.141
7:410000	YGL051W	YGL055W	-469.049	-459.304	-464.896
7:410000	YGL051W	YGL066W	-811.788	-805.921	-807.298
7:410000	YGL051W	YJL144W	714.217	720.598	714.479
7:410000	YGL051W	YOR020C	-1013.51	-1007.38	-1010.82
7:410000	YGL051W	YOL002C	-422.495	-411.325	-417.09
7:410000	YGL051W	YPL240C	748.429	752.522	752.898
7:410000	YGL051W	YGR082W	-135.807	-134.021	-135.155
7:410000	YGL051W	YJR116W	-617.27	-605.262	-611.201
7:410000	YGL051W	YNL077W	19.1949	29.0887	23.0352
7:410000	YGL051W	YNL006W	-776.019	-773.516	-774.413
7:410000	YGL051W	YNL281W	-723.988	-723.556	-721.802
7:410000	YGL051W	YGL049C	-657.363	-647.5	-652.832
7:410000	YGL051W	YDR151C	-340.584	-334.779	-331.544
7:410000	YGL051W	YNL064C	428.183	439.54	433.59
7:410000	YGL051W	YNL007C	415.254	416.999	417.959
7:410000	YGL051W	YPR158W	681.387	690.963	689.708
7:410000	YGL051W	YGL081W	201.48	208.09	206.148
7:410000	YGL051W	YGR142W	1131.43	1142.08	1136.05
7:410000	YGL051W	YGL035C	201.7	209.109	208.141
7:410000	YGL051W	YBR101C	141.389	150.624	146.181
8:110000	YHR005C	YKL138C	255.286	260.107	263.809
8:110000	YHR005C	YHR014W	-340.83	-330.769	-328.911
8:110000	YHR005C	YHL010C	-277.309	-272.312	-272.071
8:110000	YHR005C	YHR152W	-496.873	-491.001	-493.232
8:110000	YHR005C	YHR046C	489.486	498.264	531.997
8:110000	YHR005C	YKL135C	-1027.69	-1022.28	-983.504
8:110000	YHR005C	YDL055C	-1152.33	-1145.6	-1151.18
8:110000	YHR005C	YML125C	-273.447	-261.72	-262.976

8:110000	YHR005C	YEL062W	-599.239	-588.599	-583.596
8:110000	YHR005C	YNR003C	80.0063	88.1432	82.6305
8:110000	YHR005C	YFR030W	-167.142	-161.422	-134.768
8:110000	YHR005C	YNR017W	-235.424	-227.52	-219.926
8:110000	YHR005C	YHR043C	-96.3894	-84.2931	-87.2916
8:110000	YHR005C	YHL018W	-22.8576	-13.9555	-13.7713
8:110000	YHR005C	YLR303W	65.7642	76.1334	82.0157
8:110000	YHR005C	YCR023C	-759.727	-749.481	-751.441
8:110000	YHR005C	YHR040W	234.385	244.536	269.094
8:110000	YHR005C	YLR364W	-204.003	-197.99	-200.4
8:110000	YHR005C	YOR076C	-682.973	-674.503	-671.089
8:110000	YHR005C	YIL009C-A	-270.769	-260.394	-257.638
8:110000	YHR005C	YHR036W	-397.659	-385.969	-384.999
8:110000	YHR005C	YMR159C	21.7733	32.8662	26.7619
8:110000	YHR005C	YGL068W	249.253	255.449	251.557
8:110000	YHR005C	YML105C	-882.401	-870.775	-874.69
8:110000	YHR005C	YMR179W	-469.533	-465.604	-465.143
8:110000	YHR005C	YMR125W	-828.745	-817.807	-823.718
8:110000	YHR005C	YML060W	-44.8368	-39.0641	-27.051
8:110000	YHR005C	YJR110W	-457.965	-450.786	-441.788
8:110000	YHR005C	YDR244W	-613.915	-603.735	-587.552
8:110000	YHR005C	YHL009C	-574.978	-567.177	-571.948
8:110000	YHR005C	YAL031C	-403.062	-391.111	-352.029
8:110000	YHR005C	YHR054C	-624.534	-613.074	-618.496
8:110000	YHR005C	YDL064W	-574.504	-570.589	-568.551
8:110000	YHR005C	YDL054C	-464.082	-456.198	-450.371
8:110000	YHR005C	YGR199W	-1011.37	-1000.98	-983.71
8:110000	YHR005C	YOR147W	-631.787	-627.844	-616.098
8:110000	YHR005C	YHR199C-A	-187.858	-178.894	-184.694
8:110000	YHR005C	YGR109W-A	471.268	479.811	478.231
8:110000	YHR005C	YDR437W	-65.792	-59.3917	-65.4679
8:110000	YHR005C	YBR057C	-660.559	-650.757	-637.626
8:110000	YHR005C	YDR032C	-1102.68	-1091.6	-1095.4
8:110000	YHR005C	YOR044W	-4.29945	7.05514	1.13512
8:110000	YHR005C	YOR376W-A	374.2	384.197	413.016
8:110000	YHR005C	YJR010W	263.262	275.185	293.089
8:110000	YHR005C	YGR082W	-137.634	-131.314	-132.048
8:110000	YHR005C	YHR043C	314.256	326.499	322.208
8:110000	YHR005C	YER042W	-210.241	-198.106	-204.214
8:110000	YHR005C	YJR021C	-264.399	-253.805	-246.836
8:110000	YHR005C	YGR055W	614.67	618.081	620.516
8:110000	YHR005C	YNR032W	-623.188	-620.302	-563.913
8:110000	YHR005C	YMR307W	-1249.06	-1241.9	-1243.04
8:110000	YHR005C	YJR072C	-105.742	-93.6884	-97.506
8:110000	YHR005C	YNL273W	-408.555	-403.353	-408.167
8:110000	YHR005C	YDL142C	-480.499	-468.292	-455.009
8:110000	YHR005C	YHL026C	-113.481	-108.404	-85.7732

8:110000	YHR005C	YKL001C	9.25063	19.6588	13.5236
8:110000	YHR005C	YIL037C	54.3903	58.3117	56.3452
8:110000	YHR005C	YDR121W	-131.951	-121.372	-127.526
8:110000	YHR005C	YDL120W	-676.839	-666.059	-670.343
8:110000	YHR005C	YER091C	481.112	485.536	511.648
8:110000	YHR005C	YIL001W	-397.022	-392.241	-376.427
8:110000	YHR005C	YDL150W	359.364	371.2	368.728
8:110000	YHR005C	YJL146W	-664.544	-658.523	-628.375
8:110000	YHR005C	YBR115C	-31.93	-20.229	-19.1778
8:110000	YHR005C	YNL072W	-182.6	-176.185	-177.591
8:110000	YHR005C	YHL019C	-640.481	-628.521	-615.883
8:110000	YHR005C	YML092C	-600.803	-592.197	-597.62
8:110000	YHR005C	YHR127W	-267.648	-260.984	-264.028
8:110000	YHR005C	YNL279W	360.559	363.096	373.978
8:110000	YHR005C	YHR015W	58.0121	63.8379	82.6007
8:110000	YHR005C	YHL008C	333.087	338.068	382.313
8:110000	YHR005C	YHR033W	686.174	697.962	692.319
8:110000	YHR005C	YMR198W	-288.097	-281.099	-109.269
8:110000	YHR005C	YNL230C	-465.26	-455.561	-457.777
8:110000	YHR005C	YHR009C	-751.424	-739.283	-743.71
8:110000	YHR005C	YLL022C	-521.938	-514.356	-489.023
8:110000	YHR005C	YCL010C	-320.868	-317.051	-296.353
8:110000	YHR005C	YGR006W	-312.722	-304.301	-299.929
8:110000	YHR005C	YPL098C	-384.7	-379.933	-382.345
8:110000	YHR005C	YNL064C	418.462	421.364	447.644
8:110000	YHR005C	YHR021W-A	465.023	473.301	517.123
8:110000	YHR005C	YER039C	-45.9825	-35.0921	-27.9196
8:110000	YHR005C	YPL055C	-536.212	-525.343	-529.786
8:110000	YHR005C	YDR399W	614.936	621.129	615.271
8:110000	YHR005C	YKR030W	-795.482	-783.872	-788.476
8:110000	YHR005C	YCL014W	-465.52	-465.222	-444.816
8:110000	YHR005C	YDR350C	-423.257	-414.298	-402.843
8:110000	YHR005C	YML068W	-298.137	-286.415	-286.952
8:110000	YHR005C	YER112W	-328.876	-321.428	-311.4
8:110000	YHR005C	YLL062C	304.297	309.496	317.436
8:110000	YHR005C	YDR177W	-394.587	-390.042	-378.845
8:110000	YHR005C	YOR286W	-354.737	-343.164	-344.258
8:110000	YHR005C	YHR105W	-44.8243	-34.8195	-37.7429
8:110000	YHR005C	YHR052W	955.748	963.83	959.429
8:110000	YHR005C	YER155C	-1029.68	-1023.44	-988.722
8:110000	YHR005C	YHR028C	-358.174	-349.956	-350.162
8:110000	YHR005C	YFR052W	-357.128	-354.846	-339.674
8:110000	YHR005C	YCR009C	-854.658	-851.96	-840.009
8:110000	YHR005C	YDR410C	-598.212	-591.02	-584.212
8:110000	YHR005C	YGL184C	1156.96	1157.18	1207
8:110000	YHR005C	YHR029C	283.091	288.894	302.797
8:110000	YHR005C	YHR107C	-603.288	-598.633	-575.358

8:110000	YHR005C	YNL328C	15.6829	24.1045	18.0203
8:110000	YHR005C	YGL248W	-132.165	-121.377	-95.8494
8:110000	YHR005C	YCR046C	-68.2298	-57.8273	-56.4113
8:110000	YHR005C	YPL191C	-318.885	-306.945	-301.202
8:110000	YHR005C	YHR016C	-53.6943	-42.6475	-48.5526
8:110000	YHR005C	YHR022C	769.024	775.321	805.502
8:110000	YHR005C	YPL166W	211.38	222.224	226.083
8:110000	YHR005C	YHL013C	-292.44	-285.951	-277.542
8:110000	YHR005C	YOR064C	-533.968	-527.283	-528.898
8:110000	YHR005C	YBR264C	-673.327	-668.751	-641.138
8:110000	YHR005C	YOL007C	-236.207	-228.797	-200.267
8:110000	YHR005C	YKL054C	-340.721	-330.816	-333.369
8:110000	YHR005C	YNL289W	-184.318	-176.857	-142.666
8:110000	YHR005C	YPL156C	-0.849266	8.47788	22.0112
8:110000	YHR005C	YNL258C	-971.386	-965.286	-864.279
8:110000	YHR005C	YDR085C	-30.1344	-17.8273	18.9202
8:110000	YHR005C	YDR420W	-341.033	-330.921	-303.931
8:110000	YHR005C	YNR044W	188.043	191.011	212.968
8:110000	YHR005C	YLR390W	-14.837	-6.26774	-9.26171
8:110000	YHR005C	YIL021W	-863.113	-851.26	-848.696
8:110000	YHR005C	YGL210W	-527.076	-514.928	-517.753
8:110000	YHR005C	YJR086W	-108.471	-101.996	-57.3493
8:110000	YHR005C	YOR232W	-250.53	-242.807	-246.497
8:110000	YHR005C	YHR035W	-258.494	-246.81	-245.385
8:110000	YHR005C	YPL120W	-700.039	-688.111	-683.676
8:110000	YHR005C	YNL159C	-446.322	-440.701	-426.717
8:110000	YHR005C	YER039C-A	-67.094	-57.9131	-43.1394
8:110000	YHR005C	YLR350W	-56.1193	-48.2441	26.2562
8:110000	YHR005C	YHR032W	129.881	140.425	136.718
8:110000	YHR005C	YNL158W	-462.548	-456.518	-453.397
8:110000	YHL006C	YMR076C	-474.927	-471.149	-389.482
8:110000	YHL006C	YAL003W	-769.792	-759.005	-682.708
8:110000	YHL006C	YPL127C	-694.815	-684.504	-555.284
8:110000	YHL006C	YLR212C	-616.306	-606.074	-564.143
8:110000	YHL006C	YNR017W	-229.893	-226.599	-220.379
8:110000	YHL006C	YHL018W	-18.577	-13.5775	-12.6941
8:110000	YHL006C	YCR023C	-755.303	-748.053	-752.958
8:110000	YHL006C	YLL038C	4.25921	6.94963	175.813
8:110000	YHL006C	YHR040W	261.632	262.983	268.186
8:110000	YHL006C	YOR076C	-674.602	-665.018	-671.096
8:110000	YHL006C	YNL233W	-840.554	-831.649	-682.448
8:110000	YHL006C	YIL009C-A	-329.373	-321.286	-257.571
8:110000	YHL006C	YLR237W	119.558	131.596	435.966
8:110000	YHL006C	YHR036W	-402.35	-396.255	-385.29
8:110000	YHL006C	YPL153C	-286.818	-278.026	-63.5462
8:110000	YHL006C	YLR363C	-389.185	-384.762	-244.182
8:110000	YHL006C	YMR159C	19.838	26.2819	26.3528

8:110000	YHL006C	YGL068W	64.6087	68.3056	250.966
8:110000	YHL006C	YKL165C	-511.64	-502.895	-396.291
8:110000	YHL006C	YML105C	-878.544	-870.407	-875.744
8:110000	YHL006C	YLR234W	97.099	106.389	184.134
8:110000	YHL006C	YMR125W	-832.756	-821.195	-823.985
8:110000	YHL006C	YBL016W	-297.114	-285.627	2.07066
8:110000	YHL006C	YJR110W	-442.271	-435.442	-441.589
8:110000	YHL006C	YBR040W	164.796	172.102	239.815
8:110000	YHL006C	YDR244W	-601.795	-600.486	-587.694
8:110000	YHL006C	YPR122W	-103.529	-92.6928	-65.2657
8:110000	YHL006C	YHL009C	-574.244	-565.395	-571.502
8:110000	YHL006C	YHR054C	-628.312	-617.465	-618.5
8:110000	YHL006C	YDL064W	-596.736	-584.473	-568.841
8:110000	YHL006C	YGR068C	-267.09	-258.308	-3.9277
8:110000	YHL006C	YPL192C	111.002	115.099	179.738
8:110000	YHL006C	YDL054C	-459.103	-448.309	-450.081
8:110000	YHL006C	YGR146C	56.3349	60.194	406.663
8:110000	YHL006C	YOR147W	-667.189	-656.381	-615.896
8:110000	YHL006C	YHR199C-A	-195.933	-185.615	-185.02
8:110000	YHL006C	YMR236W	-1033.4	-1026.8	-873.352
8:110000	YHL006C	YLL002W	-516.322	-505.628	-328.322
8:110000	YHL006C	YGR109W-A	476.23	484.735	479.9
8:110000	YHL006C	YDR437W	-96.7034	-87.202	-66.1436
8:110000	YHL006C	YMR144W	-474.288	-471.445	-430.166
8:110000	YHL006C	YDR032C	-1101.26	-1089.41	-1095.35
8:110000	YHL006C	YOR044W	-38.0107	-26.1748	1.10358
8:110000	YHL006C	YOR376W-A	406.585	409.408	412.459
8:110000	YHL006C	YGR082W	-173.246	-169.276	-132.418
8:110000	YHL006C	YCL056C	-555.544	-547.84	-498.36
8:110000	YHL006C	YHR034C	-568.973	-562.348	-567.487
8:110000	YHL006C	YER042W	-250.604	-249.717	-204.088
8:110000	YHL006C	YDL127W	-38.7086	-30.827	94.7769
8:110000	YHL006C	YAL012W	-782.439	-771.305	-763.147
8:110000	YHL006C	YJR021C	-268.379	-264.18	-247.489
8:110000	YHL006C	YGR055W	599.485	610.701	619.662
8:110000	YHL006C	YPL124W	-463.373	-451.052	-309.133
8:110000	YHL006C	YJR072C	-102.106	-94.3468	-97.9108
8:110000	YHL006C	YNL273W	-442.511	-438.765	-408.575
8:110000	YHL006C	YHL026C	-91.1254	-81.2378	-85.7134
8:110000	YHL006C	YKL001C	9.36616	19.2311	13.3234
8:110000	YHL006C	YNL201C	-461.499	-459.926	-429.197
8:110000	YHL006C	YBR088C	-332.734	-328.107	-286.375
8:110000	YHL006C	YIL037C	38.8832	45.0916	56.995
8:110000	YHL006C	YDL120W	-691.029	-680.422	-670.271
8:110000	YHL006C	YIL001W	-383.68	-372.333	-376.275
8:110000	YHL006C	YLR183C	-186.253	-180.162	-85.6501
8:110000	YHL006C	YJL146W	-661.005	-659.794	-628.811

8:110000	YHL006C	YBR115C	-38.0123	-29.5302	-19.4919
8:110000	YHL006C	YMR288W	-465.288	-458.027	-252.016
8:110000	YHL006C	YCR089W	-245.388	-235.247	0.44638
8:110000	YHL006C	YHL019C	-620.992	-611.853	-616.901
8:110000	YHL006C	YHR127W	-315.204	-308.545	-263.314
8:110000	YHL006C	YNL279W	179.344	180.783	374.538
8:110000	YHL006C	YBL052C	-733.758	-729.337	-697.347
8:110000	YHL006C	YHR015W	-36.9105	-31.1007	83.2859
8:110000	YHL006C	YGL116W	-384.143	-374.068	-115.354
8:110000	YHL006C	YNL326C	-745.388	-744.662	-717.023
8:110000	YHL006C	YHR033W	686.304	697.161	693.291
8:110000	YHL006C	YDL003W	-211.926	-206.895	-125.466
8:110000	YHL006C	YNL230C	-510.611	-504.635	-457.818
8:110000	YHL006C	YHR009C	-759.298	-747.439	-743.307
8:110000	YHL006C	YCL010C	-407.583	-402.545	-296.565
8:110000	YHL006C	YPL267W	-279.713	-271.984	-4.08581
8:110000	YHL006C	YGR006W	-326.599	-315.935	-299.702
8:110000	YHL006C	YMR232W	105.753	107.798	229.248
8:110000	YHL006C	YNL064C	424.766	434.743	447.464
8:110000	YHL006C	YHR061C	-582.258	-581.723	-577.184
8:110000	YHL006C	YER039C	-32.9424	-23.3157	-28.605
8:110000	YHL006C	YPL055C	-580.012	-572.246	-529.996
8:110000	YHL006C	YKR030W	-820.03	-810.666	-788.458
8:110000	YHL006C	YCL014W	-527.601	-525.14	-445.132
8:110000	YHL006C	YPR133W-A	-381.367	-375.211	-334.185
8:110000	YHL006C	YDR350C	-415.921	-404.717	-403.191
8:110000	YHL006C	YML068W	-292.778	-281.272	-287.325
8:110000	YHL006C	YPR018W	-475.859	-466.438	-452.463
8:110000	YHL006C	YHR027C	-919.372	-912.463	-608.212
8:110000	YHL006C	YAL037W	143.244	149.014	420.355
8:110000	YHL006C	YLL062C	274.413	280.707	317.606
8:110000	YHL006C	YHR084W	-258.301	-248.681	8.41955
8:110000	YHL006C	YOR286W	-392.627	-381.766	-344.343
8:110000	YHL006C	YHR105W	-50.7668	-39.5725	-37.5168
8:110000	YHL006C	YOR212W	-755.379	-751.672	-615.255
8:110000	YHL006C	YGL060W	-733.389	-728.771	-633.591
8:110000	YHL006C	YPR120C	-229.606	-218.481	-139.037
8:110000	YHL006C	YGR109C	15.8276	26.2231	146.09
8:110000	YHL006C	YHR028C	-356.244	-347.524	-350.381
8:110000	YHL006C	YDR410C	-584.772	-582.519	-581.716
8:110000	YHL006C	YML047C	317.345	327.952	457.784
8:110000	YHL006C	YHR029C	263.678	265.648	303.066
8:110000	YHL006C	YNL278W	-284.143	-277.415	-33.1233
8:110000	YHL006C	YNL328C	12.0326	16.7304	17.3971
8:110000	YHL006C	YBL002W	-779.686	-771.919	-767.621
8:110000	YHL006C	YJL170C	151.98	159.834	154.934
8:110000	YHL006C	YCR046C	-122.013	-112.069	-56.6903

8:110000	YHL006C	YPL191C	-303.955	-303.246	-301.86
8:110000	YHL006C	YGL106W	-1209.66	-1202.3	-1075.16
8:110000	YHL006C	YNL078W	-471.863	-469.286	-461.326
8:110000	YHL006C	YHR031C	-311.538	-304.136	-208.244
8:110000	YHL006C	YPL166W	219.989	231.004	225.314
8:110000	YHL006C	YOR064C	-543.029	-531.057	-528.986
8:110000	YHL006C	YLR452C	-258.803	-247.647	123.99
8:110000	YHL006C	YKL054C	-345.331	-333.396	-333.398
8:110000	YHL006C	YGR047C	-733.88	-733.079	-722.637
8:110000	YHL006C	YCL055W	-119.438	-113.806	-4.91047
8:110000	YHL006C	YPL156C	11.3413	15.7685	21.5744
8:110000	YHL006C	YLR180W	806.481	815.037	810.12
8:110000	YHL006C	YKR024C	689.907	701.262	957.966
8:110000	YHL006C	YNR044W	-26.0276	-17.2439	215.617
8:110000	YHL006C	YIL021W	-852.133	-843.376	-849.539
8:110000	YHL006C	YGL210W	-534.709	-522.495	-517.722
8:110000	YHL006C	YOR232W	-350.309	-344.998	-246.648
8:110000	YHL006C	YGR152C	-478.079	-466.578	-418.474
8:110000	YHL006C	YLR315W	-201.403	-192.24	-161.699
8:110000	YHL006C	YBR071W	-328.724	-319.33	-241.711
8:110000	YHL006C	YHR035W	-257.729	-249.322	-245.793
8:110000	YHL006C	YNL159C	-445.599	-437.486	-426.705
8:110000	YHL006C	YER039C-A	-55.9604	-50.1177	-43.678
8:110000	YHL006C	YEL042W	-521.043	-510.332	-514.002
8:110000	YHL006C	YNL158W	-536.344	-531.838	-454.083
8:110000	YHL006C	YNL312W	-757.457	-747.376	-504.264
8:110000	YHL008C	YKL138C	121.693	121.9	260.957
8:110000	YHL008C	YAL003W	-675.057	-663.428	-669.479
8:110000	YHL008C	YLR212C	-565.663	-559.625	-553.842
8:110000	YHL008C	YHR046C	478.066	489.432	531.918
8:110000	YHL008C	YDL055C	-1171.65	-1165.82	-1136.83
8:110000	YHL008C	YML125C	-300.71	-290.085	-255.61
8:110000	YHL008C	YGL048C	-680.48	-673.275	-533.781
8:110000	YHL008C	YEL062W	-596.25	-584.116	-574.455
8:110000	YHL008C	YNR003C	7.05893	16.6475	87.7693
8:110000	YHL008C	YFR030W	-178.64	-169.717	-130.868
8:110000	YHL008C	YHR043C	-132.118	-119.933	-85.287
8:110000	YHL008C	YHL018W	-17.2117	-9.70056	-15.673
8:110000	YHL008C	YLR303W	8.29986	19.4903	82.3532
8:110000	YHL008C	YCR023C	-752.273	-742.955	-741.687
8:110000	YHL008C	YLL038C	144.594	154.568	174.223
8:110000	YHL008C	YHR040W	235.518	247.249	272.089
8:110000	YHL008C	YLR364W	-202.955	-194.614	-197.995
8:110000	YHL008C	YOR076C	-672.516	-661.655	-658.912
8:110000	YHL008C	YIL009C-A	-263.285	-252.686	-251.921
8:110000	YHL008C	YHR036W	-384.118	-372.585	-377.579
8:110000	YHL008C	YPL153C	-72.5108	-71.8548	-56.4723

8:110000	YHL008C	YLR363C	-246.478	-236.501	-240.686
8:110000	YHL008C	YMR159C	17.8496	29.9709	32.2945
8:110000	YHL008C	YGL068W	140.45	146.781	252.794
8:110000	YHL008C	YKL165C	-400.974	-398.43	-384.193
8:110000	YHL008C	YLR103C	-235.859	-230.036	-230.095
8:110000	YHL008C	YML105C	-868.765	-858.011	-864.013
8:110000	YHL008C	YHL006C	-158.334	-152.807	-136.429
8:110000	YHL008C	YMR125W	-824.793	-812.499	-816.941
8:110000	YHL008C	YJR110W	-468.702	-465.369	-435.373
8:110000	YHL008C	YDR244W	-579.173	-569.34	-575.267
8:110000	YHL008C	YHL009C	-568.873	-562.62	-564.898
8:110000	YHL008C	YHR054C	-626.053	-616.086	-612.083
8:110000	YHL008C	YDL064W	-565.421	-553.335	-557.947
8:110000	YHL008C	YDL054C	-455.792	-444.928	-444.627
8:110000	YHL008C	YOR147W	-612.56	-601.403	-607.298
8:110000	YHL008C	YHR199C-A	-188.261	-177.583	-183.662
8:110000	YHL008C	YMR236W	-861.054	-853.762	-859.819
8:110000	YHL008C	YLL002W	-324.424	-313.699	-319.658
8:110000	YHL008C	YGR109W-A	456.636	462.114	458.062
8:110000	YHL008C	YGR227W	-798.965	-797.755	-786.076
8:110000	YHL008C	YDR437W	-63.8434	-55.7468	-61.2917
8:110000	YHL008C	YPR103W	-386.572	-383.833	-300.819
8:110000	YHL008C	YDR032C	-1115.41	-1105.29	-1085.74
8:110000	YHL008C	YOR044W	-5.68367	6.33252	4.78073
8:110000	YHL008C	YOR376W-A	410.795	420.738	414.599
8:110000	YHL008C	YJR010W	271.27	279.536	297.403
8:110000	YHL008C	YHR034C	-568.427	-559.586	-558.451
8:110000	YHL008C	YHR043C	274.465	285.757	318.451
8:110000	YHL008C	YER042W	-205.46	-193.837	-199.953
8:110000	YHL008C	YAL012W	-771.332	-762.836	-748.366
8:110000	YHL008C	YJR021C	-251.693	-239.454	-245.067
8:110000	YHL008C	YGR055W	602.863	613.958	618.215
8:110000	YHL008C	YPL124W	-308.297	-301.608	-301.154
8:110000	YHL008C	YMR307W	-1259.97	-1255.99	-1224.06
8:110000	YHL008C	YJR072C	-142.335	-131.837	-92.6305
8:110000	YHL008C	YDL142C	-445.887	-436.938	-442.005
8:110000	YHL008C	YHL026C	-88.2216	-76.7996	-81.5768
8:110000	YHL008C	YKL001C	6.32675	17.138	18.0652
8:110000	YHL008C	YBR088C	-274.027	-273.599	-271.955
8:110000	YHL008C	YIL037C	55.2846	65.5259	60.8248
8:110000	YHL008C	YDR121W	-148.038	-135.775	-122.608
8:110000	YHL008C	YDL120W	-667.001	-655.601	-658.617
8:110000	YHL008C	YER091C	463.154	473.979	502.06
8:110000	YHL008C	YIL001W	-390.291	-378.719	-374.981
8:110000	YHL008C	YDL150W	323.181	333.043	368.066
8:110000	YHL008C	YJL146W	-623.938	-611.711	-617.764
8:110000	YHL008C	YBR115C	-31.7149	-19.8514	-24.484

8:110000	YHL008C	YHL019C	-612.908	-604.91	-608.183
8:110000	YHL008C	YML092C	-692.847	-686.474	-587.42
8:110000	YHL008C	YNL279W	360.857	366.285	375.205
8:110000	YHL008C	YHR015W	57.8031	70.0881	82.7148
8:110000	YHL008C	YGL116W	-111.13	-104.817	-107.141
8:110000	YHL008C	YHR033W	660.173	670.888	669.487
8:110000	YHL008C	YHR007C	-844.185	-841.418	-803.626
8:110000	YHL008C	YNL230C	-479.417	-467.164	-457.085
8:110000	YHL008C	YHR009C	-774.951	-765.652	-734.735
8:110000	YHL008C	YLR413W	355.227	359.996	364.541
8:110000	YHL008C	YLL022C	-483.301	-474.564	-480.027
8:110000	YHL008C	YCL010C	-316.312	-304.133	-295.988
8:110000	YHL008C	YGR006W	-307.868	-296.51	-298.701
8:110000	YHL008C	YNL064C	417.083	426.344	444.409
8:110000	YHL008C	YOL012C	-571.465	-560.829	-558.882
8:110000	YHL008C	YHR042W	-1029.38	-1027.16	-977.932
8:110000	YHL008C	YPL163C	-501.31	-500.629	-495.572
8:110000	YHL008C	YHR021W-A	490.636	500.354	514.56
8:110000	YHL008C	YER039C	-36.1565	-24.6192	-29.0366
8:110000	YHL008C	YPL055C	-534.999	-522.935	-520.56
8:110000	YHL008C	YDR399W	557.081	562.118	614.781
8:110000	YHL008C	YKR030W	-793.633	-782.24	-780.115
8:110000	YHL008C	YLR372W	-297.843	-290.333	-225.789
8:110000	YHL008C	YCL014W	-442.215	-430.079	-436.208
8:110000	YHL008C	YPR133W-A	-390.673	-385.814	-326.396
8:110000	YHL008C	YDR350C	-403.137	-391.592	-397.711
8:110000	YHL008C	YML068W	-289.285	-279.558	-280.271
8:110000	YHL008C	YER112W	-375.661	-364.087	-310.145
8:110000	YHL008C	YPR018W	-455.642	-448.821	-445.184
8:110000	YHL008C	YHR027C	-676.939	-676.791	-595.974
8:110000	YHL008C	YLL062C	293.837	305.026	317.074
8:110000	YHL008C	YDR177W	-374.878	-365.361	-370.373
8:110000	YHL008C	YOR286W	-431.444	-426.459	-338.288
8:110000	YHL008C	YHR105W	-60.0948	-48.4184	-39.7933
8:110000	YHL008C	YHR052W	899.665	911.511	954.143
8:110000	YHL008C	YPR120C	-152.402	-149.15	-128.561
8:110000	YHL008C	YHR028C	-405.369	-396.884	-344.891
8:110000	YHL008C	YFR052W	-451.218	-440.239	-335.213
8:110000	YHL008C	YML047C	443.357	444.557	447.291
8:110000	YHL008C	YGL184C	1173.62	1185.2	1197.59
8:110000	YHL008C	YHR029C	248.698	255.975	299.222
8:110000	YHL008C	YNL278W	-64.6544	-60.4887	-28.7762
8:110000	YHL008C	YHR107C	-580.815	-569.451	-568.5
8:110000	YHL008C	YNL328C	9.99002	19.5579	22.3694
8:110000	YHL008C	YBL002W	-757.756	-750.071	-754.455
8:110000	YHL008C	YJL170C	157.268	163.239	157.601
8:110000	YHL008C	YCR046C	-142.891	-132.957	-54.6766

8:110000	YHL008C	YPL191C	-308.928	-297.344	-292.802
8:110000	YHL008C	YGL106W	-1070.38	-1068.12	-1062.65
8:110000	YHL008C	YNL078W	-458.822	-451.795	-451.015
8:110000	YHL008C	YHR016C	-117.956	-107.527	-42.5422
8:110000	YHL008C	YHR031C	-228.833	-228.032	-205.213
8:110000	YHL008C	YHR022C	797.718	806.975	801.233
8:110000	YHL008C	YPL166W	212.391	223.356	221.643
8:110000	YHL008C	YOR064C	-536.382	-527.3	-522.689
8:110000	YHL008C	YKL054C	-355.835	-346.427	-327.804
8:110000	YHL008C	YLR180W	736.604	742.978	804.908
8:110000	YHL008C	YNL290W	-833.274	-833.201	-832.298
8:110000	YHL008C	YBR139W	-448.865	-439.508	-410.209
8:110000	YHL008C	YDR420W	-303.611	-292.245	-296.543
8:110000	YHL008C	YLR390W	-132.553	-122.939	-10.9003
8:110000	YHL008C	YIL021W	-839.461	-827.594	-833.454
8:110000	YHL008C	YGL210W	-525.015	-514.948	-512.1
8:110000	YHL008C	YMR314W	-342.788	-339.745	-194.46
8:110000	YHL008C	YOR232W	-301.701	-293.206	-240.879
8:110000	YHL008C	YGR152C	-409.88	-401.088	-406.453
8:110000	YHL008C	YHR035W	-249.815	-238.587	-239.89
8:110000	YHL008C	YPL120W	-698.566	-692.68	-675.73
8:110000	YHL008C	YNL159C	-426.543	-415.416	-419.524
8:110000	YHL008C	YER039C-A	-46.9751	-34.9253	-39.588
8:110000	YHL008C	YLR350W	27.1755	34.9649	28.9336
8:110000	YHL008C	YJL074C	-400.858	-399.448	-382.474
8:110000	YHL008C	YEL042W	-545.821	-536.618	-503.62
8:110000	YHL008C	YHR032W	29.0951	37.3244	135.29
8:110000	YHL008C	YNL158W	-456.983	-445.189	-446.226
8:110000	YHR007C	YKL138C	253.514	263.815	261.773
8:110000	YHR007C	YHR014W	-324.332	-317.157	-321.348
8:110000	YHR007C	YMR076C	-392.56	-390.316	-383.789
8:110000	YHR007C	YHL010C	-269.844	-266.561	-265.073
8:110000	YHR007C	YHR152W	-493.191	-488.547	-485.321
8:110000	YHR007C	YAL003W	-706.554	-695.029	-673.764
8:110000	YHR007C	YLR212C	-567.813	-559.855	-558.12
8:110000	YHR007C	YHR046C	506.918	518.857	529.872
8:110000	YHR007C	YDL055C	-1146.79	-1137.05	-1137.03
8:110000	YHR007C	YML125C	-262.803	-253.276	-259.224
8:110000	YHR007C	YGL048C	-540.26	-528.155	-532.277
8:110000	YHR007C	YML102W	-761.614	-760.403	-747.034
8:110000	YHR007C	YEL062W	-580.833	-568.881	-573.742
8:110000	YHR007C	YNR003C	76.6693	84.012	79.0796
8:110000	YHR007C	YFR030W	-143.474	-133.144	-136.478
8:110000	YHR007C	YNR017W	-220.463	-212.998	-215.707
8:110000	YHR007C	YHR043C	-90.0699	-80.0334	-82.6975
8:110000	YHR007C	YHL018W	-26.3726	-17.5025	-9.81767
8:110000	YHR007C	YLR303W	76.4151	87.4147	83.6722

8:110000	YHR007C	YCR023C	-746.488	-735.979	-741.17
8:110000	YHR007C	YLL038C	165.954	177.85	175.59
8:110000	YHR007C	YHR040W	251.153	263.179	264.918
8:110000	YHR007C	YLR364W	-204.774	-198.173	-194.359
8:110000	YHR007C	YOR076C	-673.453	-663.905	-663.156
8:110000	YHR007C	YIL009C-A	-264.869	-253.597	-257.378
8:110000	YHR007C	YHR036W	-383.043	-371.784	-377.454
8:110000	YHR007C	YPL153C	-85.3514	-77.8668	-62.6402
8:110000	YHR007C	YLR363C	-245.966	-234.416	-237.808
8:110000	YHR007C	YMR159C	16.1075	28.156	29.1072
8:110000	YHR007C	YGL068W	241.421	251.204	250.165
8:110000	YHR007C	YKL165C	-395.307	-391.816	-388.872
8:110000	YHR007C	YLR103C	-248.991	-242.933	-240.078
8:110000	YHR007C	YML105C	-866.483	-858.197	-864.325
8:110000	YHR007C	YLR234W	176.369	181.514	182.243
8:110000	YHR007C	YMR125W	-821.241	-809.292	-813.538
8:110000	YHR007C	YJR045C	-646.159	-637.504	-641.083
8:110000	YHR007C	YJR110W	-442.161	-437.545	-440.247
8:110000	YHR007C	YDR244W	-596.264	-585.549	-582.223
8:110000	YHR007C	YPR122W	-62.1152	-59.7923	-60.5244
8:110000	YHR007C	YHL009C	-567.397	-558.327	-561.881
8:110000	YHR007C	YAL031C	-347.482	-345.284	-345.311
8:110000	YHR007C	YHR054C	-615.652	-605.101	-610.44
8:110000	YHR007C	YDL064W	-570.33	-558.51	-560.802
8:110000	YHR007C	YBR067C	-320.385	-313.357	-301.538
8:110000	YHR007C	YGR068C	-75.3496	-66.1874	-1.76826
8:110000	YHR007C	YIL140W	-483.564	-479.717	-473.195
8:110000	YHR007C	YDL054C	-450.065	-438.826	-444.976
8:110000	YHR007C	YOL017W	-415.354	-412.084	-394.102
8:110000	YHR007C	YOR147W	-616.465	-604.336	-606.493
8:110000	YHR007C	YHR199C-A	-190.029	-180.415	-179.581
8:110000	YHR007C	YMR236W	-892.024	-882.467	-867.751
8:110000	YHR007C	YLL002W	-335.156	-323.357	-324.129
8:110000	YHR007C	YGR109W-A	474.495	482.755	478.094
8:110000	YHR007C	YDR437W	-71.6418	-64.2531	-70.2692
8:110000	YHR007C	YMR144W	-445.749	-436.475	-423.743
8:110000	YHR007C	YPR103W	-312.193	-300.371	-301.43
8:110000	YHR007C	YBR057C	-633.958	-630.217	-630.882
8:110000	YHR007C	YDR032C	-1117.15	-1111.71	-1088.45
8:110000	YHR007C	YOR044W	-5.74155	6.33404	0.497167
8:110000	YHR007C	YOR376W-A	396.126	407.49	412.237
8:110000	YHR007C	YGR082W	-138.29	-130.608	-131.714
8:110000	YHR007C	YCL056C	-494.525	-489.131	-489.141
8:110000	YHR007C	YHR034C	-565.872	-555.006	-558.092
8:110000	YHR007C	YHR043C	317.989	327.8	323.448
8:110000	YHR007C	YER042W	-209.968	-197.8	-203.171
8:110000	YHR007C	YAL012W	-812.855	-801.683	-752.815

8:110000	YHR007C	YJR021C	-263.893	-253.091	-240.854
8:110000	YHR007C	YGR055W	614.101	624.721	618.701
8:110000	YHR007C	YPL124W	-317.106	-306.893	-306.234
8:110000	YHR007C	YMR307W	-1240.59	-1234.72	-1231.04
8:110000	YHR007C	YJR072C	-102.642	-90.9242	-95.3781
8:110000	YHR007C	YDL142C	-463.423	-452.565	-446.474
8:110000	YHR007C	YHL026C	-90.3832	-81.0005	-85.9116
8:110000	YHR007C	YKL001C	-1.62469	7.9641	2.11987
8:110000	YHR007C	YNL201C	-440.064	-439.182	-426.021
8:110000	YHR007C	YBR088C	-284.96	-280.665	-280.921
8:110000	YHR007C	YIL037C	44.9093	54.4771	55.6269
8:110000	YHR007C	YDR121W	-139.593	-127.737	-128.969
8:110000	YHR007C	YDL120W	-668.21	-657.393	-660.028
8:110000	YHR007C	YER091C	502.666	514.47	509.092
8:110000	YHR007C	YIL001W	-375.539	-364.555	-369.487
8:110000	YHR007C	YDL150W	350.49	362.081	364.201
8:110000	YHR007C	YJL146W	-622.667	-611.683	-616.966
8:110000	YHR007C	YBR115C	-24.2678	-13.6056	-16.9157
8:110000	YHR007C	YNL072W	-173.568	-171.825	-172.77
8:110000	YHR007C	YHL019C	-614.353	-603.077	-606.478
8:110000	YHR007C	YDR507C	-562.414	-559.147	-549.999
8:110000	YHR007C	YML092C	-602.377	-590.095	-588.329
8:110000	YHR007C	YHR127W	-263.458	-259.962	-261.916
8:110000	YHR007C	YNL279W	359.94	368.866	374.285
8:110000	YHR007C	YHR015W	77.9045	88.9223	82.9001
8:110000	YHR007C	YGL116W	-121.567	-111.423	-112.321
8:110000	YHR007C	YNL326C	-708.747	-707.786	-706.776
8:110000	YHR007C	YHR033W	678.309	690.326	685.841
8:110000	YHR007C	YNL230C	-468.262	-456.388	-457.554
8:110000	YHR007C	YHR009C	-747.722	-743.181	-736.421
8:110000	YHR007C	YLL022C	-495.145	-485.012	-488.151
8:110000	YHR007C	YCL010C	-307.895	-296.455	-294.024
8:110000	YHR007C	YPL267W	-19.5312	-14.5739	-0.911493
8:110000	YHR007C	YGR006W	-313.185	-304.068	-298.805
8:110000	YHR007C	YPL098C	-381.43	-374.035	-376.326
8:110000	YHR007C	YNL064C	428.395	437.561	444.834
8:110000	YHR007C	YOL012C	-568.543	-558.112	-556.448
8:110000	YHR007C	YHR042W	-997.424	-994.268	-991.252
8:110000	YHR007C	YPL163C	-540.983	-529.36	-504.87
8:110000	YHR007C	YHR021W-A	512.836	520.513	515.135
8:110000	YHR007C	YHR061C	-579.807	-572.922	-573.232
8:110000	YHR007C	YER039C	-37.3445	-25.1424	-23.3965
8:110000	YHR007C	YPL055C	-529.837	-517.695	-521.975
8:110000	YHR007C	YDR399W	607.685	614.579	610.136
8:110000	YHR007C	YKR030W	-784.712	-772.849	-778.513
8:110000	YHR007C	YLR372W	-254.875	-244.965	-230.742
8:110000	YHR007C	YCL014W	-443.632	-431.544	-436.732

8:110000	YHR007C	YDL164C	-570.779	-564.819	-558.191
8:110000	YHR007C	YDR350C	-400.783	-390.267	-393.898
8:110000	YHR007C	YML068W	-287.739	-275.874	-280.072
8:110000	YHR007C	YER112W	-319.955	-308.371	-309.643
8:110000	YHR007C	YPR018W	-456.794	-448.962	-448.495
8:110000	YHR007C	YHR027C	-605.323	-598.499	-598.033
8:110000	YHR007C	YLL062C	303.886	316.134	313.576
8:110000	YHR007C	YDR177W	-384.759	-380.217	-375.373
8:110000	YHR007C	YOR286W	-345.809	-339.585	-341.53
8:110000	YHR007C	YHR105W	-42.4164	-31.6223	-33.3426
8:110000	YHR007C	YKL101W	-521.983	-520.715	-508.84
8:110000	YHR007C	YHR052W	940.3	952.161	949.954
8:110000	YHR007C	YER155C	-991.89	-989.916	-978.36
8:110000	YHR007C	YHR028C	-353.78	-347.17	-347.387
8:110000	YHR007C	YFR052W	-341.759	-329.547	-333.507
8:110000	YHR007C	YDR410C	-586.621	-580.281	-579.679
8:110000	YHR007C	YGL184C	1176.93	1185.08	1192.52
8:110000	YHR007C	YHR029C	295.022	306.424	300.955
8:110000	YHR007C	YNL278W	-50.4477	-42.009	-30.2388
8:110000	YHR007C	YHR107C	-578.807	-568.765	-566.97
8:110000	YHR007C	YNL328C	7.68957	18.0716	15.9583
8:110000	YHR007C	YBL002W	-770.854	-762.322	-762.684
8:110000	YHR007C	YGL248W	-96.7969	-89.3454	-93.0137
8:110000	YHR007C	YJL170C	142.239	151.611	151.651
8:110000	YHR007C	YCR046C	-63.092	-53.7736	-57.3295
8:110000	YHR007C	YPL191C	-297.109	-290.558	-296.33
8:110000	YHR007C	YGL106W	-1075.39	-1066.65	-1062.87
8:110000	YHR007C	YNL078W	-457.487	-450.104	-453.242
8:110000	YHR007C	YHR016C	-149.154	-147.969	-49.8836
8:110000	YHR007C	YHR022C	794.476	802.222	798.304
8:110000	YHR007C	YPL166W	217.252	229.364	225.726
8:110000	YHR007C	YHL013C	-278.101	-273.054	-274.138
8:110000	YHR007C	YOR064C	-530.099	-517.99	-520.221
8:110000	YHR007C	YKL054C	-340.533	-329.091	-325.042
8:110000	YHR007C	YCL055W	-27.0382	-23.5424	-6.14882
8:110000	YHR007C	YPL156C	16.3846	26.1536	26.5303
8:110000	YHR007C	YLR180W	739.281	747.069	800.649
8:110000	YHR007C	YNL290W	-842.055	-838.597	-840.411
8:110000	YHR007C	YBR139W	-432.729	-422.835	-414.206
8:110000	YHR007C	YDR420W	-316.296	-304.789	-305.731
8:110000	YHR007C	YNR044W	185.137	190.32	211.47
8:110000	YHR007C	YLR390W	-15.4641	-4.3413	-9.19938
8:110000	YHR007C	YIL021W	-853.408	-841.231	-837.466
8:110000	YHR007C	YGL210W	-522.847	-511.279	-507.886
8:110000	YHR007C	YMR314W	-201.651	-190.771	-194.977
8:110000	YHR007C	YOR232W	-254.219	-243.626	-242.866
8:110000	YHR007C	YGR152C	-430.843	-419.197	-409.948

8:110000	YHR007C	YLR315W	-157.745	-151.641	-156.981
8:110000	YHR007C	YBR071W	-283.004	-273.422	-239.307
8:110000	YHR007C	YHR035W	-251.289	-242.155	-245.886
8:110000	YHR007C	YPL120W	-701.404	-689.244	-675.342
8:110000	YHR007C	YNL159C	-429.034	-418.064	-417.952
8:110000	YHR007C	YER039C-A	-54.7091	-42.9368	-38.4374
8:110000	YHR007C	YLR350W	28.4841	35.165	29.182
8:110000	YHR007C	YEL042W	-526.642	-514.588	-507.05
8:110000	YHR007C	YHR032W	134.495	142.733	136.802
8:110000	YHR007C	YNL158W	-453.495	-441.727	-446.667
8:110000	YHR007C	YMR111C	-676.961	-672.223	-669.366
8:110000	YHR007C	YNL312W	-511.322	-508.96	-498.55
8:110000	YHR009C	YHR014W	-333.691	-329.66	-333.517
8:110000	YHR009C	YHR152W	-488.156	-478.502	-484.635
8:110000	YHR009C	YHR046C	485.264	494.628	530.953
8:110000	YHR009C	YKL135C	-972.298	-968.966	-966.71
8:110000	YHR009C	YDL055C	-1171.41	-1163.34	-1154.88
8:110000	YHR009C	YML125C	-274.713	-271.06	-272.208
8:110000	YHR009C	YML102W	-736.104	-727.953	-733.795
8:110000	YHR009C	YHR043C	-174.349	-171.744	-87.5121
8:110000	YHR009C	YLR303W	-115.561	-114.287	74.6429
8:110000	YHR009C	YCR023C	-755.129	-749.317	-752.729
8:110000	YHR009C	YHR040W	249.813	250.28	266.147
8:110000	YHR009C	YOR076C	-674.406	-666.794	-672.602
8:110000	YHR009C	YHR036W	-408.631	-396.452	-389.327
8:110000	YHR009C	YNL309W	-435.03	-426.833	-430.626
8:110000	YHR009C	YHL009C	-574.26	-565.632	-571.552
8:110000	YHR009C	YHR054C	-636.58	-629.98	-623.252
8:110000	YHR009C	YIL140W	-481.712	-473.229	-469.163
8:110000	YHR009C	YDL054C	-457.23	-450.115	-452.787
8:110000	YHR009C	YOR147W	-643.366	-642.233	-616.412
8:110000	YHR009C	YGR109W-A	477.958	487.271	483.18
8:110000	YHR009C	YGR227W	-781.154	-779.706	-780.702
8:110000	YHR009C	YHL022C	153.785	161.141	173.185
8:110000	YHR009C	YBR057C	-662.158	-650.558	-629.047
8:110000	YHR009C	YDR032C	-1111.3	-1104.62	-1106.19
8:110000	YHR009C	YOR044W	-24.3535	-23.5928	0.0114135
8:110000	YHR009C	YJR010W	188.753	197.953	272.333
8:110000	YHR009C	YHR043C	251.735	257.984	321.993
8:110000	YHR009C	YER042W	-210.037	-203.228	-209.202
8:110000	YHR009C	YMR307W	-1264.96	-1255.12	-1237.33
8:110000	YHR009C	YJR072C	-101.992	-96.1243	-100.761
8:110000	YHR009C	YKL001C	5.969	11.3433	13.0347
8:110000	YHR009C	YIL037C	46.6756	50.4235	60.349
8:110000	YHR009C	YDL120W	-684.781	-684.024	-674.052
8:110000	YHR009C	YIL001W	-387.4	-384.744	-381.395
8:110000	YHR009C	YDL150W	360.82	368.457	368.412

8:110000	YHR009C	YBR115C	-34.5256	-23.2051	-18.9004
8:110000	YHR009C	YHR015W	18.3935	27.3047	73.9866
8:110000	YHR009C	YPR115W	-254.333	-246.722	-249.153
8:110000	YHR009C	YHL008C	372.834	383.121	411.25
8:110000	YHR009C	YHR033W	687.581	698.77	692.716
8:110000	YHR009C	YMR198W	-153.202	-145.351	-98.3967
8:110000	YHR009C	YHR007C	-870.788	-860.05	-808.859
8:110000	YHR009C	YLR413W	372.324	378.395	372.911
8:110000	YHR009C	YLL022C	-496.954	-492.116	-495.359
8:110000	YHR009C	YHL009W-A	290.048	295.46	294.68
8:110000	YHR009C	YNL064C	433.333	435.948	446.36
8:110000	YHR009C	YHR042W	-1025.04	-1013.34	-987.298
8:110000	YHR009C	YHR021W-A	412.02	423.286	504.906
8:110000	YHR009C	YKR030W	-814.038	-806.76	-789.535
8:110000	YHR009C	YDR350C	-408.353	-402.325	-407.043
8:110000	YHR009C	YLL062C	310.052	316.761	315.793
8:110000	YHR009C	YHR052W	955.678	961.246	957.256
8:110000	YHR009C	YER155C	-970.982	-962.155	-968.318
8:110000	YHR009C	YHR028C	-361.606	-357.243	-359.918
8:110000	YHR009C	YCR009C	-860.887	-852.761	-808.729
8:110000	YHR009C	YDR410C	-579.111	-578.721	-574.865
8:110000	YHR009C	YHR029C	291.7	298.115	302.82
8:110000	YHR009C	YGL248W	-96.1969	-87.0335	-92.0733
8:110000	YHR009C	YJL170C	152.738	160.422	158.232
8:110000	YHR009C	YPL191C	-312.651	-307.059	-306.122
8:110000	YHR009C	YHR022C	758.919	769.962	804.517
8:110000	YHR009C	YBR264C	-664.037	-655.609	-635.083
8:110000	YHR009C	YOL007C	-207.55	-197.76	-179.754
8:110000	YHR009C	YKL054C	-342.066	-339.709	-337.055
8:110000	YHR009C	YLR180W	806.515	813.936	808.523
8:110000	YHR009C	YNL258C	-921.327	-910.94	-854.799
8:110000	YHR009C	YDR420W	-325.192	-316.867	-307.401
8:110000	YHR009C	YJR086W	-79.8506	-75.8875	-33.2633
8:110000	YHR009C	YLR315W	-164.074	-152.471	-158.347
8:110000	YHR009C	YHR035W	-257.736	-246.394	-246.468
8:110000	YHR009C	YPL120W	-705.687	-695.968	-689.129
8:110000	YHR009C	YLR350W	-17.8909	-6.4541	30.1506
8:110000	YHR009C	YHL009W-B	-48.2811	-41.4297	-31.2489
8:110000	YHR009C	YHR032W	36.1588	43.2764	128.997
8:110000	YHL003C	YHR014W	-309.026	-300.747	-303.176
8:110000	YHL003C	YHL010C	-282.747	-275.493	-272.038
8:110000	YHL003C	YHR152W	-491.118	-481.312	-479.076
8:110000	YHL003C	YAL003W	-642.622	-633.347	-638.672
8:110000	YHL003C	YHR046C	457.343	462.088	493.509
8:110000	YHL003C	YKL135C	-930.738	-929.922	-928.441
8:110000	YHL003C	YDL055C	-1109.82	-1098.95	-1081.71
8:110000	YHL003C	YML125C	-258.482	-246.456	-242.176

8:110000	YHL003C	YML102W	-717.152	-713.931	-716.755
8:110000	YHL003C	YEL062W	-580.076	-576.766	-559.036
8:110000	YHL003C	YNR003C	65.2216	76.9089	75.8074
8:110000	YHL003C	YHR043C	-99.0546	-90.8405	-80.7175
8:110000	YHL003C	YHL018W	-17.6991	-6.80453	-7.50789
8:110000	YHL003C	YLR303W	41.5067	43.4862	74.9052
8:110000	YHL003C	YCR023C	-706.429	-696.363	-700.789
8:110000	YHL003C	YLL038C	165.872	171.282	174.324
8:110000	YHL003C	YHR040W	243.812	255.981	254.293
8:110000	YHL003C	YOR076C	-642.867	-633.249	-636.142
8:110000	YHL003C	YIL009C-A	-244.401	-232.409	-238.32
8:110000	YHL003C	YHR036W	-396.442	-384.55	-384.024
8:110000	YHL003C	YLR363C	-240.556	-238.941	-232.575
8:110000	YHL003C	YMR159C	5.10005	15.9534	22.4195
8:110000	YHL003C	YML105C	-834.791	-824.817	-830.714
8:110000	YHL003C	YHL006C	-141.294	-133.576	-138.824
8:110000	YHL003C	YMR125W	-786.454	-775.682	-776.398
8:110000	YHL003C	YJR110W	-417.841	-407.859	-411.137
8:110000	YHL003C	YBR040W	233.651	238.809	237.664
8:110000	YHL003C	YDR244W	-567.968	-558.204	-556.282
8:110000	YHL003C	YHL009C	-582.88	-575.164	-580.426
8:110000	YHL003C	YER016W	-742.388	-735.824	-724.703
8:110000	YHL003C	YHR054C	-587.417	-577.049	-583.128
8:110000	YHL003C	YDL064W	-534.687	-524.151	-530.183
8:110000	YHL003C	YBR067C	-303.271	-296.542	-297.466
8:110000	YHL003C	YIL140W	-489.037	-478.13	-450.495
8:110000	YHL003C	YDL054C	-436.676	-426.109	-430.379
8:110000	YHL003C	YGR199W	-939.86	-931.549	-931.498
8:110000	YHL003C	YOR147W	-600.527	-589.805	-595.687
8:110000	YHL003C	YHR199C-A	-178.232	-169.107	-173.353
8:110000	YHL003C	YMR236W	-825.332	-819.45	-825.33
8:110000	YHL003C	YGR109W-A	437.041	447.918	445.098
8:110000	YHL003C	YDR437W	-60.2713	-54.4589	-59.6351
8:110000	YHL003C	YPR103W	-302.182	-299.671	-288.323
8:110000	YHL003C	YBR057C	-635.13	-623.004	-605.68
8:110000	YHL003C	YDR032C	-1031.44	-1020.84	-1026.14
8:110000	YHL003C	YOR044W	-27.9105	-18.9334	-19.6974
8:110000	YHL003C	YOR376W-A	375.988	386.914	392.857
8:110000	YHL003C	YJR010W	257.545	268.332	288.908
8:110000	YHL003C	YGR082W	-133.003	-127.793	-131.056
8:110000	YHL003C	YHR034C	-556.505	-545.568	-550.027
8:110000	YHL003C	YHR043C	280.834	290.496	299.874
8:110000	YHL003C	YER042W	-211.03	-199.875	-204.832
8:110000	YHL003C	YAL012W	-717.29	-706.179	-711.083
8:110000	YHL003C	YJR021C	-268.492	-260.667	-253.117
8:110000	YHL003C	YGR055W	574.223	584.872	578.774
8:110000	YHL003C	YNR032W	-579.219	-571.066	-547.424

8:110000	YHL003C	YMR307W	-1222.43	-1212.98	-1176.51
8:110000	YHL003C	YJR072C	-101.015	-89.1206	-94.3618
8:110000	YHL003C	YDL142C	-453.565	-442.529	-441.422
8:110000	YHL003C	YHL026C	-84.1983	-75.2588	-80.2573
8:110000	YHL003C	YKL001C	6.88981	18.4859	12.769
8:110000	YHL003C	YIL037C	53.318	63.2762	57.2541
8:110000	YHL003C	YDR121W	-134.646	-124.55	-116.942
8:110000	YHL003C	YDL120W	-640.037	-629.848	-633.251
8:110000	YHL003C	YIL001W	-361.341	-349.378	-355.457
8:110000	YHL003C	YDL150W	329.267	336.974	345.06
8:110000	YHL003C	YJL146W	-616.079	-604.821	-606.523
8:110000	YHL003C	YBR115C	-18.4832	-6.90571	-8.41998
8:110000	YHL003C	YHL019C	-596.72	-589.72	-593.8
8:110000	YHL003C	YDR507C	-542.385	-532.28	-522.393
8:110000	YHL003C	YML092C	-566.904	-562.658	-563.414
8:110000	YHL003C	YHR015W	69.9607	74.3008	88.2241
8:110000	YHL003C	YGL223C	-544.918	-540.756	-544.684
8:110000	YHL003C	YHL008C	344.493	351.681	371.534
8:110000	YHL003C	YHR033W	653.162	663.916	665.418
8:110000	YHL003C	YMR198W	-119.343	-111.705	-98.67
8:110000	YHL003C	YHR007C	-775.233	-768.572	-764.405
8:110000	YHL003C	YNL230C	-439.477	-427.773	-433.638
8:110000	YHL003C	YHR009C	-706.824	-701.569	-699.209
8:110000	YHL003C	YLL022C	-482.735	-474.175	-465.817
8:110000	YHL003C	YCL010C	-285.7	-274.931	-279.467
8:110000	YHL003C	YGR006W	-298.887	-287.332	-292.43
8:110000	YHL003C	YNL064C	421.053	433.071	427.156
8:110000	YHL003C	YOL012C	-549.185	-545.962	-531.289
8:110000	YHL003C	YHR042W	-962.553	-954.641	-931.871
8:110000	YHL003C	YPL163C	-498.057	-486.478	-477.053
8:110000	YHL003C	YHR021W-A	470.44	479.644	494.357
8:110000	YHL003C	YHR061C	-560.587	-554.246	-559.047
8:110000	YHL003C	YER039C	-43.8129	-32.0588	-33.8363
8:110000	YHL003C	YPL055C	-522.452	-510.931	-516.527
8:110000	YHL003C	YDR399W	547.64	559.752	567.095
8:110000	YHL003C	YKR030W	-769.924	-758.975	-762.489
8:110000	YHL003C	YLR372W	-221.367	-212.317	-215.81
8:110000	YHL003C	YCL014W	-420.109	-410.084	-415.109
8:110000	YHL003C	YDR350C	-391.071	-384.877	-382.779
8:110000	YHL003C	YML068W	-290.111	-278.502	-281.713
8:110000	YHL003C	YER112W	-294.145	-288.34	-281.3
8:110000	YHL003C	YLL062C	294.86	306.542	300.586
8:110000	YHL003C	YOR286W	-322.727	-312.991	-319.069
8:110000	YHL003C	YHR105W	-36.6861	-26.6038	-32.1596
8:110000	YHL003C	YKL101W	-496.308	-491.454	-491.951
8:110000	YHL003C	YHR052W	882.386	892.773	897.414
8:110000	YHL003C	YER155C	-947.647	-941.566	-934.126

8:110000	YHL003C	YHR028C	-339.226	-335.381	-336.921
8:110000	YHL003C	YCR009C	-889.522	-878.816	-791.442
8:110000	YHL003C	YGL184C	1119.64	1125.14	1143.22
8:110000	YHL003C	YHR029C	280.572	291.897	286.257
8:110000	YHL003C	YHR107C	-555.669	-547.663	-539.752
8:110000	YHL003C	YNL328C	12.0839	18.5197	22.89
8:110000	YHL003C	YGL248W	-98.2428	-86.875	-84.6283
8:110000	YHL003C	YJL170C	139.387	149.48	151.879
8:110000	YHL003C	YCR046C	-50.6052	-42.6369	-45.999
8:110000	YHL003C	YPL191C	-308.175	-296.187	-293.314
8:110000	YHL003C	YNL300W	-556.587	-553.821	-542.964
8:110000	YHL003C	YHR016C	-85.2773	-83.8294	-47.3287
8:110000	YHL003C	YHR022C	754.449	765.737	763.295
8:110000	YHL003C	YPL166W	202.239	213.827	208.156
8:110000	YHL003C	YOR064C	-526.602	-516.098	-512.613
8:110000	YHL003C	YOR180C	-325.676	-320.081	-321.863
8:110000	YHL003C	YBR264C	-638.808	-637.857	-601.658
8:110000	YHL003C	YOL007C	-223.661	-213.177	-183.478
8:110000	YHL003C	YKL054C	-314.492	-302.974	-306.144
8:110000	YHL003C	YNL289W	-131.642	-126.759	-123.945
8:110000	YHL003C	YML062C	-625.535	-620.043	-620.183
8:110000	YHL003C	YLR180W	764.442	775.079	777.495
8:110000	YHL003C	YNL258C	-835.242	-826.589	-816.589
8:110000	YHL003C	YBR139W	-423.812	-416.244	-418.556
8:110000	YHL003C	YLR390W	-9.6692	-9.25539	-0.443763
8:110000	YHL003C	YIL021W	-805.425	-795.965	-801.903
8:110000	YHL003C	YGL210W	-492.247	-484.33	-483.764
8:110000	YHL003C	YJR086W	-123.355	-111.603	-47.4877
8:110000	YHL003C	YOR232W	-241.084	-231.251	-237.185
8:110000	YHL003C	YLR315W	-151.632	-149.831	-150.834
8:110000	YHL003C	YBR071W	-252.784	-244.442	-245.656
8:110000	YHL003C	YHR035W	-243.986	-232.027	-233.097
8:110000	YHL003C	YPL120W	-674.621	-665.269	-640.798
8:110000	YHL003C	YNL159C	-421.087	-417.223	-404.902
8:110000	YHL003C	YER039C-A	-65.3091	-55.6989	-49.6845
8:110000	YHL003C	YLR350W	-3.17824	8.45753	21.3468
8:110000	YHL003C	YEL042W	-498.637	-487.254	-492.444
8:110000	YHL003C	YHR032W	111.162	119.915	135.243
8:110000	YHL003C	YNL158W	-423.391	-414.453	-419.844
8:110000	YHL003C	YMR111C	-649.998	-640.924	-644.267
8:110000	YHL003C	YDR225W	-883.867	-877.914	-876.128
9:70000	YIL151C	YFL045C	-1217.04	-1216.05	-1195.46
9:70000	YIL151C	YDL191W	-1125.64	-1123.91	-1099.6
9:70000	YIL151C	YDR450W	-853.757	-844.114	-827.339
9:70000	YIL151C	YJR123W	-1022.5	-1011.47	-998.159
9:70000	YIL151C	YKL180W	-484.349	-482.027	-438.632
9:70000	YIL151C	YGL031C	-910.875	-898.671	-900.631

9:70000	YIL151C	YBL072C	-1328.47	-1319.47	-1307.76
9:70000	YIL151C	YER102W	-629.644	-622.75	-609.168
9:70000	YIL151C	YGR148C	-1021.7	-1016.7	-1009.88
9:70000	YIL151C	YGR214W	-264.064	-263.304	-215.109
9:70000	YIL151C	YMR142C	-760.939	-757.774	-737.055
9:70000	YIL151C	YOL120C	-536.49	-528.252	-503.882
9:70000	YIL151C	YOL127W	-1129.58	-1120.28	-1106.31
9:70000	YIL151C	YMR242C	-410.394	-407.326	-369.177
9:70000	YIL151C	YML063W	-683.114	-672.644	-664.251
9:70000	YIL151C	YNL178W	-729.3	-726.373	-713.727
9:70000	YIL151C	YIL018W	-261.172	-255.217	-218.512
9:70000	YIL151C	YGR034W	-267.032	-260.254	-229.806
9:70000	YIL151C	YFR055W	-240.166	-232.018	-236.598
9:70000	YIL151C	YML024W	-925.942	-915.122	-912.446
9:70000	YIL151C	YOL039W	-756.911	-754.22	-753.858
9:70000	YIL151C	YDR382W	-1125.86	-1119.65	-1113.14
9:70000	YIL151C	YIL153W	-641.441	-635.833	-626.517
9:70000	YIL151C	YIL136W	770.143	782.024	837.521
9:70000	YIL151C	YJL190C	-147.775	-145.545	-119.67
9:70000	YIL151C	YPR132W	-1023.3	-1013.59	-1009.87
9:70000	YIL151C	YKL006W	-539.765	-537.466	-514.805
9:70000	YIL151C	YER074W	-471.99	-467.835	-440.712
9:70000	YIL151C	YGL147C	-371.841	-361.323	-339.242
9:70000	YIL151C	YIL148W	-557.818	-546.464	-544.801
9:70000	YIL151C	YGL103W	-1261.08	-1260.05	-1260.88
9:70000	YIL151C	YGL123W	-958.679	-958.504	-928.098
9:70000	YIL151C	YIL130W	-164.375	-164.208	-108.382
9:70000	YIL151C	YNL069C	-696.877	-687.141	-669.715
9:70000	YIL151C	YBR072W	395.239	397.151	397.743
9:70000	YIL151C	YER074W	-645.786	-638.111	-642.289
9:70000	YIL151C	YOR096W	-497.457	-489.961	-475.352
9:70000	YIL151C	YGL076C	-1202.62	-1202.51	-1182.49
9:70000	YIL151C	YLL045C	-694.466	-691.578	-685.675
9:70000	YIL151C	YOL040C	-746.15	-740.039	-728.7
9:70000	YIL151C	YBR181C	-1060.79	-1055.25	-1036.56
9:70000	YIL151C	YHR203C	-1410.37	-1404.96	-1366.24
9:70000	YIL151C	YGL135W	-1329.5	-1325	-1284.04
9:70000	YIL151C	YHR021C	-521.235	-514.439	-480.756
9:70000	YIL151C	YBR191W	-256.811	-255.277	-227.38
9:70000	YIL151C	YFR031C-A	-946.21	-940.706	-925.008
9:70000	YIL151C	YLR167W	-1144.06	-1138.68	-1127.78
9:70000	YIL151C	YGL030W	-1160.84	-1155.31	-1136.59
9:70000	YIL142W	YFL045C	-1333.51	-1325.48	-1195.96
9:70000	YIL142W	YDL191W	-1150.92	-1148.66	-1102.17
9:70000	YIL142W	YJR123W	-1079.8	-1078.94	-1000.99
9:70000	YIL142W	YIL151C	-549.002	-541.921	-399.756
9:70000	YIL142W	YKL180W	-543.376	-537.2	-440.695

9:70000	YIL142W	YGL031C	-961.983	-959.575	-900.813
9:70000	YIL142W	YER102W	-719.089	-716.945	-612.38
9:70000	YIL142W	YGR148C	-1105.72	-1105.32	-1013.89
9:70000	YIL142W	YGR214W	-298.125	-289.336	-218.654
9:70000	YIL142W	YMR142C	-817.545	-811.202	-740.995
9:70000	YIL142W	YOL120C	-613.025	-606.969	-505.144
9:70000	YIL142W	YOL127W	-1207.98	-1206.57	-1107.34
9:70000	YIL142W	YMR242C	-484.649	-478.375	-371.312
9:70000	YIL142W	YNL178W	-770.247	-760.347	-719.065
9:70000	YIL142W	YIL018W	-324.205	-318.949	-220.623
9:70000	YIL142W	YGR034W	-348.597	-344.068	-231.414
9:70000	YIL142W	YIL164C	484.047	489.717	518.035
9:70000	YIL142W	YML024W	-991.05	-984.473	-912.68
9:70000	YIL142W	YOL039W	-817.393	-817.3	-759.441
9:70000	YIL142W	YIL146C	214.793	226.626	332.913
9:70000	YIL142W	YJL190C	-211.034	-210.95	-121.6
9:70000	YIL142W	YBR189W	-442.466	-436.288	-337.731
9:70000	YIL142W	YPR132W	-1102.5	-1094.96	-1010.22
9:70000	YIL142W	YKL006W	-609.959	-608.923	-519.076
9:70000	YIL142W	YER074W	-525.262	-517.356	-443.334
9:70000	YIL142W	YMR011W	544.993	555.893	652.589
9:70000	YIL142W	YGL147C	-403.959	-398.825	-339.322
9:70000	YIL142W	YBL087C	-769.544	-762.769	-714.883
9:70000	YIL142W	YGL123W	-1010.7	-1007.85	-935.86
9:70000	YIL142W	YNL069C	-749.58	-743.158	-670.454
9:70000	YIL142W	YGL076C	-1250.61	-1246.1	-1188.37
9:70000	YIL142W	YLL045C	-765.935	-763.155	-686.834
9:70000	YIL142W	YOL040C	-799.652	-790.295	-731.932
9:70000	YIL142W	YGL135W	-1412.22	-1411.55	-1285.28
9:70000	YIL142W	YIL165C	519.557	522.406	548.298
9:70000	YIL142W	YHR021C	-590.963	-584.594	-482.028
9:70000	YIL142W	YLR167W	-1270.94	-1269.77	-1132.63
9:70000	YIL142W	YGL030W	-1200.55	-1197.78	-1136.86
9:70000	YIL153W	YFL045C	-1227.39	-1217.16	-1197.67
9:70000	YIL153W	YDL191W	-1160.35	-1153.33	-1098.31
9:70000	YIL153W	YDR450W	-851.679	-848.583	-828.458
9:70000	YIL153W	YPL131W	-831.547	-820.661	-760.503
9:70000	YIL153W	YJR123W	-1042.14	-1032.1	-998.271
9:70000	YIL153W	YIL151C	-452.046	-447.461	-397.326
9:70000	YIL153W	YKL180W	-520.748	-509.291	-439.374
9:70000	YIL153W	YGL031C	-912.079	-903.462	-901.895
9:70000	YIL153W	YBL072C	-1359.37	-1350.02	-1307.32
9:70000	YIL153W	YER102W	-673.202	-662.334	-609.462
9:70000	YIL153W	YGR148C	-1075.81	-1068.3	-1013.29
9:70000	YIL153W	YGR214W	-299.675	-288.386	-215.807
9:70000	YIL153W	YMR142C	-800.209	-788.974	-736.165
9:70000	YIL153W	YDL229W	-505.394	-497.612	-431.039

9:70000	YIL153W	YOL120C	-554.184	-543.425	-504.458
9:70000	YIL153W	YLR249W	-433.516	-432.48	-353.023
9:70000	YIL153W	YOL127W	-1146.15	-1134.73	-1106.14
9:70000	YIL153W	YMR242C	-446.518	-434.903	-370.414
9:70000	YIL153W	YMR116C	-1145.84	-1142.6	-1089.66
9:70000	YIL153W	YML063W	-681.472	-675.004	-666.603
9:70000	YIL153W	YNL178W	-755.553	-743.775	-715.925
9:70000	YIL153W	YIL018W	-271.879	-260.799	-218.507
9:70000	YIL153W	YGR034W	-289.979	-279.257	-229.835
9:70000	YIL153W	YIL164C	433.506	443.816	519.005
9:70000	YIL153W	YFR055W	-313.82	-308.884	-238.331
9:70000	YIL153W	YML024W	-939.073	-929.665	-913.285
9:70000	YIL153W	YOL039W	-822.97	-814.206	-753.652
9:70000	YIL153W	YDR382W	-1155.98	-1149.62	-1115.61
9:70000	YIL153W	YIL146C	165.697	177.675	335.615
9:70000	YIL153W	YJL190C	-170.472	-161.485	-116.678
9:70000	YIL153W	YBR189W	-404.254	-392.022	-334.956
9:70000	YIL153W	YPR132W	-1048.4	-1037.18	-1009.67
9:70000	YIL153W	YKL006W	-579.84	-569.917	-512.856
9:70000	YIL153W	YER074W	-503.187	-491.218	-441.302
9:70000	YIL153W	YMR011W	493.136	503.962	652.174
9:70000	YIL153W	YGL147C	-354.952	-344.704	-339.536
9:70000	YIL153W	YIL148W	-585.965	-577.581	-545.013
9:70000	YIL153W	YBL087C	-788.572	-777.696	-709.334
9:70000	YIL153W	YGL123W	-984.231	-973.397	-928.358
9:70000	YIL153W	YNL069C	-733.651	-725.519	-670.427
9:70000	YIL153W	YER074W	-673.068	-663.344	-640.646
9:70000	YIL153W	YOR096W	-515.777	-506.417	-473.785
9:70000	YIL153W	YGL076C	-1250.12	-1240.21	-1183.54
9:70000	YIL153W	YLL045C	-740.623	-730.224	-685.316
9:70000	YIL153W	YOL040C	-780.407	-768.646	-729.901
9:70000	YIL153W	YBR181C	-1096.15	-1089.15	-1035.63
9:70000	YIL153W	YDL081C	-809.433	-801.594	-761.383
9:70000	YIL153W	YHR203C	-1444.27	-1434.71	-1366.3
9:70000	YIL153W	YGL135W	-1380.5	-1371.32	-1284.19
9:70000	YIL153W	YIL165C	462.416	471.465	550.459
9:70000	YIL153W	YHR021C	-546.581	-536.336	-481.77
9:70000	YIL153W	YBR191W	-282.33	-275.02	-225.505
9:70000	YIL153W	YFR031C-A	-986.815	-975.707	-924.475
9:70000	YIL153W	YLR167W	-1183.68	-1173.83	-1128.3
9:70000	YIL153W	YGL030W	-1192.71	-1186.61	-1136.6
9:70000	YIL146C	YFL045C	-1239.45	-1230.14	-1195.96
9:70000	YIL146C	YDL191W	-1118.27	-1113.86	-1102.17
9:70000	YIL146C	YDR450W	-915.845	-913.994	-828.139
9:70000	YIL146C	YJR123W	-1097.3	-1088.01	-1000.99
9:70000	YIL146C	YIL151C	-500.918	-490.839	-399.756
9:70000	YIL146C	YKL180W	-540.599	-528.939	-440.695

9:70000	YIL146C	YGL031C	-941.068	-930.732	-900.813
9:70000	YIL146C	YBL072C	-1439.01	-1432	-1310.58
9:70000	YIL146C	YER102W	-712.11	-701.996	-612.38
9:70000	YIL146C	YGR148C	-1106.43	-1099.95	-1013.89
9:70000	YIL146C	YGR214W	-281.325	-270.703	-218.654
9:70000	YIL146C	YMR142C	-814.034	-805.699	-740.995
9:70000	YIL146C	YDL229W	-561.449	-560.835	-438.605
9:70000	YIL146C	YIL134W	17.6146	20.9276	94.2827
9:70000	YIL146C	YOL120C	-620.543	-608.641	-505.144
9:70000	YIL146C	YLR249W	-432.161	-431.856	-355.8
9:70000	YIL146C	YOL127W	-1201.37	-1192.15	-1107.34
9:70000	YIL146C	YMR242C	-470.332	-458.506	-371.312
9:70000	YIL146C	YML063W	-706.332	-699.025	-666.061
9:70000	YIL146C	YNL178W	-783.909	-776.544	-719.065
9:70000	YIL146C	YIL018W	-331.476	-320.254	-220.623
9:70000	YIL146C	YGR034W	-324.358	-313.919	-231.414
9:70000	YIL146C	YIL164C	514.528	520.442	518.035
9:70000	YIL146C	YFR055W	-317.518	-309.67	-238.885
9:70000	YIL146C	YML024W	-981.623	-971.614	-912.68
9:70000	YIL146C	YOL039W	-820.054	-816.996	-759.441
9:70000	YIL146C	YDR382W	-1157.8	-1156.2	-1119.81
9:70000	YIL146C	YJL190C	-201.883	-197.06	-121.6
9:70000	YIL146C	YBR189W	-464.619	-454.329	-337.731
9:70000	YIL146C	YPR132W	-1094.52	-1083.18	-1010.22
9:70000	YIL146C	YKL006W	-607.103	-601.091	-519.076
9:70000	YIL146C	YER074W	-519.135	-508.211	-443.334
9:70000	YIL146C	YMR011W	639.021	647.392	652.589
9:70000	YIL146C	YGL147C	-379.866	-368.328	-339.322
9:70000	YIL146C	YBL087C	-751.958	-745.491	-714.883
9:70000	YIL146C	YGL123W	-1016.15	-1010.54	-935.86
9:70000	YIL146C	YNL069C	-778.615	-766.448	-670.454
9:70000	YIL146C	YER074W	-755.007	-750.746	-646.776
9:70000	YIL146C	YOR096W	-563.586	-557.597	-477.044
9:70000	YIL146C	YGL076C	-1211.34	-1207.07	-1188.37
9:70000	YIL146C	YLL045C	-781.315	-773.361	-686.834
9:70000	YIL146C	YOL040C	-816.921	-805.913	-731.932
9:70000	YIL146C	YBR181C	-1140.47	-1136.02	-1040.92
9:70000	YIL146C	YHR203C	-1476.66	-1471.06	-1370.06
9:70000	YIL146C	YGL135W	-1396.12	-1386.61	-1285.28
9:70000	YIL146C	YIL165C	544.093	548.554	548.298
9:70000	YIL146C	YHR021C	-571.258	-560.003	-482.028
9:70000	YIL146C	YBR191W	-310.789	-307.494	-231.967
9:70000	YIL146C	YFR031C-A	-996.191	-995.364	-934.62
9:70000	YIL146C	YGL030W	-1185.04	-1176.87	-1136.86
9:70000	YIL148W	YDL191W	-1096.67	-1089.27	-1095.38
9:70000	YIL148W	YJR123W	-996.257	-994.233	-990.403
9:70000	YIL148W	YKL180W	-437.701	-431.047	-435.408

9:70000	YIL148W	YGL031C	-898.951	-889.274	-891.68
9:70000	YIL148W	YGR214W	-218.952	-210.801	-216.866
9:70000	YIL148W	YOL127W	-1100.29	-1097.01	-1094.14
9:70000	YIL148W	YMR242C	-369.78	-362.474	-367.324
9:70000	YIL148W	YML063W	-669.314	-661.407	-666.14
9:70000	YIL148W	YIL018W	-217.774	-212.516	-214.343
9:70000	YIL148W	YGR034W	-229.726	-222.417	-225.746
9:70000	YIL148W	YIL164C	504.072	510.536	516.391
9:70000	YIL148W	YIL142W	-901.079	-893.885	-892.604
9:70000	YIL148W	YIL153W	-628.962	-628.495	-628.138
9:70000	YIL148W	YIL146C	333.036	339.471	333.573
9:70000	YIL148W	YIL136W	802.592	811.406	816.932
9:70000	YIL148W	YPR132W	-1012.02	-1011.62	-995.572
9:70000	YIL148W	YER074W	-439.165	-430.07	-436.148
9:70000	YIL148W	YMR011W	641.997	651.624	646.068
9:70000	YIL148W	YGL147C	-339.734	-327.824	-332.457
9:70000	YIL148W	YIL152W	-353.877	-349.209	-349.833
9:70000	YIL148W	YNL069C	-671.516	-668.223	-660.888
9:70000	YIL148W	YBR072W	366.982	372.703	376.188
9:70000	YIL148W	YDR365C	834.594	836.49	866.887
9:70000	YIL148W	YGL135W	-1273.08	-1270.38	-1268.39
9:70000	YIL148W	YIL165C	533.904	538.321	544.115
9:70000	YIL148W	YHR021C	-480.83	-472.934	-476.071
9:70000	YIL148W	YGL030W	-1124.74	-1124.61	-1120.67
9:70000	YIL152W	YDL191W	-1123.48	-1122.23	-1102.17
9:70000	YIL152W	YIL134W	82.3434	90.7878	94.2827
9:70000	YIL152W	YBL087C	-743.734	-742.979	-714.883
9:70000	YIL152W	YGL076C	-1213.6	-1212.03	-1188.37
12:670000	YLR266C	YML019W	-886.706	-877.917	-883.997
12:670000	YLR266C	YDL086W	-565.037	-552.964	-559.054
12:670000	YLR266C	YMR220W	-904.133	-894.807	-898.532
12:670000	YLR266C	YLR283W	-520.643	-514.078	-517.439
12:670000	YLR266C	YDR518W	-633.631	-629.578	-632.968
12:670000	YLR266C	YEL051W	-1031.86	-1022.57	-1024.85
12:670000	YLR266C	YLR256W	135.056	138.88	139.411
12:670000	YLR266C	YJL100W	113.233	114.429	116.418
12:670000	YLR266C	YOR003W	6.33811	17.1444	12.7218
12:670000	YLR266C	YGR266W	-856.032	-850.611	-844.578
12:670000	YLR266C	YPL189C-A	487.623	497.077	496.213
12:670000	YLR266C	YML054C	750.157	756.352	754.105
12:670000	YLR266C	YMR202W	-1278.87	-1274.38	-1278.06
12:670000	YLR266C	YLR231C	-399.939	-390.379	-393.912
12:670000	YLR266C	YDR502C	-465.965	-455.593	-458.86
12:670000	YLR266C	YLR234W	181.108	188.507	185.855
12:670000	YLR266C	YGL055W	-439.664	-433.883	-420.076
12:670000	YLR266C	YOR099W	-1044.25	-1036.32	-1036.63
12:670000	YLR266C	YGR089W	-385.081	-375.5	-372.741

12:670000	YLR266C	YDR186C	-324.849	-312.728	-313.088
12:670000	YLR266C	YEL047C	-875.203	-868.9	-847.038
12:670000	YLR266C	YLR270W	-131.093	-125.638	-131.075
12:670000	YLR266C	YDL093W	-417.959	-408.409	-409.698
12:670000	YLR266C	YJL080C	-760.325	-750.808	-756.911
12:670000	YLR266C	YBR067C	-290.754	-281.89	-284.88
12:670000	YLR266C	YHR188C	-924.929	-923.732	-914.645
12:670000	YLR266C	YPL170W	-407.761	-397.437	-402.642
12:670000	YLR266C	YCR048W	-515.918	-508.79	-511.258
12:670000	YLR266C	YGR281W	-297.818	-288.288	-293.173
12:670000	YLR266C	YLR205C	91.5655	98.9101	93.9787
12:670000	YLR266C	YKL008C	-612.269	-601.326	-556.244
12:670000	YLR266C	YDR275W	-14.2326	-5.30616	-11.1596
12:670000	YLR266C	YCR061W	38.2881	45.4529	39.7346
12:670000	YLR266C	YMR110C	-316.952	-311.476	-300.398
12:670000	YLR266C	YKR046C	191.764	204.029	198.091
12:670000	YLR266C	YJR104C	-1338.18	-1326.06	-1328.17
12:670000	YLR266C	YEL035C	-471.601	-463.241	-469.215
12:670000	YLR266C	YMR038C	-786.862	-774.842	-774.844
12:670000	YLR266C	YOR334W	-210.111	-204.666	-196.41
12:670000	YLR266C	YOR356W	-597.49	-589.299	-594.071
12:670000	YLR266C	YLR288C	-615.839	-606.745	-611.85
12:670000	YLR266C	YNL010W	-1127.99	-1125.13	-1126.94
12:670000	YLR266C	YMR070W	43.4944	50.3806	58.1671
12:670000	YLR266C	YGL191W	-539.486	-531.007	-537.149
12:670000	YLR266C	YLR100W	-1003.96	-996.388	-1001.06
12:670000	YLR266C	YDR011W	-485.557	-474.904	-477.272
12:670000	YLR266C	YNL173C	-79.0441	-68.3262	-65.0751
12:670000	YLR266C	YBR085W	-258.129	-246.331	-248.495
12:670000	YLR266C	YDR529C	-501.905	-492.689	-498.407
12:670000	YLR266C	YOR085W	-1058.42	-1058.2	-1052.84
12:670000	YLR266C	YHR039C	-634.211	-624.241	-630.324
12:670000	YLR266C	YGR138C	293.651	303.461	299.59
12:670000	YLR266C	YER141W	-514.788	-505.445	-506.907
12:670000	YLR266C	YGR234W	-319.705	-313.208	-310.514
12:670000	YLR266C	YOR237W	505.056	506.081	507.625
12:670000	YLR266C	YKL150W	433.189	438.536	438.025
12:670000	YLR266C	YER011W	-645.033	-636.401	-636.465
12:670000	YLR266C	YPL027W	-571.547	-563.293	-569.382
12:670000	YLR266C	YJR105W	-1303.7	-1292.92	-1298.95
12:670000	YLR266C	YDR044W	-146.653	-137.713	-143.822
12:670000	YLR266C	YDR037W	-282.295	-278.773	-266.733
12:670000	YLR266C	YOR377W	-145.486	-140.534	-132.43
12:670000	YLR266C	YLL012W	297.723	303.492	306.254
12:670000	YLR266C	YJL133C-A	23.4673	35.6958	46.4241
12:670000	YLR266C	YGR060W	-1067.32	-1057.13	-1059.11
12:670000	YLR256W	YML019W	-901.132	-889.627	-894.223

12:670000	YLR256W	YDL086W	-569.914	-569.702	-562.062
12:670000	YLR256W	YMR220W	-916.344	-906.354	-912.197
12:670000	YLR256W	YDR518W	-704.801	-702.843	-655.513
12:670000	YLR256W	YEL051W	-1042.11	-1031.8	-1035.6
12:670000	YLR256W	YMR322C	381.573	388.218	382.07
12:670000	YLR256W	YJL100W	76.3485	84.5674	107.448
12:670000	YLR256W	YPL272C	249.167	249.505	286.014
12:670000	YLR256W	YGR266W	-884.179	-876.053	-875.855
12:670000	YLR256W	YER093C	-759.544	-748.978	-754.622
12:670000	YLR256W	YIL121W	43.2762	54.0418	47.9012
12:670000	YLR256W	YML054C	756.088	758.713	756.195
12:670000	YLR256W	YHR072W	-424.548	-422.641	-398.121
12:670000	YLR256W	YML075C	-519.545	-510.509	-511.075
12:670000	YLR256W	YDR502C	-468.63	-457.539	-463.615
12:670000	YLR256W	YER136W	-896.574	-888.693	-875.535
12:670000	YLR256W	YDR213W	-316.839	-305.453	-304.371
12:670000	YLR256W	YGR089W	-402.376	-396.422	-384.85
12:670000	YLR256W	YDR453C	736.175	739.004	757.259
12:670000	YLR256W	YEL047C	-858.465	-856.449	-853.186
12:670000	YLR256W	YDR346C	-555.737	-545.938	-546.751
12:670000	YLR256W	YLR246W	-368.814	-367.96	-356.498
12:670000	YLR256W	YJL080C	-773.488	-762.13	-767.442
12:670000	YLR256W	YBR067C	-285.783	-279.959	-280.482
12:670000	YLR256W	YHR188C	-951.248	-939.995	-930.958
12:670000	YLR256W	YLR245C	-17.1359	-8.75602	13.4231
12:670000	YLR256W	YPL170W	-419.546	-416.281	-407.229
12:670000	YLR256W	YHR003C	-437.392	-431.889	-408.964
12:670000	YLR256W	YBR183W	719.96	723.078	754.54
12:670000	YLR256W	YLR205C	79.907	88.8517	89.5845
12:670000	YLR256W	YMR110C	-325.292	-314.586	-317.076
12:670000	YLR256W	YKR046C	194.023	198.385	198.348
12:670000	YLR256W	YJR104C	-1356.42	-1350.23	-1349.28
12:670000	YLR256W	YNL264C	-964.26	-952.712	-933.729
12:670000	YLR256W	YMR038C	-793.556	-789.578	-773.218
12:670000	YLR256W	YEL024W	244.445	249.708	245.733
12:670000	YLR256W	YLR288C	-629.096	-620.927	-626.45
12:670000	YLR256W	YNL010W	-1143.53	-1135.47	-1141.62
12:670000	YLR256W	YMR070W	38.6006	41.613	59.2556
12:670000	YLR256W	YDR011W	-490.538	-481.113	-483.182
12:670000	YLR256W	YMR100W	-372.289	-360.884	-348.193
12:670000	YLR256W	YOR065W	509.911	517.638	513
12:670000	YLR256W	YNL173C	-82.973	-72.7423	-78.3349
12:670000	YLR256W	YBR085W	-259.004	-249.716	-255.406
12:670000	YLR256W	YDR529C	-506.019	-494.168	-500.138
12:670000	YLR256W	YPR095C	-454.412	-442.655	-437.683
12:670000	YLR256W	YDR218C	-615.393	-604.417	-595.773
12:670000	YLR256W	YHR039C	-639.647	-630.616	-636.737

12:670000	YLR256W	YGR138C	299.381	309.256	309.367
12:670000	YLR256W	YER141W	-514.636	-503.474	-509.477
12:670000	YLR256W	YGR234W	-327.036	-322.831	-319.141
12:670000	YLR256W	YNL280C	-855.348	-844.371	-847.731
12:670000	YLR256W	YPR065W	234.844	245.838	245.758
12:670000	YLR256W	YOL049W	-1014.77	-1002.63	-1007.26
12:670000	YLR256W	YMR244W	-285.279	-279.886	-284.694
12:670000	YLR256W	YKL150W	440.163	451.508	446.005
12:670000	YLR256W	YCR069W	-882.68	-872.905	-870.073
12:670000	YLR256W	YIL156W	-713.519	-703.496	-693.804
12:670000	YLR256W	YPL027W	-584.122	-573.785	-579.931
12:670000	YLR256W	YJR105W	-1325.07	-1324.14	-1309
12:670000	YLR256W	YHR004C	-420.076	-408.392	-388.245
12:670000	YLR256W	YDR044W	-150.831	-148.215	-144.243
12:670000	YLR256W	YGL056C	516.149	520.061	556.213
12:670000	YLR256W	YJL105W	11.7462	17.3353	36.372
12:670000	YLR256W	YLL012W	299.928	311.314	314.605
12:670000	YLR256W	YGR060W	-1073.21	-1063.37	-1069.52
12:670000	YLR256W	YBR242W	-48.114	-43.6862	13.1867
12:670000	YLR256W	YER053C-A	1160.03	1167.83	1199.29
12:670000	YLR265C	YDL086W	-522.432	-514.072	-509.764
12:670000	YLR265C	YMR220W	-851.724	-841.576	-847.265
12:670000	YLR265C	YDR518W	-636.383	-624.312	-621.344
12:670000	YLR265C	YDL085W	684.824	695.846	693.091
12:670000	YLR265C	YDL236W	-959.548	-951.315	-940.696
12:670000	YLR265C	YMR322C	338.965	347.659	367.193
12:670000	YLR265C	YLR256W	84.8453	93.8802	134.923
12:670000	YLR265C	YPR178W	-585.847	-574.869	-576.889
12:670000	YLR265C	YGL001C	-757.509	-748.098	-744.15
12:670000	YLR265C	YPL272C	264.223	268.288	264.393
12:670000	YLR265C	YPR193C	405.326	408.054	406.262
12:670000	YLR265C	YLR275W	-47.1158	-43.3705	3.79266
12:670000	YLR265C	YPL189C-A	467.087	475.342	470.095
12:670000	YLR265C	YPL107W	-196.844	-187.522	-170.76
12:670000	YLR265C	YMR202W	-1234.84	-1227.24	-1215.93
12:670000	YLR265C	YDR502C	-458.208	-448.556	-446.176
12:670000	YLR265C	YER136W	-818.427	-807.024	-812.294
12:670000	YLR265C	YGL055W	-383.043	-372.855	-378.703
12:670000	YLR265C	YOR099W	-1026.09	-1022.56	-995.761
12:670000	YLR265C	YDR213W	-291.182	-283.519	-277.942
12:670000	YLR265C	YGR089W	-363.677	-353.641	-356.898
12:670000	YLR265C	YDR297W	-0.0533998	4.64385	10.8156
12:670000	YLR265C	YDR453C	714.477	722.346	719.887
12:670000	YLR265C	YLR244C	-91.1771	-83.7036	-77.7068
12:670000	YLR265C	YMR208W	-584.578	-575.224	-572.032
12:670000	YLR265C	YLR246W	-338.948	-332.537	-331.837
12:670000	YLR265C	YDL093W	-422.509	-416.091	-395.123

12:670000	YLR265C	YJL080C	-732.052	-731.031	-706.868
12:670000	YLR265C	YBR067C	-261.665	-252.67	-257.968
12:670000	YLR265C	YHR188C	-890.606	-879.278	-875.859
12:670000	YLR265C	YPL170W	-398.087	-393.319	-384.732
12:670000	YLR265C	YER014W	-475.736	-471.11	-452.776
12:670000	YLR265C	YDR275W	-23.7232	-20.2885	-9.98052
12:670000	YLR265C	YKR046C	186.693	194.088	189.288
12:670000	YLR265C	YNL264C	-886.212	-874.836	-866.098
12:670000	YLR265C	YMR038C	-737.772	-729.463	-723.41
12:670000	YLR265C	YOR334W	-212.863	-205.893	-197.13
12:670000	YLR265C	YLR288C	-585.965	-574.024	-579.133
12:670000	YLR265C	YLR100W	-943.871	-943.446	-943.852
12:670000	YLR265C	YHR179W	-784.539	-778.932	-773.251
12:670000	YLR265C	YBR085W	-224.696	-213.226	-219.218
12:670000	YLR265C	YPR095C	-419.273	-409.72	-385.413
12:670000	YLR265C	YDR218C	-547.489	-539.684	-543.166
12:670000	YLR265C	YER141W	-470.694	-458.887	-464.937
12:670000	YLR265C	YNL280C	-796.886	-784.963	-779.319
12:670000	YLR265C	YMR244W	-258.119	-247.967	-249.737
12:670000	YLR265C	YMR134W	-425.776	-417.64	-421.232
12:670000	YLR265C	YCR069W	-816.318	-809.428	-814.54
12:670000	YLR265C	YER011W	-595.116	-590.625	-593.939
12:670000	YLR265C	YPL027W	-536.633	-532.873	-535.139
12:670000	YLR265C	YHR004C	-380.74	-377.856	-343.889
12:670000	YLR265C	YOR377W	-131.386	-129.474	-128.471
12:670000	YLR265C	YJL133C-A	45.7632	56.3025	50.516
12:670000	YLR265C	YJL167W	-1229.05	-1221.08	-1196.66
12:670000	YLR260W	YLR266C	-47.16	-42.433	-20.4084
12:670000	YLR260W	YML019W	-911.624	-911.147	-909.731
12:670000	YLR260W	YDL086W	-581.337	-569.04	-570.082
12:670000	YLR260W	YMR220W	-921.924	-913.398	-918.725
12:670000	YLR260W	YDL236W	-1040.14	-1030.42	-1024.43
12:670000	YLR260W	YEL051W	-1058.04	-1049.77	-1047.05
12:670000	YLR260W	YMR322C	376.882	383.514	384.663
12:670000	YLR260W	YLR256W	76.7758	88.6316	135.064
12:670000	YLR260W	YGL001C	-810.73	-805.8	-801.962
12:670000	YLR260W	YOR003W	-10.0573	1.84237	10.007
12:670000	YLR260W	YLR275W	-65.6282	-54.7384	-7.61844
12:670000	YLR260W	YML008C	-1144.99	-1140.13	-1141.18
12:670000	YLR260W	YMR202W	-1332.37	-1325.01	-1310.47
12:670000	YLR260W	YDR502C	-478.245	-467.796	-468.917
12:670000	YLR260W	YER136W	-895.907	-886.621	-887.76
12:670000	YLR260W	YLR234W	186.63	198.569	196.116
12:670000	YLR260W	YGL055W	-439.489	-427.359	-427.706
12:670000	YLR260W	YDR213W	-318.492	-312.046	-311.812
12:670000	YLR260W	YGR089W	-388.841	-384.571	-385.505
12:670000	YLR260W	YDR297W	0.418782	5.54792	10.1958

12:670000	YLR260W	YDR453C	764.076	770.366	768.454
12:670000	YLR260W	YDR186C	-355.219	-348.701	-326.243
12:670000	YLR260W	YLR244C	-92.1464	-89.7821	-80.2722
12:670000	YLR260W	YEL047C	-867.347	-856.944	-863.099
12:670000	YLR260W	YLR246W	-366.151	-358.831	-364.203
12:670000	YLR260W	YDL093W	-435.375	-426.068	-430.959
12:670000	YLR260W	YJL080C	-784.995	-774.28	-777.394
12:670000	YLR260W	YBR067C	-319.203	-311.839	-287.011
12:670000	YLR260W	YPL170W	-420.946	-409.163	-415.268
12:670000	YLR260W	YOL128C	772.189	777.435	839.58
12:670000	YLR260W	YHR003C	-441.28	-438.897	-404.312
12:670000	YLR260W	YLR205C	90.6546	94.0487	94.1077
12:670000	YLR260W	YKL008C	-578.003	-566.148	-570.846
12:670000	YLR260W	YKR046C	185.62	192.431	194.198
12:670000	YLR260W	YJR104C	-1371.45	-1362.2	-1365.15
12:670000	YLR260W	YNL264C	-946.056	-943.433	-940.553
12:670000	YLR260W	YMR113W	-719.411	-711.928	-706.403
12:670000	YLR260W	YEL035C	-484.091	-479.274	-483.046
12:670000	YLR260W	YMR038C	-790.162	-778.46	-784.611
12:670000	YLR260W	YGL077C	-496.564	-491.334	-482.771
12:670000	YLR260W	YLR288C	-635.4	-627.121	-633.264
12:670000	YLR260W	YNL010W	-1153.98	-1148.91	-1153.96
12:670000	YLR260W	YLR100W	-1033.23	-1024.28	-1027.86
12:670000	YLR260W	YGR131W	342.45	349.907	379.462
12:670000	YLR260W	YDR011W	-518.636	-515.997	-489.839
12:670000	YLR260W	YMR100W	-444.437	-437.448	-351.513
12:670000	YLR260W	YBR085W	-270.922	-261.188	-261.457
12:670000	YLR260W	YPR095C	-482.435	-475.599	-436.622
12:670000	YLR260W	YHR039C	-688.809	-682.898	-645.011
12:670000	YLR260W	YGR138C	297.812	303.608	309.723
12:670000	YLR260W	YER141W	-532.024	-522.263	-518.205
12:670000	YLR260W	YNR060W	615.213	616.669	620.125
12:670000	YLR260W	YOL049W	-1021.4	-1015.62	-1020.85
12:670000	YLR260W	YMR244W	-292.269	-290.255	-282.47
12:670000	YLR260W	YER011W	-662.04	-651.835	-657.735
12:670000	YLR260W	YPL027W	-585.661	-576.884	-583.032
12:670000	YLR260W	YJR105W	-1338.57	-1328.72	-1320.28
12:670000	YLR260W	YHR004C	-417.564	-405.549	-380.15
12:670000	YLR260W	YDR037W	-276.272	-271.642	-273.968
12:670000	YLR260W	YGL056C	511.704	523.64	566.149
12:670000	YLR260W	YAL039C	-115.56	-110.79	-103.973
12:670000	YLR260W	YOR377W	-158.277	-152.832	-133.944
12:670000	YLR260W	YLL012W	251.947	262.788	319.144
12:670000	YLR260W	YJL133C-A	37.7976	41.1146	40.5571
12:670000	YLR260W	YGR060W	-1088.12	-1087.28	-1078.84
12:670000	YLR260W	YJL167W	-1320.5	-1319.08	-1301.11
12:670000	YLR270W	YOR175C	-598.973	-597.1	-583.852

12:670000	YLR270W	YML019W	-901.342	-892.374	-897.769
12:670000	YLR270W	YMR220W	-914.319	-905.543	-911.618
12:670000	YLR270W	YLR283W	-573.936	-567.852	-527.263
12:670000	YLR270W	YDR518W	-665.614	-659.629	-643.79
12:670000	YLR270W	YEL051W	-1126	-1122.6	-1039.42
12:670000	YLR270W	YOR321W	-226.035	-218.355	-22.7236
12:670000	YLR270W	YJL100W	2.7634	10.4524	116.112
12:670000	YLR270W	YOR003W	-74.3477	-68.6647	14.1535
12:670000	YLR270W	YGR266W	-885.177	-877.807	-856.93
12:670000	YLR270W	YFR033C	-80.8963	-70.7738	29.4964
12:670000	YLR270W	YML008C	-1156.9	-1152.61	-1126.89
12:670000	YLR270W	YPL189C-A	485.335	491.82	499.97
12:670000	YLR270W	YHR072W	-634.17	-630.828	-389.165
12:670000	YLR270W	YLR231C	-455.796	-445.748	-397.094
12:670000	YLR270W	YLR234W	152.626	162.501	192.314
12:670000	YLR270W	YGL055W	-432.84	-422.514	-426.406
12:670000	YLR270W	YOR099W	-1152.49	-1143.94	-1050.09
12:670000	YLR270W	YGR089W	-390.794	-382.55	-379.387
12:670000	YLR270W	YML032C	-576.965	-568.672	-310.71
12:670000	YLR270W	YDR186C	-547.962	-537.238	-317.831
12:670000	YLR270W	YEL047C	-979.032	-969.948	-855.412
12:670000	YLR270W	YDL093W	-473.997	-465.764	-418.886
12:670000	YLR270W	YJL080C	-875.159	-874.116	-766.198
12:670000	YLR270W	YBR067C	-294.466	-285.369	-290.329
12:670000	YLR270W	YPL170W	-492.324	-484.285	-409.646
12:670000	YLR270W	YCR048W	-635.119	-625.1	-516.979
12:670000	YLR270W	YGR281W	-374.649	-364.536	-299.255
12:670000	YLR270W	YLR205C	1.26096	9.51546	96.5811
12:670000	YLR270W	YER014W	-506.449	-504.916	-492.376
12:670000	YLR270W	YJR048W	33.5613	40.1233	100.862
12:670000	YLR270W	YKL008C	-606.6	-594.321	-562.762
12:670000	YLR270W	YDR275W	-31.5758	-21.1749	-12.4478
12:670000	YLR270W	YCR061W	-39.2874	-28.8769	38.346
12:670000	YLR270W	YMR110C	-316.247	-311.622	-306.747
12:670000	YLR270W	YKR046C	186.183	196.909	200.026
12:670000	YLR270W	YJR104C	-1453.18	-1441.86	-1345.91
12:670000	YLR270W	YEL035C	-483.644	-476.419	-472.938
12:670000	YLR270W	YMR038C	-841.845	-829.931	-787.041
12:670000	YLR270W	YEL024W	45.1149	54.8316	252.401
12:670000	YLR270W	YLR288C	-627.214	-618.837	-623.361
12:670000	YLR270W	YNL010W	-1147.79	-1141.65	-1141.79
12:670000	YLR270W	YGL191W	-558.385	-547.487	-546.24
12:670000	YLR270W	YLR100W	-1088.46	-1076.83	-1014.84
12:670000	YLR270W	YDR011W	-601.086	-593.326	-482.588
12:670000	YLR270W	YOR065W	266.217	277.38	508.103
12:670000	YLR270W	YNL173C	-236.03	-223.823	-68.7826
12:670000	YLR270W	YBR085W	-267.252	-256.826	-253.105

12:670000	YLR270W	YDR529C	-630.132	-618.31	-506.419
12:670000	YLR270W	YOR085W	-1156.82	-1153.99	-1068.27
12:670000	YLR270W	YHR039C	-692.295	-686.046	-640.892
12:670000	YLR270W	YGR138C	222.789	235.069	300.683
12:670000	YLR270W	YER141W	-602.718	-592.338	-514.983
12:670000	YLR270W	YGR234W	-461.374	-457.016	-317.819
12:670000	YLR270W	YNL280C	-877.926	-877.832	-851.078
12:670000	YLR270W	YOL049W	-1012.64	-1011.67	-1008.56
12:670000	YLR270W	YMR134W	-438.662	-437.399	-436.664
12:670000	YLR270W	YKL150W	199.679	210.49	440.656
12:670000	YLR270W	YER011W	-688.303	-679.583	-648.196
12:670000	YLR270W	YPL027W	-587.276	-581.999	-574.075
12:670000	YLR270W	YJR105W	-1397.66	-1385.99	-1315.67
12:670000	YLR270W	YHR004C	-497.409	-486.631	-363.829
12:670000	YLR270W	YDR044W	-368.792	-365.991	-142.638
12:670000	YLR270W	YDR037W	-323.582	-315.539	-271.173
12:670000	YLR270W	YJR047C	-31.4982	-24.616	65.8748
12:670000	YLR270W	YOR377W	-154.62	-152.729	-132.339
12:670000	YLR270W	YLL012W	5.17592	7.67977	309.595
12:670000	YLR270W	YJL133C-A	39.5935	50.6571	46.5807
12:670000	YLR270W	YGR060W	-1150.84	-1141.42	-1072.61
12:670000	YLR270W	YHR048W	116.792	125.223	399.315
12:670000	YLR270W	YCR073W-A	-623.654	-616.574	-516.661
12:670000	YLR273C	YML019W	-923.458	-918.241	-908.057
12:670000	YLR273C	YDL086W	-646.901	-634.835	-568.734
12:670000	YLR273C	YMR220W	-923.704	-914.594	-919.159
12:670000	YLR273C	YLR283W	-542.261	-536.839	-538.165
12:670000	YLR273C	YDL085W	710.343	717.405	739.214
12:670000	YLR273C	YDL236W	-1066.52	-1056.92	-1025.42
12:670000	YLR273C	YMR322C	335.981	342.433	388.39
12:670000	YLR273C	YLR256W	7.1215	19.2948	138.934
12:670000	YLR273C	YOR321W	-45.5568	-38.8053	-24.5847
12:670000	YLR273C	YGL001C	-838.314	-837.161	-799.567
12:670000	YLR273C	YOR003W	-71.1214	-59.5656	11.6681
12:670000	YLR273C	YLR275W	-135.337	-130.812	-17.3988
12:670000	YLR273C	YHR072W	-527.796	-527.614	-390.364
12:670000	YLR273C	YMR202W	-1358.89	-1351.39	-1308.72
12:670000	YLR273C	YLR231C	-413.13	-406.29	-405.825
12:670000	YLR273C	YDR502C	-516.153	-512.574	-469.053
12:670000	YLR273C	YLR234W	188.764	197.87	196.025
12:670000	YLR273C	YGL055W	-461.602	-450.432	-428.891
12:670000	YLR273C	YOR099W	-1070.06	-1062.68	-1064.62
12:670000	YLR273C	YDR213W	-323.874	-319.443	-309.911
12:670000	YLR273C	YGR089W	-388.852	-379.971	-384.46
12:670000	YLR273C	YDR297W	-25.1948	-22.0523	14.9904
12:670000	YLR273C	YDR186C	-429.277	-421.622	-325.908
12:670000	YLR273C	YEL047C	-896.998	-892.367	-865.502

12:670000	YLR273C	YLR270W	-129.102	-121.366	-127.459
12:670000	YLR273C	YLR246W	-366.205	-362.875	-365.685
12:670000	YLR273C	YDL093W	-433.651	-423.959	-428.529
12:670000	YLR273C	YJL080C	-837.781	-828.503	-776.248
12:670000	YLR273C	YBR067C	-294.155	-285.047	-287.913
12:670000	YLR273C	YPL170W	-429.886	-418.521	-416.167
12:670000	YLR273C	YOL128C	638.258	647.04	846.583
12:670000	YLR273C	YHR003C	-563.267	-556.713	-407.562
12:670000	YLR273C	YGR281W	-332.303	-328.208	-289.614
12:670000	YLR273C	YLR205C	45.1126	49.4759	94.1829
12:670000	YLR273C	YKL008C	-579.804	-567.741	-571.014
12:670000	YLR273C	YDR275W	-73.4215	-62.1203	-12.8831
12:670000	YLR273C	YCR061W	36.3474	37.3736	37.5191
12:670000	YLR273C	YIL154C	-572.842	-570.688	-569.189
12:670000	YLR273C	YKR046C	181.967	192.321	197.329
12:670000	YLR273C	YJR104C	-1427.23	-1419.2	-1363.63
12:670000	YLR273C	YNL264C	-944.957	-944.167	-939.571
12:670000	YLR273C	YEL035C	-499.272	-493.775	-482.062
12:670000	YLR273C	YMR038C	-793.376	-781.187	-783.844
12:670000	YLR273C	YLR288C	-638.607	-629.876	-632.794
12:670000	YLR273C	YGL191W	-555.538	-550.327	-552.744
12:670000	YLR273C	YLR100W	-1028.46	-1019.69	-1025.77
12:670000	YLR273C	YGR131W	296.722	301.718	381.285
12:670000	YLR273C	YDR011W	-594.283	-588.321	-490.392
12:670000	YLR273C	YMR100W	-538.244	-530.869	-355.239
12:670000	YLR273C	YBR085W	-266.933	-254.874	-259.791
12:670000	YLR273C	YDR529C	-612.183	-611.33	-506.853
12:670000	YLR273C	YOR085W	-1086.6	-1082.78	-1083.13
12:670000	YLR273C	YPR095C	-490.563	-482.945	-434.21
12:670000	YLR273C	YHR039C	-753.733	-743.992	-644.357
12:670000	YLR273C	YGR138C	221.451	225.025	308.715
12:670000	YLR273C	YER141W	-546.072	-534.051	-518.767
12:670000	YLR273C	YGR234W	-392.428	-385.489	-330.65
12:670000	YLR273C	YNL280C	-882.423	-876.544	-854.042
12:670000	YLR273C	YMR134W	-443.906	-439.3	-442.655
12:670000	YLR273C	YER011W	-668.512	-659.592	-658.57
12:670000	YLR273C	YPL027W	-584.614	-577.786	-583.024
12:670000	YLR273C	YJR105W	-1371.11	-1362.41	-1320.8
12:670000	YLR273C	YHR004C	-425.481	-413.495	-371.661
12:670000	YLR273C	YGL056C	370.517	382.159	566.902
12:670000	YLR273C	YOR377W	-176.813	-166.807	-134.591
12:670000	YLR273C	YLL012W	166.588	175.077	317.228
12:670000	YLR273C	YJL133C-A	24.7359	31.5303	43.2745
13:70000	YML091C	YDR481C	-569.224	-557.513	-554.527
13:70000	YML091C	YML035C	-394.436	-387.651	-382.122
13:70000	YML091C	YIL160C	455.547	466.772	552.591
13:70000	YML091C	YOL011W	-265.949	-258.349	-261.409

13:70000	YML091C	YDL085W	502.636	505.437	720.511
13:70000	YML091C	YPR184W	564.28	570.936	622.358
13:70000	YML091C	YML124C	-1247.19	-1242.86	-1238.09
13:70000	YML091C	YCR059C	-68.7284	-62.8279	-47.9559
13:70000	YML091C	YCR098C	494.613	503.562	500.881
13:70000	YML091C	YER069W	128.844	131.41	361.63
13:70000	YML091C	YJR032W	-443.227	-442.864	-437.607
13:70000	YML091C	YPL272C	266.786	279.058	312.007
13:70000	YML091C	YDL222C	873.589	876.386	894.438
13:70000	YML091C	YNL073W	-299.534	-298.088	-266.806
13:70000	YML091C	YDR349C	-316.066	-308.583	-269.245
13:70000	YML091C	YLR267W	793.732	799.135	816.845
13:70000	YML091C	YPL019C	-174.103	-168.861	93.4827
13:70000	YML091C	YDR502C	-506.784	-494.643	-470.337
13:70000	YML091C	YIR031C	817.6	825.226	946.117
13:70000	YML091C	YDR453C	560.786	570.653	733.53
13:70000	YML091C	YDL182W	108.832	118.738	204.09
13:70000	YML091C	YCL027W	95.8008	99.4782	97.8592
13:70000	YML091C	YER073W	759.932	768.919	797.793
13:70000	YML091C	YGR031W	-300.856	-291.787	-254.282
13:70000	YML091C	YKL084W	-320.012	-319.907	-275.807
13:70000	YML091C	YKL091C	439.85	443.365	448.098
13:70000	YML091C	YGR233C	-163.104	-156.683	-84.485
13:70000	YML091C	YBL016W	9.20052	21.2007	44.9156
13:70000	YML091C	YML030W	-140.173	-139.683	-129.475
13:70000	YML091C	YAL026C	-830.307	-827.9	-829.903
13:70000	YML091C	YLR179C	-967.243	-956.379	-950.521
13:70000	YML091C	YML070W	-907.557	-904.419	-847.454
13:70000	YML091C	YDL181W	355.453	364.617	423.591
13:70000	YML091C	YML098W	-397.392	-391.102	-325.38
13:70000	YML091C	YHR199C-A	-227.494	-218.628	-197.288
13:70000	YML091C	YMR236W	-939.374	-937.191	-879.713
13:70000	YML091C	YNL244C	-672.742	-670.379	-495.604
13:70000	YML091C	YHR214C-E	86.4986	96.4648	102.49
13:70000	YML091C	YER165W	-930.178	-920.471	-857.803
13:70000	YML091C	YML087C	264.982	270.398	267.433
13:70000	YML091C	YJR060W	-259.931	-252.095	-257.938
13:70000	YML091C	YJL012C	-684.935	-672.845	-555.861
13:70000	YML091C	YER055C	-85.4303	-73.9621	-42.7508
13:70000	YML091C	YAL012W	-832.81	-825.959	-766.649
13:70000	YML091C	YIR027C	1022.61	1033.22	1201.48
13:70000	YML091C	YLR348C	-224.812	-215.998	-79.0297
13:70000	YML091C	YDL052C	-850.355	-840.326	-829.248
13:70000	YML091C	YLL018C-A	-138.278	-126.726	-81.1843
13:70000	YML091C	YHR183W	-793.193	-784.204	-765.798
13:70000	YML091C	YNL279W	329.034	340.414	362.813
13:70000	YML091C	YGR156W	-3.42785	-2.97915	31.2766

13:70000	YML091C	YHR007C	-841.694	-833.094	-819.847
13:70000	YML091C	YMR065W	-393.626	-391.895	-371.35
13:70000	YML091C	YER060W-A	-152.797	-144.468	-86.9394
13:70000	YML091C	YOR163W	-749.523	-747.917	-658.2
13:70000	YML091C	YER043C	-1015.65	-1003.86	-947.421
13:70000	YML091C	YHR047C	-354.057	-342.964	-334.353
13:70000	YML091C	YIL050W	5.2777	14.1307	13.8338
13:70000	YML091C	YDR124W	-252.995	-250.876	-236.279
13:70000	YML091C	YLL055W	146.818	155.495	163.049
13:70000	YML091C	YNL218W	-595.193	-588.134	-584.603
13:70000	YML091C	YIL051C	-1344.52	-1333.67	-1189.1
13:70000	YML091C	YDL090C	-559.133	-556.579	-517.949
13:70000	YML091C	YPL110C	-98.1495	-92.3628	-19.4095
13:70000	YML091C	YML047C	441.693	453.574	449.778
13:70000	YML091C	YKL087C	81.39	88.0806	82.1419
13:70000	YML091C	YJL157C	307.759	317.313	456.747
13:70000	YML091C	YKL127W	-920.65	-919.136	-891.355
13:70000	YML091C	YCL025C	-338.672	-329.798	-315.967
13:70000	YML091C	YML085C	-1123.99	-1122.77	-1115.9
13:70000	YML091C	YMR177W	-227.255	-221.063	-203.444
13:70000	YML091C	YGL234W	-299.322	-287.483	-135.61
13:70000	YML091C	YIL008W	-91.0225	-82.5908	-0.123519
13:70000	YML091C	YLR180W	618.626	623.701	786.53
13:70000	YML091C	YCL064C	-432.258	-423.923	-329.131
13:70000	YML091C	YPR074C	-1202.92	-1194.38	-1188.78
13:70000	YML091C	YDR111C	-252.357	-246.402	-155.874
13:70000	YML091C	YML110C	-1054.34	-1046.3	-927.904
13:70000	YML091C	YKL120W	490.645	498.635	496.829
13:70000	YML091C	YKL051W	225.807	233.952	228.107
13:70000	YML091C	YOR375C	-1445.43	-1440.75	-1407.66
13:70000	YML091C	YLL056C	-419.722	-408.814	-345.51
13:70000	YML091C	YBR208C	363.081	369.133	402.511
13:70000	YML091C	YOR032W-A	-398.597	-386.976	-373.481
13:70000	YML091C	YER081W	-235.028	-225.421	-223.665
13:70000	YML091C	YOR247W	-982.79	-971.742	-940.957
13:70000	YML091C	YIR028W	1114.32	1124.77	1268.72
13:70000	YML091C	YPR139C	-713.471	-703.663	-624.652
13:70000	YML091C	YGR238C	-177.77	-172.446	-114.871
13:70000	YML091C	YML104C	-672.811	-660.51	-666.082
13:70000	YML091C	YER072W	-893.243	-881.489	-785.239
13:70000	YML091C	YML079W	-945.055	-940.756	-735.017
13:70000	YML091C	YPL018W	360.694	368.066	384.441
13:70000	YML091C	YGR007W	-1006.91	-997.531	-1003.57
13:70000	YML091C	YGL167C	-936.148	-928.178	-777.071
13:70000	YML091C	YLR178C	455.337	457.738	570.816
13:70000	YML091C	YLR164W	222.177	231.918	328.124
13:70000	YML091C	YDR482C	-513.256	-503.104	-360.353

13:70000	YML091C	YER034W	-675.579	-673.625	-589.252
13:70000	YML091C	YGL255W	917.352	926.993	928.814
13:70000	YML091C	YGR061C	-197.782	-186.162	-100.701
13:70000	YML096W	YDR481C	-653.855	-653.816	-558.812
13:70000	YML096W	YML035C	-495.991	-487.063	-382.689
13:70000	YML096W	YPL269W	-606.422	-597.603	-474.55
13:70000	YML096W	YBR296C	773.053	776.975	805.386
13:70000	YML096W	YHL032C	-485.129	-481.429	-469.249
13:70000	YML096W	YML091C	174.133	176.042	388.447
13:70000	YML096W	YPR184W	589.588	595.44	624.977
13:70000	YML096W	YER103W	365.005	375.882	523.279
13:70000	YML096W	YOR045W	-698.894	-695.573	-687.736
13:70000	YML096W	YCR059C	-188.164	-180.557	-44.2623
13:70000	YML096W	YJL088W	985.837	989.522	1093.85
13:70000	YML096W	YCR098C	443.492	452.568	501.037
13:70000	YML096W	YCR011C	-883.515	-881.247	-852.747
13:70000	YML096W	YJR032W	-457.581	-449.153	-428.351
13:70000	YML096W	YGL038C	-642.13	-632.774	-494.105
13:70000	YML096W	YDL222C	864.04	871.364	897.654
13:70000	YML096W	YLR410W	-376.058	-368.695	-365.208
13:70000	YML096W	YER150W	470.724	472.497	698.39
13:70000	YML096W	YER054C	896.64	906.229	1056.98
13:70000	YML096W	YGR233C	-120.557	-108.814	-80.2624
13:70000	YML096W	YAL026C	-836.775	-827.657	-828.473
13:70000	YML096W	YLR179C	-971.189	-965.846	-955.235
13:70000	YML096W	YHR103W	-669.5	-657.774	-512.932
13:70000	YML096W	YDL181W	346.386	356.998	428.017
13:70000	YML096W	YBR067C	-293.42	-286.46	-292.107
13:70000	YML096W	YBL082C	-603.453	-598.166	-536.094
13:70000	YML096W	YOL152W	243.706	255.621	509.662
13:70000	YML096W	YPR013C	239.401	245.836	459.407
13:70000	YML096W	YEL052W	-672.179	-663.658	-589.949
13:70000	YML096W	YJL079C	-372.872	-363.388	-322.365
13:70000	YML096W	YDR130C	-337.978	-325.992	-174.445
13:70000	YML096W	YHR214C-E	90.6045	102.263	102.465
13:70000	YML096W	YML087C	132.408	143.711	265.52
13:70000	YML096W	YJR060W	-315.578	-305.474	-257.14
13:70000	YML096W	YJL012C	-553.34	-549.755	-552.912
13:70000	YML096W	YBR105C	-52.7207	-50.6384	-49.0849
13:70000	YML096W	YCL049C	-345.493	-337.646	-231.49
13:70000	YML096W	YNR032W	-600.189	-593.978	-537.319
13:70000	YML096W	YDR309C	-230.711	-223.575	-182.962
13:70000	YML096W	YPR035W	-1491.25	-1484.42	-1489.36
13:70000	YML096W	YOR066W	-559.719	-556.451	-554.756
13:70000	YML096W	YMR240C	-379.425	-371.235	-371.209
13:70000	YML096W	YJL213W	171.082	178.129	411.559
13:70000	YML096W	YGR156W	-30.4508	-19.3482	39.7933

13:70000	YML096W	YHR136C	880.352	890.6	903.296
13:70000	YML096W	YHR007C	-871.583	-860.35	-818.218
13:70000	YML096W	YER060W-A	-93.1009	-87.2677	-87.9858
13:70000	YML096W	YDL073W	-686.006	-676.381	-589.921
13:70000	YML096W	YJL098W	576.3	588.483	718.338
13:70000	YML096W	YOR163W	-697.561	-691.872	-657.047
13:70000	YML096W	YNL194C	1007.3	1011	1130.94
13:70000	YML096W	YIL111W	-74.6889	-65.8311	3.20495
13:70000	YML096W	YHR047C	-375.688	-367.438	-334.494
13:70000	YML096W	YPL268W	-752.243	-752.033	-615.268
13:70000	YML096W	YIL050W	-80.1115	-75.1088	14.5831
13:70000	YML096W	YHR162W	-994.945	-989.796	-840.983
13:70000	YML096W	YCL042W	20.872	30.7864	104.069
13:70000	YML096W	YNL218W	-586.733	-576.571	-581.97
13:70000	YML096W	YBL043W	373.156	381.962	470.413
13:70000	YML096W	YKL189W	-275.98	-265.89	-243.706
13:70000	YML096W	YDR019C	183.273	187.167	289.467
13:70000	YML096W	YOR374W	675.614	685.297	940.016
13:70000	YML096W	YOL016C	-342.651	-337.094	-204.691
13:70000	YML096W	YMR296C	-725.447	-720.243	-706.639
13:70000	YML096W	YPL052W	227.56	235.969	474.392
13:70000	YML096W	YAR071W	441.833	449.636	477.861
13:70000	YML096W	YDL090C	-510.746	-510.432	-510.22
13:70000	YML096W	YML047C	358.169	364.424	451.024
13:70000	YML096W	YGR243W	622.67	630.709	795.55
13:70000	YML096W	YPL014W	710.572	721.252	1057.15
13:70000	YML096W	YCL025C	-340.272	-337.025	-320.452
13:70000	YML096W	YMR177W	-224.425	-216.767	-202.369
13:70000	YML096W	YOR274W	-168.907	-159.757	28.0096
13:70000	YML096W	YPL061W	-108.102	-98.6765	38.4851
13:70000	YML096W	YNL258C	-958.902	-949.321	-839.502
13:70000	YML096W	YCL064C	-324.253	-319.779	-317.577
13:70000	YML096W	YPR074C	-1214.86	-1205.56	-1185.99
13:70000	YML096W	YDR111C	-172.209	-169.096	-153.766
13:70000	YML096W	YML121W	-658.772	-653.342	-335.436
13:70000	YML096W	YOR316C	-747.469	-738.104	-643.674
13:70000	YML096W	YKL120W	393.718	401.234	498.609
13:70000	YML096W	YKL051W	213.051	221.823	228.688
13:70000	YML096W	YOR375C	-1415.13	-1405.47	-1409.24
13:70000	YML096W	YBR208C	303.121	315.113	402.157
13:70000	YML096W	YOR032W-A	-377.469	-371.327	-371.346
13:70000	YML096W	YER081W	-235.555	-223.677	-221.603
13:70000	YML096W	YCL040W	284.084	291.289	363.697
13:70000	YML096W	YOL058W	682.333	685.231	721.311
13:70000	YML096W	YML104C	-675.752	-666.435	-668.791
13:70000	YML096W	YER072W	-787.301	-778.885	-783.39
13:70000	YML096W	YGR007W	-1005.76	-994.706	-1000.72

13:70000	YML096W	YLR121C	346.794	347.147	523.272
13:70000	YML096W	YIL120W	-282.877	-281.79	-217.952
13:70000	YML096W	YBR093C	203.181	211.108	245.717
13:70000	YML096W	YGR061C	-102.95	-97.3708	-101.282
13:70000	YML096W	YDR281C	554.436	564.216	705.744
13:70000	YML098W	YML035C	-394.003	-385.699	-386.418
13:70000	YML098W	YIL160C	503.687	507.935	578.496
13:70000	YML098W	YBR296C	798.729	806.676	808.797
13:70000	YML098W	YDL085W	716.891	720.868	740.202
13:70000	YML098W	YER103W	510.33	511.799	539.297
13:70000	YML098W	YJL088W	1101.17	1111.95	1105.78
13:70000	YML098W	YCR098C	463.412	474.151	503.676
13:70000	YML098W	YCR073C	790.997	802.988	814.502
13:70000	YML098W	YPL272C	310.457	313.724	313.45
13:70000	YML098W	YNL073W	-445.37	-441.034	-267.109
13:70000	YML098W	YOL064C	-392.854	-385.783	-381.731
13:70000	YML098W	YDR349C	-348.275	-336.853	-267.891
13:70000	YML098W	YGR166W	421.314	433.624	467.803
13:70000	YML098W	YPL019C	48.7522	59.2154	93.5737
13:70000	YML098W	YIR031C	941.24	941.251	956.473
13:70000	YML098W	YLR410W	-439.943	-431.599	-371.245
13:70000	YML098W	YGR223C	-287.612	-279.548	-268.161
13:70000	YML098W	YDL182W	207.364	211.447	208.417
13:70000	YML098W	YER150W	694.112	697.321	712.45
13:70000	YML098W	YAR008W	-310.125	-303.388	-308.716
13:70000	YML098W	YFL004W	-722.328	-717.096	-680.639
13:70000	YML098W	YER073W	726.753	737.371	805.124
13:70000	YML098W	YKL084W	-331.75	-321.022	-275.183
13:70000	YML098W	YGR233C	-133.037	-131.111	-81.3505
13:70000	YML098W	YBL016W	14.6004	20.0069	57.1402
13:70000	YML098W	YHR030C	-243.067	-241.354	-242.686
13:70000	YML098W	YML030W	-233.48	-229.452	-124.558
13:70000	YML098W	YAL026C	-841.735	-834.332	-838.856
13:70000	YML098W	YLR179C	-1010.4	-1006.43	-947.25
13:70000	YML098W	YHR103W	-523.327	-514.769	-520.043
13:70000	YML098W	YDL181W	368.489	373.595	429.939
13:70000	YML098W	YBR067C	-306.098	-299.838	-290.874
13:70000	YML098W	YOL152W	514.415	516.712	514.718
13:70000	YML098W	YNL244C	-551.054	-543.072	-494.97
13:70000	YML098W	YHR214C-E	87.9411	99.7179	102.053
13:70000	YML098W	YFR044C	-1120.39	-1114.39	-1112.8
13:70000	YML098W	YER165W	-875.417	-866.607	-861.94
13:70000	YML098W	YML087C	185.027	185.293	268.901
13:70000	YML098W	YJR060W	-318.672	-311.282	-262.101
13:70000	YML098W	YJL012C	-615.129	-606.418	-558.732
13:70000	YML098W	YER055C	-80.9403	-71.8111	-43.5584
13:70000	YML098W	YLR193C	-559.465	-554.927	-551.11

13:70000	YML098W	YAL012W	-786.772	-784.741	-768.965
13:70000	YML098W	YCL049C	-239.937	-233.751	-237.467
13:70000	YML098W	YPR035W	-1516.56	-1508.9	-1509.82
13:70000	YML098W	YNL100W	-80.9233	-79.9869	-12.1594
13:70000	YML098W	YMR240C	-391.476	-385.076	-375.351
13:70000	YML098W	YJL213W	384.967	385.752	422.562
13:70000	YML098W	YNL279W	352.677	353.024	372.838
13:70000	YML098W	YHR136C	858.802	864.498	909.353
13:70000	YML098W	YJL117W	-617.04	-604.778	-571.069
13:70000	YML098W	YHR007C	-917.822	-910.015	-807.518
13:70000	YML098W	YER060W-A	-136.324	-132.968	-89.3647
13:70000	YML098W	YOR163W	-705.253	-697.27	-657.347
13:70000	YML098W	YER043C	-988.987	-983.221	-949.531
13:70000	YML098W	YHR047C	-472.264	-467.007	-333.596
13:70000	YML098W	YPL268W	-619.515	-613.378	-618.153
13:70000	YML098W	YIL050W	-9.00302	-5.13799	14.9236
13:70000	YML098W	YHR162W	-860.778	-851.183	-838.082
13:70000	YML098W	YNL218W	-599.684	-588.986	-586.056
13:70000	YML098W	YDR019C	269.237	277.314	292.248
13:70000	YML098W	YOL016C	-286.905	-283.316	-207.832
13:70000	YML098W	YGR189C	-277.913	-271.138	-275.569
13:70000	YML098W	YPL052W	476.687	487.019	481.597
13:70000	YML098W	YAR071W	465.052	466.143	478.493
13:70000	YML098W	YML119W	-408.389	-405.452	-394.353
13:70000	YML098W	YDR529C	-514.391	-505.845	-508.68
13:70000	YML098W	YML047C	408.535	419.843	467.41
13:70000	YML098W	YOR274W	2.85558	3.58164	30.7252
13:70000	YML098W	YPL061W	29.5223	40.8572	34.6939
13:70000	YML098W	YPL156C	17.4891	20.8269	27.3482
13:70000	YML098W	YLR180W	714.85	719.694	798.118
13:70000	YML098W	YNL258C	-860.617	-854.253	-851.491
13:70000	YML098W	YCL064C	-332.676	-330.235	-319.751
13:70000	YML098W	YPR074C	-1302.11	-1298.63	-1196.88
13:70000	YML098W	YDR111C	-206.828	-198.981	-157.349
13:70000	YML098W	YML121W	-382.933	-374.297	-339.359
13:70000	YML098W	YKL120W	473.867	484.929	512.076
13:70000	YML098W	YKL051W	178.799	189.768	230.078
13:70000	YML098W	YOR375C	-1423.24	-1412.87	-1416.7
13:70000	YML098W	YOR032W-A	-379.569	-370.164	-376.254
13:70000	YML098W	YER081W	-294.921	-288.142	-226.769
13:70000	YML098W	YPR139C	-667.213	-666.617	-637.573
13:70000	YML098W	YGR238C	-143.616	-137.252	-119.226
13:70000	YML098W	YOL058W	693.878	701.987	729.797
13:70000	YML098W	YML104C	-681.408	-671.77	-677.127
13:70000	YML098W	YER072W	-885.531	-876.326	-787.9
13:70000	YML098W	YPL018W	388.382	392.151	389.299
13:70000	YML098W	YKL079W	-401.174	-398.253	-391.218

13:70000	YML098W	YGR007W	-1027.06	-1016.45	-1008.21
13:70000	YML098W	YGL167C	-787.007	-782.574	-785.126
13:70000	YML098W	YLR121C	514.666	525.719	532.823
13:70000	YML098W	YNL141W	1147.31	1156.89	1200.18
13:70000	YML098W	YIL120W	-240.651	-236.374	-222.763
13:70000	YML098W	YGL255W	795.711	805.272	937.611
13:70000	YML098W	YGR061C	-247.349	-245.381	-100.692
13:70000	YML098W	YFR006W	-968.848	-965.935	-963.98
13:70000	YML098W	YER132C	228.742	237.15	296.839
13:70000	YML092C	YDR481C	-685.976	-675.076	-554.527
13:70000	YML092C	YML035C	-499.267	-487.646	-382.122
13:70000	YML092C	YIL160C	545.535	546.658	552.591
13:70000	YML092C	YDR515W	406.623	407.663	448.888
13:70000	YML092C	YBR296C	786.498	791.494	802.224
13:70000	YML092C	YOL011W	-368.055	-362.715	-261.409
13:70000	YML092C	YER103W	316.313	322.37	517.912
13:70000	YML092C	YCR059C	-221.247	-213.6	-47.9559
13:70000	YML092C	YJL088W	1047.93	1057.89	1092.86
13:70000	YML092C	YCR098C	407.33	418.077	500.881
13:70000	YML092C	YGL038C	-643.562	-637.674	-501.71
13:70000	YML092C	YPL272C	252.499	252.678	312.007
13:70000	YML092C	YNL073W	-319.84	-314.113	-266.806
13:70000	YML092C	YOL064C	-422.686	-420.519	-378.412
13:70000	YML092C	YLR267W	718.081	721.644	816.845
13:70000	YML092C	YDR502C	-498.264	-495.488	-470.337
13:70000	YML092C	YLR410W	-426.276	-419.647	-364.613
13:70000	YML092C	YGR223C	-542.366	-532.244	-261.1
13:70000	YML092C	YER150W	518.888	528.356	697.059
13:70000	YML092C	YER054C	956.092	958.98	1054.58
13:70000	YML092C	YER073W	639.182	643.75	797.793
13:70000	YML092C	YKL091C	323.159	326.917	448.098
13:70000	YML092C	YGR233C	-167.136	-156.934	-84.485
13:70000	YML092C	YAL026C	-856.674	-849.255	-829.903
13:70000	YML092C	YLR179C	-972.99	-965.448	-950.521
13:70000	YML092C	YHR103W	-627.673	-627.242	-514.859
13:70000	YML092C	YDL181W	300.063	304.82	423.591
13:70000	YML092C	YBR067C	-293.496	-287.876	-293.368
13:70000	YML092C	YBL082C	-580.054	-578.392	-526.237
13:70000	YML092C	YOL152W	296.764	302.118	505.334
13:70000	YML092C	YDR369C	-395.899	-395.683	-391.473
13:70000	YML092C	YJL079C	-362.834	-359.73	-324.413
13:70000	YML092C	YML078W	-1324.59	-1323.99	-1171.08
13:70000	YML092C	YHR214C-E	87.3044	99.565	102.49
13:70000	YML092C	YFR044C	-1211.15	-1199.75	-1098.69
13:70000	YML092C	YER165W	-901.244	-900.279	-857.803
13:70000	YML092C	YML087C	175.853	182.184	267.433
13:70000	YML092C	YJR060W	-298.989	-291.508	-257.938

13:70000	YML092C	YJL012C	-556.998	-551.907	-555.861
13:70000	YML092C	YDR309C	-202.736	-201.046	-183.098
13:70000	YML092C	YLR348C	-89.1747	-87.7723	-79.0297
13:70000	YML092C	YPR035W	-1494.92	-1490.01	-1493.62
13:70000	YML092C	YDL052C	-848.941	-843.157	-829.248
13:70000	YML092C	YMR240C	-386.332	-377.286	-369.444
13:70000	YML092C	YHR183W	-870.355	-867.01	-765.798
13:70000	YML092C	YHR136C	857.396	865.657	896.661
13:70000	YML092C	YJL117W	-569.117	-561.627	-564.656
13:70000	YML092C	YHR007C	-855.665	-847.768	-819.847
13:70000	YML092C	YER060W-A	-107.097	-99.5646	-86.9394
13:70000	YML092C	YOR163W	-660.267	-652.08	-658.2
13:70000	YML092C	YMR300C	-20.9866	-8.89582	316.836
13:70000	YML092C	YIL111W	-93.0478	-82.9697	2.72798
13:70000	YML092C	YHR047C	-410.577	-403.16	-334.353
13:70000	YML092C	YPL268W	-691.17	-682.928	-612.843
13:70000	YML092C	YHR162W	-889.629	-881.933	-842.144
13:70000	YML092C	YNL218W	-588.398	-580.909	-584.603
13:70000	YML092C	YBL043W	429.57	431.079	464.116
13:70000	YML092C	YKL189W	-272.582	-269.678	-246.728
13:70000	YML092C	YDR019C	260.931	264.926	286.976
13:70000	YML092C	YOR374W	744.916	747.227	939.933
13:70000	YML092C	YPL052W	352.435	356.495	474.011
13:70000	YML092C	YAR071W	460.705	462.694	471.059
13:70000	YML092C	YDR529C	-677.63	-666.898	-510.18
13:70000	YML092C	YML047C	405.969	415.055	449.778
13:70000	YML092C	YGR243W	654.526	655.655	790.667
13:70000	YML092C	YPL014W	836.482	842.101	1056.63
13:70000	YML092C	YHR022C	733.48	734.467	787.511
13:70000	YML092C	YML085C	-1118.93	-1115.25	-1115.9
13:70000	YML092C	YOR274W	-78.8418	-73.458	25.0777
13:70000	YML092C	YNL258C	-962.269	-954.725	-841.644
13:70000	YML092C	YPR074C	-1224.76	-1224.26	-1188.78
13:70000	YML092C	YDR111C	-164.666	-160.478	-155.874
13:70000	YML092C	YML121W	-604.853	-592.905	-340.544
13:70000	YML092C	YKL120W	417.986	427.985	496.829
13:70000	YML092C	YKL051W	217.006	225.138	228.107
13:70000	YML092C	YOR375C	-1413.84	-1401.94	-1407.66
13:70000	YML092C	YLL056C	-352.487	-349.393	-345.51
13:70000	YML092C	YOR032W-A	-374.451	-367.58	-373.481
13:70000	YML092C	YER081W	-240.371	-230	-223.665
13:70000	YML092C	YGR238C	-223.772	-216.951	-114.871
13:70000	YML092C	YOL058W	718.852	726.09	720.057
13:70000	YML092C	YML104C	-677.935	-665.817	-666.082
13:70000	YML092C	YER072W	-801.191	-793.538	-785.239
13:70000	YML092C	YPL018W	323.772	329.157	384.441
13:70000	YML092C	YGR007W	-1006.1	-997.676	-1003.57

13:70000	YML092C	YLR121C	394.397	398.371	522.715
13:70000	YML092C	YNL141W	1169.91	1171.99	1186.89
13:70000	YML092C	YIL120W	-221.075	-217.336	-220.329
13:70000	YML092C	YGL255W	723.795	729.559	928.814
13:70000	YML092C	YBR093C	214.691	217.262	241.567
13:70000	YML092C	YGR061C	-121.537	-115.633	-100.701
13:70000	YML092C	YDR281C	552.424	562.9	699.874
13:70000	YML092C	YER132C	-28.812	-20.9887	297.791
13:70000	YML110C	YDR481C	-652.119	-647.62	-568.419
13:70000	YML110C	YML035C	-478.707	-467.287	-375.447
13:70000	YML110C	YDR515W	332.123	340.616	446.217
13:70000	YML110C	YBR296C	782.135	787.015	782.468
13:70000	YML110C	YHR005C	-585.535	-584.176	-380.066
13:70000	YML110C	YOL011W	-381.25	-380.916	-260.025
13:70000	YML110C	YOR045W	-678.88	-674.12	-671.441
13:70000	YML110C	YJL088W	1022.2	1032	1085.9
13:70000	YML110C	YCR098C	431.855	441.988	485.579
13:70000	YML110C	YER069W	278.062	281.506	363.982
13:70000	YML110C	YPL272C	223.702	233.456	313.309
13:70000	YML110C	YNL073W	-466.154	-456.39	-256.702
13:70000	YML110C	YOL064C	-402.428	-390.554	-368.265
13:70000	YML110C	YDR349C	-553.932	-553.548	-259.553
13:70000	YML110C	YDR502C	-548.631	-541.901	-460.468
13:70000	YML110C	YIR031C	916.112	918.506	942.027
13:70000	YML110C	YLR410W	-451.174	-441.756	-360.798
13:70000	YML110C	YML096W	-37.4484	-26.0455	9.46204
13:70000	YML110C	YDL182W	78.6218	87.1646	209.164
13:70000	YML110C	YER150W	597.123	599.966	692.203
13:70000	YML110C	YFL004W	-678.576	-671.655	-669.604
13:70000	YML110C	YCL027W	-47.8118	-43.086	107.877
13:70000	YML110C	YER073W	556.504	566.556	791.795
13:70000	YML110C	YGR031W	-605.391	-602.655	-251.811
13:70000	YML110C	YGR233C	-150.384	-148.829	-94.1528
13:70000	YML110C	YBL016W	-270.852	-264.443	57.8361
13:70000	YML110C	YAL026C	-823.243	-816.921	-819.567
13:70000	YML110C	YLR179C	-963.163	-962.262	-916.154
13:70000	YML110C	YDL181W	291.71	297.387	419.14
13:70000	YML110C	YBR067C	-297.111	-295.048	-286.263
13:70000	YML110C	YML098W	-432.898	-424.362	-309.324
13:70000	YML110C	YDR369C	-415.104	-409.654	-389.135
13:70000	YML110C	YHR199C-A	-193.53	-190.234	-189.006
13:70000	YML110C	YMR236W	-955.167	-951.772	-852.423
13:70000	YML110C	YML078W	-1302.27	-1293.87	-1151.46
13:70000	YML110C	YNL244C	-479.229	-471.169	-477.139
13:70000	YML110C	YHR214C-E	94.5404	106.091	100.201
13:70000	YML110C	YER165W	-914.71	-906.041	-836.941
13:70000	YML110C	YML087C	130.593	133.431	270.29

13:70000	YML110C	YJL012C	-567.838	-565.369	-557.992
13:70000	YML110C	YER055C	-252.149	-240.889	-45.4575
13:70000	YML110C	YNR028W	-484.288	-480.843	-427.745
13:70000	YML110C	YDR309C	-172.464	-169.328	-170.047
13:70000	YML110C	YDL052C	-814.033	-806.813	-812.064
13:70000	YML110C	YLL018C-A	-169.573	-168.696	-77.4478
13:70000	YML110C	YCR089W	-158.812	-152.941	25.2663
13:70000	YML110C	YML092C	-753.327	-748.044	-591.904
13:70000	YML110C	YMR240C	-367.012	-355.4	-359.028
13:70000	YML110C	YHR183W	-1028.67	-1025.68	-754.269
13:70000	YML110C	YNL279W	220.633	229.205	370.204
13:70000	YML110C	YHR136C	829.505	831.148	874.955
13:70000	YML110C	YJL117W	-571.907	-563.343	-569.479
13:70000	YML110C	YHR007C	-877.24	-871.497	-789.11
13:70000	YML110C	YOR163W	-646.889	-638.243	-644.377
13:70000	YML110C	YIL111W	-35.6169	-32.4032	-0.0715151
13:70000	YML110C	YOR317W	-149.668	-144.775	8.60129
13:70000	YML110C	YPL268W	-672.981	-665.982	-605.227
13:70000	YML110C	YIL050W	-72.8871	-72.6241	20.9169
13:70000	YML110C	YHR162W	-847.693	-835.717	-815.735
13:70000	YML110C	YNL218W	-579.907	-574.295	-578.959
13:70000	YML110C	YBL043W	439.811	440.813	471.329
13:70000	YML110C	YDR019C	279.63	291.244	292.999
13:70000	YML110C	YHR084W	-149.459	-142.152	88.4986
13:70000	YML110C	YOR212W	-681.238	-681.192	-567.756
13:70000	YML110C	YIL051C	-1206.51	-1194.51	-1167.85
13:70000	YML110C	YML119W	-413.331	-407.462	-384.328
13:70000	YML110C	YDR529C	-576.225	-568.44	-496.98
13:70000	YML110C	YPL110C	-118.603	-107.788	-18.995
13:70000	YML110C	YML047C	343.383	355.48	458.986
13:70000	YML110C	YHR013C	-857.214	-853.538	-820.971
13:70000	YML110C	YML085C	-1153.3	-1143.91	-1101.37
13:70000	YML110C	YOR274W	-54.895	-52.7514	34.1813
13:70000	YML110C	YLR452C	-189.515	-181.681	150.706
13:70000	YML110C	YPL061W	-12.8735	-10.6153	31.8257
13:70000	YML110C	YCL055W	-124.18	-115.92	10.0033
13:70000	YML110C	YPL156C	14.4945	21.4364	26.8894
13:70000	YML110C	YDR111C	-159.704	-153.252	-145.501
13:70000	YML110C	YNR044W	1.20769	7.45695	217.165
13:70000	YML110C	YKL120W	420.881	428.173	505.215
13:70000	YML110C	YKL051W	188.616	199.623	231.112
13:70000	YML110C	YOR375C	-1391.72	-1379.83	-1382.36
13:70000	YML110C	YLL056C	-422.228	-416.739	-343.325
13:70000	YML110C	YBR083W	2.05124	6.93881	91.6385
13:70000	YML110C	YER081W	-258.044	-249.147	-215.745
13:70000	YML110C	YIR028W	1250.36	1252.89	1265.97
13:70000	YML110C	YPR139C	-620.155	-618.895	-619.059

13:70000	YML110C	YGR238C	-248.793	-237.078	-109.864
13:70000	YML110C	YOL058W	718.06	727.009	720.91
13:70000	YML110C	YML104C	-668.968	-657.394	-657.47
13:70000	YML110C	YER072W	-817.946	-813.83	-777.206
13:70000	YML110C	YML079W	-829.315	-818.195	-719.541
13:70000	YML110C	YPL018W	338.791	348.325	385.915
13:70000	YML110C	YGR007W	-996.67	-990.22	-992.116
13:70000	YML110C	YNL141W	1168.86	1178	1176.24
13:70000	YML110C	YIL120W	-221.309	-213.752	-215.242
13:70000	YML110C	YGR061C	-198.584	-193.009	-97.6725
13:70000	YML110C	YFR006W	-954.513	-949.548	-949.037
13:70000	YML104C	YDR481C	-560.352	-550.926	-554.527
13:70000	YML104C	YML035C	-386.237	-378.109	-382.122
13:70000	YML104C	YIL160C	513.234	524.849	552.591
13:70000	YML104C	YHL032C	-485.458	-475.468	-469.65
13:70000	YML104C	YBR295W	64.1955	70.1028	66.7691
13:70000	YML104C	YGR088W	1102.8	1106.51	1155.43
13:70000	YML104C	YDL085W	687.213	698.84	720.511
13:70000	YML104C	YPR184W	579.755	587.459	622.358
13:70000	YML104C	YML124C	-1282.15	-1272.32	-1238.09
13:70000	YML104C	YER103W	480.878	492.039	517.912
13:70000	YML104C	YCR059C	-97.9862	-86.6472	-47.9559
13:70000	YML104C	YJL088W	1089.13	1098.72	1092.86
13:70000	YML104C	YCR098C	493.254	501.499	500.881
13:70000	YML104C	YCR011C	-855.278	-854.435	-848.813
13:70000	YML104C	YCR073C	793.881	803.186	802.588
13:70000	YML104C	YJR032W	-455.41	-449.356	-437.607
13:70000	YML104C	YPL272C	308.612	315.959	312.007
13:70000	YML104C	YDL222C	851.878	859.177	894.438
13:70000	YML104C	YNL073W	-280.221	-275.316	-266.806
13:70000	YML104C	YOL064C	-380.095	-372.349	-378.412
13:70000	YML104C	YDR349C	-283.573	-274.92	-269.245
13:70000	YML104C	YGR166W	450.097	459.24	462.137
13:70000	YML104C	YPL019C	80.7747	91.2929	93.4827
13:70000	YML104C	YDR502C	-474.644	-465.243	-470.337
13:70000	YML104C	YDR453C	712.247	717.954	733.53
13:70000	YML104C	YLR410W	-381.671	-375.085	-364.613
13:70000	YML104C	YGR223C	-264.99	-255.474	-261.1
13:70000	YML104C	YER150W	655.336	666.602	697.059
13:70000	YML104C	YER054C	1014.84	1024.86	1054.58
13:70000	YML104C	YER073W	783.751	789.969	797.793
13:70000	YML104C	YMR196W	582.264	584.214	623.525
13:70000	YML104C	YKL084W	-305.893	-296.528	-275.807
13:70000	YML104C	YKL091C	371.138	383.347	448.098
13:70000	YML104C	YGR233C	-113.038	-104.486	-84.485
13:70000	YML104C	YAL026C	-830.507	-823.893	-829.903
13:70000	YML104C	YLR179C	-973.772	-962.078	-950.521

13:70000	YML104C	YHR103W	-528.628	-516.969	-514.859
13:70000	YML104C	YML070W	-879.686	-873.184	-847.454
13:70000	YML104C	YDL181W	382.106	392.279	423.591
13:70000	YML104C	YBR067C	-295.318	-287.308	-293.368
13:70000	YML104C	YBL082C	-532.732	-528.686	-526.237
13:70000	YML104C	YOL152W	495.235	501.093	505.334
13:70000	YML104C	YEL052W	-626.167	-622.278	-597.364
13:70000	YML104C	YJL079C	-325.334	-320.385	-324.413
13:70000	YML104C	YML078W	-1178.55	-1168.52	-1171.08
13:70000	YML104C	YNL244C	-504.453	-494.408	-495.604
13:70000	YML104C	YHR214C-E	96.4681	108.64	102.49
13:70000	YML104C	YER165W	-864.961	-853.889	-857.803
13:70000	YML104C	YML087C	248.549	259.172	267.433
13:70000	YML104C	YJR060W	-268.026	-257.112	-257.938
13:70000	YML104C	YJL012C	-572.742	-562.99	-555.861
13:70000	YML104C	YBR105C	-53.554	-51.5855	-53.0364
13:70000	YML104C	YER055C	-49.1307	-46.2779	-42.7508
13:70000	YML104C	YLR193C	-570.656	-564.988	-557.658
13:70000	YML104C	YAL012W	-767.615	-764.09	-766.649
13:70000	YML104C	YCL049C	-233.335	-227.204	-232.791
13:70000	YML104C	YNR028W	-440.824	-434.779	-435.641
13:70000	YML104C	YIR027C	1198.91	1201.56	1201.48
13:70000	YML104C	YLR348C	-82.0363	-75.048	-79.0297
13:70000	YML104C	YPR035W	-1493.75	-1488.77	-1493.62
13:70000	YML104C	YNL100W	-96.9033	-93.8183	-17.4786
13:70000	YML104C	YOR066W	-564.226	-562.388	-563.756
13:70000	YML104C	YML128C	657.179	663.584	711.089
13:70000	YML104C	YMR240C	-371.739	-364.323	-369.444
13:70000	YML104C	YJL213W	395.095	401.648	406.695
13:70000	YML104C	YGR156W	1.96771	5.7945	31.2766
13:70000	YML104C	YHR136C	874.677	880.278	896.661
13:70000	YML104C	YJL117W	-579.524	-567.508	-564.656
13:70000	YML104C	YHR007C	-838.859	-831.149	-819.847
13:70000	YML104C	YER060W-A	-101.209	-90.7415	-86.9394
13:70000	YML104C	YDL073W	-649.393	-643.533	-599.599
13:70000	YML104C	YOR163W	-665.7	-654.5	-658.2
13:70000	YML104C	YMR300C	279.095	288.522	316.836
13:70000	YML104C	YNL194C	1086.67	1094.57	1128.93
13:70000	YML104C	YER043C	-961.437	-951.653	-947.421
13:70000	YML104C	YIL136W	729.053	738.152	796.139
13:70000	YML104C	YHR047C	-379.487	-367.408	-334.353
13:70000	YML104C	YPL268W	-629.927	-619.216	-612.843
13:70000	YML104C	YIL050W	-28.524	-19.9597	13.8338
13:70000	YML104C	YHR162W	-854.03	-841.941	-842.144
13:70000	YML104C	YCL042W	77.6281	82.991	99.2146
13:70000	YML104C	YLL055W	133.222	144.619	163.049
13:70000	YML104C	YNL218W	-591.573	-583.013	-584.603

13:70000	YML104C	YDR019C	283.227	292.433	286.976
13:70000	YML104C	YOR374W	912.285	915.445	939.933
13:70000	YML104C	YOL016C	-234.21	-226.205	-205.967
13:70000	YML104C	YGR189C	-289.141	-278.875	-275.208
13:70000	YML104C	YMR296C	-763.005	-760.193	-715.474
13:70000	YML104C	YPL052W	466.053	477.903	474.011
13:70000	YML104C	YDR529C	-562.246	-550.104	-510.18
13:70000	YML104C	YDL090C	-519.268	-518.716	-517.949
13:70000	YML104C	YML047C	439.91	445.216	449.778
13:70000	YML104C	YGR243W	748.957	756.904	790.667
13:70000	YML104C	YPL265W	-564.62	-561.762	-554.917
13:70000	YML104C	YPL014W	1026.47	1037.82	1056.63
13:70000	YML104C	YMR177W	-252.237	-241.376	-203.444
13:70000	YML104C	YOR274W	6.05135	15.2565	25.0777
13:70000	YML104C	YGL234W	-158.465	-151.519	-135.61
13:70000	YML104C	YIL008W	-11.7831	-8.87267	-0.123519
13:70000	YML104C	YLR180W	772.346	781.031	786.53
13:70000	YML104C	YCL064C	-339.059	-336.026	-329.131
13:70000	YML104C	YPR074C	-1208.62	-1198.38	-1188.78
13:70000	YML104C	YDR111C	-164.289	-153.742	-155.874
13:70000	YML104C	YML110C	-937.224	-936.302	-927.904
13:70000	YML104C	YML121W	-351.566	-347.284	-340.544
13:70000	YML104C	YOR316C	-647.393	-644.599	-643.532
13:70000	YML104C	YKL120W	482.339	491.76	496.829
13:70000	YML104C	YKL051W	224.429	232.003	228.107
13:70000	YML104C	YOR375C	-1413.94	-1402.15	-1407.66
13:70000	YML104C	YLL056C	-351.869	-342.123	-345.51
13:70000	YML104C	YOR032W-A	-374.446	-368.599	-373.481
13:70000	YML104C	YER081W	-226.869	-220.148	-223.665
13:70000	YML104C	YOR247W	-944.301	-938.708	-940.957
13:70000	YML104C	YPL186C	640.427	649.573	690.059
13:70000	YML104C	YIR028W	1260.42	1266.45	1268.72
13:70000	YML104C	YGR238C	-123.058	-115.671	-114.871
13:70000	YML104C	YCL040W	331.897	333.989	364.496
13:70000	YML104C	YOL058W	711.681	722.627	720.057
13:70000	YML104C	YER072W	-807.237	-796.366	-785.239
13:70000	YML104C	YPL018W	363.274	363.689	384.441
13:70000	YML104C	YNR001C	-495.935	-493.441	-464.64
13:70000	YML104C	YGR007W	-1030.95	-1018.71	-1003.57
13:70000	YML104C	YLR178C	539.462	541.95	570.816
13:70000	YML104C	YLR164W	296.412	304.114	328.124
13:70000	YML104C	YLR121C	492.658	503.371	522.715
13:70000	YML104C	YPL004C	-221.42	-213.518	-165.954
13:70000	YML104C	YNL141W	1175.23	1186.89	1186.89
13:70000	YML104C	YIL120W	-224.738	-216.192	-220.329
13:70000	YML104C	YGL255W	896.739	907.52	928.814
13:70000	YML104C	YGR061C	-135.368	-124.679	-100.701

13:70000	YML104C	YFR006W	-955.435	-951.763	-952.8
13:70000	YML104C	YDR281C	678.047	683.756	699.874
13:70000	YML104C	YER132C	276.848	289.047	297.791
15:150000	YOL090W	YER087C-B	-1003.77	-1001.46	-996.106
15:150000	YOL090W	YDL048C	455.167	457.109	472.873
15:150000	YOL090W	YDR447C	-564.922	-555.571	-560.121
15:150000	YOL090W	YKL195W	-39.39	-28.4945	7.01825
15:150000	YOL090W	YDR144C	125.986	135.088	163.654
15:150000	YOL090W	YJL185C	140.181	147.774	170.572
15:150000	YOL090W	YDR447C	-196.542	-194.293	-195.489
15:150000	YOL090W	YNR038W	49.7221	54.0072	128.444
15:150000	YOL090W	YJL165C	-293.925	-284.448	-247.078
15:150000	YOL090W	YKL096W	-459.571	-448.426	-449.227
15:150000	YOL090W	YNR055C	-637.079	-630.563	-613.467
15:150000	YOL090W	YHR197W	1031.96	1043.63	1067.97
15:150000	YOL090W	YOR084W	-87.3887	-76.2955	-61.8634
15:150000	YOL090W	YDR245W	-706.28	-701.875	-688.638
15:150000	YOL090W	YOR187W	-365.768	-360.964	-362.544
15:150000	YOL090W	YGL121C	570.619	575.807	575.156
15:150000	YOL090W	YOL028C	-9.68321	-7.38755	9.24702
15:150000	YOL090W	YGR110W	252.363	258.908	278.771
15:150000	YOL090W	YKL167C	-85.5316	-79.591	-75.189
15:150000	YOL090W	YPR117W	-461.646	-457.215	-439.564
15:150000	YOL090W	YCL054W	701.399	710.104	736.774
15:150000	YOL090W	YHR128W	-414.612	-404.348	-369.797
15:150000	YOL090W	YOL077W-A	-308.972	-297.958	-301.899
15:150000	YOL090W	YGR083C	-402.76	-395.564	-323.79
15:150000	YOL090W	YBR084C-A	-297.549	-290.65	-294.694
15:150000	YOL090W	YKL194C	74.8333	85.3236	103.299
15:150000	YOL090W	YDR450W	-787.512	-781.658	-779.303
15:150000	YOL090W	YKL085W	-11.9848	-4.40246	73.1835
15:150000	YOL090W	YLR029C	-919.364	-910.37	-914.98
15:150000	YOL090W	YML091C	368.882	380.166	410.013
15:150000	YOL090W	YMR128W	869.63	880.628	891.337
15:150000	YOL090W	YMR131C	929.402	935.985	933.607
15:150000	YOL090W	YNL055C	-1134.25	-1122.41	-1126.23
15:150000	YOL090W	YDL085W	680.077	680.69	684.234
15:150000	YOL090W	YHR065C	677.774	689.238	731.229
15:150000	YOL090W	YOL036W	-661.034	-655.087	-660.247
15:150000	YOL090W	YML125C	-306.394	-302.6	-270.46
15:150000	YOL090W	YJR123W	-935.048	-933.065	-925.272
15:150000	YOL090W	YER103W	422.766	431.265	504.452
15:150000	YOL090W	YKL110C	589.413	596.1	609.906
15:150000	YOL090W	YJR121W	-731.067	-719.249	-668.132
15:150000	YOL090W	YNL313C	309.74	311.998	336.03
15:150000	YOL090W	YMR301C	-167.508	-161.898	-142.692
15:150000	YOL090W	YEL036C	-153.783	-144.097	-94.7133

15:150000	YOL090W	YCR059C	-101.044	-94.9704	-59.0772
15:150000	YOL090W	YML093W	925.923	937.351	960.754
15:150000	YOL090W	YLL028W	-241.546	-232.608	-238.644
15:150000	YOL090W	YCR073C	740.956	743.374	764.784
15:150000	YOL090W	YOL109W	-487.415	-477.077	-468.128
15:150000	YOL090W	YGL031C	-841.808	-836.775	-837.685
15:150000	YOL090W	YBL072C	-1232.8	-1229.14	-1219.56
15:150000	YOL090W	YGL038C	-543.101	-536.67	-499.772
15:150000	YOL090W	YKR057W	-215.338	-212.181	-215.014
15:150000	YOL090W	YPL189W	-166.505	-160.792	-158.051
15:150000	YOL090W	YKR044W	151.157	154.458	226.036
15:150000	YOL090W	YPR175W	-314.717	-310.806	-298.098
15:150000	YOL090W	YCR083W	-361.294	-357.067	-351.884
15:150000	YOL090W	YGR205W	363.996	372.693	405.08
15:150000	YOL090W	YER007C-A	-755.978	-750.964	-743.382
15:150000	YOL090W	YOL061W	-310.481	-304.498	-288.714
15:150000	YOL090W	YBL068W	380.379	381.778	391.344
15:150000	YOL090W	YHR129C	-368.827	-361.676	-359.393
15:150000	YOL090W	YJL109C	945.05	955.886	957.122
15:150000	YOL090W	YER149C	-431.374	-429.355	-418.579
15:150000	YOL090W	YMR239C	583.105	594.642	643.119
15:150000	YOL090W	YNR066C	634.286	641.77	646.347
15:150000	YOL090W	YNL073W	-274.751	-264.452	-256.733
15:150000	YOL090W	YDL031W	329.2	338.609	363.269
15:150000	YOL090W	YDR337W	102.181	110.905	117.104
15:150000	YOL090W	YGL111W	504.922	515.468	528.225
15:150000	YOL090W	YHR072W	-378.777	-367.455	-340.974
15:150000	YOL090W	YBR023C	-291.124	-278.999	-266.847
15:150000	YOL090W	YDR349C	-295.614	-287.135	-260.251
15:150000	YOL090W	YLR222C	1023.75	1034.6	1090.47
15:150000	YOL090W	YER006W	929.704	938.052	935.328
15:150000	YOL090W	YCL059C	767.738	773.523	768.232
15:150000	YOL090W	YGR166W	405.012	410.981	441.867
15:150000	YOL090W	YBR121C	-925.825	-925.742	-918.296
15:150000	YOL090W	YHR085W	413.28	416.072	497.993
15:150000	YOL090W	YHR062C	-113.005	-109.012	-53.0827
15:150000	YOL090W	YDR490C	-145.111	-141.539	-104.572
15:150000	YOL090W	YPR140W	-385.574	-382.763	-356.708
15:150000	YOL090W	YGR208W	-578.094	-577.271	-533.708
15:150000	YOL090W	YPR010C	1083.99	1088.78	1084.5
15:150000	YOL090W	YER126C	769.399	780.508	811.81
15:150000	YOL090W	YGL189C	-880.679	-872.122	-877.833
15:150000	YOL090W	YPL132W	9.71022	17.5599	27.9996
15:150000	YOL090W	YNL113W	339.985	348.826	405.865
15:150000	YOL090W	YAR002C-A	-1297.32	-1289.87	-1259.96
15:150000	YOL090W	YOL127W	-1038.63	-1034.59	-1030.3
15:150000	YOL090W	YKL161C	773.827	776.71	783.144

15:150000	YOL090W	YPR191W	-155.673	-147.251	-55.308
15:150000	YOL090W	YGL190C	-384.254	-374.235	-372.993
15:150000	YOL090W	YOL022C	220.274	226.079	237.45
15:150000	YOL090W	YNL045W	-785.388	-780.222	-741.743
15:150000	YOL090W	YHR143W-A	85.9551	93.3119	169.069
15:150000	YOL090W	YIL091C	710.401	717.496	771.551
15:150000	YOL090W	YEL047C	-809.05	-804.851	-807.618
15:150000	YOL090W	YAL005C	511.149	519.005	570.093
15:150000	YOL090W	YAL029C	-369.457	-362.422	-308.254
15:150000	YOL090W	YJL131C	-12.3392	-3.36927	83.7341
15:150000	YOL090W	YML026C	-231.569	-228.229	-228.072
15:150000	YOL090W	YKL072W	218.772	230.177	238.242
15:150000	YOL090W	YLR204W	-26.1219	-20.6916	-21.0829
15:150000	YOL090W	YBR111C	-895.472	-894.918	-856.955
15:150000	YOL090W	YPR060C	-404.133	-399.015	-345.634
15:150000	YOL090W	YIR012W	272.318	279.752	318.44
15:150000	YOL090W	YKL084W	-292.44	-281.31	-261.177
15:150000	YOL090W	YPL043W	1094.44	1106.02	1119.36
15:150000	YOL090W	YLR150W	-1142.28	-1138.27	-1137.67
15:150000	YOL090W	YKR016W	-332.459	-331.172	-225.946
15:150000	YOL090W	YDL235C	-505.421	-493.61	-483.566
15:150000	YOL090W	YGR217W	-142.132	-136.205	-53.9934
15:150000	YOL090W	YGL128C	-674.009	-665.235	-605.513
15:150000	YOL090W	YJL137C	-58.2948	-57.4746	-8.1307
15:150000	YOL090W	YJL189W	-506.452	-500.907	-505.938
15:150000	YOL090W	YJR097W	769.413	774.985	869.288
15:150000	YOL090W	YER177W	-1234.1	-1225.9	-1214.25
15:150000	YOL090W	YHR207C	-705.195	-698.682	-676.033
15:150000	YOL090W	YFL054C	365.915	377.994	384.389
15:150000	YOL090W	YML070W	-862.085	-857.646	-825.231
15:150000	YOL090W	YGR175C	-553.132	-545.204	-544.958
15:150000	YOL090W	YKR026C	-362.186	-355.201	-293.135
15:150000	YOL090W	YML063W	-634.912	-629.663	-626.864
15:150000	YOL090W	YJR054W	-434.79	-425.672	-430.987
15:150000	YOL090W	YEL058W	-612.653	-608.651	-608.022
15:150000	YOL090W	YDR211W	-150.23	-149.408	-86.9928
15:150000	YOL090W	YLR175W	806.826	815.714	813.977
15:150000	YOL090W	YDL181W	318.743	328.071	385.816
15:150000	YOL090W	YKR094C	-443.213	-442.453	-441.83
15:150000	YOL090W	YEL050C	-71.2497	-61.0506	-64.8115
15:150000	YOL090W	YPL084W	-684.134	-671.939	-589.058
15:150000	YOL090W	YDR018C	385.139	388.821	411.753
15:150000	YOL090W	YIL046W	78.0315	87.579	99.2725
15:150000	YOL090W	YHR089C	736.923	744.358	739.389
15:150000	YOL090W	YKL193C	-281.95	-279.397	-231.954
15:150000	YOL090W	YOL152W	457.295	468.786	493.623
15:150000	YOL090W	YLR213C	255.755	265.223	279.25

15:150000	YOL090W	YHR176W	-182.346	-172.8	-142.073
15:150000	YOL090W	YKR063C	360.468	364.614	449.489
15:150000	YOL090W	YMR319C	265.643	274.146	301.273
15:150000	YOL090W	YBL072C	-734.248	-726.969	-730.711
15:150000	YOL090W	YNR012W	583.299	590.787	631.377
15:150000	YOL090W	YNR034W	124.596	135.584	176.893
15:150000	YOL090W	YHR156C	-186.401	-176.299	-179.352
15:150000	YOL090W	YEL052W	-630.605	-625.664	-558.414
15:150000	YOL090W	YOR312C	-510.897	-510.533	-506.017
15:150000	YOL090W	YEL055C	81.8231	91.5745	110.701
15:150000	YOL090W	YDL213C	326.576	333.77	345.693
15:150000	YOL090W	YDR430C	64.9912	76.0489	91.2505
15:150000	YOL090W	YDR165W	853.717	865.222	873.95
15:150000	YOL090W	YMR229C	1093.29	1097.15	1096.5
15:150000	YOL090W	YMR264W	-956.609	-952.649	-953.305
15:150000	YOL090W	YLR295C	-135.515	-126.476	-60.3928
15:150000	YOL090W	YLR200W	-320.489	-309.424	-241.48
15:150000	YOL090W	YHR001W-A	-170.416	-159.53	-105.777
15:150000	YOL090W	YDR060W	1160.84	1171.37	1175.86
15:150000	YOL090W	YIL079C	493.61	500.673	544.979
15:150000	YOL090W	YPL118W	-366.816	-358.209	-356.876
15:150000	YOL090W	YDR341C	-392.073	-385.563	-389.267
15:150000	YOL090W	YBL015W	355.065	357.754	445.899
15:150000	YOL090W	YPR110C	416.469	425.032	430.796
15:150000	YOL090W	YGR034W	-209.983	-207.448	-206.787
15:150000	YOL090W	YKL004W	-178.91	-172.057	-163.575
15:150000	YOL090W	YBR037C	250.016	257.149	294.043
15:150000	YOL090W	YFR044C	-1045.78	-1033.79	-1034.81
15:150000	YOL090W	YOL041C	543.644	555.079	583.951
15:150000	YOL090W	YDR032C	-1050.22	-1049.37	-1047.23
15:150000	YOL090W	YFR011C	-72.0414	-66.9102	-56.7477
15:150000	YOL090W	YIL117C	100.884	102.815	103.115
15:150000	YOL090W	YLR382C	65.8845	77.8384	89.9716
15:150000	YOL090W	YER182W	81.8847	83.9307	140.555
15:150000	YOL090W	YPL183W-A	-28.6077	-20.9317	-20.2362
15:150000	YOL090W	YMR185W	604.126	613.658	693.542
15:150000	YOL090W	YOL080C	630.941	638.037	651.412
15:150000	YOL090W	YBR230C	565.707	570.387	654.005
15:150000	YOL090W	YOL081W	-567.065	-566.892	-546.882
15:150000	YOL090W	YNL098C	5.59135	15.349	72.0557
15:150000	YOL090W	YDR300C	322.854	323.664	358.021
15:150000	YOL090W	YAL036C	783.68	794.627	815.363
15:150000	YOL090W	YKL096W-A	-1646.92	-1634.88	-1624.64
15:150000	YOL090W	YOR046C	-797.524	-788.219	-759.011
15:150000	YOL090W	YNL175C	1262.24	1271.12	1266.28
15:150000	YOL090W	YMR165C	-457.382	-456.895	-456.162
15:150000	YOL090W	YDR046C	558.331	564.805	576.577

15:150000	YOL090W	YOR004W	1024.33	1035.49	1048.95
15:150000	YOL090W	YBR105C	-67.0857	-62.8009	-52.8485
15:150000	YOL090W	YPL207W	334.546	336.721	340.674
15:150000	YOL090W	YLR394W	-349.308	-346.175	-330.383
15:150000	YOL090W	YPL239W	178.299	180.924	199.31
15:150000	YOL090W	YOR206W	1243.32	1254.99	1266.89
15:150000	YOL090W	YLR355C	-228.3	-219.778	-214.95
15:150000	YOL090W	YNR036C	-396.586	-387.501	-391.859
15:150000	YOL090W	YMR114C	-425.176	-421.746	-351.384
15:150000	YOL090W	YPL175W	368.046	378.629	413.574
15:150000	YOL090W	YJR016C	-104.815	-93.7493	-93.0249
15:150000	YOL090W	YIL127C	693.322	703.492	740.201
15:150000	YOL090W	YLR002C	873.671	884.825	912.514
15:150000	YOL090W	YDL121C	-86.4518	-75.9419	-46.0484
15:150000	YOL090W	YCR030C	-485.083	-479.062	-402.172
15:150000	YOL090W	YMR271C	690.399	697.202	691.223
15:150000	YOL090W	YIR035C	-606.928	-597.352	-590.741
15:150000	YOL090W	YCL057C-A	-763.73	-752.687	-750.327
15:150000	YOL090W	YML024W	-857.162	-851.69	-851.368
15:150000	YOL090W	YDR339C	-284.153	-273.899	-224.791
15:150000	YOL090W	YER002W	11.9346	20.3128	92.933
15:150000	YOL090W	YGL187C	-116.214	-105.541	-12.0856
15:150000	YOL090W	YML042W	-35.0022	-26.9473	-9.70803
15:150000	YOL090W	YJL181W	-417.625	-406.65	-400.914
15:150000	YOL090W	YJL066C	-135.454	-133.36	-75.7766
15:150000	YOL090W	YNR033W	-376.902	-366.141	-361.659
15:150000	YOL090W	YGR062C	-312.607	-304.535	-272.362
15:150000	YOL090W	YLR196W	1071.52	1082.39	1109.26
15:150000	YOL090W	YLL026W	581.13	588.699	668.139
15:150000	YOL090W	YOR354C	-279.255	-270.282	-270.245
15:150000	YOL090W	YPL108W	81.2224	82.3752	134.841
15:150000	YOL090W	YPL015C	-577.708	-571.196	-539.352
15:150000	YOL090W	YER045C	-246.729	-234.888	-217.043
15:150000	YOL090W	YLR168C	94.3626	103.766	99.4457
15:150000	YOL090W	YBR104W	483.828	494.634	517.087
15:150000	YOL090W	YPL012W	1231.03	1241.97	1252.14
15:150000	YOL090W	YLR194C	-31.5602	-24.3671	-27.0541
15:150000	YOL090W	YIL033C	-265.781	-255.352	-149.526
15:150000	YOL090W	YKR017C	-58.7631	-47.3768	-36.4598
15:150000	YOL090W	YNL284C	-231.076	-219.946	-190.561
15:150000	YOL090W	YJL045W	85.6913	87.4451	85.7215
15:150000	YOL090W	YGL078C	1394.85	1405.85	1407.53
15:150000	YOL090W	YGR187C	430.771	438.231	497.82
15:150000	YOL090W	YHR092C	890.974	899.679	917.374
15:150000	YOL090W	YGR128C	1157.87	1168.85	1188.82
15:150000	YOL090W	YCL051W	-215.68	-208.701	-153.268
15:150000	YOL090W	YOR207C	534.515	542.408	557.487

15:150000	YOL090W	YPL211W	920.62	929.749	940.476
15:150000	YOL090W	YNL081C	7.51823	19.3055	46.3561
15:150000	YOL090W	YDR351W	23.7117	32.0104	44.5292
15:150000	YOL090W	YER131W	321.944	324.867	325.956
15:150000	YOL090W	YFL036W	-165.875	-157.386	-151.171
15:150000	YOL090W	YPL266W	639.893	647.505	648.099
15:150000	YOL090W	YLR203C	-343.989	-338.157	-313.041
15:150000	YOL090W	YGR156W	-24.1778	-19.2935	21.8455
15:150000	YOL090W	YPL119C-A	-78.3458	-71.5433	-58.8456
15:150000	YOL090W	YKL109W	888.559	894.826	894.776
15:150000	YOL090W	YLR301W	-931.591	-922.465	-921.826
15:150000	YOL090W	YDL051W	560.82	567.715	567.073
15:150000	YOL090W	YDL208W	532.716	542.177	554.587
15:150000	YOL090W	YHR007C	-778.94	-769.827	-771.572
15:150000	YOL090W	YDR161W	632.809	642.855	652.485
15:150000	YOL090W	YCR051W	-155.136	-150.396	-150.808
15:150000	YOL090W	YDR260C	-332.154	-325.497	-330.178
15:150000	YOL090W	YDR342C	860.937	870.791	903.275
15:150000	YOL090W	YHR141C	-799.951	-792.181	-797.481
15:150000	YOL090W	YEL024W	110.696	122.168	240.86
15:150000	YOL090W	YJR132W	131.651	137.612	170.409
15:150000	YOL090W	YOL034W	-376.979	-368.991	-372.152
15:150000	YOL090W	YGR220C	-134.043	-122.01	-119.167
15:150000	YOL090W	YHR009C	-708.457	-702.155	-706.484
15:150000	YOL090W	YMR308C	143.538	154.587	174.17
15:150000	YOL090W	YDR494W	15.956	22.9533	34.3865
15:150000	YOL090W	YOR005C	-645.029	-643.085	-605.097
15:150000	YOL090W	YOR243C	268.666	277.73	287.831
15:150000	YOL090W	YER156C	-331.29	-324.426	-305.689
15:150000	YOL090W	YOL119C	212.76	220.707	216.021
15:150000	YOL090W	YLR084C	-286.975	-281.944	-273.04
15:150000	YOL090W	YPL271W	-857.6	-848.759	-803.494
15:150000	YOL090W	YHR047C	-381.052	-369.964	-339.263
15:150000	YOL090W	YFR053C	1118.54	1120.53	1147.17
15:150000	YOL090W	YOR254C	-697.246	-692.5	-663.609
15:150000	YOL090W	YJL190C	-113.061	-111.341	-112.123
15:150000	YOL090W	YNL302C	-400.013	-391.777	-396.887
15:150000	YOL090W	YOR358W	-319.054	-312.616	-273.728
15:150000	YOL090W	YHL011C	316	319.446	320.192
15:150000	YOL090W	YLR409C	1013.54	1024.66	1052.71
15:150000	YOL090W	YJR077C	-752.905	-743.793	-739.234
15:150000	YOL090W	YMR206W	333.568	340.299	350.002
15:150000	YOL090W	YOR335C	-574.36	-569.312	-568.167
15:150000	YOL090W	YPR030W	143.847	149.647	172.331
15:150000	YOL090W	YDR132C	-424.283	-418.017	-388.641
15:150000	YOL090W	YPR132W	-943.785	-939.921	-934.315
15:150000	YOL090W	YDR204W	-409.648	-409.499	-345.824

15:150000	YOL090W	YPL053C	-757.923	-747.927	-752.766
15:150000	YOL090W	YIL158W	-181.155	-176.743	-127.296
15:150000	YOL090W	YMR211W	-210.538	-206.694	-196.874
15:150000	YOL090W	YLR372W	-250.239	-243.058	-230.53
15:150000	YOL090W	YMR267W	28.8945	38.116	55.9652
15:150000	YOL090W	YOR188W	-430.385	-419.56	-391.712
15:150000	YOL090W	YNL053W	245.907	254.148	285.868
15:150000	YOL090W	YGL209W	363.026	369.964	385.237
15:150000	YOL090W	YMR093W	566.596	575.303	652.608
15:150000	YOL090W	YDR171W	981.83	986.451	1072.2
15:150000	YOL090W	YKL093W	315.719	324.347	323.153
15:150000	YOL090W	YLR332W	-173.114	-166.011	-163.124
15:150000	YOL090W	YML106W	-730.386	-723.242	-722.594
15:150000	YOL090W	YJR041C	525.106	535.38	576.046
15:150000	YOL090W	YNL247W	-250.428	-244.735	-239.505
15:150000	YOL090W	YFL042C	-297.013	-296.357	-256.545
15:150000	YOL090W	YOR276W	-709.247	-706.755	-700.859
15:150000	YOL090W	YPL040C	-8.36775	0.252752	-3.86068
15:150000	YOL090W	YGL115W	-928.969	-921.229	-897.664
15:150000	YOL090W	YOL029C	-76.2297	-68.9103	-47.6055
15:150000	YOL090W	YGR189C	-261.451	-256.267	-252.064
15:150000	YOL090W	YPL104W	-119.059	-114.206	-110.477
15:150000	YOL090W	YHR106W	-740.691	-735.779	-726.518
15:150000	YOL090W	YPR172W	-445.151	-444.569	-418.751
15:150000	YOL090W	YBR029C	-227.494	-218.265	-175.759
15:150000	YOL090W	YMR230W	-173.676	-166.659	-171.108
15:150000	YOL090W	YMR296C	-712.603	-707.136	-680.19
15:150000	YOL090W	YLR146C	-202.565	-198.097	-166.551
15:150000	YOL090W	YGL147C	-317.591	-308.435	-310.15
15:150000	YOL090W	YOR286W	-324.166	-314.008	-319.898
15:150000	YOL090W	YNL115C	-337.098	-336.972	-289.348
15:150000	YOL090W	YOL056W	-518.792	-517.026	-513.223
15:150000	YOL090W	YOR311C	-147.013	-135.316	-112.377
15:150000	YOL090W	YPL236C	-305.119	-303.751	-250.556
15:150000	YOL090W	YER001W	-61.9943	-50.4698	-56.3477
15:150000	YOL090W	YPL052W	425.748	437.927	449.451
15:150000	YOL090W	YLR375W	-676.023	-667.144	-643.095
15:150000	YOL090W	YKL155C	22.1875	29.0835	26.3065
15:150000	YOL090W	YDR513W	-265.414	-254.787	-189.47
15:150000	YOL090W	YLL046C	205.133	206.038	216.824
15:150000	YOL090W	YJR080C	-8.86284	3.1368	33.8365
15:150000	YOL090W	YMR312W	-279.905	-273.908	-255.524
15:150000	YOL090W	YJL010C	547.646	556.138	591.732
15:150000	YOL090W	YGR174C	272.837	276.672	294.336
15:150000	YOL090W	YGL123W	-854.96	-848.85	-854.885
15:150000	YOL090W	YNL137C	-98.6763	-89.5974	-89.3264
15:150000	YOL090W	YOR312C	-633.827	-630.83	-628.866

15:150000	YOL090W	YHR088W	944.987	955.469	1000.46
15:150000	YOL090W	YJR122W	-188.196	-179.664	-174.779
15:150000	YOL090W	YCR034W	-266.91	-263.315	-262.718
15:150000	YOL090W	YNL227C	401.339	408.717	440.553
15:150000	YOL090W	YER130C	-300.805	-289.748	-283.334
15:150000	YOL090W	YOR220W	-154.61	-148.896	-36.0743
15:150000	YOL090W	YHR010W	-487.52	-485.083	-480.13
15:150000	YOL090W	YGR077C	-796.006	-792.723	-791.152
15:150000	YOL090W	YJL005W	-89.3303	-88.687	-39.4011
15:150000	YOL090W	YER082C	626.657	632.937	656.891
15:150000	YOL090W	YMR295C	-1202.78	-1196.6	-1202.46
15:150000	YOL090W	YDR263C	68.6392	71.2332	78.8151
15:150000	YOL090W	YKL087C	73.4292	79.4733	84.9873
15:150000	YOL090W	YMR072W	-948.544	-939.917	-943.487
15:150000	YOL090W	YER067W	1184.32	1184.68	1213.22
15:150000	YOL090W	YDR332W	-648.511	-645.982	-636.663
15:150000	YOL090W	YER049W	339.513	343.922	354.576
15:150000	YOL090W	YJL062W-A	-121.63	-112.587	-117.361
15:150000	YOL090W	YER074W	-606.602	-603.972	-597.25
15:150000	YOL090W	YBL030C	-713.974	-702.028	-672.254
15:150000	YOL090W	YPL014W	955.342	967.349	1001.98
15:150000	YOL090W	YDR277C	1051.01	1054.89	1053.75
15:150000	YOL090W	YGR138C	288.115	296.805	293.989
15:150000	YOL090W	YGR118W	-998.817	-993.019	-996.549
15:150000	YOL090W	YGL081W	131.048	138.882	177.675
15:150000	YOL090W	YDR421W	-337.79	-328.44	-299.037
15:150000	YOL090W	YOR096W	-451.245	-449.011	-440.045
15:150000	YOL090W	YOR293W	-25.2307	-20.9387	-19.8575
15:150000	YOL090W	YDR091C	333.601	341.783	348.609
15:150000	YOL090W	YKR076W	79.7938	84.6115	85.6422
15:150000	YOL090W	YGR021W	129.002	131.681	134.021
15:150000	YOL090W	YBR284W	292.932	299.347	345.009
15:150000	YOL090W	YGR200C	588.18	599.102	632.801
15:150000	YOL090W	YHR017W	-135.085	-132.492	-15.0462
15:150000	YOL090W	YMR157C	6.88075	9.86814	10.7625
15:150000	YOL090W	YNL133C	-409.651	-404.615	-402.427
15:150000	YOL090W	YDR296W	85.4983	93.8444	94.3082
15:150000	YOL090W	YOR001W	260.75	270.598	286.674
15:150000	YOL090W	YMR286W	-51.3774	-40.2188	-45.9824
15:150000	YOL090W	YLR327C	1238.32	1240.61	1258.62
15:150000	YOL090W	YDL079C	860.593	872.456	881.973
15:150000	YOL090W	YCR043C	-665.571	-657.524	-641.961
15:150000	YOL090W	YOR274W	-15.2925	-9.28285	12.5098
15:150000	YOL090W	YNR060W	574.66	579.976	578.022
15:150000	YOL090W	YGR244C	-273.35	-269.723	-270.014
15:150000	YOL090W	YFR032C-A	-513.556	-507.198	-512.107
15:150000	YOL090W	YDL166C	-127.494	-121.294	-69.0613

15:150000	YOL090W	YIL008W	-32.5048	-25.8973	9.47069
15:150000	YOL090W	YJR034W	157.404	164.498	168.298
15:150000	YOL090W	YPL156C	22.3265	32.6312	27.6411
15:150000	YOL090W	YBL086C	347.668	350.178	426.019
15:150000	YOL090W	YHR111W	-414.875	-407.593	-411.543
15:150000	YOL090W	YIL039W	-965.3	-955.838	-947.485
15:150000	YOL090W	YER117W	-481.305	-474.397	-479.07
15:150000	YOL090W	YBR191W	-645.843	-641.092	-641.837
15:150000	YOL090W	YJR063W	486.67	493.688	582.886
15:150000	YOL090W	YKR079C	847.771	852.959	878.88
15:150000	YOL090W	YPL089C	226.312	232.779	240.443
15:150000	YOL090W	YBR162W-A	-470.384	-464.011	-452.918
15:150000	YOL090W	YMR145C	120.742	131.682	126.101
15:150000	YOL090W	YDR420W	-334.766	-325.66	-296.045
15:150000	YOL090W	YBR025C	-675.43	-673.866	-669.191
15:150000	YOL090W	YIL021W	-839.586	-832.575	-804.87
15:150000	YOL090W	YLL034C	874.278	885.531	918.783
15:150000	YOL090W	YPR033C	-612.636	-608.759	-594.912
15:150000	YOL090W	YDR041W	-15.6145	-4.49391	-1.82923
15:150000	YOL090W	YAR029W	229.501	238.076	233.167
15:150000	YOL090W	YER025W	-288.97	-284.281	-288.317
15:150000	YOL090W	YKL120W	458.016	464.848	472.319
15:150000	YOL090W	YOL094C	-324.977	-315.824	-319.389
15:150000	YOL090W	YLR118C	-568.026	-562.98	-558.492
15:150000	YOL090W	YJL011C	280.585	286.508	392.172
15:150000	YOL090W	YNL061W	945.521	952.923	951.088
15:150000	YOL090W	YBR181C	-969.14	-962.578	-958.933
15:150000	YOL090W	YKL014C	951.758	961.989	971.746
15:150000	YOL090W	YDL081C	-702.111	-693.653	-699.642
15:150000	YOL090W	YBR185C	116.601	122.998	144.086
15:150000	YOL090W	YDR324C	869.451	880.277	914.531
15:150000	YOL090W	YHR208W	-9.62403	-0.440181	-1.44549
15:150000	YOL090W	YHL024W	882.817	894.061	978.633
15:150000	YOL090W	YJL069C	605.829	610.35	682.043
15:150000	YOL090W	YGL025C	-464.601	-455.82	-446.972
15:150000	YOL090W	YGR238C	-127.971	-125.842	-108.626
15:150000	YOL090W	YHR203C	-1272.57	-1264.94	-1270.04
15:150000	YOL090W	YMR143W	-772.57	-767.208	-770.129
15:150000	YOL090W	YBL061C	277.832	287.324	314.908
15:150000	YOL090W	YKL073W	-490.618	-489.405	-476.672
15:150000	YOL090W	YGL135W	-1194.04	-1191.34	-1191
15:150000	YOL090W	YER060W	126.578	136.124	153.043
15:150000	YOL090W	YDR037W	-266.209	-254.678	-251.427
15:150000	YOL090W	YNR001C	-490.556	-480.814	-419.701
15:150000	YOL090W	YDR135C	-805.005	-796.425	-800.065
15:150000	YOL090W	YGR007W	-949.608	-943.527	-948.352
15:150000	YOL090W	YEL040W	6.62823	8.11655	59.0256

15:150000	YOL090W	YLR083C	-420.926	-413.374	-399.834
15:150000	YOL090W	YGR207C	-557.617	-549.473	-549.275
15:150000	YOL090W	YJR090C	-673.106	-665.242	-667.75
15:150000	YOL090W	YJL112W	-183.66	-176.006	-180.499
15:150000	YOL090W	YMR310C	179.26	186.178	229.853
15:150000	YOL090W	YLR164W	282.952	288.06	312.318
15:150000	YOL090W	YGR255C	-275.089	-270.747	-236.935
15:150000	YOL090W	YLL012W	210.964	216.902	285.823
15:150000	YOL090W	YBR191W	-206.525	-198.571	-203.248
15:150000	YOL090W	YGR060W	-1048.5	-1036.41	-1015.42
15:150000	YOL090W	YLR121C	480.138	491.955	505.512
15:150000	YOL090W	YKR013W	-171.851	-159.878	-146.418
15:150000	YOL090W	YDR104C	-432.183	-428.088	-428.955
15:150000	YOL090W	YDR298C	-613.882	-602.964	-546.911
15:150000	YOL090W	YGL255W	817.739	828.228	869.405
15:150000	YOL090W	YBL092W	-845.912	-839.762	-845.437
15:150000	YOL090W	YPL203W	-137.277	-134.117	-36.1996
15:150000	YOL090W	YMR108W	-25.7021	-14.997	2.44411
15:150000	YOL090W	YFR006W	-922.117	-911.875	-908.059
15:150000	YOL090W	YPR127W	-444.489	-435.532	-433.262
15:150000	YOL090W	YFL041W-A	-298.224	-293.82	-227.557
15:150000	YOL090W	YIL145C	-238.966	-229.069	-192.619
15:150000	YOL090W	YER132C	187.561	193.783	267.898
15:150000	YOL090W	YOL030W	-1052.38	-1047.86	-1048.75
15:150000	YOL090W	YOR293W	-65.5269	-62.0135	-59.543
15:150000	YOL090W	YLL023C	-553.202	-550.752	-527.246
15:150000	YOL098C	YML035C	-436.467	-435.641	-394.766
15:150000	YOL098C	YDR447C	-706.949	-697.41	-597.027
15:150000	YOL098C	YDR447C	-296.371	-285.318	-210.388
15:150000	YOL098C	YNR055C	-676.447	-674.058	-632.012
15:150000	YOL098C	YOR187W	-432.491	-428.3	-383.198
15:150000	YOL098C	YGL121C	608.51	612.492	611.914
15:150000	YOL098C	YKL192C	-867.024	-860.525	-866.043
15:150000	YOL098C	YDL061C	-859.765	-849.014	-771.937
15:150000	YOL098C	YOL077W-A	-321.084	-310.646	-307.771
15:150000	YOL098C	YDL191W	-1155.97	-1144.88	-1096.5
15:150000	YOL098C	YBR084C-A	-392.324	-382.699	-314.882
15:150000	YOL098C	YAL003W	-764.665	-752.652	-680.427
15:150000	YOL098C	YDR450W	-943.03	-935.863	-812.715
15:150000	YOL098C	YLR029C	-1051.96	-1039.83	-971.148
15:150000	YOL098C	YIL052C	-517.794	-512.574	-448.486
15:150000	YOL098C	YMR131C	956.497	964.77	984.471
15:150000	YOL098C	YNL055C	-1196.66	-1186.35	-1192.1
15:150000	YOL098C	YPL131W	-784.177	-773.396	-753.205
15:150000	YOL098C	YDL085W	682.722	684.091	716.257
15:150000	YOL098C	YDL173W	-652.682	-650.002	-626.881
15:150000	YOL098C	YDR454C	-1172.3	-1165.79	-1139.84

15:150000	YOL098C	YHL001W	-246.624	-236.908	-171.562
15:150000	YOL098C	YJL016W	-7.12521	-4.83728	-6.85327
15:150000	YOL098C	YJR123W	-1042.82	-1033.08	-993.379
15:150000	YOL098C	YGR027C	-1107.87	-1096.64	-1021.42
15:150000	YOL098C	YDL229W	-86.193	-75.9543	16.6129
15:150000	YOL098C	YKL180W	-519.282	-513.118	-442.673
15:150000	YOL098C	YLR185W	-415.234	-405.315	-347.86
15:150000	YOL098C	YLL028W	-296.116	-293.991	-261.517
15:150000	YOL098C	YKL081W	-742.241	-730.961	-647.395
15:150000	YOL098C	YGL031C	-952.269	-940.378	-893.655
15:150000	YOL098C	YER013W	-776.144	-772.495	-775.033
15:150000	YOL098C	YGL013C	-732.269	-723.447	-589.903
15:150000	YOL098C	YBL072C	-1368.73	-1358.11	-1290.2
15:150000	YOL098C	YBL027W	-501.284	-498.809	-405.432
15:150000	YOL098C	YER102W	-668.483	-657.817	-610.485
15:150000	YOL098C	YGL189C	-1144.67	-1141.36	-1085.91
15:150000	YOL098C	YKR057W	-339.807	-332.878	-235.216
15:150000	YOL098C	YPL036W	120.605	126.231	437.952
15:150000	YOL098C	YGR085C	-119.736	-108.807	-58.9357
15:150000	YOL098C	YPL189W	-191.657	-181.547	-175.12
15:150000	YOL098C	YGR148C	-1058.03	-1047.68	-1002.26
15:150000	YOL098C	YGR214W	-290.664	-284.154	-223.859
15:150000	YOL098C	YER007C-A	-815.386	-807.899	-790.003
15:150000	YOL098C	YPL272C	217.938	228.388	306.936
15:150000	YOL098C	YOL097C	-372.505	-367.239	-341.693
15:150000	YOL098C	YOR341W	1105.3	1117.59	1169.38
15:150000	YOL098C	YJL109C	1001.99	1010.9	1011.78
15:150000	YOL098C	YMR142C	-802.854	-796.211	-739.115
15:150000	YOL098C	YDL229W	-528.417	-517.974	-432.111
15:150000	YOL098C	YKL187C	-8.6485	-4.57437	0.468564
15:150000	YOL098C	YLR075W	-1787.62	-1781.6	-1704.52
15:150000	YOL098C	YDR064W	-1021.62	-1015.97	-999.989
15:150000	YOL098C	YPR163C	123.561	135.214	180.606
15:150000	YOL098C	YOR152C	-124.558	-123.539	-121.889
15:150000	YOL098C	YNL073W	-396.349	-390.36	-268.517
15:150000	YOL098C	YDR337W	50.441	56.5473	128.753
15:150000	YOL098C	YLR249W	-446.71	-435.654	-352.737
15:150000	YOL098C	YMR241W	-628.687	-627.445	-592.612
15:150000	YOL098C	YER006W	976.926	985.47	988.819
15:150000	YOL098C	YCL059C	759.007	771.255	810.413
15:150000	YOL098C	YLR267W	777.872	780.281	803.355
15:150000	YOL098C	YDR502C	-501.806	-498.353	-481.147
15:150000	YOL098C	YPR010C	1095.96	1108.09	1144.98
15:150000	YOL098C	YGL189C	-1008.36	-997.338	-935.7
15:150000	YOL098C	YIL069C	185.957	195.247	275.258
15:150000	YOL098C	YPR043W	-1177.57	-1166.95	-1138.52
15:150000	YOL098C	YPL132W	-175.426	-164.684	24.5383

15:150000	YOL098C	YOL127W	-1176.22	-1164.43	-1095.31
15:150000	YOL098C	YEL054C	207.568	215.839	339.576
15:150000	YOL098C	YEL047C	-987.53	-976.214	-857.384
15:150000	YOL098C	YML026C	-330.606	-323.501	-251.144
15:150000	YOL098C	YMR242C	-461.529	-457.195	-376.185
15:150000	YOL098C	YMR116C	-1108.75	-1101.1	-1064.13
15:150000	YOL098C	YLR204W	-53.8021	-48.8759	-17.9579
15:150000	YOL098C	YGR031W	-501.675	-492.093	-250.154
15:150000	YOL098C	YLR150W	-1279.42	-1269.25	-1208.15
15:150000	YOL098C	YDL235C	-549.74	-546.907	-513.4
15:150000	YOL098C	YDR418W	-726.251	-717.68	-649.996
15:150000	YOL098C	YGR033C	-783.877	-773.653	-584.493
15:150000	YOL098C	YHR030C	-261.568	-259.28	-234.826
15:150000	YOL098C	YJL189W	-643.87	-631.72	-533.373
15:150000	YOL098C	YIR037W	-934.164	-928.65	-908.676
15:150000	YOL098C	YFL054C	380.17	382.791	409.655
15:150000	YOL098C	YLR388W	-165.682	-162.546	-70.64
15:150000	YOL098C	YGR264C	-54.7401	-43.5706	-15.6841
15:150000	YOL098C	YPL048W	-1310.1	-1299.64	-1190.68
15:150000	YOL098C	YML063W	-742.462	-731.393	-663.968
15:150000	YOL098C	YNL044W	-906.154	-896.15	-880.061
15:150000	YOL098C	YLR388W	-251.402	-250.758	-170.06
15:150000	YOL098C	YLR175W	820.124	831.488	857.835
15:150000	YOL098C	YKR094C	-567.72	-559.196	-480.207
15:150000	YOL098C	YGL065C	-860.799	-859.08	-648.001
15:150000	YOL098C	YEL050C	-94.5037	-85.7097	-71.3841
15:150000	YOL098C	YLR344W	-217.486	-206.442	-136.251
15:150000	YOL098C	YHR089C	732.254	743.971	779.916
15:150000	YOL098C	YLR048W	-102.714	-96.8175	-15.4308
15:150000	YOL098C	YBL072C	-843.648	-837.387	-787.929
15:150000	YOL098C	YDR012W	-280.614	-268.555	-245.205
15:150000	YOL098C	YHR156C	-224.68	-222.883	-184.845
15:150000	YOL098C	YOR312C	-613.684	-605.185	-544.145
15:150000	YOL098C	YEL055C	99.2751	106.023	119.998
15:150000	YOL098C	YNL178W	-750.747	-740.98	-711.019
15:150000	YOL098C	YDR430C	-35.8781	-34.752	99.5676
15:150000	YOL098C	YDR165W	908.543	917.973	921.192
15:150000	YOL098C	YMR229C	1116.67	1128.61	1156.99
15:150000	YOL098C	YIL018W	-307.22	-304.98	-224.999
15:150000	YOL098C	YGL040C	-489.546	-478.012	-321.323
15:150000	YOL098C	YDR060W	1237.3	1242.38	1239.26
15:150000	YOL098C	YPL118W	-430.882	-424.067	-378.369
15:150000	YOL098C	YPR110C	437.818	445.33	456.452
15:150000	YOL098C	YGR034W	-335.531	-332.552	-232.78
15:150000	YOL098C	YEL026W	442.16	454.3	559.158
15:150000	YOL098C	YER165W	-932.507	-922.526	-865.661
15:150000	YOL098C	YER169W	30.3678	30.8634	206.624

15:150000	YOL098C	YPL183W-A	-30.7962	-25.6263	-20.6971
15:150000	YOL098C	YOL081W	-608.125	-596.629	-584.305
15:150000	YOL098C	YKL096W-A	-1755.86	-1747.12	-1712.79
15:150000	YOL098C	YNL175C	1313.35	1323.7	1329.17
15:150000	YOL098C	YDL130W	-715.892	-708.606	-658.824
15:150000	YOL098C	YMR118C	560.159	567.91	654.735
15:150000	YOL098C	YOR206W	1336.19	1342.25	1339.12
15:150000	YOL098C	YNR036C	-490.699	-482.502	-416.163
15:150000	YOL098C	YDL061C	-905.345	-895.656	-834.727
15:150000	YOL098C	YHL033C	-417.957	-413.563	-355.649
15:150000	YOL098C	YAL012W	-781.371	-773.009	-762.647
15:150000	YOL098C	YDL083C	-40.4693	-35.6295	62.394
15:150000	YOL098C	YMR012W	-733.927	-729.365	-648.071
15:150000	YOL098C	YMR271C	694.572	703.901	730.396
15:150000	YOL098C	YLR448W	-212.188	-203.944	-124.739
15:150000	YOL098C	YHL034C	-1190.86	-1186.69	-1173.62
15:150000	YOL098C	YCL057C-A	-797.35	-790.251	-794.896
15:150000	YOL098C	YML024W	-980.681	-970.109	-900.436
15:150000	YOL098C	YNL209W	-248.102	-235.974	-130.422
15:150000	YOL098C	YDR303C	-574.253	-574.212	-572.036
15:150000	YOL098C	YOR167C	-701.946	-696.354	-587.183
15:150000	YOL098C	YOR310C	910.096	920.622	952.103
15:150000	YOL098C	YML042W	-39.0952	-38.7594	-25.787
15:150000	YOL098C	YBL035C	-354.819	-347.668	-347.775
15:150000	YOL098C	YOL039W	-774.356	-763.034	-742.247
15:150000	YOL098C	YNR033W	-447.131	-444.422	-385.586
15:150000	YOL098C	YPL249C-A	-904.131	-900.362	-858.557
15:150000	YOL098C	YGR286C	-189.946	-186.028	-151.936
15:150000	YOL098C	YML026C	-1200.43	-1196.5	-1091.28
15:150000	YOL098C	YIL078W	-1013.42	-1006.27	-1000.73
15:150000	YOL098C	YMR194W	-243.628	-236.07	-147.714
15:150000	YOL098C	YMR182W-A	241.187	251.739	431.447
15:150000	YOL098C	YOR354C	-409.753	-397.816	-294.256
15:150000	YOL098C	YHR020W	388.754	396.183	401.352
15:150000	YOL098C	YLR023C	-187.423	-183.335	-128.994
15:150000	YOL098C	YLR168C	106.83	113.818	110.846
15:150000	YOL098C	YDR025W	-122.349	-115.572	-18.7187
15:150000	YOL098C	YLR194C	-37.3939	-31.7487	-36.3711
15:150000	YOL098C	YKL056C	-1525.74	-1516.27	-1433.46
15:150000	YOL098C	YNL284C	-311.228	-305.293	-193.955
15:150000	YOL098C	YGL078C	1476.94	1485.49	1484.74
15:150000	YOL098C	YOR107W	45.343	51.7398	55.3588
15:150000	YOL098C	YML056C	670.873	678.019	751.193
15:150000	YOL098C	YCR031C	-1028.83	-1017.09	-972.031
15:150000	YOL098C	YPL211W	982.334	984.459	987.279
15:150000	YOL098C	YNL081C	-128.238	-123.095	51.5058
15:150000	YOL098C	YDL241W	-318.384	-317.696	-292.923

15:150000	YOL098C	YER131W	232.471	240.824	336.997
15:150000	YOL098C	YJL191W	70.1459	80.5476	132.668
15:150000	YOL098C	YFL036W	-327.212	-319.584	-167.878
15:150000	YOL098C	YHR183W	-928.92	-925.015	-785.642
15:150000	YOL098C	YPL266W	667.176	675.067	684.184
15:150000	YOL098C	YHR068W	-757.022	-752.806	-693.213
15:150000	YOL098C	YDR321W	84.1136	93.3902	137.445
15:150000	YOL098C	YMR038C	-855.729	-849.248	-799.379
15:150000	YOL098C	YGL077C	-472.376	-462.755	-457.172
15:150000	YOL098C	YMR056C	-383.848	-378.053	-350.308
15:150000	YOL098C	YDR500C	-311.693	-305.682	-213.179
15:150000	YOL098C	YDL051W	567.552	575.737	593.158
15:150000	YOL098C	YPL079W	-492.583	-489.181	-396.891
15:150000	YOL098C	YDL208W	569.757	574.966	582.6
15:150000	YOL098C	YDR047W	-443.279	-443.246	-393.951
15:150000	YOL098C	YHR007C	-891.559	-888.099	-817.122
15:150000	YOL098C	YDR260C	-386.194	-376.963	-361.616
15:150000	YOL098C	YGR155W	-766.454	-759.941	-746.142
15:150000	YOL098C	YLR061W	-291.039	-286.008	-201.584
15:150000	YOL098C	YHR141C	-966.208	-955.393	-854.872
15:150000	YOL098C	YLR293C	-1320.43	-1313.8	-1270.43
15:150000	YOL098C	YOL034W	-388.706	-385.858	-388.088
15:150000	YOL098C	YGR220C	-230.01	-219.976	-124.81
15:150000	YOL098C	YMR308C	178.563	184.867	190.921
15:150000	YOL098C	YDR494W	-204.192	-195.913	33.5224
15:150000	YOL098C	YOR243C	298.93	302.077	302.106
15:150000	YOL098C	YLR197W	813.355	825.595	895.194
15:150000	YOL098C	YOL119C	210.285	214.821	220.938
15:150000	YOL098C	YML071C	-666.594	-664.798	-662.253
15:150000	YOL098C	YLR333C	-582.51	-579.554	-490.519
15:150000	YOL098C	YHR045W	-859.051	-855.808	-826.488
15:150000	YOL098C	YER110C	469.29	479.961	529.941
15:150000	YOL098C	YBR189W	-403.159	-396.761	-335.887
15:150000	YOL098C	YPL160W	-463.707	-456.19	-418.616
15:150000	YOL098C	YNL302C	-520.881	-510.582	-420.929
15:150000	YOL098C	YML126C	-1011	-1002.47	-1007.36
15:150000	YOL098C	YJR077C	-808.339	-798.032	-796.098
15:150000	YOL098C	YDR132C	-426.623	-425.328	-418.891
15:150000	YOL098C	YPR132W	-1066.08	-1055.18	-996.761
15:150000	YOL098C	YPL053C	-845.821	-844.429	-803.589
15:150000	YOL098C	YKL006W	-591.785	-585.151	-516.216
15:150000	YOL098C	YLR372W	-289.261	-281.44	-246.271
15:150000	YOL098C	YER074W	-508.9	-501.641	-443.147
15:150000	YOL098C	YFR031C-A	-260.39	-253.502	-182.563
15:150000	YOL098C	YMR267W	-66.4843	-63.1829	63.6493
15:150000	YOL098C	YHL033C	-489.875	-480.909	-436.618
15:150000	YOL098C	YDL082W	176.022	178.98	280.996

15:150000	YOL098C	YMR011W	571.702	577.978	636.248
15:150000	YOL098C	YML106W	-780.913	-777.414	-771.259
15:150000	YOL098C	YOL103W	-964.695	-958.931	-950.154
15:150000	YOL098C	YOR276W	-758.561	-754.603	-743.546
15:150000	YOL098C	YPR018W	-471.19	-460.723	-447.252
15:150000	YOL098C	YPL040C	-120.53	-108.641	-3.66688
15:150000	YOL098C	YLR432W	-14.0013	-2.15502	62.0993
15:150000	YOL098C	YGL115W	-1004.43	-1001.13	-952.979
15:150000	YOL098C	YLR449W	783.236	793.296	843.09
15:150000	YOL098C	YPL104W	-145.386	-141.14	-115.388
15:150000	YOL098C	YHR106W	-777.221	-775.645	-768.01
15:150000	YOL098C	YMR230W	-285.281	-277.085	-183.579
15:150000	YOL098C	YDL014W	670.16	682.319	746.068
15:150000	YOL098C	YDR023W	-994.941	-985.229	-953.911
15:150000	YOL098C	YGL147C	-422.228	-416.68	-334.515
15:150000	YOL098C	YOR286W	-352.429	-343.266	-338.515
15:150000	YOL098C	YOL056W	-584.477	-580.085	-553.511
15:150000	YOL098C	YNL302C	-947.564	-940.193	-880.36
15:150000	YOL098C	YER001W	-58.1424	-49.3283	-54.4934
15:150000	YOL098C	YNL255C	-110.759	-102.248	-82.114
15:150000	YOL098C	YKL155C	11.4304	19.9931	28.4338
15:150000	YOL098C	YLL046C	123.839	136.049	218.229
15:150000	YOL098C	YLR228C	485.816	488.645	504.027
15:150000	YOL098C	YGL103W	-1356.58	-1347.12	-1248.91
15:150000	YOL098C	YKL052C	-851.453	-841.768	-846.495
15:150000	YOL098C	YBR148W	73.0937	79.8447	97.3857
15:150000	YOL098C	YBL087C	-774.975	-764.382	-703.911
15:150000	YOL098C	YOL121C	-22.1891	-18.6791	85.0218
15:150000	YOL098C	YGL123W	-982.969	-971.537	-917.149
15:150000	YOL098C	YNL137C	-249.286	-237.342	-97.5439
15:150000	YOL098C	YOR312C	-737.286	-727.558	-663.076
15:150000	YOL098C	YNL069C	-726.411	-716.303	-667.606
15:150000	YOL098C	YJR122W	-237.723	-227.499	-189.638
15:150000	YOL098C	YCR034W	-342.126	-333.982	-277.143
15:150000	YOL098C	YJL034W	-703.817	-692.378	-587.502
15:150000	YOL098C	YOL091W	530.528	530.714	682.435
15:150000	YOL098C	YHR010W	-606.841	-602.146	-518.995
15:150000	YOL098C	YGR102C	-301.428	-297.563	-43.6323
15:150000	YOL098C	YKL216W	1004.46	1008.23	1004.64
15:150000	YOL098C	YGR123C	697.946	709.112	737.685
15:150000	YOL098C	YKL087C	-174.382	-162.897	84.4825
15:150000	YOL098C	YMR072W	-1008.57	-1001.82	-1006.86
15:150000	YOL098C	YJL062W-A	-158.587	-151.141	-127.624
15:150000	YOL098C	YER074W	-707.675	-698.751	-635.772
15:150000	YOL098C	YJR043C	-582.284	-573.046	-542.547
15:150000	YOL098C	YBL030C	-731.205	-728.477	-725.883
15:150000	YOL098C	YDR277C	1120.27	1121.25	1121.92

15:150000	YOL098C	YGR118W	-1099.23	-1088.96	-1063.29
15:150000	YOL098C	YOR096W	-534.139	-526.922	-470.242
15:150000	YOL098C	YLR406C	262.508	272.593	333.655
15:150000	YOL098C	YOR293W	-158.066	-156.338	-26.312
15:150000	YOL098C	YDR091C	353.545	357.364	366.731
15:150000	YOL098C	YGR021W	45.5112	48.5622	144.145
15:150000	YOL098C	YHR064C	-751.313	-743.954	-716.994
15:150000	YOL098C	YMR157C	-22.3442	-20.2988	15.8442
15:150000	YOL098C	YGL076C	-1222	-1214.14	-1179.71
15:150000	YOL098C	YDR296W	31.0474	36.193	103.889
15:150000	YOL098C	YMR286W	-90.2016	-78.6852	-40.3436
15:150000	YOL098C	YFR032C-A	-661.109	-649.612	-542.029
15:150000	YOL098C	YPL061W	-30.1468	-28.9015	20.6517
15:150000	YOL098C	YDL211C	-432.649	-426.145	-328.249
15:150000	YOL098C	YPL156C	10.4451	19.7029	25.4028
15:150000	YOL098C	YER117W	-612.101	-603.488	-522.431
15:150000	YOL098C	YBR191W	-781.009	-774.795	-685.856
15:150000	YOL098C	YPL089C	200.928	206.074	250.573
15:150000	YOL098C	YMR145C	101.853	112.556	129.987
15:150000	YOL098C	YBR025C	-742.522	-730.432	-691.377
15:150000	YOL098C	YLR441C	-486.726	-486.014	-396.816
15:150000	YOL098C	YGR285C	-1105.1	-1100.32	-1071.36
15:150000	YOL098C	YHR019C	-824.551	-817.968	-822.69
15:150000	YOL098C	YDR041W	-102.28	-95.0415	5.46813
15:150000	YOL098C	YAR029W	144.379	145.221	241.304
15:150000	YOL098C	YLL045C	-718.488	-708.657	-679.486
15:150000	YOL098C	YER025W	-327.012	-321.02	-308.031
15:150000	YOL098C	YOR315W	443.149	454.486	539.864
15:150000	YOL098C	YKL051W	195.215	195.689	211.445
15:150000	YOL098C	YLR340W	-1012.37	-1000.12	-943.397
15:150000	YOL098C	YNL301C	-1109.47	-1105.16	-1049.5
15:150000	YOL098C	YOL094C	-373.797	-366.098	-340.569
15:150000	YOL098C	YLL056C	-416.455	-410.505	-361.625
15:150000	YOL098C	YOL040C	-772.309	-762.784	-726.307
15:150000	YOL098C	YNL061W	996.419	1001.76	1004.73
15:150000	YOL098C	YBR181C	-1112.08	-1103.96	-1027.24
15:150000	YOL098C	YKL014C	1016.72	1023.45	1026.28
15:150000	YOL098C	YDL081C	-851.904	-842.775	-748.43
15:150000	YOL098C	YJR101W	-160.904	-150.374	-74.9271
15:150000	YOL098C	YBR185C	26.3217	31.0039	143.169
15:150000	YOL098C	YGR152C	-474.56	-465.996	-412.901
15:150000	YOL098C	YHR203C	-1438.34	-1427.31	-1351.61
15:150000	YOL098C	YMR143W	-893.717	-884.204	-797.313
15:150000	YOL098C	YKL073W	-661.974	-651.805	-500.825
15:150000	YOL098C	YBR048W	-844.046	-832.352	-771.084
15:150000	YOL098C	YGL135W	-1375.1	-1362.98	-1270.71
15:150000	YOL098C	YFL022C	-764.678	-754.612	-708.152

15:150000	YOL098C	YOR134W	521.568	527.075	574.386
15:150000	YOL098C	YGR007W	-1018.4	-1012.65	-1008.9
15:150000	YOL098C	YJL177W	-44.6606	-38.7583	32.6528
15:150000	YOL098C	YJR094W-A	-153.302	-145.855	-23.7195
15:150000	YOL098C	YJR090C	-766.695	-754.639	-704.015
15:150000	YOL098C	YDL075W	-703.027	-693.536	-629.641
15:150000	YOL098C	YJL112W	-223.473	-214.807	-190.715
15:150000	YOL098C	YHL001W	-975.597	-965.989	-895.174
15:150000	YOL098C	YDR482C	-453.94	-449.529	-359.794
15:150000	YOL098C	YHR021C	-580.325	-575.674	-484.188
15:150000	YOL098C	YMR217W	375.797	387.866	461.897
15:150000	YOL098C	YBR191W	-308.068	-297.053	-220.369
15:150000	YOL098C	YOR234C	-159.09	-152.773	-115.21
15:150000	YOL098C	YEL071W	-538.693	-530.72	-531.944
15:150000	YOL098C	YBL092W	-997.478	-990.912	-908.269
15:150000	YOL098C	YMR238W	-619.06	-615.546	-610.07
15:150000	YOL098C	YGR094W	-861.189	-857.892	-844.035
15:150000	YOL098C	YDR434W	-1058.15	-1051.86	-1057.7
15:150000	YOL098C	YFR006W	-947.28	-938.586	-944.662
15:150000	YOL098C	YPR127W	-466.7	-463.506	-465.407
15:150000	YOL098C	YJL136C	-895.013	-889.181	-846.138
15:150000	YOL098C	YIL133C	-130.187	-119.935	-42.804
15:150000	YOL098C	YFR031C-A	-953.877	-942.628	-916.76
15:150000	YOL098C	YOR293W	-187.376	-182.714	-67.7811
15:150000	YOL098C	YGL030W	-1203.55	-1192.33	-1125.75
15:150000	YOL097C	YDR447C	-729.294	-718.21	-594.38
15:150000	YOL097C	YDR447C	-316.301	-315.652	-205.687
15:150000	YOL097C	YNR055C	-671.276	-665.894	-629.558
15:150000	YOL097C	YOR187W	-562.171	-558.491	-385.723
15:150000	YOL097C	YKL167C	-257.681	-256.15	-78.2337
15:150000	YOL097C	YDL061C	-887.977	-880.755	-768.084
15:150000	YOL097C	YOL077W-A	-334.286	-327.385	-313.015
15:150000	YOL097C	YDL191W	-1127.2	-1121.98	-1093.12
15:150000	YOL097C	YBR084C-A	-397.931	-390.51	-315.82
15:150000	YOL097C	YAL003W	-774.321	-766.63	-679.578
15:150000	YOL097C	YDR450W	-970.046	-965.229	-818.446
15:150000	YOL097C	YLR029C	-1049.69	-1043.72	-976.309
15:150000	YOL097C	YIL052C	-584.625	-574.624	-448.08
15:150000	YOL097C	YNL055C	-1213.21	-1208.05	-1192.1
15:150000	YOL097C	YPL131W	-831.364	-822.807	-751.681
15:150000	YOL097C	YKR092C	489.389	494.469	608.604
15:150000	YOL097C	YDL173W	-694.637	-692.289	-625.236
15:150000	YOL097C	YDR454C	-1144	-1140.78	-1137.42
15:150000	YOL097C	YJR123W	-1089.4	-1078.01	-988.296
15:150000	YOL097C	YGR027C	-1115.2	-1106.36	-1025.94
15:150000	YOL097C	YDL229W	-178.082	-167.506	25.055
15:150000	YOL097C	YKL180W	-570.064	-560.557	-440.011

15:150000	YOL097C	YLR185W	-466.643	-457.385	-349.266
15:150000	YOL097C	YLL028W	-294.025	-284.528	-250.698
15:150000	YOL097C	YHR005C-A	-528.281	-523.709	-524.057
15:150000	YOL097C	YKL081W	-767.134	-766.196	-648.874
15:150000	YOL097C	YGL031C	-977.231	-966.807	-890.579
15:150000	YOL097C	YER013W	-789.833	-781.869	-772.013
15:150000	YOL097C	YGL013C	-783.976	-772.227	-583.812
15:150000	YOL097C	YBL072C	-1427.72	-1418.08	-1290.92
15:150000	YOL097C	YBL027W	-523.08	-517.088	-399.84
15:150000	YOL097C	YER102W	-718.996	-709.271	-612.299
15:150000	YOL097C	YGL189C	-1171.08	-1168.04	-1081.97
15:150000	YOL097C	YKR057W	-324.541	-314.275	-224.357
15:150000	YOL097C	YPL036W	51.272	60.7537	441.236
15:150000	YOL097C	YGR085C	-97.7099	-96.6641	-55.4833
15:150000	YOL097C	YPL189W	-183.991	-171.909	-169.441
15:150000	YOL097C	YGR148C	-1105.4	-1094.41	-999.433
15:150000	YOL097C	YGR214W	-307.551	-300.635	-222.933
15:150000	YOL097C	YAL042W	-1063.64	-1063.06	-1047.96
15:150000	YOL097C	YPL272C	237.392	246.222	308.898
15:150000	YOL097C	YOR341W	1134.09	1145.48	1172.37
15:150000	YOL097C	YMR142C	-818.65	-811.656	-732.968
15:150000	YOL097C	YDL229W	-589.538	-579.485	-426.299
15:150000	YOL097C	YKL187C	-0.215831	3.01494	4.08998
15:150000	YOL097C	YPR163C	181.059	184.49	182.792
15:150000	YOL097C	YNL073W	-554.049	-545.934	-270.545
15:150000	YOL097C	YOL120C	-646.335	-639.434	-511.441
15:150000	YOL097C	YDR337W	-88.1842	-81.9881	128.799
15:150000	YOL097C	YLR249W	-504.387	-503.78	-347.689
15:150000	YOL097C	YCL059C	771.419	783.22	808.056
15:150000	YOL097C	YLR267W	769.638	775.57	815.661
15:150000	YOL097C	YPR010C	1123.65	1134.5	1142.86
15:150000	YOL097C	YGL189C	-1057.13	-1046.63	-937.34
15:150000	YOL097C	YIL069C	241.786	245.676	281.644
15:150000	YOL097C	YPR043W	-1216.94	-1212.64	-1138.73
15:150000	YOL097C	YOL127W	-1211.99	-1201.61	-1092.1
15:150000	YOL097C	YEL054C	155.219	166.279	343.924
15:150000	YOL097C	YEL047C	-1049.48	-1044.62	-860.626
15:150000	YOL097C	YML026C	-348.829	-339.033	-243.719
15:150000	YOL097C	YMR242C	-512.83	-503.695	-372.996
15:150000	YOL097C	YLR204W	-130.161	-124.329	-18.808
15:150000	YOL097C	YGR031W	-587.305	-578.065	-248.37
15:150000	YOL097C	YLR150W	-1369.94	-1360.76	-1210.78
15:150000	YOL097C	YDL235C	-573.086	-572.008	-523.123
15:150000	YOL097C	YDR418W	-751.514	-742.81	-643.549
15:150000	YOL097C	YGR033C	-828.515	-816.735	-575.332
15:150000	YOL097C	YJL189W	-628.143	-622.701	-530.262
15:150000	YOL097C	YLR388W	-155.596	-148.349	-58.8154

15:150000	YOL097C	YPL048W	-1375.18	-1367.06	-1189.79
15:150000	YOL097C	YML063W	-745.802	-735.933	-655.87
15:150000	YOL097C	YNL044W	-932.881	-926.931	-888.065
15:150000	YOL097C	YLR388W	-249.677	-243.491	-164.131
15:150000	YOL097C	YKR094C	-589.398	-581.092	-472.429
15:150000	YOL097C	YGL065C	-934.895	-931.345	-651.921
15:150000	YOL097C	YEL050C	-144.174	-136.854	-74.7274
15:150000	YOL097C	YLR344W	-162.335	-160.292	-132.458
15:150000	YOL097C	YLR048W	-159.71	-151.303	-10.4712
15:150000	YOL097C	YBL072C	-881.381	-870.669	-786.284
15:150000	YOL097C	YDR012W	-282.922	-275.396	-248.557
15:150000	YOL097C	YHR156C	-229.358	-228.666	-186.255
15:150000	YOL097C	YOR312C	-648.833	-638.665	-541.923
15:150000	YOL097C	YNL178W	-746.549	-736.659	-706.546
15:150000	YOL097C	YMR229C	1141.69	1152.57	1155.15
15:150000	YOL097C	YIL018W	-350.747	-342.966	-224.307
15:150000	YOL097C	YGL040C	-574.035	-563.517	-315.468
15:150000	YOL097C	YPL118W	-561.351	-552.363	-374.676
15:150000	YOL097C	YGR034W	-379.229	-367.883	-229.098
15:150000	YOL097C	YEL026W	425.329	434.368	558.665
15:150000	YOL097C	YER165W	-914.986	-909.213	-857.035
15:150000	YOL097C	YFR011C	-192.256	-191.279	-53.1183
15:150000	YOL097C	YER169W	-136.965	-125.523	202.633
15:150000	YOL097C	YPL183W-A	-110.632	-106.085	-23.8572
15:150000	YOL097C	YOL081W	-626.364	-617.669	-578.623
15:150000	YOL097C	YKL096W-A	-1778.13	-1775.29	-1717.94
15:150000	YOL097C	YDL130W	-743.134	-734.839	-654.469
15:150000	YOL097C	YMR118C	593.332	599.003	659.244
15:150000	YOL097C	YNR036C	-566.875	-562.947	-419.704
15:150000	YOL097C	YDL061C	-933.415	-927.864	-832.84
15:150000	YOL097C	YHL033C	-437.863	-429.776	-350.138
15:150000	YOL097C	YAL012W	-778.616	-778.143	-768.496
15:150000	YOL097C	YDL083C	-93.3084	-82.8581	69.4668
15:150000	YOL097C	YMR012W	-789.799	-780.053	-646.086
15:150000	YOL097C	YMR271C	726.461	731.971	732.516
15:150000	YOL097C	YLR448W	-194.041	-185.843	-118.84
15:150000	YOL097C	YCL057C-A	-812.52	-808.918	-799.787
15:150000	YOL097C	YML024W	-1015.55	-1006.52	-905.19
15:150000	YOL097C	YNL209W	-297.303	-287.299	-127.416
15:150000	YOL097C	YOR167C	-671.02	-663.958	-578.195
15:150000	YOL097C	YOL039W	-820.82	-813.448	-743.159
15:150000	YOL097C	YPL249C-A	-936.63	-927.551	-857.12
15:150000	YOL097C	YMR194W	-179.097	-173.07	-138.228
15:150000	YOL097C	YMR182W-A	184.805	185.734	425.018
15:150000	YOL097C	YOR354C	-500.895	-489.697	-292.049
15:150000	YOL097C	YLR023C	-183.57	-179.651	-126.208
15:150000	YOL097C	YLR168C	110.744	118.986	112.842

15:150000	YOL097C	YDR025W	-187.091	-176.783	-14.5165
15:150000	YOL097C	YLR194C	-37.3367	-30.4024	-36.5551
15:150000	YOL097C	YNL284C	-533.091	-527.586	-194.048
15:150000	YOL097C	YOR107W	57.2039	60.2927	60.188
15:150000	YOL097C	YML056C	680.132	687.377	748.827
15:150000	YOL097C	YCR031C	-1038.87	-1032.35	-968.835
15:150000	YOL097C	YER131W	109.724	119.964	341.125
15:150000	YOL097C	YHR183W	-994.672	-991.465	-773.951
15:150000	YOL097C	YDR321W	105.372	112.853	134.02
15:150000	YOL097C	YMR038C	-812.834	-810.598	-785.718
15:150000	YOL097C	YLR325C	-299.776	-293.133	-200.31
15:150000	YOL097C	YGL077C	-507.401	-505.197	-466.989
15:150000	YOL097C	YMR056C	-362.211	-361.698	-347.67
15:150000	YOL097C	YDR500C	-304.19	-296.938	-210.658
15:150000	YOL097C	YPL079W	-494.616	-487.607	-391.185
15:150000	YOL097C	YDR260C	-364.151	-354.373	-355.151
15:150000	YOL097C	YLR061W	-315.477	-307.694	-195.934
15:150000	YOL097C	YHR141C	-935.788	-927.475	-852.89
15:150000	YOL097C	YGR220C	-364.188	-355.159	-127.047
15:150000	YOL097C	YDR494W	-353.038	-345.132	34.6565
15:150000	YOL097C	YLR197W	834.822	846.359	892.308
15:150000	YOL097C	YOL119C	215.418	222.813	219.336
15:150000	YOL097C	YLR333C	-593.334	-586.962	-489.769
15:150000	YOL097C	YHR045W	-884.736	-884.256	-829.086
15:150000	YOL097C	YJL190C	-242.085	-231.48	-114.409
15:150000	YOL097C	YER110C	476.175	484.75	528.761
15:150000	YOL097C	YBR189W	-453.244	-442.659	-330.161
15:150000	YOL097C	YPL160W	-463.582	-462.468	-419.186
15:150000	YOL097C	YNL302C	-568.082	-556.761	-416.206
15:150000	YOL097C	YML126C	-1015.56	-1012.14	-1011.6
15:150000	YOL097C	YJR077C	-829.476	-819.118	-793.727
15:150000	YOL097C	YDR132C	-428.184	-417.77	-408.006
15:150000	YOL097C	YPR132W	-1110.52	-1098.57	-996.72
15:150000	YOL097C	YKL006W	-638.834	-627.529	-508.896
15:150000	YOL097C	YER074W	-530.236	-522.411	-441.303
15:150000	YOL097C	YFR031C-A	-296.921	-288.646	-180.395
15:150000	YOL097C	YDL082W	128.658	135.14	287.137
15:150000	YOL097C	YMR011W	613.264	623.805	645.627
15:150000	YOL097C	YOL103W	-965.957	-963.073	-950.675
15:150000	YOL097C	YPR018W	-465.617	-460.829	-445.679
15:150000	YOL097C	YPL040C	-187.392	-179.397	-3.29668
15:150000	YOL097C	YLR432W	34.8992	42.3793	59.8565
15:150000	YOL097C	YGL115W	-1122.42	-1121.07	-949.764
15:150000	YOL097C	YER056C-A	-373.012	-365.862	-227.169
15:150000	YOL097C	YPL104W	-228.844	-223.399	-120.428
15:150000	YOL097C	YMR230W	-249.51	-239.47	-178.047
15:150000	YOL097C	YDL014W	716.494	726.95	746.025

15:150000	YOL097C	YGL147C	-447.518	-435.831	-331.949
15:150000	YOL097C	YOR286W	-412.638	-404.096	-342.969
15:150000	YOL097C	YOL056W	-630.172	-629.075	-555.302
15:150000	YOL097C	YNL302C	-952.04	-942.56	-872.124
15:150000	YOL097C	YER001W	-66.6692	-54.6028	-53.1904
15:150000	YOL097C	YKL155C	16.0048	20.8873	26.8455
15:150000	YOL097C	YLL046C	81.3818	93.2538	217.947
15:150000	YOL097C	YML073C	-445.525	-438.936	-308.439
15:150000	YOL097C	YKL052C	-856.106	-844.939	-846.502
15:150000	YOL097C	YBL087C	-765.93	-760.561	-707.494
15:150000	YOL097C	YOL121C	-55.4024	-49.2241	90.1548
15:150000	YOL097C	YGL123W	-1001.03	-993.157	-921.55
15:150000	YOL097C	YNL137C	-358.241	-348.5	-96.1343
15:150000	YOL097C	YOR312C	-786.572	-775.76	-656.852
15:150000	YOL097C	YNL069C	-791.904	-780.652	-668.43
15:150000	YOL097C	YJR122W	-347.709	-337.977	-191.231
15:150000	YOL097C	YJL034W	-761.066	-759.104	-581.099
15:150000	YOL097C	YOL091W	432.814	437.321	693.156
15:150000	YOL097C	YHR010W	-641.27	-630.14	-510.397
15:150000	YOL097C	YGR102C	-446.181	-442.083	-55.8661
15:150000	YOL097C	YGR123C	732.573	741.367	738.776
15:150000	YOL097C	YMR295C	-1310.29	-1306.06	-1270.85
15:150000	YOL097C	YPL265W	-582.928	-582.194	-558.803
15:150000	YOL097C	YKL087C	-247.666	-244.456	85.9282
15:150000	YOL097C	YJL062W-A	-249.548	-244.772	-132.557
15:150000	YOL097C	YER074W	-775.817	-763.563	-631.499
15:150000	YOL097C	YJR043C	-578.484	-577.488	-545.087
15:150000	YOL097C	YBL030C	-786.345	-783.732	-726.833
15:150000	YOL097C	YGR138C	262.788	264.342	304.449
15:150000	YOL097C	YGR118W	-1075.33	-1066.1	-1062.38
15:150000	YOL097C	YOR096W	-587.491	-576.876	-468.676
15:150000	YOL097C	YLR406C	295.029	301.974	339.3
15:150000	YOL097C	YOR293W	-186.965	-176.49	-21.9643
15:150000	YOL097C	YGR021W	-73.8106	-73.3781	135.794
15:150000	YOL097C	YMR157C	-122.495	-115.813	21.6721
15:150000	YOL097C	YDR296W	-136.961	-131.66	94.6067
15:150000	YOL097C	YMR286W	-142.482	-134.19	-40.8919
15:150000	YOL097C	YNR060W	590.624	597.775	619.042
15:150000	YOL097C	YFR032C-A	-660.456	-651.987	-545.546
15:150000	YOL097C	YPL061W	-26.6403	-18.7284	29.9296
15:150000	YOL097C	YDL211C	-425.095	-421.834	-333.198
15:150000	YOL097C	YPL156C	4.20666	8.64625	34.805
15:150000	YOL097C	YER117W	-609.562	-601.508	-520.25
15:150000	YOL097C	YBR191W	-820.484	-810.376	-682.317
15:150000	YOL097C	YPR036W	-1210.51	-1205.09	-1056.15
15:150000	YOL097C	YMR145C	73.7081	81.9578	133.212
15:150000	YOL097C	YBR025C	-819.891	-809.366	-698.737

15:150000	YOL097C	YLR441C	-494.034	-487.624	-389.058
15:150000	YOL097C	YLL045C	-786.938	-777.731	-677.76
15:150000	YOL097C	YOR315W	432.102	442.404	541.682
15:150000	YOL097C	YLR340W	-1073.09	-1062.84	-951.664
15:150000	YOL097C	YNL301C	-1123.46	-1114.79	-1038.46
15:150000	YOL097C	YOL094C	-401.54	-400.144	-341.318
15:150000	YOL097C	YLL056C	-407.134	-406.908	-363.689
15:150000	YOL097C	YOL040C	-807.008	-797.282	-725.506
15:150000	YOL097C	YBR181C	-1165.64	-1153.51	-1022.68
15:150000	YOL097C	YDL081C	-890.39	-878.618	-748.118
15:150000	YOL097C	YJR101W	-197.041	-184.765	-72.2999
15:150000	YOL097C	YBR185C	-175.358	-171.181	143.884
15:150000	YOL097C	YGL025C	-482.23	-479.113	-477.251
15:150000	YOL097C	YAL041W	-768.767	-764.463	-757.922
15:150000	YOL097C	YGR152C	-460.177	-456.861	-411.063
15:150000	YOL097C	YHR203C	-1478.74	-1472.05	-1359.25
15:150000	YOL097C	YMR143W	-902.604	-895.403	-797.626
15:150000	YOL097C	YKL073W	-726.051	-721.208	-500.3
15:150000	YOL097C	YBR048W	-872.774	-863.049	-770.45
15:150000	YOL097C	YGL135W	-1425.43	-1424.63	-1275.57
15:150000	YOL097C	YBR177C	-403.872	-394.636	-217.552
15:150000	YOL097C	YFL022C	-748.665	-748.482	-704.451
15:150000	YOL097C	YAL039C	-191.944	-191.006	-97.9928
15:150000	YOL097C	YJL177W	-71.9688	-64.2115	39.931
15:150000	YOL097C	YJR094W-A	-166.197	-159.335	-20.3383
15:150000	YOL097C	YJR090C	-772.711	-764.423	-705.523
15:150000	YOL097C	YDL075W	-740.149	-729.632	-631.566
15:150000	YOL097C	YJL112W	-226.999	-222.096	-188.821
15:150000	YOL097C	YHL001W	-983.934	-974.268	-887.513
15:150000	YOL097C	YDR482C	-449.483	-448.286	-361.085
15:150000	YOL097C	YHR021C	-591.639	-582.463	-480.476
15:150000	YOL097C	YMR217W	204.957	216.525	455.693
15:150000	YOL097C	YBR191W	-351.213	-339.372	-218.503
15:150000	YOL097C	YEL071W	-588.307	-579.106	-531.573
15:150000	YOL097C	YBL092W	-1023.15	-1011.66	-902.694
15:150000	YOL097C	YFR006W	-971.32	-959.892	-940.122
15:150000	YOL097C	YJL136C	-914.341	-907.5	-841.861
15:150000	YOL097C	YIL133C	-138.326	-129.839	-34.5002
15:150000	YOL097C	YFR031C-A	-967.71	-959.111	-917.663
15:150000	YOL097C	YOR293W	-224.918	-213.441	-61.414
15:150000	YOL097C	YGL030W	-1219.08	-1215.71	-1129.14
15:150000	YOL084W	YPL223C	34.265	45.179	181.379
15:150000	YOL084W	YER087C-B	-1071.43	-1063.56	-1052.59
15:150000	YOL084W	YIL160C	487.511	490.165	545.62
15:150000	YOL084W	YDR447C	-631.215	-624.348	-604.046
15:150000	YOL084W	YDR144C	111.377	114.437	175.995
15:150000	YOL084W	YJL185C	118.894	126.004	176.737

15:150000	YOL084W	YNR038W	111.089	114.225	134.344
15:150000	YOL084W	YNR055C	-649.541	-644.733	-641.71
15:150000	YOL084W	YHR197W	1087.58	1095.22	1137.95
15:150000	YOL084W	YDR245W	-753.683	-743.794	-725.167
15:150000	YOL084W	YGR110W	269.55	275.696	282.392
15:150000	YOL084W	YKL192C	-884.698	-879.558	-876.071
15:150000	YOL084W	YPR117W	-465.268	-457.915	-461.446
15:150000	YOL084W	YCL054W	755.219	763.243	779.423
15:150000	YOL084W	YHR128W	-392.969	-384.541	-385.16
15:150000	YOL084W	YOL077W-A	-317.989	-306.594	-311.594
15:150000	YOL084W	YGR083C	-376.01	-368.217	-347.583
15:150000	YOL084W	YDR450W	-847.553	-842.417	-825.309
15:150000	YOL084W	YLR029C	-1026.67	-1019.73	-985.847
15:150000	YOL084W	YMR128W	905.865	911.373	947.291
15:150000	YOL084W	YNL055C	-1226.32	-1214.97	-1206.56
15:150000	YOL084W	YDL173W	-655.813	-653.999	-635.333
15:150000	YOL084W	YOL090W	-441.991	-439.883	-424.228
15:150000	YOL084W	YML124C	-1252.86	-1245.27	-1243.63
15:150000	YOL084W	YHR065C	739.628	750.446	777.843
15:150000	YOL084W	YOL098C	-927.852	-926.51	-917.498
15:150000	YOL084W	YGR027C	-1063.89	-1051.83	-1037.57
15:150000	YOL084W	YKL110C	612.941	619.933	657.765
15:150000	YOL084W	YNL313C	338.993	346.421	369.07
15:150000	YOL084W	YMR301C	-201.772	-200.358	-136.608
15:150000	YOL084W	YEL036C	-113.118	-109.022	-103.233
15:150000	YOL084W	YML093W	982.151	989.887	1019.24
15:150000	YOL084W	YLL028W	-296.519	-291.253	-263.433
15:150000	YOL084W	YHR005C-A	-530.061	-523.876	-527.411
15:150000	YOL084W	YGL031C	-924.988	-915.25	-903.08
15:150000	YOL084W	YGL013C	-644.383	-633.075	-599.522
15:150000	YOL084W	YBL072C	-1337.77	-1335.04	-1307.09
15:150000	YOL084W	YJR032W	-471.244	-467.513	-455.595
15:150000	YOL084W	YGL189C	-1124.62	-1116.59	-1094.56
15:150000	YOL084W	YPL189W	-193.854	-182.415	-177.715
15:150000	YOL084W	YPR175W	-321.001	-316.922	-319.427
15:150000	YOL084W	YDR345C	-186.319	-182.73	-128.263
15:150000	YOL084W	YCR083W	-353.51	-350.14	-348.586
15:150000	YOL084W	YAL042W	-1091.82	-1084.51	-1067.81
15:150000	YOL084W	YBL068W	349.69	349.722	414.608
15:150000	YOL084W	YPL272C	270.838	282.144	309.68
15:150000	YOL084W	YAL017W	-384.2	-375.313	-294.947
15:150000	YOL084W	YMR239C	670.947	678.712	678.898
15:150000	YOL084W	YLR075W	-1764.05	-1758.22	-1726.84
15:150000	YOL084W	YDR064W	-1056.97	-1047.88	-1012.98
15:150000	YOL084W	YOR152C	-126.666	-123.849	-117.264
15:150000	YOL084W	YDL031W	346.401	351.307	383.694
15:150000	YOL084W	YGL111W	495.262	496.771	565.177

15:150000	YOL084W	YNL217W	-133.8	-133.606	-131.156
15:150000	YOL084W	YKR043C	-368.465	-367.427	-308.609
15:150000	YOL084W	YLR222C	1109.97	1120.36	1158.69
15:150000	YOL084W	YMR241W	-651.224	-639.222	-599.793
15:150000	YOL084W	YLR267W	793.351	801.961	811.318
15:150000	YOL084W	YHR085W	482.061	486.642	531.445
15:150000	YOL084W	YHR062C	-94.3822	-86.4354	-55.941
15:150000	YOL084W	YPR140W	-407.541	-405.162	-381.77
15:150000	YOL084W	YDR502C	-557.124	-545.853	-490.854
15:150000	YOL084W	YER126C	787.41	795.51	857.361
15:150000	YOL084W	YGL189C	-980.495	-970.875	-948.945
15:150000	YOL084W	YPR043W	-1208.43	-1204.38	-1153.18
15:150000	YOL084W	YNL113W	393.072	401.752	426.629
15:150000	YOL084W	YAR002C-A	-1349.13	-1340.12	-1338.93
15:150000	YOL084W	YOL127W	-1148.22	-1147.76	-1108.51
15:150000	YOL084W	YJL208C	-129.077	-117.104	-56.7478
15:150000	YOL084W	YLR172C	-48.8148	-46.9671	64.4705
15:150000	YOL084W	YOL022C	168.631	169.684	258.263
15:150000	YOL084W	YHR143W-A	139.093	145.421	168.013
15:150000	YOL084W	YIL091C	786.793	794.348	835.976
15:150000	YOL084W	YEL047C	-919.3	-910.311	-870.149
15:150000	YOL084W	YAL029C	-376.726	-374.808	-331.751
15:150000	YOL084W	YMR116C	-1139.63	-1132.28	-1081.34
15:150000	YOL084W	YGR031W	-331.816	-326.122	-253.31
15:150000	YOL084W	YIR012W	307.5	313.939	350.195
15:150000	YOL084W	YGR201C	-242.9	-240.424	-115.648
15:150000	YOL084W	YPL043W	1149.89	1156.96	1192.3
15:150000	YOL084W	YLR150W	-1249.62	-1239.45	-1223.96
15:150000	YOL084W	YPL196W	-605.379	-599.858	-548.132
15:150000	YOL084W	YGR033C	-674.931	-663.23	-590.333
15:150000	YOL084W	YGL128C	-671.003	-664.178	-658.096
15:150000	YOL084W	YJL189W	-571.952	-563.919	-535.853
15:150000	YOL084W	YER177W	-1332.9	-1324.24	-1312.21
15:150000	YOL084W	YPL048W	-1262.05	-1259.05	-1201.75
15:150000	YOL084W	YKR026C	-337.607	-331.887	-313.039
15:150000	YOL084W	YML063W	-685.024	-677.169	-664.48
15:150000	YOL084W	YDR211W	-159.338	-156.483	-90.5682
15:150000	YOL084W	YNL044W	-898.219	-892.064	-897.679
15:150000	YOL084W	YDL181W	399.923	402.941	409.56
15:150000	YOL084W	YGL065C	-740.402	-735.991	-657.335
15:150000	YOL084W	YEL050C	-76.4871	-66.0191	-71.997
15:150000	YOL084W	YPL084W	-650.255	-639.042	-631.181
15:150000	YOL084W	YDR018C	429.256	430.082	433.747
15:150000	YOL084W	YKL193C	-268.891	-267.503	-241.358
15:150000	YOL084W	YHR176W	-194.608	-186.532	-161.852
15:150000	YOL084W	YGL022W	-1181.87	-1173.18	-1169.54
15:150000	YOL084W	YDL213C	293.676	294.723	366.562

15:150000	YOL084W	YGL004C	-615.324	-605.59	-555.913
15:150000	YOL084W	YIL108W	-685.972	-675.205	-670.624
15:150000	YOL084W	YGL040C	-389.719	-385.62	-322.67
15:150000	YOL084W	YHR001W-A	-112.227	-103.885	-109.377
15:150000	YOL084W	YDR060W	1194.78	1195.08	1251.66
15:150000	YOL084W	YIL079C	540.821	547.871	570.853
15:150000	YOL084W	YPL118W	-379.823	-375.199	-379.11
15:150000	YOL084W	YKL212W	-575.79	-569.166	-550.115
15:150000	YOL084W	YOL041C	574.914	585.035	619.193
15:150000	YOL084W	YPL183W-A	-35.1544	-27.5681	-21.3766
15:150000	YOL084W	YOL080C	657.093	667.232	689.523
15:150000	YOL084W	YOL081W	-590.948	-586.496	-588.488
15:150000	YOL084W	YMR107W	714.148	715.206	772.511
15:150000	YOL084W	YAL036C	801.913	803.447	873.591
15:150000	YOL084W	YOR046C	-896.896	-896.778	-810.104
15:150000	YOL084W	YNL175C	1289.57	1293.67	1339.38
15:150000	YOL084W	YOR004W	1071.18	1080.34	1118.51
15:150000	YOL084W	YPL207W	295.684	299.743	362.076
15:150000	YOL084W	YOR206W	1296.3	1296.71	1350.69
15:150000	YOL084W	YNR036C	-421.936	-414.321	-420.302
15:150000	YOL084W	YMR114C	-465.076	-461.788	-390.861
15:150000	YOL084W	YLR193C	-583.278	-577.691	-562.227
15:150000	YOL084W	YDL024C	-40.3877	-31.2071	55.708
15:150000	YOL084W	YOL131W	63.0202	72.6814	201.953
15:150000	YOL084W	YJR016C	-107.512	-100.097	-106.244
15:150000	YOL084W	YLR070C	-10.6446	-1.38781	60.1229
15:150000	YOL084W	YIL127C	757.258	766.229	786.724
15:150000	YOL084W	YGR256W	60.3645	63.2515	205.132
15:150000	YOL084W	YNL093W	402.401	409.153	484.166
15:150000	YOL084W	YMR175W	471.148	481.332	524.121
15:150000	YOL084W	YLR002C	914.111	924.055	966.811
15:150000	YOL084W	YDL121C	-106.554	-94.7201	-55.7927
15:150000	YOL084W	YMR012W	-691.527	-684.604	-658.835
15:150000	YOL084W	YCL057C-A	-840.38	-836.201	-803.509
15:150000	YOL084W	YML024W	-940.021	-933.511	-913.57
15:150000	YOL084W	YDR339C	-249.291	-243.486	-243.388
15:150000	YOL084W	YDR303C	-599.628	-589.535	-578.559
15:150000	YOL084W	YDR436W	-373.251	-370.798	-331.992
15:150000	YOL084W	YER002W	93.3021	97.4189	99.958
15:150000	YOL084W	YML042W	-85.9511	-80.7973	-19.195
15:150000	YOL084W	YGR062C	-296.716	-292.283	-293.249
15:150000	YOL084W	YLR196W	1126.24	1132.2	1182.73
15:150000	YOL084W	YGR286C	-164.546	-160.89	-163.442
15:150000	YOL084W	YMR182W-A	304.339	307.262	424.676
15:150000	YOL084W	YOR354C	-329.981	-321.293	-295.32
15:150000	YOL084W	YLR023C	-232.924	-226.587	-131.577
15:150000	YOL084W	YPL108W	118.624	121.904	134.738

15:150000	YOL084W	YLR168C	82.2063	90.0603	114.868
15:150000	YOL084W	YKL056C	-1478.11	-1472.18	-1454.99
15:150000	YOL084W	YIL033C	-178.582	-174.473	-165.197
15:150000	YOL084W	YNL284C	-203.514	-198.061	-194.321
15:150000	YOL084W	YJL045W	14.5647	22.7397	85.2989
15:150000	YOL084W	YGL078C	1457.45	1464.11	1498.7
15:150000	YOL084W	YOR107W	22.9402	32.1798	57.5109
15:150000	YOL084W	YGR187C	496.702	500.067	526.043
15:150000	YOL084W	YGR128C	1207.53	1210.88	1267.29
15:150000	YOL084W	YHR183W	-890.652	-883.502	-788.365
15:150000	YOL084W	YPL119C-A	-78.3346	-69.4837	-67.4819
15:150000	YOL084W	YMR038C	-820.193	-808.743	-799.021
15:150000	YOL084W	YKL046C	-1029.9	-1020.47	-985.207
15:150000	YOL084W	YMR056C	-380.617	-373.182	-353.15
15:150000	YOL084W	YGR155W	-770.787	-769.346	-756.306
15:150000	YOL084W	YGR220C	-146.698	-143.916	-127.087
15:150000	YOL084W	YHR009C	-779.825	-768.469	-753.481
15:150000	YOL084W	YMR308C	141.183	145.97	192.394
15:150000	YOL084W	YER156C	-384.917	-378.998	-330.055
15:150000	YOL084W	YPL271W	-860.652	-849.752	-851.735
15:150000	YOL084W	YHR047C	-352.086	-345.425	-345.645
15:150000	YOL084W	YHR045W	-842.445	-834.124	-837.495
15:150000	YOL084W	YNL302C	-449.258	-443.841	-425.203
15:150000	YOL084W	YOR358W	-339.819	-333.275	-302.6
15:150000	YOL084W	YLR409C	1078.59	1082.9	1129.17
15:150000	YOL084W	YML126C	-1024.36	-1014.16	-1018.29
15:150000	YOL084W	YJR077C	-808.129	-797.575	-802.512
15:150000	YOL084W	YDR132C	-416.719	-408.605	-414.723
15:150000	YOL084W	YPR132W	-1048.05	-1046.07	-1011.55
15:150000	YOL084W	YPL053C	-827.284	-827.098	-813.573
15:150000	YOL084W	YOL113W	-110.112	-103.885	-104.058
15:150000	YOL084W	YLL055W	120.939	125.003	141.753
15:150000	YOL084W	YOR188W	-432.134	-423.02	-413.861
15:150000	YOL084W	YMR093W	679.315	684.557	693.385
15:150000	YOL084W	YMR011W	566.286	568.298	645.252
15:150000	YOL084W	YKL142W	-504.956	-504.76	-451.443
15:150000	YOL084W	YJR005W	-589.514	-584.713	-558.554
15:150000	YOL084W	YJR041C	553.021	558.755	614.342
15:150000	YOL084W	YOL095C	-272.853	-264.381	-233.507
15:150000	YOL084W	YPR018W	-454.267	-445.769	-451.871
15:150000	YOL084W	YPL040C	-56.1787	-52.3978	-0.69816
15:150000	YOL084W	YHR106W	-803.403	-795.93	-783.799
15:150000	YOL084W	YMR296C	-753.882	-745.675	-721.613
15:150000	YOL084W	YLR146C	-231.239	-219.059	-179.376
15:150000	YOL084W	YGL147C	-350.397	-341.891	-340.674
15:150000	YOL084W	YOR286W	-348.757	-338.746	-343.633
15:150000	YOL084W	YOL056W	-592.194	-583.13	-564.185

15:150000	YOL084W	YLR375W	-708.866	-702.153	-692.999
15:150000	YOL084W	YKL155C	23.9803	31.2764	26.9054
15:150000	YOL084W	YDR513W	-236.732	-226.605	-204.677
15:150000	YOL084W	YGR143W	-168.666	-168.25	-166.454
15:150000	YOL084W	YLL046C	170.593	171.677	218.883
15:150000	YOL084W	YGL103W	-1287.83	-1281.9	-1270.88
15:150000	YOL084W	YKL052C	-867.828	-857.139	-858.975
15:150000	YOL084W	YMR312W	-299.115	-291.734	-268.224
15:150000	YOL084W	YJL010C	593.265	593.864	639.786
15:150000	YOL084W	YBR148W	62.3331	74.5459	93.0481
15:150000	YOL084W	YGR174C	314.805	316.645	316.479
15:150000	YOL084W	YGL123W	-965.602	-957.825	-930.938
15:150000	YOL084W	YHR025W	-722.028	-710.924	-692.26
15:150000	YOL084W	YNL137C	-124.677	-116.32	-97.5286
15:150000	YOL084W	YHR088W	1007.4	1015.93	1072.2
15:150000	YOL084W	YJR122W	-199.305	-190.761	-189.876
15:150000	YOL084W	YNL227C	461.618	467.23	466.866
15:150000	YOL084W	YOR220W	-78.7442	-71.4646	-31.9168
15:150000	YOL084W	YGR077C	-891.961	-891.499	-856.865
15:150000	YOL084W	YGR102C	-153.2	-144.957	-50.5861
15:150000	YOL084W	YER082C	657.591	667.382	698.82
15:150000	YOL084W	YDR058C	-177.095	-171.347	-159.777
15:150000	YOL084W	YPL265W	-565.452	-558.678	-562.4
15:150000	YOL084W	YMR072W	-1055.44	-1046.43	-1017.99
15:150000	YOL084W	YDR332W	-712.902	-707.446	-687.661
15:150000	YOL084W	YJL062W-A	-131.523	-123.488	-129.631
15:150000	YOL084W	YJR043C	-554.559	-548.787	-553.531
15:150000	YOL084W	YBL030C	-736.718	-729.308	-735.306
15:150000	YOL084W	YGR118W	-1112.41	-1106.41	-1078.36
15:150000	YOL084W	YKL127W	-967.205	-956.525	-892.975
15:150000	YOL084W	YGL081W	190.706	191.856	199.01
15:150000	YOL084W	YDR421W	-345.668	-335.588	-324.724
15:150000	YOL084W	YDR091C	290.137	290.318	368.851
15:150000	YOL084W	YGR200C	613.95	619.875	677.244
15:150000	YOL084W	YGL076C	-1259.95	-1254.99	-1195.14
15:150000	YOL084W	YDR034W-B	225.805	233.39	301.682
15:150000	YOL084W	YOR001W	272.547	278.524	312.439
15:150000	YOL084W	YMR286W	-46.5876	-35.7859	-41.1882
15:150000	YOL084W	YNR060W	617.526	619.311	618.974
15:150000	YOL084W	YGR244C	-318.964	-315.651	-291.472
15:150000	YOL084W	YFR032C-A	-577.879	-568.646	-551.074
15:150000	YOL084W	YDL166C	-93.0166	-86.0523	-72.096
15:150000	YOL084W	YPL061W	-31.4566	-28.5751	20.1501
15:150000	YOL084W	YIL008W	-44.969	-36.5595	2.26589
15:150000	YOL084W	YDL211C	-381.978	-375.044	-330.804
15:150000	YOL084W	YPL156C	24.2178	26.9351	33.8152
15:150000	YOL084W	YOR063W	-1206.72	-1203.84	-1176.95

15:150000	YOL084W	YJR063W	572.142	577.689	617.858
15:150000	YOL084W	YKR079C	884.358	896.438	936.414
15:150000	YOL084W	YMR145C	121.148	127.261	131.936
15:150000	YOL084W	YBR025C	-753.644	-751.933	-704.599
15:150000	YOL084W	YGR285C	-1158.06	-1153.49	-1084.9
15:150000	YOL084W	YLL034C	925.65	929.04	984.154
15:150000	YOL084W	YML110C	-980.514	-980.513	-936.193
15:150000	YOL084W	YPR033C	-726.333	-722.034	-638.833
15:150000	YOL084W	YDR041W	-2.86639	6.93764	7.69292
15:150000	YOL084W	YDR197W	-497.853	-496.25	-475.644
15:150000	YOL084W	YAR029W	233.678	234.231	237.481
15:150000	YOL084W	YBR031W	-1429.95	-1423.58	-1367.79
15:150000	YOL084W	YOR315W	481.938	486.754	543.352
15:150000	YOL084W	YNL234W	-43.0964	-40.517	98.2148
15:150000	YOL084W	YER011W	-704.059	-701.945	-698.147
15:150000	YOL084W	YOL094C	-348.765	-340.923	-346.681
15:150000	YOL084W	YLL056C	-452.343	-444.739	-373.065
15:150000	YOL084W	YLR118C	-678.985	-672.681	-603.866
15:150000	YOL084W	YDL081C	-784.926	-783.244	-760.822
15:150000	YOL084W	YJR101W	-110.249	-100.003	-76.3199
15:150000	YOL084W	YDR324C	919.369	923.93	972.391
15:150000	YOL084W	YOR247W	-1017.27	-1011.44	-958.142
15:150000	YOL084W	YJL069C	687.084	695.112	732.659
15:150000	YOL084W	YGL025C	-491.216	-481.53	-486.486
15:150000	YOL084W	YHR203C	-1409.56	-1404.85	-1368.25
15:150000	YOL084W	YMR143W	-837.759	-835.607	-806.481
15:150000	YOL084W	YKL073W	-522.642	-512.259	-509.541
15:150000	YOL084W	YBR048W	-821.076	-820.389	-781.63
15:150000	YOL084W	YDR329C	-761.732	-760.271	-756.479
15:150000	YOL084W	YDR037W	-277.031	-265.539	-268.052
15:150000	YOL084W	YOR134W	531.988	539.56	580.526
15:150000	YOL084W	YNR001C	-483.95	-479.782	-450.376
15:150000	YOL084W	YHR160C	-61.1007	-57.7487	111.547
15:150000	YOL084W	YGR207C	-615.901	-607.31	-597.092
15:150000	YOL084W	YJR090C	-740.16	-730.418	-714.892
15:150000	YOL084W	YMR310C	203.943	211.107	252.333
15:150000	YOL084W	YGR255C	-262.874	-261.512	-253.894
15:150000	YOL084W	YDR482C	-462.241	-461.268	-366.075
15:150000	YOL084W	YAR075W	-573.008	-568.723	-539.817
15:150000	YOL084W	YDR104C	-483.378	-476.787	-462.429
15:150000	YOL084W	YDR298C	-599.98	-590.707	-595.076
15:150000	YOL084W	YEL071W	-544.844	-538.935	-538.857
15:150000	YOL084W	YDR434W	-1068.06	-1061.89	-1067.92
15:150000	YOL084W	YFR006W	-965.557	-958.949	-956.864
15:150000	YOL084W	YPR127W	-537.866	-528.278	-476.3
15:150000	YOL084W	YOL030W	-1113.65	-1105.08	-1108.09
15:150000	YOL084W	YGL030W	-1187.59	-1179.96	-1142.29

15:150000	YOL092W	YIL157C	-397.926	-397.748	-391.112
15:150000	YOL092W	YDR447C	-676.771	-674.544	-604.088
15:150000	YOL092W	YDR144C	173.822	176.392	175.706
15:150000	YOL092W	YDR447C	-309.463	-304.403	-210.239
15:150000	YOL092W	YGL121C	589.205	600.373	616.959
15:150000	YOL092W	YKL192C	-912.22	-902.973	-877.393
15:150000	YOL092W	YDL061C	-871.259	-862.717	-780.567
15:150000	YOL092W	YOL077W-A	-315.258	-306.746	-312.439
15:150000	YOL092W	YDL191W	-1161.54	-1151.38	-1103.72
15:150000	YOL092W	YAL003W	-780.988	-775.171	-689.582
15:150000	YOL092W	YLR029C	-1055.64	-1050.57	-987.948
15:150000	YOL092W	YMR128W	937.369	948.76	947.045
15:150000	YOL092W	YHR133C	-1038.3	-1029.26	-1012.86
15:150000	YOL092W	YNL055C	-1209.39	-1200.4	-1206.54
15:150000	YOL092W	YPL131W	-813.292	-802.427	-762.438
15:150000	YOL092W	YKR092C	538.432	547.188	596.175
15:150000	YOL092W	YDL085W	720.28	726.892	730.761
15:150000	YOL092W	YDR454C	-1220.95	-1212.15	-1153.43
15:150000	YOL092W	YJR123W	-1055.11	-1048.36	-1003.44
15:150000	YOL092W	YGR027C	-1089.43	-1079.12	-1037.75
15:150000	YOL092W	YGL253W	-627.007	-619.024	-516.372
15:150000	YOL092W	YDL229W	-53.7244	-47.5047	20.6399
15:150000	YOL092W	YLR185W	-445.525	-442.394	-354.147
15:150000	YOL092W	YLL028W	-293.686	-281.475	-258.37
15:150000	YOL092W	YHR005C-A	-570.162	-566.752	-528.538
15:150000	YOL092W	YKL081W	-778.508	-775.97	-656.618
15:150000	YOL092W	YGL031C	-953.698	-949.943	-902.833
15:150000	YOL092W	YER102W	-689.065	-683.533	-616.135
15:150000	YOL092W	YPL189W	-194.514	-184.517	-177.077
15:150000	YOL092W	YGR148C	-1071.26	-1063.82	-1013.76
15:150000	YOL092W	YGR214W	-316.984	-316.135	-229.851
15:150000	YOL092W	YDR345C	-138.218	-130.527	-127.425
15:150000	YOL092W	YAL042W	-1077.89	-1075.66	-1067.22
15:150000	YOL092W	YER007C-A	-843.883	-832.119	-802.608
15:150000	YOL092W	YOL061W	-314.739	-306.895	-305.707
15:150000	YOL092W	YBL068W	406.754	411.932	412.431
15:150000	YOL092W	YPL272C	228.388	237.27	309.472
15:150000	YOL092W	YDR090C	26.8046	34.0177	82.0099
15:150000	YOL092W	YJL109C	994.329	1004.75	1023.85
15:150000	YOL092W	YMR142C	-803.42	-796.221	-747.606
15:150000	YOL092W	YDL229W	-505.898	-497.397	-435.526
15:150000	YOL092W	YKL187C	-2.28507	2.52984	-1.33858
15:150000	YOL092W	YDR064W	-1030.8	-1030.02	-1017.74
15:150000	YOL092W	YGL111W	545.074	554.429	564.716
15:150000	YOL092W	YLR249W	-445.071	-435.919	-353.77
15:150000	YOL092W	YMR241W	-684.437	-672.616	-598.982
15:150000	YOL092W	YBR121C	-999.219	-991.722	-969.302

15:150000	YOL092W	YDR502C	-549.399	-537.423	-489.09
15:150000	YOL092W	YGL189C	-1002.46	-995.751	-949.708
15:150000	YOL092W	YPR043W	-1201.93	-1194.23	-1152.99
15:150000	YOL092W	YOL127W	-1168.86	-1164.1	-1108.33
15:150000	YOL092W	YOL022C	214.126	222.606	257.2
15:150000	YOL092W	YMR116C	-1117.66	-1108.51	-1082.5
15:150000	YOL092W	YGR201C	-181.144	-175.316	-114.738
15:150000	YOL092W	YPL043W	1186.35	1196.17	1192.15
15:150000	YOL092W	YLR150W	-1294.85	-1294.09	-1223.77
15:150000	YOL092W	YDR418W	-749.867	-749.219	-655.719
15:150000	YOL092W	YJL189W	-601.546	-595.518	-536.354
15:150000	YOL092W	YIR037W	-1011.63	-1000.08	-927.463
15:150000	YOL092W	YML063W	-720.276	-717.708	-664.764
15:150000	YOL092W	YNL044W	-910.297	-900.279	-897.474
15:150000	YOL092W	YPL221W	-556.82	-555.261	-529.755
15:150000	YOL092W	YKR094C	-567.237	-566.675	-480.807
15:150000	YOL092W	YEL050C	-77.3425	-67.6286	-73.149
15:150000	YOL092W	YDR012W	-312.277	-305.649	-252.164
15:150000	YOL092W	YGL022W	-1169.6	-1164.86	-1168.75
15:150000	YOL092W	YOR312C	-633.748	-631.947	-551.325
15:150000	YOL092W	YEL055C	110.641	120.469	120.795
15:150000	YOL092W	YDL213C	321.027	332.465	366.156
15:150000	YOL092W	YNL178W	-748.983	-738.8	-718.258
15:150000	YOL092W	YIL108W	-679.418	-668.896	-670.874
15:150000	YOL092W	YDR165W	897.326	904.865	930.22
15:150000	YOL092W	YGL040C	-415.047	-410.933	-321.584
15:150000	YOL092W	YDR060W	1220.1	1231.16	1251.52
15:150000	YOL092W	YKL004W	-210.609	-198.789	-171.038
15:150000	YOL092W	YDR032C	-1158.59	-1148.52	-1116.46
15:150000	YOL092W	YER165W	-986.417	-985.906	-869.497
15:150000	YOL092W	YPL183W-A	-25.2367	-18.4877	-22.7582
15:150000	YOL092W	YOL081W	-613.475	-612.939	-587.639
15:150000	YOL092W	YAL036C	845.095	854.591	872.982
15:150000	YOL092W	YOR046C	-817.739	-814.766	-810.483
15:150000	YOL092W	YDL130W	-731.017	-727.24	-665.334
15:150000	YOL092W	YPL207W	309.325	319.854	360.795
15:150000	YOL092W	YOR206W	1337.87	1349.7	1350.64
15:150000	YOL092W	YNR036C	-423.305	-420.596	-421.366
15:150000	YOL092W	YDL061C	-924.934	-916.121	-844.912
15:150000	YOL092W	YHL033C	-416.37	-412.839	-360.53
15:150000	YOL092W	YLR193C	-580.341	-574.061	-562.237
15:150000	YOL092W	YMR016C	-235.343	-232.088	-225.806
15:150000	YOL092W	YDL024C	35.8858	36.9448	57.5386
15:150000	YOL092W	YAL012W	-853.12	-847.179	-776.21
15:150000	YOL092W	YHL034C	-1260.95	-1248.94	-1193.33
15:150000	YOL092W	YCL057C-A	-805.322	-798.681	-804.571
15:150000	YOL092W	YML024W	-970.369	-964.229	-915.206

15:150000	YOL092W	YNL209W	-228.445	-222.574	-132.799
15:150000	YOL092W	YDR303C	-589.209	-581.793	-578.378
15:150000	YOL092W	YOR167C	-667.802	-664.05	-590.319
15:150000	YOL092W	YBL035C	-354.064	-348.571	-354.035
15:150000	YOL092W	YOL039W	-816.094	-807.227	-754.692
15:150000	YOL092W	YPL249C-A	-910.911	-907.551	-873.105
15:150000	YOL092W	YGR286C	-171.765	-169.752	-167.715
15:150000	YOL092W	YOR354C	-347.652	-338.719	-294.416
15:150000	YOL092W	YLR023C	-245.29	-237.252	-132.743
15:150000	YOL092W	YLR168C	88.4507	95.9129	116.507
15:150000	YOL092W	YPL012W	1321.52	1332.51	1337.22
15:150000	YOL092W	YGL078C	1466.44	1473.88	1498.28
15:150000	YOL092W	YOR107W	37.8531	39.4133	56.673
15:150000	YOL092W	YGR128C	1261.46	1269.64	1266.48
15:150000	YOL092W	YCR031C	-1031.19	-1020.51	-980.927
15:150000	YOL092W	YPL211W	951.574	962.262	996.562
15:150000	YOL092W	YJL191W	30.093	34.0661	132.598
15:150000	YOL092W	YHR183W	-943.047	-935.97	-789.321
15:150000	YOL092W	YHR068W	-800.361	-790.312	-699.178
15:150000	YOL092W	YMR038C	-843.255	-831.849	-802.271
15:150000	YOL092W	YKL046C	-1024.69	-1017.04	-991.716
15:150000	YOL092W	YGL077C	-491.163	-480.422	-472.076
15:150000	YOL092W	YMR056C	-388.406	-376.899	-353.008
15:150000	YOL092W	YDL051W	505.921	507.792	597.116
15:150000	YOL092W	YDL208W	555.941	566.989	590.666
15:150000	YOL092W	YDR161W	669.83	681.218	695.255
15:150000	YOL092W	YCR051W	-224.353	-217.52	-153.588
15:150000	YOL092W	YDR260C	-413.26	-404.29	-363.259
15:150000	YOL092W	YGR155W	-835.798	-835.513	-755.741
15:150000	YOL092W	YHR141C	-938.654	-933.235	-860.572
15:150000	YOL092W	YLR293C	-1367.95	-1360.47	-1284.57
15:150000	YOL092W	YGR220C	-165.757	-165.017	-127.326
15:150000	YOL092W	YMR308C	178.74	189.464	192.196
15:150000	YOL092W	YOR243C	253.938	261.43	304.563
15:150000	YOL092W	YER156C	-337.179	-331.18	-332.691
15:150000	YOL092W	YHR045W	-875.995	-866.635	-840.675
15:150000	YOL092W	YBR189W	-415.149	-414.5	-340.229
15:150000	YOL092W	YNL302C	-485.939	-480.943	-425.246
15:150000	YOL092W	YHL011C	275.62	279.927	342.352
15:150000	YOL092W	YML126C	-1045.79	-1033.48	-1018.86
15:150000	YOL092W	YJR077C	-809.482	-797.897	-801.977
15:150000	YOL092W	YOR335C	-658.057	-650.296	-606.912
15:150000	YOL092W	YDR132C	-436.442	-435.386	-414.332
15:150000	YOL092W	YPR132W	-1070.08	-1064.4	-1011.55
15:150000	YOL092W	YKL006W	-578.648	-574.216	-521.694
15:150000	YOL092W	YLR372W	-277.907	-266.574	-241.9
15:150000	YOL092W	YER074W	-508.699	-502.737	-452.314

15:150000	YOL092W	YFR031C-A	-261.587	-259.649	-189.219
15:150000	YOL092W	YOL113W	-117.668	-106.83	-104.393
15:150000	YOL092W	YHL033C	-490.474	-478.471	-445.524
15:150000	YOL092W	YML106W	-840.089	-833.537	-771.514
15:150000	YOL092W	YOL095C	-265.233	-259.718	-228.918
15:150000	YOL092W	YNL247W	-316.244	-311.455	-254.817
15:150000	YOL092W	YOL103W	-978.612	-970.736	-959.634
15:150000	YOL092W	YOR276W	-783.686	-772.221	-748.134
15:150000	YOL092W	YPR018W	-473.752	-462.005	-452.699
15:150000	YOL092W	YPL040C	-55.3281	-46.9037	0.0128543
15:150000	YOL092W	YDR023W	-1053.54	-1050.66	-968.132
15:150000	YOL092W	YOR286W	-359.685	-348.356	-344.16
15:150000	YOL092W	YOL056W	-587.954	-577.145	-563.984
15:150000	YOL092W	YNL302C	-943.421	-938.267	-887.908
15:150000	YOL092W	YER001W	-75.9909	-67.3076	-55.9074
15:150000	YOL092W	YLR228C	441.198	452.178	515.07
15:150000	YOL092W	YGL103W	-1329.73	-1326.41	-1271.89
15:150000	YOL092W	YKL052C	-862.786	-852.737	-858.459
15:150000	YOL092W	YMR312W	-297.404	-285.534	-268.879
15:150000	YOL092W	YBR148W	15.802	27.7501	91.391
15:150000	YOL092W	YBL087C	-795.529	-788.61	-715.521
15:150000	YOL092W	YGL123W	-982.68	-974.452	-931.987
15:150000	YOL092W	YNL137C	-163.822	-159.23	-97.0474
15:150000	YOL092W	YOR312C	-742.756	-739.927	-668.788
15:150000	YOL092W	YMR174C	440.372	447.766	469.009
15:150000	YOL092W	YNL069C	-750.406	-746.731	-678.116
15:150000	YOL092W	YJR122W	-198.053	-190.877	-188.618
15:150000	YOL092W	YCR034W	-391.043	-390.756	-280.418
15:150000	YOL092W	YKL216W	951.563	961.973	1015.91
15:150000	YOL092W	YPL265W	-604.07	-592.94	-562.832
15:150000	YOL092W	YJL062W-A	-136.493	-126.861	-130.614
15:150000	YOL092W	YER074W	-691.242	-686.23	-643.507
15:150000	YOL092W	YJR043C	-564.581	-556.088	-554.639
15:150000	YOL092W	YGR118W	-1112.8	-1101.21	-1077.69
15:150000	YOL092W	YOR096W	-547.546	-546.706	-478.127
15:150000	YOL092W	YDR091C	335.303	344.824	369.804
15:150000	YOL092W	YKR076W	16.5949	28.0525	86.4201
15:150000	YOL092W	YGR200C	667.336	675.157	675.518
15:150000	YOL092W	YHR064C	-811.413	-801.72	-728.489
15:150000	YOL092W	YMR297W	-1096.15	-1087.8	-1043.2
15:150000	YOL092W	YGL076C	-1246.03	-1237.62	-1196.78
15:150000	YOL092W	YMR286W	-47.0095	-37.1498	-41.7316
15:150000	YOL092W	YER056C	-369.165	-367.229	-361.974
15:150000	YOL092W	YFR032C-A	-627.005	-623.81	-551.293
15:150000	YOL092W	YPL156C	28.7933	39.1504	33.9375
15:150000	YOL092W	YER117W	-611.818	-607.307	-529.27
15:150000	YOL092W	YMR145C	123.185	134.981	131.849

15:150000	YOL092W	YGR285C	-1162.08	-1154.75	-1084.92
15:150000	YOL092W	YML110C	-963.016	-958.518	-941.592
15:150000	YOL092W	YDR197W	-512.941	-504.511	-476.071
15:150000	YOL092W	YLL045C	-738.844	-730.346	-689.084
15:150000	YOL092W	YER025W	-390.063	-388.595	-309.103
15:150000	YOL092W	YKL051W	199.447	203.827	207.623
15:150000	YOL092W	YNL234W	-67.4822	-65.1821	101.594
15:150000	YOL092W	YLR340W	-1046.57	-1043.66	-957.335
15:150000	YOL092W	YNL301C	-1113.25	-1111.14	-1055.89
15:150000	YOL092W	YOL094C	-349.076	-343.437	-347.256
15:150000	YOL092W	YLL056C	-438.484	-426.268	-371.877
15:150000	YOL092W	YLR118C	-641.077	-631.834	-604.605
15:150000	YOL092W	YOL040C	-786.743	-779.673	-735.691
15:150000	YOL092W	YNL061W	957.129	961.367	1011.94
15:150000	YOL092W	YKL014C	1004.7	1015.53	1039.83
15:150000	YOL092W	YDL081C	-825.85	-825.273	-761.884
15:150000	YOL092W	YJR101W	-104.198	-94.4015	-76.215
15:150000	YOL092W	YOR247W	-1044.56	-1039.98	-958.157
15:150000	YOL092W	YGR152C	-492.129	-487.889	-418.947
15:150000	YOL092W	YHR203C	-1433.69	-1432.01	-1368.1
15:150000	YOL092W	YBR048W	-862.546	-859.06	-781.773
15:150000	YOL092W	YGL135W	-1382.38	-1381.28	-1288.1
15:150000	YOL092W	YBR071W	-304.84	-298.954	-253.396
15:150000	YOL092W	YFL022C	-823.128	-819.533	-711.577
15:150000	YOL092W	YBR147W	821.054	827.164	834.818
15:150000	YOL092W	YOR134W	490.606	491.493	582.1
15:150000	YOL092W	YLR083C	-442.047	-433.124	-429.577
15:150000	YOL092W	YGR207C	-615.023	-604.523	-599.09
15:150000	YOL092W	YDL075W	-705.93	-702.798	-642.254
15:150000	YOL092W	YHL001W	-969.179	-962.832	-903.582
15:150000	YOL092W	YDR482C	-480.452	-474.72	-364.589
15:150000	YOL092W	YHR021C	-567.137	-566.955	-490.168
15:150000	YOL092W	YBR191W	-287.808	-287.686	-225.513
15:150000	YOL092W	YOR234C	-177.874	-165.765	-110.83
15:150000	YOL092W	YEL071W	-543.607	-534.306	-537.942
15:150000	YOL092W	YBL092W	-972.394	-970.588	-918.068
15:150000	YOL092W	YGR094W	-947.26	-938.239	-855.434
15:150000	YOL092W	YFR006W	-960	-951.931	-957.027
15:150000	YOL092W	YPR127W	-494.318	-487.238	-475.457
15:150000	YOL092W	YJL136C	-921.885	-919.843	-854.667
15:150000	YOL092W	YFR031C-A	-966.26	-955.027	-930.093
15:150000	YOL092W	YGL030W	-1209.7	-1200.68	-1142.36
15:150000	YOL092W	YPR028W	-1368.45	-1364.55	-1353.58
15:150000	YOL095C	YDL048C	473.193	485.12	509.474
15:150000	YOL095C	YDR447C	-603.813	-595.363	-597.027
15:150000	YOL095C	YHR002W	-313.36	-310.483	-289.863
15:150000	YOL095C	YDR447C	-211.25	-206.224	-210.388

15:150000	YOL095C	YJL165C	-302.081	-290.058	-260.493
15:150000	YOL095C	YKL096W	-461.195	-454.893	-460.333
15:150000	YOL095C	YNR055C	-635.738	-628.159	-632.012
15:150000	YOL095C	YOR084W	-92.7562	-81.2061	-67.2369
15:150000	YOL095C	YOL105C	-34.6984	-29.2884	14.839
15:150000	YOL095C	YMR227C	-412.933	-403.546	-389.769
15:150000	YOL095C	YBR084C-A	-318.954	-314.2	-314.882
15:150000	YOL095C	YAL003W	-682.13	-674.382	-680.427
15:150000	YOL095C	YDR450W	-819.289	-807.92	-812.715
15:150000	YOL095C	YML091C	415.588	423.981	432.83
15:150000	YOL095C	YKR092C	539.023	543.212	587.94
15:150000	YOL095C	YMR135C	-84.6592	-73.3171	30.9105
15:150000	YOL095C	YDL236W	-1016.61	-1009.76	-1015.65
15:150000	YOL095C	YOL090W	-425.843	-423.031	-411.908
15:150000	YOL095C	YOL036W	-695.916	-695.657	-695.196
15:150000	YOL095C	YJL016W	-9.59692	-7.05386	-6.85327
15:150000	YOL095C	YJR123W	-993.632	-987.72	-993.379
15:150000	YOL095C	YCR073C	797.995	808.885	802.739
15:150000	YOL095C	YGL031C	-897.277	-888.059	-893.655
15:150000	YOL095C	YER013W	-804.785	-792.639	-775.033
15:150000	YOL095C	YGL013C	-593.824	-588.712	-589.903
15:150000	YOL095C	YBL072C	-1296.06	-1284.08	-1290.2
15:150000	YOL095C	YER102W	-611.911	-604.6	-610.485
15:150000	YOL095C	YGL038C	-541.905	-535.535	-494.266
15:150000	YOL095C	YOR028C	1532.34	1534.01	1548.05
15:150000	YOL095C	YKR057W	-235.271	-231.346	-235.216
15:150000	YOL095C	YNR043W	-969.287	-962.95	-968.798
15:150000	YOL095C	YPL189W	-177.663	-169.157	-175.12
15:150000	YOL095C	YPR175W	-316.8	-311.792	-312.256
15:150000	YOL095C	YHR129C	-387.806	-377.422	-370.004
15:150000	YOL095C	YER149C	-447.866	-442.244	-436.107
15:150000	YOL095C	YKL037W	-43.2411	-41.9602	-43.0472
15:150000	YOL095C	YNR066C	680.885	688.685	683.035
15:150000	YOL095C	YNL073W	-273.263	-271.556	-268.517
15:150000	YOL095C	YLR249W	-353.322	-346.998	-352.737
15:150000	YOL095C	YHR072W	-371.158	-370.173	-366.628
15:150000	YOL095C	YBR023C	-285.443	-281.323	-275.48
15:150000	YOL095C	YDR349C	-276.285	-265.379	-269.689
15:150000	YOL095C	YGL096W	398.822	409.355	448.845
15:150000	YOL095C	YCL059C	809.499	815.06	810.413
15:150000	YOL095C	YGR166W	456.603	468.179	463.253
15:150000	YOL095C	YLR267W	801.769	805.334	803.355
15:150000	YOL095C	YDR490C	-113.289	-108.221	-107.451
15:150000	YOL095C	YGL189C	-937.085	-930.198	-935.7
15:150000	YOL095C	YDR528W	-45.0463	-41.8584	-42.0469
15:150000	YOL095C	YPR043W	-1139	-1134.14	-1138.52
15:150000	YOL095C	YPL132W	-12.8937	-3.42807	24.5383

15:150000	YOL095C	YER088C	-111.345	-99.6278	-50.416
15:150000	YOL095C	YOL127W	-1099.31	-1089.16	-1095.31
15:150000	YOL095C	YKL161C	810.258	815.788	815.321
15:150000	YOL095C	YDR306C	-535.935	-527.736	-483.073
15:150000	YOL095C	YER066W	-263.386	-259.632	-247.349
15:150000	YOL095C	YGL190C	-412.311	-401.391	-398.426
15:150000	YOL095C	YAL005C	593.867	599.637	594.965
15:150000	YOL095C	YML026C	-252.076	-246.255	-251.144
15:150000	YOL095C	YPR060C	-368.941	-361.895	-352.319
15:150000	YOL095C	YKL084W	-281.581	-272.856	-278.786
15:150000	YOL095C	YLR150W	-1212.01	-1202.02	-1208.15
15:150000	YOL095C	YDL235C	-519.217	-507.977	-513.4
15:150000	YOL095C	YJR055W	131.274	142.92	234.331
15:150000	YOL095C	YHR030C	-252.64	-252.072	-234.826
15:150000	YOL095C	YJR039W	-16.7781	-13.9168	-7.96288
15:150000	YOL095C	YJL189W	-535.994	-527.443	-533.373
15:150000	YOL095C	YFL054C	388.81	400.294	409.655
15:150000	YOL095C	YPL048W	-1194.62	-1186.11	-1190.68
15:150000	YOL095C	YML063W	-670.474	-661.602	-663.968
15:150000	YOL095C	YJR054W	-462.285	-451.73	-457.532
15:150000	YOL095C	YEL058W	-652.842	-645.765	-651.513
15:150000	YOL095C	YNL044W	-888.057	-877.92	-880.061
15:150000	YOL095C	YDR096W	682.816	689.731	696.92
15:150000	YOL095C	YOL117W	-328.748	-320.598	-296.472
15:150000	YOL095C	YIL105C	32.1267	33.7132	58.6187
15:150000	YOL095C	YOL152W	496.958	509.249	514.976
15:150000	YOL095C	YLR213C	291.341	300.16	296.776
15:150000	YOL095C	YNR034W	171.694	183.406	187.719
15:150000	YOL095C	YHR156C	-186.047	-179.251	-184.845
15:150000	YOL095C	YOR312C	-544.213	-540.542	-544.145
15:150000	YOL095C	YGR052W	1134.75	1136.64	1139.27
15:150000	YOL095C	YMR264W	-1015.44	-1008.57	-1001.62
15:150000	YOL095C	YOR291W	-81.9586	-79.4973	-72.6677
15:150000	YOL095C	YGL040C	-348.183	-338.279	-321.323
15:150000	YOL095C	YLR200W	-265.921	-257.152	-259.292
15:150000	YOL095C	YOR292C	-398.4	-390.008	-374.356
15:150000	YOL095C	YJL014W	-958.168	-951.673	-954.983
15:150000	YOL095C	YFR044C	-1105.19	-1093.43	-1092.9
15:150000	YOL095C	YOL081W	-637.056	-631.621	-584.305
15:150000	YOL095C	YKL096W-A	-1721.08	-1708.96	-1712.79
15:150000	YOL095C	YDR046C	591.206	597.578	607.112
15:150000	YOL095C	YBR105C	-85.5415	-78.2078	-52.825
15:150000	YOL095C	YHR195W	-151.025	-148.624	-126.999
15:150000	YOL095C	YPL175W	414.114	424.496	431.463
15:150000	YOL095C	YLL019C	-212.056	-208.165	-187.274
15:150000	YOL095C	YIR035C	-635.64	-627.373	-633.525
15:150000	YOL095C	YML024W	-905.839	-895.253	-900.436

15:150000	YOL095C	YNL209W	-133.448	-125.67	-130.422
15:150000	YOL095C	YBL035C	-369.486	-357.345	-347.775
15:150000	YOL095C	YJL181W	-437.257	-426.191	-432.311
15:150000	YOL095C	YOL039W	-742.347	-739.807	-742.247
15:150000	YOL095C	YOR354C	-330.968	-319.846	-294.256
15:150000	YOL095C	YPL015C	-576.589	-569.967	-559.082
15:150000	YOL095C	YER045C	-244.659	-235.59	-241.706
15:150000	YOL095C	YLR168C	98.8594	108.889	110.846
15:150000	YOL095C	YBR104W	526.55	538.827	538.171
15:150000	YOL095C	YLR194C	-61.5842	-52.8152	-36.3711
15:150000	YOL095C	YKL056C	-1434.19	-1427.31	-1433.46
15:150000	YOL095C	YKR017C	-62.0275	-52.5098	-41.8026
15:150000	YOL095C	YNL284C	-207.124	-204.545	-193.955
15:150000	YOL095C	YHR092C	954.332	962.064	968.578
15:150000	YOL095C	YCL051W	-220.617	-208.349	-159.939
15:150000	YOL095C	YDR351W	30.4878	37.2895	44.3178
15:150000	YOL095C	YER131W	334.437	343.14	336.997
15:150000	YOL095C	YFL036W	-174.661	-162.639	-167.878
15:150000	YOL095C	YGL077C	-470.025	-458.173	-457.172
15:150000	YOL095C	YKL109W	915.299	923.997	947.271
15:150000	YOL095C	YDR047W	-404.921	-404.433	-393.951
15:150000	YOL095C	YDR342C	947.89	958.281	953.36
15:150000	YOL095C	YHR141C	-858.307	-852.935	-854.872
15:150000	YOL095C	YOL034W	-403.194	-390.893	-388.088
15:150000	YOL095C	YGR220C	-159.605	-159.574	-124.81
15:150000	YOL095C	YDR494W	10.237	18.7372	33.5224
15:150000	YOL095C	YOL119C	193.813	194.659	220.938
15:150000	YOL095C	YJL210W	-399.066	-390.777	-332.73
15:150000	YOL095C	YLR084C	-303.38	-301.577	-302.689
15:150000	YOL095C	YMR136W	517.597	518.981	575.012
15:150000	YOL095C	YBR001C	217.381	220.559	238.109
15:150000	YOL095C	YNL302C	-423.87	-415.931	-420.929
15:150000	YOL095C	YML126C	-1007.86	-1003.58	-1007.36
15:150000	YOL095C	YJR077C	-837.605	-831.387	-796.098
15:150000	YOL095C	YDR216W	910.862	913.255	914.899
15:150000	YOL095C	YPR132W	-1000.75	-991.193	-996.761
15:150000	YOL095C	YPL053C	-808.059	-797.949	-803.589
15:150000	YOL095C	YIL158W	-162.955	-154.341	-146.358
15:150000	YOL095C	YLR143W	-619.892	-611.495	-614.48
15:150000	YOL095C	YNL218W	-609.285	-606.335	-607.073
15:150000	YOL095C	YNL053W	279.39	288.958	299.971
15:150000	YOL095C	YKL093W	334.438	342.714	336.756
15:150000	YOL095C	YAL040C	-30.1535	-28.5221	-23.8509
15:150000	YOL095C	YKR058W	519.437	520.383	532.348
15:150000	YOL095C	YPR018W	-469.202	-458.993	-447.252
15:150000	YOL095C	YPL040C	-52.2823	-42.5154	-3.66688
15:150000	YOL095C	YGL115W	-956.197	-948.401	-952.979

15:150000	YOL095C	YGR189C	-276.343	-268.296	-273.861
15:150000	YOL095C	YBR029C	-237.891	-229.351	-196.164
15:150000	YOL095C	YMR230W	-187.091	-181.975	-183.579
15:150000	YOL095C	YPR008W	-420.86	-410.39	-374.46
15:150000	YOL095C	YGL147C	-338.458	-328.716	-334.515
15:150000	YOL095C	YOL056W	-561.646	-551.464	-553.511
15:150000	YOL095C	YOR311C	-124.477	-118.33	-120.524
15:150000	YOL095C	YPL236C	-279.711	-276.144	-260.777
15:150000	YOL095C	YER001W	-59.8508	-53.6098	-54.4934
15:150000	YOL095C	YPL052W	436.82	443.758	474.579
15:150000	YOL095C	YKL155C	-4.38809	-0.481308	28.4338
15:150000	YOL095C	YGR143W	-177.122	-167.445	-160.579
15:150000	YOL095C	YLL046C	215.815	223.068	218.229
15:150000	YOL095C	YKL052C	-851.49	-841.93	-846.495
15:150000	YOL095C	YLR176C	144.859	149.818	151.624
15:150000	YOL095C	YGL123W	-917.779	-911.084	-917.149
15:150000	YOL095C	YNL137C	-137.392	-128.384	-97.5439
15:150000	YOL095C	YOR312C	-664.137	-659.038	-663.076
15:150000	YOL095C	YJR122W	-205.574	-200.464	-189.638
15:150000	YOL095C	YJL034W	-588.779	-581.352	-587.502
15:150000	YOL095C	YER130C	-302.267	-291.803	-295.145
15:150000	YOL095C	YOL116W	-640.743	-640.477	-569.55
15:150000	YOL095C	YMR295C	-1275.67	-1266.71	-1272.26
15:150000	YOL095C	YDR263C	47.3658	56.0241	82.6546
15:150000	YOL095C	YIL101C	823.208	825.197	836.143
15:150000	YOL095C	YKL087C	59.7261	71.7974	84.4825
15:150000	YOL095C	YER067W	1299.86	1302.78	1301.31
15:150000	YOL095C	YER074W	-636.957	-629.897	-635.772
15:150000	YOL095C	YJR043C	-555.257	-543.879	-542.547
15:150000	YOL095C	YPL014W	1041	1052.85	1057.25
15:150000	YOL095C	YJR127C	192.554	194.175	237.463
15:150000	YOL095C	YDR277C	1087.41	1098.32	1121.92
15:150000	YOL095C	YGR138C	286.99	296.761	295.853
15:150000	YOL095C	YGR118W	-1065.74	-1061.22	-1063.29
15:150000	YOL095C	YOR096W	-471.089	-465.618	-470.242
15:150000	YOL095C	YOR293W	-26.3361	-20.2734	-26.312
15:150000	YOL095C	YHR022C	791.638	793.251	791.75
15:150000	YOL095C	YJL118W	0.0905173	8.32824	25.4296
15:150000	YOL095C	YLR327C	1331.86	1338.18	1333.7
15:150000	YOL095C	YDL079C	903.941	915.377	929.223
15:150000	YOL095C	YOR274W	14.616	23.0657	23.2224
15:150000	YOL095C	YFR032C-A	-546.218	-537.493	-542.029
15:150000	YOL095C	YBL086C	396.961	406.615	441.617
15:150000	YOL095C	YER117W	-525.287	-521.226	-522.431
15:150000	YOL095C	YPL089C	243.08	251.356	250.573
15:150000	YOL095C	YPR036W	-1059.63	-1050.05	-1056.04
15:150000	YOL095C	YBR162W-A	-481.372	-473.401	-478.463

15:150000	YOL095C	YMR145C	104.278	112.185	129.987
15:150000	YOL095C	YDR420W	-321.483	-314.013	-313.837
15:150000	YOL095C	YBR025C	-695.552	-685.244	-691.377
15:150000	YOL095C	YPL219W	-317.273	-315.727	-301.302
15:150000	YOL095C	YIL021W	-872.982	-864.837	-857.518
15:150000	YOL095C	YDR041W	-15.5379	-10.828	5.46813
15:150000	YOL095C	YAR029W	234.676	236.178	241.304
15:150000	YOL095C	YIL036W	-305.636	-298.945	-255.543
15:150000	YOL095C	YKL120W	486.309	492.737	488.002
15:150000	YOL095C	YKL051W	202.971	207.845	211.445
15:150000	YOL095C	YLR340W	-944.434	-940.43	-943.397
15:150000	YOL095C	YOL094C	-397.777	-396.623	-340.569
15:150000	YOL095C	YBR181C	-1029.79	-1023.92	-1027.24
15:150000	YOL095C	YDL081C	-751.025	-742.646	-748.43
15:150000	YOL095C	YJR101W	-132.169	-122.931	-74.9271
15:150000	YOL095C	YHL024W	1032.53	1039.07	1037.59
15:150000	YOL095C	YGL025C	-489.507	-478.985	-478.171
15:150000	YOL095C	YAL041W	-768.584	-767.189	-765.982
15:150000	YOL095C	YGR152C	-413.53	-407.353	-412.901
15:150000	YOL095C	YGR238C	-122.957	-117.083	-118.503
15:150000	YOL095C	YGL130W	-768.825	-756.916	-748.684
15:150000	YOL095C	YHR203C	-1354.98	-1345.57	-1351.61
15:150000	YOL095C	YMR143W	-805.412	-797.656	-797.313
15:150000	YOL095C	YBL061C	321.504	333.697	328.961
15:150000	YOL095C	YKL073W	-506.154	-495.562	-500.825
15:150000	YOL095C	YGL135W	-1272.48	-1264.82	-1270.71
15:150000	YOL095C	YER060W	164.799	171.458	166.562
15:150000	YOL095C	YDR135C	-853.986	-846.582	-852.486
15:150000	YOL095C	YGR007W	-1008.93	-1002.75	-1008.9
15:150000	YOL095C	YJR090C	-705.329	-702.488	-704.015
15:150000	YOL095C	YJL112W	-202.89	-194.9	-190.715
15:150000	YOL095C	YML006C	-505.279	-503.15	-502.786
15:150000	YOL095C	YMR217W	452.99	464.547	461.897
15:150000	YOL095C	YBR191W	-223.178	-215.665	-220.369
15:150000	YOL095C	YGR060W	-1076.58	-1069.96	-1074.42
15:150000	YOL095C	YLR121C	517.011	529.232	523.616
15:150000	YOL095C	YKR013W	-169.765	-165.767	-159.935
15:150000	YOL095C	YPR149W	-113.467	-107.806	-106.924
15:150000	YOL095C	YOL087C	-445.179	-438.07	-442.74
15:150000	YOL095C	YMR238W	-612.367	-606.185	-610.07
15:150000	YOL095C	YDR434W	-1058.61	-1056.89	-1057.7
15:150000	YOL095C	YFR006W	-947.793	-942.329	-944.662
15:150000	YOL095C	YML082W	68.0553	72.5767	116.454
15:150000	YOL095C	YIL145C	-230.097	-218.178	-206.974
15:150000	YOL095C	YER132C	284.611	292.289	288.452
15:150000	YOL095C	YOR293W	-68.5689	-61.6818	-67.7811
15:150000	YOL089C	YER087C-B	-1077.78	-1067.97	-1053.88

15:150000	YOL089C	YIL160C	536.617	536.746	543.112
15:150000	YOL089C	YDR447C	-626.832	-619.862	-604.088
15:150000	YOL089C	YDR144C	168.522	173.376	175.706
15:150000	YOL089C	YDR447C	-225.432	-213.237	-210.239
15:150000	YOL089C	YNR055C	-659.494	-655.477	-641.691
15:150000	YOL089C	YGL121C	613.58	621.405	616.959
15:150000	YOL089C	YGR110W	265.977	275.303	282.455
15:150000	YOL089C	YDL061C	-806.004	-793.794	-780.567
15:150000	YOL089C	YPR117W	-476.448	-465.349	-462.39
15:150000	YOL089C	YGR174W-A	594.819	597.777	595.744
15:150000	YOL089C	YHR128W	-393	-385.871	-387.005
15:150000	YOL089C	YOL077W-A	-315.734	-310.049	-312.439
15:150000	YOL089C	YDL191W	-1116.05	-1104.1	-1103.72
15:150000	YOL089C	YBR084C-A	-338.57	-328.101	-321.609
15:150000	YOL089C	YAL003W	-714.465	-702.579	-689.582
15:150000	YOL089C	YDR450W	-843.127	-838.293	-825.583
15:150000	YOL089C	YHL032C	-497.593	-493.269	-492.441
15:150000	YOL089C	YLR029C	-996.094	-986.294	-987.948
15:150000	YOL089C	YIL052C	-471.357	-462.365	-455.494
15:150000	YOL089C	YNL055C	-1209.03	-1201.21	-1206.54
15:150000	YOL089C	YPL131W	-782.012	-769.966	-762.438
15:150000	YOL089C	YKR092C	542.785	554.366	596.175
15:150000	YOL089C	YMR135C	2.13172	4.05206	26.6037
15:150000	YOL089C	YDL085W	716.485	726.43	730.761
15:150000	YOL089C	YDL236W	-1037.91	-1027.93	-1028.66
15:150000	YOL089C	YDL223C	184.889	185.688	191.268
15:150000	YOL089C	YOL090W	-446.114	-440.581	-425.826
15:150000	YOL089C	YML124C	-1271.32	-1259.24	-1241.18
15:150000	YOL089C	YDR454C	-1176.56	-1164.8	-1153.43
15:150000	YOL089C	YML125C	-285.777	-279.382	-280.448
15:150000	YOL089C	YHL001W	-180.208	-171.349	-174.604
15:150000	YOL089C	YJL016W	-48.3226	-41.1242	-9.09653
15:150000	YOL089C	YJR123W	-1017.11	-1006.08	-1003.44
15:150000	YOL089C	YGR027C	-1046.34	-1037.33	-1037.75
15:150000	YOL089C	YGL253W	-527.521	-524.661	-516.372
15:150000	YOL089C	YDL229W	2.24609	12.4339	20.6399
15:150000	YOL089C	YKL180W	-463.647	-457.374	-449.988
15:150000	YOL089C	YMR301C	-151.091	-144.619	-138.877
15:150000	YOL089C	YLR185W	-372.758	-361.549	-354.147
15:150000	YOL089C	YLL028W	-265.36	-254.795	-258.37
15:150000	YOL089C	YKL081W	-673.592	-661.932	-656.618
15:150000	YOL089C	YCR073C	808.607	819.038	814.824
15:150000	YOL089C	YOL109W	-511.356	-502.163	-506.672
15:150000	YOL089C	YGL031C	-921.16	-911.854	-902.833
15:150000	YOL089C	YER013W	-804.426	-792.671	-781.184
15:150000	YOL089C	YGL013C	-684.59	-683.216	-598.296
15:150000	YOL089C	YBL072C	-1324.78	-1316.69	-1307.44

15:150000	YOL089C	YBL027W	-428.292	-423.982	-411.053
15:150000	YOL089C	YER102W	-634.05	-622.598	-616.135
15:150000	YOL089C	YKR057W	-252.843	-244.405	-232.441
15:150000	YOL089C	YNR043W	-998.51	-986.757	-983.906
15:150000	YOL089C	YGR085C	-67.0375	-57.4443	-58.1201
15:150000	YOL089C	YPL189W	-189.252	-178.96	-177.077
15:150000	YOL089C	YGR148C	-1032.4	-1021.42	-1013.76
15:150000	YOL089C	YPR175W	-328.317	-323.649	-320.341
15:150000	YOL089C	YGR214W	-241.692	-234.295	-229.851
15:150000	YOL089C	YDR345C	-130.272	-122.817	-127.425
15:150000	YOL089C	YER007C-A	-809.06	-800.088	-802.608
15:150000	YOL089C	YOL061W	-305.98	-302.641	-305.707
15:150000	YOL089C	YPL272C	285.011	297.083	309.472
15:150000	YOL089C	YOL097C	-348.613	-341.312	-345.518
15:150000	YOL089C	YOR341W	1179.3	1189.03	1183.73
15:150000	YOL089C	YHR129C	-381.127	-372.065	-374.897
15:150000	YOL089C	YJL109C	1019.57	1029.14	1023.85
15:150000	YOL089C	YER149C	-473.377	-463.301	-445.44
15:150000	YOL089C	YMR142C	-762.272	-753.664	-747.606
15:150000	YOL089C	YDL229W	-450.026	-438.472	-435.526
15:150000	YOL089C	YKL187C	-13.9281	-9.01121	-1.33858
15:150000	YOL089C	YKL037W	-54.3413	-51.6547	-48.2312
15:150000	YOL089C	YDR064W	-1024.28	-1024.24	-1017.74
15:150000	YOL089C	YNR066C	683.412	688.91	684.915
15:150000	YOL089C	YLR271W	-48.9337	-46.1994	-36.6484
15:150000	YOL089C	YPR163C	176.56	185.641	183.141
15:150000	YOL089C	YOR152C	-150.627	-140.049	-120.813
15:150000	YOL089C	YLR249W	-367.943	-356.508	-353.77
15:150000	YOL089C	YBR023C	-281.037	-277.637	-275.418
15:150000	YOL089C	YDR349C	-283.239	-280.912	-269.144
15:150000	YOL089C	YGL096W	452.072	455.947	455.269
15:150000	YOL089C	YMR241W	-623.246	-619.357	-598.982
15:150000	YOL089C	YCL059C	814.09	823.237	817.215
15:150000	YOL089C	YGR166W	454.077	458.437	469.087
15:150000	YOL089C	YLR267W	809.851	818.01	816.677
15:150000	YOL089C	YBR121C	-972.248	-967.445	-969.302
15:150000	YOL089C	YFR039C	-663.522	-659.437	-657.655
15:150000	YOL089C	YDR502C	-514.368	-504.126	-489.09
15:150000	YOL089C	YPR010C	1153.78	1162.53	1156.36
15:150000	YOL089C	YGL189C	-964.678	-955.103	-949.708
15:150000	YOL089C	YIL069C	265.408	276.62	281.273
15:150000	YOL089C	YPR043W	-1172.85	-1162.72	-1152.99
15:150000	YOL089C	YPL132W	2.87392	9.65446	30.0455
15:150000	YOL089C	YER088C	-70.8086	-60.757	-53.3988
15:150000	YOL089C	YAR002C-A	-1358.49	-1347.13	-1339.6
15:150000	YOL089C	YOL127W	-1130.26	-1120.36	-1108.33
15:150000	YOL089C	YKL161C	826.486	833.017	827.86

15:150000	YOL089C	YEL054C	329.96	340.759	343.911
15:150000	YOL089C	YEL047C	-891.861	-880.162	-868.819
15:150000	YOL089C	YML026C	-265.37	-255.558	-251.348
15:150000	YOL089C	YMR242C	-393.921	-389.024	-381.793
15:150000	YOL089C	YMR116C	-1088.19	-1081.17	-1082.5
15:150000	YOL089C	YGR031W	-291.425	-283.678	-252.777
15:150000	YOL089C	YKL084W	-280.127	-276.051	-278.182
15:150000	YOL089C	YLR150W	-1249.75	-1242.61	-1223.77
15:150000	YOL089C	YDL235C	-524.247	-512.639	-518.066
15:150000	YOL089C	YDR418W	-673.106	-663.909	-655.719
15:150000	YOL089C	YJR055W	210.293	214.61	225.21
15:150000	YOL089C	YGR033C	-631.564	-620.34	-586.302
15:150000	YOL089C	YJR039W	-16.357	-15.2814	-14.8343
15:150000	YOL089C	YJL189W	-554.125	-545.119	-536.354
15:150000	YOL089C	YFL054C	405.72	414.608	408.603
15:150000	YOL089C	YLR388W	-80.1249	-72.636	-64.8173
15:150000	YOL089C	YGR264C	-16.4769	-7.93369	-13.0886
15:150000	YOL089C	YPL048W	-1218.91	-1214.88	-1203.92
15:150000	YOL089C	YML063W	-680.692	-671.438	-664.764
15:150000	YOL089C	YNL044W	-911.778	-900.754	-897.474
15:150000	YOL089C	YLR388W	-183.263	-177.474	-170.686
15:150000	YOL089C	YLR175W	864.914	871.74	865.835
15:150000	YOL089C	YKR094C	-499.288	-490.161	-480.807
15:150000	YOL089C	YLR344W	-143.823	-132.945	-133.601
15:150000	YOL089C	YHR089C	781.448	791.692	788.391
15:150000	YOL089C	YOL117W	-301.04	-299.717	-300.685
15:150000	YOL089C	YOL152W	519.56	525.429	521.469
15:150000	YOL089C	YNL108C	-899.742	-894.903	-893.167
15:150000	YOL089C	YLR048W	-30.3176	-22.9204	-14.512
15:150000	YOL089C	YBL072C	-805.635	-797.231	-798.778
15:150000	YOL089C	YDR012W	-267.682	-255.371	-252.164
15:150000	YOL089C	YGL022W	-1202.63	-1190.33	-1168.75
15:150000	YOL089C	YHR156C	-187.485	-180.074	-182.3
15:150000	YOL089C	YOR312C	-567.544	-556.219	-551.325
15:150000	YOL089C	YEL055C	108.162	118.204	120.795
15:150000	YOL089C	YNL178W	-733.066	-722.268	-718.258
15:150000	YOL089C	YDR165W	927.515	935.825	930.22
15:150000	YOL089C	YMR229C	1165.8	1175	1168.97
15:150000	YOL089C	YIL018W	-249.516	-247.742	-234.312
15:150000	YOL089C	YGL040C	-354.721	-351.228	-321.584
15:150000	YOL089C	YOR292C	-395.235	-390.799	-383.391
15:150000	YOL089C	YJL014W	-996.392	-984.121	-968.25
15:150000	YOL089C	YGR034W	-254.206	-246.636	-236.706
15:150000	YOL089C	YEL026W	557.883	567.085	564.551
15:150000	YOL089C	YFR044C	-1111.65	-1111.46	-1107.35
15:150000	YOL089C	YER165W	-889.476	-877.745	-869.497
15:150000	YOL089C	YIL117C	103.906	104.395	104.966

15:150000	YOL089C	YER169W	191.166	192.529	204.73
15:150000	YOL089C	YOL080C	687.975	691.488	689.215
15:150000	YOL089C	YOL081W	-598.054	-587.73	-587.639
15:150000	YOL089C	YKL096W-A	-1740.4	-1733.64	-1737.29
15:150000	YOL089C	YMR165C	-480.136	-473.227	-475.285
15:150000	YOL089C	YDR046C	609.773	615.961	610.575
15:150000	YOL089C	YDL130W	-677.343	-669.399	-665.334
15:150000	YOL089C	YBR105C	-79.7243	-70.9786	-52.5813
15:150000	YOL089C	YMR118C	624.408	633.158	662.918
15:150000	YOL089C	YOR206W	1348.4	1356.27	1350.64
15:150000	YOL089C	YDL061C	-862.413	-850.777	-844.912
15:150000	YOL089C	YHL033C	-367.722	-362.126	-360.53
15:150000	YOL089C	YAL012W	-788.289	-779.834	-776.21
15:150000	YOL089C	YIL127C	784.295	789.557	785.801
15:150000	YOL089C	YDL083C	49.9636	58.1499	65.0061
15:150000	YOL089C	YDR098C	-1115.67	-1107.08	-1093.01
15:150000	YOL089C	YMR271C	731.775	743.355	740.592
15:150000	YOL089C	YLR448W	-134.455	-124.153	-123.172
15:150000	YOL089C	YIR035C	-640.254	-629.748	-635.647
15:150000	YOL089C	YCL057C-A	-804.715	-798.471	-804.571
15:150000	YOL089C	YML024W	-928.908	-917.634	-915.206
15:150000	YOL089C	YNL209W	-149.676	-137.664	-132.799
15:150000	YOL089C	YDR303C	-580.249	-573.836	-578.378
15:150000	YOL089C	YOR167C	-617.192	-608.213	-590.319
15:150000	YOL089C	YML042W	-37.7433	-29.8655	-18.7224
15:150000	YOL089C	YBL035C	-363.577	-353.12	-354.035
15:150000	YOL089C	YJL181W	-442.581	-442.074	-431.756
15:150000	YOL089C	YOL039W	-767.823	-755.868	-754.692
15:150000	YOL089C	YMR214W	-814.475	-805.543	-795.584
15:150000	YOL089C	YPL249C-A	-889.222	-882.84	-873.105
15:150000	YOL089C	YGR286C	-203.477	-192.695	-167.715
15:150000	YOL089C	YMR194W	-162.055	-151.817	-144.419
15:150000	YOL089C	YOR354C	-324.104	-318.15	-294.416
15:150000	YOL089C	YLR023C	-149.124	-139.45	-132.743
15:150000	YOL089C	YLR168C	114.316	122.508	116.507
15:150000	YOL089C	YDR025W	-36.6526	-26.7028	-19.4755
15:150000	YOL089C	YLR194C	-42.6006	-33.0129	-38.9629
15:150000	YOL089C	YKL056C	-1467.52	-1462.22	-1455.83
15:150000	YOL089C	YJL045W	83.9375	88.2212	87.4123
15:150000	YOL089C	YOR107W	51.99	55.2478	56.673
15:150000	YOL089C	YML056C	748.671	759.373	757.815
15:150000	YOL089C	YHR092C	970.333	980.074	979.446
15:150000	YOL089C	YCR031C	-993.693	-981.669	-980.927
15:150000	YOL089C	YDR351W	41.0509	51.5935	46.7762
15:150000	YOL089C	YER131W	320.893	331.633	341.416
15:150000	YOL089C	YJL191W	126.957	136.1	132.598
15:150000	YOL089C	YFL036W	-177.194	-169.654	-161.681

15:150000	YOL089C	YHR183W	-840.085	-837.411	-789.321
15:150000	YOL089C	YHR068W	-706.272	-698.288	-699.178
15:150000	YOL089C	YDR321W	123.161	134.117	135.266
15:150000	YOL089C	YMR038C	-857.992	-846.818	-802.271
15:150000	YOL089C	YKL046C	-1005.16	-1001.48	-991.716
15:150000	YOL089C	YLR325C	-230.441	-225.994	-209.59
15:150000	YOL089C	YGL077C	-502.389	-490.826	-472.076
15:150000	YOL089C	YMR056C	-376.837	-365.146	-353.008
15:150000	YOL089C	YDR500C	-234.488	-224.917	-213.196
15:150000	YOL089C	YLR301W	-984.835	-974.383	-976.525
15:150000	YOL089C	YDL051W	592.225	601.08	597.116
15:150000	YOL089C	YPL079W	-418.162	-411.595	-401.138
15:150000	YOL089C	YDL208W	586.063	593.749	590.666
15:150000	YOL089C	YHR007C	-808.624	-803.147	-806.865
15:150000	YOL089C	YCR051W	-156.073	-149.051	-153.588
15:150000	YOL089C	YDR260C	-370.693	-359.934	-363.259
15:150000	YOL089C	YGR155W	-786.3	-776.993	-755.741
15:150000	YOL089C	YLR061W	-216.512	-209.011	-204.441
15:150000	YOL089C	YHR141C	-882.05	-870.921	-860.572
15:150000	YOL089C	YLR293C	-1299.78	-1289.82	-1284.57
15:150000	YOL089C	YOL034W	-403.025	-392.34	-393.976
15:150000	YOL089C	YMR308C	190.51	196.963	192.196
15:150000	YOL089C	YDR494W	7.08759	8.78114	34.6416
15:150000	YOL089C	YOR005C	-650.529	-646.829	-646.694
15:150000	YOL089C	YER156C	-345.324	-337.103	-332.691
15:150000	YOL089C	YLR197W	900.56	908.295	902.455
15:150000	YOL089C	YOL119C	224.411	231.966	226.285
15:150000	YOL089C	YER043C	-971.07	-966.446	-967.622
15:150000	YOL089C	YLR333C	-513.922	-507.847	-498.591
15:150000	YOL089C	YHR045W	-861.791	-854.543	-840.675
15:150000	YOL089C	YGL146C	38.536	44.0606	44.8316
15:150000	YOL089C	YJL190C	-135.434	-131.822	-125.451
15:150000	YOL089C	YER110C	526.638	537.767	535.29
15:150000	YOL089C	YBR189W	-356.098	-347.059	-340.229
15:150000	YOL089C	YPL160W	-430.157	-420.358	-423.025
15:150000	YOL089C	YNL302C	-445.405	-434.773	-425.246
15:150000	YOL089C	YLR409C	1126.48	1132.52	1128.25
15:150000	YOL089C	YML126C	-1038.72	-1028.28	-1018.86
15:150000	YOL089C	YJR077C	-815.609	-804.949	-801.977
15:150000	YOL089C	YPR030W	183.121	185.552	183.592
15:150000	YOL089C	YDR132C	-420.625	-414.525	-414.332
15:150000	YOL089C	YPR132W	-1041	-1034.24	-1011.55
15:150000	YOL089C	YPL053C	-819.142	-807.967	-813.827
15:150000	YOL089C	YIL158W	-155.411	-146.748	-145.211
15:150000	YOL089C	YKL006W	-539.533	-530.864	-521.694
15:150000	YOL089C	YLR372W	-255.897	-245.778	-241.9
15:150000	YOL089C	YER074W	-465.691	-457.305	-452.314

15:150000	YOL089C	YFR031C-A	-200.86	-193.348	-189.219
15:150000	YOL089C	YLL055W	129.421	136.103	145.076
15:150000	YOL089C	YOR188W	-421.998	-414.714	-410.793
15:150000	YOL089C	YHL033C	-447.557	-441.092	-445.524
15:150000	YOL089C	YDL082W	275.826	283.883	286.9
15:150000	YOL089C	YMR011W	638.871	646.01	645.828
15:150000	YOL089C	YKL093W	337.089	347.739	343.427
15:150000	YOL089C	YML106W	-780.302	-771.759	-771.514
15:150000	YOL089C	YOL103W	-982.795	-973.908	-959.634
15:150000	YOL089C	YPR018W	-466.264	-454.786	-452.699
15:150000	YOL089C	YPL040C	-16.2847	-5.42616	0.0128543
15:150000	YOL089C	YLR432W	55.7116	63.0599	59.7967
15:150000	YOL089C	YGL115W	-963.208	-960.615	-961.652
15:150000	YOL089C	YER056C-A	-243.642	-241.664	-235.865
15:150000	YOL089C	YGR189C	-275.837	-270.008	-275.226
15:150000	YOL089C	YHR106W	-795.163	-788.019	-785.693
15:150000	YOL089C	YMR230W	-200.185	-188.259	-185.136
15:150000	YOL089C	YDL014W	745.998	756.716	753.64
15:150000	YOL089C	YDR023W	-975.66	-966.68	-968.132
15:150000	YOL089C	YGL147C	-357.94	-350.065	-340.51
15:150000	YOL089C	YOL056W	-580.54	-568.412	-563.984
15:150000	YOL089C	YNL302C	-894.904	-886.867	-887.908
15:150000	YOL089C	YPL236C	-274.893	-272.712	-265.563
15:150000	YOL089C	YER001W	-66.3477	-59.4791	-55.9074
15:150000	YOL089C	YNL255C	-88.1237	-80.3635	-84.0754
15:150000	YOL089C	YPL052W	477.331	482.576	481.063
15:150000	YOL089C	YLR375W	-694.868	-691.895	-692.942
15:150000	YOL089C	YKL155C	21.6208	26.0235	25.3628
15:150000	YOL089C	YLL046C	195.739	204.061	218.89
15:150000	YOL089C	YML073C	-322.665	-320.454	-318.137
15:150000	YOL089C	YGL103W	-1275.17	-1274.87	-1271.89
15:150000	YOL089C	YKL052C	-863.458	-852.839	-858.459
15:150000	YOL089C	YJL010C	634.479	640.258	638.609
15:150000	YOL089C	YBR148W	80.1526	89.5773	91.391
15:150000	YOL089C	YBL087C	-726.698	-715.592	-715.521
15:150000	YOL089C	YOL121C	68.5092	76.0637	85.4937
15:150000	YOL089C	YGL123W	-952.164	-941.306	-931.987
15:150000	YOL089C	YNL137C	-129.451	-126.996	-97.0474
15:150000	YOL089C	YOL102C	-826.978	-822.779	-809.342
15:150000	YOL089C	YOR312C	-692.389	-682.618	-668.788
15:150000	YOL089C	YOR186W	129.966	134.837	143.945
15:150000	YOL089C	YMR174C	464.717	469.284	469.009
15:150000	YOL089C	YNL069C	-690.453	-680.162	-678.116
15:150000	YOL089C	YJR122W	-197.817	-193.07	-188.618
15:150000	YOL089C	YCR034W	-299.757	-288.993	-280.418
15:150000	YOL089C	YJL034W	-624.796	-617.145	-585.976
15:150000	YOL089C	YER130C	-309.077	-306.508	-297.716

15:150000	YOL089C	YOL091W	611.617	617.624	683.741
15:150000	YOL089C	YHR010W	-538.02	-532.217	-523.295
15:150000	YOL089C	YGR077C	-872.788	-863.137	-857.209
15:150000	YOL089C	YGR102C	-109.979	-108.198	-51.1179
15:150000	YOL089C	YGR123C	739.582	750.225	745.15
15:150000	YOL089C	YMR295C	-1292.79	-1283.6	-1289.55
15:150000	YOL089C	YPL265W	-582.956	-571.745	-562.832
15:150000	YOL089C	YKL087C	47.6662	51.0108	87.0814
15:150000	YOL089C	YDR332W	-699.248	-691.281	-687.153
15:150000	YOL089C	YER074W	-665.602	-658.587	-643.507
15:150000	YOL089C	YJR043C	-583.455	-571.548	-554.639
15:150000	YOL089C	YDR277C	1124.23	1130.26	1126.11
15:150000	YOL089C	YGR138C	302.427	312.108	305.949
15:150000	YOL089C	YGR118W	-1096.68	-1085.4	-1077.69
15:150000	YOL089C	YKL127W	-902.003	-901.576	-895.936
15:150000	YOL089C	YDR421W	-340.292	-334.934	-323.77
15:150000	YOL089C	YOR096W	-493.801	-485.155	-478.127
15:150000	YOL089C	YLR406C	331.549	342.559	340.393
15:150000	YOL089C	YOR293W	-43.5833	-35.361	-27.2542
15:150000	YOL089C	YDR091C	367.36	372.873	369.804
15:150000	YOL089C	YHR064C	-747.948	-736.061	-728.489
15:150000	YOL089C	YGL076C	-1204.06	-1196.99	-1196.78
15:150000	YOL089C	YBR117C	575.26	582.898	599.125
15:150000	YOL089C	YHR022C	785.128	792.678	798.703
15:150000	YOL089C	YDL079C	945.438	951.155	945.562
15:150000	YOL089C	YFR032C-A	-573.564	-564.435	-551.293
15:150000	YOL089C	YDL211C	-344.443	-338.983	-333.041
15:150000	YOL089C	YHR111W	-446.689	-441.984	-444.932
15:150000	YOL089C	YER117W	-548.268	-537.073	-529.27
15:150000	YOL089C	YBR191W	-716.733	-711.002	-694.057
15:150000	YOL089C	YPL089C	246.081	256.521	250.917
15:150000	YOL089C	YPR036W	-1080.96	-1069.79	-1070.07
15:150000	YOL089C	YBR162W-A	-486.795	-477.484	-480.561
15:150000	YOL089C	YMR145C	116.97	126.106	131.849
15:150000	YOL089C	YDR420W	-320.006	-315.812	-319.079
15:150000	YOL089C	YBR025C	-715.109	-705.306	-704.73
15:150000	YOL089C	YLR441C	-411.701	-405.779	-400.809
15:150000	YOL089C	YGR285C	-1093.23	-1086.21	-1084.92
15:150000	YOL089C	YIL021W	-891.262	-882.055	-873.291
15:150000	YOL089C	YLL034C	981.781	986.792	982.932
15:150000	YOL089C	YHR019C	-831.379	-827.34	-831.364
15:150000	YOL089C	YDR041W	-9.90974	-9.4494	8.80161
15:150000	YOL089C	YDR197W	-480.511	-472.739	-476.071
15:150000	YOL089C	YLL045C	-701.114	-690.991	-689.084
15:150000	YOL089C	YER025W	-318.97	-310.362	-309.103
15:150000	YOL089C	YOR315W	523.923	530.111	543.389
15:150000	YOL089C	YKL051W	194.924	202.08	207.623

15:150000	YOL089C	YNL234W	80.6558	82.1883	101.594
15:150000	YOL089C	YLR340W	-966.04	-954.682	-957.335
15:150000	YOL089C	YNL301C	-1068.18	-1062.21	-1055.89
15:150000	YOL089C	YER011W	-699.181	-697.642	-697.042
15:150000	YOL089C	YOL094C	-369.706	-359.336	-347.256
15:150000	YOL089C	YLL056C	-400.799	-390.836	-371.877
15:150000	YOL089C	YOL040C	-750.197	-740.236	-735.691
15:150000	YOL089C	YBR181C	-1051.7	-1042.84	-1037.47
15:150000	YOL089C	YKL014C	1036.29	1044.99	1039.83
15:150000	YOL089C	YDL081C	-785.518	-778.386	-761.884
15:150000	YOL089C	YJR101W	-102.752	-95.5447	-76.215
15:150000	YOL089C	YDR324C	970.649	975.435	971.113
15:150000	YOL089C	YGL025C	-492.323	-481.758	-486.354
15:150000	YOL089C	YGR152C	-441.213	-430.335	-418.947
15:150000	YOL089C	YGL130W	-774.932	-765.116	-766.146
15:150000	YOL089C	YHR203C	-1384.42	-1374.62	-1368.1
15:150000	YOL089C	YMR143W	-823.092	-817.519	-807.427
15:150000	YOL089C	YBL061C	324.895	328.052	335.218
15:150000	YOL089C	YKL073W	-553.263	-550.822	-510.007
15:150000	YOL089C	YBR048W	-795.342	-783.636	-781.773
15:150000	YOL089C	YGL135W	-1307.55	-1295.96	-1288.1
15:150000	YOL089C	YBR071W	-285.399	-281.074	-253.396
15:150000	YOL089C	YFL022C	-727.444	-715.676	-711.577
15:150000	YOL089C	YBR147W	830.49	836.89	834.818
15:150000	YOL089C	YDR135C	-852.155	-849.358	-851.828
15:150000	YOL089C	YGR007W	-1040.15	-1027.98	-1018.5
15:150000	YOL089C	YJL177W	23.9466	31.0181	36.7529
15:150000	YOL089C	YLR083C	-430.322	-425.568	-429.577
15:150000	YOL089C	YJR094W-A	-37.514	-27.7477	-23.5006
15:150000	YOL089C	YJR090C	-732.204	-722.468	-713.131
15:150000	YOL089C	YDL075W	-657.224	-648.58	-642.254
15:150000	YOL089C	YJL112W	-198.387	-190.655	-193.498
15:150000	YOL089C	YMR310C	247.047	247.763	251.287
15:150000	YOL089C	YLR164W	329.932	330.232	333.44
15:150000	YOL089C	YHL001W	-920.089	-909.569	-903.582
15:150000	YOL089C	YDR482C	-401.499	-390.955	-364.589
15:150000	YOL089C	YPL123C	-44.4034	-42.6558	-35.2163
15:150000	YOL089C	YHR021C	-509.653	-501.988	-490.168
15:150000	YOL089C	YMR217W	449.362	461.317	463.077
15:150000	YOL089C	YBR191W	-236.911	-227.18	-225.513
15:150000	YOL089C	YOR234C	-115.16	-107.699	-110.83
15:150000	YOL089C	YGR060W	-1080.66	-1071.82	-1077.64
15:150000	YOL089C	YLR121C	527.52	537.988	532.075
15:150000	YOL089C	YKR013W	-160.363	-157.921	-155.794
15:150000	YOL089C	YEL071W	-551.202	-541.341	-537.942
15:150000	YOL089C	YBL092W	-928.801	-920.221	-918.068
15:150000	YOL089C	YMR238W	-622.896	-616.457	-620.989

15:150000	YOL089C	YGR094W	-864.474	-855.246	-855.434
15:150000	YOL089C	YDR434W	-1070.81	-1062	-1068.03
15:150000	YOL089C	YFR006W	-958.064	-953.48	-957.027
15:150000	YOL089C	YJL136C	-864.873	-858.502	-854.667
15:150000	YOL089C	YIL133C	-50.8736	-39.9522	-38.0413
15:150000	YOL089C	YFR031C-A	-943.442	-931.701	-930.093
15:150000	YOL089C	YOR293W	-82.7925	-72.9053	-67.0033
15:150000	YOL089C	YGL030W	-1152.97	-1141.92	-1142.36
15:150000	YOL089C	YPR028W	-1365.64	-1356.82	-1353.58
15:150000	YOL091W	YDR447C	-614.589	-611.133	-604.046
15:150000	YOL091W	YKL096W	-530.547	-525.034	-468.249
15:150000	YOL091W	YHR198C	-62.4888	-57.9953	-47.6455
15:150000	YOL091W	YLR149C	552.338	555.729	593.836
15:150000	YOL091W	YAL034C	60.2683	65.1239	217.68
15:150000	YOL091W	YPR117W	-463.962	-458.445	-461.446
15:150000	YOL091W	YOL105C	-108.053	-99.8802	17.4857
15:150000	YOL091W	YMR227C	-508.008	-499.397	-404.131
15:150000	YOL091W	YDR450W	-837.15	-830.37	-825.309
15:150000	YOL091W	YHL032C	-587.894	-580.758	-493.317
15:150000	YOL091W	YKL085W	-7.81811	-0.846961	73.5638
15:150000	YOL091W	YGR088W	927.355	937.623	1095.59
15:150000	YOL091W	YDL236W	-1080.01	-1070.61	-1026.79
15:150000	YOL091W	YML124C	-1245.73	-1238.79	-1243.63
15:150000	YOL091W	YMR008C	-409.061	-401.251	-356.426
15:150000	YOL091W	YOR178C	883.697	884.062	1040.7
15:150000	YOL091W	YER103W	473.594	482.032	535.802
15:150000	YOL091W	YJR121W	-730.467	-721.715	-723.751
15:150000	YOL091W	YHR087W	1039.06	1039.98	1205.64
15:150000	YOL091W	YER013W	-907.058	-900.016	-781.716
15:150000	YOL091W	YMR280C	485.267	490.82	579.828
15:150000	YOL091W	YNR043W	-1008.14	-995.857	-983.75
15:150000	YOL091W	YPL189W	-180.776	-177.548	-177.715
15:150000	YOL091W	YPR175W	-336.814	-330.052	-319.427
15:150000	YOL091W	YPL017C	494.097	501.211	636.645
15:150000	YOL091W	YKL035W	-134.904	-126.294	-107.842
15:150000	YOL091W	YDL199C	-97.9181	-92.111	-1.74141
15:150000	YOL091W	YKL162C	-37.131	-26.9405	55.8195
15:150000	YOL091W	YDR090C	42.705	54.7196	82.4519
15:150000	YOL091W	YER149C	-501.418	-489.349	-445.429
15:150000	YOL091W	YCR091W	386.851	398.259	484.523
15:150000	YOL091W	YKL037W	-85.0162	-75.6372	-48.7001
15:150000	YOL091W	YNL073W	-271.623	-265.695	-270.685
15:150000	YOL091W	YML054C	653.42	664.217	710.954
15:150000	YOL091W	YBR023C	-314.043	-307.86	-275.13
15:150000	YOL091W	YGL096W	319.738	325.217	454.875
15:150000	YOL091W	YGR053C	224.677	231.157	270.33
15:150000	YOL091W	YGR208W	-668.457	-658.6	-571.174

15:150000	YOL091W	YMR010W	-636.159	-626.622	-620.076
15:150000	YOL091W	YDR528W	-49.2368	-45.4282	-45.3893
15:150000	YOL091W	YPL132W	20.854	23.8203	29.9907
15:150000	YOL091W	YAR002C-A	-1364.91	-1354.42	-1338.93
15:150000	YOL091W	YDR306C	-558.374	-546.835	-492.845
15:150000	YOL091W	YDR273W	-200.478	-198.254	-172.859
15:150000	YOL091W	YPR191W	-84.5097	-77.1223	-66.5788
15:150000	YOL091W	YER066W	-374.888	-369.449	-254.894
15:150000	YOL091W	YER054C	878.875	881.928	1033.73
15:150000	YOL091W	YNL045W	-802.795	-801.373	-797.036
15:150000	YOL091W	YJL131C	72.3828	83.4925	86.9982
15:150000	YOL091W	YNL160W	516.971	528.15	606.889
15:150000	YOL091W	YKL072W	218.061	224.161	269.054
15:150000	YOL091W	YKR016W	-273.425	-262.599	-241.641
15:150000	YOL091W	YEL011W	681.867	688.757	806.341
15:150000	YOL091W	YDR202C	-423.243	-416.391	-326.727
15:150000	YOL091W	YPR091C	-359.492	-359.291	-272.517
15:150000	YOL091W	YHR207C	-736.444	-726.181	-712.475
15:150000	YOL091W	YGR175C	-602.469	-592.376	-572.75
15:150000	YOL091W	YML063W	-677.61	-676.642	-664.48
15:150000	YOL091W	YNL044W	-919.703	-910.426	-897.679
15:150000	YOL091W	YLR251W	295.893	307.401	390.833
15:150000	YOL091W	YDR504C	-250.118	-240.104	-194.587
15:150000	YOL091W	YJR008W	82.7882	93.1569	119.253
15:150000	YOL091W	YMR319C	246.432	250.879	317.644
15:150000	YOL091W	YBL013W	85.6097	90.3344	126.201
15:150000	YOL091W	YEL052W	-626.721	-623.923	-608.103
15:150000	YOL091W	YDR430C	97.0763	103.138	98.2128
15:150000	YOL091W	YBR003W	-197.458	-188.741	-186.152
15:150000	YOL091W	YMR264W	-1033.06	-1022.63	-1010.65
15:150000	YOL091W	YLR295C	-68.866	-59.3611	-62.1079
15:150000	YOL091W	YLR200W	-308.604	-298.643	-262.407
15:150000	YOL091W	YOR292C	-475.607	-466.43	-383.398
15:150000	YOL091W	YHR001W-A	-131.823	-125.941	-109.377
15:150000	YOL091W	YBL015W	424.278	430.782	478.947
15:150000	YOL091W	YLR382C	107.526	110.582	108.813
15:150000	YOL091W	YER162C	-340.761	-335.985	-302.11
15:150000	YOL091W	YOR086C	-555.951	-545.389	-534.336
15:150000	YOL091W	YOL081W	-604.918	-595.625	-588.488
15:150000	YOL091W	YNL098C	23.1616	29.4519	75.8826
15:150000	YOL091W	YJL141C	290.203	291.828	421.551
15:150000	YOL091W	YPL030W	12.1323	18.5882	59.8508
15:150000	YOL091W	YMR165C	-545.29	-537.463	-475.184
15:150000	YOL091W	YGR086C	-1093.95	-1084.23	-1066.41
15:150000	YOL091W	YBR105C	-113.727	-103.448	-54.4739
15:150000	YOL091W	YMR261C	-138.641	-130.817	-119.32
15:150000	YOL091W	YJL103C	317.109	326.041	382.557

15:150000	YOL091W	YLR446W	108.707	115.153	251.119
15:150000	YOL091W	YLR394W	-399.087	-390.625	-355.588
15:150000	YOL091W	YOL084W	828.791	833.425	888.484
15:150000	YOL091W	YFR014C	-203.443	-194.347	-165.455
15:150000	YOL091W	YHR195W	-278.425	-272.422	-134.928
15:150000	YOL091W	YHL036W	1.69922	10.6641	39.9457
15:150000	YOL091W	YLR355C	-262.02	-251.163	-233.694
15:150000	YOL091W	YIL097W	-73.8154	-65.3653	-12.6245
15:150000	YOL091W	YLL019C	-301.838	-294.156	-200.422
15:150000	YOL091W	YDR098C	-1099.22	-1096.72	-1092.12
15:150000	YOL091W	YCR030C	-460.073	-451.225	-437.81
15:150000	YOL091W	YML024W	-919.127	-912.532	-913.57
15:150000	YOL091W	YDR339C	-253.737	-250.044	-243.388
15:150000	YOL091W	YOL048C	30.1219	32.4525	163.681
15:150000	YOL091W	YDR185C	160.144	161.636	253.351
15:150000	YOL091W	YOL052C-A	1007.77	1015.16	1159.14
15:150000	YOL091W	YLR392C	-445.955	-441.661	-317.117
15:150000	YOL091W	YGL187C	-34.9789	-27.359	-16.4104
15:150000	YOL091W	YBL035C	-371.165	-359.15	-353.025
15:150000	YOL091W	YJL181W	-456.728	-449.697	-432.39
15:150000	YOL091W	YMR250W	739.129	748.836	825.228
15:150000	YOL091W	YNR033W	-384.845	-377.793	-383.002
15:150000	YOL091W	YGR248W	1195.79	1203.25	1350.69
15:150000	YOL091W	YGR286C	-183.047	-174.672	-163.442
15:150000	YOL091W	YOR354C	-303.924	-291.946	-295.32
15:150000	YOL091W	YNL100W	-126.698	-116.548	-42.5106
15:150000	YOL091W	YPL108W	58.6787	60.5842	134.738
15:150000	YOL091W	YPL015C	-653.602	-647.415	-577.323
15:150000	YOL091W	YDL214C	293.025	295.976	322.562
15:150000	YOL091W	YML100W	982.539	989.117	1111.6
15:150000	YOL091W	YPL011C	-554.251	-547.936	-477.532
15:150000	YOL091W	YNL284C	-202.966	-199.451	-194.321
15:150000	YOL091W	YLR102C	-504.352	-503.455	-459.685
15:150000	YOL091W	YML128C	564.257	573.185	652.975
15:150000	YOL091W	YNL081C	48.7089	58.6175	52.4615
15:150000	YOL091W	YPL119C-A	-98.1136	-88.8909	-67.4819
15:150000	YOL091W	YHR187W	-525.159	-515.17	-473.187
15:150000	YOL091W	YOR233W	-272.823	-260.76	-196.812
15:150000	YOL091W	YGL077C	-494.396	-482.443	-470.046
15:150000	YOL091W	YLR301W	-1004.12	-996.054	-977.669
15:150000	YOL091W	YHR007C	-808.214	-800.989	-806.552
15:150000	YOL091W	YPL109C	-352.641	-347.424	-300.669
15:150000	YOL091W	YPR161C	-764.12	-754.284	-733.558
15:150000	YOL091W	YGR220C	-143.164	-142.789	-127.087
15:150000	YOL091W	YDR494W	31.4544	38.5109	34.6203
15:150000	YOL091W	YOR005C	-648.027	-642.475	-643.612
15:150000	YOL091W	YDL206W	-247.908	-236.502	-202.333

15:150000	YOL091W	YNL194C	1021.21	1028.81	1095.16
15:150000	YOL091W	YML071C	-697.919	-686.382	-670.373
15:150000	YOL091W	YMR272C	-759.494	-748.066	-681.963
15:150000	YOL091W	YIL099W	154.399	160.431	193.38
15:150000	YOL091W	YLR084C	-358.388	-346.196	-303.386
15:150000	YOL091W	YIL077C	-171.126	-160.768	-26.541
15:150000	YOL091W	YIL136W	634.015	644.912	740.824
15:150000	YOL091W	YPL271W	-851.755	-849.244	-851.735
15:150000	YOL091W	YFR053C	1126.42	1126.75	1219.83
15:150000	YOL091W	YJR077C	-831.851	-825.441	-802.512
15:150000	YOL091W	YJR036C	-45.7838	-42.0916	85.2229
15:150000	YOL091W	YPR030W	88.3432	90.6514	180.736
15:150000	YOL091W	YDR132C	-423.844	-418.791	-414.723
15:150000	YOL091W	YPR132W	-1018.23	-1016.28	-1011.55
15:150000	YOL091W	YKL151C	123.532	126.748	152.556
15:150000	YOL091W	YOR348C	1437	1441.28	1529.52
15:150000	YOL091W	YIL158W	-225.763	-218.726	-146.938
15:150000	YOL091W	YLR143W	-687.957	-680.152	-626.707
15:150000	YOL091W	YMR211W	-213.821	-205.058	-207.612
15:150000	YOL091W	YOR188W	-435.749	-429.601	-413.861
15:150000	YOL091W	YMR181C	-87.366	-76.8197	3.07535
15:150000	YOL091W	YBR280C	-102.163	-93.1384	-43.6656
15:150000	YOL091W	YGL209W	408.024	414.291	413.263
15:150000	YOL091W	YFL042C	-300.925	-295.257	-276.974
15:150000	YOL091W	YPR018W	-464.502	-453.439	-451.871
15:150000	YOL091W	YPL040C	-3.69922	4.04082	-0.69816
15:150000	YOL091W	YGL115W	-990.017	-980.604	-961.367
15:150000	YOL091W	YHR080C	-205.016	-193.914	-87.5247
15:150000	YOL091W	YPR008W	-466.623	-466.361	-381.498
15:150000	YOL091W	YGL147C	-353.327	-352.469	-340.674
15:150000	YOL091W	YOL056W	-566.372	-558.172	-564.185
15:150000	YOL091W	YGL037C	-195.111	-189.428	-131.397
15:150000	YOL091W	YPL236C	-296.395	-286.093	-263.954
15:150000	YOL091W	YLL041C	68.6726	75.2958	143.201
15:150000	YOL091W	YGR237C	-184.607	-177.748	-98.3375
15:150000	YOL091W	YPR026W	-68.6943	-62.8794	-27.4801
15:150000	YOL091W	YGR143W	-248.766	-242.172	-166.454
15:150000	YOL091W	YKL052C	-862.979	-853.754	-858.975
15:150000	YOL091W	YNL137C	-109.664	-97.6138	-97.5286
15:150000	YOL091W	YKR093W	140.458	148.36	228.766
15:150000	YOL091W	YOR186W	110.885	110.904	145.15
15:150000	YOL091W	YOR220W	-72.7436	-68.1499	-31.9168
15:150000	YOL091W	YBR034C	434.383	435.674	442.614
15:150000	YOL091W	YBR072W	377.734	386.514	390.118
15:150000	YOL091W	YJL005W	-87.0204	-74.8958	-39.2434
15:150000	YOL091W	YML018C	227.973	234.955	267.088
15:150000	YOL091W	YOL116W	-659.325	-650.115	-568.081

15:150000	YOL091W	YMR295C	-1311.66	-1299.58	-1290.19
15:150000	YOL091W	YDR263C	-27.8538	-23.1163	84.3968
15:150000	YOL091W	YIL101C	685.26	687.342	843.071
15:150000	YOL091W	YGL058W	-874.166	-868.652	-852.445
15:150000	YOL091W	YJR043C	-559.758	-550.146	-553.531
15:150000	YOL091W	YER107C	-975.357	-972.426	-960.268
15:150000	YOL091W	YOR227W	-393.525	-386.008	-369.614
15:150000	YOL091W	YJL118W	-127.406	-123.956	13.1279
15:150000	YOL091W	YCR043C	-693.077	-680.756	-667.903
15:150000	YOL091W	YPL156C	21.2491	22.596	33.8152
15:150000	YOL091W	YIL039W	-1024.99	-1014.92	-1014.52
15:150000	YOL091W	YBR162W-A	-508.74	-496.536	-480.073
15:150000	YOL091W	YOL054W	-210.316	-198.931	-154.409
15:150000	YOL091W	YGL145W	-540.638	-538.187	-530.477
15:150000	YOL091W	YPL219W	-382.11	-371.203	-309.786
15:150000	YOL091W	YGR194C	231.771	239.808	283.884
15:150000	YOL091W	YIL021W	-886.625	-878.367	-871.727
15:150000	YOL091W	YNR002C	978.932	983.692	1140.54
15:150000	YOL091W	YDR041W	-5.76715	-4.82269	7.69292
15:150000	YOL091W	YAR029W	233.129	242.902	237.481
15:150000	YOL091W	YKL120W	435.873	441.653	498.516
15:150000	YOL091W	YKL051W	171.876	180.33	205.401
15:150000	YOL091W	YHR144C	-293.81	-282.934	-205.271
15:150000	YOL091W	YKL104C	-797.86	-789.612	-774.931
15:150000	YOL091W	YDR198C	-7.43752	-4.52399	-5.87224
15:150000	YOL091W	YJL011C	384.531	387.649	402.682
15:150000	YOL091W	YMR105C	989.479	998.405	1099.38
15:150000	YOL091W	YJR101W	-93.4147	-87.7527	-76.3199
15:150000	YOL091W	YPL186C	580.45	589.239	645.331
15:150000	YOL091W	YGL025C	-510.456	-499.192	-486.486
15:150000	YOL091W	YLR258W	654.156	662.811	773.905
15:150000	YOL091W	YGL130W	-783.12	-772.082	-762.414
15:150000	YOL091W	YCL040W	304.479	311.247	343.11
15:150000	YOL091W	YMR143W	-822.028	-821.008	-806.481
15:150000	YOL091W	YOR288C	39.7856	45.9926	61.7802
15:150000	YOL091W	YHR104W	27.8018	29.0385	78.1597
15:150000	YOL091W	YBR126C	158.302	166.951	221.398
15:150000	YOL091W	YNR001C	-473.919	-464.105	-450.376
15:150000	YOL091W	YDR135C	-903.766	-895.999	-851.807
15:150000	YOL091W	YGR007W	-1042.81	-1031.13	-1019.01
15:150000	YOL091W	YDR070C	647.501	651.389	697.991
15:150000	YOL091W	YBR030W	-239.881	-228.847	-148.024
15:150000	YOL091W	YGR060W	-1135.48	-1132.29	-1078.19
15:150000	YOL091W	YPR160W	1097.55	1106.11	1211.3
15:150000	YOL091W	YKR013W	-206.349	-204.315	-155.35
15:150000	YOL091W	YNR034W-A	1175.5	1187.4	1252.63
15:150000	YOL091W	YPL004C	-301.279	-298.719	-208.648

15:150000	YOL091W	YLR201C	-502.332	-496.866	-486.98
15:150000	YOL091W	YDR298C	-596.576	-589.185	-595.076
15:150000	YOL091W	YPL100W	-744.973	-739.921	-621.291
15:150000	YOL091W	YEL071W	-554.972	-544.817	-538.857
15:150000	YOL091W	YGL255W	912.68	921.719	923.309
15:150000	YOL091W	YDR075W	-239.053	-227.401	-200.006
15:150000	YOL091W	YPL203W	-52.3886	-47.2131	-43.6113
15:150000	YOL091W	YMR195W	-159.757	-154.897	-92.1656
15:150000	YOL091W	YMR108W	-12.3971	-2.37361	-5.64281
15:150000	YOL091W	YDR434W	-1071.18	-1062.53	-1067.92
15:150000	YOL091W	YML082W	45.7478	55.3614	112.35
15:150000	YOL091W	YLL023C	-641.797	-633.106	-565.313
15:150000	YOL091W	YOL082W	-98.9634	-89.7659	25.3106
15:150000	YOL094C	YDR447C	-642.32	-631.891	-560.121
15:150000	YOL094C	YJL185C	50.7381	56.3091	170.572
15:150000	YOL094C	YDR447C	-240.791	-229.424	-195.489
15:150000	YOL094C	YNR055C	-668.384	-666.716	-613.467
15:150000	YOL094C	YHR197W	1009.57	1016.72	1067.97
15:150000	YOL094C	YGL121C	483.529	495.425	575.156
15:150000	YOL094C	YGR110W	207.324	210.391	278.771
15:150000	YOL094C	YKL192C	-818.945	-813.112	-817.989
15:150000	YOL094C	YDL061C	-792.246	-781.222	-719.005
15:150000	YOL094C	YPR117W	-462.198	-454.588	-439.564
15:150000	YOL094C	YHR128W	-435.691	-432.238	-369.797
15:150000	YOL094C	YOL077W-A	-313.248	-303.593	-301.899
15:150000	YOL094C	YDL191W	-1075.22	-1063.13	-1025
15:150000	YOL094C	YBR084C-A	-343.788	-332.114	-294.694
15:150000	YOL094C	YAL003W	-704.071	-692.729	-641.643
15:150000	YOL094C	YDR450W	-854.743	-850.576	-779.303
15:150000	YOL094C	YLR029C	-968.852	-960.512	-914.98
15:150000	YOL094C	YMR128W	837.034	848.083	891.337
15:150000	YOL094C	YIL052C	-469.299	-459.01	-418.418
15:150000	YOL094C	YHR133C	-975.037	-970.314	-960.063
15:150000	YOL094C	YMR131C	844.745	849.639	933.607
15:150000	YOL094C	YNL055C	-1131.04	-1120.59	-1126.23
15:150000	YOL094C	YPL131W	-747.256	-735.106	-699.597
15:150000	YOL094C	YDL085W	578.883	589.28	684.234
15:150000	YOL094C	YDL173W	-650.869	-648.946	-609.973
15:150000	YOL094C	YHR065C	679.459	685.524	731.229
15:150000	YOL094C	YDR454C	-1146.27	-1134.4	-1086.64
15:150000	YOL094C	YHL001W	-254.59	-243.045	-159.282
15:150000	YOL094C	YJR123W	-968.041	-956.413	-925.272
15:150000	YOL094C	YGR027C	-1016.08	-1008.83	-966.288
15:150000	YOL094C	YGL253W	-536.427	-533.961	-489.274
15:150000	YOL094C	YDL229W	-45.0394	-33.0332	29.5302
15:150000	YOL094C	YKL180W	-454.491	-444.056	-408.215
15:150000	YOL094C	YMR301C	-214.671	-208.876	-142.692

15:150000	YOL094C	YLR185W	-356.522	-344.433	-317.459
15:150000	YOL094C	YML093W	889.426	899.809	960.754
15:150000	YOL094C	YLL028W	-280.244	-268.714	-238.644
15:150000	YOL094C	YKL081W	-693.524	-682.893	-604.424
15:150000	YOL094C	YGL031C	-886.986	-878.842	-837.685
15:150000	YOL094C	YER013W	-742.686	-737.225	-742.519
15:150000	YOL094C	YGL013C	-689.742	-678.802	-558.12
15:150000	YOL094C	YBL072C	-1292.68	-1287.95	-1219.56
15:150000	YOL094C	YBL027W	-431.845	-423.129	-373.623
15:150000	YOL094C	YER102W	-632.062	-620.094	-571.422
15:150000	YOL094C	YKR057W	-266.123	-254.589	-215.014
15:150000	YOL094C	YPL036W	101.928	104.958	420.98
15:150000	YOL094C	YGR085C	-117.314	-107.789	-55.6773
15:150000	YOL094C	YPL189W	-177.062	-166.306	-158.051
15:150000	YOL094C	YGR148C	-992.27	-980.369	-944.895
15:150000	YOL094C	YGR214W	-241.304	-230.386	-204.698
15:150000	YOL094C	YDR345C	-131.438	-125.054	-118.369
15:150000	YOL094C	YAL042W	-1015.29	-1012.77	-996.857
15:150000	YOL094C	YER007C-A	-874.587	-865.404	-743.382
15:150000	YOL094C	YOL061W	-350.664	-345.716	-288.714
15:150000	YOL094C	YPL272C	252.192	263.104	303.402
15:150000	YOL094C	YOL097C	-417.383	-406.341	-329.418
15:150000	YOL094C	YOR341W	1029.08	1035.72	1110.13
15:150000	YOL094C	YDR090C	73.8801	78.4455	89.4438
15:150000	YOL094C	YJL109C	840.165	847.765	957.122
15:150000	YOL094C	YMR142C	-724.008	-712.751	-685.455
15:150000	YOL094C	YDL229W	-458.163	-447.025	-394.33
15:150000	YOL094C	YKL187C	3.29419	9.10368	14.2933
15:150000	YOL094C	YPR163C	88.7321	95.8139	173.108
15:150000	YOL094C	YOR152C	-161.074	-157.026	-121.195
15:150000	YOL094C	YOL120C	-537.563	-531.503	-489.025
15:150000	YOL094C	YDL031W	305.889	316.556	363.269
15:150000	YOL094C	YGL111W	468.088	475.231	528.225
15:150000	YOL094C	YLR249W	-394.428	-386.136	-323.299
15:150000	YOL094C	YLR222C	1043.9	1043.99	1090.47
15:150000	YOL094C	YMR241W	-616.216	-610.08	-572.66
15:150000	YOL094C	YER006W	892.027	900.25	935.328
15:150000	YOL094C	YCL059C	720.086	729.376	768.232
15:150000	YOL094C	YLR267W	759.181	768.8	777.115
15:150000	YOL094C	YFR039C	-623.465	-618.661	-622.238
15:150000	YOL094C	YDR502C	-522.076	-514.73	-452.012
15:150000	YOL094C	YPR010C	1017.16	1026.08	1084.5
15:150000	YOL094C	YER126C	801.863	803.366	811.81
15:150000	YOL094C	YGL189C	-952.675	-943.906	-877.833
15:150000	YOL094C	YIL069C	201.405	213.507	267.697
15:150000	YOL094C	YPR043W	-1109.5	-1101.09	-1074.54
15:150000	YOL094C	YPL132W	-63.123	-57.7851	27.9996

15:150000	YOL094C	YOL127W	-1079.37	-1068.91	-1030.3
15:150000	YOL094C	YEL054C	253.919	265.425	328.819
15:150000	YOL094C	YEL047C	-932.437	-924.338	-807.618
15:150000	YOL094C	YML026C	-271.411	-259.616	-228.072
15:150000	YOL094C	YMR242C	-393.498	-384.506	-344.175
15:150000	YOL094C	YMR116C	-1066.74	-1064.3	-999.922
15:150000	YOL094C	YGR031W	-459.693	-453.388	-239.449
15:150000	YOL094C	YPL043W	1036.83	1045.91	1119.36
15:150000	YOL094C	YLR150W	-1219.02	-1213.05	-1137.67
15:150000	YOL094C	YDL235C	-508.341	-507.898	-483.566
15:150000	YOL094C	YDR418W	-653.508	-641.714	-601.387
15:150000	YOL094C	YGR033C	-687.048	-675.316	-549.026
15:150000	YOL094C	YJL189W	-553.648	-543.933	-505.938
15:150000	YOL094C	YIR037W	-920.994	-912.255	-859.118
15:150000	YOL094C	YLR388W	-108.743	-99.523	-64.0088
15:150000	YOL094C	YGR264C	-68.8447	-57.689	-12.8142
15:150000	YOL094C	YPL048W	-1229.67	-1220.46	-1124.47
15:150000	YOL094C	YML063W	-687.79	-681.349	-626.864
15:150000	YOL094C	YNL044W	-858.785	-850.365	-837.213
15:150000	YOL094C	YLR388W	-196.624	-189.034	-156.643
15:150000	YOL094C	YLR175W	693.331	697.012	813.977
15:150000	YOL094C	YPL221W	-518.473	-516.202	-490.752
15:150000	YOL094C	YKR094C	-489.806	-478.077	-441.83
15:150000	YOL094C	YGL065C	-791.947	-783.019	-611.304
15:150000	YOL094C	YEL050C	-66.6963	-59.8572	-64.8115
15:150000	YOL094C	YLR344W	-179.666	-167.746	-123.84
15:150000	YOL094C	YHR089C	613.969	617.601	739.389
15:150000	YOL094C	YLR048W	-55.0485	-44.2541	-5.8205
15:150000	YOL094C	YBL072C	-778.112	-767.293	-730.711
15:150000	YOL094C	YDR012W	-268.033	-256.612	-234.128
15:150000	YOL094C	YOR312C	-561.051	-549.493	-506.017
15:150000	YOL094C	YEL055C	-53.9866	-50.2151	110.701
15:150000	YOL094C	YDL213C	288.934	295.282	345.693
15:150000	YOL094C	YNL178W	-687.315	-675.488	-659.541
15:150000	YOL094C	YDR165W	756.4	761.292	873.95
15:150000	YOL094C	YMR229C	1044.48	1054.23	1096.5
15:150000	YOL094C	YIL018W	-249.741	-241.046	-200.672
15:150000	YOL094C	YGL040C	-394.301	-386.228	-289.275
15:150000	YOL094C	YDR060W	1074	1083.81	1175.86
15:150000	YOL094C	YPL118W	-362.904	-360.863	-356.876
15:150000	YOL094C	YJL014W	-919.789	-915.383	-902.983
15:150000	YOL094C	YPR110C	326.997	333.705	430.796
15:150000	YOL094C	YGR034W	-266.978	-256.815	-206.787
15:150000	YOL094C	YEL026W	432.947	438.3	530.677
15:150000	YOL094C	YKL004W	-249.605	-240.249	-163.575
15:150000	YOL094C	YOL041C	472.867	477.081	583.951
15:150000	YOL094C	YDR032C	-1063.21	-1059.48	-1047.23

15:150000	YOL094C	YER165W	-871.878	-859.892	-808.49
15:150000	YOL094C	YOL080C	574.14	580.645	651.412
15:150000	YOL094C	YOL081W	-551.706	-540.918	-546.882
15:150000	YOL094C	YAL036C	739.149	745.779	815.363
15:150000	YOL094C	YKL096W-A	-1684.22	-1680.11	-1624.64
15:150000	YOL094C	YNL175C	1196.71	1204.91	1266.28
15:150000	YOL094C	YDL130W	-647.13	-636.586	-613.906
15:150000	YOL094C	YOR004W	1008.44	1016.38	1048.95
15:150000	YOL094C	YMR118C	545.52	556.253	633.257
15:150000	YOL094C	YOR206W	1162.95	1172.08	1266.89
15:150000	YOL094C	YNR036C	-434.831	-434.498	-391.859
15:150000	YOL094C	YDL061C	-834.554	-822.544	-781.84
15:150000	YOL094C	YHL033C	-354.686	-345.212	-327.337
15:150000	YOL094C	YLR193C	-553.172	-550.248	-522.542
15:150000	YOL094C	YAL012W	-759.129	-755.449	-723.762
15:150000	YOL094C	YIL127C	661.265	671.303	740.201
15:150000	YOL094C	YDL083C	11.3409	21.9486	70.4064
15:150000	YOL094C	YLR002C	859.427	862.362	912.514
15:150000	YOL094C	YMR271C	507.963	511.365	691.223
15:150000	YOL094C	YLR448W	-168.339	-156.366	-109.576
15:150000	YOL094C	YHL034C	-1122.96	-1119.31	-1112.21
15:150000	YOL094C	YCL057C-A	-791.144	-783.02	-750.327
15:150000	YOL094C	YML024W	-910.575	-899.833	-851.368
15:150000	YOL094C	YNL209W	-194.313	-183.277	-112.917
15:150000	YOL094C	YDR303C	-536.964	-532.776	-527.652
15:150000	YOL094C	YOR167C	-617.087	-605.022	-550.511
15:150000	YOL094C	YOR310C	785.765	787.716	903.586
15:150000	YOL094C	YML042W	-127.719	-124.918	-9.70803
15:150000	YOL094C	YJL181W	-466.573	-464.106	-400.914
15:150000	YOL094C	YOL039W	-717.44	-705.532	-687.498
15:150000	YOL094C	YPL249C-A	-834.758	-825.802	-798.725
15:150000	YOL094C	YLR196W	1048.1	1054.92	1109.26
15:150000	YOL094C	YGR286C	-208.07	-201.531	-155.237
15:150000	YOL094C	YIL078W	-972.501	-966.408	-956.633
15:150000	YOL094C	YMR194W	-225.206	-216.045	-132.263
15:150000	YOL094C	YMR182W-A	51.4975	61.6582	400.378
15:150000	YOL094C	YOR354C	-336.799	-325.925	-270.245
15:150000	YOL094C	YHR020W	355.869	364.693	384.021
15:150000	YOL094C	YLR023C	-257.786	-248.573	-136.307
15:150000	YOL094C	YLR168C	93.1307	104.44	99.4457
15:150000	YOL094C	YPL012W	1167.96	1176.88	1252.14
15:150000	YOL094C	YDR025W	-68.0709	-56.2454	-9.38107
15:150000	YOL094C	YKL056C	-1414.05	-1410.27	-1354.23
15:150000	YOL094C	YNL284C	-238.297	-237.398	-190.561
15:150000	YOL094C	YGL078C	1309.41	1316.4	1407.53
15:150000	YOL094C	YOR107W	41.1149	44.7007	67.9331
15:150000	YOL094C	YML056C	636.383	648.059	712.248

15:150000	YOL094C	YGR128C	1118.49	1127.38	1188.82
15:150000	YOL094C	YOR207C	527.954	529.016	557.487
15:150000	YOL094C	YCR031C	-943.172	-931.507	-909.149
15:150000	YOL094C	YPL211W	844.499	851.04	940.476
15:150000	YOL094C	YER131W	248.873	257.51	325.956
15:150000	YOL094C	YJL191W	55.0181	65.6722	124.93
15:150000	YOL094C	YHR183W	-894.589	-884.576	-742.809
15:150000	YOL094C	YPL266W	539.807	545.547	648.099
15:150000	YOL094C	YHR068W	-731.745	-724.226	-658.509
15:150000	YOL094C	YDR321W	70.718	82.4693	118.956
15:150000	YOL094C	YMR038C	-807.125	-800.164	-761.859
15:150000	YOL094C	YLR325C	-238.969	-229.959	-188.67
15:150000	YOL094C	YGL077C	-442.92	-437.398	-433.351
15:150000	YOL094C	YMR056C	-407.008	-397.765	-332.253
15:150000	YOL094C	YDR500C	-238.899	-227.524	-189.933
15:150000	YOL094C	YLR301W	-922.685	-916.131	-921.826
15:150000	YOL094C	YDL051W	459.769	470.279	567.073
15:150000	YOL094C	YPL079W	-413.519	-403.708	-364.829
15:150000	YOL094C	YDL208W	400.555	405.522	554.587
15:150000	YOL094C	YHR007C	-808.147	-805.289	-771.572
15:150000	YOL094C	YDR161W	594.697	604.566	652.485
15:150000	YOL094C	YCR051W	-194.86	-184.235	-150.808
15:150000	YOL094C	YDR260C	-404.094	-393.139	-330.178
15:150000	YOL094C	YGR155W	-764.367	-755.501	-706.986
15:150000	YOL094C	YLR061W	-229.811	-219.308	-181.369
15:150000	YOL094C	YHR141C	-844.827	-833.502	-797.481
15:150000	YOL094C	YLR293C	-1257.99	-1252.01	-1203.22
15:150000	YOL094C	YGR220C	-159.363	-153.402	-119.167
15:150000	YOL094C	YMR308C	41.434	47.8554	174.17
15:150000	YOL094C	YDR494W	-80.658	-76.5748	34.3865
15:150000	YOL094C	YOR243C	184.31	192.015	287.831
15:150000	YOL094C	YER156C	-382.816	-374.311	-305.689
15:150000	YOL094C	YLR197W	767.324	772.984	848.224
15:150000	YOL094C	YLR333C	-505.258	-496.785	-466.227
15:150000	YOL094C	YPL271W	-816.952	-812.926	-803.494
15:150000	YOL094C	YHR045W	-820.335	-813.593	-784.125
15:150000	YOL094C	YJL190C	-157.921	-148.098	-112.123
15:150000	YOL094C	YER110C	445.099	456.553	504.97
15:150000	YOL094C	YBR189W	-345.204	-333.953	-307.189
15:150000	YOL094C	YPL160W	-473.918	-461.871	-389.126
15:150000	YOL094C	YNL302C	-471.974	-462.384	-396.887
15:150000	YOL094C	YHL011C	261.932	264.956	320.192
15:150000	YOL094C	YLR409C	943.381	951.532	1052.71
15:150000	YOL094C	YML126C	-972.796	-962.997	-954.323
15:150000	YOL094C	YJR077C	-747.34	-736.156	-739.234
15:150000	YOL094C	YOR335C	-578.253	-571.557	-568.167
15:150000	YOL094C	YDR132C	-401.034	-393.835	-388.641

15:150000	YOL094C	YPR132W	-991.574	-980.469	-934.315
15:150000	YOL094C	YDR256C	38.6143	43.8098	221.995
15:150000	YOL094C	YKL006W	-515.024	-504.752	-476.429
15:150000	YOL094C	YLR372W	-370.257	-369.574	-230.53
15:150000	YOL094C	YER074W	-445.345	-433.994	-406.237
15:150000	YOL094C	YFR031C-A	-214.837	-203.975	-167.554
15:150000	YOL094C	YOL113W	-150.701	-142.465	-96.66
15:150000	YOL094C	YHL033C	-439.961	-430.345	-403.13
15:150000	YOL094C	YDL082W	204.95	213.462	273.486
15:150000	YOL094C	YMR011W	543.13	547.207	605.761
15:150000	YOL094C	YML106W	-817.644	-812.781	-722.594
15:150000	YOL094C	YJR041C	528.543	531.354	576.046
15:150000	YOL094C	YOL095C	-275.428	-274.473	-219.271
15:150000	YOL094C	YNL247W	-271.558	-268.165	-239.505
15:150000	YOL094C	YOL103W	-945.196	-934.315	-888.115
15:150000	YOL094C	YOR276W	-750.007	-742.67	-700.859
15:150000	YOL094C	YPL040C	-70.9562	-62.3832	-3.86068
15:150000	YOL094C	YLR432W	-22.2488	-17.3774	59.0952
15:150000	YOL094C	YER056C-A	-262.462	-254.831	-213.876
15:150000	YOL094C	YLR449W	756.282	761.2	798.098
15:150000	YOL094C	YHR106W	-762.25	-756.108	-726.518
15:150000	YOL094C	YMR230W	-236.62	-224.922	-171.108
15:150000	YOL094C	YDL014W	595.454	599.668	705.523
15:150000	YOL094C	YDR023W	-959.71	-954.387	-911.651
15:150000	YOL094C	YGL147C	-363.386	-354.268	-310.15
15:150000	YOL094C	YOR286W	-327.374	-319.954	-319.898
15:150000	YOL094C	YNL115C	-318.21	-313.17	-289.348
15:150000	YOL094C	YOL056W	-557.223	-548.04	-513.223
15:150000	YOL094C	YNL302C	-860.147	-849.31	-823.574
15:150000	YOL094C	YER001W	-62.1806	-50.7005	-56.3477
15:150000	YOL094C	YNL255C	-142.992	-133.482	-76.0635
15:150000	YOL094C	YLR375W	-666.25	-663.214	-643.095
15:150000	YOL094C	YLL046C	99.1371	103.94	216.824
15:150000	YOL094C	YML073C	-333.473	-326.443	-291.218
15:150000	YOL094C	YLR228C	348.288	356.573	477.938
15:150000	YOL094C	YGL103W	-1286.33	-1285.12	-1200.02
15:150000	YOL094C	YKL052C	-817.045	-805.112	-798.52
15:150000	YOL094C	YMR312W	-314.064	-306.774	-255.524
15:150000	YOL094C	YJL010C	508.011	518.677	591.732
15:150000	YOL094C	YBR148W	29.0181	36.4785	85.0782
15:150000	YOL094C	YBL087C	-707.6	-695.67	-660.114
15:150000	YOL094C	YOL121C	26.2626	35.9228	84.0237
15:150000	YOL094C	YGL123W	-908.508	-899.808	-854.885
15:150000	YOL094C	YNL137C	-164.742	-155.691	-89.3264
15:150000	YOL094C	YOR312C	-680.371	-668.701	-628.866
15:150000	YOL094C	YMR174C	385.329	385.571	436.681
15:150000	YOL094C	YNL069C	-654.443	-642.61	-619.063

15:150000	YOL094C	YJR122W	-189.11	-180.429	-174.779
15:150000	YOL094C	YCR034W	-460.984	-459.629	-262.718
15:150000	YOL094C	YJL034W	-643.465	-631.699	-550.655
15:150000	YOL094C	YOL091W	330.139	341.779	638.032
15:150000	YOL094C	YHR010W	-527.856	-517.261	-480.13
15:150000	YOL094C	YGR077C	-848.44	-845.45	-791.152
15:150000	YOL094C	YGR102C	-257.976	-245.884	-42.2106
15:150000	YOL094C	YKL216W	932.734	941.503	953.334
15:150000	YOL094C	YGR123C	627.259	637.026	699.328
15:150000	YOL094C	YPL265W	-551.823	-541.614	-522.803
15:150000	YOL094C	YKL087C	-58.1483	-49.5605	84.9873
15:150000	YOL094C	YER049W	330.015	330.192	354.576
15:150000	YOL094C	YJL062W-A	-120.707	-116.965	-117.361
15:150000	YOL094C	YER074W	-644.085	-632.463	-597.25
15:150000	YOL094C	YJR043C	-525.105	-519.224	-519.373
15:150000	YOL094C	YGR118W	-1018.37	-1006.82	-996.549
15:150000	YOL094C	YOR096W	-493.373	-481.832	-440.045
15:150000	YOL094C	YLR406C	270.583	282.638	320.919
15:150000	YOL094C	YOR293W	-74.6228	-63.5053	-19.8575
15:150000	YOL094C	YDR091C	216.032	218.969	348.609
15:150000	YOL094C	YKR076W	-14.0614	-3.17299	85.6422
15:150000	YOL094C	YGR200C	557.099	560.981	632.801
15:150000	YOL094C	YHR064C	-735.981	-726.532	-673.862
15:150000	YOL094C	YMR297W	-1001.54	-998.849	-979.949
15:150000	YOL094C	YGL076C	-1156.51	-1151.59	-1114.42
15:150000	YOL094C	YHR022C	733.62	738.361	762.201
15:150000	YOL094C	YOR001W	216.076	227.315	286.674
15:150000	YOL094C	YMR286W	-65.4364	-57.0911	-45.9824
15:150000	YOL094C	YNR060W	573.229	577.691	578.022
15:150000	YOL094C	YFR032C-A	-570.742	-561.671	-512.107
15:150000	YOL094C	YDL211C	-413.667	-402.953	-320.83
15:150000	YOL094C	YPL156C	20.7484	30.3109	27.6411
15:150000	YOL094C	YER117W	-528.932	-516.764	-479.07
15:150000	YOL094C	YBR191W	-701.815	-690.922	-641.837
15:150000	YOL094C	YPR036W	-1086.23	-1078.63	-995.562
15:150000	YOL094C	YBR007C	-487.731	-487.271	-479.622
15:150000	YOL094C	YMR145C	115.843	127.706	126.101
15:150000	YOL094C	YBR025C	-746.2	-738.518	-669.191
15:150000	YOL094C	YLR441C	-431.913	-422.902	-375.608
15:150000	YOL094C	YGR285C	-1055.31	-1051.74	-1013
15:150000	YOL094C	YIL021W	-810.111	-808.302	-804.87
15:150000	YOL094C	YLL034C	834.274	842.467	918.783
15:150000	YOL094C	YHR019C	-779.54	-772.695	-778.593
15:150000	YOL094C	YDR041W	-70.9233	-70.4374	-1.82923
15:150000	YOL094C	YDR197W	-505.884	-504.249	-437.736
15:150000	YOL094C	YLL045C	-657.349	-645.951	-627.438
15:150000	YOL094C	YER025W	-386.355	-380.32	-288.317

15:150000	YOL094C	YOR315W	365.716	372.549	512.875
15:150000	YOL094C	YNL234W	-79.6378	-69.7815	92.7217
15:150000	YOL094C	YLR340W	-935.805	-923.701	-879.705
15:150000	YOL094C	YNL301C	-1025.53	-1014.38	-975.445
15:150000	YOL094C	YLL056C	-464.071	-452.927	-346.406
15:150000	YOL094C	YLR118C	-623.152	-618.648	-558.492
15:150000	YOL094C	YOL040C	-711.129	-699.352	-675.157
15:150000	YOL094C	YNL061W	883.292	888.349	951.088
15:150000	YOL094C	YBR181C	-1015.26	-1006.49	-958.933
15:150000	YOL094C	YKL014C	857.494	867.514	971.746
15:150000	YOL094C	YDL081C	-775.748	-766.047	-699.642
15:150000	YOL094C	YJR101W	-108.204	-99.6848	-71.7577
15:150000	YOL094C	YDR324C	814.182	822.816	914.531
15:150000	YOL094C	YOR247W	-938.56	-935.538	-895.698
15:150000	YOL094C	YGR152C	-442.646	-430.814	-393.451
15:150000	YOL094C	YHR203C	-1335.35	-1329.29	-1270.04
15:150000	YOL094C	YMR143W	-828.498	-822.973	-770.129
15:150000	YOL094C	YKL073W	-604.025	-594.347	-476.672
15:150000	YOL094C	YBR048W	-770.444	-758.278	-720.278
15:150000	YOL094C	YGL135W	-1265.81	-1257.64	-1191
15:150000	YOL094C	YBR071W	-313.269	-306.478	-241.187
15:150000	YOL094C	YER096W	15.0425	15.0538	90.7126
15:150000	YOL094C	YFL022C	-781.392	-771.224	-679.464
15:150000	YOL094C	YBR147W	732.575	741.561	785.496
15:150000	YOL094C	YOR134W	464.982	471.683	553.081
15:150000	YOL094C	YGR007W	-965.482	-957.613	-948.352
15:150000	YOL094C	YJL177W	-8.70987	1.70935	44.4116
15:150000	YOL094C	YLR083C	-464.44	-457.182	-399.834
15:150000	YOL094C	YJR094W-A	-87.5383	-75.516	-17.0769
15:150000	YOL094C	YGR207C	-558.895	-554.429	-549.275
15:150000	YOL094C	YJR090C	-778.891	-776.583	-667.75
15:150000	YOL094C	YDL075W	-629.658	-618.155	-582.816
15:150000	YOL094C	YJL112W	-206.918	-205.792	-180.499
15:150000	YOL094C	YMR310C	157.298	159.295	229.853
15:150000	YOL094C	YHL001W	-897.394	-885.275	-838.961
15:150000	YOL094C	YDR482C	-500.799	-491.567	-343.48
15:150000	YOL094C	YHR021C	-501.09	-490.331	-442.722
15:150000	YOL094C	YMR217W	384.422	393.526	448.111
15:150000	YOL094C	YBR191W	-257.775	-246.573	-203.248
15:150000	YOL094C	YOR234C	-155.501	-145.781	-99.0133
15:150000	YOL094C	YDR298C	-551.087	-550.941	-546.911
15:150000	YOL094C	YEL071W	-513.103	-503.999	-508.003
15:150000	YOL094C	YBL092W	-887.608	-877.223	-845.437
15:150000	YOL094C	YGR094W	-864.117	-855.715	-805.634
15:150000	YOL094C	YFR006W	-913.429	-905.647	-908.059
15:150000	YOL094C	YPR127W	-453.348	-451.056	-433.262
15:150000	YOL094C	YJL136C	-832.383	-822.132	-791.493

15:150000	YOL094C	YIL133C	-89.8749	-77.7757	-32.8919
15:150000	YOL094C	YFR031C-A	-887.044	-874.98	-850.81
15:150000	YOL094C	YOR293W	-110.788	-99.1584	-59.543
15:150000	YOL094C	YGL030W	-1101.85	-1091.25	-1054.49
15:150000	YOL088C	YDR447C	-618.402	-607.072	-605.792
15:150000	YOL088C	YDR447C	-218.717	-212.494	-211.799
15:150000	YOL088C	YKL096W	-471.313	-463.677	-463.554
15:150000	YOL088C	YNR055C	-644.351	-634.385	-640.456
15:150000	YOL088C	YHR197W	1125.76	1128.5	1134.66
15:150000	YOL088C	YDL061C	-792.955	-789.883	-780.45
15:150000	YOL088C	YOL077W-A	-320.314	-311.111	-314.782
15:150000	YOL088C	YDL191W	-1111.72	-1106.68	-1105.82
15:150000	YOL088C	YBR084C-A	-340.508	-333.901	-321.802
15:150000	YOL088C	YDR450W	-835.908	-828.591	-831.339
15:150000	YOL088C	YLR029C	-1008.35	-999.499	-990.566
15:150000	YOL088C	YMR128W	942.994	947.181	946.551
15:150000	YOL088C	YNL055C	-1238.8	-1228.24	-1208.61
15:150000	YOL088C	YPL131W	-776.103	-772.674	-763.307
15:150000	YOL088C	YOL036W	-697.251	-688.358	-694.297
15:150000	YOL088C	YJR123W	-1022.43	-1014.47	-1001.85
15:150000	YOL088C	YGR027C	-1041.71	-1033.43	-1035.58
15:150000	YOL088C	YLR185W	-363.626	-362.363	-355.676
15:150000	YOL088C	YML093W	1011.27	1014.64	1018.58
15:150000	YOL088C	YLL028W	-267.215	-258.48	-258.263
15:150000	YOL088C	YCR073C	797.871	809.686	814.881
15:150000	YOL088C	YGL031C	-923.329	-911.092	-903.002
15:150000	YOL088C	YBL072C	-1326.76	-1314.44	-1308.05
15:150000	YOL088C	YBL027W	-413.115	-412.866	-407.557
15:150000	YOL088C	YER102W	-627.694	-625.01	-618.147
15:150000	YOL088C	YGL189C	-1102.2	-1095.88	-1095.06
15:150000	YOL088C	YKR057W	-233.287	-227.702	-230.943
15:150000	YOL088C	YPL189W	-185.1	-172.904	-174.071
15:150000	YOL088C	YGR148C	-1021.88	-1017.95	-1014.41
15:150000	YOL088C	YOR341W	1180.71	1188.48	1183.9
15:150000	YOL088C	YJL109C	1005.95	1012.56	1022.91
15:150000	YOL088C	YMR142C	-754.034	-752.575	-744.484
15:150000	YOL088C	YDL229W	-443.87	-438.546	-432.774
15:150000	YOL088C	YDR064W	-1032.09	-1028.38	-1021.72
15:150000	YOL088C	YNR066C	683.468	690.003	684.184
15:150000	YOL088C	YDR337W	129.78	133.963	130.024
15:150000	YOL088C	YBR023C	-286.684	-280.146	-275.493
15:150000	YOL088C	YDR349C	-286.382	-279.152	-269.795
15:150000	YOL088C	YER006W	994.158	998.82	995.254
15:150000	YOL088C	YCL059C	814.188	820.86	815.555
15:150000	YOL088C	YGR166W	454.162	465.262	469.085
15:150000	YOL088C	YDR502C	-500.53	-498.38	-489.748
15:150000	YOL088C	YPR010C	1149.42	1157.61	1155.35

15:150000	YOL088C	YGL189C	-960.185	-949.919	-949.292
15:150000	YOL088C	YPR043W	-1158.8	-1151.68	-1154.45
15:150000	YOL088C	YPL132W	17.0257	29.0351	30.4913
15:150000	YOL088C	YOL104C	-137.139	-133.24	-131.389
15:150000	YOL088C	YOL127W	-1124.81	-1113.76	-1108.59
15:150000	YOL088C	YKL161C	824.164	826.606	826.778
15:150000	YOL088C	YPR191W	-72.9336	-71.5702	-71.5541
15:150000	YOL088C	YEL054C	342.342	345.54	344.55
15:150000	YOL088C	YEL047C	-887.234	-878.728	-872.884
15:150000	YOL088C	YFL017C	-743.12	-739.494	-739.142
15:150000	YOL088C	YML026C	-261.294	-254.261	-251.067
15:150000	YOL088C	YMR116C	-1098.62	-1094.28	-1081.69
15:150000	YOL088C	YGR031W	-270.635	-262.584	-253.806
15:150000	YOL088C	YKL084W	-288.528	-287.027	-279.215
15:150000	YOL088C	YPL043W	1186.06	1190.43	1191.36
15:150000	YOL088C	YLR150W	-1252.2	-1241.46	-1222.7
15:150000	YOL088C	YDL235C	-538.346	-533.963	-517.668
15:150000	YOL088C	YGR033C	-599.568	-598.121	-582.254
15:150000	YOL088C	YJL189W	-551.677	-544.651	-540.715
15:150000	YOL088C	YFL054C	397.608	397.983	407.646
15:150000	YOL088C	YLR388W	-64.453	-63.3693	-63.587
15:150000	YOL088C	YPL048W	-1207.23	-1203.1	-1205.86
15:150000	YOL088C	YML063W	-673.419	-662.739	-666.797
15:150000	YOL088C	YJR054W	-467.525	-465.305	-462.178
15:150000	YOL088C	YEL058W	-675.214	-669.74	-658.887
15:150000	YOL088C	YLR175W	857.447	863.206	865.148
15:150000	YOL088C	YKR094C	-489.01	-486.381	-480.234
15:150000	YOL088C	YGR001C	-871.243	-871.144	-859.353
15:150000	YOL088C	YHR089C	785.686	791.031	788.722
15:150000	YOL088C	YOL152W	519.056	523.256	520.079
15:150000	YOL088C	YBL072C	-809.273	-801.381	-796.77
15:150000	YOL088C	YDR012W	-277.245	-270.568	-253.99
15:150000	YOL088C	YHR156C	-191.278	-181.656	-181.856
15:150000	YOL088C	YOR312C	-554.66	-547.194	-550.117
15:150000	YOL088C	YDR165W	922.366	928.5	929.367
15:150000	YOL088C	YMR229C	1159.97	1169.18	1167.85
15:150000	YOL088C	YGL040C	-332.316	-323.348	-319.724
15:150000	YOL088C	YDR060W	1233.85	1238.38	1249.76
15:150000	YOL088C	YPL118W	-383.685	-380.522	-379.706
15:150000	YOL088C	YGR034W	-240.861	-234.116	-234.3
15:150000	YOL088C	YFR044C	-1110.09	-1102.88	-1106.88
15:150000	YOL088C	YIL117C	102.379	110.325	106.522
15:150000	YOL088C	YER169W	193.085	197.543	200.415
15:150000	YOL088C	YOL081W	-590.974	-580.946	-586.595
15:150000	YOL088C	YKL096W-A	-1740.71	-1731.35	-1736.03
15:150000	YOL088C	YNL175C	1333.37	1340.01	1337.68
15:150000	YOL088C	YDR046C	606.608	612.777	611.738

15:150000	YOL088C	YOR206W	1339.31	1344.95	1350.07
15:150000	YOL088C	YDL061C	-847.051	-845.121	-844.634
15:150000	YOL088C	YDL083C	59.8395	59.8653	65.7681
15:150000	YOL088C	YMR012W	-681.353	-671.71	-653.641
15:150000	YOL088C	YMR271C	737.463	742.203	740.699
15:150000	YOL088C	YLR448W	-128.833	-127.993	-122.643
15:150000	YOL088C	YIR035C	-646.759	-640.285	-635.097
15:150000	YOL088C	YCL057C-A	-820.846	-813.921	-807.59
15:150000	YOL088C	YML024W	-931.709	-922.127	-919.339
15:150000	YOL088C	YNL209W	-139.056	-134.245	-131.384
15:150000	YOL088C	YOR167C	-590.693	-586.174	-588.398
15:150000	YOL088C	YOR310C	959.22	963.133	961.854
15:150000	YOL088C	YJL181W	-445.516	-441.73	-434.751
15:150000	YOL088C	YOL039W	-760.394	-754.457	-755.602
15:150000	YOL088C	YNR033W	-393.317	-390.749	-383.867
15:150000	YOL088C	YPL249C-A	-876.689	-875.899	-871.184
15:150000	YOL088C	YMR194W	-143.916	-139.742	-143.358
15:150000	YOL088C	YOR354C	-310.128	-297.923	-293.386
15:150000	YOL088C	YLR023C	-184.282	-178.03	-129.09
15:150000	YOL088C	YER045C	-243.79	-236.082	-240.602
15:150000	YOL088C	YLR168C	114.706	122.831	116.682
15:150000	YOL088C	YBR104W	542.055	543.553	543.843
15:150000	YOL088C	YPL012W	1326.91	1330.27	1336.31
15:150000	YOL088C	YLR194C	-39.4251	-34.0234	-38.3544
15:150000	YOL088C	YNL284C	-208.944	-205.418	-195.986
15:150000	YOL088C	YGL078C	1483.76	1492.81	1498.57
15:150000	YOL088C	YOR107W	57.0702	61.4852	57.5561
15:150000	YOL088C	YHR092C	967.604	977.942	979.276
15:150000	YOL088C	YCR031C	-998.533	-991.401	-981.964
15:150000	YOL088C	YDR351W	30.4286	41.4836	46.3692
15:150000	YOL088C	YER131W	329.403	333.957	340.903
15:150000	YOL088C	YFL036W	-185.722	-178.292	-162.413
15:150000	YOL088C	YDR500C	-216.619	-215.821	-212.85
15:150000	YOL088C	YDL208W	590.81	591.27	591.173
15:150000	YOL088C	YDR047W	-399.013	-390.458	-389.166
15:150000	YOL088C	YHR007C	-816.786	-814.076	-809.461
15:150000	YOL088C	YDR260C	-370.465	-359.657	-361.621
15:150000	YOL088C	YHR141C	-874.247	-864.003	-861.978
15:150000	YOL088C	YGR220C	-135.497	-126.192	-127.875
15:150000	YOL088C	YDR494W	10.3656	21.217	35.2901
15:150000	YOL088C	YOL119C	218.123	226.395	226.305
15:150000	YOL088C	YPL271W	-867.393	-864.084	-860.118
15:150000	YOL088C	YJL190C	-125.9	-119.536	-120.191
15:150000	YOL088C	YBR189W	-348.067	-346.812	-337.773
15:150000	YOL088C	YNL302C	-440.578	-428.977	-424.965
15:150000	YOL088C	YLR409C	1121.21	1121.89	1128
15:150000	YOL088C	YJR077C	-807.089	-795.806	-801.81

15:150000	YOL088C	YDR132C	-416.77	-407.292	-413.267
15:150000	YOL088C	YPR132W	-1017.05	-1006.11	-1011.02
15:150000	YOL088C	YPL053C	-823.913	-816.189	-814.489
15:150000	YOL088C	YKL006W	-528.358	-522.324	-517.643
15:150000	YOL088C	YOL113W	-117.186	-115.243	-106.395
15:150000	YOL088C	YMR011W	651.277	658.057	652.108
15:150000	YOL088C	YJR005W	-560.67	-557.81	-556.087
15:150000	YOL088C	YPL040C	-5.3329	5.1461	0.225952
15:150000	YOL088C	YGL115W	-964.221	-954.755	-958.744
15:150000	YOL088C	YGR189C	-283.695	-277.771	-275.512
15:150000	YOL088C	YMR230W	-191.171	-180.957	-183.925
15:150000	YOL088C	YDL014W	752.223	757.736	753.551
15:150000	YOL088C	YGL147C	-346.434	-334.666	-340.224
15:150000	YOL088C	YOR286W	-347.288	-340.176	-346.038
15:150000	YOL088C	YOR311C	-130.699	-130.486	-126.874
15:150000	YOL088C	YNL302C	-893.325	-890.763	-884.889
15:150000	YOL088C	YER001W	-66.6463	-57.4149	-56.3808
15:150000	YOL088C	YPL052W	469.675	473.147	479.793
15:150000	YOL088C	YKL155C	18.5983	26.5031	23.0268
15:150000	YOL088C	YLL046C	215.36	224.892	218.757
15:150000	YOL088C	YLR228C	510.409	513.345	514.551
15:150000	YOL088C	YKL052C	-858.952	-851.557	-857.676
15:150000	YOL088C	YBL087C	-725.17	-723.68	-716.516
15:150000	YOL088C	YOL121C	85.9986	90.8937	87.6547
15:150000	YOL088C	YGL123W	-952.178	-942.76	-932.091
15:150000	YOL088C	YNL137C	-106.573	-94.3528	-96.2814
15:150000	YOL088C	YOR312C	-678.609	-669.201	-667.525
15:150000	YOL088C	YNL069C	-690.205	-685.975	-677.232
15:150000	YOL088C	YJR122W	-198.262	-189.505	-189.402
15:150000	YOL088C	YER130C	-310.691	-302.168	-298.098
15:150000	YOL088C	YHR010W	-527	-519.755	-520.822
15:150000	YOL088C	YGR123C	741.948	748.79	744.952
15:150000	YOL088C	YMR295C	-1291.59	-1287.47	-1287.41
15:150000	YOL088C	YPL265W	-563.812	-563.69	-562.811
15:150000	YOL088C	YKL087C	71.8177	82.0664	86.2916
15:150000	YOL088C	YMR072W	-1063.59	-1056.69	-1025.01
15:150000	YOL088C	YER074W	-652.465	-643.487	-640.726
15:150000	YOL088C	YGR118W	-1080.49	-1071.39	-1076.68
15:150000	YOL088C	YOR096W	-483.917	-475.4	-477.312
15:150000	YOL088C	YLR406C	336.948	340.146	339.47
15:150000	YOL088C	YOR293W	-31.055	-23.0827	-25.0183
15:150000	YOL088C	YDR091C	372.161	374.832	374.236
15:150000	YOL088C	YGL076C	-1211.55	-1209.07	-1198.41
15:150000	YOL088C	YNL133C	-430.867	-426.849	-415.081
15:150000	YOL088C	YOR001W	309.863	309.867	312.171
15:150000	YOL088C	YMR286W	-46.2516	-37.1938	-43.357
15:150000	YOL088C	YNR060W	617.999	622.183	621.219

15:150000	YOL088C	YFR032C-A	-566.185	-555.868	-553.94
15:150000	YOL088C	YPL156C	28.804	37.3553	31.9516
15:150000	YOL088C	YHR111W	-466.643	-462.769	-445.032
15:150000	YOL088C	YER117W	-537.785	-528.825	-529.739
15:150000	YOL088C	YBR191W	-707.049	-703.88	-693.322
15:150000	YOL088C	YPL089C	242.045	253.408	251.071
15:150000	YOL088C	YPR036W	-1072.59	-1064.01	-1068.94
15:150000	YOL088C	YMR145C	124.299	135.094	131.874
15:150000	YOL088C	YBR025C	-720.505	-710.521	-706.275
15:150000	YOL088C	YLR441C	-400.607	-397.216	-397.818
15:150000	YOL088C	YDR041W	-20.2096	-12.9901	9.69386
15:150000	YOL088C	YBR031W	-1393.51	-1391.4	-1371.91
15:150000	YOL088C	YLL045C	-698.918	-694.75	-688.604
15:150000	YOL088C	YOR315W	537.065	545.804	544.973
15:150000	YOL088C	YLR340W	-969.407	-963.929	-957.739
15:150000	YOL088C	YOL094C	-358.804	-353.18	-346.41
15:150000	YOL088C	YNL061W	1012	1014.51	1012.1
15:150000	YOL088C	YBR181C	-1048.29	-1037.88	-1035.6
15:150000	YOL088C	YKL014C	1027.57	1031.19	1038.4
15:150000	YOL088C	YDL081C	-766.933	-758.354	-760.9
15:150000	YOL088C	YBR185C	140.39	143.178	146.087
15:150000	YOL088C	YGL025C	-488.244	-485.988	-485.248
15:150000	YOL088C	YAL041W	-770.875	-765.884	-770.588
15:150000	YOL088C	YHR203C	-1400.08	-1388.38	-1370.48
15:150000	YOL088C	YMR143W	-819.294	-809.863	-809.739
15:150000	YOL088C	YBL061C	315.978	320.547	334.082
15:150000	YOL088C	YKL073W	-514.509	-503.669	-509.707
15:150000	YOL088C	YBR048W	-802.687	-796.246	-782.958
15:150000	YOL088C	YGL135W	-1296.35	-1286.79	-1288.66
15:150000	YOL088C	YGR207C	-601.274	-596.108	-595.265
15:150000	YOL088C	YJR090C	-720.499	-708.246	-711.405
15:150000	YOL088C	YDL075W	-652.873	-650.958	-641.419
15:150000	YOL088C	YJL112W	-196.485	-184.628	-190.622
15:150000	YOL088C	YHL001W	-912.814	-909.318	-901.451
15:150000	YOL088C	YDR482C	-378.061	-373.484	-366.222
15:150000	YOL088C	YHR021C	-496.716	-494.545	-490.086
15:150000	YOL088C	YBR191W	-243.302	-234.495	-223.493
15:150000	YOL088C	YNR065C	259.01	262.08	260.507
15:150000	YOL088C	YGR060W	-1085.89	-1084.78	-1079.64
15:150000	YOL088C	YLR121C	515.771	520.883	531.411
15:150000	YOL088C	YKR013W	-163.409	-159.003	-156.745
15:150000	YOL088C	YDR298C	-607.09	-600.773	-596.331
15:150000	YOL088C	YEL071W	-551.213	-549.513	-535.868
15:150000	YOL088C	YBL092W	-932.928	-925.77	-916.439
15:150000	YOL088C	YFR006W	-960.746	-952.895	-955.07
15:150000	YOL088C	YIL133C	-39.1958	-38.9685	-38.1856
15:150000	YOL088C	YFR031C-A	-951.224	-946.33	-929.597

15:150000	YOL088C	YOR293W	-72.175	-63.3277	-64.9099
15:150000	YOL088C	YGL030W	-1162.75	-1155.19	-1144.29
15:150000	YOL100W	YML035C	-420.955	-418.506	-391.637
15:150000	YOL100W	YDR447C	-672.058	-660.366	-589.136
15:150000	YOL100W	YDR144C	79.0133	84.1623	166.47
15:150000	YOL100W	YJL185C	90.9535	95.8356	175.545
15:150000	YOL100W	YDR447C	-257.757	-247.062	-210.134
15:150000	YOL100W	YNR055C	-697.649	-690.43	-620.551
15:150000	YOL100W	YHR197W	1046.64	1057.98	1117.52
15:150000	YOL100W	YOR187W	-396.91	-395.932	-386.89
15:150000	YOL100W	YGL121C	517.297	526.674	606.613
15:150000	YOL100W	YKL192C	-859.201	-851.829	-853.13
15:150000	YOL100W	YDL061C	-834.325	-825.035	-769.154
15:150000	YOL100W	YPR117W	-446.466	-443.097	-446.06
15:150000	YOL100W	YHR128W	-387.001	-377.286	-365.407
15:150000	YOL100W	YOL077W-A	-313.324	-302.803	-302.172
15:150000	YOL100W	YDL191W	-1141.39	-1130.96	-1099.32
15:150000	YOL100W	YBR084C-A	-373.868	-364.27	-311.309
15:150000	YOL100W	YAL003W	-735.238	-724.399	-680.279
15:150000	YOL100W	YDR450W	-901.14	-892.327	-810.652
15:150000	YOL100W	YLR029C	-1017.48	-1006.75	-960.439
15:150000	YOL100W	YMR128W	828.021	840.242	927.789
15:150000	YOL100W	YIL052C	-498.06	-494.994	-448.284
15:150000	YOL100W	YMR131C	858.846	870.669	978.233
15:150000	YOL100W	YNL055C	-1184.08	-1173.43	-1178.96
15:150000	YOL100W	YPL131W	-773.667	-765.605	-746.864
15:150000	YOL100W	YDL085W	652.741	658.052	706.978
15:150000	YOL100W	YML124C	-1228.34	-1226.6	-1228.15
15:150000	YOL100W	YHR065C	706.007	716.707	766.084
15:150000	YOL100W	YDR454C	-1187.3	-1179.95	-1127.42
15:150000	YOL100W	YHL001W	-267.788	-258.486	-171.628
15:150000	YOL100W	YJR123W	-1035.13	-1028.06	-984.32
15:150000	YOL100W	YGR027C	-1070.01	-1059.44	-1009.24
15:150000	YOL100W	YGL253W	-597.963	-593.112	-543.355
15:150000	YOL100W	YDL229W	-54.8661	-49.9524	3.87194
15:150000	YOL100W	YKL180W	-492.451	-488.348	-440.118
15:150000	YOL100W	YMR301C	-193.198	-184.716	-131.675
15:150000	YOL100W	YLR185W	-392.98	-385.692	-347.448
15:150000	YOL100W	YML093W	906.371	918.646	1002.53
15:150000	YOL100W	YLL028W	-317.804	-306.887	-247.687
15:150000	YOL100W	YHR005C-A	-526.421	-517.814	-518.778
15:150000	YOL100W	YKL081W	-742.467	-733.19	-644.946
15:150000	YOL100W	YGL031C	-927.56	-915.322	-880.265
15:150000	YOL100W	YGL013C	-690.566	-682.271	-576.48
15:150000	YOL100W	YBL072C	-1374.09	-1364.6	-1294.44
15:150000	YOL100W	YBL027W	-470.179	-467.728	-401.323
15:150000	YOL100W	YER102W	-662.992	-654.145	-616.899

15:150000	YOL100W	YGL189C	-1152.82	-1144.37	-1068.64
15:150000	YOL100W	YKR057W	-283.605	-273.46	-237.315
15:150000	YOL100W	YNR043W	-962.081	-958.465	-960.213
15:150000	YOL100W	YGR085C	-135.675	-124.452	-61.8137
15:150000	YOL100W	YPL189W	-193.274	-181.492	-168.687
15:150000	YOL100W	YGR148C	-1037.33	-1030.54	-999.511
15:150000	YOL100W	YGR214W	-266.675	-261.301	-224.512
15:150000	YOL100W	YDR345C	-140.117	-134.315	-123.354
15:150000	YOL100W	YAL042W	-1038.19	-1034.21	-1032.7
15:150000	YOL100W	YER007C-A	-850.28	-839.793	-781
15:150000	YOL100W	YOL061W	-331.041	-323.568	-290.006
15:150000	YOL100W	YBL068W	369.109	372.169	411.922
15:150000	YOL100W	YPL272C	272.844	282.137	309.698
15:150000	YOL100W	YOL097C	-413.822	-406.313	-338.235
15:150000	YOL100W	YOR341W	1016.13	1026.22	1158.16
15:150000	YOL100W	YJL109C	852.797	863.043	1002.92
15:150000	YOL100W	YMR142C	-771.827	-768.644	-734.199
15:150000	YOL100W	YDL229W	-490.524	-485.691	-437.728
15:150000	YOL100W	YKL187C	2.12612	7.14625	8.9722
15:150000	YOL100W	YLR075W	-1742.36	-1734.1	-1682.9
15:150000	YOL100W	YDR064W	-1005.47	-996.174	-989.681
15:150000	YOL100W	YPR163C	76.0123	88.2566	178.642
15:150000	YOL100W	YNL073W	-335.664	-330.814	-267.932
15:150000	YOL100W	YDL031W	328.224	338.796	378.642
15:150000	YOL100W	YDR337W	115.853	120.032	127.013
15:150000	YOL100W	YGL111W	474.275	486.058	559.884
15:150000	YOL100W	YLR249W	-409.335	-398.849	-355.183
15:150000	YOL100W	YMR241W	-637.113	-631.23	-586.586
15:150000	YOL100W	YER006W	878.008	888.841	982.487
15:150000	YOL100W	YCL059C	676.283	684.154	805.381
15:150000	YOL100W	YLR267W	748.533	757.089	799.354
15:150000	YOL100W	YFR039C	-649.56	-642.649	-646.362
15:150000	YOL100W	YDR502C	-524.033	-518.167	-481.632
15:150000	YOL100W	YPR010C	991.55	1001.37	1133.97
15:150000	YOL100W	YGL189C	-1010.3	-998.017	-925.727
15:150000	YOL100W	YIL069C	202.032	210.074	265.649
15:150000	YOL100W	YPR043W	-1149.49	-1138.76	-1123.03
15:150000	YOL100W	YPL132W	-79.6728	-71.7811	23.986
15:150000	YOL100W	YOL127W	-1141.6	-1130.06	-1086.75
15:150000	YOL100W	YLR172C	53.4074	54.5866	72.041
15:150000	YOL100W	YOL022C	217.251	222.23	259.976
15:150000	YOL100W	YEL054C	238.233	246.068	333.011
15:150000	YOL100W	YEL047C	-952.745	-941.017	-848.069
15:150000	YOL100W	YML026C	-291.821	-283.994	-252.505
15:150000	YOL100W	YMR242C	-429.074	-425.067	-374.566
15:150000	YOL100W	YMR116C	-1103.23	-1095.7	-1059.56
15:150000	YOL100W	YGR031W	-493.873	-486.691	-246.679

15:150000	YOL100W	YPL043W	1048.62	1060.57	1172.73
15:150000	YOL100W	YLR150W	-1291.24	-1279.35	-1196.01
15:150000	YOL100W	YDR418W	-709.562	-703.179	-649.529
15:150000	YOL100W	YGR033C	-735.622	-724.567	-580.528
15:150000	YOL100W	YHR030C	-241.844	-241.292	-233.284
15:150000	YOL100W	YJL189W	-585.377	-574.19	-526.721
15:150000	YOL100W	YIR037W	-971.677	-962.587	-896.582
15:150000	YOL100W	YLR388W	-123.14	-116.564	-71.3336
15:150000	YOL100W	YGR264C	-84.2224	-72.7572	-16.7956
15:150000	YOL100W	YPL048W	-1265.03	-1253.82	-1177.16
15:150000	YOL100W	YML063W	-744.236	-733.823	-665.698
15:150000	YOL100W	YNL044W	-878.947	-870.166	-869.629
15:150000	YOL100W	YLR388W	-212.161	-208.648	-168.65
15:150000	YOL100W	YLR175W	699.432	710.684	849.88
15:150000	YOL100W	YKR094C	-538.008	-532.137	-476.211
15:150000	YOL100W	YGL065C	-812.82	-803.635	-640.554
15:150000	YOL100W	YEL050C	-73.8374	-66.6689	-71.1255
15:150000	YOL100W	YGR001C	-846	-842.119	-841.329
15:150000	YOL100W	YLR344W	-190.421	-180.539	-138.995
15:150000	YOL100W	YHR089C	625.979	636.694	773.966
15:150000	YOL100W	YLR048W	-76.7182	-73.5731	-21.4069
15:150000	YOL100W	YBL072C	-822.604	-814.263	-775.883
15:150000	YOL100W	YDR012W	-265.227	-254.213	-241.571
15:150000	YOL100W	YHR156C	-259.583	-258.728	-179.266
15:150000	YOL100W	YOR312C	-589.723	-580.946	-539.292
15:150000	YOL100W	YEL055C	15.7757	27.2579	117.555
15:150000	YOL100W	YDL213C	279.392	289.047	362.248
15:150000	YOL100W	YNL178W	-739.102	-731.97	-714.328
15:150000	YOL100W	YDR430C	18.861	21.432	97.7552
15:150000	YOL100W	YDR165W	743.886	752.469	913.494
15:150000	YOL100W	YMR229C	1015.55	1022.76	1145.08
15:150000	YOL100W	YIL018W	-276.866	-274.322	-224.314
15:150000	YOL100W	YGL040C	-397.32	-386.718	-318.219
15:150000	YOL100W	YDR060W	1072.62	1084.54	1228.16
15:150000	YOL100W	YPL118W	-381.245	-374.941	-378.578
15:150000	YOL100W	YDR341C	-404.846	-399.362	-402.673
15:150000	YOL100W	YJL014W	-943.604	-940.291	-943.38
15:150000	YOL100W	YPR110C	332.042	344.224	454.964
15:150000	YOL100W	YGR034W	-300.924	-298.477	-239.564
15:150000	YOL100W	YEL026W	421.467	433.73	553.973
15:150000	YOL100W	YKL004W	-233.616	-227.143	-165.452
15:150000	YOL100W	YOL041C	495.92	506.925	611.858
15:150000	YOL100W	YDR032C	-1101.01	-1099.29	-1088.72
15:150000	YOL100W	YER165W	-915.762	-907.898	-861.753
15:150000	YOL100W	YER169W	-93.842	-90.4226	201.714
15:150000	YOL100W	YPL183W-A	-26.0575	-22.445	-22.2718
15:150000	YOL100W	YOL080C	598.147	609.533	683.482

15:150000	YOL100W	YOL081W	-579.118	-570.182	-576.172
15:150000	YOL100W	YAL036C	770.188	781.737	862.539
15:150000	YOL100W	YKL096W-A	-1778.64	-1776.4	-1698.44
15:150000	YOL100W	YNL175C	1179.6	1190	1318.67
15:150000	YOL100W	YDL130W	-692.982	-687.229	-656.003
15:150000	YOL100W	YOR004W	1027.8	1038.98	1105.21
15:150000	YOL100W	YPL207W	288.389	292.368	359.426
15:150000	YOL100W	YMR118C	548.438	559.975	646.071
15:150000	YOL100W	YOR206W	1187.72	1199.16	1328.18
15:150000	YOL100W	YNR036C	-435.296	-429.614	-411.489
15:150000	YOL100W	YDL061C	-883.482	-875.724	-838.46
15:150000	YOL100W	YHL033C	-391.674	-388.408	-353.382
15:150000	YOL100W	YLR193C	-584.861	-582.951	-551.673
15:150000	YOL100W	YMR016C	-234.021	-233.706	-220.205
15:150000	YOL100W	YAL012W	-767.328	-757.279	-748.719
15:150000	YOL100W	YIL127C	695.224	706.946	773.89
15:150000	YOL100W	YDL083C	-0.832222	3.64855	55.5077
15:150000	YOL100W	YLR002C	884.478	893.113	954.676
15:150000	YOL100W	YMR012W	-775.575	-768.325	-644.165
15:150000	YOL100W	YMR271C	497.758	504.745	723.767
15:150000	YOL100W	YLR448W	-200.581	-192.18	-130.683
15:150000	YOL100W	YHL034C	-1184.23	-1177.91	-1158.28
15:150000	YOL100W	YCL057C-A	-813.822	-807.331	-785.848
15:150000	YOL100W	YML024W	-961.172	-949.612	-904.713
15:150000	YOL100W	YNL209W	-218.424	-208.941	-132.515
15:150000	YOL100W	YDR303C	-574.233	-573.166	-568.8
15:150000	YOL100W	YOR167C	-641.615	-632.977	-579.974
15:150000	YOL100W	YOR310C	780.404	791.623	943.473
15:150000	YOL100W	YML042W	-91.7548	-85.8165	-39.5174
15:150000	YOL100W	YBL035C	-346.599	-343.974	-342.012
15:150000	YOL100W	YJL181W	-527.684	-522.382	-430.224
15:150000	YOL100W	YOL039W	-757.268	-747.566	-735.771
15:150000	YOL100W	YNR033W	-418.351	-416.933	-383.129
15:150000	YOL100W	YPL249C-A	-891.286	-890.575	-858.992
15:150000	YOL100W	YLR196W	1113	1122.95	1168.59
15:150000	YOL100W	YGR286C	-163.439	-158.664	-148.919
15:150000	YOL100W	YML026C	-1156.02	-1153.02	-1080.07
15:150000	YOL100W	YIL078W	-1006.25	-997.001	-984.537
15:150000	YOL100W	YMR194W	-253.338	-244.37	-149.679
15:150000	YOL100W	YMR182W-A	94.9682	106.862	429.78
15:150000	YOL100W	YOR354C	-336.371	-324.507	-288.576
15:150000	YOL100W	YHR020W	347.027	356.371	397.271
15:150000	YOL100W	YLR023C	-250.145	-238.727	-119.763
15:150000	YOL100W	YLR168C	92.449	100.93	99.4757
15:150000	YOL100W	YPL012W	1208.68	1220.49	1316.37
15:150000	YOL100W	YDR025W	-87.1147	-80.8594	-21.5544
15:150000	YOL100W	YKL056C	-1470.28	-1463.67	-1418.02

15:150000	YOL100W	YNL284C	-241.191	-236.093	-196.038
15:150000	YOL100W	YGL078C	1292.59	1301.69	1471.88
15:150000	YOL100W	YOR107W	32.8305	42.585	55.9712
15:150000	YOL100W	YML056C	608.196	613.663	742.295
15:150000	YOL100W	YGR128C	1158.74	1170.72	1244.41
15:150000	YOL100W	YOR207C	568.857	571.185	587.267
15:150000	YOL100W	YCR031C	-1009.19	-998.79	-963.424
15:150000	YOL100W	YPL211W	862.797	873.315	981.257
15:150000	YOL100W	YNL081C	-53.1143	-52.0857	47.7194
15:150000	YOL100W	YDL241W	-292.961	-288.836	-286.232
15:150000	YOL100W	YER131W	247.929	255.686	332.848
15:150000	YOL100W	YJL191W	49.7449	60.4224	126.404
15:150000	YOL100W	YFL036W	-400.381	-397.002	-166.402
15:150000	YOL100W	YHR183W	-925.208	-914.77	-772.496
15:150000	YOL100W	YPL266W	531.748	543.623	681.442
15:150000	YOL100W	YHR068W	-750.505	-745.637	-688.337
15:150000	YOL100W	YDR321W	39.0551	45.4176	131.31
15:150000	YOL100W	YMR038C	-820.087	-809.879	-785.372
15:150000	YOL100W	YLR325C	-256.952	-255.855	-215.854
15:150000	YOL100W	YGL077C	-453.187	-446.942	-452.755
15:150000	YOL100W	YMR056C	-395.268	-387.225	-344.225
15:150000	YOL100W	YDR500C	-269.135	-261.398	-214.329
15:150000	YOL100W	YLR301W	-961.188	-958.851	-960.243
15:150000	YOL100W	YDL051W	462.056	473.675	588.172
15:150000	YOL100W	YPL079W	-456.501	-455.562	-404.768
15:150000	YOL100W	YDL208W	436.443	448.681	578.834
15:150000	YOL100W	YDR047W	-428.76	-428.557	-387.863
15:150000	YOL100W	YHR007C	-861.359	-857.681	-812.879
15:150000	YOL100W	YDR161W	580.595	592.815	686.671
15:150000	YOL100W	YCR051W	-213.794	-203.303	-151.271
15:150000	YOL100W	YDR260C	-477.93	-465.929	-359.849
15:150000	YOL100W	YGR155W	-786.754	-784.484	-742.339
15:150000	YOL100W	YLR061W	-263.367	-258.637	-201.434
15:150000	YOL100W	YHR141C	-903.114	-891.231	-845.949
15:150000	YOL100W	YLR293C	-1305.76	-1297.21	-1253.72
15:150000	YOL100W	YGR220C	-148.76	-139.354	-126.072
15:150000	YOL100W	YHR009C	-730.32	-725.2	-728.116
15:150000	YOL100W	YMR308C	55.0121	67.2727	191.623
15:150000	YOL100W	YDR494W	-75.3817	-64.5471	37.7708
15:150000	YOL100W	YOR243C	179.62	191.787	299.581
15:150000	YOL100W	YER156C	-372.067	-361.037	-319.939
15:150000	YOL100W	YLR197W	746.883	757.878	888.174
15:150000	YOL100W	YLR333C	-536.61	-533.353	-488.411
15:150000	YOL100W	YHR045W	-826.614	-824.47	-815.8
15:150000	YOL100W	YJL190C	-168.414	-167.713	-126.46
15:150000	YOL100W	YER110C	403.65	414.016	525.575
15:150000	YOL100W	YBR189W	-373.233	-369.11	-338.772

15:150000	YOL100W	YPL160W	-529.838	-521.618	-416.071
15:150000	YOL100W	YNL302C	-492.344	-480.939	-413.459
15:150000	YOL100W	YHL011C	264.26	273.288	342.455
15:150000	YOL100W	YLR409C	1024.42	1035.88	1107.72
15:150000	YOL100W	YML126C	-1019.15	-1009.05	-1004.8
15:150000	YOL100W	YJR077C	-803.184	-792.875	-792.422
15:150000	YOL100W	YOR335C	-604.004	-596.839	-592.792
15:150000	YOL100W	YDR132C	-421.099	-415.172	-411.547
15:150000	YOL100W	YPR132W	-1026.22	-1014.75	-988.101
15:150000	YOL100W	YDR256C	72.991	73.071	222.16
15:150000	YOL100W	YKL006W	-560.12	-554.103	-511.651
15:150000	YOL100W	YLR372W	-289.703	-282.107	-243.792
15:150000	YOL100W	YER074W	-488.382	-483.788	-446.392
15:150000	YOL100W	YFR031C-A	-223.514	-217.141	-181.286
15:150000	YOL100W	YOL113W	-164.803	-164.454	-107.249
15:150000	YOL100W	YMR267W	17.6201	18.5019	61.2418
15:150000	YOL100W	YHL033C	-447.708	-444.988	-438.276
15:150000	YOL100W	YDL082W	182.395	184.828	273.403
15:150000	YOL100W	YMR011W	518.037	529.008	636.078
15:150000	YOL100W	YML106W	-807.605	-799.868	-766.932
15:150000	YOL100W	YJR005W	-555.524	-551.222	-550.447
15:150000	YOL100W	YJR041C	585.908	589.977	602.126
15:150000	YOL100W	YNL247W	-305.077	-295.698	-246.953
15:150000	YOL100W	YOL103W	-961.454	-955.989	-945.487
15:150000	YOL100W	YOR276W	-759.392	-754.889	-735.871
15:150000	YOL100W	YPR018W	-445.231	-437.89	-443.147
15:150000	YOL100W	YPL040C	-66.3149	-54.4902	-3.2168
15:150000	YOL100W	YLR432W	-28.847	-16.6046	61.8927
15:150000	YOL100W	YGL115W	-1029.52	-1028.02	-939.86
15:150000	YOL100W	YLR449W	753.663	764.71	836.497
15:150000	YOL100W	YHR106W	-759.209	-756.909	-754.491
15:150000	YOL100W	YMR230W	-257.322	-245.664	-179.572
15:150000	YOL100W	YDL014W	596.929	607.247	739.371
15:150000	YOL100W	YDR023W	-971.68	-962.889	-941.904
15:150000	YOL100W	YGL147C	-388.117	-381.065	-329.448
15:150000	YOL100W	YOR286W	-339.571	-332.407	-337.62
15:150000	YOL100W	YOL056W	-570.141	-560.605	-545.481
15:150000	YOL100W	YNL302C	-912.15	-907.774	-876.633
15:150000	YOL100W	YER001W	-81.3454	-69.663	-50.663
15:150000	YOL100W	YNL255C	-184.641	-172.409	-78.2876
15:150000	YOL100W	YLR375W	-676.974	-673.923	-673.463
15:150000	YOL100W	YKL155C	-98.9113	-98.9058	30.9444
15:150000	YOL100W	YLL046C	59.1083	69.4992	218.408
15:150000	YOL100W	YLR228C	387.782	395.598	502.018
15:150000	YOL100W	YGL103W	-1300.41	-1297.09	-1237.83
15:150000	YOL100W	YKL052C	-847.914	-837.123	-836.16
15:150000	YOL100W	YOL089C	-476.198	-473.002	-475.693

15:150000	YOL100W	YMR312W	-345.049	-336.757	-259.142
15:150000	YOL100W	YJL010C	562.51	571.606	628.68
15:150000	YOL100W	YBR148W	22.5732	31.138	99.9795
15:150000	YOL100W	YBL087C	-749.894	-741.11	-702.562
15:150000	YOL100W	YOL121C	9.4794	14.6522	81.4067
15:150000	YOL100W	YGL123W	-954.455	-942.543	-907.802
15:150000	YOL100W	YNL137C	-151.467	-139.676	-95.9677
15:150000	YOL100W	YOR312C	-714.073	-704.944	-655.701
15:150000	YOL100W	YHR088W	1055.64	1057.16	1060.94
15:150000	YOL100W	YNL069C	-707.793	-706.784	-670.904
15:150000	YOL100W	YJR122W	-195.983	-184.467	-183.882
15:150000	YOL100W	YCR034W	-388.58	-379.107	-273.789
15:150000	YOL100W	YJL034W	-695.056	-687.605	-580.082
15:150000	YOL100W	YOL091W	343.808	347.016	691.784
15:150000	YOL100W	YHR010W	-565.952	-559.211	-521.643
15:150000	YOL100W	YGR077C	-871.669	-869.03	-841.787
15:150000	YOL100W	YGR102C	-195.398	-188.508	-42.3971
15:150000	YOL100W	YKL216W	944.264	955.256	996.695
15:150000	YOL100W	YGR123C	579.634	585.398	728.899
15:150000	YOL100W	YPL265W	-596.026	-586.156	-542.052
15:150000	YOL100W	YKL087C	-80.8846	-68.8762	85.1089
15:150000	YOL100W	YMR072W	-999.014	-994.291	-998.028
15:150000	YOL100W	YER049W	362.1	364.398	376.059
15:150000	YOL100W	YJL062W-A	-134.878	-128.077	-132.3
15:150000	YOL100W	YER074W	-666.94	-659.208	-629.896
15:150000	YOL100W	YJR043C	-537.238	-530.116	-532.65
15:150000	YOL100W	YBL030C	-718.738	-718.007	-715.873
15:150000	YOL100W	YGR118W	-1069.55	-1059.55	-1049.25
15:150000	YOL100W	YDR421W	-307.276	-303.471	-301.618
15:150000	YOL100W	YOR096W	-516.959	-511.183	-473.253
15:150000	YOL100W	YLR406C	262.574	272.767	325.217
15:150000	YOL100W	YOR293W	-103.814	-96.3715	-31.441
15:150000	YOL100W	YDR091C	240.425	252.373	368.548
15:150000	YOL100W	YKR076W	-39.7852	-32.5614	90.7724
15:150000	YOL100W	YGR021W	115.284	118.302	147.269
15:150000	YOL100W	YGR200C	581.613	592.351	671.167
15:150000	YOL100W	YHR064C	-749.03	-738.996	-704.089
15:150000	YOL100W	YMR297W	-1031.21	-1029.19	-1010.69
15:150000	YOL100W	YGL076C	-1208	-1199.33	-1171.65
15:150000	YOL100W	YDR296W	89.0076	93.8885	101.484
15:150000	YOL100W	YOR001W	222.211	234.079	311.212
15:150000	YOL100W	YMR286W	-48.6498	-38.9986	-41.4577
15:150000	YOL100W	YNR060W	592.52	596.575	608.035
15:150000	YOL100W	YFR032C-A	-603.43	-591.442	-537.101
15:150000	YOL100W	YPL061W	-90.3519	-89.0354	26.0298
15:150000	YOL100W	YDL211C	-419.519	-410.344	-326.371
15:150000	YOL100W	YPL156C	12.9247	24.4388	18.4484

15:150000	YOL100W	YER117W	-572.825	-562.539	-517.144
15:150000	YOL100W	YBR191W	-748.571	-744.753	-687.063
15:150000	YOL100W	YOR063W	-1150.45	-1148.38	-1139.66
15:150000	YOL100W	YPR036W	-1181.7	-1176.29	-1044.64
15:150000	YOL100W	YMR145C	122.362	130.296	124.173
15:150000	YOL100W	YBR025C	-775.592	-765.639	-680.237
15:150000	YOL100W	YLR441C	-463.163	-460.058	-392.727
15:150000	YOL100W	YGR285C	-1096.05	-1089.04	-1063.4
15:150000	YOL100W	YLL034C	893.175	904.01	965.856
15:150000	YOL100W	YHR019C	-820.058	-811.237	-813.831
15:150000	YOL100W	YDR041W	-63.1022	-53.3652	8.15573
15:150000	YOL100W	YDR197W	-531.583	-529.403	-464.611
15:150000	YOL100W	YAR029W	153.468	153.731	235.986
15:150000	YOL100W	YBR031W	-1357.15	-1353.67	-1331.59
15:150000	YOL100W	YLL045C	-707.306	-700.688	-674.782
15:150000	YOL100W	YGR035C	82.8774	89.4464	86.0874
15:150000	YOL100W	YER025W	-381.129	-369.728	-301.724
15:150000	YOL100W	YOR315W	311.878	322.432	535.638
15:150000	YOL100W	YLR340W	-992.989	-981.237	-932.375
15:150000	YOL100W	YOL094C	-357.915	-348.038	-332.352
15:150000	YOL100W	YLL056C	-447.898	-443.225	-362.486
15:150000	YOL100W	YLR118C	-655.513	-652.488	-588.734
15:150000	YOL100W	YOL040C	-755.826	-752.349	-728.318
15:150000	YOL100W	YNL061W	893.471	904.64	999.488
15:150000	YOL100W	YBR181C	-1097.4	-1090.86	-1025.46
15:150000	YOL100W	YKL014C	857.648	869.681	1015.75
15:150000	YOL100W	YDL081C	-795.571	-784.639	-738.531
15:150000	YOL100W	YJR101W	-77.1184	-72.7139	-71.0296
15:150000	YOL100W	YBR185C	102.165	105.979	140.24
15:150000	YOL100W	YDR324C	875.387	886.406	956.023
15:150000	YOL100W	YHR208W	-11.8078	-6.74174	-4.78959
15:150000	YOL100W	YGL025C	-478.866	-476.2	-468.207
15:150000	YOL100W	YAL041W	-754.868	-749.352	-753.884
15:150000	YOL100W	YGR152C	-462.655	-454.89	-410.567
15:150000	YOL100W	YHR203C	-1428.83	-1416.7	-1345.6
15:150000	YOL100W	YMR143W	-850.035	-839.617	-785.898
15:150000	YOL100W	YKL073W	-614.654	-603.295	-491.48
15:150000	YOL100W	YBR048W	-818.055	-808.191	-767.504
15:150000	YOL100W	YGL135W	-1336.54	-1324.37	-1257.89
15:150000	YOL100W	YFL022C	-792.463	-782.197	-704.495
15:150000	YOL100W	YBR147W	767.804	772.306	823.027
15:150000	YOL100W	YOR134W	460.421	465.477	566.878
15:150000	YOL100W	YJL177W	-36.3619	-33.9217	25.4554
15:150000	YOL100W	YLR083C	-437.59	-433.817	-416.978
15:150000	YOL100W	YJR094W-A	-107.803	-99.0302	-27.276
15:150000	YOL100W	YJR090C	-859.809	-858.793	-693.257
15:150000	YOL100W	YDL075W	-666.755	-658.676	-621.288

15:150000	YOL100W	YHL001W	-946.009	-936.662	-894.922
15:150000	YOL100W	YDR482C	-511.603	-499.911	-350.038
15:150000	YOL100W	YHR021C	-537.17	-532.133	-487.153
15:150000	YOL100W	YMR217W	339.896	349.268	460.399
15:150000	YOL100W	YBR191W	-278.812	-268.174	-219.105
15:150000	YOL100W	YOR234C	-170.769	-164.985	-117.266
15:150000	YOL100W	YEL071W	-529.534	-523.063	-528.523
15:150000	YOL100W	YBL092W	-949.864	-945.459	-902.43
15:150000	YOL100W	YGR094W	-879.536	-873.411	-832.337
15:150000	YOL100W	YFR006W	-945.291	-938.599	-934.478
15:150000	YOL100W	YPR127W	-472.751	-467.122	-456.266
15:150000	YOL100W	YJL136C	-870.439	-866.299	-840.55
15:150000	YOL100W	YIL133C	-102.587	-93.6533	-46.0574
15:150000	YOL100W	YFR031C-A	-951.031	-941.458	-922.634
15:150000	YOL100W	YOR293W	-138.132	-129.915	-70.6773
15:150000	YOL100W	YGL030W	-1167.66	-1156.85	-1114.09
15:150000	YOL096C	YML035C	-488.191	-485.276	-413.708
15:150000	YOL096C	YDR447C	-693.529	-685.126	-604.088
15:150000	YOL096C	YDR144C	105.91	115.354	175.706
15:150000	YOL096C	YJL185C	53.3921	65.1455	174.64
15:150000	YOL096C	YDR447C	-351.991	-341.756	-210.239
15:150000	YOL096C	YHR197W	1032.11	1040.86	1136.84
15:150000	YOL096C	YDR245W	-758.549	-756.591	-726.097
15:150000	YOL096C	YKL192C	-968.005	-966.273	-877.393
15:150000	YOL096C	YDL061C	-895.354	-883.947	-780.567
15:150000	YOL096C	YCL054W	754.663	760.686	779.252
15:150000	YOL096C	YHR128W	-397.091	-391.839	-387.005
15:150000	YOL096C	YOL077W-A	-325.35	-315.133	-312.439
15:150000	YOL096C	YDL191W	-1209.29	-1199.11	-1103.72
15:150000	YOL096C	YBR084C-A	-425.319	-421.804	-321.609
15:150000	YOL096C	YAL003W	-836.112	-828.186	-689.582
15:150000	YOL096C	YDR450W	-888.197	-882.837	-825.583
15:150000	YOL096C	YLR029C	-1075.49	-1065.06	-987.948
15:150000	YOL096C	YMR128W	847.087	854.776	947.045
15:150000	YOL096C	YMR131C	764.477	767.669	992.135
15:150000	YOL096C	YNL055C	-1250.37	-1238.24	-1206.54
15:150000	YOL096C	YPL131W	-855.167	-844.516	-762.438
15:150000	YOL096C	YKR092C	577.399	581.473	596.175
15:150000	YOL096C	YDL085W	573.203	578.141	730.761
15:150000	YOL096C	YDL173W	-745.577	-742.619	-635.427
15:150000	YOL096C	YML124C	-1243.43	-1238.18	-1241.18
15:150000	YOL096C	YHR065C	725.219	736.935	777.781
15:150000	YOL096C	YDR454C	-1254.72	-1247.89	-1153.43
15:150000	YOL096C	YJR123W	-1078.15	-1070.35	-1003.44
15:150000	YOL096C	YGR027C	-1092.46	-1081.04	-1037.75
15:150000	YOL096C	YGL253W	-735.335	-724.394	-516.372
15:150000	YOL096C	YDL229W	-72.9663	-66.4223	20.6399

15:150000	YOL096C	YMR301C	-186.847	-181.061	-138.877
15:150000	YOL096C	YLR185W	-483.435	-477.606	-354.147
15:150000	YOL096C	YML093W	947.474	957.979	1018.96
15:150000	YOL096C	YLL028W	-284.43	-272.814	-258.37
15:150000	YOL096C	YKL081W	-862.972	-857.166	-656.618
15:150000	YOL096C	YGL031C	-955.324	-945.786	-902.833
15:150000	YOL096C	YGL013C	-713.326	-702.928	-598.296
15:150000	YOL096C	YBL072C	-1384.09	-1377.07	-1307.44
15:150000	YOL096C	YER102W	-714.393	-706.684	-616.135
15:150000	YOL096C	YGL189C	-1155.99	-1152.15	-1095.3
15:150000	YOL096C	YPL189W	-198.385	-186.989	-177.077
15:150000	YOL096C	YGR148C	-1112.19	-1105.92	-1013.76
15:150000	YOL096C	YDR345C	-184.557	-176.711	-127.425
15:150000	YOL096C	YAL042W	-1104.68	-1098.44	-1067.22
15:150000	YOL096C	YER007C-A	-911.858	-904.758	-802.608
15:150000	YOL096C	YOL061W	-435.583	-424.622	-305.707
15:150000	YOL096C	YBL068W	273.386	282.64	412.431
15:150000	YOL096C	YPL272C	211.456	222.949	309.472
15:150000	YOL096C	YDR090C	72.748	74.5241	82.0099
15:150000	YOL096C	YMR142C	-850.022	-846.921	-747.606
15:150000	YOL096C	YDL229W	-533.119	-524.581	-435.526
15:150000	YOL096C	YMR239C	666.25	671.656	678.604
15:150000	YOL096C	YDR064W	-1075.17	-1070.16	-1017.74
15:150000	YOL096C	YNL073W	-286.823	-283.979	-271.602
15:150000	YOL096C	YDL031W	318.03	326.734	383.021
15:150000	YOL096C	YGL111W	420.842	428.45	564.716
15:150000	YOL096C	YLR249W	-490.284	-478.547	-353.77
15:150000	YOL096C	YLR222C	1115.91	1124.13	1158.37
15:150000	YOL096C	YMR241W	-734.137	-722.764	-598.982
15:150000	YOL096C	YER006W	814.133	815.705	996.035
15:150000	YOL096C	YLR267W	761.819	767.209	816.677
15:150000	YOL096C	YFR039C	-662.181	-658.242	-657.655
15:150000	YOL096C	YPR140W	-454.371	-449.268	-383.636
15:150000	YOL096C	YDR502C	-588.715	-576.934	-489.09
15:150000	YOL096C	YER126C	783.047	794.182	858.076
15:150000	YOL096C	YGL189C	-1003.18	-992.227	-949.708
15:150000	YOL096C	YPR043W	-1255.54	-1245.52	-1152.99
15:150000	YOL096C	YPL132W	-107.793	-101.308	30.0455
15:150000	YOL096C	YOL127W	-1201.01	-1192.9	-1108.33
15:150000	YOL096C	YLR172C	-106.842	-96.3038	63.3679
15:150000	YOL096C	YOL022C	138.826	150.477	257.2
15:150000	YOL096C	YIL091C	819.53	819.743	834.485
15:150000	YOL096C	YEL047C	-947.623	-936.904	-868.819
15:150000	YOL096C	YML026C	-378.897	-374.806	-251.348
15:150000	YOL096C	YMR116C	-1181.72	-1170.04	-1082.5
15:150000	YOL096C	YGR031W	-451.63	-448.628	-252.777
15:150000	YOL096C	YIR012W	322.931	323.994	346.579

15:150000	YOL096C	YGR201C	-317.341	-312.591	-114.738
15:150000	YOL096C	YPL043W	1094.81	1104.07	1192.15
15:150000	YOL096C	YLR150W	-1291.13	-1280.54	-1223.77
15:150000	YOL096C	YGR033C	-774.656	-766.594	-586.302
15:150000	YOL096C	YJL189W	-621.122	-612.568	-536.354
15:150000	YOL096C	YIR037W	-1101.78	-1089.86	-927.463
15:150000	YOL096C	YPL048W	-1291.87	-1281.93	-1203.92
15:150000	YOL096C	YKR026C	-340.198	-339.696	-315.06
15:150000	YOL096C	YML063W	-720.28	-710.873	-664.764
15:150000	YOL096C	YNL044W	-901.429	-895.642	-897.474
15:150000	YOL096C	YPL221W	-618.684	-618.07	-529.755
15:150000	YOL096C	YKR094C	-614.074	-612.95	-480.807
15:150000	YOL096C	YGL065C	-893.686	-887.949	-659.141
15:150000	YOL096C	YEL050C	-103.096	-92.3036	-73.149
15:150000	YOL096C	YBL072C	-875.15	-873.987	-798.778
15:150000	YOL096C	YDR012W	-403.752	-398.493	-252.164
15:150000	YOL096C	YGL022W	-1186.38	-1180.37	-1168.75
15:150000	YOL096C	YHR156C	-212.984	-209.071	-182.3
15:150000	YOL096C	YOR312C	-686.772	-684.95	-551.325
15:150000	YOL096C	YEL055C	-35.7898	-29.5703	120.795
15:150000	YOL096C	YDL213C	218.477	229.054	366.156
15:150000	YOL096C	YNL178W	-781.619	-773.805	-718.258
15:150000	YOL096C	YDR430C	73.798	74.4603	96.6311
15:150000	YOL096C	YIL108W	-706.023	-694.989	-670.874
15:150000	YOL096C	YGL040C	-396.113	-386.477	-321.584
15:150000	YOL096C	YPL118W	-388.583	-382.924	-380.552
15:150000	YOL096C	YDR341C	-558.1	-549.05	-416.441
15:150000	YOL096C	YKL212W	-578.314	-576.046	-550.258
15:150000	YOL096C	YJL014W	-981.902	-979.375	-968.25
15:150000	YOL096C	YPR110C	229.047	231.057	457.231
15:150000	YOL096C	YKL004W	-343.123	-334.936	-171.038
15:150000	YOL096C	YOL041C	534.411	546.026	618.864
15:150000	YOL096C	YDR032C	-1179.59	-1176.87	-1116.46
15:150000	YOL096C	YPL183W-A	-92.1335	-86.3965	-22.7582
15:150000	YOL096C	YOL080C	645.957	655.88	689.215
15:150000	YOL096C	YOL081W	-591.997	-581.615	-587.639
15:150000	YOL096C	YAL036C	751.045	762.471	872.982
15:150000	YOL096C	YOR046C	-874.456	-865.159	-810.483
15:150000	YOL096C	YNL175C	1179.56	1179.92	1339
15:150000	YOL096C	YOR004W	1042.22	1054.14	1119
15:150000	YOL096C	YPL207W	209.043	219.843	360.795
15:150000	YOL096C	YPL239W	68.5947	77.6618	213.398
15:150000	YOL096C	YOR206W	1216.4	1219.54	1350.64
15:150000	YOL096C	YNR036C	-427.933	-420.444	-421.366
15:150000	YOL096C	YDL061C	-978.544	-970.325	-844.912
15:150000	YOL096C	YLR193C	-634.507	-632.382	-562.237
15:150000	YOL096C	YDL024C	-23.3785	-19.4752	57.5386

15:150000	YOL096C	YAL012W	-890.289	-878.037	-776.21
15:150000	YOL096C	YJR016C	-106.07	-100.902	-106
15:150000	YOL096C	YIL127C	747.534	757.578	785.801
15:150000	YOL096C	YGR256W	98.2919	98.6397	208.142
15:150000	YOL096C	YLR002C	895.729	907.163	967.63
15:150000	YOL096C	YDL121C	-77.8532	-76.1742	-55.545
15:150000	YOL096C	YMR012W	-711.52	-705.179	-657.018
15:150000	YOL096C	YHL034C	-1354.77	-1345.33	-1193.33
15:150000	YOL096C	YCL057C-A	-892.549	-882.837	-804.571
15:150000	YOL096C	YML024W	-967.349	-956.834	-915.206
15:150000	YOL096C	YNL209W	-240.506	-230.381	-132.799
15:150000	YOL096C	YDR303C	-642.521	-635.173	-578.378
15:150000	YOL096C	YOR167C	-716.692	-712.904	-590.319
15:150000	YOL096C	YML042W	-103.106	-91.6003	-18.7224
15:150000	YOL096C	YOL039W	-865.361	-856.572	-754.692
15:150000	YOL096C	YJL066C	-188.238	-187.795	-87.7925
15:150000	YOL096C	YNR033W	-395.25	-391.399	-383.262
15:150000	YOL096C	YPL249C-A	-935.802	-933.761	-873.105
15:150000	YOL096C	YLR196W	1095.5	1107.71	1181.7
15:150000	YOL096C	YIL078W	-1105.3	-1094.38	-1015.96
15:150000	YOL096C	YMR182W-A	142.4	151.978	427.807
15:150000	YOL096C	YOR354C	-327.668	-315.353	-294.416
15:150000	YOL096C	YLR023C	-343.228	-335.113	-132.743
15:150000	YOL096C	YLR168C	92.6006	102.577	116.507
15:150000	YOL096C	YPL012W	1180.14	1185.36	1337.22
15:150000	YOL096C	YKL056C	-1518.34	-1516.78	-1455.83
15:150000	YOL096C	YNL284C	-208.632	-201.17	-194.924
15:150000	YOL096C	YJL045W	54.4085	57.0721	87.4123
15:150000	YOL096C	YOR107W	-16.6097	-12.5168	56.673
15:150000	YOL096C	YGR128C	1146.67	1156.44	1266.48
15:150000	YOL096C	YOR207C	408.419	419.224	597.681
15:150000	YOL096C	YCR031C	-1074.07	-1063.86	-980.927
15:150000	YOL096C	YPL211W	810.028	820.059	996.562
15:150000	YOL096C	YNL081C	5.63597	6.59225	51.6065
15:150000	YOL096C	YHR183W	-982.062	-971.314	-789.321
15:150000	YOL096C	YPL266W	468.032	470.215	689.412
15:150000	YOL096C	YHR068W	-905.544	-898.52	-699.178
15:150000	YOL096C	YMR038C	-847.787	-839.328	-802.271
15:150000	YOL096C	YKL046C	-1039.17	-1034.6	-991.716
15:150000	YOL096C	YGL077C	-479.232	-472.402	-472.076
15:150000	YOL096C	YMR056C	-397.166	-388.095	-353.008
15:150000	YOL096C	YDL208W	407.689	413.551	590.666
15:150000	YOL096C	YHR007C	-824.496	-818.733	-806.865
15:150000	YOL096C	YDR161W	575.433	585.244	695.255
15:150000	YOL096C	YDR260C	-497.444	-489.261	-363.259
15:150000	YOL096C	YHR141C	-991	-982.866	-860.572
15:150000	YOL096C	YJR132W	98.9331	106.584	189.439

15:150000	YOL096C	YLR293C	-1415.8	-1403.57	-1284.57
15:150000	YOL096C	YGR220C	-138.401	-127.816	-127.326
15:150000	YOL096C	YHR009C	-781.745	-776.026	-757.623
15:150000	YOL096C	YMR308C	78.1127	87.8836	192.196
15:150000	YOL096C	YDR494W	-48.386	-40.8611	34.6416
15:150000	YOL096C	YOR243C	97.8237	99.5612	304.563
15:150000	YOL096C	YER156C	-398.268	-388.527	-332.691
15:150000	YOL096C	YHR045W	-863.715	-857.222	-840.675
15:150000	YOL096C	YOR254C	-765.924	-759.801	-698.839
15:150000	YOL096C	YNL302C	-501.625	-492.163	-425.246
15:150000	YOL096C	YOR358W	-385.987	-379.54	-301.687
15:150000	YOL096C	YHL011C	80.2775	86.3879	342.352
15:150000	YOL096C	YLR409C	1021.49	1030.91	1128.25
15:150000	YOL096C	YML126C	-1058.63	-1046.53	-1018.86
15:150000	YOL096C	YJR077C	-807.648	-796.644	-801.977
15:150000	YOL096C	YDR132C	-444.684	-440.675	-414.332
15:150000	YOL096C	YPR132W	-1091.67	-1082.56	-1011.55
15:150000	YOL096C	YDR256C	13.512	15.7105	240.657
15:150000	YOL096C	YKL006W	-618.509	-615.44	-521.694
15:150000	YOL096C	YLR372W	-426.153	-416.092	-241.9
15:150000	YOL096C	YER074W	-560.507	-559.128	-452.314
15:150000	YOL096C	YOL113W	-154.513	-146.913	-104.393
15:150000	YOL096C	YMR267W	56.4204	58.0081	61.639
15:150000	YOL096C	YHL033C	-584.424	-574.419	-445.524
15:150000	YOL096C	YMR011W	510.512	518.617	645.828
15:150000	YOL096C	YML106W	-996.685	-988.537	-771.514
15:150000	YOL096C	YJR005W	-564.165	-561.983	-557.303
15:150000	YOL096C	YJR041C	498.731	510.028	612.306
15:150000	YOL096C	YOL095C	-296.508	-289.585	-228.918
15:150000	YOL096C	YNL247W	-414.226	-402.388	-254.817
15:150000	YOL096C	YOL103W	-997.264	-996.105	-959.634
15:150000	YOL096C	YOR276W	-899.664	-888.989	-748.134
15:150000	YOL096C	YPR018W	-458.086	-450.106	-452.699
15:150000	YOL096C	YPL040C	-46.4775	-34.2702	0.0128543
15:150000	YOL096C	YLR432W	-174.957	-171.143	59.7967
15:150000	YOL096C	YLR449W	581.799	587.45	847.774
15:150000	YOL096C	YHR106W	-815.647	-810.04	-785.693
15:150000	YOL096C	YPR172W	-558.199	-554.868	-448.385
15:150000	YOL096C	YMR230W	-351.932	-350.919	-185.136
15:150000	YOL096C	YDR023W	-1118.57	-1107.5	-968.132
15:150000	YOL096C	YGL147C	-375.893	-366.696	-340.51
15:150000	YOL096C	YOR286W	-400.412	-392.417	-344.16
15:150000	YOL096C	YOL056W	-593.723	-584.065	-563.984
15:150000	YOL096C	YNL302C	-986.227	-984.336	-887.908
15:150000	YOL096C	YER001W	-97.3473	-90.5002	-55.9074
15:150000	YOL096C	YLL046C	78.8331	82.7105	218.89
15:150000	YOL096C	YLR228C	264.257	273.83	515.07

15:150000	YOL096C	YGL103W	-1299.66	-1298.7	-1271.89
15:150000	YOL096C	YKL052C	-866.616	-856.143	-858.459
15:150000	YOL096C	YMR312W	-318.807	-311.115	-268.879
15:150000	YOL096C	YJL010C	571.567	579.78	638.609
15:150000	YOL096C	YBR148W	-17.0788	-5.37324	91.391
15:150000	YOL096C	YBL087C	-837.977	-830.83	-715.521
15:150000	YOL096C	YGL123W	-1001.01	-989.233	-931.987
15:150000	YOL096C	YHR025W	-711.538	-705.97	-691.929
15:150000	YOL096C	YNL137C	-125.039	-113.016	-97.0474
15:150000	YOL096C	YOR312C	-778.362	-773.612	-668.788
15:150000	YOL096C	YHR088W	1027.23	1035.1	1071.35
15:150000	YOL096C	YMR174C	314.506	320.097	469.009
15:150000	YOL096C	YNL069C	-782.592	-777.675	-678.116
15:150000	YOL096C	YJR122W	-192.297	-182.504	-188.618
15:150000	YOL096C	YCR034W	-522.609	-515.229	-280.418
15:150000	YOL096C	YOL091W	529.581	533.124	683.741
15:150000	YOL096C	YHR010W	-633.649	-632.475	-523.295
15:150000	YOL096C	YGR102C	-302.091	-292.587	-51.1179
15:150000	YOL096C	YER082C	651.088	659.583	698.035
15:150000	YOL096C	YKL216W	880.152	889.193	1015.91
15:150000	YOL096C	YPL265W	-599.138	-587.689	-562.832
15:150000	YOL096C	YKL087C	-60.0088	-51.5386	87.0814
15:150000	YOL096C	YMR072W	-1079.7	-1067.8	-1019.69
15:150000	YOL096C	YER049W	231.231	240.994	376.677
15:150000	YOL096C	YJL062W-A	-173.2	-167.613	-130.614
15:150000	YOL096C	YER074W	-716.236	-711.079	-643.507
15:150000	YOL096C	YJR043C	-559.381	-554.496	-554.639
15:150000	YOL096C	YGR118W	-1161.61	-1150.58	-1077.69
15:150000	YOL096C	YKL127W	-932.976	-932.012	-895.936
15:150000	YOL096C	YOR096W	-556.666	-550.365	-478.127
15:150000	YOL096C	YDR091C	188.657	196.592	369.804
15:150000	YOL096C	YKR076W	-60.0195	-52.277	86.4201
15:150000	YOL096C	YGR200C	565.588	577.615	675.518
15:150000	YOL096C	YHR064C	-846.433	-835.443	-728.489
15:150000	YOL096C	YGL076C	-1303.65	-1291.88	-1196.78
15:150000	YOL096C	YDR296W	95.1393	96.7592	102.951
15:150000	YOL096C	YOR001W	224.807	233.515	311.777
15:150000	YOL096C	YMR286W	-52.7752	-42.8101	-41.7316
15:150000	YOL096C	YER056C	-365.389	-364.445	-361.974
15:150000	YOL096C	YNR060W	603.046	604.493	618.833
15:150000	YOL096C	YFR032C-A	-632.786	-623.411	-551.293
15:150000	YOL096C	YPL061W	-100.829	-98.0501	20.2044
15:150000	YOL096C	YDL211C	-529.909	-522.458	-333.041
15:150000	YOL096C	YPL156C	28.6996	39.2358	33.9375
15:150000	YOL096C	YER117W	-659.224	-651.49	-529.27
15:150000	YOL096C	YKR079C	903.513	908.277	936.699
15:150000	YOL096C	YMR145C	123.974	134.073	131.849

15:150000	YOL096C	YBR025C	-844.827	-839.827	-704.73
15:150000	YOL096C	YGR285C	-1173.37	-1162.46	-1084.92
15:150000	YOL096C	YLL034C	874.404	884.818	982.932
15:150000	YOL096C	YML110C	-1123.33	-1117.21	-941.592
15:150000	YOL096C	YHR019C	-931.418	-928.698	-831.364
15:150000	YOL096C	YDR041W	-6.64711	3.45692	8.80161
15:150000	YOL096C	YAR029W	218.427	218.915	237.38
15:150000	YOL096C	YBR031W	-1431.94	-1429.46	-1364.63
15:150000	YOL096C	YLL045C	-774.352	-767.815	-689.084
15:150000	YOL096C	YER025W	-552.763	-549.853	-309.103
15:150000	YOL096C	YOR315W	324.813	329.212	543.389
15:150000	YOL096C	YNL234W	-94.5996	-92.2656	101.594
15:150000	YOL096C	YLR340W	-1092.6	-1086.68	-957.335
15:150000	YOL096C	YOL094C	-358.009	-353.078	-347.256
15:150000	YOL096C	YLL056C	-470.972	-461.082	-371.877
15:150000	YOL096C	YLR118C	-733.497	-722.271	-604.605
15:150000	YOL096C	YOL040C	-813.179	-806.975	-735.691
15:150000	YOL096C	YNL061W	834.127	838.789	1011.94
15:150000	YOL096C	YDL081C	-839.804	-834.924	-761.884
15:150000	YOL096C	YJR101W	-86.5237	-81.2121	-76.215
15:150000	YOL096C	YBR185C	134.216	138.066	145.648
15:150000	YOL096C	YDR324C	867.189	877.89	971.113
15:150000	YOL096C	YOR247W	-1112.68	-1106.64	-958.157
15:150000	YOL096C	YHR203C	-1459.81	-1451.82	-1368.1
15:150000	YOL096C	YMR143W	-895.732	-891.992	-807.427
15:150000	YOL096C	YKL073W	-613.472	-605.553	-510.007
15:150000	YOL096C	YBR048W	-880.113	-873.607	-781.773
15:150000	YOL096C	YGL135W	-1412.44	-1403.75	-1288.1
15:150000	YOL096C	YBR071W	-304.467	-299.939	-253.396
15:150000	YOL096C	YER096W	-5.31166	-3.69296	98.491
15:150000	YOL096C	YFL022C	-878.368	-869.889	-711.577
15:150000	YOL096C	YOR134W	388.264	397.195	582.1
15:150000	YOL096C	YNR001C	-504.558	-502.251	-452.021
15:150000	YOL096C	YLR083C	-535.342	-525.109	-429.577
15:150000	YOL096C	YGR207C	-685.702	-676.629	-599.09
15:150000	YOL096C	YJR090C	-797.169	-793.055	-713.131
15:150000	YOL096C	YDL075W	-729.532	-724.898	-642.254
15:150000	YOL096C	YHL001W	-1034.6	-1030.93	-903.582
15:150000	YOL096C	YDR482C	-571.806	-563.672	-364.589
15:150000	YOL096C	YHR021C	-607.334	-606.362	-490.168
15:150000	YOL096C	YMR217W	274.728	276.308	463.077
15:150000	YOL096C	YBR191W	-304.814	-301.197	-225.513
15:150000	YOL096C	YOR234C	-359.422	-352.333	-110.83
15:150000	YOL096C	YDR104C	-473.248	-470.872	-464.539
15:150000	YOL096C	YEL071W	-554.552	-545.872	-537.942
15:150000	YOL096C	YBL092W	-972.524	-966.792	-918.068
15:150000	YOL096C	YGR094W	-1028.99	-1022.28	-855.434

15:150000	YOL096C	YDR434W	-1071.49	-1063.6	-1068.03
15:150000	YOL096C	YFR006W	-968.293	-962.662	-957.027
15:150000	YOL096C	YPR127W	-543.258	-533.498	-475.457
15:150000	YOL096C	YOL030W	-1107.68	-1102.83	-1107.3
15:150000	YOL096C	YFR031C-A	-1001.63	-991.529	-930.093
15:150000	YOL096C	YOR293W	-200.156	-199.24	-67.0033
15:150000	YOL096C	YGL030W	-1225.86	-1214.15	-1142.36
15:150000	YOL096C	YPR028W	-1401.71	-1401.59	-1353.58
15:150000	YOL087C	YDR447C	-635.901	-626.817	-605.792
15:150000	YOL087C	YDR447C	-252.963	-251.171	-211.799
15:150000	YOL087C	YNR055C	-663.038	-656.443	-640.456
15:150000	YOL087C	YOR084W	-302.827	-298.911	-80.193
15:150000	YOL087C	YOL077W-A	-358.186	-353.447	-314.782
15:150000	YOL087C	YBR084C-A	-352.31	-347.557	-321.802
15:150000	YOL087C	YAL003W	-735.452	-734.829	-688.941
15:150000	YOL087C	YDR450W	-858.425	-851.978	-831.339
15:150000	YOL087C	YLR029C	-1022.21	-1020.52	-990.566
15:150000	YOL087C	YNL055C	-1242.81	-1238.92	-1208.61
15:150000	YOL087C	YPL131W	-793.636	-792.089	-763.307
15:150000	YOL087C	YOL036W	-800.59	-795.673	-694.297
15:150000	YOL087C	YJR123W	-1038.92	-1030.45	-1001.85
15:150000	YOL087C	YGR027C	-1051.13	-1045.45	-1035.58
15:150000	YOL087C	YLL028W	-273.149	-264.332	-258.263
15:150000	YOL087C	YCR073C	688.632	699.242	814.881
15:150000	YOL087C	YOL109W	-515.005	-511.86	-507.051
15:150000	YOL087C	YGL031C	-927.411	-917.071	-903.002
15:150000	YOL087C	YGL013C	-622.682	-614.829	-592.328
15:150000	YOL087C	YBL072C	-1352.49	-1341.42	-1308.05
15:150000	YOL087C	YER102W	-659.983	-659.802	-618.147
15:150000	YOL087C	YGL189C	-1117.74	-1115.91	-1095.06
15:150000	YOL087C	YKR057W	-278.563	-275.109	-230.943
15:150000	YOL087C	YPL189W	-192.981	-180.818	-174.071
15:150000	YOL087C	YGR148C	-1051.5	-1048.6	-1014.41
15:150000	YOL087C	YPL272C	279.855	283.224	309.638
15:150000	YOL087C	YOR341W	1027.06	1029.82	1183.9
15:150000	YOL087C	YGR287C	392.06	394.503	477.181
15:150000	YOL087C	YDL229W	-461.376	-458.66	-432.774
15:150000	YOL087C	YNR066C	658.277	663.409	684.184
15:150000	YOL087C	YDR337W	116.206	118.347	130.024
15:150000	YOL087C	YHR072W	-428.736	-423.983	-375.023
15:150000	YOL087C	YBR023C	-300.169	-291.265	-275.493
15:150000	YOL087C	YDR349C	-404.216	-393.028	-269.795
15:150000	YOL087C	YCL059C	690.44	693.703	815.555
15:150000	YOL087C	YGR166W	284.168	295.895	469.085
15:150000	YOL087C	YLR267W	818.223	822.478	823.04
15:150000	YOL087C	YPR010C	962.186	963.316	1155.35
15:150000	YOL087C	YGL189C	-972.175	-965.019	-949.292

15:150000	YOL087C	YPR043W	-1186.48	-1182.96	-1154.45
15:150000	YOL087C	YPL132W	4.86134	16.4538	30.4913
15:150000	YOL087C	YER088C	-159.319	-154.172	-54.0351
15:150000	YOL087C	YOL127W	-1135.37	-1126.09	-1108.59
15:150000	YOL087C	YEL047C	-920.589	-917.498	-872.884
15:150000	YOL087C	YAL005C	510.511	515.111	591.406
15:150000	YOL087C	YML026C	-288.09	-283.383	-251.067
15:150000	YOL087C	YKL072W	207.06	212.745	266.942
15:150000	YOL087C	YGR031W	-348.743	-339.619	-253.806
15:150000	YOL087C	YLR150W	-1248.73	-1239.35	-1222.7
15:150000	YOL087C	YDL235C	-590.217	-582.317	-517.668
15:150000	YOL087C	YGR033C	-626.491	-625.811	-582.254
15:150000	YOL087C	YJL189W	-556.26	-553.517	-540.715
15:150000	YOL087C	YPL048W	-1240.94	-1238.68	-1205.86
15:150000	YOL087C	YGL229C	86.26	87.0129	182.255
15:150000	YOL087C	YGR175C	-601.951	-599.51	-577.406
15:150000	YOL087C	YML063W	-698.878	-688.913	-666.797
15:150000	YOL087C	YEL058W	-713.838	-712.721	-658.887
15:150000	YOL087C	YKR094C	-515.983	-514.554	-480.234
15:150000	YOL087C	YGR001C	-862.404	-858.789	-859.353
15:150000	YOL087C	YLR312C	346.296	350.031	523.061
15:150000	YOL087C	YIL046W	-115.054	-108.038	126.804
15:150000	YOL087C	YBL072C	-821.853	-814.401	-796.77
15:150000	YOL087C	YDR012W	-297.59	-294.884	-253.99
15:150000	YOL087C	YHR156C	-248.9	-239.842	-181.856
15:150000	YOL087C	YOR312C	-603.188	-598.71	-550.117
15:150000	YOL087C	YNL178W	-726.548	-722.878	-717.959
15:150000	YOL087C	YIL108W	-709.468	-705.739	-667.752
15:150000	YOL087C	YMR229C	971.696	974.308	1167.85
15:150000	YOL087C	YGL040C	-323.318	-319.32	-319.724
15:150000	YOL087C	YPL118W	-404.089	-403.386	-379.706
15:150000	YOL087C	YGR034W	-274.272	-268.567	-234.3
15:150000	YOL087C	YFR044C	-1160.58	-1153.43	-1106.88
15:150000	YOL087C	YIL117C	103.066	108.576	106.522
15:150000	YOL087C	YER169W	-2.70899	0.0477629	200.415
15:150000	YOL087C	YOL081W	-650.169	-648.57	-586.595
15:150000	YOL087C	YKL096W-A	-1784.56	-1777.12	-1736.03
15:150000	YOL087C	YDR046C	467.336	468.299	611.738
15:150000	YOL087C	YNR036C	-429.286	-429.213	-425.439
15:150000	YOL087C	YPL175W	171.938	174.045	431.595
15:150000	YOL087C	YMR012W	-689.29	-686.208	-653.641
15:150000	YOL087C	YIR035C	-701.613	-696.371	-635.097
15:150000	YOL087C	YML024W	-938.877	-932.667	-919.339
15:150000	YOL087C	YNL209W	-163.497	-162.126	-131.384
15:150000	YOL087C	YOR167C	-627.135	-626.156	-588.398
15:150000	YOL087C	YBL035C	-366.529	-366.026	-357.027
15:150000	YOL087C	YJL181W	-473.846	-463.905	-434.751

15:150000	YOL087C	YOL039W	-794.067	-791.493	-755.602
15:150000	YOL087C	YNR033W	-384.808	-377.757	-383.867
15:150000	YOL087C	YPL249C-A	-891.733	-891.629	-871.184
15:150000	YOL087C	YOR354C	-298.464	-287.223	-293.386
15:150000	YOL087C	YLR023C	-299.082	-296.125	-129.09
15:150000	YOL087C	YER045C	-313.392	-309.444	-240.602
15:150000	YOL087C	YLR168C	98.9285	107.781	116.682
15:150000	YOL087C	YBR104W	318.672	324.975	543.843
15:150000	YOL087C	YLR194C	-118.333	-117.869	-38.3544
15:150000	YOL087C	YKL056C	-1488.71	-1488.4	-1457.86
15:150000	YOL087C	YNL284C	-200.077	-190.624	-195.986
15:150000	YOL087C	YOR107W	-7.98326	1.92718	57.5561
15:150000	YOL087C	YHR092C	629.72	640.92	979.276
15:150000	YOL087C	YCR031C	-1009.82	-1007.21	-981.964
15:150000	YOL087C	YNL081C	45.3268	51.1051	48.156
15:150000	YOL087C	YDR351W	-119.571	-111.08	46.3692
15:150000	YOL087C	YER131W	290.679	295.147	340.903
15:150000	YOL087C	YFL036W	-336.986	-324.914	-162.413
15:150000	YOL087C	YDR047W	-403.068	-396.852	-389.166
15:150000	YOL087C	YHR007C	-813.962	-808.135	-809.461
15:150000	YOL087C	YDR260C	-517.231	-511.043	-361.621
15:150000	YOL087C	YHR141C	-899.279	-892.843	-861.978
15:150000	YOL087C	YGR220C	-137.932	-127.71	-127.875
15:150000	YOL087C	YDR494W	22.2371	32.7009	35.2901
15:150000	YOL087C	YOL119C	34.3039	34.446	226.305
15:150000	YOL087C	YJL190C	-152.045	-146.849	-120.191
15:150000	YOL087C	YBR189W	-366.248	-365.133	-337.773
15:150000	YOL087C	YNL302C	-453.968	-444.16	-424.965
15:150000	YOL087C	YML126C	-1043.04	-1042.69	-1023.35
15:150000	YOL087C	YJR077C	-842.483	-830.222	-801.81
15:150000	YOL087C	YDR132C	-434.493	-424.257	-413.267
15:150000	YOL087C	YPR132W	-1039.12	-1028.85	-1011.02
15:150000	YOL087C	YPL053C	-838.653	-830.53	-814.489
15:150000	YOL087C	YKL006W	-549.009	-544.7	-517.643
15:150000	YOL087C	YMR011W	558.134	566.179	652.108
15:150000	YOL087C	YLR332W	-177.647	-175.353	-168.03
15:150000	YOL087C	YOL095C	-374.614	-370.487	-216.403
15:150000	YOL087C	YPR018W	-471.433	-468.753	-453.635
15:150000	YOL087C	YPL040C	-8.28735	1.93227	0.225952
15:150000	YOL087C	YGL115W	-995.571	-984.862	-958.744
15:150000	YOL087C	YMR230W	-239.936	-232.683	-183.925
15:150000	YOL087C	YGL147C	-354.043	-342.591	-340.224
15:150000	YOL087C	YOR286W	-387.059	-384.064	-346.038
15:150000	YOL087C	YOL056W	-569.78	-566.472	-565.051
15:150000	YOL087C	YOR311C	-243.187	-236.384	-126.874
15:150000	YOL087C	YNL302C	-919.207	-918.189	-884.889
15:150000	YOL087C	YER001W	-114.53	-106.712	-56.3808

15:150000	YOL087C	YLL046C	115.239	124.113	218.757
15:150000	YOL087C	YKL052C	-863.544	-854.819	-857.676
15:150000	YOL087C	YOL121C	23.9076	24.7157	87.6547
15:150000	YOL087C	YGL123W	-947.615	-942.952	-932.091
15:150000	YOL087C	YNL137C	-102.414	-90.3637	-96.2814
15:150000	YOL087C	YOR312C	-705.682	-698.84	-667.525
15:150000	YOL087C	YNL069C	-706.082	-702.285	-677.232
15:150000	YOL087C	YJR122W	-204.202	-197.033	-189.402
15:150000	YOL087C	YER130C	-438.187	-431.991	-298.098
15:150000	YOL087C	YOL091W	601.118	601.41	696.06
15:150000	YOL087C	YHR010W	-565.154	-558.691	-520.822
15:150000	YOL087C	YMR295C	-1300.85	-1293.37	-1287.41
15:150000	YOL087C	YKL087C	49.8074	59.4357	86.2916
15:150000	YOL087C	YER074W	-663.856	-654.615	-640.726
15:150000	YOL087C	YJR043C	-557.562	-557.421	-554.802
15:150000	YOL087C	YPL014W	759.177	760.504	1062.96
15:150000	YOL087C	YGR138C	226.414	230.572	305.827
15:150000	YOL087C	YGR118W	-1113.18	-1105.58	-1076.68
15:150000	YOL087C	YOR096W	-508.647	-501.483	-477.312
15:150000	YOL087C	YOR293W	-61.9893	-55.5547	-25.0183
15:150000	YOL087C	YMR286W	-67.3659	-59.4535	-43.357
15:150000	YOL087C	YFR032C-A	-574.613	-568.073	-553.94
15:150000	YOL087C	YPL061W	-62.2577	-53.5655	27.7589
15:150000	YOL087C	YPL156C	25.8149	35.5356	31.9516
15:150000	YOL087C	YER117W	-572.618	-566.763	-529.739
15:150000	YOL087C	YBR191W	-724.498	-720.127	-693.322
15:150000	YOL087C	YPL089C	99.2346	108.976	251.071
15:150000	YOL087C	YPR036W	-1151.59	-1146.19	-1068.94
15:150000	YOL087C	YBR162W-A	-491.137	-489.673	-480.187
15:150000	YOL087C	YMR145C	92.8965	103.798	131.874
15:150000	YOL087C	YDR420W	-379.269	-378.187	-313.332
15:150000	YOL087C	YBR025C	-786.711	-780.958	-706.275
15:150000	YOL087C	YDR041W	4.06511	15.8312	9.69386
15:150000	YOL087C	YLL045C	-721.321	-719.159	-688.604
15:150000	YOL087C	YOR315W	378.611	384.955	544.973
15:150000	YOL087C	YLR340W	-1011.06	-1007.99	-957.739
15:150000	YOL087C	YKL104C	-786.667	-784.397	-776.38
15:150000	YOL087C	YOL094C	-473.44	-464.746	-346.41
15:150000	YOL087C	YBR181C	-1088.21	-1078.7	-1035.6
15:150000	YOL087C	YDL081C	-781.594	-773.482	-760.9
15:150000	YOL087C	YNL294C	-269.462	-267.093	-243.512
15:150000	YOL087C	YGL025C	-486.825	-481.355	-485.248
15:150000	YOL087C	YAL041W	-776.435	-771.155	-770.588
15:150000	YOL087C	YHR203C	-1405.53	-1398.78	-1370.48
15:150000	YOL087C	YMR143W	-848.711	-841.476	-809.739
15:150000	YOL087C	YBL061C	80.619	91.2349	334.082
15:150000	YOL087C	YKL073W	-549.861	-540.369	-509.707

15:150000	YOL087C	YBR048W	-809.541	-806.319	-782.958
15:150000	YOL087C	YGL135W	-1340.79	-1334.47	-1288.66
15:150000	YOL087C	YDR135C	-881	-879.235	-851.409
15:150000	YOL087C	YJR090C	-804.406	-793.074	-711.405
15:150000	YOL087C	YDL075W	-664.174	-660.929	-641.419
15:150000	YOL087C	YJL112W	-243.103	-231.687	-190.622
15:150000	YOL087C	YHL001W	-948.894	-947.735	-901.451
15:150000	YOL087C	YDR482C	-527.024	-525.409	-366.222
15:150000	YOL087C	YHR021C	-524.809	-524.316	-490.086
15:150000	YOL087C	YMR217W	382.433	385.17	461.698
15:150000	YOL087C	YLL012W	184.547	187.484	304.85
15:150000	YOL087C	YBR191W	-247.961	-238.877	-223.493
15:150000	YOL087C	YNR065C	197.53	200.745	260.507
15:150000	YOL087C	YGR060W	-1133.22	-1130.17	-1079.64
15:150000	YOL087C	YLR121C	397.41	402.574	531.411
15:150000	YOL087C	YKR013W	-209.246	-203.944	-156.745
15:150000	YOL087C	YPR149W	-264.646	-263.744	-125.419
15:150000	YOL087C	YBL092W	-931.549	-924.495	-916.439
15:150000	YOL087C	YFR006W	-972.427	-967.892	-955.07
15:150000	YOL087C	YOL030W	-1116.19	-1112.83	-1105.93
15:150000	YOL087C	YFR031C-A	-952.87	-948.615	-929.597
15:150000	YOL087C	YOR293W	-102.19	-94.9307	-64.9099
15:150000	YOL087C	YGL030W	-1168.72	-1168.51	-1144.29
15:150000	YOL082W	YNR038W	67.4475	72.8008	146.243
15:150000	YOL082W	YKL096W	-571.269	-561.617	-467.843
15:150000	YOL082W	YHR197W	1120.28	1129.87	1125.97
15:150000	YOL082W	YOR084W	-390.187	-378.646	-74.8707
15:150000	YOL082W	YDR245W	-754.277	-746.845	-722.142
15:150000	YOL082W	YOR187W	-441.428	-430.893	-382.229
15:150000	YOL082W	YKL167C	-138.423	-128.572	-77.6291
15:150000	YOL082W	YCL054W	712.502	718.387	780.001
15:150000	YOL082W	YHR128W	-395.463	-391.569	-384.762
15:150000	YOL082W	YOL077W-A	-319.392	-309.575	-314.004
15:150000	YOL082W	YGR083C	-397.248	-388.88	-348.35
15:150000	YOL082W	YML091C	190.7	201.532	429.767
15:150000	YOL082W	YPR155C	-117.304	-114.882	54.9457
15:150000	YOL082W	YNL055C	-1207.81	-1197.17	-1201.02
15:150000	YOL082W	YML124C	-1229.92	-1223.53	-1225.61
15:150000	YOL082W	YHR065C	757.552	768.318	774.632
15:150000	YOL082W	YOL036W	-757.294	-752.306	-686.206
15:150000	YOL082W	YER103W	326.481	337.443	520.454
15:150000	YOL082W	YJL205C	-371.33	-368.27	-328.093
15:150000	YOL082W	YJR121W	-902.915	-900.867	-716.529
15:150000	YOL082W	YNL313C	270.2	272.287	360.917
15:150000	YOL082W	YEL036C	-318.08	-316.295	-90.8595
15:150000	YOL082W	YCR059C	-344.33	-343.5	-59.1052
15:150000	YOL082W	YML093W	1008.69	1016.88	1010.98

15:150000	YOL082W	YLL028W	-305.855	-298.538	-257.36
15:150000	YOL082W	YOL109W	-552.343	-545.473	-497.189
15:150000	YOL082W	YMR280C	423.052	431.049	574.683
15:150000	YOL082W	YJR032W	-514.616	-510.523	-451.909
15:150000	YOL082W	YGL038C	-652.186	-647.708	-502.819
15:150000	YOL082W	YPL189W	-198.081	-191.167	-169.539
15:150000	YOL082W	YKR044W	-38.4525	-28.1726	227.2
15:150000	YOL082W	YPR175W	-320.899	-320.623	-319.632
15:150000	YOL082W	YGR205W	48.28	58.4264	420.89
15:150000	YOL082W	YMR239C	546.058	547.275	662.747
15:150000	YOL082W	YNR066C	534.476	543.848	676.499
15:150000	YOL082W	YBR162C	-784.773	-776.924	-736.047
15:150000	YOL082W	YDL031W	362.428	369.748	381.132
15:150000	YOL082W	YDR337W	44.8786	50.3599	131.882
15:150000	YOL082W	YHR072W	-734.865	-734.677	-369.851
15:150000	YOL082W	YBR023C	-564.31	-559.976	-268.529
15:150000	YOL082W	YKR043C	-353.282	-352.068	-313.382
15:150000	YOL082W	YLR222C	1111.05	1119.63	1152.07
15:150000	YOL082W	YHR140W	115.651	120.129	163.327
15:150000	YOL082W	YHR062C	-87.702	-82.3094	-65.8618
15:150000	YOL082W	YDR490C	-368.808	-360.362	-116.205
15:150000	YOL082W	YPR140W	-450.882	-448.577	-375.415
15:150000	YOL082W	YGR208W	-684.698	-675.931	-561.736
15:150000	YOL082W	YER126C	848.854	854.584	850.908
15:150000	YOL082W	YER088C	-54.3319	-49.3518	-51.8625
15:150000	YOL082W	YAR002C-A	-1360.47	-1353.93	-1331.23
15:150000	YOL082W	YER150W	457.417	460.763	661.702
15:150000	YOL082W	YER066W	-307.472	-300.395	-234.2
15:150000	YOL082W	YGL190C	-465.026	-458.102	-395.122
15:150000	YOL082W	YJL208C	-113.096	-101.072	-53.2266
15:150000	YOL082W	YNL045W	-916.668	-907.809	-790.515
15:150000	YOL082W	YIL091C	733.459	735.029	827.94
15:150000	YOL082W	YDR129C	-776.341	-773.314	-734.108
15:150000	YOL082W	YAL005C	179.613	183.86	586.579
15:150000	YOL082W	YKL072W	-54.4642	-47.138	267.637
15:150000	YOL082W	YLR204W	-37.4668	-28.5034	-16.3214
15:150000	YOL082W	YBR111C	-936.531	-935.18	-905.483
15:150000	YOL082W	YPR060C	-408.291	-404.619	-372.737
15:150000	YOL082W	YIR012W	299.423	303.05	346.112
15:150000	YOL082W	YDL235C	-629.763	-619.961	-508.396
15:150000	YOL082W	YGR217W	-435.404	-428.169	-57.599
15:150000	YOL082W	YGL128C	-692.885	-684.518	-647.106
15:150000	YOL082W	YDR202C	-520.476	-512.556	-315.925
15:150000	YOL082W	YJL137C	-72.6991	-72.35	7.8837
15:150000	YOL082W	YMR244C-A	-356.799	-352.813	-327.086
15:150000	YOL082W	YJR097W	732.406	733.433	943.17
15:150000	YOL082W	YMR251W-A	-1013.15	-1012.83	-968.364

15:150000	YOL082W	YER177W	-1330.86	-1319.25	-1288.33
15:150000	YOL082W	YHR207C	-817.922	-807.133	-712.28
15:150000	YOL082W	YFL054C	303.673	315.834	404.449
15:150000	YOL082W	YGR175C	-712.902	-701.461	-566.366
15:150000	YOL082W	YKR026C	-353.781	-345.41	-313.702
15:150000	YOL082W	YJR054W	-596.345	-594.938	-453.464
15:150000	YOL082W	YEL050C	-91.3714	-80.2833	-76.7627
15:150000	YOL082W	YGR001C	-886.622	-882.74	-846.351
15:150000	YOL082W	YPL084W	-751.699	-741.894	-628.154
15:150000	YOL082W	YOL152W	217.526	229.346	514.38
15:150000	YOL082W	YLR213C	58.5427	63.4697	292.834
15:150000	YOL082W	YHR176W	-173.994	-163.621	-147.636
15:150000	YOL082W	YKR063C	110.387	114.297	495.958
15:150000	YOL082W	YNR034W	16.9958	24.363	196.338
15:150000	YOL082W	YHR156C	-229.924	-219.03	-176.003
15:150000	YOL082W	YEL052W	-746.962	-735.818	-607.687
15:150000	YOL082W	YGL004C	-619.99	-613.198	-545.215
15:150000	YOL082W	YLR295C	-193.666	-182.54	-62.311
15:150000	YOL082W	YLR200W	-449.299	-441.033	-253.543
15:150000	YOL082W	YPL118W	-430.106	-421.928	-376.316
15:150000	YOL082W	YKR018C	-936.969	-936.837	-903.08
15:150000	YOL082W	YOL041C	611.858	618.982	613.8
15:150000	YOL082W	YFR011C	-98.9071	-88.5481	-49.5075
15:150000	YOL082W	YPL183W-A	-28.8087	-20.2314	-22.9152
15:150000	YOL082W	YER162C	-359.096	-357.879	-297.085
15:150000	YOL082W	YOL080C	686.702	692.608	689.209
15:150000	YOL082W	YBR230C	244.398	247.812	677.613
15:150000	YOL082W	YOL081W	-645.253	-643.038	-578.707
15:150000	YOL082W	YDR300C	378.309	383.035	398.226
15:150000	YOL082W	YDR046C	294.313	306.156	605.424
15:150000	YOL082W	YOR004W	1108.78	1115.67	1109.57
15:150000	YOL082W	YGR086C	-1206.41	-1204.86	-1059.39
15:150000	YOL082W	YOL084W	674.147	674.691	904.667
15:150000	YOL082W	YHL036W	-186.947	-184.042	36.4975
15:150000	YOL082W	YNR036C	-476.425	-464.443	-417.69
15:150000	YOL082W	YMR114C	-397.761	-396.511	-380.527
15:150000	YOL082W	YPL175W	-36.975	-27.0727	429.201
15:150000	YOL082W	YJR016C	-193.21	-187.187	-111.137
15:150000	YOL082W	YIL127C	766.12	773.995	782.413
15:150000	YOL082W	YLR002C	950.408	961.641	962.062
15:150000	YOL082W	YDL121C	-68.2418	-58.816	-48.8858
15:150000	YOL082W	YCR030C	-657.809	-654.558	-442.898
15:150000	YOL082W	YOL092W	-461.23	-455.447	-384.586
15:150000	YOL082W	YIR035C	-757.996	-748.231	-625.071
15:150000	YOL082W	YCL057C-A	-798.233	-790.877	-796.572
15:150000	YOL082W	YDR339C	-410.119	-408.734	-251.305
15:150000	YOL082W	YER002W	33.3881	39.734	101.519

15:150000	YOL082W	YJL181W	-571.54	-566.694	-425.2
15:150000	YOL082W	YNR033W	-478.758	-467.179	-375.986
15:150000	YOL082W	YGR062C	-347.608	-336.639	-290.344
15:150000	YOL082W	YLR196W	1170.64	1178.88	1175.14
15:150000	YOL082W	YLL026W	275.63	279.443	695.501
15:150000	YOL082W	YPL015C	-621.241	-615.668	-565.637
15:150000	YOL082W	YER045C	-385.493	-375.962	-234.056
15:150000	YOL082W	YLR168C	104.06	108.304	117.201
15:150000	YOL082W	YBR104W	233.757	243.789	542.884
15:150000	YOL082W	YLR194C	-59.6322	-54.0636	-36.0037
15:150000	YOL082W	YKR017C	-96.6197	-87.1576	-38.8249
15:150000	YOL082W	YLR102C	-490.572	-489.242	-422.525
15:150000	YOL082W	YJR003C	-25.9155	-20.0423	57.8731
15:150000	YOL082W	YCL051W	-356.499	-349.384	-157.902
15:150000	YOL082W	YOR207C	591.881	597.411	592.712
15:150000	YOL082W	YDL241W	-311.95	-304.845	-287.935
15:150000	YOL082W	YDR351W	-277.151	-274.568	48.7275
15:150000	YOL082W	YGR156W	-139.151	-128.878	31.7922
15:150000	YOL082W	YPL119C-A	-102.626	-97.219	-71.6645
15:150000	YOL082W	YLR301W	-973.276	-967.739	-960.969
15:150000	YOL082W	YDR047W	-516.48	-515.968	-381.938
15:150000	YOL082W	YHR007C	-865.711	-857.048	-795.202
15:150000	YOL082W	YCR004C	-1107.38	-1102.93	-951.807
15:150000	YOL082W	YDR342C	544.667	555.577	949.747
15:150000	YOL082W	YEL024W	-37.0594	-34.8487	257.095
15:150000	YOL082W	YJR132W	158.179	166.946	194.353
15:150000	YOL082W	YOL034W	-402.764	-399.254	-393.178
15:150000	YOL082W	YGR220C	-232.651	-230.523	-122.636
15:150000	YOL082W	YOR005C	-662.794	-660.284	-641.338
15:150000	YOL082W	YJL210W	-412.506	-406.149	-330.608
15:150000	YOL082W	YML071C	-692.755	-690.591	-668.757
15:150000	YOL082W	YMR272C	-792.993	-780.959	-662.53
15:150000	YOL082W	YKL026C	-2.1951	-2.02462	111.259
15:150000	YOL082W	YLR084C	-420.809	-419.302	-290.14
15:150000	YOL082W	YPL271W	-899.764	-892.563	-848.675
15:150000	YOL082W	YHR047C	-480.555	-478.047	-343.624
15:150000	YOL082W	YFR053C	861.13	865.89	1196.23
15:150000	YOL082W	YOR254C	-688.457	-685.868	-684.462
15:150000	YOL082W	YOR358W	-312.919	-302.634	-291.714
15:150000	YOL082W	YJR077C	-813.325	-804.921	-794.658
15:150000	YOL082W	YMR206W	274.826	276.003	374.633
15:150000	YOL082W	YPR030W	146.96	154.436	177.535
15:150000	YOL082W	YDR132C	-447.2	-437.557	-413.299
15:150000	YOL082W	YDR204W	-433.041	-429.465	-360.106
15:150000	YOL082W	YPL053C	-926.536	-919.336	-802.425
15:150000	YOL082W	YPL222W	24.2935	29.0404	102.108
15:150000	YOL082W	YOR348C	1301.23	1308.02	1529.11

15:150000	YOL082W	YIL158W	-173.95	-166.268	-134.838
15:150000	YOL082W	YLR143W	-653.698	-646.696	-625.919
15:150000	YOL082W	YMR267W	-88.8075	-84.1741	62.4552
15:150000	YOL082W	YOR188W	-470.805	-461.466	-400.043
15:150000	YOL082W	YNL053W	-151.851	-149.613	299.961
15:150000	YOL082W	YGL209W	337.297	345.987	416.362
15:150000	YOL082W	YMR093W	611.625	618.23	690.875
15:150000	YOL082W	YDL021W	541.224	545.011	601.662
15:150000	YOL082W	YLR332W	-226.218	-223.46	-162.543
15:150000	YOL082W	YJR041C	565.109	573.565	605.121
15:150000	YOL082W	YPL040C	-125.913	-120.504	2.16425
15:150000	YOL082W	YGL115W	-1083.39	-1079.54	-946.743
15:150000	YOL082W	YPL104W	-149.038	-139.207	-116.728
15:150000	YOL082W	YOR048C	-569.276	-563.192	-443.968
15:150000	YOL082W	YBR029C	-338.248	-326.502	-191.794
15:150000	YOL082W	YMR296C	-773.182	-765.397	-702.754
15:150000	YOL082W	YLR146C	-252.068	-241.648	-179.962
15:150000	YOL082W	YOR286W	-342.826	-333.492	-338.777
15:150000	YOL082W	YNL115C	-348.193	-343.486	-312.932
15:150000	YOL082W	YOR311C	-620.766	-617.171	-123.418
15:150000	YOL082W	YGL037C	-289.952	-283.804	-141.127
15:150000	YOL082W	YER001W	-67.8673	-59.2384	-52.9116
15:150000	YOL082W	YPL052W	236.57	244.41	476.251
15:150000	YOL082W	YLL041C	-79.7751	-75.5647	138.909
15:150000	YOL082W	YLR375W	-694.328	-688.213	-686.221
15:150000	YOL082W	YKL155C	-30.4351	-22.5601	21.9586
15:150000	YOL082W	YKL052C	-847.698	-839.714	-845.05
15:150000	YOL082W	YJL010C	625.953	632.364	636.355
15:150000	YOL082W	YHR025W	-750.943	-739.537	-683.63
15:150000	YOL082W	YKR093W	13.6746	18.1842	237.641
15:150000	YOL082W	YHR088W	1001.91	1007.09	1065.3
15:150000	YOL082W	YJR122W	-239.686	-237.275	-182.431
15:150000	YOL082W	YER130C	-564.544	-552.734	-294.53
15:150000	YOL082W	YOR220W	-269.415	-262.339	-43.8309
15:150000	YOL082W	YIL155C	473.217	477.511	579.472
15:150000	YOL082W	YBR034C	308.955	312.99	445.541
15:150000	YOL082W	YER082C	623.553	628.5	694.682
15:150000	YOL082W	YOL116W	-593.834	-592.681	-534.775
15:150000	YOL082W	YMR295C	-1331.05	-1320.91	-1269.89
15:150000	YOL082W	YDR263C	31.7149	33.9247	87.9694
15:150000	YOL082W	YMR072W	-1018.42	-1010.62	-1014
15:150000	YOL082W	YER067W	1141.18	1141.9	1285.63
15:150000	YOL082W	YER049W	358.544	361.54	370.63
15:150000	YOL082W	YJL062W-A	-149.527	-137.978	-130.599
15:150000	YOL082W	YBL030C	-783.118	-771.375	-722.434
15:150000	YOL082W	YPL014W	658.849	660.661	1056.89
15:150000	YOL082W	YGR138C	221.526	228.771	303.968

15:150000	YOL082W	YGL081W	18.1068	19.3548	198.949
15:150000	YOL082W	YDR421W	-318.309	-310.048	-306.455
15:150000	YOL082W	YGR021W	51.8924	52.9818	140.621
15:150000	YOL082W	YBR284W	84.9564	96.9157	349.077
15:150000	YOL082W	YMR157C	-12.2197	-6.6341	23.4305
15:150000	YOL082W	YDR296W	12.7375	16.4193	97.3032
15:150000	YOL082W	YMR286W	-66.2992	-54.6529	-39.5191
15:150000	YOL082W	YDL079C	763.129	766.736	937.964
15:150000	YOL082W	YCR043C	-703.493	-694.367	-665.145
15:150000	YOL082W	YOR274W	-190.078	-181.029	16.6891
15:150000	YOL082W	YNR060W	611.46	618.061	612.682
15:150000	YOL082W	YGR244C	-290.981	-284.788	-288.225
15:150000	YOL082W	YDL166C	-145.31	-143.255	-76.5997
15:150000	YOL082W	YIL008W	-34.9642	-24.8408	11.2296
15:150000	YOL082W	YJR034W	68.1379	73.7531	173.169
15:150000	YOL082W	YPL156C	25.9419	36.0628	34.4197
15:150000	YOL082W	YIL039W	-1091.76	-1081.8	-1002.35
15:150000	YOL082W	YKR079C	840.736	852.101	925.803
15:150000	YOL082W	YBR162W-A	-523.228	-512.762	-469.942
15:150000	YOL082W	YMR145C	124.206	135.484	134.458
15:150000	YOL082W	YDR420W	-418.022	-407.215	-307.37
15:150000	YOL082W	YGL145W	-559.621	-557.108	-536.956
15:150000	YOL082W	YLL034C	972.612	980.574	976.061
15:150000	YOL082W	YPR033C	-672.788	-662.925	-630.613
15:150000	YOL082W	YAR029W	100.661	111.64	231.208
15:150000	YOL082W	YKL104C	-797.037	-793.553	-764.681
15:150000	YOL082W	YOL094C	-343.828	-334.663	-339.879
15:150000	YOL082W	YJL011C	344.85	347.453	417.411
15:150000	YOL082W	YER062C	-433.076	-424.046	-388.644
15:150000	YOL082W	YHR208W	-62.3482	-57.1728	-16.1582
15:150000	YOL082W	YHL024W	751.596	761.85	1033.45
15:150000	YOL082W	YJL069C	638.548	645.541	737.248
15:150000	YOL082W	YGL025C	-514.761	-504.383	-482.094
15:150000	YOL082W	YAL041W	-758.26	-752.335	-758.215
15:150000	YOL082W	YGR238C	-345.395	-343.9	-122.953
15:150000	YOL082W	YCL040W	126.078	135.434	325.35
15:150000	YOL082W	YBL061C	-260.354	-253.664	334.011
15:150000	YOL082W	YDR329C	-770.342	-767.854	-744.84
15:150000	YOL082W	YER060W	13.2766	16.1526	155.197
15:150000	YOL082W	YGL237C	38.5683	38.9085	129.17
15:150000	YOL082W	YDR037W	-402.833	-396.752	-267.769
15:150000	YOL082W	YNL256W	-349.578	-347.859	-259.149
15:150000	YOL082W	YNR001C	-510.727	-498.71	-444.866
15:150000	YOL082W	YAL039C	-109.042	-108.466	-97.1596
15:150000	YOL082W	YDR135C	-951.64	-945.709	-838.375
15:150000	YOL082W	YGR195W	-592.754	-590.945	-568.499
15:150000	YOL082W	YEL040W	-320.941	-320.425	56.0793

15:150000	YOL082W	YJR090C	-832.632	-825.229	-706.552
15:150000	YOL082W	YJL112W	-304.523	-294.845	-187.619
15:150000	YOL082W	YMR310C	237.854	244.219	256.564
15:150000	YOL082W	YLR164W	314.271	321.829	340.357
15:150000	YOL082W	YLL012W	-52.9784	-52.7397	305.913
15:150000	YOL082W	YNR065C	-2.57853	-1.95271	258.837
15:150000	YOL082W	YGR060W	-1169.86	-1159.22	-1065.3
15:150000	YOL082W	YLR121C	331.495	339.694	529.749
15:150000	YOL082W	YKR013W	-496.442	-489.956	-150.692
15:150000	YOL082W	YDR298C	-752.694	-750.316	-586.599
15:150000	YOL082W	YPR149W	-332.349	-321.219	-125.8
15:150000	YOL082W	YGL255W	575.282	582.106	912.4
15:150000	YOL082W	YFR006W	-950.514	-942.016	-948.149
15:150000	YOL082W	YFL041W-A	-303.754	-294.086	-225.7
15:150000	YOL082W	YER132C	-188.775	-182.679	281.408
15:150000	YOL082W	YOL030W	-1100.98	-1096.78	-1089.26
16:510000	YPL020C	YML124C	-1241.11	-1234.01	-1222.96
16:510000	YPL020C	YIL069C	278.95	281.382	278.985
16:510000	YPL020C	YPR191W	-192.774	-192.097	-67.0603
16:510000	YPL020C	YPR020W	-317.443	-313.328	-288.404
16:510000	YPL020C	YML026C	-255.39	-255.253	-248.289
16:510000	YPL020C	YML063W	-670.658	-665.902	-656.002
16:510000	YPL020C	YOL152W	363.861	370.933	505.146
16:510000	YPL020C	YBL072C	-779.051	-773.99	-777.49
16:510000	YPL020C	YGR281W	-445.267	-435.845	-304.707
16:510000	YPL020C	YKR034W	1495.56	1499.6	1496.31
16:510000	YPL020C	YPL054W	588.755	589.243	656.032
16:510000	YPL020C	YEL063C	60.5406	62.5979	74.4922
16:510000	YPL020C	YPL024W	-217.706	-214.83	-201.211
16:510000	YPL020C	YGR086C	-1127.21	-1126.3	-1011.92
16:510000	YPL020C	YFR055W	-238.417	-232.873	-235.178
16:510000	YPL020C	YIR032C	649.132	657.18	659.515
16:510000	YPL020C	YDR260C	-363.955	-362.661	-355.227
16:510000	YPL020C	YPL022W	-896.182	-892.177	-886.794
16:510000	YPL020C	YLR333C	-484.232	-483.059	-475.597
16:510000	YPL020C	YPR132W	-1015.48	-1013.27	-986.571
16:510000	YPL020C	YOR348C	1348.86	1350.85	1498.39
16:510000	YPL020C	YOR286W	-359.477	-356.435	-334.724
16:510000	YPL020C	YPL046C	-376.975	-369.634	-333.497
16:510000	YPL020C	YEL072W	-229.419	-229.199	-223.84
16:510000	YPL020C	YGR138C	241.32	249.173	297.924
16:510000	YPL020C	YDL210W	1301	1308.27	1304.14
16:510000	YPL020C	YHL024W	858.278	869.88	1034.88
16:510000	YPL020C	YAL024C	-463.142	-459.573	-384.998
16:510000	YPL020C	YNR001C	-543.724	-539.036	-434.11
16:510000	YPL020C	YDR033W	-152.278	-140.187	-93.285
16:510000	YPL020C	YFL014W	696.019	699.182	815.6

16:510000	YPL020C	YBL092W	-897.681	-897.546	-896.871
16:510000	YPL020C	YDR379W	-725.426	-713.345	-709.876
16:510000	YPL016W	YJL147C	-199.94	-194.51	-193.461
16:510000	YPL016W	YEL006W	-397.26	-386.722	-362.34
16:510000	YPL016W	YPL020C	-773.252	-762.806	-752.178
16:510000	YPL016W	YML124C	-1176.58	-1166.11	-1167.88
16:510000	YPL016W	YKL047W	-555.492	-544.846	-533.049
16:510000	YPL016W	YPL107W	-167.431	-157.474	-160.011
16:510000	YPL016W	YPR020W	-389.942	-388.136	-298.602
16:510000	YPL016W	YPL024W	-231.376	-221.559	-193.728
16:510000	YPL016W	YOR376W-A	369.547	376.992	378.769
16:510000	YPL016W	YIR027C	1135.24	1137.55	1151.34
16:510000	YPL016W	YIR029W	923.672	927.184	945.839
16:510000	YPL016W	YDR131C	-484.678	-475.229	-477.361
16:510000	YPL016W	YDL052C	-801.208	-797.019	-799.706
16:510000	YPL016W	YMR240C	-357.957	-349.168	-353.165
16:510000	YPL016W	YDR260C	-337.533	-333.032	-333.116
16:510000	YPL016W	YLR142W	830.709	841.048	861.972
16:510000	YPL016W	YPL022W	-854.462	-851.104	-845.29
16:510000	YPL016W	YPL038W-A	-111.701	-105.571	-102.17
16:510000	YPL016W	YGR154C	402.616	414.662	419.035
16:510000	YPL016W	YML068W	-299.266	-288.16	-288.26
16:510000	YPL016W	YGL075C	-469.86	-462.646	-459.743
16:510000	YPL016W	YEL072W	-231.181	-223.743	-207.049
16:510000	YPL016W	YGR138C	283.246	284.07	304.294
16:510000	YPL016W	YDR421W	-316.736	-314.446	-292.198
16:510000	YPL016W	YCL025C	-362.391	-350.375	-307.062
16:510000	YPL016W	YIR028W	1186.84	1187.6	1214.33
16:510000	YPL016W	YDR033W	-215.694	-214.735	-123.218
16:510000	YPL016W	YPL039W	-359.688	-347.938	-332.177
16:510000	YPL016W	YER039C-A	-71.7252	-62.8107	-59.364
16:510000	YPL016W	YER065C	-346.879	-335.212	-333.891
16:510000	YPL016W	YHR099W	-260.781	-249.738	-254.844
16:510000	YPL024W	YKL138C	176.792	180.789	255.164
16:510000	YPL024W	YIR021W	-42.8097	-40.5396	-37.2542
16:510000	YPL024W	YKL195W	-28.4171	-28.0978	18.1942
16:510000	YPL024W	YBR084C-A	-346.796	-336.268	-325.23
16:510000	YPL024W	YKR092C	563.483	572.95	602.453
16:510000	YPL024W	YKL180W	-467.375	-461.688	-452.088
16:510000	YPL024W	YHR038W	325.552	328.19	421.102
16:510000	YPL024W	YPL272C	285.866	292.917	300.688
16:510000	YPL024W	YDL229W	-476.61	-474.97	-445.466
16:510000	YPL024W	YJL063C	71.597	81.6027	121.352
16:510000	YPL024W	YNL073W	-307.439	-302.521	-266.411
16:510000	YPL024W	YGL068W	188.325	197.001	242.15
16:510000	YPL024W	YIL069C	267.227	278.487	283.462
16:510000	YPL024W	YMR225C	11.8546	19.1639	47.3982

16:510000	YPL024W	YML026C	-262.102	-254.288	-255.097
16:510000	YPL024W	YMR116C	-1101.46	-1099.03	-1091.02
16:510000	YPL024W	YIL070C	480.366	489.964	536.776
16:510000	YPL024W	YLR150W	-1265.78	-1264.9	-1235.77
16:510000	YPL024W	YLR259C	-896.57	-890.212	-829.897
16:510000	YPL024W	YML063W	-671.148	-662.393	-668.415
16:510000	YPL024W	YLR048W	-28.4957	-16.4846	-6.39392
16:510000	YPL024W	YBL072C	-820.92	-812.44	-802.881
16:510000	YPL024W	YHR199C-A	-211.332	-210.217	-210.25
16:510000	YPL024W	YIL018W	-249.668	-245.063	-231.596
16:510000	YPL024W	YLR069C	196.278	204.698	261.095
16:510000	YPL024W	YGL040C	-430.59	-428.747	-343.729
16:510000	YPL024W	YBR282W	114.887	116.216	123.169
16:510000	YPL024W	YML129C	61.0199	63.363	139.447
16:510000	YPL024W	YDR508C	318.681	327.036	367.583
16:510000	YPL024W	YDL130W	-683.457	-679.147	-671.749
16:510000	YPL024W	YBR146W	-80.9146	-70.3356	-51.2944
16:510000	YPL024W	YDL083C	42.0392	45.6055	59.0001
16:510000	YPL024W	YJL047C	-756.891	-750.696	-744.393
16:510000	YPL024W	YNL185C	219.797	230.931	281.349
16:510000	YPL024W	YNL209W	-176.87	-169.883	-138.045
16:510000	YPL024W	YDR131C	-521.266	-519.065	-516.938
16:510000	YPL024W	YPL031C	-671.332	-664.073	-670.231
16:510000	YPL024W	YFR007W	-156.85	-148.583	-88.5827
16:510000	YPL024W	YOR354C	-383.7	-374.214	-304.372
16:510000	YPL024W	YDR025W	-42.7103	-36.5395	-24.9033
16:510000	YPL024W	YLL009C	388.954	399.067	431.929
16:510000	YPL024W	YER131W	301.395	305.874	329.845
16:510000	YPL024W	YMR240C	-398.594	-387.03	-376.809
16:510000	YPL024W	YDL045W-A	-143.847	-133.074	-104.354
16:510000	YPL024W	YGR138C	13.7829	21.4201	19.4169
16:510000	YPL024W	YDR500C	-235.177	-224.699	-214.986
16:510000	YPL024W	YPL079W	-424.384	-414.948	-401.879
16:510000	YPL024W	YDR260C	-376.872	-365.091	-363.522
16:510000	YPL024W	YLR061W	-223.503	-213.469	-202.905
16:510000	YPL024W	YDR194C	-226.497	-216.218	-206.463
16:510000	YPL024W	YPL022W	-912.615	-904.411	-909.474
16:510000	YPL024W	YPL098C	-442.688	-434.437	-393.425
16:510000	YPL024W	YLR333C	-510.294	-498.257	-493.124
16:510000	YPL024W	YGL140C	-606.908	-598.244	-599.647
16:510000	YPL024W	YPR132W	-1017.87	-1007.09	-1012.18
16:510000	YPL024W	YER074W	-466.802	-459.266	-451.563
16:510000	YPL024W	YMR011W	614.435	624.194	645.42
16:510000	YPL024W	YPR133W-A	-358.755	-356.265	-347.4
16:510000	YPL024W	YPL040C	-105.634	-96.4098	-17.694
16:510000	YPL024W	YOR286W	-354.858	-350.556	-348.175
16:510000	YPL024W	YML073C	-329.262	-319.208	-310.908

16:510000	YPL024W	YIL131C	-313.896	-306.567	-287.752
16:510000	YPL024W	YNL137C	-176.779	-168.709	-105.019
16:510000	YPL024W	YJR122W	-224.62	-213.477	-201.091
16:510000	YPL024W	YKL087C	-22.8164	-12.0979	70.5188
16:510000	YPL024W	YJL062W-A	-147.413	-139.698	-134.754
16:510000	YPL024W	YCR046C	-87.2973	-75.0844	-61.3065
16:510000	YPL024W	YMR060C	-221.899	-216.908	-186.157
16:510000	YPL024W	YHR022C	791.661	795.425	795.622
16:510000	YPL024W	YBR191W	-732.004	-726.777	-703.261
16:510000	YPL024W	YML009C	-164.231	-158.115	-129.305
16:510000	YPL024W	YPL072W	19.4095	28.344	79.1216
16:510000	YPL024W	YJL112W	-241.058	-229.895	-202.232
16:510000	YPL024W	YDR115W	53.679	59.0299	124.708
16:510000	YPL024W	YHL001W	-920.908	-916.403	-908.426
16:510000	YPL024W	YHR021C	-508.326	-505.085	-497.487
16:510000	YPL024W	YGL249W	84.8844	86.8109	90.5835
16:510000	YPL024W	YBL092W	-940.68	-931.874	-921.098
16:510000	YPL024W	YBR041W	-359.954	-348.92	-321.272
16:510000	YPL024W	YHR099W	-291.234	-281.176	-275.793
16:510000	YPL024W	YDR379W	-736.117	-725.434	-728.631
16:510000	YPL024W	YIL133C	-54.1214	-44.716	-38.6918
16:510000	YPL024W	YOR293W	-91.8671	-81.5807	-70.9047
16:510000	YPL023C	YJL147C	-205.833	-200.572	-194.31
16:510000	YPL023C	YKL138C	212.022	223.016	253.698
16:510000	YPL023C	YEL006W	-392.789	-383.9	-371.737
16:510000	YPL023C	YKL195W	-23.9916	-13.2074	22.3664
16:510000	YPL023C	YOR187W	-385.177	-375.423	-373.588
16:510000	YPL023C	YBR084C-A	-316.686	-308.26	-312.455
16:510000	YPL023C	YKL047W	-561.908	-555.868	-540.861
16:510000	YPL023C	YDL229W	-0.485933	2.33194	7.77807
16:510000	YPL023C	YKL180W	-456.574	-448.821	-441.152
16:510000	YPL023C	YHR038W	333.206	344.322	416.77
16:510000	YPL023C	YBL072C	-1308	-1303.22	-1292.39
16:510000	YPL023C	YER102W	-634.161	-625.595	-611.755
16:510000	YPL023C	YGL189C	-1092.69	-1083.27	-1074.59
16:510000	YPL023C	YDL229W	-448.177	-438.294	-427.041
16:510000	YPL023C	YJL063C	95.7792	107.622	119.665
16:510000	YPL023C	YNL073W	-282.814	-271.573	-259.608
16:510000	YPL023C	YGL068W	224.772	234.337	242.808
16:510000	YPL023C	YPL107W	-192.452	-187.807	-174.855
16:510000	YPL023C	YIL069C	245.726	257.14	278.985
16:510000	YPL023C	YMR225C	32.8676	43.7482	48.0133
16:510000	YPL023C	YML026C	-260.501	-250.094	-248.289
16:510000	YPL023C	YMR116C	-1056.11	-1050.48	-1053.37
16:510000	YPL023C	YIL070C	514.896	524.738	529.198
16:510000	YPL023C	YLR150W	-1216.21	-1208.31	-1202.28
16:510000	YPL023C	YLR259C	-882.682	-872.543	-807.801

16:510000	YPL023C	YML063W	-660.926	-650.415	-656.002
16:510000	YPL023C	YLR048W	-35.9953	-24.1378	-4.39776
16:510000	YPL023C	YBL072C	-786.963	-776.72	-777.49
16:510000	YPL023C	YDR430C	45.9176	56.2803	94.6049
16:510000	YPL023C	YIL018W	-230.373	-223.931	-223.103
16:510000	YPL023C	YLR069C	223.193	235.402	256.834
16:510000	YPL023C	YGL040C	-366.262	-364.31	-331.689
16:510000	YPL023C	YBR037C	247.824	253.822	320.747
16:510000	YPL023C	YLR382C	78.4831	89.4555	106.623
16:510000	YPL023C	YML129C	60.4819	71.3462	135.66
16:510000	YPL023C	YDR508C	264.195	266.677	367.177
16:510000	YPL023C	YDL130W	-669.281	-661.182	-654.316
16:510000	YPL023C	YBR146W	-55.3031	-46.873	-49.9306
16:510000	YPL023C	YDL083C	48.7227	56.1754	60.3454
16:510000	YPL023C	YMR012W	-656.904	-653.136	-646.925
16:510000	YPL023C	YNL185C	254.321	264.605	280.67
16:510000	YPL023C	YNL209W	-152.757	-141.142	-127.299
16:510000	YPL023C	YDR131C	-522.076	-514.16	-501.842
16:510000	YPL023C	YPL031C	-658.751	-651.543	-654.508
16:510000	YPL023C	YFR007W	-147.327	-135.679	-87.4119
16:510000	YPL023C	YOR354C	-326.634	-315.542	-296.776
16:510000	YPL023C	YDR025W	-50.3748	-39.8797	-23.3452
16:510000	YPL023C	YLL009C	413.55	423.761	423.409
16:510000	YPL023C	YDL120W	-748.112	-742.076	-691.929
16:510000	YPL023C	YER131W	294.508	305.305	326.977
16:510000	YPL023C	YMR240C	-378.245	-367.373	-366.14
16:510000	YPL023C	YDL045W-A	-122.443	-111.032	-100.033
16:510000	YPL023C	YDR500C	-234.451	-222.503	-209.637
16:510000	YPL023C	YPL079W	-406.623	-395.54	-388.096
16:510000	YPL023C	YDR260C	-414.171	-404.769	-355.227
16:510000	YPL023C	YLR061W	-223.258	-211.12	-194.298
16:510000	YPL023C	YPL022W	-900.472	-888.701	-886.794
16:510000	YPL023C	YPL098C	-410.383	-399.552	-377.12
16:510000	YPL023C	YLR333C	-511.477	-501.071	-475.597
16:510000	YPL023C	YGL140C	-609.653	-598.594	-577.442
16:510000	YPL023C	YPR132W	-1000.14	-988.134	-986.571
16:510000	YPL023C	YER074W	-450.628	-442.045	-440.05
16:510000	YPL023C	YMR011W	558.207	567.047	638.693
16:510000	YPL023C	YML068W	-321.089	-313.627	-305.357
16:510000	YPL023C	YPL040C	-73.8949	-62.4843	-14.8148
16:510000	YPL023C	YOR286W	-337.912	-329	-334.724
16:510000	YPL023C	YML073C	-318.492	-306.909	-300.27
16:510000	YPL023C	YIL131C	-314.001	-301.726	-272.274
16:510000	YPL023C	YNL137C	-134.308	-122.678	-100.664
16:510000	YPL023C	YKL087C	2.68366	14.5122	74.1047
16:510000	YPL023C	YCR046C	-62.19	-52.77	-56.4447
16:510000	YPL023C	YMR060C	-233.898	-222.009	-174.349

16:510000	YPL023C	YKL054C	-406.677	-394.56	-345.662
16:510000	YPL023C	YBR191W	-690.247	-684.89	-684.182
16:510000	YPL023C	YIL098C	357.918	367.98	389.391
16:510000	YPL023C	YDL210W	1294.91	1301.24	1304.14
16:510000	YPL023C	YML009C	-131.879	-125.79	-128.072
16:510000	YPL023C	YBR181C	-1034.63	-1029.89	-1026.23
16:510000	YPL023C	YPL072W	43.5234	55.0063	78.9347
16:510000	YPL023C	YOR232W	-250.528	-242.933	-245.592
16:510000	YPL023C	YBR048W	-774.412	-772.356	-773.958
16:510000	YPL023C	YJL112W	-303.091	-296.367	-194.812
16:510000	YPL023C	YDR115W	90.9547	102.643	122.975
16:510000	YPL023C	YHL001W	-896.002	-892.362	-893.979
16:510000	YPL023C	YHR021C	-494.498	-487.877	-485.224
16:510000	YPL023C	YGL249W	61.6782	71.0213	86.7768
16:510000	YPL023C	YBL092W	-897.702	-891.501	-896.871
16:510000	YPL023C	YBR041W	-419.112	-412.592	-307.76
16:510000	YPL023C	YHR099W	-290.272	-281.748	-262.727
16:510000	YPL023C	YDR379W	-716.073	-705.603	-709.876
16:510000	YPL023C	YIL133C	-77.5925	-65.353	-39.4362
16:510000	YPL023C	YOR293W	-87.4598	-75.8661	-69.1993
16:510000	YPL031C	YKL138C	27.2745	37.6897	255.164
16:510000	YPL031C	YOR187W	-454.164	-450.43	-383.262
16:510000	YPL031C	YBR084C-A	-437.238	-431.274	-325.23
16:510000	YPL031C	YKR092C	475.709	486.32	602.453
16:510000	YPL031C	YHR038W	52.6618	62.0825	421.102
16:510000	YPL031C	YPL036W	28.7296	30.5229	402.802
16:510000	YPL031C	YPL272C	204.863	207.843	300.688
16:510000	YPL031C	YJL063C	-3.1533	5.76887	121.352
16:510000	YPL031C	YNL073W	-458.299	-452.626	-266.411
16:510000	YPL031C	YGL068W	140.972	147.863	242.15
16:510000	YPL031C	YIL069C	94.7146	99.2262	283.462
16:510000	YPL031C	YMR225C	5.57506	13.2353	47.3982
16:510000	YPL031C	YPR191W	-225.474	-220.462	-65.2809
16:510000	YPL031C	YML026C	-375.112	-373.024	-255.097
16:510000	YPL031C	YIL070C	439.93	449.081	536.776
16:510000	YPL031C	YLR259C	-1006.95	-994.841	-829.897
16:510000	YPL031C	YML063W	-741.39	-734.711	-668.415
16:510000	YPL031C	YDL181W	252.741	257.007	427.994
16:510000	YPL031C	YLR048W	-208.725	-204.138	-6.39392
16:510000	YPL031C	YBL072C	-901.045	-898.175	-802.881
16:510000	YPL031C	YDR430C	-117.496	-110.909	94.4637
16:510000	YPL031C	YLR069C	71.8544	80.1161	261.095
16:510000	YPL031C	YBR282W	111.012	111.435	123.169
16:510000	YPL031C	YLR382C	-146.147	-143.684	106.885
16:510000	YPL031C	YML129C	-229.048	-222.743	139.447
16:510000	YPL031C	YDR508C	77.0504	78.548	367.583
16:510000	YPL031C	YBR146W	-81.6546	-74.0156	-51.2944

16:510000	YPL031C	YGR086C	-1215.18	-1208.39	-1041.69
16:510000	YPL031C	YNL185C	162.916	169.815	281.349
16:510000	YPL031C	YFR007W	-328.733	-323.006	-88.5827
16:510000	YPL031C	YOR354C	-436.79	-430.551	-304.372
16:510000	YPL031C	YLL009C	390.529	399.875	431.929
16:510000	YPL031C	YMR240C	-376.82	-370.646	-376.809
16:510000	YPL031C	YDL045W-A	-163.426	-154.064	-104.354
16:510000	YPL031C	YGR138C	16.2329	16.3168	19.4169
16:510000	YPL031C	YDR500C	-385.738	-384.59	-214.986
16:510000	YPL031C	YDR260C	-464.063	-459.846	-363.522
16:510000	YPL031C	YPL022W	-933.164	-925.135	-909.474
16:510000	YPL031C	YPL098C	-518.005	-510.289	-393.425
16:510000	YPL031C	YLR333C	-702.982	-695.056	-493.124
16:510000	YPL031C	YPL271W	-882.182	-874.713	-845.204
16:510000	YPL031C	YGL140C	-685.232	-682.936	-599.647
16:510000	YPL031C	YPR132W	-1101.44	-1095.47	-1012.18
16:510000	YPL031C	YPL040C	-140.265	-136.621	-17.694
16:510000	YPL031C	YOR286W	-348.679	-343.909	-348.175
16:510000	YPL031C	YML073C	-490.279	-489.907	-310.908
16:510000	YPL031C	YNL137C	-245.302	-235.1	-105.019
16:510000	YPL031C	YJR122W	-260.656	-257.957	-201.091
16:510000	YPL031C	YJL062W-A	-151.129	-145.883	-134.754
16:510000	YPL031C	YCR046C	-71.8592	-64.6042	-61.3065
16:510000	YPL031C	YGR138C	222.788	227.607	305.659
16:510000	YPL031C	YMR060C	-359.45	-357.727	-186.157
16:510000	YPL031C	YIL098C	239.372	246.309	394.528
16:510000	YPL031C	YML009C	-158.739	-152.59	-129.305
16:510000	YPL031C	YPL072W	-64.5888	-54.2318	79.1216
16:510000	YPL031C	YJL112W	-272.665	-262.329	-202.232
16:510000	YPL031C	YDR115W	-15.2335	-5.46563	124.708
16:510000	YPL031C	YBL092W	-1009.83	-1007.35	-921.098
16:510000	YPL031C	YBR041W	-517.106	-505.518	-321.272
16:510000	YPL031C	YHR099W	-291.618	-291.025	-275.793
16:510000	YPL031C	YOR293W	-227.333	-226.858	-70.9047
16:510000	YPL022W	YKR009C	-343.466	-342.838	-336.689
16:510000	YPL022W	YPL020C	-758.147	-756.426	-757.241
16:510000	YPL022W	YML091C	362.421	365.951	440.056
16:510000	YPL022W	YML124C	-1238.74	-1230.84	-1222.96
16:510000	YPL022W	YPL107W	-176.21	-175.318	-174.855
16:510000	YPL022W	YIR031C	906.71	917.091	938.112
16:510000	YPL022W	YPR191W	-164.069	-159.478	-67.0603
16:510000	YPL022W	YJR152W	1026.79	1035.6	1042.64
16:510000	YPL022W	YPR020W	-326.783	-317.835	-288.404
16:510000	YPL022W	YMR251W-A	-930.092	-927.133	-896.77
16:510000	YPL022W	YML063W	-666.058	-665.128	-656.002
16:510000	YPL022W	YDL181W	292.151	292.337	424.536
16:510000	YPL022W	YOL152W	414.22	422.602	505.146

16:510000	YPL022W	YGR281W	-426.846	-423.961	-304.707
16:510000	YPL022W	YKR034W	1475.39	1484.03	1496.31
16:510000	YPL022W	YHR037W	-292.445	-288.412	-176.671
16:510000	YPL022W	YEL063C	35.1481	38.5465	74.4922
16:510000	YPL022W	YPL024W	-238.574	-227.555	-201.211
16:510000	YPL022W	YFR055W	-276.933	-276.324	-235.178
16:510000	YPL022W	YIR027C	1167.41	1176.33	1193.84
16:510000	YPL022W	YIR032C	623.811	630.222	659.515
16:510000	YPL022W	YIR029W	953.856	964.62	980.248
16:510000	YPL022W	YGL187C	-133.02	-131.123	-26.5246
16:510000	YPL022W	YEL024W	121.502	122.38	243.571
16:510000	YPL022W	YLR333C	-477.513	-474.822	-475.597
16:510000	YPL022W	YOR348C	1390.51	1395.94	1498.39
16:510000	YPL022W	YGR154C	429.845	435.318	436.127
16:510000	YPL022W	YPL046C	-421.643	-409.759	-333.497
16:510000	YPL022W	YEL072W	-284.632	-272.596	-223.84
16:510000	YPL022W	YGR138C	247.435	251.018	297.924
16:510000	YPL022W	YDL210W	1268.18	1280.13	1304.14
16:510000	YPL022W	YHL024W	893.554	900.495	1034.88
16:510000	YPL022W	YIR028W	1233.45	1240.36	1248.82
16:510000	YPL022W	YNR001C	-480.499	-468.763	-434.11
16:510000	YPL022W	YDR033W	-131.476	-119.492	-93.285
16:510000	YPL022W	YPL039W	-364.71	-359.361	-352.344
16:510000	YPL022W	YDR379W	-717.626	-705.36	-709.876
